# Comparative morphological and transcriptomic analyses reveal chemosensory genes in the poultry red mite, *Dermanyssus gallinae*

**DOI:** 10.1038/s41598-020-74998-7

**Published:** 2020-10-21

**Authors:** Biswajit Bhowmick, Yu Tang, Fang Lin, Øivind Øines, Jianguo Zhao, Chenghong Liao, Rickard Ignell, Bill S. Hansson, Qian Han

**Affiliations:** 1 Laboratory of Tropical Veterinary Medicine and Vector Biology, School of Life and Pharmaceutical Sciences, Hainan University, Haikou, 570228 Hainan China; 2 Norwegian Veterinary Institute, Ullevaalsveien 68 P.boks 750 Sentrum, 0106 Oslo, Norway; 3 Disease Vector Group, Unit of Chemical Ecology, Department of Plant Protection Biology, Swedish University of Agricultural Sciences, 23053, Alnarp, Sweden; 4 Department of Evolutionary Neuroethology, Max Planck Institute for Chemical Ecology, 07745 Jena, Germany

**Keywords:** Molecular biology, RNAi, Transcriptomics, Peripheral nervous system, Scanning electron microscopy, Parasite physiology

## Abstract

Detection of chemical cues via chemosensory receptor proteins are essential for most animals, and underlies critical behaviors, including location and discrimination of food resources, identification of sexual partners and avoidance of predators. The current knowledge of how chemical cues are detected is based primarily on data acquired from studies on insects, while our understanding of the molecular basis for chemoreception in acari, mites in particular, remains limited. The poultry red mite (PRM), *Dermanyssus gallinae*, is one of the most important blood-feeding ectoparasites of poultry. PRM are active at night which suck the birds' blood during periods of darkness and hide themselves in all kinds of gaps and cracks during the daytime. The diversity in habitat usage, as well as the demonstrated host finding and avoidance behaviors suggest that PRM relies on their sense of smell to orchestrate complex behavioral decisions. Comparative transcriptome analyses revealed the presence of candidate variant ionotropic receptors, odorant binding proteins, niemann-pick proteins type C2 and sensory neuron membrane proteins. Some of these proteins were highly and differentially expressed in the forelegs of PRM. Rhodopsin-like G protein-coupled receptors were also identified, while insect-specific odorant receptors and odorant co-receptors were not detected. Furthermore, using scanning electron microscopy, the tarsomeres of all leg pairs were shown to be equipped with sensilla chaetica with or without tip pores, while wall-pored olfactory sensilla chaetica were restricted to the distal-most tarsomeres of the forelegs. This study is the first to describe the presence of chemosensory genes in any *Dermanyssidae* family. Our findings make a significant step forward in understanding the chemosensory abilities of *D. gallinae.*

## Introduction

Mites are highly diverse arthropods of the subphylum chelicerata, one of the most diverse groups in animal radiations^1^. These eight-legged arthropods exhibit a tremendous variation in lifestyle, ranging from saprophagous to herbivorous and from predatory to parasitic feeding behaviors^2^. Some of these species pose a threat to both animal and human health, such as ticks, mites, spiders and scorpions, and may also vector pathogens of importance to animals, insects, plants and humans. The poultry red mite (PRM), *Dermanyssus gallinae* (de Geer, 1778), is an obligatory hematophagous ectoparasitic mite that infests a wide range of hosts including wild birds, rodents and mammals^1, 3,4^. The PRM is considered the major pest for the poultry industry, with the annual cost in damage and control estimated at €231 million in European countries alone^5^. PRM sucks blood mainly at night, while they hide themselves in cracks and crevices during daytime so that chickens cannot peck and eat them. Their complex behavior makes them difficult to control with conventional acaricides and other treatment practices^5^. Hence, an increased understanding of the peripheral olfactory system and specific olfactory organs, including the identification of chemosensory receptors and the ultrastructural characteristics of chemosensory sensilla in mites and ticks, is of great interest for the development of novel plant-based repellent products and botanical acaricides^6^. Unlike insects, olfactory organs in mites and other arachnids are located exclusively on the legs^7^. The four pairs of walking legs are covered with specialized hairs, called sensilla, which protect sensory neurons from the external environment. Three major types of chemosensory sensilla have so far been characterized in arachnids, including the foreleg tarsi of the mites *Varroa destructor*^8,9^, *Dermanyssus prognephilus*^10^ and *D. gallinae*^11^. Sensilla chaetica are classified into different morphological types based on location, size, shaft morphology, structure of socket and the presence and location of pores: sensilla chaetica with or without a tip pore (Sc-tp) and sensilla chaetica with wall pores (Sc-wp). In contrast to wall-pore (wp) and tip-pore (tp) sensilla, non-pore (np) sensilla are considered to be mechanosensory functions to sense a mechanical distortion of the exoskeleton or seta^12^. These unique structures may advantageously allow for the coordination of mechano-, hygro-, thermo and chemoreception^13^. Even though the identification of structure and the internal arrangement of sensilla is an effective approach to judge their functions^12^, very few studies have been conducted in the acarines as a whole, and especially in mites^14^. Thus, detailed ultrastructural studies using scanning electron microscopy would improve our knowledge of PRM sensory biology dramatically.

The vast majority of receptors that detect chemosensory stimuli and convert ligand binding into neural activity belong to one of three membrane-bound receptor families: gustatory receptors (GRs), odorant receptors (ORs) and ionotropic receptors (IRs). While insects make use of all of these receptor families, non-insect arthropods rely on GRs and IRs for their chemical communication^15,16^. The GRs detect non-volatile compounds, including bitter and sweet compounds, salts and some gustatory pheromones, but also carbon dioxide in various insect species^17–20^. The IRs are involved in olfaction and gustation, but also have non-chemosensory functions, including detection of humidity and temperature^21^. Proteins from other gene families have also been shown to contribute to taste and olfaction. Members of the odorant binding protein (OBP) and chemosensory protein (CSPs) families in insects are highly concentrated in the sensillum lymph, and have been shown to bind odorant molecules^22,23^. OBP-like proteins have been well characterized in chelicerata, performing roles analogous to those of insect OBPs and CSPs^24,25^. Besides OBPs and CSPs, another potential chemosensory protein family, Niemann Pick type C2 (NPC2), has been identified in both insects and non-insect arthropods. Genes that encoded NPC2 proteins have undergone extensive duplication and functional differentiation that generating highly divergent sequences in mites and tick species^25,26^. NPC2 proteins have recently been reported in adult predatory mites, suggesting a putative role in detecting of the female sex pheromones^27^.Other proteins commonly expressed in the chemosensory system include sensory neuron membrane proteins (SNMPs), which are related to scavenger proteins of the CD36 family, and several classes of G protein-coupled receptors (GPCRs) which allow neurons to sense a variety of extracellular signals, including e.g. hormones and neurotransmitters^28^.

## Results

### Identification of sensilla on the legs of *D. gallinae*

**Figure 1:**
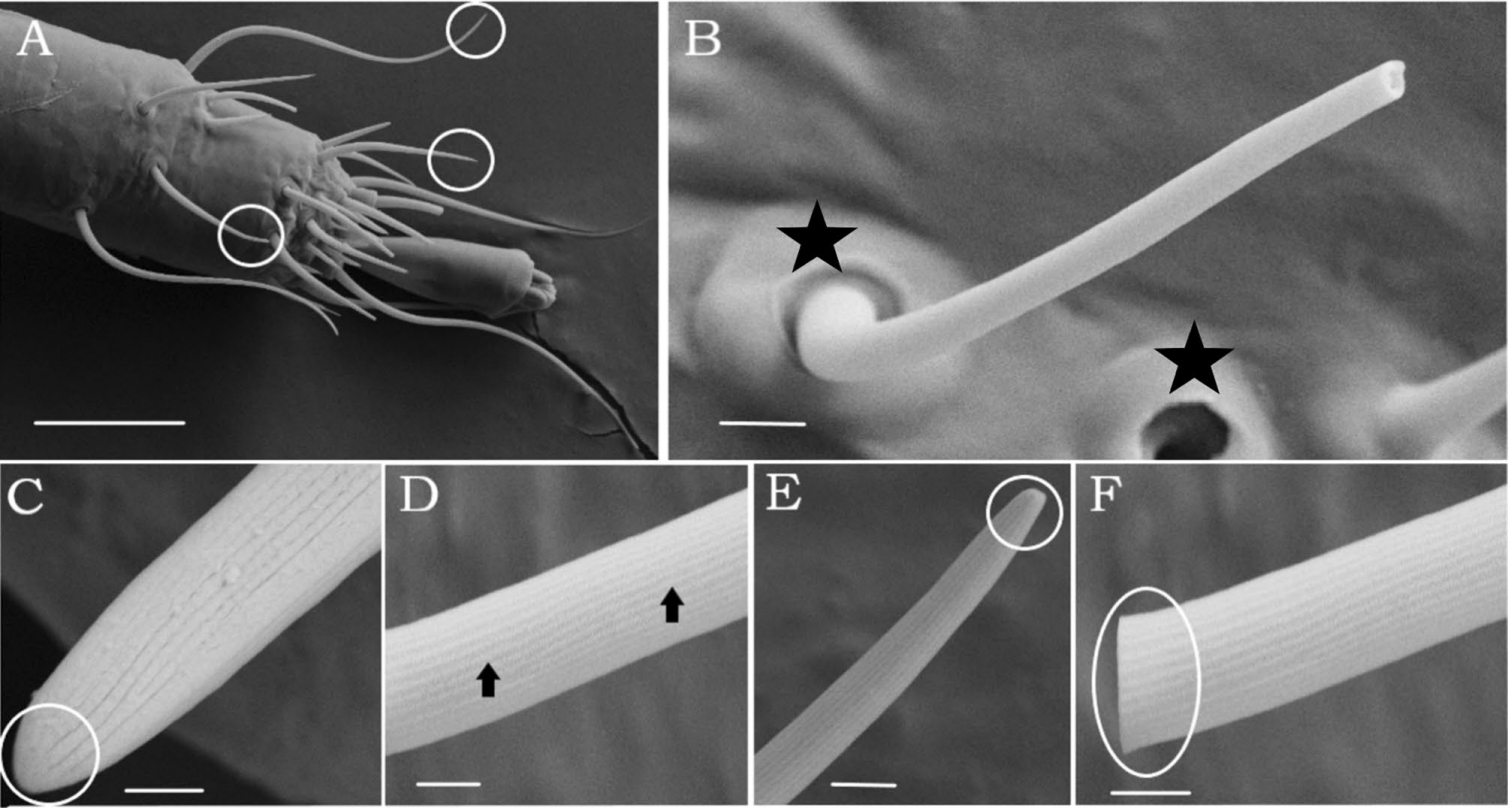
SEM of sensilla chaetica with or without tip-pore (Sc-tp). (**A**) Circles indicating sensilla chaetica with a tip pore or without pores on distalmost tarsomere of first leg. Different types of sensilla are not discriminated in this figure because of low magnifications. (**B**). Shaft broken in its apical part revealing a pore and structure of socket (white square) (**C**) No pore sensilla. (**D**) Lateral view of the Sc-tp displaying longitudinal grooves (arrows). (**E**) Tip-pore sensilla showed indistinct pore on tarsomere I (circle) F. Tip-pore sensilla showed visible tip pore on tarsomere III and delimited by a round margin (circle). *Scale-bars*: (**A**) 20 μm, (**B**) 2 μm, (**C**) 300 nm, (**D**–**F**), 1 μm.

**Figure 2:**
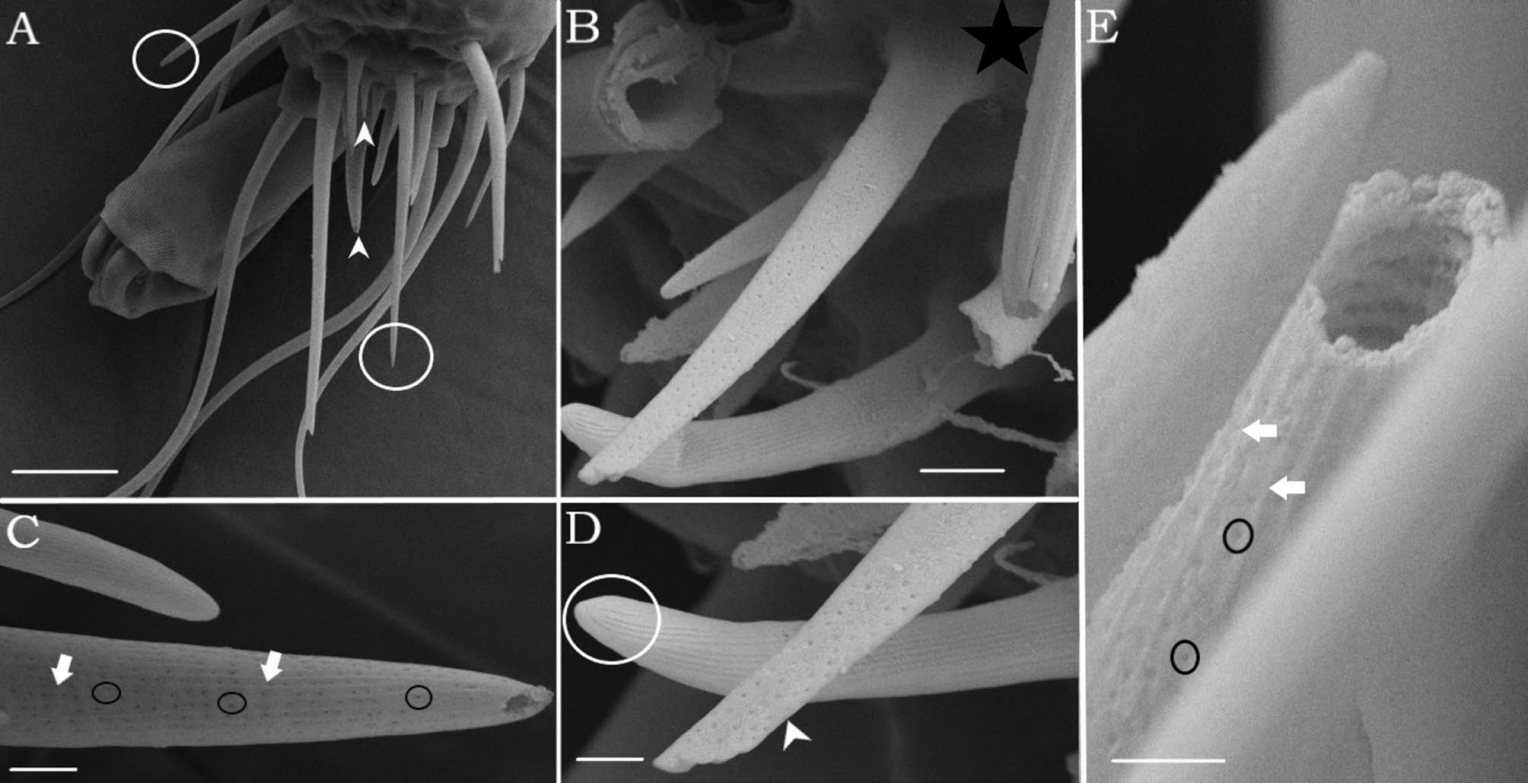
SEM of sensilla chaetica with wall-pores (Sc-wp). (**A**) Lateral view of different types of hair sensilla on the distalmost tarsus (DT-I), circles indicate Sc-tp and arrowhead indicate Sc-wp sensillum (**B**) Structure of socket with an incomplete articulation (white square) (**C**) Shaft with longitudinal ridges (white arrows) and numerous pores housed in longitudinal grooves (black circle) (**D**) Comparing tip pore (circle) and wall pore sensilla (white arrows) (**E**) White arrows represent longitudinal ridges, and circles indicate lines of wall pores. *Scale-bars*: (**A**), 10 μm, (**B**) 2 μm, (**C**) 1 μm, (**D**,**E**), 200 nm.

Using SEM, the forelegs and hindlegs of *D. gallinae* were analyzed, demonstrating a higher number of sensillum types on the anterior foreleg tarsi than on the posterior ones (Supplementary Figure S1). The Sc-tp, which was the longest sensillum type, displayed a regular distribution pattern on the tarsi (Fig. 1A). This sensillum type has a prominent socket and an articulating membrane (Fig. 1B), with the sensillum wall having continuous longitudinal ridges along the shaft, and the shaft being gradually tapered toward the tip (Fig. 1D,E). Even though tip pores on the Sc-tp of the distalmost tarsomeres (DT) I-II were indistinct in the SEM (Fig. 1E), tip pores on DT III-IV were delimited by a round margin (Fig. 1F). In contrast to Sc-tp, all (Sc-wp) were restricted to tarsi I (Fig. 2A), and their shafts were inserted in a shallow membranous cuticle with an incomplete articulation (non-socketed) (Fig. 2B). The shaft tapered toward the tip (Fig. 2C), and the thick wall contained a large number of evenly distributed pores (Fig. 2E).

### Mapping, transcriptome analysis and functional annotation

**Figure 3:**
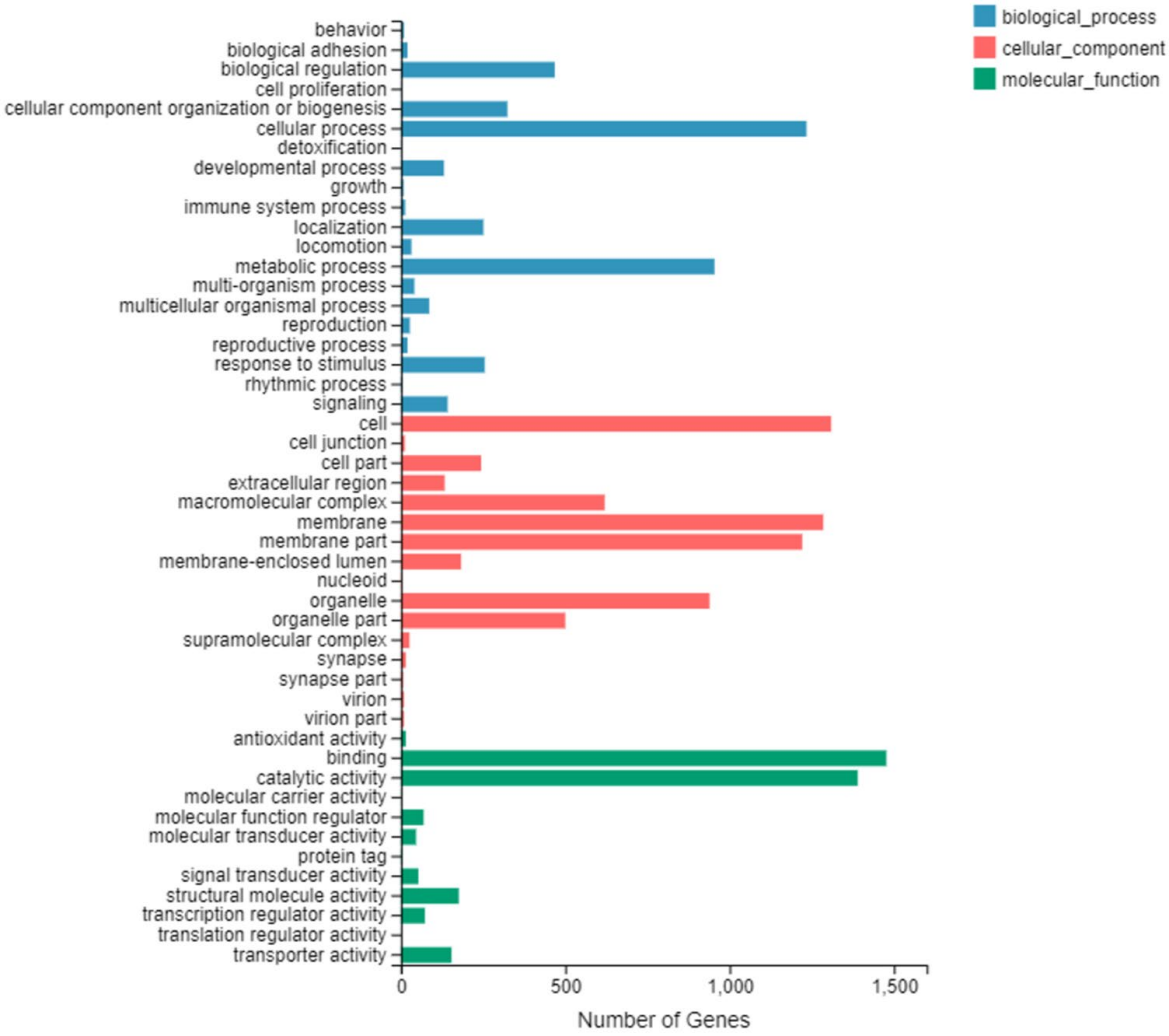
Gene ontology (GO) classification of differentially expressed genes. The X-axis indicates the number of unigenes in each category. The Y-axis represents three main ontologies: biological process, cellular component, and molecular function.

To identify olfactory genes, RNA extracts of PRM forelegs and hindlegs were sequenced separately using a BGISEQ-500 HiSeq platform. On average, 22.5 and 21.7 million raw reads were generated for the foreleg and hindleg samples, respectively. After quality control screening, 69.42% of the reads per sample mapped to the *D. gallinae* reference genome (Supplementary Table S1). While the resulting 25,604 sequences were identified as unigenes, these might not necessarily represent unique genes. The read sequences from the six transcriptomes were submitted to the sequence read archive (SRA) at NCBI under the accession number of PRJNA602095. Of the 25,604 unigenes, 14,203 (55.47%) showed significant similarity to genes encoding for known proteins in the NCBI non-redundant protein database (NR), whereas 10,285 unigenes (40.16%) were identified based on GO annotation. GO annotation was used to categorize annotated genes into functional groups based on three main categories: molecular functions, cellular components and biological process. Among the biological process terms, the most represented biological processes were cellular (1,234 unigenes) and metabolic processes (954 unigenes). In the cellular component terms, the genes expressed were predominantly cell (1,309 unigenes), membrane (1,285 unigenes), and membrane part (1,221 unigenes). In the molecular function category, binding (1,477 unigenes) and catalytic activity (1,390 unigenes) had a huge preponderance and were the most highly expressed categories (Fig. 3). In general, GO term enrichment analysis indicated that forelegs were mostly associated with binding, catalytic activity, signal transducer and transporter activity in the molecular function category.

### No gustatory receptors (GRs), olfactory receptors (ORs) and chemosensory proteins (CSPs) found in *D. gallinae* transcripts

BLASTx and BLASTn searches did not identify any transcripts putatively encoding for GRs and Ors, and CSPs in the *Dermanyssus* transcriptome. To further validate these findings, tBLASTn (*e*-value ≤ 1) and protein domain based searches did not identify any matches.

### Identification of ionotropic glutamate receptor (iGluR) families

**Figure 4:**
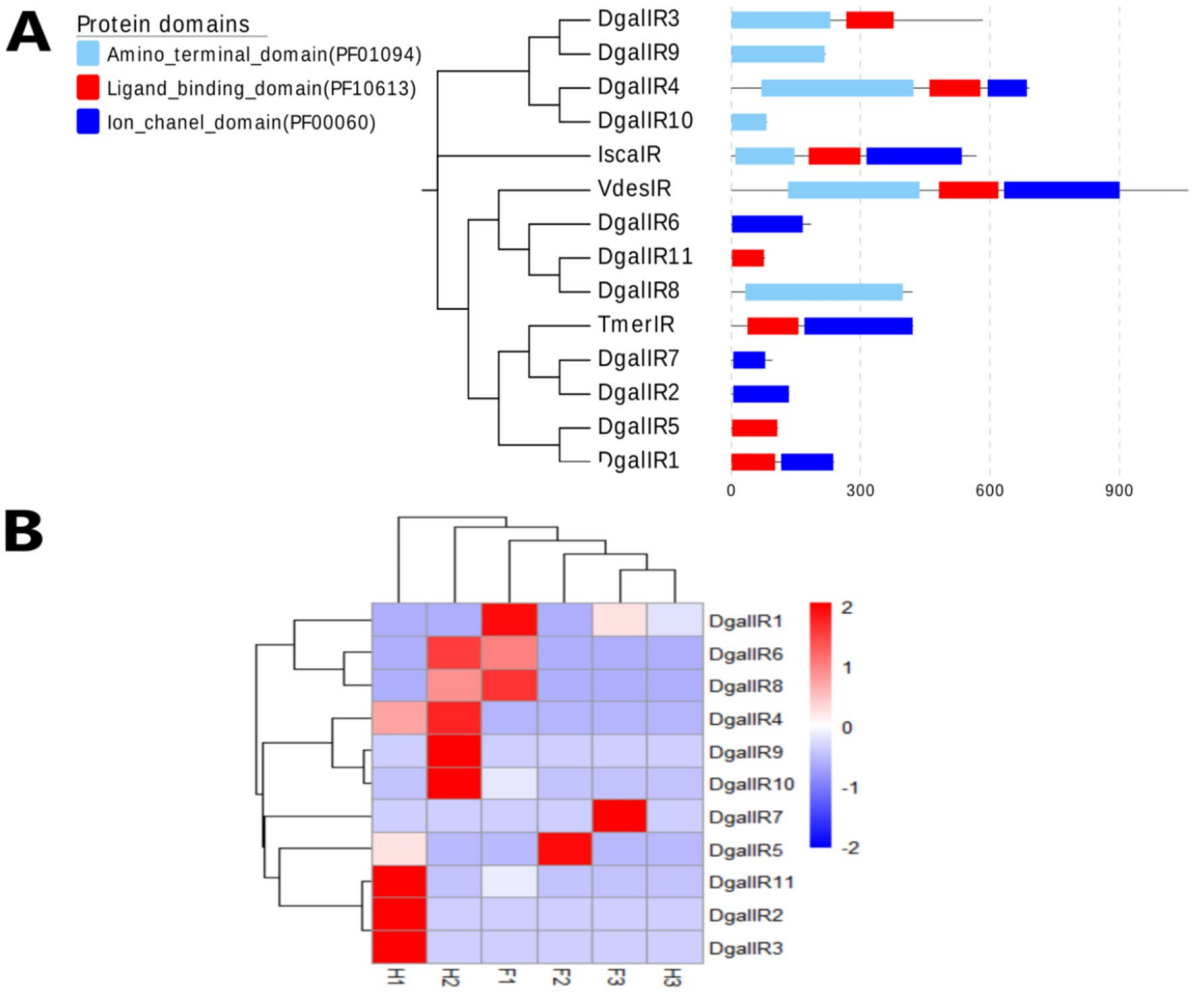
The protein domain organization of iGluRs/IRs and gene expression. (**A**) The protein domain organization of iGluRs/IRs in PRM is shown in histogram. Phylogenetic tree of the IR/iGluR family based on amino acid sequences of *D. gallinae* (DgalIR), *Tropilaelaps mercedesae* (TmerIR), *Varroa destructor* (VdesIR) and *Ixodes scapularis* (IscaIR) and the corresponding protein domains of each member. (**B**) Hierarchical clustering of the differentially expressed genes (DEGs). Blue to red colors represent gene expression levels (i.e., FPKM values from − 2 to 2).

Eleven transcripts encoding IR/iGluR homologs were identified, five of which contained the specific domain signature of the ionotropic glutamate receptors (Pfam domain-PF00060). Out of the eleven transcripts, only one transcript (DgalIR4) encoded all of the characteristic domains of the IR/iGluR proteins, namely, the amino-terminal domain (Pfam domain-PF01094), the ligand-binding domain (Pfam domain-PF10613) and the ion channel domain (Pfam domain-PF00060). The protein domain organization of iGluRs/IRs is shown in histogram (Fig. 4A). Among the IRs, all sequences showed complete ORFs, with > 112 amino acids. However, read counts for the identified IR/iGluR transcripts were low in both forelegs and hindlegs: in the forelegs, IR transcript expression levels ranged from 0.06 to 0.13 FPKM, and in the hindlegs from 0.01 to 1.12 FPKM. None of the identified IR/iGluR transcripts were significantly found in the forelegs (Fig. 4B). The phylogenetic analysis demonstrated that eight transcripts were clustered with the iGluR sub-group, and one gene was close to the NMDA sub-group (Supplementary Table S2). Another two identified transcripts from the *D. gallinae* formed a distinct sub-group along with a sequence of *V*. *destructor* mites, suggesting that a mite-specific IR sub-group is present. The IR phylogeny did not reveal any potential orthologous relationships with insect IRs, for example there were no relatives of the divergent class of insect IRs (Supplementary Figure S2).

### Identification of putative OBP-like and NPC2 proteins in *Dermanyssus* sequences

**Figure 5:**
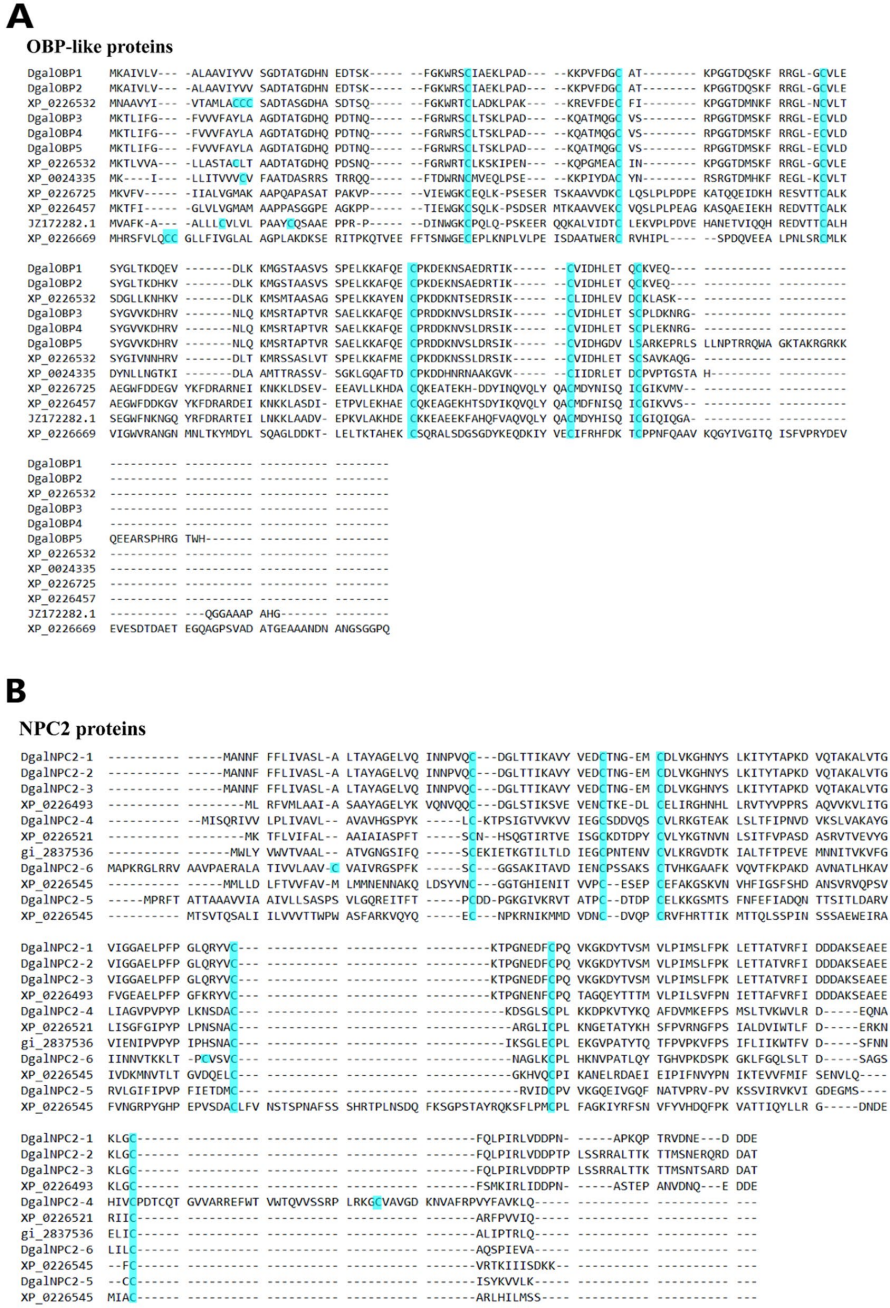
Alignment of the putative OBP-like and NPC2 sequences of PRM together with reference sequences. (**A**) Alignment of the five putative OBP-like sequences of PRM together with those of *V*. *destructor* sequences (XP_022653281.1; XP_022653293.1; XP_022645714.1; XP_022666940.1; XP_022672530.1), *Amblyomma americanum* (JZ172282.1) and *Ixodes scapularis* (XP_002433530.1). The highly conserved six cysteine (**C**) residues are shown in blue. (**B**) Alignment of the six putative NPC2 sequences of PRM together with those of *V*. *destructor* sequences. The highly conserved six cysteine (**C**) residues are shown in blue.

**Figure 6:**
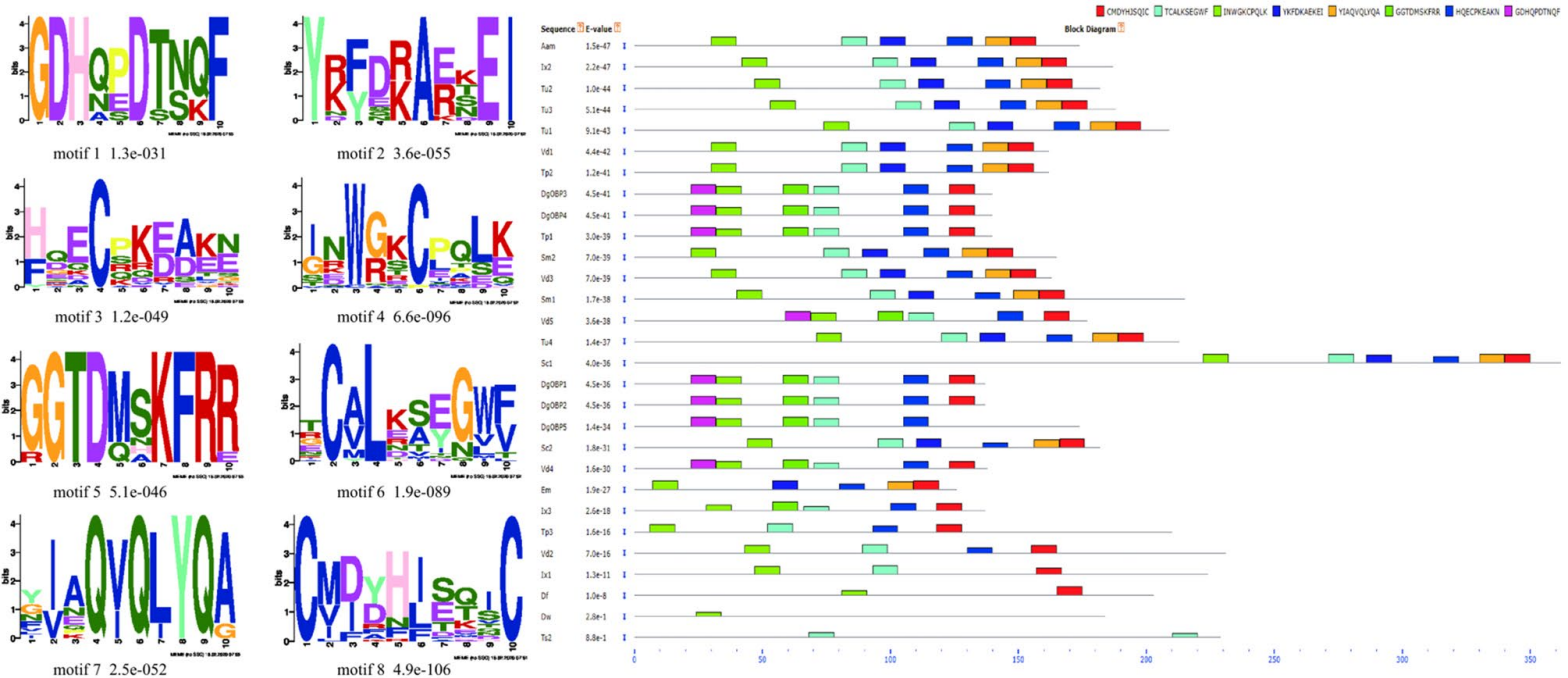
Motif-pattern analysis of OBP protein from *D. gallinae* and other classes including insecta and arachnida. The parameters setting in this study for motif predictions were: minimum width of motif = 6, maximum width = 10 and maximum number of motifs to find = 8. Motif-based sequence analyses were predicted by using web-based version 5.1.0 of the MEME server. Eight amino acid motifs with various widths and corresponding e-values were identified, and the lower part indicates approximate locations of each motif on the protein sequence. Different motifs are shown by different colored boxes, where small number indicate high conservation.

A total of five and six candidate OBP- and NPC2-encoding genes were identified, respectively, in the transcriptomic analysis of *Dermanyssus* chemosensory tissues. The complete ORFs were identified for five OBP candidate genes, revealing > 137 amino acids encoding for proteins with high homology to known OBP-like proteins from other mite and tick species. SignalP revealed an 18-amino-acid-long signal peptide cleaved between position 18 and 19. Alignment of amino acid sequences from these species revealed that all OBP-like sequences contain six conserved cysteine (C) residues (Fig. 5A). Two OBP-like unigenes (DgalOBP1 and DgalOBP2) revealed higher levels of expression in forelegs, whereas three genes (DgalOBP3, DgalOBP-4 and DgalOBP-5) had expression in both forelegs and hindlegs (Supplementary Figure S3B). The phylogenetic analysis showed that all of *Dermanyssus* OBPs were clustered together on the same subclade with high bootstrap support (Supplementary Table S3). In addition, *Dermanyssus* OBPs were clustered with a clade containing OBPs of the mites *V. destructor* and *Tropilaelaps mercedesae*, with more than 80% branch support (Supplementary Figure S3A). Conserved OBP motifs were predicted to better understand the protein’s evolution and function. Since a high number of OBP genes have been reported in both insects and arachnids, we performed a motif-pattern analysis between these two classes (Supplementary Table S4). Only motif 1 (CMDYHJSQIC) and motif 2 (TCALKSEGWF) encoded the OBP family were present in all the OBP orthologs except for *Trichomalopsis sarcophagae* (Ts2), *D. willistoni* (Dw) and *D. ficusphila* (Df) (Fig. 6). Since motif 1 and 2 were found in all OBP proteins including the five *Dermanyssus* OBPs, the results provide confidence of their identification as bonafide OBP-like encoding genes and infer a functional similarity with other OBPs.

Six NPC2 proteins were annotated from the *D. gallinae* transcriptomes. These putative NPC2 genes consisted of complete ORFs with lengths ranging from 471 to 591 bp nucleotides, and all sequences had an N-terminal signal peptide that is a common feature of the secretory proteins. To compare sequence similarity with other acari, a multiple sequence alignment showed that all six cysteines were conserved in all NPC2 proteins (Fig. 5B). Phylogenetic analysis of the NPC2 genes revealed three groups of *D. gallinae* NPC2 genes, with two groups being unique to arachnida (Supplementary Figure S4A; Table S5). The differential expression of the chemosensory-related proteins between the forelegs and hindlegs comparisons was less prevalent (Supplementary Figure S4B). The conserved motifs are important elements of functional domains. A total of 32 sequences from both insects and arachnids including six *Dermanyssus* NPC2 were used to compare and search for shared motif patterns (Supplementary Table S6). The results showed that motif 1 (CPLKKGKDYT) and motif 2 (PFPGPKSDAC) were present in all the NPC2 orthologs. In addition, the homologous NPC2 from different acari species had similar motif patterns (Supplementary Figure S5).

### Identification of candidate sensory neuron membrane proteins (SNMPs)

Five candidate SNMPs transcripts were identified based on similarity to known SNMPs, within the CD36 superfamily (PF01130.21), of mites, ticks and insects. None of the identified transcripts were significantly found in the forelegs (Supplementary Figure S6B). To extend and specify this classification, the constructed phylogenetic tree revealed that acarian SNMP sequences were clearly separated from those of insects with the exception of three SNMPs (TrpWZC0, Var27012 and IscPD82) that showed proximity to the *D. melanogaster* clusters (Supplementary Figure S6A, Table S7).

### Identification of candidate G protein-coupled receptors (GPCRs)

**Figure 7:**
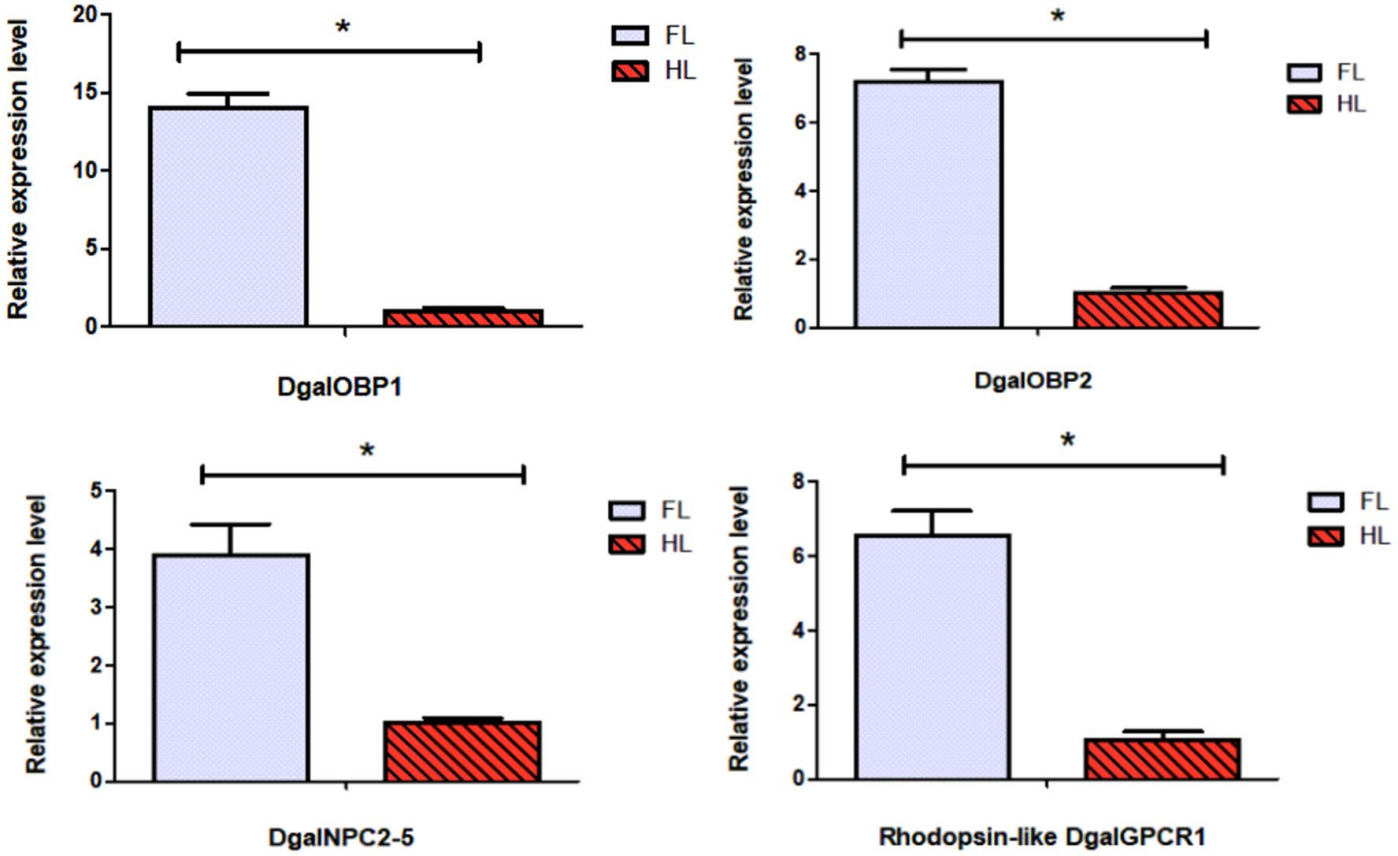
Expression profiles of putative chemosensory genes in *D. gallinae* using qRT-PCR. FL, forelegs; HL, hindlegs. Error bars represent means ± SEM, and asterisks above indicate significant differences between forelegs and hindlegs (*p < 0.05).

One putative GPCR was identified exclusively in the transcriptome of forelegs and contained a rhodopsin-like domain (PF00001.21). Protein domain and phylogenetic analyses of the putative GPCR transcript revealed that the transcript belongs to the putative clade A, rhodopsin-like GPCRs showing GPCR and photoreceptor activity (Supplementary Figure S7, Table S8). We also found a foreleg-biased expression of a G protein, and expression of this was verified by RT-PCR (qPCR) (Fig. 7).

### Differentially expressed gene (DEG) validation using qRT-PCR

In order to verify the differentially expressed gene identified through the transcriptome analysis, the expression pattern of identified gene was analyzed using qRT-PCR, including 2 OBPs (DgalOBP1, DgalOBP2), 1 NPC2 (DgalNPC2-5) and 1 GPCR (DgalGPCR1). The expression levels were largely consistent with the transcriptome profile analyses concluding that the RNA-seq data were reliable (Fig. 7). RNA-seq expression data are presented in supplementary file (Supplement 3).

## Discussion

Compared to other arthropods in general and to ticks in particular, the molecular basis of chemoreception in mites is largely unexplored. Most arachnids including PRM are active at night and thus do not primarily depend on visual sense. Their most important sensory input comes from mechanical stimuli such as air currents, touch and vibrations. To better understand how mites perceive olfactory chemical cues, chemosensory genes were identified using transcriptomic analysis of *D*. *gallinae*, along with an ultrastructural study of sensilla. Despite genes encoding chemosensory proteins and the ultrastructure of mite sensilla have been described, the association between those two elements have not been examined. Our results suggest that the forelegs are equipped with sensilla containing neurons with chemo- and mechanosensory related functions. In general, an improved understanding of the olfactory system may provide a good start of controlling this problematic mite.

Three types of setiform sensilla were distinguished, which is typical for all acari: no pore (np), wall-pored (wp) and tip-pored (tp) sensilla. Although each sensillum type has a characteristic ultrastructure, functional properties can only be established with certainty by data from physiological recordings, which we so far lack. Wall-pore sensilla are, in general, considered to have an olfactory function^12^. The exclusive presence of wp sensilla on the distal-most tarsomeres of the front legs indicate that these are important sensory (Fig. 2) appendages also present in ticks (Haller’s organ) and other mite species^13,29,30^. Moreover, the presence of tp sensilla suggests a contact-chemoreceptive (gustatory function) and contact-mechanoreceptive function (Fig. 1), as previously described in other arachnids. These results are consistent with ticks and other types of arachnids^13,31^.

In forelegs, four molecular function categories might be connected to olfaction (odorant binding), enzyme activity (catalytic) and signal transduction (signal transducer activity) (Fig. 3). These results are likely to reflect that forelegs play an important chemosensory role. Our study did not identify any transcripts putatively encoding gustatory receptors, despite well-documented morphological evidence of gustatory-like tp sensilla. This result might reflect that the transcriptome sequencing depth, method and number of samples were not sufficient, and therefore, further research is needed to validate this result. Another striking difference was the small number of chemosenory genes in PRM compared with other homologous species (Supplementary Figure S8), which possibly indicates differences in their ability to discriminate volatile chemical compounds and communicate with conspecifics. This difference may have evolved as a result of very different nesting behaviours, feeding habits and host-specificity of the poultry red mite. For example, most endoparasitic mite species do not actively seek out their hosts. Transmission occurs through close or direct physical contact. The loss of active host-seeking behavior might thus have resulted in a reduction both in number of genes involved in chemosensation and in sensillum numbers. Both commonly observed in species that transition from ectoparasites to endoparasites^7^.

OBPs have recently been reported in some chelicerata, demonstrating that these proteins are structurally reminiscent of insect odorant binding proteins, and therefore named OBP-like. These proteins were first described in the tick *Amblyomma americanum* and the honey-bee mite *V. destructor*, suggesting a likely involvement in acari chemodetection^24,30^. These secreted proteins have six cysteine amino acid residues as is also observed in the classical OBP family. The arrangement pattern of six conserved cysteines in the *D. gallinae* OBP family is comparable to the patterns in other OBP-like proteins of chelicerata (Fig. 5). A major finding of our study is the identification of transcripts encoding OBP-like soluble proteins, some of which were highly and differentially expressed in the forelegs. This was also evident in *Ixodes scapularis* ticks, where some of the OBP-like transcripts were highly expressed in the forelegs^32^. Phylogenetic analysis revealed that all *Dermanyssus* OBPs were clustered within arachnida only, indicating that this branch evolved after the splitting of insects and arachnids. The continued annotation of OBP genes from chelicerate genomes will likely clarify this issue. In addition, soluble proteins of a different class (NPC2), which was also detected in the *varroa* proteomic project, have been suggested to function as semiochemical transporters in chelicerates^24,26^. This study found that NPC2 was expressed not only in chemosensory sensilla, but also in other parts of the body, suggesting roles in both chemical detection as well as in other functions. The results of this study were consistent with other studies in arthropods, which showed wide expression of NPC2 proteins across the body^33,34^.

Regarding CSPs, we were unable to detect gene expression of chemosensory proteins. This finding is in agreement with previous studies in ticks (*Amblyomma americanum*) and mites (*V*. *destructor*) species based on genomic and transcriptome data^24,30^. However, the CSPs have been reported in the stored‐food mite *Tyrophagus putrescentiae*^35^. In the transcriptome analysis we also identified sensory neuron membrane proteins (SNMPs), which have key functions in pheromone detection in insects. The role of SNMPs in chemoreception in acari is still unknown. Similarly, there is little information available on the identified GPCRs, and the molecular characterization of most GPCRs remains unknown in acari. Identification of G proteins are also relevant for the understanding of acari biology in general, as these proteins could be targeted for next generation pesticides. Most information on acari GPCRs were obtained from tick transcriptome datasets and recombinant receptor systems^36^. Previous experiments in ticks indicated that prediction of GPCRs using transcriptome analysis might be a challenging task. As such, the transcriptome study of the bovine tick *Boophilus microplus* did not allow to detect the kinin receptor, a neuropeptide GPCR^37^, which should be present^38^, underscoring the often low expression of GPCRs as a challenge for detection. Likewise, only two G proteins were identified in *I. scapularis* ticks and all were expressed at low to non-existent levels in legs^32^. All of these findings acknowledge limitations in the prediction of tick GPCRs. In agreement with this, only one GPCR transcript was detected in the present study. In addition, the presence of putative rhodopsin-like GPCR transcripts, having a foreleg bias expression in *Dermanyssus* mite, might code for olfaction. A similar finding was observed in the American dog tick *Dermacentor variabilis* with foreleg-biased expression of GPCRs, suggesting that tick olfaction via the Haller’s organ involves a GPCR-mediated pathway like that of vertebrates and nematodes^29^. In crustaceans, like other non-insect arthropods, GPCRs might be possible candidates for chemoreceptor proteins^39^. We thus suggest that mite olfactory receptors might represent a completely novel type of 7-transmembrane receptor (7TM) family proteins that have yet to be identified.

## Conclusions

The chemosensory genes reported here represent a significant contribution to the pool of identified olfactory genes in non-insect arthropods in general. Mites constantly move their first pair of legs in the air in the manner of the antennae of insects, along with ultrastructural features of the sensory organs, and the results of transcriptomic analyses are all strongly in favour of the assumption that the forelegs serve an olfactory function. These results highlight the importance of an integrative approach in systematic studies, combining morphological and molecular characteristics, previously not being conducted in other mite and tick species. This finding could potentially contribute to find new strategies for controlling this pest.

## Materials and methods

### Mites collection and scanning electron microscope (SEM)

*D. gallinae* was a kind gift from Professor Pan, China Agricultural University, China. Upon arrival, mites were kept in vivo using a rearing system by feeding on chickens, as previously described^40^. The care and use of chickens in this study were approved by the Hainan University Institutional Animal Care and Use Committee. We also confirmed that all experiments were performed in accordance with institutional guidelines and regulations. Mites identification to the species level were carried out using a scanning electron microscope in accordance with the morphological keys of Moss and Di Palma^41,42^. Sample preparation for SEM examination has been previously described^31^. Briefly, specimens were cleansed with ultrasonic cleaning solution (Liquinox^®^) using an ultrasonic cleaner (Digital ultrasonic cleaner^®^). Samples were subjected to supercritical drying (Quorum^®^ K850) with CO_2_ after being dehydrated in a graded series of ethanol (from 45 to 100%). Dehydrated mites were fixed on stubs with double sided conductive carbon adhesive tabs and then sputter-coated with gold using Anhui Beq SBC-12 coating apparatus. Morphological analyses and photographs were done with a GeminiSEM microscopy at the HU, China.

### Total RNA isolation, cDNA library preparation, bioinformatics and differentially expressed genes (DEGs) analysis

Total RNA extracted from the forelegs and hindlegs of *D. gallinae* mites were used for RNA sequencing. A total of 150 adult mites (300 forelegs and 300 hindlegs) were dissected by using a scalpel blade (size no. 10) under a dissecting microscope, and three biological replicates per group were generated. RNA was extracted from each group using Trizol^®^ reagent, according to the manufacturer’s protocol. High-quality samples were subsequently sequenced at the Beijing Genome Institute (BGI-Shenzhen, China), using a BGISEQ-500 platform. For generating first-strand cDNA libraries, RNA was purified using DNA polymerase I and deoxyribonucleotide triphosphate (dNTPs), following RNase H treatment. Then, the cDNA was generated using superscript III reverse transcriptase with random hexamer primer sets. A cDNA library was sequenced on a BGISEQ-500 platform. Following sequencing, clean reads were obtained by removing those containing adapters or unknown nucleotides at greater than 5% and of low-quality reads from the dataset. All clean reads were converted to the FASTQ file format. All clean reads were converted to the FASTQ file format. All of the downstream analyses were based on clean data with high-quality reads^43^. The reference transcriptome of *D. gallinae* was obtained from NCBI (GAIF00000000)^44^ and used for building the index by HISAT2. The generated index files were used to align the clean reads of the six RNA-seq samples to the reference genome. Bowtie2 was used to compare clean reads to reference sequences, and RSEM was used to calculate gene expression levels for each sample^45–47^. Gene expression levels were estimated based on the FPKM value (fragments per kilobase of exon per million fragments mapped) using RSEM (RNA-Seq by expectation–maximization) method with default parameters. Differential expression analysis of chemosensory genes in forelegs versus hindlegs were performed by the DEseq2 R package. The p-values were adjusted using q-values, with q-value ≤ 0.001 and log_2_ (fold change) ≥ 2 set as the threshold for significantly differential expression using the Benjamini and Hochberg method^48,49^. Differentially expressed genes (DEGs) were cut-off by a false discovery rate (FDR) at 0.05, and then a gene ontology (GO) term enrichments were performed using Blast2GO software^50^.

### Identification, annotation and phylogenetic analysis of chemosensory-related genes

Chemosensory-related genes from the transcriptomes of *D. gallinae* were identified by the functional annotation results based on its molecular function, cellular component and biological process. Standalone tblastn searches (BioEdit software 5.0.6) were performed using previously identified olfactory sequences of IRs, GRs, GPCRs, OBPs, NPC2 and SNMPs from other mite and tick species as queries in order to identify additional candidate chemosensory gene families^51,52^. All candidate gene families were manually verified using the BLASTx program against non-redundant protein databases available at the NCBI with a cut-off e-value < 10^−6^. The open reading frames (ORFs) of all candidate genes were determined by using the open reading finder at NCBI. Thereafter, all transcripts with BLAST hits was included in an InterProScan tool search available at the EMBL-EBI^53^. The presence of putative N-terminal signal peptides were predicted using the SignalP4.0 server, and the transmembrane domains were predicted using the TMHMM2.0 server. In addition, olfactory soluble proteins, such as OBP and NPC2 protein sequences from the arthropod that include insects, mites, ticks were used for motif-pattern analysis^54^. We have also tested signature patterns of cysteine (C) residues on the Swiss-Prot to see if the signature pattern was specific to the group of family of proteins. (“C1-X16-C2-X18-C3-X37-C4-X15-C5-X9-C6” where X represents any amino acid; prosite pattern notation, https://prosite.expasy.org/prosuser.html#conv_pa). All candidate chemosensory unigene names were represented as follows: first letter of the genus and first three letter of species (e.g. *D*. *gallinae*-Dgal) followed by a member of one of these protein families (e.g. IR, NPC2, OBP). For the qualitative report of gene family transcripts, the translated ORFs of *D. gallinae* chemosensory transcripts that contain the relevant conserved domains were retrieved and used to construct phylogenetic analysis. Amino acid sequences for each gene family were aligned using MAFFT in the “Auto” strategy method with BLOSUM62 scoring matrix and other default parameters^55^. The maximum-likelihood (ML) trees were constructed with PhyML using the best-fit substitution model WAG + G + I as determined by ProtTest 2.4. Branch support was estimated using a fast and accurate maximum likelihood-ratio test (aLRT) and subsequently edited in iTOL webserver^56–58^. The species maximum-likelihood phylogenetic tree was conducted based on the alignment result of mitochondrial cytochrome c oxidase genes (CO1) from different species using the software in MEGA7.0^59^.

### Quantitative real-time PCR validation for differentially expressed genes (DEGs)

A total of 4 chemosensory-related transcripts identified by RNA-seq to be expressed deferentially between forelegs and hindlegs were selected for real-time quantitative PCR (qPCR) analysis. RNA extraction was performed as mentioned above. Gene-specific primer sets were designed based on the ORF, and Fun14-like gene was used as housekeeping gene^60^. The primers were designed from the transcriptome-derived nucleotide sequences using Primer3 v.0.4.0. The primers used for real-time PCR are listed in Supplementary Table S9. The thermal cycle conditions were as follows: 96 °C for 8 min, followed by 40 cycles of 96 °C for 5 s, 56 °C for 15 s and 71 °C for 15 s. Following the real-time PCR cycles, the specificity of the SYBR green PCR signal was confirmed by melting curve analysis and agarose gel electrophoresis. Relative quantification data were analyzed using the comparative 2^–ΔΔCt^ method with three biological repeats each containing three technical replicates^61^.

### Data analysis

The data were analyzed using prism 5.01 software, and the values are represented as means ± standard errors of means (SEM) of three biological replicates. The differences between two groups were analyzed using t-tests. Roche LightCycler® 96 System with analysis software was used for qPCR data normalization and quantification. Differences were considered to be significant at (*) p values of < 0.05.

## Supplementary information

Supplementary Information 1.

Supplementary Information 2.

Supplementary Information 3.
