# Supplementary material for: Comparative morphological and transcriptomic analyses reveal chemosensory genes in the poultry red mite, Dermanyssus gallinae
Source: Sci Rep. 2020 Oct 21;10:17923. doi: 10.1038/s41598-020-74998-7 (PMC7578799; doi:10.1038/s41598-020-74998-7)
Supplement: Supplementary file 1 — Supplementary Information 1. [file 41598_2020_74998_MOESM1_ESM.pdf]

Comparative morphological and transcriptomic analyses reveal chemosensory genes in the poultry red mite, *Dermanyssus gallinae*

Biswajit Bhowmick[a], Yu Tang[a], Fang Lin[a], Øivind Øines[b], Jianguo Zhao[a], Chenghong Liao[a], Rickard Ignell[c], Bill S. Hansson[d] and Qian Han[a*]

[a]Laboratory of Tropical Veterinary Medicine and Vector Biology, School of Life and Pharmaceutical Sciences, Hainan University, Haikou, Hainan 570228, China.

[b]Norwegian Veterinary Institute, Ullevaalsveien 68 P.boks 750 Sentrum, 0106 Oslo, Norway.

[c]Disease Vector Group, Unit of Chemical Ecology, Department of Plant Protection Biology, Swedish University of Agricultural Sciences, Alnarp, Sweden

[d]Department of Evolutionary Neuroethology, Max Planck Institute for Chemical Ecology, 07745, Jena, Germany.

*Correspondence: Qian Han Email: qianhan@hainanu.edu.cn

Supplementary Materials-1

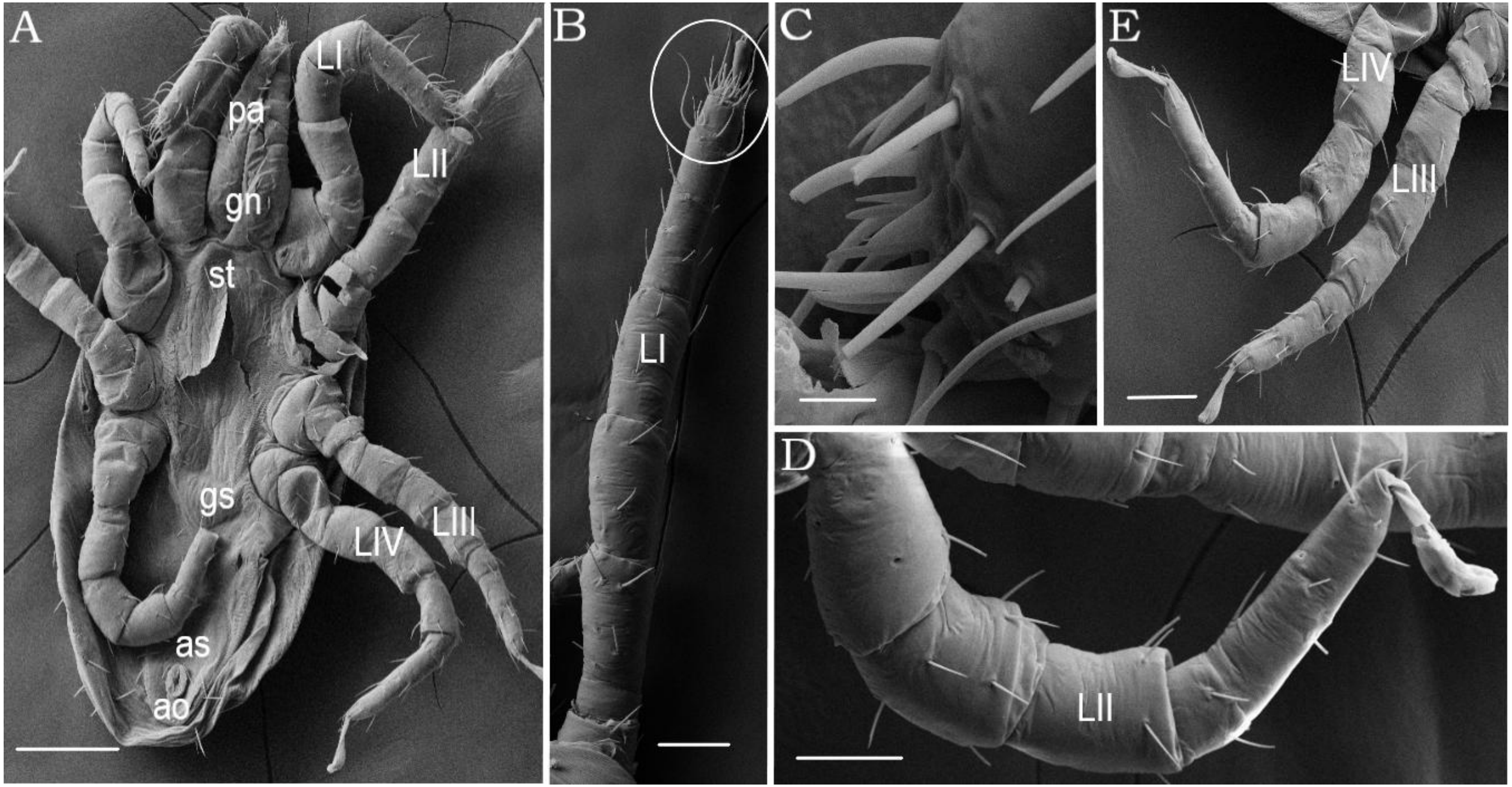
A
pa
LI
LII
gn
st
gs
LIV
LIII
as
ao
B
LI
C
E
LIV
LIII
D
LII

Figure S1. Scanning electron micrographs of PRM. A Ventral view of an adult PRM. B The first leg (LI), indicating the distal sensilla-rich structure (circle). C Different types of sensilla on the distal-most tarsomere (DT-I) of LI. D The second leg (LII). E The third leg (LIII) and fourth leg (LIV). Different types of sensilla are not discriminated in this figure because of low magnifications. *Scale-bars*: A, 100 µm B, D, E, F, 20 µm, C, 2 µm. *Abbreviations*: st, sternal plate; gn, gnathostoma; as, anal shield; ao, anal opening; ch, chelicera; Pa, pedipalp; (LI–IV), leg; gs, genitoventral shield

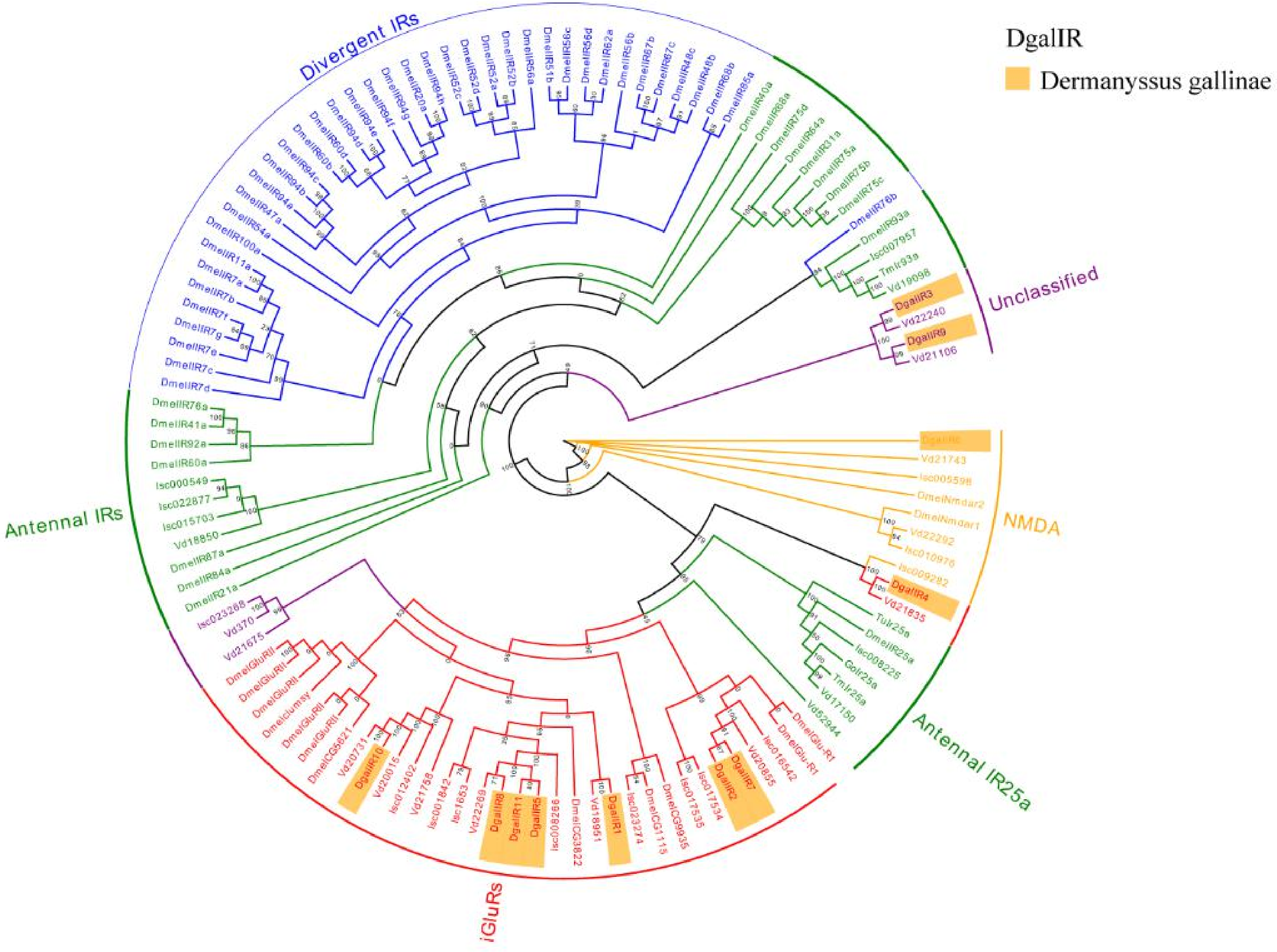
DgalIR
Dermanyssus gallinae
Divergent IRs
Unclassified
NMDA
Antennal IRs
Antennal IR25a
iGluRs

Figure S2. Phylogenetic tree of putative *D. gallinae* iGluRs and IRs along with insect and non-insect arthropods iGluRs and IRs. To further distinguish putative IR gene families from the transcriptome of *D. gallinae*, all of the IRs in our transcriptomes were aligned with IRs from *Varroa destructor*, *Tetranychus urticae*, *Galendromus occidentalis*, *Ixodes scapularis*, *Tropilaelaps mercedesae* and *Drosophila melanogaster* by MAFFT. The *D. gallinae* genes are shown in light orange. The amino acid sequences and a comprehensive list of acronyms are listed in Supplementary file 2, Table S2.

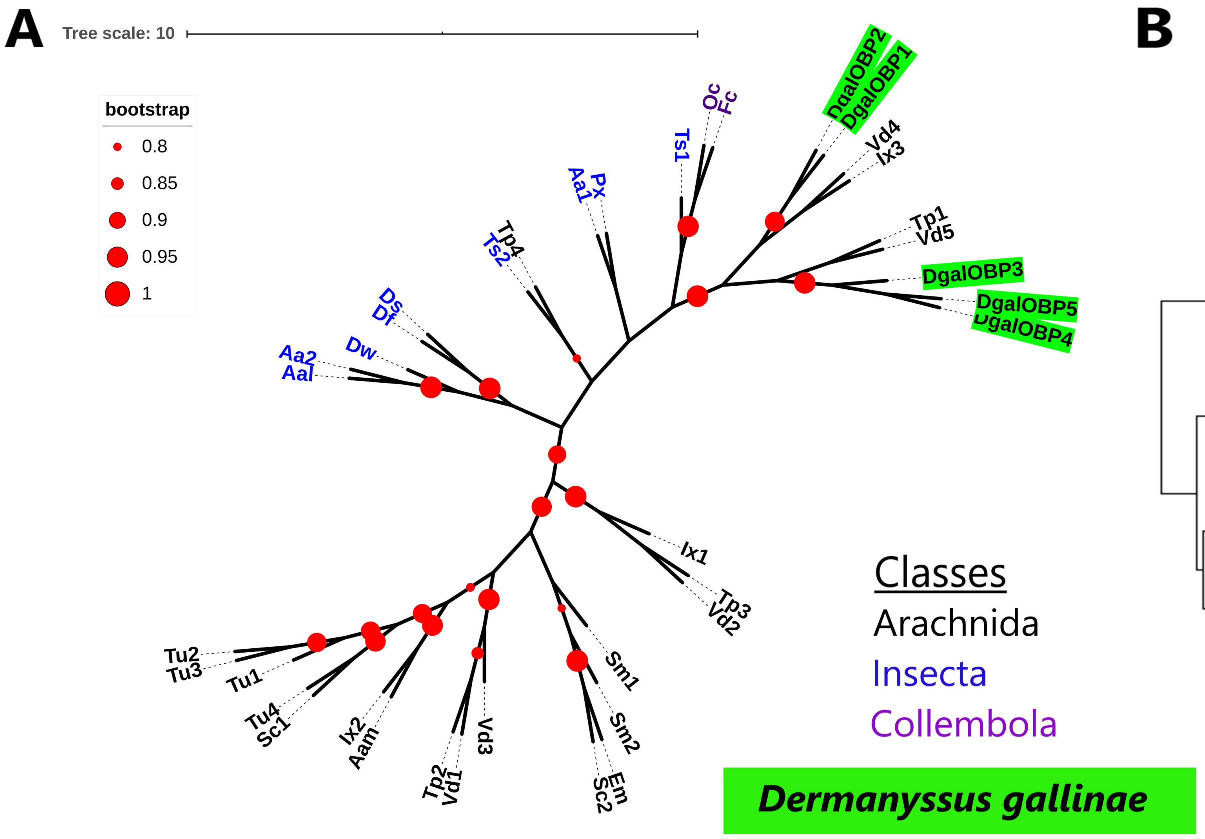

A
Tree scale: 10
bootstrap
0.8
0.85
0.9
0.95
1
DgalOBP2
DgalOBP1
Vd4
Ix3
Tp1
Vd5
DgalOBP3
DgalOBP5
DgalOBP4
Oc
Fc
Ts1
Px
Aa1
Tp4
Ts2
Ds
Df
Dw
Aa2
Aal
Ix1
Tp3
Vd2
Tu2
Tu3
Tu1
Tu4
Sc1
Ix2
Aam
Tp2
Vd1
Vd3
Sm1
Sm2
Em
Sc2
Classes
Arachnida
Insecta
Collembola
Dermanyssus gallinae

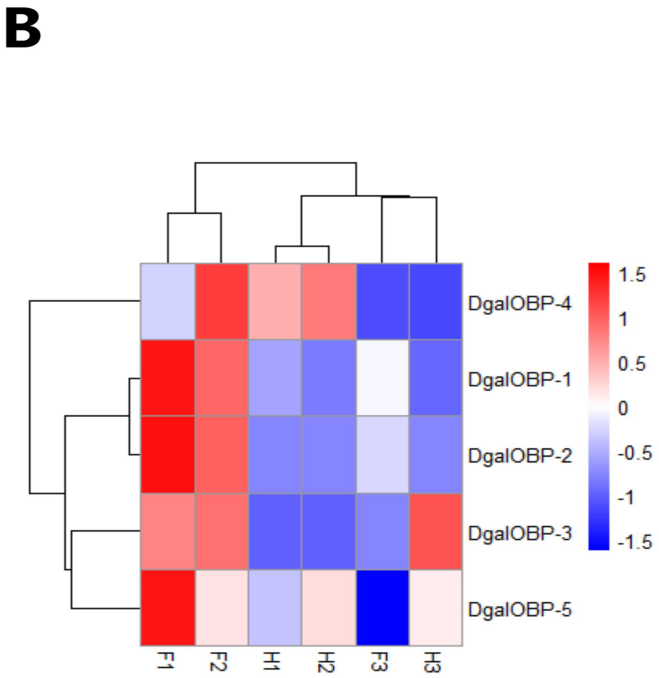

B
DgalOBP-4
DgalOBP-1
DgalOBP-2
DgalOBP-3
DgalOBP-5
F1
F2
H1
H2
F3
H3
1.5
1
0.5
0
-0.5
-1
-1.5

Figure S3 Phylogenetic unrooted tree of odorant-binding protein (OBPs) and hierarchical clustering analysis of the differentially expressed genes (DEGs). (A) Phylogenetic unrooted tree of odorant-binding protein (OBPs). Included are OBPs from *D. gallinae* (Dg: 5), *V. destructor* (Vd: 5), *I. scapularis* (Ix: 3), *A. americanum* (Aam: 1), *T. mercedesae* (Tp: 4), *Stegodyphus mimosarum* (Sm: 2), *Tetranychus urticae* (Tu: 4), *Sarcoptes scabiei* (Sc: 2), *Trichomalopsis sarcophagae* (Ts: 2), *Aedes albopictus* (Aal: 1), *Aedes aegypti* (Aa: 2), *Papilio xuthus* (Px: 1), *Folsomia candida* (Fc: 1), *D. sechellia* (Ds: 1), *D. ficusphila* (Df: 1), *Euroglyphus maynei* (Em: 1), *Orchesella cincta* (Oc: 1) and *D. willistoni* (Dw: 1). Leaves are coloured according to the organisms classes: arachnida (black), collembola (purple) and insecta (blue). *Dermanyssus* transcripts are highlighted in green.The amino acid sequences and a comprehensive list of acronyms are listed in S3. (B) Hierarchical clustering analysis of the differentially expressed genes (DEGs). Blue to red colors represent gene expression levels (i.e., FPKM values from −1.5 to 1.5). To clearly describe the DEGs of each olfactory-related family, we performed cluster analysis using a pheatmap package in R software based on FPKM-values, represented as a heatmap.

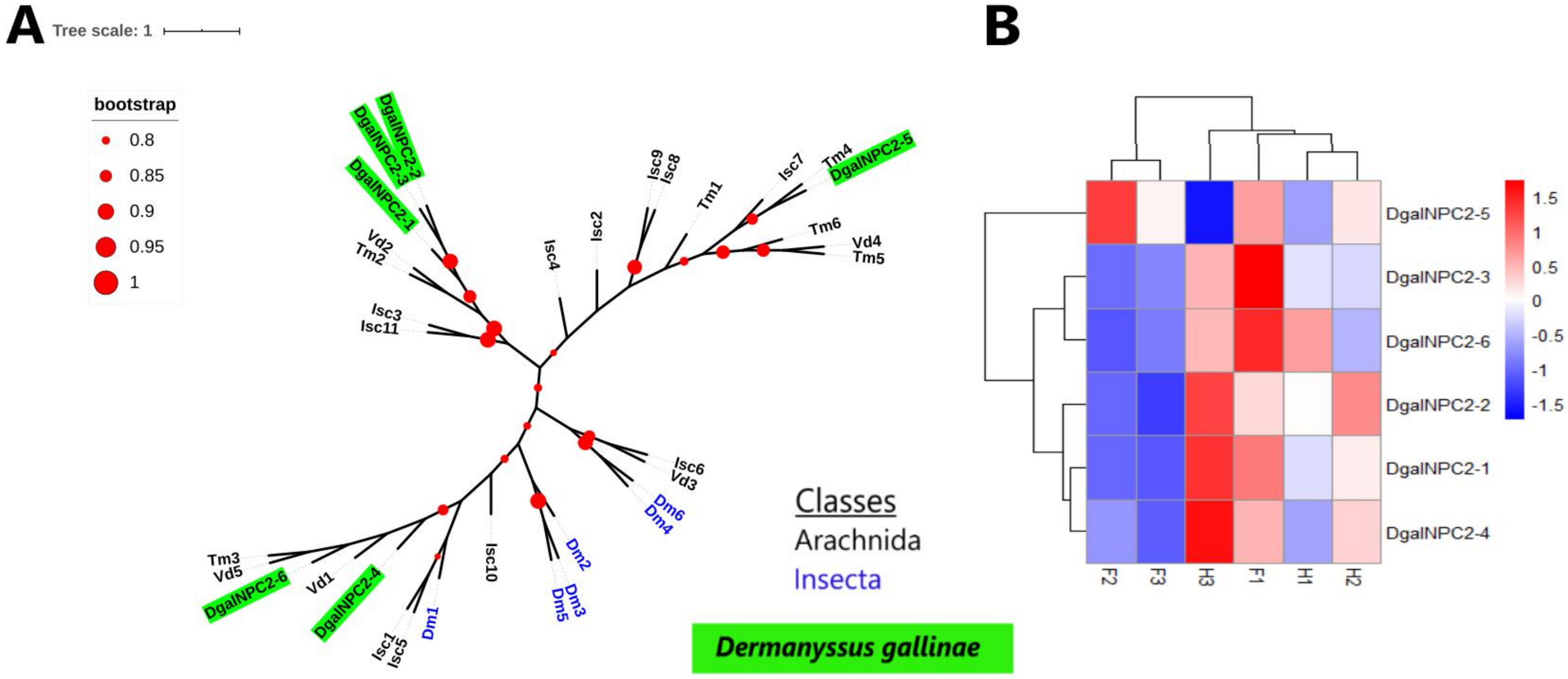

Figure S4. An unrooted phylogenetic tree of NPC2 proteins and hierarchical clustering analysis of the differentially expressed genes (DEGs). (A) An unrooted phylogenetic tree of NPC2 proteins. The evolutionary tree is constructed by aligning the NPC2 sequences of *V. destructor* (Vd:

6), *Dermanyssus gallinae* (Dg: 6), *Drosophila melanogaster* (Dm: 6), *Ixodes scapularis* (Isc: 11) and *Tropilaelaps mercedesae* (Tm: 6). Leaves are colored according to the organisms classes: Insecta (blue) and Arachnida (black). *Dermanyssus* transcripts are highlighted in green. The amino acid sequences and a comprehensive list of acronyms are listed in S5. (B) Hierarchical clustering analysis of the differentially expressed genes (DEGs). Blue to red colors represent gene expression levels (i.e., FPKM values from −1.5 to 1.5). To clearly describe the DEGs of each olfactory-related family, we performed cluster analysis using a pheatmap package in R software based on FPKM-values, represented as a heatmap.

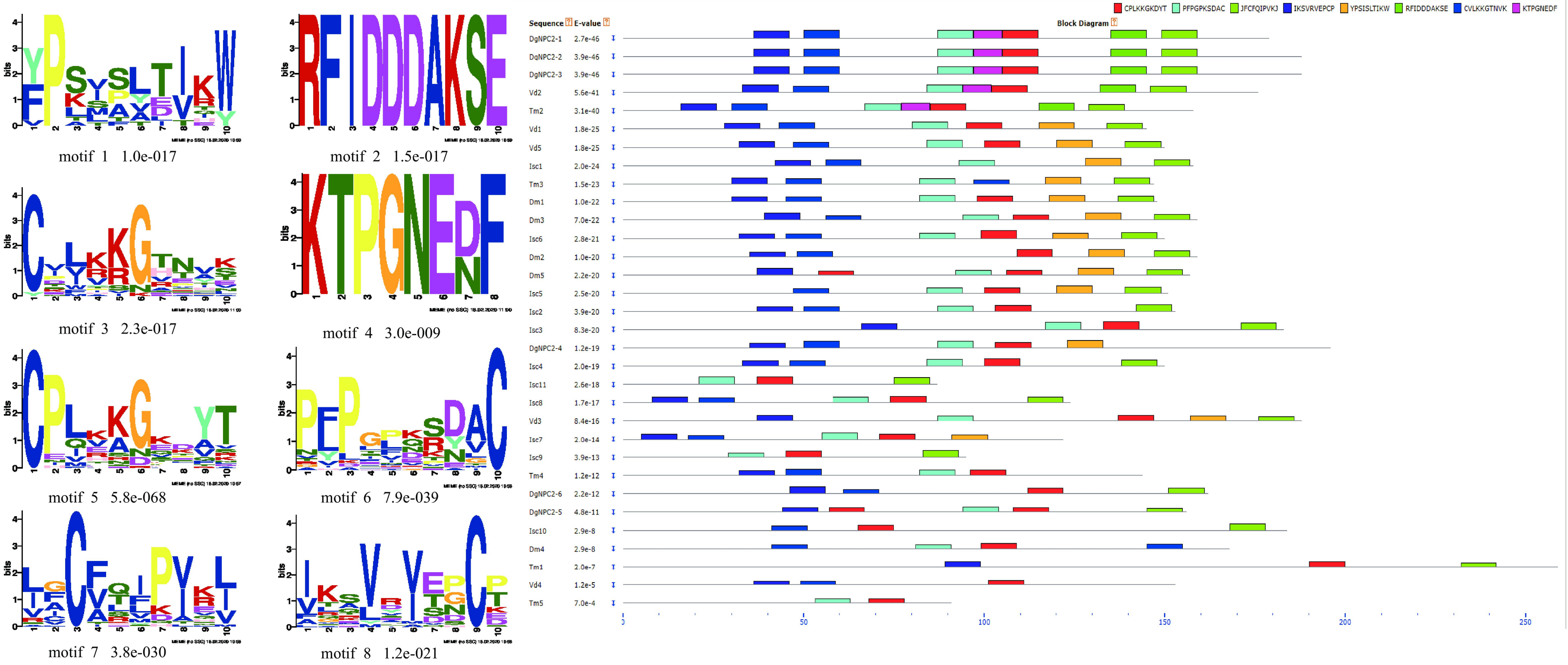
motif 1 1.0e-017
motif 2 1.5e-017
motif 3 2.3e-017
motif 4 3.0e-009
motif 5 5.8e-068
motif 6 7.9e-039
motif 7 3.8e-030
motif 8 1.2e-021
CPLKKGKDYT
PFPGPKSDAC
JFCFQIPVKJ
IKSVRVEPCP
YPSISLTIKW
RFIDDDAKSE
CVLKKGTNVK
KTPGNEDF
Sequence
E-value
Block Diagram
DgNPC2-1 2.7e-46
DgNPC2-2 3.9e-46
DgNPC2-3 3.9e-46
Vd2 5.6e-41
Tm2 3.1e-40
Vd1 1.8e-25
Vd5 1.8e-25
Isc1 2.0e-24
Tm3 1.5e-23
Dm1 1.0e-22
Dm3 7.0e-22
Isc6 2.8e-21
Dm2 1.0e-20
Dm5 2.2e-20
Isc5 2.5e-20
Isc2 3.9e-20
Isc3 8.3e-20
DgNPC2-4 1.2e-19
Isc4 2.0e-19
Isc11 2.6e-18
Isc8 1.7e-17
Vd3 8.4e-16
Isc7 2.0e-14
Isc9 3.9e-13
Tm4 1.2e-12
DgNPC2-6 2.2e-12
DgNPC2-5 4.8e-11
Isc10 2.9e-8
Dm4 2.9e-8
Tm1 2.0e-7
Vd4 1.2e-5
Tm5 7.0e-4
0
50
100
150
200
250

Figure S5. Motif analysis of NPC2 protein from *D. gallinae* and other classes including insecta and arachnida. The parameters setting in this study for motif predictions were: minimum width of motif = 6, maximum width = 10 and maximum number of motifs to find = 8. Motif-based sequence analyses were predicted by using web-based version 5.1.0 of the MEME server. Eight amino acid motifs with various widths and corresponding *e*-values were identified, and the lower part indicates approximate locations of each motif on the protein sequence. The results showed that motif 1 (CPLKKGKDYT) and motif 2 (PFPGPKSDAC) were present in all the NPC2 orthologs except for *I. scapularis* (Isc1), *Tropilaelaps mercedesae* (Tm3,1), *D. melanogaster* (Dm2,4), *D. gallinae* (DgNPC2) and *V. destructor* (Vd4). In addition, the homologous NPC2 from different acari species had similar motif patterns; for example, *D. gallinae* (DgNPC2-1, 2 and 3), *V. destructor* (vd2) and *T. mercedesae* (Tm2) had same motif-pattern as 4-7-2-8-1-6-3. Different motifs are shown by different colored boxes, where small number indicate high conservation. Protein sequences of NPC2 used in this analysis are listed in Supplementary file 2, Table S6.

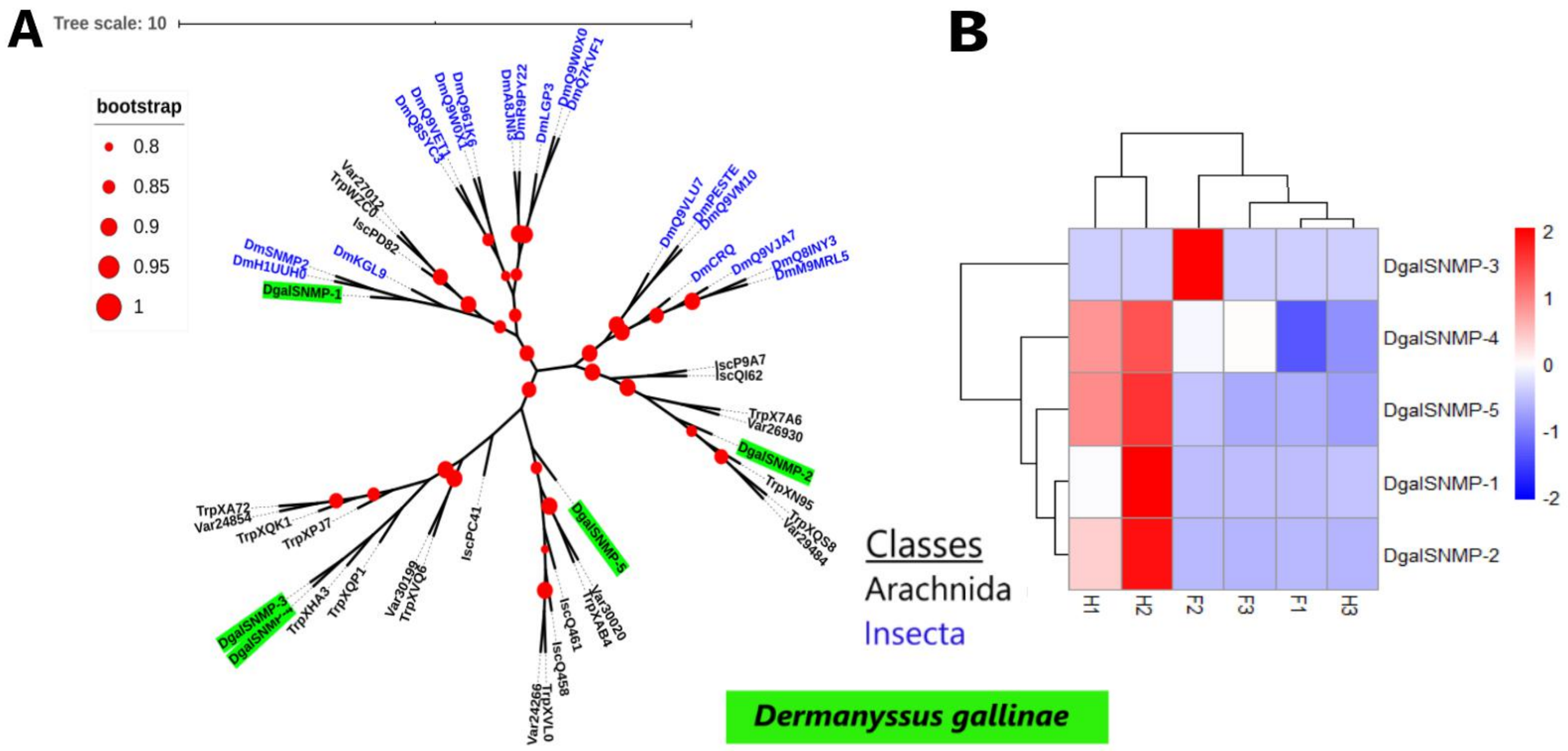

Figure S6. An unrooted phylogenetic tree of SNMP proteins and hierarchical clustering analysis of the differentially expressed genes (DEGs). (a) An unrooted phylogenetic tree of SNMP proteins. The tree is constructed by aligning the SNMP sequences of *V. destructor* (Var: 7), *D. gallinae* (Dg: 5) *T. mercedesae* (Trp: 12), *Ixodes scapularis* (Isc: 6) and *Drosophila melanogaster* (Dm: 19). Leaves are coloured according to

the organisms classes: Insecta (blue) and Arachnida (black). *Dermanyssus* transcripts are highlighted in green. (b) Hierarchical clustering analysis of the differentially expressed genes (DEGs). Blue to red colors represent gene expression levels (i.e., FPKM values from −2 to 2). To clearly describe the DEGs of each olfactory-related family, we performed cluster analysis using a pheatmap package in R software based on FPKM-values, represented as a heatmap.

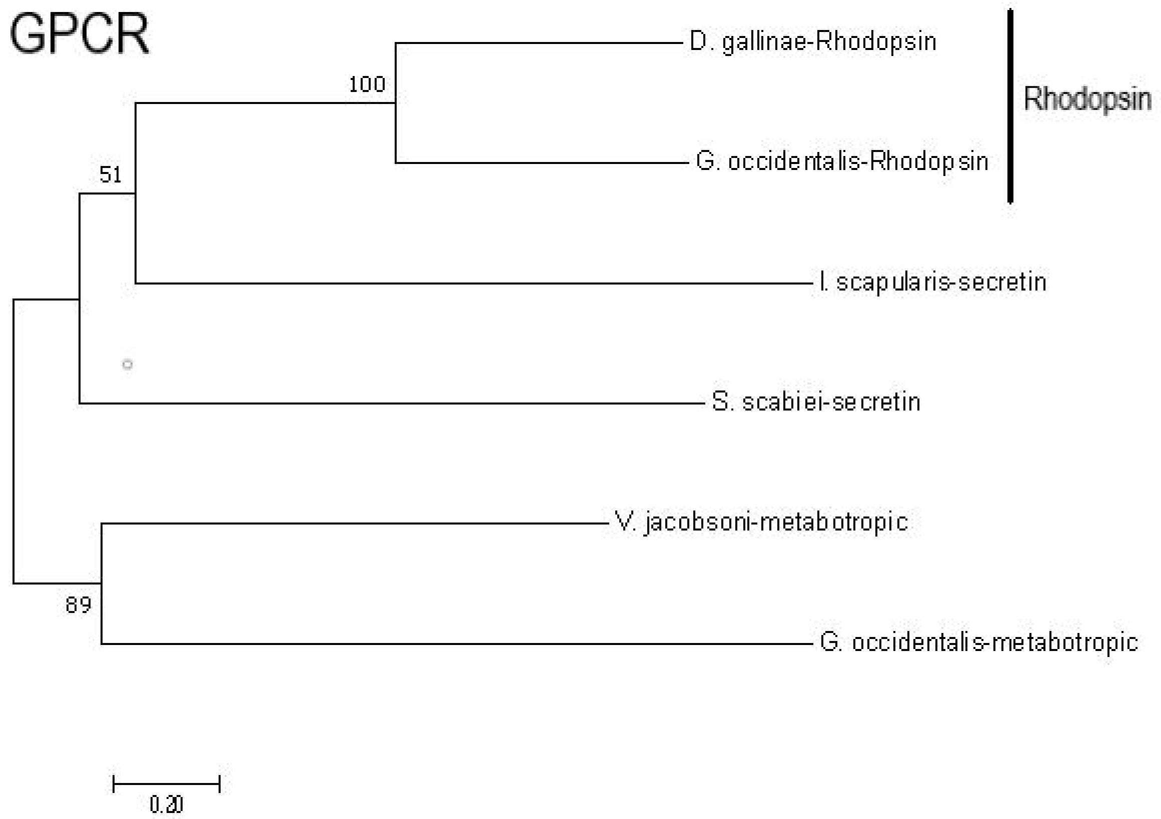

Figure S7. Phylogenetic tree of rhodopsin-like GPCR of *D. gallinae* and other mite species. The amino acid sequences are listed in Supplementary file 2, Table S8.

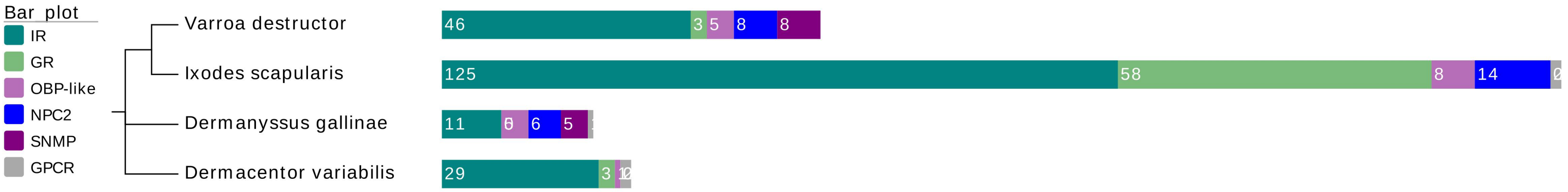

Figure S8. The number of chemosensory genes in different mite and tick species are presented in bar plot. A phylogenetic tree showing evolutionary relationships between various species are illustrated on the left. The number of chemosensory genes are obtained from *V. destructor, I. scapularis, D. variabilis.* The tree image was further viewed and graphically edited using the iTOL online tool with the number of identified chemosensory genes.
