# Supplementary material for: Comparative morphological and transcriptomic analyses reveal chemosensory genes in the poultry red mite, Dermanyssus gallinae
Source: Sci Rep. 2020 Oct 21;10:17923. doi: 10.1038/s41598-020-74998-7 (PMC7578799; doi:10.1038/s41598-020-74998-7)
Supplement: Supplementary file 2 — Supplementary Information 2. [file 41598_2020_74998_MOESM2_ESM.pdf]

Comparative morphological and transcriptomic analyses reveal chemosensory genes in the poultry red mite, *Dermanyssus gallinae*

Biswajit Bhowmick[1], Yu Tang[1], Fang Lin[1], Øivind Øines[2], Jianguo Zhao[1], Chenghong Liao[1], Rickard Ignell[3], Bill S. Hansson[4] and Qian Han[1*]

[1]Laboratory of Tropical Veterinary Medicine and Vector Biology, School of Life and Pharmaceutical Sciences, Hainan University, Haikou, Hainan 570228, China.

[2]Norwegian Veterinary Institute, Ullevaalsveien 68 P.boks 750 Sentrum, 0106 Oslo, Norway.

[3]Disease Vector Group, Unit of Chemical Ecology, Department of Plant Protection Biology, Swedish University of Agricultural Sciences, Alnarp, Sweden

[4]Department of Evolutionary Neuroethology, Max Planck Institute for Chemical Ecology, 07745, Jena, Germany.

*Correspondence: Qian Han Email: qianhan@hainanu.edu.cn

Supplementary Materials-2

Table S1. Summary of the transcriptome sequencing data from *D. gallinae* samples.

Sample	Total raw reads (Million)	Total clean reads (Million)	Total Mapping (%)
F1 (Forelegs)	24.02	23.53	72.51
F2 (Forelegs)	21.73	21.49	68.43
F3 (Forelegs)	21.73	21.29	67.16
H1 (Hindlegs)	21.74	21.47	69.06
H2 (Hindlegs)	21.75	21.51	65.82
H3 (Hindlegs)	21.72	21.30	73.56

Table S2. Protein sequences of IRs used to construct phylogenetic tree.

>TuIr25a ; XP_015792522.1 ionotropic receptor 25a-like [Tetranychus urticae]<br>MIVINLLFLTLLINVQYCLTYYLNDFIIGVVTGPNGGFVERSFNAALEQLNLTSITISETKWIRGS<br>NNTAVSDICDSLVTRSHQYPSLIFDFTPQSVDNENGQFSTAIKKYSRLSGTPTVSTSLSLGATKNP<br>WNRLNSIENNYLIHLYRPGDVIPMIIQDIITLYNITEAVILHDQSFILDHKLTGLLDKSTVDYDVK<br>RLDADTLEDTMNELKASKVHNYFVLAKIANIDRILEMASSLGMFRFADSWFMITKGRGQIKCD<br>NCTHGSILLYQPSRVKSIEYDHSPGLNNISKEKRIDAFFIYDLVRLTLMSVDEMVKTNVWPINLT<br>FPDCGKSLTTRQIDERKGLNLRDQMSLEGFFGLFGQFVISPSTTPNPLSNQLSDGFDQFIDYQEII<br>MRVSKVELWNYQPFRYYLVSEWIAEPPRGRFILTGAIQAASKPLYRIVTIQQPPFVMKWPNGTW<br>YGYCIDLLEAIQDRLAEEDKFDYELYESPDGKYGDKDPTTGKWDGIMGEIQSDKAQIGIGSVT<br>VMAERESIVDFTVPYYDLVGISILMKKTTTPSHMFKFLTVLTNEVWLSVLVCYLATCAAMFIID<br>RLSPYSYRNTKNKRKDQNASRDFNLKECFWFCMTSLTPQGGGEAPINLSGRFVAAIWWIFGFV<br>LVATYTANLAAFLTVSRLDSPIDGLDKLVEQYRIRYAPVKDTLSMIYFKRMAYIEKKFYEIWKD<br>MALDENLDKKERGKLTVWDYPLTQKYSNILKQIEMTGMPKTFEEGVARVKKSKNSAEGFALL<br>ADATKIRYAAMTNCDLQQVGNEFSRKPIALAVQENSPLKDKLSSAILKLLNLRKLESLKEKWW<br>SNNPEKADCEDPKKASDGISIRNIGGVFIVISVGVVLACITLIWEHCIVKKFRKSSAKVSNVFRQ<br>EIGKKESSKL YNFPMPYNPKMKEKI
>GoIr25a; XP_003743738.1 ionotropic receptor 25a [Galendromus occidentalis]<br>MWSSPRSLSPASRGWNGVNRSSEDPSWELVSAVITEKAFPIAKEAIERGLSRAKNDGKNIKWSF<br>IELTNVELDHKKLCSMIMEKKPSLVINTVRTPGSNYYKLTMTIKEVLKNLGMPTLDLSYGISDE<br>YSMSGWSTKKGDQGNYLIHVTPPGDSYTQAVRELSLKMDLATAGILYDKTVLIDHKYARLLEN<br>VPTRHIMREVGESAADFWNKTQVIQSTDVSNYFVVGSISHLSNALEVAKKYDLRQRHHCWVL<br>VTKQEGEPSCDGCTNLEALFVHPVKVDDTRVGKEGPYGADKLDGLFYEEISEYTVKRIFATEK<br>YSKEPIVATCPGVGSARPNNNLYAALNSAKLKGIYGDVVFDMKVGYQQIRMKVEHLNFTTAK<br>NRDTQDKGYFTYQGDANVAVWKEGQSSLEQYGAVTFYKVVTIVQPPFVYRNNETKKFRGYCI

DLIDEIKKILKFEYEIYEVADSHFGSKNKEGTWSGLIGDLVQKKADIALGPIAVMAERETVVDFT<br>VPYYDLVGLSILMKKPEVKPSLFKFLTVLETNVWGCILAAYFFTSFLMFIFDRLSPYSYRNNKE<br>KYKDDDEKREFTLKECLWFCMTSLTPQGGGEAPRNLSGRLVAATWWLFGFIIIASYTANLAAFL<br>TVSRLESPIESLDDLSKQYKVKYAPQKDTTASTYFQRMANIEEKFYEIWKNMSLDDSLTDDQR<br>AELAVWDYPVSDKFTKIWWTMQEANLPESFEAGVQRVKDSLTSEGFAFIADATQIKYATMTNC<br>DLTQIGSEFSRKPLALAVQQNSPLRDQLSSAILKLLNQRRLESLKETWWNQNSEKKECDEEGK<br>NNDGISIKNIGGVFIVIFIGVVMACITLVFEYFWYKNKKPRSKVVSVKEHMPQKR
>TmIr25a; Tropilaelaps mercedesae IR25a mRNA for ionotropic receptor, complete cds (LC438511.1) Honey bee parasitic mites<br>MWVPLRISYLLVITLLLFENVLGQTLNIAVIVEKRFELARQAIDNGLKKAKNDGKPIEWKFIVL<br>DTEGETKGLCNMILANKPSLVINTVRTPGSNYYKLTKAIKETLKNLAMPTLDLSYGSTDEYQLS<br>GWNKNTENERSYLIHVTPPGDSYTQAVRELSIRMDLATAGILYDKSVIIDHKYARLLENVPTRHI<br>MREVSESYADFENKTKVIQSTDVSNYFVVGTIQHLSNALEAAQKNNWKPRHHCWVLLTKQEG<br>EPLCESCANHEVFFVHPVDADDKRVGTEGAYGNEKLDGMFYEEIAYYTVMRIFASPKYSKEPIT<br>ASCAGESTARNDYNLTEALTSSKLQIRYGEVLFPNTKHSRSGHQIIRMKVAHFNYTTAKVRQEN<br>EKGYFVYSGDSTTWQKNEQSSMEQFGSVTFYRVVTIVQPPFVYEGNGTGPAFRGYCIDLLDEI<br>KKILKFEYTVYGVEDKHFGSKNKDTGQWNGLIGDLVKKKAEIALGPIAVMAERETVVDFTVP<br>YYDLVGLSILMKKPEIKPSLFKFLTVLETNVWGCILAAYFFTSFLMFIFDRLSPYSYRNNKEKYK<br>DDDEKREFTLKECLWFCMTSLTPQGGGEAPRNLSGRLVAATWWLFGFIIIASYTANLAAFLTVS<br>RLESPIESLDDLSKQYKVKYAPQADTTASTYFFRMANIEEKFYEIWKNMSLDDSLTDDQRAELA<br>VWDYPVSDKYTKIWWTMQEADLPKTFEEGVARVKNSQGGDGFAFIADATQIKYATMTNCDLT<br>QIGSEFSRKPLALAVQQNSPLRDQLSSAILKLLNQRKLESLKETWWNQNPEKKNCDDEGKNND<br>GISIKNIGGVFIVIFIGVVMACITLVFEYFWYKNKKPRSKVVSVKDHMPQKK
>TmIr93a IR93a Tropilaelaps mercedesae IR93a mRNA for ionotropic receptor, complete cds (LC438512.1) Honey bee parasitic mites<br>MWPRLIFFASLAAGWRTRAEEIKAVVLPDSQIVVDEQDVRDFIFNRTGEYNATVGAAGYYISFL<br>ATNIMSVSELNERVNPESESNINLNLLVIALDCDDTYKAMDVQMKKFPTSMLIALNRPYCPRFD<br>ADDQQVANKKPFALSIPVTQGPTDVLPILADFRAMLPLSRWSEITIIVPYSYTEEYLEQIMRAVE<br>GVRSNDEVNSAASIAFWEYCPPSALADGNCGLNSESMKRILDSYRNNDNKKVDREFIIIGDEEL<br>VLDFFERGVDYGIFTLFREFFIILTTPYSDTIKDLIFEKIASFANIVIASTDVKDEDCPVSGTCHIDL<br>PMNAYMNAVNKNPDLFERNVDKFNHKYTIKKAVLTELRESKSCGFCSKYTIRTLTVKAGRKTF<br>DPVAVWDYFDGLQMVEDTSRVSYQLFPRFLGNMGGVNLRIGVINEPPMTEVTSKEGRVISVNG<br>TLVDLLKELARTLNFTYTFIMPPEPVPGLKQNDGRWSGLIGQLVRQETDLALYDFTPTPDRQSA<br>VNFTVAFDEAPYKFLVPKPQPNYKYLFLDPFTWDTWLAVLGTVLIIGPILWCVHINSKFYDYYD<br>MRDGKGLFKLSNCEWYCFGAIIQQGGIHLPDAISGRILVGFWWLFVIVTLTTYSGNLVADLTFP<br>KIRNPFDTVDSLLNSHMSWGAFRGQAVIEILKLQQQGPLTKLSMKLTHIETAHEGWALKEVAD<br>GNMALIGSEVTLYHYIGKQFIATGQCDYAVAKEEIIREIKVLAVRPGFPFLARFNTLLTRMVETGL<br>VIRWKKKYWPKENECTVESKPQAGDIRRITIAHMEGSFWVLGAGFLISFLLLGVELLRKRRELR<br>DPQAGKRISPTDIKENLFRKTGHSDKSHYKTDYGAHGSGAGPGGKGGQTGYSFDLSNTPFRGY<br>SGFPNRTDLIPYNYP ARRY
>DgalIR1 DgIr763lcl|ORF2; GAIF01020763.1 TSA: Dermanyssus gallinae isotig25947 transcribed RNA sequence

MMLKESAKFLTGNERFEGYCVDLLQELSKELKFTYEIRLAADGGYGIKSDTGHWNGMIGEVV EGKADLAIADLTITSAREAAVDFTMPFMNTGISILFKKPTQKATSLFGFLSPFSTGVWTYVVGAY LGVSCVLFLVGRMSPYEWDNPHPCRQNDQVLENSFSLLNSLWFTIGSLMQQGSDLAPKAMST RTVAGIWYFFTLIMISSYTANLAAFLTVEKTVYPVESAEDLAKQTCSRR
>DgalIR2 DgIr194lcl|ORF3; GAIF01026194.1 GAIF01026194.1|TSA: Dermanyssus gallinae isotig14279 transcribed RNA sequence<br>MNPLSREIWMCIIFAYVGVSVVLFLVSRFSPHEWRYEETFVGPTVSNDFSLYNSLWFSLGAFMQ QGCDICPRSVSGRIVGGVWWFFTLIIISSYTANLAAFLTVERMVTPINPADDLAKQTEVEYGTLE SSSTQ
>DgalIR3 DgIr4048lcl|ORF3; GAIF01024048.1 GAIF01024048.1|TSA: Dermanyssus gallinae isotig18401 transcribed RNA sequence<br>MEAIQNSGVKISIAKKVKSEAEASDAVLELSSLGRLRPDNIEKIVLNVNKPTLMRTVLSGMIKE VKSFKNLNTQFILASLVTDDFWQNIRFRDAGSSLNVTAFRLVDSELPHVLRFHKKMMQNANY RDSFKNQGTETYLFLDAVTALLTNYDRALQEKPTLLDAYLQADDGTKESPGFNCMDQSRYFD NGKDLASQLKQVKLPEGQTGAVEFLADGTRLQPTIAVIHGSHVGHVKLGSWSPKKGLMLTDK PEALPQIKAAGAITPAKEISVGGILSPPYLMLKRGSDSDYTGLVPELLEALANIVPFAYKIHVVS SGQALGRADYNGSWSGLMSEVINKKVEIAIADIPLTAERQKFIEYTQPFLMDDLAVVVSRKSQ AGRPSLSIFFVTIFSWQLWLSILGVFVTFVLLAYLLNRLASTKENADSFSRTLWLSVGSFLCRTP GPEGRCRASRLLLLFWWLFLAVVTVLYVSAFVAIVQTVVFGYGPDVFSMRKFVQDAAGGIVD IGYVRDVVPGKVLKTPRSGEFATIYSQMLSTPRGRAVFSVEDGVHSVSTNFAFLGLHSQIESYL DDCSKRSYIVSANF
>DgalIR4 DgIr3943lcl|ORF12; GAIF01033943.1 GAIF01033943.1|TSA: Dermanyssus gallinae isotig11463 transcribed RNA sequence<br>MRLRPATAEVLAMTFTMRPGRQMGPTRGILVVVTILTIIAATLIEPTRAAVKIGGIVEAGQSRT KELLEEILKERSSNLPTQNRMHPEIAYVEANATFGISNTACAQVGQGVSSIVSPCTGIVHTTLDS LSRRLSLPYINTAPDLVDVTGRNRPRESSDDTGDRGFTIVMVPSTAKATADLIRENKWKDIYFI YDKPEGLDELELILSHLPKEDEVQVKGVHQMTSNTSAMDYLQAIENAAWDVEKHIILAIDVK MAKRTVELSYRKKRAYYHFVIPRFTVRHFWSEVRTFDQSTVATFQIVDEDEFRSMKAKRAGV STPKDPEDEHLNEAEALLMDAGQLLFEAVQSLSREQPLAAGEIFKELSQISASVSCYRFPLEVY AQGQIIADVMKKMRFKGSTGQVRFNEYGRRTDFTLEVMELTSLVDMEKVGVWNERDGLRFS NEHERQAKRARVRQDVSKNIIITTIIERPFLFFKNENDTRPHIDNVDGYLKELIVRVMQDIPGRP AFDIHFVKDGKFGYKDAQKPSGWNGMIGEILRKDADVAIAPLTVTSERNKVVHFSQPFMATGI GAIARNDADDLLNIRTPFTFLRPLDWKIWLLFLGAYGVVALILLLVNLVNLAVATPPVDPPTPC RHLYNSIWYAFGMFMSEECNNCRPKSLGSRVIACSWWLFILCVLSIYTAQLWINDPF
>DgalIR5 DgIr780lcl|ORF3; GAIF01028780.1 GAIF01028780.1|TSA: Dermanyssus gallinae isotig14118 transcribed RNA sequence<br>MYKENWREAGLTGNDKYEGYCIDLLDAIASSEHFNNNMKYVIREVADNSYGRKDADGRWN GMIGELLNGKADLAVADLTITYVREEAVDFTMPFMTLGISILYKKTAK
>DgalIR6 DgIr6943lcl|ORF1; GAIF01016943.1|TSA: Dermanyssus gallinae isotig24381 transcribed RNA sequence<br>MRITPPRGYKFSLFRTYWLVWAVLFGAAVNVDCPRGYTARFMSNVWAMFAVVFLAIYTANL AAFMITREEYYDLSGIEDSRLRYPHLMEPPFRFGTIPYGNTEQVLQRNKPTMYEYMRPYNRSN

VNEGIKAVKKGTLDAFIYDATVLDYFVGQDDECRLLTVGSWYAMTGYGFALPKKSKIFTDV
>DgalIR7   DgIr466 lcl|ORF4; GAIF01003466.1 GAIF01003466.1|TSA: Dermanyssus gallinae isotig14278 transcribed RNA sequence<br>MNPLSREIWMCIIFAYVGVSVVLFLVSRFSPHEWRYEETFVGPTVSNDFSLYNSLWFSLGAFM<br>QQGCDICPRYIFGQHSHQADEAAIDNKTQREL
>DgalIR8   DgIr541 lcl|ORF2; GAIF01006541.1 GAIF01006541.1|TSA: Dermanyssus gallinae isotig08537 transcribed RNA sequence<br>MNGLGAPVLALALLGVAVADIKIGGLFEAGEDIVEMAFNSAVERINSHGLAEKGERYTPTSDR<br>LLTRVEHIERADSFQASRKVCTLLEEGVAAVFGPQSTDASFAVRSACETLDVPHIETHWDYRS<br>RTTNHSLNLYPHPSALGKAYLDFIKYKDWKNFAILYEENDALVRLQEILKDATLMKENRVTV<br>RQFEIGTEYRKTLKDIGKTGIKNIVLDVPIKNLYTVLKHAQQVDMMSEYHNYFITSLDAHTIDT<br>EDFQYGGTNISAFRLIDFSSKEIDEVARDWIQRRHRFSSKKSSSVQSSFNTGDQDFYKNLTTKV<br>ALMYDAVRLFATALKDLHPDSGPKIQVAPLSCEREHKWSQGNAFVKYMRMINIKGLTGNIRF<br>NKDGHRTDMRLTVLDMAHSGWREASEWTMLGCISVTANYTRE
>DgalIR9   DgIr849 lcl|ORF1; GAIF01028849.1 GAIF01028849.1|TSA: Dermanyssus gallinae isotig14310 transcribed RNA sequence<br>MLTSYSSHFHIPLATSLSPRSFSLRSDYAISTQVSLAEATGSLLRRYRWSQAAYIYDDSASPDILE<br>AIQNSGVKISIAKKVNNEAEASDAVLELSSLGRLKPEKIEKIVLNVNRPSLAKAVLSGMIREVKS<br>FKNLNTQFIFANPVSDDFWQYIRFRDAGSSLNVTAFRLVDSELPHVTRFHKQMMQLSSHRSLF<br>HNQGTQSYLFLDAVTALLTGFEPS
>DgalIR10   DgIr982 lcl|ORF1; GAIF01026982.1 GAIF01026982.1|TSA: Dermanyssus gallinae isotig09650 transcribed RNA sequence<br>MAVDKVNRDMGLLSATRFQAKVFRVRSRDSFSAARHVCELARHKVAAFVGPRSPSVKELVK<br>ASAYRLNVPHISTDPELHERR
>DgalIR11   DgIr664 lcl|ORF5; GAIF01023664.1 TSA: Dermanyssus gallinae isotig14117 transcribed RNA sequence<br>MYKENWREAGLTGNDKYEGYCIDLLDAIASSEHFNNNMKYVIREVADNSYGRKDADGRWN<br>GMIGELLNGVRTTHWTTH
>DmelIR7a<br>MFHHLWLLMGLRSLAMGALHPPQPEAMTPLVAAALEILAEQVSPSQSTLAVMDLTQDAEHRD<br>ERQEQLMTIILRSVGSEMALRTFQKPPAEVPASFVVFLVNSAQAFNTLGFHFTDIHSTREFNFLIL<br>LTHRMSSRAERLQVLRDISRTCVRFHTSNVILLTEKRDGVVLVYAYRLLNMDCDLSVNLELIDI<br>YKNGLFRHGHEARSFNRVLSLSGCPLQVSWYPLPPFVSFIGNSSDPEERAQIWRLTGIDGELIKL<br>LASIFDFRILLEEPCNKCLSPDIKDDCSGCFDQVIISNSSILIGAMSGSHQHRSHFSFTSSYHQSSL<br>VFIMHMSSQFGAVAQLAVPFTVIVWLALVVSSLLLVLVLWMRNRLVCGRSDLASHALQVLTTL<br>MGNPLEARSLPRSSRLRILYAGWLLLVLVLRVVYQGKLFDSFRLPYHKPLPTEISELIRSNYTLIN<br>QEYLDYYPRELTVLTRNGSKDRFDYIQGLGKEGKFTTTSLIATMEYYNMMHWSTSRLTHIKEHI<br>FLYQMVIYLRRHSLLKFAFDRKIKQLLSAGIIGYFVREFDACQYRKPFEEDYEVTPIPLDSFCGL<br>YYISLIWLSAAVVAFILELLSQRIVWLRRIFE
>DmelIR7b<br>MKYWLYILSCCSLVASTMESSSDWDLAEALAQVVANSEMGRFKTLYIYTHTNSQTGGHLEEL<br>LDQVLMIVPNNLQARRLLLQQSMEYKPYVHAVLALVDGLPSLSAIYARIRATQDLSHTLIYMS

MPTDAYGEEMQATLRFLWRLSVLNVGVVLRPPGDHILMVSYFPFSALHGCQVISANVVNRYQ<br>VGTKRWASQDYFPSKLGNFYGCLLTCATWEDMPYLVWRPDGSGSFVGIEGALLQFMAENLNF<br>TVGLYWMNKEEVLATFDESGRIFDEIFGHHADFSLGGFHFKPSAGSEIPYSQSTYYFMSHIMLV<br>TNLQSAYSAYEKLSFPFTPLLWRAIGLVLILACLLLMLLVRWRHHHELPRNPYYELLVLTMGGN<br>LEDRWVPQRFPSRLVLLTWLFATLVLRSGYQSGMYQLLRQDTQRNPPQTISEVLAQHFTIQLAE<br>VNEARILASLPELRPEQLVYLEGSELQSFPALAQQSGSSARVAILTPYEYFGYFRKVHPMSRRLH<br>LVRERIYTQQLAFYVRRHSHLVGVLNKQIQHAHTHGFLEHWTRQYVSAVDEKDESVARIASTS<br>YSTLDGIDGDPSLSESEEDQQVAPVRQNVLSMRELAALFWLILWANLGAVVVFVLELLLPRIKL<br>RKILRKMKSDIKKQISKLVRK
>DmelIR7c<br>MLHSAVHNVSLVYALVWAIDNYYGMATSTPLAVVQFPTSRESRRLHNDLIDAALGRSSGTGRI<br>QFLLEDDRVEMTETDTDPPPPSGLTGRPIAIWFLDSLRSYFRLEMYLNQLGSPYKRNGFFLVIYT<br>GLEDQPMESLKIMFRRLLNMYVLNVNVFLQRDGTVHLYTYYPYGPHHCQSSLPVYYTAFQDL<br>AAPANGFGLTKPLFPRKLTNMHGCEMVVATFEHRPYVIIEDDPKTPGGRSIHGIEGLIFRSLAER<br>MNFTIKLVEQKDKNRGEILPDGNFTGILKMMVDGEVNLTFVCFMYSKARSDLMLPSTSYTSFPI<br>VLVVPSGGSISPMGRLTRPFRYIIWSCILVSLIFGFVLICLLKITALPGLRNLVLGRRNRLPFMGM<br>WASLLGGLALYNPQRNFARYILVMWLLQTLILRAAYTGQLYLLLQDVEMRSPIKSLSEVLAKD<br>YEFRILPALRTIFKDSMPTTNFHAVLSLEESLYRLRDEDDPGITVALLQPTVNQFDFRSGPNKRHL<br>TVLPDPLMTAPLTFYMRPHSYFKRRIDRLIMAMMSSGIVARYRKMYMDRIKRVSKRRNLEPKP<br>LSIWRLSGIFVCCAGLYLVALIVFILEILTTNHRRLRRAFNVINRYAA
>DmelIR7d<br><br>MDIRCVVALLLGLCKVQAVVWPHQHLLEEQLASQISATLQKIFINGLAVYNFGVFISTSYEEMD<br>RDRVILVHQVLNRNLYPPNFPVAVVLASKMNRKITAQVFTQLLFVQNAEQAIAIAEGVNRNGLC<br>VIVLLTSQPERPIMTKIFTYFMQERYNINVVILVPRLHGVQAFNVRPYTPTSCSSLEPVEIDIKDG<br>DLWDVFPRRLKNLHGCPLSVIVWDIPPYMRINWKSSDPMDGLDGLDGLLLRIVARKMNFTLK<br>LIPNEPNGLIGGSSFMNGTFTGAYKMLRERRANITIGCAACTPERSTFLEATSPYSQMSYIIVLQA<br>RGGYSIYEVMLFPFEKYTWLLLSTILGLHWIVGSRWRMPSPILAGWMLWIFVIRASYEASVFNF<br>IQNSPVKPSPRTLDQALSGGFRFITDHASYRMTLKIPSFQGKTLISAGQPVDVFDALLKAPWKT<br>GAFTSRAFLADHLVRHRKHRNQLVILAEKIVDNMLCMYFPHGSYFAWEINKLLFNMRSFGIFQ<br>HHSQILAWDNLPTTTDTDTPGKRIHSSTESVATGFAESMSFVVAALNCLMGALCISIVVFGLELL<br>SRRRHWTGLEWLFERV
>DmelIR7e<br>MNISALLNSYYDLSGEQMNHINEFVARAVLHVVHHYILSVTPSLVLTLCCRSNHTCNFYNKMM<br>STLFREWGLAPLQIVNVLRGVPWHPVPGRRHFNVIFTDSFAAFEEIRMEYYSREYNYNEHYFIF<br>LQARDRLLQGEMRLIFDYCWRYRLIHCSIQVQKSNGDILFYSYYPFGEHGCSDMEPQLINRYN<br>GSMLVEPDLFPRKLRNFFGCPLRCALWDVPPFLTLDEDQEEVLRVNGGYEGRLLLALAEKMNF<br>TIAVRKVHVNMRDEALEMLRRDEVDLTLGGIRQTVARGMVATSSHNYHQTREVFGVLASSYE<br>LSSFDILFYPYRLQIWMGILGVVALSALIQLIVGRMLRERMGSRFWLNLELVFVGMPLLECPRS<br>HTARLYCVMLMMYTLIIRTIYQGLLYHLIRTHQLNRWPQTIESLVQKNFTVVLTPIVQEVLDEIP<br>SVQHMRFRLLEANSELDPLYFLEANHQLRQHVTASALDIFIHFNRLSADKVHQRGEQGSGAHF<br>EIVPEDIISMQLTMYLAKHSFLIDQLNEEIMWMRSVGLLSVWSRWELSESYLRNEQSFQVLGT<br>MELYAIFLMVLVGLIVGLLVFILELVSMRSIYLRKLFT

>DmelIR7f<br>MNTTSDSNAGSSLSSGSGYSIYKSYLENSRIDMQGEDANLYVARALRLVIENVLAQLSTTLVVT<br>ISTRHLGTAHWFEYMMNILMDSWRMVAVQLLRIRPDLVVNPVPGRKRVSLLMVDSYQGLLDT<br>NITASNANFDDPDYYFIFLQARDHLIPKELQLILDHCLAHFWLHCNVMIQTAQVEVLVYTYYPY<br>TADACQKAYPIPVNTFDGRKWKASQMFPDKLSQMHGCPLTVLTWHQPPFVELVWDPKHNRSR<br>GSGFEIQLVEHLARRMNFSLELVNIALLRPNAYRLAEGSSEGPIEKLLQRNVNISMGYFRKTARR<br>NQLLTTPMSYYSANLVAVLQLERYRIGSLALLVFPFELSVWMLLLLALLIHLGIHLPSARRGNEE<br>DGGGGLQVVALLLGAALARLPRSWRHRFIAAHWLWASIPLRISYQSLLFHLIRLQLYNTPSFSL<br>DQLLAEGFQGICTANTQRLLLEMPQLARDPDSIQSVDTPFDWDVLNVLTRNRNRKIFAVANQD<br>VTLSFLHSSAHPNAFHVVKQPVNVEYAGMYMPKHSFLYEKMDDDIRRLDASGFIHAWRRASF<br>ASVHRKEQVHMTSRRYINHAKLSGIYMVMAGLYLLAGLLFAGEVLLRQRN
>DmelIR7g<br>MNVTSLLNFESMKYIGAQTQAASINHHVAQALRVFIEDFYQRIAPAFIVVLSCRRPSPMNFYRNI<br>MQLLYESVDTMIVQLVLVELGRPRRIAGPRTHNLLLVDSLDALLDIEIHTYTAQSDTSEYYFIFL<br>QQRDALIPHDMQGVFAYCWRHQLINCNVMTQSSGGQVLLHTYFPYAPGQCNDSQPTRINMFL<br>GESWKHRDYFPSKLHNLNGCPLIVLARKVSPFLDLDEGQRELRGLEGRLLQELSRRMNFSIQFS<br>GLQDQLKNRTTWTEKQLLQKLVQERIAHLAIGYVRKRIQYATNLTPVFPHYSNRVVGCLLLNA<br>HNLTSLEIWSFPFQALTWICLLLFASWLIFGLIVRSMYSALLFFILRYHLHQRLPGNLQDLTHGD<br>YAAVMGRTTLQDLREVPSLQDLLGLKSVIVTSEREEEVLRTLDRCTLREGAGSHPLFFGLISQD<br>ALLHLTQRGHRAGAYHIIPQDVLEQQLAIYLQKHSHLASHLDHLVMSIRSVGLVHHWAGQMA<br>SERYFRSRFLYREKRIRQPDLWAVYILTAGLYLLSLVVFICELLASRRAGL
>DmelIR11a<br>MRFAILWLFSGCLLPGIQVGIWVVVRAQPTGRDVLLSRLGNQQNELNTRRLANASSYLTRNYI<br>ANRINTLVVREICVECPYELSERQRQLVDQILASLAPELSVLLHKGTAEETTWEYTLFVVNDHT<br>AFTGQVFIFPDELLEREFFCIVVVSEIQSRQFVRQTVGSIVKSNLQMHFVNVVVVAQLEDGTVG<br>TYSYKLFKANCTPGITVRQINHFDRITGKPQQSMPDLYPVRNGHLGDCPFNVGAAHMPPHLIY<br>KRHKDPPPASNVSIPAEDLAGIDWDLLQLLAKALKFRIQLYMPQEPSQIFGEGNVSGCFRQLAD<br>GTVSIAIGGLSGSDKRRSLFSKSTVYHQSNFVMVVRRDRYLGRLGPLILPFRGKLWGVIIVILLL<br>AVLSTCWLRSRLGLSHPIEDLLTVIVGNPIPDHRLPGKGFLRYLLASWMLLTLVLRCAYQARLF<br>DVLRLSRHRPLPKDLSGLIKDNYTMVANGYHDFYPLELTCRQPLDFSARFERVQRAAPDERLTT<br>IALISNLAYWNHKHPNISRLTFVRQPIYMYHLVIYFPRRFFLRPAIDRKIKQLLSAGVMAHIERRY<br>MQYENKRKVASNDPVLLRRITKSIMNGAYRIHGLVIVLATGMFILELLAGRSNGRLRRWMEWV<br>HQ
>DmelIR20a<br>MLASLNRSTGLSAELLDLYGLVVHFLLSGEHTTLVYFNPAGLDCSWGVLWQRNLTAHPQIVW<br>QRNYSYPDLYYQFNAKLLVLACLPMDSRAAIQLEILANSLSHLRTVVRLLIEVAGPDQVTLARQ<br>YLSFCLRRSMLHVELYFRDYHHSLILYSFRAFPSFELVMRWISVGQGVKLFLHKLDDLRGHRLR<br>VIPDLSPPNTFFYRDARGDNQVTGYLWDFLATFAGRLNAGLEVVRPSWRAGSASDSSYMLEYS<br>AKGLIDVGLTTTLITKWNLWAIHQYTYPLLVSSWCTMLPVEKPLATPDLFGRIVCPTLAMTLLLI<br>ILVTWLVFRQLRCLTRLKNSRPARIVPHLLTLLLLTTCSAQLLSLLIFPPYHVRIASFEDLLRGDQ<br>KILGMRNEFYNFDGAFRARYAGVFYLIDDPNELYDLRNHFNTTWAYTMPYIKWLVIKTQQRHF<br>SKPLFRWSKDLCFFDFMPTSVIVAPDSIYWESIKDFTFRIHQAGLMKHWIRKSFYDMIKAGKMS<br>IKDYSDLETLKPLNIGDLEIVWRVCGAAIAVASAIFIMELLYFYINVFFNSL

>DmelIR25a<br>MILMNPKTSKILWLLGFLSLLSSFSLEIAAQTTQNINVLFINEVDNEPAAKAVEVVLTYLKKNIR<br>YGLSQLDSIEANKSDAKVLLEAICNKYATSIEKKQTPHLILDTTKSGIASETVKSFTQALGLPTIS<br>ASYGQQGLRQWRDLDEAKQKYLLQVMPPADIIPEAIRSIVIHMNITNAAILYDDSFVMDHKYK<br>SLLQNIQTRHVIAIAKDGKREREEQIEKLRNLDINNFFILGTLQSIRMVLESVKPAYFERNFAWH<br>AITQNEGEISSQRDNATMFMKPMAYTQYRDRLGLLRTTYNLNEEPQLSSAFYFDLALRSFLTIK<br>EMLQSGAWPKDMEYLNCDDFQGGNTPQRNLDLRDYFTKITEPTSYGTFDLVTQSTQPFNGHSF<br>MKFEMDINVLQIRGGSSVNSKSIGKWISGLNSELIVKDEEQMKNLTADTVYRIFTVVQAPFIMR<br>DETAPKGYKGYCIDLINEIAAIVHFDYTIQEVEDGKFGNMDENGQWNGIVKKLMDKQADIGL<br>GSMSVMAEREIVIDFTVPYYDLVGITIMMQRPSSPSSLFKFLTVLETNVWLCILAAYFFTSFLMW<br>IFDRWSPYSYQNNREKYKDDEEKREFNLKECLWFCMTSLTPQGGGEAPKNLSGRLVAATWWL<br>FGFIIIASYTANLAAFLTVSRLDTPVESLDDLAKQYKILYAPLNGSSAMTYFERMSNIEQMFYEI<br>WKDLSLNDSLTAVERSKLAVWDYPVSDKYTKMWQAMQEAKLPATLDEAVARVRNSTAATGFA<br>FLGDATDIRYLQLTNCDLQVVGEEFSRKPYAIAVQQGSHLKDQFNNAILTLLNKRQLEKLKEK<br>WWKNDEALAKCDKPEDQSDGISIQNIGGVFIVIFVGIGMACITLVFEYWWYRYRKNPRIIDVAE<br>ANAERSNAADHPGKLVDGVILGHSGEKFEKSKAALRPRFNQYPATFKPRF
>DmelIR31a<br>MNLLISMFILILAAGEGEIIPSMEESVVTNFVKSLVKTKQAIVFSCLFKDFKEISLALMRINQFVS<br>VVNLNQSYSLTSILTRENYARTSVMVNARCSGSSELLFEASENRYFNKTYQWFLWGVDLEVQS<br>LFPLNLNVGPNAQITYVNETADGYAYWDIHSKGRHLKSNLEINLIATLINDTLNIARDIFHLQSID<br>FRGQFNGLTLRGSVIDKEDIISNEQIESILSRPTKDAGVAAFIKYHYELLGLLRERFNFTVNFRNS<br>RGWAGRLGNTTFRLGLLGIVMRNEADIAASGAFNRINRFAEFDTIHQSWKFETAFLYRYTSDLD<br>THGKSGNFLSPFSDRVWLFCLLTLGAFSIIWVLFEIIDYKILRIRVNSQKLEHLNQKSSVICIKTTC<br>IERILQTFGACCQQGLDPNPVDRSVRFLVMTLFLFSLVMYNYYTSSVVGGLLSSSDQGPSTVDE<br>ITASPLKISFEDIGYYKVLFRESQNRSITRLIEKKLSSSRSLNELPIFSHIEDAVPYLKAGGFAFHCE<br>VVDAYPVISEYFDANEICDLREVSGLMEVEILNWILHKNSQYTEIFKTAMCNAQEKGFVERILR<br>RRQIKKPACQSLYTVYPVSLSGVLPGFVILICGFGASLLLLCLEKVYAHFGPRKFCGF
>DmelIR40a<br>MHKFLALGLLPYLLGLLNSTRLTFIGNDESDTAIALTQIVRGLQQSSLAILALPSLALSDGVCQK<br>ERNVYLDDFLQRLHRSNYKSVVFSQTELFFQHIEENLQGANECISLILDEPNQLLNSLHDRHLG<br>HRLSLFIFYWGARWPPSSRVIRFREPLRVVVVTRPRKKAFRIYYNQARPCSDSQLQLVNWYDG<br>DNLGLQRIPLLPTALSVYANFKGRTFRVPVFHSPPWFWVTYCNNSFEEDEEFNSLDSIEKRKVR<br>VTGGRDHRLLMLLSKHMNFRFKYIEAPGRTQGSMRSEDGKDSNDSFTGGIGLLQSGQADFFL<br>GDVGLSWERRKAIEFSFFTLADSGAFATHAPRRLNEALAIMRPFKQDIWPHLILTIIFSGPIFYGII<br>ALPYIWRRRWANSDVEHLGELYIHMTYLKEITPRLLKLKPRTVLSAHQMPHQLFQKCIWFTLR<br>LFLKQSCNELHNGYRAKFLTIVYWIAATYVLADVYSAQLTSQFARPAREPPINTLQRLQAAMIH<br>DGYRLYVEKESSSLEMLENGTELFRQLYALMRQQVINDPQGFFIDSVEAGIKLIAEGGEDKAVL<br>GGRETLFFNVQQYGSNNFQLSQKLYTRYSAVAVQIGCPFLGSLNNVLMQLFESGILDKMTAAEY<br>AKQYQEVEATRIYKGSVQAKNSEAYSRTESYDSTVISPLNLRMLQGAFIALGVGSLAAGVILLL<br>EIVFIKLDQARLWMLCSRLQWIRYDRKV
>DmelIR41a<br>MFIDLSWSLVLSAIVGKYLNESTICIFWNDKFEFQLLHKSDYISFVGINIKSFDDNGGHYIIDTGL<br>KKKELQNKHLFLDELVIKIIISIEVTHCETFVVFDKDIDRFVNAFNKASVYSIWRSLHNKFVFAHI<br>ANESPESRNHFFEDQPNILFVVRDHSSASSFDIKTNKFVGRKAENPSQMILVDRYLASEQRFQF

GKSLFADKLNNLQGREVIIAGFDYPPYTVIKHNMSTNAQDMGVSGESDFKNVYIDGTETRIVL
NFCEQFNCTIQIDSSAANDWGKVYPNMSGDGALGMLINRKADICIGAMYSWYEDYTYLDLSM
YLVRSGITCLVPAPLRLTSWYLPLEPFKETLWAAILLCLCAEATGLVLAYKSEQALYVLPGYREG
WWTCTSFGVCTTFKLFISQSGNSKAYSLTVRVLLFACFLNDLIITSIYGGGLASILTIPSMDEAAD
TVTRLRFHRLQWAANSEAWVSAIRASDEALVKDILYNFHIYSDDELLRLAQDQHMRIGFTVER
LPFGHFAIGNYLGPQAIDQLVIMKDDIYFQYTVAFVPRLWPLLDKLNTLIYSWHSSGFDKYWEY
RVVADNLNLKIQQQVQETMTGTKDIGPVPLGMSNFAGFIIVWILGSAIATLTFLLELSLTYILKQS
NLK

>DmelIR47a
MRQIKLLVWLLVVGVVSSTEQLQFLKNFLEAVHKERSISTILLIQRKVHKNDFLHGLYPIFWPIIC
LDEKRVELVNNFNKDFLALVYMESEADTLLLSALAADLNHIRDARIMIWLQMSPSENFLDRIVF
QASKQFLNLVVIENTLKTRRFYPFPQPKVQVIDKPFEEKEIYPALWRNFMGKNAIAVPDLVPPRS
FNSFDPKTGHRRESGSIYNVFKAFTQRYNITMLLKWPLIRNTTQEEIIGKSVRGEIDLPITGQLISF
RHPNGSRSQPLLGMTALSIAVPCGPELPMFDRFFLFYGLATPITITGYYVLLNTIEIILGTLSDRIK
RHPRRKKILNLVLNLRVFSCILSLPTPQGNRLRSVKGQLTMVMSITGLILSCIVAAQTSTILTMKP
QYRHIKNFQELSDSNITVVCNHLNYLTIKQQMDPKFMAKFMQNIWIVNSIEQMKMIFDLNTSY
AYQTFSYKKDPFTLLQMHTTRKAFCRTPGLDLVSGLAYTAVLEKNSIYALALQDYTLKAFSAGL
VYYWAEESIRDLISTVGRTQFEKLPIVIGYQSLKLQDYNVCWKILLIGGALAFCVFIVEVVVGLI
NRRI

>DmelIR48b
MILQQSSNLLKLLLLLAISSVRTQGLNDIIIELNQRLLISNNFLYCNQSDKLNEYEIKYLQHMPPIS
LMIFTSIESMNFTQVEYNLGADNKLFLIMGNEEPPYDFLHALNLHFQFAEYIIVIDEPVDLKKST
KWLDFVNHLWQQGYVQLLIYTSYDEKLYHKIIFPETVIEETLVEQYISIRGSFNNLYGYPVRVAA
YNNAPRSMLYVNRWGKHIFAGFYMRFLRAFIDARNGSFVPVLTPSNSPGNCTLNLVNETVDVC
ADALAANPAAFSLTHGFRIASANVLVTHAKPLHSYRYLTAPFQWSVWACLVIYVLLVVNFLSFI
GWLRSGKWEFSKYLLEVFSSLLFSGFYLKEIRGRERYILFGVLFIAGFVYSTEYLGLLKSMLISE
VFEKQIDTFEALVESNITLMVDPYDKILFAKYNMPEILSPIMELVSFETLLKHRNRFDQDYAYILF
SDRMALYDYAQQFLKHPKLLRIPIDFSFLYTGIPMRKRWFLKHHLGRAWYWAFESGLTRKLAL
DADFEAVRVGYLSFLITEHVEAQPLNVDYFVMPAIALAIGYILALLSFVIEMTAWRIREFLGCRK
ATMTSTGCSEGGHVDVD

>DmelIR48c
MSLLRIILIIIFLRIVSSIPDTIISHLSAELQIKIQIYFGLGNDLYDFSRLDGNYQKIIISHNISEEFKTY
HDEPVLIIIRLERDLNLNLATLDVLRSYLTDRQYNDILLIDNDEENLNSYVDIRKAYWNAGFSQ
VLIYNSQQRTWSIKPYPYLQIRPTSLKEYIENRNTRNLMGYPLRVLVTNDPPHCFVDKDELPGSP
NRYKGSIVTMLKIFADQLNATFQANPFREFRRYSTADCVQMVSDDEIDACGSIFIRTYTYATSQP
VRLNRVVIMAPFGNIEKFYYFFRPFDLYVWIGTGIIVVYIAVMGSLLHRWHFKEWNVGQYLLL
AVQTLLNRELSLPQSSSGKFMLLLLLFAIGFILSNLYVALLSMMLTTKLYQRPIENLADLKAANV
NILLQTHNIRPNSVYGSSEELRERFLLVEESQHLEKRNGLDPSYAYVDSEDRMDFYLYQQKFLR
RRRMKKLSNPVGYTWAVQVIKQNWVLEKHYNDHVQRFFETGLQNKLVDDVHELAVKAGFL
HFFPTQTQTIEPLRLEDIVMAAMVLGGGHALAVICFLVELFA

>DmelIR51b

MCKVLTLLVVILLLALTNAAYNVTLLKSVLSLISTREPWINTPIFVGHNTQGGDLNDLIIWLHQT

MGVTSLTMNLFLQPEHIRPLGHFKITRYNGIALFFCHDKHDIMWLTLDRNLRKLRRIRLIIILRN<br>QRSGSQGAIKSIFNALWQYQFLNVLVLQRDQLYSYTPYPAMRFFKLDIHTEPLFPHAARNFHGY<br>VVSTPAENDIPRVFHVHDPLTKSRKVLGYAYRTFVEYLDHYNASLRLTNPDENLDPTTSVNMN<br>HIVQLIIDGQLEISLHPYVFTPPTATKSYPLLIYPNCLIVPMRNEIPRHMYLLRPFQLYSWYILLFA<br>VFYITGILYCISPKLNKSSWPQRLGLNFLDAISKILFISPPITIYRPTWRHLIIFLQLSVLGFMSTSW<br>YNIELDSFFTTIVVGEQVNSMDQLVHQQQRVLVKEYEINTFLRHVEPRLVEKVSRLLVPVNASE<br>QVSALLSFNRSFAYPFTEERWQFFAMQQQYAFKPIFRFSSACLGSPHIGYPMRVDSHLETSLNHF<br>ILKIQDTGLLNHWVVSDFNDAMRAGYVRFVDNVLGYQSIDVDTLRLGWCVLGIGWILSALVF<br>SCEYWHLYPWRFIA
>DmelIR52a<br>MALGWSVIILGFIGQLSAQILNYTQSRDLELLEGSLFRVLSRLNLEEEYNTLLIYGKECVFHSLL<br>RKLEISAVTVPSGSTDYDWSFSTAILILSCGYDAENEENSYTLMKLQRTRRLIYLEDNSEPESVC<br>MRYSLKEQHNIAMVKSDFDQSDTFYSCRLFQTPNYVEGHFFKDQPIYIENFQNMRGATIRTVA<br>DSLVPRTILYRDEKSGETKMMGYLGHMINTYAQKLNAKLHFIDTSKLGAKKPSVLDIMNWVN<br>EDIVDIGTALASSLQFKNMDSVWYPYLLTGYCLMVPVPAKMPYNLVYSMIVDPLVLSIIFVMLC<br>LFSVLIIYTQHLSWKNLTLANILLNDKSLRGLLGQSFPFPPNPSKHLKLIIFVLCFASVMITTMYE<br>AYLQSYFTQPPSEPYIRSFRDIGNSSLKMAISRLEVNVLTSLNNSHFREISEDHLLIFDDLSEYLVL<br>RDSFNTSFIFPVSVDRWNGYEEQQKLFAEPAFYLATNLCFNQFMLFSPPLRRYLPHRHLFEDHM<br>MRQHEFGLVTFWKSQSFIEMVRLGLASMEDLSRKRNEEVSLLLDDISWILKLYLGAMFISSFCF<br>ILEILRCGERCKRLWRCRW
>DmelIR52b<br>MTWLVILLCFLGYMAAHIADISVQNQSLMDNELINLLLKLRNEEFYDTLLVYGKDCEFHSVIK<br>NVDVAVVLVSDSMNFEWNFSSLTLILSCGPDIDNGGPNSTSIKLQRNRRLVLLKEDFQPSNICNI<br>YTQKEQYNIALVRENFTKSKSIYTCRYFQDPNVDEVNLSGTKPIFIEQFQNMKGKAIRIVPDLLP<br>PRVMLYQDANDGELKMIGYVANLITNFAQKVNATLQLDFLKPSTSITEISRMAKDDELDMGITL<br>EASLNTSNLETSSYPYLLTSYCLMVQVPAKFPYNLVYALIVDPLVLGIIFVLFLLLSVLLIYSQKM<br>SWQDLSVANILLNDKSLRGLLGQSFPFPLNASKKLRLIFTILCFASIMLTTMYEAYLQSFFTNPPS<br>EPEICSFQDVGSYNRRIAMSALEVNGLIKTNNSHFREIRMDDLEIFDNMPECYELRDAFNLSYN<br>YVVTGDRWRSYAEQQTLFKEPVFYFARDLCFSRLIFLSVPLRRHLPYRHLFDEHMMQQHEFGF<br>VNYWMSHSFFDMVRLGLTSLKDLSRPLAYTPSLLMDDISWIMKIYLAAIVLCVFCFLLEIGVDK<br>WKRWMKFRNLQILNTC
>DmelIR52c<br>MVWLIIILFCLGNSSSQILDVTNNSHLDFDYRLFGLLQRLQVEKSYDTLLVYGEDCAIPSLFERL<br>QVPAVLVSSGSTNFDWNFSSLTLILSCNFQDEREENYRTLMKLQTSRRLILLKGHIKPESVCDFY<br>SKKEQHNVAMVKENFYQLEVVYSCRLFQDQNYEKLNLFDGKSIYKDQFRNMHGAPIRTLSDK<br>EPPRTIPYIDSKTGEEKFKGYVGMLISQFVKKVNATMQIREDLIKDDEEVSFVDITNFTSNDILDI<br>GICEARTLEMSNYDAISYPYLMSSYCFMAPLPDSLPFSDVYMAIVAPSILIMFLIIFCICSVLIIYIQ<br>ERSYRSLTIRSVLMNDICLRGFLAQPFPFPRQYNRKLKLIFMLVCFSSLISTTMYTAYLQAFLWG<br>PPIEPRLTSFDDVKKSRYTMAINIYEREFLEALNVSLEDVEIYDYGKFSKLRSTFNTNYLFPVTAL<br>QWFTINEEQKLFKYKIFYYCDAFCLNQFDILSIPLRRHLPYRDIFEEHMLLQKEFGLTKYWIDQS<br>YRDMIRANLTTFKDFSPLLENDYIEVHNLYWVFTMYFVGMGMGLCFFILEILRPLRYWRNCKI<br>KCEYCYAFLKNFAK
>DmelIR52d<br>MVRIIIILLCLGYTKARILDATNTNHTDLEERLLSLLLRLQQEQFFNTLLIYGEDCAFSSLSRRLQ

VPTILVSSGSTSFEWNYSSLALILTCEFKAEREENYQTLKKLQMNRRLILLNGNIKPDSVCDFYS<br>KKDQYNIAMVNNNFHQVGIIYACRLFQERNYEKVYLSEGNPIYVDQFRNMQGALLKSITFNLI<br>PGSMAYRDPKTGQEKHIGYVANLLNNFVEKVNATLDMQVKLHKAGKKTSFYNITKWASEDLV<br>DIGMSYAAYFEMTNFDTISYPYLMTSTCFMVPLPDMMPNSEIYMGIVDPPVLVVLIAIFCIFSVM<br>LNYIKQRSWRSLSLVNVLLNDICLRGFLAQPFPFPRQSNRKLKLISMLVCFFSVITTTMYTSYLQ<br>SFMWGPPIDPKMCSFADLENSRYKLAIRRYDIEMLRPFNVSMDHVVVFDESSQLEYLRDSFDD<br>NYMYPMSALSWSAFKEQQKLFAFPLFYYSEKLCLKPISFFSFPIRRHLPYRDLFEEHMLQQNEF<br>GLSTYWIDRSFSDMVRLKLATMNDFSPPRLEDYIEVSDLSWVFGMYFTGLGISCCCFGLELLGL<br>PSWTRRLRLTNWLRVRN
>DmelIR54a<br>MWTVITGIVLWAPVLVAGSAVDFIFRAAAEHSLSVIMIRIDYCPYNWAKDIFENQTIPVVVLSDS<br>ETFINIRMFSRPLHVACLPGHELQKDLALLENFTSSLMDFPSQKKIVYISNNFSDPTRMDYIFETC<br>YHRRIWNIVGLLASDEHRYFYRYHLYPSFRTEYRSLESSTIFDKDFPNMHGHPLTVMPDQWLPR<br>SVLYVDRRTGKQILAGSVGRFFHVLSWKLNATLQLSKKVTTGRFLNATALKELSESFSVDVPAS<br>LTIMERVEQLASTSYPMEVTHVCLMVPVARRIPIKDIYFILSSASNMFLAIVIVSSYGLALNLLRN<br>MTHRDVRLVDFVLNDKALRGILGQSFNLPLSRSFSTRLIFLMLGIVGLNVSSIFGAGLDTLMAH<br>PPRQFQARSFAGLRRTKIPLVTTEEDFPTWMKLRVPMLVVNVSEYNHLRNGRNTSNAYFASRL<br>YWNLFSEQQKRFTRELFIYSTDDCLWSLALLSFQWPQNSLFTEPVSQLILEVNANGLYDFWVG<br>MHYYDMTAAGLSGLEDPSLQLKEREHPTSLRIVDFQWMWQAYGTFMVIAILVFLLEVSWHRI<br>TSLFVSLVY
>DmelIR56a<br>MGSRFFIRNLILFGLLASSNMQIPFGELEKKFELDVDFLLGVTELVGHIQGLYSITVYADCIDIHP<br>SIQQRIMDKFMVPVNTIGSNLSRPNYHKLDNSRIRIVLFTGLNDTILVNLNKTDVPYSDNFYML<br>AYASAIKNKCIELDFIEEVFTLLWKMSIQNAILLIRGEFMMEMWSYLYMGKIHKIKLTKPNSYLE<br>SLRKYNYRFSLEVINDPPAIFWYNSSEQADVTGGGNLSVSGPLGLIIINFLRHLNVTIDIVPIPGK<br>QTSQYELFQQPDNLRAENGVNMVGSALLKYSPMVTQSRMCLLVSNRRMIPFSRFLDRLVSPG<br>VHKLTFVSSIGIFVIKYFSHRPRSFVDAIFCTIRFFFAIPLPSIILNRLPVVDRFIEVFIIIFVQILLSSNI<br>SITTSALTTGFWEPPIINVETMRASGLHILTEDPTILQAFKENILPSSLADLVILVDEDTYFHHVTT<br>LNNSYVYVVQAHNWQIFRLYQQQMTNEPFEIASEELCSKWRILGIPLNPKSPLRFMFKDYFYRI<br>LESGLREQWVHSGFKKFCEFNNLKKLPVDSVDSWQPLSIEFYSNVIRAYIIGLVIATLAFVAELL<br>HNGYRRKNVKKT
>DmelIR56b<br>MLLDTDLASGVIRSPYSFDIPHAFIFNETQFVVPKFCGPYMEIVKHFAEVYHYQLFLDSLESLPK<br>KSVVEQDIISGKYNLSLHGVIIRPEETSDFFNATQHSYPLELMTNCVMVPLAPELPKWMYMVW<br>PLGKYIWTCLFLGTFYVALLLRYVHWREPGNATRSYTRNVLHAMALLMFSANMNMSVKLKH<br>ASIRVIIFYTLLYIFGFILTNYHLSHMTAFDMKPVFLRPIDTWSDLIHSRLRIVIHDSLLEELRWLP<br>VYQALLASPSRSYAYVVTQDAWLFFNRQQKVLIQPYFHLSKVCFGGLFNALPMASNASFADSL<br>NKFILNVWQAGLWNYWEELAFRYAEQAGYAKVFLDTYPVEPLNLEFFTTAWIVLSAGIPISSLA<br>FCLELFIHRRKQRRPQYERFECYDY
>DmelIR56c<br>MQHLLNLLAPFGRMNVFQEIVWFVSPHQRLDQLDEFIMRIDEAFGKSATQTVVNNNTEMRMI<br>YSSARRNHMSFVFTTGAEDPIMKVFSKVLLGRHFYVSMVIYVDKVGDMHPIYDLLTFAYNQQ<br>FFNSMVHFESMEGVNQLFGVSKFPVMSFENRTDFLKYMGKIWKQVQNARSDVGGFGFTTPLR<br>QDLPHLFQSQGHYDGSTYRIIETFVRFINGSFKELIMPPDSLGGQVINMKDALQLIRERKMEFCA

HAYALFMSDEELEKSYPLLVVQWCLMVPLYNSVSTYFYPLQPFDWNVWFFALGALLALVLLE<br>LMWLRMFGGWSGYRGAVLNSFCYIINVPIEGQLQQPCLLRFLLLATVFFHGFFLSAYYTSNLGS<br>ILTVNLFHAQINTMNDIVSAQLPVMIIDYEMEFLLNLNKELPQEFLELLRPVDSAVFSEHQTSFN<br>SSFAYFVTEDHWEFLDEQQKHLKQRLFKLSSICFGSYHLAFPLQMDSSLWRDIEYFTFRIHSSGL<br>LNFYARSSFGSALHAGLVQRMPDTQEYTSAGLQHLAIAFILLLVMSFLAGIVFVLETLSR
>DmelIR56d<br>MDNRAAELILRERNIFPTNGSDNITLLNNMFVLEMFYRITQLYHFKNFIFYISERLDLNNKDSQE<br>FFHNFWTYFPMAPNLIITREHHLGIPMMQFISTPSLVMVFTTGKDDPIMELASHNQQGIHWLKT<br>IFVLFPSLQSRDFETNPESLAQFTAEIKDVYDWVWRKQFINTFLITIKDNVFILDPYPTPSIVNKT<br>GVWQAEEFFHKYAKNMKGYLVRTPILYDMPRVFKSDRPTNRYEKNFIHGTSGNLFLGFLEFVN<br>ATLMDTSANVTADYLNMTNLLDLVSQGVYETLIHSFTEITTKFVVSYSYPIGINDCCIMVPYRN<br>QSPADQYMHEALQENVWVLISLFTLYITVAIYLCSPLRPRDLSAAFLQSICTLTYSVPTFIIRTPTL<br>RMRYLYILLAIWGIVTSNLYISRMTSYFTTAPPVRQINTVQDVVEANLRIKMLAIEYERMAKSPL<br>QYPESYLNQVDLVDKHMLDLHRDPFNTSFGYTVSSDRWRFLNLQQLHLRKPIFRLTEICEGPFY<br>HVFPLHKDSHMRSVMTEYIMIAQQAGLMNHWERETFWEAVHLHRIHVHLFDDEPMALSLDFF<br>SSLLRTWTLGLILAGLAFAAEMKWHEHVTFKRRPVIRITRKPRSFLRRFMKL
>DmelIR60a<br>MWCNNPGLIIIIFLGQILNLCQGIVNLSNETANTVIFMLPEKDLGPDVWKAGVGCLDSFAQIFFF<br>RNPKERFTRAYNLMLVHAFHLSSPADQIQEGFSKLINEAVTNPGPPDREELFQMRVASDYNITN<br>GTEDKGELILADNYVIVVDSVDRLKELMKKKIVEMRSWNPGARFLVLFHNATCRNRPLGVAS<br>NIFKDLMEMFYVHRVALLYANSTMNYNLLVNDYYSNVNCRILNVQSVGQCHDGKLYPNNAV<br>VKASMQDYVSGFSPRNCTFFACSSISAPFVEADCILGLEMRILGFMKNRLKFDVNQTCSLESRG<br>EMDGPANWTGLLGKVQNNECDFVFGGYYPDNEVADHFWGSDTYLQDAHTWYIKMADRRPA<br>WQALVGIFEAYTWIGFILILIISWLFWFTLVMILPEPKYYQQLSLTAINALAVTISIAVQERPICETT<br>RLFFMALTLYGLNVVATYTSKMIATFQDPGYLHQLDELTEVVAAGIPFGGHEESRDWFENDDD<br>MWIFNGYNISPEFIPQSKNLEAVKWGQRCILSNRMYTMQSPLADVIYAFPNNVFSSPVQMIMK<br>AGFPFLFEMNSIIRLMRDVGIFQKIDADFRYNNTYLNRINKMRPQFPETAIVLTTEHLKGPFFILV<br>VGSCWAALTFIGELIIHRWRTQLVSTSEQQDRRSDKRRRRRRRRKPEKDNRWQRQVQVAPVVR<br>FTPVKRRKVFQGQTSQK
>DmelIR60b<br>MRRSLYLIIAIGLVDVHCVSLRYILNALENELQYRAILLVESASEIESCWEQKYIQGAVPILNFNA<br>NQSLYLKDALNTNILALVCLNENVESTMQALYENLEDMRDTPTILFVLSDSKVQDVFLECLRR<br>KMLNVLAFKGLDRGFVYSFRAFPTFRVIERNVMDILQYFEQQLEDLGGHTLTTLPDNIIPRTVV<br>YKSPDGSRQLAGYLYPFLRNYVSTINATLKVCWHLVPEDGMIQLGEVVRLSEIHDVDFPLGMH<br>GIEHGSTSQNVPLEVSSWFLMLPMEPSLSRAQFFIMLGFEKVTPVLLLLTILLSTAHRIEMGLRPS<br>WRCYVLGDRVLQGTLGQAFFLPRRLSVKLMLVYSLILLNGFTFSNYSITSLETWLVHPPSGHPI<br>HSWEQMRTLNLKVLIVPSELDSMTKALGKQFTESNSDLFELSKSGNFQDKRLAMDQSYAYPVT<br>CTLWPLLEHAQIRLPKPEFRRSREMVLIPLLIMAMPLPKNSMFHKSLNRYRALTHQSGLYEFWF<br>KRSFNELVALRKIHYKVNGDHQIYRDFEWQDFSYVWLGFVGGTIASILVLLAEIGYHRWQLNQ<br>N
>DmelIR60d<br>MRLAIYVAFLSSIGNRSGFLSSLLMSLGKELHYKTILLVGGSSTCWSLEPFETGVPILNLRGENN<br>AYPQDTFNSQMLALACLQTESEDAVKLLYRSLKDMRDTPTLLFASSEEHIHDTLFLGCFRENML<br>NVLALTASSKEFIYSYQAFPTFRVIKRKLVEIHRYFEPQLKDLGGHIVSALPGNIMPRTMCYRNA

EGERQLAGYLNTFIRNYVESINGTLRISWGLVPEDDMRHLTISRLSKIQHVDFPLGIIPLYNKTDK<br>QHVYMEISSWFLMLPMETSVPRAHLFVKLGLERLLPIIVVVGAVLGNAHRIEVGLGPSWRCYY<br>LADKVLRGALAQPIVLPRRLSPKLMLIYSLLLLSGFFLSNYYMASLTTWLVHPPASDRILEWDQ<br>LRYLHLKVLTIPEEFKYMSLILGTDFMTAYGSIFQLTNSTDFQRRRISMDPSYAYPVTTSLWPFLE<br>LSQVRLRRPLFRRSYDMVLQPFQVMSLPLPRNSIFHKSLLRYAALTRETGLYYYWFRRSYYELV<br>ALGKISYKEEEGNPYCDLKWNDFRIVWLAFLGGTIISCLALLLEVAHYRWHLGNSSL
>DmelIR62a<br>MYLQFLFALFLSRYQIVATENFDRAFELALFLDRIGRVHRLHAITIVNSLGSVDPSYLDDLHRGL<br>MCNSSNHFYMLPQMTATDKDSSHVHFSSLQDEETIYLVFARDSKDAVIYLQAERARGRRYTRT<br>MFLLRKQESQKDIKYFFELLWKLQFRSALVVVAARNFYQMDPYPTVRVIRMRRLSSYDPHHVF<br>PPANRKNFRGYRMRLPVQQDVPNTFWYKNRRTKAWELAGLGGILINQLMMHLNVTMDLFRF<br>EVNGSSLLNMAALTDLIVKGKVELSPHLYDTLQSNTSVDYSYPTQVAPRCFMIPLDNEISRSLY<br>VFLPFSLTMWLCLLFVLLVVHFVYVRRLIPDGHFWAILGVPGAGQVRYGNRKPVRRFSTFLILF<br>GIFILGQTYSTKLTSSLTVTLIRRPDNSLEELFLLPYRILVLPTDVYAIVDSLGHAEQFSTKFSCTD<br>AENFSQKRISMHPEYIYPISTIRWRFFDMQQRFLRKKRFYFSKICHGSFPYQYQLRVDSHLKDA<br>LHRFLLHVQQAGLHDLWLDTCYRKAHRMGYLKDFSTLAELEEKLRLRPLALNLLVPAFSLFLC<br>GMLGSGIAFLVEIRHSFGCRQKPPSINRNPGD
>DmelIR64a<br>MHWWLLVFLPLSCQGLPEHELLELELDYGLAEPQRTSLLQSSLILQFSQDYKHIPRITYFTCQKP<br>HLQTPNQIPNAAEHRDAFAAKNFQLIKSLYESELFVRIVLLDVLAQSPTSGRPNRPGNGPTGGFS<br>QTPSQAQSNSEWLEGVLRMEALRQIAVVDLACGAVSRRFLELASAKMLYSEKFHWLLIEDFAW<br>HGRTQTAEGSGKRDDGEMEEEEPPGQQIQATDDEDLPSIESFLGGMNLYMNTELTLAKRMSEA<br>AHYTLFDVWNPGLNYGGHVNLTEIGSFTPTEGIQLHTWFRTTSTVRRRMDMQHARVRCMVV<br>VTNKNMTGTLMYYLTHTMSGHIDTMNRFNFNLLMAVRDMFNWTFVLSRTTSWGYVKNGRF<br>DGMIGALIRNETDIGGAPIFYWLERHKWIDVAGRSWSSRPCFIFRHPRSTQKDRIVFLQPFTNDV<br>WILIVGCGVLTVFILWFLTTIEWKLVPHDGSALIKPKGGAPPRHHYQQQQQQEQVEAPVRPITA<br>VSVVVSKEKVEEKQEEYEDSTPIDAGTLWQRCYQKLNKYIKDRKAKQKKAPERVGLFLESVL<br>FFVGIICQQGLGFSTSFVSGRCIVITSLLFSFCIYQFYSASIVGTLLMEKPKTIKTLSDLVHSSLKV<br>GMEDILYNRDYFLHTKDPVSMELYAKKITSVPTTKENEADEDEPVDPNPVSTDPAKSYRDIVHS<br>HETGAHAKDNAASNWLDPETGLLRHLGFAFHVDVAAAYKIIAETFSEQDICDLTEVSMFPPQK<br>TVSIMQKNSPMRKVISYGLRRVTETGILTYHFNVWHSRKPPCVKKIETSDLHVDMDTVSSALLI<br>LLFSYAITLMILGTEILYSKWHNRIQLKWVGAT
>DmelIR67b<br>MELLYLNTLQSLSLLEGNRLVQTVQELNNIYQTELNVFLEFGNGADILESAQGTFVPTLWIKNP<br>QNQKVMKGNFTSCTLTILYLEDEHLDRGLYYLANWLWEYHHLEVLIFFNGGSYDKLIQIFSRCF<br>NEGFVNVLVMLPGSDELYTFMPYQDLKILNLKSIKEFYSLSRKKMDLNGYNITSGLVIAGAPR<br>WFSFRDRQNRLILTGYMLRMIVDFTNHFNGSVRLMNVLTVNDGLELLANRTIDFFPFLIRPLKS<br>FSMSNILYLENCGLIVPTSRPLPNWVYLLRPYAFDTWIAWLIMLIYCSLALRILSKGQISISAAFL<br>KVLRLVMYLSGSRDMGTRPTTRRLFLFVILTTSGFILTNLYVAQLSSNSAAGLYEKQINTWEDL<br>DKSDSIWPLIDVDIKTMEKLIPDRTKLLKKIVPTLEADVDTYRRNLNTSCIHSGFFDRIDFALYQ<br>QKFLRFPIFRKFPHLLYQQPLQISAAFGRPYLQLFNWFVRKIFESGIYLKMKDDAYRHGIQSGLL<br>NLAFRDRHLEVKSNDVEYYYLIAGLWFGGLTLATVCFLLELLIGYAKIKVTISCKMNIM
>DmelIR67c<br>MFCWLIFLNIILLSDRSESWSAREVIHQFNHDQQLQLNIYLDCNDVELQIGQEVSNLFVNSTAD

KMKILGRFSSHSLIIACFKDSTRNRTLNGVKELLWGLQYLPILFVVDSNMDFYFQQALRHGFIH<br>VLALNFMNGSLYTYKPYPKVEVHQIKDMQKFYKLTKLRNLQGQAVRTTVETMTPRCFRYRNR<br>HGQLVYAGYMYRMVKEFISTYNGTEEHVFGNVDTVPYKEGLAALKNGEIDMMPRIIHALEWY<br>YFYRSHILYNIKTYIMVPWAEPLPKSLYFIQPFRGTVWITIMVSFVYASIVIWWIRYRQQGNSSLT<br>QSFMDVLQLLFQLPLSKIWHFNMGTHQVVSFIVLFVFGFMLTNLYTAQLSSYLTTGLFKSQINT<br>FDDLFREKRTLLVESFDAEVLHNMTKEKIIQKEFESIILITSIEEVFKHRKSLNTSYAYEAYEDRIA<br>FELSQQRYLRVPIFKILKEVYDQRPVFVALRHGLPYVELFNNYLRRIFESGIWIKLQEDSFLEGIA<br>SGEISFRKSKSREIKIFDKDFYFFAYILLGMGWCVSTIALFLELWSFKYSVTNVLHEG
>DmelIR68a<br>MRCLWILIVAFISLAMATSIPIPIANPAPLSGYEMQLKILLQKILWVANVKRCFAVITDDLHYPIYD<br>RIFFESVGRRVIPFFVMRTNESDDLQRPSRQVELFVKAIKSSDCELNVITILNGWQVQRFLGYIY<br>DNRSLNMQKKFVLLHDLRLFESDMIHLWSVFIDAIFLKRQLDNKYTISTIAFPGILSGVLVMKNI<br>ANWELGKGLNGRILFADKTSNLFGTSLPVAISEHVPMVLWANATKSFQGVEVEIMNALGKALN<br>FKPVYYKPNQTENMDWTELDGGASVAYGSGNPDGYAQNGTHIDSMLVDEVAAHSARFAIGDL<br>HLFQVYLKLVELSAPHNFECLTFLTPESSTDNSWQTFILPFSAGMWVGVLLSLFVVGTVFYAISF<br>LNAIINGNVSSEFFRCLRPNRNVPMDPKIYRRISFRIAISRYRSSKGDRMPRDLFDGYTNCILLTY<br>SMLLYVALPRMPRNWPLRVLTGWYWIYCILLVATYRASFTAILANPAARVTIDTLEDLLRSHIPP<br>STGATENRQFFLEANDEVARKVGEKMEVFGYSDDLTSRIAKGQCAYYDNEFYLRYLRVADESG<br>SALHIMKECVLYMPVVLAMEKNSALKPRVDASIQHLAEGGLIAKWLKDAIEHLPAEALAQQE<br>ALMNIQKFWSSFVALLIGYVISMLTLLAERWHFKHIVMKHPMYDVYNPSLYYNFKRIYPQH
>DmelIR68b<br>MKFLVGLLLQWYLPGIYALAEIACRIAVEQNVQVTYLYRCASCPASFDADYSALELDLYRCVG<br>SRLPVITRNMEAHELEPFRRTDSLSIFQIPAAEKGDSLVRRILDMLNPHQRRKHMHKYLFVWPN<br>AGRHQLLRLFRGSWAKKLLYGLAITGRENGTFDFDPFAWGGLQVIQRLDGEVPYARKVKDLR<br>GYPLRFSMFTDPLMAMPRSPVETAGYQAVDGVAARVVGEMLNASVTYVFPEDNESYGRCLPN<br>GNYTGVVSDIVGGHTHFAPNSRFVLDCIWPAVEVLYPYTRRNLHLVVPASAIQPEYLIFVRVFRR<br>TVWYLLLVTLLVVVLVFWVMQRLQRRIPRRGVIQFQATWYEILEMFGKTHVGEPAGRLSSFSS<br>MRTFLMGWILFSYVLSTIYFAKLESGFVRPSYEEQVDRVDDLVHLDVHIYAVTTMYDAVRSALT<br>EHQYGLLENRSRQLPLGIATSYYQPVVRRRDRRAAFIMRDFHARDFLAITYDSQAERPAYHIAR<br>EYLRSMICTYILPRGSPFLHRLESLYSGFLEHGFFEHWRQMDLITRVGASPDAEEFLEDLGDQT<br>DTDSGSNELAIRNKKVVLTLDILQGAFYLWSVGIGISCLGFAVEHAHWFWRRQTLRNAVEART<br>S
>DmelIR75a<br>MQLVQLANFVLDNLVQSRIGFIVLFHCWQSDESLKFAQQFMKPIHPILVYHQFVQMRGVLNWS<br>HLELSYMGHTQPTLAIYVDIKCDQTQDLLEEASREQIYNQHYHWLLVGNQSKLEFYDLFGLFN<br>ISIDADVSYVKEQIQDNNDSVAYAVHDVYNNGKIIGGQLNVTGSHEMSCDPFVCRRTRHLSSLQ<br>KRSKYGNREQLTDVVLRVATVVTQRPLTLSDDELIRFLSQENDTHIDSLARFGFHLTLILRDLLH<br>CKMKFIFSDSWSKSDVVGGSVGAVVDQTADLTATPSLATEGRLKYLSAIIETGFFRSVCIFRTPH<br>NAGLRGDVFLQPFSPLVWYLFGGVLSLIGVLLWITFYMECKRMQKRWRLDYLPSLLSTFLISFG<br>AACIQSSSLIPRSAGGRLIYFALFLISFIMYNYYTSVVVSSLLSSPVKSKIKTMRQLAESSLTVGLE<br>PLPFTKSYLNYSRLPEIHLFIKRKIESQTQNPELWLPAEQGVLRVRDNPGYVYVFETSSGYAYVE<br>RYFTAQEICDLNEVLFRPEQLFYTHLHRNSTYKELFRLRFLRILETGVYRKQRSYWVHMKLHC<br>VAQNFVITVGMEYVAPLLLMLICADILVVVILLVELAWKRFFTRHLTFHP
>DmelIR75b

MLQLHNLILHNLIHMAKLSHVLILHCSLSHLALLAQSKNIFTQFQPLHSDIQLNDDFLNHNILKL<br>GVFLDINCDKSGTVLDMASAKRFFSHRYHWLIYDRSMNFSVLESHFKEAQIFVDADVTYVTH<br>DPFSKNFLLYDVYNKGRQLGGELNITADREIFCNKTNCRVERYLSELYTRSALQHRKSFTGLTM<br>RATAVVTALPLNVSIKEIFDFMNSKYRIQLDTYARLGYQARQPLRDMLDCKFKYIFRDRWSDG<br>NATGGMIGDLILDKADLAIAPFIYSFDRALFLQPITKFSVFREICMFRNPRSVSAGLSATEFLQPF<br>SGGVWLTFALLLLLAGCLLWVTFILERRKQWKPSLLTSCLLSFGAGCIQGAWLTPRSMGGRMA<br>FFALMVTSYLMYNYYTSIVVSKLLGQPIKSNIRTLQQLADSNLDVGIEPTVYTRIYVETSEEPDV<br>RDLYRKKVLGSKRSPDKIWIPTEAGVLSVRDQEGFVYITGVATGYEFVRKHFLAHQICELNEIPL<br>RDASHTHTVLAKRSPYAELIKLSELRMLETGVHFKHERSWMETKLHCYQHNHTVAVGLEYAA<br>PLFIILLGAIILCMGILGLEVIWHRHCTLH
>DmelIR75c<br>MTSWPLYRLIVFNLLEINLSNLMVFHCWSIKEAFPLVEMLNQNGIFSQYIDVQNPDNLANVHK<br>EYLDSDLVRLGVFLDLGCDKAELVTNQSSRARLYNQNLHWLLYDEAGNFTKLTQLFEGANLS<br>LNADVTYVSREDEERFILHDVYNKGSHLGGKLNITVDQTLQCNRSHCQVKEYLSELHLRPRLQ<br>HRMDLSSVTFRLAALVSVLPINSSEEELLEFLNSDRDSHMDSISRIGNRLIMHTQEILGFKLHYI<br>WCGTWSVQDAFGGAIGMLTNESAELCTTPFVPSWNRLHYLHPMTEQAQFRAVCMFRTPHNA<br>GIKAAVFLEPFMPSVWFAFAGLLIFAGVLLWMIFHLERHWMQRCLDFIPSLLSSCLISFGAACIQ<br>GSYLMPKSAGGRLAFIAVMLTSFLMYNYYTSIVVSTLLGSPVRSNIRTIQQLADSSLDVGFDTVP<br>FTKTYLVSSPRPDIRSLYKQKVESKRDPNSVWLSPEEGVIRVRDQPGFVYTSEASFMYHFVEKH<br>YLPREISDLNEIILRPESAVYGMVHLNSTYRQLLTQLQVRMLETGITSKQSRFFSKTKLHTFSNSF<br>VIQVGMEYAAPLFISLLVAYFLALLILILEICWARYAKKKFSTIIPQNQ
>DmelIR75d<br>MKVQVAHWLPLIFFLLVSGTPRVAGSWRSEYSRQDPDPKTRWGNQLPDMLVAYYRHHGVHSL<br>MLVVCHTDIADFRLWKLWQHFNLNNFYVQVSTESSLRDLQHVDALDEHKDAPPPKSFHANNS<br>THWETSFLLPALPYKMGILLLEFSSECALNLLRWSAASEHNYFTTNRFWLLLTEDPGDIDLLED<br>PEIFIPPDSELRVLHYENVGNFSCSLIDLYKVAAWKPLKRTLVGHNIRNSRHVIHALQHFGSAITY<br>RQDLEGIVFNSAIVIAFPDLFTNIEDLSLRHIDTISKVNHRLMLELANRLNMSYNTYQTVNYGW<br>RQPNGSFDGLMGRFQRYELDLAQLAIFMRLDRIALVDFVAETYRVRAGIMFRQPPLSAVANIFA<br>MPFENDVWVSILMLLIITTVVLVLELFFSPHNHDMSYMDTLNFVWGAMCQQGFYVEVRNRSA<br>RIIVFTTFVAALFLFTSFSANIVALLQSPSDAIQSLSDLGQSPLEIGVQDTQYNKIYFTESTDPVTK<br>NLYHKKIASKGENIYMRPLLGMEKMRTGLFAYQVELQAGYQIVSDTFSEPEKCGLMELEPFQL<br>PMLAIPTRKNFPYKELIRRQLRWQREVSLVNREERKWIPQKPKCEGGVGGFVSIGITECRYALGI<br>FGCGAAVSFVLFLFEFIFRHFKQVYRIIKGYREVQR
>DmelIR76a<br>MENLLVESYYFSTVLSFFAQQFFADSHATCIFWHPAFDFRLETVHPMPLIIMDWHRWANRSDQD<br>VYDYKIKEDEFEGKGIPYNDWTLRLTVAIERSHCETFIAFQEQIPEFARYFYHASIYSIWRSLRNR<br>FMFVYTKEFEDKKDSYLSGYIFQDQPNILVITSQYLNSSTFEIKTNRFVGPRNFNKNPEPVEFYIL<br>QRFDAKGTKATWETQSAMSSKMRNLKGREVVIGIFDYKPFMLLDYEKPPLYYDRFMNTTDVT<br>IDGTDIQLMLIFCELYNCTIQVDTSEPYDWGDIYLNASGYGLVGMILDRRNDYGVGGMYLWYE<br>AYEYMDMTHFLGRSGVTCLVPAPNRLISWTLLLRPFQFVLWMCVMLCLLLESLALGITRRWEH<br>SSVAAGNSWISSLRFGCISTLKLFVNQSTNYVTSSYALRTVLVASYMIDIILTTVYSGGLAAILTLP<br>TLEEAADSRQRLFDHKLIWTGTSQAWITTIDERSADPVLLGLMEHYRVYDANLISAFSHTEQM<br>GFVVERLQFGHLGNTELIENDALKRLKLMVDDIYFAFTVAFVPRLWPHLNAYNDFILAWHSSG

FDKFWEWKIAAEYMNAHRQNRIVASEKTNLDIGPVKLGIDNFIGLILLWCFGMICSLLTFLGEL
WRGQG

>DmelIR84a
MIKLQVKVISWPLIILTAFLRVLQIESINTNFLELAAFEDFLRSEHLSHVLVVRGDDADGDWKIE
CHQKLLANYRVQFYRPEMSANFEDLMFYGSPRTAVLVLNSEHVLVRRQVFGVASEAGYFNNSL
AWFILGSGRESLPVEQLIDQLLSGYRMGIDADITVALRGPDNASMLFYDVYRISRQANTPLIIEK
KGLWTHSGGYQKFGNFKNTWVIRRRNFLNVTLIGSTVLTEKPPGFGDMEYLADDKQLQQLDP
MQRKTYQLFQLVERMFNLSLAISLTDKWGELLDNGSWSGVMGQVTSREADFAVCPIRFVLDR
QPYVQYSAVLHTQNIHFLFRHPRRSHIKNIFFEPLSNQVWWCVLALVTGSTILLLFHVRLERML
SNMENRFSFVWFTMLETYLQQGPANEIFRLFSTRLLISLSCIFSFMLMQFYGAFIVGSLLSESAR
SIVNLQALYDSNLAIGMENISYNFPIFTNTSNQLVRDVYVKKICKSGEHNIMSLQQGAERIIQGR
FAFHTAIDRMYRLLLELQMDEAEFCDLQEVMFNLPYDSGSVPKGSPWREHLAHALLHFRATG
LLQYNDKKWMVRRPDCSLFKTSQAEVDLEHFAPALFALALAMVASALVFLLELFLHWLPDFR
RRLGTMST

>DmelIR85a
MSIQWLKHILLLAILVNLAGTRENHIPLDLKKSSIVMVKMSQILCKARIKVLFVYFENQTSHEH
TGQILKEVTKCDISNQNTPLEAVKDDGILMYMVMITTNISQPLELSLIRKKSAAKHRSHVFLLV
RDADTVSDAWMRASFRQFWKIWLLNIVILYWRDGRLNAYRYNPFMDNYLIPVDNKPNEVPTL
EQLFPKTIPNMQRKPLRMCIYKDDVRAIFWRQGTILGTDGLLAAYVAERLNATMMITRPHSYN
NHNLSSDICFLEVAKEYVDVAMNIRFLVPDTFRKQAESTVSHTRDDLCVIVPKAKTAPTFWNIF
RSFGSLVWALILVSVLVANVFCYILKSEVGRVPMQLFAGALTMPMTQIPPNHSIRLFLIFWLYFG
LLICSAFKGNLTSMMVFQPYLPDINQLGALARSHYHIIIRPRHVKHIQHFLTLGHKHESRIREQM
LEVSDTQMYEMMRNNDIRFAYLEKYHIARFQVNSRVHMHLGRPLFHLMNSCLVPFHAVYIVP
YGSPYLGFLDSLIRSSHEFGFERYWDRIMNSAFIKSGVKVVNRRRGSGNDEPVVLKLQHFHAVF
ALWLVGIGMACIVLAWEHLTHNYNLAVTKRRD

>DmelIR92a
MLLQPLVMHLSQLLRIIVGQYFAEFPSILIVYNNSASTTPLQLEYLSALELVLRELSKPIRLQWIN
VAFLKDLNDLEDQVMGALNSSVTEGFITILSQTHHFIHARYYATRNANVRLKDKRYLFLCEDES
PAELLCMDILQFYPHHLMVRPGTETAPTGPTGPHPDPRRGGGASVSTKNKDDGEGGAGNKTTS
PYRDINFELWTQKFVGAVGNLDALLLDAFLPNETFANRVELYPNKLLNLQRRSLLVGSITYVPY
TITNYVPAGQGDVDPIHPQWPNRSLTFDGAEANVMKTFCQVHNCHLRVEAYGADNWGGIYD
NESSDGMLGDIYEQRVEMAIGCIYNWYDGITETSHTIARSSVTILGPAPAPLPSWRTNIMPFNNR
AWLVLISTLVICGTFLYFMKYVSYRLRYSGTQVKFHHSRKLEKSMLDIFALFIQQPSAPLSFDRF
APRFFLATILCATITLENIYSGQLKSMLTFPFYSAPVDTIEKWAQSGWKWSAPSIIWVHTVQSSD
LETEQILARNFEVHDYSYLSNVSFMPNYGFGIERLSSGSLSVGDYVSTEALENRIVLHDDLYFD
YTRAVSIRGWILMPELNKHIRTCQETGLYFHWELEFIDKYMDKKKQEVLMDLANGHKVKGAP
QALDVRNIAGALFVLAFGVAFAGCALVAELLIHRMDLSK

>DmelIR93a
MNPGEMRPSACLLLLAGLQLSILVPTEANDFSSFLSANASLAVVVDHEYMTVHGENILAHFEKI
LSDVIRENLRNGGINVKYFSWNAVRLKKDFLAAITVTDCENTWNFYKNTQETSILLIAITDSDC
PRLPLNRALMVPIVENGDEFPQLILDAKVQQILNWKTAVVFVDQTILEENALLVKSIVHESITNH
ITPISLILYEINDSLRGQQKRVALRQALSQFAPKKHEEMRQQFLVISAFHEDIIEIAETLNMFHVG
NQWMIFVLDMVARDFDAGTVTINLDEGANIAFALNETDPNCQDSLNCTISEISLALVNAISKITV
EEESIYGEISDEEWEAIRFTKQEKQAEILEYMKEFLKTNAKCSSCARWRVETAITWGKSQENRK

FRSTPQRDAKNRNFEFINIGYWTPVLGFVCQELAFPHIEHHFRNITMDILTVHNPPWQILTKNSN
GVIVEHKGIVMEIVKELSRALNFSYYLHEASAWKEEDSLSTSAGGNESDELVGSMTFRIPYRVV
EMVQGNQFFIAAVAATVEDPDQKPFNYTQPISVQKYSFITRKPDEVSRIYLFTAPFTVETWFCLM
GIILLTAPTLYAINRLAPLKEMRIVGLSTVKSCFWYIFGALLQQGGMYLPTADSGRLVVGFWWI
VVIVLVTTYCGNLVAFLTFPKFQPGVDYLNQLEDHKDIVQYGLRNGTFFERYVQSTTREDFKH
YLERAKIYGSAQEEDIEAVKRGERINIDWRINLQLIVQRHFEREKECHFALGRESFVDEQIAMIV
PAQSAYLHLVNRHIKSMFRMGFIERWHQMNLPSAGKCNGKSAQRQVTNHKVNMDDMQGCFL
VLLLGFTLALLIVCGEFWYRRFRASRKRRQFTN

>DmelIR94a
MALPKQLKFINIFLVLLIIYGSSDGTENQHEIFLNRLLQAVHNERSVETLFLLHHSNLANCSLQD
WNPPRIPTIRSNELTVFNVEKTFNHNALALVCLMKNSYREILNTLAKSFDCMRQERIILMIHRKS
DSKFIEDITHEVKNLQFLHLIVLIVQEKYNGQVFASTLRLQSFPEPHFKRIRNVFAIQRIFYRPINF
HGKVLNAIPNDIPILFVALNEMFTEYARRYNSTLRIQNRTIKEDIEITEDNYDIDMKIQLHNSQNF
LHHMNIAMDIGSNSLIILVPCATELRGLDIFKELGVRTLTWLALLFYIIFVLVEMLFVFISNRFNG
RNFTMRYTNPLINLRAVRAILGQTSPISNRYSLSIQHFFVFMSLFGTLFGGFFDCKLRSFLTKRPY
YSQIENFSELRKSGVTVVVDHTTRQFIEQEINANFFRDEVPNVRTTTIQELINHVYSYDRKFAFV
ANSIPWRTFREEMKSINQKILCDSKNLTILENVPLTFSIRRNAIFSHHLRNFIINAADSGMITCWF
KMAGKVIRKHIKTTLRESEQQPSHLPLSFDHFKWLWAVLCIAYVMSFMVFVMEILWSKYQRRT
RSVSIV

>DmelIR94b
MSLIFNLLFILILSQAVSQETEFLQLKYLNNIVRSMIKLHKMETLVIVKHHLDNNCSLQNWNAH
GMGIIRTNDQGKLIMKDTFNSRTLAIICIGQNSHITLLRNVFETFGKVQQKKIILWTQMELKEKF
FQEISKKSRDLKLLNLLVLKAVTKDKLLIYRLNPFPSPHFKRIENIWTPNDTLFMDTKFNFHGMT
AVVKHDYNWTIQMGNIRKFPISRIEDKEVIEFALKYNLTLQFFNDVERFDIELRKRIILKSNSTQP
IDSGIPMVFSSLLIVVPCGNYLSIQDVIKVSGIEKWIFYIILVYVIFVLIEITFLGVTILISRQSRHQM
IPNTLVNLCAFRAILGLPFPETRRTSLSLRQLFLAIALFGMIFSIFINCKLSSMLTNPCPRPQVNNF
EELKTSGLTVVMDHDAENFIEKEIGVDFFNQYMPRKVTLTFTERAKLLFSLKGNHAFTLFSESF
AIIESYQRSKGLRAHCTSEDLIVAERVPRIYILENNSILDRPLRRFIRQMQESGITNHWLKNIPSSL
EKNLMQITIPYDRERVHPLSIEHLTWLWCILILGYSISMIVFFVEMSLKRRKKNLENRAPNICIC

>DmelIR94c
MSKVFKLLVLPLIYLSLTKGSKNPQLKFLRELINVIEEGREIRTIMVIKHSRDEYCHLDQWNPRG
SPILRTNEMGSIRISGYFNDQAVILACMGENSDYGLLKSLANAMDNMRQERIILWSEREPTKML
MDYISQQADRYNFAQIIIVTMNEDVDAVPSLHQLNPYPTPRFRQITNISNIRRTSFFGCGLSFQGK
TAILKESVVSNIRFKVWSPSGPIPLSELKDYEIVQFAVKYNLSLKLYDQNESKSDHFDIQLGPLFI
TKDFPTQMAFVSPNTACSLIVIVPCSPKWRFMDVLHKLGVLKLIGCLLIAYAVFVLIETLILWLT
HRISGREVRLTSLNQLLNPRAFRGILGLPFPEFRRSSISLRQLFLVISVFGLVYSNFVSCTLSALLT
KPAQNPQVRNFKELRDSGLITIMDKYTHSFIEKHIDPEFFDHVLPHYLILQKKEALRMIWNFND
SYSYVMYTTTWKSLNTVQKSFDERVFCESESLTIAWNLPRMYVLGNNSVLKWMLSRYITYMP
QTGIPDSWTEQLPKVLKLLYNVTSPRRIKEGAVPLSIQHLSWIWHLLFIGESIATLVFIVEILLQKS
NQHTSNMRERSSEDDDFV

>DmelIR94d
MGQLHLLLVALVLLSPGGDSFYHSLIHHLNRELKIEYVLLLGNFDTTWLDILWQLPVSVLQIKE
HSRETYSLLENPSHNVLTIAFVNDSPEDILEILYRNLRMLNTQPVLLVIRKSTIRVNSLLEWCWH
HQLLKVVAIAQDFMESLIVYSYNPFPVLQFIERRLDNSTVIFEKRLENLHGYEVPIALGGSSPRLI

VYRDLEGKLIFSGPVGNFMKSFEQRYNCRLVQPYPFDESAISPARDLIASVQNGSVQIALGAIYP
QVPYTGYSYPIELMSWCLMMPVPEEVPHSQLYSMVFSPMAFGITIVAMVLISLTLSMALRLHG
YRVSFSEYFLHDSCLRGVLSQSFYEVLRAPALIKAMYLVICLLGLLITSWYNSYFSTFVTSAPRF
PQLTSYESIRHSNIKIVIWKPEYEMLLFFSENMEKYSSIFQLQEDYKEFLHLRDSFDTRYGYMMP
MEKWSLMKEQQRVFSSPLFSLQDDLCVFHTVPIVFPMVKNSIFKEPFDRLILDVTATGLLSRWR
DMSFTEMIKAGQLGLEDRGHPKEFRAMKVGDLIQIWRFVGWMLGLATIVFLLELICFWRHKM
WQNMKYMFCRNKNI

>DmelIR94e
MDCPKWILSGLCLISLVSGATVIELLGTLKLELDFEYVLLMKNRNFSLSDQVWNGTSLTKDVM
DEVQVPVLQFNENVSYFLHNSISRRLVTLGFMSDANLDEHRGLLTALVANLRHMTTSRVIFLVQ
SKASTDFLYELFRNCWRKKLLNVIVIFQDFETTSTFYSYSNFPILQIEERIYETSLQTLPIFPDRLR
NLHGYEMPVILGGTAPRMIAYRNKKGNVVYDGTVGHFMTAFQQKYNVKFVQPLQAKNPLDF
APSMQTVGAVRNETVEISISLTFPTIPPFGFSYPYEQMNWCVMLPVEADVPPFEYYTRVFELAAF
LLTLGTLVLISCLLASALSLHGYATNISEFLLHDSCLRGVLGQSFVEVFRAPTLVRGIYLEICVLGI
LITAWYNSYFSSYVTSAPKQPPFRTYDDILASKLKVVAWKPEYAELVGRLLEFRKYETMFLVEP
DFNRYLALRDTLDTRYGYMITTNRWVLINEQQKVFSRPLFQKRDDFCFFNNIPFGFPLHENSVF
MEPVQKLIMELAETGLYYHWITTGFSELIDAGEMHFVDLSPHREFRAMQIQDLQYVWYGYAF
MVVLSSLVWLLENLAYTVKSKTIFPTHFMQRNKK

>DmelIR94f
MWQQVLLAETSNWFRSDVLQRFWTHLRVEIRFRTMLNYRLESCDCWFDNVLGSDNSTALLW
NDQTYPHYLRRRQDTDILVVSCLRFHQYQEVLLALSLMLDQMRSMPVVLQLCGDEDSMQEL
NSARLLLKHSQDLKMPNVVLLSSTFFTSATLYSYEMFPEFNVQKLVYQAYLTLFPYKLGNLKG
HPIRTVPDNSEPLTIVRKTLNGSIAIDGLVWQFMIEFAKHINATLQLPIEPHPEKSIKLVQILDLVR
NQTVDIAASLRPYSLNVQRSSTHIYGSPMMVGNWCMMLPTERVIGSHEALTRLMKSPWTWLI
LLLFYSVHRFLAQKTRLRSSLIHLIKLLINLSLICFLQAQLSAYFIGPQKVNHISNMQQVEESGLK
IRGMRGEFMEYPIDMRSRYASSFLLHDLFFDLAQYRNSLNTSYGYTVTSVKWELYKEAQRHFR
RPLFRYSEEICVQKLSLFSLIQQSNCIYCYRSRIFILRMHEAGLIRLWYRRSYYVMVTAGRFPIGD
LSTVHRAQPIRWTEWQNVVLLHGVGLLFSVVVFVIELTVHYANVCLNNL

>DmelIR94g
MSTAVNSVHSKLVSLISRGQELTSIFFYAPAKEKCHLEDTISSATWGLPLVIWRTDRTVILNGFIGE
GLLVLACLPGFHWRALLGSLARSLKYLRQARILIELMQDRDEFLVSEVLQFCLSQDMINVNAIF
DDFPETENLSSFEAYPSFEVVNQTFTPDTQVSDLYPNKMLNLRGGVIRTMPDYSEPNTILYQDK
EGNKEILGYLWDLLEAYAHKHNAQLQVVNKYADDRPLNFIELLDAAQSGIIDVGASIQPMSMG
SLSRMHEMSYPVNQASWCTMLPVERQLHVSELLTRVIPYPTLALLLLLWIFYEVLRGRWRRHS
RLQSIGWLVLATLVSSNYVGKLLNLFTDPPSLPPVNSLAALMESPVRIISIRSEYSAIEFTQRTKY
SAAFHLALHASILIGLRNAFNTSYGYTITSEKWKIYEEQQKRSSKPVFRYSKDLCFYEMIPFGLV
IPENSPHRAPLHSYTLLLRQAGLHDFWVNRGFSYMVKAGKINFTAVGERYEAKTLTITDLRNVF
IIYVSVLLISLILFTCELFVSWVNYWLGF

>DmelIR94h
MLSNISFSSAPELVDLYGLVLKFLVSSETTLFYFNPTGQKCSWETLPRTILSNHPQIIWFREETYP
GLYKRHSSNLFVMACLSSTSYDGQLQLLAESLTRYRSVRVLIEVQDKEGSFLASQILLLCQQHS
MLNVVLYFSRWTRTLNVFSYLAFPYFKLLKQRLSGSLRPKIFINQLKDLQGYKIRVQPDLSPPNS
FSYRDRHGECQVGGFLWRIVENFSKSLKGDTQVLYPTWAKAKVSAAEYMIQFTRNGSSDIGVT

TTMITFKHEERYRDYSYPMYDISWCTMLPVEKPLSVEILFSHVLSPGSALLLILAFILFFLIVPQLI<br>KCLGITFRGRLIGMASRIFALVMLCSSSAQLLSLLMSPPLHTRIKSFDDLLTSGLKIFGIRSELYFL<br>DGGFRAKYASAFHLTENPNELYDNRNYFNTSWAYTITSVKWNVIEAQQRHFAHPVFRYSTDLC<br>FSSETPWGLLIAPESFYREPLQHFTLKINQAGLITQWMTQSFHEMVRAGRMTIKDYSRTNLMK<br>PLRIQDLRKCWVIFAVGLGTSTVVFTIELLLIYTNVFLNSL
>DmelIR100a<br>MATTLQLIMLALVGGTLGQANNTDHKQVLTSIVKQLEGGLELHLRTSEDGGNDLVQFLMQEK<br>SSIIISAKQEEVPSRAKIMRHHFFIFDGVHQMQEIRTSLFNTDGFYILALENNTIEDDVLLMEFAA<br>DVWLQHGHSRIYYVQLSKKSVLLFNPFLQRLVVVQDSKTYSRIYKDLEGYHLRIYIFDSVYSSV<br>IGDGENKVLSVTGADAKLAKTVARQLNFTADFVWPDDEFFGGRLANGEYSGGVGRAHRGEV<br>DIIFAGFFIKDYLTTHIQFSAAVYMDELCLYVKKAQRIPQSILPLFAVHMDVWLCFLLVGLLGAL<br>VWLILRAVNLILGIEGVPDGSRATRISYFGAARRIFVDTWVIWVRVNVGRFPPFHSERIFVASLCL<br>VSVIFGALLESSLATVYIRPLYYRDVNTLRELDESGQPIYIKHPAFKDDLFYGHNSEVYRRLDAK<br>MMLVAEGEERLIEMVSKRGGFAGVTRSASLQLSDIRYVMTKKVHKIPECPKNYHIAYVLPRPSP<br>YLEEVNRIVLRLVAGGIVGLWTGEAKERAKWSIQRFPEYLAELDVGRWKVLTLSDVQLAFYAL<br>TIGCLLSAIVCMAEILLGRQRRLHSPK
>DmelCG11155<br>MVRKKREIVIKENIQGRSYLKKICCSYIILSILVISNALPPVIRVGAIFTEDERESSIESAFKYAIYRI<br>NKEKTLLPNTQLVYDIEYVPRDDSFRTTKKVCSQLEAGVQAIFGPTDALLASHVQSICEAYDIP<br>HIEGRIDLEYNSKEFSINLYPSHTLLTLAYRDIMVYLNWTKVAIIYEEDYGLFNLMHSSTETKAE<br>MYIRQASPDSYRQVLRAIRQKEIYKIIVDTNPSHIKSFFRSILQLQMNDHRYHYMFTTFDLETYD<br>LEDFRYNSVNITAFRLVDVDSKRYLEVINQMQKLQHNGLDTINGSPYIQTESALMFDSVYAFAN<br>GLHFLNLDNHQNFYIKNLSCTSDQTWNDGISLYNQINAAITDGLTGTVQFVEGRRNIFKLDILK<br>LKQEKIQKVGYWHPDDGVNISDPTAFYDSNIANITLVVMTREERPYVMVKEDKNLTGNLRFEG<br>FCIDLLKAIATQVGFQYKIELVPDNMYGVYIPETNSWNGIVQELMERRADLAVASMTINYARES<br>VIDFTKPFMNLGIGILFKVPTSQPTRLFSFMNPLAIEIWLYVLAAYILVSFALFVMARFSPYEWK<br>NPHPCYKETDIVENQFSISNSFWFITGTFLRQGSGLNPKATSTRIVGGCWFFFCLIIISSYTANLAA<br>FLTVERMISPIESASDLAEQTEISYGTLEGGSTMTFFRDSKIGIYQKMWRYMENRKTAVFVKTY<br>EDGIKRVMEGSYAFLMESTMLDYAVQRDCNLTQIGGLLDSKGYGIATPKGSPWRDKISLAILEL<br>QEKGIIQILYDKWWKNTGDVCNRDDKSKESKANALGVENIGGVFVVLLCGLALAVVVAIFEFC<br>WNSRKNLNTENQSLCSEMAEELRFAMHCHGSKSRHRPRKRSCLNCSSVPTYVPSNVSTSNVG<br>VYYNYFN
>DmelCG3822<br>MRSSGVLVLPLLLLQLILNCRKAQSLPDIIKIGGLFHPADDHQELAFRQAVDRINADRSILPRSKL<br>VAQIERISPFDSFHAGKRVCGLLNIGVAAIFGPQSSHTASHVQSICDNMEIPHLENRWDYRLRRE<br>SCLVNLYPHPNTLSKAYVDIVRHWGWKTFTIIYENNDGIVRLQELLKAHGMTPFPITVRQLSDS<br>GDYRPLLKQIKNSAEAHIVLDCSTERIHEVLKQAQQIGMMSDYHSYLVTSLDLHTVNLDEFRY<br>GGTNITGFRLINEKIVSDVVRQWSIDEKGLLRSANLTTVRSETALMYDAVHLFAKALHDLDTSQ<br>QIDIHPISCDGQSTWQHGFSLINYMKIVEMKGLTNVIKFDHQGFRTDFMLDIVELTPAGIRKIGT<br>WNSTLPDGINFTRTFSQKQQEIEANLKNKTLVVTTILSNPYCMRKESAIPLSGNDQFEGYAVDLI<br>HEISKSLGFNYKIQLVPDGSYGSLNKLTGEWNGMIRELLEQRADLAIADLTITFEREQAVDFTTP<br>FMNLGVSILYRKPIKQPPNLFSFLSPLSLDVWIYMATAYLGVSVLLFILAKFTPYEWPAYTDAHG<br>EKVESQFTLLNCMWFAIGSLMQQGCDFLPKALSTRMVAGIWWFFTLIMISSYTANLAAFLTVE<br>RMDSPIESAEDLAKQTRIKYGALKGGSTAAFFRDSKISTYQRMWSFMESARPSVFTASNGEGV

ERVAKGKGSYAFLMESTSIEYVTERNCELTQVGGMLDTKSYGIATPPNSPYRTAINSVILKLQEE<br>GKLHILKTKWWKEKRGGGKCRVETSKSSSAANELGLANVGGVFVVLMGGMGVACVIAVCEF<br>VWKSRKVAVEERLSAILNE
>DmelCG5621<br>MWFVKMISTEASFPLGFILTSLLLAFPGCRGERTNVGLVYENTDPDLEKIFHLAISKANEENEDL<br>QLHGVSVSIEPGNSFETSKKLCKMLRQNLVAVFGPTSNLAARHAMSICDAKELPFLDTRWDFG<br>AQLPTINLHPHPATLGVALRDMVVALGWESFTIIYESGEYLPTVRELLQMYGTAGPTVTVRRYE<br>LDLNGNYRNVLRRIRNADDFSFVVVGSMATLPEFFKQAQQVGLVTSDYRYIIGNLDWHTMDL<br>EPYQHAGTNITGLRLVSPDSEQVQEVAKALYESEEPFQNVSCPLTNSMALVYDGVQLLAETYK<br>HVNFRPVALSCNDDSAWDKGYTLVNYMKSLTLNGLTGPIRFDYEGLRTDFKLEVIELAVSGMQ<br>KIGQWSGEDGFQENRPAPAHSLEPDMRSLVNKSFVVITAISEPYGMLKETSEKLEGNDQFEGFG<br>IELIDELSKKLGFSYTWRLQEDNKYGGIDPKTGEWNGMLREIIDSRADMGITDLTMTSERESGV<br>DFTIPFMSLGIGILFRKPMKEPPKLFSFMSPFSGEVWLWLGLAYMGVSISMFVLGRLSPAEWDN<br>PYPCIEEPTELENQFSFANCLWFSIGALLQQGSELAPKAYSTRAVAASWWFFTLILVSSYTANLA<br>AFLTVESLVTPINDADDLSKNKGGVNYGAKIGGATFNFFKESNYPTYQRMYEFMRDNPQYMT<br>NTNQEGVDRVENSNYAFLMESTTIEYITERRCTLTQVGALLDEKGYGIAMRKNWPYRDTLSQA<br>VLEMQEQGLLTKMKTKWWQEKRGGGACSEAPDSDASSLGFANLGGVYLVMFVGSCFGSIYG<br>LVNCVVSVYLRARENKVSFKTELLDEIRFILQCSGNTKAVKYPKNSSRSNASSKSKGSSMSVDS<br>LPEDTSEADASGKHNHKK
>DmelCG9935<br>MLIASGFLLFQFLSYGLGVPPLVRIGAIFSNQPGMYNSELAFRYAIHRLNMDKSLLPETTVDYY<br>VEYVNRFDSFETVQKVCKLIRVGVQAVFSPTDSVLATHINSICDALDIPNIGRSAHDFSINVYPSK<br>QLVNYAFNDVIQYLNWTRFGILHEKENGIINLHQLSRSFHGEVHMRQVSRDSYVSALNEFKGK<br>EIHNIIIDTNSNGISILLKNILQQQMNEYKYHYLFTSFDLETYDLEDFKYNFVNITSFRLVDTADV<br>GVKQILKDIGLYSHHIFKKPYLNLHIKKSTILESEPALMFDSVYVFAIGLQTLEQSHSLTLLNISCE<br>EENSWDGGLSLINYLNAVEWKGLTGPIQFKDGQRVQFKLDLIKLKQHSIVKVGEWTPHGHLNI<br>TEPSMFFDAGSMNVTLVVITILETPYVMMHYGKNFTGNERFYGFCVDILETISREVGFDYILDL<br>VPDRKYGAKDPETGEWNGMVAQLMKYADLAVGSMTITYARESVIDFTKPFMNLGISILFKVPT<br>SEPTRLFSFMNPLAIEIWIYVLIAYFLVSLCIYIVGKLSPIEWKCINACDLENISIGNQFSLTDSFWF<br>TIGTFMQQSPDIYPRAMSTRIISSTWGFFSLIIVASYTANLAAFLTTERMINPIENAEDLASQTEIS<br>YGTLDSGSTMTFFRDSVIETYKKIWRSMDNKKPSAFTTTYEDGIKRVNQGNYAFLMESTMLDY<br>IVQRDCNLTQIGGLLDTKGYGIATPKGSPWRDKISLAILELQERGDIQMLYDKWWKNTDETCT<br>RKNTSKQSKANSLGLESIGGVFVVLIAGIIVAAVVAFFEFWYNFRYNYEATPSQSVVNNKYNQD<br>GILESERNYTPPDRSFWIEIAEELRYASWCMNKQKRPALTRTCSKCTIPKGQRINKL
>Dmelclumsy<br>MYSLFLTHFLLIALPVLADIDRSQFMVGSIFTSDKDESEIAFRTAVDRANILERNVELVPIVVYAN<br>TDDSFIMEKMVCNLISQGVIAIFGPSTGSSSDIIASICDTLDIPHIVYDWIPNESIPDREHSTMTLN<br>VHPDNLLLSQGLAEIVQSFAWRSFTVVYETDKELQQLQDILQVGEPISNPTTVKQLGPGDDHRP<br>FLKEIKLSTDNCLILHCAPDNLLKILQQANELKMLGEYQSVFIPLLDTHSIDFGELSGVEANITT<br>VRLMDPSDFHVKNVVHDWEEREKREGRYFKVDPNRVKSQMILLNDAVWLFSKGLTELGIFEE<br>LTAPDLECRRKKPWPFGKRIIEFIKARSEETSTGRIDFNENGQRSFFTLRFMELNSDGFLDLATW<br>DPVNGLDVLNDDEESEKRVGQKLSNKTFIVSSRLGAPFLTLREPQEGEILTGNSRYEGYSIDLIN<br>EIAKMLNFKFEFRMSPDGKYGALNKVTQTWDGIVRQLIDGNADLGICDLTMTSSRRQAVDFTP<br>PFMTLGISILFSKPPTPPTDLFSFLSPFSLDVWIYMGSAYLFISLLLFALARMAPDDWENPHPCKE

PEEVENIWSIMNTTWLSIGSLMGQGCDILPKAASTRLVTGMWWFFALMMLNSYTANLAAFLT<br>NSRQANSINSAEDLAAQSKIKYGAMAGGSTMGFFRDSNFSTYQKMWTAMESASPSVFTKTND<br>EGVERVQKGKNLYAFLMESTTLEYNVERKCDLVQIGGWLDYKSYGIAMPFNSPYRKQISAAVL<br>KLGELGQLAELKRKWWKEMHGGGNCEKSDEDGGDTPELGLENVGGVFLVLGLGLLSAMVL<br>GCTEFLWNVKSVAIEEKISLKEAFKSEALFAARIWITTKPVHTSSESGSSNSSSSSSSRSKHSFKS<br>QGLSMKSLKSSGYQDVEASVHSKLKKIGSMFSLKSQKTVTPPPEIGWKLDKSTQIDVVPTSDV<br>DQELIPEVEPHLPHRHHHHHHHRHHHHHHQPDQEHDRNPSPPE
>DmelGlu-R1<br>MHSRLKFLAYLHFICASSIFWPEFSSAQQQQQTVSLTEKIPLGAIFEQGTDDVQSAFKYAMLNH<br>NLNVSSRRFELQAYVDVINTADAFKLSRLICNQFSRGVYSMLGAVSPDSFDTLHSYSNTFQMPF<br>VTPWFPEKVLAPSSGLLDFAISMRPDYHQAIIDTIQYYGWQSIIYLYDSHDGLLRLQQIYQELKP<br>GNETFRVQMVKRIANVTMAIEFLHTLEDLGRFSKKRIVLDCPAEMAKEIIVQHVRDIKLGRRTY<br>HYLLSGLVMDNHWPSDVVEFGAINITGFRIVDSNRRAVRDFHDSRKRLEPSGQSQSQNAGGPN<br>SLPAISAQAALMYDAVFVLVEAFNRILRKKPDQFRSNHLQRRSHGGSSSSSATGTNESSALLDC<br>NTSKGWVTPWEQGEKISRVLRKVEIDGLSGEIRFDEDGRRINYTLHVVEMSVNSTLQQVAEWR<br>DDAGLLPLHSHNYASSSRSASASTGDYDRNHTYIVSSLLEEPYLSLKQYTYGESLVGNDRFEGY<br>CKDLADMLAAQLGIKYEIRLVQDGNYGAENQYAPGGWDGMVGELIRKEADIAISAMTITAER<br>ERVIDFSKPFMTLGISIMIKKPVKQTPGVFSFLNPLSQEIWISVILSYVGVSFVLYFVTRFPPYEW<br>RIVRRPQADSTAQQPPGIIGGATLSEPQAHVPPVPPNEFTMLNSFWYSLAAFMQQGCDITPPSIA<br>GRIAAAVWWFFTIILISSYTANLAAFLTVERMVAPIKTPEDLTMQTDVNYGTLLYGSTWEFFRRS<br>QIGLHNKMWEYMNANQHHSVHTYDEGIRRVRQSKGKYALLVESPKNEYVNARPPCDTMKVG<br>RNIDTKGFGVATPIGSPLRKRLNEAVLTLKENGELLRIRNKWWFDKTECNLDQETSTPNELSLS<br>NVAGIYYILIGGLLLAVIVAIMEFFCRNKTPQLKSPGSNGSAGGVPGMLASSTYQRDSLSDAIMH<br>SQAKLAMQASSEYDERLVGVELASNVRYQYSM
>DmelGlu-R1B<br>MRFGLKLSCLWPSFLLWLTWSSGGGGGSGVGVSAQPSLTEKIPLGAIFEQGTDEVQSAFKYAM<br>LNHNLNVSSRRFELQAYVDVINTADAFKLSRLICNQFSRGVYSMLGAVSPDSFDTLHSYSNTFQ<br>MPFVTPWFPEKVLTPSSGFLDFALSMRPDYHQAIIDTIQFYGWRKIIYLYDSHDGLLRLQQIYQG<br>LRPGNESFQVELVKRISNVSMAIEFLHTLEQIGRFENKHIVLDCPTEMAKQILIQHVRDLRLGRR<br>TYHYLLSGLVMDDRWESEIIEFGAINITGFRIVDTNRRLVREFYDSWKRLDPQMSVGAGRESIS<br>AQAALMYDAVFVLVEAFNKILRKKPDQFRNNVQRRSQTLMVAQAAASTSSDGYNYSASGGG<br>GGNGGAGGGFAGSDSGGSGGMASRALDCNTAKGWVNAWEHGDKISRYLRKVEIEGLTGDIK<br>FNDDGRRVNYTLHVVEMTVNSAMVKVAEWNDDAGLQPLNAKYVRLRPHVEFEKNRTYIVTT<br>VLEEPYIMLKQVAFGEKLHGNNRFEGYCKDLADLLAKELGINYELRLVKDGNYGSEKSSAHG<br>GWDGMVGELVRKEADIAIAAMTITAERERVIDFSKPFMSLGISIMIKKPVKQTPGVFSFMNPLS<br>QEIWVSVIFSYIGVSIVLFFVSRFSPHEWRLVQQQPQQSQSPDPHAHHEQLANQQPPGIIGGAPL<br>PAPPGPPTPGAQTAAGAAALQAALSAGSPGSGGSSSAVVNEFSVWNSFWFSLAAFMQQGCDLS<br>PRSVSGRIAAASWFFFTLILISSYTANLAAFLTVERMVTPINSPEDLAMQTEVQYGTLLHGSTW<br>DFFRRSQIGLHNKMWEYMNSRKHVFVPTYDEGIKRVRNSKGKYALLVESPKNEYVNAREPCD<br>TMKVGRNLDTKGFGIATPLGSALKDPINLAVLTLKENGELIKLRNKWWYEKAECSTHKDGETS<br>HSELSLSNVAGIFYILIGGLLVSVFVAILEYCFRSRDSRSASSGSGMGLGMGLGGGMSGGSLGK<br>ANGSMMLGPSSAVPGGMPSSHQRSTLTDTMHAKAKLTIQASRDYDNGRVGYLNCASLQYYPP<br>AQLSATPPDAGDSLHMNAHGQVXHEHAQESRLXRTPCAPTIRRIMANSRQETEKMMGGGHG<br>GTVTVSVPATCSSSMNGSHEGSLALSPSSGSATLMRHSPDINQHPYGK

>DmelGluRIIA
MRLCPVVIYAFIIIIGFLEGIIALGGDDRNEITVGAIFYENEKEIELSFDQAFREVNNMKFSELRFV
TIKRYMPTNDSFLLQQITCELISNGVAAIFGPSSKAASDIVAQIANATGIPHIEYDLKLEATRQEQL
NHQMSINVAPSLSVLSRAYFEIIKSNYEWRTFTLIYETPEGLARLQDLMNIQALNSDYVKLRNL
ADYADDYRILWKETDETFHEQRIILDCEPKTLKELLKVSIDFKLQGPFRNWFLTHLDTHNSGLR
DIYNEDFKANITSVRLKVVDANPFERKKTRLTKVDQILGNQTMLPILIYDAVVLFASSARNVIA
AMQPFHPPNRHCGSSSPWMLGAFIVNEMKTISEDDVEPHFKTENMKLDEYGQRIHFNLEIYKP
TVNEPMMVWTPDNGIKKRLLNLELESAGTTQDFSEQRKVYTVVTHYEEPYFMMKEDHENFR
GREKYEGYAVDLISKLSELMEFDYEFMIVNGNGKYNPETKQWDGIIRKLIDHHAQIGVCDLTIT
QMRRSVVDFTVPFMQLGISILHYKSPPEPKNQFAFLEPFAVEVWIYMIFAQLIMTLAFVFIARLS
YREWLPPNPAIQDPDELENIWNVNNSTWLMVGSIMQQGCDILPRGPHMRILTGMWWFFALM
MLSTYTANLAAFLTSNKWQSSIKSLQDLIEQDKVHFGSMRGGSTSLFFSESNDTDYQRAWNQ
MKDFNPSAFTSTNKEGVARVRKEKGGYAFLMETTSLTYNIERNCDLTQIGEQIGEKHYGLAVPL
GSDYRTNLSVSILQLSERGELQKMKNKWWKNHNVTCDSYHEVDGDELSIIELGGVFLVLAGG
VLIGVILGIFEFLWNVQNVAVEERVTPWQAFKAELIFALKFWVRKKPMRISSSSDKSSSRRSSGS
RRSSKEKSRSKTVS

>DmelGluRIIB
MHGLQFLVLLALAIASGANEDTLVIKIGAIFFDTEMKLADAFSAALEEVNAINPALKLDAIKRY
VTVDDSIVLQDISCDLIGSGVAAIFGPSSKTNSDIVEVLCNMTGIPHLQFDWHPQQSNRERMNH
QLTVNVAPMELFLSAAFSDILASKTFDWKSFTIAYERSSHLIRLQHILAWKQLHKAGIKMQEFE
RGDDYRILWKRINNAREKFVLLDCPSDILVDVINASIGYNMTGSFNHLFLTNLDTHLSGIDGFYS
RDFTVAVAAVRIRTYVPPPVHDEIDVFDNSVDTRFSSLGSQLVYDSIVLFYNALLEISQRPGFYIP
NFSCGRGFWQPGPRLVEQMKQITPKMVKPPFKTQRLQINADGQREDFNLEVYNPIIDRVTHIW
NKEFQLVDFEKLRENSTQALKQKRLQNKEDFSQKPIRYTVATRVGKPYFSWREEPEGVHYEGN
ERFEGYAVDLIYMLAQECKFDFNFEPVRDNKYGSYDANTDEWDGIIRQLIDNNAQIGICDLTIT
QARRSVVDFTVPFMQLGISILSYKEPPPKADIYAFLNPYNAEVWLFVMIAMMITAFALIFTGRID
QYEWDQPVENVNREMERQNIWHLSNALWLVLGSMLNQGCDLLPRGLPMRLLTAFWWIFALLI
SQTYIAKLAAFITSSKIAGDIGSLHDLVDQNKVQFGTIRGGATSVYFSESNDTDNRMAWNKMLS
FKPDAFTKNNEEGVDRVKLSKGTYAFLMETTNLQYYVQRNCELTQIGESFGEKHYGIAVPLNA
DFRSNLSVGILRLSERGELFKLRNKWFNSNESTCDSNVPTIDDGQFDMDSVGGLFVVLIVGVV
VGLVIGVAEFLWHVQRISVKEKIPPMLALKAEFYFVIRFWLTRKPLHTYRQSRDSTSTGYSSLEQ
ITSASSAKKKKKTRRIEK

>DmelIR87a
MSTPEQRFWLAALLFLLSQHSEVRGFGINLMKVQTEDKGQEACILALLRKYFDSGDGLSGSVL
CINRNYQLPNIEEQLLRGVNNYENYPWSLLITNSREGPSPAKFLMNEKPQCYFLIVDNLEDEDL
DEVFEHWKGMVNWNPLAQFVVYLASLEETDEEMNDLMVELLLTFINKKIFNVNVIGQSEENQ
FYYGKTVFPYHPDNNCGNRVISVELLDACDYPSEETDSEDENDEDEGDGAQEEDDGPQEEGD
GEQEEEDGPQEQEDGDQAKGDEGQENDDGGLENKVENEFRIGASDDDELENDLSSNSSEPEAI
IEEFFRAKFEDKFPRDLSGCPLTASFRPWEPYIFRNSEEQPVDDYYYGLQGDEDDYNDTSPNYG
ESDDESYADPGEDGDGAIPDTETQSGGKLKLSGIEYEMVQTIAERLHVSIEMQGENSNLYHLFQ
QLIDGEIEMIVGGIDEDPSISQFVSSSIPYHQDELTWCVARAKRRHGFFNFVATFNADAGFLIGIF
VVTCSLVVWLAQRVSGFQLRNLNGYFPTCLRVLGILLNQAIPAQDFPITLRQLFALSFLMGFFFS
NTYQSFLISTLTTPRSSYQIHTLQEIYSNKMTVMGTSEHVRHLNKDGEIFKYIREKFQMCYNLV
DCLNDAAQNEHIAVAVSRQHSFYNPRIQRDRLYCFDRRESLYVYLVTMLLPKKYHLLHQINPVI

QHIIESGHMQKWARDLMRRMIHEEITRVREDPFKALTFDQFRGAIAFSGGLLLVASCVFAFELC<br>YVKYVYRTEKRERKTKKITKKVHNIKIQHD
>DmelIR21a<br>MSYYWVALVLFTAQAFSIEGDRSASYQEKCISRRLINHYQLNKEIFGVGMCDGNNENEFRQKR<br>RIVPTFQGNPRPRGELLASKFHVNSYNFEQTNSLVGLVNKIAQEYLNKCPPVIYYDSFVEKSDG<br>LILENLFKTIPITFYHGEINADYEAKNKRFTSHIDCNCKSYILFLSDPLMTRKILGPQTESRVVLVS<br>RSTQWRLRDFLSSELSSNIVNLLVIGESLMADPMRERPYVLYTHKLYADGLGSNTPVVLTSWIK<br>GALSRPHINLFPSKFQFGFAGHRFQISAANQPPFIFRIRTLDSSGMGQLRWDGVEFRLLTMISKRL<br>NFSIDITETPTRSNTRGVVDTIQEQIIERTVDIGMSGIYITQERLMDSAMSVGHSPDCAAFITLAS<br>KALPKYRAIMGPFQWPVWVALICVYLGGIFPIVFTDRLTLSHLMGNWGEVENMFWYVFGMFT<br>NAFSFTGKYSWSNTRKNSTRLLIGAYWLFTIIITSCYTGSIIAFVTLPAFPDTVDSVLDLLGLFFR<br>VGTLNNGGWETWFQNSTHIPTSRLYKKMEFVGSVDEGIGNVTQSFFWNYAFLGSKAQLEYLV<br>QSNFSDENISRRSALHLSEECFALFQIGFLFPRESVYKIKIDSMILLAQQSGLIAKINNEVSWVMQ<br>RSSSGRLLQASSSNSLREIIQEERQLTTADTEGMFLLMALGYFLGATALVSEIVGGITNKCRQIIK<br>RSRKSAASSWSSASSGSMLRTNAEQLSHDKRKANRREAAEVAQKMSFGMRELNLTRATLREIY<br>GSYGAPETDHGQLDIVHTEFPNSSAKLNNIEDEESREALESLQRLDEFMDQMDNDGNPSSHTF<br>RIDN
>DmelIR76b<br>MATGIELLVAAALCVACPPLNDSPPTNLIQMGENGTLSPVTELPMDVDASEAGFDADAPVETLE<br>TINRKKPKLREMLDWIGGKHLRIATLEDFPLSYTEVLENGTRVGHGVSFQIIDFLKKKFNFTYE<br>VVVPQDNIIGSPSDFDRSLIEMVNSSTVDLAAAFIPSLSDQRSFVYYSTTTLDEGEWIMVMQRP<br>RESASGSGLLAPFEFWVWILILVSLLAVGPIIYALIILRNRLTGDGQQTPYSLGHCAWFVYGALM<br>KQGSTLSPIADSTRLLFATWWIFITILTSFYTANLTAFLTLSKFTLPYNTVNDILTKNKHFVSMRG<br>GGVEYAIRTTNESLSMLNRMIQNNYAVFSDETNDTYNLQNYVEKNGYVFVRDRPAINIMLYRD<br>YLYRKTVSFSDEKVHCPFAMAKEPFLKKKRTFAYPIGSNLSQLFDPELLHLVESGIVKHLSKRNL<br>PSAEICPQDLGGTERQLRNGDLMMTYYIMLAGFATALAVFSTELMFRYVNSRQEANKWARHGI<br>GRTPNGQSVAPSRWLRGWRRLNSGHGQLLGASTHGQNVTPPPPYQS<br>IFNGGSHGDPLNRWRRPLANGNALGNGVLLGGDSEGGVRRLINGRDYMVFRNPNGQSQLVPV<br>RSPSAALFQYSYTE
>DmelGluRIIC<br>MWQRILLLGCMWSAFFMCRSRGQQINIGAFFYDDELELEKEFMTVVNAINGPESEQTMRFYPL<br>IKRLKPEDGSVTMQEHACDLIDNGVAAIFGPSSKAASDIVALVCNSTGIPHIEFDISDEGIQAEKP<br>NHQMTLNLYPAQAILSKAYADIVQNFGWRKFTIVYDADDARAAARLQDLLQLREVHNDVVRV<br>RKFHKDDDFRVMWKSIRGERRVVLDCEPNMLVELLNSSTEFGLTGQYNHIFLTNLETYTDHLE<br>ELAADNETFAVNITAARLLVNPDPPPYSLPYGYVTQRDNIVYESSDPPRTLIHDLIHDALQLFAQ<br>SWRNASFFYPDRMVVPRITCDFAASGGRTWAMGRYLARLMKGTSGVNNTNFRTSILQFDEDG<br>QRITFNIEVYDPLDGIGIAIWDPRGQITQLNVDVKAQKKMIYRVATRIGPPYFSYNETARELNLT<br>GNALYQGYAVDLIDAIARHVGFEYVFVPVADQQYGKLDKETKQWNGIIGEIINNDAHMGICDL<br>TITQARKTAVDFTVPFMQLGVSILAYKSPHVEKTLDAYLAPFGGEVWIWILISVFVMTFLKTIVA<br>RISKMDWENPHPCNRDPEVLENQWRIHNTGWLTVASIMTAGCDILPRSPQVRMFEATWWIFAII<br>IANSYTANLAAFLTSSKMEGSIANLKDLSAQKKVKFGTIYGGSTYNLLADSNETVYRLAFNLM<br>NNDDPSAYTKDNLEGVDRVRKNRGDYMFLMETTTLEYHREQNCDLRSVGEKFGEKHYAIAVP<br>FGAEYRSNLSVAILKLSERGELYDLKQKWWKNPNASCFEEPDPDATPDMTFEELRGIFYTLYAG<br>ILIAFLIGITEFLVYVQQVALEERLTFKDAFKKEIRFVLCVWNNRKPIVAGTPISSVRTTPRRSLDK

SLDRTPKSSRRVVIGRSSEEMREMAQGSGSSSGSNNAGRGEKEARV
>DmelGluRIID<br>MHFCWISLIILSLSRVQAQFYGGNAYEASSGQSIRLGLITDDATDRIRQTFEHAISVVNNELGVPL<br>VGETEQVAYGNSVQAFAQLCRLMQSGVGAVFGPAARHTASHLLNACDSKDIPFIYPHLSWGSN<br>PDGFNLHPSPEDIANALYDIVNQFEWSRFIFCYESAEYLKILDHLMTRYGIKGPVIKVMRYDLN<br>LNGNYKSVLRRIRKSEDSRIVVVGSTTGVAELLRQAQQVGIMNEDYTYIIGNLNLHTFDLEEYK<br>YSEANITGIRMFSPDQEEVRDLMEKLHQELGESEPVNSGSTFITMEMALTYDAVRVIAETTKHL<br>PYQPQMLNCSERHDNVQPDGSTFRNYMRSLEIKEKTITGRIYFEGNVRKGFTFDVIELQTSGLV<br>KVGTWEEGKDFEFQRPPQAVNFNDIDDGSLVNKTFIVLISVATKPYASLVESIDTLIGNNQFQGY<br>GVDLIKELADKLGFNFTFRDGGNDYGSFNKTTNSTSGMLKEIVEGRADLAITDLTITSEREEVID<br>FSIPFMNLGIAILYVKPQKAPPALFSFMDPFSSEVWLYLGIAYLGVSLCFFIIGRLSPIEWDNPYPC<br>IEEPEELENQFTINNSLWFTTGALLQQGSEIAPKALSTRTISAIWWFFTLIMVSSYTANLAAFLTIE<br>NPTSPINSVKDLADNKDDVQYGAKRTGSTRNFFSTSEEPIYIKMNEYLNAHPEMLMENNQQGV<br>DKVKSGTKYAFLMESTSIEFNTVRECNLTKVGDPLDEKGYGIAMVKNWPYRDKFNKALLELQ<br>EQGVLARLKNKWWNEVGAGVCSAKSDDDGPSELGVDNLSGIYVVLVIGSIISIIISILCWCYFV<br>YKKAKNYEVPFCDALAEEFRIVIRFSENERPLKSAQSIYSRSRNSSQSIESLKTDSEENMPVED
>DmelGluRIIE<br>MFFNHFVILWSLFSIHISVNWAQYENFGGYDNYQSLESVPIGLLTDQNTEQMNIVFDHAIDVAN<br>QEVGTSLTSLKEEVNYGDAYQSYGKLCRMLETGIAGVFGPSSRHTAVHLMSICDAMDIPHIYSY<br>MSENAEGFNLHPHPADLAKALYSLITEFNWTRFIFLYESAEYLNILNELTTMLGKSGTVITVLRY<br>DMQLNGNYKQVLRRVRKSVDNRIVVVGSSETMPEFLNQAQQVGIINEDYKYIIGNLDFHSFDL<br>EEYKYSEANITGLRLFSPEKMAVKELLMKLGYPTDQDEFRNGSCPITVEMALTYDAVQLFAQT<br>LKNLPFKPMPQNCSQRTESVRDDGSSFKNYMRTLRLTDRLLTGPIYFEGNVRKGYHLDVIELQP<br>SGIVKVGTWDEDRQYRPQRLAPTTAQFDSVDNSLANKTFIILLSVPNKPYAQLVETYKQLEGNS<br>QYEGYGVDLIKELADKLGFNFTFVNGGNDYGSYNKSTNESTGMLREIMTGRADLAITDLTITS<br>EREQALDFTIPFMNLGIAILYLKPQKATPELFTFMDPFSEEVWWFLGFSFLGVSLSFFILGRLSPS<br>EWDNPYPCIEEPEELENQFTLGNSIWFTTGALLQQGSEIGPKALSTRTVASFWWFFTLIVVSSYT<br>ANLAAFLTIEKPQSLINSVDDLADNKDGVVYGAKKTGSTRNFFMTSAEERYKKMNKFMSENP<br>QYLTEDNMEGVNRVKTNTHYAFLMESTSIEYNTKRECNLKKIGDALDEKGYGIAMRKDWPHR<br>GKFNNALLELQEQGVLEKMKNKWWNEVGTGICATKEDAPDATPLDMNNLEGVFFVLLVGSC<br>CALLYGIISWVLFVMKKAHHYRVPLRDALKEEFQFVIDFNNYVRVLKNSASIYSRSRQSSMSVA<br>SVAQESQ
>DmelNmdar1<br>MAMAEFVFCRPLFGLAIVLLVAPIDAAQRHTASDNPSTYNIGGVLSNSDSEEHFSTTIKHLNFDQ<br>QYVPRKVTYYDKTIRMDKNPIKTVFNVCDKLIENRVYAVVVSHEQTSGDLSPAAVSYTSGFYSI<br>PVIGISSRDAAFSDKNIHVSFLRTVPPYYHQADVWLEMLSHFAYTKVIIIHSSDTDGRAILGRFQ<br>TTSQTYYDDVDVRATVELIVEFEPKLESFTEHLIDMKTAQSRVYLMYASTEDAQVIFRDAGEYN<br>MTGEGHVWIVTEQALFSNNTPDGVLGLQLEHAHSDKGHIRDSVYVLASAIKEMISNETIAEAP<br>KDCGDSAVNWESGKRLFQYLKSRNITGETGQVAFDDNGDRIYAGYDVINIREQQKKHVVGKF<br>SYDSMRAKMRMRINDSEIIWPGKQRRKPEGIMIPTHLRLLTIEEKPFVYVRRMGDDEFRCEPDE<br>RPCPLFNNSDATANEFCCRGYCIDLLIELSKRINFTYDLALSPDGQFGHYILRNNTGAMTLRKE<br>WTGLIGELVNERADMIVAPLTINPERAEYIEFSKPFKYQGITILEKKPSRSSTLVSFLQPFSNTLWI<br>LVMVSVHVVALVLYLLDRFSPFGRFKLSHSDSNEEKALNLSSAVWFAWGVLLNSGIGEGTPRSF<br>SARVLGMVWAGFAMIIVASYTANLAAFLVLERPKTKLSGINDARLRNTMENLTCATVKGSSVD

MYFRRQVELSNMYRTMEANNYATAEQAIQDVKKGKLMAFIWDSSRLEYEASKDCELVTAGEL<br>FGRSGYGIGLQKGSPWTDAVTLAILEFHESGFMEKLDKQWIFHGHVQQNCELFEKTPNTLGLK<br>NMAGVFILVGVGIAGGVGLIIIEVIYKKHQVKKQKRLDIARHAADKWRGTIEKRKTIRASLAM<br>QRQYNVGLNSTHAPGTISLAVDKRRYPRLGQRLGPERAWPGDAADVLRIRRPYELGNPGQSPK<br>VMAANQPGMPMPMLGKTRPQQSVLPPRYSPGYTSDVSHLVV
>DmelNmdar2<br>MMPSRVKLKRGTDGPTPTPTPMPTTMRKHTPIATLNTASCQHNSTTSRRKRILTPPSGPISLLLLT<br>VLTLLILDTRSCQGLRLTNGGGSLSKGAAANKEQLNIGLIAPHTNFGKREYLRSINNAVTGLTKT<br>RGAKLTFLKDYSFEQKNIHFDMMSLTPSPTAILSTLCKEFLRVNVSAILYMMNNEQFGHSTASA<br>QYFLQLAGYLGIPVISWNADNSGLERRASQSTLQLQLAPSIEHQSAAMLSILERYKWHQFSVVT<br>SQIAGHDDFVQAVRERVAEMQEHFKFTILNSIVVTRTSDLMELVNSEARVMLLYATQTEAITILR<br>AAEEMKLTGENYVWVVSQSVIEKKDAHSQFPVGMLGVHFDTSSAALMNEISNAIKIYSYGVE<br>AYLTDPANRDRRLTTQSLSCEDEGRGRWDNGEIFFKYLRNVSIEGDLNKPNIEFTADGDLRSAE<br>LKIMNLRPSANNKNLVWEEIGVWKSWETQKLDIRDIAWPGNSHAPPQGVPEKFHLKITFLEEA<br>PYINLSPADPVSGKCLMDRGVLCRVAADHEMAADIDVGQAHRNESFYQCCSGFCIDLLEKFAE<br>ELGFTYELVRVEDGKWGTLENGKWNGLIADLVNRKTDMVLTSLMINTEREAVVDFSEPFMET<br>GIAIVVAKRTGIISPTAFLEPFDTASWMLVGIVAIQAATFMIFLFEWLSPSGYDMKLYLQNTNVTP<br>YRFSLFRTYWLVWAVLFQAAVHVDSPRGFTSRFMTNVWALFAVVFLAIYTANLAAFMITREEF<br>HEFSGLNDSRLVHPFSHKPSFKFGTIPYSHTDSTIHKYFNVMHNYMRQYNKTSVADGVAAVLN<br>GNLDSFIYDGTVLDYLVAQDEDCRLMTVGSWYAMTGYGLAFSRNSKYVQMFNKRLLEFRAN<br>GDLERLRRYWMTGTCRPGKQEHKSSDPLALEQFLSAFLLLMAGILLAALLLLLEHVYFKYIRK<br>RLAKKDGGHCCALISLSMGKSLTFRGAVFEATEILKKHRCNDPICDTHLWKVKHELDMSRLRV<br>RQLEKVMDKHGIKAPQLRLASSSDLLNHHHLKERPPLLGNLSLAASAQDLYRWSYKTEIAEME<br>TVL
>Vd18951<br>MLFLIGTQLSRSRPPSTRPSPQSRTGNSSADWADSRRLFVYSSLLLLFVTRCAALPPVINVGGLF<br>DNEDDEQDFAFRVAIERVNNDQSILLKSRLVPRVERVEKDDCFRATKKVCTLLREGIAGIFGPTS<br>DVTSMHVQSICDALDVPHVEMRWDFQLQRDDLSINLFPKPSILAQAYVDLIKTWDWKSFALV<br>YEDHEGIRLKDFIKGARREGWKIQMYQFVPNEPYRDLFWKIKKSEQN<br>RVVLDVHRKNLYECLKHAQQVGMLTESHSYLISSLDLHTVDLEEFKYGQTKITGLRLVDVESP<br>ELQNFLDDWKRLAQQPKHVRAPPAPGPHLIKTETALMYDAVKLFAMGLQQLDLTKAVDFPVIS<br>CDAAESSSDGSSLINLMRPITLQGLTGDISFDSQGFRSTFQLDVMSLKTDGLQKVGYWNPKQR<br>VVVEDNSTSDYDSLRLRNKTLIVSTVLTDPYMMLKESAKFLTGNERFEGYCVDLLQELSKDLG<br>FSYEIRLAADGAYGIKSDTGVWNGMIGEVVYGKADLAIADLTITSAREAAVDFTMPFMNTGISI<br>LFKKPTQKATSLFGFLSPFSTEVWTYVVGAYLGVSCVLFLVGRMSPYEWDNPHPCRQNDQVL<br>ENSFSLLNSMWFTIGSLMQQGSDLAPKAMSTRTVAGIWYFFTLIMISSYTANLAAFLTVEKTVY<br>PVESAEDLAKQTKIQYGCVKSGSTRAFFKESKIPTFMKMHKFMEERNTYVATSAEGKQRVSNG<br>DYAFLMESASIEFLVERNCNLTQIGSLLDNKGYGIVTVKNSPFRQVLSSGILQLQEAGKLHSFKE<br>KWWKERKGGGKCTDDTKKSSAVTELSLANVGGVFVVLLLGLLLAALVGIAEFLWKARSLSKE<br>DKVSICSEMLKQLKFAVSCRSSTMPVRSSRQTPELDTVDANNLSNRYGISTLPSFTSDF
>Vd20015<br>MSSDDVSDWPQSSPATKTRPTAAWQVPRLELRLKAGSAKTQAVMNNQWNQRVNDTNRYSYY<br>KYGIKKNKTNYQDLLNSQDFDKVTAGQRVIPGDRVSNLRNLHRFQCRCLDTRTPFIDYQLEPR<br>SRDHVLSSMVVNSIQTSASSFLCPTRSSLEKQIDVNVFLLEIYNTEITTNNHNDLTLKSKAVTTTS

TVLNACKNHGGMISNICRNDSNEVYADDKDGDEQKGGDDDNYSSTTSTLDHNSQQLAIGKPE
LLSLWRSPVWPAHMPSKAESKYLRDPEAKPRKRYRLNRQRSLDVTARNDEPLVCIRQLIHRLA
SRYVATSLYTVSGTGMGNALLPLMVVLAAAACLPSAVDGAKSSTDAFAIGGVFDQGENSHLDE
HLSRAVERVNRDGALLRGRELQSISFKTNPGDSFVASKCVCELARQGVVALVGPTSWQVATYV
ASAAARLHVPHLKTSAGGFRERRSPFSLQLHPAVNQLNKAYYDLIKSRKWKSFAVLYDNDNVF
VALKDVLNASLNPPNVLMYPYNPALSFKKMLKDIGSKNIYNIILHLELTKVPQLFREAEEVNQT
TLYHDYIVMGLDFHTLDLREFYSLKANVTAFRIIDPDRVAVQLVRRDWALTQGLQPDSGVSASH
SKGLGSPGKRGLTGTALFWRKPRDLSSGLPASIDQMSTAEALLYDAVSLVAAGVEALLEKDKE
AAKDNKAAKGKDNSKDKDLRAGPLVLSQLDCNRSAHWEHGKALLEAIKGARIRGLSGDLRL
NRRTGTRDEFGLDVVELKYTGLKKIASWSPREGLKLNAANRTEQEADLSRTLEGMTLRVVTV
LNEPYTMLHPPSENRTGNDRFYGYAIDLIAALASLYKFNYTFYISPDRKYGSRQPDGKWNGMV
GELIQRKADIAVVDLTITYEREQVVDFTIPFMNTGISILFKKPEKSEPAIFSFLYPFSIVVWFYTLTV
YTFVSILVYVLGRFTPYEWVPSHPCDPQSEPENQFSSLQNAFWFTMGSIMQQGSDLVPRAISTR
VLASIWYFFTLILISSYTANLAAFLTAARMGAPIENANDLAKQTKIAYGCLGGGSTYGFFKKHN
DPVMKRMWTYMETARPAVFTSSNKEGIERVLRGDYAYLMEALSIEYLVERNCNLTQIGGLLDN
KGYGIATPLKSPLRSALTSAILILQEKGVLHSLKMKWWKQQGGLVKCDDEKGASSTSEMDMG
SVGGVFLTLIVGCTVGVIIVVCEFCWKRAKLPYGQRGHILCEFFKEMRHVLACQGSRPNFDSR
EDMDDQTQDMDGAVGTGAAGALHDEADAPGVALADSLHLHQLQRQIANLNSMSSGESGVVA
KTKIARPATLQDIRELRLHDLQYHQQFRQKQQHQQYNGNSHGSGDPQSGSKAHIGSGSFSRQT
SASHLSRTGTLQSRHSNHSVRSSHTTDSVHSMPSVHSNHWGVYEN

>Vd20731

MSSFAASLAATIDPVRSQLLLSLASLSILLSALHRSQAIDHVNIGGIFDQEDEGLANHFTLAVDRV
NRDLGMLGATKFQAKMFRVTPKDSFGASKRVCELARHKVAAFVGPSSPSVKELVKATAFRLN
VPHVSTSWELHERQSPYTLRMYPEADLLARAYRDIISEKKWKKFAIFYDSDDVLVMLKYVLA
DGFNQTAPDVLLYEFDPDLSYEKMLKDIGTRNHFNVIIGLRPDRVKEALLAAEGSYSSFYHSYL
ILGLDFHSYKMEEVVMQRYLANVTMFRLVDPSRSEFQEVNNRPRSNSQLPTDSSIFGHVDAPN
VWAPPSTTPPDDLDVERLTTEEALMYDAVSLIANGVHNLLRMNILGFQQLDCNKTNSNWDYG
QLFLDHMKELTIRGLTGDVRLDKEGKRDMFTLDVLELNKGSKQWSFLKKSSWDPTTGINKPE
GDEAVHDVEFSVSGMILKVVTVENAPYTIINHSLTGQDRFYGYAIDLIKMLAEKANFTPEFYIAP
DSAYGSHIGGGVWNGMIGDLMKHKADIAIVDLTTTAERESVVDFTTPFMNTGISILLRTPEMQP
PSLFSFLHPFSPLVWFYTLTVYILITVAVYVLGRFTPYEWVPSHPCDPDSEPENQFGTISDCFWFT
MGSIMQQGSDLVPRAVSTRTLASLWYFFCLILISSYTANLAAFLTAARMSSPIENANDLAKQTDI
AYGAKDGGSTKRFFQNSNNTVYKRMWAYMESQKPSVFPKTNEEGIQRVLKGDYAYLMEVTSI
DYLVERNCNLTKIGGLLDNKGYGIATPQGSQVRGILEHHLIIMQEKGVLQELKDLWWKIPGEPC
DRVKDDSAEMSMESLGGVFYTLYGGVLIGVFVALIEFWWEKSQAPYGERDHILVEFLRELKLV
LTCTGSRPNPESADNSIADDAASRRSGHSRQASQVFATLNT

>Vd20855

MHITIETSSKTGQSCTLNGSGHIHRLTTYQNHRGPAWGKCRSFTLQRPLKANDAAFKIGTSPRR
QIEEHPLRSNSSVEAAYVTRTNHGQWPSVRHKWSSPSSSCLPNLINKTWPRWHSRWHLAVAV
FVIDCSLLMLPNHVGAAKGLPDRIPVGAIFLKEGETELRAAFEHAIEVHNYRDSHFSVAPHVEL
VESDDPFQVAKKICSILKNGTFAMVATTTASSYETLVSYSNTFQIPIVSPSFPYHTRLRPARYGISM
RPDYLKAILDLIQHYRWKQIVYIYDSDAGLLKLQNLYKLTTDEYKLEIIKVRRVTSASEANKFL
VNYDNETRDQLKHVVIDCEANLTREIIIKHVKHPIVGRRTFHFLLGGLIMDEFANNPAIEFGAVN
ITGFRLVQNNSRAYEEVFTEKSRHQWPDPKTKTITSEAALMYDGTRVMLGAFKQLLNEYPAIFA

KNFRRGEVYNNNTRGIDCKREKVIPWEHGNEIFHRLRNIKGIRGLSGNISFDPYGLRSNYTVDI<br>VEVTVNSELTTIAEWNSQMGFVFAAPKYRRIFVDSGFKSNKTYIVTSILEEPYLMRRKMEPGVN<br>LTGNSQFEGYCKDLADLIAEKLNFTYELRLVKDGKYGGQSADSESGWNGMVGELIRNEADM<br>AIAPLTITSARERVIDFTKPFMSLGISIMIKKPMKKKPGVFSFMNPLSREIWMCIIFAYVGVSVVL<br>FLVSRFSPHEWRYEETFVGPTVSNDFSLYNSLWFSLGAFMQQGCDICPRSVSGRIVGGVWWFF<br>TLIIISSYTANLAAFLTVERMVTPINSADDLAKQTEVEYGTLESSSTQDFFRKSKIAVYARMWEF<br>MHTRKNVFAATYEQGIQRVRESKGKYAFLMESTKNDYINERRPCDTMKVGGNLDAKGYGVG<br>TPLGSSLRDQLNLAVLSFKENGDLARLQNKWWYDRSECKTGDANKESTQNELTLSNVAGCFY<br>ILIGGLVLAMLVALLEFCYKSRLEATRSKTTMYNAMKAKVRMSITGTQNGTGPSGGGNVAGG<br>VGVQGLAEDRIRKSLAEAALYGDSPSSPG
>Vd21106<br>MQLHWVLVGALGALAYSAHEVLAKEKLRIPIGTILLPNQEVYENILRYAVKNFDSSLFDTRLVA<br>MRLSNADFVQINMKVCAMMREGVLAFVVAPDSAPALREMLTSYSSHFHIPLATSLASRSFSRSE<br>YAISTQVSLADATASLLRRYRWSQVAYIFDDSASPDILEAIQNSGVKISIAKKVNNEAEASDAVL<br>ELSSLGRLKPEKIEKIVLNVNRPSLAKTVLSGMIREVKSFKNLNTQFILANPVSDDFWQYIRFRD<br>AGSSLNVTAFRLVDSELPHVTRFHKQMMQLSSHRSLFHNQGTQSYLFLDAVTALLTGFDRVLK<br>DNPRLLDRNLRHHPGSVYFNGTKGLDCMDQSRFFEYGDILAAFIKQIKIPQGQSGPVEFHDNG<br>ERVQPTITVIQGSHKGHVRLGTWTAKKGLMLADKPEALPQIKAPGAITPAKELSVGSVLNSPYL<br>IQRQTDGEFSGFVPDFLEALSNVVPFAYKIQVVRQRDYGRRSENGTWSGLMAEVINKKVEIAV<br>SDMALTAERQEAVECTQPFFVDDLAVVMGRTTPAGRPSLSFFFVRVFSWQLWACVAAILAVFIIF<br>SYILNHLAQSRQRNSSEKNRLLCLVWFTLASVFARSQGPHVNSKAYRILLASWWLFLAVMTIT<br>YLSACIALLQAVLFGSGPDLFNKDKFCRDAANGAVDVGYMRHGAVEKMIKNQAETQLSFYAQ<br>LLNNSRIRIVSGIAEGMRHVGPNYAFLGLKSQVDSYIGRDCGIKSYTLIPQFAQYALVMPKMSP<br>YRDMFNQGIEKLRESGVLADLKEKWIDSLVRCPREVEVLEVPAPIRYTALCGMFIIVLAGFILAL<br>LAGVIEFCWSAKRDSSKSEKPIQKVMWDKAHDSLKPFTQKDPALGNDEEMTLNPSQANGTPK<br>QVRVMV
>Vd21675<br>MRWIAFVLVLCTTVLVQAGDLPVMKIGALLDEDEQVASGALMDTLVARINADHKILPRFRLHV<br>VRVKVTNSFAAAKATCALIDDGCVALIGPTQHESHRMAQEIAARLQVPFFARGRPGASVGMTG<br>ASPAGGDNSGPSSSCSTCFDLLPPSRALSRAFADILKAKKWKNYALIYEKNQDLVELAELIKNK<br>NILLYQYQRGERGTKRLIREIGSDPQYNYIVQLPADRANDFFRLAGIVGLLSEYFSYFVTAQDFH<br>TWQLPAEALSNITALQLLHQEFVSLPSDSNGKRQRGGPKQQQFIHQHQASQQQQFQQQQQAQ<br>QQLQTPPAPLKYHTALLHDYIFVFARAVGTLSRTQNMAPVSDASCHKPRNGWPMGLTIATQIKA<br>TSMQGLFGNIQFDTFGARSNFSAFVTEHKHGTIVKVGTWAPSSGFSMTRNVSWDRVHAEHSLR<br>NRPLRVVTLLSPPFVMWRQQQNSAKSERNGDLEGFCIDLLNELSRMLQLKFDIRLVNDSQHGS<br>RDRQGNWNGLIKDILDMEADVALADLTVNVERARVVSFTTPFLPSQLELLYRPPSTAGDLVSLV<br>KELFSPLSQEVWLSGVACVFMATFAMSYASKFSPRDRVRRSSSKQANIQTWTKPRFSFGDIFHL<br>VACCALRQPPISKRPRGVATRFLTSVLCFTSFVFWSVYTARLTAQFVQRRMHFDELHDAGDLLR<br>VKGIRFGYVANTSSQLFLQGANYEPFSTIWSLIMGAGDISTLPSLEEGLRLVQAGGYAMFTETPA<br>AEYHQCSISDCSVLRLATGIETPGYALALPRGKACMKNTVP
>Vd21743<br>LNPNRPNVTYVMIVPSTRFESVRRKYRSTINEALNQIKNGKVPGNHLNKHYSLQFQLVYLSLA<br>PTPREILDTLCTEILNASVLSVGYFTNSETFGSNAASVEYVHQLLGYLGIPVISWNPDNIALDER<br>VTDAHILGLSPSVDHQVNSIFDFLDRYQWTQFAIITTQLAGHENFIRAIREKVFHKQHKYSLIAA

HTLPRGGDADKYFNLLQELADGEVRVIVLFCGTEDAREIFLASSRHGITGKNYAWIVTQAVIGS<br>AERSPAEFPTGLIGMYYNFTLPVLLDEMEKSMYIFASALERLVNHTTEENRNQLSTGLTCNASE<br>YSYWRKGEEFYKYLKETEWTSKKFNSVAFNFDGTRKRVDLDILNLDARNVWEKIGDWGESG<br>VDIKDIIWPGEERKPPKGVPEKFNLKVTFMEERPFVIVGLPDPETGECESSRAVKCRIAPESALIG<br>LNDTMARRHPNYYRCCMGFCIDLLEKFAQDLGFTYDLSKVEDGMWGVKDKNGKWNGLIAA<br>LLNRHTDIVVTSIKINSDRQEAVDFTVPFLETGIAIVVAKRTGIISPKAFLEPFDTVSWLLILLVGI<br>QVAAFSIFIFEFLSPDGYDMKIAPPRNYKFSLFRTYWLVWAVLFGAAVNVDCPRGYTARFMSNV<br>WAMFAVVFLAIYTANLAAFMITREEYYDLSGIEDSRLRYPHLMEPPFRFGTIPYGNTEQVLQRN<br>KPTMYEYMRPYNRSNVNEGIKAVKKGTLDAFIYDATVLDYFVGQDDECRLLTVGSWYAMTG<br>YGFALPKKSKYLQMFNRQMIEYREHGDLERLQRFWLQGACKPDKRKRNAGNPLDINQFMSA<br>FLLLGCGVLLTVVLLILEHVYFRYCRKQLAKANFGSCFSLISLSMGKSLSFRDAVYSAQGMLLR<br>RATENRFLQAAEHERLQADVARLARELDVLRQKIKTLESDLTTTKAGSNGGSLDELRGTGIDTT<br>GSIPGSQRVARPQAPKRTPFGGPLAISGSHPNVADKIVGLRQASIHSNTSVGAESPTRSLARFTGS<br>HEDLLRHRDLTRTKIHSNEPLVISQASILPAVPPHSVLFQQQQAMVKAHIMEKETVL
>Vd21758<br>MLITDDISMLSIRSIILFLVVTLFAGCVEAGQPFSVNIGGIFASNSPLVRVFDFAIRRAPESSRLPIV<br>RDAFIGMKKVIPDIHSSFDASKAVCQLLSRAPSIVVGPTVVPVSHILASTCRAFHVPFLSIGPLLS<br>RTSAASPRKLPQMLNLHPPSDILGRAYFDVLNMTEWRSLTIVYDREDAFLRLAPILRAAPSLTTL<br>RPLDKSVSPGTLFKEVLRSKDTHIVLDVDTQLIPELLRSAKDVGLMTEYHNFVITSLDLHTLDL<br>SPFFPSRANITFLQLLDLKSKENLKMIRDFRRFQLMDGIRDNLNLTTEQALLIDSVSFVVRTLYE<br>LSASSKLRSPPPFKCQSPRPWQTGDLVIKKMLRSSFRGVTGIITLNRHGLRSRFQVNVMRIMWN<br>GVAKIATWNQDSGIRTQASYPATFRKELENLMASKVFNVTTIVNPPYTMLKSDYRNRTGNDRF<br>EGFCMELMETLAANIGFRYSVHLVKDNSYGSRQPDGTFDGMIKELINMEADMAIVDLSITAER<br>MQAVDFTQPFLKTGISILFRKPAQGGLTLFLFMKPFSIDVWICMLTAFTGVTVLYYLIASISPTEN<br>RDLGEDDLSQRDPQLLDQEMKIMSRFWFTIGSIMQQGCDLNPVSLSCRTIASIWWFFTLIVVSS<br>YTASLAATLTAERLVSSIDSADDLARQSTIEYGCLASGSSRKFFEVVLLPLQDNSHSLKTAVLFF
>Vd21835<br>MRLQAKNVEMHRPGFGFIVLLVLLGPALAAVKIGGIIAAGQPRTKELLEEILKERSSGLPTKNR<br>MHPELAYVEPNATFGISNTACAQVGQGVSSIVSPCTGIVHATLQSLASRLAIPYINTAPDLVDTTF<br>NTKQLGESEKSITIVMAPSTAKATADLIRKNQWKDIYFIYDKPEGLEELELILSHLLEEDEVQVK<br>GIHQVTSNTSAMTYLQEKENAAWDVEKHIILAIDVKMAKRIIQHSYSKRRAYYHFVVPRFTVR<br>HFWSEVRAFDQSTVATFQIVEETEFKTMKGMRAGVSTPKNSEDEHIHEEEALLMDAGRLLSDT<br>MVALSGKQPLAAEDIFKELSQITAAVTCYRSPLEEYPQGQTIADVMRKIHFKGSTGQVRFNDHG<br>RRTGYTLDVLELTSDADMERVGKWNEQEGLAFSNEHERNSKRARIKQELSKSIIISTIIERPFLFF<br>KNTNETNPHIDNVDGYIKDLIVRLMKDIPGRPQFNIHFVKDGKFGYKDSQQASGWNGFIGEILR<br>KDAHMAIAPLMITSERSKVVHFSQPFMATAIGAVARNDADDLLNIRTPFTFLRPLDWKIWLLFL<br>GAYGTVAIVLLLINLINLAVATPPVDPPTPCRHFYNSLWYAFGMFMSEECNNCRPKSLGSRVIAC<br>SWWFFILCVLSIYTAQLWINDPFAPTGEKLPRCLKELLYQDRIEYGLIRGSATEDFFQNTDDSEY<br>KAMYRRILSFNDSVIESYPKAIDRVRNNSKFIVFADSNFIRYTSAHTPCDTIQVGTDLRRGQYAV<br>AMSKHFALSKKIDDIISSLRDEGFLAELEDRWFGAHALDCPADNLVIAPSLAYGGEKPLDIMKP<br>LEVRRVLGWFIVLGLGLIIALFITICEVYCKKPKKQRTPEEIVERQTAREAKKSRSKGKKDKAEE<br>VETQQLTAHEAPIGNSQMDDATRMGVGIANAADDATLKKGGCLPREMKIDEEYLDGDDGGFR<br>RSELPPIADVEPFTDTDSRDAIQSPVSGQSTIKMHSGNTTEAVNAGLIGIKPSSQSPPTLILKSPQQ<br>PCQPIQPILTQSQPKMPITTQIQPTFQTQPLTTKSTIPQMSKAPQVAQMGRSMSQPVASLPPLSQR

VPPVQLPPTMAPIKNTAPAQATPTSAPRTPSPAPAGPPIPKSTLPSMSPVGPMSPLSPASPASPASP<br>NALPPLSGLSKLGGDPPPPAVVNQGPLSRMELEGQPELKSIDRSPADTPGTPHDDLERIDPILGN<br>LGSLAGLPPPPANIRSTIQNQDSIGYESSEV
>Vd22269<br>MSGVVAPVLALALLGVAVADIKIGGLFETGEDIVEMAFNSAVERINSHGLAEPGERYTPTSDRLL<br>TRVEHIERADSFQASRKVCTLLEEGVAAVFGPQSTDASFAVRSACETLDVPHIETHWDYRSRTT<br>NHSLNLYPHPSALGKAYLDFIKYKDWKNFAILYEENDALVRLQEILKDATLMKENRVTVRQFEI<br>GTEYRKTLKDIGKTGIKNIVLDVPIRSLYTVLKHAQQVDMMSEYHNYFITSLDAHTIDTEDFQY<br>GGTNISAFRLIDFSSKEIDEVARDWIQRRHRFSSKKSSSVQSNFNTGAQDFYKNLTTKVALMYD<br>AVRLFATALKDLHPDSGPKIQVAPLSCEREHKWSQGNAFVKYMRMINIKGLTGNIRFNKDGHR<br>TDMRLTVLDMAHSGWREAGEWTMHGGISITANYTRELEEARLSLLNKTLVVTTILQPPYVMY<br>KENWRDNGLTGNDKYEGYCIDLLDAIASSEHFDNNMKYVIREVADNSYGRKDADGRWNGMI<br>GELLSGKADLAVADLTITFVREEAVDFTMPFMTLGISILYKKTAKPPPGLFSFLDPLSLEVWIYM<br>MTAFLGVSLFLFVLARFSPYEWVNPHPCEGNPEELDNQFTIWNTLWFTIGCLMQQGCDVTPRA<br>LSTRVAAGMWWFFTLIMVSSYTANLAAFLTVERLVSPIESVEDLAKQTTIQYGCLRSGSTQSFF<br>KDSQFPTYAKMWHVMQSQRPTVFAESNQKGIDRVLRGKYAYLMESTSIEYNIERNCDLTQIGS<br>LLDNKGYGIATPPGSPYRTMLSQAILRLQENGDLHVLKERWWKKKRISKKCPKDIINKGTSAM<br>TVASVGGVFIVLLVGSCVAIISAIVEFVWRAKKIVPEERDPVCVELCRELKFTLTCGSKKTPDTPS<br>LG
>Vd22292<br>MHNIMTVVQKALKALEQIEAMRRRVKTSVHYPTIFECRSGRAGANLLLSLLVVPGVFASSVPT<br>VTADLHSNGNSASVLDPEIISKAVLDADLSVNAINLAAEELPRVYNVGAVLSHPEGIAYFEEAIL<br>DAMATYNIDLVAKSVHMSLNPIRMAENVCEQLISQQVYAVVISSPLKGELSPAAVSYTCGFYSIP<br>VIGISSRHSSLSDKNLHRTFLRTVPPYSHQADVWMKLLRHLGYQSVVFVHSSDNDGRATLGRF<br>HSVAAQHKNAVHIEHVIEFESGVSETDLTAALRNASRRHCRVYVLYADTSEASKVFDVVKKLD<br>MTTSGYVWIVSEQALKAPNCPDGVLGLELVNAVDERAHIRDSVNLIALALKKLQKDSKVAAP<br>QLNCSNLEHNWDAGLKLVSILKEQVLESGENGHVKFDQRGDRLNSDYDIFNIQRDKESYLVK<br>VGEYMYSEYDMEMELKINLNAVSWPGKATEKPLGFVIPKHLRVATLAERPFVWSRKVNSSTEC<br>SSVEILCPWYNRTADTGHDEAFCCSGYCMDLLHFLASKLNFTFDLYLVEDGQYGNLDSNVEG<br>KRVWTGLIGDLVRKKADMVVAPLTITPERSMEVDFTKPFKYQGITILAKKQDKSSTLASFLQPF<br>QKSLWILVVVSVHVVALGLYLLDRFSPFGNYRVLPSERDEDGLNLSSALWFAWGVLLNSGMAE<br>GTPRSFSGRVLGMVWAGFAMIVVASYTANLAAFLVLEKPESSLAGINDPRLRNPSENFTYATVR<br>GSAVDTYFKRQVELQNMYRIMEGKNFDTVDHGIHALMNGNIDAFIWDSSRLEYEAARHCELL<br>TAGEQFGRSGYGVALQRNSFWVDKVTLALLEMHESGFMEQLDSLWIHNGGRRCESKLERTPA<br>TLGLTNMAGVFILVGAGIFGGLVLIIIEINYKRYLAKQKRRMQIARKAAAKWRGIVERRRSLRR<br>PSHHRIMLPSNIQQLPVLIGSLASGMPSAQTVVPAHSGASKHENLGHQGLSRLKSASTSIKSSHE<br>TRSDLADLPPPPPPPARVQATPGTRMNASTRYLLGAQENKPVSFDINV
>Vd370<br>MGYRSGSRHLAGVTTLSVLFLCGWMNTTGAVPARVHKDVGTIGIIVEDQDLAKHFLYIASKLV<br>DSNLKAVSKVIQNDDILEAIQSMCALMESDVNVVFGPTSPVSSLHVSSMARHFDLPHIEFRWDP<br>SLIDDEHSLNLFPDPVQIAEAQTALMEYWKWRDIALIYESDDDIIEMRRLLQELQEDGITVNYF<br>KKDKGETFRTLLRQIKERDIRNVVVDLEQKAEREKDKEGKIESFLKQAMQVAMLNENYNYFF<br>LSLDFHVAKLNDFMHSRANITALRLVDMKHHIADDLLRGQDSPQDNKKGSASGRHNQQKAA<br>MARARRDRNDRRLLKRDEHNGEADEEEADEDASFPPGLETEVALIYDAIMLFKLGVKDLVN

KKSSYTKALPTAKCNGRKSLDSEHLGTELTKTMLRRTFEGATGRVNFNEKGIREDFVLFVTELG<br>TDGLEEIGIWSKSSGLNITNDENEFELSKEEKTKIDDIHLRIVTVMIPPWIMKDEKLSTEDRPRYK<br>GFLIDLLEDMKKRSPRPLDYELYTSPDGSYGVETRYENGTITWNGMIGELVKEKANVAIADLIV<br>SDERLRAVDFTLPFMTAQLVAVVKAPVPRKPGGVWSFFLPLTREVWMYTVVAGLLTALIMYMC<br>ARFCLSEYVNTGDGVNGPAEEVDNQLGIVSCLLFVFTTLLHQRMHLDPNATATRVLAGFWYFF<br>TFVILAIIVGNLCESVLYEDEDFSKLETIQGLLRIRGMKFTCIEHGATCRTLMTSHAPVLWEINKR<br>MFKDETPKVKNMTHALAKISQDEHLAFIMEYASAKYLVASSCDLVMTKPFANHASYGIALNK<br>KNPGKTNRLLSEIVLNLQAKGILQEREERWWKTKRFCDVDNDGDFDDVDDGDDDSINEQKVE<br>LRTLSPGDLSGAFILLFLGLCFALLINIGEVIVEHLTREKRDTYFNMITVVDHIRFAFKMQTNQV<br>DLSETSLPRNHWKDQLQKRRENRTRENGQKSNDPTSPRKTDSLSSKIPQYQ
>Vd52944<br>MTWLCAVLASVTVVSISFSTGVHGGTYKIAGVFSAETGQVESKVFARAVDRVNTDNREFPEVQ<br>MQAIPIMLKEGQTWENYQELCRQFESGVVAIVGAESSVEDSLISSVSSAVRIPHIRTGFNRELED<br>PDDLSLGLAPSPSDLAEVVRELILHMGWNHIVYIYDTSTGLLRMNELEEDPEFKGRRLVPIDLS<br>VDANPWDNIIKLSIRHVVVDIESKSVHQVLKEISMSGLSQDYYSFIMPNSDIRLPENSTQRTFFID<br>ADVYTFSLVDPDNREMHWIYRMLEMNSSENEPNLPFIYDSVLQIGHTLNNLTKLQRPIRSSPGN<br>CTLKVAFKDGRKLYDSIRESTKFEGVTGFVEFDDKGARKVKHVHVKLLKDGKLTKVADWRPL<br>DGLTFIKELDPKGEPDEALEHDEGLNNRALVVTTILLPPFLMRDDSPHLKGNERYSGFIVDLLE<br>KLARLLKFQYLIKEVEDGAYGTYNATTNSYNGMIGEILLGKADIAVAPFTINAERYQILDFSFPF<br>LEAGLGLLARREVDPLEHNFLSPFSAIMWFSLFGTMMFATFVITFIARLSPREWSSVTDTVIETH<br>FTVGNCFWYLYSTLMFQRFQHRPNSLGVIVAFFAWFIFCLAIMVSYGTVLGDFIIKYNERPALSK<br>IDDLRSENNRVKFSAIKTGSTMRFFEETEYPNYKQLYAKLDKDAAVRNLSEAIQRVKNEDFALF<br>LETPYIEYFVGHDCSLQQVEGTVGSVSYGIAMSQGSSLQKYLSAGVLSLRREGSLQSMRAKW<br>WNEQHAGRHCPDHLHIVRPTPFTVRFGRVNMLFVFLLGGIILSVAITIYNYVRKQMTIEDKNEF<br>RYFLPTLIREVETLFRKRPVPTNPSPEPTAA
>Vd17150 (25a, MF069507.1)<br>MEILLRVVYELLVTLYFVENGLGQTLNIAVIVEKKFELARQAVEAGLQRAKNDGKTIHWQFVV<br>MDADDELKDLCDIIMENQPSLVINTVRTPGSNYYKLTKEIKQTVRNLAIPTLDLSYGSTDEYQM<br>SGWNGKKPEENDYLIHVTPPGDSYTQAVRELSIKMDLATAGILYDKSVIIDHKYARLLENVPTR<br>HIMRQVSESSADFVNKTKVIQSTDVSNYFVVGTISHLSNALHSAQENDWKPRHHCWVLITKQE<br>GEPVCEDCMNHEVLFVHPADKEGKRIGIDGAYGSEKLDGLFYEEITYYMITRIFADNKYSKQPI<br>RASCREKPATRDDYNLLNALESSKLSTRYGDIYFTDAGNKPGYQAINMKVAHFNYTASKTRQD<br>NEKGFYVYGGDAKAAMWTKADQTALEQFGSVTFYKVVTLIQPPFVYKGNGSGPEFYGYCIDL<br>IDEIKKILKFEYNIYAVEDKHFGSKDKETGLWNGLIGELVKKKAEIALGPIAVMAERETVVDFTV<br>PYYDLVGLSILMKKPEIKPSLFKFLTVLETNVWGCILAAYFFTSFLMFVFDRLSPYSYRNNKEK<br>YKDDDEKREFTLKECLWFCMTSLTPQGGGEAPRNLSGRLVAATWWLFGFIIIASYTANLAAFLT<br>VSRLESPIESLDDLSKQYKVKYAPQGQTTASTYFHRMANIEEKFYEIWKNMSLDDTLTDDQRA<br>ELAVWDYPVSDKYTKIWWTMQEASLPLTFEEGVARVKNSQGSDGFAFIADATQIKYATMTNCD<br>LTQIGSEFSRKPLALAVQQNSPLRDQLSSAILKLLNQRKLESLKEAWWNGNPEKKNCDDEGKN<br>NDGISIKNIGGVFIVIFIGVVMACITLVFEYFWYKNKKPRSKVVSVKDHMPQKK
>Vd18850<br>MAVKSMEYQINRVTTVHHRYEANIPADQQWGVIFPNGSATGMIGMLTRNESDWAANAFGQTL<br>DRHRVVTLSSEFMVQDLSILAGRTFGFQDNYFGLFGAFESNVWTCLLMATLVMPVILVLSRLLE<br>RNEGNVNRTLFSRMLVLYGRNLMSVVRTILFESIEDRDLPKGNSGRILIGLWLTSIYLTMSIFQGT

MKASLLISSGSSRIDSMRDLVVRKQVVPIIWRGTAYHKLFQRADMDIYQTVYSRAMEKNGILP<br>GNQLYTNENFMNVLQGRAVIINEQSSIMYNLGRSCDALRGHPGEFYFAKEPVYTHGLSMSLRK<br>TLHPMLKRVINKTIRELQETGNIRKWLNIAMADFFTCEIVNGAPTQEEPTVYTLGLSKLKGPLII<br>YGMGAVATLITLCAELAWESKCFCFIWQNGKLIA
>Vd19098<br>MTEVTSREGRVVNVNGTLVELLKELARKLNFTYTFIMPPEPVPGLKQSDGRWSGLIGLLVRQEI<br>DLALYDFTPTPDRQQAVNFTVAFDESPYKFLVPKPQPNYKYLFLDPFTWDTWLAVLGTVLIIGPI<br>LWCVHINSKFYDYYDMRDGKGLFKLANCEWYCFGAIIQQGGIHLPDAISGRILVGFWWLFVIV<br>TLTTYSGNLVADLTFPKIRNPYDTVSSLLNSDITWGAFKGHAVIEILKLQQQGLLTQLSAKLAHI<br>ETAHESWALKEVADGKMALIGSEVTLFHYIGKQFIATNLCQYAVAKKEIIRQVKVLAVRPGFPFL<br>ARFNTLLTRIVETGLIIRWKKKYWPKENECTVDSKPQAGDIRRITIAHMEGSFWILGVGFLVSFT<br>LLALEIFRKRRELRDPNGGRPQRIGPRDFKDNLFRKTNYSDKDHYNTDYGAQVGGAGPGAGK<br>GGQSGYGFEPSNTPFRGYSGFPNRTDLIPYN<br>YPARRY
>Vd22240<br>TDDFWQSIRFRDAGSSLNVTAFRLVDSELPHVMRFHKKMMQNPNYRDSFKNQGTESYLFLDA<br>VTALLTNYDRALQDKPTLLEAYLPQEDGSKGTVGFNCMDQSRFFEHGKDLASLLKQIKLPEGQ<br>TGTVEFLADGSRLQPTIAVIHSSHVGHVKLGSWSPKKGLMLTDKPEALPQIKAPGAITPNKEISV<br>GGILSPPYLMLKRGSDADFTGLIPELLEALANVMPFSYKIHAVPRGQAGARTDNNASWTGLISE<br>VINKKVEIAIADIPLTADRQQHVEYTQPFLMDDLSVVVSRKSQAGRPSLSICFITIFSWQLWLCIL<br>GVFFTFVLLAYLLNRLVSPQEEADSFGRTLWLSAGSFLCRSPGPEGRCRASRLLLLFWWFFLAM<br>VTVLYVSAFVAIVQTVVFGYGPDVFTMRKFVQDAASGTVDIGYVRDVVPGKVLK
>Isc000549<br>MAHSFFNKTLLDDFVESVLFWYPYDPHAAFSETPQGIKVTTGLLGDIIRELSHSMKFNETPQGI<br>KVTTGLLGDIIRELSNSMKFNYTVSFTPERTLGIRHPNGSWTGLIGSLQRDEGDFALGLMIPTNS<br>RNAIARPTTEVYIDEITIFAGRTHSKSMNVFSYVWVCVVIAMFTLAFLSTGFDYFYEHTVRNRK<br>SFSERFFTHLWEYFENLLGKGSSVESDWHATRLLSGFWWIGVTVLVFAFAGQMRACLMVKSQ<br>EAMIRNVDDLARRSNVKPYTLSGSILTTILRNSKKPSYTKVFQRIVKGRGQTELHQIYGPKILKE<br>VVQGKSVVIADRGSFTYKVARTCRNYTDGEFYIAAEPILNFRFVMYLSATMDPRTQWEINRRL<br>VWLRENGIVGKWIKMLGDWEHCRQESSRVTLSFEDTYAIFVMWALLIAASLVAFGCEKLHHA<br>CAAAKERPSQRPGPILGWGVTTVSRKRPKVAKPFQAYNFKV
>Isc005598<br>ILRRLCEDLLPDDVVAVLYLTNSAVFGSNAASVQYVLQLLGYLGVPVIAWNADNIGLDQRVSQ<br>ARVLQLAPSVVHQVAAIFAILERYAWYQFAVVTTQLGGHEDFVRAKNEHIYSFSVLDVYTLRG<br>HTRQEFRSQLEPVATGEARVLLLFASKEDSREVFAAVGQLGMTSKNYVWIVTQSVIGAHPGLAP<br>QEYPAGLLGIHYNTSLAKLHDEIERAVLVLGHGLELFVNEPANANLSLNPGLRCYEVGRAHWS<br>QGDHFFRYLRNVSIEAKGSPNIEFLPDGTLKYVELDIVNLNKDGYWDRIGGWTQKGIDIKDIV<br>WPGNELVPPRGVPEKFNLKVTFMEERPFLNIGQPDNETGECESGRAVKCRVVPEAMLQGINDT<br>AARKNSTYYKCCLGFCIDLLQKFAQDLGFTYDIHRVEDGTWGVKDNGTWNGLIAELLDKKAD<br>IVVTSIKINSDRQMAVDFTVPFLETGIAIVVAKRTGIISPKAFLEPFDTVSWLLILLVSIQVAAFSIF<br>VFEWMSPSGYDMRITPPREISLFAEHRFSLFRTYWLVWAILFGAAVNVDCPRGYTARFMSNVW<br>AMFAVVFLAIYTANLAAFMITREEYYNLAGIEDNRLQNPQQTDPPFRFGTIPRGNTEAVLKQNK<br>PQLYAYMRPFNRSTVTEGIRDVKRGELDAFVYDATVLDYLVGQDDECRLLTVGSWYAMTGYG<br>FALPKKSKYLAMFNRQMVEYRERGDLERLQRFWLQGACKPDKPKRNASNPLDVNQFMSAFL

LLGCGVLLTLLLLFLEHIYFRYLRARPWLRPKITLVTHPRHHHAQSMGKSLSFREAVYEARMRR<br>RCRDPACDNQLWKVRHQLDIARLRVSQLE
>Isc007957 (XP_029841705.1 ionotropic receptor 93a [Ixodes scapularis])<br>MNNKNMMTFHREFVSVVTQPIDDDFQKLVVGFPDGANVLAAYPEIADNDCPVEPGCQLPLAM<br>ETVAKTIGDKLEKGTYRTTEFFTTKFIFSNTSKSLLLASGKCGQCARFIIRSVAKVQGIQEFLKIG<br>EWTPAVGLKMTHKQFFPGIMGNLGGIRLTIGVINDPPMSVVEMSPDRKTVKNVTGTMADMVE<br>ALAKGLNFTYTWKVPKEEIPGSKENGNWNGLIGMLATGEADLGAYGFSVTKERSEVVNFTSA<br>YDESPYKILVPKPRANYKYLFLDPFTWDTWVAVLVSLVLIGPILWGIHCASPFYDYHGLRDNKG<br>LFLLQNCEWYCFGAIIQQGGIHLPEAISGRILVGFWWLFVIVTLTTYSGNLVADLTFPKIRNPVDS<br>VENLVAHRGYMRWGAFKGQAVFELLKSQEQGPLKVLSDRMNVFEPNHEMWVLDQVRLGYM<br>ALIGSEVNMFHYLGRELNRTGECDFAVARGEVIRDVKSLAVAPNFAFLERLNNEPDHDGRPPPR<br>RLKRLVESGLVMRWKKKYWPQDNECTVESKPQAGDIRKITLRHMTGSFWVLGVGFFSSFAAL<br>FVEFVRRKRELTAPPTHKPPTVIHTKSPFFTRTEYSGKDTLTTDRFATDYGGRGPRDNAGFAFSP<br>PNSPFRYNGYPNNRSDLIPYNYPARS
>Isc008225 (IR25a)<br>MQPPFIVRKNASNGENQFEGYCIDLINAIKEILKFEYVITEVKDKMFGSIDKNTKQWNGMIREL<br>VDKNADIALGPISVMAERETVVDFTVPYYDLVGISILMKKPEVNFLMYLFDRLSPYSYRNNKE<br>KYKDDEEKRDFNLKECLWFCMTSLTPQGGGEAPRNLSGRLVAATWWLFGFIIIASYTANLAAFL<br>TVSRLDSPIESLDDLAKQYKIKYAPQKDTSSMTYFERMAYIEEKFYE
>Isc008266<br>MGTPEVLCLLLLGCLAAHADIKIGGIFETGDDVLEMAFSSAVDRVNTMGLAEPGTQFVAAGSR<br>LLARVEHIERLDSFQASRKVCSLLEEGVAAVFGPQSGEASAAVRSACDVLDVPHIETHWDYRA<br>RPDNHSVNLFPHPAALGKAYLDFIKHKDWRTFAILYEENDALIRLQEILKDPLMRERKVVVRQF<br>ESGVEYRKILKDVGKSGIKNIVLDVPTERIHTALKHAQQVDMMSEYHNYFITSLDAHTVDMED<br>FQYGGTNISGFRLVDVNAKEFDEVARDWLTRRLRFSKKGEKEENFYRTKVALMYDAVRLFATA<br>LRDLQPDNGPKIQVRPLSCETEEPWAQGNSFVKYMRMINVQGLTGNIRFNPNGHRTDMRLAIL<br>EITHNGLREAGEWTMHGGINITTNYSRQLEEARLHLMNKTLIVTTLVQAPYTMLKENHAELEG<br>NAKYEGYCIDLLEEISKLPDISLKYKIREVADKAHGRRDDKNEWNGMIGELLHGKADLAIADL<br>TITYVREEVVDFTMPFMNLGISILYRKADKKPPWLFSFLAPLSLEVWIYMSTAFLGVSLFLFVVA<br>RFSPYEWVNPHPCDPNPTELENRFTIWNTLWFTIGCLMQQGCDVTPRALSTRVAAGMWWFFT<br>LIMVSSYTANLAAFLTVERLVSPIESVEDLAKQTTIQYGSLQSGSTQSFFKESEFPTYKKMWHV<br>MQAARPSVFTESNQKGIERVRRGKYAYLMESTSIEYAIERNCDLTQIGSLLDNKGYGIATPPGSP<br>YRTMLSQAILQLQESGTLHVLKERWWKKRHIVKKCPKEEASASKGTSALGLANVGGVFVVLL<br>TGSCIACITAIFEFIWRSRKAVPEEREPVCMELCRELKFTLTCGGKKPVQNKSEDRQPPGENGLP<br>FMPLTGYNAVVSKDTFS
>Isc009282<br>MAPGSRPRMRASGRRSAPQWARSHRSHGAFRAAPRPVAGAMARLAPPVLVAWLLLVPAVLLV<br>AVDSTPALLAALPPRSAPQLARALRRALARWGAGPGDASATSWVVAAFSAVPDTRAALSALCT<br>ALEGHAPLLVVMPVLAYTGGYLDRAAQEASPLYLSLDPGLPELTEALFGLLSQNRWYHFVLVT<br>DESAASSMLAQRLGSLCHGVPWRPMLRLALPGPTQPLLGPLAKITASQARIVLLFVEAPMVRA<br>VLDAARTLGLLAGDHLWLFVERDDPNPASPHPCGGDVEGSALKGCPLGALSLQLRQSHVERT<br>DAPRLLAGLLHDGLSRWGPIEYPLALRNASGAPTPSCWEEPSARREMFSQRLHGYLRAAAAD<br>AVQSQRFPPPVFDILNLVPAEGGAQWRSVGNVTKGSAVLDAVLWPSTGEPTLMGPRPFGQPRFR<br>VVTAYAAPFVMAATRLRNGSCLTGVPCLQVSTAISDELAALFAHYHRTGGHTRHPGYNVTCCA

GIAIDLLKSLARDLSFDFDLYLVADGSFGTDRGGRWGGITADLISGAAHVAATAYSVTSSRSRRV<br>DFSVPFFHSGVSCLAYAVKRDVPLSAFLVPFSVPLWLAIFLSLKVTALAAALYEWFSPFGLNPW<br>GRQRTRNFSLASALWVMWSLLFSHLVAFKAPKSWPNKVLINLWGCFSVIFLASYTANIAAHFA<br>GLFFQMQVHDFHDTSVPEGYVFAENKRLWEHIQRFGVASLEQGLESLRLGTLDVLIGDTAVLN<br>YYRANEPSCRLRLLGDSIFDDAYAVGMARGFPLTAAVSELILRYNAVGYLDQLHGKWYGRAHC<br>LQDGLLQRLDKPMPLGVRAVAGLFIMLLVGLLAGSLVLILEHLVFRYALPGLRARSKQCFWKSP<br>NLMFFSQKLYRFINTVELVSPHHSAKEIVSNLREGQIASLFQKSVKRKIKEEARRRKSKSQFFEM<br>IQEIRRVVRQQQTERDEESPTSPHEGPFSATSSIGPLLVREPSPPSGSSSSSAPTSSWVHGALLSVS<br>LEELPSVAPPACRRRRSHSLGDLSHMRPAWQPRRAPPSCASDLDDVRLRALSREQLLHRWRDS<br>ERRLLNLLREALREKQALERKLAFLHSALRNKPP
>Isc010976<br>GASFSFRRLLKKQKLLRGETGRVRFDDRGDRLDSDYDILNVVRGNHAPVGQYWSMSMELDL<br>ELDRVVWPGNRTETPLGYVIPKHLKVATIAERPFVWVRPVPDESDCLPSEIFCPWTNSSDGIAYP<br>FCCEGYCMDLLQSLSQKLNFTYTLYQVQDGQYGTFDFVNGSTHKRWSGMVGDLVRGVADMII<br>APLTITPERSMEIEFTKPFKYQGITILAKKANKSSTLASFLQPFQKTLWILVMVSVHVVALALYLL<br>DRFSPFGRYKLPNSDATEEDALNLSSAIWFAWGVLLNSGIAEGTPRSFSGRVLGMVWAGFAMI<br>VVASYTANLAAFLVLEKPESSLSGINDPRVGGVFSEYFTRVSSPGDETTRGRPQREMALASATRI<br>LPHTKTFSSGGAPNIVNDLQLDAFIWDSSRLEFEAAQHCELVTAGEQFGRSGYGIGLQRNSFWV<br>DKVTLTLLEMHESESPLSVSFIILSSRQGEDTVLPRAWLGPFISAGVFILVGAGIVGGIGLIVIEIV<br>YKKHQTRKQRRLELARHAADKWRGAVEVSQHTPTLFPTMRHRGVQLVAPSAVPLTLSGLPQN<br>GANIHQGLNSHWNDGHRSLLHHK
>Isc012402<br>GGIFESGSQKAQLSFRYAIDQINKDISILPRTELMPIIHEVKQKDSFTASKALCTFFKQRVSAIIGPT<br>SEAVTSFTKSACTNLNVPHLQTSWEPKSNRNPYTLNLFPDPEVFGKAYLDLIINKKWKSFTVLY<br>EHQDSLILLKDVINSSLAQNHSVSLVQVNPKLSFKKMLKDIGYQKQFNIILDVKTESLPLLFRQA<br>MEVNMMTYYHNYIITSLDLHTLDLREFVGLQANITAFRLVDPSRPEVMNVLRDIKIGSALWRY<br>GADNHTSPAHIDTDTALTFDAVALLANGLHNADRVLQTLDLQSLNCEHPKPWRHGSSLLDAM<br>KMITIRGLTGDVRLEEKGVREEFTLDVLELKARGLKQVATWHPRTGLSYFANYTLSEKEFTDEL<br>EGFTLKVTTVETKPYTMVYPDSENKTGNDRFYGYAIDLIAMLAKEIGFKHEFYLTEDGKYGSP<br>QPNKEWNGMIKELLDRKADAAIVDLTITYERESAVDFTIPFMNTGISILFKKAEKSDPSLFSFLYP<br>FSVDVWLYMLTAYLGVSLLMFIMGRFTPYEWVSPHPCVPEEGDLQNQFNLPNSFWFTTGAIM<br>QQGSEIAPIAISTRIASSMWWFFSLIMISSYTANLAAFLTAQRMTAPIENANDLAKQTKIVYGCK<br>EEGSTCLFFKNSDDPLMKRMWTTMLAARPKAFADSNEKAVERVKKGNYAFLMESSSIEYVVE<br>RDCNLTQIGGLLDSKGYGIATPPGDNL
>Isc1653 CW012407-PA conserved hypothetical protein|protein_<br>coding|DS907449:516555:547561:-1|gene:ISCW012407 ,>EEC16531.1 conserved hypothetical protein<br>[Ixodes scapularis]<br>MGHPPYVIAKSSGRFIGVAAVNGTSELRLTGFAIELLDILARALGFDYNVRVSGEDYGSLSPNGT<br>WTGLVRQVYEREADLGVGDISITRERLEYVDFTAPFMQNGIGILYRNVDRQFEEIPHFLRPWTS<br>ELWLYVLTAVLATSLLFSFLSRLSSAEWVCKRRAGPSCRKLAGKEHLKNRFTLINSFWFVLASL<br>LQQGTDMFPRYELQLLLFFVELAKLT
>Isc015703<br>MNSSVLPHLIVAFPDSPPFMTPKYRNGKLYLDGIFGHILQAVAQFVPFSYELVYDRKRTYGVRLP<br>NGTFNGIMGLLQSGEADLAAAPLIVRQDRAEAINFTPVIYTTYLSLLAVAGEPTANAFSYILAFD

WQVWAILFVSIPILSALLAAIETRRARNKHKYLREFHDNFWDLLRTISYEGLDAAIRTTPGRLM<br>MATWWLTVLVLTNAFAGHMKACMSIKTEPPRFSSIDDVVLHPKIRPMIWKDTGYEFYIRTSEKE<br>SLRMLSRMAQKHGGFVPLSEMQSPAAVAQVYSGRAVYVNDRDSMLYLLSKQCGRTGSLYVAP<br>ELIFARFCAMAYSRKLAHHISRRIDLGVQAVIDSGLATKWYADGLVEWERCRMMQEENAGGE<br>MSYKPLLFQDLSAVFILWGLSVSFALMAFVLESLAFWMERR
>Isc016542<br>LLKLQQIFHMTSNKKLDVRVVRRFMNVSDANAFLHEMELDDRESRKYVVLDSSAEVAKEIIIN<br>HVRDLYTGRRNFHFLLTSLYFNNQILELGVINVTGFRMLQSNRDEFKIFHKSWEKLDHSKWPG<br>AGTAYVSADVLMHDATKVILDAYSRLLKNKSDIFRNNFRRGEVYNRGTKGIDCQSWPAVTWE<br>HGKVIADYMKQTQIKGLTGNISFDSQGYRVNFSIDVVEMTINSEMVKIADWTDTRGLSSHPPK<br>YKRVKQDNEFENKTYIVTSILEEPYLMFKKPEPGQVLVGNDQFEGCKDLADLVAEHIRITYVLK<br>LVNDSKYGGRDVNSPAGWNGMVGELIRQEADMAIAPLTITSARERVIDFTKPFMSLGISIMIKK<br>PMKMKPGVFSFMNPLSREIWMCIIFAYVGVSVVLFLVSRFSPHEWRYEETFVGPSVSNDFSLYN<br>SLWFSLGAFMQQGCDICPRSVAGRIVGSVWWFFTLIIISSYTANLAAFLTVERMVTPINSADDLA<br>KQTEVEYGTLSFSSTQEFFRRSKIAVYARMWEFMNTRKHVFTSTYEEGIRRVRESKGKYAFLM<br>ESTKNDYINERHPCDTMKVGRNLDAKGYGVATPLGSNLRDRLNLAVLSMKENGDLARLENK<br>WWYDRSECRSGDSKESTQNELTLSNVAGCFYILIGGLVLAMVVALLEFCYKSRMEAARSKMT<br>YEAMKAKVRMSITGTQNGDRGRVRQGSL
>Isc017534<br>MGVEMNGGSSGLMVSFSARMIGLVVCFVALSWHGCQAVRVGVLLRRGHNDVWREYEDAMT<br>NFNNSDVASRLGIDIKTHRATFDDNLFITAQELCKQLNAGVTTVLLPSRGFSDAALYSLFGSTNV<br>PRITTTVQQRCGSKDPPVLQEDVWNETTTWTPSSTSTLPPWFSDGSTTERTPDDEEDPTTLGVT<br>MMPDLAPAVIDLADVWGFKSLVFVYDSDHALVTLQQFMDSGRVRVQQARRVSRSAEAHSMLS<br>SLEKGDQQGRKLVVLDCAYDLAKDIVIRHVRDVYMGRRNYHYVLVKPIVSERYLEGVSEFAA<br>LNITAFRFQNTEGITQAYNYNYRTTLIINAYRTLKEDPPPKNADLFEDRVSSGYSGGTSCGRVAY<br>LTKDQGQVVDAYLKNVVFDGKTGRVAFDSEGCRVNFTVDVMQVNGKNQWIQTGTWSKSSGF<br>VRSGERNDSVPPGHKDYVYRVTTILEDPYLMVKNKKYETDGTNQTYEGFCKDLIDAISRLTGI<br>KYQLHLVKDDHYGSVSIDGWNGMIGEIVSNDADIALAGLTITSARKNVVDFTHPFLTSGIAALI<br>KKPSKLQKGVGVNTFLAPFELHLWIGLGASLGAVLLFLFIFGAAVMKNDHCCYQAGDEDVSK<br>DAVLKTVCESLEMVAPLPTGSFMARSISGRIVSSFWWMFVVLVFSTYTTTLTPLLTLRDAEYSSI<br>STLEELSMQNKVKFGTLRTGGVQEYFEESQYRTNKLIWEAMKTERHVLVGTRQEGVGRVRSS<br>DGKYVFFTESLFADYVNGRRPCDTKVIGEVFATQYFGLAVTPGSPLREKLNEAIVNMTESGEID<br>EMKAKWWASECDAPPPDEPMRLELFLHIIFYLACLLIVGIAVALLELLVRGCNKWRFSRLRAVS<br>ARIDRERALRRSRQGDVTGVFPSCPAGVSDR
>Isc017535<br>LGVILKPDLNIVQSVYEGVMYNYSNSNVAQEIKFKVKTSFATLEDTDLFSTSRTLCEQLRGNVT<br>TVVLSSTLHSSLALQSLFKNTNVPYVATSYQEHCSVNVGGNLNLPSADSLGVSLLPDYLPAVAE<br>VVDHLAWDTFVYVYDSDNGPSKLQRLLSHQFKNSVSMRYAKRISNSSDANDFLRLLETTDRES<br>RKYVLLDCRFETAKRIIIDHVRDIYMGRRNYHFLLVNPVVNELTYEKVPEFVAVNITGLRLVGE<br>DLSQQSSLDPSQEPREKKITEEDERRAGPQPRPRLEAEAELLEEGDQLLQADSPSTTTCGDRPSV<br>PQELGEIITRNLREVRPKDTEVDVCSCNFVLLVPSIVLQTLLCFYVLLQRSFQGLTGSIRFTSDGC<br>RIDYNVHVVQLNVNNEAIKIAEWSDTKGFEPVIKPARVEVNDTGVLDKDKTYVIQSVLKPSSW<br>QRVPDAQDRMGNDRYDGYCKDLIDALARELDIKYELRAAEETVYGRRDHKVRGGWTGLIGE<br>VLRKASQNAQSPSTATVINAERKEAVDFSQPFMTTGIAALMLKPSDLPGRGMFTFLAPFSLELW

IFFVSSFGLVFVIMFFVSFFTTRVSSTKPGMEHSACGTIYKSLCYSLEAFTPHYIDSYYARPYEVL<br>RSTGSYRRRYPIFPTLSRSISGRVIGNIWWLFIVFVFSAYTASMVPFLSKESRIRPIRSVEDLPLQS<br>QVDYGFSRQSMAKKYFENPNLNSTAHRRMWEVMNSKPDVFKNSNAEGVDAVRSSKGNYVFF<br>MEANAVFVNTQRPCDTMQLGGTFGVRSFAVAVPKGSSLRKHLDEAIAHLSETGELDKLKKKW<br>WTQKSYCQYPERKKDETVMPLDNFVFFILGGGVALGILVGLIEFI
>Isc022877<br>MAAKYRNTTLRLSNVGWYPYVFTVNQPDGTFRLKGITGSMIDYILASMGIKYQLVDPSDHKW<br>GNLEPDGSWSGMIGQINRNEADLALGPFVLTEREAMVANPSPPYTDEDMIILSGVAKEHKSNV<br>YGYISAFDLHVWMVLLCFLGFVATVAVVAEYLLSNPEMSIRQRRQRLTRIFWTFVASVFLESSTF<br>NFTSHSQRLLFGFWFLMIVVLMNTFVSYMESALMLKEETDRVETIEQLALHPDIQPMVFEAAG<br>FLQVIREAEGQAFQKVLRMMEEYDHEEEVHGEELFTPPYLSMVLKRKAVFLMERISLLARVSQ<br>HCYDVELKGQGLFHLGRKRILQIKMVFYVRKTMDPVLQASATPPQEEIFKRVRWIMDSGLNLH<br>WHEDLIPDPGPCWTKFHPDHLHDYASLNYQDFAVIFYLLLCSHGLAFCGYLSELVLFHWCCLK<br>SRSRIGPNSYLN
>Isc023268<br>LYQVVFQAAIDIVNEDSSVNLEPDILPVVDILPEDDSYEAVRDACRLLEQGVAAIFGPMSGVSSV<br>HVSSVCDSLNVPHLETRWDPFMEDSDDSINIFPHPNVLAEAYTALMKYWEWSALAILYDEDDA<br>IVQLKDLLQELREDGIEVSYFFRNSSDSFRDVLKKIKEAEYVNIFVAIEDEDLEVFLKQAMQIEM<br>LGEEYNYLFMSLDFHVADLSDFIYAKANITALRIVDVEDPVADIFQKKIDENLLRTLSLATLRLTI<br>VKHFVAKKKYLELRQVIGTKSRHSMSSEVLATPAITYGIYNAHFPQVAFDGLTGTVKFDEHGFR<br>KDFQLYVTELGTDGLEEIGTWSPTSGLNITKDEEEKGDDVVLRVVVISVSFVLHKPFVIVNNDE<br>DGSPKFSGFCVDLLQELSAMLDFKYELHASYDGRYGSLQEDGQWNGMVGELTNGNADLAM<br>ADLTITEQRERVVDFTLPFMSTGLAIVTKKGEPMRPGGIWSFFLPLTREVWVYVILATTCTIFVM<br>YIGARLSFREWTLVEDGKGEECMENRFNLFNCFLFVVTTLLHQRITLDPRAPATRVLAGFWYFF<br>TFIILAIIVSNLCESILWEDDGPDYDTAQELLRARGFTYVVVHHGSSQKFLQDSKVPLHQRISSL<br>VERNYRNLPTSVGDGLQRVKDEDKVAFVMETASAAYATQEDCNFSRTDPFVFHSAYGFATPRD<br>SPYRSKLSEGILKLQENGRLQWIQQRWWNPKR
>Isc023274<br>CNLISSGVAAIFGPNDPMIGAHIQSLSDSLDIPHIESRLDLEPDAKDCSVNLHPDPHITGKSMRDL<br>VDYLNWTRIAVLYQDDISLISLQELVRPFPPKNVQYLFRRAENFTATLMDIKSRGIYSMIIDVRPE<br>NLTAFLRAILQIQMNDYKYHYHFATYDIEAYNLEDFQYNFVNITAYRMVDSDNQTVRHIMKDM<br>EKFQPFGQNILNRTNVIITEPALMFDAVYAFAHGLNDLQRSTTLRPANVSCEDEVSWTEGTSLFN<br>LISMVDFYGLTGNIRFNEGNRTSLKLDLLKLRQNFLEKVGEWSTNDGLNITNHNAFHDFGTSNI<br>TLRVTTIESRPYVMMKDNKNLTGNDRFDGFCIDLLRTIADLLGFNYELYLVPDKKFGVENTSTG<br>EWNGMVREIIDKNADLAVAPMTINYARESVIDFTKPFMNLGIGILFMLPSGMPVKLFSFMSPLD<br>VDIWLYVLAAYILVSFTMFIVARSDGAKTSRTKKSRRYTEPQTRVLLYNLNIFFKLSCQKQLQSC<br>DLNPKAMSTRIVGTIWWFFTLILISSYTANLAAFLTVERMITPIESVEDLAEQSKIAYGTLDSGST<br>MTFFRDSKIETYQKMWRYMENRPTVFVSTYEDGARVLQGNYAFLMESTMLDFMVQRDCNLT<br>QVGGLLDSKGYGIATPMGSPWRDKISLAILDLQEKGVIQMLYNKWWKSPGLTCNRDDKNKEG<br>KANALGLANIGGVFVVLLCGLAVAIVTAVMEFCINSRKHAQTDRQSLCSEMAEELRFAVRCRA<br>SRQRPALRRQCSKCIPGTTYVPSAMDLPQENGIMQMLE

Table S3. Protein sequences of OBP used to construct phylogenetic tree.

Accession number	organism	Abbriviated name
XP_022672530.1	*Varroa destructor*	Vd1
XP_022666940.1	*Varroa destructor*	Vd2
XP_022645714.1	*Varroa destructor*	Vd3
XP_022653293.1	*Varroa destructor*	Vd4
XP_022653281.1	*Varroa destructor*	Vd5
JZ172282.1	*Amblyomma americanum*	Aam
OQR79252.1	*Tropilaelaps mercedesae*	Tp1
OQR73552.1	*Tropilaelaps mercedesae*	Tp2
OQR80083.1	*Tropilaelaps mercedesae*	Tp3
OQR74725.1	*Tropilaelaps mercedesae*	Tp4
EEC12103.1	*Ixodes scapularis*	Ix1
XP_002399632.1	*Ixodes scapularis*	Ix2
XP_002433530.1	*Ixodes scapularis*	Ix3
KFM70022.1	*Stegodyphus mimosarum*	Sm1
KFM79180.1	*Stegodyphus mimosarum*	Sm2
XP_025016893.1	*Tetranychus urticae*	Tu1
XP_015782945.1	*Tetranychus urticae*	Tu2
XP_015791022.1	*Tetranychus urticae*	Tu3
XP_015794342.1	*Tetranychus urticae*	Tu4
KPM10930.1	*Sarcoptes scabiei*	Sc1
KPM10909.1	*Sarcoptes scabiei*	Sc2
OXU16759.1	*Trichomalopsis sarcophagae*	Ts1
OXU23289.1	*Trichomalopsis sarcophagae*	Ts2
EAT43854.1	*Aedes aegypti*	Aa1
EAT36416.1	*Aedes aegypti*	Aa2
XP_019538846.2	*Aedes albopictus*	Aal
KPI92605.1	*Papilio xuthus*	Px
OXA59970.1	*Folsomia candida*	Fc

XP_002039957.1	*Drosophila sechellia*	Ds
XP_017053738.1	*Drosophila ficusphila*	Df
OTF72123.1	*Euroglyphus maynei*	Em
ODM87445.1	*Orchesella cincta*	Oc
XP_002066115.1	*Drosophila willistoni*	Dw

>DgalOBP1 (GAIF01000051.1 lcl|ORF1)<br><br>MKAIVLVALAAVIYVVSGDTATGDHNEDTSKFGKWRSCIAEKLPADKKPVFDGCATKPGGTD<br>QSKFRRGLGCVLESYGLTKDQEVDLKKMGSTAASVSSPELKKAFQECPKDEKNSAEDRTIKC<br>VIDHLETQCKVEQ
>DgalOBP2 (GAIF01009917.1 lcl|ORF5)<br><br>MKAIVLVALAAVIYVVSADTATGDHNEDTSKFGKWRSCIAEKLPADKKPVFDGCATKPGGTD<br>QSKFRRGLGCVLESYGLTKDHKVDLKKMGSTAASVSSPELKKAFQECPKDEKNSAEDRTIKC<br>VIDHLETQCKVEQ
>DgalOBP3 (GAIF01034646.1 lcl|ORF7)<br><br>MKTLIFGFVVVFAYLAAGDTATGDHQPDTNQFGRWRSCLTSKLPADKQATMQGCVSRPGGT<br>DMSKFRRGLECVLDSYGVVKDHRVNLQKMSRTAPTVRSAELKKAFQECPRDDKNVSLDRSIK<br>CVIDHLETSCPLDKNRG
>DgalOBP4 (GAIF01001373.1 lcl|ORF7)<br><br>MKTLIFGFVVVFAYLAAGDTATGDHQPDTNQFGRWRSCLTSKLPADKQATMQGCVSRPGGT<br>DMSKFRRGLECVLDSYGVVKDHRVNLQKMSRTAPTVRSAELKKAFQECPRDDKNVSLDRSIK<br>CVIDHLETSCPLEKNRG
>DgalOBP5 (GAIF01024919.1lcl|ORF6)<br><br>MKTLIFGFVVVFAYLAAGDTATGDHQPDTNQFGRWRSCLTSKLPADKQATMQGCVSRPGGT<br>DMSKFRRGLECVLDSYGVVKDHRVNLQKMSRTAPTVRSAELKKAFQECPRDDKNVSLDRSIK<br>CVIDHGDVLSARKEPRLSLLNPTRRQWAGKTAKRGRKKQEEARSPHRGTWH
>Vd1<br>MKVFVIIALVGMAKAAPQAPASATPAKVPVIEWGKCEQLKPSESERTSKAAVVDKCLQSLPLP<br>DPEKATQQEIDKHRESVTTCALKAEGWFDDEGVYKFDRARNEIKNKKLDSEVEEAVLLKHDA<br>CQKEATEKHDDYINQVQLYQACMDYNISQICGIKVMV
>Vd2

MHRSFVLQCCGLLFIVGLALAGPLAKDKSERITPKQTVEEFFTSNWGECEPLKNPLVLPEISDA<br>ATWERCRVHIPLSPDQVEEALPNLSRCMLKVIGWVRANGNMNLTKYMDYLSQAGLDDKTLE<br>LTKTAHEKCSQRALSDGSGDYKEQDKIYVECIFRHFDKTCPPNFQAAVKQGYIVGITQISFVPR<br>YDEVEVESDTDAETEGQAGPSVADATGEAAANDNANGSGGPQ
>Vd3<br>MKTFIGLVLVGMAMAAPPASGGPEAGKPPTIEWGKCSQLKPSDSERMTKAAVVEKCVQSLPLP<br>EAGKASQAEIEKHREDVTTCALKAEGWFDDKGVYKFDRARDEIKNKKLASDIETPVLEKHAE<br>CQKEAGEKHTSDYIKQVQLYQACMDFNISQICGIKVVS
>Vd4<br>MNAAVYIVTAMLACCCSADTASGDHASDTSQFGKWRTCLADKLPAKKREVFDECFIKPGGTD<br>MNKFRRGLNCVLTSDGLLKNHKVDLKKMSMTAASAGSPELKKAYENCPKDDKNTSEDRSIKC<br>LIDHLEVDCKLASK
>Vd5<br>MYENRLLHLNGICGGGIQADRCEPAYLPDTVVRIIVKMKTLVVALLASTACLTAADTATGDHQP<br>DSNQFGRWRTCLKSKIPENKQPGMEACINKPGGTDMSKFRRGLGCVLESYGIVNNHRVDLTK<br>MRSSASLVTSPELKKAFMECPKDDKNSSLDRSIKCVIDHLETSCSAVKAQG
>Aam JZ172282.1 Amblyomma americanum cDNA, mRNA<br>sequenceMVAFKAALLLCVLVLPAAYCQSAAEPPRPDINWGKCPQLQPSKEERQQKALVIDTCL<br>EKVPLPDVEHANETVIQQHREDVTTCALHSEGWFNKNGQYRFDRARTEILNKKLAADVEPKV<br>LAKHDECKKEAEEKFAHQFVAQVQLYQACMDYHISQICGIQIQGAQGGAAAPAHG
>Tp1<br>MKTLVIALIAGTACLAAADIATGDHQPDTNQFGQWRTCLKSKIPQDKQATMDNCVKKPGGTD<br>MAKFRRGISCVLESYGVVNGHRVDLRKMGSTASSVRSPELKKAFQECPKDDKNASLDRSIKCV<br>IDHLETSCPLVKAQG
>Tp2<br>MKVFVVMALMGMAMAAPQAPAAPTSAKAPVIEWGKCDQLKPSENERTSKAAVVDKCLQSLP<br>LPDPEKATQQEIDKHRESVTTCALKAEGWFDDKGVYKFDRARNEIKNKKLDGEIEAAVLVKH<br>DACQKEATEKHEDYINQVQLYQACMDYNISQICGIKVMV
>Tp3<br>VEQFFTSNWGECEPLKNPLVLPEISDGSTWERCRAHIPLTPEQVEEALPNLSRCMLKVIGWVRP<br>NGNMNLTKYMDYLSQAGLDEKTLELTKIAHEKCSQRALSDGAGNYKEQDKIYVECIFRHFDK<br>TCPPNFQAAVKQGYIVGITQISFVPRYDDVEMEVDSDADAEDPNGAYPGPGADDANASSGGSS<br>NNNNKDDSNNNNKADSNGDAQ
>Tp4<br>MSKVLTLYTNGFNVHKLGALLVSMAYSFSPTEDRPVIEQCLGQHDNETVMELIKCYMEKMNI<br>MGGGNMCEVKKLKDLLASKMTAAPPVATAVLQKYAECICQNSGAEGMVCVQQKTMDYAKTF<br>CSAIVGDD
>Ix1<br>MWTSAGKSALVTLGVLLAVGVGLGDAGPVDPKKTDKPEKQTIHEFFSSNWGACEPLKNPLVIP<br>EMSNPTTWVKCQKHLPEHPQDIEEYLPRLSRCMLKSTGWVRSNGNMNLSKYLQYLSLLGLDD<br>ETMNGTREAHKGCIAKAYKEKDYKTQDKVYVECIFKFFTKSCSANFQGAVKEGYIVGITQLSF<br>MPRMGPLEVVDDVEAEVSEDDAPQDVLPVTNSTSTS
>Ix2MVAFKVSVAVLLCVGSAFAIPAGGKDLDAAAQTQAGVPPRPQINWGKCPQLQPSENERQQ

KALVIDQCLDKVPLPDVDTANDTVIQQHRQDVTTCALKTEGWFNSNGQYRFDRARTEILNKK<br>LAKDVEPQVLSKHDECKKEAEEKFAKNFVAQVQLYQACMDYHISQICGIQIAQAAEAHPQGA<br>QQG
>Ix3<br>MKILLITVVVCVFAATDASRRSTRRQQFTDWRNCMVEQLPSEKKPIYDACYNRSRGTDMHKF<br>REGLKCVLTDYNLLNGTKIDLAAMTTRASSVSGKLGQAFTDCPKDDHNRNAAKGVKCIIDRL<br>ETDCPVPTGSTAH
>Sm1<br>MLLSVLLTLFGVGVILAAPPTTVQPQNEASLESQNYPVPQIDWGKCPQLKPTEADKEQKRLVIQ<br>KCLDQNPPPALETVTDPKQIDDHRELVTTCALKTEGWFNEDGSYKFNRAKTEIESKKLDSQVE<br>KNISKRHQECQDEAVKRFPGGYIQQVQLYQACMDYHIAEICEIELIPPPGGFPIEEFGDYDESPL<br>QPEQPEKSQETTTQPQAQPASAK
>Sm2<br>MKCFVLAVLCFAAVAIAQEVPQVNWGKCPELEPTAEEQKSKGEVIQQCLEKNPPPAEGASLTQE<br>QIDAHRETITTCALKTEGWFGADNKYNYQRARKEIQSKNLKPEAEILAKHDECSKEAKENFPD<br>NNIPQVQLYQACMDFHITKICEIKITPPATEAPAAASA
>Tu2<br>MINSLSHSVSLCLIVLVVNSSNCAPQATPNRQGGFSGQLQGPPQPPQINWGKCPQLEPVESDKK<br>AKADIISSCLKSIALPENMTQETVEKHRTEVARCALNAENWFTEDGKYRYDKAESEIKAKKLS<br>QEVEPQILRQHGECRKEAEGQFPSGIAQIQLYQACMDYHISQICGITIVGPSSPQ
>Tu3<br>MILITLCLVVLAINSSYGQPQRFLAPQPTSDQLEGSPQGGFPGQPQGPPQPPQINWGKCPQLEPK<br>ESDKKAKADIISSCIKSIPLPENVTQESIEKHRTEVAICALNAENWFSADMKYRYDKAESEIRAK<br>KLVSDVEPLVLRQHGECRKEAEGQFPSGIAQVQLYQACMDYHISQICGITIVGASSPQ
>Tu4<br>MNYLSLCVFIIFFLTIGFSESARLPRSTSSPATSSSTTTTSSTVNNKKNEENQPSTSSASSSAGSATG<br>GPTINWGRCPQLEPTNEEKIKKAAVITKCLESTPVPVNITRETVELHREQVAACALRMEGWFNG<br>QGLYRFDKAENEIKAKKLTREVEEKVLSFHRQCKDEAEEKYPVSSNQLIAQIQLYQACMDYFIS<br>EVCGIEVQIAGSEGSSE
>Sc1<br>MMAIHQLFLTTVASLWSVMLMTAVVALPQPLNTSTNNKPADSISTASSSSIDSNVPLMASASSPS<br>GQNLRSSSQQPMLLTLEQQQELLDQLAQLQRQTQQLLAAQQSLAAVAAAAAVNGNNNGVSIG<br>TPLDGNSNNDAGSHSPITSGSIQYHRQQNQQQTHPTTQQLNNPTSNQQSAAIRNDNIDQGSLEQ<br>SQSKSNQLNQMINNAQNSQSQSMSGLPQSPQINWGKCPELEPSEQEKLAKANVITKCLETTPLP<br>ANITRESVEQHREQIAGCALRSEGWFTPNGGYDFSKAEKEIRNKHLQADLENQVLNYHGQCR<br>VESEEKYPTSNSSIIAQIQLYQACMDYFISDVCGIEVNDTDSPQF
>Tu1<br>MKNLQSITILCLIFGFIVSTVYSQPLGSSDSNPDTQDTKEASTSVNDKSSDQEGSSESDEDAPTPV<br>AGPHRPPTINWGKCPQLEPKEAEKKQKADIIRTCLKSITLPENITQESVEKHRTEVAKCALNAEN<br>WFAEDGSYRYDKAESEIKNKKLQPEIEPQIVNQHSECRKEAEEQFPSGIAQIQLYQACMDYHIS<br>MICGIQIVGTGYSQ
>Sc2<br>MIANMNLYALAIILLLAHLCDAQSTEKLDKSKLKQQQQLPQRPKIDWGKCPQLEPSEKDIKSKS<br>NILQRCLKENPPPNQDSITPELIIGHQKNVTECALKIENWFDKKGDYKFEKAEKEIKNKKLSSE

MQKALVGSHQDCAEVSKTQKKQSKTPMDVVEQVQIYQGCMDAHITQHCQIKIGA
>Ts1<br>MSSNLKMINMIGSWILNVTSSNKSRYWKNKYEACFDYKEGSVIICGQYLISMSRHLIITLTLFSA<br>VFVVKSLSPEEREARDKCLKENGFSREPDFIGNDAVDMRTKCYAACALRGYGIMNEDGSIDIN<br>KILEHISDTKNKDIDVKKSLIIPCAEKKGETDCDTGYLITNCVVLASLSQEDIDARNKCLKEHGF<br>TIEPKYVSAYKTIDTRAKCYASCLMRETGVIKEDGSIDLNKVLEKISDPENKTLDEDVKKSFIPC<br>TEKKGDNDCDTVHQILTCIVATISILKESMKIV
>Ts2<br>MLANTARLNAFAMCMLQQFNVMDSNGMVNPDVMSYSIISTVPNATAGISQQCISKRGIDAVNT<br>ARMIMNCYLRANQMVLALSRRDRTYIIRINYITVACPVLAYLQASFSPLIENDFHAYEADCGAK<br>SGASDESIEAARRARQLPQSPQMNAFALCMMQKYKVMAADGSVNPDVRSYGIITDGPDNTWR<br>VSEHCRDLDGNSSGETARMIMNCYLDNNQLVMGLTPRVSA
>Aa1<br>MQVLNFLCLVLLCLVLEKVAVAEECIKFEDHKDEILNCCKYQPPYPKDDVKECVQEAQGKSGG<br>DKHEFFACLLECYLPKIGIINGDSIDEDKISEHLQSLDENARDILLAAYKECDESTTGTTRAQCSS<br>YALDLETCVLQKLDQQCPDEFYNPSEICDKLKSGVEICH
>Aa2<br>MKATVTSVLVLLAISHATLADPAAPDNVPASCLNKNFNVDPFECCKTPKLLDEGTVKECVHSFP<br>PPQNAQDEIKPDCMSECVMNSTRIFDRRQNVNDAKAMETFLEKLNGKSVWAEIVQKAVKQCL<br>DDADNRKEEFSRDMKALQQKFPKERICSPAAGFIMECVHVSVYKNCPASIFKDNLAGCPAIKK<br>HLNVDNCPFYTIFPEKKAPKPVKRH
>Aal<br>MKAIVASVLVLLALSHTTRADASAPDDVPATCLNKNFNVDPFECCKTPKLLDETVVKQCVKSY<br>PPPQDVKDEIKPDCMSECVMNSTQIFDRRQNVNDAKAMETFLEKLNGKSVWADIVQKAVMQ<br>CLDDADNRKEEFNRDMKALQQRFPKERICSPAAGFIMECVHVVVYKNCPASVFKDSLTGCPAI<br>KKHLNVDNCPFYTIFPEKKGNKAKKH
>Px<br>MSKLYCVFLFLGLAVSLRHVRALSQEDIAAIKTGLRPLIAECGKEFGVDEADIKKAKESGKIESL<br>DPCLFACIGKKMGMINDKGEFDVEKSSETVKKFVTDKDEQKQILEIIEKCSSVNDEAVSDDKGC<br>DRAVLLHKCMEPYKDQGNSLGDLQKSYADLVIECAKEFPITTEDIAQLQNKQLPDKESVKCLF<br>ACAYKKAGMMDDEGKMSVEGALEVINKYLADDPDTMKKAIDFTNACSTVNDAEVSDGTKG<br>CDRAALMFRCSIDKADEFDINL
>Fc<br>MQPTLHFVLLCTIGVLHFQDGVFAAEEKPCNAVVADLTKKQLGGISACTKALKFKDAKEKAK<br>KTTCIMKCVLKKEALLDEKGQLTLEQFDKFLAKHFPDSMTEKANSTFRPCVDGALKGMDDTE<br>EFCKDYDPMIKCLLSTFPNESDKSCKQIMADMTKRQLSGITKCIKGMKFKNGKEKAAKMNCI<br>MKCAMENEGLVSKTGDISREQIDEILIREFPSSLLERANATFQPCVEKYAGTMDANPETDPLCAT<br>YDPVIKCLISALPNLCPPA
>Df<br>MLRTGILIFVVLSQFLEGPLAIRIHCRHTERIHEENIHHCCKHPDGHNDVTEMCAKQTNFKLPNP<br>NEEAIEDVTVDQAMVGTCWAKCVFDHYNLMENNTLDMAKVRHYYKRYHKIDPEYETEMLN<br>AYESCHSKSEDATEQFLSLPIVRAFATSKFCKPTSSIIMSCVIYHFFHNCPKNRWSNTTECVETLA<br>FAKKCKDVLTTM
>Em

QLPRAPKINWGRCPQLEPTQRDIEAKSSILQECLRQNPPPQSESITQEQIIQREYKYEKAEKEIKN<br>KNLAKEMEKVLLESHKQCSQIAKDRKQAVKNLSNVEQVQIYQGCMDAHITQNCQIQISAV
>Oc<br>MMKLCLLVTACILAVISVQAQDLKSCRAVFSEQNRKQLTWINGCVKQLGYKSAREKTQKSTCV<br>WKCVLDNFNMLNADGNMDVNQYNHHLQEEWPAHMLDRANSTFQPCLKIGSETTQDEFCKG<br>YDPLIKCMTRNFANLCKGIK
>Dw<br>MSASHLLILTCCLVLAQNVRGQTQINCQRPPQLVDPSLCCRDGGRDQITEQCAQKIVGSAANGP<br>PSIETATCLAECILTTSNYITEPQKINATNIRNNLSAKFSDKEYVEAMSMAFTKCEPQAQQRLAVI<br>QQQQRQQPKCSPFSAILLGCTYMEYFKNCPANRWTQSTECALAKTFVTQCGLGA

Table S4. Protein sequences of OBP used to construct motif analysis

Accession number	E-value	organism	Abbriviated name
XP_022672530.1	6e-116	Varroa destructor	Vd1
XP_022666940.1	3e-173	Varroa destructor	Vd2
XP_022645714.1	5e-117	Varroa destructor	Vd3
XP_022653293.1	7e-97	Varroa destructor	Vd4
XP_022653281.1	2e-129	Varroa destructor	Vd5
JZ172282.1		Amblyomma americanum	Aam
OQR79252.1	2e-99	Tropilaelaps mercedesae	Tp1
OQR73552.1	8e-117	Tropilaelaps mercedesae	Tp2
OQR80083.1	3e-156	Tropilaelaps mercedesae	Tp3
EEC12103.1	3e-167	Ixodes scapularis	Ix1
XP_002399632.1	1e-135	Ixodes scapularis	Ix2
XP_002433530.1	2e-98	Ixodes scapularis	Ix3
KFM70022.1	2e-156	Stegodyphus mimosarum	Sm1
KFM79180.1	2e-117	Stegodyphus mimosarum	Sm2
XP_025016893.1	2e-131	Tetranychus urticae	Tu1
XP_015782945.1	2e-125	Tetranychus urticae	Tu2
XP_015791022.1	1e-135	Tetranychus urticae	Tu3
XP_015794342.1	2e-155	Tetranychus urticae	Tu4
KPM10930.1	0.0	Sarcoptes scabiei	Sc1
KPM10909.1	9e-130	Sarcoptes scabiei	Sc2
OXU23289.1	4e-169	Trichomalopsis sarcophagae	Ts2
XP_017053738.1	1e-151	Drosophila ficusphila	Df
OTF72123.1	5e-85	Euroglyphus maynei	Em
XP_002066115.1	2e-132	Drosophila willistoni	Dw

>DgalOBP1 (GAIF01000051.1 lcl|ORF1)

MKAIVLVALAAVIYVVSGDTATGDHNEDTSKFGKWRSCIAEKLPADKKPVFDGCATKPGGTDQSKFRRGLGCVLESYGLTKDQEVDLKKMGSTAASVSSPELKKAFQECPKDEKNSAEDRTIKCVIDHLETQCKVEQ
>DgalOBP2 (GAIF01009917.1 lcl|ORF5)<br>MKAIVLVALAAVIYVVSADTATGDHNEDTSKFGKWRSCIAEKLPADKKPVFDGCATKPGGTDQSKFRRGLGCVLESYGLTKDHKVDLKKMGSTAASVSSPELKKAFQECPKDEKNSAEDRTIKCVIDHLETQCKVEQ
>DgalOBP3 (GAIF01034646.1 lcl|ORF7)<br>MKTLIFGFVVVFAYLAAGDTATGDHQPDTNQFGRWRSCLTSKLPADKQATMQGCVSRPGGTDMSKFRRGLECVLDSYGVVKDHRVNLQKMSRTAPTVRSAELKKAFQECPRDDKNVSLDRSIKCVIDHLETSCPLDKNRG
>DgalOBP4 (GAIF01001373.1lcl|ORF7)<br>MKTLIFGFVVVFAYLAAGDTATGDHQPDTNQFGRWRSCLTSKLPADKQATMQGCVSRPGGTDMSKFRRGLECVLDSYGVVKDHRVNLQKMSRTAPTVRSAELKKAFQECPRDDKNVSLDRSIKCVIDHLETSCPLEKNRG
>DgalOBP5 (GAIF01024919.1lcl|ORF6)<br>MKTLIFGFVVVFAYLAAGDTATGDHQPDTNQFGRWRSCLTSKLPADKQATMQGCVSRPGGTDMSKFRRGLECVLDSYGVVKDHRVNLQKMSRTAPTVRSAELKKAFQECPRDDKNVSLDRSIKCVIDHGDVLSARKEPRLSLLNPTRRQWAGKTAKRGRKKQEEARSPHRGTWH
>Vd1<br>MKVFVIIALVGMAKAAPQAPASATPAKVPVIEWGKCEQLKPSESERTSKAAVVDKCLQSLPLPDPEKATQQEIDKHRESVTTCALKAEGWFDDEGVYKFDRARNEIKNKKLDSEVEEAVLLKHDACQKEATEKHDDYINQVQLYQACMDYNISQICGIKVMV
>Vd2<br>MHRSFVLQCCGLLFIVGLALAGPLAKDKSERITPKQTVEEFFTSNWGECEPLKNPLVLPEISDAATWERCRVHIPLSPDQVEEALPNLSRCMLKVIGWVRANGNMNLTKYMDYLSQAGLDDKTLELTKTAHEKCSQRALSDGSGDYKEQDKIYVECIFRHFDKTCPPNFQAAVKQGYIVGITQISFVPRYDEVEVESDTDAETEGQAGPSVADATGEAAANDNANGSGGPQ
>Vd3<br>MKTFIGLVLVGMAMAAPPASGGPEAGKPPTIEWGKCSQLKPSDSERMTKAAVVEKCVQSLPLPEAGKASQAEIEKHREDVTTCALKAEGWFDDKGVYKFDRARDEIKNKKLASDIETPVLEKHAECQKEAGEKHTSDYIKQVQLYQACMDFNISQICGIKVVS
>Vd4<br>MNAAVYIVTAMLACCCSADTASGDHASDTSQFGKWRTCLADKLPAKKREVFDECFIKPGGTDMNKFRRGLNCVLTSDGLLKNHKVDLKKMSMTAASAGSPELKKAYENCPKDDKNTSEDRSIKC

LIDHLEVDCKLASK
>Vd5<br>MKTLVVALLASTACLTAADTATGDHQPDSNQFGRWRTCLKSKIPENKQPGMEACINKPGGTDM<br>SKFRRGLGCVLESYGIVNNHRVDLTKMRSSASLVTSPELKKAFMECPKDDKNSSLDRSIKCVID<br>HLETSCSAVKAQG
>Tp1<br>MKTLVIALIAGTACLAAADIATGDHQPDTNQFGQWRTCLKSKIPQDKQATMDNCVKKPGGTD<br>MAKFRRGISCVLESYGVVNGHRVDLRKMGSTASSVRSPELKKAFQECPKDDKNASLDRSIKCV<br>IDHLETSCPLVKAQG
>Tp2<br>MKVFVVMALMGMAMAAPQAPAAPTSAKAPVIEWGKCDQLKPSENERTSKAAVVDKCLQSLP<br>LPDPEKATQQEIDKHRESVTTCALKAEGWFDDKGVYKFDRARNEIKNKKLDGEIEAAVLVKH<br>DACQKEATEKHEDYINQVQLYQACMDYNISQICGIKVMV
>Tp3<br>VEQFFTSNWGECEPLKNPLVLPEISDGSTWERCRAHIPLTPEQVEEALPNLSRCMLKVIGWVRP<br>NGNMNLTKYMDYLSQAGLDEKTLELTKIAHEKCSQRALSDGAGNYKEQDKIYVECIFRHFDK<br>TCPPNFQAAVKQGYIVGITQISFVPRYDDVEMEVDSDADAEDPNGAYPGPGADDANASSGGSS<br>NNNNKDDSNNNNKADSNGDAQ
>Ix1<br>MWTSAGKSALVTLGVLLAVGVGLGDAGPVDPKKTDKPEKQTIHEFFSSNWGACEPLKNPLVIP<br>EMSNPTTWVKCQKHLPEHPQDIEEYLPRLSRCMLKSTGWVRSNGNMNLSKYLQYLSLLGLDD<br>ETMNGTREAHKGCIAKAYKEKDYKTQDKVYVECIFKFFTKSCSANFQGAVKEGYIVGITQLSF<br>MPRMGPLEVVDDVEAEVSEDDAPQDVLPVTNSTSTS
>Ix2<br>MVAFKVSVAVLLCVGSAFAIPAGGKDLDAAAQTQAGVPPRPQINWGKCPQLQPSENERQQKAL<br>VIDQCLDKVPLPDVDTANDTVIQQHRQDVTTCALKTEGWFNSNGQYRFDRARTEILNKKLAK<br>DVEPQVLSKHDECKKEAEEKFAKNFVAQVQLYQACMDYHISQICGIQIAQAAEAHPQGAQQG
>Ix3<br>MKILLITVVVCVFAATDASRRSTRRQQFTDWRNCMVEQLPSEKKPIYDACYNRSRGTDMHKF<br>REGLKCVLTDYNLLNGTKIDLAAMTTRASSVSGKLGQAFTDCPKDDHNRNAAKGVKCIIDRL<br>ETDCPVPTGSTAH
>Sm1<br>MLLSVLLTLFGVGVILAAPPTTVQPQNEASLESQNYPVPQIDWGKCPQLKPTEADKEQKRLVIQ<br>KCLDQNPPPALETVTDPKQIDDHRELVTTCALKTEGWFNEDGSYKFNRAKTEIESKKLDSQVE<br>KNISKRHQECQDEAVKRFPGGYIQQVQLYQACMDYHIAEICEIELIPPPGGFPIEEFGDYDESPL<br>QPEQPEKSQETTTQPQAQPASAK
>Sm2<br>MKCFVLAVLCFAAVAIAQEVPQVNWGKCPELEPTAEEQKSKGEVIQQCLEKNPPPAEGASLTQE<br>QIDAHRETITTCALKTEGWFGADNKYNYQRARKEIQSKNLKPEAEILAKHDECSKEAKENFPD<br>NNIPQVQLYQACMDFHITKICEIKITPPATEAPAAASA
>Tu1

MKNLQSITILCLIFGFIVSTVYSQPLGSSDSNPDTQDTKEASTSVNDKSSDQEGSSESDEDAPTPV<br>AGPHRPPTINWGKCPQLEPKEAEKKQKADIIRTCLKSITLPENITQESVEKHRTEVAKCALNAEN<br>WFAEDGSYRYDKAESEIKNKKLQPEIEPQIVNQHSECRKEAEEQFPSGIAQIQLYQACMDYHIS<br>MICGIQIVGTGYSQ
>Tu2<br>MINSLSHSVSLCLIVLVVNSSNCAPQATPNRQGGFSGQLQGPPQPPQINWGKCPQLEPVESDKK<br>AKADIISSCLKSIALPENMTQETVEKHRTEVARCALNAENWFTEDGKYRYDKAESEIKAKKLS<br>QEVEPQILRQHGECRKEAEGQFPSGIAQIQLYQACMDYHISQICGITIVGPSSPQ
>Tu3<br>MILITLCLVVLAINSSYGQPQRFLAPQPTSDQLEGSPQGGFPGQPQGPPQPPQINWGKCPQLEPK<br>ESDKKAKADIISSCIKSIPLPENVTQESIEKHRTEVAICALNAENWFSADMKYRYDKAESEIRAK<br>KLVSDVEPLVLRQHGECRKEAEGQFPSGIAQVQLYQACMDYHISQICGITIVGASSPQ
>Tu4<br>MNYLSLCVFIIFFLTIGFSESARLPRSTSSPATSSSTTTTSSTVNNKKNEENQPSTSSASSSAGSATG<br>GPTINWGRCPQLEPTNEEKIKKAAVITKCLESTPVPVNITRETVELHREQVAACALRMEGWFNG<br>QGLYRFDKAENEIKAKKLTREVEEKVLSFHRQCKDEAEEKYPVSSNQLIAQIQLYQACMDYFIS<br>EVCGIEVQIAGSEGSSE
>Sc1<br>MMAIHQLFLTTVASLWSVMLMTAVVALPQPLNTSTNNKPADSISTASSSSIDSNVPLMASASSPS<br>GQNLRSSSQQPMLLTLEQQQELLDQLAQLQRQTQQLLAAQQSLAAVAAAAAVNGNNNGVSIG<br>TPLDGNSNNDAGSHSPITSGSIQYHRQQNQQQTHPTTQQLNNPTSNQQSAAIRNDNIDQGSLEQ<br>SQSKSNQLNQMINNAQNSQSQSMSGLPQSPQINWGKCPELEPSEQEKLAKANVITKCLETTPLP<br>ANITRESVEQHREQIAGCALRSEGWFTPNGGYDFSKAEKEIRNKHLQADLENQVLNYHGQCR<br>VESEEKYPTSNSSIIAQIQLYQACMDYFISDVCGIEVNDTDSPQF
>Sc2<br>MIANMNLYALAIILLLAHLCDAQSTEKLDKSKLKQQQQLPQRPKIDWGKCPQLEPSEKDIKSKS<br>NILQRCLKENPPPNQDSITPELIIGHQKNVTECALKIENWFDKKGDYKFEKAEKEIKNKKLSSE<br>MQKALVGSHQDCAEVSKTQKKQSKTPMDVVEQVQIYQGCMDAHITQHCQIKIGA
>Ts2<br>MLANTARLNAFAMCMLQQFNVMDSNGMVNPDVMSYSIISTVPNATAGISQQCISKRGIDAVNT<br>ARMIMNCYLRANQMVLALSRRDRTYIIRINYITVACPVLAYLQASFSPLIENDFHAYEADCGAK<br>SGASDESIEAARRARQLPQSPQMNAFALCMMQKYKVMAADGSVNPDVRSYGIITDGPDNTWR<br>VSEHCRDLDGNSSGETARMIMNCYLDNNQLVMGLTPRVSA
>Df<br>MLRTGILIFVVLSQFLEGPLAIRIHCRHTERIHEENIHHCCKHPDGHNDVTEMCAKQTNFKLPNP<br>NEEAIEDVTVDQAMVGTCWAKCVFDHYNLMENNTLDMAKVRHYYKRYHKIDPEYETEMLN<br>AYESCHSKSEDATEQFLSLPIVRAFATSKFCKPTSSIIMSCVIYHFFHNCPKNRWSNTTECVETLA<br>FAKKCKDVLTTM
>Em<br>QLPRAPKINWGRCPQLEPTQRDIEAKSSILQECLRQNPPPQSESITQEQIIQREYKYEKAEKEIKN<br>KNLAKEMEKVLLESHKQCSQIAKDRKQAVKNLSNVEQVQIYQGCMDAHITQNCQIQISAV
>Dw<br>MSASHLLILTCCLVLAQNVRGQTQINCQRPPQLVDPSLCCRDGGRDQITEQCAQKIVGSAANGP<br>PSIETATCLAECILTTSNYITEPQKINATNIRNNLSAKFSDKEYVEAMSMAFTKCEPQAQQRLAVI

QQQQRQQPKCSPFSAILLGCTYMEYFKNCPANRWTQSTECALAKTFVTQCGLGA

Table S5. Protein sequences of NPC2 used to construct phylogenetic tree.

Accession number	organism	Abbreviated name
XP_022652195.1	*Varroa destructor*	Vd1
XP_022649311.1		Vd2
XP_022654520.1		Vd3
XP_022654596.1		Vd4
gi_283753684		Vd5
NP_608637.1	*Drosophila melanogaster*	Dm1
NP_650331.1		Dm2
AAN71436.1		Dm3
ADL59625.1		Dm4
NP_001247376.1		Dm5
AEN25572.1		Dm6
OQR69913.1	*Tropilaelaps mercedesae*	Tm1
OQR78448.1		Tm2
OQR69614.1		Tm3
OQR72593.1		Tm4
OQR76180.1		Tm5
OQR69865.1		Tm6
EEC00381.1	*Ixodes scapularis*	Isc1
EEC07080.1		Isc2
EEC07323.1		Isc3
EEC03680.1		Isc4
XP_002435260.1		Isc5
EEC08723.1		Isc6
EEC06676.1		Isc7
EEC13424.1		Isc8
EEC13423.1		Isc9
EEC18444.1		Isc10
EEC16220.1		Isc11

>DgalNPC2-1 GAIF01015586.1 lcl|ORF7<br>MANNFFFLIVASLALTAYAGELVQINNPVQCDGLTTIKAVYVEDCTNGEMCDLVKGHNYSLKIT<br>YTAPKDVQTAKALVTGVIGGAELPFPGLQRYVCKTPGNEDFCPQVKGKDYTVSMVLPIMSLFP<br>KLETTATVRFIDDDAKSEAEEKLGCFQLPIRLVDDPNAPKQPTRVDNEDDDE
>DgalNPC2-2 GAIF01031731.1 lcl|ORF2

MANNFFFLIVASLALTAYAGELVQINNPVQCDGLTTIKAVYVEDCTNGEMCDLVKGHNYSLKIT<br>YTAPKDVQTAKALVTGVIGGAELPFPGLQRYVCKTPGNEDFCPQVKGKDYTVSMVLPIMSLFP<br>KLETTATVRFIDDDAKSEAEEKLGCFQLPIRLVDDPTPLSSRRALTTKTTMSNERQRDDAT
>DgalNPC2-3 GAIF01008081.1 lcl|ORF7<br>MANNFFFLIVASLALTAYAGELVQINNPVQCDGLTTIKAVYVEDCTNGEMCDLVKGHNYSLKIT<br>YTAPKDVQTAKALVTGVIGGAELPFPGLQRYVCKTPGNEDFCPQVKGKDYTVSMVLPIMSLFP<br>KLETTATVRFIDDDAKSEAEEKLGCFQLPIRLVDDPTPLSSRRALTTKTTMSNTSARDDAT
>DgalNPC2-4 GAIF01004272.1 lcl|ORF7<br>MISQRIVVLPLIVAVLAVAVHGSPYKLCKTPSIGTVVKVVIEGCSDDVQSCVLRKGTEAKLSLTFI<br>PNVDVKSLVAKAYGLIAGVPVPYPLKNSDACKDSGLSCPLKKDPKVTYKQAFDVMKEFPSMS<br>LTVKWVLRDEQNAHIVCPDTCQTGVVARREFWTVWTQVVSSRPLRKGCVAVGDKNVAFRPV<br>YFAVKLQ
>DgalNPC2-5 GAIF01014813.1 lcl|ORF6<br>MPRFTATTAAAVVIAAIVLLSASPSVLGQREITFTPCDDPGKGIVKRVTATPCDTDPCELKKGSM<br>TSFNFEFIADQNTTSITLDARVRVLGIFIPVPFIETDMCRVIDCPVVKGQEIVGQFNATVPRVPVKS<br>SVIRVKVIGDEGMSCCISYKVVLK
>DgalNPC2-6 GAIF01031088.1 lcl|ORF3<br>MAPKRGLRRVAAVPAERALATIVVLAAVCVAIVRGSPFKSCGGSAKITAVDIENCPSSAKSCTVH<br>KGAAFKVQVTFKPAKDAVNATLHKAVIINNVTKKLTPCVSVCNAGLKCPLHKNVPATLQYTGH<br>VPKDSPKGKLFGQLSLTDSAGSLILCAQSPIEVA
>Vd1 XP_022652195.1 epididymal secretory protein E1-like [Varroa destructor]<br>MKTFLVIFALAAIAIASPFTSCNHSQGTIRTVEISGCKDTDPYCVLYKGTNVNLSITFVPASDASR<br>VTVEVYGLISGFGIPYPLPNSNACARGLICPLKNGETATYKHSFPVRNGFPSIALDVIWTLFDER<br>KNRIICARFPVVIQ
>Vd2 XP_022649311.1 epididymal secretory protein E1-like [Varroa destructor]<br>MLRFVMLAAIASAAYAGELYKVQNVQQCDGLSTIKSVEVENCTKEDLCELIRGHNHLLRVTYV<br>PPRSAQVVKVLITGFVGEAELPFPGFKRYVCKTPGNENFCPQTAGQEYTTTMVLPILSVFPNIET<br>TAFVRFIDDDAKSEAEEKLGCFSMKIRLIDDPNASTEPANVDNQEDDE
>Vd3 XP_022654520.1 uncharacterized protein LOC111247622 [Varroa destructor]<br><br>MTSVTQSALIILVVVTTWPWASFARKVQYQECNPKRNIKMMDVDNCDVQPCRVFHRTTIKMT<br>TQLSSPINSSSAEWEIRAFVNGRPYGHPEPVSDACLFVNSTSPNAFSSSHRTPLNSDQFKSGPST<br>AYRQKSFLPMCPLFAGKIYRFSNVFYVHDQFPKVATTIQYLLRGDNDEMIACARLHILMSS
>Vd4 XP_022654596.1 epididymal secretory protein E1-like isoform X1 [Varroa destructor]<br>MMLLDLFTVVFAVMLMMNENNAKQLDSYVNCGGTGHIENITVVPCESEPCEFAKGSKVNVH<br>FIGSFSHDANSVRVQPSVIVDKMNVTLTGVDQELCGKHVQCPIKANELRDAEIEIPIFNVYPNIK<br>TEVVFMIFSENVLQFCVRTKIIISDKK
>Vd5 gi_283753684<br>MWLYVWVTVAALATVGNGSIFQSCEKIETKGTILTLDIEGCPNTENVCVLKRGVDTKIALTFTP<br>EVEMNNITVKVFGVIENIPVPYPIPHSNACIKSGLECPLEKGVPATYTQTFPVPKVFPSIFLIIKWT<br>FVDSFNNELICALIPTRLQ

>Dm1<br>MLRYAVIACAALVVFAGALEFSDCGSKTGKFTRVAIEGCDTTKAECILKRNTTVSFSIDFALAEE<br>ATAVKTVVHGKVLGIEMPFPLANPDACVDSGLKCPLEKDESYRYTATLPVLRSYPKVSVLVKW<br>ELQDQDGADIICVEIPAKIQ
>Dm2<br>MKLSLGLFVIFAALIGFTSSTDVSQCPKSKSKALAAGDVSISNCPKSKCILKRNTEASIQMKIRPE<br>RDFQELTSDIQGIILDVPLPFPGYYGTSACPHIYDEAGEKKVGCPLKAGQVYTYKNSFKILPVYP<br>TVSLEIHWGLGDKHGDAACFQIPAKIKA
>Dm3<br>MLRPSSLQAVAIAIVLISSSASAEVVNFEPCPDSVDTCTIQQVRVSPCPEALNNAACNIRRKHNSE<br>MSFDFTPNFDADTLVASLGWAKSENVELPLLTLDSAACKYTPCPVRSGVKQTYTTLVPIEAKFP<br>LSPYTIRWALKDPVSQKRCCFTIDIKVVR
>Dm4<br>MLRIVVTLALILATVNATNVQQCKNKPFPLDVNIKDCEEPPCVVYKGTIAVMEVHFLGNNNNI<br>KSITATTTAKVLGMNLPYALPDEVSDVCRNLLYGAICPIDKDEDVTYQFNFYVEPSFPEITADVT<br>VTLNDAQNEPITCFVVSCKIRKGATAAQMDGYLLDWTNPL
>Dm5<br>MLRLSSLLPVAFALVLSSVSAEIVNFQTCEDSVDSCSISQVRVTPCPEANANAACHIRRRHRFTM<br>SFDFTPHFDADTLVASLGWAKSENVELPLLTMDQEACKYTTCPVRSGVTQTYTNNMPADARFP<br>LSPYTIRWALKDPVSQKRCCFTIDIKVVR
>Dm6<br>MSSFKKLSLCLVLSIMWTSVADSTPIRQCADSNYPQPLMVQIDDCDALPCDLWKGTEAKIDIQF<br>VATRNTMKKLSAEVHLTSLGVTIPYDLEASRGNVCSNLLHGAYCPLDAGEDVTYQLLLPVTTN<br>QPEVPTRLEVRLLDSDDENRVVSCFLADTRVKKPRSAV
>Tm1<br>MFCSIAYYVVSSVLIFVLADVYAGLNGSSPPPKPRPTVPENQEFYRTLANVTLDRYMDLISMPQ<br>VPSRYMRNDDDHPNKWIFEEDDDDYIRSYQVTSCPHERNPVVKYSNLRVHPDPLIFPGTIRITS<br>DIQVLEDVTEPITVQVRMRRRMVYFWMDLPCVAAYGSCLYENICDTTIKCPIFYELLGLPCGCP<br>IKKGNYRTHNKAYDVPDVLPTWLTSGDYQVILKASSNNRPLFCVRFKLSVEGPDDRNRLLSFL<br>SKIR
>Tm2<br>MRTGVCQRQSTDGLSTIKSVAVENCKNSELCEWTKGRNHELKVTYTAPKNVETVKVLISG<br>LVGGAELPFPGLKRYICKTPGNENFCPQTAGQEYTATITLPILSVFPKIETTAFVRFIDD<br>DAKSEAEEKLGCFHMQIKLIDDPEAPKVERVDNQEDDE
>Tm3<br>MALNRFVVLIALVAVGYASPFKSCDSKDGSILSLDIEGCPDDNYECILHKGDDAKISITF<br>TPNVNSNTVMAKAFGIIAGIPIPYAIPESNACLHGLVCPLKSGVVTKYSQTFPVQRTYPS<br>LALEVKWMLIDDRGDAIVCAKIPVRLQ
>Tm4<br>MLRLSVIVFLAISAFGQQVVVPKKCAETGGGILKKLVVSPCTAEPCVMKKGRNATLTFEY<br>IADQNSTTAVIDARVKAFGMKVPVPGIDTDLCKIISCPVKEGATYTAQITVPVPKIPIKK

TTLEVKIIGDKGLSSCFTHPLFLK
>Tm5<br>MVSFNVPAIALAVALTIHQEVDGQRKLKSYTDCGRDTNTVRVRPSVIFGKLNVTLPGVDS<br>ELCGKHLKCPIKANELQDADIEVPVFKTYPS
>Tm6<br>MKRLGQTLLICGFLCAQIVHLQKKVINDMQICGGNGVVQLVIVEPCSNEPCMFQRPGQIK<br>VHIEAGFDRPSETAKLSASAMVKSRNLTLPGVPNNLCRLTSCPLNGRGDIRNLSFTVPIK<br>SFYPPGTYELSFVASGDDGMEICRRLNVTIITDQ
>Isc1<br>SPLHTTTDAMARHLLPGTLLLAALCGLTEAIKWTDCGSSEGQVASVSVTGCEHTDTCNLK<br>KGTDVSLIIDFTSNVKSPTVQVKAYGVLEHVPIPFDVPQPDACQSGVTCPVQPAGKYNYR<br>GSFPIKPMYPSISLDIKWELLDDKDQYLVCVLIPVKID
>Isc2<br>MAASLRLELLALVVTRLMAIFEASEFTVVDYTTCGGEALQVRVDPCEQLPCSFKKGRSVK<br>IEMDFAASKNQSQVSVGIMGKAGGMLLPLPFNQKDGCKGSGLDCPLVAGRNYTVSRAVRV<br>YRIYPKMEILAVFEIRGNDGEVLACVQFPVHIQ
>Isc3<br>MGEKPLFKQLFFTSQHHSSRDILTFIMAGSMVFFALCAFAFVSAATAADALSVVKVDQCT<br>DGSTKNVKEVRMTHCESLPCNVKLADKPRFEVDFVAERDSNVMRVKVQGQIGALNPENFP<br>GFKSDACSNMGVECPLVAGKQYTAKSQLTMSPTFPPTQAKAIFKGVDAGGELFCFTVPVE<br>LKQ
>Isc4<br>MNRLCYAASLVLVSAALVSAEFDVVKHEKCGGEFGEVRIDPCPELPCIFKKGTPLKLQVDFVAA<br>DSFKTLQMKLLGELSKGVWLPFPNFGRNACKKNGLTCPLESGKPYTLQSTLNVLSSFPTVRGS<br>RGVYMKGDNQTIFCFLVPVKVSD
>Isc5<br>MQHHFEAALLVCVIALLGQCSAIWKSCNADNGQVLDMRVTPGGDREVYILRKGTNVTLNIEF<br>WSSVDSGSAKVRAHGMVMGVPIPLKMPNEDACVDCGISCPMRNGEKYLYKQGFEVKASYPK<br>MSATIKWALGDDNGGTVACVLIPVEIVD
>Isc6<br>MASSVVLALAVVVCAGFVVGDLVAFKDCGHGNVKSVNIVSCKKAPCKVKIGTRVVFEASFVA<br>PFDVTSAFNDIGAYIERHRFRLPEPRVDACNGYLSPRCPMRKGQTYTYRYTMPVREIYPATPATI<br>EYKLTNEEGRLIGCTSIPVQIFR
>Isc7<br>MPGAVIQSLIVTPCTSDPCVIPVGTQINITFEVESNQDSDRVLFDPRVTVLGVQLPIPGVEKDACR<br>SGAVVCPVHKGKLFAGTISAYVYNFVPSLTVTTTWKMVGAQGIIACGATNVTIVRK
>Isc8<br>FLPTAGGDVTHVRVQPCTHEPCSIKRGETVLIEVPFRANQDSDKLATKISAQLEDLELPLPSLKK<br>DGCREQGIRCPLEKNEKYVFTYSLLLIYLLFQLNTTAKLSLTGAKGVVFCVTFPVNLTD
>Isc9<br>MSIILFSTDQNSAKLSTKISAVIDNKNELVLPSVKRNTCRNQGIKCPLEQGTDYVFEYNLEIKPSF<br>PTLDTTAKLNITGAEGPVFCVTFPIHLVE
>Isc10

MLSTRNMVLLAGSALALLLVVPACVRGQDAPGEPTFRNFYNCTWAKGDPGEQAQAYLGGCG SEEICPLYRGSEAKLELQFSPRSDTSGVFRRIFGLFDKVLVPYGREVNVCNSTTSIDDNVRCSEES HGLRQGRLYRHSGAFLVKPFFPKVQLNVTVYLYDKSPQKVPVACVQIPVQIKDKDRS
>Isc11 MRDSDVMRLKVQGQIGQLQPENFPGFKSDACSQMGVECPLVAGKQYTAKSQLTMSPTFPPTQ AKAIFKGVDAAGELFCFTVPVELKQ

Table S6: Protein sequences of NPC2 used to construct motif analysis.

Accession number	E-value	organism	Abbreviated name
**XP_022652195.1**	2e-31	*Varroa destructor*	Vd1
**XP_022649311.1**	5e-128		Vd2
XP_022654520.1			Vd3
XP_022654596.1			Vd4
gi_283753684			Vd5
NP_608637.1	5e-104	*Drosophila melanogaster*	Dm1
NP_650331.1	2e-113		Dm2
AAN71436.1	2e-112		Dm3
ADL59625.1	6e-120		Dm4
NP_001247376.1	1e-112		Dm5
OQR69913.1	0.0	*Tropilaelaps mercedesae*	Tm1
OQR78448.1	6e-114		Tm2
OQR69614.1	1e-103		Tm3
OQR72593.1	4e-98		Tm4
OQR76180.1	8e-61		Tm5
EEC00381.1	5e-112	*Ixodes scapularis*	Isc1
EEC07080.1	2e-108		Isc2
EEC07323.1	4e-132		Isc3
EEC03680.1	9e-106		Isc4
XP_002435260.1	1e-108		Isc5
EEC08723.1	6e-106		Isc6
EEC06676.1	1e-82		Isc7
EEC13424.1	2e-85		Isc8
EEC13423.1	3e-63		Isc9
EEC18444.1	6e-134		Isc10
EEC16220.1	9e-57		Isc11

>DgalNPC2-1 GAIF01015586.1 lcl|ORF7<br>MANNFFFLIVASLALTAYAGELVQINNPVQCDGLTTIKAVYVEDCTNGEMCDLVKGHNYSLKIT<br>YTAPKDVQTAKALVTGVIGGAELPFPGLQRYVCKTPGNEDFCPQVKGKDYTVSMVLPIMSLFP<br>KLETTATVRFIDDDAKSEAEEKLGCFQLPIRLVDDPNAPKQPTRVDNEDDDE
>DgalNPC2-2 GAIF01031731.1 lcl|ORF2<br>MANNFFFLIVASLALTAYAGELVQINNPVQCDGLTTIKAVYVEDCTNGEMCDLVKGHNYSLKIT<br>YTAPKDVQTAKALVTGVIGGAELPFPGLQRYVCKTPGNEDFCPQVKGKDYTVSMVLPIMSLFP<br>KLETTATVRFIDDDAKSEAEEKLGCFQLPIRLVDDPTPLSSRRALTTKTTMSNERQRDDAT
>DgalNPC2-3 GAIF01008081.1 lcl|ORF7<br>MANNFFFLIVASLALTAYAGELVQINNPVQCDGLTTIKAVYVEDCTNGEMCDLVKGHNYSLKIT<br>YTAPKDVQTAKALVTGVIGGAELPFPGLQRYVCKTPGNEDFCPQVKGKDYTVSMVLPIMSLFP<br>KLETTATVRFIDDDAKSEAEEKLGCFQLPIRLVDDPTPLSSRRALTTKTTMSNTSARDDAT
>DgalNPC2-4 GAIF01004272.1 lcl|ORF7<br>MISQRIVVLPLIVAVLAVAVHGSPYKLCKTPSIGTVVKVVIEGCSDDVQSCVLRKGTEAKLSLTFI<br>PNVDVKSLVAKAYGLIAGVPVPYPLKNSDACKDSGLSCPLKKDPKVTYKQAFDVMKEFPSMS<br>LTVKWVLRDEQNAHIVCPDTCQTGVVARREFWTVWTQVVSSRPLRKGCVAVGDKNVAFRPV<br>YFAVKLQ
>DgalNPC2-5 GAIF01014813.1 lcl|ORF6<br>MPRFTATTAAAVVIAAIVLLSASPSVLGQREITFTPCDDPGKGIVKRVTATPCDTDPCELKKGSM<br>TSFNFEFIADQNTTSITLDARVRVLGIFIPVPFIETDMCRVIDCPVVKGQEIVGQFNATVPRVPVKS<br>SVIRVKVIGDEGMSCCISYKVVLK
>DgalNPC2-6 GAIF01031088.1 lcl|ORF3<br>MAPKRGLRRVAAVPAERALATIVVLAAVCVAIVRGSPFKSCGGSAKITAVDIENCPSSAKSCTVH<br>KGAAFKVQVTFKPAKDAVNATLHKAVIINNVTKKLTPCVSVCNAGLKCPLHKNVPATLQYTGH<br>VPKDSPKGKLFGQLSLTDSAGSLILCAQSPIEVA
>Vd1 XP_022652195.1 epididymal secretory protein E1-like [Varroa destructor]<br>MKTFLVIFALAAIAIASPFTSCNHSQGTIRTVEISGCKDTDPYCVLYKGTNVNLSITFVPASDASR<br>VTVEVYGLISGFGIPYPLPNSNACARGLICPLKNGETATYKHSFPVRNGFPSIALDVIWTLFDER<br>KNRIICARFPVVIQ
>Vd2 XP_022649311.1 epididymal secretory protein E1-like [Varroa destructor]<br>MLRFVMLAAIASAAYAGELYKVQNVQQCDGLSTIKSVEVENCTKEDLCELIRGHNHLLRVTYV<br>PPRSAQVVKVLITGFVGEAELPFPGFKRYVCKTPGNENFCPQTAGQEYTTTMVLPILSVFPNIET<br>TAFVRFIDDDAKSEAEEKLGCFSMKIRLIDDPNASTEPANVDNQEDDE
>Vd3 XP_022654520.1 uncharacterized protein LOC111247622 [Varroa destructor]<br>MTSVTQSALIILVVVTTWPWASFARKVQYQECNPKRNIKMMDVDNCDVQPCRVFHRTTIKMT<br>TQLSSPINSSSAEWEIRAFVNGRPYGHPEPVSDACLFVNSTSPNAFSSSHRTPLNSDQFKSGPST<br>AYRQKSFLPMCPLFAGKIYRFSNVFYVHDQFPKVATTIQYLLRGDNDEMIACARLHILMSS
>Vd4 XP_022654596.1 epididymal secretory protein E1-like isoform X1 [Varroa destructor]<br>MMLLDLFTVVFAVMLMMNENNAKQLDSYVNCGGTGHIENITVVPCESEPCEFAKGSKVNVH<br>FIGSFSHDANSVRVQPSVIVDKMNVTLTGVDQELCGKHVQCPIKANELRDAEIEIPIFNVYPNIK

TEVVFMIFSENVLQFCVRTKIIISDKK
>Vd5 gi_283753684<br>MWLYVWVTVAALATVGNGSIFQSCEKIETKGTILTLDIEGCPNTENVCVLKRGVDTKIALTFTP<br>EVEMNNITVKVFGVIENIPVPYPIPHSNACIKSGLECPLEKGVPATYTQTFPVPKVFPSIFLIIKWT<br>FVDSFNNELICALIPTRLQ
>Dm1<br>MLRYAVIACAALVVFAGALEFSDCGSKTGKFTRVAIEGCDTTKAECILKRNTTVSFSIDFALAEE<br>ATAVKTVVHGKVLGIEMPFPLANPDACVDSGLKCPLEKDESYRYTATLPVLRSYPKVSVLVKW<br>ELQDQDGADIICVEIPAKIQ
>Dm2<br>MKLSLGLFVIFAALIGFTSSTDVSQCPKSKSKALAAGDVSISNCPKSKCILKRNTEASIQMKIRPE<br>RDFQELTSDIQGIILDVPLPFPGYYGTSACPHIYDEAGEKKVGCPLKAGQVYTYKNSFKILPVYP<br>TVSLEIHWGLGDKHGDAACFQIPAKIKA
>Dm3<br>MLRPSSLQAVAIAIVLISSSASAEVVNFEPCPDSVDTCTIQQVRVSPCPEALNNAACNIRRKHNSE<br>MSFDFTPNFDADTLVASLGWAKSENVELPLLTLDSAACKYTPCPVRSGVKQTYTTLVPIEAKFP<br>LSPYTIRWALKDPVSQKRCCFTIDIKVVR
>Dm4<br>MLRIVVTLALILATVNATNVQQCKNKPFPLDVNIKDCEEPPCVVYKGTIAVMEVHFLGNNNNI<br>KSITATTTAKVLGMNLPYALPDEVSDVCRNLLYGAICPIDKDEDVTYQFNFYVEPSFPEITADVT<br>VTLNDAQNEPITCFVVSCKIRKGATAAQMDGYLLDWTNPL
>Dm5<br>MLRLSSLLPVAFALVLSSVSAEIVNFQTCEDSVDSCSISQVRVTPCPEANANAACHIRRRHRFTM<br>SFDFTPHFDADTLVASLGWAKSENVELPLLTMDQEACKYTTCPVRSGVTQTYTNNMPADARFP<br>LSPYTIRWALKDPVSQKRCCFTIDIKVVR
>Tm1<br>MFCSIAYYVVSSVLIFVLADVYAGLNGSSPPPKPRPTVPENQEFYRTLANVTLDRYMDLISMPQ<br>VPSRYMRNDDDHPNKWIFEEDDDDYIRSYQVTSCPHERNPVVKYSNLRVHPDPLIFPGTIRITS<br>DIQVLEDVTEPITVQVRMRRRMVYFWMDLPCVAAYGSCLYENICDTTIKCPIFYELLGLPCGCP<br>IKKGNYRTHNKAYDVPDVLPTWLTSGDYQVILKASSNNRPLFCVRFKLSVEGPDDRNRLLSFL<br>SKIR
>Tm2<br>MRTGVCQRQSTDGLSTIKSVAVENCKNSELCEWTKGRNHELKVTYTAPKNVETVKVLISG<br>LVGGAELPFPGLKRYICKTPGNENFCPQTAGQEYTATITLPILSVFPKIETTAFVRFIDD<br>DAKSEAEEKLGCFHMQIKLIDDPEAPKVERVDNQEDDE
>Tm3<br>MALNRFVVLIALVAVGYASPFKSCDSKDGSILSLDIEGCPDDNYECILHKGDDAKISITF<br>TPNVNSNTVMAKAFGIIAGIPIPYAIPESNACLHGLVCPLKSGVVTKYSQTFPVQRTYPS<br>LALEVKWMLIDDRGDAIVCAKIPVRLQ
>Tm4<br>MLRLSVIVFLAISAFGQQVVVPKKCAETGGGILKKLVVSPCTAEPCVMKKGRNATLTFEY<br>IADQNSTTAVIDARVKAFGMKVPVPGIDTDLCKIISCPVKEGATYTAQITVPVPKIPIKK<br>TTLEVKIIGDKGLSSCFTHPLFLK
>Tm5

MVSFNVPAIALAVALTIHQEVDGQRKLKSYTDCGRDTNTVRVRPSVIFGKLNVTLPGVDS<br>ELCGKHLKCPIKANELQDADIEVPVFKTYPS
>Isc1<br>SPLHTTTDAMARHLLPGTLLLAALCGLTEAIKWTDCGSSEGQVASVSVTGCEHTDTCNLK<br>KGTDVSLIIDFTSNVKSPTVQVKAYGVLEHVPIPFDVPQPDACQSGVTCPVQPAGKYNYR<br>GSFPIKPMYPSISLDIKWELLDDKDQYLVCVLIPVKID
>Isc2<br>MAASLRLELLALVVTRLMAIFEASEFTVVDYTTCGGEALQVRVDPCEQLPCSFKKGRSVK<br>IEMDFAASKNQSQVSVGIMGKAGGMLLPLPFNQKDGCKGSGLDCPLVAGRNYTVSRAVRV<br>YRIYPKMEILAVFEIRGNDGEVLACVQFPVHIQ
>Isc3<br>MGEKPLFKQLFFTSQHHSSRDILTFIMAGSMVFFALCAFAFVSAATAADALSVVKVDQCT<br>DGSTKNVKEVRMTHCESLPCNVKLADKPRFEVDFVAERDSNVMRVKVQGQIGALNPENFP<br>GFKSDACSNMGVECPLVAGKQYTAKSQLTMSPTFPPTQAKAIFKGVDAGGELFCFTVPVE<br>LKQ
>Isc4<br>MNRLCYAASLVLVSAALVSAEFDVVKHEKCGGEFGEVRIDPCPELPCIFKKGTPLKLQVDFVAA<br>DSFKTLQMKLLGELSKGVWLPFPNFGRNACKKNGLTCPLESGKPYTLQSTLNVLSSFPTVRGS<br>RGVYMKGDNQTIFCFLVPVKVSD
>Isc5<br>MQHHFEAALLVCVIALLGQCSAIWKSCNADNGQVLDMRVTPGGDREVYILRKGTNVTLNIEF<br>WSSVDSGSAKVRAHGMVMGVPIPLKMPNEDACVDCGISCPMRNGEKYLYKQGFEVKASYPK<br>MSATIKWALGDDNGGTVACVLIPVEIVD
>Isc6<br>MASSVVLALAVVVCAGFVVGDLVAFKDCGHGNVKSVNIVSCKKAPCKVKIGTRVVFEASFVA<br>PFDVTSAFNDIGAYIERHRFRLPEPRVDACNGYLSPRCPMRKGQTYTYRYTMPVREIYPATPATI<br>EYKLTNEEGRLIGCTSIPVQIFR
>Isc7<br>MPGAVIQSLIVTPCTSDPCVIPVGTQINITFEVESNQDSDRVLFDPRVTVLGVQLPIPGVEKDACR<br>SGAVVCPVHKGKLFAGTISAYVYNFVPSLTVTTTWKMVGAQGIIACGATNVTIVRK
>Isc8<br>FLPTAGGDVTHVRVQPCTHEPCSIKRGETVLIEVPFRANQDSDKLATKISAQLEDLELPLPSLKK<br>DGCREQGIRCPLEKNEKYVFTYSLLLIYLLFQLNTTAKLSLTGAKGVVFCVTFPVNLTD
>Isc9<br>MSIILFSTDQNSAKLSTKISAVIDNKNELVLPSVKRNTCRNQGIKCPLEQGTDYVFEYNLEIKPSF<br>PTLDTTAKLNITGAEGPVFCVTFPIHLVE
>Isc10<br>MLSTRNMVLLAGSALALLLVVPACVRGQDAPGEPTFRNFYNCTWAKGDPGEQAQAYLGGCG<br>SEEICPLYRGSEAKLELQFSPRSDTSGVFRRIFGLFDKVLVPYGREVNVCNSTTSIDDNVRCSEES<br>HGLRQGRLYRHSGAFLVKPFFPKVQLNVTVYLYDKSPQKVPVACVQIPVQIKDKDRS
>Isc11<br>MRDSDVMRLKVQGQIGQLQPENFPGFKSDACSQMGVECPLVAGKQYTAKSQLTMSPTFPPTQ<br>AKAIFKGVDAAGELFCFTVPVELKQ

Table S7. Protein sequences of SNMP used to construct phylogenetic tree.

Long name	Organism	Abbriviated name
TRINITY_DN24266_c1_g1	*Varroa destructor*	Var24266
TRINITY_DN24854_c0_g1	*Varroa destructor*	Var24854
TRINITY_DN26930_c0_g1	*Varroa destructor*	Var26930
TRINITY_DN27012_c0_g1	*Varroa destructor*	Var27012
TRINITY_DN29484_c0_g1	*Varroa destructor*	Var29484
TRINITY_DN30020_c0_g1	*Varroa destructor*	Var30020
TRINITY_DN30199_c0_g1	*Varroa destructor*	Var30199
Q7KVF1	*Drosophila melanogaster*	DmQ7KVF1
Q9W0X0	*Drosophila melanogaster*	DmQ9W0X0
A0A0B4LGP3	*Drosophila melanogaster*	DmLGP3
CRQ	*Drosophila melanogaster*	DmCRQ
Q9VM10	*Drosophila melanogaster*	DmQ9VM10
PESTE	*Drosophila melanogaster*	DmPESTE
A8JNI3	*Drosophila melanogaster*	DmA8JNI3
R9PY22	*Drosophila melanogaster*	DmR9PY22
SNMP2	*Drosophila melanogaster*	DmSNMP2
Q961K6	*Drosophila melanogaster*	DmQ961K6
Q8INY3	*Drosophila melanogaster*	DmQ8INY3
Q9VLU7	*Drosophila melanogaster*	DmQ9VLU7
H1UUH0	*Drosophila melanogaster*	DmH1UUH0
Q9VJA7	*Drosophila melanogaster*	DmQ9VJA7
M9MRL5	*Drosophila melanogaster*	DmM9MRL5
A0A0B4KGL9	*Drosophila melanogaster*	DmKGL9
Q8SYC3	*Drosophila melanogaster*	DmQ8SYC3
Q9VET1	*Drosophila melanogaster*	DmQ9VET1
Q9W0X1	*Drosophila melanogaster*	DmQ9W0X1
A0A1V9XAB4	*Tropilaelaps mercedesae*	TrpXAB4
A0A1V9XVQ6	*Tropilaelaps mercedesae*	TrpXVQ6
A0A1V9X7A6	*Tropilaelaps mercedesae*	TrpX7A6
A0A1V9XVL0	*Tropilaelaps mercedesae*	TrpXVL0
A0A1V9XA72	*Tropilaelaps mercedesae*	TrpXA72
A0A1V9WZC0	*Tropilaelaps mercedesae*	TrpWZC0
A0A1V9XN95	*Tropilaelaps mercedesae*	TrpXN95
A0A1V9XQP1	*Tropilaelaps mercedesae*	TrpXQP1
A0A1V9XHA3	*Tropilaelaps mercedesae*	TrpXHA3
A0A1V9XPJ7	*Tropilaelaps mercedesae*	TrpXPJ7
A0A1V9XQK1	*Tropilaelaps mercedesae*	TrpXQK1
A0A1V9XQS8	*Tropilaelaps mercedesae*	TrpXQS8
B7PC41	*Ixodes scapularis*	IscPC41
B7Q461	*Ixodes scapularis*	IscQ461
B7QI62	*Ixodes scapularis*	IscQI62
B7PD82	*Ixodes scapularis*	IscPD82
B7Q458	*Ixodes scapularis*	IscQ458
B7P9A7	*Ixodes scapularis*	IscP9A7

>DgalSNMP-1 lcl|ORF3 GAIF01013616.1 GAIF01013616.1:1-930 + PF01130.21<br>0.5, 0.38, 0.36, 6.48, 32.29, 0.7<br>MEAISALNSEESKKGKFGLAIDKNETEPGIFNMYTGATGVTDLNRIYSVNNQHELSIWPTEHDN<br>VCNQVDGTFGYLRPPMNETNEQVVFVPDMCRPITIHYQKDVTFHGIRLRRFTLTPENFFSSKQK<br>PENFCYDSKMKMPSGVAEVGPCKQGAPILMSMPHFMHADPSFSEKVKGMTPDIEKHTFTMDH<br>EPTTGMTVAVRGRLQANIAVRPVDLLGADDLPTVVMPLFWQDMYVEAGPGVVAFLKKVLG<br>YPGLAKIGLVILLLVGLIVLFIGAGLLINYSRRVRQTG
>DgalSNMP-2 lcl|ORF1 GAIF01030464.1 GAIF01030464.1:1-1157 + PF01130.21<br>0.19, 0.2, 0.05, 1.96, 4.9, 0.1<br>MGVVSYTTFPSILEQQVNANLVIDPSNEVYESWKESPIPIYAKMYLFNYTNPNEVIEGERPKLQ<br>QLGPFVYRERRHKVNITFNGNGTVSYRQVVSYEFLPHLSRGTLDDSLYTLNVPLIGTAYRNRK<br>YLPDEQEPMAASMEEAFQMYNQSLIIHRKVRELLFEGYEDPMLRIAKTMGWSPFSRFGYQIGR<br>NNSNDGLYTIFTGSNGMDKYGTVESWQGRQKVTGFRKFCNFVNGTSGEMWPPYTHTTHSHL<br>NFFAPALCRSLRLDFLRNETVKGIRVRASRESVVTPAPSQSSKSLLAVRIAGHLRHFVSFHSKID
>DgalSNMP-3 GAIF01028952.1 GAIF01028952.1:1-882 + PF01130.21 lcl|ORF1<br>0, 0.2, 0, 0, 0, 0<br>MDFNGDRNLDYYMNSQCSVSGTNGQIFPSFYATPAQVTVFFPFLCKPWTLRSYKHFILQGDEEI<br>VRFIADKATFLRESTSDKCVNWKPTGISGLFDASNCHLDNKVGNEFVNYFAPVLSWPHFLHAD<br>VKLHDDVHGLRPESRKHEFFIDVHSRSGVTVDMRLRYQVNVRLYPSRIMEATIPKIMFPIYWQE<br>IA
>DgalSNMP-4 lcl|ORF1 GAIF01014656.1 GAIF01014656.1:1-610 + PF01130.21<br>2.42, 3.98, 4.08, 5.09, 5.72, 2.98<br>MSKRYADYDLKMYTMPVVLSLPHFLHGDPLLLANISGLHPDEERHRFYIDVHAGSGLTVDMR<br>FRFQVNMRLVPLETEQVPSIIFPIFWIEDANLPSDRNKLVHILRKSSVVRWSALAACLCLLATSA<br>LMCGLLL
>DgalSNMP-5 Icl|ORF20 GAIF01027311.1 GAIF01027311.1:1-2641 +<br>7.55, 10.23, 6.79, 31.36, 42.21, 5.35<br>MLNDTDDGYLTVYAGIDDATRSRRNFIVQWRSRETIPYYSGRCNRLWGQTQSCSLHSVVHHLQ<br>TLAYFWRLYVGPGHCASTAHVWKTALSSCALLVVSTYFHRRPAMMPLTASSWTAGRGACLMA<br>PVNMAFPPSSACRISSTLRKNFFRMSMDFNPTSRSITFISTSTRC
>Var24266<br><br>MKLIRGKQKSAPFLNFLKMGVNAGRGAGVICVVVGAFLVVLGAAVLINFTATFQFILSRKVVL<br>SSTAQGFPIWRDVSDHLDTKTAWYFFNLTNPEEFMQGTQPILQEVGPFWYKTILKKINIEFLDN<br>KTVTFEEHREFEFDAENSVLPNTTRITMINVPVMSALQRLKPLGYFARLAVDPVIKAAIADQQL<br>TANYSVAELTYDGVYNNLVALSKIKEGFLEAIFALNSKDNKKGKFGFGVDKHDPTTFNMFTGG<br>TGVMDLNRIYSINHKHVLSMWPNIENTTCNIVDGTFGYLRPPMNESNEQKVYIPDMCRPVTIR<br>YEKDVNTNGIRLRRFILGKENFYNREAEPANACYDEIYREPSGVAEAGPCKQGAPILLSMPHFM<br>HADPSFSEKVIGMNADPERHTFIMDHEPMTGMTVTVRGRLQANFRVTQLDCLDGEKLPELLM<br>PLFWQELYVEAGPGIVAFLHKVLSYPGLAKIALIIVLLLGIIISIIGAGFLMNHSRRTRQTEQLNPT<br>KYHKMATQEATRKPELDQLV
>Var24854

MKSRGIAGLGISCAFFLVACLSLLAYLKIPSFLQLAVSRSGLSPGSFAADLFSAIPFDIRQNVYMF<br>NITNSEDFFNGAKPRFKQLGPYIFRIGLRKQMTWSEGDLLTFRENRKFWFDADLSHGHLNDTV<br>YTVDPIYAMAQEVIDELPEFLERLFRPLLKSRKVLFKHSVDELLYNGYSDSLAELAHLIKPNLPV<br>IGGKIGYLRAFNDTDDGDITVFAGTNPFTKDKRNLITQWNHKKTIPYYGDGCNGIWGANAELF<br>PSFLSSSPPAQIGVFLPLLCRPWSLHFNETRQENGIELARFATGLDIFSPSNSSQIEQCAQPAGWPR<br>GVFDVRACQHSFPALISLPHFLHSDEKILDAVDGLTPNVTEHDFHLDIFPLLGIPVRPAIRAQINV<br>RIWRHLDTSQSNNPPIVYPVLWQEIVLSESAISYLSTMIWYIILLPETTLRVILMLLTILSVSLAVS<br>YAYTSFSKVSDEETESILASAEDSAASS
>Var26930<br>MCGGSFCQSELNKPGGHRSEASVGRCTPHLAVTCSLCIGLFFCVFGMVSYAAFGPILRQQVKA<br>NLIIDPSNEVYENWKETPVPIYVSMFLFNYTNPDDIILGAKPKLQQLGPFVYRERRQKVNITFNG<br>NGTVSYRQLLSYEHLPELSASSLDVKLYTLNVPMIGAAYKNRKTPSNEEQPMASALEEMFSKM<br>NQSLLIHRTVRELLFDGYEDQMLKLAKQWSWSPTTRFGYQIDRNNSNDGIYTVFTGENGMAN<br>YGTIESWQGRHRVIGFRKSCSFINGTTGEMWPPYTLTSKSSLLFFTSPLCRSLRLDFLRNEVVKG<br>IQVLRYHLNERLFDYSIEENQCFCSKKKGKDPECFPNGVLDLNAPIVVSLPHLLYASPSITEAVE<br>GLSPDPQLHEFFMDVEPSMGIPLRVSAKVQMNVIVDAFKYFKYFEMFETRKFLPTFWIETAAV<br>VNDDFAFKIRLVVQDLGSYVSFASYAWVLIGLFIIMFTLGYVIAHTRRSRMGQPSPYGYSTIKM<br>AEVVTGR
>Var27012<br>MILTGVPRWVAFIVFAASAVVFILGVVGYYMVPSIIGAKVSQQMRLVENGSTLKRWANVAVPIY<br>FSAFMFNITNPEEFANGEKPQVQEIGPYVYLQKRRKLITHIDRDVVTYKDYKSYHFLPEYSIGTP<br>DDRLYALNVPLVAMDKLIGSKLPGDLAKSLLQPILEGLLEKHNEKLVVQRNVSEMLFDGYAVP<br>MMNELSKLAAAFMPDTKLPAISNFGLFYNKNNTADQWFTVGTGAGEYPFATILEWNNHTSLD<br>FWSRDNCNKINGTDGGQFSPFVRTDQKLFVFATDLCRSIYFEYEKNTQVKGVDTKRFTVPDAIF<br>ASGKRQPENQCFCEAINRCHEGGVIPLSSCRKGAPVMLSAPHFYQGDAKLVQDVAGLRPQKK<br>YHETYLDVQTMTGLVLRAAKRLQINIDLKQSDLLYPLANVSSRVMPIAWVEERMEATGPMTD<br>GLKEKLIFPQKMGVLISTAAALGGAIGAIAAILVIVVRQAGTTRSAKLQPSK
>Var30020<br>MPRHVLTISPVKTVSAEIERDPLERYNAMTADLQNGKQKEKTKTPSKCCCCSYKVCSLLLFILS<br>FIMTVTAVVLQSQFDAFFDRALKDQMHLGPSGLVFPAWRVSDLRMRMRVYFFNITNPVEVQLG<br>DKPILNELGPYTWRIYMEKFDIKFHENSTLSYREKKWYQFLREESIGGYNDIITTVNVPYAAVA<br>QRLKDGGTVAKSTATFTLNGLGQTVMHAKQVGELTFEGYPDFLILVANAVEAGKKEDSGFGTI<br>LGTGLRIFTNVLAAEPDVQGSFGYFVDRNNTDDGIVTIFTGEDNRQKINRVNVVNGHTELTTW<br>PQHKCNKIKGTMGHLRPPMSSRENPLLIFVPDICRSLPLRYENVSVYEGLSTLRFVAGRETFNNS<br>GPDPCFAGSRKFKSGVMDIGPCKKGAPLVISFPHYLFADEAHQNSVIGLNPRKEAHQFYLESDP<br>LTGVTVSVRARFQVGVVMERVHGLGNLGTIFDGTIPLFWQELQLEALPPTVFQLSILRQLPIYA<br>KQTALVLIGTAILISLIAVFLVYRYVHSTYVL
>Var29484<br>MKKSLKNELIGVLCHTEENGLFTMCSGGQRLSEQEVRSDQRYDSAPTGCSPLLRVIISLCIGLLF<br>CILGVVSYTTFAPILEQQVKANLVIDPSNEVFESWQEPPITINVKMYLFNYTNPEKILAGLKPTV<br>DELGPFVYRERRQKVNITFNGNGTVSYRQIVSYDFLPHLSVGNLDDELFTLNVPMIGSAYKNR

KALLNEESMMAHTIEELFEKHNQTFLIQRKVRELLFEGYQDPLLRVAKEMGWSSSTRFGYQVN<br>RNNSDDGIYTIYTGEEGMENYGTVDSWDGKKRVLAFRRSCSFINGTTGFTISSMTEFIQSTRGK<br>KGWRTTARWIAGTARKGYSLSALRYHLSQRLFDYSIKENQCFCTKKKGKDPECFPNGLLDLNR<br>CQPDAPIVASLPHHLYAASSVSEAVDGLSPDPELHEFFMDVEPTMGIPLRVSARLQLNVIVDAFK<br>YFKQFQVFKKRVFLPTFWTETTAMINDDIAFKIRLVTEDLANYVTFAALAWVLIGLIVIICTFGY<br>VIAYTRRSVRLIQKIYVDYNSIWDISNPDPSFLKDRLVF
>Var30199<br>MGLKKSYVVLISASLLVSFLGCIGILTLHPLFQTLLSHRLPLKPGSESYNGWKKVPFPIYQRFYY<br>FNVTNPDEFLNFGEKPVLEEVGPYTWRSEWVKEAVEWHANGTLQYRERKRFWFDREQSAGD<br>QEDVIFTINTPLVAASQKIRNASPLIKLAFLIFLNAANETLFIRRSIRQLTYEGYPDVLAHISHVIDP<br>NVPVKDGRFAYMSGKNDTDEGLFNVYTGSVDLRYFNRIDKWNGRTELPWWESGTPCAKLLG<br>TNGELVHPIVSSDEHIYFFNPVFCKPWRLTRAPDVDSLGITLTRFVAGPEVLFNSSREPANRCFET<br>PGKSLPSGGMDLSRCQFGLPLVLSYPHFYAADSKYLEKVEGLSPNQERHQFQIDIEPRLGITLGL<br>SARAQINVKLERVDFLKYFRSVPELVFPVFWQDVVIEQTPAFADHIRVLLDRPLFYAQFAFRFMT<br>MCGSLVALGAYIYVVILAKKESKKDRFGPLLDDEEINEDEHGEGDVAEDSASGQSQGH
>DmQ7KVF1<br>MNGPKHKFCTKLSSTYLRKWWITIVVAAALIIGGIVVACEFTVLIDAVVDRMVALRPGAK<br>TFGWWAKPPVEPRISLYIYNVTNADDFLSNGSKAIVDEVGPYVYSETWEKVNIVENDNGT<br>LSYNLRKIYSFREDLSVGPEDDVVIVPNIPMLSATSQSKHAARFLRLAMASIMDILKIKP<br>FVQVSVGQLLWGYEDPLLKLAKDVVPKEQKLPYEEFGLLYGKNGTSSDRVTVNTGVDDIR<br>RYGIIDNFNGRTHLPHWTTDACNTLAGTDGSIFPPHIDHDRILHVYDKDLCRLLPLVFEK<br>EVMTSNEVPGYRFTPPEWVFADVDSHPDNMCFCPAGKPSCSPNGLFNVSLCQYDSPIMLS<br>FPHFYLADESLRTQVEGISPPMKEKHQFFFDVQPKMGTTLRVRARIQINLAVSQVFDIKQ<br>VANFPDIIFPILWFEEGIDNLPDEVTDLMRFAEQVPPKIRVALIVGLCALGVILLLLSTF<br>CLIRNSHRQSTLHLEGSNYLATAQVDMNKKQNKDNQPARY
>DmQ9W0X0<br>MQLDDILHINNCKADCSSLSTTPNPKTDLVNMNGPKHKFCTKLSSTYLRKWWITIVVAAA<br>LIIGGIVVACEFTVLIDAVVDRMVALRPGAKTFGWWAKPPVEPRISLYIYNVTNADDFLS<br>NGSKAIVDEVGPYVYSETWEKVNIVENDNGTLSYNLRKIYSFREDLSVGPEDDVVIVPNI<br>PMLSATSQSKHAARFLRLAMASIMDILKIKPFVQVSVGQLLWGYEDPLLKLAKDVVPKEQ<br>KLPYEEFGLLYGKNGTSSDRVTVNTGVDDIRRYGIIDNFNGRTHLPHWTTDACNTLAGTD<br>GSIFPPHIDHDRILHVYDKDLCRLLPLVFEKEVMTSNEVPGYRFTPPEWVFADVDSHPDN<br>MCFCPAGKPSCSPNGLFNVSLCQYDSPIMLSFPHFYLADESLRTQVEGISPPMKEKHQFF<br>FDVQPKMGTTLRVRARIQINLAVSQVFDIKQVANFPDIIFPILWFEEGIDNLPDEVTDLM<br>RFAEQVPPKIRVALIVGLCALGVILLLLSTFCLIRNSHRQSTLHLEGSNYLATAQVDMNK<br>KQNKDNQPARY
>DmLGP3<br>MQLDDILHRITIVVAAALIIGGIVVACEFTVLIDAVVDRMVALRPGAKTFGWWAKPPVEP<br>RISLYIYNVTNADDFLSNGSKAIVDEVGPYVYSETWEKVNIVENDNGTLSYNLRKIYSFR

EDLSVGPEDDVVIVPNIPMLSATSQSKHAARFLRLAMASIMDILKIKPFVQVSVGQLLWG<br>YEDPLLKLAKDVVPKEQKLPYEEFGLLYGKNGTSSDRVTVNTGVDDIRRYGIIDNFNGRT<br>HLPHWTTDACNTLAGTDGSIFPPHIDHDRILHVYDKDLCRLLPLVFEKEVMTSNEVPGYR<br>FTPPEWVFADVDSHPDNMCFCPAGKPSCSPNGLFNVSLCQYDSPIMLSFPHFYLADESLR<br>TQVEGISPPMKEKHQFFFDVQPKMGTTLRVRARIQINLAVSQVFDIKQVANFPDIIFPIL<br>WFEEGIDNLPDEVTDLMRFAEQVPPKIRVALIVGLCALGVILLLLSTFCLIRNSHRQSTL<br>HLEGSNYLATAQVDMNKKQNKDNQPARY
>DmCRQ<br>MCCKCCGETQRKVWVFGLGSVFLLLGILIVVFWPGIADNLVEDGLTLKPGTDAYESWLEA<br>PIPIYLSFYMFNWTNPEDIRNPDIKPNFVEMGPYTFLEKHKKENYTFYDNATVAYYERRT<br>WFFDPERSNGTLDDMVTAAHAITATVADEMRDQRKIVKKIINFMLNHEGGKLYVTKPVGE<br>WIFEGYQDNITDFLNLFNTTKIDIPYKRFGWLADRNESLTYDGLFTIHTGTDDISNLGRL<br>THWNGKSETGFYEMPCGIVNGTTGDMFPPKMNVNDEITIFATDACRFMNLRPRGTYENHG<br>LTATKWVGTEETLDSGENYPNQACFCDEARFDECPKTGVVECKACRDKAPIYSSFPHFYL<br>ADQSYVDAVSGMKPEKEKHEFFLAVEPITGVPVQVHGRIQINMMIEPDDDFDIYRGVQKV<br>LMPMFWFDQYAELSSELASKAKLAINLSSYGIIFGYSMIAFASVFLITGITLTVTKKWVR<br>RTPEDEDILTN
>DmQ9VM10<br>MPTQNSAMWGQKSNRKLIIGIFGFCLGLFGILCGMFWVDLFDWIMHKEMALAPDTRVYEN<br>WKSPPIDLSLDIYLYNWTNPEDFGNLSTKPILEQVGPYRFIERPDKVDIHWHPENASVTY<br>RRRSLFYFDAAGSNGSLDDEITTLNAVALSAAATAKYWPPVKRSLVDVGLKMYGAEMSVQ<br>KSIDELLFTGYNDAMIDVAMAMPIFGDEVKVPFDKFGWFYTRNGSADLTGVFNVFTGADQ<br>LAKLGQMHSWNYQENTGFFDSYCGMTNGSAGEFQPQHLKPGDSVGLFTPDMCRTIPLDYV<br>ETVDIEGLEGYKFSGGPRSVDNGTQYPENLCFCGGQCVPSGVMNISSCRFGSPVFMSYPH<br>FFNADPYYPDQVEGLSPNQKDHEFYMVVQPSTGIPLEVAARFQVNMLVEPIQGISLYTGI<br>PRIFFPLVWFEQKVRITPDMADQLKVLPIVMLSGHIFAGICLIVGITLLCWTPVQILLAS<br>CRNRRYDLRTKTKTNGQYKSRSQFSSAEELKSKASTLVCEKSVKGSPDSSPLLEKGRKPT<br>IIKSQTGESVATASTAISDNKQD
>DmPESTE<br>MTSRTRHCARLGIVLLGICCIASGIYLFRNWIDMFTRMRGQEMALSPNSRSFEGWKVSPL<br>PLDFDIYLFNWTNPDDFYVGSNKKPHFEQLGPYRFREKPDKVDIEWHNHNASVSFHKKSW<br>FYFDAAGSNGSLWDKVTTVNSVAHSAARRAAVDWFARTGVNIANKLYRQGVTITKTVDEM<br>LFKGYEHPFISVGKLLRPQDVPYKRIGYHYPRNGSSEFDGDINMFTGADDIAKMGQIHTW<br>NNLTHTGAFEGTCGQVHGSMGEFFPPNLGTKDTVYMYMPKMCRAIPLDYVETVTVHGVTA<br>YKFSGTRHAVDNGTLYPDTRCYCVGGKCMPSGVINIGPCSFNASVYMSFPHFYMADPSYL<br>EAIEGLRPEREKHEFFMALEPNAGVPMDVGGGFQANYYMEPIPGITLYENVPTVMIPMMW<br>CEERVRVSEEIAADIALVPLIVLLGQIVTGILLAGGLICTCWYPTRQVTHFCHSDPKAKA<br>SVLRPLNAFGVNSSAATAPVAQLFRNNISSSGNERVGVRLLDYNRDSGIRLESGTMESSH<br>RERLISEDSPDVVVR
>DmA8JNI3<br>MGLEKHYLRYGRTARDHLLDWATCGRGRRQQQQQQQQTHAQPSQGSTATGRRSRPHVTH<br>RGTPLSMLISNGVRISNNRLAVIIIGIITLILGIILSSMPWLDYFILKNLRLWNDTLSYH<br>YWQRPGVIRLTKLYIYNVTNPDGFLRGEKPHLQEVGPFVYREDMQKVNVKFHENNYTVSY<br>QHKKILQFVPELSIDKDTPITTPNIPLLTLTSLSPKLGYLLSKTISVVVTAAQFKPFINV

TAEQLAFGYDDALVSLAHRFYPKHMRPMERMGLLLGRNGTLTEVSSVKTGMDSMDQFGYI<br>DQLNGLDHLPHWSEPPCTSIAGSEGSFFPPRELTKSEVVHIYDKDLCRIIPLKYVESLEK<br>DGIAADLFRLPNNSYGDSAHNPENKCYDTSEYEPIQGLQNISPCQYGAPVYISNPHFFES<br>HPDLLNSVEGLKPEREKHETYFKIQPKLGVPLEGKVRIQLNLKVTRAKDVYPVRDFRDFV<br>FPVMWLEEGISELTPAIKRWIYLGTVIAPSAVPIGSYLMILGGAFAIIFSFVRAYQNFMF<br>AQDPTLEILEMGRRSLRRGSSFIAHQQHRLLVHHRDSYSLLRHGPMATCLGEGNREEDAQ<br>PIIDVSLSGGISQES
>DmR9PY22<br>MPWLDYFILKNLRLWNDTLSYHYWQRPGVIRLTKLYIYNVTNPDGFLRGEKPHLQEVGPF<br>VYREDMQKVNVKFHENNYTVSYQHKKILQFVPELSIDKDTPITTPNIPLLTLTSLSPKLG<br>YLLSKTISVVVTAAQFKPFINVTAEQLAFGYDDALVSLAHRFYPKHMRPMERMGLLLGRN<br>GTLTEVSSVKTGMDSMDQFGYIDQLNGLDHLPHWSEPPCTSIAGSEGSFFPPRELTKSEV<br>VHIYDKDLCRIIPLKYVESLEKDGIAADLFRLPNNSYGDSAHNPENKCYDTSEYEPIQGL<br>QNISPCQYGAPVYISNPHFFESHPDLLNSVEGLKPEREKHETYFKIQPKLGVPLEGKVRI<br>QLNLKVTRAKDVYPVRDFRDFVFPVMWLEEGISELTPAIKRWIYLGTVIAPSAVPIGSYL<br>MILGGAFAIIFSFVRAYQNFMFAQDPTLEILEMGRRSLRRGSSFIAHQQHRLLVHHRDSY<br>SLLRHGPMATCLGEGNREEDAQPIIDVSLSGGISQES
>DmSNMP2<br>MIHWSLIVSALGVCVAVLGGYCGWILFPNMVHKKVEQSVVIQDGSEQFKRFVNLPQPLNF<br>KVYIFNVTNSDRIQQGAIPIVEEIGPYVYKQFRQKKVKHFSRDGSKISYVQNVHFDFDAV<br>ASAPYTQDDRIVALNMHMNSVLQISENDPGLALLLVHLNANLKAVFNDPRSMFVSTSVRE<br>YLFDGVRFCINPQGIAKAICNQIKESGSKTIREKSDGSLAFSFFGHKNGSGHEVYEVHTG<br>KGDPMRVLEIQKLDDSHNLQVWLNASSEGETSVCNQINGTDASAYPPFRQRGDSMYIFSA<br>DICRSVQLFYQTDIQYQGIPGYRYSIGENFINDIGPEHDNECFCVDKLANVIKRKNGCLY<br>AGALDLTTCLDAPVILTLPHMLGASNEYRKMIRGLKPDAKKHQTFVDVQSLTGTPLQGGK<br>RVQFNMFLKSINRIGITENLPTVLMPAIWVEEGIQLNGEMVAFFKKKLINTLKTLNIVHW<br>ATLCGGIGVAVACLIYYIYQRGRVVEPPVK
>DmQ961K6<br>MSHTKDAEMSMPARQESDRTSAPSVTKSLTLNSTRSGVGTGNFGGYGMTNGAEKPSVLHM<br>IMEALGLRNQAQNREPTSKDIGTLIMLGVMFLLFVISVTGFFVMWFTEYYNNTILENLIL<br>AENSDTANSWLNPDPKYDTLLKAHIFHYPNIDDYLAGRADKIKVVDVGPLTYQEHTVKDE<br>VSFNKNFTVSFRDRKSYKFLPEKSSIGEHEVVRVPNVPLISAAGPVKRMKAFERLAVSTW<br>IKQFKEPLFKNLTVSEYLWGYEDSIIKLKSLGRGRRRFGLLMSRNGTSVDSVQLNTGEDD<br>ITKFSIITQFNGMPQLDYWEGDECNRIDGSEPSMFSPHLLQDRSTVNVFLQVLCRKVPLH<br>FEKEVTIYNDIDVLRYRTPMDVFSHPSKNPANQCYCQNTELCLPSGVINATKCYGDAPIF<br>PSFPHFFTGDPVLYQDFEGIEPDAAVHQTYADIHPRFGFPISGASRVQINIMLDKTPILR<br>NQGDRLKNTTILPLMWIEITSGDFSEEVLHTLYLSTFGLNAIQQTLKYGTLLISVTSFSL<br>IVAGVYYLNSRREQQLQESKTSAELEALNGGENVIVVHHIDMPVPLPVQDGGIHQGT
>DmQ8INY3<br>MCCSCCGVTQQKAWVFGLGTFFAVSGLLAIICWPGFIDSGIMKALPLTPTSKTFEKWEEL<br>PIPVYVYMYLWNWTNAADVQANGVKPIFAQLGPYVYREERKKMDLEWHDNGTVTFNPRRT<br>WFWEEELSGGKQTDLITAPHLPSLAASNQMRNSNVFLKFMFNEALNANGGHLFVTHTAAE<br>WLFESFYDEFLHYAMTLNNPLVPKIESDHFAWFLNRNGSKDFEGPFTVHTGVGDIKEMGE<br>IKYWKGQNHTGWYDGECGRLNGSTTDLFVPDEPKEKALTIFIPDTCRIINLEFTGQSETI

QGITGWKYEITPNTFDNGQINGNAKCWCPLERQPDNCPATGATDLGPCADGVPMYLSADH
FMYADESYGSTISGYNPNYDQNNFYIIMERKMGVPLKVNANVMITLYIEPDSVIDILKGL
PSFYAPLFTTASRAEIDEALASELRLALNLPAIGRYTGVGFLCLGCILIAVGILLTIKRK
WYGQAELDKLALKDNEETPYEPEVPANVVERNF

>DmQ9VLU7
MIGKQRIWQTSVLGLLLASFSLFSVLYMEQIERWMLEWFMVLRPGTMISSLWQSPAMDIN
VDLYIFNWTNSEKFSDPTVKPRFEELGPYRFTERMQKVNVEWHDENSTVSYRRRSRFDFD
PKLSVGRPTDPIVAPNLLIVGLYQKMVMWSPMLRSLMLLALNIYGKEQAMIRPASDWMFD
GFDTPMIKMSKMVPPSLVPEMKFPYEKIGYAYPRNGSMEIYGHHNVYTGRDEFQKLGQIA
RWRYNNVTEASPRCKLKGSAGEFHPIPLVKGRPISYFLPDLCRELQVDYSGTTIFEGIDA
FVYRGSARNMANGTDNPDNSCYCQDNCQEVRSGLLNISSCWYGTPVFASYPHFFKADPYY
GEQVEGMKPDKDRHEMVIMLEPKTGMVLEIKARIMANLLVEPKTHLIYRTARRTFFPLIW
ADYNVRITDDLLVYVKLMPILEFVGKICGILGVVLGLLMVFWYPHQMIWQKSLMHKIEIS
SIETNSSLEPVKNNDVEDSPLLKGLQYAAAKDGAGNGN

>DmH1UUH0
MIHWSLIVSALGVCVAVLGGYCGWILFPNMVHKKVEQSVVIQDGSEQFKRFVNLPQPLNF
KVYIFNVTNSDRIQQGAIPIVEEIGPYVYKQFRQKKVKHFSRDGSKISYVQNVHFDFDAV
ASAPYTQDDRIVALNMHMNAFLQVFEREITDIFQGFANRLNSRLNQTPGVRVLKRLMERI
RGKRKSVLQISENDPGLALLLVHLNANLKAVFNDPRSMFVSTSVREYLFDGVRFCINPQG
IAKAICNQIKESGSKTIREKSDGSLAFSFFGHKNGSGHEVYEVHTGKGDPMRVLEIQKLD
DSHNLQVWLNASSEGETSVCNQINGTDASAYPPFRQRGDSMYIFSADICRSVQLFYQTDI
QYQGIPGYRYSIGENFINDIGPEHDNECFCVDKLANVIKRKNGCLYAGALDLTTCLDAPV
ILTLPHMLGASNEYRKMIRGLKPDAKKHQTFVDVQSLTGTPLQGGKRVQFNMFLKSINRI
GITENLPTVLMPAIWVEEGIQLNGEMVAFFKKKLINTLKTLNIVHWATLCGGIGVAVACL
IYYIYQRGRVVEPPVK

>DmQ9VJA7
MRCGCCSVRQQKIWVFSMGGLILAIGIFLASAWPAVSRQWIRSLLPLAPNSFIYNRWVTT
PTPVYSTVFLFNWTNPGDLNTEGVKPHFEQLGPYTFSDFKVKEDLEWNQPQVTYFGKRTW
HFLPEKSNGSLDDVVIAPHFPTLTAALYSRRLRRILRKVMNFTLNREGGATHMTHTAGQW
LFDGYYDHLLDFVEQLHSPLLPVYDNTFGWFYERNNSKTAEGNFTLHTGRGDLSQLGDLL
LWNGENTTGFYPGECGKVNGSTGELWSPYRTWDQPTTIFLPDAARYLNLFPKENLTIDGI
DVWRYESTNLTIDNGQLSPDTECFCLKNRECPRNGVLDYGPPAFNGPFYMSHPHFFLTDQ
MYRENTTGLQPEESEHGMYVIMEPTLGIPLSLRGQLMISTKVVRDEAIDLFKNVAYDHYA
PLFTMRLHAELDEGLTKLLKLALNAAIIGMYTGIGLVLLGMSIVVTGIFITKKHKWHSEQ
RPDKHLQCTQN

>DmM9MRL5
MCCSCCGVTQQKAWVFGLGTFFAVSGLLAIICWPGFIDSGIMKALPLTPTSKTFEKWEEL
PIPVYVYMYLWNWTNAADVQANGVKPIFAQLGPYVYREERKKMDLEWHDNGTVTFNPRRT
WFWEEELSGGKQTDLITAPHLPSLAASNQMRNSNVFLKFMFNEALNANGGHLFVTHTAAE
WLFESFYDEFLHYAMTLNNPLVPKIESDHFAWFLNRNGSKDFEGPFTVHTGVGDIKEMGE
IKYWKGQNHTGWYDGECGRLNGSTTDLFVPDEPKEKALTIFIPDTCRIINLEFTGQSETI
QGITGWKYEITPNTFDNGQINGNAKCWCPLERQPDNCPATGATDLGPCADGVPMYLSADH
FMYADESYGSTISGYNPNYDQNNFYIIMERKMGVPLKVNANVMITLYIEPDSVIE

>DmQ8SYC3
MKEFSKMFARRYSSVSRDVEASNQRQSAVTPGSGLSTDADPAVSCAAQVALQRIQRRKSL
KANRSMNPIDILSSFFNEPIQGKRNRVPTRLFILLVIGFLAIITSTLIKVLEPYDLIFKW
KLIMTEGGEIFNLWAQPPVDLYIKIYLFNITNANAFLAGREQLRVEQVGPYVYKEIMTHE
NVTFNSNNTMSSTPSHPLVWQEEMSGNCREDDEVVMLNIAMLAISHLTANQPFLVRMALK
TLLLSTKSEPIVRTTAKEFMFGYPSALATLGNTFLPNWISFEKVGLIDRMYDFSTDFETF
YTGVPNPALSGLYASYRGETTLPQWDGDHCSNIEFASDGTKFKSFIQPNETVKFFRKSMC
RPINLYRVGNEKTYGSLKGYNYVFEDNAFDNGATNEANKCFCRKGDCQPVGLIDVTDCYY
GFPISLSFPHFMNGDVGLQQNVTGISPDPDKHSSTFVIQPESGLPLSLSVKVQINMHFKD
LSNFPVVSVFNHLTVPMLWFEIMMSKLPDSLDSRFNFYLNILPLVNPLGFWSGILLGISL
LGYAITKATLHMSTFSRRAANITDVKYVRANLIQNIAGEAANHAYSPCDIKLLDDISVVR
HPVNKQTVQDSGFSSKTTYALDMEPALLDTSESSDEGRDCDIVTISRNSTSTRFCADEKK
CWLGDDIRVECFTESRAPQTILKREEQIH

>DmKGL9
MQVPRVKLLMGSGAMFVFAIIYGWVIFPKILKFMISKQVTLKPGSDVRELWSNTPFPLHF
YIYVFNVTNPDEVSEGAKPRLQEVGPFVFDEWKDKYDLEDDVVEDTVSFTMRNTFIFNPK
ESLPLTGEEEIILPHPIMLPGGISVQREKAAMMELVSKGLSIVFPDAKAFLKAKFMDLFF
RGINVDCSSEEFSAKALCTVFYTGEIKQAKQVNQTHFLFSFMGQANHSDSGRFTVCRGVK
NNKKLGKVVKFADEPEQDIWPDGECNTFVGTDSTVFAPGLKKEDGLWAFTPDLCRSLGAY
YQHKSSYHGMPSMRYTLDLGDIRADEKLHCFCEDPEDLDTCPPKGTMNLAACVGGPLMAS
MPHFYLGDPKLVADVDGLNPNEKDHAVYIDFELMSGTPFQAAKRLQFNLDMEPVEGIEPM
KNLPKLILPMFWVEEGVQLNKTYTNLVKYTLFLGLKINSVLRWSLITFSLVGLMFSAYLF
YHKSDSLDINSILKDNNKVDDVASTKEPLPSANPKQSSTVHPVQLPNTLIPGTNPATNPA
THHKMEHRERY

>DmQ9VET1
MKLFTNKNQRNAKLLRSAALGMTLLFFGSLVIISDPLQSILDTQLSLKPGTLLHRLWLLP
PLDVYINVYMFNYTNVDEFTSGRASKLQLQEVGPYVYKEVLSNHNVTYNESNNTITYSPK
REYVFAPERSVGDPKIDRIRAPNIPLMGVTTLASSLSMFAALGLSAITRQLNSQPMLEMS
VHDYMWGYEDHLVELASRFVPNWIDFSSFGIMEKLFREGNESNVFNMNLPEPKDKYGMRM
TDAPRGYTVDSINGERGFKGWQYNEETNGTMCNRIWGSHDATLFPLDMDENDEFFLYRRT
FCRRLPVKFNRTTTFNGLDAFEFVMEPDSFDSEVDNANSSCYCKNNRCLKKGVGNVSPCY
YNIPLAITYPHFMHADPSLLEPFDGLQPNVSRFTSTFVVQPQLGAPMQGTHLRLQANQVV
GKVNFNRMMTPFENMILPLLWVDLNIDVLCLTLRLLIHGIKWGFPLLQWSAALGMLLAGV
YQLCSALLLCFWPTARQFQKVDQVERGTAVQVIGVGLGLATGLRKPSIHLDPEEQHHLLL
GGGSFIPKMAKT

>DmQ9W0X1
MKSVQWVKILLCLILACVNVFLFVVSWGVDYRILVAREHIRFRQEMPTMDSWINSPFGKL
KNYVFNVTNAEEFRSGRDSRLKVKEIGPIVYRIVGFNDILDRNETNVRYRKHRYRVVEFL
PEESVAPDVLNWTITSTNNVILGAATKVKHTAPLAAFGFDAALMMEDIFVTDSVYYFLWE
FTRPLLQTLSRISNIRPNVAVLYNALKEKEEVYTVNIGPKRGIENFFRIETLNGEVIIRE
QLPHTRQYDSNSCPFNVSGALDNSLFPPFVQPDTPLSIVAIESCRVLPLTYQRQERYNGL
DTFRYTLLQSHQKPPGCLDTSYGVKLPDGMFDVSQCVINDAPSAFSMPHFYGSSYNWSQH
YEGYTPNAEDHEPYILLEPVTGIPVTEKYRFQSNIPIPDLRRFSSRLSRFSNMMIPSFWY
EFEMGQLPGFVTSLMWINVNIVQHIQPYCMVLFLVLALWSVLKAIRVACGDLGYVVLFRK

LCGDLGTVTALETKFQATSASPDVAVK
>TrpXAB4<br>MTTNVRNGKQKETPQTPGKCCGCSSKVCSLLLLILSFVMTLTAVLIHTQFDSFFDRALRD<br>QMHLGPSGLVFPAWRVSDLRMRMRVYFFNITNPAEVQLGDKPVLTELGPYTWRIYMEKFD<br>INFYKNNTLSYREKKWYEFLREESVGGYDDIITTVNVPYAAVAQRLKDSGTVTKSTATLT<br>LSGLGQTIMHAKRVGELTFEGYPDILILVANAVETGRKEESGFGSFLGAGLRIFTNVLAA<br>EPEVQGNFGYFVDRNNTDDGVMTIFTGEDDRQKINRVNVVNGQTELSTWPEQKCNKIRGT<br>MGHIRPPMSPPSSPVVIFVPDICRTLPLHFENTSDYEGLTTLRFVAGSDTFNNSGPDPCF<br>AGPRKLKNGLMDIAPCKKGAPLVISFPHYLYADEEHLHSVIGLNPRKEAHQFYLDSDPLT<br>GVTVSVRARFQVGVVMERVHGLGNLGTMFVGNIPLFWQELQLEALPPTVFQLSLLRNLPQ<br>YAKHAALGLVGLAILTGLVAVILAYRDWRYGRSSATKVISITPDHHPIERTYHNHVYENE<br>DEVVKNGRAGAIQDKEAQIQELEKRIRSEGHVGRSKAPPS
>TrpXVQ6<br>MVYGRSHVVLICAGIAVAAFGVTGLLTLKGLFNALLSRRLPLEPGSESYDGWKKIPFPIY<br>QRFYYFNVTNPDQFLRSGEKPILKEIGPYTWRSEWVKEAVEWNANGTLQYRERKRFWFDR<br>NNSVGDQDDVIVTINTPLVAASQKIRNASPLIKLAISIFLNAANETLFIKRSVRQLTYEG<br>YPDVLAQISHLIDPNVPVKDGRFAYMTGKNNTDEGLFNVFTGRDDLQRFNRIEKWNGKDE<br>LPWWESGTPCAKLLGTNGELVHPISSTDEHIHFFNPVFCKSWRLTRGPDVDSLGITLTRF<br>IAGPEILYNSSKAPSHRCFEKPGISLPSGGMYLSRCQFGLPLVLSYPHFYAADPVYLEHI<br>EGLSPDESRHQFYTDVEPRLGITLGLSARAQINVKLERVDFLKYFRHVPELVLPVFWQEI<br>KIEQTPAFAEHINELLERPLFYARLSYGFMIACGVMTILGTLTYVLVLYGRQHK
>TrpX7A6<br>LRVDIFQNLVIDPSNEVYQSWQKNPVPIHVKMYLFNYTNPKEVIQGAKPKLKQLGPFVYR<br>EHREKVNITFNGNGTVSYRQVLSYEYLQEYSVGSLDDKIYTLNVPMIGAAYKNRKSPPGE<br>EEPMANAMEDIFKGMNQSLLVHRAVRELLFEGYEDQMLLLAKQFSWSPTTRFGYQFGRNN<br>SNDGLYTIFTGADEMANYGTVEHWEGRERVKGFRKSCSFVNGTTGEMWPPYTVTAEQPLV<br>FFVSALCRSLRLDFLRNETVKRIQVLRFHLSERLFDYTIEENQCYCSKKKGKDLECFPNG<br>VLDINKCQPEAPLVVSLPHLLYTSPAISEAVEGLSPNADLHEFFMDVEPTMGIPLRVSAR<br>LQMNVIIDSFKYFKQFEVFKKRAFLPTFWLETTATINDDLAFKIRLVTQDLEGYVICASF<br>FWVVLGLIVIIVTLGYVIAHTRKSRLEQPSLRSYTNVTMVPANR
>TrpXVL0<br>MKLTGGKRRPTALLEYVGMGFTAGRTAGIICVMLGACLVVLGAAVLVNFTATFRFILGKK<br>VVLAPTSQGFPIWRDVSQHLDTRVAWYFFNLTNGKEFLDGKKPVLKEVGPFWFRTTISKK<br>NIHFSDNKTVTFEEHRSFQFDLINSVLPYDTRITMINVPVMTALQKLMGLSRVTRLLADS<br>AILSLIGDQNLLTNYTVAELTYEGQYNKLVAISKIKEGVMEAISALSSEDDKKGKFGFAV<br>DKNETHPAIFNMYTGASGVMDLNRIHSVNNQHVLSIWPTQDMHENNACNIVDGTFGYLRP<br>PMNESNEQKVYIPDMCRAVTLHYEKDVSFHGIRLRRFTLGKENFYNRKQEPKNACYDEQF<br>REPSGVSEVGPCKQGAPVLMSMPHFMYANPKFSKMVEGMNPNVEQHSFIMDHEPTTGMTV<br>SVRGRLQANFRVIPLDMMEGGNIPNALMPLFWQEMYVEAGPGLVAFLHKVTGYPSLAKIA<br>LIIVLLIGFIVSIIGAGFLIK
>TrpXA72<br>MKSRGITTIGICALFALVAFLSVTAFIKVPALLEQFVLSRSGISRDSLAEYLFSVIPFDI<br>RQNVYMLNITNPDEFFKGAKPHFQQLGPYVFRVNLRKNMTWSDGDLLTYRENRKFWYDAA<br>LSNGHLNDSINTVDPVYTAAQEVIDELPLIVQKFVRPLLESRKVLFKHTVRELLYDGYTD

PLAELAHLIKPDLPVVNGKIGYLRSMNDTDDGDMTVFGGTNPSTRDKRNLVIEWRHKKTI<br>PYYSGGCNRIWGANAELYPSFNLSSPPEKIGLFLPLFCRPWSLHYNGTRWENGLKLARFG<br>TGLDIFSSSNSTGIKECAQPGSWPQGLFDVRACQHDFPALLSLPHFLHADDKILQDVDGL<br>RPDVTKHDLHLDVFPLLGIPVRPAVRVQLNVRIWRHLDLSRSGNPSIVYPVLWQEIVLSE<br>QSVSYIASMIWYVIQLPQILLQVIAFILFFVSVCFIIIHCYAASQKASGDDTVSIMRSTD<br>DMAAIG
>TrpWZC0<br>MNGEKPAVREIGPYVYLQKRRKFITHIDKNVVTYKAYKSYHFVPEYSNGIPDDKLYALNV<br>PMVAMDKLVGAKLPGDLAKSLLQPILEGLIDKHQEKLVVERKVSEMLFDGYPIPMLTEVS<br>KLAAAFMPDAKLPAADRFGLFYNTNDTADQLFTVGTGAGDYPFATILKWNNLTSLNFWGS<br>EYCNKINGTDGAQFSPFIETNQKLFVFATDLCRSIYFEFEKPTQVKGVDTKRFTVPDAVF<br>ASATQRAENRCFCEGSGRCHEGGVIPLSSCRRGAPIMLSAPHFYQGDAKLVQDVEGLRPE<br>KKNHETFLDVQTMTGLVLRAAKRLQINIDLKQSDLLYPLANLTSRVMPIAWVEERMEATR<br>SMTEALTQRLIQPQKMGVMASSAAVLGGAIGAIAALLVIVVRQANTSRSAKLQPSK
>TrpXN95<br>MVSYEHLPHLSVGSLDDKVYTLNVPMIGSAYKNRKNSPEEELLMANAMEEIFEKYNQTIL<br>VHRKVRELLFEGYEDQLLVIAKGMGWSPVQRFGYQIGRNNSDDGVYTIYTGAEGMDKYGI<br>IESWEGKKRVPGFRKSCGFVNGTTGEMWPPYTLTNQTRLIFFVAPICRSLQLDFLRNETV<br>KEIRVLRYHLNQRLFNYSFEENQCFCTKKKGKDLECVPNGMLDLNRCQPDAPLTASLPHQ<br>LYSTPTVSKAVDGLAPDPELHDFFMDVEPTMGIPLRVSARLQMNVVVDSFRYFEQFAVFK<br>EKLFLPTFWIETTATVNDELAFKIRLVTEDLANYVTLASFAWVLMGLIIIICTLGYIISH<br>TRRSRRPRPSSEYRAVKIIATTR
>TrpXQP1<br>MVSIKLKVALQVLAVVLSVFSLGLIVLSSSASSLVSRIAKGQANLESEDVLENFISPPVP<br>VYQRIYMFNLTNSKEVRKGAKPSVDEIGPYVFRVDMLKKNVTLRDGILSFKQYKAYHFVP<br>ELSGGLSLQDQIITVNPILHFFFLTVDSFDNNTHLRRPLFFKKTVDELLYNGFEDDILDN<br>LEDTDIPTYEGRVGYLVGTNNTIEGAYEVFSGSDEDRGKLNNIISFFGRRHFTYYEEPCN<br>EVEGSNGELYPPFSAAPESIALFNPQLCRPWALHFNGSVSKNGLQLARFVAKDDFLSATG<br>NATVDECLDLHDGADWKALFNAAPCHNSIPVWLSLPHFLKADATLLSDVVGLSPDPKKHE<br>VYLDVYPKVGFTVDMRIRVQVNLKLKRGPKDGQTPEVIYPILWQEIMVYPEGSAKIARIL<br>YEKVELPRALLTRISVVLAVLCFIAALVCVGIRLRLSDTSVSLDAVTGPYPPNWKLLGME<br>FSSREPSNAETGRELVIKTFRTEKLCH
>TrpXHA3<br>MYALRRAAKDAIIKPDSQAAGDFTAERVFELRCYLFNLTNEGSIGSVETPPELVQRGPYV<br>FRVVRKRLAVFEDSGLMRYSDTETFYFDPSKSKGTLRDEILTPDPLVALLTYEKATSNSV<br>KLSGLKRLRKRKTFTVRTVGQLLFDGYNDDFLTKRDSSTSALKLPEKLGYFYGRNGSTSK<br>WFTIETGANDSSEVGTIRLINGKKQLPFYRAPCNVIEGTNGMHGPPFSPWDPPYFVKIFI<br>QTLCRPWRLQLAHKRLNFYGLTVAQFHADREIFGWTGNQKYDSCLQPNPTGFWGTFDATP<br>CSHNLPIVLSLPHFLHGTEMLGKLIRGLSPDDNLHRLYLNIYPILLGGFLQATGVTVDAA<br>IRVQLNLPLNLIKYEKSHVIYPVLWHETLIPETTKEQIVALLSFEVQPFLDVYPLAALIV<br>GLCYFMVGGLVWYIWKRVQQTNATSIDKTVMGLDGETLDSPNAGREKYFVQCKTVAQPGK<br>GLEESQTRDLTDSIATTTRTFVKSGSKRPGPVTHKGAIARVTPVAKKPVPTPEKRTNIPM<br>PSPQQRSPQKLSPQKLSPQKLSPQKPSPQKPSPQQSSRQQSSRQQSSRQQSTRQQSSRQQ<br>SSSQQSSRQQSSPQQHRSQLARLLQLLQQSKPWISRGNTRSEDRTTHDEHFEEGWGSYDD

YTPPIDQRNRSRRVEDESLIDHDSILTPSDIEDSFERPKSH

>TrpXPJ7
YPDSLAEIAHLIEPKLPIIDGKVGFLLGKNATNDGLYSVFSGANNHLDLLNVITEFQNST
TIPYYDEPCNEIRGTNGELYPPFFDAPPESIEIFSPVMCRPWPLHRNGSTTVRGINSVVF
SSGEDLFTKTNNETMDRCFEKMPYMNGVKGTFDASACQFGQPIVITLPHFFKADPELHKL
VTGLEPDARKHDFSLSVFEFGIVTDLQVRLQVNIPVRVRPGANTGPLILYPIWWQELIFL
EQDRNKLSDIIWDQAVRPKVLIQSFLIGLGLLLIATFIIVAITFYIIDRKGKTPDDDELL
RDEERVERRFEVSDRLSR

>TrpXQK1
MELTPVRIAVFFSIALFVAICFGAYPLVPDVIRAIARTRLALVPGSATADQSKALPVNIT
QRVYMFNITNYSAFMDKGAVPNVRQLGPYTFASEWVKNLSWNGDQLLFRENRTFYFLPDL
SNGTLDDEIVTVDLVVVGAIQMVDKLPERIQKFIRPLLDLRPILCKHKVREFLYEG

>TrpXQS8
LQNLVIDPSNEVYESWQEPPIDIYVKLYLFNYTNPEKMQAGLKPKVEELGPFVYRWLPC

>IscPC41
MWPSVQKLPLVNGSEAFELWRDIPLPAFQRVYFFNLTNPYEFLQEGKKPKLQEVGPYTFG
VSMVKTNIVWNPNHTVSYREVRTFHFDREKSVGGQDDVIVSINAPLVGAGALLKRANPAL
RLVMAVVINKLNEQLIVNHTVGELLYDGYPDFLAAASHMLDPTIPTSDGKFGYMHGRNAT
DDGLYTVYTGEDQMDLYNIITRWNGKENLTAWKGTCNMINGTSSFPQGSFKLVREGTDSL
YGISAVRFRVDNRTFDNGTTYPPNACFDTKRTMASGAVDIGPCQHNLPAALSFPHFYLAD
PSYSDKVEGMKPDPDRHSFTLDMEPDDDSSLLINDDKVDEPSDKENGIIHG

>IscQ461
MNCIKERISLCVISLLSVVLGMLTLLGFDNIFRLILNKVIRRISLCVISLLSVVLGMLTL
LGFDNIFRLILNKEVSLAEHSRAFPMWRDVGHDTLVRFYFFNTTAQLLKEQNFIIRGIAS
LTLSGLGQRIFISRTVGQLTFGGYPDILILLGSVVDSGRPRPGQPGFNIQDVFRNGYRAV
TKVLGSLVDPNQQGTNGKFGYMINKNDTVDGEYTIFTGENDISQVNKVYEFNKQRSIPTE
NMGYEDFMGLKVKRFVAGPLAFDSGQKFRENSCFAAGKELPEGGTDLGPCKQGAPLVLSF
PHFLYADPAYLADVDGLHPDVTKHQFFFNSEPTLGVTVNVRGRIQYVEARGDTLKMLRTI
VNLPEYVQWFSVGIIIVSSIALASTAFALFRPRTRRGDITKVRPINGASKAKYADTHCAD
ILPNKVERIIQNGLERLPNTFGITAARNNFINELKSGNNNNPRRPSEPSAPPEVNIYKS

>IscQI62
SAAHTVRFDTEEFEFFKEAVSEIFVAKNSTFFVRRTMRELLFDGYDEPILTAAKSLGIEV
PSTKFGWLLGKNGTDDGEYTIFTGSKGMHNYGEMDKYNGLRSIRLSYLGDVETRGVKLRR
YWGEGRLFDYAEKDNRCFCTGACFPSGVLNISACQQGAPVAVSFPHFLYGDPSYQQAVEG
IAPDPKRHQMYLDIEPLASADKGLLSKVRLVTEEIPQYVTVASFGAVIIGGIVLLATLVY
AIRYTRRPKPVRPTYIPVTVLK

>IscPD82
MINGTDGGQFAPFVSKDSELYVFATDLCRSMRFNYERDTEVHGLKTMRFTPPATLFASAD
EDENNRCFCTTSTCPKSGVVYVSTCQKGAPIVLSSPHFYLGDNEYVNGVAGLSPVKEQHE
TFLDIHPLTGLVLRASKRLQINIDLKNYDKLHKVTVIPDHSHDVSNHLSDGSVE

>IscQ458
MWGGDECNLIRGTFGNVRPPMATTDEQRVFIPDIKRSVLLRYVHDSKVGSVHTRRFAVTP
EVFASGKTRPENACYNKRTLPDGAADMGPVAKGAPVAVSLPHFLYGNQSEFGVEGLTPDE

DKHLLYVDSEPKKS
>IscP9A7<br>MAPVCLLQNLILEPGNEVYAHWQEVPIPIYIKYYFFNVTNPNEVLEQTEKPRLEELGLRE

Table S8. Protein sequences of GPCR used to construct phylogenetic tree.

>DgalGPCR1 D. gallinae-Rhodopsin<br>MCRLSCTYINLFICASNFIVVALTMERFYAVAVPMKARTRGFSGRPNWSIGVCFFLAIVLYLPSV<br>YNYRLMAIEHSIDESTTLPSFRCYVCQWSRFQEQQDGRVYVCIREIFSRVIPVFIVVILNVGMVIS<br>LKHHQKSRAKLSECRASQAIASPDAADSDEDRLKRLLIVVMILFVVCNAPTVFRFLLMRSSGIN<br>ELLPEIFVFCANLLALFNIACNFYVYCFCSTAVRTRVWELLFKVVPGYTTTTSTEKKHTLT
>G. occidentalis-Rhodopsin<br>MNADNISAFFDDWASNFTDNTQSENVTVVPTVIRGSKQIADGICSHFFMGPRSRNVSPAIDWVR<br>SIEFHILLPTIAVLGFVGNAINLIVLRSFTGRTFFYLFWLAVSDLGTMIFDSLFSLSFSIAPTNSLMA<br>FLQCHMVPAGAGISLTCSNFIVVVLTVDRFRAVCYPIETRAKENNSRPRLKVLLCFVSALVVFGP<br>NTYRRRVEAVVFDLGERSTMTTLCYVCQRRRFSNNTLITFYRILLAVFSKIIPVMIVATVNISIVV<br>GLTRHQSERSANLHLGPLRQETAEDRLKKLILTITTIFVICLVPIAFRQLYYRQEPNTLGFAVFKA<br>LSLVMIHLNTACNFYVYCFYSEAIRNQLRKISGHLLNTAKKNLTRTTTILSSTSPSTPSSPGTPDT<br>AISFISFDPSVDSKL
>I. scapularis-secretin<br>MAPTSRHKTCGNADPPRYRTAYACEGSQLHIACDDGHLVQLIRANYGRFSISICNEHGTLDWS<br>VDCMSHRSFRDLPKGTPGGDLVPPLPGTVHAAAPPAVVQADNARTPPPPSPEHVLDIGFPFRTT<br>TTEETTEAPSTEAATQEVSTSLDLLTDFCQPVTVREISWNWTRAGEVAVYPCPGGARGDARW<br>QCGVNPVKWLSERPDLSGCNSFWVGDLERRIHDGVSVVNLASELAQKTRVKVLYGGDLLHT<br>TSIVQQLLSKMEDKLDQALDSYQRQQVVEELMNYVVDFSSNLLEEHQRSSWGDLPPMEQRA<br>AASSLLEGLERNAMLLVKSHSANMNYSRLQSNIMVAVRVLGLQSVTDLSFPDPKMVRGTPW<br>ADLGDSIYLPYKTLLESARNGVVKVAFFSYRQMEAILSAPSPPGGDGVGGNMTRLVNSRVMA<br>AALGRRRVLGLSQPVLVTLRHNKEENVSDPECVFWDFSKRDWSERGCWVESSNQTHTTCAC<br>NHLTNFAVLMDVRAVQLSYSNEVALQVITYIGCFISIICMALTIITFHFFRSLESDRTVIHKNLCT<br>CLLVAEIVFVAGITQTSQRVVCGVVAGLLHYFFLAAFLWMFVEGFQLYVMLIEVFDSEKSRLG<br>WYYVLAYGAPAVVVTVAACVDPTSYGTERYCWLRADNYFILSFVGPVVAILLANLVFLSIAIY<br>MMCRHASLASSVKNKEQSKMANLRIWIRSATVLVFLLGLTWAFGLLYLNAESVVMAYLFTFL<br>NSLQGLFIFVFHCLRNDKVRKEYRQLVRQCPWAPPCVRREDKCPPAERPSYAHSNGTAHSAPS<br>GAALSVRTSCTSTFAHPAPADHIYETIDQEDAFPLSATVGGGCARFVPIDPGECHIRSRSDASHQ<br>SNSSLGCDRRPLIRQRIGGSALSDAGERPVDPALVMAVFDGDRVKCTLQGTAPGRLGGKALVT<br>KHPCQPSAHLSTDC
>S. scabiei-secretin<br>MRLPIVLSQYDTTLPTMSDPTNNDVSVHIPYKVLAQNANDQGQVKVIFLMYRRLSNLLRVRN<br>PIYDFPIHLQQYAYKHSTNERSNDFEYPIYQYQRQQHQFHHPNPLFRSIPQPPINTIINSDIVGLIL<br>PSDLQHSSYDRLPLPLNGMIELTFRHLRTENVTNGRCAYWDMKRIDEDTFGDWSTAGCETVS<br>TNQTHTICRCNHLTNFAVLMDINNISFESNDELVLRLITRIGCSISIIALALTLLTLSVYRSLNSIR<br>NSIHKHLCGCLLVAEVIFLLGIDETRMKLVCGLIAIALHYFFLASFLWMFFEGLQLYTMLVQVF

EHRHNRLTIYSLVAYGAPALLVIVAALVDPLSYGTSQHCWLRTDNHFIWSFLGPALAILLANIL<br>FLSLALSIMCRHTPSSIISTTTFAVHQKQQQQQHLKVYPSTLTPSPSYSITEHSRMHSIRFVNSKFI<br>T
>V. jacobsoni-metabotropic<br>MLFAIDRINEDETLLPGVRLGATILDTCSSDSYALNQSLEFIRASINTADPHNYYCHGGGNLTLK<br>PEASAAISGVIGGSYSEVSLQVANLLRLFRIPQISPASTGTALSDKTRYDFFARTVPPDTFQALA<br>MVAVVETFNWSYVSLVYSEGQYGESGQEQFTKEARHHNICIAISEKIPHSATEQQYLSALERL<br>QQKPNAKVVVLFVRADHAEGVLRAAKELKRKYRDRLQSFDFYWIASDGWGARALSEDMQE<br>AAEGAITIELESRRIEAFDRYMLDLTVENNERNPWFRDYWEALFECKIRNTSSEKEKLPQGISG<br>SNSSLPSCSENLNISSTKDGYKQENKVQFVVDAVYAMAHAIDSVREKVCGAHGVCPRLKEVD<br>GGLIYQALLNVSFVDLVNSTVRFDEKGDAQAPYIIYNYRRNYSSGELQYIQVGKWLGVEEGLE<br>LMAGDITFGRRETHDRVIPVSVCSAECGIGEVKKVQAGDHCCWICTRCEPHEFVVNESTCADC<br>GPSRWPHPHKRSCFDLPVQHMQWNNLFSAVPIAVALLGIVLTCGTIAVFVRNNETPIVKASGR<br>ELSYMLLSGILICYLMTFVLLLKPTPVSCTAQRFGVGLGFSIIYGSLLTKTNRISRIFDSARRSAR<br>RPSFISPKSQMVITCMLISVQVMATLVWFVLEPPATRTEYPEGRRNQVILKCKIRDSSFLVSLVY<br>NMFLITTCTVYAIKTRKIPENFNESKFIGFTMYTTCIIWLAFVPIYFGTGNNFQVQITTLCVSISLS<br>AYVALFCLFSPKVYIILFHPDKNVRKLTMNSATYKKAPTSSTTGGGGGAGGGPPGHNLGSGHG<br>TAVNNQPPTTCTTGTADSCVETVKLVPVATISQHTLQQTSPEMTVAQALRGAPPCSQPTQIEG<br>GRCFQGANTGECVTSDQPRLPGQYSSASRATPSSSPPATGNKSPCDQGSTMMKAVDDRGGGD<br>CDSMQSL
>G. occidentalis-metabotropic<br>MARCPYLNSVPAALLLPGLLLVVVLVTEADTSHHHHRHLGFDEASLYGFDDFPPESRFHKTNN<br>NYHQAPNHVNGVHDTYSTHNTGLNFSWPVKRVAHKKGEVMLGALMMVHERHANWTCGPI<br>MPQGGIQALECMLFTIDYVNRQEWMPSNVSFGYYILDDCDSDTYGLEQAVDFIKGSIGRLHDN<br>DDRITCSQKSSARGSSNSRPSSPATVDVISGVLGAASSVTSIQVANLLRLFRIPQISFFSTSPELSN<br>KQRFEYFLRTVPSDRDQAAAMVELVKLLRWTYISIVYEESNYGIQAFSVLESLLGQQGICIAAK<br>EKLTKDSGQANDKAYDYIVDKLKAKSRARGVIVFGSDQEVAHLMSAVARGNATGMFSWIGS<br>DGWSARSLVSDGHEAQVEGTISVQPMAHPIPEFESYFRNLTAQGNKRNPWFIEYWEYTFKCR<br>WPNETITPYNQAYNRTCSGHEKLSDNTQTGGFELERQLQFVSDAVLAFAYAVRDMHQQLCPG<br>FSGLCRKMKPIEGSQLLSYLKRVTFDGLTSDEFRFAENTLDGPSRYNILHFKQTKPGTYDWVKI<br>GEYRDGQLTLNQTELQFRLEERNTSAEDGSILTGTTPSPPISVCSLPCERGSAKKFLEGEKCCW<br>QCMPCQKYEVLRTETECVECARGTLPDEDHQVCLEIPAQHVNPHSSWATGAMAFASAGSILT<br>LCVIYVFVKYRDTPVVRASGRELSAVLLLGILFCYCVTFVLVQKPTDLFCGLQRTGVGLSFTIV<br>YAAILTKSNRIARIFRAGKRTSRRPSFISPKSQLIICGILVCIQVLVTAVWLLFSPPKAVKHYPTRE<br>QSQLLCQASLGSSYVVSFAYPVMLVCVCTAYAVITRKIPEAFNESKYIGFTMYTTCIIWLSFIPL<br>YLTTSSQLAIGVTVMCVSISLSATVTLVCMFAPKLCIILLHPEKNVRQPVGMIQNNRYGALRSM<br>QRRHVECATQSDDLELGVTVMAKSAVSLASSCAATQTEEEGEEAEETDRLWRQNGIGGDVQL

Table S9. Primer sequences for quantitative RT–PCR.

Genes	Forward primer (5'-3')	Reverse primer (5'-3')	length
DgalOBP1	TGCGTTCTTGAGAGCTA TGG	TTCATCCTTCGGACACTCTG	126 bp
DgalOBP2	GGCTTTGGCTGCTGTAA TCTA	GCAACTTCTCCGCGATACAA	110 bp
DgalGPCR1	TCTGCGCGAGCAACTTT A	AGACTCCGATGCTCCAATTC	120 bp
DgalNPC2-5	TATCGTCGTGCTACCTC TGAT	TCGTCAGAACAGCCTTCAATC	126 bp
Fun14-like (HKG)	TAA CTG GCT CAC CCG AAC TC	CTT TCT CCA CAG CCT TCC AG	208 bp
