# Supplementary material for: Comparative morphological and transcriptomic analyses reveal chemosensory genes in the poultry red mite, Dermanyssus gallinae
Source: Sci Rep. 2020 Oct 21;10:17923. doi: 10.1038/s41598-020-74998-7 (PMC7578799; doi:10.1038/s41598-020-74998-7)
Supplement: Supplementary file 3 — Supplementary Information 3. [file 41598_2020_74998_MOESM3_ESM.pdf]

Gene ID	F1 FPKM	F2 FPKM	F3 FPKM	H1 FPKM	H2 FPKM	H3 FPKM
GAIF01000002.1	29.25	30.59	28.02	92.43	69.33	6.35
GAIF01000003.1	0	0	0	0	0	0.15
GAIF01000004.1	0.07	0	0	12.17	0	0.15
GAIF01000005.1	0.39	0.08	0	0.27	0.93	0.17
GAIF01000006.1	12.77	12.29	26.87	25.94	15.2	28
GAIF01000008.1	0	0	0.14	0	0	0
GAIF01000010.1	0	0	0	0	0.33	0
GAIF01000012.1	77.04	84.34	61.39	74.27	13.38	0
GAIF01000013.1	0.46	1	0.89	2.41	7.63	0.25
GAIF01000015.1	2.08	1.23	0.17	0.75	0.64	0
GAIF01000017.1	0.06	0	0	0.07	0.61	0
GAIF01000018.1	1.12	1.01	0.06	1.03	0.82	0
GAIF01000019.1	0.73	0.56	0.23	0.27	0.1	0
GAIF01000021.1	0	0	0	0.87	1.21	0.28
GAIF01000022.1	0.26	0.38	0.48	1.22	2.54	0.58
GAIF01000024.1	0	0	0.17	0	0	0
GAIF01000025.1	4.99	0.68	0.07	8.36	15.34	1.27
GAIF01000027.1	261.19	447.09	361.6	193.57	389.79	297.1
GAIF01000028.1	1.88	2.26	1.34	1.07	1.03	0
GAIF01000030.1	0.28	0.76	0.19	6.59	16.44	0.07
GAIF01000031.1	0	0.24	0	0	0	0
GAIF01000034.1	0.73	2.14	0.03	0.29	0.04	0.1
GAIF01000036.1	52.91	63.67	109.15	16.3	21.79	130.09
GAIF01000038.1	0	0.21	0	0.9	0.05	0
GAIF01000040.1	7.02	5.1	3.9	4.24	2.69	1.6
GAIF01000042.1	0.2	0.19	0.18	0.35	0.19	0.39
GAIF01000044.1	2.41	0.1	0.1	0.31	2.04	0.1
GAIF01000047.1	11.12	9.97	16.14	23.77	14.7	16.63
GAIF01000048.1	243.69	309.25	960.6	0	0.04	358.97
GAIF01000050.1	0.22	0.89	1.33	55.07	65.75	23.19
GAIF01000051.1	1057.25	843.44	407.93	175.08	61.51	5.25
GAIF01000052.1	0	0	2.67	0	0.35	0
GAIF01000053.1	8.82	7.01	2.77	16.22	22.21	0.94
GAIF01000054.1	0.54	0	0	1.63	10.5	0.12
GAIF01000055.1	0	0.38	0.16	0.19	0.69	0
GAIF01000057.1	0.56	0.38	0.15	0.32	1.42	0.15
GAIF01000058.1	0	0.03	0.01	0	0	0
GAIF01000059.1	0.89	1.1	0.28	1.03	0	0
GAIF01000061.1	0	0	0.55	0	0	0
GAIF01000064.1	0	0.05	0	0	0	0
GAIF01000065.1	0.63	0	0	1.11	1.12	0
GAIF01000066.1	1.4	0.34	0.06	3.06	16.55	1.48
GAIF01000067.1	0	0	0	0	0.23	0

GAIF01000068.1	0	0	0	0.07	0	0
GAIF01000069.1	0	0	0	0	0.21	0
GAIF01000070.1	0	6.02	5.94	0	0	0.56
GAIF01000071.1	0.21	0.11	0.06	1.03	3.88	0.11
GAIF01000072.1	0	0	0	0.13	0.1	0
GAIF01000075.1	9.26	0	0	10.19	30.07	18.64
GAIF01000076.1	0.08	0.08	0	0	0	0
GAIF01000077.1	0.13	0.14	0	0.21	1.39	0.66
GAIF01000079.1	36.29	33.36	24.76	86.06	86.9	17.36
GAIF01000081.1	11.79	7.3	8.4	27.86	21.88	72.11
GAIF01000082.1	0.49	0	0	2.68	8.43	0.27
GAIF01000083.1	0.13	0	0.27	0	0	0
GAIF01000084.1	0.31	0	0	0.87	3.85	1.1
GAIF01000086.1	10.3	10.87	14.14	28.04	14.66	6.51
GAIF01000087.1	1.41	4.06	2.61	0.76	1.03	0.17
GAIF01000089.1	0	0	0	0	0.13	0
GAIF01000090.1	27.23	11.55	10.02	82.04	55.33	1.19
GAIF01000092.1	0.06	0	0.05	0.15	2.94	0
GAIF01000094.1	1.65	0.58	0.24	3.58	1.63	3.05
GAIF01000095.1	9.58	14.86	7.29	20.76	13.21	2.68
GAIF01000097.1	32.68	2.79	4.56	1.45	0.25	0.31
GAIF01000098.1	0.32	0.08	0.12	0.54	0.47	0.12
GAIF01000100.1	0.19	0.18	0.37	2.87	4.08	16.7
GAIF01000102.1	7.41	10.64	4.71	11.17	10.48	1.22
GAIF01000103.1	0.33	0	0	0	0	0
GAIF01000104.1	6.72	14.79	8.78	19.14	15.09	2.2
GAIF01000105.1	0.16	0.18	0.54	0	0	0
GAIF01000106.1	0	0	4.61	0	0	0
GAIF01000107.1	3.26	0.72	1.17	1.39	0.72	0.37
GAIF01000108.1	0	0	0	0	0.1	0
GAIF01000109.1	5.29	2.97	5.69	7.89	10.23	3.43
GAIF01000110.1	17.82	10.62	21.52	37.95	30.87	124.34
GAIF01000111.1	0.13	0	0	0	0	0
GAIF01000112.1	14.32	18.78	5.14	36.01	49.78	3.32
GAIF01000113.1	0.06	0.03	0	0.48	2.45	0.14
GAIF01000114.1	0	1.03	2.74	0.1	0	0.66
GAIF01000115.1	8.34	11.83	2.41	24.66	23.38	2.82
GAIF01000116.1	935.35	758.22	587.59	716.25	1011.58	676.03
GAIF01000119.1	0	0	0	0.14	1.2	0
GAIF01000120.1	0	0	0	0.08	0.47	0.04
GAIF01000121.1	10.62	6.76	6.39	14.83	20.44	1.4
GAIF01000122.1	0	0.06	0.12	0	0	0.06
GAIF01000123.1	0	0	0.42	0	0	0
GAIF01000127.1	0	0.17	0	0.27	0.28	0

GAIF01000128.1	0	0.21	0	0.56	1.15	0
GAIF01000129.1	30.25	33.39	20.64	46.68	42.93	12.48
GAIF01000130.1	145.25	110.92	99.79	186.97	99.53	21.78
GAIF01000131.1	6.1	9.58	15.42	8.72	8.09	8
GAIF01000133.1	12	6.64	7.74	27.08	21.34	12.66
GAIF01000134.1	0	0.28	0	0	0	0
GAIF01000135.1	15.49	12.63	10.75	49.2	26.52	3.2
GAIF01000138.1	0	0	0	0	0.13	0
GAIF01000139.1	3.39	5.88	3.46	10.24	8.72	1.06
GAIF01000140.1	0.71	0.36	0	6.27	14.51	0.05
GAIF01000141.1	0.7	0.91	0.77	0.49	0.17	0
GAIF01000142.1	1.19	1.3	1.31	0	0	0
GAIF01000143.1	0	0	0	0	0.5	0.1
GAIF01000144.1	0.09	0.06	0.1	0.72	2.72	0.4
GAIF01000146.1	5.16	0	13.67	1.38	0	12
GAIF01000147.1	0	0.85	0.72	2.86	0	0
GAIF01000148.1	0.33	0.65	0.06	1.27	6.04	0.06
GAIF01000149.1	0	0	0	0	2.26	0
GAIF01000150.1	64.68	110.05	120.58	198.37	184.16	227.93
GAIF01000153.1	5.75	1.06	0.54	19.46	82.87	7.96
GAIF01000154.1	0	0	0	0	0.09	0
GAIF01000155.1	5.74	5.61	2.58	12.52	12	1.64
GAIF01000156.1	0.2	0.21	0.59	0.11	0	0.05
GAIF01000157.1	0.08	0	0.1	0.69	4.29	0.1
GAIF01000158.1	3.13	0.23	5.37	2.35	0.14	1.08
GAIF01000159.1	0	0.09	0	0.1	0.41	0
GAIF01000161.1	0.43	0.31	0.55	1.91	6.29	0.71
GAIF01000164.1	0.05	0.07	0.1	0.08	0.59	0
GAIF01000165.1	0	0	0	0.42	0.63	0.2
GAIF01000166.1	0.13	0.1	0	0.15	0.81	0.14
GAIF01000167.1	14.64	20.85	10.98	38.3	46.1	2.99
GAIF01000168.1	0.16	0	0.09	0.55	0.09	0.69
GAIF01000169.1	2.58	0	0.17	7.89	18.14	3.7
GAIF01000171.1	0	0.06	0	0	0	0
GAIF01000172.1	0.4	1.39	0	3.89	4.48	0.72
GAIF01000173.1	0	0	0	0	0.33	0
GAIF01000174.1	201.39	257.27	422.49	197.62	152.16	541.84
GAIF01000175.1	4098.6	7174.02	3216.73	4940.36	4759.06	1200.15
GAIF01000178.1	3.68	6	0.92	3.17	6.77	0.37
GAIF01000180.1	51.22	47.36	39.35	100.98	41.74	27.24
GAIF01000181.1	0	0	0	0	0	0.03
GAIF01000183.1	0	0	0	0	0.36	0.04
GAIF01000184.1	0	0	0	0.09	0.38	0.26
GAIF01000185.1	19.41	0.08	1.43	3.92	0	0.08

GAIF01000189.1	0	0	0	0.06	0.04	0
GAIF01000191.1	0	0	0	0.24	0.73	0.45
GAIF01000194.1	0	0	0.28	0.17	1.08	0
GAIF01000195.1	0	0	0	0.99	1.28	0
GAIF01000196.1	3.62	4.1	1.21	7.48	7.19	0.17
GAIF01000198.1	11.03	12.93	2.59	31.44	49.19	1.56
GAIF01000199.1	105.55	147.86	33.7	328.38	252.92	14.54
GAIF01000200.1	3.66	0.47	0.48	0	0.07	0.07
GAIF01000201.1	2.42	1.63	1.01	4.82	12.49	8.78
GAIF01000203.1	0	0	0	0.52	1.43	0
GAIF01000204.1	2.2	1.15	4.02	0	0	1.07
GAIF01000205.1	0.28	0	0	1.57	3.3	0.19
GAIF01000206.1	143.59	5.18	0.32	895.89	1847.95	0.97
GAIF01000207.1	0	0.11	0	0	0.12	0
GAIF01000209.1	0	0	0.04	0.13	0.9	0
GAIF01000210.1	0.56	0.44	0.09	0	0.1	0
GAIF01000211.1	0	0	0	0	0.46	0.03
GAIF01000212.1	0	0	0	0	0.15	0
GAIF01000213.1	0	0.28	0	1.19	3.85	0
GAIF01000214.1	54.06	1.99	184.18	7.82	0	1.13
GAIF01000215.1	59.75	57.44	57.16	84.47	45.06	28.4
GAIF01000217.1	22.84	32.23	42.08	52.87	32.29	16.94
GAIF01000218.1	0.07	0.23	0	0.5	0.09	0.87
GAIF01000219.1	0	0	0	0	0	0.06
GAIF01000220.1	12.41	9.79	11.3	15.41	11.55	14.13
GAIF01000221.1	1.67	3.24	1.54	1.33	0	1.07
GAIF01000222.1	0	0	0	0	0	0.07
GAIF01000223.1	0	0.12	0	0	0	0
GAIF01000224.1	368.15	50.53	183.77	233.62	120.56	838
GAIF01000225.1	0	0.17	0	0.18	0.19	8.99
GAIF01000227.1	0.77	2.01	0.57	4.83	4.13	0
GAIF01000229.1	7.87	15.33	4.39	51.91	131.16	2.28
GAIF01000230.1	0.08	0.25	0.09	0.71	3.7	0
GAIF01000231.1	2.4	0.36	0.37	8.73	26.11	102.83
GAIF01000232.1	123.27	139.12	83.9	111.79	79.97	30.43
GAIF01000233.1	0	0.07	0	0	0	0
GAIF01000234.1	3.63	8.26	5.76	8	5.59	1.78
GAIF01000236.1	5.61	2.91	5.13	5.43	2.25	5.74
GAIF01000237.1	0.35	0.23	0.31	0.49	0.25	0.08
GAIF01000239.1	1.36	0	0	4.62	15.66	0.95
GAIF01000240.1	3.88	6.18	7.61	2.73	1.33	3.36
GAIF01000243.1	12.05	8.77	19.1	22.57	19.76	34.61
GAIF01000246.1	162.41	105.97	67.11	155.06	115.73	59.05
GAIF01000247.1	0.82	1.42	3.22	9.94	7.18	0.48

GAIF01000248.1	56.19	78.01	41.56	32.78	53.47	24.74
GAIF01000249.1	1.31	0.61	0.36	6.72	15.86	0.56
GAIF01000250.1	0	0	1.91	0	0.05	1.77
GAIF01000251.1	34.27	0	7.63	118.67	18.87	107
GAIF01000253.1	0.3	0	0	1.74	5.22	0.66
GAIF01000256.1	0.04	0.04	0	0	0	0
GAIF01000259.1	0.85	0.47	0	3.71	3.38	1.08
GAIF01000263.1	46.9	6.08	0	25.76	4.81	1.16
GAIF01000264.1	1.39	3.09	3.2	1.16	1.16	0.52
GAIF01000265.1	10.6	9.22	10.57	3.02	3.36	1.06
GAIF01000266.1	0.56	0	0	1.63	3.73	1.21
GAIF01000267.1	19.17	14.76	21.63	17.61	11.85	15.84
GAIF01000268.1	80.88	22.15	6.62	112.3	86.66	3.38
GAIF01000269.1	0	0	0	0	0.04	0
GAIF01000270.1	0	0.04	0.04	0	0.02	0.08
GAIF01000271.1	131.44	184.1	170.52	129.23	108.16	67.87
GAIF01000275.1	0.19	0.2	0.14	0.72	1.64	0.41
GAIF01000276.1	7.6	2.15	2.33	6.56	0	4.64
GAIF01000277.1	10.28	14.41	25.82	8.86	9.35	1.83
GAIF01000278.1	0.73	0.64	0	1.02	3.69	0.16
GAIF01000279.1	4840.14	4818.91	7417.05	2974.45	2189.64	12806.42
GAIF01000280.1	72.45	48.05	79.72	195.02	68.77	109.23
GAIF01000282.1	0	0	0	0.13	0	0
GAIF01000283.1	1.32	2.78	2.06	0	4.06	4.66
GAIF01000284.1	0.12	0.13	0	0	0	0
GAIF01000285.1	182.65	81.85	71	92.58	229.62	216.23
GAIF01000286.1	0	0	0.17	0.74	2.29	1.75
GAIF01000287.1	267.63	103.85	50.85	232.11	194.3	155.49
GAIF01000289.1	6.78	13.92	8.01	19.69	11.68	2.61
GAIF01000291.1	16.96	8.58	6.93	16.88	17.32	6.25
GAIF01000293.1	621.86	183.83	222.71	517.78	298.07	3345.63
GAIF01000294.1	1.26	2.74	1.56	0.18	0	0
GAIF01000296.1	0	0	0	0	0.27	0
GAIF01000297.1	0	0	0	1.1	7.25	0
GAIF01000298.1	0	0	0	0.55	2.59	0
GAIF01000299.1	12.14	15.94	8.88	50.11	35.83	4.02
GAIF01000300.1	15.64	23.22	20.11	47.02	36.85	11.68
GAIF01000301.1	0	0	0	0.16	0	0
GAIF01000302.1	0	0.18	0	0	0	0
GAIF01000305.1	3	6.35	5.23	9.61	8.91	1.77
GAIF01000306.1	0.74	2.6	1.11	1.07	1	0
GAIF01000307.1	0	0	0	0.07	0	0
GAIF01000308.1	33.19	22.02	24.61	37.92	44.22	32.38
GAIF01000309.1	0	0.09	0.35	0.66	0.52	0

GAIF01000310.1	1.31	0.44	0.47	6.24	17.52	0.75
GAIF01000311.1	14.59	15.55	11.69	11.51	9.47	1.29
GAIF01000313.1	1.3	6.19	7.27	24.9	18.89	1.32
GAIF01000316.1	0	0	0	0	0.08	0
GAIF01000318.1	0	0.04	0	0.16	0.04	0
GAIF01000320.1	0	0	0	0.06	0	0
GAIF01000321.1	290.92	56.7	122.5	495.41	228.74	306.12
GAIF01000322.1	0.89	0.12	0.49	1.41	6.53	0.12
GAIF01000323.1	6.47	11.51	23.47	0	0	0
GAIF01000325.1	38.03	12.61	16.96	61.88	21.15	62.02
GAIF01000326.1	0.03	0.03	0.25	0.26	0.34	0
GAIF01000327.1	1.23	2.57	0.52	2.46	1.86	1.04
GAIF01000328.1	0.41	1.38	0.77	3.73	6.45	1.9
GAIF01000329.1	3.01	0.34	0.34	12.16	33.94	4.92
GAIF01000330.1	0.32	0.35	0	0	1.44	0
GAIF01000333.1	6.61	11.38	4.24	26.33	13.94	0.82
GAIF01000337.1	46.94	56.99	14.71	56.52	30.11	6.13
GAIF01000339.1	8.01	14.67	9.94	9.88	10.73	2.79
GAIF01000340.1	28.97	23.88	21.29	61.55	37.65	25.49
GAIF01000342.1	0	0	0.08	0	0	0
GAIF01000343.1	1.14	0.84	1.28	2.29	2.41	0.86
GAIF01000346.1	0.26	0.1	0	1.57	5.87	0.07
GAIF01000347.1	0	0	0	0.14	0	0
GAIF01000349.1	7.24	3.67	2.57	8.08	12.94	22.19
GAIF01000351.1	0.64	0.38	0.16	0.49	1.95	0.31
GAIF01000352.1	0	0.08	0	0	0	0
GAIF01000353.1	1.38	0.52	0.53	3.56	3.93	1.32
GAIF01000355.1	0	0	0	0.2	0.19	0
GAIF01000356.1	0.51	0.07	0.08	0.23	0	0.32
GAIF01000357.1	0	0	0	0	0.26	0
GAIF01000358.1	0.08	0.04	0.04	0.25	1.7	0.11
GAIF01000359.1	0.88	0.18	0.46	2.66	0.68	0.41
GAIF01000360.1	0	0	0	0.09	0	0
GAIF01000361.1	0	0.17	0	0	0.1	0
GAIF01000362.1	0	0	0	0.37	0.2	0
GAIF01000364.1	0.76	0	0	2.09	10.46	1.04
GAIF01000365.1	0	0.01	0.02	0.08	0.23	0.01
GAIF01000366.1	3.35	0.46	0.08	2.44	10.52	6.04
GAIF01000367.1	0.25	0.09	0	0.59	3.27	0.47
GAIF01000368.1	0	0.04	0.04	0.48	1.41	0.21
GAIF01000369.1	6.26	1.03	0.32	8.77	39.47	32.85
GAIF01000370.1	0.13	0	0	0.16	0.86	0.05
GAIF01000372.1	0	0	0	0.29	0.41	0
GAIF01000374.1	0	1.45	114.77	0	0	0

GAIF01000375.1	0.6	0.24	0.08	4.44	20.65	1.24
GAIF01000376.1	165.62	245.62	139.04	143.88	127.35	36.49
GAIF01000377.1	0.33	0.17	0.17	0.18	0.24	0.14
GAIF01000378.1	0.23	0.62	0.37	0.26	0.14	0.25
GAIF01000380.1	3075.2	2986.63	3022.67	2199.9	1743.99	1477.17
GAIF01000381.1	0	0	0	0	0	0.17
GAIF01000384.1	1.95	0	0.27	1.77	0.73	1.07
GAIF01000385.1	6.92	12.97	5.45	9.2	7.54	2.45
GAIF01000387.1	0.41	0.23	0.98	1.09	0.75	0
GAIF01000389.1	0.36	0	0.89	0	0	0
GAIF01000390.1	6.95	14.76	16.05	22.79	25.31	22.3
GAIF01000392.1	0	0.07	0.33	0	0	0
GAIF01000393.1	0.11	0	0.75	0	0	0.25
GAIF01000394.1	6.37	6.65	15.08	19.14	9.68	16.73
GAIF01000395.1	0	0	0	0	0.16	0
GAIF01000396.1	4.67	0.14	0.09	0.48	0.05	0.14
GAIF01000397.1	0	0	0	0	0.45	0
GAIF01000398.1	0.13	0.05	0	0.37	1.46	0
GAIF01000399.1	0	0	0	0	2.04	0
GAIF01000400.1	0	0	0	0.16	0	0
GAIF01000401.1	49.82	37.57	48.78	121.43	106.02	66.35
GAIF01000402.1	0.06	0.03	0	2.66	13.01	0.08
GAIF01000403.1	1.25	1.77	1.1	4.06	7.82	0.28
GAIF01000404.1	0.77	0.84	0	4.4	17.67	2.54
GAIF01000405.1	1.14	4.42	0	2.66	3.57	0
GAIF01000406.1	10.87	6.2	4.22	17.83	13.3	14.48
GAIF01000407.1	0.7	0	0	0.95	7.73	0
GAIF01000408.1	0	0	0	0.07	0	0
GAIF01000409.1	0	0	0	0.26	2.16	0
GAIF01000410.1	4.02	0.86	0.46	8.4	1.21	0.23
GAIF01000411.1	107.12	133.92	122.26	184.6	151.08	89.87
GAIF01000413.1	0	0.05	0	0	0	0
GAIF01000415.1	0.42	0.31	0.41	0.44	0.28	0
GAIF01000416.1	0	0	0	1.95	0	0
GAIF01000417.1	0	0	0	0	0.31	0
GAIF01000418.1	0.21	0	1.16	1.15	0.25	0.39
GAIF01000419.1	0	0	0	0	0.17	0
GAIF01000420.1	5.64	6.57	4.72	13.56	9.37	0.15
GAIF01000421.1	60.09	101.52	132.93	74.09	66.07	66.22
GAIF01000422.1	0.22	0.2	0.2	0.56	0.89	0.29
GAIF01000427.1	0	0	0	0.18	1.91	0.23
GAIF01000428.1	0	2.41	1.22	0	0	2.08
GAIF01000429.1	0	0	0	0.02	0	0
GAIF01000430.1	1.18	0.37	0.06	3.46	14.34	0.43

GAIF01000431.1	0	0	0	0.07	0.08	0
GAIF01000432.1	0	0.03	0	0	0.03	0
GAIF01000433.1	0	0	0.12	0	0.18	0
GAIF01000434.1	24.11	15.28	20.02	18.33	21.29	11.62
GAIF01000435.1	1.16	0.24	0.2	0	0.97	0
GAIF01000436.1	7.23	9.32	6.51	14.9	8.01	2.04
GAIF01000437.1	0	0	0.23	0.73	5.91	0
GAIF01000439.1	0	0.11	0	0.11	0	0
GAIF01000441.1	0.57	0.1	0.52	1.98	8.66	1.25
GAIF01000442.1	0.01	1.03	1.77	0.28	1.28	0.21
GAIF01000443.1	1.39	0.8	0	0.38	1.08	0.18
GAIF01000445.1	0	0.01	0	0.11	0	0
GAIF01000447.1	7	4.97	1	35.34	21.24	1.01
GAIF01000449.1	0	0.27	0	0.93	3.1	0
GAIF01000451.1	0.81	1.02	0.59	2.34	4.68	0.15
GAIF01000452.1	0	0.03	0.06	0.03	0	0
GAIF01000453.1	1.31	0.1	0	3.26	10.8	1.96
GAIF01000456.1	0	0	0	0.09	0	0
GAIF01000457.1	36.88	87.28	20.44	57.21	80.42	22.65
GAIF01000458.1	0	0	0	0	0.02	0
GAIF01000460.1	6.02	9.81	6.31	16.67	17.1	1.51
GAIF01000461.1	6.39	8.24	5.31	11.23	8.12	0.91
GAIF01000463.1	26.33	27.27	44.31	33.27	26.75	25.5
GAIF01000464.1	0	0	0	0	0.93	0.08
GAIF01000465.1	1.32	1.44	0.32	4.09	17.64	0.16
GAIF01000467.1	4.54	2.62	0.61	15.55	30.05	5.06
GAIF01000469.1	1.39	1.83	0.97	0.85	0	0
GAIF01000471.1	0	0.06	0	0.13	0.39	0
GAIF01000472.1	2.87	3.5	2.51	3.22	2.66	1.75
GAIF01000473.1	0.04	0	0.05	0.15	0.85	0.05
GAIF01000475.1	0.13	0	0	0	0.17	0
GAIF01000476.1	0.31	0	0	1.02	1.8	0
GAIF01000477.1	0	0	0	0.59	2.14	0
GAIF01000478.1	0.48	0.39	0.78	1.66	2.86	0.39
GAIF01000479.1	2420.58	2064.64	2755.17	2458.34	1463.41	3849.35
GAIF01000480.1	2.42	1.25	0	5.91	5.66	0.59
GAIF01000481.1	1.83	0.06	0.31	0.65	2.61	1.35
GAIF01000482.1	10.85	6.76	4.67	37.74	36.85	0.41
GAIF01000483.1	0	0	0	0	0.24	0
GAIF01000484.1	1.32	0.36	0.58	3.26	4	1.45
GAIF01000485.1	0.44	0	0	0.31	0	0
GAIF01000486.1	0.09	0.12	0.05	0.64	2.01	0
GAIF01000487.1	0	0	0	0.13	0	0.04
GAIF01000489.1	0.48	0.1	0	1.88	8.07	0.17

GAIF01000491.1	14.28	13.02	8.18	51.25	29.86	3.32
GAIF01000492.1	2.21	0.17	0.25	6.81	16.2	0.84
GAIF01000493.1	4.26	5.85	3.51	16.4	12.36	1.53
GAIF01000494.1	16.4	13.69	12.49	4.45	4.48	1.32
GAIF01000496.1	30.93	10.95	14.03	48.77	27.04	202
GAIF01000498.1	260.63	102.8	96.45	165.59	85.74	187.33
GAIF01000499.1	58.01	20.7	23.55	58.13	58.08	68.65
GAIF01000500.1	0.04	0.04	0	0	0.05	0
GAIF01000505.1	0.04	0.18	0.09	0.24	0.1	0.09
GAIF01000506.1	42.05	66.69	51	40.14	38.74	8.5
GAIF01000508.1	185.09	339.11	152.76	92.52	128.42	40.01
GAIF01000509.1	63.4	75.92	134.41	104.17	55.8	84.03
GAIF01000510.1	0.41	0	0	0.42	3.03	0.38
GAIF01000511.1	19.41	1.58	0	26.77	38.5	12.84
GAIF01000512.1	0.29	0	0.21	1.35	3.86	0.43
GAIF01000513.1	0.03	0.63	0	0.34	0.61	0.48
GAIF01000514.1	0.32	0.09	0	3.91	21.72	0
GAIF01000517.1	0	0	0	0	0.14	0
GAIF01000519.1	4.53	0.82	0.04	1.22	0.64	0
GAIF01000520.1	0	0	0.47	0.2	0	0.28
GAIF01000521.1	2.67	4.98	2.3	12.96	14.78	1.25
GAIF01000522.1	26.04	0.45	0	18.46	31.98	0
GAIF01000525.1	19.44	18.27	25	21.79	24.22	31.92
GAIF01000526.1	0.05	0	0	0.03	0	0
GAIF01000527.1	18.39	27.02	47.31	36.41	28.53	31.72
GAIF01000528.1	5.13	4.14	3.56	11.59	19.05	12.73
GAIF01000530.1	0	0	0.26	0	0	0
GAIF01000531.1	6.67	12.43	7.11	2.18	1.18	0.06
GAIF01000532.1	0	0.09	0.04	0	0	0.04
GAIF01000533.1	0.22	0.24	0.24	0.26	0.27	0
GAIF01000535.1	2.12	1.39	0	0	0.52	0
GAIF01000538.1	0.32	0.51	0.48	0.43	0.33	0.31
GAIF01000540.1	28.65	31.87	24.3	8.85	5.07	3.34
GAIF01000541.1	0.74	1.08	1.09	0.72	0.4	0.14
GAIF01000542.1	0.17	0.09	0.06	0	0	0
GAIF01000543.1	0	0	0	0	0	1.09
GAIF01000544.1	2.7	0.43	0.26	10.77	59.76	22.03
GAIF01000545.1	0	0	0	0	0.21	0
GAIF01000547.1	11.39	8.72	10.26	12.83	8.16	19.17
GAIF01000550.1	0	0	0	0	0.15	0
GAIF01000551.1	2.73	0.05	0	0.49	0.06	0
GAIF01000553.1	0	0	0	0	0.17	0
GAIF01000554.1	0.03	0	0	0	0	0
GAIF01000555.1	2165.9	1227.07	954.88	1459.25	1316.5	3266.95

GAIF01000557.1	0.02	0.01	0	0.11	0.36	0.03
GAIF01000558.1	0.43	0.81	0.12	2.47	7.04	0
GAIF01000559.1	0	0.1	0	0	0	0
GAIF01000560.1	0	0.06	0	0.37	1.61	0
GAIF01000561.1	0	0.17	0	0.39	0.18	0.11
GAIF01000562.1	0.11	0.04	0.04	0	0	0
GAIF01000563.1	0	0	0	0	0.37	0
GAIF01000566.1	17.68	30.68	17.36	65.86	41.58	6.07
GAIF01000567.1	1.27	0.13	0.06	7.15	28.33	1.27
GAIF01000571.1	6.52	6.81	1.32	12.07	30.11	4.09
GAIF01000572.1	0.76	0.06	0	0.13	3.02	0
GAIF01000573.1	198.23	106.83	68.14	4.18	3.94	19.09
GAIF01000574.1	0	0.89	0	0.18	0	0.9
GAIF01000575.1	0	0	0	0.15	0.16	0
GAIF01000576.1	0.66	0.18	0	0.67	3.9	1.46
GAIF01000577.1	0	0	0	0	0.1	0
GAIF01000579.1	0.08	0	0	0.2	1.16	0
GAIF01000580.1	0.8	1.45	0.29	4.78	8.31	0.15
GAIF01000581.1	7.75	10.19	0.05	0	1.32	0
GAIF01000583.1	2.07	0.87	1.59	2.09	3.87	8.81
GAIF01000584.1	1.05	0.31	0.53	1.75	1.76	0.08
GAIF01000585.1	0	0	0	0	0.15	0
GAIF01000586.1	0.12	0	0	0	0.36	1.03
GAIF01000587.1	0.38	0.56	0.56	1.34	7.86	0.28
GAIF01000588.1	0.03	0.07	0.04	0.08	1.74	0.15
GAIF01000589.1	0	0.11	0	0.67	2.96	0.16
GAIF01000590.1	1.21	1.32	0.81	1.92	3.86	0.1
GAIF01000592.1	3.58	0.51	0	0.84	2.06	0
GAIF01000595.1	1.73	0.8	0.27	8.63	22.38	0
GAIF01000596.1	2.21	0.88	0.5	8.12	17.73	4.49
GAIF01000598.1	12.11	9.13	12.22	19.09	11.95	20.28
GAIF01000599.1	0.34	0	0	0.13	0.97	0
GAIF01000600.1	0	0	0	0.12	0.13	0
GAIF01000601.1	23.66	8.87	9.72	35.68	62.64	47.05
GAIF01000602.1	0	0	0	0	0	1
GAIF01000603.1	18.76	37.72	23.45	53.52	49.82	5.58
GAIF01000604.1	0.36	1.09	0.19	1.42	2.04	0.28
GAIF01000605.1	0	0	0	0	0.15	0
GAIF01000606.1	0	0	0	0	0.08	0
GAIF01000608.1	10.16	8.92	34	6.39	2.3	16.08
GAIF01000610.1	0.16	0.17	0	5.84	17.56	0
GAIF01000611.1	0	0	0	0	1.36	0
GAIF01000612.1	0.17	0	0	0.29	1.72	0.46
GAIF01000613.1	0	0.14	0.21	0.15	0	0.14

GAIF01000616.1	0.33	0.57	0	1.54	6.26	1.06
GAIF01000617.1	0	0.12	0.09	0	0	0
GAIF01000618.1	0	0	0.09	0	0	0.28
GAIF01000619.1	1.43	0.16	0	2.5	8.8	0.47
GAIF01000620.1	1.73	2.2	0.51	3.55	4.17	0.19
GAIF01000621.1	1.68	0.09	0.04	2.68	11.22	1.12
GAIF01000622.1	0	0	0	1.24	6.44	2.95
GAIF01000624.1	16.44	19.53	9.63	13.65	7.74	2.19
GAIF01000628.1	0.03	0.06	0	0	0.03	0
GAIF01000629.1	0.81	0	0	0.93	12.98	0
GAIF01000632.1	6.43	11.17	12.93	36.43	43.59	5.71
GAIF01000633.1	0.26	0.28	0	1.19	4.92	0
GAIF01000634.1	1.28	0.71	0.18	7.95	20.91	0.38
GAIF01000636.1	0	0	0	0.48	2.27	0
GAIF01000637.1	1.57	1.07	0.34	0.2	0.14	0.49
GAIF01000638.1	0.31	0	0	1.99	4.13	5.23
GAIF01000640.1	5.75	0	0	0	0	0
GAIF01000641.1	0	0	0	0	0.1	0
GAIF01000642.1	3.8	3.78	1.8	28.48	42	7.24
GAIF01000643.1	1.33	0	0	0	0	0.27
GAIF01000644.1	0	0	0	0	0.27	0.11
GAIF01000645.1	0.56	0.85	0.17	1.38	3.48	0.13
GAIF01000646.1	0.35	0.71	0	0.27	0.14	0
GAIF01000647.1	1.26	0.51	0.39	2.08	10.11	0.57
GAIF01000649.1	0	0.1	0.2	0	0	0
GAIF01000650.1	23.21	21.5	13.05	43.1	42	3.59
GAIF01000651.1	93.29	77.09	36.89	71.09	51.29	7.18
GAIF01000652.1	0	0	0	0.3	0.94	0.14
GAIF01000653.1	0.04	0.03	0	0.75	2.95	0.02
GAIF01000654.1	0.14	0.16	0	1.09	2.44	0
GAIF01000658.1	4.57	3.69	5.07	1.76	0.34	2.36
GAIF01000659.1	1.77	0.34	0	9.45	22.44	0.23
GAIF01000660.1	28.86	47.94	19.7	54.17	43.1	3.6
GAIF01000662.1	0.04	0.05	0	0.05	0.73	0
GAIF01000663.1	0.9	1.09	2.2	0.7	1.09	0.77
GAIF01000664.1	0.05	0	0	0	0	0
GAIF01000665.1	0.81	0.62	1.51	3.05	5.06	0.62
GAIF01000666.1	0	0.56	0.35	0	0	0.15
GAIF01000667.1	14.97	9.49	19.03	35.75	18.75	45.5
GAIF01000668.1	0	0.51	0.34	1.36	9.94	0.69
GAIF01000669.1	400.66	143.58	87.01	124.83	141.35	133.7
GAIF01000671.1	0.16	0.18	0.53	0.25	0.65	0.71
GAIF01000675.1	17.1	27.55	24.98	19.03	13.4	6.4
GAIF01000676.1	12.41	17.44	8.72	40.01	23.83	2.65

GAIF01000677.1	0.11	0	0	0	0	0
GAIF01000678.1	14.31	8.61	8.8	27.1	16.98	21.26
GAIF01000679.1	0.48	0.36	0.28	0	1.53	0.52
GAIF01000680.1	0	0.04	0	0.09	0.08	0
GAIF01000681.1	0.06	0	0.06	0.07	1.5	0.19
GAIF01000683.1	18.78	19.8	17.97	61.65	80.35	18.81
GAIF01000687.1	0	0.14	0	0.45	1.1	0
GAIF01000688.1	57.12	60.38	54.67	326.35	206.64	82.28
GAIF01000689.1	13.87	18.45	7.34	35.59	67.13	2.09
GAIF01000690.1	0.5	0.54	0.09	4.08	18.27	0.12
GAIF01000691.1	4.02	2.06	0.08	8.89	13.13	9.78
GAIF01000692.1	0	0	0	0.12	1.45	0
GAIF01000693.1	0	0	0	0.05	0	0
GAIF01000694.1	0	0.13	0	0.14	0.15	0
GAIF01000695.1	0	0	0	0.03	0.2	0
GAIF01000696.1	0	0	0	0	0.87	0
GAIF01000698.1	0.78	0.21	0	2.73	0.76	0.48
GAIF01000699.1	31.06	35.05	32.49	2.85	4.22	3.36
GAIF01000701.1	0	0.03	0	0.15	0	0
GAIF01000705.1	0.18	0	0	0	0	0
GAIF01000706.1	38.15	37.11	44.01	44.22	33.41	27.3
GAIF01000707.1	0.01	0	0	0.06	0	0
GAIF01000708.1	4.9	5.61	1.48	0	0	0
GAIF01000709.1	0	0	0.06	0	0	0
GAIF01000710.1	0.61	0.93	0.16	3.43	3.25	0.47
GAIF01000711.1	0.27	0.39	0.05	1.06	4.41	0
GAIF01000713.1	555.36	651.57	1105.74	228.15	157.85	264.19
GAIF01000715.1	2.31	3.36	0	0	0	0
GAIF01000718.1	20.56	10.91	13.69	53.04	81.99	11.99
GAIF01000719.1	7.64	4.33	0.85	6.73	3.51	0.81
GAIF01000720.1	66.21	95.82	63.06	54.4	4.35	13.09
GAIF01000721.1	0.02	0	0	0.14	1.01	0.07
GAIF01000722.1	0.28	1.72	0	0.76	0.94	0
GAIF01000723.1	0.1	0	0.16	0	0	0
GAIF01000725.1	0.25	0.31	0.55	0.87	1.12	0.04
GAIF01000727.1	4.77	10.37	0	38.74	57.34	26.27
GAIF01000728.1	0.04	0.04	0	0.08	0.21	0
GAIF01000729.1	2.53	3.78	2.34	1.05	0.57	0.13
GAIF01000730.1	0	0	0	0	0.44	0
GAIF01000731.1	0	0	0.46	0.51	8.62	0
GAIF01000732.1	0.17	0.09	0	0.1	0.3	0
GAIF01000734.1	49.25	32.81	11.56	80.21	91.52	5.16
GAIF01000735.1	8.18	4.55	3.31	1.07	2.81	1.21
GAIF01000736.1	87.15	89.17	85.67	260.47	192.79	40.41

GAIF01000737.1	0	0	0	0	0.07	0
GAIF01000742.1	69.44	1.41	18.04	110.69	101.5	116.93
GAIF01000745.1	1203.4	871.24	932.48	5.07	12.26	17.66
GAIF01000747.1	28.37	19.17	5	46.54	45.56	4.85
GAIF01000748.1	1.11	2.02	0.44	5.29	10.7	0.15
GAIF01000751.1	1.08	0.78	0.26	5.58	6.79	0.13
GAIF01000752.1	20.9	1.25	4.05	6.15	12.75	52.59
GAIF01000754.1	0.3	0.28	0.28	0.53	0.24	0
GAIF01000755.1	1.69	1.05	1.86	5.15	6.88	1.78
GAIF01000756.1	1.8	0.76	0.33	7.64	19.68	0.33
GAIF01000757.1	0.9	0	0	0.98	0.14	0
GAIF01000760.1	0	0	0	0	0.16	0
GAIF01000761.1	0.07	0	0	0	2	0
GAIF01000762.1	60.82	132.38	109.13	41.68	59.82	44.37
GAIF01000763.1	0.98	0.57	1.49	0.88	0.27	0.91
GAIF01000764.1	0	0.05	0.03	0.08	0.98	0
GAIF01000766.1	2343.57	2275.82	3797.64	1059.24	619.55	533.48
GAIF01000767.1	5.59	10.35	1.42	13.9	19.68	5.81
GAIF01000768.1	1.34	0.29	0.13	0	1	0.3
GAIF01000769.1	0	0	0	0	0.09	0
GAIF01000771.1	5.13	0.64	1.02	1.17	0.2	0.93
GAIF01000772.1	0	0	0	1.61	0.9	0.21
GAIF01000773.1	34.31	7.62	7.34	33.77	43.43	25.74
GAIF01000774.1	5.25	5.48	6.35	1.14	0.8	0.43
GAIF01000776.1	2.43	0.02	0	7.56	16.61	6.82
GAIF01000777.1	0	0	0	0	0.88	0
GAIF01000778.1	7.37	2.65	1.34	9.5	7.34	3.62
GAIF01000779.1	5.86	6.8	5.06	9.58	12.17	1.96
GAIF01000780.1	0.22	0.34	0.32	1.35	6.85	1
GAIF01000781.1	35.01	15.74	7.58	42.49	16.63	0.69
GAIF01000782.1	13.71	8.56	23.96	5.63	0.78	15.4
GAIF01000783.1	1.72	0.13	0	4.8	23.14	2.54
GAIF01000784.1	0	0	0	0	0.03	0
GAIF01000785.1	0	0	0	1.79	3.12	0.1
GAIF01000786.1	12.64	14.45	6.05	24.26	16.98	1.11
GAIF01000787.1	21.3	18.56	2.39	51.64	61.84	0.65
GAIF01000788.1	0.12	0	0	0	1.12	0
GAIF01000792.1	0.05	0.03	0.05	0.05	0.12	0.55
GAIF01000793.1	1.22	4.13	5.85	5.42	4.58	2.11
GAIF01000794.1	0.21	0.12	0.2	2.43	8.34	0.09
GAIF01000797.1	0.04	0	0	0.05	0	0
GAIF01000798.1	0	0	0	0	1.23	0
GAIF01000799.1	0	0	0	0.13	0.79	0
GAIF01000800.1	0	0.11	0	0.34	3.05	0.22

GAIF01000801.1	22.58	28.7	19.73	9.42	9	7.47
GAIF01000803.1	0.4	0.29	0	3.12	11.31	0.15
GAIF01000804.1	0.91	0.57	0.02	3.93	11.7	0.59
GAIF01000806.1	16.53	14.36	21.97	38.27	21.07	15.19
GAIF01000807.1	0.06	0.48	0.55	0.32	0.26	0.15
GAIF01000808.1	3.22	4.12	4.15	1.09	1.33	0.66
GAIF01000811.1	5.14	4.28	1.32	21.08	32.38	0.62
GAIF01000812.1	8.28	5.56	2.29	40.97	31.65	2.13
GAIF01000813.1	23.67	23.98	17.94	48.92	54.25	15.53
GAIF01000814.1	0.07	0	0	0	0	0
GAIF01000815.1	20.21	7.97	12.9	24.29	23.91	18.45
GAIF01000817.1	0.35	0.24	0.07	0.3	0	0.14
GAIF01000818.1	1.08	0.65	0.79	1.26	1.01	0.26
GAIF01000819.1	0	0.59	1.02	0	0	0.34
GAIF01000820.1	0.31	0.08	0.09	4.33	11.76	0.34
GAIF01000821.1	0.14	0.15	0	0.14	1.07	0
GAIF01000822.1	0.13	0.2	0	0.41	0.28	0
GAIF01000823.1	0.13	0	0.14	0	0	0.24
GAIF01000824.1	13.21	13.69	8.04	18.03	14.82	4.72
GAIF01000825.1	2.75	1.71	1.72	0.57	1.06	0.65
GAIF01000828.1	32.49	34.51	63.02	46.85	33.57	92.37
GAIF01000829.1	1082.97	720.78	341.63	752.35	502.15	65.49
GAIF01000830.1	30.95	58.09	13.78	73.31	67.64	4.08
GAIF01000832.1	0.44	0.19	0	0.31	0	0
GAIF01000835.1	0.23	0.05	0	1.62	12.35	0.45
GAIF01000836.1	0.11	0	0	0	0	0
GAIF01000839.1	0.12	0	0	0	0	0
GAIF01000841.1	0	0	0	0.04	0	0
GAIF01000842.1	0.33	0.38	0	0.25	1.48	0
GAIF01000843.1	0.2	0	0	0	0	0.01
GAIF01000844.1	7.99	1.59	1.07	42.58	122.45	125.76
GAIF01000845.1	0.11	0	0	0.25	0	0.12
GAIF01000846.1	0	0	0	0.05	0	0
GAIF01000847.1	0	0	0	0	0	0.09
GAIF01000848.1	0	0	0	0	0.15	0
GAIF01000849.1	0.14	0	0	1.67	3.62	0
GAIF01000851.1	6.67	2.42	2.38	8.23	20.46	45.77
GAIF01000852.1	0	0	0	0.47	0	0
GAIF01000853.1	5.22	3.93	6.12	18.42	16.74	8.67
GAIF01000856.1	0.62	0.24	0.79	0.47	1.27	0.18
GAIF01000859.1	10.2	11.35	13.84	28.89	22.09	5.47
GAIF01000860.1	11.85	23.74	23.66	30.81	21.11	7.23
GAIF01000861.1	0.15	0.47	0.61	0.93	0.97	0.59
GAIF01000866.1	4.66	7.92	9.76	14.45	10.5	6.3

GAIF01000867.1	2.71	1.59	0	7.7	20	6.06
GAIF01000872.1	6.15	4.67	4.31	10.84	7.85	1.55
GAIF01000873.1	1.72	2.61	2.82	0.2	0.41	0
GAIF01000874.1	0	0.09	0	0	0.1	0
GAIF01000875.1	0	0	0	0	0.09	0
GAIF01000877.1	0.44	1.21	0.49	0.83	2.41	0
GAIF01000878.1	1.59	1.38	0.42	1.92	4.81	0.28
GAIF01000879.1	1.62	0.04	0	5.12	21.47	9.46
GAIF01000880.1	2.51	0.7	0	3.06	4.16	0.27
GAIF01000882.1	45.34	31.29	31.41	65.82	28.37	14.57
GAIF01000883.1	0	0.13	0	0.27	0.99	0
GAIF01000884.1	0.09	0.04	0	0.73	1.75	0
GAIF01000885.1	130.4	63.79	166.27	89.92	28.91	75.86
GAIF01000886.1	0	0	0	0.04	0.04	0.03
GAIF01000887.1	0	0.14	0.13	0.87	0.42	0.73
GAIF01000888.1	0.04	0	0	0	0	0.04
GAIF01000889.1	1.45	0.27	0.06	5.01	1.27	6.21
GAIF01000892.1	1.75	0.31	0.56	0.72	0.2	0.06
GAIF01000893.1	9.61	5.58	4.51	2.95	1.53	0.32
GAIF01000894.1	0	0	0	0	0.64	0
GAIF01000896.1	24.41	20.07	16.34	65.78	57.85	11.28
GAIF01000897.1	0.78	0.07	0	3.72	16.9	0.5
GAIF01000899.1	21.88	19.67	22.25	45.65	39.45	16.12
GAIF01000900.1	1.65	3.18	2.61	15.3	19.81	1.21
GAIF01000901.1	22.69	32.12	26.95	19.38	12.36	4.56
GAIF01000903.1	1.32	3.2	1.56	0.47	0.37	0
GAIF01000904.1	0	0	0	0.11	0.29	0
GAIF01000905.1	0	0	0	0.36	0.1	0
GAIF01000906.1	4.25	2.37	0.13	6.27	20.03	1.39
GAIF01000907.1	0	0.21	0.74	0	0	0.11
GAIF01000908.1	0.05	0	0	0	0	0
GAIF01000912.1	1.21	0	0.51	1.48	4.63	1.65
GAIF01000913.1	0.41	0.44	0.27	0.47	0.29	0
GAIF01000915.1	0	0	0	0	0.07	0
GAIF01000916.1	0	0	0	0	0.6	0
GAIF01000917.1	0	0	0	0	0	0.15
GAIF01000918.1	33.49	20.54	15.55	7.81	7.23	1.1
GAIF01000922.1	0.02	0	0	0.24	0.04	0.09
GAIF01000923.1	30.41	40.51	30.64	87.74	84.29	30.68
GAIF01000924.1	0.87	1.35	0.54	2.44	4.77	0.55
GAIF01000925.1	5.71	2.12	1.01	20.55	44.45	14.61
GAIF01000927.1	9.78	8.67	9.36	23.58	29.33	10.85
GAIF01000928.1	18.63	9.38	18.16	23.04	34.74	87.03
GAIF01000929.1	6.71	2.02	1.34	15.16	26.61	16.04

GAIF01000931.1	0	0.09	0	0	0	0
GAIF01000932.1	0	0	0	0.21	0	0
GAIF01000935.1	144.21	143.1	247.12	0	0.26	0.08
GAIF01000936.1	4.15	5.02	5.9	12.47	12.69	2.6
GAIF01000937.1	0	0	0	0.49	0	0
GAIF01000940.1	0.2	0.38	0	0.78	0.77	0
GAIF01000942.1	9.73	8.22	9.23	20.45	10.6	5.97
GAIF01000943.1	0	0.06	0	0	0	0
GAIF01000944.1	0	0	0	0	0.06	0
GAIF01000945.1	0	0.18	0.12	0	0.53	0
GAIF01000946.1	0	0	0	0	0	0.05
GAIF01000947.1	0	0	0	0.35	0.52	0.04
GAIF01000952.1	0	0.04	0.01	0.11	0.61	0
GAIF01000954.1	36.52	56.45	39.41	77.02	83.78	14.69
GAIF01000957.1	0	0	0	0	0.34	0
GAIF01000958.1	0	0.61	1.93	0	0	0.35
GAIF01000959.1	62.77	73.76	43.25	145.77	119.12	10.36
GAIF01000960.1	4.71	3.62	4.23	6.87	3.53	3.74
GAIF01000961.1	8.91	7.39	5.9	39.42	29.37	5.23
GAIF01000962.1	0.64	0.94	0.87	1.1	0.97	0.72
GAIF01000964.1	0	0.07	0.61	0.45	0.22	0.2
GAIF01000966.1	25.19	18.02	32.93	27.5	14.75	6.21
GAIF01000967.1	10.24	6.41	2.81	21.32	28.63	1.04
GAIF01000968.1	0.14	25.75	81.32	0.78	7.07	11.92
GAIF01000970.1	0	0	0	0.1	0	0
GAIF01000971.1	0.97	0.81	0.67	4.92	10.91	0.76
GAIF01000972.1	29.12	25.13	26.66	115.96	142.35	44.4
GAIF01000973.1	6.52	5.61	5.82	11.32	14.44	4.03
GAIF01000974.1	0	0	0.2	0.14	0.5	0.07
GAIF01000975.1	34.75	22.78	23.44	96.87	46.33	45.73
GAIF01000976.1	2.45	5.18	3.78	8.69	5.08	5.16
GAIF01000977.1	3.49	0	0	16.86	16.24	21.66
GAIF01000978.1	0.09	0.1	0	0.11	0	0.1
GAIF01000979.1	0.33	0.07	0	0.23	0.16	0
GAIF01000981.1	5.2	3.38	3.41	10.14	61.07	1.85
GAIF01000982.1	0.43	1.16	1.08	3.06	4.56	0.38
GAIF01000984.1	23.42	26.46	21.41	29.26	35.23	32.98
GAIF01000986.1	1.19	0.77	0.07	0.94	1.28	0.19
GAIF01000989.1	58.18	33.09	22.88	66.74	109.78	81.3
GAIF01000990.1	17.71	20.57	0.01	25.72	18.29	0
GAIF01000993.1	142.36	48.88	180.97	191.31	108.13	578.01
GAIF01000995.1	0.19	0.21	0.05	2.83	5.87	0.16
GAIF01000996.1	13.54	5.34	4.96	16.98	28.91	27.08
GAIF01000999.1	0.14	0.05	0	0.58	1.2	0

GAIF01001001.1	0.15	0	0	0.88	4.74	0
GAIF01001002.1	0	0	0	0.12	1.57	0.11
GAIF01001005.1	3.32	0	0.56	13.51	19.86	0
GAIF01001006.1	0	0	0	0	0.14	0
GAIF01001008.1	7.34	1.4	0	13	18.88	4.08
GAIF01001011.1	0	0	0	0.05	0.21	0
GAIF01001014.1	1.14	0.87	0.85	0.13	0.49	0.5
GAIF01001015.1	6.87	4.46	1.6	0	16.12	4.81
GAIF01001017.1	1.58	0.9	0.56	8.9	27.97	0.28
GAIF01001018.1	0.32	0	0.25	1.76	6.31	0.65
GAIF01001019.1	0.58	0	0	0.79	5.15	2.76
GAIF01001021.1	0.13	0	0	0	0	0
GAIF01001022.1	0	0.15	0	0	0	0
GAIF01001023.1	3.02	2.28	3.06	6.29	5.72	1.43
GAIF01001024.1	0.14	0.23	0.1	0	0.06	0.09
GAIF01001026.1	0	0	0	0	0.23	0
GAIF01001027.1	4.99	4.83	6.17	13.15	9.38	9.29
GAIF01001029.1	0	0	0	0	0.04	0
GAIF01001031.1	1.54	0.19	0.31	7.17	23.97	4.7
GAIF01001032.1	1.77	0.48	0.44	7.12	15.78	1.69
GAIF01001034.1	6.38	5.97	5.86	5.99	2.04	2.96
GAIF01001035.1	0	0.27	0	5.88	13.61	0.4
GAIF01001037.1	184.05	211.83	466.64	206.95	153.19	236.49
GAIF01001038.1	6.21	7.42	6.37	7.45	5.83	0.45
GAIF01001040.1	9.62	10.38	11.77	8.18	12.71	5.42
GAIF01001041.1	169.8	564.93	603.02	5.61	18.77	50.3
GAIF01001042.1	0.16	0	0	1.15	10.69	0.73
GAIF01001043.1	0	13.57	11.44	0.14	0.55	3.61
GAIF01001044.1	0.03	0	0	0.11	0.88	0.11
GAIF01001045.1	0.27	0	0	0	0	0
GAIF01001047.1	0	0.1	0	0	0.11	0
GAIF01001048.1	0.27	0.15	0	0.05	0	0
GAIF01001049.1	0.23	0	0	0	0	0
GAIF01001050.1	1.01	0.11	0	3.35	10.07	0.61
GAIF01001051.1	1	0.2	0	3.36	15.56	0.3
GAIF01001052.1	352.3	793.79	491.5	72.48	78.58	123.23
GAIF01001054.1	1.87	3.37	0.35	10.94	19.5	0.1
GAIF01001055.1	3.55	2.95	2.41	4	4.77	3.91
GAIF01001056.1	1.61	0.22	0.08	7.88	7.96	0.25
GAIF01001057.1	0	0	0	0	0.66	0
GAIF01001058.1	0.09	0.1	0.1	0	0	0
GAIF01001062.1	2.34	2.17	0.9	2.04	0.99	1.16
GAIF01001065.1	6.15	4.89	5.58	1.78	0.57	0.78
GAIF01001066.1	9.8	7.66	4.65	0.89	0.9	0.36

GAIF01001068.1	7.39	3.59	3.97	1.83	1.51	0.17
GAIF01001069.1	0	0	0.63	0.4	0.34	0
GAIF01001070.1	0	0	0	0	0.07	0
GAIF01001071.1	0.28	0.15	0	0.8	0.33	0
GAIF01001073.1	10.97	3.42	15.4	4.02	4.28	6.13
GAIF01001074.1	33.08	17.23	7.71	95.69	95.64	7.47
GAIF01001075.1	5.1	3.34	2.9	1.9	2.1	0.17
GAIF01001076.1	4.09	4.29	2.68	2.51	0.85	0.1
GAIF01001079.1	6.45	5.71	10.82	15.82	11.96	15.02
GAIF01001080.1	12.61	9.44	1.99	7.53	29.19	4.34
GAIF01001081.1	0.04	0	0.04	0	0.17	0.04
GAIF01001084.1	9.6	0	0.74	5.21	0.6	1.31
GAIF01001085.1	0.72	4.26	0.26	1.24	6.15	1.06
GAIF01001086.1	0	0	0	0	3.13	0
GAIF01001087.1	0.21	0	0.23	3.14	10.65	0.34
GAIF01001088.1	5.52	1.28	3.37	2.09	2.13	1.49
GAIF01001089.1	514.21	595.82	542.64	766.51	467.2	236.79
GAIF01001090.1	0.07	0	0	0	0	0
GAIF01001091.1	12.59	8.72	11.87	19.91	10.09	26.31
GAIF01001092.1	0	0	0.06	0	0	0
GAIF01001094.1	14.85	21.67	19.35	31.44	12.03	14.93
GAIF01001095.1	0	0	0	0.3	0.63	0
GAIF01001096.1	0	0	0	0.08	0.04	0
GAIF01001097.1	48.26	63.34	25.26	108.77	107.06	12.37
GAIF01001098.1	5.15	3.67	0.39	9.06	15.37	0.39
GAIF01001099.1	0.3	0	0	1.47	7.15	0.27
GAIF01001100.1	40.73	14.91	9.91	5.2	5.22	10.41
GAIF01001101.1	0.04	0.05	0	0.4	0.33	0.05
GAIF01001102.1	1.81	1.53	2.53	1.69	2.02	3.28
GAIF01001103.1	0	0	0	0	0.07	0
GAIF01001104.1	12.51	29.02	17.84	5.37	4.5	1.09
GAIF01001105.1	25.42	0.14	0.61	11.74	0	0.42
GAIF01001108.1	31.87	32.41	26.74	62.55	52.79	12.05
GAIF01001111.1	0.09	0.12	0.13	0.19	0.16	0.08
GAIF01001112.1	1.66	0.47	0.99	3.79	0.57	2.61
GAIF01001115.1	5.99	0.71	0.32	23.81	80.67	21.8
GAIF01001116.1	0	0	0	0	0	0.16
GAIF01001117.1	122.59	153.57	138.59	201.41	128.34	45.56
GAIF01001121.1	4.02	3.08	3.07	10.14	2.81	3.72
GAIF01001122.1	0	0.15	0	0.81	6.81	4.79
GAIF01001123.1	10.75	8.37	11.71	24.73	28.4	12.97
GAIF01001124.1	0	0	0	0.12	1.68	0.24
GAIF01001125.1	20.05	18.26	1.69	20.11	33.15	3.4
GAIF01001126.1	0.31	0.02	0.52	1.36	5.98	0.36

GAIF01001127.1	3.97	4.83	3.48	2.03	2.86	0.87
GAIF01001128.1	58.53	73.88	78.57	42.55	27.15	26.03
GAIF01001129.1	4.18	0.98	1.27	6.13	6.54	3.93
GAIF01001131.1	26.48	20.5	16.96	30.56	30.67	5.25
GAIF01001132.1	0.11	0.12	0.87	0.26	0	0
GAIF01001133.1	0	0	0	0.19	0	0
GAIF01001134.1	0	0	0	0.16	0.4	0.15
GAIF01001137.1	3.59	3.68	0.15	6.69	15.94	4.79
GAIF01001138.1	0	0.12	0	0.13	0.07	0
GAIF01001139.1	0.49	0	0.33	1.51	8.61	1.51
GAIF01001140.1	0.09	0	0	0	0	0
GAIF01001141.1	2.18	6.82	2.33	10.28	9.37	0.31
GAIF01001143.1	1.19	0	0.15	3.14	9.32	6.31
GAIF01001144.1	0	0	0	0.06	0.04	0
GAIF01001146.1	0.35	0.07	0.07	1.07	7.75	0.29
GAIF01001147.1	60.68	59.59	31.69	26.4	22.05	9.52
GAIF01001149.1	1.02	1.66	1.12	7.32	17.86	0
GAIF01001150.1	0.98	0	0	5.46	0.47	0
GAIF01001151.1	0.07	0	0.02	0.4	1.82	0.21
GAIF01001152.1	69.77	80.13	85.1	543.88	566.51	76.89
GAIF01001153.1	0.14	0	0	0.54	3.53	0
GAIF01001157.1	0.02	0.01	0	0	0	0.04
GAIF01001158.1	9.06	17.68	11.41	29.86	14.75	3.38
GAIF01001160.1	1.57	0	0.42	0	0	0
GAIF01001161.1	0.39	0	0	0	0	0.92
GAIF01001162.1	899.94	1981.6	1942.8	992.79	511.78	318
GAIF01001164.1	8.32	13.88	15.95	32.24	17.76	5.47
GAIF01001165.1	0.19	0.1	0	1.52	7.89	1.34
GAIF01001166.1	0.78	0.96	0.23	1.23	1.53	0.2
GAIF01001167.1	88.39	35.79	28.13	132.07	211.15	236.71
GAIF01001169.1	20.98	8.55	23.03	54.78	37.84	31.79
GAIF01001170.1	8.21	6.3	6.36	15.25	16.79	1.95
GAIF01001172.1	30.65	5.58	1.74	15	14.08	14.57
GAIF01001173.1	1.21	0	0.44	0.23	0.48	0.22
GAIF01001174.1	0	0	0	0.12	0	0
GAIF01001176.1	0	0	0	0	0.65	0
GAIF01001177.1	6.07	5.19	6.3	22.62	24.05	4.12
GAIF01001178.1	0	0	0	0.21	0.64	0.07
GAIF01001179.1	0.3	0.11	0.74	0.26	0.05	0.87
GAIF01001182.1	6.18	6.41	0.98	1	0.24	0.06
GAIF01001183.1	0.57	0.76	0.28	0.96	0.31	0
GAIF01001185.1	33.31	35.71	39.81	31.02	22	37.58
GAIF01001186.1	0.81	0.75	1.04	0.85	0.49	0.35
GAIF01001187.1	0.22	0.48	0.84	0.64	0.13	0.12

GAIF01001188.1	0	0.2	0	2.26	5.58	0.41
GAIF01001189.1	0.02	0	0	0	0	0
GAIF01001190.1	5.05	2.77	2.16	21.58	27.96	2.26
GAIF01001191.1	0.12	0.11	0	0.46	0.25	0
GAIF01001192.1	21.69	24.55	4.45	0.22	0.45	0.21
GAIF01001194.1	0.81	0.18	0	1.87	6.39	0.18
GAIF01001198.1	5.58	1.52	0	2.2	4.59	0.86
GAIF01001199.1	0	0.12	1.37	0.66	3.14	0.25
GAIF01001200.1	0.12	0.46	0.27	0.42	0.8	0.07
GAIF01001201.1	22.02	5.76	8.71	40.22	14.16	2.01
GAIF01001202.1	0	0.07	0	0.38	0.51	0
GAIF01001203.1	0.54	0.46	0.49	0	0.26	0.52
GAIF01001204.1	0	0	0.18	0.59	2.83	0
GAIF01001205.1	0	0	0.04	0	0.04	0
GAIF01001206.1	0	0	0	0.23	0.68	0
GAIF01001207.1	19.21	18.1	13.93	49.68	60.97	10.06
GAIF01001208.1	0	0	0	0	0.08	0
GAIF01001209.1	27.13	13.82	5.58	63.34	53.28	21.78
GAIF01001210.1	0.3	0.55	0	1.13	1.97	0
GAIF01001211.1	0	0	0	0.82	0.55	0
GAIF01001213.1	0.59	0	0	0.51	0.89	0.16
GAIF01001214.1	0.06	0	0	0.28	0.79	0
GAIF01001215.1	0.62	0	0.29	1.44	7.37	4.4
GAIF01001216.1	0.25	1.21	1.09	1.43	1.49	0.14
GAIF01001217.1	4.63	2.11	1.39	12.52	22.09	1.3
GAIF01001219.1	164.5	40.57	99.63	69.89	66.04	561.35
GAIF01001220.1	0	0.3	0	0	0	0
GAIF01001221.1	0	0	0	0.3	0.31	0
GAIF01001222.1	0	0.15	0	0	0.54	0
GAIF01001223.1	0.88	0.43	0.32	0	0.24	0.11
GAIF01001224.1	0.81	0.11	0.22	2.35	11.68	6.91
GAIF01001228.1	15.69	15.48	15.57	30.97	19.33	6.52
GAIF01001229.1	1.02	0	0.09	3.85	21.08	7.88
GAIF01001230.1	0	0	0	0	0.07	0
GAIF01001231.1	25.18	17.05	20.22	33.39	35.89	30.22
GAIF01001232.1	17.06	34.19	8.93	44.97	27.83	0.01
GAIF01001234.1	0.03	0	0.03	0.33	0.23	0.1
GAIF01001237.1	0	0	0.3	0	0.26	0
GAIF01001238.1	0.61	0	0	1.77	4.65	6.05
GAIF01001240.1	26.35	18.08	14.5	30.3	25.01	33.05
GAIF01001241.1	1.81	1.43	1.45	1.91	1.78	0.36
GAIF01001242.1	0	0	0	0	0.09	0
GAIF01001243.1	0	0	0	0	0.39	0
GAIF01001244.1	4.2	4.85	2.78	1.48	1.06	0.93

GAIF01001247.1	264.6	281.13	659.29	194.31	125.71	431.01
GAIF01001249.1	0.15	0	0	0.35	0	0
GAIF01001251.1	50.78	26.81	35.44	32.74	20.35	10.13
GAIF01001252.1	17.56	8.76	7.51	25.2	29.94	8.16
GAIF01001257.1	0.84	0.17	0.08	0.09	1.29	0
GAIF01001258.1	0.17	0.12	0.12	1.57	8.63	0.12
GAIF01001259.1	0	0	0	0.16	0.16	0.59
GAIF01001260.1	53.32	43.46	40.02	94.79	62.18	31.57
GAIF01001267.1	0.7	0.5	1.17	2.26	0.46	6.36
GAIF01001268.1	0	0	0	0	0.01	0
GAIF01001269.1	0.12	0.09	0.03	0.12	0.07	0.02
GAIF01001273.1	0.96	0.72	0.33	1.54	1.96	0
GAIF01001276.1	0	7.72	3.4	0	0	1.71
GAIF01001277.1	0	0	0	0	0	0.24
GAIF01001282.1	12.91	22.72	20.56	0.67	0.47	1.84
GAIF01001283.1	8.24	9.12	13.31	18.04	9.53	12.84
GAIF01001285.1	3.12	4.15	4.7	1.21	1.11	2.42
GAIF01001286.1	3.93	0.16	0	5.89	29.41	7.8
GAIF01001287.1	1.04	0.17	0	1.51	0.82	1.8
GAIF01001288.1	33.47	44.16	28.29	6.01	3.37	2.16
GAIF01001290.1	1.28	0.25	0.51	0	0	0
GAIF01001291.1	0.66	0.13	0.1	1.2	1.38	0.99
GAIF01001292.1	1.15	1.82	1.66	2.89	1.39	0.18
GAIF01001293.1	17.86	12.56	2.75	26.43	62.94	2.22
GAIF01001294.1	0	0	0.07	0	0	0
GAIF01001295.1	0	0	0	0	0.12	0
GAIF01001297.1	0	0	0	0	0.12	0
GAIF01001298.1	4.25	3.79	3.75	14.42	8.11	7.38
GAIF01001299.1	0	0	0.32	0	0.1	0
GAIF01001301.1	16.44	16.66	14.91	124.72	126.01	12.63
GAIF01001302.1	12.43	10.31	2.31	30.31	62.06	0.93
GAIF01001303.1	13.56	9.89	10.47	7.01	4.63	2.89
GAIF01001305.1	0	0	0.05	0	0	0
GAIF01001306.1	0	0	1.65	0.64	1.41	0
GAIF01001309.1	3.51	2.8	0.3	24.88	41.41	1.04
GAIF01001310.1	0.23	0.13	0	0.27	2.66	0.26
GAIF01001311.1	13.58	19.89	5.53	32.27	29.78	0.26
GAIF01001314.1	686.86	374.36	681.31	2837.1	1875.62	5507.43
GAIF01001315.1	0.37	0.22	0.19	0.53	0.45	0.03
GAIF01001317.1	2369.08	818.23	608.8	153.76	156.64	375.86
GAIF01001318.1	0.27	0	0	0.16	0	0
GAIF01001319.1	17.46	16.4	13.57	9.21	3.27	0.75
GAIF01001320.1	0	0	0	2.27	1.49	0.18
GAIF01001322.1	0	0	0	0	0.17	0

GAIF01001323.1	0	0	0	0.21	1.21	0
GAIF01001324.1	0	0.13	0.06	0.07	0.07	0
GAIF01001326.1	0	0	0	0.5	3.31	0.04
GAIF01001328.1	0	0	0	0	0.31	7.53
GAIF01001329.1	0	0	0.11	0	1	0
GAIF01001330.1	0	0.17	0	0.49	0.05	0
GAIF01001331.1	0.09	0.09	0	0.05	0	0
GAIF01001332.1	8.43	16.76	14.33	27.25	17.38	3.23
GAIF01001333.1	0.45	0.28	0.08	0	0.08	0.08
GAIF01001334.1	0.69	0.11	0	2.42	21.77	16.01
GAIF01001335.1	9.4	20.97	9.72	31.77	37.4	1.94
GAIF01001337.1	0	0	0	0	0.12	0
GAIF01001339.1	3.61	2.49	0.98	0.77	0.31	0.14
GAIF01001340.1	24.98	0	7.71	20.29	31.54	4.82
GAIF01001341.1	0	0.2	0.61	0	0	0.2
GAIF01001343.1	0.97	0.19	1.07	0.91	1.64	1.07
GAIF01001344.1	0.14	0	0	0	0.15	0
GAIF01001345.1	0	0	0	0	0.18	0
GAIF01001346.1	36.87	41.66	24.49	9.38	13.73	19.34
GAIF01001347.1	0.1	0.28	0.12	0.5	0.83	0
GAIF01001350.1	0.88	0.31	0	8.98	28.04	0
GAIF01001355.1	0	0	0	0.48	0.25	0
GAIF01001356.1	1.42	0.46	0	7.58	24.46	0.37
GAIF01001359.1	5.65	3.46	3.56	10.48	24.35	5.02
GAIF01001362.1	0.17	0	0	0.1	0	0
GAIF01001364.1	23.28	18.79	16.79	12.26	9.96	5.28
GAIF01001365.1	0	0	0.25	0	0	0
GAIF01001366.1	12.71	27.79	8.01	72.04	81.58	18.2
GAIF01001367.1	0.19	0.07	0.17	0.15	0.42	0.12
GAIF01001368.1	454.32	151.72	51.88	26.93	18.25	6.49
GAIF01001369.1	2	1.54	1.59	12.76	28.76	1.28
GAIF01001370.1	8.25	3.59	2.98	15.73	18.89	5.99
GAIF01001371.1	1.83	4.72	2.76	10.61	10.63	0.67
GAIF01001373.1	21.99	60.43	0.97	41.69	50.4	0.11
GAIF01001375.1	0	0	0	0.77	3.03	0.07
GAIF01001376.1	0.7	0.26	0.23	4.43	22.94	1.25
GAIF01001377.1	0.12	0	0.05	0.61	0	0
GAIF01001381.1	0	0	0.12	0.25	2.44	0.24
GAIF01001382.1	0.55	0	0	1.72	2.6	0.37
GAIF01001383.1	761.98	50.7	147.09	359.6	146.41	1651.64
GAIF01001385.1	5.28	2.38	4.8	14.34	10.99	22.41
GAIF01001386.1	556.87	358.12	288.04	1411.9	2349.43	119.76
GAIF01001387.1	33.12	0	0.01	0.02	18.57	31.6
GAIF01001388.1	0	0.14	0	0.1	0.36	0.05

GAIF01001389.1	0	0	0	0	0.08	0
GAIF01001391.1	0	0	0	0	0.92	0
GAIF01001392.1	0.73	0.64	1.46	0.7	1.23	0.14
GAIF01001393.1	0	0	0.07	0	0.07	0
GAIF01001394.1	0.95	1.2	0.17	5.13	13.47	3.65
GAIF01001396.1	0.42	1.37	0.81	1.7	0.5	0.92
GAIF01001397.1	0.45	0.38	0.38	0	0	0.08
GAIF01001398.1	1.61	0.44	1.15	0.35	0.37	0.24
GAIF01001399.1	11.98	6.99	3.66	17.3	28.85	2
GAIF01001400.1	21.83	13.96	21.11	55.4	24.46	25.44
GAIF01001403.1	3.29	5.1	2.96	13.57	6.03	0.77
GAIF01001404.1	0	0.26	0.18	0	0.05	0.79
GAIF01001405.1	4.73	0	0	5.19	26.92	6.26
GAIF01001406.1	0.44	0.46	0.47	0.25	0.15	0.31
GAIF01001407.1	17.32	27	31.06	10.72	6.25	6.58
GAIF01001409.1	1.09	0	0	0.69	1.81	0
GAIF01001412.1	0.27	0.24	0	0	0.21	0
GAIF01001414.1	0.2	0	0	0.23	0.24	0.88
GAIF01001415.1	26.17	10.45	8.99	44.52	58.42	26.35
GAIF01001416.1	8.3	6.69	3.82	16.7	25.33	1.99
GAIF01001417.1	0.09	0.1	0	1.46	5.99	0.1
GAIF01001420.1	90.39	23.66	2.05	149.42	285.02	9.39
GAIF01001421.1	4.3	1.53	0.34	16.52	40.17	4.31
GAIF01001422.1	0	0	0.14	0	0	0
GAIF01001425.1	0	0	0	0	0.34	0.04
GAIF01001427.1	3.6	2.08	3.83	6.92	4.19	3.1
GAIF01001428.1	2.76	2.81	0.4	17.78	50.74	0.2
GAIF01001429.1	0	0	0	0.02	0	0
GAIF01001430.1	0	0	0	0.13	0	0
GAIF01001431.1	0.48	0	0.09	0.09	0.19	0
GAIF01001432.1	87.43	38.03	76.84	239.05	189.2	211.97
GAIF01001433.1	52.4	38.86	31.21	63.52	24.49	29.57
GAIF01001434.1	2.43	3.33	1.17	20.12	38.63	1.08
GAIF01001436.1	4.7	1.27	0.39	22.1	67.35	3.81
GAIF01001440.1	0	0	0	0.07	0.61	0
GAIF01001441.1	0	0	0	0	0.2	0
GAIF01001443.1	0	0	0	0.11	0	0
GAIF01001444.1	4.46	5.08	1.6	0.61	0	0.08
GAIF01001445.1	1.32	0	0	2.75	16.04	0.51
GAIF01001446.1	0	0	0.32	1.52	0.73	0.17
GAIF01001447.1	5.47	7	6.69	3.34	1.58	2.23
GAIF01001448.1	0	0.04	0	0.18	1.6	0.08
GAIF01001449.1	42.77	38.02	47.33	48.59	52.26	56.74
GAIF01001450.1	2.15	0.13	0.14	0	0	0

GAIF01001451.1	0.7	0.05	0.41	3.3	1.74	1.59
GAIF01001452.1	32.57	12.57	11.62	77.91	83.8	11.09
GAIF01001453.1	0	0	0	0	0.25	0
GAIF01001454.1	0	0	0	0	0.86	0
GAIF01001455.1	7.55	6.61	3.46	9.06	8.2	1.3
GAIF01001456.1	685.63	334.04	524.92	156.87	206.96	213.37
GAIF01001457.1	11.63	13.77	10.81	11.07	4.39	3.75
GAIF01001459.1	0	0	1.11	0	0	0
GAIF01001460.1	0.27	0.07	0.37	1.23	3.35	0.45
GAIF01001462.1	0	0	0.11	0	0	0
GAIF01001466.1	0	0	0	0.06	0.63	0
GAIF01001467.1	6.98	8.55	5.58	30.81	34.85	1.03
GAIF01001469.1	0	0	0	0	0.21	0
GAIF01001470.1	0.1	0	0	1.63	2.05	0
GAIF01001471.1	4.69	4.97	6.85	3.31	2.94	3.6
GAIF01001472.1	0.05	0.08	0.1	0.17	0.06	0
GAIF01001473.1	0.18	0.2	0	2.34	10.57	0
GAIF01001476.1	0	0	0	0	0.15	0
GAIF01001477.1	0.31	0.11	0	0.61	5.42	0
GAIF01001478.1	9.64	0.63	0	8.57	17.58	0
GAIF01001479.1	16.18	21.56	13.08	56.25	35.83	5.18
GAIF01001480.1	8.73	5.89	2.89	26.81	38.66	2.87
GAIF01001482.1	0	0.05	0.21	0	0	0.15
GAIF01001487.1	0.34	0	0	0.33	0.27	0.99
GAIF01001488.1	44.5	16.23	12.28	33.62	56.22	37.08
GAIF01001489.1	234.99	210.98	142.22	380.8	208.01	123.15
GAIF01001490.1	108.3	60.31	36.92	112.01	248.93	82.16
GAIF01001491.1	0	0.52	0.7	0	0	0
GAIF01001492.1	0.36	0.17	0	0.56	0	0.19
GAIF01001496.1	0	0.14	0	0	0	0
GAIF01001498.1	0	0	0	0.03	0.01	0
GAIF01001499.1	130.43	131.47	120.82	224.13	315.52	1255.95
GAIF01001500.1	13.56	4.98	7.3	17.22	19.27	33.42
GAIF01001501.1	0.14	0	0.08	0.16	0	0.22
GAIF01001502.1	0.08	0	1.01	0	0.02	0.14
GAIF01001503.1	0	0	0	0	0.06	0
GAIF01001504.1	6.08	10.37	14.72	24.6	17.08	11.06
GAIF01001505.1	1.31	1.57	0.9	4.58	8.34	0.47
GAIF01001506.1	5.11	5.79	3.85	21.5	16.27	1.29
GAIF01001507.1	0	0	0	0	0.11	0
GAIF01001508.1	0.85	0.17	0.17	0.9	3.32	0.26
GAIF01001512.1	0	0	0	0	0.22	0
GAIF01001513.1	20.23	20.64	14.81	28.5	31.9	5.41
GAIF01001514.1	0	0	0	0.32	0.17	0

GAIF01001515.1	2.75	2.89	0.42	5.77	10.34	2.68
GAIF01001516.1	0.87	0.54	0.27	4.12	6.07	0.21
GAIF01001517.1	25.89	26.14	49.8	69.3	46.07	37.47
GAIF01001519.1	0	0	0	0.1	1.85	0
GAIF01001520.1	1.07	1.17	0.59	0.62	0.32	0.15
GAIF01001521.1	0.2	0.03	0	0.02	1.47	0
GAIF01001522.1	0	0.14	0.21	0	0.04	0.14
GAIF01001523.1	0.18	0.23	0	0.21	0.29	0
GAIF01001525.1	0	0	0	0.12	0.6	0.33
GAIF01001528.1	14.76	15.73	10.33	34.64	23.89	3.57
GAIF01001529.1	1.97	0.23	0.54	1.83	2.83	0.34
GAIF01001533.1	0.04	0	0.29	2.51	5.52	0.1
GAIF01001534.1	0	0	0	1.6	0.57	0
GAIF01001535.1	0.32	0.17	1.31	0.37	0.1	0.17
GAIF01001536.1	16.58	9.35	5.9	3.07	3.53	1.3
GAIF01001537.1	0	0	0	0.06	0.12	0
GAIF01001538.1	0.54	0	0	0.32	0.33	0.6
GAIF01001539.1	0.04	0	0.05	0.15	0.48	0.1
GAIF01001540.1	7.35	12.9	9.15	17.32	12.59	1.72
GAIF01001541.1	0.18	0.39	0.13	0.49	1.24	0.13
GAIF01001542.1	0	0	0	0	0.05	0
GAIF01001543.1	0.7	0.37	0.21	0.51	0.43	0.02
GAIF01001544.1	43.68	39.62	86.36	14.86	12.69	69.44
GAIF01001545.1	0.11	0	0.17	0.13	0	0.06
GAIF01001546.1	0.06	0.03	0	0	0.08	0
GAIF01001547.1	0	0	0	0	1.99	0.32
GAIF01001548.1	0	0.16	0	2.3	18.49	0.07
GAIF01001550.1	0	0	0	0	0.11	0
GAIF01001551.1	2.79	3.93	0.9	4.57	7.9	0
GAIF01001552.1	0	0	0.12	0.16	0	0
GAIF01001553.1	4.66	5.63	2.21	0.6	0.15	0.14
GAIF01001554.1	1.5	0	0	1.02	0	1.67
GAIF01001556.1	0.38	0.26	0.57	0.53	0.56	0.13
GAIF01001557.1	0.47	0.18	0.08	3.19	16.97	0.05
GAIF01001558.1	0.18	0	0	0.39	3.55	0.19
GAIF01001559.1	41.26	113.97	120.66	30.13	46.99	39.34
GAIF01001560.1	0.24	0	0	0	0.14	0
GAIF01001562.1	167.76	120.47	262.1	377.2	229.46	335.84
GAIF01001563.1	0.11	0.22	0.13	0.03	0.35	0
GAIF01001564.1	0.27	0.54	0.27	0.48	0.48	0
GAIF01001565.1	59.36	90.68	60.86	135.91	177.42	17.33
GAIF01001567.1	0.2	0	0	0	0	0
GAIF01001569.1	0	0	0	0	0.11	0
GAIF01001570.1	0	0	0.19	0	0	0

GAIF01001572.1	0	0.41	0	0.47	1.47	0
GAIF01001574.1	0	0	0	0	0	0.14
GAIF01001575.1	47.56	63.95	44.7	83.4	44.03	9.74
GAIF01001577.1	0.05	0	0	0.17	0.03	0
GAIF01001579.1	0	0	0	0.29	0.9	0.07
GAIF01001580.1	0.83	0	0	3.86	16.61	7.41
GAIF01001581.1	0.1	0.1	0	0.43	1.42	0.16
GAIF01001582.1	18.02	16.62	16.82	91.19	71.5	11.53
GAIF01001583.1	0	0	0	0	0.28	0.03
GAIF01001584.1	0.12	0	0	0	0	0
GAIF01001585.1	0	0.08	0	0.58	0	0
GAIF01001586.1	0	0	0	0	0.23	0
GAIF01001587.1	166.22	59.49	30.25	10.31	10.46	0
GAIF01001588.1	0.02	0	0	0	1.61	0.53
GAIF01001590.1	0	0	0	0	0.21	0
GAIF01001591.1	0.2	0	0.07	0.15	0.15	0
GAIF01001593.1	0.34	0.18	0	0	0.4	0
GAIF01001594.1	0.09	0	0.2	1.56	7.34	0.4
GAIF01001595.1	0	0.18	0	0.09	0.97	0.09
GAIF01001597.1	0.72	0.43	0	2.78	7.82	0.35
GAIF01001598.1	0	0.09	0.26	0.09	0.19	0.09
GAIF01001599.1	7.68	9.71	4.38	29.23	29.57	1.87
GAIF01001600.1	0	0	0	0	3.19	0
GAIF01001601.1	0	0	0.02	0	0.05	0.02
GAIF01001602.1	7.95	8.01	18.23	4.61	2.81	10.05
GAIF01001603.1	11.83	12.79	3.49	27.54	39.95	3.01
GAIF01001604.1	218.28	175.98	156.46	522.44	454	104.8
GAIF01001605.1	0	0.06	0	0	0	0
GAIF01001608.1	0	0	0	10.15	0	0
GAIF01001609.1	7.64	4.99	1.76	44.75	28.53	1.57
GAIF01001611.1	3.72	2.23	3.55	1.8	0.13	1.18
GAIF01001612.1	10.52	0.5	0.45	14.5	9.94	2.86
GAIF01001614.1	0.73	0.34	0.57	2.61	5.78	0.75
GAIF01001615.1	0	0.17	0	0	0	0
GAIF01001617.1	0.09	0.06	0	0.34	0.15	0
GAIF01001618.1	0.02	0	0	0	0.02	0
GAIF01001619.1	12.83	3.18	5.8	17.03	9.93	2.33
GAIF01001621.1	0	1.53	1.79	0	0	0
GAIF01001622.1	181.9	120.2	51.3	211.96	251.78	127.26
GAIF01001623.1	0	0.05	0	0	0	0
GAIF01001625.1	0	0.29	0	0.38	0.33	0
GAIF01001626.1	0	0	0	0	0	0.08
GAIF01001627.1	99.98	96.23	32.86	275.45	330.21	9.94
GAIF01001628.1	425.76	292.48	443.17	517.93	223.51	658.37

GAIF01001630.1	16.26	19.62	16.79	34.62	27.08	0.87
GAIF01001631.1	6.47	4.94	4.1	2.38	1.35	1.72
GAIF01001632.1	0.99	2.6	3.11	11.58	5.65	1.02
GAIF01001633.1	2.73	2.52	1.25	5.91	7.57	1.06
GAIF01001634.1	8.32	12.01	12.26	7.45	6	2.49
GAIF01001635.1	0.13	0.07	0	0	0	0
GAIF01001636.1	805.75	530.27	337.16	557.12	424.09	124.21
GAIF01001637.1	0	0	0.66	0	0	0
GAIF01001638.1	0	0	0.5	0	0.55	0.17
GAIF01001639.1	8.48	16.82	6.79	21.31	13.44	3.3
GAIF01001640.1	0.83	0.42	0.63	1.71	0.78	0.14
GAIF01001641.1	31.29	13.62	16.67	10.35	0	5.49
GAIF01001642.1	14.42	19.77	7.63	53.21	68.55	3.6
GAIF01001643.1	0	0.09	0	0.37	0.39	0
GAIF01001644.1	0.12	0.03	0	0	0.01	0
GAIF01001645.1	0.15	0.18	0	0	0.1	0
GAIF01001646.1	4.48	5.43	10.4	6.2	4.56	6.26
GAIF01001648.1	10.91	6.4	7.11	21.45	28.79	24.65
GAIF01001649.1	16.21	15.48	2.55	41.68	68.35	5.12
GAIF01001650.1	13.92	20.98	15.28	36.42	21.59	7
GAIF01001653.1	10.7	8.98	1.59	17.48	29.25	24.82
GAIF01001654.1	0.03	0	0	0	0	0
GAIF01001655.1	86.11	75.65	54.6	204.79	97.35	46.79
GAIF01001656.1	0	0	0	0.09	0.09	0
GAIF01001658.1	2.71	2.05	0.15	3.87	7.54	2.49
GAIF01001659.1	0.44	0.08	0.03	0.1	0.07	0
GAIF01001661.1	0	0	0	0.05	0	0
GAIF01001662.1	19.06	8.4	31.35	53.8	27.17	90.61
GAIF01001663.1	1.86	0	0.06	0.86	0.06	0
GAIF01001664.1	0.26	0	0	0.75	6.22	0.43
GAIF01001666.1	12.52	15.48	20.82	10.47	10.82	8.42
GAIF01001667.1	0	0.65	0	0	0	0
GAIF01001668.1	0.31	0	0	0.12	1.66	0
GAIF01001669.1	25.55	7.8	9.22	30.46	14.94	51.1
GAIF01001670.1	8.1	10.97	7.7	32.73	30.31	4.26
GAIF01001672.1	0.31	0	0	1.11	4.8	0.09
GAIF01001674.1	0.52	0.31	0.52	0.57	1.22	0.62
GAIF01001676.1	6.02	8.06	4.83	0.26	0.2	0
GAIF01001678.1	0.12	0	0	0.21	1.84	0
GAIF01001680.1	0.04	0	0	0	0.07	0
GAIF01001681.1	0.18	0	0	1.73	4.12	0.1
GAIF01001682.1	0.55	1.6	0	0.88	1.97	0
GAIF01001685.1	8.05	10.04	10.21	8.31	8.67	15.08
GAIF01001686.1	20.52	16.78	16.92	75.74	90.32	10.05

GAIF01001687.1	0	0.1	0.05	0.16	0.56	0
GAIF01001688.1	0	0.11	0.11	0.12	0	0
GAIF01001689.1	0.06	0.07	0	0.07	0.53	0
GAIF01001690.1	0	0	0	1.25	0.22	0.2
GAIF01001691.1	0.25	0	0	0.58	0.91	0.14
GAIF01001692.1	0	0	0	0	0.07	0
GAIF01001693.1	0.48	0.45	0.18	0.93	0.69	0
GAIF01001694.1	0	0	0	0	0.23	0
GAIF01001695.1	0	0	0	0.5	1.56	0.32
GAIF01001696.1	1.6	1.2	0.86	6.73	17.19	0.56
GAIF01001697.1	0.71	1.54	0.2	2.68	2.81	0.03
GAIF01001699.1	1.5	2.18	2	13.72	12.3	0.75
GAIF01001700.1	0	0	0.03	0.09	0.21	0
GAIF01001701.1	9.52	13.28	7.36	33	24.3	2.36
GAIF01001702.1	8.45	4.23	7.05	22.71	32.28	16.48
GAIF01001703.1	0	0	1.21	0	0	0.35
GAIF01001704.1	0	0.1	0	0.53	3.24	0.3
GAIF01001705.1	1.26	0.86	0	0.76	0	0
GAIF01001706.1	0.17	0	0	2.18	11.1	0.28
GAIF01001707.1	0	0	0	0.18	0.19	0
GAIF01001709.1	0	0.3	0.12	0	0.07	0
GAIF01001710.1	0	0	0	0	0.11	0
GAIF01001711.1	0	0.16	0	0.19	0.14	0
GAIF01001712.1	61.34	43.31	90.28	162.42	162.24	273.27
GAIF01001713.1	1.78	0.93	0.07	2.44	3.24	0
GAIF01001714.1	0.09	0	0	0.11	0.79	0.1
GAIF01001715.1	0	0	0	0.35	0.32	0
GAIF01001717.1	0.07	0.02	0	0.04	0.01	0.01
GAIF01001718.1	6.94	12.03	5.59	42.08	21.17	4.98
GAIF01001721.1	1.06	1.46	3.15	2.39	1.51	0
GAIF01001723.1	0	0.18	0	0.32	0.26	0
GAIF01001724.1	1.54	3.3	2.32	3.58	7.61	2.87
GAIF01001725.1	0	0.04	0.14	0	0	0.98
GAIF01001728.1	0	0	0	0.17	0	0
GAIF01001729.1	0	0	0	0	2.35	1.19
GAIF01001733.1	1.79	0.97	0.39	4.77	8.6	3.74
GAIF01001734.1	0	0	0	0.15	0.24	0
GAIF01001738.1	5.43	0.01	0.02	0	1.46	0.01
GAIF01001739.1	0	0.07	0	0.07	0.31	0
GAIF01001741.1	144.68	183.26	557.76	186.47	120.12	303.06
GAIF01001744.1	0	2.5	0	0.77	1.78	0.69
GAIF01001746.1	0	0	0	0.12	0	0.06
GAIF01001747.1	12.79	5.02	6.42	27.67	34.04	4.87
GAIF01001748.1	1.38	0.82	0.73	2.6	17.5	0

GAIF01001750.1	0.14	0	0	0	0	0
GAIF01001752.1	0.7	0.66	0.82	0.16	0.06	0
GAIF01001753.1	2.18	1.67	0.82	0.99	0.25	0
GAIF01001754.1	2.73	1.78	0.15	6.65	10.17	0.45
GAIF01001755.1	8	15.14	16.21	22.74	18.62	6.48
GAIF01001756.1	3.62	3.87	2.2	12.55	14.62	1.96
GAIF01001758.1	0.25	0	0	1.16	9.59	0.27
GAIF01001759.1	0	0.16	0.41	0.09	0	0.16
GAIF01001760.1	54.17	98.87	124.38	19.06	22.98	32.49
GAIF01001761.1	5.29	0.65	0.13	14.94	29.01	0.53
GAIF01001762.1	0	0.2	0	0.1	0.43	0
GAIF01001763.1	27.8	27.55	16.8	50.9	51.27	12.64
GAIF01001764.1	0	0	0	0.14	0	0
GAIF01001765.1	72.79	39.69	26.85	257.24	251.41	85.7
GAIF01001766.1	86.66	61.03	47.73	171.89	115.86	28.55
GAIF01001767.1	0	0	0	0	0.34	0
GAIF01001770.1	0.08	0.18	0.22	0.63	0.5	0.08
GAIF01001772.1	0.1	0	0	0.12	0	0
GAIF01001773.1	0	0	0	0	0.15	0
GAIF01001774.1	0.23	0	0	0.21	0	0
GAIF01001776.1	70.95	46.39	105.42	41.06	14	64.73
GAIF01001779.1	1.86	0	0	0	0	0
GAIF01001780.1	29.14	36.89	51.18	13.53	10.51	80.28
GAIF01001781.1	6.45	6.83	9.95	26.15	32.89	6.93
GAIF01001782.1	0	0	0	0.57	2.13	0
GAIF01001783.1	1.17	0.51	0.41	0.87	0.73	0
GAIF01001784.1	1.99	0	7.49	0.82	1.71	4.19
GAIF01001785.1	0	0	0	0	0.08	0
GAIF01001787.1	0.09	0.05	0.05	0.25	0.21	0.05
GAIF01001788.1	3.08	0	0.57	1.26	2.33	10.24
GAIF01001789.1	26.75	30.53	19.49	13.37	9.53	13.88
GAIF01001790.1	54.18	63.14	66.63	207.35	98.36	53.14
GAIF01001791.1	0	0	0	0	3.82	0
GAIF01001792.1	24.84	22.54	20.36	38.35	31.85	9.06
GAIF01001793.1	2.32	0.18	0	5.2	22.54	4.57
GAIF01001794.1	841.99	5342.08	4582.37	40.22	126.63	218.44
GAIF01001796.1	7.54	16.95	10.1	30.1	14.6	3.24
GAIF01001797.1	15816.06	12068.87	12032.04	8659.34	2807.82	1858.21
GAIF01001799.1	12.41	10.57	13.99	17.91	15.57	5.27
GAIF01001801.1	4.63	0.28	0	1.5	4.53	2.15
GAIF01001802.1	0.02	0	0	0.03	0	0
GAIF01001804.1	0.07	0.12	0.23	0	0.92	0
GAIF01001805.1	1.29	0	0.12	0.12	0	0.35
GAIF01001806.1	0	0.2	0.15	0.16	0	0.26

GAIF01001807.1	0	0.59	0.9	0	1.57	0.9
GAIF01001808.1	0	0	0	0	0.19	0.28
GAIF01001810.1	2.47	2.41	1.23	9.09	8.09	0.61
GAIF01001811.1	6.66	2.02	0.82	9.57	14.16	3.72
GAIF01001812.1	85.86	110.13	100.71	133.15	119.16	49.42
GAIF01001813.1	0.49	0.45	0	0.08	0	0
GAIF01001815.1	0.19	0.3	0.21	0.98	5.39	0.21
GAIF01001816.1	4.17	4.93	2.66	8.26	5.53	0.37
GAIF01001817.1	0.23	0.34	0.09	1.81	10.33	0.27
GAIF01001818.1	0	0	0	0	0.24	0
GAIF01001819.1	0	0.08	0	0.17	0	0.16
GAIF01001820.1	93.16	149.98	68.02	176.74	126.93	15.81
GAIF01001822.1	0.05	0	0.06	0	2.85	0.48
GAIF01001823.1	0	0	0	0.05	0.32	0.07
GAIF01001825.1	4.44	5.9	1.52	5.36	0.43	0.16
GAIF01001827.1	0.13	0	0	0.84	5.57	0.07
GAIF01001828.1	3.15	11.44	2.92	12.35	9.23	0.35
GAIF01001829.1	0.05	0	0	0	0.29	0
GAIF01001830.1	12.99	5.28	3.08	25.16	38.12	9.77
GAIF01001831.1	13.85	0.25	0.34	21.04	1.1	1.35
GAIF01001832.1	0.11	0	0	0	0	0
GAIF01001833.1	0.01	0	0	0.02	0.18	0
GAIF01001838.1	3.6	1.06	3.67	0.63	0.49	1.79
GAIF01001839.1	0.46	0.17	0	0.38	1.43	0.17
GAIF01001840.1	14.49	4.84	12.66	6.19	9.42	3.52
GAIF01001841.1	0	0	0	0	0.36	0
GAIF01001844.1	0	0	0.27	0	1.06	0.17
GAIF01001845.1	3.01	3.27	4.08	9.44	3.62	1.17
GAIF01001846.1	0.21	0.17	0.58	0.67	0.06	0.06
GAIF01001848.1	0	0.93	0	6.93	14.36	1.41
GAIF01001850.1	8.46	6.21	7.44	1.41	0.64	0.42
GAIF01001851.1	0.06	0	0	0.42	0.14	0
GAIF01001852.1	1.36	0	0.25	0.44	0.09	0
GAIF01001857.1	0.12	0	0	0	0	0
GAIF01001858.1	0	0	0	0.07	0	0
GAIF01001859.1	9.86	10.61	4.21	48.59	58.92	1.21
GAIF01001861.1	72.15	37.31	7.84	100.05	67.24	23.46
GAIF01001863.1	0	0	0	0.14	0	0
GAIF01001865.1	43.83	32.22	45.31	36.07	35.43	20.21
GAIF01001866.1	0.08	0.25	0.08	0	0.09	0
GAIF01001867.1	11	12.82	10.33	28.69	34.5	4.25
GAIF01001868.1	0	0.09	0	0.71	0.06	0.13
GAIF01001869.1	1.67	0	0.92	1.74	0.8	0.55
GAIF01001870.1	2.4	0.51	3.99	1.35	0.19	2.73

GAIF01001872.1	0	0	0	4.41	0	0.2
GAIF01001874.1	6.24	9.93	4.01	24.21	24.17	2.01
GAIF01001875.1	0	0	0	0	0	0.16
GAIF01001877.1	1.49	0.32	0.16	5.42	10.72	0.17
GAIF01001878.1	18.4	27.51	16.76	35.71	27.13	3.64
GAIF01001879.1	10.09	5.23	4.48	2.16	1.73	1.32
GAIF01001880.1	3.03	0.9	0.36	14.92	74.87	8.67
GAIF01001881.1	5.05	7.96	4.21	26.04	23.91	1.98
GAIF01001883.1	40.37	33.28	48.33	87.04	70.09	67.82
GAIF01001884.1	0.11	0.12	0	0	0.06	0
GAIF01001885.1	10.59	7.66	17.67	22.64	13.5	15.1
GAIF01001886.1	0.51	0.42	0.42	0.3	0	0
GAIF01001887.1	0.8	0.87	0	3.83	11.09	0
GAIF01001888.1	1.46	0.41	0.54	2.23	5.88	2.57
GAIF01001889.1	207.51	92.04	70.15	199.07	175.03	76.56
GAIF01001891.1	1.79	0.81	2.54	0.78	0.36	0.58
GAIF01001892.1	2.8	0.09	0	1.43	2.28	1.18
GAIF01001893.1	19.32	75.24	38.15	3.86	1.65	0
GAIF01001894.1	0	0	0	0.01	0.27	0
GAIF01001895.1	7.5	4.41	14.04	18.96	0.04	6.11
GAIF01001898.1	0	0.03	0	0	0	0
GAIF01001902.1	3.09	2.84	0.84	2.6	2.36	0.69
GAIF01001903.1	0.18	0	0.05	0	0	0
GAIF01001904.1	542.17	647.79	909.15	283.08	235.07	1176.44
GAIF01001905.1	35.37	45.74	29.42	0.25	0.53	0
GAIF01001908.1	0	0.06	0	0	0	0
GAIF01001909.1	0	0	0.82	0.37	0.26	0
GAIF01001910.1	0.21	0.22	0.47	1.36	0.77	0.23
GAIF01001912.1	14.78	24.13	15.46	12.98	9.04	1.32
GAIF01001914.1	1.72	0.43	0.15	6.43	24.8	0.74
GAIF01001916.1	0.9	2.5	1.28	0.64	0.66	1.21
GAIF01001917.1	0.93	0.12	2.95	0.08	0.01	1.93
GAIF01001918.1	285.46	326	512.77	260.07	206.55	309.79
GAIF01001919.1	0	0	0	0	0.84	0.15
GAIF01001921.1	0.21	0	0	0	0.51	0
GAIF01001922.1	1.97	2.04	0	10.57	38.51	5.9
GAIF01001923.1	0.12	0	0	0.14	0.45	0
GAIF01001925.1	0.1	0.11	0	0	0.5	0
GAIF01001926.1	2.03	2.71	3.63	1.17	1.43	0.15
GAIF01001928.1	0	0	0	0.47	4.45	0.45
GAIF01001931.1	0	0	0	0.15	0	0.14
GAIF01001932.1	0.07	0	0	0.15	0	0.22
GAIF01001936.1	253.19	175.27	168.45	95.54	72.12	131.52
GAIF01001937.1	401.22	804.99	300.12	310.39	561.4	210.85

GAIF01001938.1	78.35	59.57	41.76	127.5	88.45	25.5
GAIF01001942.1	25.26	24.29	29.01	38.8	25.87	19.62
GAIF01001943.1	0.09	0.05	0.08	0.3	1.16	0.7
GAIF01001945.1	0	0	0	0	0.35	0
GAIF01001947.1	5.49	6.08	2.52	2.55	1.92	2.75
GAIF01001948.1	9.75	2.19	0	22.63	56.82	6.3
GAIF01001949.1	0.15	0	0.39	1.6	5.09	1.97
GAIF01001950.1	0	0	0	0.08	0	0
GAIF01001951.1	0	0	0	0.02	0.02	0
GAIF01001953.1	0.03	0.03	0.06	0.37	0.45	0.06
GAIF01001954.1	0	0	0	0.08	0.44	0
GAIF01001956.1	2668.54	1452.12	2037.81	47.64	27.04	9.79
GAIF01001958.1	0	0	0	0	0.19	0
GAIF01001959.1	4.59	3.03	1.65	7.51	21.7	0.41
GAIF01001960.1	16.84	3.4	1.01	22.92	128.21	296.55
GAIF01001962.1	19.81	18.99	10.65	55.6	57.81	2.93
GAIF01001963.1	0	0	0	0	0.23	0
GAIF01001964.1	0.99	4.3	3.1	2.62	2.55	0.16
GAIF01001965.1	0.35	0	0	0.41	0.85	0
GAIF01001966.1	35.1	45.93	57.1	41.33	40.72	39.19
GAIF01001968.1	1	0.14	0.18	0.1	0	0
GAIF01001969.1	13.19	22.23	15.87	13.51	8.11	1.32
GAIF01001970.1	10.59	18.13	6.39	5.93	7.24	0.41
GAIF01001971.1	97.03	26.65	24.77	10.57	4.72	2.11
GAIF01001973.1	0	0	0	0.09	0.55	0
GAIF01001974.1	0	0.07	0.16	0.09	0.54	0.17
GAIF01001975.1	0	0	0.32	0	0	0
GAIF01001976.1	0	0	0	0	0.13	0
GAIF01001980.1	0	0.1	0	0.59	2.68	0.32
GAIF01001981.1	31.08	19.54	16.71	51.49	35.49	17.55
GAIF01001983.1	2.7	3.26	1.5	7.31	8.11	0.31
GAIF01001984.1	2.32	1.73	0.14	4.46	3.51	0.23
GAIF01001985.1	0.18	0.2	0	0	0.44	0
GAIF01001986.1	0	0	0	0	0.13	0
GAIF01001987.1	1.12	1.46	0.74	1.82	1.75	0
GAIF01001991.1	0.18	0.02	0.1	0.05	0	0.02
GAIF01001992.1	2.89	0	2.77	0.8	0	0.14
GAIF01001993.1	52.16	51.64	5.81	60.87	61.08	15.22
GAIF01001994.1	4.12	2.55	4.75	2.92	4.53	21.9
GAIF01001998.1	1.61	0	0	1.87	4.2	0
GAIF01001999.1	20	31.32	41.66	30.28	29.47	15.75
GAIF01002000.1	15.39	12.59	19.54	25	15.09	14.67
GAIF01002001.1	0.03	0	0	0	0	0
GAIF01002002.1	0	0	0	0	0	0.06

GAIF01002003.1	0.53	0	0	0.2	0.32	0
GAIF01002004.1	117.8	33.39	44.46	69.24	49.95	214.62
GAIF01002005.1	0	0.09	0	0	0	0
GAIF01002006.1	1.08	0.69	0	2.93	5.19	0
GAIF01002007.1	0	0	0.64	1.27	0.51	0
GAIF01002009.1	0	0	0	0.17	0	0
GAIF01002010.1	4.08	11.96	6.04	7.35	8.82	0.64
GAIF01002011.1	0	0	0	0	0	0.1
GAIF01002012.1	0.31	0.33	0.07	0.21	0.59	0
GAIF01002013.1	0.94	0	0	2.98	20.77	0.27
GAIF01002014.1	4.9	2.2	2.8	5.98	0	0
GAIF01002016.1	4.2	7.35	2.61	15.26	21.19	0.3
GAIF01002019.1	4.84	5.01	3.54	17.66	10.26	1.27
GAIF01002020.1	4.27	1.63	3.03	1.43	0.85	0.27
GAIF01002021.1	0.31	0	0.17	1.07	1.29	0
GAIF01002023.1	1664.73	1919.6	2497.86	273.97	169.7	196.45
GAIF01002024.1	76.98	123.44	96.16	191.56	142	78.71
GAIF01002025.1	19.92	28.75	25.35	11.66	12.84	3.18
GAIF01002027.1	0.26	0.05	0	0	0	0
GAIF01002029.1	0.33	0.72	0.36	2.87	4.95	1.27
GAIF01002031.1	0.32	0.32	0.36	0.19	0.04	0
GAIF01002032.1	0	0	0	1.1	5.39	0.9
GAIF01002033.1	0	0.01	0	0	0	0
GAIF01002034.1	12.23	8.95	7.85	2.92	0.65	1.2
GAIF01002036.1	0.38	0.12	0	0.57	6.65	0.3
GAIF01002038.1	0.06	0	0	0	0	0
GAIF01002039.1	5.25	16.79	4.86	34.59	42.37	2.09
GAIF01002042.1	9.72	5.79	11.62	7.69	9.96	17.83
GAIF01002043.1	1.87	3.65	1.63	1.34	2.71	0.23
GAIF01002044.1	0	0	0	0	1.24	0
GAIF01002045.1	0.03	0.06	0	0	0	0.03
GAIF01002046.1	0	0	0.05	0	0	0
GAIF01002047.1	0.05	0.32	0	0	0	0
GAIF01002048.1	0	0.15	0	0	0	0
GAIF01002049.1	0.06	0.03	0.13	0.11	0.37	0.03
GAIF01002050.1	0.11	0.24	0.06	0.04	0.04	0
GAIF01002051.1	7.52	9.33	4.64	16.23	18.72	1.89
GAIF01002052.1	0.42	0.36	0.26	0.82	0.85	1.24
GAIF01002053.1	0	0	0	0	0.26	0
GAIF01002054.1	2.51	3.26	0.53	13.3	24.33	0.92
GAIF01002055.1	0.68	0.63	0	0	0	0
GAIF01002057.1	18.62	35.36	14.73	49.87	48.8	4.08
GAIF01002058.1	4.97	2.97	2.57	11.3	21.55	3.65
GAIF01002059.1	5.85	4.28	3.51	11.36	7.04	1.17

GAIF01002060.1	0	0	0.16	0	0	0
GAIF01002061.1	36.61	7.92	8.89	22.61	43.73	171.99
GAIF01002062.1	0	0	0	0.13	0.27	0.12
GAIF01002063.1	333.43	575.24	389.49	313	301.97	69.05
GAIF01002068.1	0.11	0.3	0.37	0.39	0.14	0
GAIF01002069.1	0.32	0.2	0	0.11	0.05	0
GAIF01002070.1	0	0	0	0.09	0	0
GAIF01002072.1	0.04	0	0	0	0	0
GAIF01002074.1	13.54	29.44	25.6	28.74	20.98	5.1
GAIF01002075.1	44.61	48.83	58.38	125.67	135.93	39.35
GAIF01002077.1	6.25	15.52	10.62	13.97	14.19	0.26
GAIF01002078.1	0	0	0.06	0	0.06	0
GAIF01002080.1	0	0	0	0	2.97	0
GAIF01002081.1	0.29	0.87	0.44	0.13	6.29	0.06
GAIF01002082.1	2.56	0.68	0.14	6.78	31.08	1.1
GAIF01002084.1	0.11	0	0	0.09	0	0.21
GAIF01002086.1	0.12	0.18	0	2.27	4.67	0.64
GAIF01002087.1	261.31	202.76	221	249.33	152.8	72.47
GAIF01002090.1	15.47	14.76	24.65	27.23	14.8	16.49
GAIF01002091.1	7.14	11.85	9.22	33.02	16.73	10.36
GAIF01002092.1	0.08	0.06	0.12	0.71	0.92	0
GAIF01002093.1	7.6	4.52	1.17	4	2.29	0.52
GAIF01002095.1	2.03	1.32	0.09	0.19	0.39	0.09
GAIF01002096.1	13.82	12.13	7.99	66.85	35.73	5.21
GAIF01002098.1	0.5	0.11	0.11	2.32	17.68	0.77
GAIF01002100.1	4.28	0.07	0.15	13.71	45.94	1.43
GAIF01002101.1	5.14	0.59	0.37	12.94	22.35	43.31
GAIF01002102.1	2.17	1.32	1.02	6.28	13.43	1.53
GAIF01002104.1	0	0	0	0	0.05	0
GAIF01002105.1	0	0.45	0.98	0	0	1.29
GAIF01002108.1	0.63	0.28	0.03	4.9	15.85	0.14
GAIF01002109.1	0	0.26	0	0.14	0.58	0
GAIF01002111.1	22.92	29	8.52	76.72	80.07	8.04
GAIF01002112.1	0	0.1	0	0	0	0
GAIF01002114.1	297.24	271.97	102.32	499.73	696.16	226.57
GAIF01002115.1	0	0	0.2	0.59	0	0
GAIF01002116.1	10.13	17.66	10	33.87	27.83	3.41
GAIF01002118.1	0	0	0.03	0.07	0.49	0
GAIF01002120.1	0	0	0	0	0.13	0
GAIF01002121.1	1.95	1.09	0.64	1.72	11.39	1.53
GAIF01002122.1	11.67	18.83	18.1	38.84	15.54	7.86
GAIF01002123.1	13.16	3.87	4.94	12.54	4.2	2.81
GAIF01002124.1	0	0	0	0.15	0.15	0
GAIF01002125.1	0	0	0	0	0.16	0

GAIF01002127.1	16.97	9.36	0	244.12	66.03	6.56
GAIF01002128.1	13.01	0	0	6.65	3.98	1.69
GAIF01002129.1	2.12	0.47	0.8	1.95	0	1.72
GAIF01002130.1	0.48	0	0	0	0	0
GAIF01002134.1	0.04	0.08	0.04	0.08	0.17	0.08
GAIF01002135.1	76.76	49.42	36.4	40.22	23.82	13
GAIF01002139.1	15.91	9.91	0.07	31.05	20.63	19.74
GAIF01002140.1	0	0	0	0.33	2.22	0.16
GAIF01002141.1	0.48	0.09	0	2.05	10.92	0.09
GAIF01002145.1	0.4	0.07	0	6.01	33.85	1.4
GAIF01002146.1	0	0.16	0.33	0	0.18	0
GAIF01002148.1	0.47	0.65	1.58	3.01	5.28	0.69
GAIF01002149.1	25.9	32.54	20.63	37.64	36.74	34.9
GAIF01002150.1	1.17	1.75	0.32	3.56	11.5	0.4
GAIF01002151.1	2.92	0.8	0	6.45	25.99	0.8
GAIF01002153.1	0	1.23	0.83	0	0	0.21
GAIF01002154.1	0	0	0.33	0	0	0
GAIF01002155.1	10.59	14.2	16.18	23.41	13.9	5.2
GAIF01002156.1	0.06	0	0	0	0	0
GAIF01002157.1	1.04	0.19	0.95	0.66	0	0.33
GAIF01002159.1	0	0	0	0	5.86	0
GAIF01002160.1	0	0	0	0.04	0.05	0
GAIF01002161.1	1.53	1.58	2.74	12.52	55.38	2.75
GAIF01002162.1	0.37	0.2	0	3.67	17.69	0.41
GAIF01002164.1	13.08	9.98	5.59	21.09	21.28	4.4
GAIF01002165.1	16.17	12.05	19.54	42.3	23.29	47.35
GAIF01002166.1	0.11	0	0.04	0.32	0.21	0.04
GAIF01002167.1	63.11	48.81	60.7	74.98	54.5	27.4
GAIF01002168.1	11.15	10.28	12.97	28.06	21.66	9.64
GAIF01002169.1	2.43	0.79	0.11	4.91	8.88	0.1
GAIF01002172.1	89	115.84	101	13.76	6.21	3.35
GAIF01002174.1	0	0.12	0	0	0	0
GAIF01002176.1	7.14	6.67	8.17	14.94	17.07	6.65
GAIF01002177.1	3.58	3.01	1.49	6.65	5.02	0.77
GAIF01002178.1	103.21	183.12	41	61.53	123.71	60.7
GAIF01002179.1	19.77	22.69	20.89	49.95	46.31	5.97
GAIF01002180.1	19.09	15.23	19.43	43.47	47.15	19.23
GAIF01002181.1	7.82	10.67	6.73	6.59	8.72	2.47
GAIF01002184.1	15.97	22.38	27.38	35.37	19.99	9.31
GAIF01002185.1	0.25	0	0	0.29	0.31	0
GAIF01002186.1	0.16	0.1	0	1.04	3.67	0.21
GAIF01002187.1	56.86	56.43	31.16	13.82	5.73	0.87
GAIF01002190.1	0.09	0.1	0	0	0.59	0
GAIF01002191.1	0.5	0.48	0.1	6.07	20.56	0.14

GAIF01002192.1	122.01	173.82	225.68	108.92	83.42	83.24
GAIF01002193.1	9.37	6.01	7.17	25.28	27.87	7.3
GAIF01002194.1	20.7	36.74	32.13	59.37	45.5	15.21
GAIF01002195.1	2.09	1.14	0.38	10.25	46.06	1.54
GAIF01002196.1	12.6	17.55	19.27	23.82	18.39	12.95
GAIF01002197.1	0.46	0.05	0	0.26	2.47	3.07
GAIF01002201.1	4.2	1.32	2.56	20.02	7.4	9.39
GAIF01002202.1	3.64	4.52	1.91	37.09	31.14	2.62
GAIF01002204.1	4.41	6.71	2.07	0.29	0.15	0
GAIF01002205.1	0	0	0	0	0.11	0
GAIF01002206.1	3.25	0.58	0.47	2.6	3.32	1.11
GAIF01002207.1	0	0.28	0.86	0	0	0.48
GAIF01002208.1	12.19	14.79	15.71	11.69	4	2.67
GAIF01002210.1	32.97	12.54	32.45	24.91	20.4	10.91
GAIF01002211.1	27.15	0.87	0.44	1.08	0.16	0
GAIF01002213.1	3.93	3.18	3.15	11.82	17.87	4.94
GAIF01002216.1	1.41	0.59	0.36	1.76	2.87	0.6
GAIF01002218.1	0.14	0	0	0	0	0
GAIF01002219.1	138.6	74.66	33.16	221.36	224.4	47.41
GAIF01002220.1	0	0	0	0	0.02	0
GAIF01002221.1	0	0.14	0	0	0	0
GAIF01002222.1	0	0.05	0.05	0	0.1	0
GAIF01002224.1	1516.88	1663.72	2424.51	1243.68	653.7	471.15
GAIF01002226.1	3.64	22.78	9.36	1.72	1.3	0.53
GAIF01002227.1	26.2	24.4	25.84	57.82	40	11.67
GAIF01002229.1	0	0.03	0	0.15	0.19	0.07
GAIF01002230.1	0.73	0.1	0.1	5.51	30.08	0.4
GAIF01002231.1	0	0	0	0.09	1.39	0.17
GAIF01002232.1	0	0	0	0.07	0	0
GAIF01002233.1	0	0.06	0	0.12	0	0
GAIF01002235.1	0	0	0.12	0.12	1.07	0.11
GAIF01002237.1	0.35	0.09	0.24	0.15	0.21	0.02
GAIF01002238.1	0	0.54	0.37	8.11	74.35	0.37
GAIF01002239.1	1.86	1.98	0.5	8.41	17.89	2.11
GAIF01002240.1	0	0	0.03	0.03	0	0
GAIF01002241.1	19.46	28.08	23.45	65.1	84.62	14.18
GAIF01002242.1	0	0.76	1.92	0	0	3.15
GAIF01002243.1	0.13	0.23	0.16	0.44	0.8	0
GAIF01002244.1	37.68	48.51	18.6	69.11	72.81	7.84
GAIF01002245.1	17.78	5.71	4.36	21.54	33.86	20.63
GAIF01002246.1	0	0.06	0.06	0	1.28	0.12
GAIF01002249.1	3.08	12.15	2.54	7.9	10.81	0.14
GAIF01002251.1	0.28	0.31	0	3.31	12.22	0
GAIF01002252.1	0	0	0	0	0.17	0

GAIF01002253.1	0	0	0	0	0.18	0
GAIF01002254.1	51.24	54.45	36.09	84.41	105.95	37.08
GAIF01002255.1	34.31	53.68	43.75	23.49	35.9	11.16
GAIF01002257.1	0.31	0.31	0.1	1.2	4.61	0.68
GAIF01002258.1	0	0	0	0	1.81	0
GAIF01002259.1	0.59	0.1	0	0.99	4.46	4.02
GAIF01002261.1	0	0	0	0.11	0.22	0
GAIF01002262.1	7.37	9.37	18.2	4.39	2.64	9.55
GAIF01002263.1	0.13	0	0.07	149.12	0	0
GAIF01002264.1	0.45	0.28	0.1	2.54	7.49	0.25
GAIF01002265.1	33.31	24.26	3.51	6.27	2.15	0.13
GAIF01002266.1	0	0.03	0	0	0	0
GAIF01002268.1	0	0	0	0	0	0.09
GAIF01002269.1	7.82	6.94	5.11	27.16	21.94	2.7
GAIF01002270.1	0.03	0	0	0	0.02	0.02
GAIF01002271.1	0.05	0	0	0	0.09	0
GAIF01002273.1	0	0.15	0	0.95	2.13	0
GAIF01002274.1	13.99	9.23	8.96	36.13	38.26	1.6
GAIF01002275.1	168.85	243.43	238.55	106.6	89.88	85.48
GAIF01002276.1	0.29	0	0.13	1.42	4.42	0.26
GAIF01002278.1	96.04	64.1	61.28	118.64	76.44	32.23
GAIF01002280.1	0.62	0.86	0.61	1.58	0.11	0.18
GAIF01002282.1	0	0.04	0.04	0	0.07	0
GAIF01002283.1	0	0	0	0	1.61	0
GAIF01002284.1	32.26	39.92	42.64	67.8	51.16	22.18
GAIF01002285.1	0	0	0	0.2	0	0
GAIF01002286.1	0.3	0.56	0.18	0.6	0.06	0.42
GAIF01002287.1	14.54	6.79	8	16.73	20.86	24.29
GAIF01002288.1	0.12	0	0	0.2	0.07	0.7
GAIF01002290.1	9.5	10.75	10.43	17.38	15.85	4.2
GAIF01002291.1	0	0.18	0	0.38	0.98	0.39
GAIF01002293.1	0.31	0.25	0.08	1.25	3.88	0
GAIF01002295.1	1.87	0.35	1.07	3.4	5.77	3.14
GAIF01002297.1	0.06	0	0.07	0.07	0.29	0
GAIF01002298.1	0.5	0	0	0	0.6	0.14
GAIF01002299.1	0	0	0.06	0.12	1.11	0
GAIF01002303.1	0	0	0	0	0.1	0
GAIF01002304.1	0.06	0.03	0.07	0.21	0.89	0.03
GAIF01002305.1	11.21	8.38	15.98	14.07	9.74	20.16
GAIF01002306.1	0	0	0.12	0.66	3.06	0
GAIF01002307.1	0	1.15	0	2.27	2.16	0
GAIF01002308.1	0.39	0	0	1.02	12.49	0.86
GAIF01002309.1	0	0.52	0.33	0	0	0
GAIF01002311.1	0.72	0.46	0	0.28	0.5	0

GAIF01002312.1	10.1	5.34	12.87	22.51	55.15	61.35
GAIF01002314.1	0.39	0.11	0.05	0.96	4.9	0.05
GAIF01002316.1	1.14	2.21	0.98	4.01	27.71	0.56
GAIF01002318.1	12.56	23.35	15.13	34.11	19.46	4.74
GAIF01002319.1	42.82	58.92	120.24	51.35	58.88	49.22
GAIF01002320.1	11.69	6.14	6.85	4.71	2.86	2.95
GAIF01002321.1	0.07	0.07	0	0.15	0.08	0
GAIF01002323.1	12.73	9	12.41	34.62	56.58	30.69
GAIF01002324.1	3.32	3.88	6.97	10.25	5.13	3.22
GAIF01002325.1	0.57	0	0	0	0.14	0
GAIF01002326.1	5.08	3.99	2.48	14.19	11.31	0.52
GAIF01002327.1	0	0.04	0	0.29	0.93	0.08
GAIF01002329.1	0.29	0.28	0.41	0.35	0.9	0.35
GAIF01002330.1	508.74	214.08	786.72	22.16	6.96	62.11
GAIF01002331.1	5.24	5.7	0	6.09	5.18	0
GAIF01002332.1	13.84	29.09	25.56	29.02	53.01	32.01
GAIF01002333.1	0	0.16	0	0	0.18	0
GAIF01002334.1	0.16	0.07	0	0.6	2.92	0.14
GAIF01002336.1	0.69	0	0.08	2.93	10.6	0.08
GAIF01002338.1	0.52	0.11	0.11	1.33	1	0.23
GAIF01002339.1	0.33	0.73	0.55	2.23	3.91	0
GAIF01002340.1	31.5	29.96	22.61	107.77	59.43	11.67
GAIF01002341.1	0	0	0	3.21	4.28	0.98
GAIF01002342.1	2.75	9.05	5.25	13.86	11.84	0.88
GAIF01002343.1	0.53	0	0	1.03	4.06	0.05
GAIF01002344.1	0	0.15	0	0.55	0.19	0.05
GAIF01002345.1	0.85	0	0	0	1.76	0
GAIF01002346.1	22.16	25.92	19.31	37.94	26.09	4.91
GAIF01002347.1	0	0	0	0.36	0.84	0
GAIF01002348.1	1.32	0.56	1.03	0.35	0.1	0.04
GAIF01002349.1	0.92	0.6	0	2.45	8.19	1.48
GAIF01002350.1	0	0	0	0	0.49	0
GAIF01002351.1	3.32	4.57	0	1.05	1.2	0.03
GAIF01002352.1	0.57	0.67	0.32	0.72	0	0.16
GAIF01002353.1	1.15	2	0.88	3.47	3.88	0.51
GAIF01002354.1	1.26	0.37	0.18	4.45	28.62	72.19
GAIF01002355.1	11.48	11.88	6.36	6.61	4.09	1.47
GAIF01002356.1	0.16	0.43	0.04	0	0.13	0
GAIF01002357.1	14.07	6.64	8.77	13.27	5.47	25.75
GAIF01002358.1	0.11	0.06	0.06	0.13	0.34	0.06
GAIF01002360.1	0.11	0.33	0.62	0	0.59	0
GAIF01002361.1	3.38	5.5	1.45	9.73	23.97	0
GAIF01002363.1	0	0	0	0	0.11	0
GAIF01002364.1	5.62	12.66	6.42	24.91	24.95	2.58

GAIF01002366.1	3.36	2.96	0	23.05	47.18	0.18
GAIF01002367.1	67.47	65.76	91.14	76.31	77.82	85.75
GAIF01002368.1	15.81	9.13	6.62	32.38	16.28	104.07
GAIF01002369.1	6.95	0.83	0	14.65	14.48	26.97
GAIF01002370.1	22.87	27.03	56.49	3.24	1.92	5.86
GAIF01002371.1	29.2	20.72	9.67	39.71	41.11	8.2
GAIF01002372.1	29.53	16.95	25.18	1.74	0.55	0.51
GAIF01002373.1	104.15	133.78	155.4	140.72	115.24	65.87
GAIF01002374.1	0.06	0.21	0.1	0.11	0.27	0.02
GAIF01002375.1	0.7	0.19	0	4.27	12.54	1.06
GAIF01002377.1	1568.46	1193.32	686.68	307.52	201.18	171.19
GAIF01002378.1	0.07	0	0	0	0	93.79
GAIF01002381.1	0.07	0	0	0.04	0.04	0.08
GAIF01002382.1	0.8	0	0	0.59	0.85	0
GAIF01002383.1	17.51	11.76	15.08	31.88	13.83	7.38
GAIF01002385.1	0	0	0	0.28	0.29	0
GAIF01002386.1	5.5	5.23	4.68	5.74	3.64	1.06
GAIF01002388.1	0	0.18	1.12	0	0.04	0.4
GAIF01002389.1	0	0	0	0.06	0.05	0
GAIF01002390.1	74.42	70.01	14.31	150.34	154.76	12.32
GAIF01002391.1	0	0.07	0	0	0.16	0
GAIF01002392.1	192.31	76.04	76.77	81.16	189.22	38.53
GAIF01002393.1	9.53	9.98	6.28	29.76	27.95	5.25
GAIF01002395.1	0	0	0	0	0.05	0
GAIF01002397.1	0	0.03	0	0.04	0.04	0
GAIF01002398.1	0.11	0.06	0	0	0	0.13
GAIF01002399.1	59.74	19.15	10.84	0	0	0.12
GAIF01002400.1	11.38	11.06	11.98	22.99	25.15	18.89
GAIF01002401.1	84.28	167.91	110.3	148.63	151.29	28.72
GAIF01002403.1	2.01	3.68	1.91	6.17	3.66	0.35
GAIF01002405.1	56.61	47.15	43.42	151.33	72.67	22.79
GAIF01002406.1	0.01	0.1	0.01	0.02	0.2	0.01
GAIF01002408.1	0.2	0.06	0	0.07	0.15	0.07
GAIF01002409.1	22.76	17.65	22.89	29.72	25.41	33.21
GAIF01002411.1	0.85	0.4	0.76	0.85	0.29	0.04
GAIF01002413.1	0	0	0	0.26	0.38	0
GAIF01002414.1	0	0.06	0	0	0	0
GAIF01002415.1	24.94	10.95	11.39	9.68	8.85	4.51
GAIF01002417.1	0	0.11	0.22	0.12	0.12	0
GAIF01002419.1	0	0	0	0.26	0	0
GAIF01002420.1	1.57	0.2	0.15	3.49	11.62	1.02
GAIF01002421.1	1.15	0.63	0.18	6.1	15.71	1.36
GAIF01002423.1	0.17	0	0	0.39	0.2	0
GAIF01002425.1	0	0	0	0.1	0.32	0.1

GAIF01002427.1	6.78	0.59	1.98	4.4	0.22	1.59
GAIF01002428.1	0.12	0	0	0	0	0
GAIF01002429.1	0.4	0.57	0.22	0.97	1.43	0
GAIF01002431.1	0	0	0	0	0	1.76
GAIF01002433.1	7.96	30.97	0	10.53	0	7.94
GAIF01002434.1	3.33	2.61	3.82	4.72	5.52	1.69
GAIF01002435.1	0	0	0	0.32	0	0.31
GAIF01002439.1	1.03	0.09	0.19	0.65	4.12	0
GAIF01002440.1	0	0.03	0.07	0	0	0
GAIF01002441.1	0.28	0	0	3.29	17.42	0.06
GAIF01002443.1	21.04	40.5	13.36	50.37	50.91	4.71
GAIF01002444.1	48.2	77.38	60.53	86.34	53.36	16.74
GAIF01002445.1	0.26	0.39	0	0.38	0	0
GAIF01002446.1	1.67	1.11	0.32	2.09	3.06	0.16
GAIF01002448.1	39.72	56.52	24.87	129.81	79.84	11.69
GAIF01002449.1	0	0	0	0.05	0	0
GAIF01002450.1	0.1	0	0	1.35	7.74	0
GAIF01002451.1	0.35	0	0	1.37	6.95	4.68
GAIF01002452.1	0.81	0.4	0.25	0.06	0.33	0.04
GAIF01002454.1	12.7	0	1.23	14.95	4.55	4.94
GAIF01002456.1	0.09	0.14	0	0	0	0
GAIF01002458.1	1.94	2.53	2.13	5.7	5.29	0.86
GAIF01002459.1	371.91	209.79	211.06	516.35	380.05	2091.38
GAIF01002460.1	0.1	0.11	0	0.12	1.56	0.22
GAIF01002462.1	0.07	0	0	0.25	0.88	0
GAIF01002463.1	4.88	7.84	4.42	2.09	2.55	0.58
GAIF01002464.1	0	0	0	0.15	0	0
GAIF01002466.1	15.46	15.41	11.55	26.42	31.89	47.68
GAIF01002467.1	0.08	0	0	0	0.18	0
GAIF01002468.1	8.13	11.59	10.05	13.38	17.67	3.99
GAIF01002469.1	0.98	0	0.14	3	14.38	1.37
GAIF01002470.1	0.3	0.08	0	1.11	7.74	0.16
GAIF01002474.1	0.23	0	0	0.49	0.37	0
GAIF01002475.1	0.32	0	0	0.5	0	0.24
GAIF01002476.1	0	2.08	1.07	3.32	5.72	0
GAIF01002477.1	0.21	0.05	0.05	0.39	1.33	0.19
GAIF01002478.1	1	0.4	0	1.9	1.56	1.28
GAIF01002479.1	0	0	0	0.03	0.04	0
GAIF01002480.1	0.03	0	0.06	0.33	1.42	0.09
GAIF01002482.1	0	0	0	0	0.21	0
GAIF01002483.1	0	0.15	0	0.4	2.29	0
GAIF01002484.1	0	0	0	0.6	0.79	0
GAIF01002485.1	2.78	0.98	1.85	3.4	3.13	0.66
GAIF01002486.1	0.09	0	0	0	0	0

GAIF01002487.1	0	0.03	0	0	0	0
GAIF01002489.1	6.33	7.41	1.76	6.07	4.58	0.31
GAIF01002490.1	0	0	0	0.06	0.03	0
GAIF01002491.1	0.77	0.12	0.44	4.49	0	1.43
GAIF01002492.1	0	0	0	0	15.77	71.82
GAIF01002493.1	0	0.03	0.18	0.1	0.03	0.03
GAIF01002496.1	0.16	0.15	0.05	0.4	1.02	0.1
GAIF01002497.1	16.35	21.19	35.86	98.49	37.97	42.67
GAIF01002498.1	0	0.03	0	0	0	0
GAIF01002499.1	0.18	0	0	0	0	0
GAIF01002500.1	0	0.09	0.09	0.48	0.49	0
GAIF01002501.1	17.47	12.02	13.96	45	23.83	37.56
GAIF01002502.1	0.18	0	0.2	0.93	0.64	0
GAIF01002504.1	0	0.08	0	0	0	0
GAIF01002506.1	333.48	172.19	291.27	145.36	72.13	152.5
GAIF01002507.1	0	0.12	0	0	0	0
GAIF01002508.1	0	0.09	0.04	0	0	0
GAIF01002509.1	31.76	22.63	37.56	10.88	6.94	7.28
GAIF01002515.1	0.08	0.05	0	0	0.1	0
GAIF01002516.1	0.05	0	0	0	0	0
GAIF01002517.1	0.67	0	0	0.77	3.47	0.73
GAIF01002518.1	0	0	0	0.25	0	0
GAIF01002519.1	1.1	0.78	0.94	6.53	16.68	1.58
GAIF01002520.1	40.15	36.47	24.54	24.08	37.34	4.27
GAIF01002521.1	15.43	8.85	34.73	17.16	12.75	0
GAIF01002522.1	1.33	2.24	0.12	4.07	12.08	0.25
GAIF01002524.1	0	0	0	0.05	0.53	0.63
GAIF01002525.1	0.21	0.15	0.08	0.24	3.81	0
GAIF01002527.1	0.06	0.12	0.25	1.55	2.28	0.62
GAIF01002528.1	211.26	226.87	55.25	70.37	164	12.96
GAIF01002530.1	0.18	0	0.1	0	0	0.19
GAIF01002533.1	0.14	0	0.14	0.25	0	0
GAIF01002534.1	0	0	0.36	0.88	1.69	0
GAIF01002536.1	0.96	1.6	0.06	9.33	26.83	0.34
GAIF01002537.1	0	0.2	0	0	0.62	2.22
GAIF01002538.1	18.69	22.07	17.72	34.12	24.23	11.53
GAIF01002539.1	1.81	1.57	0.4	10.21	19.56	0.2
GAIF01002540.1	0	0	0.07	0	0	0
GAIF01002541.1	7.97	5.82	5.02	9.25	25.92	20.87
GAIF01002544.1	0	0.1	0	0	0.05	0
GAIF01002545.1	0	0	0.17	0	0	0
GAIF01002546.1	0	0	0	0	0.87	0
GAIF01002548.1	0.85	0.73	1.6	0.71	0.8	0.2
GAIF01002549.1	3.67	4.34	1.68	5.66	6.22	1.43

GAIF01002550.1	21.88	10.25	14.81	20.98	17.32	21.95
GAIF01002552.1	0.23	0	0	0.14	0	0
GAIF01002553.1	0	0	0.09	0.28	1.11	0
GAIF01002555.1	0	0	0	0.67	0.17	0
GAIF01002556.1	0	0	0	0	0.32	0
GAIF01002558.1	0.06	0.88	0.92	0	0.1	0.18
GAIF01002559.1	5.03	2.49	3.41	1.47	0.66	0
GAIF01002561.1	4.36	1.92	1.79	8.16	2.69	7.97
GAIF01002562.1	2	4.95	4.19	1.67	0.65	0.32
GAIF01002565.1	0	0	0	0	4.36	2.3
GAIF01002566.1	17.99	10.45	25.51	33.84	15.58	27.95
GAIF01002567.1	4.37	0	0	4.78	5.33	4.54
GAIF01002568.1	0	0	0	0	0.16	0
GAIF01002569.1	26.9	39.62	18.91	52.2	33.38	2.82
GAIF01002570.1	0.22	0.03	0.04	0.14	0.06	0.38
GAIF01002571.1	1.01	3.61	0.56	1.86	4.68	0.24
GAIF01002572.1	0	18.58	12.46	0	0	3.52
GAIF01002573.1	0.5	0.55	0	1.47	8.08	0.06
GAIF01002574.1	0	0	0	0	0.31	0
GAIF01002575.1	28.9	53.71	35.73	71.89	67.55	9.45
GAIF01002576.1	0.4	0	0	0.23	0.48	0
GAIF01002577.1	25.75	126.51	68.32	27.14	6.03	2.48
GAIF01002578.1	0.8	0	0.52	0.86	5.9	0.52
GAIF01002580.1	30.68	21.41	17.04	58.45	44.3	15.24
GAIF01002581.1	0.2	0.2	0	2.58	12.11	0.11
GAIF01002582.1	0	0	0	79.65	0	0.13
GAIF01002583.1	12.17	15.81	8.26	42.33	52.98	4.09
GAIF01002584.1	0.22	0.1	0.01	0.05	0.01	0.04
GAIF01002585.1	109.69	110.94	70.09	12.12	12.61	22.79
GAIF01002586.1	0	0	0	0	0	0.42
GAIF01002587.1	0	0	0	0	0	0.38
GAIF01002589.1	0.55	0	0	3.66	19.13	0.76
GAIF01002592.1	198.18	84.25	54.18	103.84	117.85	69.47
GAIF01002593.1	0	0.07	0	0.71	2.06	0.04
GAIF01002594.1	0	1.16	0.55	0.15	0	0.05
GAIF01002595.1	0	0	0	0	0	67.84
GAIF01002596.1	32.45	17.18	23.42	44.76	51.13	64.18
GAIF01002599.1	0.14	0	0	0.08	0.09	0
GAIF01002601.1	28.83	45.19	31.49	18.62	21.67	2.56
GAIF01002603.1	7.22	3.86	2.69	29.83	37.09	1.08
GAIF01002605.1	0	0	0	0	0.27	0
GAIF01002606.1	23.17	15.28	12.03	51.99	58.46	3.53
GAIF01002607.1	14.63	25.37	5.83	49.81	60.02	2.77
GAIF01002608.1	158.41	154.05	219.76	50.27	24.75	30.63

GAIF01002609.1	0.1	0	0.05	0.17	0	0.05
GAIF01002610.1	5.24	2.92	3.37	6.67	2.92	5.91
GAIF01002611.1	317.39	355.87	402.85	785.49	632.15	253.46
GAIF01002613.1	13.5	6.87	13.22	28.86	44.54	50.98
GAIF01002614.1	0.15	0	0	0.09	0.54	0
GAIF01002615.1	0	0	0	0	0.11	0
GAIF01002616.1	0.13	0.14	0	0.31	0.8	0.15
GAIF01002617.1	0.31	0	0	3.98	17.06	0.86
GAIF01002618.1	1.53	1.66	0	12.79	10.49	4.77
GAIF01002619.1	0.69	0.22	0	8.12	6.47	1.13
GAIF01002621.1	11.34	2.6	2.9	2.47	2.13	1.71
GAIF01002622.1	0.56	0.18	0.18	2.9	6.58	1.24
GAIF01002623.1	15.39	23.18	14.64	68.57	37.78	4.46
GAIF01002624.1	1.04	0	0.11	0.36	0.35	0.19
GAIF01002625.1	11.43	10.46	5.46	25.88	22.24	3.31
GAIF01002627.1	0.61	0	0.4	0.99	10.82	0.13
GAIF01002628.1	1.26	0.31	0.71	2.22	0.67	0.35
GAIF01002629.1	0.21	0.38	0.36	0.55	0.21	0.16
GAIF01002630.1	0.22	0	0	2.02	5.51	0.24
GAIF01002631.1	32.8	15.99	4.75	59.89	54.76	24.86
GAIF01002632.1	2.22	0.19	0.09	12.86	27.69	0.18
GAIF01002633.1	149.27	84.7	19.68	86.24	174.83	12.13
GAIF01002634.1	161.45	43.06	27.97	14.53	19.5	8.19
GAIF01002635.1	13.19	9.09	6.57	24.18	10.58	3.89
GAIF01002636.1	0	0	0	0	0.05	0
GAIF01002638.1	0.91	0	0	0	0	0
GAIF01002639.1	16.67	27.12	20.09	19.06	14.81	5.33
GAIF01002640.1	0.05	0.13	0	0.12	0.33	0
GAIF01002642.1	0	0	0	0	0	0.03
GAIF01002643.1	0	0	0	0.16	0	0.3
GAIF01002647.1	0	0	0	0.29	1.25	0.06
GAIF01002648.1	0.02	0.06	0	0	0	0
GAIF01002649.1	0.03	0	0	0	0	0
GAIF01002653.1	0	0	0	0	0	0.05
GAIF01002654.1	1.34	0	0	6.14	21.51	0.16
GAIF01002656.1	191.54	65.09	71.63	209.11	65.46	18.88
GAIF01002657.1	0.22	0	0	0	2.17	0.41
GAIF01002658.1	0.2	0	0	0.06	0	0
GAIF01002659.1	24.46	10.55	10.44	31.95	27.41	14.73
GAIF01002660.1	0	0.35	0	0	0	0
GAIF01002661.1	182.05	115.41	130.19	16.32	6.73	8.91
GAIF01002663.1	1.62	0.96	0.81	0.17	0	0.16
GAIF01002664.1	11.82	4.13	2	18.67	4.76	1.38
GAIF01002666.1	512.9	46.01	74.75	275.42	115.17	710.29

GAIF01002669.1	118.4	23.16	54.23	74.08	76.65	236.83
GAIF01002670.1	10.58	16.37	18.12	22.52	10.76	6.93
GAIF01002671.1	26.46	13.56	16.8	41.85	56.24	157.06
GAIF01002672.1	0.54	0.29	0.07	1.54	6.17	0
GAIF01002673.1	0	0	0	0.39	0.4	0
GAIF01002674.1	0	0	0	0	0.12	0
GAIF01002675.1	0.19	0.2	0	0.43	0.66	0.61
GAIF01002676.1	1.42	1.92	0.5	1.85	6.84	0.06
GAIF01002677.1	0	0	0	0.02	0.29	0
GAIF01002679.1	175.39	72.39	71.59	382.32	610	1933.64
GAIF01002680.1	2.29	0.43	0.05	8.5	37.69	1.08
GAIF01002681.1	38.83	17.75	27.39	24.08	10.85	37.68
GAIF01002682.1	0.03	0.03	0	0.03	0	0.03
GAIF01002685.1	111.05	116.99	49.67	168.52	134.77	13.42
GAIF01002686.1	0	0	0	17.6	45.48	0
GAIF01002687.1	0.26	0.14	0	0	0	0
GAIF01002688.1	0	0.24	0	0	0.26	0
GAIF01002689.1	3.62	2.41	0.81	39.58	125.08	2.85
GAIF01002690.1	0.29	0.68	0	0.12	0.64	0
GAIF01002691.1	0.17	0.17	0.15	0.23	0.28	0
GAIF01002692.1	1.27	1.05	0.24	7.3	21.02	0.05
GAIF01002695.1	0.03	0.06	0	0.19	0.73	0
GAIF01002696.1	0.85	1.02	0.21	2.93	6.43	0.44
GAIF01002697.1	0	0	0	0	0.14	0
GAIF01002699.1	6.4	6.44	2.32	17.51	21.09	9.76
GAIF01002701.1	0.04	0.04	0.04	0	0.03	0
GAIF01002702.1	53.02	62.32	29.29	139.76	146.57	11.46
GAIF01002703.1	0	0.21	0	0.11	0.36	0
GAIF01002704.1	1.65	0.97	0	7.21	22.73	0
GAIF01002705.1	0	0	0.15	0.63	3.58	0.3
GAIF01002706.1	4.06	9.02	5.94	18.05	13.18	1.83
GAIF01002707.1	0	0	0	0	0.61	0
GAIF01002708.1	6.97	4.61	2.74	25.8	18.5	4.61
GAIF01002711.1	7.29	3.88	5.07	1.51	1.99	3.89
GAIF01002712.1	4.46	2.43	3.81	0.58	0.6	3.01
GAIF01002713.1	0	0.07	0	0.32	1.65	0
GAIF01002716.1	1.24	1.15	1.16	3.89	0.42	0.19
GAIF01002717.1	0.19	0.34	0.41	3.4	7.57	0.07
GAIF01002718.1	13.32	7.3	9.36	8.16	3.84	7.99
GAIF01002720.1	53.28	80.62	79.02	274.19	192.77	40.38
GAIF01002723.1	440.21	103.69	203.92	324.57	73.89	140.63
GAIF01002724.1	0.46	0.4	0.1	1.6	0.33	0
GAIF01002725.1	0.27	0	0	0	0	0
GAIF01002726.1	0.03	0	0.03	0.34	1.24	0.06

GAIF01002728.1	11961.62	13151.98	31159.75	8097.02	4758.47	26674.68
GAIF01002729.1	11.59	10.35	10.59	23.26	11.52	5.52
GAIF01002730.1	1.23	0	0	1.16	0	0.25
GAIF01002732.1	0	0	0	0	0.42	0.15
GAIF01002734.1	0.12	0	0	0	0	0
GAIF01002735.1	32.2	20.3	15.49	34.81	13.26	22.6
GAIF01002737.1	5853.46	7499.23	12331.98	3368.03	2773.71	14706.47
GAIF01002738.1	0	0	9.32	0	0	0
GAIF01002739.1	0	0	0.07	0	1.06	0
GAIF01002741.1	7.58	3.93	7.39	25.56	29.56	16.39
GAIF01002742.1	10.27	6.73	8.8	51.61	126.93	116.28
GAIF01002743.1	0	8.28	1.95	0.14	0.14	2.18
GAIF01002744.1	8.03	5.02	6.04	15.02	10.74	37.55
GAIF01002745.1	0.11	0.24	0.12	0.91	8.63	0.12
GAIF01002746.1	31.92	23.03	23.54	78.9	46.4	98.97
GAIF01002747.1	0.31	0	0	0.32	2.06	0
GAIF01002748.1	0.19	0	0	0.04	0	0.03
GAIF01002751.1	2.12	1.58	0.14	0.43	0.2	0
GAIF01002752.1	32.65	33.4	8.7	8.24	8.36	17.98
GAIF01002753.1	370.36	206.79	142.11	243.8	82.12	34.45
GAIF01002754.1	0.06	0.12	0.02	0.15	0.16	0.02
GAIF01002755.1	49.63	47.49	52.12	41.41	21.58	17.63
GAIF01002756.1	3.29	3.76	3.31	3.82	2.18	1.15
GAIF01002757.1	1.79	0	7.03	0	0	0
GAIF01002758.1	5.57	4.92	5.39	4.85	3.46	1.06
GAIF01002760.1	7.38	10.89	9.01	33.7	22.95	3.81
GAIF01002762.1	0	0	0	0	0.16	0
GAIF01002764.1	1.74	0.17	0.87	1.29	1.9	0.52
GAIF01002766.1	0	0	0	0	0.11	0
GAIF01002767.1	0	0	0	0.14	0	0
GAIF01002768.1	0	0	0	0.13	0	0
GAIF01002769.1	0.13	0.31	0.14	0.52	0.5	0.04
GAIF01002770.1	0	0.11	0	0	0	0
GAIF01002771.1	47.75	23.79	40.69	40.07	43.68	81.97
GAIF01002772.1	0.79	0.58	2.18	0.46	0.12	1.11
GAIF01002773.1	4.58	12.1	4.85	28.3	27.95	3.07
GAIF01002774.1	2.98	1.53	1.63	9.02	9.64	2.64
GAIF01002778.1	7.85	14.31	20.64	28.13	17.64	8.51
GAIF01002779.1	2.45	0.29	0.13	5.09	22.58	0.81
GAIF01002781.1	0	0.13	0	0	0	0
GAIF01002784.1	0	0	0	0.12	0	0
GAIF01002785.1	0	0	0	0.51	0.97	0
GAIF01002786.1	0	0.01	0	0	0.02	0
GAIF01002788.1	0	0	0	0.03	0.55	0

GAIF01002790.1	0.19	0.17	0	0	0	0
GAIF01002792.1	0	0	0	0.09	0	0
GAIF01002793.1	0	0.32	0	1.85	6.59	0.33
GAIF01002794.1	6.26	10.14	4.52	9.31	9.23	0.64
GAIF01002795.1	94.07	78.65	18.67	264.17	279.67	12.41
GAIF01002796.1	0.11	0.18	0	0.25	0.64	0
GAIF01002799.1	430.43	243.83	187.92	1800.72	762.13	202.02
GAIF01002800.1	9.9	15.68	12.38	29.95	19.61	2.84
GAIF01002801.1	0.52	0.38	0	1.8	6.54	0.1
GAIF01002805.1	0	0	0	0.54	1.37	0
GAIF01002806.1	1351.79	1090.14	1772.34	566.46	754.57	6067.05
GAIF01002807.1	27.03	18.47	36.69	72.61	53	292.96
GAIF01002808.1	17.03	29.99	5.75	27.01	55.39	0.29
GAIF01002809.1	0	0.1	0.1	0	0	0
GAIF01002810.1	20.99	18.53	25.38	25.03	26.8	8.2
GAIF01002811.1	1.75	0.78	0	0.27	1.39	0
GAIF01002812.1	0	0	0	0.33	0.69	0
GAIF01002815.1	0.35	0.12	0	3.13	11.89	0.09
GAIF01002819.1	20.43	14.18	14.81	21.99	16.54	34.45
GAIF01002820.1	0.66	0	0	1.39	4.89	1.91
GAIF01002822.1	5.75	3.89	5.95	7.55	1.96	3.71
GAIF01002823.1	7.16	16.26	28.5	5.61	9.91	4.88
GAIF01002825.1	8.07	1	3.91	19.19	2.36	0.98
GAIF01002826.1	0.37	0.15	0.48	0	0	0
GAIF01002827.1	0	0.04	0	0	0.04	0
GAIF01002828.1	15.05	21.07	9.51	29.44	29.02	5.35
GAIF01002829.1	0	0	0	0.19	0.24	0
GAIF01002830.1	0.92	0.54	0.27	6.47	32.21	0.83
GAIF01002834.1	0	0.2	0	0	0	0
GAIF01002836.1	0	0	1.52	0	0	0
GAIF01002837.1	0	0.07	0	0.15	0.38	0
GAIF01002838.1	0	0.4	0.45	0	0.09	0.94
GAIF01002839.1	0.21	0.12	0.16	2.05	4.71	0.45
GAIF01002840.1	109.64	137.17	282.93	43.74	39.85	168.2
GAIF01002841.1	0	0	0	0.3	0.63	0.43
GAIF01002844.1	0	0	0	0	0	0.02
GAIF01002845.1	0.12	0.53	0.13	1.49	2.58	0
GAIF01002847.1	0.37	0	0	0.74	1.32	0
GAIF01002849.1	0.19	0.17	0.16	0.48	1.49	0.14
GAIF01002851.1	0.85	0.94	0.94	0.11	0	0
GAIF01002852.1	0	0	0.13	0	0	0
GAIF01002853.1	25.02	13.47	7.31	92.31	179.49	27.04
GAIF01002854.1	0	0	0	0.14	2.46	0
GAIF01002857.1	0	0	0	3.48	12.61	11.56

GAIF01002859.1	0	0	0	0	0.35	0
GAIF01002860.1	2.34	2.59	3.79	0	0	0.05
GAIF01002862.1	0.05	0.46	0.12	0.37	0.57	0
GAIF01002863.1	18.05	23.46	10.11	41.68	36.1	3.6
GAIF01002864.1	22.12	15.88	11.04	55.72	69.97	11.56
GAIF01002865.1	0.5	0	0.16	0.26	0.26	0.02
GAIF01002866.1	3.68	0.52	0.07	12.34	45.03	13.26
GAIF01002867.1	38.97	30.6	0	52.56	26.02	10.45
GAIF01002868.1	0	0	0	0.03	0.04	0
GAIF01002870.1	0	0	0.26	0.76	4.35	0
GAIF01002871.1	0.23	0	0	0	0.14	0
GAIF01002872.1	0	0.07	0.07	0	0	0
GAIF01002873.1	0	0.1	0	0	0.2	0
GAIF01002874.1	0	0	0.2	0	0.02	0
GAIF01002875.1	7.23	7.93	10.36	17.36	8.89	5.94
GAIF01002876.1	13.51	12.94	37.79	25.84	14.82	15.18
GAIF01002877.1	1.9	0.3	0.1	2	6.42	5.39
GAIF01002879.1	13.97	10.81	7.62	41.68	53.91	11.27
GAIF01002880.1	0	0	0.03	0	0	0
GAIF01002882.1	1.68	2.99	7.45	2.08	2.03	0.96
GAIF01002883.1	0.15	0.06	0.16	0.07	0.11	0.22
GAIF01002884.1	0.17	0	0	0.19	0	0.37
GAIF01002886.1	143.75	211.3	183.24	469.24	168.1	61.74
GAIF01002887.1	0.14	0	0.02	1.38	7.45	0.13
GAIF01002888.1	0	0	0	0	0	0.58
GAIF01002889.1	0.65	0.8	0.09	0.19	0.29	0
GAIF01002890.1	0	0	0	0	0.15	0
GAIF01002892.1	10.87	9.92	18.8	4.44	4.27	9.33
GAIF01002894.1	20.48	18.98	10.4	106.35	109.27	14.65
GAIF01002896.1	2594.36	958.94	3077.01	614.49	74.55	446.19
GAIF01002897.1	0	0.09	0	0	0	0
GAIF01002898.1	8.8	15.61	19.97	25.38	10.4	6.42
GAIF01002899.1	14.73	8.3	4.13	39.02	44.23	15.53
GAIF01002900.1	0	0	0.73	0	0	0.15
GAIF01002901.1	2.28	0.95	0.66	2.1	5.57	0.94
GAIF01002902.1	0	0	0	0.98	3.45	0.09
GAIF01002903.1	82.68	43.92	43.74	90.81	86.48	187.79
GAIF01002904.1	50.11	10.52	1.13	119.96	384.37	5.39
GAIF01002905.1	0	0	0	0	0.84	0.03
GAIF01002907.1	1.44	1.08	0	8.66	25.94	0.5
GAIF01002908.1	17.33	25.75	16.68	81.06	83.22	5.64
GAIF01002909.1	0.6	0.84	0.47	1.01	1.1	0.4
GAIF01002910.1	0	0	0.16	0	0	0
GAIF01002913.1	0	0.07	0	0	0	0

GAIF01002914.1	0.22	0.16	0.24	1.34	5.88	0
GAIF01002916.1	0	0	0	0.1	0	45.43
GAIF01002918.1	0	0	0	0.68	0.84	0.18
GAIF01002919.1	0	0	0	0.7	4.25	0.09
GAIF01002921.1	534.67	139.4	156.2	166.8	164.29	287.56
GAIF01002922.1	0	0	0	1.23	3.81	0
GAIF01002927.1	1.75	1.26	0.08	2.86	7.85	0.08
GAIF01002928.1	4.01	0	0	8.26	26.32	1.67
GAIF01002929.1	2.49	0.73	0.15	11.48	34.17	0.44
GAIF01002930.1	0.17	0.04	0	0.39	2.13	0.06
GAIF01002932.1	0.67	0.16	0.05	0.06	0.17	0.16
GAIF01002935.1	0	0	0	0.22	0.8	0
GAIF01002936.1	0.17	0.09	0.19	1.39	3.17	0
GAIF01002937.1	2.95	2.85	1.62	8.24	7.91	1.47
GAIF01002938.1	0.85	0.76	0.29	0.48	0.74	0.03
GAIF01002939.1	0	0.02	0	0	0	0
GAIF01002940.1	2.63	0	0.41	0.88	0.79	1.2
GAIF01002942.1	73.47	122.59	75.43	206.84	121.92	45.47
GAIF01002943.1	1.45	3.15	0.6	0.64	0.19	0
GAIF01002944.1	0.16	0.09	0	0.1	0.4	0
GAIF01002945.1	13.7	23.37	20.48	45.67	34.13	6.71
GAIF01002946.1	0	0	0	0	0	0.09
GAIF01002948.1	4.85	61.25	66.12	0	0	0
GAIF01002949.1	0	0	0	0	0.18	0
GAIF01002950.1	0.33	0	0	2.12	12.79	2.75
GAIF01002952.1	53.34	45.52	39.69	57.43	64.83	34.22
GAIF01002953.1	390.58	110.11	180.21	266.67	204.91	2597.57
GAIF01002955.1	41.84	6.75	11.92	6.12	5.31	8.49
GAIF01002956.1	0	0.01	0	0	0.04	0
GAIF01002957.1	1.93	2.65	0.59	0.55	1.34	0.06
GAIF01002958.1	0.3	0	0	0.18	0.18	0.33
GAIF01002959.1	0.21	0.15	0.15	0.24	0	0
GAIF01002960.1	7.41	6.6	12.05	7.94	1.09	5.21
GAIF01002961.1	0	0.29	0.07	0.62	3.54	0.07
GAIF01002962.1	0.26	0.1	0	2.46	10.34	0.26
GAIF01002963.1	0	0.42	0.42	0.11	0.12	0.25
GAIF01002964.1	10.68	14.54	6.3	6.44	8.58	0.63
GAIF01002965.1	0	0	0	0	0.01	0
GAIF01002966.1	0	0.23	0	0	0	0.17
GAIF01002967.1	30.5	27.32	19.85	48.65	36.64	8.11
GAIF01002968.1	11.07	15.37	19.39	20.67	11.3	9.78
GAIF01002969.1	0	0.16	0.13	0.04	0	0.16
GAIF01002970.1	17.27	15.86	9.54	101.08	132.04	10.81
GAIF01002972.1	46.53	26.67	24.19	44.57	46.97	92.2

GAIF01002973.1	0.98	0.32	0.32	1.82	4.7	0.11
GAIF01002974.1	38.6	26.27	24.87	36.11	29.4	30.64
GAIF01002975.1	3.78	1.28	1	1.81	0	0.72
GAIF01002976.1	0	0	0	0	0.4	0
GAIF01002978.1	0	0.07	0	0	0	0.07
GAIF01002980.1	12.04	15.78	15.96	42.12	21.49	9.25
GAIF01002981.1	3.76	1.49	1.72	10.49	24.38	16.79
GAIF01002982.1	0	0	0.12	0	0	0
GAIF01002983.1	0.05	0	0	0.33	2.17	0.05
GAIF01002984.1	0.11	0.16	0.04	0.65	1.12	0
GAIF01002985.1	8.03	6.67	4.49	11.69	13.48	1.09
GAIF01002986.1	15.39	22.01	10.76	40.25	27.51	3.02
GAIF01002987.1	0	0	0	0	0.09	0.16
GAIF01002991.1	0	0	0	0.05	0.11	0.1
GAIF01002992.1	13.83	31.03	9.85	22.03	27.78	3.57
GAIF01002993.1	0.56	0	0	0	0	0
GAIF01002994.1	0	0.14	0.14	0.15	0	0
GAIF01002998.1	11.74	12.56	18.32	36.15	32.91	12.83
GAIF01002999.1	56.55	40.71	41.21	82.06	75.27	13.28
GAIF01003000.1	0.21	0	0	0	0	0
GAIF01003002.1	281.41	145.47	139.63	222.92	138.4	54.09
GAIF01003006.1	0.03	0	0	0	0.04	0
GAIF01003007.1	1.39	2.59	0	2.54	14.33	7.66
GAIF01003008.1	1.4	0.87	0.66	3.71	4.44	0.55
GAIF01003010.1	79.64	25.6	67.52	121.29	79.69	16.28
GAIF01003011.1	11.37	8.3	7.16	24.49	26.62	2.31
GAIF01003013.1	20.91	16.14	34.07	88.86	37.99	71.74
GAIF01003015.1	0	0	0.09	0	0.03	0
GAIF01003016.1	0	0	0	0	0.17	0
GAIF01003017.1	0.07	0	0	0.11	0.62	0
GAIF01003018.1	0.16	0	0.12	1.48	7.6	0.59
GAIF01003019.1	0	0	0	0.08	2.21	0
GAIF01003020.1	1.89	0.73	0.05	6.65	19.53	0.63
GAIF01003022.1	1.86	2.02	1.88	7.3	8.06	2.3
GAIF01003023.1	2.7	1.52	0.21	3.1	2	0.16
GAIF01003025.1	0.22	0.1	0.1	0.67	0.48	0.2
GAIF01003026.1	2.09	3.35	2.78	17.33	39.67	0.66
GAIF01003027.1	0	0	0.04	0	1.84	0.4
GAIF01003028.1	0	0	0	0.35	0.37	0
GAIF01003029.1	0	0	0	0.18	0.1	0
GAIF01003031.1	0	0.91	0	0	0	0
GAIF01003033.1	0	0	0	0.06	0.07	0
GAIF01003034.1	5.4	4.6	4.39	16.3	14.25	3.45
GAIF01003035.1	7.26	6.72	6.04	30.52	22.12	2.81

GAIF01003036.1	0.86	0.41	0	1.23	1.52	1.46
GAIF01003037.1	7.22	9.49	11.24	28.12	24.61	5.64
GAIF01003040.1	6.58	8.04	2.19	3.69	3.25	0.89
GAIF01003041.1	262.07	170.2	96.52	0	1.5	0.38
GAIF01003042.1	0.18	0.14	0.04	1.09	2.53	0.02
GAIF01003043.1	0.11	0	0.12	0.12	0	0
GAIF01003045.1	3.97	4.88	7.54	14.09	10.91	4.11
GAIF01003046.1	0.37	0	0.11	2.26	4.34	0.34
GAIF01003047.1	0.04	0	0	0	0.06	0.04
GAIF01003050.1	2.28	1.31	2.36	2.81	0.65	0.74
GAIF01003051.1	0	0	0	0.12	0.13	0
GAIF01003053.1	0.25	0.4	0.41	0	0.15	0
GAIF01003054.1	8.91	13.99	7.91	37.68	29.11	1.69
GAIF01003056.1	0.93	0.48	0.04	2.2	6.9	0.18
GAIF01003058.1	9.72	6.08	6.87	25.94	35.98	8.79
GAIF01003060.1	0.07	0	0	0.08	0.08	0
GAIF01003061.1	0	0	0	0.09	0	0
GAIF01003062.1	0	0	0	0	0.09	0
GAIF01003063.1	0.44	0.47	0.16	0	0	0
GAIF01003064.1	0.08	0	0	0	0	0
GAIF01003065.1	0.49	0.28	0.18	0.34	0.28	0.04
GAIF01003066.1	28.94	41.4	25.69	85.67	105.07	8.13
GAIF01003067.1	1.11	177.33	396.59	6.66	31.56	34.99
GAIF01003068.1	84.38	27.13	32.31	15.83	23.75	15.64
GAIF01003069.1	0.59	0	0	1.28	8.36	0.19
GAIF01003070.1	0.21	0	0	2.97	0	0
GAIF01003071.1	64.95	85.2	91.88	102.54	77.54	21.17
GAIF01003072.1	1.99	3.77	1.01	0.83	0.37	0.11
GAIF01003073.1	0.85	1.18	0.42	0.36	0.46	0
GAIF01003074.1	0.21	0	0	0.25	0.38	0.12
GAIF01003075.1	151.02	70.36	69	169	126.57	631.76
GAIF01003076.1	0	0	0.09	0.28	1.55	0
GAIF01003079.1	1	1.04	0.68	3.54	4.01	0.47
GAIF01003080.1	0.07	0	0	0	0	0
GAIF01003081.1	5.81	3.97	3.74	7.36	13.79	1.58
GAIF01003083.1	0.25	0.09	0.3	0.04	0.03	0.4
GAIF01003084.1	3451.76	1958.33	1161.28	2413.92	2801.32	1728.09
GAIF01003085.1	0	0.11	0	0	0	0
GAIF01003086.1	6.84	2.89	2.79	25.67	63.91	41.81
GAIF01003089.1	0.06	0.54	0.54	0	0.07	3.96
GAIF01003091.1	12.33	20.45	22.29	69.02	35.8	11.95
GAIF01003092.1	125.39	186.09	251.73	112.72	75.98	256.23
GAIF01003094.1	0.19	0.04	0	0.54	8.37	0
GAIF01003096.1	0.27	0	0	0.12	0	0

GAIF01003097.1	0.09	0	0	0.04	0.01	0.16
GAIF01003099.1	102.69	101.18	166.92	49.76	31.19	83.45
GAIF01003100.1	11.12	7.49	14.57	24.92	21.74	30.86
GAIF01003101.1	0.32	0.18	0.18	2.64	8.2	0.18
GAIF01003102.1	0	0	0	1.92	5.14	0.1
GAIF01003107.1	25.66	22.12	16.6	31.75	29.07	10.49
GAIF01003109.1	79.45	27.64	45.31	115.89	2.56	10.24
GAIF01003110.1	22.8	27.28	18.87	38.5	39.22	24.39
GAIF01003112.1	6.24	1.2	2.72	4.69	6.04	5.75
GAIF01003113.1	0.14	0	0.05	0.05	0.15	0.19
GAIF01003118.1	0.72	0.26	0	7.42	31.94	1.6
GAIF01003119.1	11.18	24.92	6.45	41.41	70.47	0.5
GAIF01003120.1	0.02	0	0	0	0	0
GAIF01003123.1	0.46	0.25	0.17	3.35	16.01	0.08
GAIF01003124.1	23.72	29.6	13.52	2.87	0.91	0.55
GAIF01003125.1	25.09	25.55	22.23	70.38	43.27	15.1
GAIF01003128.1	408.7	183.74	171.91	575.77	597.86	128.98
GAIF01003130.1	5.53	4.44	8.97	7.67	5.78	16.42
GAIF01003131.1	0.52	2.08	0.53	6.87	38.42	1.47
GAIF01003133.1	2.5	0.29	0	9.64	44.23	1.38
GAIF01003135.1	0	0	0.06	0.07	0.27	0
GAIF01003136.1	0	0	0	0	0.22	0
GAIF01003137.1	0.3	0.16	0	0	0	0
GAIF01003138.1	0	0	0	0	0.23	0
GAIF01003140.1	26.7	13.2	1.03	6.58	4.84	12.58
GAIF01003141.1	0	0	0	0.12	0	0
GAIF01003142.1	0.09	0	0	0	0	0
GAIF01003143.1	62.54	42.08	21.76	102.04	121.61	49.05
GAIF01003145.1	42.24	42.19	25.56	208.58	201.36	19.45
GAIF01003146.1	34.97	47.06	23.54	315.61	196.09	17.59
GAIF01003147.1	992.21	312.78	295.83	409.47	421.94	2087.88
GAIF01003148.1	3.82	8.5	5.48	8.49	4.8	0.18
GAIF01003149.1	0.17	0	0	0.58	2.5	0.27
GAIF01003150.1	33.47	43.17	57.55	49.4	30.05	28.55
GAIF01003151.1	0	0	0	0	0.14	1.08
GAIF01003152.1	0.08	0.13	0.08	4.86	16.54	0.17
GAIF01003153.1	0.14	0.1	0.1	0.89	0.87	0.1
GAIF01003154.1	33.04	12.03	26.47	39.15	16.94	13.02
GAIF01003156.1	137.73	35.64	54.91	33.51	16.97	83.74
GAIF01003157.1	297.34	111.84	131.83	68.08	50.14	104.43
GAIF01003158.1	4.29	5.32	2.29	33.61	41.52	1.68
GAIF01003159.1	3.58	5.96	4.56	18.07	17.23	2.51
GAIF01003161.1	0.2	0.16	0.05	1.32	9.11	0.05
GAIF01003162.1	0.03	0.03	0	0.28	0	0

GAIF01003164.1	0.82	0.64	0	5.59	2.83	0.65
GAIF01003165.1	15.06	10.8	9.13	34.13	33.05	8.55
GAIF01003166.1	1.75	0	0	1.85	8.84	0.59
GAIF01003169.1	0.11	0.12	0	0.13	0.65	0
GAIF01003170.1	0.21	0.1	0.1	2.22	8.17	0.05
GAIF01003172.1	0	0	0	0	0.15	0
GAIF01003173.1	1.21	2.27	1.33	0.78	0.32	0
GAIF01003174.1	4.53	8.15	11.38	24.79	21.41	5.51
GAIF01003175.1	0	0.08	0	0.17	0	0
GAIF01003177.1	4.06	3.04	3.64	4.13	3.39	2.15
GAIF01003178.1	0	0	0.15	0	0	0
GAIF01003179.1	20.01	22.32	30.32	32.02	28.64	5.18
GAIF01003180.1	4.84	7.27	7.85	1.58	2.57	2.59
GAIF01003182.1	0.96	0.5	0	0.37	0.6	0.36
GAIF01003184.1	18.01	13.8	18.52	15.69	38.69	4.33
GAIF01003188.1	0	0	0	0	0.06	0.05
GAIF01003189.1	4.09	3.45	0.87	13.09	25.05	22.13
GAIF01003190.1	0	2.41	0.35	0.59	1.09	0
GAIF01003191.1	1022.03	615.6	886.18	840.88	705.95	1203.8
GAIF01003192.1	0	0	0	0	0.04	0
GAIF01003195.1	0	0	0	0	0	0.48
GAIF01003196.1	0.29	0.47	0.95	0	0	0
GAIF01003198.1	0.18	0.05	0	0.36	1.85	0.05
GAIF01003199.1	8.72	11.38	6.3	38.15	46.84	2.97
GAIF01003201.1	0.62	1.76	0.39	8.28	3.12	0.7
GAIF01003203.1	209.57	330.7	681.57	220.21	189.53	257.36
GAIF01003206.1	0	0	0	0.45	0.19	0.19
GAIF01003207.1	0.27	0	0.1	0.15	0.13	0
GAIF01003209.1	0.08	0	0	0	0.2	0
GAIF01003210.1	0	0	0	0	0	8.8
GAIF01003213.1	0.24	0.09	0.03	0.35	0.62	0.2
GAIF01003214.1	0.95	0.36	0.41	4.31	17.67	7.64
GAIF01003215.1	6.97	4.93	5.87	3.24	0.98	1.28
GAIF01003216.1	32.97	33.42	20.52	55.04	45.32	13.35
GAIF01003217.1	3.13	1.31	1.33	0	0	0
GAIF01003222.1	0.23	0	0	2.72	1.31	0
GAIF01003223.1	20.63	11.16	23.93	23.26	16.75	39.78
GAIF01003224.1	5.94	23.88	6.73	35.9	10.74	0
GAIF01003225.1	9.61	10.06	6.04	30.68	36.48	1.78
GAIF01003226.1	2.26	3.62	1.61	1.04	2.39	0.64
GAIF01003227.1	0	0	0	0	0.12	0
GAIF01003228.1	0	0	0.03	0	0	0
GAIF01003229.1	0.44	0.6	0.24	0.9	1.07	0
GAIF01003231.1	18.6	33.69	8.72	41.6	47.01	1.75

GAIF01003233.1	0	0.12	0.12	0.21	1.45	0.55
GAIF01003235.1	0.14	0.53	0.57	0	0	0
GAIF01003236.1	4.21	2.13	0.43	6.45	12.48	0.38
GAIF01003239.1	0	0.02	0	0.15	0.09	0
GAIF01003241.1	0	0.42	0.17	0	0	0
GAIF01003244.1	1.14	1.39	1.01	2.97	1.62	0.47
GAIF01003247.1	5.98	5.38	7.01	12.12	9.25	21.12
GAIF01003248.1	4.68	5.19	3.18	11.95	13.73	0.82
GAIF01003250.1	0	0	0	0.84	0.56	0
GAIF01003252.1	0.1	0.05	0.05	1.25	4.87	0.05
GAIF01003257.1	14.62	16.16	4.63	28.09	35.38	1.6
GAIF01003259.1	162.28	81.73	46.95	231.58	317.17	38.19
GAIF01003260.1	0	0.04	0	0	0	0.1
GAIF01003261.1	0.9	0.19	0.79	1.04	2.37	0.4
GAIF01003262.1	0.1	0	0	0	0	0
GAIF01003265.1	0.04	0.27	0	0.04	0	0
GAIF01003266.1	0	0	0.1	0	0.14	0.1
GAIF01003267.1	0	0	0	0	0.42	0
GAIF01003268.1	7.86	5.79	7.52	19.42	15.65	5.87
GAIF01003270.1	610.12	276.66	278.85	435.21	321.08	73.65
GAIF01003271.1	0.47	0.1	0.47	1.49	0.57	0.42
GAIF01003272.1	0	0	0.07	0	0.17	0
GAIF01003273.1	0.05	0.06	0.11	1.07	4.48	0.17
GAIF01003274.1	30.86	19.72	25.31	54.57	38.4	72.45
GAIF01003275.1	2.36	1.45	0	1.99	1.39	0.8
GAIF01003276.1	1.94	2.09	2.45	8.59	4.5	0.74
GAIF01003278.1	29.6	4.59	2.08	11.34	5.64	5.66
GAIF01003279.1	1.24	55.31	26.68	10.02	108.69	37.26
GAIF01003280.1	0	0.09	1.24	0	0	0
GAIF01003281.1	1.88	0	0.28	1.9	0.91	3.74
GAIF01003282.1	22.5	19.48	24.7	37.48	34.45	22.04
GAIF01003286.1	0.11	0	0.37	0	0	0
GAIF01003288.1	1.4	0.38	0	1.49	1.78	0
GAIF01003291.1	21.01	29.69	10.48	77.19	97.29	0.6
GAIF01003295.1	39.49	35.85	17.79	60.04	63.36	8.5
GAIF01003297.1	1.99	0.43	0.38	0.75	1.61	1.1
GAIF01003299.1	0	0	0	0.18	0.15	0
GAIF01003302.1	0	0	0	0.1	0.21	0
GAIF01003304.1	0.14	95.23	28.87	0	0.45	0
GAIF01003305.1	0.01	0.12	0	0.15	1.37	0
GAIF01003309.1	20.45	25.14	31.28	48.19	32.78	12.74
GAIF01003310.1	0.03	0	0	0.43	1.02	0.06
GAIF01003311.1	88.44	39	7.54	79.21	53.97	5.03
GAIF01003312.1	1.31	2.55	1.52	1.12	1.08	1.52

GAIF01003313.1	0.73	0.11	0.34	3.14	5.88	5.84
GAIF01003314.1	6120.29	24692.38	20679.25	243.48	604.67	924.15
GAIF01003315.1	0	0	0	0.21	1.04	0.05
GAIF01003317.1	1.73	0.18	0	6.39	26.08	0.54
GAIF01003318.1	0.28	0.35	0.12	3.03	7.59	0.31
GAIF01003319.1	0	0	0	0	3.89	0.58
GAIF01003321.1	1.06	0.29	0.1	1.23	1.91	0.1
GAIF01003322.1	0	0	0	0.91	0.97	0
GAIF01003325.1	0	0	0	0.11	0	0
GAIF01003326.1	0.23	0	0	1.49	4.49	0.13
GAIF01003328.1	100.11	114.03	46.74	93.15	75.21	33.77
GAIF01003329.1	0.05	0	0	0	0.74	0
GAIF01003330.1	64.51	95.88	121.08	59.72	47.73	34.57
GAIF01003331.1	0	0	0	0	0.2	0
GAIF01003332.1	1.12	0.6	0.33	8.76	0.37	0.29
GAIF01003333.1	1.23	0.73	0.45	1.36	0.61	0.34
GAIF01003334.1	0	1.05	2.24	0	0	0.66
GAIF01003335.1	204.57	111.07	85.65	58.28	37.71	20.48
GAIF01003336.1	0	0	0	0.06	0.48	0
GAIF01003337.1	0	0	0	0.11	0.23	0.11
GAIF01003338.1	0.24	0.7	0.09	0.74	1.22	0.18
GAIF01003339.1	43.89	20.57	16.92	93.3	65.77	15.36
GAIF01003340.1	2.78	1.73	2.39	8.22	11.34	0
GAIF01003342.1	3.25	1.09	1.26	3.48	5.11	0.38
GAIF01003343.1	0.26	0.25	0.69	0.85	0.7	0.9
GAIF01003345.1	33.74	18.78	22.29	45.75	41.55	9.31
GAIF01003346.1	0	0.19	0	0.41	1.06	0
GAIF01003347.1	1.97	0	1.83	1.76	1.64	3
GAIF01003348.1	9.25	13.56	9.07	17.18	19.24	2.23
GAIF01003349.1	27.16	21.16	17.7	49.64	40.73	7.77
GAIF01003351.1	5.19	4.73	2.65	3.26	2.48	1.8
GAIF01003354.1	56.97	69.49	40.82	32.32	20.96	29.71
GAIF01003355.1	0	0.24	0	0.07	0.11	0.05
GAIF01003356.1	0.01	0.04	0	0	0.06	0
GAIF01003357.1	17.95	8.16	10.9	8.51	3.68	6.24
GAIF01003359.1	8.87	7.22	7.91	29.86	40.05	3.59
GAIF01003360.1	0	0.94	0.66	0.15	0.8	0.07
GAIF01003361.1	0	0	0.1	0.16	0.56	0.05
GAIF01003362.1	44.38	80.59	82.26	13.51	5.68	19.61
GAIF01003363.1	0	0.13	0.54	0	0	0
GAIF01003364.1	0.28	0	0.15	1.78	6.55	0
GAIF01003366.1	12.12	4.82	13.72	35.28	73.88	155.76
GAIF01003367.1	0.54	0	0.19	0.8	0	0.48
GAIF01003372.1	191.48	110.85	54.59	203.16	187.78	115.26

GAIF01003373.1	11	11.48	8.34	28.95	19.1	6.8
GAIF01003374.1	2.37	0	0	2.68	8.4	0
GAIF01003377.1	0	0	0	0	0.15	0.27
GAIF01003378.1	0	0.11	0	0	0	0
GAIF01003379.1	0	0	0	0.26	0.27	0
GAIF01003380.1	0.09	0	0	0.21	4.08	0.1
GAIF01003381.1	1.49	2.5	0.69	1.34	2.06	0.25
GAIF01003382.1	0.22	0	0	0.77	1.42	0
GAIF01003385.1	0.97	0.47	0.99	0.38	0.22	0.08
GAIF01003386.1	0	0	0	0	0.5	0
GAIF01003388.1	0	0	0.09	0	0	0.23
GAIF01003390.1	6.23	11.17	6.54	21.7	23.73	3.55
GAIF01003391.1	10.69	12.19	2.42	48.73	46.05	1.21
GAIF01003392.1	41.75	37.46	30.06	73.7	62.78	19.32
GAIF01003395.1	0	0.15	0	0.47	3.72	0.3
GAIF01003396.1	0.46	0.41	0	0.54	4.85	0.22
GAIF01003397.1	0	0	0	0	0.54	0
GAIF01003398.1	0.11	0	0	0.26	1.15	0.06
GAIF01003399.1	0.14	0.6	0	0.17	1.3	0.44
GAIF01003400.1	2.19	4.77	4.37	6.2	3.69	2.85
GAIF01003401.1	0.17	0.04	0.09	0.34	2.53	2.27
GAIF01003403.1	0.06	0	0	0	0.46	0
GAIF01003406.1	4.07	2.71	1.01	4.42	2.68	0.87
GAIF01003407.1	0	0	0	0.14	0.44	0
GAIF01003408.1	3.07	3.95	6.08	8.22	17.58	2.31
GAIF01003409.1	0	0	0	0	0	0.29
GAIF01003410.1	0	0	0	0.12	0.37	0
GAIF01003411.1	21.6	18.8	13.58	44.3	27.89	7.84
GAIF01003412.1	0	0	0	0.09	0	0
GAIF01003413.1	0	0	0.2	0	0	0
GAIF01003415.1	0	0	0	0	0	0.53
GAIF01003416.1	40.95	12.62	14.01	4.58	1.55	0.99
GAIF01003418.1	0	0	0	0	0.3	0
GAIF01003420.1	1.21	0.74	0.83	8.88	68.62	2.18
GAIF01003421.1	0	0	0	0.1	0.1	0
GAIF01003422.1	14.06	10.53	13.22	24.44	22.11	14.84
GAIF01003423.1	0.14	0	0	0.5	2.09	0
GAIF01003424.1	5.51	6.55	7.3	7.98	4.03	2.57
GAIF01003425.1	17.39	18.36	15.56	40.32	20.25	5.18
GAIF01003426.1	0.52	0.1	0.2	1.75	7.01	0.85
GAIF01003427.1	0.97	0.3	0.15	3	6.04	0.1
GAIF01003428.1	0	0	0	0.13	0.22	0
GAIF01003429.1	0.49	0.31	0.33	0.69	0.31	0
GAIF01003430.1	3.37	1.9	0.51	7.18	16.43	15.66

GAIF01003432.1	30.15	30.29	36.86	5.79	3.88	4.7
GAIF01003433.1	6.08	4.76	1.9	1.45	5.23	0.49
GAIF01003434.1	0	0	0.1	0	0.22	0
GAIF01003438.1	0.17	0.09	0	0.2	0.93	0.09
GAIF01003441.1	12.65	16.23	16.93	34.7	25.96	10.04
GAIF01003442.1	0.09	0.03	0.06	0.74	5.39	0.48
GAIF01003443.1	7.89	11.37	11.24	25.42	15.14	3.06
GAIF01003444.1	0	0	0	0	0.33	0
GAIF01003445.1	30.21	8.16	7.75	20.04	9.84	16.35
GAIF01003446.1	19.12	23.02	15.14	53.22	45.1	6.13
GAIF01003449.1	0.09	0	0	0	0	0
GAIF01003450.1	0	0	0	0	4.48	0
GAIF01003451.1	0	0.08	0	0.08	0.12	0
GAIF01003453.1	0	0	0	0	0.58	0
GAIF01003454.1	0.31	0	0	0	0	0
GAIF01003455.1	207.72	121.25	52.23	65.19	84.82	27.28
GAIF01003456.1	0.11	0	0	0	0	0.28
GAIF01003457.1	0	0	0	0.25	0.12	0
GAIF01003459.1	0	0	0	0	0.13	0
GAIF01003460.1	0.04	0	0	0.58	0	0
GAIF01003461.1	0	0.17	0.35	0	0	0.96
GAIF01003463.1	8.69	14.46	7.6	8.43	8.2	0.5
GAIF01003464.1	0.4	0.7	0	2.79	4.44	0.18
GAIF01003465.1	14.44	16.71	12.86	47.99	134.02	28.77
GAIF01003466.1	0	0	0.08	0	0	0
GAIF01003467.1	14	17.99	22.18	29.95	15.99	9.34
GAIF01003469.1	10.3	7.75	8.2	26.07	56.4	26.53
GAIF01003470.1	158.15	20.99	9.39	95.24	150.46	11.12
GAIF01003472.1	27.92	15.97	8.82	82.31	118.16	12.47
GAIF01003473.1	19.37	11.7	5.18	41.05	52.69	12.63
GAIF01003474.1	4.09	3.01	5.37	2.33	0.37	6.05
GAIF01003475.1	0.25	0	0.05	0.73	3.29	0.28
GAIF01003476.1	0	0	0.11	0.12	0.12	0.13
GAIF01003477.1	1.37	0	0	0.89	1.34	0
GAIF01003478.1	0.3	0.16	0	0.53	1.27	0
GAIF01003479.1	1.24	0.23	0	4.17	12.14	1.31
GAIF01003480.1	26.73	10.21	6.88	9.12	24.59	6.99
GAIF01003481.1	0	0.31	0.08	0	0	0.08
GAIF01003482.1	0.92	0.26	0.13	1.96	14.12	0
GAIF01003484.1	34.84	82.14	27.56	96.57	160.9	12.58
GAIF01003485.1	0	0	0	0.14	1.72	0
GAIF01003486.1	17.13	14.06	3.62	7.17	27.05	15.65
GAIF01003487.1	0	0	0	0.29	0.74	0
GAIF01003489.1	0	0	0	0.62	3.43	0.44

GAIF01003490.1	0	0.05	0	0	0	0
GAIF01003491.1	0.73	0.64	0.96	1.02	0.18	0
GAIF01003492.1	1.05	0.38	0.13	4.59	23.1	0.26
GAIF01003493.1	0	0	0.01	0.13	2.38	0
GAIF01003494.1	0	0	0	0	0.15	0.05
GAIF01003495.1	1.83	2.91	0.15	7.19	10.84	0.16
GAIF01003496.1	0.3	0.11	0.22	1.15	0.18	0
GAIF01003497.1	4.86	3.77	0.69	6.04	11.44	0.69
GAIF01003498.1	0	0	0	0.07	0.14	0.59
GAIF01003499.1	3.27	6.08	2.43	18.35	14.72	1.33
GAIF01003500.1	48.51	70.81	55.28	110.18	62.54	12.89
GAIF01003501.1	0.5	0.72	1.16	0.81	0.32	1.2
GAIF01003503.1	36.54	45.68	70.33	56.06	54.59	28.72
GAIF01003504.1	0	0	0	0	0.08	0.07
GAIF01003505.1	0.26	0	0	0.95	1.11	0
GAIF01003506.1	0.11	0	0	0.98	1.66	0.25
GAIF01003507.1	3.89	3	1.09	15.18	44.59	1.39
GAIF01003508.1	0.08	0	0	0.19	0.84	0.05
GAIF01003509.1	7.21	8.85	5.7	34.9	33.71	2.26
GAIF01003510.1	0	0	0	0.12	0.87	0
GAIF01003511.1	0	0	0.05	0	0.05	0
GAIF01003512.1	23.72	14.18	21.84	44.29	67.27	36.94
GAIF01003513.1	23.72	15.75	19.49	20.22	10.84	13.35
GAIF01003514.1	0	0	0	0.56	0.14	0
GAIF01003515.1	50.87	49.73	37.99	116.11	83.11	16.7
GAIF01003516.1	28.93	53.34	19.31	71.07	78.56	6.4
GAIF01003517.1	0.91	1.28	0.72	0.3	0	0
GAIF01003518.1	0	0.66	0	0	0	0
GAIF01003521.1	0	0	0	0	0.06	0
GAIF01003522.1	0.12	0.37	0.07	0.9	3.38	0.48
GAIF01003523.1	54.23	20.76	29.88	61.14	55.99	87.17
GAIF01003524.1	0.87	0.16	0	2.8	18.29	0.43
GAIF01003525.1	0.23	0.1	0	0.05	1.96	0
GAIF01003526.1	0.86	0	7.96	4.97	0	4.61
GAIF01003527.1	44.13	47.69	12.15	60.77	52.03	2.75
GAIF01003528.1	0.17	0.04	0.06	1.17	5.98	0.9
GAIF01003529.1	0.57	0.21	0.62	0	0	0.21
GAIF01003532.1	0	0.49	0.06	0	0	0
GAIF01003534.1	7.56	6.65	5.12	22.8	27.03	1.39
GAIF01003537.1	0	0.27	0.27	0.1	0	0.55
GAIF01003538.1	2.47	0	0	2.34	1.4	0.73
GAIF01003539.1	0	0.13	0.09	0	0	0
GAIF01003540.1	0	0	0	0	0.09	0
GAIF01003541.1	0	0	0	0	0.4	0

GAIF01003542.1	0.04	0	0	0	0.05	0
GAIF01003543.1	2.25	2.74	2.1	2.93	1.89	0.45
GAIF01003547.1	1.57	1.6	0.4	3.86	6.92	1.31
GAIF01003549.1	0	0	0	0.1	0.11	0
GAIF01003550.1	0.03	0.04	0	0	0	0
GAIF01003552.1	0	0	0.29	0	0	0
GAIF01003553.1	2.49	1.93	3.52	7.43	5.14	0.39
GAIF01003555.1	0.83	0	0.46	0.24	0.25	0
GAIF01003556.1	6.14	4.08	0.56	4.75	4.72	3.01
GAIF01003557.1	6.47	11.91	9.74	11.72	11.5	2.59
GAIF01003560.1	0	0	0	0.06	0.12	0
GAIF01003561.1	55.46	123.54	171.79	48.52	63.33	173.09
GAIF01003562.1	0.16	0.09	0	1.32	7.82	0.18
GAIF01003563.1	0.74	1.09	0.66	0.55	0.24	0.3
GAIF01003565.1	324.23	267.37	248.16	214.56	145.43	370.2
GAIF01003567.1	0.09	0	0	0	0.31	0.28
GAIF01003569.1	2.68	3	2.86	2.47	0.95	0.17
GAIF01003570.1	0.08	0	0	0.09	0.52	0
GAIF01003571.1	202.85	153.2	111.34	120.09	165.48	57.54
GAIF01003574.1	0.56	0.51	0.38	1.82	2.89	0.38
GAIF01003575.1	1030.19	72.99	191.51	1492.18	250.31	222.28
GAIF01003576.1	3.79	5.84	3.9	14.68	10.87	1.64
GAIF01003578.1	1.29	0.49	0	2.07	4.94	0.39
GAIF01003579.1	0.42	0.39	0.26	0	0.94	0
GAIF01003581.1	55.97	64.42	109.06	28.82	17.45	25.03
GAIF01003582.1	0	0	0	0.18	0.95	0.17
GAIF01003583.1	0	0	0	0	0.07	0
GAIF01003586.1	16.54	17.05	12.63	44.17	26.98	7.57
GAIF01003589.1	0.13	0	0	0	3.38	0
GAIF01003590.1	4.83	0.46	0.43	17.31	68.22	2.7
GAIF01003592.1	0.81	2.65	0.36	10.37	19.3	0.27
GAIF01003593.1	0	0.14	0	0	0.1	0
GAIF01003595.1	0	0.09	0.09	0.58	0	0.17
GAIF01003596.1	0.12	0.27	0.18	0.14	0.28	0.36
GAIF01003597.1	6.58	4.29	3.32	28.72	9.18	3.48
GAIF01003598.1	0	0	0.16	1.22	5.76	0
GAIF01003599.1	0	0.4	0.34	0	0.06	0
GAIF01003601.1	0.12	0	0	0	0.14	0
GAIF01003602.1	0	0	0	0	1.17	0
GAIF01003604.1	17.14	6.21	0.97	50.64	233.46	11.72
GAIF01003605.1	3.72	1.4	1.76	5.01	5.38	1.79
GAIF01003608.1	0.01	0	0	0.09	0.01	0
GAIF01003610.1	0	0.06	0	0	0.71	0.06
GAIF01003612.1	0.48	0.34	0.12	3.26	6.6	0.19

GAIF01003613.1	0	0	0	0.35	0	0
GAIF01003614.1	1.24	2.16	0.27	4.32	5.07	0.82
GAIF01003616.1	7.53	5.71	0	12.55	7.41	0
GAIF01003617.1	0	0.27	0.23	0.04	0.13	0.08
GAIF01003618.1	0	0.23	0.15	3.08	9.9	0.23
GAIF01003619.1	0.08	0	0	0.2	0.92	0.21
GAIF01003620.1	0	0.34	0.07	0.15	0	0
GAIF01003621.1	0.05	0	0	0.12	0.77	0.29
GAIF01003622.1	14.04	56.79	17.99	3.23	21.11	0.97
GAIF01003626.1	31.28	30.32	76.27	35.28	50.16	197.7
GAIF01003627.1	0	0	0	0	0.37	0
GAIF01003629.1	13.79	11.8	4.41	36.92	29.73	2
GAIF01003630.1	58.61	33.64	15.77	126.77	175.02	21.98
GAIF01003631.1	0	0	0	0	0	0.05
GAIF01003632.1	0.68	0.63	0.85	0.68	0.23	0.43
GAIF01003634.1	38.23	25.17	21.27	75.09	28.72	9.12
GAIF01003635.1	0	0	0	0	0.11	0
GAIF01003636.1	0	0	0	0	1.71	1.69
GAIF01003638.1	2.77	2.63	3.51	6.48	7.05	0.69
GAIF01003639.1	2.7	1.73	0.19	8.09	20.29	2.49
GAIF01003640.1	0	0	0	0	0.49	0.11
GAIF01003643.1	3.25	4.95	3.14	13.73	23.19	1.58
GAIF01003644.1	0.4	0.14	0	2.01	6.87	0
GAIF01003646.1	0	0	0.16	0	0	0
GAIF01003647.1	7.23	15.94	8.94	9.25	5.02	2.41
GAIF01003648.1	1.92	2.1	0	3.76	7.02	0
GAIF01003649.1	42.87	77.57	74.92	8.49	15.64	10.99
GAIF01003651.1	0	0.06	0.06	0	0.11	0
GAIF01003652.1	0.07	0	0	0.91	6.21	0.24
GAIF01003654.1	0.77	0.93	0.05	3.56	7.11	0.25
GAIF01003657.1	0.26	0.11	0	0.23	0.67	0.22
GAIF01003658.1	0.11	0	0	0	0	0.35
GAIF01003659.1	0	0.14	0	0	0	0
GAIF01003661.1	0	0.35	0.79	0	0	0
GAIF01003662.1	0	0	0	0.14	0	0
GAIF01003663.1	1.5	1.04	1.05	4.12	12.14	0.15
GAIF01003664.1	13.43	14.7	19.51	36.11	30.37	19.09
GAIF01003665.1	5.91	14.11	8.55	8.64	10.17	1.14
GAIF01003666.1	26.52	34.68	58.91	64.78	38.63	36.83
GAIF01003667.1	0	0	0	0	0.13	0
GAIF01003669.1	55.41	48.69	63.3	57.67	24.59	30.65
GAIF01003670.1	0.19	0.13	0	3.58	7.56	0.07
GAIF01003671.1	407.38	146.44	143.01	594.07	664.66	1094.97
GAIF01003672.1	0.74	0	0	0.14	0	0

GAIF01003673.1	0.14	0.04	0.04	0.12	0.21	0
GAIF01003674.1	0.82	1.68	0	3.1	4.67	0
GAIF01003676.1	7.39	6.8	4.79	28.28	38.4	3.13
GAIF01003677.1	1.19	0.43	0	0.61	1.11	0.29
GAIF01003678.1	0.04	0	0	0	0	0
GAIF01003679.1	0	0	0	0	0.52	0
GAIF01003680.1	4.22	1.8	2.62	1.81	0.01	0
GAIF01003681.1	0.36	0	0.08	0.08	0.87	0
GAIF01003686.1	0.24	0.45	0.59	0.23	0.1	0
GAIF01003687.1	25.02	15	17.28	12.46	15.48	2.35
GAIF01003689.1	0.18	0	0	0	0	0
GAIF01003690.1	6.48	8.53	11.45	24.64	17.85	4.97
GAIF01003692.1	0.11	0.06	0.06	0.5	2.71	0
GAIF01003694.1	2.2	6.09	5.79	2.65	2.3	1.24
GAIF01003695.1	0.13	0.27	0.41	0.24	0.15	0
GAIF01003698.1	11.38	13.28	14.66	20.31	11.54	4.41
GAIF01003699.1	6.32	1.58	0.16	5.14	13.09	1.2
GAIF01003700.1	0.3	0	0	1.49	6.87	0.65
GAIF01003701.1	0.05	0	0	0.09	0.03	0
GAIF01003702.1	163.26	276.34	265.03	117.26	100.54	82.25
GAIF01003703.1	357.3	2569.04	1870.78	25	72.35	54.2
GAIF01003704.1	3.44	1.7	2.13	2.17	1.75	1.91
GAIF01003706.1	0	0.46	0.3	0	0	0.13
GAIF01003707.1	8.17	3.08	0.2	5.5	19.89	8.3
GAIF01003708.1	0	0.09	0	0	0	0
GAIF01003709.1	48.76	25.62	19.83	75.56	43.8	87.7
GAIF01003710.1	0	0	0	0.24	1.11	0
GAIF01003711.1	2.18	0.79	0.27	11.53	51.38	0.53
GAIF01003712.1	0	0	0	0	0.1	0
GAIF01003713.1	1.8	2.34	1.43	1.58	0.54	1.5
GAIF01003715.1	0	0	0	0.4	0.21	0.19
GAIF01003716.1	0.41	0.27	0.18	0.38	0.39	0
GAIF01003718.1	0.56	1.27	0.4	5.33	12.86	0.07
GAIF01003719.1	0.18	0.1	0	0.33	1.02	0.26
GAIF01003720.1	0	0	0	0	0.03	0
GAIF01003722.1	0	0.09	0	0	0.1	0
GAIF01003724.1	6.46	5.87	12.96	15.11	9.19	0
GAIF01003725.1	0.57	0.17	0.06	0	0	0.34
GAIF01003726.1	0	0	0	0	2.16	0
GAIF01003728.1	56.18	36.76	14.08	9.38	12.98	0.74
GAIF01003729.1	0	0	0	0.32	2.52	0.15
GAIF01003730.1	2.05	1.61	1.26	2.58	2.52	1.45
GAIF01003731.1	0.11	0.16	0.25	0.09	0.27	0.08
GAIF01003732.1	243.47	3.23	4.32	8.12	0.68	1.77

GAIF01003733.1	0	0	0	0	1.22	2.4
GAIF01003734.1	0	0	0	0.11	0.45	0.2
GAIF01003735.1	0.27	0.06	0.03	1.23	4.49	0.63
GAIF01003736.1	0.92	0.22	0.12	0.16	0	0
GAIF01003739.1	0	0	0	0.17	1.03	0.08
GAIF01003740.1	0	0	0	0.03	0.04	0
GAIF01003741.1	71.75	113.71	90.47	24.36	26.52	12.1
GAIF01003744.1	0.2	0	0	1.35	4.62	0.06
GAIF01003745.1	0	0	0	0.43	5.47	0
GAIF01003747.1	4.62	4.71	1.08	7.73	6.17	0.67
GAIF01003748.1	0	0	0	0.16	0.51	0.31
GAIF01003750.1	192.22	206.06	265.68	279.82	147.19	92.27
GAIF01003752.1	0.03	0	0.03	0.07	0.34	0
GAIF01003753.1	10.99	14.86	17.83	38.63	17.27	5.79
GAIF01003754.1	0	0.14	0	0	0.16	0
GAIF01003755.1	3.89	0	3.26	10.2	8.66	15.51
GAIF01003756.1	22.12	133.68	155.02	0	6.02	3.37
GAIF01003757.1	0.09	0.11	0.29	0.03	0.41	0.07
GAIF01003758.1	0.34	0.07	0.03	1.94	6.11	1.49
GAIF01003760.1	2111.34	1453.54	2565.21	692.2	411.3	439.63
GAIF01003762.1	0	0	0	0	0.39	0
GAIF01003763.1	11.36	3.31	3.11	15.67	17.63	14.95
GAIF01003764.1	0	0	0	0	0.19	0
GAIF01003765.1	3247.25	1552.54	1144.35	1744.73	1567.59	1168.07
GAIF01003766.1	0	0	0	0	0	0.09
GAIF01003767.1	0	0	0	0	1.17	0
GAIF01003769.1	0.14	0.16	0	0.85	4.26	0.08
GAIF01003770.1	1.11	0.58	0.6	1.6	1.66	0
GAIF01003771.1	0.62	0.67	0.27	0.86	0.74	0
GAIF01003772.1	133.2	33.78	54.37	52.64	37.48	38.6
GAIF01003773.1	1.22	1.51	0	0.35	0.08	0.2
GAIF01003774.1	0	0	0	0	0	0.04
GAIF01003775.1	1.24	0.43	0	0	0.3	0.24
GAIF01003776.1	0.11	0	0	0	0.12	0
GAIF01003777.1	0.42	0.09	0.09	4.5	19.25	0.37
GAIF01003778.1	1.16	2.65	1.31	9.96	22.53	1.71
GAIF01003780.1	0.02	0	0	0	0	0.04
GAIF01003781.1	7.77	15.02	4.13	18.5	21.14	0.63
GAIF01003782.1	0	0	0	0	0.38	0
GAIF01003783.1	1.57	1.87	0.79	3.85	2.84	0.65
GAIF01003785.1	0.11	0.06	0.06	0.39	1.29	0.06
GAIF01003786.1	13.66	10.98	11.81	27.31	47.9	16.68
GAIF01003789.1	0.28	0.15	0	1.35	23.97	34.25
GAIF01003790.1	0	0	0	0	0.48	0

GAIF01003791.1	0.78	1	1.49	1.64	1.5	0.98
GAIF01003792.1	3.08	2.99	0.48	18.87	34.08	0.85
GAIF01003794.1	7.61	5.74	7.87	17.56	10.05	13.67
GAIF01003796.1	0.77	0.46	0.54	3.75	8.36	3.79
GAIF01003797.1	135.48	216.05	299.12	134.02	105.58	79.99
GAIF01003798.1	114.4	113.99	154.87	65.46	66.28	234.79
GAIF01003799.1	0.49	0.21	0.11	1.13	0	0.21
GAIF01003802.1	13.47	5.67	23.55	39.37	13.81	9.45
GAIF01003803.1	8.1	2.82	5.6	16.72	14.04	21.4
GAIF01003804.1	9.52	4.02	1.12	1.39	0.33	0.1
GAIF01003805.1	18.33	6.63	2.17	1.25	2.98	0.65
GAIF01003806.1	0	0	0	0.06	0.12	0
GAIF01003807.1	10.13	11.18	5.71	99.08	111.81	6.22
GAIF01003808.1	1.19	0.73	1.06	1.95	1.48	0
GAIF01003809.1	5.63	13.11	1.26	9.53	22.79	0.95
GAIF01003810.1	1.83	2.2	1.03	4.22	14.68	0.37
GAIF01003811.1	8.05	2.28	0.35	12.98	18.96	2.84
GAIF01003814.1	2.06	1.77	0.24	12.73	22.26	0.48
GAIF01003815.1	0	0	0	0	0.16	0
GAIF01003817.1	33.58	44.9	27.3	74.9	84.24	10.64
GAIF01003819.1	0	0.18	0.18	0.75	1.55	0
GAIF01003820.1	17.55	20.82	20.15	15.16	9.54	5.12
GAIF01003821.1	40.81	70.82	97.38	105.28	98.31	31.96
GAIF01003822.1	4.39	8.5	4.49	3.66	2.99	0.47
GAIF01003824.1	46.79	39.33	20.12	41.25	35.06	3.21
GAIF01003825.1	0.16	0.17	0.52	0.91	3.78	0.17
GAIF01003826.1	0.29	0	0	0.67	3.82	0.74
GAIF01003827.1	0.7	0	0.05	0.81	3.98	0.21
GAIF01003828.1	1.09	0.94	2.16	0.53	0.51	1.1
GAIF01003829.1	0.22	0.18	0	2.81	7.64	0.06
GAIF01003830.1	0	0	0	0.97	4.2	0
GAIF01003831.1	0.18	0.17	0.23	0	2.01	0
GAIF01003832.1	0	0	0	0.32	2.2	0.14
GAIF01003833.1	3.61	4.14	3.89	4.42	3.02	2.62
GAIF01003835.1	4.43	9.97	2.53	15.64	16.21	0.14
GAIF01003836.1	0.05	0	0	0.41	1.28	0.08
GAIF01003837.1	6.37	3.87	3.74	15.09	10.43	10.82
GAIF01003838.1	0.23	0.06	0.08	1.21	5.22	0.19
GAIF01003839.1	0.73	2.01	2.58	26.46	24.64	87.67
GAIF01003840.1	0	2.25	4.75	0	0	0
GAIF01003843.1	0.4	0.44	1.32	0.46	0.5	0.59
GAIF01003844.1	16.73	30.37	16.65	28.29	17.28	9.47
GAIF01003846.1	0.08	0.09	0.09	0.28	2.39	0
GAIF01003847.1	26.58	14.33	12.15	19.61	30.61	15.21

GAIF01003848.1	0.19	0	0	0.55	1.25	3.23
GAIF01003850.1	0.11	0.29	0.21	0.44	0.43	0.04
GAIF01003852.1	65.14	55.1	85.2	247.81	217.5	247.55
GAIF01003854.1	0.87	0.8	0	2.39	1.77	0.82
GAIF01003855.1	5.36	7.49	7.34	4.97	1.93	0.59
GAIF01003856.1	381.73	291.62	484.16	105.56	45.01	155.47
GAIF01003857.1	0	0	0	0	0.45	0
GAIF01003858.1	0	0	1.54	0	0	0
GAIF01003859.1	0.12	0.16	0	0.81	1.88	0
GAIF01003860.1	29.35	20.95	13.62	10.82	12.91	1.13
GAIF01003862.1	1.34	0.89	0	4.6	8.19	0.19
GAIF01003867.1	6.99	0.37	3.65	48.45	62.19	7.14
GAIF01003869.1	0.1	0	0	1.32	2.86	0
GAIF01003870.1	58.75	92.84	193.73	94.08	68.4	53.4
GAIF01003872.1	9.3	0	1.82	0.02	0	0
GAIF01003873.1	0	2.8	0	2.53	2.87	0
GAIF01003875.1	0	0.15	2.04	0	0	0
GAIF01003876.1	0	0	0	0.13	0.14	0
GAIF01003877.1	0	0	0	0	1.45	0
GAIF01003881.1	0.12	0	0.13	0.28	1.87	0.39
GAIF01003883.1	0	0	0	0.39	1.74	0.17
GAIF01003884.1	0.04	0	0.04	0.34	0.31	0.2
GAIF01003886.1	405.89	136.89	32.43	372.3	425.25	28.67
GAIF01003887.1	0	0.18	0	0.22	0.21	0.37
GAIF01003888.1	0	0	0	0.07	2.63	0.01
GAIF01003889.1	0.04	0	0	0	2.23	0
GAIF01003890.1	0.29	0	0	1.18	4.02	0.64
GAIF01003891.1	0	0	0.53	0	0	0
GAIF01003893.1	74.6	124.62	51.88	162.5	106.8	19.5
GAIF01003895.1	56.54	42.78	130.69	121.57	69.63	184
GAIF01003896.1	1.89	1.42	0.29	3.8	8.05	1.31
GAIF01003897.1	89.25	64.03	161.63	35.24	16.6	66.93
GAIF01003898.1	0	0	0	0	0.36	0
GAIF01003899.1	0.04	0	0	0	0	0.21
GAIF01003900.1	4.62	6.35	5.07	2.76	1.22	0.07
GAIF01003901.1	2.76	1.24	1.23	3.09	8.17	10.39
GAIF01003902.1	5.01	3.43	0.59	0.66	0.04	0.03
GAIF01003903.1	0.92	1.04	1.68	1.61	0.85	1.76
GAIF01003904.1	0.3	0	0	1.78	11.15	0.15
GAIF01003905.1	0	0	0	0	0.64	0
GAIF01003907.1	11.93	11.47	13.21	42.71	31.6	12.73
GAIF01003909.1	7.94	5.45	8.02	1.45	1.84	0.31
GAIF01003910.1	2.65	1.1	2.36	6.01	7.44	4.59
GAIF01003911.1	0	0.08	0.13	0.49	4.69	0.09

GAIF01003912.1	8.33	4.99	11.53	8.55	3.92	14.9
GAIF01003913.1	0	0	0	0	0.22	0
GAIF01003914.1	6.93	12.05	3.24	19.18	14.12	2.4
GAIF01003915.1	3.46	4.48	4.53	3.57	6.96	0.62
GAIF01003916.1	34.59	26.65	39.38	30.52	44.42	58.65
GAIF01003917.1	0.03	0.07	0	0.82	4.48	0
GAIF01003919.1	5.19	7.28	20.91	0	0	5.92
GAIF01003920.1	0.16	0.37	0	1.63	3.96	0
GAIF01003922.1	1.46	1	0.2	1.91	9.69	0.81
GAIF01003923.1	0	0.13	0.13	0.54	0.56	0.52
GAIF01003924.1	4.98	3.9	3.65	2.26	0.05	0.84
GAIF01003927.1	22.27	24.47	15.79	10.62	7.24	2.85
GAIF01003928.1	0	0	0	0	0.11	0.1
GAIF01003930.1	0	0	0	24.2	29.49	1.93
GAIF01003931.1	3.85	2.92	1.47	11.92	16.22	0.71
GAIF01003933.1	0.3	0.45	0	0.28	0.24	0
GAIF01003934.1	2.21	2.95	1.31	3.66	5.3	0.21
GAIF01003935.1	0	0	0	0	0.54	0
GAIF01003936.1	0	0	0	0	0.83	0
GAIF01003937.1	297.73	595.67	288.06	39.43	63.96	19.67
GAIF01003938.1	0.56	0.2	0.14	0.8	0.22	0.55
GAIF01003939.1	1.68	0.15	0	4.38	1.55	2.73
GAIF01003940.1	0	0	0	0	0.17	0
GAIF01003941.1	6.74	9.82	12.38	36.88	19.94	12.06
GAIF01003942.1	45.08	44.85	20.96	79.74	90.88	6.88
GAIF01003944.1	0	0	0	0	0.04	0
GAIF01003946.1	0	0	0	0.09	0.73	0
GAIF01003948.1	11.79	8.82	8.86	2.23	0.9	0.74
GAIF01003949.1	71.43	33.83	26.84	134.39	165.39	59.33
GAIF01003950.1	0.13	0	0.15	0.05	0.03	0
GAIF01003951.1	0	0	0.05	0.29	0.48	0.11
GAIF01003953.1	0	0.06	0	0.08	0.26	0
GAIF01003955.1	1.1	0	0.03	0.53	0.04	0
GAIF01003957.1	0	0.11	0.11	0.12	0.12	0
GAIF01003959.1	1.35	1.08	0.47	2.91	4.59	0.56
GAIF01003961.1	0.29	0	0	0.11	0.18	0
GAIF01003962.1	0	0	0	0	0.03	0.08
GAIF01003963.1	0	0	0	0	0.49	0
GAIF01003964.1	0.01	0	0	0	0	1.69
GAIF01003965.1	4.22	1.85	0	0.61	0.38	0
GAIF01003967.1	0	0.11	0	0.06	0.22	0
GAIF01003968.1	1.46	0.74	0.19	0.59	0.62	0
GAIF01003969.1	27.65	21.01	9.22	74.05	110.4	3.28
GAIF01003971.1	0.05	0.05	0.05	0	0	0.11

GAIF01003973.1	0.77	0	1.37	1.9	2.33	2.87
GAIF01003974.1	0.09	0.09	0.1	0.05	0.05	0.14
GAIF01003975.1	23.72	52.68	41.27	30.29	22.27	7.34
GAIF01003977.1	0.49	0	0	0.28	0.92	0
GAIF01003978.1	0.03	0	0.3	0	0	0
GAIF01003979.1	0	0	0	3.39	7.43	0
GAIF01003980.1	19.28	11.83	21.67	24.64	22.76	49.67
GAIF01003981.1	0.08	0	0	0	0	0
GAIF01003982.1	0	0	0	0	0.15	0.03
GAIF01003984.1	21.27	34.51	17.69	60.28	55.02	5.2
GAIF01003985.1	4001.17	2946.9	1977	2715.99	2123.19	2195.99
GAIF01003987.1	0.06	0	0	0	0	0
GAIF01003989.1	0	0.23	0.23	0	0	0.06
GAIF01003990.1	0.59	0	0	0.32	3.4	2.24
GAIF01003991.1	0.08	0.13	0.26	0.64	0.71	0
GAIF01003994.1	0.26	0	0	0	2.15	0
GAIF01003995.1	6.62	8.48	7.55	20.91	17.02	5.56
GAIF01003996.1	4.29	1.78	1.73	8.02	19.84	0.56
GAIF01003997.1	0	0.07	0.15	0.32	0.98	0
GAIF01003998.1	0.11	0.22	0.39	0.45	0.18	0
GAIF01003999.1	24.51	37.72	57.95	8.39	1.46	15.36
GAIF01004000.1	0.11	0	0	0.12	1.41	0.08
GAIF01004001.1	2.16	1.15	1.02	5.07	4.64	0.82
GAIF01004003.1	0.3	0	0	0.17	0.18	0
GAIF01004004.1	0.12	0	0	0.49	0	0.13
GAIF01004005.1	6.19	10.27	5.87	35.41	26.44	1.11
GAIF01004006.1	88.81	95.42	121.23	120.56	140.05	313.33
GAIF01004007.1	9.39	14.14	8.73	35.5	18.39	7.3
GAIF01004008.1	188.55	102.76	166.27	108.44	35.28	68.31
GAIF01004010.1	4.02	5.71	2.97	5.7	12.31	3.56
GAIF01004011.1	0.1	0.55	0.33	0	0.12	0.27
GAIF01004013.1	0	0	0	0	0.12	0
GAIF01004014.1	2.89	0.27	0	0.81	0	0
GAIF01004015.1	0	0.04	0	0	0.04	0
GAIF01004016.1	56.55	85.49	67.48	108.39	77.83	15.12
GAIF01004017.1	0.4	0	0.13	0.67	1.25	0.13
GAIF01004018.1	0	0	0	0	2.38	0
GAIF01004019.1	11.5	17.91	31.45	23.56	16.55	10.44
GAIF01004020.1	0	0	0	0	0.09	0
GAIF01004022.1	0.09	0	0	0	0.29	0.06
GAIF01004023.1	22.75	15.55	14.4	33.94	22.84	8.95
GAIF01004024.1	22.12	34.41	3.16	28.13	68.59	4.23
GAIF01004025.1	0	0	0.07	0.3	0.95	0
GAIF01004026.1	0.41	1.87	1.56	0.12	0.12	0

GAIF01004027.1	1.18	2.92	0.61	3.31	3.23	0.02
GAIF01004029.1	0.05	0.16	0.08	0.06	0.09	0
GAIF01004030.1	585.1	317.25	586.75	142.33	84.51	285.4
GAIF01004033.1	0	0	0	0	0.12	0
GAIF01004034.1	0	0	0	0.14	0	0
GAIF01004035.1	20.55	16.66	22.96	40.7	20.05	44.95
GAIF01004036.1	0.16	0	0.09	0.14	0.37	0.18
GAIF01004037.1	0	0	0	16.16	20.6	0
GAIF01004038.1	7.15	1.15	0.61	14.72	37.54	4.42
GAIF01004041.1	0	0	0	0	0.07	0
GAIF01004042.1	0.14	0.06	0.12	0.6	3.35	0
GAIF01004043.1	44.41	46.64	15.59	30.32	60.22	56.54
GAIF01004044.1	0.04	0	0	0	0	0
GAIF01004045.1	0.04	0	0	0	0.17	0
GAIF01004046.1	1784.87	340.52	548.26	914.46	316.38	2004.09
GAIF01004047.1	4.37	0	0	4.84	15.52	16.28
GAIF01004051.1	14.57	10.62	25.44	25.92	24.57	17.8
GAIF01004052.1	1.01	0.45	0	9.01	20.37	0.68
GAIF01004053.1	3.99	5.7	5.51	12.98	7.34	1.22
GAIF01004055.1	27.49	12.06	1.99	21.17	20.37	6.06
GAIF01004056.1	2.23	0.64	0	3.5	43.32	84.87
GAIF01004057.1	2.15	1.51	1.77	0.18	0.19	0
GAIF01004058.1	0.81	0.21	0.11	4.9	18.77	0.25
GAIF01004059.1	0	0.11	0	0	0	0
GAIF01004060.1	40.15	71.57	10.29	71.32	93.71	2.07
GAIF01004062.1	0	0	0	0.23	0.36	0
GAIF01004063.1	63.48	126.61	201.69	505.24	318.97	165.29
GAIF01004064.1	0	0	0	0.88	2.99	0.78
GAIF01004065.1	0.07	0.07	0	0	0.25	0
GAIF01004066.1	0.42	0.68	0	1.43	1.84	0
GAIF01004068.1	0.38	0.21	0.16	3.38	1.9	0.11
GAIF01004070.1	16.95	11.26	17.73	35.83	34.5	17.53
GAIF01004071.1	0	0	0	0	0.04	0
GAIF01004072.1	0.2	0.04	0.04	5.06	17.29	0.44
GAIF01004073.1	0.41	0.32	0.92	1.06	1.32	0.75
GAIF01004074.1	0	0.15	0	0.09	0.5	0
GAIF01004075.1	0	0	0	0.39	0.55	0
GAIF01004077.1	24.67	19.11	40.5	60.76	42.8	63.66
GAIF01004079.1	0.06	0	0	0	0	0
GAIF01004080.1	0.16	0.27	0.15	0.09	0.2	0
GAIF01004081.1	0.21	0.23	0.4	0.94	1.03	0.23
GAIF01004082.1	0	0	0	0.06	0.06	0
GAIF01004083.1	193.04	55.82	88.24	125.13	83.53	672.61
GAIF01004085.1	120.18	81.12	115.57	141.54	40.78	48.35

GAIF01004087.1	0	0	0.07	0.11	0.5	0
GAIF01004089.1	0.12	0.13	0	0	0	0
GAIF01004090.1	0	0	0	0.18	0	0
GAIF01004092.1	0.07	0.04	0.06	0.56	2.03	0.32
GAIF01004093.1	0	0.01	0	0	0	0
GAIF01004095.1	0.58	0.46	0.1	0.63	0.71	0.59
GAIF01004096.1	0.61	0.52	0.95	0.77	0.02	0
GAIF01004098.1	0	0	7.01	20.92	20.63	1.36
GAIF01004099.1	161.48	185.47	380.23	156.54	104.9	277.56
GAIF01004100.1	1.42	3.35	3.21	2.02	0.48	2.26
GAIF01004101.1	5.32	7.56	11.86	27.33	16.98	5.92
GAIF01004102.1	1.16	3.04	2.39	3.51	3.64	0.17
GAIF01004103.1	3.74	1.5	4.76	4.31	3.3	4.76
GAIF01004104.1	13.01	7.74	6.23	36.34	36.59	29.03
GAIF01004105.1	5.99	18.23	4.38	28.96	58.57	11.88
GAIF01004106.1	29.28	15.04	33.53	3.32	2.64	3.82
GAIF01004107.1	0.13	0	0	1.85	8.77	0
GAIF01004109.1	0.84	0.61	1.53	1.78	3.02	0.62
GAIF01004111.1	1.02	0.1	0.03	0.14	0.04	0
GAIF01004112.1	0	0	0	0	0.12	0
GAIF01004113.1	0.35	0.57	1.15	1.63	0.63	0.39
GAIF01004114.1	0	0	0	0.43	3.57	0
GAIF01004115.1	0	0	2.76	0	0	2.04
GAIF01004116.1	0.44	0.58	0.2	0.92	1.17	0.1
GAIF01004118.1	0.22	0.12	0.13	0.41	1.26	0
GAIF01004120.1	0.18	0	0	0	1.25	0.4
GAIF01004121.1	0.22	0.37	0.49	0.13	0.14	0
GAIF01004122.1	0	0	0	0.21	0	0
GAIF01004123.1	17.12	3.53	1.73	102.28	237.73	28.63
GAIF01004126.1	3.49	3.06	2.1	11.38	16.4	1.99
GAIF01004127.1	0	0	0	0.02	0.04	0
GAIF01004129.1	5.73	1.26	0.68	9.38	88.11	7.1
GAIF01004131.1	2.07	2.17	6.09	0.32	0.48	0.93
GAIF01004133.1	0	0	0	0.11	0.11	0
GAIF01004134.1	0	0	0	0	0.07	0
GAIF01004135.1	162.24	355.03	1036.29	78.38	40.52	114.07
GAIF01004136.1	1.72	1.05	1.06	0.08	0.08	0.3
GAIF01004141.1	0	0	0	0.05	0.11	0
GAIF01004142.1	0.2	0	0.65	0.39	0.18	0
GAIF01004143.1	0.06	0.1	0	0.27	2.47	0
GAIF01004144.1	7.62	7.28	6.45	1.6	0.22	0.3
GAIF01004145.1	3.69	0.8	0.27	13.91	45.67	14.38
GAIF01004146.1	0.96	2.96	0.7	4.27	4.04	0.35
GAIF01004147.1	0	0	0.1	0	0	0

GAIF01004148.1	0.11	0	0	0	0	0
GAIF01004149.1	212.55	137.29	53.89	0.8	0.65	0
GAIF01004150.1	0.52	1.27	0.93	1.66	0.62	0.64
GAIF01004152.1	26.74	67.57	43.37	6.29	3.33	0.53
GAIF01004154.1	9.37	14.26	8.23	6.52	4.51	2.06
GAIF01004155.1	0	0	0.08	0	0.21	0.21
GAIF01004156.1	0	0.38	0	0.26	0	0
GAIF01004158.1	0.77	0.26	0	1.74	10.33	0.27
GAIF01004159.1	0.93	0.83	0.41	0.4	2.04	0.83
GAIF01004160.1	0.31	0	0	1.83	7.17	0
GAIF01004161.1	3.59	2.03	2.21	8.27	17.96	3.41
GAIF01004162.1	0.15	0	0.06	2.26	6.77	0.45
GAIF01004164.1	0.52	0.53	0.26	0.23	2.64	0.32
GAIF01004165.1	25.47	23.88	20.11	63.04	60.69	24.54
GAIF01004166.1	1.71	2.04	0.38	6.74	7.6	0.19
GAIF01004168.1	1.36	0	1.23	2.28	2.77	0
GAIF01004169.1	0	0.82	0	0	0	0
GAIF01004170.1	0	0.03	0	0.03	0	0
GAIF01004171.1	0.02	0	0	0	0	0
GAIF01004172.1	0.04	0	0	1.13	0.56	0.21
GAIF01004173.1	0.32	0.31	0.84	1.52	0.96	0.43
GAIF01004174.1	0	0	0	0	0.38	0
GAIF01004175.1	0.65	0	0.14	0	0	0
GAIF01004176.1	0.13	0.09	0	0.58	2.59	0.27
GAIF01004178.1	0	0	0	0.01	0	0
GAIF01004179.1	68.78	9.3	44.28	38.63	27.59	151.06
GAIF01004183.1	4.54	2.41	1.93	6.79	9.23	3.26
GAIF01004184.1	2581.08	1376.95	633.74	1864.59	2109.47	5352.67
GAIF01004185.1	0.21	0.12	0.33	0.26	0.66	0.97
GAIF01004186.1	3.49	5.94	2.07	24.1	35.64	0.71
GAIF01004189.1	0.02	0.23	0	0.16	0.26	0
GAIF01004190.1	1.01	0.18	0	8.38	27.85	2.59
GAIF01004191.1	0	0	0.15	0	0	0.3
GAIF01004192.1	17.42	26.94	13.74	22.16	10.71	3.98
GAIF01004193.1	17.46	14.45	14.44	54.12	60.47	21.04
GAIF01004194.1	0	0	0	0.35	1.09	0
GAIF01004195.1	11.04	21.85	13.4	13.99	15.32	2.08
GAIF01004196.1	0.43	4.26	0	6.53	12.71	0
GAIF01004198.1	32.01	17.87	20.07	24.76	27.6	73.78
GAIF01004200.1	3.19	1.73	0.97	7.75	17.06	2.47
GAIF01004201.1	0	0.41	0.48	0	0.07	0.27
GAIF01004203.1	57.25	31.62	16.02	63.96	36.03	7.56
GAIF01004204.1	0.07	0	0	0.25	0.8	0.75
GAIF01004205.1	0.08	0.09	0	0.36	0.38	0

GAIF01004208.1	1.7	1.01	0.51	2.69	4.46	1.36
GAIF01004209.1	5.48	7.32	6.52	13.08	13.55	2.2
GAIF01004210.1	0	0	0	0	0.4	0.25
GAIF01004212.1	2.39	4.02	2	2.42	0.31	0.06
GAIF01004213.1	26.01	14.88	20.67	5.18	2.1	2.03
GAIF01004214.1	8.67	14.88	16.78	13.27	9.97	5.66
GAIF01004215.1	0.13	0.1	0	0	1.43	0
GAIF01004221.1	0	0	0	0.1	0	0.1
GAIF01004222.1	0	0	0	0	0.04	0
GAIF01004223.1	0.11	0.31	0.12	0.54	1.24	0.04
GAIF01004224.1	0.03	0	0	0.08	0.61	0.04
GAIF01004226.1	0.73	0	0.33	2.11	6.78	0.29
GAIF01004227.1	0.61	0.11	0.12	0.33	0.33	0.23
GAIF01004230.1	0	0.21	0	0	0	0
GAIF01004231.1	0.78	0.49	0.78	0.45	0.08	0.07
GAIF01004232.1	14.49	6.63	4.57	23.62	33.19	16.33
GAIF01004233.1	0	0	0	0.73	2.42	0.34
GAIF01004234.1	0	0.03	0	0	0	0
GAIF01004237.1	0	0	0	0	0.15	0
GAIF01004238.1	66.21	46.08	48.13	57.81	0	16.12
GAIF01004240.1	0	0	0	0.31	0.64	0.15
GAIF01004241.1	0.11	0	0	0	0.63	0
GAIF01004243.1	15.86	25.41	19.04	2.74	5.42	5.06
GAIF01004245.1	12.73	7.51	8.75	23.6	22	14.17
GAIF01004246.1	0	1.55	2.03	0	0	0.09
GAIF01004247.1	10.56	25.31	13.04	18.7	20.06	1.87
GAIF01004248.1	0.41	0.21	0	0	0.24	0
GAIF01004249.1	1.3	2.4	0.43	3.08	4.08	0.12
GAIF01004250.1	3.42	0.67	0.06	8.12	31.77	4.9
GAIF01004252.1	2.01	2.28	2.01	3.85	0.42	1.83
GAIF01004253.1	1.15	1.12	0	3.86	7.44	0
GAIF01004254.1	0	0	0	0.62	0.33	0
GAIF01004257.1	0	2.17	0.14	0	0	0
GAIF01004258.1	0.13	12.72	0	17.63	11.89	4.07
GAIF01004259.1	23.62	16.05	14.69	25.38	21.86	7.52
GAIF01004263.1	0.55	0	0.36	6.34	20.88	0.18
GAIF01004264.1	0	0.06	0	0.08	0.3	0
GAIF01004265.1	0	0	0	0	0.07	0
GAIF01004266.1	0.42	0	0	3.85	29.32	0.14
GAIF01004269.1	0.35	0	0	0.82	2.98	0
GAIF01004271.1	0	0	0	0	0.66	0
GAIF01004272.1	257.67	166.33	138.63	176.13	241.32	339.68
GAIF01004274.1	0	0.05	0	0	0	0
GAIF01004275.1	18.73	13.22	14.43	26.78	18.06	20.8

GAIF01004276.1	0	0	0	0.14	0	0
GAIF01004277.1	31.32	71.08	112.06	57.27	59.86	18.4
GAIF01004279.1	0	0	0	0.88	2.27	0
GAIF01004280.1	312.71	332.91	406.79	13.25	10.51	10.69
GAIF01004281.1	261.03	318.89	212.43	567.15	429.32	95.09
GAIF01004282.1	0	0	0	0.57	1.77	0.09
GAIF01004283.1	0	0	0	0.09	0	0
GAIF01004284.1	167.45	102.55	94.09	269.9	112.41	88.09
GAIF01004285.1	0	0	0	0.05	0.43	0
GAIF01004287.1	0	0	0	0	1.47	0.71
GAIF01004289.1	16.5	19.73	29.03	46.48	27.62	19.89
GAIF01004290.1	13.95	6.57	0.84	22.45	21.57	6.07
GAIF01004291.1	0.02	0.02	0.02	0.07	0.43	0
GAIF01004292.1	0.12	0.25	0.25	0	0.28	0
GAIF01004295.1	0.11	0	0	1.6	10.52	0.06
GAIF01004296.1	732.97	578.07	560.75	455.11	297.98	222.32
GAIF01004297.1	0.12	0.04	0.09	0.43	2.06	0
GAIF01004298.1	29.23	20.39	12.99	64.33	118.85	31.46
GAIF01004299.1	32.26	0	0	89.86	95.46	13.6
GAIF01004300.1	0	0	0	0.09	0.18	0
GAIF01004301.1	12.64	9.87	9.97	30.04	22.57	12.84
GAIF01004302.1	0	0	0	4.47	0	0.13
GAIF01004305.1	0	0.43	0.22	0.09	0	0.04
GAIF01004306.1	114.25	61.1	58.54	96.23	72.08	51.61
GAIF01004307.1	0	0	0	0	0.14	0
GAIF01004308.1	87.22	143.44	165.24	111.21	100.32	107.94
GAIF01004309.1	0	0.11	0.06	0	0	0
GAIF01004310.1	1.47	4.53	0.6	33.82	30.76	12.07
GAIF01004311.1	1.51	0.62	0.09	1.99	2.61	0.19
GAIF01004312.1	3.74	10.77	4.96	12.1	30.73	0.67
GAIF01004314.1	3.59	0	0	18.55	35.63	13.92
GAIF01004315.1	20.78	11.06	6.67	50.23	62.32	7.74
GAIF01004316.1	0.38	0	0	0.22	0	0
GAIF01004317.1	0	0	0	0.1	0.4	0
GAIF01004318.1	0.23	0	0.09	1	2.47	1.12
GAIF01004319.1	0	0	0	0	0	0.05
GAIF01004320.1	0.44	0.18	0.18	1.79	9.53	1.39
GAIF01004321.1	1.86	1.82	1.41	5.92	11.38	0.3
GAIF01004324.1	3.41	3.61	4.44	9.88	9.71	2.43
GAIF01004325.1	0	0	0	0.14	0.3	0
GAIF01004326.1	0	0	0	0	0	0.21
GAIF01004327.1	0	0	0	0.16	0.83	0.15
GAIF01004329.1	28.65	25.96	15.88	36.34	16.89	4.8
GAIF01004331.1	0.12	0	0.17	0.05	0.14	0

GAIF01004332.1	4.07	1.54	5.08	4.52	28.05	5.62
GAIF01004333.1	7.94	5.2	1.31	25.41	38.8	3.74
GAIF01004334.1	1.74	0.82	0.22	6.68	7.13	0.08
GAIF01004335.1	0.22	0.31	0	1.03	7.77	0
GAIF01004336.1	17.81	12.23	17.5	56.51	81.5	22.57
GAIF01004337.1	0.65	0.24	0.08	1.9	4.32	0.08
GAIF01004338.1	0.14	0	0	0	0	0
GAIF01004339.1	0.14	0.25	0.62	0	2.08	0.12
GAIF01004341.1	0.19	0.63	0	0.66	0.66	0.55
GAIF01004342.1	62.91	69.99	51.21	133.05	155.38	125.39
GAIF01004343.1	0.35	0.29	0.34	0.63	1.44	1.62
GAIF01004344.1	2.21	1.36	1.12	2.03	3.36	0.3
GAIF01004345.1	0	0	0	0	0	0.13
GAIF01004346.1	22.93	18.49	23.79	61.43	71.78	30.46
GAIF01004349.1	21.98	14.74	10.27	50.83	41.99	2.23
GAIF01004351.1	28.45	10.27	12.97	8.62	4.51	1.22
GAIF01004355.1	8.21	12.95	6.72	22.81	29.14	1.78
GAIF01004356.1	0	0.07	0.03	0	0.28	0
GAIF01004359.1	0	0	0	0	0.15	0
GAIF01004360.1	7.77	9.55	7.09	9.02	10.21	3.17
GAIF01004362.1	265.42	447.68	351.75	262.41	249.7	110.01
GAIF01004364.1	6.8	2.11	2.77	14.74	21.43	35.13
GAIF01004366.1	0.05	0.05	0.05	0.04	0.17	0
GAIF01004369.1	0	0	1.01	0	0	0.13
GAIF01004370.1	69.57	46.6	34.45	15.4	6.68	4.74
GAIF01004372.1	0	0	0	0	0.07	0
GAIF01004373.1	2.34	0	0	0.36	0	0
GAIF01004374.1	3.68	1.22	0	10.41	11.17	0.35
GAIF01004375.1	3.85	3.14	1.58	5.72	13.29	9.93
GAIF01004376.1	0	0	0	4.54	20.4	0.42
GAIF01004377.1	12.09	12.33	15.91	48.04	44.01	12.23
GAIF01004381.1	0	0	0	0.36	0.98	0
GAIF01004382.1	1.63	0.62	0.23	3.95	11.92	0.47
GAIF01004383.1	0.15	0.08	0.25	0.04	0.11	0.17
GAIF01004384.1	5.19	9.48	7.97	14.09	8.61	2.06
GAIF01004385.1	5.28	3.53	3.19	14.19	22.73	2.65
GAIF01004386.1	1.51	1.03	0.21	1.97	0.91	0
GAIF01004387.1	0.07	0	0	0.08	0	0
GAIF01004388.1	0.22	0	0	0.75	7.34	0.17
GAIF01004389.1	75.51	71.95	80.63	31.27	14.89	15.81
GAIF01004390.1	0	0.06	0	0.07	0.81	0.06
GAIF01004391.1	0.1	0.03	0	0.06	0.01	0
GAIF01004393.1	23.6	26.51	10.77	48.01	60.32	3.01
GAIF01004395.1	0.42	0.43	0	0.28	0	0

GAIF01004396.1	1.22	0.93	1.47	6.07	8.33	0.54
GAIF01004398.1	2.86	8.04	3.13	4.17	8.62	0.89
GAIF01004402.1	0.01	0.06	0.17	0.42	0.94	0.09
GAIF01004404.1	0.16	1.23	2.42	0.68	0.58	2.9
GAIF01004405.1	6.78	7.21	3.18	7.28	4.85	2.56
GAIF01004406.1	7.23	6.64	13.97	16.54	15.71	9.07
GAIF01004407.1	2.82	0.58	0.13	3.1	11.6	9.45
GAIF01004408.1	5.23	4.8	4.56	10.54	8.86	8.26
GAIF01004409.1	4.96	2.06	4.08	2.05	1.9	5.49
GAIF01004410.1	0.06	0.12	0	0	1	0
GAIF01004411.1	0.09	0.19	0	0.1	0.41	0
GAIF01004412.1	0.16	0	0.06	0	0	0
GAIF01004414.1	0.69	0	0	0.4	1.1	0
GAIF01004415.1	4.32	9.14	0.91	6.89	27.3	0
GAIF01004416.1	0	0	0.09	0	0.1	0
GAIF01004417.1	0	0	0.09	0	0	0
GAIF01004419.1	7.85	8.19	15.44	4.01	6.08	16.79
GAIF01004421.1	0.21	0.11	0	0.24	0.5	0
GAIF01004422.1	0	0	0	0	0	0.25
GAIF01004423.1	34.31	25.33	12.93	31.3	37.55	6.81
GAIF01004424.1	26.93	27.19	21.69	35.54	28.78	4.53
GAIF01004425.1	0	0	0	0.28	0.94	0.1
GAIF01004426.1	0.92	1.35	0.93	0.67	0.68	0.24
GAIF01004428.1	0.6	0.54	0.22	1.85	2.51	0.22
GAIF01004429.1	3.74	0.69	1.67	2.47	1.32	1.89
GAIF01004430.1	7.05	8.68	12.32	28.05	16.69	15.29
GAIF01004431.1	5.04	3.85	9.38	3.5	0.2	1.42
GAIF01004432.1	0.48	0	0	0	0.14	0
GAIF01004433.1	0	0	0	0	0.08	0
GAIF01004434.1	17.83	12.29	9.96	57.49	52.74	10.87
GAIF01004436.1	11.91	21.95	22.41	53.17	30.96	7.12
GAIF01004437.1	6.94	10.41	11.45	7.65	7.94	6.93
GAIF01004439.1	0	0.35	0	0	0	1.04
GAIF01004441.1	0.02	0.02	0	0.06	1.24	0
GAIF01004442.1	2.35	3.65	1.48	10.46	8.74	0.86
GAIF01004443.1	0.13	0.04	0	0	0.12	0.04
GAIF01004444.1	98.87	40.35	49.83	78.59	49.69	187.42
GAIF01004445.1	3.97	1.94	0.53	1.35	4.12	2.94
GAIF01004447.1	11.16	3.63	0	40.14	42.34	2.46
GAIF01004448.1	0	0	0	0.22	0	0
GAIF01004450.1	0	0	0	0.06	0.39	0.06
GAIF01004451.1	0	0	0	0.07	0	0
GAIF01004452.1	0	0	0	0	0.58	0
GAIF01004453.1	1.05	0.76	1.67	2.31	1.13	1.03

GAIF01004454.1	6.98	1.08	0.14	3.18	7.35	0.27
GAIF01004455.1	6.17	3.72	1.61	10.64	19.71	9.64
GAIF01004456.1	15.4	14.07	14.66	32.48	51.56	42.61
GAIF01004457.1	0	0	0.05	0.01	0	0
GAIF01004458.1	1.45	2.1	1.13	2.14	3.74	0.73
GAIF01004461.1	0	0.09	0.16	0	0	0.21
GAIF01004462.1	0.06	0.06	0	0	0	0
GAIF01004463.1	10.37	10.99	6.44	2.55	1.89	0.69
GAIF01004464.1	0	0.24	0.97	0	0	1.05
GAIF01004466.1	2.1	0.74	0.89	1.02	2.69	0.07
GAIF01004468.1	0	0.08	0	0	0	0
GAIF01004470.1	0.36	0	0.08	1.09	0.64	2.25
GAIF01004472.1	35.68	20.84	14.36	32.32	64.18	10.51
GAIF01004473.1	33.07	19.17	26.38	40.89	40.75	76.26
GAIF01004474.1	1.11	1.62	0.83	0.29	0.16	0
GAIF01004475.1	0.2	0	0	0	2.89	0
GAIF01004476.1	0	0	0	0.07	0	0
GAIF01004478.1	0.01	0	0.03	0	0.94	0
GAIF01004479.1	0.11	0	0	0.07	0.23	0
GAIF01004480.1	12.74	15.08	20.88	89.17	54.09	34.78
GAIF01004482.1	0.2	0.39	0.06	0.06	0.25	0
GAIF01004483.1	0	0	0.07	0	0.08	0
GAIF01004484.1	13.76	32.94	23.67	29.32	11.77	3.35
GAIF01004487.1	1.3	0.29	0.09	1.86	3.96	0.23
GAIF01004488.1	0.06	0	0	0	0	0
GAIF01004489.1	10.4	10.96	10.89	14.3	10.65	5.29
GAIF01004490.1	34.37	35.81	50.83	80.74	36.74	35.38
GAIF01004491.1	0.29	0.08	0	1.66	7.14	0.63
GAIF01004495.1	0	0.1	0	0	0.03	0
GAIF01004497.1	12.25	10.9	5.17	2.92	0.94	0.21
GAIF01004498.1	0.09	0.05	0.05	0.05	0.34	0
GAIF01004499.1	0	0.43	0.22	0	0	0
GAIF01004500.1	0	0	0	0.43	1.22	0
GAIF01004501.1	2.9	0.92	0.13	10.95	13.09	0.67
GAIF01004503.1	0.66	0	0.22	0.12	0.42	0.55
GAIF01004504.1	0.37	0	0	1.16	9.73	0
GAIF01004505.1	6.63	13.07	13.58	4.82	3.19	2.34
GAIF01004506.1	4.01	2.18	3.96	16.74	37.74	2.35
GAIF01004507.1	0.38	0.46	0.21	1.97	5.85	0.1
GAIF01004509.1	2.05	1.07	0.45	13.23	15.09	0.54
GAIF01004511.1	0	0	0.22	0	0.25	0
GAIF01004512.1	43.16	16.78	16.18	25.35	33.44	29.71
GAIF01004514.1	42.42	27	31.64	44.3	42.13	33.35
GAIF01004515.1	34.05	0	0	0	18.29	42.12

GAIF01004517.1	0.5	0.41	0	3.61	14.66	0.96
GAIF01004518.1	26.82	25.53	19.72	29.11	37.87	22.14
GAIF01004519.1	562.74	4.28	23.62	9.13	1.51	0.83
GAIF01004520.1	0.09	0.4	0.11	0.65	1.05	0.05
GAIF01004521.1	15.46	19.25	13.93	39.25	43.49	25.93
GAIF01004524.1	19.99	37.34	35.59	21.39	52.69	16.86
GAIF01004525.1	0.08	0	0	0	0	0.09
GAIF01004526.1	0	0	0	1.72	7.63	0.39
GAIF01004527.1	6.91	12.4	10.29	32.59	23.72	4.85
GAIF01004528.1	5.8	9.56	5.6	0.2	0	0.46
GAIF01004529.1	2.11	3.94	5.92	6.55	4.46	0.28
GAIF01004530.1	0	0	0	0	0.17	0
GAIF01004531.1	0.15	0	0	1.19	2.98	1.29
GAIF01004532.1	4732.92	1172.48	2414.16	5832.03	5473.43	5592.56
GAIF01004533.1	0.67	1.62	0.08	0.95	0.81	0
GAIF01004534.1	0	0.22	0.22	0	0.32	0.15
GAIF01004535.1	0	0.21	0	0	0.24	0
GAIF01004537.1	0.84	0.33	0	0.79	0.65	0.08
GAIF01004538.1	0.03	0.06	0.06	0	0	0
GAIF01004539.1	2.56	5.56	4.92	5.14	2.69	1.03
GAIF01004542.1	21.93	8.71	16.13	37.64	58.13	152.18
GAIF01004543.1	30.16	41.71	52.87	47.51	44.21	24.36
GAIF01004545.1	14.81	21.79	16.94	26.14	10.35	4.02
GAIF01004549.1	22.06	34.02	36.72	23.93	11.1	10.97
GAIF01004550.1	9.39	11.03	11.55	23.87	26.99	4.76
GAIF01004551.1	0	0.03	0	0	0	0
GAIF01004552.1	17.31	17.99	3.55	94.79	142.49	4.54
GAIF01004554.1	0	0	0	0.22	0.41	0
GAIF01004555.1	1.1	0	0	3.48	12.51	0
GAIF01004557.1	0	0.22	0.22	0	0	0.07
GAIF01004558.1	10.01	13.44	4.68	27.19	41.11	1.84
GAIF01004559.1	0	0	0	0	0	0.43
GAIF01004560.1	0	0	0	0.06	0	0
GAIF01004561.1	22.51	15.56	24.47	40.58	35.87	23.51
GAIF01004562.1	9.54	2.04	0.34	0.91	0	0.17
GAIF01004563.1	14.79	29.22	21.57	29.62	19.69	9.21
GAIF01004565.1	0.82	0	0.08	3.82	15.99	0.26
GAIF01004566.1	0.15	0	0.05	0	0.38	0
GAIF01004567.1	4.67	6.96	10.23	11.2	7.98	3.48
GAIF01004569.1	0	0	0	0.01	0.39	0
GAIF01004571.1	119.35	113.48	118.95	104.35	55.02	41.51
GAIF01004572.1	0.31	0.08	0.6	0	0	0
GAIF01004574.1	0	0	0	0.07	0.57	0
GAIF01004575.1	0	8.17	2.11	0	0	0.35

GAIF01004576.1	0	1.47	0.25	0.98	0.9	0
GAIF01004577.1	0.43	0.31	0	0	0	0
GAIF01004579.1	0.11	0.1	0.02	0.5	1.8	0.07
GAIF01004582.1	0.14	0.15	0	0.31	0.33	0
GAIF01004583.1	235.18	248.68	237.82	152.76	62.23	58.78
GAIF01004584.1	18.36	30.19	23.86	50.81	44.85	10.57
GAIF01004585.1	0.83	1.09	2.38	1.35	2.61	0.37
GAIF01004588.1	30.25	20.01	0	18.27	8.71	0
GAIF01004589.1	2.1	0.73	1.36	0.89	2.41	2.73
GAIF01004590.1	0	0	0	0.24	1.37	0
GAIF01004591.1	0.28	0.1	0	0	0.56	0.1
GAIF01004592.1	13.63	8.35	13.99	19.88	20.28	42.35
GAIF01004593.1	0	0.2	0.2	0.77	0.47	0
GAIF01004594.1	0.21	0	0	0.08	0	0
GAIF01004595.1	0.92	1.52	3.86	1.38	3.51	0.22
GAIF01004596.1	0.01	0.12	0	1.08	2.76	0
GAIF01004600.1	0.54	0.17	0.08	2.33	5.84	0.17
GAIF01004601.1	0	0	0	0	0	171.25
GAIF01004602.1	10.84	18.19	8.06	1.52	0.58	0.05
GAIF01004604.1	1.04	1.01	0.45	0.59	1.08	0.82
GAIF01004605.1	0.04	0.01	0	0	0.08	0
GAIF01004607.1	9.22	6.4	6.48	3.77	3.88	5.39
GAIF01004610.1	0	0	0	0	0.95	0
GAIF01004611.1	0.26	0.29	0	1.98	5.69	0.29
GAIF01004613.1	37.88	53.25	37.36	69.54	32.74	10.92
GAIF01004614.1	0	0	0.13	0	0	0
GAIF01004615.1	0.11	0	0	0	0.14	0
GAIF01004616.1	0.06	0	0	0.38	2.32	0.03
GAIF01004618.1	2.16	0.31	0.16	0.33	0.52	0.32
GAIF01004619.1	18.13	16.93	39.54	39.59	19.51	35.43
GAIF01004620.1	0	0	0	0.01	0	0
GAIF01004622.1	2.79	1.06	1.23	7.46	3.36	1.85
GAIF01004623.1	6.2	6.58	5.76	23.4	19.2	1.84
GAIF01004625.1	0.09	0.31	0	0.33	0.23	0.1
GAIF01004626.1	0.53	0.08	0	0.56	2.03	0.14
GAIF01004628.1	0	0.05	0.05	0.18	0	0
GAIF01004629.1	0	0	0	1.17	1.55	0
GAIF01004630.1	20.16	23.69	16.44	26.9	24.3	4.65
GAIF01004632.1	0.94	0.67	0.55	0.37	0.63	0.4
GAIF01004635.1	5.17	7.29	3.38	3.37	1.96	0.5
GAIF01004636.1	304.79	359.1	430.41	167.82	150.35	319.01
GAIF01004637.1	21.24	38.45	44.04	62.02	32.21	16.29
GAIF01004638.1	1.26	0.24	0.24	1.8	4.18	1.79
GAIF01004639.1	2.87	0.59	0.9	6.6	1.57	0.9

GAIF01004641.1	0.63	0.69	0.26	11.58	43.6	0
GAIF01004642.1	11.64	9.47	14.92	34.72	26.36	19.35
GAIF01004643.1	10.62	10.99	11.33	13.16	13.39	4.39
GAIF01004644.1	2.91	0.61	0.15	3.85	9.11	1.25
GAIF01004645.1	6.09	6.25	10.69	7.46	3.25	12.07
GAIF01004646.1	0.12	0.07	0	0.21	0.44	0
GAIF01004647.1	26.68	30.77	23.48	58.3	37.03	11.67
GAIF01004648.1	0	0.04	0	0	0.08	0
GAIF01004649.1	11.98	13.12	20.17	38.25	28.91	13
GAIF01004650.1	0.27	0	0	1.03	6.72	0
GAIF01004653.1	0	0	0	0	0.38	0
GAIF01004656.1	9.71	9.81	10.33	9.77	4.93	2.93
GAIF01004657.1	0	0	0	0.06	0.03	0
GAIF01004658.1	0.88	0.32	0.32	0	0	0
GAIF01004659.1	0.37	0	0	0.85	2.21	0.14
GAIF01004661.1	5.77	7.4	5.68	7.21	16.9	21.52
GAIF01004662.1	0.09	0.01	0	0.02	0.07	0
GAIF01004664.1	0.1	0	0	0	0	0
GAIF01004665.1	0	0.04	0	0.36	1.62	0.04
GAIF01004666.1	6.23	8.73	4.75	0.88	0.52	0.03
GAIF01004671.1	0	0.02	0	0.18	0.42	0.02
GAIF01004672.1	25.05	34.26	30.71	54.96	32.15	13.27
GAIF01004673.1	1.24	0.73	1.37	0.17	0	0.05
GAIF01004675.1	0.09	0	0	0.93	5.96	0
GAIF01004676.1	9.15	17.85	3.55	0.9	1.52	0.52
GAIF01004680.1	0.35	0	0	0.27	0.28	0
GAIF01004681.1	4.16	3.02	2.16	0.54	0.14	0
GAIF01004682.1	0.06	0.07	0.04	0.01	0.05	0.06
GAIF01004686.1	6.09	2.12	2.68	15.55	34.87	26.85
GAIF01004687.1	0.18	0.06	1.93	4.63	1.41	12.29
GAIF01004689.1	0	0.18	0.74	0	0	0.19
GAIF01004690.1	0	0	0	0.33	1.5	0
GAIF01004691.1	0.53	0.34	0	3.43	4.31	0.35
GAIF01004692.1	0.61	1.33	0	2.76	14.25	0.67
GAIF01004693.1	0	0	0	0	1.57	0
GAIF01004694.1	0	0	0	0	0	0.07
GAIF01004695.1	14.04	31.31	25.72	8.65	15.97	6.92
GAIF01004696.1	3.7	4.68	0.68	5.94	10.73	0
GAIF01004697.1	0	0	0	0	0.25	0
GAIF01004698.1	19.03	13.3	18.64	40.96	31.56	21.36
GAIF01004699.1	74.34	91.84	76.7	119.55	70.45	18.44
GAIF01004700.1	0.3	0.85	0.13	2.22	4.75	0.13
GAIF01004701.1	0	0	0.04	0.68	3.1	0
GAIF01004703.1	1372.85	972.64	311.54	2954.74	3386.33	954.29

GAIF01004704.1	19.72	20.19	12.52	79.47	56.3	14.3
GAIF01004706.1	0.26	0	0	0.24	0.31	0.19
GAIF01004708.1	17.6	15.7	11.8	43.58	37.05	8.49
GAIF01004709.1	68.11	6.04	154.32	0.06	0	18.73
GAIF01004711.1	0	0.28	0.19	0.3	0.61	0
GAIF01004712.1	0.04	0.05	0.2	0.1	0.16	0
GAIF01004713.1	0.88	0.18	0	2.37	13.42	0
GAIF01004714.1	2.54	4.57	2.06	5.9	13.16	0.37
GAIF01004715.1	60.1	31.45	41.78	69.1	33.44	35.13
GAIF01004717.1	0.09	0.25	0.05	0.11	0.22	0
GAIF01004718.1	2.75	0.82	0.27	13.07	30.71	0.55
GAIF01004719.1	0	0	0	0	0.19	0
GAIF01004722.1	3.04	0	0	0	0	0
GAIF01004723.1	14.72	17.18	14.46	39.85	43.76	17.18
GAIF01004724.1	8.34	6.11	4.42	12.95	6.16	5.9
GAIF01004725.1	0.12	0.25	0	0.42	0	0.14
GAIF01004726.1	0	0	0	0	0	0.16
GAIF01004727.1	0	1.65	1.42	0	0	0.46
GAIF01004729.1	0.06	0	0	0.43	1.66	0.04
GAIF01004730.1	0	0.04	0.04	0.31	1.29	0
GAIF01004731.1	0	1.5	0.61	0	0.18	0.11
GAIF01004733.1	0	0.12	0	0.13	0.81	0.25
GAIF01004735.1	0	0.67	0	0.06	2.33	0
GAIF01004736.1	0.24	0	0.54	0.28	0.44	0
GAIF01004737.1	0	0	0	0	1.01	0
GAIF01004740.1	0	0	0	0	0.22	0
GAIF01004741.1	35.79	0	1.26	21.68	9.35	7.37
GAIF01004742.1	1.23	0.53	0	0.47	1.01	0.15
GAIF01004743.1	4.31	4.02	3.94	0	5.61	5.61
GAIF01004745.1	1.47	15.38	21.21	20.64	27.65	3.06
GAIF01004747.1	0	0	0	0	0	0.42
GAIF01004748.1	0	0	0	0	0.06	0
GAIF01004749.1	0.05	0	0	0.2	2.79	0
GAIF01004751.1	6.9	6.61	2.05	3.8	4.62	2.14
GAIF01004752.1	0.38	0	0	1.61	5.6	0.28
GAIF01004753.1	0	0	0.11	2.2	2.02	0
GAIF01004755.1	0.31	0	0	1.23	2.65	0
GAIF01004759.1	29.09	42.7	49.1	81.65	39.13	15.83
GAIF01004760.1	6.4	5.06	1.6	16.21	37.44	3.05
GAIF01004764.1	0.29	0.63	0.35	3.76	9.67	0.12
GAIF01004766.1	0	0	0	0.03	0	0
GAIF01004767.1	5.89	1.29	0.12	15.52	46.72	2.42
GAIF01004768.1	0	0.16	0	0.17	0	0
GAIF01004769.1	0	0	0	0	0.07	0

GAIF01004770.1	3.6	3.73	1.59	12.72	17.24	0.32
GAIF01004771.1	62.73	13.84	27.65	40.81	31.5	174.26
GAIF01004772.1	0	0	0	0	0.02	0
GAIF01004774.1	0	0	0	0.06	0.06	0
GAIF01004775.1	0	0	0.21	0	0	0
GAIF01004777.1	0.7	0.05	0.08	1.07	9.73	0.31
GAIF01004779.1	0.1	0	0	0.06	0.83	0
GAIF01004780.1	0.13	0.31	0.23	0.06	0.03	0
GAIF01004781.1	0	0	0	0.16	0	0
GAIF01004782.1	21.05	10.67	12.78	25.4	36.7	47.07
GAIF01004783.1	8.88	1.57	0	15.36	21.85	5.69
GAIF01004784.1	1.3	0.9	0.67	0.34	0.68	0.01
GAIF01004786.1	0	0	0	0	0.24	0
GAIF01004787.1	0.41	0.17	0.22	13.64	0.25	0.28
GAIF01004788.1	134.82	85.03	49.52	186.96	223.41	22.44
GAIF01004790.1	0	0.34	0.17	0.67	0.34	0
GAIF01004791.1	0.29	0.04	0.04	1.04	5.54	0.55
GAIF01004792.1	25.19	18.85	38.81	50.81	25.57	31.25
GAIF01004793.1	0.05	0	0	0.06	0.25	0.34
GAIF01004794.1	12.47	15.84	20.5	24.61	16.89	10.46
GAIF01004795.1	0.53	0.29	0.37	0.23	0	0.15
GAIF01004797.1	353.46	197.7	44.7	1798.33	1517.47	76.01
GAIF01004798.1	0.12	0.4	0	0.29	0.6	0.27
GAIF01004799.1	0.93	0.08	0	2.15	9.96	0.63
GAIF01004800.1	0	0	0	0.43	0.15	0
GAIF01004801.1	0.12	2.89	1.8	0	0.11	0
GAIF01004802.1	7.62	5.62	8	7.31	10.29	4.4
GAIF01004803.1	0.21	0	0.16	0	3.16	5.26
GAIF01004804.1	0	0	0	0.43	2.9	0
GAIF01004805.1	0.22	0.12	0	2.33	6.05	0.06
GAIF01004806.1	0	0	0	0.18	0.53	0
GAIF01004808.1	21.64	19.85	18.41	20.08	11.25	5.93
GAIF01004809.1	0.11	0	0	0	0	0
GAIF01004810.1	0	0	0.08	0.09	0.09	0
GAIF01004813.1	0.05	0	0	0	0.2	0.06
GAIF01004814.1	12.68	11.81	12.97	12.38	8.03	4.42
GAIF01004815.1	0.25	0	0.48	0	0.31	0
GAIF01004816.1	7.79	13.67	5.72	34.54	52.2	4.02
GAIF01004818.1	2.69	3.1	4.42	1.56	0.2	1.11
GAIF01004819.1	0	0.06	0	0	0	0
GAIF01004821.1	0	0	0	0	0.27	0
GAIF01004822.1	0.09	0	0	0	0	0
GAIF01004823.1	272.15	268.17	215.16	288.01	340.98	108.89
GAIF01004826.1	0	0	0	0.2	1.54	0.09

GAIF01004827.1	0.81	0.48	1.08	0.72	0.83	0.45
GAIF01004828.1	0.19	0.07	0	1.56	7.39	0.14
GAIF01004829.1	0	0	0.33	0	0.19	0
GAIF01004831.1	0	0	0	0.38	0.04	0
GAIF01004833.1	0	0.69	0	0.7	1.18	0
GAIF01004838.1	12.38	18.94	0	38.22	61.7	39.32
GAIF01004839.1	171.74	255.28	334.82	97.51	54.29	98.91
GAIF01004840.1	51.78	29.63	50.43	36.54	53.91	134.38
GAIF01004841.1	0.88	0.93	0	0.58	0.83	0.74
GAIF01004842.1	0	0	0	0.34	1.41	0.16
GAIF01004844.1	21.43	16.66	16.99	52.15	15.92	15.09
GAIF01004845.1	1.66	6.11	6.52	2.2	4.4	0
GAIF01004846.1	0.2	0	0	0.31	0.36	0
GAIF01004847.1	14.63	23.13	14.39	44.13	34.63	3.46
GAIF01004849.1	0.84	0.91	0.31	2.11	2.19	0
GAIF01004851.1	0	0	0	0	0.02	0
GAIF01004852.1	1.09	2.6	1.7	4.54	5.1	1.08
GAIF01004853.1	15.13	0.95	0.96	21.63	47.47	2.89
GAIF01004854.1	3.61	2.73	1.16	0.24	0	0.24
GAIF01004855.1	0	1.5	0.97	0	0	0.65
GAIF01004858.1	1.09	2.2	0	15.15	35.69	0
GAIF01004860.1	0	0	0.32	0	0	0
GAIF01004861.1	0	0.1	0	0.07	0.16	0
GAIF01004863.1	2.24	2.89	3.91	1.45	2.29	2.1
GAIF01004864.1	4.1	1.32	2.9	3.4	5.41	5.07
GAIF01004865.1	38.16	44.32	29.32	54.55	60.45	4.91
GAIF01004866.1	0.39	0.23	0.53	1.33	4.88	0.55
GAIF01004867.1	4	4.85	6.78	11.28	11.64	9.16
GAIF01004868.1	0.25	0.2	0.05	0.19	0.26	0.36
GAIF01004869.1	0	0	0	0	0.14	0.26
GAIF01004873.1	0.06	0.26	0.1	0.11	0	0
GAIF01004874.1	0	0	0	0.35	3.04	0.16
GAIF01004879.1	92.83	36.97	41.5	92.66	55.41	28.74
GAIF01004880.1	1.42	0.26	0.65	10.82	48.67	2.21
GAIF01004881.1	0	0.02	0	0	0.02	0
GAIF01004882.1	488.83	149.82	60.47	525.34	627.31	82.11
GAIF01004883.1	0.09	0.14	0.03	1.24	4.24	0
GAIF01004885.1	0.36	0	0	0	0	0.2
GAIF01004886.1	0.13	0	0.25	0.11	0	0.13
GAIF01004887.1	10.86	10.6	6.69	2.83	3.86	0.24
GAIF01004888.1	0	0	0	0	0.5	0
GAIF01004889.1	0.58	4.39	2.21	0.68	1.27	1.78
GAIF01004890.1	0	0.05	0	0	0.58	0.3
GAIF01004891.1	0.31	0.2	0.2	0.28	0.07	0.07

GAIF01004894.1	65.74	80.01	89.53	63.18	43.6	36.04
GAIF01004897.1	2.8	3.22	0	7.04	20.41	3.37
GAIF01004898.1	0.08	0	0	0	0	0
GAIF01004903.1	10.46	16.58	10.59	31.39	23.77	6.02
GAIF01004904.1	0.36	0	0.79	0.63	0.22	0.53
GAIF01004905.1	0	0.96	0	2.24	0.37	0
GAIF01004906.1	0.66	0.22	0	0.38	0.08	0.22
GAIF01004907.1	1.24	1.68	0.81	2.36	4.16	0.53
GAIF01004910.1	885.19	887.51	682.08	219.43	112.97	81.27
GAIF01004911.1	0.37	0	0	0.68	1.48	0.1
GAIF01004912.1	24.79	30.81	24.69	9.51	12.52	5.65
GAIF01004913.1	0.59	0.07	0	1.23	0.51	0
GAIF01004914.1	2.13	0	0	2.41	6.26	0
GAIF01004915.1	8.85	1.19	2	31.87	61.89	8.6
GAIF01004916.1	0	0	0.06	0	0.12	0.06
GAIF01004917.1	1.62	3.32	3.7	0.26	0.22	0
GAIF01004919.1	0	0	0	1.46	1.36	1.79
GAIF01004920.1	4.6	4.41	0.24	2.04	2.11	0
GAIF01004921.1	4.59	13.18	11.17	0	0	0
GAIF01004922.1	0.07	0	0	0	0	0
GAIF01004923.1	219.56	288.85	242.59	87.21	45.24	53.05
GAIF01004924.1	10.28	9.67	14.06	23.21	20.63	13.62
GAIF01004925.1	130.26	297.31	82.62	8.41	5.92	1.33
GAIF01004926.1	6.21	5.39	1.7	1.06	2.23	2.38
GAIF01004927.1	0.59	0	0	0	0	0
GAIF01004928.1	0.85	0.14	0.11	1.17	0.77	0.76
GAIF01004929.1	11.56	18.34	13.43	31.38	17.84	4.56
GAIF01004930.1	7.35	0	0.23	12.03	2.42	1.68
GAIF01004931.1	29.57	20.63	24.16	18.73	22.91	10.82
GAIF01004932.1	0	0	0	0.13	1.62	0.25
GAIF01004934.1	0	0	0	0	0	0.54
GAIF01004937.1	5.48	5.05	4.85	14.16	10.31	2.56
GAIF01004939.1	0	0	0	0	0.03	0
GAIF01004940.1	0	0.03	0	0.06	0	0
GAIF01004941.1	15.31	5.64	6.85	17.29	24.79	38.15
GAIF01004942.1	0	0	0.05	0	0.17	0
GAIF01004944.1	0	0.03	0.03	0.09	0	0
GAIF01004945.1	0.19	0.06	0	0.73	3.01	0.21
GAIF01004946.1	4.91	1.6	0.81	8.84	24.52	4.33
GAIF01004947.1	15.34	7.54	5.58	3.75	2.56	1.02
GAIF01004948.1	0	0	0	0.14	0.2	0
GAIF01004949.1	0	0	0	0.23	0.84	0
GAIF01004951.1	39.88	5.92	5.57	50.31	67.17	222.88
GAIF01004952.1	1.11	1.84	0.45	20.62	53.04	0.14

GAIF01004953.1	0.18	0.1	0	2.76	10.01	0.3
GAIF01004955.1	0	0	0	0	0.25	0
GAIF01004957.1	28.27	14.33	13.42	52.96	53.92	11.07
GAIF01004958.1	3.51	3.92	3.31	0.68	0.59	0.21
GAIF01004959.1	0	0	0	0	0.27	0
GAIF01004960.1	0.02	0	0	0	0	0
GAIF01004961.1	0	0.38	0	0	0	0
GAIF01004962.1	0.18	0.07	0.07	0.77	4.98	0.07
GAIF01004963.1	3.73	3.13	1.4	0.99	2.82	0
GAIF01004964.1	2.24	3.95	3.26	7.85	14.95	0.8
GAIF01004965.1	30.39	80.12	87.39	64.41	62.8	19.95
GAIF01004966.1	0.21	0.45	0.08	3.33	8.63	0.15
GAIF01004968.1	7.85	10.92	8.57	29.46	16.09	1.93
GAIF01004969.1	20.36	23.85	22.4	32.77	18.64	15.96
GAIF01004970.1	0	0.02	0	0.03	0.05	0
GAIF01004971.1	3.95	2.52	1.22	2.43	2.53	2.21
GAIF01004973.1	14.54	19.47	6.6	11.43	46.38	13.24
GAIF01004975.1	4.15	7.14	4.55	2.72	2.54	0.52
GAIF01004976.1	0	0	0	0	0.06	0
GAIF01004979.1	1.19	1.28	0.42	0	0	0
GAIF01004981.1	0	2.57	1.76	0	0	0.17
GAIF01004982.1	0.02	0.04	0	0.35	0	0
GAIF01004986.1	0.3	0.05	0	0.12	0.06	0.05
GAIF01004987.1	21.64	34.07	24.83	59.67	44.73	7.48
GAIF01004990.1	0	0	0	0	0	0.14
GAIF01004992.1	0	0.17	0.19	0	0	0
GAIF01004994.1	5.7	15.54	23.9	19.71	26.12	50.11
GAIF01004996.1	3.09	2.36	5.62	2.94	2.61	0
GAIF01004997.1	3.37	3.75	0.15	28.66	47.47	0.95
GAIF01004999.1	0	0	0	0	0.1	0
GAIF01005000.1	14.18	17.77	10.26	39.58	31.77	5.02
GAIF01005001.1	0.56	0.2	0.21	1.86	1.81	0.93
GAIF01005004.1	0.68	0.04	0.14	5.66	23.04	0.87
GAIF01005006.1	0	0	0	0	0.18	0
GAIF01005008.1	10.05	5.67	2.5	35.76	48.79	0.3
GAIF01005009.1	0.16	0	0	0	0	0
GAIF01005013.1	0	0	0.26	0	0	0
GAIF01005014.1	0	0	0	0	0.76	0
GAIF01005016.1	1.66	1.2	0	7.37	17.95	0.3
GAIF01005017.1	2.03	2.32	1.12	5.08	7.46	0.34
GAIF01005018.1	0.29	0.17	0.79	2.19	3.76	0.67
GAIF01005019.1	0	0	0.89	0	0	0.43
GAIF01005020.1	1.3	0	0	0.85	1.39	5.77
GAIF01005021.1	20.93	11.69	5.55	51.32	53.76	8.16

GAIF01005022.1	0	0	0	0	0.18	0
GAIF01005023.1	56.72	34.51	56.39	108.43	46.29	27.53
GAIF01005027.1	0.12	0.07	0	0.06	0	0
GAIF01005028.1	1.61	0.25	0.17	5.86	39.47	9.44
GAIF01005031.1	0.11	0.12	0	0.21	2.27	0
GAIF01005032.1	0.7	0.11	0.11	2.54	13.62	0.11
GAIF01005034.1	0.67	0.18	0	4.48	19.16	0.09
GAIF01005035.1	0.15	0	0	0.72	1.99	0
GAIF01005036.1	0	0.14	0	0	0	0
GAIF01005037.1	0.02	0.13	0.58	0.48	0.52	0.29
GAIF01005038.1	0.37	0.1	0	0	0	0.33
GAIF01005039.1	0.27	0.13	0	0.79	3.54	0.08
GAIF01005040.1	65.1	67.51	133.41	185.2	144.4	112.28
GAIF01005043.1	0.41	0.15	0	1.18	13.36	0
GAIF01005046.1	0.35	0.33	0.17	1.95	4.33	0
GAIF01005049.1	2.68	3.34	0	27.68	78	0.34
GAIF01005051.1	0	0.12	0.13	0.13	0.05	0.21
GAIF01005053.1	1.83	2.43	0.82	4.16	3.17	0.37
GAIF01005054.1	3.49	1.02	0.74	8.11	27.94	2.96
GAIF01005055.1	0	0	0.49	0.28	0	0.16
GAIF01005056.1	5	1.41	4.94	1.97	1.08	2.53
GAIF01005058.1	3.44	0	0	4.29	10.82	2.14
GAIF01005059.1	0.03	0	0	0.2	0.34	0.06
GAIF01005060.1	0.03	0	0.03	0.09	0.89	0
GAIF01005062.1	0	0	0	0	0.88	0
GAIF01005063.1	0	0.04	0	0.65	3.79	0.13
GAIF01005064.1	41.29	48.7	37.48	46.55	37.21	27.86
GAIF01005065.1	0	0	0	0	0.71	1.04
GAIF01005066.1	2.67	7.74	6.7	8.26	7.03	1.26
GAIF01005067.1	0	0.05	0	0	0	0
GAIF01005069.1	13.61	3.65	5.61	20.46	5.55	7.13
GAIF01005070.1	12.12	8.63	15.14	31.43	16.18	34.63
GAIF01005071.1	0	0.08	0.65	0	0	0
GAIF01005072.1	0.12	0.13	0.13	0.27	0.42	0.13
GAIF01005073.1	0	0	0.34	0	0	0
GAIF01005074.1	5.11	0	4.01	2.35	1.59	10.49
GAIF01005076.1	0	0	0	0	0.09	0
GAIF01005078.1	0.19	0	0	0	0	0
GAIF01005079.1	0	2.21	5.95	0	0	1.77
GAIF01005080.1	10.34	12.09	5.17	32.92	34.88	0.91
GAIF01005081.1	7.88	8.39	0	9.04	11.93	0.32
GAIF01005082.1	1.1	1.52	1.44	3.76	2.97	0.22
GAIF01005083.1	1.69	0.62	0.45	7.14	6.29	0.46
GAIF01005084.1	0.28	0	0.08	0	0	0.04

GAIF01005085.1	0.11	0	0	0.04	0	0
GAIF01005086.1	27.44	38.13	62.36	26.39	17.49	10.26
GAIF01005089.1	33.99	13.93	1.59	292.01	767.61	77.34
GAIF01005093.1	21.37	15.43	9.76	39.96	17.38	6.28
GAIF01005094.1	48.73	9.07	9.65	35.33	28.83	29.75
GAIF01005095.1	15.07	7.17	7.47	25.55	20.65	12.92
GAIF01005096.1	3.43	0.09	0.4	7.68	23.58	0
GAIF01005097.1	0.31	1.27	0.67	0.48	5.11	0.06
GAIF01005098.1	21.74	22.39	17.89	7.95	3.04	8.21
GAIF01005099.1	3.82	5.48	3.81	4.01	5.12	0.87
GAIF01005101.1	29.25	37.05	25.63	73.84	61.36	15.09
GAIF01005103.1	0	0.06	0.02	0	0.31	0
GAIF01005104.1	4.49	4.54	1.7	13.37	40.06	1.61
GAIF01005105.1	0	0	0.39	0	0.21	0.19
GAIF01005106.1	13.68	13.33	28.75	46.57	36.15	16.92
GAIF01005107.1	0	0	0	0	0.19	0
GAIF01005108.1	725.41	3598.08	1227.23	52.72	181.1	28.71
GAIF01005109.1	3.51	4.87	3.05	2.73	3.53	0.04
GAIF01005110.1	0	0	0	0.13	0	0
GAIF01005111.1	0.11	0	0	0.5	0.73	0
GAIF01005112.1	6.26	4.17	1.96	0.43	0.13	0.12
GAIF01005116.1	0	0	0	0.08	0	40.05
GAIF01005117.1	0.06	0.17	0.04	0.59	2.69	0
GAIF01005118.1	28.36	21.54	20.1	18.68	9.8	8.09
GAIF01005119.1	0	0	0	1.39	1.7	1.64
GAIF01005121.1	0	0	0	0.07	0.22	0
GAIF01005122.1	10.48	5.51	1.77	20.18	41.96	114.84
GAIF01005123.1	13.67	5.95	10.08	6.12	1.88	6.03
GAIF01005125.1	16.69	26	20.8	21.99	9.77	5.22
GAIF01005126.1	0	0.31	0.32	0	0	0
GAIF01005128.1	32.92	45.32	43.99	10.33	5.82	3.61
GAIF01005129.1	38.32	24.89	14.72	58.48	44.12	19.74
GAIF01005130.1	0.07	0	0	0	0	0
GAIF01005131.1	8.83	0	1.52	11.22	7.44	24.24
GAIF01005132.1	34.84	11.91	13.04	20.84	17.92	19.8
GAIF01005133.1	0.18	0.39	0.99	0	0.22	0.19
GAIF01005134.1	0.15	0	0	0.52	1.79	0
GAIF01005135.1	2.09	0.7	0.7	1.28	0.63	0.51
GAIF01005136.1	13.42	9.69	13.3	22.61	26.24	11.6
GAIF01005138.1	18.08	16.51	5.83	17.94	32.49	10.96
GAIF01005139.1	371.33	1175	1137.44	280.35	214.85	317.39
GAIF01005140.1	0.39	2.13	0	4.4	3.93	0.14
GAIF01005141.1	0.44	0	0.16	0	0.28	0
GAIF01005142.1	1.37	0.16	0	1.63	2.93	0.68

GAIF01005143.1	43.63	29.79	18.65	35.74	71.92	14.15
GAIF01005144.1	30.47	23.83	7.28	38.22	31.72	2.46
GAIF01005145.1	8.99	0	14.58	22.13	0	0
GAIF01005146.1	0	0	0	0.13	0	0.11
GAIF01005147.1	111.13	90.83	80.19	50.46	30.29	15.75
GAIF01005149.1	0	0	0	0.04	0.08	0.08
GAIF01005150.1	57.23	63.99	43.48	56.13	49.57	9.88
GAIF01005155.1	0	0.7	1.03	0	0	0.24
GAIF01005156.1	0.16	0	0	0	0.56	0
GAIF01005157.1	15.86	18.87	18.11	8.34	7.67	11.34
GAIF01005158.1	0.17	0	0.05	0	0.45	0.08
GAIF01005159.1	22.12	29.06	48.56	47.41	27.89	40.28
GAIF01005160.1	251.92	248.46	412.95	314.45	198.11	372.16
GAIF01005162.1	6.9	4.22	3.57	11.69	6.01	5.07
GAIF01005163.1	0.47	0.17	0.12	0.06	0.06	0
GAIF01005167.1	3.01	3.51	3.01	6.39	5.3	2.41
GAIF01005168.1	4.46	4.13	3.38	1.63	2.56	0.83
GAIF01005170.1	0.9	0.51	0	0.19	1.77	0.74
GAIF01005172.1	2.19	0	0.06	6.72	20.1	0.52
GAIF01005173.1	10.25	9.23	2.52	55.34	40.47	2.37
GAIF01005174.1	22.87	0.24	5.79	46.2	16.4	29.32
GAIF01005176.1	0.49	0.21	0.21	0	0.12	0
GAIF01005177.1	100.33	109.95	145.26	19.28	101.63	17.75
GAIF01005179.1	0	0	0	0.07	0	0
GAIF01005180.1	0.05	0.08	0	0.54	1.21	0.24
GAIF01005184.1	0.13	0.14	0	0.67	4.96	0.5
GAIF01005187.1	13.4	4.26	7.5	34.06	12.27	52.73
GAIF01005188.1	0	0	0	0.44	0	0
GAIF01005189.1	0.78	0	0.49	0.36	3.61	1.17
GAIF01005192.1	0	0	0	0	0	0.23
GAIF01005193.1	7.65	9.77	8.12	0.68	0.69	1.79
GAIF01005194.1	0	0	0	0.02	0.38	0
GAIF01005196.1	6.56	0.03	0.42	26.87	53.27	0.73
GAIF01005197.1	0	0	0	0	0.09	0
GAIF01005198.1	0.7	0.38	0	4.47	14.66	0.11
GAIF01005200.1	2.16	0	0	2.23	4.04	1.85
GAIF01005201.1	0.33	1.6	0.37	0.93	1.56	0.36
GAIF01005202.1	0	0	0.08	0	0.09	0
GAIF01005203.1	0	0	0	0.08	0.26	0
GAIF01005205.1	0	0	0	0	0.46	0
GAIF01005207.1	3.63	1.37	1.34	3.14	2.4	1.35
GAIF01005208.1	61.44	70.77	38.15	86.61	104.32	10.55
GAIF01005214.1	0.25	0.13	0	0.43	2.38	0.14
GAIF01005215.1	2.26	2.6	0.51	6.48	0	0.17

GAIF01005216.1	8.88	7.32	7.64	27.14	52.96	5.48
GAIF01005218.1	72.9	85.87	31.09	152.62	120.76	12.81
GAIF01005219.1	38.68	47.2	33.7	19.44	22.93	57.67
GAIF01005221.1	7.36	6.1	3.74	28.73	33.02	3.9
GAIF01005223.1	45.42	17.63	20.59	8.6	7.14	40.55
GAIF01005224.1	12.41	9.1	6.53	31.02	34.85	7.57
GAIF01005225.1	0	0	0	0.28	1.56	0
GAIF01005227.1	0.09	0.31	0.28	0	0	0
GAIF01005228.1	0	0	0	0	1.44	0
GAIF01005229.1	0	0	0	0.01	0.4	0
GAIF01005230.1	1.55	0.5	0.1	3.76	13.06	0
GAIF01005231.1	1.03	0.27	0.19	1.47	0.63	0.46
GAIF01005233.1	418.94	313.92	176.14	498.19	343.71	309.48
GAIF01005234.1	0.05	0.05	0.09	0	0	0
GAIF01005235.1	2.54	4.81	0.72	18.09	98.32	0
GAIF01005240.1	3.74	2.74	1.88	3.75	2.06	0.89
GAIF01005241.1	6.03	12.64	4.58	13.26	12.44	2.19
GAIF01005242.1	0	4.25	9.2	0.1	0	61.65
GAIF01005243.1	0.17	0.05	0	0.2	0	0
GAIF01005244.1	0.56	1.05	1.5	2.91	5.23	2.33
GAIF01005245.1	93.78	94.71	176.93	300.91	191.57	229.67
GAIF01005246.1	1.01	0.12	0	2.47	8.21	0.12
GAIF01005249.1	4.97	2.44	0	9.22	15.25	7.38
GAIF01005252.1	0	0	0	0	0.13	0
GAIF01005253.1	0	0	0.41	0	0	0
GAIF01005254.1	0	0.41	0.21	0	0.07	0
GAIF01005255.1	3	4.03	7.21	1	0.56	3.5
GAIF01005257.1	0	0	0	1.25	7.04	0
GAIF01005258.1	0.16	0	0	0	0.19	0.57
GAIF01005259.1	9.26	14.88	14.72	13.63	7.31	2.44
GAIF01005260.1	2.16	0.39	0.1	4.69	31.24	2.77
GAIF01005261.1	1.78	0.14	0.14	7.38	28.44	4.2
GAIF01005262.1	22.34	9.23	4.66	12.06	39.09	9.48
GAIF01005263.1	2.51	1.6	0.37	8.65	23.07	0.15
GAIF01005264.1	0	0	0.13	0.16	0.22	0
GAIF01005266.1	13.59	11.85	16.29	7.22	4.12	7.66
GAIF01005267.1	207.24	201.08	84.17	639.45	651.54	58
GAIF01005268.1	0	0	0	0	0.09	0
GAIF01005269.1	45.5	40.38	25.55	70.89	94.4	29.06
GAIF01005270.1	1.66	2.04	0.37	10.03	15.25	0.28
GAIF01005271.1	4.79	1.56	3.07	1.53	1.8	0
GAIF01005272.1	0	0	7.17	0	0	0
GAIF01005273.1	0	0	0	0	0.07	0
GAIF01005274.1	22.83	12.56	25.07	41.62	21.16	46.18

GAIF01005276.1	0	0	0	0.28	0	0
GAIF01005277.1	0.09	0.47	0	4.38	12.79	0
GAIF01005279.1	0	0	0	0.34	0.17	0
GAIF01005281.1	44.63	43.26	32.84	56.65	75.94	0
GAIF01005282.1	0.37	0	0	0.14	0.08	0.14
GAIF01005283.1	1.72	0	0.26	0.2	0	0
GAIF01005284.1	0.08	0	0	0.07	0.38	0
GAIF01005285.1	4.19	5.23	5.71	3.88	2.99	1.53
GAIF01005287.1	7.56	10.05	5.73	27.88	45.76	4.7
GAIF01005288.1	0	0.16	0	0.98	1.47	0
GAIF01005289.1	1.44	2.64	0.92	0.88	1.19	0
GAIF01005291.1	44.29	24.69	28.84	92.78	43.55	28.54
GAIF01005292.1	0.03	0.03	0.09	0.2	0.69	0.06
GAIF01005293.1	0	0.11	0	0	0.13	0
GAIF01005297.1	4.4	4.75	5.89	3.79	4.47	4.25
GAIF01005298.1	0	0	0.05	0	0	0
GAIF01005300.1	6.05	7.24	4.77	27.2	25.52	1.52
GAIF01005301.1	1.6	2.04	1.09	5.14	8.14	1.66
GAIF01005302.1	0.29	0.06	0	0.84	5.64	1.45
GAIF01005303.1	0.09	0	0	0.74	3.15	0.25
GAIF01005305.1	2.23	0.69	0.87	0.95	1.11	0.35
GAIF01005308.1	0.22	0.24	0	0.13	0	0
GAIF01005310.1	0.28	0	0.08	2.24	8.7	0.23
GAIF01005313.1	35.54	52.28	39.69	75.36	39.63	7.59
GAIF01005314.1	0	0	0	0.1	0.4	0
GAIF01005315.1	40.63	8.08	6.19	18.34	34.47	12.85
GAIF01005317.1	0	0	0	0	0.27	0
GAIF01005318.1	0	0	0	0.13	0	0
GAIF01005319.1	1.97	0.75	0.75	9.57	32.53	2.84
GAIF01005320.1	35.54	27.62	17.01	17.84	18.49	9.16
GAIF01005321.1	25.1	34.26	20.65	29.42	15.05	4.96
GAIF01005322.1	9.13	3.98	7.34	17.64	20.15	60.24
GAIF01005323.1	6.65	6.98	12.37	17.2	9.24	7.29
GAIF01005324.1	1.2	0	0	0.29	0	0
GAIF01005325.1	7.23	4.64	6.07	17.14	28.55	9.66
GAIF01005326.1	69.17	45.73	41.94	12.63	9.76	3.87
GAIF01005327.1	6.98	14.94	5.78	25.76	26.22	3.98
GAIF01005328.1	9.24	6.96	10.03	34.61	21.47	4.86
GAIF01005330.1	0	0	0	0	0.06	0
GAIF01005331.1	0	0	0	0	0.23	0
GAIF01005333.1	1.05	2.72	1.77	4.59	2.42	0.53
GAIF01005334.1	0.27	0.13	0.04	0.04	0.14	0.08
GAIF01005335.1	0	0	0	0	1.2	0
GAIF01005337.1	210.72	221.87	106.08	433.88	322.22	39.62

GAIF01005338.1	0	0	0	0	0.03	0
GAIF01005340.1	58.15	61.61	65.44	96.31	85.45	81.58
GAIF01005341.1	177.61	317.44	326.94	117.41	97.91	76.9
GAIF01005342.1	0	0.71	0	0	0	0.15
GAIF01005343.1	3.23	4.13	0.9	1.81	3.57	8.39
GAIF01005344.1	0.62	0.36	0.01	2.03	3.83	0.04
GAIF01005345.1	0.29	0.3	0	0	0.86	0
GAIF01005346.1	6.64	10.93	9.57	17.27	12.9	0.96
GAIF01005347.1	1.27	0.08	0	2.69	6.58	0.16
GAIF01005348.1	4.03	1.03	0.35	0.78	2.27	0
GAIF01005349.1	0	0	0	0.44	1.61	0
GAIF01005353.1	475.33	282.88	131.93	124.14	240.8	13.47
GAIF01005354.1	0	0	0.08	0	0	0
GAIF01005355.1	1.02	0.1	0.3	2.36	11.8	0.92
GAIF01005356.1	0	0.09	0	0	0	0
GAIF01005357.1	10.06	6.21	3.16	23.52	27.65	22.85
GAIF01005358.1	0	0	0	0	0.58	0
GAIF01005359.1	5.25	7.86	5.09	6.31	2.5	1.04
GAIF01005360.1	0.06	0.54	0.07	0.1	0.9	0.05
GAIF01005361.1	0	0	0	0.1	0.52	0
GAIF01005362.1	0	0	0	0	0	0.07
GAIF01005363.1	0.92	0	0	4.56	21.58	0.29
GAIF01005365.1	637.21	578.67	466.76	519.1	305.31	212.57
GAIF01005366.1	13.11	19.54	8.16	9.29	9.02	2.45
GAIF01005367.1	36.57	44.66	25.23	49.22	74.88	5.76
GAIF01005368.1	18.14	7.16	4.5	20.37	14.43	14.19
GAIF01005370.1	0.34	0.29	0.17	0.79	0	0
GAIF01005373.1	61.99	70.63	67.84	59.09	31.03	24.67
GAIF01005375.1	2.31	3.78	5.89	13.68	26.19	5.1
GAIF01005376.1	0	0.49	0.5	0	0	0
GAIF01005378.1	0	0	0	0	0.09	0
GAIF01005381.1	0	0.15	0	0.03	0	0
GAIF01005382.1	0	0	0	0	0	0.14
GAIF01005383.1	19.96	16.87	29.12	43.15	18.07	26.74
GAIF01005384.1	6.97	1.3	2.04	19.03	10.68	21.44
GAIF01005385.1	0.26	0.14	0.33	0.15	0.33	0.04
GAIF01005387.1	0	1.68	0.8	0	0	0
GAIF01005388.1	0.71	0	0	1.02	1.08	0.2
GAIF01005389.1	0.14	0	0	0.42	2	0
GAIF01005390.1	2.97	6.73	7.23	4.73	3.03	1.76
GAIF01005391.1	0.14	0	0	0.16	0.32	0
GAIF01005393.1	0	0.15	0	0.24	0.85	0.31
GAIF01005395.1	0	0	0	0	0	0.15
GAIF01005396.1	0.12	0.13	0	0	0.15	0

GAIF01005398.1	4.39	0.95	0.78	5.13	11.12	1.08
GAIF01005399.1	0.97	0.13	0.53	0.42	1.31	0.4
GAIF01005400.1	26.77	17.11	23.14	48.19	22.98	59.47
GAIF01005401.1	6.08	0.52	0.5	7.66	6.93	0.96
GAIF01005402.1	1.61	0	0	0	1.94	2.67
GAIF01005403.1	7.23	0.75	0.27	10.56	19.63	0.7
GAIF01005404.1	124.85	113.83	151.39	566.94	304.19	153.31
GAIF01005406.1	0.84	0	0	0.73	1.01	0
GAIF01005407.1	99.2	102.07	220.64	291.67	123.5	151.03
GAIF01005411.1	17.2	24.54	25.48	21.91	16.25	7.83
GAIF01005412.1	33.24	75.26	26.56	15.25	25.22	3.04
GAIF01005413.1	0	0	0	0	0.16	0
GAIF01005414.1	0	0.12	0	0	0	0
GAIF01005415.1	1.43	0.21	0.49	1.78	0.18	0.22
GAIF01005416.1	0	0.04	0.04	0.04	0.08	0
GAIF01005417.1	424.27	371.11	493.72	624.24	311.54	607.01
GAIF01005418.1	7.02	3.02	0.92	12.04	13.73	11.88
GAIF01005419.1	0.21	0.11	0	0.18	0.06	0.06
GAIF01005420.1	11.95	23.39	11.07	12.25	10.73	1.09
GAIF01005421.1	10.87	10.51	11.11	11.97	17.72	2.54
GAIF01005422.1	0.9	2.75	4.66	0.98	2.24	4.8
GAIF01005424.1	0	0	0	0.29	0.15	0.14
GAIF01005426.1	1.03	0.26	0	0.37	1.21	0
GAIF01005428.1	0	0.05	0	0	0.2	0
GAIF01005430.1	0.21	0	0	0	0	0
GAIF01005432.1	0	0	0	0	0.29	0
GAIF01005433.1	17.92	15.07	9.59	46.07	67.05	10.28
GAIF01005434.1	0	0	0.03	0	0	0
GAIF01005436.1	0.23	0.25	0.26	3.52	8.79	0
GAIF01005437.1	0	0	0	0	0.15	0
GAIF01005438.1	65.6	52.15	40.29	133.16	86.29	27.98
GAIF01005440.1	0.14	0	0	0.16	0	0
GAIF01005441.1	75.35	98.23	297.22	55.5	43.19	196.64
GAIF01005442.1	0	0	0	0	2.05	0
GAIF01005443.1	11.82	1.39	1.09	47.05	117.67	177.03
GAIF01005444.1	0	0	0	0	0.11	0.11
GAIF01005445.1	32.79	23.04	8.29	9.01	12.35	1.19
GAIF01005446.1	0	0	0	0.06	0	0
GAIF01005448.1	1.46	0.65	0.19	10.34	11.02	0.28
GAIF01005449.1	16.58	7.95	9.48	31.64	17.98	146.34
GAIF01005450.1	0	0	0	0	0.13	0
GAIF01005453.1	0	0.09	0.25	0	0.02	0.02
GAIF01005454.1	2.54	0.29	0.12	6.65	23.14	0.29
GAIF01005455.1	5.48	4.76	3.95	4.69	5.02	3.15

GAIF01005456.1	0.12	0.91	0.66	0.83	1	0.13
GAIF01005457.1	2.17	1.18	0	11.33	26.27	2.22
GAIF01005458.1	12.86	6.31	1.96	37.71	40.04	2.66
GAIF01005460.1	2.35	2.66	0	16.58	29.96	0.38
GAIF01005462.1	0.25	0.37	0.37	2.65	4.57	0.42
GAIF01005463.1	3.94	4.68	4	6.41	4.74	0.67
GAIF01005464.1	1.88	0.86	0.34	12.59	30.76	0.34
GAIF01005465.1	0	0	0	0	0.66	0
GAIF01005466.1	0	0	0	0	0.04	0
GAIF01005470.1	0.21	0.57	0.23	0.24	0	0.11
GAIF01005471.1	0	0.23	0	0	0	0
GAIF01005473.1	5.01	6.31	4.42	2.6	2.85	0.44
GAIF01005474.1	0	0	0	0.86	6.49	0
GAIF01005475.1	13.48	18.33	9.08	16.06	28.59	48.84
GAIF01005477.1	0	0	0	0	0	0.22
GAIF01005478.1	0	0.13	0.25	0	0	0
GAIF01005479.1	0.62	0	0	0	0	0
GAIF01005480.1	0.14	0.04	0.04	0	0.09	0
GAIF01005482.1	18	18.68	0	24.35	12.61	0
GAIF01005485.1	0.06	0.13	0	3.98	0.59	0.13
GAIF01005487.1	8.75	11.91	2.11	6.19	1.11	0.55
GAIF01005489.1	1.94	1.28	1.34	8.88	8.45	0.11
GAIF01005490.1	1.49	0.58	0.43	1.03	3.91	1.61
GAIF01005491.1	0.2	0.21	0.22	0.92	1.43	0.22
GAIF01005493.1	140.45	18.11	68.39	126.39	10.21	194.32
GAIF01005496.1	27.77	44.56	27.22	28.9	29.5	6.83
GAIF01005497.1	0.06	0	0.13	0	0	0
GAIF01005498.1	78.44	35.03	25.73	0.32	0.73	1.09
GAIF01005500.1	3.87	0.08	0.15	2.7	2.44	6.4
GAIF01005501.1	0.07	0	0	0.17	0.62	0
GAIF01005502.1	0.02	0	0	0.05	0.11	0
GAIF01005503.1	0.07	0	0.08	1.09	3.03	0.4
GAIF01005504.1	0.04	0	0.05	0.05	0	0
GAIF01005505.1	8.22	10.98	6	24.08	32.14	3.05
GAIF01005506.1	0.75	0.08	0.04	1.44	8.67	0.79
GAIF01005508.1	0.38	0	0	1.44	0.69	0.31
GAIF01005510.1	7.23	137.78	271.93	0	0	13.75
GAIF01005511.1	58.35	60.34	35.73	75.09	63.93	15.09
GAIF01005513.1	0.14	0.24	0	1.09	3.3	0
GAIF01005516.1	0	0	0	0	1.17	0
GAIF01005517.1	0.11	0	0.32	0.07	0.2	0
GAIF01005518.1	0	0	0	0	0	0.25
GAIF01005519.1	0.22	0.24	0.24	0.38	0.66	0
GAIF01005520.1	0.3	0.09	0.16	0.06	0.03	0.1

GAIF01005523.1	0	0	0	0	0.02	0
GAIF01005524.1	0.04	0.03	0	0.01	0	0
GAIF01005525.1	2.18	3.15	4.39	1.12	3.39	1.47
GAIF01005526.1	4.03	7.17	7.39	12.33	9.9	2.64
GAIF01005528.1	444.76	201.45	299.82	532.36	401.17	2211.06
GAIF01005529.1	4.57	4.02	4.49	6.47	5.19	4.23
GAIF01005530.1	0.27	0	0.07	1.17	4.19	0.1
GAIF01005531.1	0.03	0	0	0.07	0.38	0
GAIF01005532.1	2.67	2.5	4.34	0.32	0.11	0.2
GAIF01005534.1	12.3	15.52	10.35	91.99	72.28	9.97
GAIF01005536.1	32.44	55.41	96.71	21.01	27.38	69.54
GAIF01005537.1	3.27	2.95	3.72	7.73	6.79	4.11
GAIF01005538.1	0.32	0.24	0.11	0.67	0.37	0.45
GAIF01005540.1	1.6	1.25	2.03	0.38	0.89	0.18
GAIF01005543.1	541.46	430.67	1023.54	0.43	1.43	1.21
GAIF01005544.1	0	0	0.19	0	0	0
GAIF01005546.1	0	0	0	0	0.19	0
GAIF01005547.1	0	0	0	0	0	2.75
GAIF01005550.1	0	0.58	0.5	0	0	0.19
GAIF01005551.1	1.38	1.25	2	1.24	0.46	0.71
GAIF01005552.1	7.15	3.79	4.9	23.14	42.8	31.67
GAIF01005554.1	0.17	0	0	0	0.29	0.05
GAIF01005556.1	0.75	0.46	0.28	2.66	7.26	0.13
GAIF01005557.1	27.05	27.39	47.81	43.28	21.47	40.1
GAIF01005559.1	0.66	0.88	0.3	2.55	4.24	0.15
GAIF01005560.1	0	0	0	0	0.08	0
GAIF01005562.1	15.43	16.79	33.19	60.36	53.09	22.87
GAIF01005563.1	9.23	7.63	8.94	19.02	13.97	5.88
GAIF01005565.1	106.79	76.15	147.9	246.68	224.38	515.4
GAIF01005566.1	0.66	0.16	0	2.84	7.7	0
GAIF01005568.1	0	1.78	1.53	0	0.15	6.02
GAIF01005569.1	37.31	19.61	39.41	31.3	30.12	72.54
GAIF01005570.1	11.08	7.94	7.79	29.43	33.36	5.23
GAIF01005572.1	0	0.17	0	1.41	8.42	0.17
GAIF01005573.1	930.09	722.34	1140.09	848.13	483.56	2500.86
GAIF01005574.1	0.48	0.12	0.08	3.72	16.17	0.08
GAIF01005577.1	1.32	1.98	0.24	2.63	20.57	0.06
GAIF01005578.1	5.09	9.71	21.52	0.11	0.17	1.14
GAIF01005579.1	59.51	50.81	72.59	54.98	44.41	45.79
GAIF01005580.1	0.21	0.04	0.23	0.95	4.77	0.08
GAIF01005581.1	0	0	0	0.55	0.53	0
GAIF01005582.1	0	0	0	0.2	0.21	0
GAIF01005583.1	0	0	0	0	0.42	0
GAIF01005584.1	3.61	1.43	1.43	2.68	0.89	1.5

GAIF01005585.1	0	0	0	0.11	0	0
GAIF01005586.1	0	0.29	0	0.15	0.32	0.29
GAIF01005587.1	0	0.04	0	0.89	1.3	0
GAIF01005589.1	0.43	0.11	0.07	0.04	0.06	0
GAIF01005590.1	0.22	0.12	0	0.72	1.89	0
GAIF01005591.1	1.94	4.34	5.04	6.71	3.7	1.78
GAIF01005592.1	2.25	1.36	1.87	0.47	0	0.08
GAIF01005593.1	0.19	0.08	0.02	1.41	5.21	0.24
GAIF01005594.1	0	0.04	0.08	0.47	0	0.04
GAIF01005595.1	93.43	6.09	6.15	1.7	1.55	7.08
GAIF01005596.1	198.43	276.87	289.62	261.86	189.78	17.86
GAIF01005597.1	45.25	43.45	97.07	141.3	100.77	185.6
GAIF01005598.1	7.22	6.6	5.42	33.23	43.71	3.35
GAIF01005599.1	0	3.43	1.5	0	0.05	0
GAIF01005601.1	22.4	16.16	13.36	44.83	40.25	14.43
GAIF01005602.1	159.52	85.32	64.78	130.06	126.62	64.81
GAIF01005603.1	14.6	10.19	7.72	32.83	32.75	8.28
GAIF01005605.1	32.2	33.41	25.38	71.25	84.68	24.77
GAIF01005606.1	0	0.05	0	0	0	0
GAIF01005607.1	8.11	8.34	10.89	13.55	7.03	5.84
GAIF01005608.1	17.83	22.25	13.33	20.1	18.79	2.32
GAIF01005610.1	8.98	5.92	2.99	4.26	4.62	7.91
GAIF01005611.1	107.63	23.34	5.24	1448.64	767.79	2.26
GAIF01005613.1	0	0	0	0.23	0.48	0
GAIF01005615.1	8.43	7.33	2.19	4.8	1.94	0
GAIF01005616.1	3.9	3.8	1.78	0.24	0.24	0
GAIF01005619.1	11.04	18	12.5	16.06	11.31	4.2
GAIF01005621.1	0	0.05	0	0	0.04	0
GAIF01005622.1	0.35	0	0.1	0.23	1.99	0
GAIF01005623.1	0	0.09	0	0.09	0.63	0.04
GAIF01005625.1	0	0	0	1.4	7.55	0
GAIF01005626.1	102.4	61.76	38.32	169.67	181.22	116.28
GAIF01005627.1	0	0	0.08	0	0	0
GAIF01005629.1	0	0	0	0.31	6.08	0
GAIF01005631.1	8.87	16.33	8.17	52.51	48.67	1.28
GAIF01005632.1	0.44	0.19	0.1	2.97	15.88	0.49
GAIF01005633.1	5.78	14.16	7.99	18.99	20.55	2.45
GAIF01005634.1	0.8	0.87	0	8.53	34.87	0.74
GAIF01005635.1	15.02	15.98	6.41	22.77	55.96	35.08
GAIF01005638.1	20	21.74	30.34	49.79	34.62	9.08
GAIF01005639.1	13.64	21.25	17.76	41.04	21.64	7.31
GAIF01005642.1	4.64	4.02	1.17	11.66	13.56	0.43
GAIF01005643.1	135.86	151.51	146.32	16.92	5.74	4.27
GAIF01005644.1	0	0	0	0	0.16	0

GAIF01005646.1	0	0	0	0	0.29	0
GAIF01005647.1	0.03	0	0	0.08	0.08	0.04
GAIF01005648.1	0	0	0	0.95	3.07	0.18
GAIF01005649.1	0	0	0.07	0	0	0
GAIF01005650.1	2819.37	1126.26	2077.21	1480.8	1333.87	5590.27
GAIF01005651.1	56.92	26.16	18.17	58.15	58.39	63.87
GAIF01005652.1	0.19	0	0	0	2.4	0.05
GAIF01005653.1	0.62	0.41	0	1.3	7.14	0
GAIF01005654.1	0.19	0.04	0	0.26	1.19	1.17
GAIF01005655.1	27.81	21.08	14.43	46.83	49.17	9.07
GAIF01005656.1	0	0	0.32	0	0	0.22
GAIF01005658.1	2712.7	3791.04	4291.24	1994.06	858.9	597.36
GAIF01005660.1	0.11	0.03	0	0	2.21	0
GAIF01005662.1	19.75	29.93	25.44	19.93	16.11	6.02
GAIF01005663.1	333.28	283.21	383.35	874	498.1	610.42
GAIF01005666.1	0	0	0	0	1.21	0
GAIF01005667.1	0.44	0.1	0.34	0.15	0.11	0.39
GAIF01005668.1	3.18	4.32	8.29	8.3	3.34	9.63
GAIF01005669.1	0.07	27.66	108.7	0.08	1.41	84.64
GAIF01005670.1	0	0.2	0.82	0	0	0
GAIF01005673.1	0.08	0	0	0	0	0
GAIF01005675.1	1.53	2.81	1.93	7.36	13.14	4.4
GAIF01005679.1	0.08	0.07	0	0.46	0.29	0.18
GAIF01005680.1	9.44	3.39	1.17	1.5	0.59	0.05
GAIF01005681.1	0	0	0	0	0.74	0
GAIF01005682.1	0.33	0.4	0.49	0.1	0.86	0.49
GAIF01005683.1	1.45	0.26	0.53	0.14	0.14	0
GAIF01005684.1	0.02	0	0	0	0	0
GAIF01005687.1	1.83	5.05	0.05	3.11	4.33	0.01
GAIF01005689.1	7.48	4.18	2.17	40.52	45.88	0.59
GAIF01005691.1	2.47	5.63	1.55	14.07	16.56	0.26
GAIF01005692.1	84.43	92.66	10.24	17.64	32.55	15.89
GAIF01005693.1	5.09	7.21	0	3.14	4.68	0
GAIF01005694.1	0.09	0.1	0.1	0.38	0.22	0
GAIF01005696.1	0	0	0	0	0.21	0
GAIF01005700.1	0	0	0	0.33	0.76	0.62
GAIF01005702.1	68.3	2.94	3.62	8.04	0.71	0.19
GAIF01005703.1	0	0	0	0.08	0	0
GAIF01005704.1	5.51	0	1.05	0.4	0.49	0.77
GAIF01005705.1	9.82	6.85	8.06	23.89	20.7	7.76
GAIF01005706.1	0	0	0	0.14	0.11	0
GAIF01005707.1	0.36	0	0	0	0	0
GAIF01005708.1	0.23	0	0	0.26	0.96	0
GAIF01005709.1	30.09	30.3	14.33	42.13	35.61	13.15

GAIF01005710.1	0.11	0	0	0.34	0.8	126.22
GAIF01005711.1	0	10.67	0	0	0	0
GAIF01005712.1	36.55	28.35	21.44	9.67	13.46	7.48
GAIF01005713.1	0.08	0	0.09	0	0	0
GAIF01005714.1	0	0	0	0.11	0	0
GAIF01005715.1	0.23	0.07	0.07	0.37	1.65	0.14
GAIF01005716.1	2198.73	4025.92	4758.35	243.63	222.65	334.64
GAIF01005717.1	32.2	9.43	11.09	32.09	13.54	6.45
GAIF01005718.1	1.23	3.61	0.41	5.28	3.55	0
GAIF01005719.1	0.18	0.2	0.2	0.14	0.07	0.07
GAIF01005720.1	0	0	0	0	0.09	0
GAIF01005722.1	0	0	0	0	0.04	0
GAIF01005723.1	166.39	58.74	33.72	198.42	295.32	200.17
GAIF01005725.1	107.29	134.93	129.83	73.77	58.72	58.52
GAIF01005727.1	6.42	3.12	3.71	16.08	11.38	3.72
GAIF01005728.1	0	0	0	0	0.08	0
GAIF01005729.1	0.7	0.19	0.06	0.59	4.39	0
GAIF01005733.1	0.07	0.04	0.04	0.17	0.23	0
GAIF01005735.1	5.41	3.36	1.13	11.05	25.07	0.14
GAIF01005737.1	0.11	0	0	0.75	2.6	0.71
GAIF01005739.1	0.89	1.05	0.35	1.78	1.94	0.53
GAIF01005740.1	3.69	3.91	4.66	4.32	2.08	1.15
GAIF01005741.1	0.08	0.04	0.04	0.09	0.05	0.04
GAIF01005742.1	0	0.09	0	0.12	0	0
GAIF01005743.1	1.34	0.36	0.37	1.56	7.67	0.18
GAIF01005744.1	0.28	0.15	0.15	1.6	0.17	0
GAIF01005745.1	0	0	0	0	0	0.16
GAIF01005746.1	0	0	0	0	0.07	0
GAIF01005747.1	16.18	16.18	12.21	98.19	53.06	8.65
GAIF01005749.1	0.04	0.04	0.05	0	0.03	0
GAIF01005750.1	0	0	0	0.06	0.17	0
GAIF01005751.1	227.75	271.43	282.3	171.84	111.8	87.68
GAIF01005753.1	0	0	0	0.76	4.09	0
GAIF01005754.1	0	0	0	0	0.8	0
GAIF01005755.1	0.83	0	0.18	0	0.31	0
GAIF01005756.1	0.46	0.06	0	0	0.27	0
GAIF01005757.1	32.83	18.28	22.99	61.76	52.22	22.96
GAIF01005758.1	3.89	2.14	1.37	10.46	21.94	2.15
GAIF01005760.1	19.39	7.39	58.27	4.75	4.35	9.68
GAIF01005761.1	110.11	137.09	325.52	108.25	116.07	213
GAIF01005763.1	19.71	20.02	15.52	9.78	2.65	1.57
GAIF01005764.1	0	0	0	0.52	0	0
GAIF01005765.1	0.14	0	0.71	0	0	0
GAIF01005766.1	11.39	4.45	5.08	3.26	4.96	2.38

GAIF01005767.1	1.07	0.39	0	1.32	1.37	0
GAIF01005768.1	0.04	0	0	0.02	0.23	0.01
GAIF01005769.1	0.11	0.21	0.24	0	0	0
GAIF01005770.1	0	0.04	0	0.14	0.05	0.14
GAIF01005771.1	17.22	16.52	14.47	35.46	54.57	6.47
GAIF01005772.1	0	0.05	0	0.22	0.45	0.1
GAIF01005773.1	16.21	9.24	2.39	22.21	38.25	1.54
GAIF01005775.1	0	0	0	0	0.18	0
GAIF01005777.1	0	0	0	0.19	0	0
GAIF01005778.1	42.8	26.12	26	33.58	37.72	28.94
GAIF01005780.1	0.49	1.2	0.49	3.48	0.3	0.27
GAIF01005782.1	4.06	1.84	3.47	9.7	3.8	3.36
GAIF01005783.1	0.6	0.43	0	2.79	4.69	0.55
GAIF01005784.1	0.86	1.24	0.47	7.78	0.34	0.47
GAIF01005786.1	0	0	0	0	0.61	0
GAIF01005787.1	7.92	7.33	4.66	17.8	21.3	10.89
GAIF01005788.1	0.03	0.07	0	0	0.08	0
GAIF01005789.1	0.48	0.26	0	1.94	8.99	2.45
GAIF01005790.1	2.01	1.07	1.67	1.04	0.38	0.25
GAIF01005791.1	0.32	0	0	0.73	1.26	0
GAIF01005792.1	0.58	0.72	0.08	0.86	1.71	0
GAIF01005793.1	18.27	0.51	0.48	3.85	1.48	0.19
GAIF01005795.1	0	0.17	0	2.62	7.92	0
GAIF01005798.1	8.65	16.9	5.51	11.89	11.09	1.21
GAIF01005799.1	3.97	4.44	3.17	1.12	0.87	0
GAIF01005800.1	57.64	31.82	16.94	84.08	81.18	8.5
GAIF01005802.1	1.75	1.71	0	10.16	15.79	0
GAIF01005803.1	0	0	0	0	26.21	0
GAIF01005804.1	7.7	1.09	0.88	25.42	72.03	39.23
GAIF01005805.1	0	1.24	1.76	0	0	0.08
GAIF01005806.1	0	0	0	0	0.16	0
GAIF01005808.1	0.18	0.22	0	0.06	0.18	0
GAIF01005809.1	1.13	0	0.34	0.84	0	0.79
GAIF01005810.1	0.07	0	0	0	0	0
GAIF01005816.1	2.82	0.95	0.53	15.46	31.05	0.55
GAIF01005817.1	0	0	0	0	0.38	0.15
GAIF01005818.1	9.26	7.18	5.3	33.74	40.81	4.02
GAIF01005819.1	1.65	0.92	0.21	1.15	2.75	0.07
GAIF01005820.1	3.39	1.33	0.64	4.14	16.3	0.71
GAIF01005821.1	0	0	0	0	0.03	0
GAIF01005822.1	2.78	3.68	1.21	6.49	6.77	0.15
GAIF01005823.1	1.59	1.9	0.92	2.66	3.38	1.21
GAIF01005824.1	0.25	0	0.09	0	0	0.12
GAIF01005828.1	0	0.18	0.09	0.28	1.95	0.27

GAIF01005829.1	99.8	130.05	176.43	245.45	103.86	43.96
GAIF01005830.1	129.1	114.84	25.14	336.91	418.79	154.38
GAIF01005831.1	1.59	3.36	3.66	7.41	5.26	3.93
GAIF01005834.1	0	0	0.17	0	0	0
GAIF01005835.1	0.87	0.24	0.24	3.53	4.18	0.48
GAIF01005836.1	1455.1	1211.4	2572.53	709.37	447.41	1273.78
GAIF01005837.1	119.07	177.51	17.89	111.12	205.59	14.63
GAIF01005838.1	4.42	0	0.84	1.66	3.81	13.59
GAIF01005839.1	2.46	1.89	2	19.34	21.14	1.61
GAIF01005840.1	0.65	0.09	0	1.65	3.53	0.18
GAIF01005843.1	135.69	64.09	74.25	0.18	0.37	0.85
GAIF01005844.1	0.83	1.81	2.57	0.65	0.73	1.42
GAIF01005845.1	1.07	2.18	0	1.73	3.13	0
GAIF01005846.1	0	0.52	0	0	0	0
GAIF01005847.1	3.55	0.79	1.8	1.75	6.51	1.79
GAIF01005848.1	4.54	2.4	2.99	0.9	0.55	0.64
GAIF01005850.1	0	0	0.3	0	0	0
GAIF01005851.1	0.41	0.18	0	1.42	2.54	0.63
GAIF01005852.1	15.81	14.82	11.29	61.54	109.59	33.03
GAIF01005855.1	10.74	19.11	14.5	57.6	38.6	9.03
GAIF01005856.1	0.7	0.63	0.77	0.34	0.49	1.41
GAIF01005857.1	0	0	0	0.28	1.22	0.37
GAIF01005858.1	82.94	103.72	63.69	129.33	74.07	16.32
GAIF01005859.1	0	0.04	0	0	0.12	0
GAIF01005860.1	0	0	0	0	0.27	0
GAIF01005863.1	19.97	11.94	15.4	33.45	17.75	16.54
GAIF01005865.1	3.68	1.94	0.49	17.08	32.04	0.37
GAIF01005867.1	0.04	0.09	0	0.06	0.29	0
GAIF01005868.1	38.58	42.36	31.59	98.86	59.26	15.36
GAIF01005869.1	75.41	38.59	62.28	35.58	45.85	180.21
GAIF01005871.1	57.53	62.31	69.24	106.87	86.19	49.52
GAIF01005872.1	0.18	1.06	0.1	3.39	7.98	0
GAIF01005873.1	0.27	0.14	0.1	0.46	1.33	0
GAIF01005874.1	2.13	3.6	1.21	2.75	3.42	0
GAIF01005875.1	28.51	21.57	26.84	58.65	33.71	23.53
GAIF01005877.1	0	0	0	0.02	0	0.01
GAIF01005878.1	6.53	2.92	0.6	9.28	13.06	0.12
GAIF01005879.1	5.6	1.21	0.46	4.69	8.8	3.38
GAIF01005880.1	1037.82	1186.07	1829.6	548.62	520.33	1741.72
GAIF01005881.1	14	14.35	5.29	51.74	110.44	16.36
GAIF01005883.1	0	5.52	1.06	0	0.95	0
GAIF01005884.1	0.09	0	0	0	0.3	0
GAIF01005885.1	12.21	7.3	9.43	19.81	31.96	6.97
GAIF01005887.1	0	0	0	0.32	0.4	0.16

GAIF01005888.1	9.05	12.94	15.62	42.67	30.22	10.55
GAIF01005891.1	6.72	2.73	0.54	4.7	8.71	3.11
GAIF01005892.1	0.24	0.19	0	0.2	0.87	0
GAIF01005893.1	2.46	1.62	2.68	2.32	1.55	3.33
GAIF01005894.1	0	0	0.11	0	0	0
GAIF01005895.1	100.02	83.43	84.4	33.93	17.5	16.96
GAIF01005896.1	24.21	3.24	1.39	60.55	279.99	29.78
GAIF01005897.1	17.3	21.83	27.28	53.06	52.91	10.88
GAIF01005898.1	0	0	0	0	0	0.07
GAIF01005899.1	32.18	23.83	21.87	62.52	67.82	49.54
GAIF01005900.1	12.44	12.38	11.81	28.02	16.48	3.95
GAIF01005901.1	0	0.03	0	0.1	0.18	0
GAIF01005904.1	9.47	10.75	10.39	33.4	18.45	4.57
GAIF01005906.1	0	0	0	0	1.41	0.42
GAIF01005907.1	23.02	33.23	8.14	36.44	31.55	2.35
GAIF01005909.1	1900.91	5973.33	5687.4	1959.98	1445.61	821.91
GAIF01005910.1	36.39	13.89	7.79	0	0.05	0.13
GAIF01005911.1	0.06	0.03	0.03	1.14	4.53	0.14
GAIF01005912.1	20.39	15.05	32.68	62.61	82.63	55.18
GAIF01005913.1	0	0	0.1	0	0	0.1
GAIF01005914.1	1.58	2.12	2.87	2.85	1.72	0.82
GAIF01005916.1	1.18	0	0	3.33	6.47	0.32
GAIF01005917.1	50.88	34.48	57.01	43.28	23.89	38.66
GAIF01005918.1	11.77	10.13	8.62	23.06	23.4	3.06
GAIF01005919.1	0.73	0.53	0.36	2.21	7.7	0.8
GAIF01005920.1	0	0	0	0	0.14	0
GAIF01005921.1	0	0	0	0	0	0.05
GAIF01005922.1	0	0	0	0	0.06	0
GAIF01005924.1	0.19	0	0.11	0.22	2.1	0
GAIF01005925.1	0.09	0.03	0	0.8	5.43	0.04
GAIF01005926.1	0	0	0	0	0.33	0
GAIF01005927.1	0	0	0.02	0	0	0
GAIF01005928.1	1.34	2.24	1.43	0.89	0.63	0.37
GAIF01005930.1	0	0	0	0	0.01	0
GAIF01005931.1	1.89	1.83	1.48	7.99	8.76	0.37
GAIF01005933.1	0.36	1.57	1.19	0.21	0	0
GAIF01005936.1	3.18	4.03	0	14.82	8.01	0
GAIF01005937.1	20.06	31.22	29.57	34.35	22.37	9.18
GAIF01005938.1	1.15	3.05	3.62	3.83	3.17	0.64
GAIF01005939.1	0.16	0	0	0	0	0
GAIF01005940.1	500.14	186.02	278.37	100.31	51.31	104.9
GAIF01005941.1	0	0	0	0	0.19	0
GAIF01005942.1	0.11	0	0	0	0	0
GAIF01005943.1	121.88	207.04	64.61	184.87	181.23	8.75

GAIF01005944.1	7.04	12.63	14.23	15.17	8.6	7.63
GAIF01005946.1	57.2	0.62	27	144.86	82.95	104.18
GAIF01005947.1	3.84	4.11	2.14	22.26	31.63	2.34
GAIF01005948.1	20.25	13.7	27.24	58.74	46.9	22.09
GAIF01005950.1	1.9	2.69	1.46	4.05	5.57	1.19
GAIF01005951.1	0	0	0	0	0	0.12
GAIF01005952.1	0	0	0	0	25.13	0
GAIF01005955.1	1.72	0.07	0.27	11.42	77.24	5.22
GAIF01005956.1	0.21	0	0	0.12	0	0
GAIF01005958.1	15.91	9.65	9.75	47.11	55.07	22.55
GAIF01005960.1	0.7	0.69	0.41	0.53	0.33	0.6
GAIF01005961.1	17.43	20.31	20.64	13.77	10.68	8
GAIF01005962.1	1.39	0.15	0.06	7.37	32.23	2.09
GAIF01005963.1	0	0.09	0	0	0.05	0
GAIF01005964.1	0	0	0	0.13	0.77	0
GAIF01005965.1	1.69	0.61	0	7.18	20.29	0
GAIF01005966.1	10.17	15.16	5.1	38.89	62.55	2.78
GAIF01005967.1	0.44	0.16	0.32	0	0	0
GAIF01005968.1	0	0	0	0.47	4.41	0
GAIF01005969.1	9.3	10.16	7.23	25.29	31.94	1.65
GAIF01005970.1	10.67	14.18	17.68	25.61	11.97	8.57
GAIF01005971.1	29.82	49.36	22.2	77.65	70.49	5.16
GAIF01005972.1	14.69	5.11	5.65	8.57	2.6	3.78
GAIF01005973.1	0	0	0	0	0.02	0
GAIF01005974.1	0.1	0.06	0.11	0	0.37	0.06
GAIF01005977.1	0	0	0.11	0.36	0	0.19
GAIF01005978.1	0	0	0	0.07	0.38	0
GAIF01005979.1	56.4	49.16	39.9	101.14	48.38	21.43
GAIF01005980.1	0.02	0.04	0.09	0.3	1.71	0.06
GAIF01005981.1	31.36	10.69	13.39	35.22	23.35	27.17
GAIF01005982.1	0	0	0	0.06	0.06	0
GAIF01005983.1	0	0.33	0.18	0.08	0.08	0
GAIF01005984.1	0.15	0.33	0	0.25	1.12	0.06
GAIF01005985.1	1.04	0.28	0.38	3.92	22.5	1.72
GAIF01005986.1	7.66	14.47	8.78	70.98	103.13	2.68
GAIF01005987.1	0.24	0	0.07	0.34	0.91	0.53
GAIF01005988.1	0	0	0	0	0.42	0
GAIF01005989.1	0.63	1.03	0.69	2.94	7.8	0
GAIF01005990.1	0.37	0.52	0.4	0	0.81	0.32
GAIF01005991.1	0	0	0	0.13	0.15	0
GAIF01005992.1	9.05	17.18	16.09	15.83	6.13	3.37
GAIF01005993.1	2.6	3.72	0	8.22	22.91	1
GAIF01005994.1	52.2	43.68	36.68	195.6	95.83	64.26
GAIF01005995.1	0	0	0	0.07	0.07	0

GAIF01005996.1	70.78	93.73	59.93	65.36	92.55	15.93
GAIF01005997.1	0.04	0.07	0	0.13	0.2	0.04
GAIF01005998.1	0	0	0	0.84	5.29	0.23
GAIF01005999.1	0	0.18	0	0	0	0
GAIF01006000.1	15.48	7.86	9.01	35.99	30.42	35.55
GAIF01006002.1	286.05	100.02	72.27	302.41	323.22	58.67
GAIF01006003.1	17.19	33.57	55.98	54.42	21.94	19.85
GAIF01006004.1	0.18	0	0	0.75	2.22	0
GAIF01006005.1	18.25	15.99	13.84	62.32	46.73	6.94
GAIF01006006.1	1.09	0.89	0	0.95	1.97	1.66
GAIF01006007.1	0.12	0.71	0.59	0.92	0.16	0
GAIF01006008.1	42.61	59.06	77.13	110.6	46.55	37.82
GAIF01006010.1	0	0	0	0	0.15	0
GAIF01006011.1	5.12	10.04	12.89	12.81	13.16	4.09
GAIF01006012.1	0.11	0.12	0	0.12	0.26	0.24
GAIF01006013.1	0.09	0.1	0.21	0.66	0.8	0.21
GAIF01006015.1	0	0	0.03	0	0	0
GAIF01006016.1	0.09	0.39	0.17	0	0.48	0
GAIF01006017.1	0.09	0	0	0.67	6.18	0.1
GAIF01006018.1	2.49	3.09	0.22	30.88	49.65	1.48
GAIF01006019.1	0.1	0.11	0	0	0	0
GAIF01006020.1	0.02	0	0	0	0	0
GAIF01006021.1	21.44	10.03	6.33	27.74	62.66	14.14
GAIF01006024.1	3.38	10.2	2.61	0.87	1.65	0.14
GAIF01006025.1	0.11	0	0	0.13	0	0.12
GAIF01006026.1	0.07	0	0	0.23	0.5	0
GAIF01006028.1	0.22	0	0.25	5.09	28.12	0.37
GAIF01006029.1	0.1	0.11	0	0.7	0.49	0
GAIF01006030.1	0.28	0	0	1.9	6.91	0.2
GAIF01006031.1	0	0	0	0	0	0.03
GAIF01006032.1	0.48	1.3	0.26	2.23	0.29	0.18
GAIF01006033.1	1.65	0.9	0.34	8.22	19.21	0.97
GAIF01006034.1	54.4	67.54	102.54	77.37	66.29	70.81
GAIF01006035.1	0	0.07	0	0	0	0
GAIF01006037.1	0.58	0	0.32	1.01	9.25	0.32
GAIF01006038.1	27.25	15.08	22.13	36.83	29.53	61.71
GAIF01006039.1	4.71	2.49	3.35	6.25	7.67	10.23
GAIF01006041.1	0.19	0.21	0.41	0.22	0.45	0.21
GAIF01006043.1	0.06	0	0.03	0.1	0.17	0
GAIF01006044.1	17.22	28.42	13.87	7.3	26.26	26.32
GAIF01006045.1	1.3	3.61	2.06	4.02	5.21	0.08
GAIF01006046.1	0	0.09	0.09	0	0	0
GAIF01006047.1	0	0	0	0.13	0	0
GAIF01006049.1	128.96	55.01	94.73	271.05	125.36	347.91

GAIF01006050.1	130.84	104.83	78.75	70.71	42.31	25.34
GAIF01006051.1	0	0	0	0.07	0.55	0.03
GAIF01006052.1	18.34	27.07	30.49	41.52	39.2	11.5
GAIF01006054.1	0.27	0.3	0.1	1.47	3.27	0.3
GAIF01006056.1	12.86	26.5	12.39	37.21	28.87	4.11
GAIF01006058.1	0.07	0	0.07	0.53	3.56	0
GAIF01006059.1	0.29	0	0	0.32	2.81	1.91
GAIF01006061.1	0	0.04	0	0.04	0.22	0
GAIF01006063.1	236.06	233.68	171.89	137.15	96	58.38
GAIF01006064.1	511.54	297.71	276.84	240.23	166.36	308.87
GAIF01006065.1	0.14	0.05	0.17	0.05	0.11	0.1
GAIF01006066.1	16.35	15.05	13.67	79.92	66.36	11.94
GAIF01006067.1	14.84	16.34	16.81	2.97	1.6	0.6
GAIF01006068.1	0	0	0	0.11	0	0
GAIF01006069.1	0	0	0	0.06	0.19	0
GAIF01006070.1	0	0	0.05	0.42	0.84	0.21
GAIF01006071.1	0.47	0.21	0	3.07	7.39	4.59
GAIF01006072.1	0.79	0	0	0.27	0.9	0.05
GAIF01006074.1	11.49	12.74	7.03	11.12	7.98	1.3
GAIF01006076.1	0.12	0.26	0.39	0.28	0	0.13
GAIF01006078.1	0	0	0	0.05	0.09	0
GAIF01006080.1	44.49	27.56	21.54	49.98	57.88	120.66
GAIF01006081.1	0.06	0	0.12	0	0	0.06
GAIF01006082.1	2.39	1.32	1.74	1.3	1.46	1.13
GAIF01006084.1	0.54	0.51	0.57	0.44	0.63	0.34
GAIF01006085.1	584.83	590.91	325.38	402.31	358.72	69.46
GAIF01006087.1	0.09	0.05	0.1	0.46	2.15	0
GAIF01006088.1	0.4	0.32	0.65	0	0	0
GAIF01006089.1	0	0	0	0	0.07	0
GAIF01006090.1	34.42	0.68	0.21	4.51	0.45	0.9
GAIF01006091.1	0.04	0.16	0.08	1.26	4.98	0.36
GAIF01006092.1	39.81	24.42	43.05	8.69	8.28	22.28
GAIF01006093.1	0.21	0	0	0.61	2.66	0.11
GAIF01006094.1	66.46	22.61	20.44	32.69	45.57	25.1
GAIF01006095.1	0	0.08	0	0.01	0.44	0.07
GAIF01006100.1	27.67	54.44	23.22	90.6	80.91	5.07
GAIF01006101.1	4.87	19.36	9.02	4.64	3.93	0.08
GAIF01006103.1	0.4	0.1	0.02	0.1	1.89	1.25
GAIF01006104.1	1.33	0	0.06	0.06	0.06	0
GAIF01006105.1	0	0	0	0.01	0	0
GAIF01006106.1	0	0	0	0	0.64	0
GAIF01006107.1	39.72	48.87	76.38	127.03	60.13	37.56
GAIF01006108.1	2.53	0.8	1.41	0	0	0
GAIF01006110.1	0	1.53	1.16	0.14	0	0.26

GAIF01006113.1	0.15	0	0	0	0	0
GAIF01006114.1	0	0.05	0	0.03	0.14	0
GAIF01006115.1	0	0	0	1.82	0	0
GAIF01006116.1	10.06	7.41	4.14	18.14	34.37	16.48
GAIF01006118.1	0.07	0	0	0.91	0.08	0.08
GAIF01006119.1	0	0	0.43	0.12	0.07	0
GAIF01006120.1	0.13	0	0.07	0.07	1.06	0.07
GAIF01006121.1	0.85	0.79	0	3.22	8.96	0.2
GAIF01006122.1	0.29	0	0.63	0	0.17	0
GAIF01006123.1	0	0	0	0	0	0.04
GAIF01006124.1	0.33	0	0.15	0.85	1.76	0.66
GAIF01006125.1	25.03	31.88	37.81	94.51	95.46	21.07
GAIF01006126.1	19.77	22.49	27.95	34.43	30.07	12.4
GAIF01006128.1	99.79	180.4	247.56	561.85	347.81	87.89
GAIF01006129.1	0	0	0	0	0.19	0
GAIF01006133.1	83.64	44.99	74.6	107.11	42.23	87.68
GAIF01006135.1	0	0.07	0.04	2.63	9.01	1.36
GAIF01006136.1	3.33	10.32	9.53	10.66	24.89	2.89
GAIF01006139.1	12.69	14.93	9.97	59.79	108.56	25.15
GAIF01006141.1	0.06	0	0.06	0	0.14	0.07
GAIF01006142.1	4.44	0	191.75	0	0	0
GAIF01006143.1	0	0	0.01	0	0	0
GAIF01006145.1	0	0	0	0	0.55	0.07
GAIF01006146.1	0.13	0.14	0.14	0	0	0
GAIF01006147.1	0	0	0	1	0.16	0
GAIF01006148.1	36.37	31.6	31.26	46.36	66.7	10.34
GAIF01006149.1	0	0	0	0	0.13	0
GAIF01006151.1	0	0	0	0	0.07	0
GAIF01006154.1	0	0	0	0	0.18	0
GAIF01006155.1	615.16	395.11	197.42	590.58	1121.08	2282.41
GAIF01006156.1	0.06	0	0	0	0	0
GAIF01006157.1	6	7.25	1	12.74	13.99	0.66
GAIF01006158.1	3.15	4.15	0	0	0	0
GAIF01006160.1	0.21	0.23	0	0.25	0.51	0
GAIF01006162.1	0.37	0	0	0.52	3.12	0
GAIF01006163.1	0	0.12	0	0.23	0.89	0.07
GAIF01006164.1	0	0	0	0.41	0.57	0
GAIF01006165.1	2.14	0.95	0.43	1.49	1.76	0.43
GAIF01006166.1	0.3	0.18	0.24	0.15	0.1	0.03
GAIF01006168.1	0	0	0	0	1.7	0
GAIF01006169.1	0.15	0.81	2.1	0.77	0.48	0
GAIF01006171.1	0.12	0	0	0	0.73	0
GAIF01006172.1	10.89	9.69	9.38	3.86	3.04	3.74
GAIF01006174.1	7.47	5.81	4.76	14.4	31.91	5.26

GAIF01006175.1	0.05	0.05	0	0	0	0.05
GAIF01006176.1	25.76	25.07	24.38	10.9	10.27	5.96
GAIF01006177.1	1.91	1.31	2	3.04	8.4	2.08
GAIF01006178.1	14.06	6.46	12.71	9.34	4.52	6.46
GAIF01006179.1	0.61	0.21	0.48	0.3	0.09	0.28
GAIF01006180.1	0	0.05	0	0.11	0.87	0
GAIF01006182.1	27.16	19.38	9.56	66.14	78.74	5.3
GAIF01006183.1	0.83	1.75	0.49	1.23	0	0.1
GAIF01006184.1	0	0	0	0.2	0	0
GAIF01006185.1	15.9	13.77	11.64	5.7	4.84	1.08
GAIF01006186.1	8.41	3.6	1.76	18.59	9.63	4.72
GAIF01006187.1	0	0	0	0.11	0.47	0.11
GAIF01006189.1	14.69	13.34	30.11	26.42	12.23	17.37
GAIF01006190.1	0	0	0	0	0.03	0
GAIF01006191.1	0.1	0	0	0.37	1.68	0
GAIF01006194.1	5.57	3.81	4.95	11.26	8.63	7.72
GAIF01006195.1	0.34	0.37	0.56	1.58	6.94	0.14
GAIF01006199.1	0	0	0	0.03	0.84	0
GAIF01006200.1	0.81	1.22	0.81	0.4	0.5	0.06
GAIF01006201.1	19.06	24.79	14.93	55.12	54.15	5.68
GAIF01006202.1	35.32	35.09	31.99	86.43	42.92	15.33
GAIF01006204.1	0	0.07	0	0.16	0.57	0
GAIF01006205.1	132.85	114	113.72	236.6	193.71	78.46
GAIF01006207.1	0	0.84	0	0	0	0
GAIF01006210.1	0	0	0	0	0.34	0
GAIF01006211.1	0	2.61	0	1.24	0.79	0
GAIF01006214.1	1.31	0.63	3.52	2.78	1.66	1.07
GAIF01006215.1	0	0	0	0.11	0.33	0
GAIF01006216.1	0.01	0.01	0.01	0.02	0	0
GAIF01006217.1	0	0.07	0	0	0.16	0
GAIF01006219.1	0	0	0	0.12	0.24	0
GAIF01006221.1	0	0	0	0	0.03	0
GAIF01006226.1	22.38	21.2	13.81	117.26	130.77	5.76
GAIF01006227.1	0	0	0	0.09	0.11	0
GAIF01006228.1	32.61	16.99	5.88	8.47	5.78	2.07
GAIF01006229.1	12.87	3.9	2.19	8.66	9.78	7.17
GAIF01006230.1	0	0	0	0	0	0.07
GAIF01006231.1	3.54	1.17	2.24	7.89	17.36	61.38
GAIF01006234.1	0	0	0	0.15	0	0
GAIF01006238.1	0.23	0.25	0	3.53	6.37	0.25
GAIF01006239.1	3.46	0.69	0.2	13.22	29.04	0.4
GAIF01006240.1	9.67	10.28	19.95	15.94	21.54	3.38
GAIF01006241.1	2.2	1.3	1.53	0.67	2.33	0.44
GAIF01006243.1	0.59	0.11	0.22	0.11	0.36	0

GAIF01006244.1	0	0.15	0	0.79	1.35	0.56
GAIF01006245.1	0.14	0.15	0	0.16	0.65	0
GAIF01006246.1	2.45	3.68	0.32	7.61	20.29	0.87
GAIF01006247.1	0	0.31	0	0.67	2.78	0
GAIF01006248.1	0	0	0.15	0.32	0.5	0
GAIF01006249.1	13.28	101.47	7.25	3.39	33.1	2.1
GAIF01006250.1	0.89	0.79	1.35	4.91	11.12	0.43
GAIF01006253.1	0	0	0	0.24	0.34	0
GAIF01006254.1	0	0	4.27	4.51	7.01	10.7
GAIF01006255.1	0.45	0	0	0	0.27	0
GAIF01006256.1	3.14	1.83	3.33	3.65	0.81	5.56
GAIF01006257.1	0	0.03	0	0.04	0	0
GAIF01006260.1	30.93	16.94	2.04	90.97	231.88	4.61
GAIF01006261.1	3.98	2.42	0.87	7.76	17.04	2.11
GAIF01006262.1	0	0	0	0	0.18	0
GAIF01006263.1	0.15	0.16	0.15	0.23	1.38	0.15
GAIF01006265.1	0	0	0	0	0	0.15
GAIF01006266.1	0	0	0	0.07	0.3	0
GAIF01006269.1	17.88	6.67	7.26	30.39	36.02	33.82
GAIF01006271.1	1	1.44	1.22	0.28	1.55	0.21
GAIF01006272.1	13.03	8.7	16.45	25.49	11.98	15.59
GAIF01006274.1	1.07	0.95	0.22	9.35	18.28	0.66
GAIF01006275.1	0.07	0	0.16	0	0	0
GAIF01006276.1	0	0	0	0	0.21	0
GAIF01006277.1	0	0.17	0.11	0.42	0.37	0.06
GAIF01006278.1	40	44.8	43.54	66.35	38.61	31.22
GAIF01006279.1	6	14.32	9.32	9.17	14.95	11.93
GAIF01006280.1	0	0	0	0	0	0.12
GAIF01006281.1	0	0.8	0.06	0	0	0
GAIF01006282.1	3.71	7.53	1.65	9.28	8.67	0.39
GAIF01006283.1	0.44	0	0.24	24.65	8.87	1.7
GAIF01006284.1	0	0	0	0	0.07	0
GAIF01006285.1	0.81	0.08	0.08	0.68	1.15	0.81
GAIF01006286.1	0	0	0	0.02	0.05	0
GAIF01006287.1	0.07	0.04	0	0	0	0
GAIF01006288.1	0	0.3	0	0	0	0
GAIF01006289.1	0.07	0	0.15	0.7	4.11	0.07
GAIF01006290.1	0.31	0	0	0.37	0	0.02
GAIF01006293.1	0	0	0	0	0.74	2.13
GAIF01006294.1	2.12	1.65	0.88	1.95	4.52	1.15
GAIF01006295.1	8.04	4.98	9.4	6.13	3.92	5.39
GAIF01006296.1	0	0.2	0	0.21	0.56	0
GAIF01006297.1	0.25	0.41	0	0.59	3.06	0.56
GAIF01006298.1	60.08	32.08	2.56	10.24	10.07	0.35

GAIF01006299.1	6.65	7.81	5.19	5.28	3.37	4.25
GAIF01006301.1	0	0	0	0.05	0	0
GAIF01006302.1	0	0	0	0.75	0	0.11
GAIF01006303.1	0.6	0.52	0.13	2.79	17.2	4.11
GAIF01006304.1	0	0	0	0.07	0.04	0
GAIF01006307.1	0	0	0	0.48	0.95	0
GAIF01006309.1	30.51	38.3	50.5	46.21	31.16	23.95
GAIF01006310.1	0	0.12	0.24	0	0.13	0
GAIF01006311.1	0.54	0.67	1.36	0.58	0.02	1.95
GAIF01006313.1	1.39	3.74	3.53	0.51	0.62	0.24
GAIF01006314.1	3.12	2.91	2.5	3.05	16.29	4.01
GAIF01006315.1	312.88	366.48	922.39	68.05	74.35	305.8
GAIF01006316.1	0.05	0.54	0.22	0.22	0.48	0.15
GAIF01006317.1	65.05	0.92	1.61	0.98	0.07	0
GAIF01006318.1	39.02	21.78	8.28	71.15	107.04	4.17
GAIF01006320.1	28.79	15	23.7	18.67	9.16	7.74
GAIF01006321.1	0	0	0	0.6	3.86	0.13
GAIF01006325.1	11.17	16.52	10.43	34.4	39.57	11.26
GAIF01006326.1	0	0	0	0	0.24	0
GAIF01006328.1	1.9	6.47	0	0	0	0
GAIF01006330.1	6.93	7.43	2.83	12.42	28.11	4.91
GAIF01006332.1	0	0	0	0	0	0.36
GAIF01006333.1	0	0	0	0	0.37	0
GAIF01006334.1	0.28	0.06	0	1.81	8.32	2.03
GAIF01006335.1	0	0	0	0	0.15	0
GAIF01006337.1	5.51	7.75	8.96	25.35	21.26	5.85
GAIF01006338.1	0	0.19	0.28	0.17	0.24	0.06
GAIF01006340.1	19.16	18.52	18.32	26.47	18.31	11.61
GAIF01006341.1	0.25	0	0	2.04	2.32	1.02
GAIF01006342.1	22.35	9.34	8.46	37.71	39.08	10.75
GAIF01006343.1	0	0	0	0.85	2.4	0
GAIF01006345.1	0	0	0.06	0	0.07	0
GAIF01006346.1	0	0	0	0	0.09	0
GAIF01006347.1	5.36	6.2	3.98	24.94	17.32	3.36
GAIF01006351.1	0	0.04	0	0.13	0.36	0
GAIF01006352.1	0.17	0	0	0.17	0.35	0.37
GAIF01006353.1	1.31	2.42	3.1	0.45	0.21	0.12
GAIF01006354.1	1.86	6.46	3.2	7.97	4.2	1.03
GAIF01006355.1	0.6	0.52	1.12	5.67	1.66	0.89
GAIF01006358.1	17.05	9.45	23.21	32.56	13.86	56.39
GAIF01006359.1	0.58	2.34	2.95	0.91	0.96	0.33
GAIF01006360.1	9.26	7.16	4.5	1.24	0.21	0.2
GAIF01006361.1	13.59	22.15	4.88	2.97	13.94	0
GAIF01006362.1	1.02	0.44	0.89	0.71	0.73	0.45

GAIF01006363.1	284.36	56.37	152.54	209.81	58.36	502.27
GAIF01006364.1	1.11	0.31	0	0.34	0	0
GAIF01006365.1	16.05	15.92	10.12	43.06	30.53	5.5
GAIF01006366.1	0	0.15	0	0.47	2.45	0.15
GAIF01006367.1	10.83	1.57	1.75	19.84	3.67	0.89
GAIF01006368.1	5.5	2.66	0.61	0.64	0	0.71
GAIF01006369.1	2.23	1.89	0.33	1.94	1.53	0.14
GAIF01006370.1	0.92	0	0	0.65	1.45	0.53
GAIF01006372.1	2.49	3.81	0.25	7.31	16.48	0.74
GAIF01006374.1	8.25	5.76	9.31	6.76	12.33	45.53
GAIF01006378.1	2.06	1.74	0.92	3.56	13.3	1.48
GAIF01006379.1	0	0	0.16	0	0	0.16
GAIF01006380.1	5.69	6.03	4.32	31.91	35.29	3.97
GAIF01006382.1	174.73	209.39	202.01	1469.77	750.71	367.71
GAIF01006383.1	33.99	49.18	41.14	49.51	44	15.67
GAIF01006384.1	0	0	0.69	0	0.19	0
GAIF01006385.1	1.32	0.92	0.52	0.52	0.17	0
GAIF01006387.1	26.57	19.4	10.02	6.56	10.49	7.48
GAIF01006388.1	0	0	0	0	0.09	0
GAIF01006389.1	0	0	0	0	0.44	0.36
GAIF01006390.1	1.4	0.29	0.15	8.64	43.7	1.83
GAIF01006395.1	0.04	0	0	0.36	1.02	0.4
GAIF01006396.1	0.35	0.28	0	0.92	2.3	0.18
GAIF01006399.1	0.01	0	0.01	0	0	0
GAIF01006400.1	0.55	0.05	0.27	0.06	0.44	0.22
GAIF01006401.1	10.65	22.9	14.08	8.76	31.31	3.44
GAIF01006402.1	22.31	25.67	38.16	21.5	19.82	40.56
GAIF01006403.1	14.83	19.9	11.35	75.52	135.75	15.95
GAIF01006404.1	1.6	0.14	0.28	1.56	5.05	0.15
GAIF01006405.1	0.05	0.05	0.05	1.05	3.26	0.21
GAIF01006407.1	0	0	0	0	2.87	0
GAIF01006408.1	1.43	0.98	0.5	0.5	1.11	0.53
GAIF01006410.1	192.24	209.86	417.48	178.38	128.32	185.46
GAIF01006411.1	37.34	46.87	23.92	54.81	27.98	22.27
GAIF01006413.1	0	0.07	0	0.23	0.31	0.06
GAIF01006414.1	9.39	6.89	8.79	18.83	16.78	17.23
GAIF01006415.1	272.41	125.05	85.51	373.68	573.71	88.77
GAIF01006416.1	5.5	2.64	4.44	5.25	4.86	6.06
GAIF01006417.1	0.91	1.49	0.15	3.18	5.55	0.25
GAIF01006418.1	0.05	0.05	0	0.96	0.61	0.1
GAIF01006419.1	13.11	28.51	14.4	45.65	15.77	0
GAIF01006420.1	12.1	13.16	20.88	31.44	21.49	11.43
GAIF01006422.1	11.73	1.89	6.24	1.23	0.57	1.17
GAIF01006423.1	0.2	0	0.11	0	0.12	0

GAIF01006424.1	4.45	6.81	2.62	2.58	1.98	0
GAIF01006425.1	21.2	12.47	12.75	5.56	7.6	1.44
GAIF01006426.1	0	0	0.3	3.19	14.16	0
GAIF01006427.1	1.21	0.51	0.05	3.78	17.21	0.1
GAIF01006428.1	0.39	0	0	0	0.63	0
GAIF01006429.1	0.5	0.95	0	4.8	8.59	0
GAIF01006431.1	16.39	22.67	24.82	18.14	12.66	10.46
GAIF01006432.1	0.18	0.1	0.23	0.44	0	0
GAIF01006433.1	0.03	0	0	0	0	0
GAIF01006434.1	0	0	0	0.06	0.06	0
GAIF01006435.1	2.1	1.51	0	2.37	2.43	0.99
GAIF01006436.1	0.16	0.36	0.35	0.56	3.4	0
GAIF01006438.1	0.85	0.66	0.27	3.37	10.91	0
GAIF01006440.1	62.61	48.32	44.47	62.09	32.82	15.39
GAIF01006441.1	1.83	5.45	4.63	4.6	5	2.18
GAIF01006442.1	0	0.39	0	2.27	5.97	0
GAIF01006443.1	0.13	0	0.14	0.15	1.36	0.14
GAIF01006444.1	0.28	0	0.19	0.4	0.18	0.32
GAIF01006446.1	0.2	0	0	2.63	13.38	3.47
GAIF01006447.1	6.2	1.1	0.28	1.76	1.98	0
GAIF01006449.1	0.57	0.12	6.68	0.59	4.23	7.57
GAIF01006450.1	0.13	0.05	0	0.15	0.72	0
GAIF01006451.1	0	0	0	0.01	0.06	0
GAIF01006452.1	0	0	0	0	0.08	0
GAIF01006453.1	23.57	15.22	35.88	72.01	41.06	45.28
GAIF01006454.1	0.73	1.35	0.72	1.37	1.23	0.56
GAIF01006455.1	13.64	10.62	16.65	23.63	19.98	9.27
GAIF01006456.1	1.13	1.3	1.88	2.93	12.93	2.47
GAIF01006458.1	0.1	0	0.15	0	0.06	0
GAIF01006459.1	2.29	0.29	0	0.94	3.08	0.15
GAIF01006461.1	0	0	0	0	0.16	0
GAIF01006462.1	4.14	2.6	3.04	13.84	11.19	6.82
GAIF01006465.1	0.13	0.05	0.1	1.56	4.17	0
GAIF01006467.1	0.23	0.06	0.28	0.49	0.82	0.06
GAIF01006468.1	1340.02	1031.69	2236.78	353.04	232.13	685.35
GAIF01006469.1	0	0	0	0.08	0	0
GAIF01006470.1	0	0	0	0	0.9	0
GAIF01006471.1	0.87	0	0.03	0.79	0	0
GAIF01006473.1	16.78	26.92	25.99	38.9	25.35	10.1
GAIF01006476.1	78.16	69.47	37.43	111.48	112.86	52.95
GAIF01006478.1	3.84	4.58	0.75	16.75	28.76	1.23
GAIF01006479.1	0.24	0.04	0	0.32	0.57	0
GAIF01006481.1	0	0	0	0	0.39	0
GAIF01006485.1	17.63	18.07	17.82	32.35	33.33	11.04

GAIF01006486.1	5.95	10.1	8.87	29.64	29.78	3.36
GAIF01006487.1	35.28	33.04	35.68	29.87	22.41	69.73
GAIF01006490.1	0.12	0	0	0	0.28	0
GAIF01006492.1	0	0	0	0	0.25	0
GAIF01006493.1	0	0	0.31	1.3	4.04	0
GAIF01006494.1	0.03	0	0	0.35	1.79	0.06
GAIF01006495.1	0.27	0	0	0	0	0.3
GAIF01006496.1	0.65	0.18	0	4.84	9.64	0.18
GAIF01006498.1	0	0	0	0	1.43	0
GAIF01006499.1	1.6	0.32	0.8	0.68	1.4	0
GAIF01006500.1	1.18	0.39	0.1	3.36	8.71	0.5
GAIF01006501.1	0.06	0	0	0	0.07	0
GAIF01006502.1	14.5	15.15	12.39	68.9	38.05	16.31
GAIF01006504.1	0	0	0	0.04	0.05	0
GAIF01006505.1	4.69	5.86	1.72	6.97	24.15	0.27
GAIF01006506.1	0.79	0.29	0.23	1.47	21.28	0.29
GAIF01006508.1	57.2	71.74	68.26	37.69	36.59	55.3
GAIF01006510.1	0	1.26	3.33	0	0	0
GAIF01006511.1	77.99	70.11	27.57	236.7	406.97	313.72
GAIF01006514.1	0.17	0	0.27	0.45	0.38	0.3
GAIF01006515.1	1.96	1.72	0.54	0	0	0
GAIF01006516.1	2.49	1.64	0.5	1.18	2.57	0
GAIF01006517.1	0	0	0	0	1.04	0
GAIF01006518.1	0.13	0.05	0.15	0.56	0.16	0
GAIF01006519.1	0.04	0	0	0	0.13	0
GAIF01006524.1	0.44	0.48	0	1.34	4.9	0.2
GAIF01006526.1	0	0	0	0	0.1	0
GAIF01006528.1	34.56	43.55	36.77	81.99	33.67	7.23
GAIF01006529.1	973.17	627.85	476.64	672.42	282.02	264.12
GAIF01006530.1	0	0	0	0	0.12	0
GAIF01006532.1	31.11	84.63	70.53	10.84	0.68	7
GAIF01006533.1	3.13	1.89	5.5	0.67	1.36	1.22
GAIF01006534.1	2.63	2.76	2.05	8.3	7.39	1.54
GAIF01006535.1	0	0	0	0	0.22	0
GAIF01006536.1	6.87	3.59	3.94	4.85	0.87	1.91
GAIF01006537.1	0	0.07	0	0	0.08	0
GAIF01006538.1	52.49	38.39	16.49	33.39	12.87	18.03
GAIF01006539.1	0.4	0.15	0	0.31	1.45	0.15
GAIF01006540.1	13.52	9.23	17.46	35.26	13.09	25.7
GAIF01006541.1	0.12	0	0	0	0	0
GAIF01006542.1	65.09	58.32	40.42	144.53	72.66	35.62
GAIF01006543.1	0.21	0.09	0	0	0	0
GAIF01006544.1	0.27	0.59	0	1.25	0.4	0
GAIF01006545.1	57.15	34.84	22.08	70.95	79.83	19.92

GAIF01006548.1	0	2.51	0	15.65	10.08	0
GAIF01006549.1	0.16	0.18	0.36	0.05	0	0.27
GAIF01006553.1	3.05	0.68	0.37	9.19	8.51	0.45
GAIF01006554.1	4.43	10.11	5.29	8.19	8.86	0.74
GAIF01006556.1	0	0	0	0	0.67	0
GAIF01006557.1	2.2	0.23	0.16	8.07	10.24	0.39
GAIF01006560.1	1.94	0	0	0	2.34	0
GAIF01006561.1	0.6	0.18	0.33	0.7	1.66	0.34
GAIF01006563.1	13.48	21.89	12.88	26.66	8.16	2.8
GAIF01006564.1	0	0	0	0.15	0	0
GAIF01006566.1	3.03	6.77	3.05	13.2	11.71	0.59
GAIF01006568.1	4.25	2.32	2.77	5.92	11.25	5.44
GAIF01006569.1	6.47	7.93	8.04	7.96	5.32	3.14
GAIF01006570.1	0	0.24	0	2.23	7.26	0
GAIF01006571.1	18.22	10.32	5.88	11.55	7.18	4.22
GAIF01006572.1	0.06	0	0	0.07	0.22	0
GAIF01006573.1	0	0.14	0	0.07	0.23	0.07
GAIF01006574.1	0.05	0	0.15	0.05	1.17	0.05
GAIF01006576.1	6.77	0.87	0.39	8.69	33.44	34.38
GAIF01006582.1	19.43	16.89	32.83	25.57	12.1	30.16
GAIF01006583.1	0	0	0	0	0.39	0
GAIF01006584.1	0.66	0.76	0.31	4.58	10.96	0.23
GAIF01006585.1	0.03	0.03	0	0	0.16	0
GAIF01006586.1	7.81	0.81	0.62	18.21	127.98	1.58
GAIF01006587.1	1.15	0.56	0	0	0.6	0.43
GAIF01006588.1	0	0.36	4.1	0	0	0
GAIF01006590.1	19.08	30.53	23.51	88.77	55.91	8.63
GAIF01006591.1	0.56	0.2	0.27	3.1	9.49	0.07
GAIF01006592.1	0	0	0	0	0	0.09
GAIF01006593.1	5.51	11.28	3.03	19.76	19.3	1.25
GAIF01006594.1	0.67	0	1.82	7.12	7.55	0.04
GAIF01006595.1	0	0.13	0	0	0	0
GAIF01006596.1	0	0	0	0	0.34	0
GAIF01006597.1	20.56	23.35	39.9	41.76	16.89	12.79
GAIF01006598.1	8.27	4.55	3.75	13.01	21.34	3.01
GAIF01006600.1	0	0.06	0	0.8	0.51	0
GAIF01006602.1	41.73	72.91	39.62	13.45	6.91	0.79
GAIF01006604.1	0	0.98	1.37	0	0	0.31
GAIF01006607.1	14.7	12.18	20.55	17.77	10.73	24.88
GAIF01006608.1	0	0	0	0.09	0.32	0
GAIF01006609.1	0.59	0.35	0.35	4.29	28.99	0.42
GAIF01006611.1	8.29	21.54	10.26	40.17	32.86	1.47
GAIF01006612.1	0.05	0	0	0.22	1.14	0
GAIF01006613.1	5.67	6.32	2.42	15.54	10.01	2.38

GAIF01006614.1	0.27	0.58	0	1.09	4.18	0
GAIF01006616.1	0	0	3.13	0	0	0
GAIF01006617.1	0	0	0	0	0	0.26
GAIF01006619.1	10.67	5.61	2.99	0.5	0.43	0.16
GAIF01006620.1	33.36	28.01	22.5	48.39	23.12	14.41
GAIF01006621.1	0.38	0.1	0.1	1.53	8.06	2.81
GAIF01006622.1	17.54	18.78	18.44	51.55	27.48	12.07
GAIF01006624.1	71.96	54.33	104.31	10.18	2.85	7.45
GAIF01006625.1	16.97	9.54	22.49	28.36	13.55	15.64
GAIF01006626.1	0	0	0.15	0	0	0
GAIF01006628.1	0	0	0	0.17	0.71	0
GAIF01006630.1	19.78	15.48	11.27	64.36	41.2	14.55
GAIF01006632.1	0	0	0	0.27	1.41	0
GAIF01006633.1	0.14	0.75	0.9	0.48	0.66	0.15
GAIF01006634.1	0.06	0.03	0	0.56	1	0
GAIF01006635.1	0	0	0.14	0	0	0
GAIF01006636.1	0	0	0	0.59	0.61	1.39
GAIF01006637.1	0.08	0	0.18	0.48	0.95	0
GAIF01006638.1	0.87	0.71	0.33	5.09	0	0.2
GAIF01006641.1	0.41	0.2	0.1	0.22	0.53	0.1
GAIF01006642.1	0.09	0.1	0.1	0.11	0.45	0.21
GAIF01006643.1	14.83	16.03	9.16	19.63	21.32	2.11
GAIF01006644.1	1.01	1.1	0.65	2.63	8.99	0.56
GAIF01006646.1	0	0	0	0.03	0.12	0
GAIF01006647.1	10.37	10.17	9.79	32.1	21.22	7.63
GAIF01006649.1	0.37	0.3	0	1.39	1.44	0.1
GAIF01006650.1	0.06	0	0.08	0.26	0.49	0
GAIF01006651.1	23.96	9.73	8.73	4.96	1.79	0.88
GAIF01006652.1	2.91	2.78	1.76	1.57	1.63	0.94
GAIF01006653.1	0.51	0.41	0	2.67	0.42	0.99
GAIF01006655.1	0	0	0	0	0.08	0
GAIF01006658.1	0	0.09	0.05	0.29	0.9	0.09
GAIF01006661.1	0	0	0	0	0.09	0
GAIF01006663.1	0.08	0	0	0.1	0.6	0.55
GAIF01006664.1	635.11	411.73	423.05	666.51	385.53	342.33
GAIF01006665.1	63.8	13.74	23.4	37.62	20.95	41.03
GAIF01006667.1	23.25	13.52	11.06	31.59	41.79	6.58
GAIF01006669.1	0	0	0.06	0	0	0
GAIF01006670.1	0	0	0	0.04	0.95	0.22
GAIF01006672.1	0	0	0	0	0.2	0
GAIF01006674.1	0	0	0	0.64	1.57	0
GAIF01006676.1	0	0.06	0	0	0.54	0
GAIF01006677.1	2.79	1.01	0.82	8.41	29.61	1.13
GAIF01006678.1	0.33	0.12	0	0.64	2.13	0.24

GAIF01006679.1	12.23	13.45	8.72	17.86	10.6	4.36
GAIF01006680.1	33.04	32.18	24.42	67.14	48.16	21.23
GAIF01006681.1	6.87	0.54	0.59	5.37	51.03	6.9
GAIF01006682.1	38.94	31.93	16.93	49.14	46.53	16.22
GAIF01006683.1	0	0	0	0	0.14	0
GAIF01006684.1	1.56	0.25	0.04	2.69	15.03	26.15
GAIF01006685.1	5049.11	3327.68	5666.93	464.84	100.53	377.91
GAIF01006686.1	10.28	14.01	10.83	14.99	17.54	20.03
GAIF01006688.1	0	0.06	0	0.26	0	0.03
GAIF01006691.1	0.54	0.76	1.01	1.88	3.5	0.42
GAIF01006692.1	0	0	0	0	0.83	0
GAIF01006693.1	0.62	0.62	0.47	0	0.39	0
GAIF01006694.1	5.82	2.34	0.51	13.6	27.62	2.89
GAIF01006695.1	1.1	0	0	3.47	8.7	1.91
GAIF01006696.1	34.72	11.72	15.86	22.02	14.91	65.7
GAIF01006697.1	3.62	4.34	5.35	6.16	4.41	3.19
GAIF01006700.1	0.7	0.56	1.59	2.03	1.28	0.31
GAIF01006702.1	0.74	0.27	0.09	4.28	14.16	0.14
GAIF01006703.1	0.12	0	0	0	0	0
GAIF01006704.1	35.23	24.97	26.75	26.03	35.07	7.7
GAIF01006705.1	0.33	0	0	0	0.87	0
GAIF01006707.1	0.07	0.08	0	0.04	0.44	0.04
GAIF01006708.1	0	0.16	0	0	0	0
GAIF01006709.1	19.58	20.02	15.52	47.85	48.18	12
GAIF01006710.1	0	0	0	0.12	0	0
GAIF01006712.1	0.29	0	0.37	0	0	0
GAIF01006713.1	5.19	2.26	0.57	0	0	0
GAIF01006714.1	0	0	0	0.31	0	0
GAIF01006715.1	52.98	59.08	102.33	169.51	120.19	59.6
GAIF01006717.1	0	0	0	0.1	0.64	0
GAIF01006718.1	1.2	2.14	0.08	3.6	3.9	0.08
GAIF01006719.1	0.51	0.42	0.14	2.78	7.92	0.24
GAIF01006720.1	0	0	0	0	0.19	0
GAIF01006721.1	11.22	3.92	4.6	9	5.81	2.36
GAIF01006723.1	4.9	8.68	3.54	16.58	9.6	1.08
GAIF01006724.1	5.08	3.63	2.93	24.35	0.8	0.88
GAIF01006725.1	0	0	0	0.31	2.43	0.29
GAIF01006726.1	0	0	0	0	0.11	0
GAIF01006728.1	0.31	0	0.23	0.12	2.38	0
GAIF01006729.1	2.15	0.98	0.38	6.04	6.68	3.29
GAIF01006731.1	4.27	6.03	3.77	3.5	6.98	1.16
GAIF01006732.1	209.79	285.46	562.59	219.29	172.6	405.14
GAIF01006734.1	0.23	0	0	0	0.09	0
GAIF01006735.1	0	0.05	0	0	0	0.84

GAIF01006737.1	0.04	0.05	0	0	0.37	0
GAIF01006738.1	0.83	0.3	0.85	0.96	0.66	0.3
GAIF01006740.1	0.14	0.04	0.34	0.36	0.33	0.11
GAIF01006743.1	0	0.08	0	0.09	0.09	0.08
GAIF01006744.1	0.01	0	0	0	0	0
GAIF01006745.1	209.34	268.47	129.7	225.61	155.95	49.81
GAIF01006747.1	8.38	3.75	4.63	28.91	50.98	3.05
GAIF01006748.1	0	0	0	0.2	0.21	0
GAIF01006749.1	1.12	0.26	0	2.77	10.44	0.09
GAIF01006750.1	86.48	51.35	40.48	141.48	172.15	17.2
GAIF01006753.1	0.53	0	0	0	0.17	0.09
GAIF01006754.1	3.44	6.29	6.56	4.43	2.14	11.92
GAIF01006755.1	0	1.57	0.55	0	0.15	0
GAIF01006756.1	20.79	9.06	4.82	34.92	46.67	5.55
GAIF01006758.1	9.33	9.84	12.47	40.16	36.06	7.27
GAIF01006759.1	0.5	0	0	0.29	0	0
GAIF01006761.1	0.06	0	0	0	0.33	0
GAIF01006762.1	0.09	0.41	0.68	0.41	1.14	0
GAIF01006764.1	0.09	0	0	0.44	0.68	0
GAIF01006765.1	0.07	0.08	0	0.35	2.07	0
GAIF01006766.1	4.77	3.33	3.51	2.1	1.45	0.61
GAIF01006770.1	1.64	1.03	1.84	6.63	9.77	1.19
GAIF01006773.1	0.13	0	0	0.15	0	0
GAIF01006775.1	0	0	0	0	0.12	0
GAIF01006776.1	441.31	440.48	126.75	693.83	976.8	258.32
GAIF01006777.1	0	0.05	0	0	0	0
GAIF01006778.1	0	0	0	0.08	0.51	0
GAIF01006779.1	6.99	6.98	3.37	2.11	2.19	0.95
GAIF01006783.1	0.68	0.47	0.17	0.3	2.76	0.54
GAIF01006784.1	0.03	0.03	0.03	0.03	0.07	0
GAIF01006785.1	10.75	0	0.34	4.52	3.75	6.01
GAIF01006786.1	0.58	0	0.58	0.83	0	0
GAIF01006787.1	71.64	63.16	45.45	135.65	193.96	194.11
GAIF01006789.1	0	0.17	0	0.18	0.75	0
GAIF01006790.1	2.99	2.03	1.86	6.36	5.03	4.42
GAIF01006791.1	0	3.19	0	5.09	8.19	0
GAIF01006792.1	0.03	0.07	0	0.04	1.5	0
GAIF01006794.1	11.11	9.2	16.35	39.85	35.37	28.8
GAIF01006795.1	0	0	0.26	0.41	0.56	0.26
GAIF01006796.1	0.24	0.2	0	2.6	9.67	0.73
GAIF01006797.1	13.07	8.44	2.9	57.09	26.87	2.05
GAIF01006798.1	1.12	1.39	2.09	2.18	2.1	1.81
GAIF01006799.1	0.12	0.18	0.05	0.06	0	0.03
GAIF01006800.1	0.07	0.21	0.13	0.09	0.09	0

GAIF01006801.1	0	0	0	0	0.14	0
GAIF01006802.1	49.93	55.3	22.06	56.55	42.37	7.06
GAIF01006803.1	42.19	31.29	21.7	147.86	177.84	18.08
GAIF01006805.1	0	0.58	0.1	0	0	0.68
GAIF01006806.1	0	0	0	0	0	0.1
GAIF01006807.1	0.17	0.11	0	0	0	0.05
GAIF01006808.1	0	0.06	0	0	0	0
GAIF01006810.1	0.11	0.04	0	0.39	2.74	0.05
GAIF01006812.1	30.22	38.18	35.45	92.42	81.68	23.14
GAIF01006815.1	0	0.39	0.6	0	0	0.26
GAIF01006816.1	4.59	6.93	4.43	9.49	6.76	1.45
GAIF01006817.1	0	0	0	0	1.26	0
GAIF01006818.1	0	0	0	0	0.43	0.07
GAIF01006819.1	0.92	0.4	0.29	0.11	0.51	0
GAIF01006820.1	0	0.01	0	0.01	0	0
GAIF01006822.1	0.07	0.07	0	1.17	5.81	0.14
GAIF01006825.1	0	0.12	0.35	0.25	0.15	0
GAIF01006828.1	0.87	0.16	0.66	0.67	0.35	0.16
GAIF01006829.1	0	0	0	0	1.91	0
GAIF01006830.1	0	0	0	0.15	0.16	0
GAIF01006832.1	37.26	26.11	12.57	45.38	61.46	63.55
GAIF01006834.1	172.32	94.38	140.13	7.15	16.28	59.99
GAIF01006838.1	49.85	51.21	22.36	144	113.91	5.64
GAIF01006839.1	0.68	0.93	0.79	0.58	1.25	0.4
GAIF01006841.1	35.38	16.82	9.34	94.26	127.41	9.71
GAIF01006842.1	0	0.54	0.58	0	0.04	0.14
GAIF01006844.1	44.68	29.35	33.85	53.97	36.33	50.16
GAIF01006846.1	9.77	17.79	13	15.59	8.37	2.69
GAIF01006847.1	13.63	7.1	0.85	14.86	13.25	18.25
GAIF01006848.1	70.21	15.06	10.04	51.41	62.9	182.91
GAIF01006849.1	7.21	1.18	0.26	6.54	43.71	1.59
GAIF01006851.1	0	0	0	0.35	1.08	0.82
GAIF01006852.1	0	0.05	0	0	0	0
GAIF01006854.1	0	0	0	0	0.19	0
GAIF01006855.1	0.15	0.08	0	0.09	0.18	0
GAIF01006856.1	6.67	5.35	0.36	8.2	9.79	0
GAIF01006857.1	9.71	3.48	6.36	13.08	17.17	19.8
GAIF01006858.1	0	0	0	0	0.09	0.04
GAIF01006859.1	36.48	27.65	15.34	3.44	0	7.51
GAIF01006860.1	0.62	0.65	0	0.56	0	0
GAIF01006861.1	0.07	0.08	0	0	0.09	0
GAIF01006862.1	0.94	1.41	1.38	0.79	2.09	1.05
GAIF01006863.1	3.61	1.8	0.17	0.86	6.84	1.59
GAIF01006864.1	8.96	3.79	4.5	10.27	21.21	28.53

GAIF01006865.1	1.88	0.37	1.4	2.11	2.83	0.82
GAIF01006866.1	1.14	0.76	0.09	9.25	34.16	0.05
GAIF01006867.1	4.7	3.54	8.59	7.72	1.88	3.28
GAIF01006868.1	0.17	0	0	0	0	0
GAIF01006869.1	0.37	0.09	0.12	0.16	0.12	0.12
GAIF01006870.1	0	0	0	0.1	0	0
GAIF01006872.1	47.37	28.83	86.05	134.77	58.38	376.5
GAIF01006873.1	0.1	0.11	0	0.12	0.49	0.23
GAIF01006875.1	0.34	0.28	0.19	0.1	0.1	0.05
GAIF01006877.1	0	0	0	0	0.13	0
GAIF01006879.1	0	0	0	0	0.13	0
GAIF01006881.1	0.75	0.96	0.07	4.03	6.71	0
GAIF01006882.1	0.76	0.12	0	7.16	25.41	0.42
GAIF01006883.1	0.11	0.06	0	1.22	3.2	1.16
GAIF01006884.1	0	0	0	0.11	0	0
GAIF01006886.1	1.71	1.21	0.45	0.89	0.21	0
GAIF01006887.1	0	0	0	0	0.32	0
GAIF01006888.1	0.19	0.05	0.05	0.06	0.06	0.05
GAIF01006889.1	0.64	0	0.17	3.23	9.22	0.92
GAIF01006890.1	0.04	0	0	0.26	1.02	0.05
GAIF01006892.1	21.15	1.59	1.43	1.39	0.35	0
GAIF01006893.1	0.59	0.04	0.03	1.28	0.15	0
GAIF01006894.1	187.43	330.42	476.66	823.84	589.19	109
GAIF01006897.1	8.51	12.81	6.81	43.76	63.72	9.44
GAIF01006899.1	0	0	0	0.32	1.48	0.09
GAIF01006900.1	183.78	98.22	44.21	132.87	169.17	147.56
GAIF01006901.1	0	0	0	0	0.09	0
GAIF01006902.1	1.17	0.85	0.21	5.21	21.47	0.86
GAIF01006903.1	0.55	0.18	0.24	4.44	15.43	0.18
GAIF01006905.1	0.71	0.39	0.31	0.66	0.17	0.16
GAIF01006906.1	0	0.26	0	0	0	0.17
GAIF01006907.1	0.37	0.08	0	0.63	2.16	0
GAIF01006908.1	0.09	0.1	0	0.86	3	0.1
GAIF01006911.1	11.85	15.74	12.3	1.31	1.16	0.06
GAIF01006912.1	0	0	0	0.22	1.43	0
GAIF01006913.1	0.06	0.06	0	0	0	0
GAIF01006914.1	0.23	0.25	0.2	0.64	4.72	0.15
GAIF01006915.1	0	0	0	0.18	0.73	0.84
GAIF01006916.1	1.96	1.6	2.16	0.95	0.2	1.44
GAIF01006917.1	0.39	0	0	0.25	2.23	0
GAIF01006918.1	0	0	0	0	0.05	0
GAIF01006919.1	0	0	0.64	0	4.08	0.23
GAIF01006921.1	0.06	0	0.02	0	0	0
GAIF01006924.1	0	0	0.3	0	0	0

GAIF01006925.1	4.65	8.29	3.34	18.11	10.82	1.18
GAIF01006926.1	0	0	0	0	0.37	0.08
GAIF01006927.1	0	0	0	0.25	4.31	0
GAIF01006929.1	0	0.03	0	0	0	0
GAIF01006930.1	6.68	7.48	7.26	9.55	6.81	5.2
GAIF01006931.1	0	0	0	0.59	4.32	0
GAIF01006933.1	0.22	0.71	0	0.5	3.38	0
GAIF01006934.1	0.04	0	0	0	0	0
GAIF01006935.1	0.06	0	0	0.39	2.24	0.06
GAIF01006937.1	0	0	0.16	0	0	0
GAIF01006938.1	8.06	4.44	5.42	26.54	30.85	15.79
GAIF01006939.1	9.67	3.3	6.46	17.4	21.77	40.42
GAIF01006940.1	0.36	0.59	0	2.21	6.01	0
GAIF01006941.1	0	0	0	0	0.33	0
GAIF01006942.1	0.2	0	0	0.39	0.57	0.22
GAIF01006943.1	0	0	0	0	0.1	0
GAIF01006944.1	256.81	289.8	396.43	212.72	170.36	198.56
GAIF01006945.1	0	0	0	0.11	0.2	0
GAIF01006946.1	0	0	0	0.32	0	0
GAIF01006947.1	0	0	0	0	0.07	0
GAIF01006949.1	0	0.29	0.96	0	0	0.1
GAIF01006950.1	0.19	0.28	0.06	1.44	12.4	0.29
GAIF01006954.1	5.16	8.74	5.29	8.14	6.99	1.21
GAIF01006955.1	0.48	0	0.24	6.98	12.31	0
GAIF01006959.1	5.4	6.87	2.42	16.9	16.91	1.64
GAIF01006960.1	4.07	5.04	2.98	1.71	1.5	0.62
GAIF01006961.1	0	0.05	0	0	0.05	0
GAIF01006963.1	22.85	12.8	20.41	49.71	126.51	59.83
GAIF01006964.1	10.43	3.33	0.13	10.11	13.87	0
GAIF01006965.1	0.1	0	0	0.37	3.27	0.46
GAIF01006967.1	1.73	0.12	0	2.01	9.24	0.48
GAIF01006968.1	7.97	6.4	10	18.07	17.47	7.11
GAIF01006969.1	0	0	0	0	0.46	0.08
GAIF01006970.1	4.03	1.2	0	1.04	0	0.32
GAIF01006972.1	0.07	0.04	0	0.53	4.47	0.47
GAIF01006973.1	0.17	0.09	0	2.06	7.94	0.47
GAIF01006974.1	35.14	29.18	19.5	40.52	39.86	8.84
GAIF01006975.1	13.35	39.72	62.19	37.86	22.71	11.41
GAIF01006976.1	6.59	6.68	8.7	25.44	11.19	0
GAIF01006977.1	0	0	0	0	18.05	0
GAIF01006978.1	0	0.18	0.14	0.64	4.88	0
GAIF01006979.1	387.08	953.43	1246.66	160.74	464.86	393.02
GAIF01006980.1	0.03	0	0	0.21	1.18	0
GAIF01006982.1	7.67	4.11	5.57	1.83	0.61	0.37

GAIF01006983.1	0.2	0.06	0	0.17	0.21	0
GAIF01006984.1	12.75	12.21	11.31	28.49	28.87	4.6
GAIF01006985.1	0	0	0	0.06	0.12	0
GAIF01006987.1	16.29	15.96	1.27	72.11	120.46	8.67
GAIF01006988.1	13.23	12.73	6.91	29.97	18.36	5.33
GAIF01006989.1	3.93	3.55	1.03	1.76	0.84	0.13
GAIF01006991.1	0.07	0	0	0.05	0.17	0
GAIF01006992.1	0.7	0.9	0	0	0	0
GAIF01006994.1	2.69	5.17	3.09	1.15	1.43	0.13
GAIF01006996.1	0.12	0	0	0	0	0
GAIF01006997.1	0.21	0	0.11	0	0.07	0
GAIF01006999.1	0.04	0.04	0	0.29	1.09	0
GAIF01007000.1	0	0.1	0.2	0.41	0	0.2
GAIF01007003.1	0.04	0	0.06	0.08	0	0.26
GAIF01007005.1	0.2	0.02	0.01	1.75	0.08	0
GAIF01007006.1	2.12	3.07	1.77	1.76	1.34	0.11
GAIF01007007.1	1.19	0.73	0	4.41	0	0.8
GAIF01007008.1	3.99	3.22	2.6	15.97	17.97	0.98
GAIF01007009.1	0.03	0	0	0	0	0
GAIF01007011.1	0.28	0.31	0.34	0.21	0.4	0
GAIF01007012.1	16.55	18.92	12.18	2.91	2.49	0.26
GAIF01007014.1	0	0	0	0	0.25	0
GAIF01007015.1	0.49	0	0	1.43	5.03	2.17
GAIF01007016.1	9.11	0	0	11.21	13.76	2.87
GAIF01007018.1	185.52	183.79	370.53	196.74	143.71	215.53
GAIF01007019.1	0	0	0	0	0.13	0
GAIF01007020.1	0.31	0	0	0.21	0	0
GAIF01007021.1	0.06	0	0	0	0.07	0.06
GAIF01007023.1	0.68	0	1.05	1.74	2.3	0
GAIF01007025.1	9.78	9.66	12.83	6.65	18.45	9.27
GAIF01007027.1	7.05	6.09	6.33	12.08	9.82	6
GAIF01007030.1	0.08	0	0	0	0.09	0
GAIF01007031.1	0	0.29	0	0	0	0
GAIF01007033.1	0	0	0	0.12	0.34	0
GAIF01007035.1	0	0.31	0.24	0	0.06	0.05
GAIF01007036.1	0	0	0	0	0.01	0
GAIF01007037.1	2.56	1.67	1.97	2.82	0.31	2.4
GAIF01007038.1	3.11	1.66	0.7	8.77	23	6.3
GAIF01007039.1	1.06	0	0.46	0	0	0
GAIF01007040.1	1.36	0.51	0.2	7.99	24.16	0.58
GAIF01007041.1	0	0	0	0.14	0.67	0
GAIF01007042.1	0	0	0.09	0.18	0	0
GAIF01007043.1	0.06	0.07	0	1.02	3.19	0.07
GAIF01007044.1	35.03	18.65	2.15	59.16	92.9	24.85

GAIF01007045.1	0.17	0.22	0.04	0.09	0.38	0
GAIF01007046.1	0.36	0.06	0	1.73	7.66	0
GAIF01007048.1	0.1	0	0	0.23	1.39	0
GAIF01007049.1	0	0	0	0.23	2.7	0
GAIF01007051.1	0.9	0	0.21	2.84	5.23	0
GAIF01007052.1	0.09	0	0	0	0	0
GAIF01007055.1	35.86	48.77	37.96	90.75	68.26	8.07
GAIF01007058.1	0	0.13	0.4	0	0	0
GAIF01007059.1	0	0.07	0	0.08	0	0
GAIF01007061.1	13.51	13.29	5.69	15.07	16.18	1.53
GAIF01007065.1	0	0	0	0	0	0.14
GAIF01007066.1	113.99	123.45	110.11	146.04	126.99	225.66
GAIF01007067.1	0	0	0	0	0.07	0
GAIF01007068.1	0.08	0	0	0.09	0.39	0.27
GAIF01007071.1	1399.28	521.04	312.45	885.08	1536.1	2979.27
GAIF01007073.1	29.21	17.97	24.46	21.4	13.61	75.46
GAIF01007074.1	0	0.07	0	0.76	9.65	0.05
GAIF01007076.1	3.78	2.38	4.71	7.52	8.16	1.86
GAIF01007077.1	0.2	0	0	0.06	0.18	0
GAIF01007078.1	132.52	126.25	117.91	280.83	190	66.69
GAIF01007079.1	0.45	0	0	0.17	0	0
GAIF01007080.1	3.13	1.91	3.16	2.33	0.75	1.93
GAIF01007081.1	0	0.1	0	0	0	0
GAIF01007082.1	0.17	0	0	0	0	0
GAIF01007083.1	53.36	29.39	54.06	17.73	5.73	18.98
GAIF01007084.1	0.32	0.52	0.23	0.55	1.34	0.18
GAIF01007085.1	27.83	10.96	6.35	59.08	83.03	29.3
GAIF01007086.1	166.87	98.45	165.06	7.9	0	5
GAIF01007088.1	0	0	0	0	0.06	0
GAIF01007089.1	2.52	1.4	4.05	3.65	1.96	5.37
GAIF01007090.1	0	0	0	0.09	0.29	0.09
GAIF01007092.1	20.69	5.72	1.74	39.69	73.67	39.26
GAIF01007093.1	0.04	0	0	0.33	1.39	0
GAIF01007094.1	6.22	14.81	10.49	15.42	12.64	0.15
GAIF01007095.1	0	0.33	0.92	0.64	0.38	0
GAIF01007096.1	0.36	0	0	0.28	0.58	0.13
GAIF01007097.1	5.9	1.24	0.44	15.9	17.5	4
GAIF01007098.1	0	0	0	0	0.09	0
GAIF01007099.1	4.63	1.08	0.39	36.37	88.64	0.94
GAIF01007100.1	0	0	0	0.03	0.13	0
GAIF01007101.1	0	0	0	0.22	1.45	0
GAIF01007102.1	20.56	5.86	3.34	72.95	136.21	26.54
GAIF01007103.1	0.14	0	0	0	0	0
GAIF01007104.1	5.15	0.71	0.11	37.48	116.86	3.93

GAIF01007106.1	0	0	0	0	1.53	0
GAIF01007107.1	0.24	0.11	0.03	1.19	5.45	0.99
GAIF01007108.1	0	0.76	0.57	0	0.04	4.28
GAIF01007109.1	16.27	14.8	0	0	24.99	8.3
GAIF01007110.1	9.86	18.11	10.99	21.9	12.04	1.43
GAIF01007111.1	4350.09	4890.12	9290.64	2386.26	1669.52	6245.17
GAIF01007112.1	0.46	0.13	0.32	0.54	1.26	0.38
GAIF01007113.1	1.01	0.05	0.2	3.47	15.04	0.91
GAIF01007114.1	0	0	0	0.54	6.75	0.34
GAIF01007116.1	3.55	0.5	0.76	4.21	7.52	31.3
GAIF01007117.1	0.07	0.22	0	1.54	3.93	0.44
GAIF01007118.1	3.4	0.34	0	0.28	6.54	0.32
GAIF01007120.1	0.06	0.12	0	0	0	0
GAIF01007121.1	0	0.05	0	0	0	0
GAIF01007123.1	0	0	0	0.38	0.62	0
GAIF01007126.1	0.08	0	0	0	0	0
GAIF01007127.1	0.1	0	0	0	0	0
GAIF01007128.1	3.27	2.7	0.57	11.52	7.54	3.6
GAIF01007129.1	0.29	0.42	0	0.89	0.7	0.11
GAIF01007130.1	0.09	0.07	0.34	0	0	0
GAIF01007131.1	0	0	0	0	0.34	0
GAIF01007132.1	0.16	0.38	0.26	0.41	1.08	0.26
GAIF01007133.1	0.28	0.12	0.19	5.9	12.13	0.34
GAIF01007134.1	0	0.3	0	0	0.8	0
GAIF01007135.1	0	0.15	0	0.33	1.37	0.31
GAIF01007136.1	1.18	0	0.42	0	0	0
GAIF01007137.1	0.2	0.04	0.04	0.58	4.79	0.04
GAIF01007138.1	0	0	0.16	0	0	0
GAIF01007139.1	0	0	0	0.45	0	0
GAIF01007142.1	0	0	0	0	0.07	0.07
GAIF01007143.1	62.12	28.51	21.23	107.16	142.05	120.23
GAIF01007144.1	25.27	6.73	7.21	8	4.94	2.71
GAIF01007145.1	0	0	0	0.09	1.01	0
GAIF01007147.1	0	0	0	0.66	2.33	0
GAIF01007148.1	16.89	28.17	15.54	91.47	107.5	12.58
GAIF01007149.1	0	0	0	0.36	2.03	0
GAIF01007150.1	2.66	0.98	0.01	6.56	5.75	4.68
GAIF01007152.1	0	0	0	0	0.21	0
GAIF01007153.1	0	0	0	0	1.22	0
GAIF01007155.1	0	0.07	0	0	0.22	0
GAIF01007157.1	19.1	9.26	3.08	0	0.11	0
GAIF01007158.1	0	0	0	0.08	0.32	0
GAIF01007159.1	1.22	0.05	0.15	0.48	0.82	1.04
GAIF01007160.1	22.85	24.69	8.11	77.28	35.52	3.22

GAIF01007161.1	0	0.33	0	0	0	0
GAIF01007162.1	0	0	0	0	1.85	0
GAIF01007165.1	168.9	63.55	33.58	133.51	146.4	22.32
GAIF01007166.1	0.29	0.32	0.06	0.29	1.07	0
GAIF01007167.1	3.11	0.81	0.69	13.19	42.45	6.26
GAIF01007168.1	685.86	436.15	336.11	552.51	391.52	316.5
GAIF01007169.1	0.07	0.07	0.23	0.59	0.48	0.05
GAIF01007170.1	0.62	1.29	0.74	1.5	0.93	0.11
GAIF01007171.1	1346.42	1216.86	849.6	738.99	495.21	233.37
GAIF01007173.1	8.39	17.96	6.82	8.87	6.24	0.6
GAIF01007174.1	4.06	2.22	3.97	5.09	1.69	2.16
GAIF01007175.1	0.19	0.07	0	0.15	0.46	0
GAIF01007177.1	257.76	227.89	152.63	59.06	130.42	19.66
GAIF01007178.1	1.43	0.41	0.91	2.37	1.09	0.64
GAIF01007179.1	0	0.13	0.14	0	0.3	0
GAIF01007180.1	0	0.28	1.48	0	0	1.15
GAIF01007181.1	11.43	5.61	0.48	34.84	49.05	5.34
GAIF01007182.1	0	0.36	0.55	0	0	0
GAIF01007183.1	0.19	0.04	0.25	1.78	5.41	1.07
GAIF01007184.1	3.08	1.41	0.34	4.27	8.37	11.83
GAIF01007186.1	0	0	0	0	0.1	0
GAIF01007187.1	5.53	2.06	1.26	3.99	22.14	14.98
GAIF01007188.1	0.07	0.08	0.08	0	0	0
GAIF01007189.1	841.78	368.5	439.76	535.49	202.33	441.74
GAIF01007190.1	0	0.07	0	0.04	0.05	0
GAIF01007191.1	24.14	38.47	36.08	38.32	32.33	2.56
GAIF01007192.1	0.23	0	0	1.57	4.88	0.75
GAIF01007193.1	0.05	0.03	0	0.03	0	0
GAIF01007194.1	1.21	0	0	1.64	8.97	1.7
GAIF01007195.1	0.04	0.04	0.01	0.1	0.02	0
GAIF01007197.1	12.88	19.87	6.23	51.29	33.54	2.13
GAIF01007199.1	0	0.54	0	0.57	0	0.27
GAIF01007201.1	0.05	0.11	0	0.23	1.07	0
GAIF01007202.1	95.58	13.25	2.1	296.26	703.41	43.12
GAIF01007203.1	0.08	0	0	0	0	0
GAIF01007205.1	0	0	0	0	0.57	0
GAIF01007206.1	1.57	0.62	0.31	4.8	8.24	0.16
GAIF01007208.1	0	0	0	0.35	1.99	0
GAIF01007210.1	13.05	4.83	2.79	1	0.83	1.57
GAIF01007212.1	0	0	0	0	0.18	0
GAIF01007213.1	0	0	0	0.23	1.06	0.11
GAIF01007215.1	4.59	8.47	3.7	17.05	16.02	1.41
GAIF01007217.1	0	0	0	0	0.08	0
GAIF01007220.1	53.72	99.99	23.74	148.95	133.21	8.27

GAIF01007221.1	68.33	68.92	115.08	206.7	107.46	75.65
GAIF01007222.1	0.52	0.65	0	3.38	5.66	1.23
GAIF01007223.1	15.58	15.18	16.57	34.14	20.75	7.72
GAIF01007225.1	0	0.04	0.04	0	0	0
GAIF01007227.1	0	0	0	0	0.37	0.06
GAIF01007228.1	3.92	5.3	0	1.82	0	0.29
GAIF01007229.1	3.9	1.32	1.77	21.29	81.89	45.98
GAIF01007230.1	0.94	0.09	0	5.57	31.89	0.69
GAIF01007232.1	8.58	11.86	7.81	37.14	27.33	4.26
GAIF01007233.1	0.87	0.12	0.06	0.13	0	0.06
GAIF01007234.1	0.01	0	0.03	0	0	0
GAIF01007235.1	57.49	42.92	37.21	118.5	76.62	23.4
GAIF01007236.1	0	0	0	0.1	0	0
GAIF01007238.1	5375.79	3541.95	2369.47	4047.1	3449.12	2308.36
GAIF01007239.1	3.72	2.16	0.23	19.21	33.13	0.4
GAIF01007240.1	0	0	0	0	0.02	0
GAIF01007242.1	1.84	1.26	1.43	9.87	20.42	0.4
GAIF01007245.1	0	0	0	0	0.12	0
GAIF01007246.1	0.31	0.27	0.08	3.17	12.6	0.08
GAIF01007247.1	0	0.22	0	0	0.1	0
GAIF01007248.1	0	0.05	0	0	0	0.27
GAIF01007249.1	0.06	0.25	0.34	0.23	0.18	0
GAIF01007253.1	1.01	0.22	0	0.45	10.09	0.63
GAIF01007254.1	0.02	0.02	0.02	0	0.48	0
GAIF01007255.1	1.11	0.13	0	3.74	19.81	2.73
GAIF01007256.1	9.85	16.33	12.47	12.6	13.97	2.5
GAIF01007257.1	0	0	0	0	5.52	0
GAIF01007259.1	49.54	36.03	18.36	13.56	13.31	7.68
GAIF01007260.1	12.38	16.22	6.55	13.51	34.63	3.12
GAIF01007261.1	0.07	0	0.18	0	0	0
GAIF01007262.1	45.1	19.32	20.57	36.82	59.43	226.53
GAIF01007263.1	0.12	0	0.25	0	0	0
GAIF01007264.1	1.63	0.75	0	3.46	2.22	0.54
GAIF01007267.1	39.67	34.5	14.47	108.8	57.98	6.38
GAIF01007268.1	8.32	4.02	1.01	10.37	12.6	2.21
GAIF01007269.1	25.21	11.11	22.59	53.05	36.08	78.18
GAIF01007271.1	0.62	0.47	0.26	0.29	0	0.21
GAIF01007272.1	3.62	3.17	0.21	19.15	50.52	8.53
GAIF01007273.1	0	0	0	0	0.08	0
GAIF01007274.1	17.29	6.91	15.33	41.49	24.07	13.67
GAIF01007276.1	0.16	0	0.17	0.18	0	0
GAIF01007277.1	0.39	0.25	0.59	0.23	0	0.32
GAIF01007278.1	0.44	0.28	0.62	1.48	1.2	0.65
GAIF01007279.1	0	0	0	0	0.17	0

GAIF01007280.1	40.64	32.27	21.25	30.08	30.83	5.65
GAIF01007282.1	0	0	0	0.05	0	0
GAIF01007283.1	0.05	0	0	0	0.24	0
GAIF01007284.1	23.88	7.55	9.9	1.7	0.44	2.69
GAIF01007286.1	0.26	0.09	0	1.51	3.87	0.77
GAIF01007288.1	2.68	5.92	1.68	14.77	25.83	0.1
GAIF01007289.1	0.11	0	0	2.65	7.58	0.48
GAIF01007290.1	0	0	0	0.35	0	0
GAIF01007291.1	31	19.66	33.85	51.97	19.89	34.93
GAIF01007292.1	0.27	0	0	0.05	0.05	0
GAIF01007294.1	0	0.08	0.08	0	0	0.01
GAIF01007295.1	0.21	0.45	0.23	1.68	10.2	1.14
GAIF01007297.1	49.39	50.77	95.89	156.21	63.03	109.65
GAIF01007298.1	0.13	0	0	0	0	0
GAIF01007299.1	6.36	9.02	10.79	18.79	14.34	4.44
GAIF01007301.1	0	0	0.6	0	0	0
GAIF01007302.1	0	0.21	0	0	0	0
GAIF01007303.1	0	0	0	0	0.16	0
GAIF01007305.1	0	0	0	0	0.78	0
GAIF01007307.1	9.03	13.34	12.78	48.46	27	8.54
GAIF01007308.1	0	0	0	0.86	1.97	0
GAIF01007309.1	0	0	0	0.97	1.04	0
GAIF01007312.1	5.16	0.73	7.32	4.6	2.83	1.69
GAIF01007313.1	0.16	0.26	0	0.44	1.65	0.18
GAIF01007314.1	0	0	0.03	0.03	0.16	0
GAIF01007315.1	43.03	26.81	54.44	1.53	0	1.74
GAIF01007316.1	1.04	0.31	0.26	8.5	34.25	0.29
GAIF01007319.1	17.88	32.74	38.08	68.38	63.02	33.58
GAIF01007320.1	0.59	1.28	0.09	0.78	2.22	0.18
GAIF01007321.1	0.4	0.09	0	0.24	0.52	0.06
GAIF01007324.1	0	0	0	0	0.25	0
GAIF01007326.1	28.96	20.15	37.02	109.34	52.94	33.47
GAIF01007327.1	5.57	13.14	5.51	22.94	24.72	3.46
GAIF01007329.1	90.54	81.66	77.28	83.4	54.3	13.27
GAIF01007330.1	4.37	0.22	1	26.63	208.45	7.72
GAIF01007331.1	1.02	0.19	0.84	0	0.1	0
GAIF01007332.1	0.83	0.95	0.34	1.56	0.73	0.07
GAIF01007334.1	0.05	0.06	0	0	0	0
GAIF01007338.1	54.06	16.19	7.57	120.52	177.15	57.66
GAIF01007339.1	0	0	0	0	0.05	0
GAIF01007341.1	441.48	343.32	437.78	12.17	7.29	5.42
GAIF01007342.1	0.15	0	0	0.52	1.19	0
GAIF01007343.1	40.16	16.27	4.48	26.52	31.6	39.85
GAIF01007344.1	39.92	22.54	26.2	27.78	18.8	23.38

GAIF01007345.1	0.24	0	0	0.14	0.43	0
GAIF01007346.1	4.64	0	5.37	0.65	3.73	2.35
GAIF01007347.1	2.61	0	0.08	0.51	1.63	0.43
GAIF01007348.1	0.03	0	0.03	0.09	0.37	0.03
GAIF01007349.1	5467.64	4625.38	6328.77	3094.41	2573.26	8011.3
GAIF01007350.1	0	0.14	0.09	0	0	0.14
GAIF01007351.1	0.56	0.24	0.67	0.13	0	0.18
GAIF01007352.1	168.92	184.69	292.91	133.9	102.25	167.68
GAIF01007353.1	0	0.1	0	0.67	3	0.21
GAIF01007354.1	0.92	0.74	1.07	3.2	6.94	1.16
GAIF01007355.1	0	0	0.16	0	0.21	0
GAIF01007356.1	1.6	0.67	1.5	6.18	9.78	0
GAIF01007357.1	0.29	0	0	0.29	7.12	0.26
GAIF01007358.1	1.76	2.82	0.51	12.64	14.12	2.98
GAIF01007360.1	7.35	6.64	7.28	13.44	4.5	9.63
GAIF01007361.1	0	0	0	0.13	0.22	0
GAIF01007362.1	7.57	5.94	5.63	19.77	10.16	4.03
GAIF01007363.1	743.88	353.21	462.45	332	107.6	99.28
GAIF01007364.1	0.47	0.51	0	1.8	2.4	0
GAIF01007365.1	0	0	0	0	0.25	0
GAIF01007366.1	0.12	0	0	0.27	0.42	0.51
GAIF01007368.1	9.2	21.29	16.57	0.22	0.17	0.71
GAIF01007369.1	13.99	24.02	33.07	11.83	12.9	23.13
GAIF01007371.1	0	0	0	0.14	0	0.14
GAIF01007372.1	0.51	0	0.45	0.67	2.14	0.54
GAIF01007373.1	0.46	0	0	1.07	4.46	5.36
GAIF01007375.1	0	0	0	0	1.84	0
GAIF01007376.1	0	0	0	0.63	2.99	0
GAIF01007377.1	0.99	0.56	0.99	0.84	0.67	0.18
GAIF01007378.1	20.4	25.66	25.24	21.2	10.44	5.37
GAIF01007380.1	0.04	0	0	0	0	0
GAIF01007383.1	0	0.23	0	0.4	0.75	0
GAIF01007384.1	0	0	0.15	0.03	0	0
GAIF01007385.1	8.99	9.77	7.81	37.97	19.8	5.63
GAIF01007386.1	0	0.06	0.06	0	0	0
GAIF01007387.1	110.83	29.65	27.11	0.26	0.4	0.31
GAIF01007388.1	0	0	0	0	0	0.11
GAIF01007390.1	0	0	0	0	0.16	0
GAIF01007391.1	0	0	0	0.88	0	0
GAIF01007392.1	0.12	0	0	0.29	0.44	0
GAIF01007393.1	0	0.13	0.13	0	0.29	0
GAIF01007394.1	0.23	0.11	0	0.08	0.79	0
GAIF01007395.1	23.43	35.12	22.01	23.81	31.91	3.14
GAIF01007396.1	6.05	11.32	11.26	0.37	0	0.97

GAIF01007397.1	17.99	13.41	12.52	44.46	36.14	10.32
GAIF01007398.1	10.64	9.36	7.61	23.88	24.4	2.39
GAIF01007400.1	3194.79	1391.53	681.43	1382.71	1163.21	2788.74
GAIF01007401.1	60.32	69.99	64.96	100.91	118.19	114.63
GAIF01007402.1	0.89	0.02	0.02	2.11	6.47	1.94
GAIF01007404.1	69.01	20.78	2.49	8.87	17.72	0.73
GAIF01007406.1	17.59	10.41	6.08	10.84	10.19	7.05
GAIF01007408.1	0.17	0	0	0.39	0.5	0
GAIF01007409.1	14.33	16.58	12.77	32.15	19.66	6.51
GAIF01007410.1	3.66	2.72	3.28	0.45	0.12	0.37
GAIF01007411.1	11.86	10.68	3.46	60.22	109.93	1.2
GAIF01007412.1	3.8	1.69	1.53	1.58	1.9	2.09
GAIF01007413.1	0.1	0.22	0.52	0.05	0.45	0.45
GAIF01007414.1	3.39	2.66	2.79	6.55	8.94	2.28
GAIF01007415.1	11.43	3.16	0	13.06	13.14	2.51
GAIF01007416.1	0	0	0	0	0.01	0
GAIF01007417.1	0.03	0	0	0	0.27	0
GAIF01007418.1	0	0	0	0	0.05	0
GAIF01007419.1	2.47	5.59	2.71	14.8	34.12	1.31
GAIF01007420.1	0	0	0	0	0.22	0
GAIF01007422.1	1.62	2.13	0.23	0.74	1.52	0.72
GAIF01007423.1	0	0.29	0.22	0	0	0
GAIF01007424.1	0	0	0	0.24	0.28	0
GAIF01007426.1	16.1	18.94	14.87	38.72	35.28	9.38
GAIF01007427.1	0	0	0.93	0	4.07	0
GAIF01007428.1	0	0	0	0	0.59	0
GAIF01007430.1	1.75	0.26	0	1.29	1.44	0.53
GAIF01007431.1	0	0	0	1.17	3.27	0.22
GAIF01007432.1	33.13	63.53	27.11	84.49	48.74	8.35
GAIF01007433.1	0	0.05	0	0.35	1.25	0.52
GAIF01007434.1	14.22	16.98	4.62	8.94	9.24	0.72
GAIF01007435.1	3.21	0.98	0.29	0.39	0.16	0.69
GAIF01007437.1	0	0	0	0.46	0.29	0
GAIF01007438.1	2.25	1.35	2.22	4.61	2.21	1.1
GAIF01007439.1	1.75	3.21	1.36	1.19	0.26	0.09
GAIF01007441.1	0	0	0	0.24	1	2.51
GAIF01007442.1	6.17	7.45	12.04	12.47	6.6	10.33
GAIF01007443.1	0	0	0	0	0.13	0.12
GAIF01007446.1	0	0.16	0.16	0.17	0	0.16
GAIF01007448.1	0.19	0.2	0	0.22	0.67	0
GAIF01007449.1	0	0	0	0.18	4.4	0.09
GAIF01007450.1	0	0	0	0	0.05	0
GAIF01007451.1	0	0.07	0.21	0.95	5.09	0
GAIF01007452.1	48.69	6.22	14.32	29.4	5.62	25.44

GAIF01007454.1	0.33	0	0	1.14	3.66	0.24
GAIF01007456.1	1.67	1.62	1.95	5.99	10.53	1.32
GAIF01007457.1	0	0	0	0	0.53	0
GAIF01007458.1	1.49	0.19	0.26	3.81	14.77	21.29
GAIF01007459.1	113.73	137.9	251.11	105.02	77.15	127.9
GAIF01007461.1	0.11	0.12	0	0	0	0
GAIF01007462.1	6.34	1.94	0.83	20.55	29.93	1.38
GAIF01007463.1	0.31	0.16	0.16	0	0.23	0.16
GAIF01007464.1	0.24	0	0	0.34	0.58	0.27
GAIF01007465.1	13.47	14.96	20.66	0.5	0.26	0.08
GAIF01007466.1	81.12	36.86	26.99	92.08	37.75	17.29
GAIF01007467.1	37.6	46.35	54.82	1.03	0.47	0.22
GAIF01007468.1	0	0	0	0	0.42	0
GAIF01007469.1	0.13	0.07	0.14	0.13	0.03	0
GAIF01007470.1	0	0	0	0	0.12	0
GAIF01007471.1	6.54	5.31	4.96	13.94	15.91	12.03
GAIF01007472.1	0	0	0.13	0	0.34	0
GAIF01007473.1	0.31	0	0	0.59	0.16	0.34
GAIF01007474.1	28.52	32.95	49.98	108.85	63.49	21
GAIF01007475.1	0.74	0.4	0.65	1.09	1.19	0.25
GAIF01007478.1	244.39	467.8	167.25	444.48	448.26	47.3
GAIF01007479.1	5.44	3.34	1.61	6.29	6.38	5.25
GAIF01007480.1	553.78	419.45	164.36	76.44	216.91	307.79
GAIF01007482.1	0	0.05	0	0	0	0
GAIF01007483.1	45.11	72.65	45.99	84.37	96.53	91.12
GAIF01007484.1	52.92	44.16	48.88	95.19	47.79	120.15
GAIF01007485.1	5.07	5.65	5.61	6.09	5.72	1.81
GAIF01007487.1	103.79	75.1	169.76	270.84	111.97	254.95
GAIF01007489.1	5.77	10.53	4.95	2.66	0.89	0.16
GAIF01007490.1	0.9	0.74	0	0.69	1.81	0
GAIF01007491.1	22.86	23.96	23.15	55.9	39.27	12.29
GAIF01007492.1	17.32	27.1	4.78	14.22	64.91	4.32
GAIF01007493.1	0.13	0	0	0.12	0	0
GAIF01007495.1	0.08	0.04	0	0	0	0
GAIF01007496.1	1.23	3.24	1.35	1.41	1.93	0.9
GAIF01007497.1	0.26	0	0.09	0	0.08	0.5
GAIF01007498.1	31.48	20.82	13.43	62.44	44.6	7.72
GAIF01007501.1	0	0	0	0.05	0	0
GAIF01007503.1	0.42	0	0.21	0.77	0.52	0.52
GAIF01007505.1	1.22	1.2	1.37	6.29	13.18	0.75
GAIF01007506.1	67.06	93.64	59.13	165.44	185.63	20.77
GAIF01007508.1	43.44	61.45	109.71	171.13	182.76	33.43
GAIF01007509.1	5.02	4.26	4.65	2.55	2.26	0.17
GAIF01007510.1	12.19	9.19	8.21	18.81	10.98	4.59

GAIF01007511.1	5.34	10.42	10.57	9.76	0	1.66
GAIF01007512.1	0	0	0	0.1	1.08	0.36
GAIF01007515.1	0	1.13	0	0.01	29.75	3.83
GAIF01007518.1	18.67	12.59	3.82	11.38	20.8	0
GAIF01007519.1	1.26	1.55	1.12	0.51	0.4	1.05
GAIF01007520.1	8.83	3.58	5.02	29.62	20.08	13.71
GAIF01007521.1	0.33	0	0.02	0.87	0.62	0.17
GAIF01007522.1	0.13	0.07	0.07	0.08	0.08	0.15
GAIF01007523.1	0.34	0.36	0.22	2.57	7.66	0.07
GAIF01007524.1	0.51	0.5	0.1	0.21	0	0
GAIF01007527.1	23.23	30.11	71.33	0	14.54	21.97
GAIF01007528.1	0.22	0.18	0	1.11	2.56	0.12
GAIF01007529.1	0	0	0	0.07	0.08	0
GAIF01007530.1	0.66	1.12	0.18	0.19	0	0.04
GAIF01007531.1	0.25	0.05	0.14	1.08	0.15	0.19
GAIF01007534.1	0	0.05	0.05	0.16	0.27	0.05
GAIF01007536.1	0	0.65	0	0	0	0.27
GAIF01007537.1	0	0.03	0	0	0	0
GAIF01007538.1	32.21	33.29	32.65	43.61	17.45	13.43
GAIF01007539.1	5.63	9.94	6.18	38.9	40.17	3.75
GAIF01007540.1	0.03	0.03	0	0	0	0
GAIF01007542.1	195.16	135.63	292.84	84.22	24.96	173.62
GAIF01007543.1	0	0.16	0.16	0	0	0
GAIF01007544.1	0.19	0.1	0.16	0.6	3.41	0.21
GAIF01007545.1	75.2	31.53	18.63	26.04	19.43	1.64
GAIF01007546.1	0.33	0.24	0.24	0	0.26	0
GAIF01007547.1	3.14	5.42	2.3	3.65	2.15	0.88
GAIF01007548.1	13.93	12.42	2.42	7.55	10.95	0
GAIF01007549.1	10.31	8.76	9.25	53.32	23.57	4.89
GAIF01007550.1	0	0	0.47	0	0.54	0.25
GAIF01007551.1	0.48	0.15	0.15	2.82	4.91	0.2
GAIF01007552.1	2.48	3.23	4.1	9.53	7.33	3.44
GAIF01007553.1	0.57	0	0	0	0	0
GAIF01007554.1	0.54	0.07	0.15	1.64	7.04	3.11
GAIF01007556.1	0	0	0	0.28	0	0
GAIF01007558.1	0	0	0.1	0	0.05	0.19
GAIF01007561.1	0	0.17	0.26	0.28	1.15	0.09
GAIF01007562.1	3.69	1.05	0.5	14.23	17.94	1.59
GAIF01007563.1	0.37	0.2	0.2	0.53	0	0.1
GAIF01007564.1	0	0	0	0.21	0.64	0
GAIF01007565.1	9.35	24.48	21.54	30.67	32	3.66
GAIF01007566.1	4.19	3.52	0.91	2.48	1.68	0
GAIF01007567.1	3.97	6.18	1.16	34.54	55.36	0.87
GAIF01007569.1	0.68	0	0.13	1.16	7.34	2.02

GAIF01007570.1	0.67	0	0	0.78	3.37	0
GAIF01007573.1	0	0.28	0	0.5	0.9	0.12
GAIF01007574.1	0	1.68	2.51	0	0	0.5
GAIF01007575.1	26.22	19.74	6.64	0	0	0
GAIF01007577.1	1837.76	1829.31	508.41	1798.84	3168.28	534.43
GAIF01007578.1	37.09	18.36	18.6	63.54	80.61	66.92
GAIF01007580.1	0	0	0.4	0	0	0
GAIF01007582.1	1.32	2.89	5.22	2.26	2.3	8.23
GAIF01007583.1	129.33	84.01	47.35	241.32	453.14	47.79
GAIF01007584.1	0	0	0	0	0.29	0
GAIF01007585.1	13.58	11.03	16.29	44.83	24.74	19.2
GAIF01007586.1	12.67	16.42	18.06	20.81	10.05	13.37
GAIF01007587.1	1.73	0.97	0.53	6.57	7.38	0.67
GAIF01007589.1	0.53	0.32	0.38	2.84	24.31	40.42
GAIF01007591.1	0.41	0.26	0	5.84	29.24	0.13
GAIF01007592.1	4.2	5.68	4.61	4.57	6.75	0.94
GAIF01007593.1	0	0	0	0.46	0.35	0
GAIF01007594.1	0	0	0	0	0.07	0
GAIF01007595.1	0.1	0.75	0.16	0	0	0
GAIF01007596.1	0	0	0.04	0.05	0	0.09
GAIF01007598.1	0.59	0	0	0	0	0
GAIF01007599.1	0	0.51	0	0.22	0.11	0
GAIF01007600.1	0.16	0	0	0.16	1.77	0
GAIF01007601.1	1.96	0.45	0.06	12.78	32.64	0.51
GAIF01007602.1	0.08	0.13	0.09	0.19	0.84	0.02
GAIF01007605.1	0.24	0	0	0.56	3.21	0.09
GAIF01007606.1	32.7	30.11	30.23	30.63	16.08	4.61
GAIF01007607.1	0.14	0.22	0.15	0.16	0	0
GAIF01007609.1	0.99	0.64	0	15.62	27.41	0
GAIF01007610.1	37.67	25.79	36.18	66.69	36.18	26.62
GAIF01007611.1	0	0	0.17	0	0.16	0
GAIF01007613.1	0	0	0.12	0.06	0.57	0
GAIF01007614.1	0	0	0	0	0.01	0
GAIF01007615.1	0	0.16	0	0	0	0
GAIF01007616.1	0.54	0.2	0	0.6	0.58	0
GAIF01007617.1	0	0.24	0	0	0.41	0.08
GAIF01007618.1	1.65	0.04	0.08	0.57	2.08	0.21
GAIF01007619.1	2.7	0.31	0	5.03	9.5	1.06
GAIF01007620.1	0.06	0.06	0.03	0	0.14	0
GAIF01007621.1	0	0.01	0.04	0.03	0.08	0.01
GAIF01007623.1	2.78	4.26	2.69	3.68	4.8	1.16
GAIF01007626.1	14.07	7.98	10.99	45.67	95.63	6.65
GAIF01007627.1	0	0	0	0	0.71	0
GAIF01007628.1	11.14	6.91	9.6	1.74	0.58	2.71

GAIF01007629.1	0	0	0	0.44	0.92	0
GAIF01007630.1	1.2	0.36	0	4.8	4.98	0.12
GAIF01007632.1	9.09	13.14	12.06	23.59	28.54	3.86
GAIF01007633.1	54.89	10.16	39.75	60.35	25.99	20.28
GAIF01007634.1	2.84	1.22	0.15	0	0	0
GAIF01007635.1	0	0	0	0.11	0.75	0
GAIF01007637.1	0	0	0	0	0.25	0
GAIF01007638.1	0	0	0	0	1.54	0
GAIF01007640.1	49.63	21.29	2.99	20.38	29.35	0.7
GAIF01007641.1	0.29	0.07	0	0.29	0.98	0
GAIF01007642.1	5.55	4.38	0.7	12.13	17.76	9.84
GAIF01007643.1	0.12	0.18	0.11	0.18	0.82	0
GAIF01007644.1	0.06	0.33	0.41	0	0	0.14
GAIF01007645.1	0	0	0.11	0.12	0	0
GAIF01007646.1	0.13	0.07	0.07	0.07	0	0
GAIF01007647.1	0	0	0.15	0	0	0
GAIF01007648.1	12.24	18.63	18.5	21.39	13.77	6.98
GAIF01007650.1	0.06	0.14	0	0.14	0	0
GAIF01007653.1	0.37	0.2	0	1.66	6.85	0.59
GAIF01007654.1	0.09	0	0	0.33	1.74	0.21
GAIF01007656.1	0.76	0	0	2.2	8.47	0
GAIF01007657.1	0	0	0	0	0	0.15
GAIF01007658.1	0.03	0	0	0	0	0
GAIF01007659.1	0	0.08	0	0	0	0
GAIF01007660.1	0	0	0	0	0.07	0
GAIF01007661.1	0.15	0.55	0.67	0.53	0.43	0.17
GAIF01007662.1	505.11	208.19	143.04	518.47	271.8	550.46
GAIF01007663.1	106.72	55.84	70.12	47.71	67.44	69.34
GAIF01007664.1	0.13	0.07	0	0.08	0.64	0
GAIF01007666.1	8.02	2.63	1.11	0	0	0
GAIF01007667.1	0	0	0	0.65	2.02	0
GAIF01007668.1	0.02	0	0	0.17	0	0
GAIF01007670.1	47.56	54.56	63.67	81.34	55.29	45.53
GAIF01007671.1	5.48	1.02	0.26	12.94	24.04	4.4
GAIF01007672.1	10.09	9.57	3.16	9.83	10.96	8.73
GAIF01007673.1	0	0	0	0.12	0.48	0
GAIF01007674.1	0	0	0	0.47	3.08	0
GAIF01007675.1	99.84	142.07	132.33	213.22	228.13	307.43
GAIF01007676.1	0.01	0.01	0	0.02	0.15	0
GAIF01007677.1	11.4	5.46	12.09	0.45	0.46	1.06
GAIF01007679.1	8.69	2.04	4	8.32	4.66	5.31
GAIF01007680.1	332.53	197.05	174.08	86.83	72.16	22.06
GAIF01007681.1	0.34	0.09	0	0.15	1.76	0.02
GAIF01007682.1	23.18	0	3.06	11.92	1.59	19.73

GAIF01007683.1	3.47	3.42	2.26	14.1	16.31	1.32
GAIF01007684.1	67.49	83.66	69.86	153.19	91.7	69.66
GAIF01007685.1	46.1	59.57	44.73	65.42	33.73	14.81
GAIF01007687.1	55.21	74.41	49.51	51.7	36.82	11.31
GAIF01007688.1	0	0	0	0.32	2.03	0.17
GAIF01007689.1	30.28	26.45	21.31	7.99	9.02	7.35
GAIF01007690.1	1.23	0	0.83	0.93	2.47	0.23
GAIF01007692.1	4.23	5.04	4.06	2.99	2.01	0.85
GAIF01007693.1	1.32	0.45	0.08	4.9	8.08	14.42
GAIF01007695.1	0	0	0	0.09	0.45	0
GAIF01007696.1	1.18	3.08	0.99	1.44	1.08	0.08
GAIF01007697.1	0	0.37	0	0.39	1.63	0
GAIF01007698.1	0	0	0	0	0.28	0
GAIF01007700.1	0	0	0	0	1.31	0
GAIF01007701.1	13.19	11.09	9.18	22.95	20.41	10.97
GAIF01007703.1	61.66	64.91	96.62	103.44	47.69	76.45
GAIF01007704.1	0	0	0	0.27	1.03	0
GAIF01007706.1	1.37	0.64	2.1	0.2	0	0.51
GAIF01007707.1	0.92	0.27	1.37	0.58	2.31	1.2
GAIF01007709.1	2.87	7.05	3.38	8.89	10.34	0.39
GAIF01007712.1	0.04	0	0	0	0.09	0
GAIF01007713.1	0	6.32	9.44	0	0	0.38
GAIF01007714.1	0.58	0	0	0	0.84	0
GAIF01007715.1	19.05	10.41	11.35	1.27	4.5	1.2
GAIF01007716.1	0	0	0	0.21	0.45	0.1
GAIF01007718.1	0	0.21	0.32	0	0	0
GAIF01007719.1	0.96	0	0	3.34	9.68	1.09
GAIF01007720.1	0	0	0	0.01	0	0
GAIF01007721.1	0.06	0.09	0.18	0.06	0.2	0.04
GAIF01007722.1	0.02	0	0	0	0.02	0
GAIF01007725.1	2.17	2.36	1.39	8.17	6.95	0.6
GAIF01007726.1	0.87	0.54	0	7.92	21.05	0
GAIF01007727.1	0.19	0.05	0.05	0.03	0	0.03
GAIF01007728.1	27.46	19.84	11.3	18.33	16.95	6.76
GAIF01007729.1	0	0	0.06	0.06	0	0.06
GAIF01007731.1	0.79	0.34	0.78	0.28	0	0.26
GAIF01007732.1	1.01	0	0	6.86	29.66	0.56
GAIF01007734.1	13.74	6.96	10.21	11.48	11.5	46.84
GAIF01007735.1	0.02	0	0.08	0.14	0.32	0.03
GAIF01007736.1	0.03	3.31	1.19	0.16	0	0.37
GAIF01007738.1	0.77	0	0	0.1	0	0
GAIF01007739.1	2.74	2.75	3.36	6.12	6.59	1.28
GAIF01007740.1	0	0.28	0.14	0.88	0.76	0.84
GAIF01007742.1	200.04	168.78	124.22	42.05	49.4	54.39

GAIF01007743.1	4.78	7.79	3.03	5.29	4.22	0.74
GAIF01007744.1	104.39	47.18	55.03	43.99	33.71	52.39
GAIF01007746.1	0	0	0	0	0.31	0
GAIF01007747.1	0.37	0	0.31	1.78	11.32	0.46
GAIF01007750.1	0	0.15	0	0	0	0
GAIF01007751.1	0	0	0	0	0.05	0
GAIF01007752.1	0.1	0.24	0.04	0	0.04	0
GAIF01007754.1	3.66	4.42	0.17	6.02	17.94	0
GAIF01007755.1	18.84	24.68	35.67	74.04	63.7	18.31
GAIF01007756.1	0	0	0.23	0.73	0	0
GAIF01007757.1	0	0.07	0	0	0.66	0
GAIF01007758.1	37.9	34.8	44.24	72.21	61.44	115.18
GAIF01007759.1	3.62	4.7	1.66	5.14	3.99	0.11
GAIF01007763.1	0	0	0	0.65	3.41	0
GAIF01007764.1	5.22	3.45	2.22	5.85	2.97	0.66
GAIF01007766.1	189.77	262.28	360.86	758.44	461.28	278.65
GAIF01007767.1	0.04	0	0.05	0	0	0
GAIF01007768.1	0.13	0	0.28	0.29	0	0
GAIF01007769.1	0.03	0.07	0	0.03	0.04	0
GAIF01007770.1	0.54	0.18	0.23	0.59	0.8	0.34
GAIF01007771.1	0	0	0	0	0.05	0
GAIF01007772.1	0	0	0	0.07	0.08	0
GAIF01007773.1	0.06	0.06	0.06	0.45	2.45	0.15
GAIF01007774.1	2.09	0.82	0.42	6.9	18.48	5.64
GAIF01007775.1	0	0	0.19	0	0	0
GAIF01007776.1	26.22	0	10.49	0	37.38	0
GAIF01007777.1	5.22	5.6	3.44	4.65	4.51	0.17
GAIF01007779.1	0	0	2.08	4.35	0.08	0
GAIF01007780.1	0	0	0	0	0.09	0
GAIF01007781.1	34.65	38.01	39.8	20.18	15.18	5.19
GAIF01007782.1	330.23	609.79	685.54	261.26	231.05	82.8
GAIF01007783.1	0	0	0	0	0.17	0
GAIF01007784.1	25.48	18.58	13.72	75.03	108.73	8.03
GAIF01007785.1	0	0.09	1.03	0	0.1	1.13
GAIF01007787.1	0	2.65	14.79	12.99	0	11.93
GAIF01007788.1	15.87	5.61	3.22	4.37	9.36	1.9
GAIF01007789.1	0	0.13	0	0.08	0	0
GAIF01007790.1	0.01	0	0	0	0	0.05
GAIF01007791.1	0.18	0.1	0	0.62	1.17	0
GAIF01007792.1	1.05	0.32	0	0.34	0	0
GAIF01007794.1	0.52	0.28	0	0.3	2.04	0.57
GAIF01007796.1	4.84	6.93	5.93	1.12	0.06	0.11
GAIF01007797.1	53.75	95.69	99.8	144.21	133.05	20.91
GAIF01007798.1	5.97	3.01	1.42	7.79	16.43	3.05

GAIF01007799.1	0.91	0	0	0	0.13	0.13
GAIF01007800.1	10.8	9.56	8.36	43.33	50.94	9.6
GAIF01007801.1	0	0	0	0.3	1.87	0
GAIF01007802.1	1777.7	1377.29	1715.53	1133.27	784.23	1933.12
GAIF01007804.1	0	0.12	0	0.26	0.54	0
GAIF01007805.1	0	0.07	0	0.07	0.07	0
GAIF01007809.1	0.86	0.23	0	4.47	1.03	0.47
GAIF01007811.1	20.65	16.61	17.77	26.68	23.13	8.67
GAIF01007812.1	0	0	0	0	0.04	0
GAIF01007813.1	215.14	163.34	85.23	122.79	89.21	27.46
GAIF01007815.1	0.03	0	0	0.04	0.53	0.08
GAIF01007816.1	0.12	1.21	0.32	0.23	0	0.27
GAIF01007817.1	52.45	0	0	0	0	0
GAIF01007819.1	2.34	0.27	0	4.62	11.47	0.97
GAIF01007820.1	1.78	1.22	0.72	3.21	3.71	1.63
GAIF01007821.1	0.1	0.04	0	0.04	0.08	0.02
GAIF01007822.1	0	0.26	0.33	0	0	0.07
GAIF01007823.1	0	0	0	0.1	0.1	0
GAIF01007825.1	8.4	15.51	5.55	46.02	30.53	2.45
GAIF01007826.1	3.98	4.72	3.72	2.65	1.24	1.62
GAIF01007827.1	0	0.08	0	0.08	0.83	0
GAIF01007828.1	0	0.13	0.2	0.49	1.64	0.07
GAIF01007829.1	3.61	0.89	0.43	9.15	21.39	8.65
GAIF01007831.1	1.18	1.86	0.08	5.18	16.85	0.12
GAIF01007832.1	1.78	0.24	0	2.19	6.15	2.33
GAIF01007834.1	0	0	0	0.06	0.04	0
GAIF01007836.1	0	0	0	0.07	0.07	0
GAIF01007837.1	0	0.4	0.67	0.42	0.15	0
GAIF01007838.1	0.13	0.1	0.29	0.54	0.22	0
GAIF01007840.1	4.77	3.15	3.49	10.91	4.35	13.39
GAIF01007841.1	14.9	7.56	10.31	19.52	27.03	94.72
GAIF01007842.1	0	0	0	0	0.02	0
GAIF01007843.1	2.58	0.92	0.31	6.98	19.12	5.57
GAIF01007846.1	7.77	7.42	8.36	32.13	37.3	4.81
GAIF01007847.1	3.63	1.63	1.09	14.58	26.03	0.42
GAIF01007848.1	0.48	0.22	0.07	9.72	23.49	0.23
GAIF01007849.1	0	0	0	0.2	0.46	0
GAIF01007850.1	1.7	0.84	0.17	0.64	0.46	0
GAIF01007851.1	0.48	0	0	2.14	8.51	0
GAIF01007853.1	0.28	0.1	0.42	0.23	0.34	0
GAIF01007854.1	0.22	0.08	0	0.17	0	0.16
GAIF01007855.1	11.26	6.13	3.56	17.44	25.15	7.42
GAIF01007857.1	35.53	37.65	38.08	40.13	31.28	17.39
GAIF01007858.1	0	0	0	0	0.09	0

GAIF01007860.1	0	0.04	0.19	0	0.01	0.02
GAIF01007861.1	0	0.21	0	0	0	0
GAIF01007862.1	0	0.18	0	0	0	0
GAIF01007863.1	0	0	0	0.39	0.2	0
GAIF01007864.1	0	0	0	0	0.5	0
GAIF01007865.1	3.13	0.38	0	1.62	3	1.78
GAIF01007867.1	12.92	12.77	22.48	22.79	11.3	17.76
GAIF01007869.1	0	0	0	0	0	0.21
GAIF01007871.1	0	0	0	0	0.09	0
GAIF01007873.1	46.89	39.01	22.57	75.67	76.94	45.6
GAIF01007874.1	0	0.32	0.34	2.9	15.95	0.55
GAIF01007875.1	1.22	0.83	0.34	5.21	12.64	2.03
GAIF01007876.1	20.52	12.27	3.9	83.57	96.8	4.25
GAIF01007877.1	0	0.08	0.16	0.08	0.08	0
GAIF01007878.1	0	0	0	0.26	0.55	0
GAIF01007879.1	1.45	0.07	0.01	0.2	1.01	0.09
GAIF01007881.1	6.61	3.33	0	8.67	5.87	0
GAIF01007882.1	0	0	0	0	0.85	0
GAIF01007883.1	0	0	0	0.14	0.09	0
GAIF01007885.1	0	0	0.05	0	0.06	0.05
GAIF01007886.1	0.32	0.34	0	0.61	1.26	0
GAIF01007888.1	0.13	0	0	1.24	1.18	0.63
GAIF01007889.1	114.05	294.15	928.13	71.14	34.58	104.21
GAIF01007890.1	0.84	0.36	0.12	2.49	14.77	0
GAIF01007892.1	43.89	77.29	61.05	125.97	153.52	20.8
GAIF01007893.1	0.14	0	0	0.16	0	0
GAIF01007894.1	0.03	0.03	0	0.41	0	0
GAIF01007895.1	4.56	2	0.32	16.33	34.78	30.16
GAIF01007896.1	1.25	1.36	5.32	1.04	1.72	3.77
GAIF01007897.1	1.66	0.21	0.31	1.74	1.23	0.31
GAIF01007898.1	0.76	1.02	0.37	4.93	13.73	0.05
GAIF01007899.1	30.96	38.58	8.36	165.86	100.6	3.53
GAIF01007900.1	0.61	0.91	0.07	2.54	10.84	0.04
GAIF01007901.1	0.06	0	0	0.07	0.07	0
GAIF01007903.1	0	0	0.04	0	0	0
GAIF01007904.1	0	0.17	1.05	0	0	0.18
GAIF01007905.1	2.99	4.33	2.55	5.01	19.73	5.12
GAIF01007906.1	45.41	126.6	47.68	53.37	241.49	6.65
GAIF01007909.1	0.34	0.73	0.12	0	0.54	0
GAIF01007910.1	0.28	0	0	1.04	4.75	0
GAIF01007911.1	5.37	8.76	3.98	4.64	5.18	0.62
GAIF01007912.1	0.47	0.77	3.04	1.84	1.22	1.57
GAIF01007913.1	31.23	11.7	18	19.72	13.47	7.84
GAIF01007914.1	80.28	99.2	76.07	403.59	324.32	69.87

GAIF01007915.1	1.87	1.07	0	8.02	15.8	1.65
GAIF01007916.1	0	0	0	0.07	0	0
GAIF01007917.1	35.74	13.09	18.32	5.22	3.24	6.7
GAIF01007918.1	1.01	1.41	0	5.02	9.89	0.16
GAIF01007919.1	9.53	8.12	0.48	22.53	46.55	2.9
GAIF01007920.1	2.17	3.05	0.76	2.81	6.88	0.75
GAIF01007921.1	0.08	0.09	0	0.92	1.52	0
GAIF01007923.1	0	0.37	0	0.11	0.3	0.14
GAIF01007924.1	74.2	109.74	94.46	75.27	45.88	25.64
GAIF01007925.1	0	0	0	1.22	9.72	0
GAIF01007926.1	0.58	0.47	0.16	1.51	5.03	0.16
GAIF01007927.1	0.02	0	0	0.11	0.5	0
GAIF01007929.1	0	0	0	0.16	0.17	0
GAIF01007930.1	0.47	0.18	0.03	1.93	11.18	0.21
GAIF01007932.1	0	0	0	1.3	0	0
GAIF01007934.1	20.08	31.79	15.76	17.89	14.62	1.84
GAIF01007935.1	6.82	6.33	2.19	20.07	28.39	3.66
GAIF01007936.1	0.6	0.42	0.14	2.8	0.9	0.29
GAIF01007937.1	35.95	28.6	12.87	97.48	104.9	169.13
GAIF01007938.1	0.36	0.1	0.2	0.14	0.7	0
GAIF01007939.1	39.98	43.45	53.11	83.53	69.74	54.49
GAIF01007940.1	2.44	0.22	0	1.11	2.98	0
GAIF01007941.1	0.45	0.34	0.25	1.01	0.54	0.17
GAIF01007942.1	0	0.7	0.77	0	0	0.13
GAIF01007944.1	0	0	0	5.38	0	0.12
GAIF01007945.1	98.67	4.8	12.74	5	0.82	1.83
GAIF01007946.1	2.64	0.5	0.2	8.88	53.92	0.5
GAIF01007948.1	2.1	0.33	0.13	1.84	1.98	0
GAIF01007949.1	0	0	0	0	0.04	0
GAIF01007951.1	0.73	0	1.6	5.92	22.78	30.51
GAIF01007952.1	7592.61	7550.1	12677.15	4146.38	2770.75	7090.38
GAIF01007954.1	108.62	54.48	59.41	80.02	105.47	83.51
GAIF01007957.1	0	0	0.11	0.06	0	0
GAIF01007958.1	0	0.07	0.07	0	0	0
GAIF01007959.1	168.07	112	152.87	209.8	287.78	256.78
GAIF01007960.1	0	0.14	0	0.48	2.91	0.73
GAIF01007961.1	0	0	0	0.33	2.77	0
GAIF01007965.1	0.77	2.1	0	3.4	9.55	0.08
GAIF01007967.1	0.21	0	0.04	0.37	0.02	0.25
GAIF01007968.1	0	0	0.28	0	0	0
GAIF01007969.1	12.21	13.02	9.25	38.21	47.32	12.56
GAIF01007972.1	215.73	246.91	179.79	173.54	137.91	29.94
GAIF01007973.1	5.02	0.93	2.64	3.97	9.39	3.67
GAIF01007974.1	6.84	10.69	4.51	3.32	4.04	0.14

GAIF01007976.1	0	0	0	0.06	0.1	0
GAIF01007977.1	0	0	0	0	0.23	0
GAIF01007978.1	26.68	21.66	47.27	100.54	85.65	41.37
GAIF01007979.1	0.03	0.03	0	0.26	1.56	0.06
GAIF01007980.1	0	0	0	0.04	0	0
GAIF01007983.1	0	0.03	0.03	0.31	0.82	0
GAIF01007984.1	0	0.04	0	0.04	0.04	0
GAIF01007985.1	57.27	55.12	26.22	40.62	47.17	4.51
GAIF01007986.1	3.34	1.19	0.15	7.17	13.22	2.95
GAIF01007990.1	13.92	18.27	10.18	20.35	42.4	3.19
GAIF01007992.1	23.92	23.07	18.37	76.15	56.24	21.27
GAIF01007996.1	1.26	0.73	0	0.98	4.19	0
GAIF01007998.1	0	0	0	0	0.26	0
GAIF01007999.1	10.54	4.8	3.29	9.12	6.9	1.76
GAIF01008000.1	595.58	137.93	209.74	488.48	137.97	351.68
GAIF01008002.1	0	0	17.39	0.73	0	0
GAIF01008003.1	4.99	0.73	0.3	11.9	40.93	3.47
GAIF01008004.1	70.91	43.24	33.68	104.2	88.47	30.58
GAIF01008005.1	12.35	13.06	4.9	0.13	0	0
GAIF01008006.1	0.1	0.08	0.11	0.27	0.7	0
GAIF01008007.1	0.12	0	0.23	0.17	0.1	0
GAIF01008010.1	1.85	1.38	1.24	3.16	6.43	0.41
GAIF01008011.1	0.13	0.17	0.45	0.22	0	0.1
GAIF01008012.1	2.74	3.05	1.6	2.9	3.71	0.96
GAIF01008014.1	0	0.03	0	0	0	0
GAIF01008015.1	0	0.06	0	0	0.06	0
GAIF01008016.1	1.41	1.25	0.89	6.18	5.78	0.57
GAIF01008017.1	0.25	0	0.05	0.14	0	0
GAIF01008018.1	2.29	3.59	0.56	5.6	2.14	0
GAIF01008019.1	1.4	0.77	0.27	0.64	3.88	0.14
GAIF01008021.1	1.46	0.88	0.63	2.33	3.83	0.09
GAIF01008022.1	20.99	26.15	49.99	97.36	111.84	23.95
GAIF01008024.1	0.12	0.04	0	0.71	8.03	0.14
GAIF01008026.1	0	0.2	0	0	0	0
GAIF01008027.1	8.99	1.18	0.93	6.49	17.88	8.03
GAIF01008029.1	4.57	9.19	2.47	11.54	11.44	0.62
GAIF01008030.1	0.11	0.13	0.12	0.26	0	0
GAIF01008031.1	0.2	0.42	0.32	0.68	1.41	0.11
GAIF01008032.1	0.08	0	0	0	1.78	0
GAIF01008033.1	1.16	0.52	0.08	1.79	6.6	0.25
GAIF01008034.1	51.72	38.65	32.38	61.78	49.89	51.55
GAIF01008035.1	0.41	0	0	1.21	4.23	0
GAIF01008036.1	0	0.18	0.04	0.13	0.69	0.16
GAIF01008037.1	0.1	0	0.23	0.48	0.5	0

GAIF01008038.1	0	0.17	0	0	0	0
GAIF01008040.1	3.53	3.19	4.23	11.15	8.59	1.79
GAIF01008042.1	13.55	0	0	0	17.98	1.08
GAIF01008043.1	0.04	0.55	0.44	4.08	1.27	0.62
GAIF01008045.1	0	0	0	1.6	0	0
GAIF01008046.1	0.05	0.08	0.08	0.08	0.25	0
GAIF01008047.1	25.77	30.4	31.92	31.62	28.6	10.92
GAIF01008049.1	1.34	1.69	1.94	1.11	0.56	0.94
GAIF01008051.1	0	0	0	0	0.13	0
GAIF01008053.1	1.01	0	0	3.53	18.49	0
GAIF01008055.1	14.94	21.25	21.73	49.65	22.78	9.31
GAIF01008056.1	4.99	29.32	9.39	51.88	70.67	16.14
GAIF01008058.1	58.01	44.11	26.35	14.02	14.1	2.67
GAIF01008059.1	0.12	0	0.05	0	0.31	0
GAIF01008061.1	0.35	0.19	0	2.05	2.76	0
GAIF01008062.1	0.68	0	0	1.59	3.12	0.3
GAIF01008063.1	8.48	9.28	12.42	24.8	12.49	5.51
GAIF01008064.1	0	0	0	0.71	4.41	0.34
GAIF01008065.1	0	0.04	0	0	0	0
GAIF01008067.1	0	0.03	0	0.04	0	0
GAIF01008068.1	0.02	0	0	0	0.1	0
GAIF01008069.1	2.94	5.47	2.89	16.48	11.89	0.53
GAIF01008071.1	0	0	0	0	11.55	0
GAIF01008072.1	1.1	0.26	0.44	0.3	0.16	0.04
GAIF01008074.1	0	0.4	0	0	5.44	0
GAIF01008076.1	21.64	11.29	17.18	7.71	7.9	5.59
GAIF01008079.1	0.24	0.39	0	1.24	6.13	0.13
GAIF01008081.1	101.7	44.87	48.24	61.5	59.63	76.24
GAIF01008082.1	0	0	0	0	0.15	0.28
GAIF01008083.1	5.94	4.21	3.37	19.54	18.45	4.3
GAIF01008084.1	22.38	17.71	32.02	67.54	56.94	29.75
GAIF01008085.1	0.1	0	0	0.61	3.02	0.23
GAIF01008087.1	0	0	0	0	0.52	0
GAIF01008088.1	0.43	0	0	0.13	0	0
GAIF01008090.1	0.33	0	0.06	0	0	0
GAIF01008092.1	0.02	0.05	0.08	0.03	0	0
GAIF01008093.1	11.41	6.1	8.67	25.5	8.81	2.41
GAIF01008094.1	35.7	8.24	12.98	12.65	19.87	38.4
GAIF01008095.1	0	0.09	0.2	1.6	4.76	0.38
GAIF01008096.1	2.28	0.26	0	8.77	33.01	0.4
GAIF01008097.1	935.14	46.58	60.6	876.62	1631.23	6389.96
GAIF01008099.1	0	0	0	0.27	2.06	0.1
GAIF01008100.1	10.23	11.35	9.42	20.9	12.67	1.66
GAIF01008101.1	0	5.23	0.1	0	0	0.1

GAIF01008102.1	12.69	8	4.55	24.17	24.61	2.85
GAIF01008103.1	266.57	946.84	491.28	32.76	33.3	16.97
GAIF01008104.1	0	0	0	0	0.15	0
GAIF01008105.1	0	0	0	0.28	2.21	0
GAIF01008106.1	11.53	21.72	17.17	46.25	25.28	9.13
GAIF01008107.1	3.52	5.6	0.76	10.81	28.24	0
GAIF01008108.1	0	0	0	0	0.04	0
GAIF01008109.1	9.18	1.7	1.07	16.1	37.35	3.01
GAIF01008110.1	0	0.12	0	0.27	1.38	0
GAIF01008111.1	0.18	0	0	0.13	0.05	0
GAIF01008112.1	0.11	0.36	0.48	1.41	0.93	0
GAIF01008113.1	6.4	5.05	1.67	7.89	17.42	0.09
GAIF01008114.1	10.59	8.15	4.99	15.75	26.76	7.25
GAIF01008115.1	4.34	7.34	4.76	2.18	2.9	0.06
GAIF01008116.1	36.56	60.05	59.49	52.57	35.36	17.08
GAIF01008117.1	9.28	5.88	6.49	17.26	23.16	17.31
GAIF01008118.1	5.18	7.8	8.3	12.19	8.08	1.03
GAIF01008120.1	69.78	36.68	40.2	19.09	21.38	9.86
GAIF01008121.1	0.43	0.04	0.12	0.15	0.12	0.1
GAIF01008123.1	0	0	0	0	0.26	0
GAIF01008124.1	92.77	125.97	34.23	80.14	69.72	4.14
GAIF01008126.1	0.06	0	0.07	0	0	0
GAIF01008127.1	0.33	0.09	0	0.1	0	0
GAIF01008128.1	2.86	2.92	0.95	11.48	23.06	0.86
GAIF01008129.1	0.37	0.16	0	1.66	6.36	0.75
GAIF01008130.1	3.85	4.19	6.13	0.22	0	0
GAIF01008131.1	2.93	2.07	0	5.94	18.83	0.16
GAIF01008132.1	4.54	1.29	0.47	17.93	83.66	12.02
GAIF01008133.1	1004.07	420.16	529.22	1156.53	525.74	2098.34
GAIF01008134.1	2.25	2.99	5.49	4.64	1.65	1.24
GAIF01008135.1	18.25	13.01	10.47	57.11	68.48	8.57
GAIF01008138.1	0	0	0	0.21	0.87	0
GAIF01008139.1	4.17	2.49	5.58	0.45	1.24	0.97
GAIF01008140.1	129.08	61.4	53.25	14.11	30.54	10.44
GAIF01008142.1	0	0.07	0	0	0	0
GAIF01008143.1	2.21	0.25	0	12.89	68.89	0.92
GAIF01008144.1	189.58	126.18	95.25	191.51	223.91	85.03
GAIF01008145.1	6.22	2.91	3.89	4.58	3.82	6.42
GAIF01008146.1	8.27	11.91	2.45	30.9	44.1	4.01
GAIF01008147.1	3.02	1.64	0	18.86	29.78	0
GAIF01008149.1	0	0	0	0	5.24	0
GAIF01008153.1	4.98	7.13	0	58.33	103.92	0
GAIF01008154.1	0.04	0.05	0	0.88	8.32	0
GAIF01008155.1	0.28	0.36	0.31	0.71	0.34	0.26

GAIF01008156.1	30.87	22.52	15.36	32.99	43.17	16.66
GAIF01008157.1	0.36	0	0	0.24	0	0.06
GAIF01008158.1	0	0.3	0.25	0	0.17	0
GAIF01008159.1	0	0	0	0.18	0.68	0
GAIF01008160.1	0.12	0	0	1.03	4.3	0.04
GAIF01008161.1	0.15	0	0	0.28	0.06	0
GAIF01008162.1	220.17	39.99	3.92	533.81	1324.4	26.75
GAIF01008167.1	2.93	3.61	3.43	1.81	3.05	2.8
GAIF01008168.1	19.29	17.61	13.23	23.05	24.52	32.42
GAIF01008170.1	0	0.32	0	1.48	13.43	0.32
GAIF01008171.1	0.13	0.04	0.04	0	5.28	0.2
GAIF01008172.1	0	0	0	0	0.62	0
GAIF01008174.1	0	0	0	0	0	0.17
GAIF01008175.1	2.85	3.95	5.77	2.26	1.33	1
GAIF01008177.1	27.39	27.44	6.45	34.4	62.16	44.77
GAIF01008178.1	0.59	0	0.13	1.78	13.73	0.65
GAIF01008182.1	0.83	1.29	0.78	1.51	1.56	0.65
GAIF01008184.1	11.07	22.68	10.86	37.43	44.01	3.26
GAIF01008185.1	0	0.48	0	0.67	0	0
GAIF01008186.1	0	0	0.05	0.2	0.88	0
GAIF01008187.1	0	0	0	0.02	0	0
GAIF01008190.1	13.69	17.01	11.77	33.01	22.65	8.39
GAIF01008191.1	1.52	0.16	0.11	4.84	15.39	0
GAIF01008192.1	0	0	0.14	0	0	0
GAIF01008193.1	0.11	0.23	0.23	0.73	3.03	0.35
GAIF01008194.1	381.38	590.99	776.05	1998.59	1259.86	320.87
GAIF01008196.1	0.25	0	0.13	1.24	4.88	0.31
GAIF01008197.1	0.74	0	2.09	0.76	0	0
GAIF01008198.1	0.19	0.05	0	0.43	2.06	0.02
GAIF01008200.1	0	0.22	0.15	0	0	0.04
GAIF01008201.1	0	0	0	0	0.18	0.08
GAIF01008203.1	0.12	0	0	0	4.34	1.41
GAIF01008205.1	38.19	92.27	60.81	73.61	55.42	7.44
GAIF01008207.1	62.99	73.12	62.28	160.26	88.26	23.98
GAIF01008208.1	10.79	8.59	1.88	57.43	4.75	0.15
GAIF01008209.1	0.47	0.04	0.24	0.08	1.81	0.12
GAIF01008210.1	0.52	0	0	0.91	3.62	0
GAIF01008211.1	21.58	12.56	7.37	40.95	46.16	26.92
GAIF01008212.1	13.4	9.88	4.46	6.29	3.18	1.38
GAIF01008213.1	0	0	0	1.11	6.13	0
GAIF01008216.1	0.72	0	1.32	3.52	3.36	9.51
GAIF01008217.1	70.74	38.08	19.93	87.62	62.68	10.14
GAIF01008220.1	81.31	24.09	69.65	40.43	16.41	114.15
GAIF01008221.1	0	0	0.19	0.1	0.07	0.03

GAIF01008222.1	0	0.08	0	114.63	0	0.33
GAIF01008223.1	71.75	98.5	60.84	83.62	31.75	11.1
GAIF01008224.1	13.86	15.87	14.42	19.06	9.87	6.43
GAIF01008226.1	0	0	0	0.29	0	0
GAIF01008230.1	0	0	0.42	0	0	0
GAIF01008231.1	2.05	2.37	3.14	1.42	3.61	0.9
GAIF01008232.1	0.11	0	0.04	0.16	1.53	0.04
GAIF01008233.1	0.08	0.45	0.54	0	0	0
GAIF01008234.1	0.05	0	0	0	0	0
GAIF01008235.1	0	0.06	0.1	0	0.07	0
GAIF01008237.1	84.73	153.34	18.3	134.01	229.85	7.43
GAIF01008238.1	42.23	63.36	48.23	53.65	52.39	8.6
GAIF01008240.1	0	0.42	0.28	0	0	0.16
GAIF01008241.1	0.04	0.13	0.09	0.25	0.56	0.02
GAIF01008242.1	0.21	0.35	0	2.22	8.56	0.12
GAIF01008243.1	0.09	0.1	0.21	0	0.45	0
GAIF01008244.1	1.23	1.12	0	0.38	0.16	0.08
GAIF01008245.1	0.03	0	0	0.16	0.27	0
GAIF01008246.1	0.03	0.03	0	0.07	0.23	0
GAIF01008248.1	0.11	0.19	0	0.58	1.13	0.05
GAIF01008250.1	7.38	8.12	6.56	2.24	3.95	1.95
GAIF01008253.1	0.18	0	0.2	0.21	0.43	0
GAIF01008257.1	0	0	0	0	0.17	0
GAIF01008258.1	0.15	0.22	0.11	0.2	2.03	0.06
GAIF01008260.1	0	0.05	0	0.22	0.66	0
GAIF01008261.1	0.95	0.78	0.52	0.28	0.29	0
GAIF01008262.1	0.45	0.18	0.15	0.11	0.1	0.28
GAIF01008263.1	0.23	0.1	0	0.78	6.31	0.57
GAIF01008264.1	30.49	5.66	7.05	32.45	53.49	65.19
GAIF01008265.1	6.36	14.24	4.17	43.69	48.32	2.56
GAIF01008266.1	0	0	0	0.1	0.53	0
GAIF01008268.1	0.21	0	0	0	0	0.11
GAIF01008271.1	0.06	0	0	0.15	0.08	0
GAIF01008272.1	0	0	0	0	0.1	0
GAIF01008273.1	0	0	0	0	0.82	0
GAIF01008274.1	0.36	0	0.22	0	0	0.22
GAIF01008277.1	0	2.88	2.53	0.63	0	1.77
GAIF01008278.1	26.85	29.84	24.34	79.16	63.25	20.93
GAIF01008280.1	0	0	0	0	0.15	0
GAIF01008282.1	5.38	2.77	2.48	4.36	21.38	3.28
GAIF01008283.1	0.07	0	0	0	0.74	0
GAIF01008285.1	70.61	66.64	68.78	80.88	60.53	78
GAIF01008286.1	198.94	125.21	189.71	139.78	184.37	87.86
GAIF01008287.1	5.89	6.54	1.21	1.35	1.18	0

GAIF01008288.1	24.15	27.08	11.71	27.67	38.29	25.69
GAIF01008289.1	0	0	0	0	2.04	0
GAIF01008290.1	0	0	0	0.24	0.24	0
GAIF01008291.1	0.33	0.04	0.04	2.26	9.54	0.25
GAIF01008293.1	0	0.16	0	0	0.02	0
GAIF01008294.1	50.05	97.8	51.67	19.19	14.45	0.85
GAIF01008297.1	0	0	0	0	0.02	0
GAIF01008298.1	23.97	17.09	47.17	11.54	4.22	23.96
GAIF01008300.1	0.23	0.56	0.23	0.65	0.72	0
GAIF01008302.1	1115.94	1798.48	1233.09	679.03	646.88	127.05
GAIF01008303.1	19.36	15.79	9.04	56.07	23.19	4.11
GAIF01008304.1	0	0	0	0	0	2.8
GAIF01008307.1	341.89	320.29	211.06	498.46	407.16	289.14
GAIF01008308.1	0.1	0.07	0	0	0.1	0
GAIF01008311.1	0	0	0	0.52	0.89	0
GAIF01008312.1	15.4	8.34	8.37	28.56	16.64	8.46
GAIF01008314.1	3.36	4.01	1.62	10.98	16.49	0.92
GAIF01008316.1	0	0	0	0	0.19	0
GAIF01008319.1	0.37	0.5	0.1	0.85	4.3	0.2
GAIF01008322.1	0	0	0	0.62	0.89	0
GAIF01008324.1	2.48	0.24	0.06	3.86	8.46	20.85
GAIF01008325.1	1.24	3.51	0.25	0.45	0.74	1.01
GAIF01008326.1	53.86	38.02	32.87	79.33	53.27	33.26
GAIF01008328.1	3.39	2.26	3.13	1.31	0.57	0.13
GAIF01008329.1	1.44	6.01	0.02	0.23	0.01	0.13
GAIF01008330.1	0	0.01	0	0.01	0	0
GAIF01008331.1	0	15.14	8.66	29.35	19.4	2.42
GAIF01008332.1	10.9	29.91	31.16	10.21	26.48	12.61
GAIF01008334.1	0.39	0.47	0.32	2.04	5.22	0.71
GAIF01008336.1	1591.46	1511.12	2056.25	779.32	452.47	340.75
GAIF01008337.1	16.35	18.12	11.43	48.13	37.33	5.5
GAIF01008338.1	0	0	0	0	0.07	0
GAIF01008339.1	0	0.27	0	0.97	2.92	0
GAIF01008340.1	93.88	127.43	82.08	95.93	68.87	18.74
GAIF01008341.1	2.05	2.36	1.19	5.04	8.71	1.46
GAIF01008343.1	101.45	54.09	62.6	152.29	149.74	120.85
GAIF01008344.1	5.98	0	0.79	4.01	0.4	8.23
GAIF01008345.1	0.72	0.43	0.51	0.29	0	0.05
GAIF01008346.1	0.19	0.07	0	0.44	1.38	0.14
GAIF01008347.1	0.19	0	0.1	0.33	0.68	0.31
GAIF01008349.1	0	0	0	0	0.24	0.22
GAIF01008350.1	0.07	0	0	1.02	5.14	0.45
GAIF01008351.1	39.92	41.36	41.31	58.65	21.79	10.56
GAIF01008354.1	0.21	0	0	0.61	2.65	0

GAIF01008355.1	0	0	0	0	0.32	0
GAIF01008356.1	0	0.22	0	0	0.12	0
GAIF01008357.1	6.08	3.1	1.61	14.41	31.31	2.24
GAIF01008358.1	0	0	0	0.22	0	0
GAIF01008360.1	0	0.28	0	0.15	0	0
GAIF01008361.1	4.16	4.52	1.29	7.06	8.98	0.12
GAIF01008365.1	0	0	0	2.28	15.07	0.89
GAIF01008367.1	2.21	1.6	0.17	0.67	0.44	0
GAIF01008368.1	0.12	0.13	0.13	0.14	1.15	0.13
GAIF01008369.1	6.87	11.62	8.06	18.94	15.74	2.01
GAIF01008371.1	0	0.26	0	0	0.44	0
GAIF01008373.1	0.14	0.15	0.3	1.12	1.5	0.46
GAIF01008374.1	0	0	0	0	0.94	0
GAIF01008375.1	2.8	0.94	0.47	10.2	20.57	0.95
GAIF01008376.1	0	0	0	0.26	0.45	0
GAIF01008377.1	0.48	0.06	0.26	0.56	2.95	0.53
GAIF01008378.1	0.07	0.02	0.04	0.04	0.1	0
GAIF01008379.1	0.27	0	0	0.16	0.16	0
GAIF01008380.1	2	3.86	0.46	51.39	3.84	1
GAIF01008381.1	375.49	82.95	102.96	5.58	3.58	7.33
GAIF01008382.1	43.26	46.78	71.2	73.08	29.25	36.99
GAIF01008383.1	15.47	20.87	20.15	26.32	17.7	11.46
GAIF01008384.1	0	7.88	5.55	0	0.18	2.75
GAIF01008385.1	0.19	0.31	0	0.47	0.26	0
GAIF01008386.1	2.78	1.2	1.69	1.33	1.79	0.4
GAIF01008388.1	1.26	0.26	0	1.93	1.44	1.74
GAIF01008389.1	0.2	0.09	0.06	3.14	7.63	0.03
GAIF01008390.1	0.29	0	0.22	1.11	2.53	0.09
GAIF01008391.1	5.38	8.17	1.8	7.72	11.52	0.54
GAIF01008392.1	15.6	13.26	7.54	58.01	31.11	14.07
GAIF01008393.1	0.11	0.06	0.13	0.33	0.96	0
GAIF01008394.1	0	0	0	0	0.19	0
GAIF01008395.1	11.04	9.23	6.66	30.68	11.52	18.71
GAIF01008396.1	0.83	0.16	0.21	2.05	5.74	0.17
GAIF01008399.1	9.47	5.52	3.08	13.9	16.02	5.82
GAIF01008400.1	16.68	14.98	27.91	14.83	7.49	8.95
GAIF01008401.1	0.42	0.34	0	0.36	1.76	0
GAIF01008403.1	0.1	0	0	0	0	0.06
GAIF01008404.1	79.79	37.56	29.95	53.33	32.91	96.32
GAIF01008405.1	0.08	0	0	0	0.3	0
GAIF01008406.1	0	0	0	0.15	1.36	0
GAIF01008409.1	0	0.15	0.16	1.15	1.49	0
GAIF01008411.1	34.47	31.16	27.03	178.35	222.96	39.56
GAIF01008412.1	0	0	0	0.08	0	0

GAIF01008413.1	0	1.16	22.43	0	0.72	10.43
GAIF01008414.1	0	0	0.15	0	0	0
GAIF01008415.1	0	0	0	1.37	5.85	0
GAIF01008417.1	0	0	0	0.07	0.31	0
GAIF01008418.1	0.15	0	0	0	0.54	0.16
GAIF01008419.1	21.88	14.03	18.66	4.81	0	21.83
GAIF01008420.1	0.32	0	1.18	0.48	0	0.99
GAIF01008421.1	0	0	0	0.14	0.14	0
GAIF01008422.1	0	0	0	0.22	0.05	0
GAIF01008423.1	0	0	0	0.11	0	0
GAIF01008424.1	0.02	0	0	0.11	0.24	0
GAIF01008425.1	0	0	0	0.44	0	0
GAIF01008426.1	0	0	0.12	0.12	0.14	0
GAIF01008428.1	0.36	0	0.7	0.71	0.16	0
GAIF01008429.1	1.55	0.73	0.83	3.25	14.56	1.05
GAIF01008432.1	0	0	0	0	0.04	0
GAIF01008433.1	10.3	12.62	14.43	17.31	12.55	5.44
GAIF01008434.1	0.13	0.29	0.44	0	1.45	0.29
GAIF01008436.1	37.84	9.72	4.5	8.98	7.16	0
GAIF01008437.1	0.94	0.2	0.16	2.78	9.8	3.19
GAIF01008438.1	0	0	0	0	0.14	0
GAIF01008440.1	0.11	0.04	0	1.19	5.67	0.08
GAIF01008442.1	0	0.09	0.09	0	0.1	0.33
GAIF01008443.1	0	0	0	0.05	0	0
GAIF01008444.1	13.57	17.41	15.66	39.17	38.66	25.42
GAIF01008445.1	0	1.46	0	0	1.09	0
GAIF01008448.1	13.31	10.74	8.48	22.92	18.07	8.52
GAIF01008449.1	0.25	1.62	7.34	0.6	1.37	7.73
GAIF01008452.1	0	0	0	0	0.15	0
GAIF01008454.1	0.94	1.16	0.7	5.66	11.45	0.08
GAIF01008456.1	0.78	0	0	2.62	7.88	0
GAIF01008458.1	3.44	0	0.01	2.87	0.83	0.21
GAIF01008459.1	6.75	8.37	8.98	1.11	0.29	1.75
GAIF01008460.1	0.37	0	0	0.57	0.44	0.27
GAIF01008462.1	0.04	0.29	0.14	0.1	0.52	0
GAIF01008463.1	0	7.69	16.77	0	0	1.71
GAIF01008464.1	2.32	0.23	0	16.52	56.51	0.62
GAIF01008466.1	42.8	109.58	117.86	7.56	7.22	6.97
GAIF01008468.1	34.32	57.46	20.76	42.75	63.71	13.45
GAIF01008469.1	10.73	6.78	8.9	1.92	0.72	6.23
GAIF01008470.1	1.75	0.35	0	6.61	34.32	0.43
GAIF01008472.1	0	0.04	0	0	0	0
GAIF01008473.1	3.11	1.33	0.27	13.86	47.35	0.45
GAIF01008474.1	3.24	0.65	2.3	4.71	1.07	1.29

GAIF01008475.1	0.05	0	0	0.12	0.09	0
GAIF01008476.1	5.78	3.1	3.55	5.64	7.41	1.36
GAIF01008478.1	8.14	0.93	7.06	0.65	0	0.19
GAIF01008479.1	0	0.67	1.15	0	0	0
GAIF01008480.1	0.18	0.2	0.3	0.74	0.98	0
GAIF01008481.1	0.37	0.04	0	0.16	0.16	0
GAIF01008482.1	639.22	214.03	148.75	1651.23	126.18	378.27
GAIF01008484.1	3.06	8.78	5.54	11.4	17.6	3.52
GAIF01008485.1	0	0	0	0	0.31	0
GAIF01008486.1	7.31	6.95	5.58	23.34	32.18	4.14
GAIF01008487.1	21.64	28.55	26.05	44.51	19.2	9.04
GAIF01008488.1	0.04	0.03	0	0	0	0
GAIF01008490.1	0.13	0	0	1.78	4.15	0
GAIF01008492.1	116.9	106.25	107.75	228.42	254.33	111.9
GAIF01008493.1	0.2	0.52	0.15	0.95	4.58	0.08
GAIF01008494.1	22.01	30.53	31.8	44.98	38.42	10.24
GAIF01008495.1	0	0	0	0	0.11	0
GAIF01008496.1	0.2	1.44	1.12	0.12	0	0.22
GAIF01008498.1	135.89	67.33	52.65	90.54	112.33	77.14
GAIF01008499.1	7.8	4.15	5.03	9.8	8.77	8.55
GAIF01008503.1	1.14	0.26	1.11	0	0.92	0.14
GAIF01008504.1	0.16	0	0	0.3	2.48	0.11
GAIF01008505.1	0.76	0.63	0.28	0.73	2.15	0.72
GAIF01008507.1	53.83	51.19	36.36	113.68	142.03	24.27
GAIF01008508.1	165.25	157.16	193.77	124.92	106.96	504.71
GAIF01008511.1	0.64	0.91	0.57	4.88	11.42	6.64
GAIF01008512.1	0.03	0	0	0	0.27	0
GAIF01008513.1	0.17	0	0	0.4	3.69	0.19
GAIF01008514.1	0.71	1.69	1.44	0.84	0	0
GAIF01008515.1	137.99	172.41	366.95	173.95	118.27	261.92
GAIF01008516.1	0.83	0.53	0	3.47	15.34	0
GAIF01008518.1	135	25.58	8.33	34.85	74.66	16.58
GAIF01008519.1	1610.61	1291.31	1310.83	1020.43	1173.01	6522.14
GAIF01008520.1	0	0.23	0.09	0	0.35	0
GAIF01008522.1	0	0	0	0	1.46	0
GAIF01008523.1	0.04	0.13	0.28	0.07	0.1	0
GAIF01008524.1	0.88	0.85	0.5	0.76	0.19	0.97
GAIF01008525.1	0	0.04	0	0	0	0
GAIF01008526.1	0	0	0	2.5	0	0
GAIF01008527.1	0	0	0	0	0.11	0
GAIF01008530.1	0.32	0.25	0.05	1.94	7.43	0.14
GAIF01008531.1	4037.3	904.97	2012.86	47.84	35.8	127.94
GAIF01008532.1	0.39	0.05	0.3	0.24	0.83	0.25
GAIF01008533.1	0.07	0	0	0.22	0.75	0

GAIF01008534.1	0	1.19	0.61	0	0	0
GAIF01008535.1	0.98	0.15	0.07	8.04	12.05	0.8
GAIF01008536.1	0.07	0	0.15	0.08	0	0
GAIF01008537.1	0	0	0	3.07	7.19	0
GAIF01008538.1	0.69	1.41	0.71	0.35	0.32	0.08
GAIF01008540.1	43.42	25.78	15.55	4.27	4.09	1.43
GAIF01008541.1	258.44	153.81	323.05	26.43	12.7	30.44
GAIF01008542.1	4.85	0.93	0.78	11.25	34.87	6.75
GAIF01008543.1	0	0	0	0	0.19	0
GAIF01008544.1	0	0	0	0	0.25	0
GAIF01008545.1	0	0	0	0.17	0.35	0.16
GAIF01008546.1	0	0	0.1	0.32	3.29	0.3
GAIF01008549.1	194.36	51.07	74.66	507.55	746.5	4435.69
GAIF01008550.1	0.05	0	0	0.17	0	0
GAIF01008551.1	0	0.87	0	0.09	0	0
GAIF01008553.1	0.42	0.45	0.15	4.69	15.72	0.27
GAIF01008554.1	0.12	0	0	0	1.57	0
GAIF01008555.1	1.14	0.22	0	1.58	6.4	0.45
GAIF01008556.1	0	0	0	0	0	0.19
GAIF01008558.1	0	0.27	0.28	0	0	0
GAIF01008559.1	3.58	3.76	4.9	2.63	1.97	2.98
GAIF01008560.1	3.99	2.69	0.3	1.91	3.14	0
GAIF01008562.1	1.07	0.74	0.06	1.52	2.93	0.06
GAIF01008563.1	2.55	0.2	0.5	14.17	44.61	40.27
GAIF01008565.1	0.26	0.07	0	1.11	6.54	0.28
GAIF01008566.1	7.17	5.04	15.07	4.65	2.19	10.46
GAIF01008568.1	0	2.3	1.81	0	0.09	0
GAIF01008570.1	10.94	7.71	5.43	21.91	22.69	2.93
GAIF01008571.1	10.66	8.06	7.25	23.65	19.63	10.51
GAIF01008572.1	0	1.1	0.52	0.63	1.94	1.88
GAIF01008574.1	94.84	57.41	39.4	133.49	104.49	49.63
GAIF01008576.1	0	0.34	0.29	0	0	0.29
GAIF01008577.1	0.21	0	0	0.38	2.17	0.1
GAIF01008578.1	0.27	0.03	0.07	1.21	3.6	0.4
GAIF01008579.1	0.14	0	0	0.15	0.17	0
GAIF01008580.1	0.64	0.35	0	0	3.49	0
GAIF01008581.1	20.07	6.34	0	44.51	56.1	18.1
GAIF01008582.1	1477.39	6130.6	3981.01	80.41	200.55	136.61
GAIF01008583.1	9.46	3.07	4.07	17.62	33.83	11.73
GAIF01008587.1	0.54	0.19	0.2	0.83	3.01	0.2
GAIF01008588.1	0.32	0	0.09	0.09	1.06	0
GAIF01008589.1	0	0.88	1.12	0	0.08	0.07
GAIF01008591.1	1.25	0.32	0.46	9.66	2.21	0
GAIF01008592.1	17.75	23.35	22.99	52.94	18.14	18.19

GAIF01008593.1	0.45	0.35	0.41	0.57	1.32	0.35
GAIF01008594.1	20.41	27.7	29.18	27.01	17.11	12.28
GAIF01008595.1	0.14	0	0.23	0.24	0	0.15
GAIF01008596.1	0	0.13	0	4.06	1.06	0.33
GAIF01008597.1	0	0	0	0.12	0	0
GAIF01008598.1	0.09	0.05	0.05	0.64	0.94	0.2
GAIF01008599.1	1.28	1.73	0	0.22	0.21	2.5
GAIF01008600.1	0	0	0	0	0.04	0
GAIF01008601.1	0	0	0	0.06	0	0
GAIF01008604.1	1.95	1.95	2.49	0.12	0.06	0.35
GAIF01008605.1	525.7	502.35	605.87	345.34	241.59	345.99
GAIF01008607.1	0	0.14	0	0.29	2.86	0
GAIF01008609.1	1.44	3.54	1	2.38	2.46	0.04
GAIF01008610.1	0	0.01	0	0	0	0.01
GAIF01008611.1	0.32	0	0	0	0	0
GAIF01008613.1	0.15	0	0.16	0	0	0
GAIF01008614.1	0	0.08	0	0	0	0
GAIF01008616.1	9.34	4.98	0.84	16.56	16.53	2.99
GAIF01008617.1	12.35	18.38	11.51	39.22	31.42	2.64
GAIF01008619.1	6.85	3.25	9.42	4.73	5.03	10.89
GAIF01008620.1	11.1	9.01	12.34	24.55	18.68	23.81
GAIF01008621.1	5.42	1.44	0.36	9.82	18.92	2.84
GAIF01008622.1	24	10.63	14.64	31.98	17.1	23.19
GAIF01008623.1	0	0.89	0	0.18	0	0.9
GAIF01008624.1	0.84	1.21	0.09	0.15	0.05	0
GAIF01008625.1	0	0.25	0	0	1.84	0.22
GAIF01008627.1	0.13	0.5	0	0.88	1.37	0.05
GAIF01008630.1	0	0	0	0	0.17	0
GAIF01008631.1	16.55	12.67	7.95	44.65	55.24	6.16
GAIF01008632.1	101.65	55.64	63.49	101.28	168.66	54.53
GAIF01008633.1	1.45	0.1	0.53	5.15	11.72	1.7
GAIF01008634.1	0.4	0	0	1.39	3.56	0.57
GAIF01008636.1	0.31	0.23	0.34	0.16	0.12	0.27
GAIF01008638.1	16.84	24.33	24.84	52.91	36.89	9.09
GAIF01008643.1	0.46	0.37	0.13	1.69	8.13	0.35
GAIF01008645.1	0	0.17	0.18	0	0.08	0
GAIF01008647.1	12.85	8.33	3.74	14.81	34.58	0.84
GAIF01008649.1	6.8	1.48	0.25	6.57	23.98	18.48
GAIF01008650.1	0	0	0	0	8.09	0
GAIF01008654.1	0.09	0.17	0.03	0.18	0.33	0.03
GAIF01008656.1	1.72	0.06	0.25	2.8	11.25	17.15
GAIF01008657.1	329.97	279.43	261.22	394.45	217.57	61.07
GAIF01008658.1	0	0	0.03	0	0.03	0
GAIF01008659.1	1.66	0.68	0.23	2.65	4.75	0.46

GAIF01008660.1	2.18	1.52	0.17	0.54	0.37	0.51
GAIF01008661.1	0	0.04	0	0	0.09	0
GAIF01008664.1	7.46	13.68	9.27	37.3	29.32	3.12
GAIF01008665.1	0.2	0.26	0	1	5.14	0.11
GAIF01008666.1	0	0	0	0	0.11	0
GAIF01008667.1	3.82	2.92	0.47	29.96	33.77	0.79
GAIF01008668.1	0	0.05	0	0	0.22	0
GAIF01008670.1	0.35	0	0	2.97	13.18	5.11
GAIF01008673.1	3.32	0.16	0.11	11.39	41.89	2.02
GAIF01008674.1	0	0.15	0.15	0.63	4.77	0.15
GAIF01008676.1	6.36	1.76	2.87	0	0.37	0.87
GAIF01008677.1	1.15	0.75	0.56	3.7	6.65	0.34
GAIF01008678.1	6.4	7.29	8.7	7.54	15.63	7.39
GAIF01008679.1	0.03	0	0	0.07	0	0
GAIF01008680.1	75.02	36.2	4.89	114.53	212.44	10.77
GAIF01008682.1	10.9	8.28	9.84	8.58	1.82	2.46
GAIF01008683.1	0.27	0	0	0	0	0
GAIF01008684.1	0	1.01	0	0.6	1.01	0.29
GAIF01008685.1	0.54	0.11	0	0.34	1.12	0.23
GAIF01008686.1	2.28	3.52	1.79	14.17	22.13	0.73
GAIF01008687.1	6.83	8.75	6.65	22.05	30.51	2.2
GAIF01008688.1	0.09	0.05	0.15	0.46	1.01	0
GAIF01008689.1	14.73	6.2	12.43	20.67	8.47	15.09
GAIF01008691.1	0	0.35	0	0	0	0
GAIF01008693.1	0	0	0	0.25	1.92	0
GAIF01008694.1	3.11	1.81	1.06	1.93	1.92	0.75
GAIF01008695.1	0.13	0	0	0.3	7.27	0.28
GAIF01008696.1	0	0	0	0	0.13	0
GAIF01008697.1	5.86	0.27	0.27	4.85	0.37	0
GAIF01008698.1	52.5	48.63	49.73	44.98	40.24	21.85
GAIF01008699.1	5.26	6.84	2.22	15.12	22.94	0.42
GAIF01008701.1	1.78	0.75	0.87	6.9	19.18	1.2
GAIF01008702.1	7.13	8.56	10.14	27.48	10.8	3.11
GAIF01008703.1	1.11	0.97	0.45	6.37	20.54	1.11
GAIF01008706.1	0.09	6.02	5.89	0	0	0.75
GAIF01008708.1	0.15	0.21	0.05	1.02	3.56	0.05
GAIF01008709.1	0	0.31	0.45	0	0	0
GAIF01008710.1	8.03	5.76	4.33	22.51	31.73	3.67
GAIF01008711.1	0.32	0.76	0.63	0.59	0.69	0
GAIF01008713.1	0.33	0.65	0.15	0.93	8.18	0.15
GAIF01008714.1	2683.76	2390.7	2129.35	1774.52	1783.93	8018.12
GAIF01008716.1	0	0.19	0.58	0.62	0.23	0.39
GAIF01008717.1	0	0	0	0.23	0.47	0.22
GAIF01008718.1	0	0.2	0.41	0	0	0

GAIF01008719.1	0.21	0	0	0.49	2.14	0.31
GAIF01008720.1	0.2	0.07	0	0	0.08	0
GAIF01008721.1	20.07	27.07	25.54	66.01	63.09	18.6
GAIF01008722.1	0	2.56	0.73	0	0.04	0.16
GAIF01008723.1	3.5	2.67	0	6.3	12.6	5.4
GAIF01008725.1	0.06	0	0	0.11	0.39	0
GAIF01008726.1	0.08	0.04	0.14	0	0	0
GAIF01008727.1	0	0	0.09	0	0	0.2
GAIF01008728.1	0.09	0	0	0	0.38	0
GAIF01008729.1	0.28	0.2	0.24	1.66	7.1	0.49
GAIF01008731.1	21.16	8.89	9.19	34.64	44	17.95
GAIF01008733.1	0.06	0	0	0.07	0.18	0
GAIF01008734.1	4.22	9.52	2.85	0.94	1.75	0
GAIF01008737.1	3.25	1.22	0.74	9.5	19.01	1.79
GAIF01008738.1	0	0	0.18	0	1.06	0
GAIF01008739.1	0	0.24	0	0	0	0
GAIF01008740.1	0	0.32	0.07	0	0	0
GAIF01008741.1	6.87	4.5	1.96	14.08	23.42	1.66
GAIF01008742.1	0	0	0	0.22	0.3	0
GAIF01008743.1	0	0	0	0.13	1.01	0
GAIF01008744.1	1.45	0	0	0.37	0.52	0
GAIF01008746.1	29.14	10.09	1.5	40.01	66.62	1.18
GAIF01008747.1	24.43	20.28	5.54	16.98	23.17	12.21
GAIF01008748.1	0.19	0.21	0	1.11	1.84	0
GAIF01008749.1	0.44	1.04	0	0.09	0.06	1.06
GAIF01008751.1	0	17.58	53.4	1.18	3.23	163.32
GAIF01008752.1	0.03	0.03	0	0.23	2.2	0
GAIF01008753.1	0	0	0	0	0.28	0
GAIF01008754.1	0	0	0	0.56	0	0
GAIF01008755.1	0	0	0.12	0	0	0
GAIF01008756.1	0	0.19	0.39	0	0	0
GAIF01008757.1	0.64	0.19	0.05	0.45	0	0
GAIF01008761.1	21.41	9.94	12.17	40.71	24.12	74.96
GAIF01008762.1	0.76	0.74	0.3	0.66	0.24	0.3
GAIF01008763.1	0	0.18	0	0.19	0.03	0
GAIF01008764.1	0	0.17	0	0	0	0
GAIF01008765.1	4.02	4.24	3.39	7.88	5.3	3.43
GAIF01008766.1	12.11	6.06	7.03	43.26	70.75	76.43
GAIF01008767.1	0	0	0	0	0.2	0.18
GAIF01008769.1	8.87	3.55	3.47	14.91	17.29	24.27
GAIF01008770.1	0	0	0	0	0.47	0
GAIF01008771.1	47.75	18.44	19.42	8.44	7.71	54.26
GAIF01008772.1	0	0	0	7.42	3.73	0
GAIF01008773.1	0.11	0.11	0	0	0	0

GAIF01008775.1	0.11	0.11	0.07	5.02	7.5	0.53
GAIF01008776.1	0	0	0	0	0.14	0
GAIF01008777.1	0	0.03	0	0.04	0.09	0
GAIF01008780.1	6.21	2.77	1.06	3.87	1.96	0
GAIF01008782.1	0.13	0	0.15	0.16	0.49	0.15
GAIF01008783.1	0	0.19	0	0.9	4.56	2.38
GAIF01008784.1	0	0	0.71	0	0	0
GAIF01008785.1	15.56	10.55	17.63	37.16	75.67	57.03
GAIF01008786.1	1.07	0.52	0.73	5.09	5.18	0
GAIF01008787.1	0.46	0.18	0.37	0.61	1.96	1.03
GAIF01008791.1	1.81	1.07	0.99	3.46	10.36	2.45
GAIF01008792.1	1.57	0.22	0	4.45	14.79	3.07
GAIF01008793.1	2.79	1.9	1	3.61	5.3	6.58
GAIF01008794.1	0.05	0.05	0.1	0.99	0	0
GAIF01008795.1	0	0	0	0.08	0.18	0.08
GAIF01008796.1	0.15	0.11	0.22	0.12	0.12	0.05
GAIF01008797.1	0	0	0	0	1.23	0
GAIF01008799.1	0	0	0	0.08	0	0
GAIF01008801.1	0.39	0	0	0	0.71	0.22
GAIF01008802.1	0.01	0	0	0.02	0.02	0.01
GAIF01008803.1	0.14	0	0.12	0	1.04	0
GAIF01008804.1	0.42	0	0	2.92	15.23	0
GAIF01008805.1	1.22	0.31	0.1	2.08	6.03	0.33
GAIF01008806.1	12.55	11.37	6.96	20.34	20.91	6.05
GAIF01008807.1	0	0	0	0	0	2.38
GAIF01008808.1	37.03	82.49	17.32	72.58	116.72	35.17
GAIF01008810.1	3.06	4.48	1.48	16.43	20.15	7.91
GAIF01008813.1	0	0	0.21	0	0	0
GAIF01008815.1	1.95	1.11	0.23	9.65	15.6	0.1
GAIF01008816.1	4.09	2.33	1.02	5.62	28.49	1.54
GAIF01008817.1	0	0	0	0	0	0.03
GAIF01008818.1	20.27	20.45	14.75	34.4	53.68	4.76
GAIF01008819.1	0.17	0	0	2.07	0.2	0.73
GAIF01008821.1	0	0	0	0	1.17	0
GAIF01008822.1	0.19	0.1	0	0.87	3.82	0
GAIF01008823.1	0	0	0	0	0.2	0
GAIF01008824.1	2.32	2.86	1.01	14.91	28.02	0.47
GAIF01008827.1	0.17	0	0	0.19	0.5	0.18
GAIF01008828.1	0.16	0.25	0	0	0.54	0.09
GAIF01008830.1	0.34	0.36	0	0	5.81	0
GAIF01008831.1	13.6	4.11	0	33.08	130.04	1.82
GAIF01008832.1	1.05	0	0	0	0	0
GAIF01008833.1	25.89	18.47	26.12	27.09	15.22	14.93
GAIF01008834.1	3.21	14.24	6.2	3.89	4.03	0

GAIF01008835.1	0.05	0	0	0	0	0
GAIF01008836.1	1.56	0.35	0	14.59	62.38	0.65
GAIF01008837.1	15.75	2.28	1.26	158.19	433.31	198.6
GAIF01008839.1	0.21	0	0	0	0.17	0
GAIF01008842.1	0	0	0	0	0.07	0
GAIF01008844.1	0.28	0	0	0.23	0.05	0.04
GAIF01008847.1	1.12	1.23	1.52	3.82	1.79	0.59
GAIF01008848.1	0.11	0.09	0	0	0	0
GAIF01008849.1	0.06	0.02	0	0	0.03	0
GAIF01008850.1	0.04	0	0	0	0.26	0
GAIF01008851.1	0.2	0.22	0.88	0	0	0
GAIF01008852.1	0	0	0	0	0.18	0
GAIF01008853.1	0	0	0	2.08	1.57	0.58
GAIF01008854.1	22.76	27.11	39.68	66.38	35.35	31.14
GAIF01008855.1	3.69	6.91	1.4	8.26	12.39	2.1
GAIF01008856.1	0	1.1	0.5	0	0	0.1
GAIF01008857.1	0	0	0.08	0.1	0.11	0.05
GAIF01008859.1	63.68	101.15	85.4	82.37	67.76	46.61
GAIF01008860.1	1.53	1.33	1.91	0.24	0.12	0.23
GAIF01008861.1	0	0	0	0.09	0.18	0
GAIF01008862.1	3.57	4.42	1.3	12.88	34.07	0.22
GAIF01008864.1	20.3	15.65	13.07	16.95	4.23	3.42
GAIF01008865.1	0.32	0.1	0.1	2.15	7.69	0.25
GAIF01008866.1	9.61	5.58	5.83	10.53	7.79	6.51
GAIF01008868.1	19.58	19.72	29.74	45.86	42.3	17.18
GAIF01008869.1	49.24	0.81	0.1	3.67	0.45	0.62
GAIF01008870.1	0.72	0.13	0	3.05	15.68	0
GAIF01008872.1	3.31	2.1	0.81	9.92	14.04	1.22
GAIF01008873.1	51.33	53.75	15.13	11.99	17.61	0
GAIF01008874.1	0	0	0	0.02	0.26	0
GAIF01008875.1	0	0	0	17.01	0	0.09
GAIF01008877.1	2.06	1.46	0.76	4.12	8.27	0
GAIF01008878.1	0.22	0.04	0.04	0.17	0.53	0.04
GAIF01008881.1	8.62	16.96	9.07	19.59	16.38	2.05
GAIF01008882.1	2.19	4.48	0.78	24.28	43.71	0.12
GAIF01008884.1	0	0	0	0	0.98	0
GAIF01008885.1	216.81	78.76	65.91	20.64	14.91	19.06
GAIF01008886.1	5.46	13.86	8.23	17.92	11.07	1.75
GAIF01008887.1	0	0	0	0	0.18	0
GAIF01008888.1	11.16	11.27	11.7	9.53	5.54	2.62
GAIF01008889.1	0	0.1	0	0	0	0
GAIF01008890.1	0.07	0	0.16	0.42	2.36	0
GAIF01008891.1	0.51	1.11	0.67	1.3	4.66	0.11
GAIF01008892.1	87.77	128.06	207.1	61.24	56.98	128.56

GAIF01008893.1	0.07	0	0	0	0	0
GAIF01008896.1	0.12	0	0	0.11	0.07	0
GAIF01008897.1	0.28	0.24	0.18	0.13	0.27	0.43
GAIF01008899.1	16.01	8.68	13.14	19.69	23.93	73.86
GAIF01008900.1	0.34	0.81	0.14	1.44	3.83	4.41
GAIF01008901.1	22.18	12.21	7.74	6.24	8.44	3.22
GAIF01008902.1	19.68	15.53	24.22	33.02	13.56	25.69
GAIF01008903.1	17.08	5.01	9.08	11.26	10.78	10.97
GAIF01008904.1	11.77	9.08	11.39	28.91	30.22	37.87
GAIF01008905.1	5.31	5.25	3.41	5.56	2.85	0.75
GAIF01008906.1	42.52	21.36	24.64	41.79	23.58	136.77
GAIF01008907.1	27.66	43.48	45.62	46.34	39.38	17.35
GAIF01008909.1	0.29	0.11	0.11	1.63	3.74	0.39
GAIF01008911.1	3.65	2.14	2.35	15.64	13.5	2.48
GAIF01008912.1	0	0	0	0	0.26	0.24
GAIF01008915.1	10.18	20.11	4.56	55.94	87.53	4.32
GAIF01008916.1	1.33	0.65	0.07	5.47	20.42	0.56
GAIF01008917.1	14.34	7.08	3.4	2.91	2.85	1.04
GAIF01008918.1	0.15	0	0	0.54	2.55	0
GAIF01008919.1	0	0.17	0	1.24	7.51	0
GAIF01008920.1	0	0	0.12	0	0	0
GAIF01008924.1	11.75	13.16	19.74	29.05	17.71	8.86
GAIF01008928.1	53.38	94.66	46.46	38.04	43.7	18.82
GAIF01008929.1	21.1	16.64	17.64	17.59	23.01	9.51
GAIF01008931.1	4.83	1.97	3.06	1.26	2.18	4.64
GAIF01008932.1	0	0	0	0.17	0	0
GAIF01008933.1	0.34	0.09	0	0.2	3.28	0
GAIF01008934.1	0.73	0.36	0.37	0.44	0.18	0
GAIF01008935.1	16.57	17.43	32.16	38.71	26.37	26.38
GAIF01008936.1	1.29	1.05	1.77	13.32	10.88	6.59
GAIF01008937.1	712.97	122.08	165.29	1248.29	2251.83	2551.43
GAIF01008938.1	0.43	0.15	0	0	0.06	0
GAIF01008939.1	110.88	57.55	23.07	188	128.33	6.48
GAIF01008941.1	0.03	0	0	0	0	0.03
GAIF01008943.1	0.69	0	0	1.01	1.7	1.19
GAIF01008945.1	0.49	0.23	0.15	2.12	12.31	0.08
GAIF01008948.1	29.31	0.64	1.36	1.31	0.19	0.12
GAIF01008950.1	2.07	0.18	0	3.83	7.94	14.72
GAIF01008951.1	0	0	0	0	0.05	0
GAIF01008952.1	0	0	0	0.5	0.59	0
GAIF01008953.1	89.59	41.25	22.91	17.8	23.07	51.86
GAIF01008954.1	0.64	0	0	0	0.31	0
GAIF01008955.1	13.68	22.81	13.46	32.82	35.83	6.37
GAIF01008956.1	42.93	46.09	37.06	57.07	45.45	18.04

GAIF01008957.1	1.06	1.52	1.35	6.52	2.67	1
GAIF01008958.1	0.94	1.31	0.62	6.52	3.7	0.37
GAIF01008959.1	0.94	0.4	0.25	4.2	11.95	0.53
GAIF01008962.1	45.84	60.36	52.09	46.4	33.71	7.31
GAIF01008965.1	2.78	7.68	1.29	10.64	14.26	0.37
GAIF01008966.1	0.44	1.27	0.23	2.01	0.7	0.08
GAIF01008967.1	5.36	9.15	4.44	1.53	0.5	0
GAIF01008968.1	5.17	2.69	0.74	0.64	0.6	0.27
GAIF01008969.1	341.02	655.95	344.42	27.42	94.33	132.74
GAIF01008970.1	39.27	11.33	10.82	0.07	0.07	0.07
GAIF01008971.1	71.62	54.17	41.93	148.91	140.3	11.63
GAIF01008972.1	1.46	0.53	0.27	6.41	19.25	1.34
GAIF01008973.1	0	0	0	0.09	0.11	0.16
GAIF01008975.1	1.3	1.09	0.88	0.64	1.39	0.06
GAIF01008979.1	0	4.9	6.5	0	0.07	0.43
GAIF01008980.1	15.93	24.01	16.15	15.57	1.03	2.4
GAIF01008981.1	0.09	0.15	0	0.48	1.22	0
GAIF01008983.1	0.49	0.21	0.11	3.88	7.21	0.54
GAIF01008985.1	0.13	0	0	0.15	0.56	0.14
GAIF01008986.1	19.69	3.88	2.35	1.82	1.54	2.36
GAIF01008987.1	0.49	0.11	0.19	0.88	0.57	0.04
GAIF01008988.1	1.69	0.65	0	1.54	2.46	3.31
GAIF01008990.1	164.83	80.85	66.11	172.48	217.63	29.57
GAIF01008992.1	248.55	409.81	386.47	186.65	328.73	919.34
GAIF01008993.1	0	0	0.06	0.45	1	0.18
GAIF01008994.1	0.03	0.1	0.03	0.22	0.57	0.03
GAIF01008995.1	0	0	0	0	0.1	0
GAIF01008996.1	0	0	0	0	1.29	0.24
GAIF01008997.1	0.05	0.19	0	0	0.34	0
GAIF01008998.1	0.05	0.11	0	0	0.12	0
GAIF01008999.1	0.24	0.09	0	1.74	9.76	0.09
GAIF01009000.1	148.26	47.47	124.13	0	0	0
GAIF01009001.1	0	0	0	0	0.03	0
GAIF01009002.1	0.84	0.21	0.07	1.2	8.08	0.35
GAIF01009003.1	39.77	52	57.24	91.9	72.27	21.21
GAIF01009005.1	1.75	8.25	3.5	13.69	7.41	1.1
GAIF01009006.1	7.8	2.71	1.26	0.38	1.11	0.44
GAIF01009007.1	1.89	2.23	0.78	1.65	1.42	0.17
GAIF01009008.1	0.76	0.31	0.78	2.85	11.56	4.77
GAIF01009012.1	21.8	15.72	10.97	80.42	78.8	12.78
GAIF01009013.1	16.02	0	0	4.68	8.41	0
GAIF01009014.1	0	0.15	0	0.16	0.32	0
GAIF01009015.1	0	0	0	0.27	1.21	0
GAIF01009016.1	0.17	0.06	0.12	2.01	6.24	0.31

GAIF01009018.1	0.76	0.41	0.1	0.22	0.68	0
GAIF01009019.1	0	0	0	0.14	0.38	0.05
GAIF01009020.1	16.66	10.75	11.93	71.83	63.66	16.46
GAIF01009024.1	51.3	6.03	7.13	95.51	159.03	160.95
GAIF01009025.1	1215.86	134.75	193.27	55.12	8.77	34.73
GAIF01009026.1	3.83	6.89	2.69	3.89	2.69	0.18
GAIF01009028.1	2.44	1.3	1.27	0.8	2.27	0.41
GAIF01009031.1	0.98	1.72	0.49	3.88	5.94	0.11
GAIF01009033.1	0	0.07	0	0	0.08	0.04
GAIF01009035.1	0.83	0.48	1.36	1.89	3.89	1.41
GAIF01009036.1	0	0	0	3.16	14.18	0
GAIF01009039.1	67.27	0.44	3.59	42.72	9.84	57.01
GAIF01009040.1	0.41	0	0	0	0.62	0
GAIF01009041.1	0.05	0.05	0	0.12	0	0
GAIF01009042.1	0	0	0	0.11	0.76	0
GAIF01009043.1	0.13	0.07	0.07	0.31	2.73	0.07
GAIF01009045.1	18.92	40.08	27.79	53.75	62.87	8.79
GAIF01009046.1	0	0.34	0	0.41	0.52	0
GAIF01009047.1	508.75	296.54	523.98	426.09	69.38	63.58
GAIF01009049.1	32.85	23.56	21.69	53.16	33.7	15.54
GAIF01009050.1	1.05	0.3	0.1	2.93	10.8	6.82
GAIF01009051.1	9.77	11.57	5.38	43.86	43.12	2.46
GAIF01009052.1	0	0	0	0	1.1	0
GAIF01009053.1	0	0.08	0.55	0.02	0	0
GAIF01009054.1	0	0	0.34	0	0	0
GAIF01009055.1	25.45	20.12	14.54	7.23	7.05	2
GAIF01009056.1	0	0	0	0	0.22	0
GAIF01009057.1	5	2.74	2.81	14.2	15.13	8.15
GAIF01009058.1	14.12	2.33	1.74	27.34	73.77	45.4
GAIF01009060.1	0.98	0.4	0.64	0.41	0.53	0
GAIF01009061.1	5.24	0	0	2.78	0	5.49
GAIF01009062.1	14.51	5.89	1.4	31.4	16.52	18.96
GAIF01009063.1	4.52	5.19	5.04	11.51	18.3	8.87
GAIF01009065.1	38.6	41.86	51.49	47.93	31.51	28.87
GAIF01009066.1	55.85	10.18	36.19	26.81	9.34	23.31
GAIF01009067.1	0.18	0.73	0	2.15	7.05	0
GAIF01009069.1	0	0.13	0.13	0	0	0.27
GAIF01009070.1	0.54	0.12	1	0.2	0	0.35
GAIF01009071.1	19.28	20.69	21.46	18.58	12.62	36.98
GAIF01009072.1	0	0	0	0.05	0.05	0
GAIF01009073.1	0.03	0.04	0.04	0.11	0.2	0.07
GAIF01009074.1	0.12	0	0	0.29	0.68	0
GAIF01009075.1	0.18	0.2	0.2	0.95	2.74	0.2
GAIF01009076.1	0	0	0	1.09	5.19	0

GAIF01009077.1	0	0	0	0	0.14	0
GAIF01009078.1	0	0	0	0.18	0.26	0
GAIF01009081.1	0	0	0.14	0.15	0.05	0.05
GAIF01009083.1	0	0	0.32	0	0	0
GAIF01009085.1	0.29	0	0	0.18	3	0.19
GAIF01009086.1	309.93	245.94	202.13	388.96	274.84	56.84
GAIF01009087.1	0.72	0	0	0	0	0
GAIF01009088.1	9.91	0.44	1.04	8.19	23.5	128.91
GAIF01009089.1	0.33	0	0	1.06	2.42	0.85
GAIF01009090.1	115.41	25.32	105.57	73.97	33.14	102.17
GAIF01009092.1	0.98	1.5	0.39	5.49	17.26	0.15
GAIF01009095.1	0	0.11	0.12	0.18	0.45	0.04
GAIF01009100.1	0	0	0	0	0.12	0
GAIF01009101.1	0	0	0	0.27	1.6	0.13
GAIF01009102.1	0	0.1	0	0	1.35	0
GAIF01009103.1	0.08	0.18	0.08	0.75	0.72	0
GAIF01009104.1	5.54	10	7.9	13.12	7.55	4.09
GAIF01009105.1	0.04	0.07	0.07	0.31	0	0
GAIF01009106.1	19.74	28.35	9.05	32.52	32.9	4.58
GAIF01009108.1	0	0.38	0.08	0.16	0	0.16
GAIF01009109.1	0	0	0.06	0.11	0.15	0
GAIF01009110.1	0.79	0	0	0	1.22	0
GAIF01009112.1	0	0.03	0.06	0	0.22	0
GAIF01009114.1	0	0	0	0	0.2	0
GAIF01009115.1	14.09	19.63	14.41	37.49	44.94	7.26
GAIF01009116.1	1.19	0	0	2.46	9.25	1.61
GAIF01009118.1	11.96	8.23	8.15	5.24	7.72	5.84
GAIF01009120.1	11.42	3.12	0.82	40.2	64.24	10.39
GAIF01009121.1	24.76	49.23	52.5	40.95	23.52	13.12
GAIF01009123.1	0.96	0.3	0.2	1.54	7.05	0.1
GAIF01009124.1	1.56	0.42	0.57	5.13	17.51	2.44
GAIF01009125.1	16.25	27.81	18.37	50.63	40.72	6.31
GAIF01009126.1	16.68	9.75	2.45	10.28	0.56	0
GAIF01009127.1	4.37	2.38	0	2.54	7.88	0
GAIF01009128.1	4.65	5.79	5.23	14.78	9.63	3.49
GAIF01009130.1	0	0	0	0	0.22	0
GAIF01009131.1	0.1	0.06	0	0	1.39	0.32
GAIF01009132.1	3.86	5.58	4.4	3.24	4.33	5.07
GAIF01009133.1	0	23.16	0	12.37	40.44	0
GAIF01009134.1	4.84	10.67	2.11	2.72	1.28	0.39
GAIF01009136.1	42.58	23.76	18.32	87.15	87.32	25.03
GAIF01009137.1	0	0	0.35	0.38	1.55	0
GAIF01009139.1	0.1	0	0	0.08	1.19	0.1
GAIF01009140.1	3.42	3.21	2.48	6.45	12.19	2.11

GAIF01009142.1	2.12	0	8	3.85	0	0
GAIF01009143.1	60.93	65.31	55.39	30.72	15.2	6.79
GAIF01009145.1	12	9.79	9.88	50.14	44.37	6.72
GAIF01009146.1	0.47	0.12	0	1.73	5	0
GAIF01009147.1	0.43	0.24	0.12	0.63	0.13	0.36
GAIF01009148.1	0.02	0.01	0	0.03	0.12	0
GAIF01009149.1	3.4	2.71	4.19	13.72	4.61	1.47
GAIF01009150.1	0.54	0	0	0.6	2.81	0
GAIF01009151.1	0	0.12	0	0.32	0.4	0
GAIF01009152.1	0	0	0	0.03	0	0
GAIF01009153.1	8.43	23.57	6.9	21.11	22.4	2.69
GAIF01009154.1	0.17	0.18	0.18	2.34	0.2	0.18
GAIF01009155.1	65.66	32.02	13.62	111.17	100.03	21.28
GAIF01009156.1	0.23	0	0	0	0	0
GAIF01009157.1	0.09	0	0.1	0.05	0	0
GAIF01009158.1	0.48	0.3	0	0.95	1.57	0
GAIF01009159.1	0	0	0	0	0.73	0.17
GAIF01009162.1	0.16	0.28	0.18	5.85	28.79	2.6
GAIF01009164.1	57.67	13.05	3.08	39.43	121.09	546.06
GAIF01009165.1	0.48	0.36	0.23	0.29	2.86	0.03
GAIF01009167.1	0.29	0.63	0	1.56	0.81	1.17
GAIF01009168.1	0.09	0.11	0.11	0.28	2.78	0.01
GAIF01009169.1	0.51	0.48	0.15	0.59	0.81	0
GAIF01009171.1	3.39	4.78	4.12	2.13	1.54	0.5
GAIF01009172.1	0.05	0	0.05	0.39	1.93	0.21
GAIF01009173.1	0.14	0.15	0	0	0	0.15
GAIF01009174.1	2.95	2.42	2.61	7.36	4.72	4.9
GAIF01009177.1	2.8	0.54	0.72	2.48	0.2	0.18
GAIF01009178.1	0.73	1.16	0.42	3.42	9.04	0.57
GAIF01009179.1	1.6	1.07	0	3.46	3.01	0.53
GAIF01009180.1	0	0	0.09	0	0.21	0.14
GAIF01009181.1	0.24	0.14	0.12	0.12	0	0
GAIF01009182.1	0.9	1.25	0	0.38	0	0
GAIF01009183.1	11.81	7.16	3.33	44.58	54.91	3.51
GAIF01009186.1	0	0.06	0	0.32	3.41	0
GAIF01009187.1	0	0	0	0.09	0.09	0
GAIF01009188.1	2.69	2.7	0.64	7.58	27.38	0
GAIF01009189.1	164.55	64.8	7.19	216.24	264.64	3.83
GAIF01009190.1	0.1	0.11	0	0	0.7	0
GAIF01009191.1	12.2	10.92	9.77	44.63	59.02	8.54
GAIF01009192.1	2.53	3.5	1.39	5.2	9.81	1.39
GAIF01009193.1	0	0	0	0.01	0.01	0
GAIF01009194.1	0.17	0.07	0.07	1.06	2.48	0.04
GAIF01009197.1	0	0	0.41	1.41	0.77	0.3

GAIF01009198.1	2.61	1.81	3.11	3.67	10.74	2.08
GAIF01009199.1	15.78	5.48	6.5	27.47	37.17	30.64
GAIF01009200.1	0.02	0.07	0	0	0.05	0.02
GAIF01009201.1	0	0	0.13	0	0	0
GAIF01009202.1	0	0	0	0.23	0	0.04
GAIF01009203.1	0	0	0	0.09	0.73	0
GAIF01009204.1	6.77	10.56	9.4	26.61	33.21	4.82
GAIF01009205.1	0	0	0	1.04	1.07	0.4
GAIF01009206.1	0	0	0	0	0.05	0
GAIF01009207.1	0	2.5	0	0	2.63	0
GAIF01009208.1	0	0	0	0.14	0.58	0
GAIF01009210.1	0	0	0	0	0.14	0
GAIF01009212.1	1.12	3	1.9	9.43	9.82	0.65
GAIF01009213.1	11.24	0	12.34	26.09	4.51	33.01
GAIF01009215.1	4.59	8.55	7.6	0.87	0.23	0.1
GAIF01009216.1	0.01	0.03	0	0.03	0.07	0
GAIF01009217.1	0.41	0	0	2.55	10.18	0.18
GAIF01009218.1	0	0	0	0	0.39	0
GAIF01009220.1	0	0	0	0	0.49	0
GAIF01009224.1	63.29	34.61	78.76	60.23	25.72	19.36
GAIF01009225.1	528.93	298.75	158.89	1074.73	873.54	129.88
GAIF01009226.1	0.15	0.33	0	0.35	3.38	0
GAIF01009227.1	62.04	36.13	17.99	55.86	75.44	140.8
GAIF01009229.1	0.03	0	0	0.35	1.89	0
GAIF01009230.1	3.4	3.43	1.8	16.07	9.01	0.95
GAIF01009233.1	0.79	0.43	0.29	2.13	4.42	0.87
GAIF01009234.1	0.1	0	0	0.33	0.48	0.05
GAIF01009235.1	33.04	56.46	27.09	42.91	54.8	18.78
GAIF01009237.1	7.54	6.41	4.06	2.39	1.15	2.27
GAIF01009238.1	0.25	0.09	0.09	0.1	0	0
GAIF01009241.1	0	0.01	0	0	0	0
GAIF01009242.1	0	2.86	3.03	0	0	0.87
GAIF01009243.1	26.77	51.05	50	31.02	29.58	13.4
GAIF01009244.1	9.54	11.72	3.2	36.98	38.3	1.6
GAIF01009246.1	79.05	73.34	257.66	69.33	40.67	212.93
GAIF01009247.1	0.24	0.05	0.02	0.65	1.15	0.02
GAIF01009248.1	0.05	0	0.03	0	0.13	0
GAIF01009252.1	21.41	18.86	32.82	36.65	14.76	18.77
GAIF01009254.1	0.37	0.14	0.54	1.75	1.82	0.36
GAIF01009255.1	0	0.1	0	0.83	5.14	0
GAIF01009256.1	0.85	0	0	0.71	0.58	0
GAIF01009257.1	0	0	0	0	0.25	0
GAIF01009258.1	18.8	11.64	10.73	1.01	0.35	0.32
GAIF01009259.1	0	0	0	0	0.24	0

GAIF01009260.1	724.39	419.73	182	293.35	198.45	41.22
GAIF01009261.1	84.66	22.37	11.66	18.87	22.53	0
GAIF01009265.1	0.14	0	0	0	0.17	0.31
GAIF01009266.1	3.34	2.32	3.05	10.78	11.85	1.3
GAIF01009267.1	1.87	0.11	0.11	10.4	24.16	44.61
GAIF01009269.1	5.77	4.43	7.26	13.68	20.6	3.64
GAIF01009270.1	800.53	577.92	491.62	0.5	2.08	1.36
GAIF01009271.1	0.89	0.2	0.38	0	0.27	0
GAIF01009272.1	25.18	18.1	12.97	54.95	69.42	5.92
GAIF01009274.1	0	0	0.2	0.21	1.59	0.1
GAIF01009275.1	0.3	0	0	1.64	3.72	0
GAIF01009277.1	0	0.02	0	0.14	0.67	0
GAIF01009279.1	11.87	15	9.63	19.3	0	3.81
GAIF01009281.1	0.29	0.27	0.4	0.19	0.15	0.09
GAIF01009282.1	0	0	0	0	0.36	0
GAIF01009283.1	3.43	2.83	0	6.1	5.8	0
GAIF01009286.1	23.44	10.89	11.14	48.96	75.6	155.46
GAIF01009288.1	1.89	3.02	4.21	0.7	0.29	0
GAIF01009290.1	0.18	0	0	0.29	1.75	0.26
GAIF01009291.1	0	0	0	0	16.64	0
GAIF01009292.1	7.91	8.55	10.27	35.75	18.04	4.99
GAIF01009293.1	1.54	0.65	0.26	2.34	20.43	0.79
GAIF01009294.1	105.04	122.38	65.81	17.39	0	0
GAIF01009296.1	0.09	0	0	0.28	1.43	0.07
GAIF01009298.1	1.26	0.64	0.09	3.11	6.44	0.09
GAIF01009300.1	0	4.6	2.91	0	0	0
GAIF01009301.1	0.21	0.15	0	0.08	0.17	0
GAIF01009302.1	4.79	2.28	1.99	0.35	0.31	0
GAIF01009303.1	0	0	0	0	0.07	0
GAIF01009304.1	0	0	0	0	0.72	0
GAIF01009306.1	0	0	0.04	0.04	0.09	0
GAIF01009307.1	0.43	0.23	0.89	0.59	0.36	0.37
GAIF01009308.1	0.98	0	0	0	0	0
GAIF01009309.1	24.5	35.38	32.37	73.37	56.02	9.94
GAIF01009310.1	0.2	0	0	0	1.58	0
GAIF01009311.1	0	0	0	0	0.38	0
GAIF01009312.1	0	0.04	0	0.13	0.7	0
GAIF01009313.1	0.14	0	0	0.23	2.76	0
GAIF01009314.1	0	0	0	0	0.15	0
GAIF01009315.1	15.65	11.06	18.44	7.97	3.05	15.9
GAIF01009316.1	0	0	0.16	0	0	0
GAIF01009317.1	3.99	4.62	1.27	3.44	2.32	1.84
GAIF01009318.1	0	0.13	0	0.26	0	0
GAIF01009319.1	0.02	0.02	0	0	0	0

GAIF01009320.1	0	0	0	0.37	0.63	1.11
GAIF01009321.1	0.1	0.42	0.34	0.4	0.75	0.18
GAIF01009324.1	25.72	40.2	26.42	54.99	69.84	14.17
GAIF01009325.1	10.88	7.06	3.72	9.49	9.44	4.93
GAIF01009327.1	0.34	0.06	0.22	1.18	3.05	0.12
GAIF01009328.1	0	0	2.81	0.37	0.11	0
GAIF01009329.1	13.6	2.93	1.26	5.73	9.43	2.69
GAIF01009330.1	0	0	0	0	1.96	0
GAIF01009331.1	0	0.1	0	0	0	0
GAIF01009332.1	0	1.6	1.94	0.09	0.09	0.81
GAIF01009333.1	528.19	356.25	30.28	397.86	439.91	162.02
GAIF01009334.1	13.44	10.94	4	20.99	21.67	3.96
GAIF01009335.1	3.49	10.07	25.29	0	0.2	11.62
GAIF01009336.1	0.3	1.13	0.65	3.26	4.8	0
GAIF01009337.1	0	0	0.05	0	0	0.11
GAIF01009338.1	0.63	0.11	0.81	0.12	0.13	0.12
GAIF01009339.1	33.43	69.22	89.27	25.57	23.86	18.93
GAIF01009340.1	3087.59	2082.44	4946.84	1011.42	492.95	2848.51
GAIF01009341.1	0	0	0	0	0.15	0
GAIF01009342.1	0.19	0	0.26	0.16	0.19	0.11
GAIF01009343.1	0.13	0	0.23	0.31	0	0
GAIF01009345.1	0	0	0	0	0.13	0
GAIF01009346.1	4.34	3.78	7.55	12.75	14.62	8.32
GAIF01009347.1	0	0	0	1.12	3.91	0
GAIF01009348.1	7.52	7.24	15.01	18.86	15.33	9.78
GAIF01009349.1	0	5.71	1.72	0	0.05	0.09
GAIF01009350.1	0	0.1	0	0	0	0
GAIF01009351.1	0	0	0	0.35	2.87	0
GAIF01009352.1	0	0.08	0.08	1.26	3.23	0.15
GAIF01009353.1	5.66	4.23	5.34	2.09	0.66	1.96
GAIF01009354.1	0.74	0.16	0	5.03	12.02	0.57
GAIF01009355.1	0.71	0.39	0	2.47	5.34	0.2
GAIF01009356.1	3.73	4.89	2.67	9.91	17.52	1.32
GAIF01009357.1	0	0	0	0.04	0.1	0
GAIF01009358.1	37.92	55.61	37	77.99	189.6	9.89
GAIF01009359.1	85.29	82.71	62.36	36.61	27.24	13.4
GAIF01009360.1	0	0	0	0.77	1.81	0.49
GAIF01009361.1	0	0	0	0	4.3	0
GAIF01009363.1	3.26	3.89	4.07	4.91	4.38	2.87
GAIF01009364.1	0	0	0	0	0.15	0
GAIF01009365.1	6.87	0	3.69	24.62	43.35	3.45
GAIF01009366.1	1.6	0.57	1.76	0.95	1.67	1.12
GAIF01009369.1	15.52	6.32	2.36	2.32	1.93	0.99
GAIF01009372.1	0	0	0	0.03	0	0

GAIF01009373.1	0.89	3.57	0	0	0	0
GAIF01009374.1	4.39	5.05	7.67	15.55	8.44	3.46
GAIF01009375.1	55.16	113.91	63.97	64.47	101.45	34.42
GAIF01009376.1	0.18	0	0	0.76	3.29	0.6
GAIF01009380.1	0	0.03	0	0	0.18	0.03
GAIF01009383.1	0.08	0	0	0	0.16	0
GAIF01009384.1	0.14	0.08	0.16	0.25	0.17	0.08
GAIF01009385.1	2977.21	1765.98	1875.54	1699.31	1490.65	4633.11
GAIF01009386.1	0	0.19	0.32	0.24	0.14	0.1
GAIF01009387.1	0.6	0.46	0.75	2.38	1.13	2.63
GAIF01009389.1	0.69	1.31	0.85	3.24	8.72	1.13
GAIF01009390.1	4.77	5.19	4.56	4	1.94	1.46
GAIF01009392.1	26.42	25.02	37.22	56.7	41.99	26.48
GAIF01009393.1	20.82	22.27	24.97	34.01	22.05	19.33
GAIF01009394.1	0	0	0	0	0.05	0
GAIF01009396.1	0.21	0	0	0	0	0
GAIF01009397.1	0.14	0.07	0.07	0	0.03	0
GAIF01009398.1	0.25	0	0	5.81	10.93	0
GAIF01009399.1	0	17.29	0	0	0	0
GAIF01009401.1	0	0.15	0	0.47	1.15	0
GAIF01009402.1	0.08	0	0	0	0.46	0
GAIF01009403.1	0	0.01	0	0.01	0.09	0
GAIF01009405.1	0	0	0	0.08	0.16	0
GAIF01009406.1	14.47	21.15	24.44	40.47	27.7	11.38
GAIF01009407.1	14.62	10.93	12.91	29.82	19.76	7.44
GAIF01009408.1	0.21	0.05	0.05	0.05	0	0
GAIF01009409.1	0.33	0.08	0	2.24	2.59	0.02
GAIF01009411.1	0.11	0	0	0.04	0.41	0
GAIF01009412.1	1.12	0.1	0.31	5.54	29.82	0.62
GAIF01009413.1	0.03	0.18	0.19	0.39	0.45	0
GAIF01009415.1	74.74	51.66	35.99	235.5	332.9	29.24
GAIF01009416.1	21.8	13.86	31.34	54.88	29.89	74.73
GAIF01009417.1	14.23	9.7	6.11	7.66	5.91	1
GAIF01009418.1	0	0.18	0	0.53	3.28	0
GAIF01009420.1	0	0	0	2.93	6.15	0
GAIF01009421.1	0	0	0	0	0.34	0
GAIF01009425.1	0.32	0.87	0	0.19	0.19	0.53
GAIF01009427.1	17.85	12.13	12.39	73.77	107.25	48.61
GAIF01009428.1	0	0	0	0.02	0.06	0
GAIF01009429.1	0	0	0	0.06	0.03	0.03
GAIF01009431.1	0	0	0.05	0	0	0
GAIF01009433.1	0	0	0	0.09	0	0
GAIF01009434.1	0	0	0	0	0.03	0
GAIF01009436.1	1.34	0	0	4.74	20.98	3.16

GAIF01009437.1	1.44	1.44	1.22	1.43	1.34	0.61
GAIF01009438.1	19.23	7.7	9.36	69.06	97.77	47.15
GAIF01009440.1	29.87	20.27	9.49	73.32	63.85	3.25
GAIF01009441.1	3.35	2.08	2.18	9.37	17.44	15.23
GAIF01009442.1	84.2	77.46	50.18	400.85	593.39	45.63
GAIF01009443.1	52.77	33.73	14.62	3.45	1.45	2.19
GAIF01009444.1	2.44	3.98	2.18	7.44	6.61	0.34
GAIF01009445.1	0	0	0	0	0	0.02
GAIF01009446.1	0.29	0.08	0	0.25	0.09	0.12
GAIF01009447.1	54.68	48.27	37.29	23.01	28.23	4.66
GAIF01009448.1	0.06	0	0	0	0	0
GAIF01009449.1	3.82	6.59	3.58	35.53	54.72	1.4
GAIF01009450.1	0.32	0.12	0.25	0.26	0.67	0.12
GAIF01009451.1	0	0	0	0	0.19	0
GAIF01009452.1	0.55	0.45	0	1.91	6.45	0
GAIF01009453.1	0	0	0	0.06	0.06	0
GAIF01009454.1	0	0	0	0	0.49	0
GAIF01009455.1	0.51	0	0	0.66	2.91	2.22
GAIF01009456.1	12.66	5.36	7.17	23.53	25.52	10.37
GAIF01009457.1	0	0	0	0.28	0.8	0.13
GAIF01009458.1	238.19	202.29	160.99	244.93	202.3	94.64
GAIF01009461.1	0	0	0	0.47	0.15	0
GAIF01009464.1	0.56	0.22	0.34	0.18	0.49	0.4
GAIF01009465.1	0	0	0	0	1.68	0
GAIF01009466.1	150.13	176.02	218.84	186.95	124.79	197.8
GAIF01009467.1	0.85	0.37	0.18	1.32	2.78	0.1
GAIF01009468.1	174.04	144.74	122.56	233.32	234.99	145.27
GAIF01009469.1	10.43	4.67	7.15	7.96	7.66	7.62
GAIF01009470.1	29.84	38.02	26.31	16.44	8.36	8.5
GAIF01009471.1	0	0	0	0.04	0.13	0
GAIF01009472.1	0.29	0.19	1.28	0.39	0.65	0.19
GAIF01009473.1	0	0.48	0	0	0.4	0.12
GAIF01009477.1	49.66	31.04	17.63	15.53	17.68	2.2
GAIF01009478.1	0	0	0	0	0.99	0
GAIF01009479.1	0.23	0.14	0.03	0.32	0.51	0
GAIF01009480.1	0.63	0.17	0	5.79	26.38	0.4
GAIF01009481.1	0.94	0.9	0	1.91	6.4	0.32
GAIF01009482.1	140.75	28.17	34.28	10.4	6.4	11.61
GAIF01009483.1	12.71	14.65	10.46	21.26	42.02	9.38
GAIF01009484.1	5.57	1.15	0.62	1.64	1.19	1.09
GAIF01009485.1	0	0	0	0	0.14	0
GAIF01009486.1	0	0	0.01	0	0	0
GAIF01009487.1	0.74	0.4	1.63	0.86	0.3	0.41
GAIF01009488.1	3.25	3.41	2.09	2.47	1.88	0.12

GAIF01009489.1	16.15	9.57	4.54	22.66	34.95	0
GAIF01009490.1	39.11	33.01	31.94	42.7	32.71	25.68
GAIF01009491.1	0	0	0.03	0	0.06	0
GAIF01009492.1	6.02	5.75	0.6	9.95	32.47	17.89
GAIF01009493.1	30.6	12.49	8.15	0	0.1	0.05
GAIF01009494.1	0	0	0	0	0.06	0
GAIF01009495.1	53.16	87.28	27.69	3.33	0.86	0.4
GAIF01009499.1	0	0	0	0.68	0.7	0
GAIF01009500.1	0.05	0.05	0	0.32	2.28	0
GAIF01009505.1	27.01	14.75	7.15	48.45	54.49	31.44
GAIF01009506.1	0.13	0.14	0.11	0.04	0.08	0.76
GAIF01009507.1	2.31	2.84	1.65	8.45	17.33	3.28
GAIF01009508.1	0	0	0	0.08	0.35	0
GAIF01009509.1	0.35	1.43	0.68	0.92	0.21	0
GAIF01009510.1	7.18	4.19	3.08	21.83	25.6	2.32
GAIF01009511.1	55.44	70.31	52.95	131.71	69.86	16.8
GAIF01009512.1	19.86	26.85	30.96	14.37	23.22	6.51
GAIF01009513.1	0	0	0	0	0.14	0
GAIF01009515.1	0.1	0	0	0.47	2.18	0
GAIF01009516.1	0.07	0	0	0.08	0.25	0
GAIF01009517.1	0.43	0.31	0.32	1.73	4.07	0.16
GAIF01009518.1	11.99	8.45	5.81	23.31	11.13	4.92
GAIF01009519.1	140.93	188.79	244.88	367.25	319.47	16.56
GAIF01009520.1	0	0	0	0	0.09	0.15
GAIF01009521.1	0	0	0	0	0.25	0.15
GAIF01009522.1	12.05	23.55	40.85	22.15	22.32	30.48
GAIF01009523.1	8.93	7.4	8.77	23.1	13.61	5.41
GAIF01009525.1	11.02	11.83	12.53	32.78	20.72	13.83
GAIF01009526.1	14.39	6.78	3.25	10.2	37.13	1.29
GAIF01009527.1	18.83	21.17	10.16	4.5	1.29	0.24
GAIF01009528.1	9.07	2.8	4.85	4.74	5.42	20.1
GAIF01009531.1	0	0	0	0	0.17	0
GAIF01009532.1	0.65	0.2	0	4.83	11.89	0.61
GAIF01009533.1	0.68	0	0	0.61	0.99	0
GAIF01009534.1	15.96	22.2	25.03	97.8	52.48	11.77
GAIF01009535.1	45.47	36.71	33.81	13.58	9.75	4.96
GAIF01009536.1	30.88	36.94	30.13	50.64	44.75	34.98
GAIF01009538.1	0	0	0.26	0.27	0.56	0
GAIF01009541.1	269.9	151.77	47.37	358.21	225.22	189.99
GAIF01009543.1	2.82	0.4	0	3.8	17.72	8.22
GAIF01009545.1	0	3.38	4.37	0	0	0
GAIF01009547.1	0	0	0	0	0.11	0
GAIF01009549.1	1104.1	312.47	63.59	1067.42	472.34	67.25
GAIF01009550.1	0	0.16	0	0.17	0	0

GAIF01009551.1	55.62	37.5	76.77	66.55	34.12	31.14
GAIF01009552.1	3.97	5.33	3.08	9.22	5	0
GAIF01009553.1	3.68	4.93	2.43	6.3	6.98	0.8
GAIF01009554.1	10.68	10.66	13.68	63.57	76.73	6.85
GAIF01009557.1	0	0.04	0.13	0.22	0.91	0
GAIF01009558.1	5.02	8.52	5.78	1.85	1.32	0.13
GAIF01009559.1	0	0	0	0	0.18	0
GAIF01009560.1	10.84	11.96	13.27	31.5	18.1	8.68
GAIF01009563.1	0.88	1.41	0.61	1.78	0.56	0.56
GAIF01009564.1	0.14	0.69	0.08	0	0	0
GAIF01009565.1	0.64	0.56	0.28	0.44	0.77	0
GAIF01009566.1	4.32	2.81	2.25	14.29	16.04	0.86
GAIF01009567.1	0	0	0.08	0	0	0
GAIF01009568.1	127.86	139.67	116.02	120.21	82.92	19.68
GAIF01009571.1	4.61	5.54	7.6	4.05	2.16	3.04
GAIF01009573.1	36.99	13.79	24.43	13.05	3.57	19.32
GAIF01009574.1	0.48	0	0.09	0.09	0.29	0
GAIF01009577.1	11.72	23.44	13.83	45.41	22.73	4.26
GAIF01009578.1	4.22	7.75	5.79	3.83	2.22	1.02
GAIF01009583.1	0	0	0	0.07	0	0
GAIF01009584.1	0	0	0	0.05	0	0
GAIF01009585.1	15.84	21.56	31.24	35.32	24.99	11.72
GAIF01009587.1	0.46	0.18	0.23	0.39	2.39	0.13
GAIF01009588.1	11.54	14.38	6.61	8.51	4.3	1.73
GAIF01009589.1	0.13	0.27	0	6.56	13.9	0
GAIF01009591.1	0	0.16	0	0.64	1.99	0.19
GAIF01009592.1	0	0	0	0	0.15	0
GAIF01009594.1	0.04	0	0	0	0	0
GAIF01009595.1	0.22	0.12	0	1.46	8.01	2.41
GAIF01009596.1	3.31	0.86	0.52	2.56	0	0
GAIF01009597.1	7.6	1.4	0.71	16.04	33.57	12.69
GAIF01009598.1	0.19	0.13	0.32	0.2	0.73	0.13
GAIF01009599.1	79.18	39.58	72.5	82.27	37.03	42.72
GAIF01009604.1	7	10.6	3.3	64.13	52.55	1.67
GAIF01009605.1	14.23	14.96	16.99	32.66	12.22	22.4
GAIF01009607.1	60.69	38.3	14.2	70.06	120.31	35.43
GAIF01009608.1	0	0.1	0	0	0.05	0
GAIF01009609.1	2.25	0.8	0.91	8.79	9.05	2.16
GAIF01009610.1	1.7	3.24	1.73	0.97	1.2	0.07
GAIF01009611.1	0	0.33	0.14	0.69	0.71	0.13
GAIF01009612.1	8.2	0	0.29	2.76	1.75	30.18
GAIF01009613.1	0	0	0	0.14	0.37	0
GAIF01009615.1	0.21	0.16	0.05	0.37	1.16	0
GAIF01009616.1	0	0.04	0	0.04	0.29	0

GAIF01009617.1	0.11	0.24	0	0.65	5.48	0
GAIF01009619.1	0.07	0.17	0	0.34	0.37	0.14
GAIF01009620.1	2.51	2.57	2.75	7.03	4.27	1
GAIF01009621.1	1.92	0.34	0.07	1.8	28.21	1.09
GAIF01009622.1	0	0	0.09	1.37	2.59	0.17
GAIF01009623.1	0	0	0	1.08	8.24	0
GAIF01009624.1	36.43	36.91	18.51	44.22	57.69	26.34
GAIF01009625.1	0.31	0.38	0.04	1.55	4.29	0.04
GAIF01009626.1	0.27	0.22	0.23	0.24	1.24	0
GAIF01009627.1	0.3	0.33	0.33	1.05	2.37	0.33
GAIF01009628.1	0.75	0.05	0	0.94	7.49	0.04
GAIF01009629.1	99.14	461.52	563.61	5.14	9.23	13.22
GAIF01009631.1	0	0	0	0	0.04	0
GAIF01009632.1	1.36	0.11	0.32	6.66	28.53	2.89
GAIF01009634.1	1.26	0.19	0.39	2.71	13.37	22.13
GAIF01009636.1	106.27	87.61	80.59	69.87	36.79	35.76
GAIF01009637.1	0.21	0.03	0	1.68	4.76	0.17
GAIF01009639.1	28.39	24.57	15.25	131.43	72.1	11.46
GAIF01009640.1	0.35	0.16	0.25	0	0	0
GAIF01009643.1	43.3	35.32	35.44	46.7	32.89	36.71
GAIF01009647.1	0	0	0	0.05	0.57	0.09
GAIF01009648.1	0.14	0.15	0.15	0.16	0.37	0.23
GAIF01009649.1	5.21	0.83	0	1.51	2.09	0.36
GAIF01009650.1	2.1	0.26	0.15	0.2	0	0.71
GAIF01009652.1	0.1	0.05	0	0.57	2.47	0.05
GAIF01009653.1	2.32	0.72	0.18	2.5	4.78	0
GAIF01009654.1	0.13	0	0	0	0.17	0
GAIF01009655.1	284.88	366.51	465.9	193	151.49	300.8
GAIF01009656.1	0.3	0.11	0	1.16	7.75	0.53
GAIF01009657.1	1	0.21	0.37	3.5	15.83	1.93
GAIF01009660.1	0	0.12	0	0	0.14	0
GAIF01009662.1	1.31	0.65	0.23	3.53	13.65	0.66
GAIF01009663.1	3.27	1.41	0.96	6.92	7.92	1.06
GAIF01009665.1	0	0.57	0	1.22	9.95	0.58
GAIF01009666.1	0	0	0	0.06	0.2	0
GAIF01009667.1	7.64	7.13	6.24	4.22	1.92	1.33
GAIF01009668.1	0	0	0	0.06	0	0
GAIF01009671.1	48.49	503.03	198.36	37.72	46.34	37.98
GAIF01009672.1	1.85	0.74	0.69	3.5	4.75	0.7
GAIF01009673.1	2.77	1.87	0.94	2.88	7.46	0.95
GAIF01009674.1	0.08	0	0	0.32	0.8	0
GAIF01009675.1	0.08	0.14	0	0.04	0	0.09
GAIF01009677.1	0.17	0.31	0.13	0.13	1.17	0
GAIF01009679.1	3.09	8.49	0.16	7.96	24.92	0

GAIF01009680.1	0.26	0.42	0.14	1.8	1.55	0
GAIF01009681.1	38.02	58.71	123.3	44.04	35.52	25.47
GAIF01009682.1	0.07	1.46	0.29	0.16	0.32	0
GAIF01009683.1	0.12	0.04	0	0.18	1.06	0.17
GAIF01009684.1	24.27	2.25	0.3	1.69	8.66	1.22
GAIF01009685.1	0.11	0.06	0.19	0.19	0.14	0.19
GAIF01009686.1	0.08	0	0.05	0.11	0.05	0.05
GAIF01009688.1	0	0.34	0.24	0	0.6	0
GAIF01009689.1	0.59	0	0	1.08	7.42	0
GAIF01009692.1	0	0	0	0.98	2.41	0
GAIF01009693.1	0	0	0.04	0	0	0
GAIF01009694.1	0.06	0	0	1.48	0.8	0.22
GAIF01009695.1	0	0	0	0.07	0.15	0
GAIF01009696.1	0	0	0	0.08	0.63	0
GAIF01009698.1	0.1	0	0	0	0	0.11
GAIF01009700.1	0.22	0.23	0.25	0.35	0.52	0.2
GAIF01009701.1	0	0	0.19	0	0	0
GAIF01009702.1	0.16	0.18	0.18	1.12	2.14	0
GAIF01009703.1	10.09	4.45	4.58	24.81	71.15	54.3
GAIF01009704.1	32.79	23.11	67.5	35.31	23.83	49.34
GAIF01009705.1	124.4	226.63	41.87	41.71	71.55	24.52
GAIF01009710.1	0.34	0	0.53	0.4	5.93	1.58
GAIF01009711.1	0	0	0	0.42	0.59	0.69
GAIF01009712.1	0	0	0	0	0	0.9
GAIF01009714.1	3.09	0	0	7.99	34.68	17.33
GAIF01009715.1	3.97	1.63	1.45	27.95	149.7	2.04
GAIF01009718.1	0	0.1	0	0	0	0
GAIF01009719.1	0.59	0.2	0	3.71	13.22	0.22
GAIF01009720.1	18.92	8.13	20.45	8.16	8.54	24.77
GAIF01009722.1	3.82	4.39	4.43	4.67	39	0
GAIF01009725.1	0	0	0.22	0	0	0
GAIF01009727.1	0.17	0.06	0.12	0.06	0	0
GAIF01009729.1	207.25	269.77	393.81	921.99	385.44	221.18
GAIF01009732.1	0	0	0	0.62	2.89	0
GAIF01009734.1	1.49	1.11	1.18	0	1.15	0.23
GAIF01009735.1	27.7	43.81	49.56	118.37	58.56	30.61
GAIF01009736.1	0.47	0	0.17	1.99	11.43	28
GAIF01009737.1	707.21	811.73	1731.7	549.28	413.08	914.75
GAIF01009738.1	0	0	0	0.19	0.97	0
GAIF01009739.1	1.87	0.91	0.5	4.28	13.37	1.35
GAIF01009740.1	0	0	0	0.42	2.95	0.13
GAIF01009741.1	1.17	4.24	2.68	0.79	0.35	0.11
GAIF01009742.1	3.08	2.26	2.6	1.79	2.85	1.24
GAIF01009743.1	6.55	181.99	28.53	5.43	25.5	27.46

GAIF01009744.1	0.29	0.32	0.16	0.57	0.53	0.22
GAIF01009746.1	0	2	1.66	0.18	0	0.01
GAIF01009748.1	25.61	21.16	22.16	49.37	21.88	20.55
GAIF01009749.1	0.81	0	0	0	0	0.06
GAIF01009750.1	0	0.65	2.39	0	0	0.26
GAIF01009751.1	0.03	0	0.04	0.15	0.05	0
GAIF01009752.1	2.66	1.1	0.97	11.51	40.22	5.03
GAIF01009754.1	2.34	3.63	1.65	5.52	3.31	0.64
GAIF01009755.1	0.13	0.07	0.07	1.09	7.47	0.69
GAIF01009756.1	0	0	0	0.31	1.28	0
GAIF01009757.1	17.03	21.46	12.41	15.15	7.66	1.4
GAIF01009758.1	0.08	0.18	0	0	0.1	0
GAIF01009759.1	0	0	0	0.12	0.12	0.11
GAIF01009761.1	25.45	10.27	9.58	5.35	4.64	2.52
GAIF01009762.1	9.94	1.48	0.15	3.79	14.16	0.83
GAIF01009763.1	2.66	2.89	1.64	8.65	5.51	1.06
GAIF01009766.1	2.71	0	0	7.15	9.25	3.98
GAIF01009768.1	0	0.1	0.03	0.18	0.84	0.03
GAIF01009769.1	0	0	0	0.01	0	0.01
GAIF01009771.1	0	0	0	0.06	0.25	0.1
GAIF01009772.1	480.93	678.42	635.18	636.01	390.14	199.12
GAIF01009773.1	5.61	4.92	3.33	16.1	9.47	0.34
GAIF01009774.1	0	0.15	0	0.08	0.42	0
GAIF01009775.1	0.19	0.21	0.05	0	0	0
GAIF01009776.1	10.86	6.03	9.53	5.41	3.68	5.61
GAIF01009777.1	8.14	13.97	14.43	19.28	17.3	4.02
GAIF01009778.1	13.47	13.25	10.38	23.31	18.62	5.67
GAIF01009779.1	0.08	0	0	0.53	1.48	0
GAIF01009780.1	40.39	41.44	25.61	74.74	82.46	55.07
GAIF01009782.1	0	0.57	0.37	0	0	0
GAIF01009783.1	19.5	31.27	22.08	47.71	34.8	6.22
GAIF01009784.1	0	0.22	0	0.47	0.73	0.22
GAIF01009785.1	0.03	0	0	0	0	0
GAIF01009787.1	1.37	1.48	0.75	4.88	5.01	0.48
GAIF01009788.1	0.2	0.08	0	2.59	11.92	0
GAIF01009789.1	15.08	26.05	21.65	18.69	78.71	137.11
GAIF01009791.1	0	0	0	0	0.05	0
GAIF01009792.1	0.57	0.93	0.73	2.31	1.03	0
GAIF01009793.1	0.33	0.84	0.85	2.18	1.73	0.49
GAIF01009795.1	0	0	0	1.65	0.87	0
GAIF01009796.1	0.21	0	0	0	0.18	0
GAIF01009799.1	6.74	7.79	14.74	32.29	25.35	11.11
GAIF01009800.1	8.12	3.46	0.94	1.8	3.07	0.5
GAIF01009802.1	1.37	3.58	2.11	1.11	0.33	0.15

GAIF01009804.1	0	0	0	0	0.04	0
GAIF01009805.1	0.3	0.22	0.66	0.06	0	0.06
GAIF01009806.1	0.1	0.1	0.1	0.27	2.56	0
GAIF01009807.1	29.76	32.35	31.01	66.88	39.41	16.4
GAIF01009808.1	0.08	0.17	0	0.09	0	0
GAIF01009810.1	0.03	0.03	0	0	0.11	0
GAIF01009811.1	0.11	0	0.12	0.12	0.16	0
GAIF01009812.1	0	0	0	0	0.01	0
GAIF01009813.1	33.06	28.15	17.8	169.52	251.54	68.97
GAIF01009814.1	70.8	78.38	70.03	116.26	67.9	21.24
GAIF01009816.1	6.96	5.4	0	23.16	17.37	0.87
GAIF01009817.1	0	0	0	0	0.62	0
GAIF01009819.1	0.14	0.23	0.08	0.21	0.25	0
GAIF01009820.1	35.56	24.54	30.33	64.76	35.03	54.56
GAIF01009821.1	0.78	0.34	0.07	4.59	21.52	0.36
GAIF01009823.1	59.57	45.01	18.88	7.23	6.36	0
GAIF01009824.1	14.72	33.73	14.95	42.47	70.81	4.17
GAIF01009826.1	0	0	0	0	0	0.14
GAIF01009827.1	0.25	0.15	0.24	0.18	1.96	0.09
GAIF01009828.1	0	0	0	0	0.61	0.25
GAIF01009829.1	83.83	56.81	0	89.08	102.47	51.02
GAIF01009830.1	4.02	0.57	1.06	8.94	12.63	3.28
GAIF01009831.1	0	7.58	0	0	0	0
GAIF01009832.1	18.76	12.89	20.43	48.42	19.49	24.97
GAIF01009833.1	0.54	0	0	0.37	2.23	0.09
GAIF01009836.1	3.39	0	1.85	5.83	1.18	8.09
GAIF01009837.1	12.18	8.76	11.13	1.84	0.59	1.5
GAIF01009838.1	0	0	0	0	1.51	0
GAIF01009839.1	0.98	0.4	0	0	0.15	0
GAIF01009840.1	1.51	1.16	1.73	1.77	1.29	0.45
GAIF01009842.1	0	0	0	0.54	1.48	0.17
GAIF01009843.1	5.46	2.94	4.11	12.39	35.06	15.56
GAIF01009844.1	157.39	171.41	199.77	105.01	96.62	178.33
GAIF01009847.1	0	0.26	0	0.22	0.94	0
GAIF01009848.1	0	0.5	0.19	0.34	1.2	0
GAIF01009849.1	4.49	8.57	3.72	10.41	25.12	0.52
GAIF01009850.1	0	0.1	0	0	0	0
GAIF01009851.1	0	0	0	0	0.01	0
GAIF01009853.1	0	0	0.06	0	0	0.04
GAIF01009855.1	302.59	1.21	3.66	12.62	0.67	0.73
GAIF01009856.1	0	0	0	0.17	0.36	0
GAIF01009857.1	0	0	0	0	0.66	0
GAIF01009858.1	0.07	0	0	0.31	0.16	0
GAIF01009859.1	0	0.15	0	0	0	0

GAIF01009860.1	3.93	6.88	8.94	15.57	13.81	3.26
GAIF01009861.1	0.32	0	0	0.18	0.38	0
GAIF01009862.1	29.64	12.38	14.69	54.87	37.31	40.8
GAIF01009863.1	19.58	8.75	13.2	21.5	16.9	13.34
GAIF01009864.1	7.36	8.99	6.16	0.36	1.28	0.37
GAIF01009865.1	1.69	0	0.14	3.18	8.78	2.58
GAIF01009869.1	16.45	27.93	11.99	68.22	90.53	7.91
GAIF01009870.1	0.09	0.1	0	0.27	0.11	0
GAIF01009871.1	0.19	0	0	0	0	0.13
GAIF01009872.1	11.12	32.75	30	60.54	55.91	5.97
GAIF01009873.1	0	0	0	0	0.12	0
GAIF01009874.1	20.42	27.69	21.19	54.88	51.36	24.56
GAIF01009877.1	0	1.45	0.33	0.9	0.43	0.38
GAIF01009878.1	0.1	0	0.11	0	2.74	0
GAIF01009879.1	5.88	8.74	7.83	17.52	8.53	0.78
GAIF01009880.1	0	0	1.75	0.06	0	0.05
GAIF01009881.1	0	0	0	0.48	4.95	0
GAIF01009882.1	0	0	0.82	0	0	0.12
GAIF01009883.1	0	0.08	0.08	0	0	0
GAIF01009884.1	3.21	0.38	0.96	10.19	16.92	1.36
GAIF01009885.1	0.15	0	0	0.6	1.52	0
GAIF01009886.1	0	0	0	0.1	0	0
GAIF01009887.1	0	0	0.36	0.25	2.99	0
GAIF01009889.1	1.71	0.86	0.72	2.73	4.05	0.43
GAIF01009890.1	0	0	0	0.11	1.06	0
GAIF01009892.1	0	0	0	0	0.54	0
GAIF01009893.1	4.76	5.33	4.81	17.89	17.75	1.98
GAIF01009894.1	124.82	102.52	29.5	363.14	689.61	49.82
GAIF01009895.1	0	0	0	0.18	0.4	0
GAIF01009896.1	2.81	3.08	2.47	1.21	2.42	0.37
GAIF01009899.1	0	0.41	0	0	0	0
GAIF01009900.1	0.21	0.91	0.81	0.73	0.63	0.69
GAIF01009901.1	1.57	1.49	2.15	2.96	8.49	0.43
GAIF01009902.1	6.4	8.43	6.95	19.47	10.28	2.04
GAIF01009903.1	21.57	31.84	53.78	5.13	6.46	27.9
GAIF01009904.1	12.67	16.51	7.91	9.97	3.96	0
GAIF01009910.1	0.12	0	0	0	0	0
GAIF01009911.1	0	0.03	0.08	0	0.04	0
GAIF01009912.1	23.29	48.66	77.88	33.2	18.6	17.14
GAIF01009913.1	0	0	0	0	0.48	0
GAIF01009914.1	9.47	0.98	0.34	26.23	59.01	10.01
GAIF01009915.1	0.68	0.98	0.25	4.97	12.39	0.41
GAIF01009916.1	0	0	0	0	0.08	0
GAIF01009917.1	836.65	645.65	197.13	0	0.25	7.53

GAIF01009918.1	0	0	0	25.7	0	0
GAIF01009919.1	0.18	0.2	0.16	0.04	0	0
GAIF01009920.1	50.29	39.97	16.81	127.22	192.54	70.76
GAIF01009921.1	0	0	0	0.64	0.17	0.28
GAIF01009922.1	20.29	13.01	5.52	0	0.21	0
GAIF01009923.1	0	0	0	0.55	3.32	0
GAIF01009924.1	4.87	4.76	2.73	12.08	24.05	0
GAIF01009925.1	0	0.1	0.1	0.11	0.11	0
GAIF01009927.1	0.13	0	0.07	1.12	9.76	0.07
GAIF01009929.1	0	0	0	3.04	5.27	0
GAIF01009930.1	0.1	0.34	0.23	1.46	4.28	0.46
GAIF01009931.1	17.48	11.91	6.91	18.61	19.71	1.58
GAIF01009932.1	1.17	2.79	3.61	0	2.51	6.41
GAIF01009933.1	0	0	0	0.68	1.26	0
GAIF01009934.1	0.08	0	0	2.38	6.2	1.53
GAIF01009935.1	0	22.25	24.36	0	0	7.64
GAIF01009936.1	76.03	37.74	44.26	96.96	78.1	174.55
GAIF01009937.1	0.38	0.24	0.34	9.71	46.82	0.97
GAIF01009938.1	0.77	0	0	0	1.23	0
GAIF01009939.1	1.85	10.97	13.11	18.06	27.35	8.93
GAIF01009940.1	0	0	0	0	0.07	0
GAIF01009941.1	0.28	0.55	0.12	1.62	5.72	0.37
GAIF01009942.1	0.08	0	0	0.1	0	0
GAIF01009944.1	0.49	0	0	4.83	6.67	0
GAIF01009945.1	0	0.28	0.11	0	0.04	0.07
GAIF01009947.1	0.53	0	0.49	2.64	6.82	0.18
GAIF01009949.1	0.03	0	0	0	0	0
GAIF01009950.1	7.38	0	0.51	12.58	0.83	11.43
GAIF01009954.1	0	0.18	0	0	0	0
GAIF01009955.1	53.79	8.43	9.18	33.62	24.36	23.16
GAIF01009958.1	2.43	1.92	0	8.44	27.9	0.16
GAIF01009962.1	6.34	8.11	9.74	12.26	3.89	4.81
GAIF01009965.1	0	0	0	0	0.03	0
GAIF01009968.1	33.41	0	1.78	16.92	3.62	30.12
GAIF01009969.1	1.05	1.21	3.87	3.86	1.29	2.68
GAIF01009970.1	0.46	0.1	0	1.49	4.84	0
GAIF01009971.1	0.96	0.85	1.53	0.35	0.54	1.13
GAIF01009972.1	0	0.08	0	0.18	1.29	0.17
GAIF01009973.1	20.92	15.89	8.99	53.44	73.04	14.24
GAIF01009974.1	20.41	31.51	16.86	57.61	31.22	9.51
GAIF01009975.1	2.01	2.27	1.48	3.12	1.44	0.16
GAIF01009976.1	2.24	1.77	0	6.17	21.41	1.22
GAIF01009977.1	14.36	22.15	20.31	32.18	26.75	10.87
GAIF01009978.1	0.92	0.12	0.1	4.74	7.08	1.28

GAIF01009979.1	0	0.01	0	0	0	0
GAIF01009980.1	0	0.12	0	0	0.37	0
GAIF01009982.1	55.84	21.39	55.22	18.33	3.33	4.58
GAIF01009983.1	0	0	0	0	0.03	0.06
GAIF01009984.1	1.34	0.13	0	0.81	3.7	0.06
GAIF01009985.1	487.31	398.15	724.38	90.04	2.48	195.07
GAIF01009986.1	0	0	0.07	1.35	4.49	0.27
GAIF01009987.1	0.23	0.08	0	1	1.69	0.34
GAIF01009988.1	115.64	139.48	55.44	188.17	167.27	14.73
GAIF01009989.1	0.41	0	0	0.51	0.77	0.69
GAIF01009991.1	37.31	59.61	39.89	8.23	42.15	4.06
GAIF01009993.1	0	0.12	0.25	0	0	0
GAIF01009995.1	1.13	2.92	0.47	7.31	7.49	0
GAIF01009997.1	21.91	15.69	16.87	58.16	50.14	7.8
GAIF01009998.1	3.95	3.67	6.25	3.18	7.36	3.58
GAIF01009999.1	2.11	0	0	1.32	0.68	1.57
GAIF01010000.1	0	1.11	0	0	4.25	0
GAIF01010001.1	36.55	13.44	21.48	43.26	40.83	186.15
GAIF01010002.1	19.78	7.83	10.45	24.45	23.64	13.01
GAIF01010006.1	0	0	0	0.04	0	0
GAIF01010007.1	0.18	0	0	0.82	4.36	0
GAIF01010009.1	0	0	0	0	0.35	0
GAIF01010010.1	5.33	0.88	3.5	11.58	0.86	3.88
GAIF01010011.1	0	0	0	0	0	0.22
GAIF01010012.1	85.09	109.3	75.92	100.2	101.2	20.88
GAIF01010014.1	0.35	0.03	0.03	1.94	16.44	0.29
GAIF01010015.1	150.7	152.49	155.27	96.28	58.63	47.91
GAIF01010017.1	0	0	0	0	1.05	0
GAIF01010018.1	0.09	0	0	0.37	0	0
GAIF01010019.1	0	0	0	0	0	0.19
GAIF01010020.1	1.67	1.11	0.36	5.23	12.46	0.92
GAIF01010021.1	0.11	0.12	0.12	0.52	1.48	0.12
GAIF01010025.1	0	0	0	0	0.17	0.15
GAIF01010026.1	0	0	0.36	0	0	0.09
GAIF01010027.1	2.78	0.49	0	1.04	0	0
GAIF01010028.1	64.32	58.52	107.82	104.98	48.79	82.72
GAIF01010034.1	81.42	50.9	42.01	135.99	80.82	91.86
GAIF01010035.1	0	0	0	0.96	31.43	0.07
GAIF01010038.1	7.1	7.87	3.03	0	19.63	2.6
GAIF01010040.1	0	1.28	0	0.84	0.75	0.84
GAIF01010041.1	0.08	0.09	0	0.38	1.46	0
GAIF01010043.1	12.68	3.61	1.2	1.27	2.63	4.64
GAIF01010046.1	4.61	2.4	0.22	8.38	14.95	8.4
GAIF01010048.1	0	0	0	0.8	1.07	0

GAIF01010053.1	4.55	5.31	5.92	1.94	13.26	8.79
GAIF01010054.1	5.02	12.23	4.29	4.74	3.5	0.2
GAIF01010055.1	0.26	0	0	0.45	1.7	0
GAIF01010057.1	0.8	0	0.61	7.35	1.26	1.63
GAIF01010059.1	2.48	3.16	1.07	6.76	13.74	2.5
GAIF01010060.1	17.01	17.52	17.63	41.34	27.86	9.99
GAIF01010061.1	0	0	0	0	0.48	0
GAIF01010063.1	0.64	0.26	0	8.54	73.71	0.18
GAIF01010064.1	0	0	0	0	0.01	0
GAIF01010065.1	0	0	0	0.91	4.08	0.84
GAIF01010066.1	11.27	13.03	23.02	24.2	11.71	7.98
GAIF01010067.1	1.58	0.34	0.35	0	0	0
GAIF01010068.1	0	1.18	0.11	0	0.08	0.07
GAIF01010069.1	13.78	11.59	18.35	7.46	1.55	3.44
GAIF01010070.1	0.91	0.73	1.12	2.55	35.68	2.48
GAIF01010071.1	0	0.46	0	1.14	2.7	0.15
GAIF01010072.1	0.98	0.11	0.75	5.17	16.82	7.84
GAIF01010073.1	0.56	0.33	0.64	1.97	2.55	0.17
GAIF01010074.1	0.46	0.12	0	1.27	9.27	0.34
GAIF01010075.1	0.08	0	0.08	0.22	0	0.08
GAIF01010076.1	16.56	12.98	19.37	59.69	25.62	18.84
GAIF01010077.1	0	0	0	0	0.29	0
GAIF01010078.1	0.23	0	0.08	0.89	3.51	0
GAIF01010080.1	75.01	158.19	239.44	159.83	128.52	64.82
GAIF01010082.1	0	0	0	0	0.05	0
GAIF01010083.1	0	0.13	0	0.43	1.1	0
GAIF01010084.1	9.3	9.01	11.06	14.88	17.15	5.94
GAIF01010086.1	0	0.03	0	0	0.04	0
GAIF01010088.1	0	0.09	0.28	0	0	0
GAIF01010091.1	49.19	21.63	9.92	64.48	71.37	80.1
GAIF01010092.1	0.13	0.14	0.72	1.53	0.47	1.6
GAIF01010093.1	0.07	0.31	0	0.25	0.52	0
GAIF01010095.1	2.24	1.21	0	1.53	0.84	2.78
GAIF01010096.1	54.21	22.39	60.45	75.09	45.75	14.71
GAIF01010098.1	0.5	0.55	0.21	0	0	0.14
GAIF01010099.1	0.27	0	0	1.55	1.93	0
GAIF01010100.1	83.98	41.87	17.05	152.25	169.71	15.48
GAIF01010101.1	0.36	0	0.13	0.83	3.86	0.13
GAIF01010102.1	0.07	0	0	0	0	21.57
GAIF01010105.1	0.18	0.17	0.27	7.13	5.06	0.19
GAIF01010106.1	0	0	0	0	0.25	0
GAIF01010107.1	16.51	13.51	6.24	46.42	47.52	3.33
GAIF01010108.1	0.44	0.52	0.19	2.68	8.44	0.19
GAIF01010109.1	0.2	0	0	0	0	0

GAIF01010110.1	1.42	0.83	0.18	1.58	3.45	8.24
GAIF01010111.1	1.21	0.27	0.07	5.54	16.52	1.94
GAIF01010112.1	0.13	0.17	0.11	0	0	0.19
GAIF01010113.1	0.55	0.13	0.13	1.5	1.48	0.67
GAIF01010115.1	7.13	0.16	0.17	13.24	23.95	3.97
GAIF01010116.1	16.63	27.17	18.18	112.47	57.52	10.75
GAIF01010118.1	40.09	15.82	12.37	49.51	60.07	51
GAIF01010119.1	0	0	0	0.46	0.24	0
GAIF01010122.1	36.6	26.46	15.84	60.67	17.1	12.32
GAIF01010123.1	6.44	3.41	6.01	14.44	17.09	58.9
GAIF01010124.1	0	0.06	0.06	0.68	1.73	0.08
GAIF01010125.1	0.23	0.68	0.38	0.46	0.07	0
GAIF01010126.1	0.09	0.02	0.02	0.95	10.31	0.03
GAIF01010127.1	7.5	6.12	2.06	10.26	16.59	0.59
GAIF01010130.1	7.43	4.47	3.71	22.99	17.97	6.24
GAIF01010131.1	67.8	93.12	129.05	81.41	78.13	290.48
GAIF01010133.1	0.17	0.06	0.06	0.59	1.5	1.31
GAIF01010135.1	0.16	0.17	0	0.46	1.42	0
GAIF01010136.1	0.76	0.05	0.2	1.97	7.48	0.3
GAIF01010138.1	18.63	17.87	9.28	33.79	45.05	2.8
GAIF01010139.1	0.4	0	0	0.36	2.18	0.09
GAIF01010140.1	0.34	0	0.09	0.1	0	0.09
GAIF01010141.1	0.29	0.84	0.86	0.83	1.41	0.21
GAIF01010143.1	0	0.06	0	0	0	0
GAIF01010148.1	0.69	0.37	0.53	0.4	0.62	0
GAIF01010150.1	0	0	0	0.26	0.71	0.24
GAIF01010151.1	8.89	13.05	9.36	21.02	13.16	4.27
GAIF01010152.1	20.08	26.04	23.67	33.19	21.01	13.43
GAIF01010153.1	9.03	1.2	0.27	2.66	15.67	0
GAIF01010154.1	9.08	11.41	7.09	41.4	28.72	10.13
GAIF01010155.1	0	0	0	0.09	0	0
GAIF01010156.1	16.24	9.04	4.76	33.12	46.07	27.4
GAIF01010157.1	4.38	2.15	2.17	0.99	0.58	0.29
GAIF01010158.1	0	0	0	0.96	0.5	0
GAIF01010160.1	0	0.68	0	0	0	0
GAIF01010163.1	0.11	0	0	1.48	8	1.39
GAIF01010164.1	0.87	0.79	0.64	1.7	1.25	0.41
GAIF01010165.1	0.09	0.05	0	0.1	0.26	0
GAIF01010166.1	38.11	56.21	54	23.92	21.71	19.04
GAIF01010167.1	123.83	101.68	81.07	153.75	151.37	247.75
GAIF01010168.1	5.63	8.96	4.99	13.56	13.56	2.29
GAIF01010169.1	3.08	2.66	4.6	6.84	10.35	2.83
GAIF01010170.1	9.81	5.91	3.81	9.1	11.32	2.81
GAIF01010171.1	0	0	0	1.6	1.76	0

GAIF01010172.1	0.03	0.11	0.15	0.12	0.13	0.02
GAIF01010173.1	1.35	1.93	0.15	9.22	25.57	1.99
GAIF01010175.1	0.1	0	0	0.12	0.25	0
GAIF01010177.1	43.06	65.62	0	79.05	31.51	15.92
GAIF01010178.1	0.61	0.48	0	5.64	21.76	0.86
GAIF01010179.1	53.2	47.46	45.26	44.52	20.17	10.51
GAIF01010180.1	0	0	0	0.19	0	0
GAIF01010181.1	1591.48	1079.36	717.98	937.05	541.1	443.71
GAIF01010182.1	2.58	2.98	1.03	1.45	1.22	0.17
GAIF01010183.1	0.97	0.44	0	3.9	13.71	1.51
GAIF01010184.1	91.17	64.7	37.79	69.44	64.5	15.18
GAIF01010186.1	436.26	1112.84	675.69	41.22	142.04	134.13
GAIF01010188.1	10.11	9.02	10.96	37.92	24.32	21.58
GAIF01010189.1	4.39	4.86	3.73	11.01	14.62	4.73
GAIF01010190.1	0	0.15	0	0	0	0
GAIF01010191.1	0.14	0	0	5.97	1.46	1.34
GAIF01010194.1	0.09	0.28	0	0.8	0.52	0.19
GAIF01010195.1	23	30.82	41.29	68.9	28.4	28.86
GAIF01010196.1	0.05	0	0	0	0	0
GAIF01010197.1	1.11	0.99	0.86	0.72	0.54	0.25
GAIF01010199.1	7.1	3.07	3.76	4.66	0	1.27
GAIF01010200.1	0	0	0	0	0.33	0
GAIF01010201.1	0	0	0	0	0.09	0
GAIF01010202.1	0	0.06	0	0	0	0
GAIF01010203.1	10.66	8.07	13.36	26.43	16.46	28.78
GAIF01010204.1	0	3.89	0	0	0.1	0
GAIF01010205.1	0.2	0	0.11	0.92	2.16	0.33
GAIF01010206.1	0.23	0	0	0	0.14	0
GAIF01010207.1	15.41	16.63	32.46	69.55	36.92	21.26
GAIF01010208.1	0.06	0.13	0.07	0.28	0.39	0
GAIF01010209.1	1.64	2.95	4.12	0.61	0.61	0.4
GAIF01010210.1	22.77	21.66	6.38	90.94	90.22	3.86
GAIF01010211.1	0	0	0.14	0	0	0
GAIF01010212.1	0	0	0	0	0.1	0
GAIF01010215.1	1.44	0.26	1.32	0.56	0.72	0.13
GAIF01010216.1	0	0	0	0.05	0.1	0
GAIF01010217.1	0.06	0	0	0.92	1.43	0.47
GAIF01010219.1	0	0	0	0.34	0.47	0
GAIF01010222.1	0	0	0	0.12	0.24	0
GAIF01010224.1	17.42	16.43	22.87	38.88	36.43	12.71
GAIF01010225.1	0	0	0	0	0.28	0
GAIF01010226.1	0	0	0	0.15	0.31	0
GAIF01010228.1	1.75	3.12	0.98	6.83	5.62	1.98
GAIF01010229.1	0.41	0.09	0.15	0.16	0.13	0

GAIF01010230.1	0.36	0	0.11	1.34	5.93	0
GAIF01010231.1	9.4	7.6	13.27	16.49	11.37	51.08
GAIF01010232.1	6.81	6	10.06	18.97	8.64	7.09
GAIF01010233.1	0.1	0	0	0.3	0.35	0.04
GAIF01010235.1	28.22	25.01	40.17	25.49	8.53	11.67
GAIF01010236.1	0	0	0	0.04	0.15	0
GAIF01010237.1	0	0	0	0	0.05	0
GAIF01010238.1	0	0	0	0	0	0.1
GAIF01010239.1	0.99	0.06	0	0	1.46	0.36
GAIF01010241.1	0	0.08	0.16	0	0	0
GAIF01010242.1	4.64	3.78	1.34	8.48	10.04	1.53
GAIF01010243.1	1.16	0.6	0	0.57	0.99	0
GAIF01010244.1	0.14	0	0	0	0.19	0.03
GAIF01010246.1	12.5	25.12	25.33	31.51	25.92	4.1
GAIF01010247.1	0	0	0	0	0.47	0
GAIF01010248.1	6.77	6.27	4.58	27.05	37.99	9.95
GAIF01010249.1	73.18	107.79	95.34	19.78	34.01	19.04
GAIF01010250.1	2.42	0	2.59	1.86	3.08	0.6
GAIF01010251.1	7.29	12.56	11.78	11.66	4.55	5.08
GAIF01010252.1	3.16	1.5	1.41	7.33	5.7	1.41
GAIF01010253.1	0.17	0.06	0	0.13	0.14	0
GAIF01010255.1	0	0.36	0.12	0	0.26	0.12
GAIF01010256.1	0.04	0.04	0	0.13	0.26	0.04
GAIF01010257.1	5.45	2.07	2.88	12.18	6.8	1.26
GAIF01010258.1	4.05	2.46	1.31	1.52	0.43	0.52
GAIF01010259.1	13.67	15.81	10.63	69.34	81.81	2.26
GAIF01010260.1	0.57	1.4	0.38	1.5	0.91	0.13
GAIF01010261.1	0	0	0	0	0.99	0
GAIF01010262.1	0.33	0.36	0.85	0.13	0.4	0
GAIF01010263.1	0.25	0.05	0	1.71	10.76	0
GAIF01010265.1	46.74	27.81	27.26	0	0	0
GAIF01010266.1	0.09	0.1	0	0.89	2.54	0.3
GAIF01010268.1	5.91	12.85	6.18	19.03	17.03	1.91
GAIF01010269.1	7.87	0.33	0.25	19.6	103.64	68.87
GAIF01010271.1	1.09	2.08	1.8	0.95	0.49	0.6
GAIF01010272.1	0	0	0.13	0	0	0
GAIF01010273.1	0.08	0	0	0	0	0
GAIF01010275.1	0	0.04	0.04	0	0	0
GAIF01010276.1	0.02	0.03	0	0	0.17	0
GAIF01010277.1	0	0.14	0.14	0.26	0	0
GAIF01010279.1	4.72	1.1	0.11	31.45	71.91	4.15
GAIF01010280.1	16.5	18	15.28	29.43	42.6	34.62
GAIF01010281.1	0	0.17	0	0.09	0.37	0
GAIF01010282.1	0.05	0	0	0	0	0

GAIF01010284.1	0	0	0	2.64	2.15	0.51
GAIF01010285.1	78.43	17.74	27.66	110.18	108.87	102.02
GAIF01010286.1	0	0	0.16	0	0	0.08
GAIF01010287.1	0	0	0	0	0.06	0
GAIF01010288.1	0.08	0	0	0.09	0.19	0
GAIF01010289.1	11.42	9.91	14.14	15.32	10.09	9.56
GAIF01010291.1	0.87	0.3	0.55	0.42	0.77	0.5
GAIF01010293.1	6.8	3.64	0.61	1.16	1.88	0.12
GAIF01010294.1	0	0.32	0	0	0.4	0.01
GAIF01010297.1	4.41	1.38	1.06	8.17	9.7	2.93
GAIF01010298.1	0	0.17	0	0.43	3.02	0
GAIF01010299.1	0	0.74	0	0	0	0.25
GAIF01010300.1	36.52	37.79	15.46	22.17	22.9	1.85
GAIF01010301.1	0	0	0	0	1.7	0
GAIF01010302.1	47.98	59.6	80.77	131.98	55.18	70.26
GAIF01010303.1	0	0.1	0	0	0.11	0
GAIF01010304.1	0.19	0.02	0	0.62	2.47	0.11
GAIF01010305.1	0	0	0	0.16	2.27	0
GAIF01010307.1	11.42	11.54	7.22	6.16	3.95	0.95
GAIF01010308.1	20.94	10.89	9.66	4.23	1.22	0.22
GAIF01010309.1	4.27	8.16	5.5	32.81	15.58	2.19
GAIF01010310.1	124.14	161.99	219.43	192.62	136.16	48.59
GAIF01010311.1	16.93	17.68	13.83	73.22	47.48	9.54
GAIF01010312.1	0	0	0	0.12	0	0
GAIF01010313.1	0	0.87	0.16	0	0	0.24
GAIF01010314.1	0.52	0	0	0	0	0
GAIF01010315.1	1.18	3.74	3.72	6.97	4.27	0.4
GAIF01010317.1	0.62	0.54	1.23	2.61	9.9	23.51
GAIF01010318.1	6.94	5.1	7.12	12.02	17.6	15.94
GAIF01010319.1	19.06	11.04	9.19	3.51	1.5	0.79
GAIF01010320.1	3.93	7.52	2.94	12.59	9.65	0.87
GAIF01010321.1	0.14	1.44	1.36	0.06	0.23	0.3
GAIF01010323.1	0.04	0.04	0	0	0.39	0.04
GAIF01010324.1	38.53	51.35	49.68	157.74	184.54	27.45
GAIF01010325.1	0.98	1.68	0	2.41	2.75	0.82
GAIF01010326.1	2.59	3.22	2.87	3.25	5.1	2.03
GAIF01010327.1	0	0	0	2.1	0	0
GAIF01010328.1	1.35	1.89	1.17	10.22	8.5	0.62
GAIF01010330.1	0.07	0	0.08	0	0	0
GAIF01010331.1	1.28	1.02	2.06	2.68	10.28	1.51
GAIF01010333.1	0.26	0	0.29	0	0	0
GAIF01010335.1	1.7	0.85	0.3	6.49	11.09	0.2
GAIF01010336.1	0	0	0	0.12	0.74	0
GAIF01010337.1	0.11	0	0	0	0	0

GAIF01010338.1	0.21	0	0	0.41	1.27	0
GAIF01010339.1	13.34	69.14	0	19.45	89.75	122.26
GAIF01010342.1	0.19	0.1	0	0.26	1.82	0
GAIF01010343.1	0.19	0.37	0.21	1.85	1.74	0.11
GAIF01010345.1	0.25	1.23	0.69	0.15	0.3	0
GAIF01010346.1	6.69	4.8	2.8	6.15	5.04	3.92
GAIF01010349.1	0	0	0	0.05	0	0
GAIF01010350.1	1.81	1.65	0.18	5.57	13.16	0.05
GAIF01010351.1	18.42	12.59	11.14	1.41	0.27	0.24
GAIF01010352.1	6.67	3.79	0.85	4.51	9.13	0.2
GAIF01010353.1	25.4	12.48	14.93	6.29	1.66	1.29
GAIF01010354.1	0	0.23	0.34	0	0	0
GAIF01010355.1	0.3	0.11	0	0.23	0.36	0.66
GAIF01010356.1	0.14	0	0.47	0.17	0	0
GAIF01010357.1	0	0	0	0	0.13	0
GAIF01010358.1	0	0	0.05	0.05	0.74	0.24
GAIF01010359.1	0	0	0	0.05	0.1	0
GAIF01010360.1	0	0	0	0.33	0.45	0.1
GAIF01010361.1	1453.98	411.71	656.75	720.16	1136.34	4895.06
GAIF01010362.1	0	0	22.05	0	0	0.03
GAIF01010363.1	0.22	0.36	0	1.55	6.14	0.98
GAIF01010364.1	18.47	15.71	14.75	28.42	22.35	10.5
GAIF01010366.1	0	0	0.39	0	0	0.13
GAIF01010367.1	0	0.32	0	1.02	3.68	0
GAIF01010369.1	2.11	1.44	0.61	2.28	0.7	0.21
GAIF01010370.1	2.07	1.98	3.18	4.23	3.98	7.21
GAIF01010371.1	0.68	0.43	0.18	2.65	7.6	0.22
GAIF01010373.1	1.2	1.1	1.31	0.96	0.22	0.1
GAIF01010375.1	20.89	26.11	13.99	34.86	22.66	5.37
GAIF01010376.1	0	0	0	0	0	0.02
GAIF01010377.1	0	0	0	0	0	0.08
GAIF01010378.1	0	0	0	0	0.14	0
GAIF01010381.1	0	0.08	0	0.08	0	0.15
GAIF01010382.1	3.32	1.85	0.16	7.63	12.08	0.55
GAIF01010383.1	0.83	0.06	0	1.61	4.48	2.09
GAIF01010384.1	0.14	0.02	0.01	0.68	6.66	0.66
GAIF01010385.1	0.21	0.62	0	1.11	1.1	0.13
GAIF01010386.1	0.17	0.09	0	3.1	6.42	0.66
GAIF01010387.1	0.2	0.04	0	1.39	6.75	0.04
GAIF01010388.1	0.17	0.15	0.11	0.43	1.74	0.02
GAIF01010390.1	0	0	0	0.05	0.05	0
GAIF01010391.1	0	2.45	4.75	0	0	0
GAIF01010393.1	0	0	0	0	0.13	0
GAIF01010394.1	6.15	8.63	8.42	13.7	9.38	3.2

GAIF01010395.1	13.28	15.8	15.22	8.98	8.06	3.82
GAIF01010397.1	33.25	51.25	46.96	59.64	53.19	40.18
GAIF01010398.1	21.83	2.83	28.32	9.75	30.31	0.08
GAIF01010399.1	0	0.15	0	0	0.16	0
GAIF01010400.1	0	0.35	0	0	0.78	1.81
GAIF01010403.1	0	0	0	0	0.11	0
GAIF01010405.1	25.7	17.63	14.76	67.96	87.98	8.57
GAIF01010406.1	4.11	1.22	0	24.49	77.44	20.1
GAIF01010407.1	0.3	0.13	0	0.55	1.04	0.06
GAIF01010408.1	1.3	0.4	0.2	3.46	10.86	0.92
GAIF01010409.1	0	0	0	0.11	0.6	0
GAIF01010410.1	0	11.4	0	0	0	0
GAIF01010411.1	0	0	0	0.73	3.79	0.35
GAIF01010412.1	4.2	5.32	3.79	4.51	3.12	0.89
GAIF01010413.1	0	0.19	0	0	0.11	0
GAIF01010414.1	12.07	12.39	9.38	14.35	21.63	4.9
GAIF01010416.1	59.15	37.8	91.55	135.71	174.54	328.86
GAIF01010417.1	0.91	0.1	0	0	0.05	0
GAIF01010418.1	0	0.13	0	0.68	0.79	0
GAIF01010419.1	0	0	0	0.05	0.1	0
GAIF01010420.1	0	0	0	0	0.02	0
GAIF01010422.1	0.91	0.4	0.2	0.32	0	0.1
GAIF01010425.1	0.11	0.12	0	0.39	1.35	0
GAIF01010426.1	0	0	0	0	0.43	0
GAIF01010427.1	6.54	8.62	9.13	22.4	12.74	8.01
GAIF01010428.1	0	0	0.12	0	1.23	0
GAIF01010431.1	0	0.51	0	0	0.56	0
GAIF01010433.1	8.87	7.25	10.77	4.88	5.49	7.65
GAIF01010434.1	1.37	0.92	0.53	6.09	14.56	0.87
GAIF01010435.1	72.86	42.38	28.56	96.86	97.42	19.38
GAIF01010436.1	0	0	0.25	0	0	0
GAIF01010437.1	1.33	0.07	0.07	6.04	16.28	0.61
GAIF01010438.1	0	0	0	2.69	1.26	0
GAIF01010439.1	0.77	0.17	0	1.08	4.29	8.37
GAIF01010440.1	6.11	5.09	1.82	0.43	0.13	1.22
GAIF01010441.1	0.06	0	0	0.34	0.35	0
GAIF01010442.1	0.91	0.16	0.21	0.28	0.58	0.11
GAIF01010443.1	0	0	0.03	0	0	0
GAIF01010444.1	0	0	0	0.03	0	0.05
GAIF01010445.1	0	0	0	1.46	7	0
GAIF01010449.1	57.76	34.09	14.02	156.7	183.79	19.08
GAIF01010450.1	23.37	5.03	3.18	3.22	2	1.02
GAIF01010451.1	0	0.05	0.05	0.12	0.18	0
GAIF01010454.1	0	0	0	0.36	2.82	0.04

GAIF01010455.1	0	0.27	0	0	0	0
GAIF01010456.1	2.09	0	0	7.11	20.88	2.01
GAIF01010457.1	0	0	0	0	0.12	0.11
GAIF01010458.1	6	2.51	0.13	6.03	14.57	1.91
GAIF01010459.1	6.71	9.5	12.34	55.52	25.95	11.92
GAIF01010460.1	3.13	0	0	3.63	23.17	22.03
GAIF01010461.1	0.27	0.1	0	0.2	0.84	0.29
GAIF01010463.1	0.45	0.25	0	1.57	2.85	0
GAIF01010466.1	0	0.05	0	0.05	0	0
GAIF01010467.1	2.87	1.44	1.13	4.34	1.72	0.47
GAIF01010468.1	0	0	0	0	0.05	0
GAIF01010470.1	0	0	0	0	0.2	0.05
GAIF01010472.1	87.86	115.27	157.69	117.02	113.03	102.48
GAIF01010473.1	0	0	0	0	0.45	0
GAIF01010475.1	0.66	0.18	0.27	0	1.39	1.46
GAIF01010476.1	0	0.08	0	0.17	0.53	0
GAIF01010479.1	0.28	0	0	1.64	4.42	2.39
GAIF01010480.1	39.96	37	58.73	133.21	78.45	72.42
GAIF01010481.1	115.89	137.95	341.01	92.36	77.37	234.27
GAIF01010484.1	8.71	10.6	11.13	30.32	16.1	9.02
GAIF01010485.1	12.08	6.57	5.49	23.55	18.69	2.99
GAIF01010486.1	0	4.4	1.11	0	0	0
GAIF01010487.1	0.08	0.09	0	0.09	0.1	0.09
GAIF01010490.1	1.43	0.76	0.84	11.32	6.96	1.85
GAIF01010491.1	54.86	81.42	137.46	62.85	57.66	40.69
GAIF01010492.1	0	0	0	0	0.06	0
GAIF01010494.1	0.03	0	0	0.1	0.18	0
GAIF01010495.1	3.91	0	8.36	7.71	4.92	0
GAIF01010500.1	0.06	0.07	0	0	0.07	0
GAIF01010501.1	1.8	1.45	1.45	1.85	0	0
GAIF01010502.1	2002.55	872.35	588.24	1216.12	1741.55	5343.82
GAIF01010504.1	0	3.78	1.69	1.7	1.41	0.72
GAIF01010505.1	0.33	0	0.12	1.41	2.92	1.22
GAIF01010506.1	0.21	0.15	0.08	0.24	0.33	0.08
GAIF01010507.1	5.81	0	5.81	0.55	0	4.65
GAIF01010510.1	0	0	0	0	0.15	0
GAIF01010511.1	0	0	0	0	1.26	0
GAIF01010512.1	23	27.94	26.59	22.31	15.49	6.1
GAIF01010513.1	13.83	18.44	19.54	20.41	14.42	5.06
GAIF01010514.1	0.35	0	0	2.05	3.83	0.13
GAIF01010515.1	744.69	1136.41	2853.19	1288.54	1137.95	2610.64
GAIF01010516.1	0	0.09	0	0.1	0	0
GAIF01010520.1	0	0	0	0	0.15	0
GAIF01010521.1	0	0	0	0.27	2.43	0

GAIF01010523.1	0.9	0	0.15	0.29	0.68	2.45
GAIF01010524.1	0	0	0	0	0.41	0
GAIF01010525.1	0.15	0.21	0.06	1.21	3.99	0.22
GAIF01010526.1	18.14	23.29	13.22	39.61	10.02	27.61
GAIF01010527.1	0	0	0.04	0	0.23	0
GAIF01010528.1	13.09	26.69	22.84	38.18	28.04	7.73
GAIF01010529.1	70.38	73.89	59.26	33.72	40.41	69.28
GAIF01010530.1	1.9	1.82	2.59	11.23	74.02	3.27
GAIF01010531.1	3.37	1.68	0.89	9.12	25.8	1.75
GAIF01010532.1	0	0	0	1.6	0	0.17
GAIF01010536.1	0	0	0	0.08	0.19	0
GAIF01010537.1	0.22	0.39	0.24	0	0.13	0
GAIF01010538.1	14.04	5.7	7.79	29.94	33.99	16.81
GAIF01010540.1	1.88	1.02	1.93	9.82	5.23	1.68
GAIF01010542.1	0.06	0.56	0.28	0.22	0.92	0.21
GAIF01010545.1	0.32	0	0.18	3.67	16.99	0.09
GAIF01010547.1	0.34	0	0	0.04	0.19	0.49
GAIF01010548.1	0	0	0.13	0	0	0
GAIF01010549.1	0.03	0	0	0	0.04	0.03
GAIF01010550.1	0	1.1	4.85	0	0	0.77
GAIF01010551.1	0	0	0	0	0.19	0
GAIF01010552.1	29.85	48.1	72.08	50.17	46.71	11.31
GAIF01010554.1	0.52	0.28	1.15	0.76	0.31	0.14
GAIF01010555.1	0	0	0	0	0.17	0
GAIF01010556.1	0.3	0	0	0.53	6	1.25
GAIF01010557.1	0.09	0	0	0	0	0
GAIF01010558.1	0.08	0	0	0.1	0	0
GAIF01010559.1	0	0.02	0	0	0	0
GAIF01010560.1	0.13	0	0	0	0	0.85
GAIF01010561.1	0.05	0	0	0.06	0.29	0
GAIF01010562.1	2.28	0.83	0.16	2.77	16.8	2.47
GAIF01010563.1	0	0.08	0	0	0.26	0
GAIF01010564.1	1780.16	1957.82	2867.49	1454.2	1010.3	914.16
GAIF01010565.1	0	1.62	0.37	0	0.04	0.04
GAIF01010567.1	12.14	8.17	4.7	10.2	5.56	4.08
GAIF01010568.1	0	0	0	0.03	0	0
GAIF01010569.1	3.79	3.86	3.91	8.65	3.41	4.67
GAIF01010570.1	0.41	0.31	0	0	2.67	0.46
GAIF01010572.1	0	0	0.14	0	0.77	0
GAIF01010575.1	11.09	10.31	10.11	5.89	6.24	4.17
GAIF01010576.1	0	0	0	0	0.24	0.08
GAIF01010577.1	275.57	199.02	123.44	284.77	238.84	97.85
GAIF01010579.1	0	0	0	0	0.43	0
GAIF01010582.1	0	0.24	0.58	0	0	0

GAIF01010583.1	1.85	1.81	0	6.66	14.24	0.41
GAIF01010584.1	1.15	0.27	0	0.86	2.28	0
GAIF01010585.1	0.6	0.25	0.15	0.75	2.73	0.05
GAIF01010586.1	15.09	15.68	12.99	21.97	12.91	7.45
GAIF01010587.1	0	0	0	0	0.5	0
GAIF01010588.1	0	0	0	0	0.16	0
GAIF01010590.1	5.74	9.16	4.27	21.59	27.54	1.22
GAIF01010591.1	4.68	2.2	0.99	0.31	0.32	0.42
GAIF01010592.1	48.48	39.32	28.52	107.67	78	16.43
GAIF01010593.1	3.66	0.4	0.4	7.44	26.86	0.2
GAIF01010594.1	15.17	18.17	6.74	22.68	18.66	2.33
GAIF01010598.1	8.04	8.4	9	6.91	4.74	5.03
GAIF01010599.1	0.31	0	0.16	0	0.41	0.15
GAIF01010601.1	2.14	1.21	0	2.85	5.04	0.37
GAIF01010602.1	13.62	11.3	10.1	13.68	11.76	5.3
GAIF01010604.1	0	1.02	0.41	0	0	0
GAIF01010607.1	0.61	0	0.59	3.81	0.97	3.49
GAIF01010608.1	1.97	2.27	0.97	1.08	2.97	0.3
GAIF01010609.1	0	0	0	0	0.16	0
GAIF01010610.1	0	0	0	0	0.22	0
GAIF01010615.1	0	0.42	0.19	0	0.5	0.04
GAIF01010617.1	4.43	3.18	2.69	5.66	2.83	2.01
GAIF01010618.1	25.41	25.21	16.39	46.72	30.56	19.6
GAIF01010619.1	1.25	1.26	1.37	5.68	2.57	1.27
GAIF01010620.1	0	0.1	0	0	0	0
GAIF01010624.1	0	0	0.04	0	0	0
GAIF01010625.1	7.61	13.21	5.99	12.88	13.89	4.67
GAIF01010626.1	0.4	0.25	0	0.79	0.9	0.2
GAIF01010627.1	0	0	0	0	0.26	0
GAIF01010628.1	0.05	0.02	0.02	0.13	0.11	0.15
GAIF01010629.1	0	0	0	0	0.36	0
GAIF01010630.1	0.94	0.75	1.27	0.24	0.44	0.75
GAIF01010633.1	11.02	12.95	12.84	6.17	0	1.1
GAIF01010634.1	0.11	0	0	0.12	0.25	0
GAIF01010635.1	0	0.03	0	0	0	0
GAIF01010636.1	0	0	0	0	0	0.22
GAIF01010637.1	0	0.12	0.25	0	0	0
GAIF01010638.1	0.87	0.12	0.12	0.25	0.07	0
GAIF01010639.1	0.32	0.53	0.08	1.21	3.76	0.15
GAIF01010640.1	0.04	0	0	0.08	0.39	0.07
GAIF01010642.1	0	0	0	0	0.36	0
GAIF01010643.1	9.5	7.77	6.83	9.13	5.84	4.09
GAIF01010645.1	11.4	12.44	14.88	7.98	4.69	3.99
GAIF01010646.1	1.52	1.62	1.67	3.5	3.75	3.62

GAIF01010647.1	0.54	0.69	0.5	0.11	0.11	0
GAIF01010648.1	4.84	7.18	3.26	4.09	4.9	0
GAIF01010649.1	0	0.36	0	0.55	0.86	0
GAIF01010650.1	0.63	0.17	0.51	1.18	1.13	0.17
GAIF01010651.1	194.79	155.42	233.5	612.1	330.03	1834.64
GAIF01010653.1	0.9	0	0	1.67	3.74	4.47
GAIF01010655.1	12.62	2.31	0.92	87.58	276.78	0.83
GAIF01010656.1	16.55	11.98	11.06	20.02	12.29	11.43
GAIF01010657.1	0.19	0.08	0.16	0.04	0.18	0.02
GAIF01010658.1	0	2.24	0	0	0	0
GAIF01010659.1	0.17	0.19	0	0.79	0.41	0
GAIF01010660.1	0.3	0.2	0.12	0.04	0.04	0.15
GAIF01010661.1	15.84	3.19	2.58	0	0	0
GAIF01010662.1	4.88	0.42	0.19	0	0.5	0.04
GAIF01010665.1	0.9	1.33	0.55	0.79	0.16	0
GAIF01010667.1	0.15	0.33	0	0.71	0.55	0
GAIF01010668.1	6.31	7.25	4.66	13.65	15.46	1.87
GAIF01010669.1	83.14	59.57	54.59	117.21	69.48	59.11
GAIF01010670.1	0.26	0	0	0	0	0
GAIF01010671.1	0	0	0	0	0.37	0
GAIF01010672.1	0.19	0.09	0.09	0.06	0.03	0
GAIF01010673.1	1.8	1.11	0.52	6.73	7.44	1.21
GAIF01010677.1	0.06	0	0	0.07	0.28	0
GAIF01010679.1	25.47	37.59	37.1	37.4	21.2	7.88
GAIF01010682.1	0.18	0	0	0	0	0
GAIF01010683.1	3.51	0.71	0	8.78	15.93	2.79
GAIF01010684.1	0	0	0	0.14	0	0
GAIF01010685.1	11.35	10.53	4.94	27.31	30.38	1.43
GAIF01010686.1	14.51	30.12	8.38	9.56	7.8	3.54
GAIF01010687.1	0	0.48	0.19	0	0	0.05
GAIF01010689.1	0	0	0	0	0.39	0
GAIF01010690.1	117.98	134.26	212.72	198.19	141.02	173.14
GAIF01010691.1	0.12	0	0	0.57	2.55	0.94
GAIF01010693.1	0	0	0	0	0	0.06
GAIF01010695.1	31.02	11.26	9.19	45.54	72.16	30.53
GAIF01010696.1	8.99	7	7.3	4.56	4.72	6.69
GAIF01010699.1	0.13	0	0	0.54	4.84	0
GAIF01010700.1	0.11	0.26	0.53	0.44	0.59	0.69
GAIF01010701.1	4.33	6.84	13.93	10.54	8.99	3.87
GAIF01010702.1	0	0	0	0.15	1.14	0
GAIF01010704.1	0.9	0.33	0	3.75	22.03	1.24
GAIF01010705.1	115.99	53.65	52.96	150.19	169.55	86.43
GAIF01010706.1	15.76	6.42	4.24	31.63	33.53	5.23
GAIF01010707.1	0.02	0.27	0.02	0.18	0.28	0.01

GAIF01010708.1	4.5	6.68	2.68	19.37	19.16	0.91
GAIF01010710.1	9.99	14.09	10.23	1.57	0.65	0.45
GAIF01010711.1	0.09	0.14	0.03	0.13	1.35	0
GAIF01010712.1	16.37	7.76	6.05	37.26	18.13	8.93
GAIF01010713.1	6.73	6.12	4.79	10.37	5.11	1.95
GAIF01010715.1	0.23	0	0	0.26	1.64	0
GAIF01010716.1	0	0	0	0.09	0	0
GAIF01010717.1	60.87	27.57	10.31	3.17	2.32	0.48
GAIF01010721.1	0	0	0	0.1	0.2	0
GAIF01010722.1	424.34	295.66	137.06	62.28	58.65	18.42
GAIF01010723.1	0	0	0.01	0	0	0
GAIF01010725.1	21.35	21.32	8.51	5.97	4.29	0.1
GAIF01010727.1	0.07	0.03	0	0.68	0	0.24
GAIF01010728.1	0	22.79	93.77	1.45	6.01	164.07
GAIF01010729.1	0	0.07	0	0.29	0.45	0
GAIF01010730.1	156.69	52.78	5.94	174.85	324.34	49.07
GAIF01010731.1	0.52	0	0.46	0.54	0.44	0.36
GAIF01010732.1	3.47	1.63	1.55	0.61	0.39	0.16
GAIF01010735.1	0.69	0.25	0.13	3.88	18.73	1.91
GAIF01010736.1	0.69	0.5	0.38	1.86	3.86	0.76
GAIF01010737.1	10.01	12.01	11.94	14.56	13.17	4.26
GAIF01010738.1	15.18	12.84	6.01	39.43	42.55	9.36
GAIF01010740.1	0.42	0.13	0.33	0	0.07	0
GAIF01010741.1	0.1	0.31	0	0.32	0.34	0
GAIF01010743.1	0.17	0	0	0.59	0.61	0
GAIF01010745.1	0	0	0	0	0.14	0
GAIF01010746.1	0.14	1.39	0.22	1.6	1.1	0.07
GAIF01010747.1	0.4	3.67	2.77	0	0.43	0.91
GAIF01010748.1	0.52	0.16	0.08	2.43	13.65	0.33
GAIF01010749.1	0.02	0.09	0.05	0.41	2.04	0.07
GAIF01010750.1	4.32	1.73	0.78	3.87	1.58	1.27
GAIF01010753.1	0.12	0.1	0.07	1.95	6.09	0.33
GAIF01010755.1	6.11	6.4	12.26	2.75	3.39	6.46
GAIF01010756.1	9.79	13.6	3.04	4.67	3.92	1.38
GAIF01010757.1	39.67	20.19	10.19	27.28	24.95	4.67
GAIF01010758.1	30.24	54.09	43.09	74.19	43.05	7.51
GAIF01010759.1	0.96	0.57	0.88	0.22	0	0.33
GAIF01010760.1	0.1	0	0	0	0.5	0
GAIF01010763.1	35.07	17.78	13.46	18.69	11.6	3.51
GAIF01010765.1	0.1	2.65	1.42	0	0.24	0
GAIF01010766.1	75.31	40.16	42.56	98.02	46.2	25.78
GAIF01010767.1	0.66	1.33	26.9	26.33	0.01	16.91
GAIF01010768.1	0	0	0	0.06	0.59	1.56
GAIF01010770.1	0	0	0.21	0	0	0.26

GAIF01010771.1	0	0.06	0	0	0.07	0
GAIF01010773.1	3.41	4.78	4.41	10.08	8.39	2.22
GAIF01010774.1	0	1.08	0.6	0	0.16	1.2
GAIF01010775.1	0	0	0	0	0.16	0
GAIF01010777.1	0.14	0	0	0.08	0.2	0
GAIF01010779.1	1.34	0.49	0	0.73	0.43	0.49
GAIF01010780.1	0	0	0	0.98	4.76	0
GAIF01010782.1	0	0	0	0	0.19	0
GAIF01010783.1	0.38	0	0.05	0.71	0.35	0
GAIF01010784.1	7.82	10.8	6.65	1.64	2.63	0.39
GAIF01010785.1	0.12	0	0	0	0.28	0
GAIF01010786.1	1.67	0.21	0.08	6.75	32.21	2.05
GAIF01010788.1	0	0	0	0	0.55	0
GAIF01010789.1	1.39	1.73	0.55	4.74	5.87	0.93
GAIF01010791.1	9.56	3.47	1.32	11.43	13.28	4.18
GAIF01010792.1	0	0.03	0	0.25	0.26	0
GAIF01010793.1	0.17	0	0	0.33	0.03	0
GAIF01010795.1	0	1.89	3.63	0	0	3.26
GAIF01010796.1	0	0	0	0	0.06	0
GAIF01010797.1	7.18	5.73	4.16	26.97	18.23	2.87
GAIF01010798.1	0	0	0	0.2	0.84	0
GAIF01010799.1	23.89	15.74	24.23	9.03	4.48	8.32
GAIF01010801.1	17.11	18.77	24.99	45.42	28.09	17.38
GAIF01010803.1	0.06	0.14	0	0.42	1.34	0
GAIF01010804.1	13.6	20.02	13.79	17.98	12.21	2.85
GAIF01010805.1	96.07	145.13	205.83	138.66	110.32	77.01
GAIF01010806.1	0.21	0.08	0	0.08	0	0
GAIF01010807.1	0.25	0.3	0.43	0.06	0.16	0.09
GAIF01010809.1	10.9	8.85	10.39	22.79	26.63	8.48
GAIF01010810.1	0.69	0.38	0	0.44	0.22	0
GAIF01010811.1	0.15	0.16	0	0	0.06	0
GAIF01010812.1	0	0.08	0	0	0.08	0
GAIF01010814.1	4.82	7.79	6.35	5.72	4.68	1.94
GAIF01010815.1	6.45	11.8	9.74	16.95	10.62	2.38
GAIF01010816.1	2142.43	4019.23	7366.89	1371.24	819.85	1989.61
GAIF01010818.1	69.77	39.84	20.49	60.43	45.26	41.29
GAIF01010819.1	0	5.16	0.45	7.09	5.88	1.3
GAIF01010820.1	0	2.33	9.58	0.17	0.17	4.1
GAIF01010822.1	26.81	20.2	20.81	32.53	40.62	14.19
GAIF01010823.1	577.24	219.17	267.5	172.4	164.38	307.91
GAIF01010824.1	230.78	69.43	56.47	149.66	83.29	114.46
GAIF01010825.1	0.08	0.08	0	0	0	0
GAIF01010827.1	0.19	0.07	0.56	0	0.08	0
GAIF01010828.1	18.66	11.93	22.7	24.82	23.63	50.13

GAIF01010831.1	0	0.06	0	0	0	0
GAIF01010832.1	3.04	0.71	0	14.52	34.8	1.68
GAIF01010834.1	16.95	22.51	8.76	73.04	57.65	2.75
GAIF01010836.1	2.25	1.35	0.13	17.49	49.61	0.07
GAIF01010837.1	33.66	48.8	43.58	94.84	90.02	12.2
GAIF01010840.1	0	0.13	1.33	0	0	0
GAIF01010841.1	0	0	0	0.25	0.79	0
GAIF01010844.1	170.9	200.57	161.54	35.81	28.34	7.05
GAIF01010845.1	4.53	1.03	0.61	5.26	13.79	19.05
GAIF01010846.1	2.7	4.23	4.95	8.93	10.26	2.63
GAIF01010847.1	1.33	0.39	0.19	2.47	7.32	2.26
GAIF01010849.1	0.27	0.12	0.12	0.69	2.14	0.18
GAIF01010851.1	13.01	8.74	7.01	52.86	51.23	10.19
GAIF01010852.1	0	0.35	0	0	0.13	0
GAIF01010853.1	0.14	0	0.16	0.17	0	0
GAIF01010855.1	1.27	0.04	0.05	0.48	0.35	0.05
GAIF01010856.1	45.04	44.08	16.98	98.89	106.55	31.57
GAIF01010857.1	6.07	3.79	6.57	13.58	14.82	10.37
GAIF01010858.1	0.15	0.11	0.15	0.35	0.89	0.11
GAIF01010859.1	36.57	34.54	22.6	50.28	57.42	18.79
GAIF01010860.1	0	0	0.04	0	0	0
GAIF01010862.1	0.58	0.6	0.74	1.52	2.1	1.14
GAIF01010863.1	13.59	3.22	5.02	21.76	25.44	29.94
GAIF01010865.1	0.54	0.59	0.8	4.11	4.26	0.1
GAIF01010866.1	0.1	0.08	0	0	0.09	0
GAIF01010867.1	10.9	24.19	18.78	7.03	3.82	2.84
GAIF01010868.1	0	0.14	0.5	0	0	0.03
GAIF01010869.1	1.93	2.29	1.16	5.3	6.34	0.77
GAIF01010870.1	0.23	0	0	0.95	2.83	0.52
GAIF01010871.1	2.3	1.54	1.02	3.94	1.11	0.07
GAIF01010873.1	0	0	0	0	0.41	0
GAIF01010874.1	0.27	0	0	0.21	1.72	0
GAIF01010875.1	0	1.42	2.77	0	0.17	0.26
GAIF01010876.1	0.1	0	0	0.12	1.26	0
GAIF01010877.1	0.05	0	0	0	1.22	0
GAIF01010878.1	0.74	0.65	0.34	3.14	4.96	0.18
GAIF01010879.1	0.14	0.15	0	1.86	5.99	0
GAIF01010881.1	0	0	0	0	0.04	0
GAIF01010882.1	0	0.2	0.15	0.38	1.24	0.1
GAIF01010883.1	2.24	1.28	0.25	0.87	3.4	0.48
GAIF01010884.1	4.4	4.61	0	7.02	14.11	0
GAIF01010885.1	3.86	3.33	1.41	1.92	3.01	0.63
GAIF01010886.1	0.88	0.32	0.11	6.58	17.75	0.65
GAIF01010887.1	0	1.59	0	1.6	1.26	0

GAIF01010889.1	0	0	0	0.05	0.07	0
GAIF01010891.1	5.6	10.87	2.34	35.27	50.81	0.59
GAIF01010893.1	0	0	0	0.04	0	0.03
GAIF01010895.1	0	0	0	0.31	0	0
GAIF01010896.1	0.49	0	0	0.43	4.15	3.26
GAIF01010897.1	0.3	0.63	0.4	1.04	0.46	0.07
GAIF01010898.1	0.41	0.03	0.45	0.37	1.51	0.19
GAIF01010900.1	0.11	0.17	0.02	1.53	2.31	0.83
GAIF01010901.1	145.17	68.45	69.98	204.09	102.88	156.49
GAIF01010903.1	2	0.19	0.29	0.3	0	0
GAIF01010904.1	18.19	21.43	16.05	53.23	47.12	8.04
GAIF01010905.1	2.63	1.83	2.17	3.09	0	0.67
GAIF01010907.1	14.14	8.44	10.69	9.12	2.31	7.33
GAIF01010908.1	0	0.07	0.03	0	0.03	0
GAIF01010910.1	0.33	0.31	0.16	0	0	0
GAIF01010914.1	13.14	10.73	8.5	35.67	46.14	5.05
GAIF01010915.1	0.41	0.17	0.11	0.36	0.49	0.17
GAIF01010917.1	0	0	0	0	0.18	0
GAIF01010918.1	0.05	0	0	0.18	0.39	0
GAIF01010919.1	5.1	2.65	0.49	11.32	37.87	4.64
GAIF01010920.1	1.26	0.42	0	2.44	1.97	1.26
GAIF01010921.1	0	0	0	0.1	0	0
GAIF01010927.1	359.57	116.36	119.51	292.11	97.21	90.02
GAIF01010929.1	3103.01	3166.42	3283.45	1864.82	1827.14	6427.63
GAIF01010931.1	0	1.62	0	0	0	0.09
GAIF01010933.1	13.83	16.03	14.72	17.78	10.55	6.8
GAIF01010936.1	1.44	0	0.65	5.86	1.04	0.52
GAIF01010937.1	4.08	2.8	1.79	14.5	10.49	1.74
GAIF01010939.1	0	0.34	0	0	0	0
GAIF01010941.1	299.43	305.33	724.17	318.41	171.13	525.67
GAIF01010942.1	2.06	0.41	0	6.28	9.69	15.33
GAIF01010943.1	0.18	0.57	0.19	2.99	5.84	0.45
GAIF01010944.1	269.7	125.16	47.33	1195.32	1078.57	155.57
GAIF01010945.1	33.96	2	1.65	68.93	112.67	202.44
GAIF01010947.1	0	0	0	0	0.05	0
GAIF01010948.1	1.95	0.49	0.66	4.04	9.29	6.34
GAIF01010949.1	2.66	1.74	0.17	13.67	42.85	0.99
GAIF01010950.1	20.43	42.65	71.05	98.48	92.74	126.54
GAIF01010951.1	0	0.14	0	0.87	2.42	0
GAIF01010953.1	0	0.03	0	0	0.04	0
GAIF01010954.1	0.19	0.04	0.17	0.27	0.19	0.04
GAIF01010955.1	40.89	35.1	37.5	46.17	17.92	17.88
GAIF01010956.1	0	0.46	0.09	0	0	0.09
GAIF01010957.1	0.15	0.27	0	0.26	0	0

GAIF01010960.1	0	0	0	0.03	0	0
GAIF01010962.1	0	0	0	0	0	0.06
GAIF01010966.1	0.2	0.06	0.17	0.12	1.35	0
GAIF01010967.1	4.08	9.43	17.13	1.49	1.86	1.24
GAIF01010969.1	21.92	23.52	16.9	53.08	35.79	4.72
GAIF01010970.1	0	0.15	0	0	0.32	0
GAIF01010971.1	0	0	0	0	0.17	0
GAIF01010973.1	0	3.1	1.38	0	0	1.21
GAIF01010974.1	0	0.2	0.54	0	0	0
GAIF01010975.1	1.35	1.15	0.66	60.43	0	0.83
GAIF01010976.1	0.19	0.07	0.05	0.05	0.25	0
GAIF01010977.1	0	0	0	0.09	0.26	0
GAIF01010978.1	9.07	13.74	6.66	65.55	94.29	1.65
GAIF01010979.1	0	0	0	0.19	0.1	0.26
GAIF01010980.1	28.87	13.02	21.88	36.62	18.9	110.17
GAIF01010981.1	0.78	0.1	0.07	1.2	1.1	0.91
GAIF01010982.1	0	0	0	0	0	0.25
GAIF01010983.1	0	0.12	0	0.27	1.34	0.13
GAIF01010985.1	0.63	0.14	0	1.88	3.19	0.14
GAIF01010986.1	115.71	54.42	42.31	155.6	79.74	11.51
GAIF01010987.1	0.1	0	0	0	0	0
GAIF01010988.1	8.22	4.27	4.2	14.96	18.2	6.59
GAIF01010989.1	0.1	0.11	0.11	2.09	5.9	3.31
GAIF01010992.1	0	0	0	0.22	0.11	0
GAIF01010993.1	24.86	38.56	47.79	59.18	35.91	15.47
GAIF01010994.1	0	0	0	1	4	0.25
GAIF01010999.1	6.16	7.93	2.28	9.48	13.45	0.76
GAIF01011001.1	0	0.11	0.23	0	0	0
GAIF01011002.1	0	0.19	0	0.4	0	0
GAIF01011003.1	3.85	1.98	1.59	1.77	1.76	1
GAIF01011004.1	0.05	0	0	0	0.06	0
GAIF01011005.1	33.28	42.94	43.84	95.18	66.74	14.79
GAIF01011006.1	0	0.57	1.16	0.12	0	0.12
GAIF01011007.1	0.19	0.15	0.05	1.47	4.75	0.47
GAIF01011008.1	0	0	0	0.03	0	0
GAIF01011009.1	15.17	9.99	11.64	12.31	13.01	5.21
GAIF01011010.1	0.52	0.4	0.57	0.91	0.69	0.34
GAIF01011011.1	0	1.5	1.18	0	0	0
GAIF01011012.1	0	0	0	0	0.13	0
GAIF01011013.1	1.43	1.49	0.35	12.61	9.19	0
GAIF01011014.1	139.9	64.83	37.17	95.62	108.96	242.73
GAIF01011015.1	42.1	56.18	42.32	30.67	24.33	25.11
GAIF01011017.1	1.62	3.43	2.01	0.84	1.38	0.56
GAIF01011018.1	9.1	6.85	5.86	10.46	6	1.06

GAIF01011019.1	15.4	15.66	22.01	27.25	29.08	11.64
GAIF01011021.1	0	0	0	0	0.5	0
GAIF01011022.1	0.09	0	0.21	0.6	4.71	0.16
GAIF01011023.1	0	0.86	0.71	0	0	0.24
GAIF01011024.1	0.2	0.11	0	1.37	4.39	3.15
GAIF01011025.1	50.59	65.03	27.57	105.65	102.27	8.28
GAIF01011026.1	15.28	22.58	37.02	18.97	22.19	6.07
GAIF01011027.1	12.64	15.52	11.82	16.89	10.97	1.96
GAIF01011028.1	0	0	0	0	0.82	0
GAIF01011030.1	5.21	4.91	6.35	17.21	18.11	4.39
GAIF01011032.1	0	0	0	0	1.08	0
GAIF01011035.1	0	0.13	0	0	0	0
GAIF01011037.1	0	0.12	0.12	0	0.13	0
GAIF01011038.1	2.26	0.94	0.19	0.7	1.04	0.1
GAIF01011039.1	78.22	83.2	109.36	53.5	26.78	26.08
GAIF01011040.1	7.3	8.89	0	15.46	13.35	0
GAIF01011043.1	0	0.71	0.48	0.19	0.07	0.06
GAIF01011045.1	12.59	16.43	2.88	22.84	27.33	6.52
GAIF01011047.1	0	0	0	0	0.17	0
GAIF01011049.1	0.5	0.49	0.89	4.77	2.2	0
GAIF01011050.1	33.03	25.56	21.21	155.97	171.15	18.22
GAIF01011051.1	0.29	0.21	0.52	0.55	1.26	0
GAIF01011052.1	38.05	50.56	37.28	118.22	93.2	16.63
GAIF01011053.1	1.03	0.27	0	1.91	1.13	1.31
GAIF01011056.1	0.29	0.13	0.19	0	0	0
GAIF01011060.1	0	0	0	0	0.18	0
GAIF01011063.1	0.04	0.07	0	0	0	0.22
GAIF01011064.1	25.02	18.53	19.4	46.85	54.33	76.47
GAIF01011065.1	0	0.08	0	0.13	0.84	0
GAIF01011066.1	0	0	0	0.3	0.76	0.29
GAIF01011067.1	0	0	0	0.04	0.27	0
GAIF01011069.1	1.14	0.2	0.07	4.81	20.29	1.26
GAIF01011070.1	0.6	0.92	0.46	3.91	5.21	0
GAIF01011071.1	0	0	0.06	1.53	2.59	0
GAIF01011073.1	3.33	1.92	1.51	3.98	2.47	3.35
GAIF01011074.1	0.32	0	0.14	0.52	1.44	1.47
GAIF01011075.1	91.31	92.94	60.46	136.65	112.1	61.43
GAIF01011076.1	320.16	235.39	169.33	325.57	322.31	54.6
GAIF01011078.1	12.25	14.15	7.45	16.25	18.42	47.49
GAIF01011079.1	0.06	0	0	0	0	0
GAIF01011080.1	0.13	0.05	0	0.05	0.46	0
GAIF01011083.1	0	0.26	0	0.28	1.73	0
GAIF01011084.1	0.12	0.04	0.14	0	0.15	0
GAIF01011085.1	0	0	0	13.08	16.04	0

GAIF01011086.1	12.99	12.2	14.63	25.45	21.9	8.07
GAIF01011087.1	84.48	110.39	59.37	95.83	82.57	9.27
GAIF01011088.1	0.03	0	0	0	0	0
GAIF01011089.1	0	0	0.12	0	0	0
GAIF01011090.1	0.59	0.4	0.53	0.81	0.66	0.45
GAIF01011091.1	0	0	0	0.28	0.14	0
GAIF01011093.1	0	0	0.07	0	0.15	0
GAIF01011095.1	0	0	0.05	0	0	0.08
GAIF01011098.1	3.8	4.11	3.48	24.99	31.5	3.06
GAIF01011099.1	0.01	0	0	0	0	0
GAIF01011100.1	0.09	0.09	0	1.1	3.1	0.09
GAIF01011101.1	0.04	0	0.04	0	0	0
GAIF01011103.1	1.45	1.04	1.35	0.54	0.96	0.17
GAIF01011104.1	28.61	27.37	28.49	58.42	45.5	18.26
GAIF01011108.1	55.58	40.19	57.83	98.97	46.31	85.88
GAIF01011109.1	45.44	33.46	5.33	3.26	1.58	2.17
GAIF01011111.1	0	0	0	0.46	0.67	0
GAIF01011115.1	1.22	0	0	1.81	8.51	0
GAIF01011117.1	0	0.37	0	0	0.07	0.06
GAIF01011119.1	0.38	0.15	0.05	0.94	6.96	1.2
GAIF01011120.1	1.17	0.12	0	4.46	10.54	1.87
GAIF01011121.1	21.58	31.74	17.8	65.52	61.11	8.75
GAIF01011122.1	0.28	0.15	0	1.96	10.62	0
GAIF01011123.1	0	0	0	0.22	0.99	0
GAIF01011125.1	1.2	0.52	2.1	0.17	0	0.16
GAIF01011127.1	0	0	0	0	0.05	0
GAIF01011128.1	0	0	0	0.4	0	0
GAIF01011130.1	0.4	0.22	0.27	0.46	1.85	0.41
GAIF01011131.1	16.43	13.38	14.63	19.73	10.36	7.09
GAIF01011133.1	0	0	0.08	0.04	0	0
GAIF01011135.1	11.71	5.9	6.71	5.77	2.82	1.64
GAIF01011136.1	0.02	0	0	0	0.19	0.06
GAIF01011139.1	6.4	1.97	8.94	5	1.35	1.81
GAIF01011141.1	150.84	184.2	155.44	245.07	230.27	85.38
GAIF01011143.1	0.15	0	0.08	15.73	0.09	0.33
GAIF01011145.1	0.07	0	0	0	0.71	0
GAIF01011146.1	6.24	7.02	3.88	26.14	17.75	1.51
GAIF01011147.1	0.26	0	0	0.15	0.16	0.14
GAIF01011148.1	2.36	0.1	0.1	1.47	4.58	2.4
GAIF01011149.1	55.73	6.65	0.88	40.58	63.46	6.96
GAIF01011151.1	8.36	19.67	14.22	8.04	4.74	1.12
GAIF01011152.1	0	0	0	0.08	0	0
GAIF01011153.1	2.93	1.37	3.59	3.46	1.3	2.24
GAIF01011154.1	0	0.15	0.03	0	0.06	0

GAIF01011155.1	0	0	0.04	0	0.55	0.04
GAIF01011156.1	0	0.15	0	0	0	0
GAIF01011157.1	0	0	0	0	0	0.03
GAIF01011159.1	0.05	0	0	0.06	0	0
GAIF01011160.1	3.71	6.13	6.04	9.12	4.85	2.68
GAIF01011161.1	6.14	13.25	33.22	29.91	31.67	46.84
GAIF01011163.1	3.21	2.58	1.73	9.16	6.9	1.62
GAIF01011164.1	0	0.06	0	0.18	2.2	0
GAIF01011166.1	1.33	0.13	0	3.89	9.98	0
GAIF01011167.1	0.09	0.1	0	0.13	0.61	0
GAIF01011169.1	0	0.33	0	0.38	0	0.28
GAIF01011171.1	0	0	0	0.11	0	0
GAIF01011173.1	8.47	15.87	7.4	1.35	1.83	3.14
GAIF01011174.1	5.73	3.75	7.96	16.48	7.99	9.22
GAIF01011175.1	12.33	5.32	1.5	10.38	15.12	17.64
GAIF01011176.1	0	0	0.06	0	0	0
GAIF01011177.1	0	0	0	1.13	4.47	0
GAIF01011178.1	0.73	0.63	0.48	0.17	1.57	0
GAIF01011181.1	0	0	0	0.32	0.17	0
GAIF01011183.1	5.32	7.89	5.19	2.43	2.38	0.45
GAIF01011184.1	4730.06	4758.69	4551.96	2369.3	1568.41	3983.3
GAIF01011185.1	0.14	0	0.15	0.98	1.86	0
GAIF01011187.1	0	0	0	0	0.06	0
GAIF01011190.1	0.57	0.16	0.12	7.08	34.71	0.5
GAIF01011191.1	0.12	0.27	0.24	0.5	0.31	0
GAIF01011192.1	0.16	0.29	0.52	0	0	0
GAIF01011193.1	3.67	4.27	4.69	1.58	0.62	1.6
GAIF01011194.1	5.32	6.87	8.76	33.38	13.59	8.43
GAIF01011195.1	27.1	29.61	39.26	61.76	26.88	17.39
GAIF01011196.1	0	0	0	16.36	26.8	1.05
GAIF01011197.1	3.74	9.45	5.92	15.84	15.75	2.43
GAIF01011198.1	1775.69	1272.96	890.67	164.07	188.4	451.05
GAIF01011199.1	3.46	5.6	8.45	2.96	9.91	2.91
GAIF01011202.1	0.11	0	0	0.78	2.7	0
GAIF01011203.1	0.31	0.08	0.03	1.39	4.73	0.52
GAIF01011204.1	0	0	0	0	1.79	0.15
GAIF01011205.1	3.34	0.11	0	0.36	0	0
GAIF01011206.1	97.25	72.46	24.62	102.68	119.56	28.53
GAIF01011208.1	0.27	0.96	0.6	4.2	1.23	0.23
GAIF01011210.1	0	0	0	0.11	1.57	0.1
GAIF01011212.1	15.25	15.99	23.92	0.63	0.52	0.48
GAIF01011213.1	0.49	0.27	0	1.14	6.62	0
GAIF01011214.1	0.06	0.06	0	0.45	1.54	1.96
GAIF01011215.1	1.35	1.36	0.91	10.26	42.38	0.92

GAIF01011216.1	35.98	38.65	22.9	3.03	17.93	33.56
GAIF01011218.1	0	1.11	0.57	0	0	0
GAIF01011220.1	0.02	0.32	0	0.09	0.07	0
GAIF01011221.1	0.08	0.09	0	1.04	2.55	0.36
GAIF01011222.1	0.9	0.14	0.14	3.03	1.75	0.61
GAIF01011224.1	0	0	0	0	0.2	0
GAIF01011225.1	452.29	717.42	633.6	47.83	33.58	74.14
GAIF01011226.1	4.13	1.88	0.29	7.58	23.88	4.55
GAIF01011228.1	0	0.07	0	0	0	0.22
GAIF01011231.1	0.59	0.4	0.17	5.27	23.83	0.51
GAIF01011232.1	0.14	0	0	0	0.5	0
GAIF01011233.1	9.56	0.89	0.41	36.65	156.94	99.39
GAIF01011237.1	0.2	0	0	0.11	1.14	0.04
GAIF01011238.1	2.98	2.63	0.62	25.27	42.45	0.33
GAIF01011239.1	0.14	0	0.08	0.13	0.42	0.04
GAIF01011240.1	0	0	0	0.22	0	0
GAIF01011241.1	47.7	34.44	43.6	74.42	95.54	27.14
GAIF01011242.1	7.25	7.28	3.01	25.8	46.08	4.88
GAIF01011244.1	9.23	19.35	13.68	46.93	30.21	3.46
GAIF01011245.1	6.87	3.78	2.26	32.22	24.62	3.97
GAIF01011248.1	0	0	0	0	0.21	0
GAIF01011249.1	0.22	0	0	0	0	0
GAIF01011250.1	0	0	0	0	0.04	0
GAIF01011252.1	0	0	0	0	2.16	0
GAIF01011254.1	0	0	0	0	0.19	0
GAIF01011255.1	0	0	0	0.96	6.41	0
GAIF01011257.1	372.42	20.87	26.96	360.13	901.61	2492.35
GAIF01011258.1	0	0.33	0.36	0.17	0.44	0
GAIF01011259.1	15.87	11.41	11.15	28.36	38.18	17.12
GAIF01011260.1	0	0	0.06	0	0	0.07
GAIF01011262.1	0	0	0.04	0.35	0	0.21
GAIF01011263.1	2.77	2.13	2.25	10.83	10.91	0.53
GAIF01011264.1	0.43	0.1	0.1	2.57	14.67	0.45
GAIF01011265.1	2.84	3.78	2.8	15.22	12.78	4.07
GAIF01011266.1	0.62	0	0	1.2	6.49	0.12
GAIF01011267.1	24.73	37.31	28.8	36.21	32.85	10.91
GAIF01011268.1	108.43	151.06	194.27	155.37	114.27	137.13
GAIF01011269.1	0	0	0	0	0.08	0
GAIF01011271.1	9.11	6.32	2.01	3.08	3.36	1.02
GAIF01011273.1	10.79	12.62	14.43	3.68	1.74	1.1
GAIF01011274.1	0	0	0	0.13	2.2	0
GAIF01011275.1	0	0.06	0	0	0	0
GAIF01011276.1	0	0	0	0	0.08	0
GAIF01011280.1	3.76	1.1	0	11.43	32.85	4.03

GAIF01011281.1	20.48	16.99	19.52	29.39	17.71	12.87
GAIF01011282.1	1.34	0	0	5.31	6.98	0.3
GAIF01011283.1	88.44	155.6	137.52	268.77	284.09	40.08
GAIF01011284.1	0	0.11	0	0	0.12	0
GAIF01011285.1	0.3	0.19	0.24	2.22	4.2	0.49
GAIF01011286.1	0	0	0	0.05	0.1	0
GAIF01011288.1	39.32	30.63	29.01	94.74	44.47	40.6
GAIF01011289.1	0.27	0.12	0	1.95	8.29	0.17
GAIF01011290.1	22.57	15.59	27.89	29.32	16.05	21.27
GAIF01011291.1	0	0	0	0.3	1.96	0
GAIF01011293.1	22.42	30.66	26.09	63.23	67.86	18.25
GAIF01011297.1	58.78	57.34	0	59.78	5.93	0
GAIF01011299.1	2.27	0.25	0	10.43	23.86	0
GAIF01011300.1	0.24	0	0.06	2.27	10.6	0.52
GAIF01011302.1	4.63	7.92	8.62	13.52	9.44	3.01
GAIF01011303.1	0.38	0.21	0.14	2	7.28	0
GAIF01011304.1	0.7	0.38	0.77	0.81	0.42	1.54
GAIF01011306.1	26.71	33.45	10.46	98.12	61.27	4.8
GAIF01011307.1	0.17	0	0	0	0	0
GAIF01011308.1	0.03	0	0	0	0	0
GAIF01011310.1	0	0	0	0.14	0.59	0
GAIF01011311.1	1.56	0.26	0.07	2.44	8.37	2.89
GAIF01011316.1	0	0	0.96	0.37	0	0
GAIF01011317.1	0.17	0	0.04	0	0.04	0
GAIF01011319.1	0	0	0	0	0.13	0
GAIF01011320.1	0.03	0.07	0.07	0.66	3.18	0
GAIF01011322.1	7.23	5.19	0.38	4.94	10.83	2.86
GAIF01011325.1	1.77	4.92	7.92	3.19	4.14	13.41
GAIF01011326.1	0.15	0.34	0	0.67	0.81	0
GAIF01011327.1	0	0.1	0	0.1	2.48	0
GAIF01011330.1	38.74	55.04	44.67	54.25	29.97	14.2
GAIF01011331.1	11.82	7.16	1.73	11.95	32.59	6.99
GAIF01011332.1	0	0	0.75	0	0	0
GAIF01011334.1	27.37	35.63	77.7	36.96	25.28	37.02
GAIF01011335.1	0	0	0	0	0.12	0
GAIF01011340.1	8.71	8.68	11.42	6.58	0.45	6.33
GAIF01011342.1	11.25	0.77	0	13.01	20.51	36.76
GAIF01011343.1	0	0	0	0	0.12	0
GAIF01011344.1	0	0	0.09	0.1	0.61	0.19
GAIF01011345.1	52.69	22.96	25.29	29.79	26.24	90.1
GAIF01011346.1	0.14	0.09	0.07	0	1.02	0.18
GAIF01011350.1	0.38	0.27	0	2.2	5.92	0.14
GAIF01011351.1	0.07	0	0.32	0.12	0	0.08
GAIF01011352.1	35.74	43.75	32.27	114.05	142.39	27.39

GAIF01011353.1	15.24	10.78	3.43	4.06	7.42	2.08
GAIF01011354.1	5.69	1.25	0.08	0.3	2.24	0.04
GAIF01011355.1	0.05	0	0	0.06	0.03	0
GAIF01011357.1	0	0	0.04	0.04	0	0
GAIF01011358.1	7.42	13.44	17.22	6.93	4.4	3.11
GAIF01011359.1	1.27	1.7	0.86	0.91	0.59	0
GAIF01011360.1	0.22	0.14	0	0.85	0.63	0
GAIF01011361.1	0	0.11	0	0.13	2.07	0
GAIF01011362.1	17	4.02	0.67	39.13	135.11	15.02
GAIF01011365.1	87.95	93.25	214.33	49.71	38.3	172.96
GAIF01011366.1	0.79	0.93	1.23	1.76	1.66	5.07
GAIF01011367.1	0.24	0.06	0	0.9	2.79	0.07
GAIF01011368.1	1.19	0	0	2.98	15.55	0.3
GAIF01011369.1	0	0	0	0.47	1.15	0
GAIF01011370.1	6.37	4.77	7.5	14.03	8.77	18.1
GAIF01011371.1	0	0	1.58	0	0	4.63
GAIF01011372.1	0.19	0.07	0.07	5.6	22.51	0.07
GAIF01011373.1	0.69	0.22	0.36	1.15	0.15	0
GAIF01011374.1	23.49	19.15	12.69	55.92	51.1	0.02
GAIF01011375.1	0	0	0	0	0.07	0
GAIF01011376.1	24.83	27.86	26.94	7.67	4.63	2.8
GAIF01011378.1	0.23	0	0	1.23	3.39	0.26
GAIF01011379.1	0.34	0.18	0	0.99	2.04	0
GAIF01011380.1	0.62	0	0	1.56	0	0
GAIF01011382.1	7.31	5.99	13.32	23.36	17.41	7.14
GAIF01011383.1	0	0	0	0.4	0	0
GAIF01011384.1	690.3	1081.48	699.48	205.45	612.72	81.85
GAIF01011385.1	0.06	0.06	0	0	0	0
GAIF01011386.1	0	0	0	0	0.3	0
GAIF01011387.1	20.68	11.04	6.2	82.45	89	3.39
GAIF01011388.1	0	0	0	0	0.16	0
GAIF01011389.1	0	0	0	0	0	0.14
GAIF01011391.1	0.1	0.21	0	0.66	3.22	0.11
GAIF01011392.1	312.06	961.88	1721.8	320.97	362.69	346.16
GAIF01011393.1	23.68	60.45	43.37	78.07	56.95	8.86
GAIF01011395.1	47.57	72.2	61.73	122.06	71.59	29.85
GAIF01011396.1	537.74	396.57	781.99	564.49	21.08	228.71
GAIF01011397.1	0	0	0	0	0.17	0
GAIF01011398.1	0.67	0.22	0	0.1	0.2	0
GAIF01011400.1	0.08	0.09	0.19	0	0	0
GAIF01011401.1	0	0	0	0	0.06	0
GAIF01011402.1	18.95	12.95	6.24	50.11	35.16	3.88
GAIF01011403.1	11.53	17.5	6.27	7.82	3.29	0.38
GAIF01011404.1	0	0	0	0	0	0.06

GAIF01011409.1	0.3	0.11	0.05	1.27	5.39	0
GAIF01011410.1	0	0	0	0	0.1	0
GAIF01011412.1	8.16	8.04	3.54	9.69	19.16	4.18
GAIF01011413.1	7.89	12.14	7.9	3.72	5.97	0
GAIF01011414.1	0	0	0.03	0	0	0
GAIF01011415.1	5.85	1.73	0.25	19.22	27.68	1.26
GAIF01011416.1	0.12	0	0.1	0.02	0.09	0.03
GAIF01011418.1	0.03	0	0.28	0.62	0	0
GAIF01011419.1	0.75	0.15	0.06	1.31	0.14	0.39
GAIF01011420.1	0.05	0	0	0.16	0.76	0
GAIF01011421.1	2.86	7.97	3.43	8.93	8.76	0.4
GAIF01011422.1	0.04	0	0	0	0	0
GAIF01011423.1	64.67	77.01	155.13	26.74	22.47	80.18
GAIF01011424.1	0.64	0.69	0.7	1.36	0.68	0.33
GAIF01011425.1	0.22	0	0	0.13	0.26	0
GAIF01011426.1	0.72	0.26	0	2	22.24	43.44
GAIF01011427.1	1.33	0.8	0.43	0.85	0.85	0.09
GAIF01011428.1	0	0	0	0	0.29	0
GAIF01011429.1	37.03	14.11	12.39	32.38	20.03	2.96
GAIF01011430.1	0.22	0.12	0	1.95	6.07	0.37
GAIF01011431.1	0	0	0	0.41	1	0
GAIF01011432.1	1.34	1.42	0.26	3.36	2.56	0.81
GAIF01011433.1	0	0	0	0	0.08	0
GAIF01011434.1	47.47	58.72	42.59	6.55	6.09	4.67
GAIF01011435.1	16.88	11.73	6.02	46.62	56	3.36
GAIF01011436.1	0	0	0	0	0.37	0.82
GAIF01011439.1	0.3	0.16	0.16	0.18	0	0
GAIF01011440.1	0.26	0.07	0	1.44	5.73	0.07
GAIF01011442.1	0	0.4	0.2	0	0	0
GAIF01011443.1	0.3	0	0	0.35	0	0.06
GAIF01011444.1	0.17	0	0	0.15	0.48	0.13
GAIF01011445.1	1	1.12	0.64	0.34	0	0
GAIF01011446.1	1.85	0.88	0.76	6.03	2.64	0.25
GAIF01011447.1	3.95	2.37	3.09	26.49	61.98	2.49
GAIF01011448.1	0	0	0	0	0	0.17
GAIF01011449.1	1.18	1.2	0.26	3.58	4.33	0
GAIF01011452.1	1.18	0.86	1.84	1.62	0.91	0
GAIF01011453.1	0.25	0	0.21	0	0	0
GAIF01011454.1	0.09	0	0	0.13	0.14	0
GAIF01011455.1	42.11	42.91	20.63	42.46	50.64	10.23
GAIF01011456.1	0	0	0	0	0.01	0
GAIF01011457.1	0.3	0.08	0	2.24	6.35	0
GAIF01011458.1	819.94	708.32	926.9	435.05	281.47	283.63
GAIF01011461.1	0.31	0	0	0.48	5.87	0

GAIF01011465.1	0.26	0.33	0.39	0.24	0.22	0.39
GAIF01011466.1	0.71	1.11	0.48	2.21	0.94	0.45
GAIF01011467.1	0.02	0	0	0	0.01	0
GAIF01011468.1	2.06	1.72	0	11.02	14.65	0.17
GAIF01011469.1	5.74	2.89	2.04	10.66	14.82	7.75
GAIF01011470.1	2318.88	1348.92	1634.63	1508.13	847.12	1964.71
GAIF01011474.1	0	0.06	0.06	0.06	0.8	0.04
GAIF01011477.1	0	2.39	0	1.15	3.35	4.79
GAIF01011478.1	105.09	62.13	44.33	121.35	157.87	220.18
GAIF01011480.1	0.05	0	0.03	0	0.03	0
GAIF01011481.1	1.83	1.42	0	3.89	7.66	0
GAIF01011482.1	2.8	0.8	1.14	6.72	9.02	1.01
GAIF01011483.1	7.68	9.08	22.19	14.81	10.05	7.59
GAIF01011484.1	0.06	0	0	0	0.81	0
GAIF01011485.1	4.46	1.62	0	0	0	0
GAIF01011486.1	6.98	8.47	6.16	19.88	19.07	2.24
GAIF01011488.1	31.73	18.43	17.45	6.8	9.19	7.5
GAIF01011491.1	136.22	184.15	405.38	94.97	71.55	275.49
GAIF01011494.1	0.11	0.12	0.12	0.25	0.39	0
GAIF01011496.1	0	0	0	0	0.2	0
GAIF01011497.1	0	0	0	0	0.31	0
GAIF01011498.1	9.96	34.17	8.35	32.87	3.23	7.91
GAIF01011499.1	199.94	191.34	174.43	108	57.45	27.77
GAIF01011500.1	13.57	15.95	15.34	10.63	16.32	4.12
GAIF01011501.1	0.32	0	0.18	0.38	4.86	0.53
GAIF01011502.1	70.95	89.1	37.58	89	44.39	60.38
GAIF01011503.1	4	1.4	0	16.75	46.12	1.44
GAIF01011504.1	0.34	0.13	0	0.21	0.36	0
GAIF01011506.1	0.17	0.37	0	0.69	1.23	0.09
GAIF01011507.1	0	0	0	0	0.09	0.06
GAIF01011510.1	18.27	13.05	7.55	50.67	55.19	7.81
GAIF01011511.1	0.9	0	9.62	8.93	8.73	7.53
GAIF01011512.1	4.96	9.18	4.23	15.39	15.6	0.6
GAIF01011514.1	0	0	0	0	0.04	0
GAIF01011516.1	0.83	0.18	0	1.92	7.93	1.25
GAIF01011517.1	0	1.05	1.06	0	0	0
GAIF01011518.1	342.18	278.17	386.1	95.43	44.5	62.01
GAIF01011520.1	0.05	0	0.1	0	0	0
GAIF01011521.1	0	0.26	0.37	0.72	1.94	0
GAIF01011522.1	0.63	0.86	0.69	0.18	0.57	0.7
GAIF01011524.1	4.77	2.66	5.36	11.41	10.6	12.6
GAIF01011525.1	0	0.11	0.15	0	0	0.07
GAIF01011526.1	4.12	5.85	0.88	14.6	22.97	1.51
GAIF01011528.1	0.01	0	0	0.06	0	0.38

GAIF01011529.1	2.59	0.65	0.47	6.71	24.27	2.91
GAIF01011530.1	28.41	34.18	17.38	19.32	15.26	2.95
GAIF01011532.1	2736.16	812.97	1151.99	1694.19	2459.13	9045.91
GAIF01011534.1	18.52	20.68	20.97	34.03	19.28	14.37
GAIF01011535.1	19.29	34.47	36.79	45.53	33.02	15.88
GAIF01011539.1	1.42	0.84	0.07	13.82	22.41	0.21
GAIF01011540.1	0	0	0	0	0.13	0
GAIF01011541.1	0	0	0	0	0.85	0
GAIF01011542.1	0	0	0	0	0.04	0
GAIF01011543.1	0.17	0	0	0.29	1.09	0
GAIF01011544.1	0.54	0	0.89	0.31	0	0
GAIF01011547.1	1.6	0.24	0.05	0	0.1	0.1
GAIF01011549.1	18.22	20.65	9.08	55.27	43.83	6.08
GAIF01011550.1	11.77	6.38	4.66	22.96	24.89	46.25
GAIF01011551.1	0	0.17	0.13	2.73	7.53	0.23
GAIF01011555.1	0	0	0.16	0	0.15	0
GAIF01011556.1	12.52	16.07	15.17	62.18	52.17	9.5
GAIF01011558.1	0.4	0.5	0	4.04	15.57	0.63
GAIF01011559.1	0.27	0	0	0	9.57	0.18
GAIF01011560.1	3.18	0.82	0.17	1.05	5.82	0
GAIF01011562.1	22.44	15.86	38.8	44.33	19.75	104.59
GAIF01011563.1	0.04	0.08	0	0	0.09	0
GAIF01011566.1	0	0.11	0.11	0	1.35	0.06
GAIF01011568.1	0	0.43	0	0.46	4.97	0
GAIF01011569.1	27.59	21.76	22.1	33.46	27.72	11.83
GAIF01011570.1	0	0	0	0	0.6	0
GAIF01011572.1	0.13	0	0	0.94	4.4	0.14
GAIF01011573.1	0	0	0	0	0.02	0
GAIF01011574.1	10.67	13.38	15.39	51.72	41.51	8.02
GAIF01011576.1	11.67	4.64	4.46	21.31	28.61	9.16
GAIF01011577.1	10.44	10.75	8.6	22.29	25.45	9.15
GAIF01011578.1	9	8.77	8.49	15.87	6.34	4.22
GAIF01011579.1	0	0	0	0	1.26	0
GAIF01011580.1	0	0.71	0.44	0	0	0.18
GAIF01011583.1	0.05	0	0	0	0	0
GAIF01011584.1	0	0	0	0	0.22	0
GAIF01011585.1	17.29	8.9	8.81	7.51	11.82	15.09
GAIF01011587.1	58.88	130.42	75.08	210.09	201.59	28.83
GAIF01011588.1	0	0	0	0.03	0.03	0
GAIF01011589.1	0.4	0	0	1	4	0.33
GAIF01011590.1	0.03	0	0	0	0	0
GAIF01011591.1	0	0	0	0	0.16	0
GAIF01011592.1	13.07	18.51	16.64	44.55	42.89	6.74
GAIF01011594.1	0.42	0	0	0.16	0	0

GAIF01011596.1	0	0	0	0	0.23	0
GAIF01011597.1	0.08	0.17	0.26	2.14	8	0.44
GAIF01011598.1	0.52	0.31	0.84	0.54	0.7	0.97
GAIF01011600.1	6.45	5.54	0.99	12.42	35.81	1.43
GAIF01011601.1	0	0.15	0	0.31	1.14	0
GAIF01011602.1	0	0	0	0	0.08	0
GAIF01011603.1	1.41	1.21	4.09	0	0	2.83
GAIF01011604.1	0.02	0.08	0	0.04	0.02	0.08
GAIF01011605.1	0.04	0.05	0	0	0	0
GAIF01011606.1	0	1.36	1.37	0	0.07	0.29
GAIF01011607.1	0.26	0.46	0	2.53	5.82	0
GAIF01011608.1	0.4	0.29	0.15	0.92	1.44	0.29
GAIF01011610.1	0	0	0	0.13	0	0
GAIF01011612.1	4.68	3.87	1.99	18.73	3.5	3.66
GAIF01011613.1	0	0.28	0	0	0.16	0
GAIF01011614.1	0.77	0	0.24	0	0.07	0.06
GAIF01011615.1	0.37	0	0.06	1.42	7.98	0.99
GAIF01011616.1	0.47	1.94	0.54	0.21	5.97	0.04
GAIF01011617.1	0.86	1.89	0.95	4.98	4.97	0.45
GAIF01011618.1	0	0.24	0	0.38	0.79	0
GAIF01011619.1	10.27	15.3	17.76	5.67	6.54	1.79
GAIF01011620.1	0.07	0.38	0.07	0	0.12	0
GAIF01011622.1	0	0	0	0.15	0.09	0
GAIF01011623.1	22.37	19.93	13.2	30.28	19.89	6.94
GAIF01011624.1	10.1	17.93	3.2	46.35	70.02	0.87
GAIF01011625.1	18.99	21.67	15.17	40.18	28.98	4.21
GAIF01011626.1	0.14	0.15	0.91	0.48	0.66	0
GAIF01011629.1	0	0	0	0	0.08	0
GAIF01011631.1	11.33	19.04	16.59	23.91	13.83	5.3
GAIF01011632.1	8.24	13.08	7.56	7.65	5.46	0.77
GAIF01011633.1	0	0.11	0	0	0.25	0
GAIF01011634.1	0	0	0.12	0	0.55	0.87
GAIF01011635.1	1.59	0.92	1.16	2.95	3.44	0.93
GAIF01011636.1	930.08	194.12	139.5	916.19	1243.17	11354.3
GAIF01011637.1	27.56	26.27	13.7	9.47	10.57	2.01
GAIF01011638.1	107.22	225.99	105.82	220.51	117.71	25.05
GAIF01011639.1	63.12	82.03	65.69	124.89	156.44	46.67
GAIF01011641.1	18.34	27.97	18.86	9.93	6.25	1.73
GAIF01011643.1	8.46	5.4	4.54	4.22	1.35	0.91
GAIF01011645.1	57.93	45.46	21.42	188.41	147.21	31.86
GAIF01011647.1	17.26	7.18	3.94	25.35	33.25	8.63
GAIF01011649.1	0	0	0	0	0.39	0
GAIF01011650.1	0.16	1.15	0.85	0.39	0.54	0.91
GAIF01011652.1	0.28	0	0	0.03	0	0

GAIF01011653.1	62.38	43.86	81.37	30.76	7.44	60.75
GAIF01011656.1	0	0	0	0.36	0	0
GAIF01011657.1	0.84	0	0	1.27	3.68	2.54
GAIF01011658.1	2.28	4.09	0.08	1.85	5.56	2.49
GAIF01011659.1	0.13	0.14	0.14	1.16	1.65	0.14
GAIF01011661.1	0	0	0	0.04	0	0
GAIF01011662.1	10.3	0	0.1	10.58	0.66	1.41
GAIF01011664.1	0.65	0.28	0.14	0	0	0
GAIF01011665.1	7.18	11.49	10.95	22.2	14.5	3.37
GAIF01011666.1	9.37	7.55	4.51	16.61	16.46	1.91
GAIF01011667.1	1.26	2.17	1.78	4.09	1.29	0.66
GAIF01011668.1	0	0	0	0.09	0.55	0
GAIF01011669.1	6.64	2.86	7.25	3.51	2.04	6.59
GAIF01011670.1	0.77	0.84	1.53	0.36	0.56	0
GAIF01011672.1	0.12	0	0	0.28	6.46	0
GAIF01011673.1	3.69	0.89	0.34	1.89	7.76	27.33
GAIF01011674.1	1.19	4.8	7.24	0.24	0	1.14
GAIF01011675.1	0.1	0.29	0	0	0.08	0
GAIF01011678.1	0.35	0	0	1.77	6.35	0.23
GAIF01011679.1	0.14	0	0	0	0.98	0
GAIF01011680.1	0.2	0.53	0	1.13	1.64	0
GAIF01011681.1	0	0.3	0.1	0	0	0
GAIF01011682.1	0	0	0	0.31	0	0
GAIF01011683.1	29.07	39.51	42.04	114.85	78.26	36.21
GAIF01011685.1	0	0.05	0	0	0	0
GAIF01011686.1	0	0	0	0	0.1	0
GAIF01011687.1	107.45	170.66	150.37	123.72	96.93	33.38
GAIF01011691.1	0	0.26	0	0.3	0.16	0.04
GAIF01011693.1	10.19	13.09	10.2	23.48	6.22	1.34
GAIF01011694.1	0.12	0	0	0	0.29	0
GAIF01011696.1	23.74	29.06	49.05	164.64	77.64	27.01
GAIF01011697.1	0	0	0.03	0	0.12	0
GAIF01011699.1	0.24	0.52	0	2.78	8.84	0.26
GAIF01011700.1	0.08	0.03	0	0.44	3.12	0.25
GAIF01011701.1	0	0.1	0.44	0	0.36	0.22
GAIF01011702.1	13.57	5.9	6.06	8.75	8.28	8.22
GAIF01011708.1	0.95	0.32	0.08	4.47	14.69	0.32
GAIF01011709.1	2.28	2.87	2.63	5.98	12.11	0.4
GAIF01011710.1	52.55	69.56	46.81	79.38	24.48	12.9
GAIF01011711.1	12.83	0.22	7.04	59.79	33.09	79.12
GAIF01011716.1	0	0	0	0	0.31	0
GAIF01011717.1	2.21	0.21	0.04	5.43	18.33	29.91
GAIF01011718.1	0.35	1.6	0.44	1.22	3.9	0.29
GAIF01011721.1	0.94	0.51	0	0.43	0.29	0

GAIF01011722.1	35.77	18.44	27.27	95.42	203.68	316.25
GAIF01011723.1	5.65	0.29	0.44	5.3	0	1.11
GAIF01011725.1	0.49	1.5	0.43	0.57	0.47	0
GAIF01011727.1	0	0	0	0.02	0.51	0.04
GAIF01011729.1	0.03	0.09	0.06	0.4	2.15	0.09
GAIF01011730.1	0.14	0.07	0	0	0.03	0
GAIF01011731.1	24.97	37.59	70.92	21.4	17.35	11.96
GAIF01011732.1	0.7	0.86	0.5	3.9	6.89	0.73
GAIF01011733.1	0.14	0.21	0.05	0.16	0.11	0
GAIF01011736.1	3.48	4.66	1.61	18.2	18.14	0.39
GAIF01011737.1	2	3.94	1.44	0.51	0.75	0
GAIF01011738.1	22.73	32.16	7.73	0.66	0.46	0.21
GAIF01011739.1	5.54	6.44	2.22	17.95	26.42	0.8
GAIF01011740.1	0.07	0.04	0	0	0.04	0
GAIF01011741.1	0.1	0	0	0.45	4.89	0
GAIF01011742.1	0	0.09	0	0	0	0
GAIF01011743.1	0	0.09	0	0.09	0.96	0
GAIF01011744.1	2.22	2.13	1.16	0.35	0	0.34
GAIF01011745.1	8.37	18.27	18.89	28.75	18.19	7.16
GAIF01011747.1	9.94	12.19	4.68	22.49	16.93	1.91
GAIF01011748.1	4.41	1.84	0	7.08	12.55	11.32
GAIF01011749.1	8.97	12.61	7.36	41.57	33.88	3.97
GAIF01011750.1	0	0	0	0	0.08	0
GAIF01011751.1	8.01	18.95	8.62	35.86	40.78	1.68
GAIF01011753.1	12.09	20.48	18.18	51.46	36.91	11.29
GAIF01011754.1	48.18	104.4	73.51	144.13	128.28	16.92
GAIF01011755.1	13.96	25.42	21.46	65.3	62.3	51.83
GAIF01011756.1	28.87	26.45	19.46	57.67	30.49	40.97
GAIF01011757.1	0	0.04	0	1.15	4.23	0.08
GAIF01011759.1	0.86	0.06	0.22	2.59	7.14	5.87
GAIF01011760.1	0	0.61	0.97	0	0	0.62
GAIF01011761.1	6.94	0.64	0	2.06	0	0
GAIF01011762.1	0	0.14	0.28	0.88	3.8	0
GAIF01011763.1	22.43	23.88	15.02	78.92	46.27	12.18
GAIF01011764.1	0.94	0.2	0	0.81	0.25	0
GAIF01011765.1	4.97	5.07	3.23	25.94	21.32	2.4
GAIF01011766.1	14.46	8.86	12.48	24.93	25.26	15.54
GAIF01011767.1	0	0	0	0.21	0.22	0
GAIF01011768.1	0.14	0.08	0	1.63	6.13	0.15
GAIF01011769.1	0	0.73	0.73	0	0.04	0.08
GAIF01011772.1	0.61	0.6	0.32	0.45	0.43	0.58
GAIF01011773.1	21.01	17.81	6.83	63.94	49.72	4.26
GAIF01011774.1	9.41	7.89	1.18	46.06	64.64	0.49
GAIF01011775.1	0.07	0.08	0	0.09	0	0

GAIF01011776.1	4.92	0.12	0.47	0	8.51	0
GAIF01011777.1	0.25	0	0.07	0.22	1.66	0.07
GAIF01011778.1	15.49	9.72	8.97	55.6	65.25	21.32
GAIF01011779.1	5.23	2.57	1.22	13.8	16.92	3.57
GAIF01011780.1	0	0.18	0	0	0.49	0.22
GAIF01011782.1	10.52	9.41	3.79	34.74	17.16	2.89
GAIF01011783.1	0	0	0	0.62	2.82	0
GAIF01011784.1	0.05	0.05	0	0	0.05	0
GAIF01011786.1	0	0.17	0.17	0.01	0	0.05
GAIF01011788.1	0	0	0	0.04	0.56	0
GAIF01011789.1	15.6	14.03	17.86	46.52	41.13	12.63
GAIF01011790.1	5.36	1.86	4.71	7.59	2.71	18.07
GAIF01011791.1	417.19	207.45	170.84	214.56	68.64	142.5
GAIF01011792.1	51.06	66.93	55.07	80.19	55.68	12.26
GAIF01011793.1	0.29	0	0	0	0	0
GAIF01011794.1	0	0	0	0.01	0.04	0
GAIF01011796.1	10.7	17.12	0	34.49	117.11	0.98
GAIF01011797.1	0	0.09	0	0.28	0.97	0
GAIF01011798.1	0	0.48	0.15	0	0	0.1
GAIF01011800.1	0.48	0.2	0	0.38	0.38	0.14
GAIF01011801.1	0.17	0	0	0	0	0
GAIF01011802.1	0	0	0	0	0.38	0
GAIF01011803.1	0	0.32	0	0	1.33	0
GAIF01011804.1	0	0	0	0	0	0.05
GAIF01011805.1	0.77	0.09	0.06	1.79	15.76	0.45
GAIF01011807.1	0	0.05	0	0.06	0.18	0
GAIF01011808.1	0	0	0	0	0.19	0
GAIF01011809.1	0	0.29	0	0	0	0
GAIF01011811.1	2.5	1.88	0	6.72	34.08	1.01
GAIF01011812.1	0.22	0	0	0.39	1.61	0.74
GAIF01011814.1	0.11	0.05	0	0.46	0.48	0
GAIF01011815.1	0	0	0	0	0	0.12
GAIF01011816.1	0.4	0.33	0	0.58	0.97	0
GAIF01011817.1	27.96	18.51	19.8	34.22	24.74	13.27
GAIF01011818.1	0	0.63	0	0	0	0
GAIF01011819.1	0.35	0.2	0.03	1.7	7.72	0
GAIF01011821.1	5.5	0.76	0.55	2.11	0.8	0.09
GAIF01011822.1	0.43	0.18	0.06	1.89	5.94	0.36
GAIF01011823.1	0.09	0.05	0	0.35	0.6	0.24
GAIF01011825.1	0.49	0.26	0.32	7.33	28.86	0.11
GAIF01011826.1	0.21	0.23	0.23	0	0.26	0
GAIF01011829.1	0.4	0.22	0	1.36	6.01	0.04
GAIF01011830.1	0.15	0.49	0.08	2.28	4.63	0.25
GAIF01011831.1	3.35	1.63	2.03	0.67	0.56	0.25

GAIF01011833.1	1.93	3.89	3.68	1.14	0	0.23
GAIF01011834.1	0	0	0	0	0.17	0
GAIF01011835.1	26.29	12.48	18.25	29.42	16.01	18.09
GAIF01011840.1	61.39	62.28	101.39	25.75	30.48	153.04
GAIF01011842.1	0.25	0.05	0	0.12	1.21	0.23
GAIF01011845.1	1.34	2.55	3.56	6.48	10.34	7.63
GAIF01011846.1	0.14	0.15	0.31	0	0.17	0
GAIF01011847.1	28.08	17.17	6.84	25.97	39.78	12.05
GAIF01011851.1	0	0	0	0.03	0	0
GAIF01011852.1	0.08	0	0	0.15	0.08	0
GAIF01011853.1	0	0	0	0.07	0	0
GAIF01011856.1	27.95	35.84	13.48	7.71	2.86	0.14
GAIF01011858.1	0	0	0	0.17	0.71	0
GAIF01011859.1	7.64	16.98	7.07	23.82	15.29	1.28
GAIF01011862.1	0.9	0.49	0.99	1.69	4.18	0
GAIF01011863.1	11.01	3.83	2.65	39.8	61.18	4.2
GAIF01011864.1	8.4	1.06	0.08	0	0	0
GAIF01011865.1	0	0	0	0	0.05	0
GAIF01011866.1	68.49	46.13	41.95	38.27	18.3	10.54
GAIF01011867.1	25.69	25.22	32.04	0.72	0.45	0.14
GAIF01011868.1	0.67	0	0	1.55	15.42	0.61
GAIF01011869.1	0	0	0.05	0	0	0.05
GAIF01011870.1	3.37	3.45	0	3.84	0	0
GAIF01011871.1	0	0.02	0	0	0.09	0
GAIF01011872.1	0	0	0	0.02	0.27	0
GAIF01011874.1	0	0	0	0.15	0.15	0
GAIF01011875.1	0	0.09	0	0.1	0.42	0
GAIF01011876.1	0	0.09	0	0.5	7.39	0
GAIF01011877.1	0.83	0.1	0	1.04	2.48	0
GAIF01011878.1	0	0	0	0	0.4	0
GAIF01011879.1	0.81	0.68	0.41	3.01	9.83	0.17
GAIF01011880.1	6.39	5.86	5.7	2.43	4.16	3.44
GAIF01011881.1	0	0	0	0.22	0	0
GAIF01011882.1	0.32	0	0.43	0	1.12	1.17
GAIF01011883.1	1.11	0.83	0.98	1.3	1.84	1.98
GAIF01011884.1	0	0	0	0	0	203.14
GAIF01011885.1	1.3	1.18	0.98	0.68	1.68	0.62
GAIF01011889.1	121.1	127.3	128.29	137.72	106.18	66.58
GAIF01011890.1	0	0	0.07	0	0	0
GAIF01011892.1	1225.3	1079.47	1255.89	2.37	4.27	2.73
GAIF01011894.1	0	0	0	0.11	0.24	0
GAIF01011896.1	0.16	0.09	0.04	0.05	0	0
GAIF01011897.1	4.19	2.68	0	2.6	9.94	2.21
GAIF01011898.1	1.17	3.07	1.59	3.6	2.91	0.36

GAIF01011899.1	43.71	9.5	47.98	0	0	0
GAIF01011901.1	1.11	0.4	0.61	0.64	0.22	0.25
GAIF01011902.1	2.76	6	3.03	3.2	3.32	0
GAIF01011903.1	1.16	0.37	0	4.12	24.05	3.28
GAIF01011904.1	0	0.01	0.04	0.47	2.18	0.22
GAIF01011905.1	0	0	0	0	0.17	0
GAIF01011906.1	21.26	17.59	3.19	109.16	82.94	4.5
GAIF01011908.1	0	0	0.05	0	0	0
GAIF01011909.1	17.41	13.03	12.21	16.14	16.36	9.97
GAIF01011910.1	0.25	0	0	0.32	0	0
GAIF01011911.1	0.8	0.74	0.75	2.25	1.78	0
GAIF01011913.1	2.26	0.57	0.66	18.5	53.62	3.56
GAIF01011915.1	36.79	31.81	36.42	47.66	15.91	25.97
GAIF01011916.1	17.97	11.33	9.61	52.32	74.4	6.18
GAIF01011917.1	1693.56	1250.16	1556.63	811.27	549.32	694.36
GAIF01011919.1	35.8	19.04	20.33	76.15	86.36	19.98
GAIF01011920.1	0	0.09	0	0	0	0
GAIF01011921.1	0	0	0.03	0	0	0
GAIF01011923.1	0.54	0	0	2.97	12.17	0.23
GAIF01011926.1	23.45	15.58	15.34	41.29	35.12	29.71
GAIF01011928.1	0	0.09	0.27	0	0.2	0.09
GAIF01011929.1	3.7	10.91	6.05	20.82	11.77	0.91
GAIF01011930.1	0	0	0	0.04	0.09	0
GAIF01011931.1	0.11	0.24	0.24	1.01	0.26	0.12
GAIF01011933.1	1.08	0	0.61	0	0	0
GAIF01011935.1	0.02	0.11	0	0.14	0.16	0.01
GAIF01011936.1	5.58	2.74	3.64	1.29	0	1.9
GAIF01011937.1	0.25	0.05	0.05	0.37	1.83	0
GAIF01011938.1	0.09	0.09	0.09	0.53	0.45	0.13
GAIF01011939.1	30.68	29.6	25.77	67.85	43.23	6.14
GAIF01011940.1	0.09	0.09	0	1.61	1.25	0.19
GAIF01011941.1	0	0	0	0	0	0.4
GAIF01011944.1	0	1.55	13.15	0	0	0.09
GAIF01011945.1	19.82	37.67	17.53	52.44	38.18	3.52
GAIF01011947.1	11.33	20.06	13.43	17.33	16.41	4.2
GAIF01011948.1	0.93	1.37	2.25	1.61	1.89	2.21
GAIF01011950.1	0	0	0	0.18	0.17	0
GAIF01011951.1	0	0.03	0.1	0.34	0	0
GAIF01011952.1	7.89	38.09	49.71	39.73	66.76	69.96
GAIF01011953.1	7.71	10.37	0	7.09	14.39	0.39
GAIF01011954.1	0	0.73	2.22	0	0.15	0.4
GAIF01011955.1	5.33	1.46	4.27	2.51	0.59	2.37
GAIF01011957.1	0	0.62	1.47	0	0	0.53
GAIF01011958.1	0	0.03	0	0.34	1.23	0.16

GAIF01011961.1	56.7	42.76	102.12	13	6.6	15.08
GAIF01011963.1	0.36	0.39	0.26	0	0.14	0
GAIF01011964.1	10.09	19.84	14.15	22.43	17.9	4.54
GAIF01011965.1	0	0.07	0	0	0	0
GAIF01011967.1	0.38	0	0	0	0	0
GAIF01011968.1	2.23	0.9	1.29	0	0	0
GAIF01011971.1	0	0.38	0	2.22	4.96	0.38
GAIF01011973.1	58.25	57.12	50.63	72.58	76.86	107.38
GAIF01011974.1	0	0	0	0.52	0.57	0
GAIF01011975.1	1.91	3.74	0.36	0	0.17	0.53
GAIF01011976.1	64.63	50.67	93.79	83.2	41.44	22.43
GAIF01011977.1	31.95	48.9	15.01	59.29	74.03	1.92
GAIF01011981.1	77.28	61.12	34.13	94.93	61.92	25.32
GAIF01011982.1	3.22	1.75	3.24	5.6	8.17	8.28
GAIF01011984.1	145.31	70.14	139.21	82.37	100.74	16.49
GAIF01011985.1	1.84	0.53	0	10.2	25.63	0.36
GAIF01011986.1	0	0	0	0.03	0.42	0
GAIF01011987.1	0	0	0	0	0.23	0.3
GAIF01011990.1	13.11	14.26	28.79	24.56	23.34	31.2
GAIF01011991.1	0.15	0.16	0	0.56	2.22	0
GAIF01011992.1	0	0	0	0.18	0.1	0
GAIF01011995.1	5.86	4.94	5.5	16.55	9.36	7.39
GAIF01011997.1	1.08	0.7	0.79	2.58	8.36	0.47
GAIF01011998.1	5.9	2.2	0.55	6.78	6.91	1.2
GAIF01012001.1	15.67	9.64	1.12	36.9	70.12	3.02
GAIF01012002.1	0.66	0.36	0	4.22	6.86	0
GAIF01012004.1	0.8	0.17	0.18	2.78	10.94	0.44
GAIF01012005.1	7.89	8.76	4.78	68.35	141.01	9.22
GAIF01012006.1	0	0	0	0.26	0	0
GAIF01012007.1	0	0	0	0	0.06	0
GAIF01012008.1	9.93	3.81	3.58	19.49	45.37	40.46
GAIF01012011.1	0	0	0	0	0.74	0
GAIF01012012.1	2.93	6.37	6.34	16.63	8.85	1.13
GAIF01012013.1	2.02	1.94	0.6	9.49	12.12	0.85
GAIF01012014.1	0	0	0.15	0	0	0
GAIF01012016.1	1.29	0.94	0.28	12.38	23.7	0.09
GAIF01012018.1	0.29	1.34	0	4.67	9.74	0.09
GAIF01012019.1	0.06	0.06	0.32	0	0	0.32
GAIF01012020.1	0.1	0	0	0	0	0
GAIF01012021.1	32.92	17.8	5.96	43.66	45.83	15.69
GAIF01012022.1	0.19	0.29	0.33	0.67	0.39	0.64
GAIF01012023.1	184.04	63.51	63.02	353.59	590.44	2448.96
GAIF01012024.1	40.73	2.12	0.4	18.6	240.24	616.58
GAIF01012025.1	0	1.36	0	0	0	0

GAIF01012027.1	9.13	13.1	9.83	33.63	37	5.37
GAIF01012028.1	30.17	0.12	0	0.02	0.79	0.05
GAIF01012031.1	0	0	0	0.47	1.16	0.25
GAIF01012034.1	0	0	0	0	0.86	0
GAIF01012037.1	0.06	0.06	0	0	0	0
GAIF01012038.1	0.81	0.15	0.15	2.03	31.05	1.58
GAIF01012039.1	0.03	0	0.04	0.31	0.32	0.1
GAIF01012040.1	8.17	4.91	4.54	28.62	21.14	6.54
GAIF01012041.1	0.1	0.06	0.23	0.58	0.62	1.39
GAIF01012042.1	0	0	0	0	0.13	0
GAIF01012043.1	0.56	0.3	0	1.73	3.04	0.15
GAIF01012044.1	0.05	0	0	0.27	1.62	0.31
GAIF01012045.1	0.07	0.07	0.05	0.2	0.07	0.04
GAIF01012047.1	0.47	0.53	0.72	0.14	0.08	0.32
GAIF01012050.1	0	0	0.03	0.07	0.21	0
GAIF01012052.1	0	0	0	0	0.16	0
GAIF01012053.1	0	0.22	0.56	0.65	0.58	0
GAIF01012055.1	0.33	0	0.04	0.28	0.33	0.1
GAIF01012057.1	0	0	0	0	0.1	0
GAIF01012058.1	0	0	0	0	0.37	0.01
GAIF01012059.1	0	0	0.65	3.04	2.58	0
GAIF01012061.1	5.95	7.46	6.03	5.44	3.85	1.13
GAIF01012062.1	11.56	20.89	12.19	6.79	3.97	3.31
GAIF01012063.1	0.31	0.25	0	0.27	0.33	0.08
GAIF01012064.1	0	0	0.19	0.2	0.2	0
GAIF01012066.1	4.26	2.91	0.53	2.39	2.56	0
GAIF01012067.1	0	0	0	0	0	0.08
GAIF01012069.1	0.06	0.03	0	0.26	1.12	0.2
GAIF01012071.1	0	0	0	1.45	3.53	0
GAIF01012072.1	0.16	0	0	0	0	0
GAIF01012077.1	1.97	5.44	1.59	3.05	3.17	0.15
GAIF01012078.1	0	0	0	1.7	3.52	0
GAIF01012081.1	1.84	2.52	1.61	4.2	6.27	1.64
GAIF01012083.1	0	0	0.33	0	0	0
GAIF01012084.1	1.41	1.5	0	2.88	1.64	2.85
GAIF01012086.1	4.89	4.79	5.37	25.9	59.81	8.12
GAIF01012087.1	0.06	0	0.06	0.07	0.21	0
GAIF01012088.1	0	5.35	4.75	0	0	0
GAIF01012089.1	0	0	0.5	0.51	2.12	0
GAIF01012090.1	0	0	0	0	2.63	0
GAIF01012091.1	17.4	11.1	13.33	22.43	20.59	16.53
GAIF01012092.1	0	0	0	0.7	2.16	0
GAIF01012093.1	7	1.14	0.11	14.07	36.06	9.44
GAIF01012096.1	0.46	0.24	0.42	2.17	4.56	0

GAIF01012097.1	0.1	0.22	0.11	1.4	4.13	0.47
GAIF01012098.1	0	0	0	2.08	1.12	15.09
GAIF01012099.1	0.28	0.1	0.1	0.54	1.45	0
GAIF01012101.1	1.25	1.11	2.94	0.59	0	0.39
GAIF01012103.1	24.19	17.15	18.92	2.5	1.06	2.37
GAIF01012104.1	0.09	0	0	0	0	0
GAIF01012105.1	0	0	0.17	0.36	5.79	0
GAIF01012107.1	0	0.08	0.16	0	0	0.16
GAIF01012108.1	0	0	0.14	0	0	0
GAIF01012109.1	0.27	0.24	0	1.19	6.93	0
GAIF01012111.1	1.03	1.4	2.15	0	0	0
GAIF01012113.1	82.11	52.68	52.96	83.92	45.46	20.19
GAIF01012114.1	14.07	5.88	1.78	32.1	93.11	1.97
GAIF01012115.1	2.46	0.53	0.08	2.36	3.29	0
GAIF01012116.1	1.39	1.63	2.84	6.71	6.94	4.47
GAIF01012118.1	0.3	0.26	0.26	0.21	0.36	0.33
GAIF01012119.1	14.7	18.37	6.41	43.21	84.18	0.98
GAIF01012121.1	0.07	0	0	0.04	0.17	0
GAIF01012122.1	0.18	0.85	0.25	0.18	0.75	0
GAIF01012123.1	0.22	0.06	0.06	0.65	1.22	0
GAIF01012124.1	0	0	0	0	0.14	0
GAIF01012125.1	8.49	9.98	9.08	19.03	23.94	3.8
GAIF01012126.1	51.02	26.48	12.96	23.44	30.9	8.62
GAIF01012127.1	0	0	0.25	0	0	0
GAIF01012128.1	23.08	45.19	6.75	30.47	28.3	1.36
GAIF01012129.1	0	0	0	0	0.37	0
GAIF01012131.1	12.04	12.2	5.54	5.26	3.22	0.91
GAIF01012132.1	8.86	19.7	15.54	24.87	14.32	3.21
GAIF01012133.1	69.79	64.34	43.12	104.69	123.85	71.75
GAIF01012134.1	0	0.25	0	0.27	4.18	0
GAIF01012135.1	0.37	0.1	0.19	0.99	1.27	2.54
GAIF01012136.1	0	0	0	0	0	0.37
GAIF01012140.1	57.1	36.94	68.58	51.21	29.22	31.46
GAIF01012142.1	8.18	10.27	6.12	5.35	2.68	0.49
GAIF01012144.1	0	0	0	0	0	0.14
GAIF01012145.1	0.04	0	0.04	0.19	0.4	0.04
GAIF01012146.1	700.15	634.08	892.01	362.4	236.56	217.34
GAIF01012147.1	0	0.01	0	0	0.49	0.04
GAIF01012148.1	0	0	0	0	0.03	0
GAIF01012150.1	2.26	0.86	0.02	0.86	5.08	0.45
GAIF01012152.1	17.24	13.27	8.01	2.77	1.12	0.44
GAIF01012153.1	6.3	1.83	0.49	15.14	41.51	3.35
GAIF01012154.1	0.11	0.11	0	1.58	7.58	2.08
GAIF01012155.1	0	0.13	0.88	0	0.21	0.44

GAIF01012159.1	7.53	4.48	4.03	16.51	24.02	2.88
GAIF01012160.1	0.4	0	0	1.78	5.68	3.55
GAIF01012162.1	104.33	337.28	326.06	16.46	38.03	23.44
GAIF01012163.1	0	0.08	0	0	0	0
GAIF01012165.1	0.21	0	0.02	0.21	0	0
GAIF01012166.1	0.18	0	0	0	0.22	0
GAIF01012168.1	0	0	0	0	0.89	0
GAIF01012169.1	0	0	0	0	0.22	0
GAIF01012170.1	7.34	3.51	2.98	5.56	6.28	0.24
GAIF01012172.1	69.47	21.43	25.49	96.29	120.52	77.11
GAIF01012173.1	0	0	0	0	0.33	0
GAIF01012174.1	0.45	0.37	0	0.78	2.44	0
GAIF01012175.1	0.12	0.4	0.13	2.98	13.66	0.13
GAIF01012176.1	12.85	15.91	10.19	11.28	13.16	2.19
GAIF01012177.1	0.58	0.06	0.34	0.1	0	0.28
GAIF01012179.1	14.25	7.35	7.36	20.28	27.8	25.82
GAIF01012180.1	52.86	44.53	56.73	56.35	31.07	45.64
GAIF01012185.1	0.17	0	0	0	0	0
GAIF01012188.1	1.01	1.08	1.51	1.16	0.7	0.26
GAIF01012191.1	1.55	1.52	0.6	0.52	0	0.23
GAIF01012192.1	6.05	5.02	2.17	1.5	1.39	0.27
GAIF01012194.1	40.33	70.73	91.55	35.17	33.83	14.46
GAIF01012196.1	1.37	1.49	0.98	6.5	6.16	0.2
GAIF01012197.1	12.32	16.01	20.91	37.24	20.21	11.64
GAIF01012198.1	8.61	10.31	2.83	30.42	47.48	1.51
GAIF01012200.1	4.41	0.5	0.32	5.27	12.48	30.27
GAIF01012203.1	0.41	0	0	0.19	0.89	0.09
GAIF01012206.1	88.04	5.6	33.84	1.98	0.5	0.58
GAIF01012207.1	0	0	0.6	0	0.39	0
GAIF01012208.1	2.34	1.66	1.38	1.36	0.87	0.4
GAIF01012210.1	0.36	0.13	0	0.46	2.88	0.48
GAIF01012212.1	2.23	1.58	1.65	1.46	0.6	1.11
GAIF01012215.1	4.23	3.15	1.11	24.13	40.47	0.68
GAIF01012217.1	0	0	0	0	0.43	0
GAIF01012218.1	0.5	1.28	0	0.19	1.01	0
GAIF01012219.1	28.31	4.43	4.66	18.32	4.38	2.14
GAIF01012220.1	0	0	0	0.01	0	0
GAIF01012221.1	0.19	0.14	0.07	0	0	0
GAIF01012222.1	0	0.12	0	0	0	0
GAIF01012224.1	0.08	0	0	0	0	0
GAIF01012225.1	14.21	17.74	8.9	30.4	25.68	2.62
GAIF01012226.1	8.83	14.49	7.78	0	0	0.17
GAIF01012227.1	1.82	0.38	0.22	0.68	1.88	0.47
GAIF01012228.1	0.22	0	0	0.51	0.81	0

GAIF01012231.1	0.09	0.1	0	0.94	2.5	0
GAIF01012232.1	7.52	7.59	8.99	13.08	15.69	6.03
GAIF01012233.1	17.98	18.35	19.74	15.93	27.21	15.91
GAIF01012234.1	0	0	0.08	0.08	0.43	0.08
GAIF01012238.1	31.36	27.65	53.23	81.82	46.54	88.23
GAIF01012239.1	36.42	42.03	63.37	72.18	53.15	48.82
GAIF01012240.1	0	0.03	0.03	0.07	0.04	0
GAIF01012241.1	31.01	17.19	26.24	89.23	53.54	124.05
GAIF01012242.1	0	0.06	0.02	0.64	2.02	0
GAIF01012243.1	0	0	0	1.96	5.76	0
GAIF01012245.1	0.24	0	0	0	6.97	0
GAIF01012246.1	31.32	20.97	24.27	68.16	100.58	41.47
GAIF01012247.1	33.93	87.42	30.48	49.95	49.24	9.81
GAIF01012248.1	0.86	4.81	3.72	3.77	1.83	0.96
GAIF01012250.1	0.6	0.57	0.59	0.65	0	0.03
GAIF01012251.1	5.93	7.88	2.12	30.84	52.86	1.56
GAIF01012252.1	0	0	0	0	0.05	0
GAIF01012256.1	38.26	5.37	11.32	35.93	29.61	78.35
GAIF01012257.1	0.36	0	0	0	0	0
GAIF01012258.1	0	0.15	0	0	0.65	0.3
GAIF01012259.1	0.95	0.69	1.17	1.18	0.15	0.63
GAIF01012260.1	108.19	26.74	8.11	10.38	18.49	1.88
GAIF01012261.1	1706.4	1125.81	709.01	1314.48	778.03	156.65
GAIF01012262.1	15.12	14.27	14.2	21.08	15.33	5.45
GAIF01012264.1	13.07	29.75	10.7	120.71	164.51	4.39
GAIF01012265.1	0.44	0.38	0	0	0.26	0
GAIF01012266.1	0	0	0	0	0	0.1
GAIF01012268.1	14.61	9.54	4.44	39.4	38.54	2.7
GAIF01012269.1	28.28	13.43	4.66	93.48	141.1	9
GAIF01012274.1	1476.46	923.06	861.86	21.53	14.43	11.84
GAIF01012275.1	18.14	27.75	34.72	41.56	26.48	13.34
GAIF01012276.1	0	1.33	0.3	0	0	0
GAIF01012277.1	0.75	0.21	0	8.51	17.61	0.43
GAIF01012279.1	0.38	0.25	0.41	1.41	2.71	0.1
GAIF01012280.1	1.01	0.85	0.25	7.65	28.35	0.12
GAIF01012282.1	0	0	0	0	0.4	0
GAIF01012284.1	0.31	0.72	0.05	1.95	1.17	0.1
GAIF01012285.1	1.95	0	0	0.81	0.97	0.36
GAIF01012287.1	1.54	1.83	0	7.64	19.71	2.01
GAIF01012289.1	0	0	0	1.12	2.6	0
GAIF01012290.1	0	0	0	0.04	0.05	0
GAIF01012291.1	0.25	0.14	0.14	0	0	0
GAIF01012292.1	256.03	622.29	280.06	208.37	1007.35	53.37
GAIF01012295.1	40.77	22.92	28.37	60.57	43.29	44.27

GAIF01012296.1	13.16	8.41	15.88	26.55	39.98	55.6
GAIF01012297.1	226.8	107.95	68.98	120.75	81.95	64.33
GAIF01012299.1	7.14	10.68	5.16	44.95	22.12	3.99
GAIF01012300.1	0.14	0	0	0.74	1.38	0.66
GAIF01012302.1	7.96	7.87	9.74	34.73	44.57	3.54
GAIF01012304.1	0	0	0	2.51	9.87	0
GAIF01012305.1	7.97	2.75	0.28	12.92	49.76	0.7
GAIF01012307.1	1.46	0	0.27	3.11	15.07	1.29
GAIF01012309.1	12.15	17.59	11.87	47.59	32.49	5.87
GAIF01012311.1	0.04	0.23	0	0	0.05	0
GAIF01012312.1	0	0	0	0	0.11	0
GAIF01012313.1	0	0	0.19	0	0	0.08
GAIF01012314.1	59.64	49.75	55.97	140.02	123.39	26.2
GAIF01012315.1	0.18	0	1.31	0	0	0
GAIF01012316.1	7.7	8.72	4.32	18.12	30.67	3.01
GAIF01012317.1	1086.63	457.27	605.68	712.12	947.62	7085.11
GAIF01012319.1	1.09	0.2	0	2.42	18.97	0.1
GAIF01012320.1	0.32	0.33	0	0.36	0.12	0
GAIF01012321.1	5.67	8.45	4.27	2.16	1.7	0.2
GAIF01012322.1	7.71	11.67	14.78	14.93	12.65	6.7
GAIF01012323.1	13.75	44.92	8.34	43.03	85.85	0.73
GAIF01012324.1	69.58	43.1	10.35	203.29	282.82	22.81
GAIF01012327.1	7.37	6.21	4.65	9.97	12.47	9.17
GAIF01012328.1	0	0	0	0.14	1.41	0
GAIF01012329.1	7.12	2.11	0	1.71	2.37	1.77
GAIF01012330.1	16.24	10.57	5.78	41.44	63.69	2.99
GAIF01012332.1	0	0	0	0	0	0.02
GAIF01012333.1	4.11	1.94	4.91	1.14	0.98	2.46
GAIF01012334.1	0.1	0.35	0.33	0.18	0.12	0
GAIF01012336.1	0	0	0.12	0	0	0
GAIF01012337.1	0.1	0	0.11	1.14	3.3	0
GAIF01012339.1	0	0	0	0.55	0	0.29
GAIF01012340.1	0	0.05	0	0	0	0
GAIF01012341.1	0	0	0.23	0	0	0
GAIF01012342.1	0	23.82	21.92	0	0.34	2.69
GAIF01012343.1	24.16	11.51	14.8	49.27	78.72	26.05
GAIF01012344.1	0	0.47	0.38	0.2	0.53	0.49
GAIF01012345.1	0.53	1.16	1.1	1.2	1.22	0.17
GAIF01012348.1	0	0	0.1	0	0	0.2
GAIF01012349.1	0	0.08	0.15	0	0.5	0
GAIF01012351.1	0	0	0.07	0.08	0	0
GAIF01012353.1	0	0	0	0	0	0.06
GAIF01012354.1	0	0	0.04	0	0	0
GAIF01012355.1	4.95	10.08	9.59	23.89	22.19	6.62

GAIF01012356.1	11.17	13.43	10.37	28.95	22.3	4.19
GAIF01012357.1	0	0	0	0.07	0.28	0
GAIF01012358.1	0.02	0	0	0	0.03	0
GAIF01012359.1	0.47	0.06	0.13	3.4	12.54	0.65
GAIF01012361.1	0	0.09	0	0	0.1	0
GAIF01012362.1	0.11	0	0	4.73	18.3	0
GAIF01012363.1	0	0.63	0.13	0	1.26	0
GAIF01012365.1	0	0	0	0	0.18	0
GAIF01012368.1	7.24	2.02	0	5.9	28.94	4.71
GAIF01012369.1	0.15	0.15	0.12	1.12	4.21	0.47
GAIF01012370.1	198	197.71	82.21	699.76	800.03	83.14
GAIF01012371.1	5.12	4.33	6.72	13.38	13.35	5.96
GAIF01012372.1	0.03	0	0	0.12	0.16	0
GAIF01012373.1	6.13	7.46	7.19	30.49	34.16	4.13
GAIF01012374.1	0.6	0	0	0.1	0.1	0
GAIF01012375.1	0	0	0.05	0.4	1.01	0.11
GAIF01012376.1	0	6.35	0	2.09	1.24	0
GAIF01012377.1	156.17	25.95	2.09	47.09	53.95	0
GAIF01012380.1	0	0.01	0	0.01	0	0
GAIF01012383.1	40.83	22.71	24.46	37.91	65.26	39.38
GAIF01012384.1	0.11	0.24	0.48	0.77	0.15	0.12
GAIF01012385.1	4460.22	2535.84	4106.12	3328.01	1958.73	10845.81
GAIF01012387.1	17.91	26.64	4.04	22.9	52.84	13.72
GAIF01012389.1	0	0	0	0	0	0.12
GAIF01012390.1	0	0	0	0.19	0.12	0
GAIF01012391.1	32.53	28.96	12.98	40.98	36.51	36
GAIF01012392.1	0.78	0.34	1.54	1.45	0.19	0.34
GAIF01012393.1	0	0.05	0	0	0	0
GAIF01012394.1	0	0	0.06	0	0	0
GAIF01012396.1	0	0.43	0.18	0.06	0	0.06
GAIF01012397.1	0	0	0.08	0.09	0	0
GAIF01012398.1	7.01	11.92	19.06	2.22	1.24	26.42
GAIF01012399.1	2.01	2.73	1.55	5.37	11.49	4.99
GAIF01012400.1	342.21	163.27	85.72	65.76	46.9	20
GAIF01012401.1	0	0.16	0	0	0.09	0
GAIF01012402.1	0.57	0.31	0.04	0.05	0.16	0
GAIF01012404.1	0	0	0	0	0	0.31
GAIF01012405.1	9.66	11.13	7.78	16.83	18.06	2.89
GAIF01012406.1	0	2.74	1.2	0	0	0.37
GAIF01012409.1	0.13	0.03	0.03	0.18	1.38	0.14
GAIF01012410.1	0	0	0	0	0.05	0
GAIF01012412.1	0.42	0.68	0.46	0.24	1.25	0
GAIF01012415.1	0	0	0.05	0.09	0.07	0
GAIF01012416.1	8.98	9.97	1.48	12.42	13.21	7.89

GAIF01012418.1	0	0	0	0.05	0.44	0
GAIF01012420.1	7.94	7.75	9.45	20.2	23.92	6.66
GAIF01012421.1	0	0.13	0	0	0	0.65
GAIF01012425.1	0	0	0	0	0.1	0
GAIF01012426.1	0	0	0	0.63	0.16	0.15
GAIF01012428.1	1.87	1.76	0.75	0.69	1.02	0
GAIF01012431.1	0	0	0	0	0.25	0
GAIF01012432.1	0.45	0.28	0.04	4.71	15.06	0.42
GAIF01012433.1	4.48	2.55	7.73	1.24	0.38	2.7
GAIF01012435.1	0.54	0.24	0	0	0.07	0.4
GAIF01012436.1	0.36	0	0	0.93	5.05	0.39
GAIF01012437.1	0	0	0.11	0.12	0.44	0
GAIF01012438.1	1.31	0.28	0.18	4.53	11.63	17.08
GAIF01012439.1	0.25	0.69	0.21	0.22	0.08	1.47
GAIF01012440.1	4.96	1.26	0.12	0.74	1.27	0
GAIF01012442.1	0.26	0	0	0	0	0
GAIF01012443.1	6.35	6.34	4.18	22.58	23.59	4.18
GAIF01012445.1	0.13	0.36	0.29	4.12	0	0.07
GAIF01012446.1	0.1	0.14	0	0.93	0.55	0.22
GAIF01012447.1	0.13	0	0	0.39	0	0.13
GAIF01012450.1	0.99	1.08	0.57	0.88	0.9	0.6
GAIF01012451.1	10.23	9.72	10.21	32.72	38.87	7.36
GAIF01012452.1	0.11	0	0	0	0	0
GAIF01012453.1	0	0	0.22	0.63	2.94	0
GAIF01012454.1	24.07	43.46	18.48	56.63	64.73	5.93
GAIF01012456.1	0.38	0.1	0	0	0.22	0
GAIF01012457.1	0	0	0	0.16	0	0
GAIF01012458.1	15.92	10.95	9.14	18.35	29.5	14.64
GAIF01012459.1	19.85	10.65	20.02	61.74	54.5	18.81
GAIF01012461.1	1.51	0.28	0.7	0.89	1.54	5.2
GAIF01012462.1	0	0.07	0	0	0.08	0
GAIF01012464.1	0.1	0	0	0.17	0.12	0
GAIF01012465.1	0.92	0.57	0.4	5.56	17.34	0.43
GAIF01012466.1	0.1	0	0	0	0	0.05
GAIF01012467.1	0	0.03	0	0.03	0.04	0
GAIF01012468.1	5.08	2.98	3	0.76	0.47	0.14
GAIF01012469.1	0.74	0.41	0.31	3.99	0.8	0.1
GAIF01012470.1	0	0.4	0	1.23	1.19	0.04
GAIF01012472.1	0.02	0.05	0.03	0.06	0.39	0.01
GAIF01012473.1	14.51	8.49	8.58	19.43	9.39	24.6
GAIF01012474.1	0	0	0.07	0	0.42	0
GAIF01012477.1	0.19	0.14	0	0	0.08	0
GAIF01012478.1	0.08	0.27	0.09	0.1	0.79	0.18
GAIF01012479.1	1.65	1.96	3.19	69.02	46.14	304.7

GAIF01012481.1	2.31	0.42	0.22	12.41	45.36	0.98
GAIF01012482.1	0	0	0	0.27	0.87	0
GAIF01012483.1	0	0	0.06	0	0	0
GAIF01012484.1	0.46	0.2	0	0	0	0.31
GAIF01012485.1	37.79	55.31	92.93	40.68	36.77	40.22
GAIF01012486.1	0	0	0.32	0	0	0
GAIF01012488.1	0.51	0.18	0.37	0.29	0.51	0
GAIF01012490.1	0	0.24	0	0	0	0
GAIF01012491.1	1.08	0.35	0.53	0.18	0.75	0.23
GAIF01012492.1	0	0.08	0.2	0	0.04	0.4
GAIF01012493.1	0.12	0	0	6.7	4.49	2.95
GAIF01012494.1	18.17	19.64	28.7	30.71	21.97	12.84
GAIF01012496.1	17.11	13.48	10.26	47.04	63.75	3.22
GAIF01012498.1	19.64	6.16	5.58	39.79	68.97	171.59
GAIF01012499.1	0.08	0	0	0.06	0.27	0
GAIF01012500.1	19.5	15.72	10.55	42.82	27.56	7.55
GAIF01012502.1	7.61	16.29	3.46	19.82	17.84	0.21
GAIF01012506.1	0	0	0	0.06	0.26	0
GAIF01012507.1	28.67	20.84	35.01	69.27	53.66	88.19
GAIF01012509.1	0	0	0	0.18	0.57	0
GAIF01012510.1	0	0.14	0	0	0	0
GAIF01012512.1	10.2	16.17	10.37	39.47	43.39	4.15
GAIF01012513.1	0.19	0.19	0	0	0	0
GAIF01012514.1	0.09	0	0.36	0.11	0.5	0
GAIF01012515.1	11.36	7.28	21.16	28.73	31.72	14.09
GAIF01012517.1	210.82	159.08	79.6	208.85	181.61	30.7
GAIF01012518.1	0.26	0	0	0	0	0
GAIF01012521.1	15.6	9	3.98	19.28	16.74	0.73
GAIF01012522.1	6.92	6.88	4.04	8.43	9.75	0
GAIF01012523.1	157.12	157.3	164.3	97.33	105.03	463.02
GAIF01012524.1	4.78	1.02	0.24	2.04	2.47	1.2
GAIF01012525.1	0.14	0	0	0	0.16	0.45
GAIF01012527.1	0.39	0.31	0.15	0.74	0.68	0
GAIF01012528.1	0	0	0	0.11	0	0
GAIF01012529.1	0	0	0	0	0.1	0
GAIF01012530.1	0.03	0.04	0	0	0.13	0
GAIF01012532.1	0.59	0.85	0.65	0	0.19	0
GAIF01012533.1	0.14	1.46	0	1.37	5.72	0.17
GAIF01012536.1	0.05	0	0.05	0.05	0.11	0.05
GAIF01012537.1	2.57	2.8	0.26	10.99	24.47	1.55
GAIF01012538.1	0	0.05	0.02	0	0.17	0.16
GAIF01012539.1	61.27	39.46	16.28	14.9	19.02	16.55
GAIF01012540.1	0	0.05	0.26	0.01	0.03	0.13
GAIF01012541.1	15	19.29	13.34	39.83	33.66	4.21

GAIF01012542.1	0.21	0	0	0.12	0.51	0
GAIF01012545.1	0.27	0.15	0.3	1.35	1.81	0.98
GAIF01012548.1	0.24	0.17	0.35	1.46	10.21	0.09
GAIF01012549.1	0	0	0	0	2.19	0
GAIF01012551.1	0.29	0	0	0.68	1.16	0
GAIF01012552.1	0	0	0	0	0	0.12
GAIF01012554.1	0	0	0	0.14	0	0
GAIF01012555.1	0	0.37	0	0	0.56	0
GAIF01012556.1	0.19	0.07	0.16	2.3	9.87	0
GAIF01012557.1	4.86	0	0.43	7.45	2.81	18.66
GAIF01012558.1	0.09	0	0.05	0	0.17	0
GAIF01012559.1	0	0	0.09	0.09	0	0
GAIF01012560.1	0.15	0.07	0	0	0	0
GAIF01012561.1	0	0	0	0.1	0	0
GAIF01012562.1	0	0.15	0	1.4	4.68	0.15
GAIF01012565.1	17.07	12.9	11.78	7.32	6.28	8.61
GAIF01012567.1	0	0	0.17	0	0.09	0
GAIF01012569.1	14.92	36.69	17.72	45.97	31.17	5.39
GAIF01012570.1	3.86	2.86	3.58	3.06	1.46	1.41
GAIF01012571.1	6.24	0	0	4.06	0	0.11
GAIF01012572.1	2.11	1.45	0.49	6.39	23.82	1.66
GAIF01012573.1	0.79	1.3	0.87	9.22	5.73	6.57
GAIF01012574.1	0.09	0.14	0.09	0.45	0.31	0
GAIF01012575.1	0	0.47	0.05	0	1.12	0.16
GAIF01012577.1	24.83	12.01	10.47	36.09	49.13	23.22
GAIF01012578.1	0	0	0	0	0.36	0
GAIF01012579.1	0	0	0	0	0.11	0
GAIF01012581.1	0	0	0	0.01	0	0
GAIF01012582.1	0.77	0.28	0.14	0.15	0	0.43
GAIF01012584.1	0	0	0	0.16	0.31	0
GAIF01012586.1	7.42	7.72	9.2	20.05	16.24	6.7
GAIF01012587.1	0	0.04	0	0	0	0
GAIF01012589.1	10.41	17.15	27.23	44.79	32.41	11.64
GAIF01012590.1	211.85	128.88	22.66	4328.54	3689.93	6.61
GAIF01012591.1	0.32	0.35	0.71	0.94	7.56	1.07
GAIF01012593.1	0.68	1.28	0.15	0.63	1.85	0.15
GAIF01012594.1	0.47	0.64	0.13	0.27	0.14	0
GAIF01012595.1	0.15	0	0	0	0.18	0
GAIF01012596.1	1.26	0.34	0.09	3.02	13.71	1.39
GAIF01012597.1	42.15	24.43	19.49	62.02	121.36	175.28
GAIF01012599.1	7.41	6.25	3.61	15.61	19.46	3.36
GAIF01012600.1	0.12	0	0	0.44	2.29	0.21
GAIF01012602.1	0.22	0.32	0.32	0.39	0.5	0
GAIF01012603.1	32.13	38.84	19.57	31.36	18.3	0.01

GAIF01012604.1	6.01	2.75	2.86	2.02	2.47	2.18
GAIF01012605.1	9.25	8.46	5.15	24.67	1.54	1.35
GAIF01012606.1	111.58	188.94	109.69	302.32	261.36	62.81
GAIF01012607.1	0	0	0	0	0.11	0
GAIF01012608.1	0	0	4.16	1.45	0	0.42
GAIF01012610.1	24.2	2.68	3.05	17.2	5.91	4.52
GAIF01012612.1	0	0	0	0	0.1	0
GAIF01012613.1	0.68	0.38	0.11	0.83	0.98	0.26
GAIF01012614.1	0.46	1	0	0.35	0.3	0.21
GAIF01012615.1	0	0	0	0.27	1.02	0
GAIF01012616.1	0.05	0	1.78	0.68	0	0
GAIF01012617.1	0.35	0.46	0.08	0.73	1.1	0.04
GAIF01012619.1	0.18	0.19	0	1.63	6.43	1.16
GAIF01012621.1	28.07	17.79	11.37	45.52	46.41	10.01
GAIF01012622.1	0	0	0	0	0.13	0
GAIF01012625.1	130.34	60.02	99.71	99.63	51.93	69.75
GAIF01012626.1	5.11	5.12	0.51	32.34	50.27	0.29
GAIF01012627.1	0	0	0	0.11	0	0
GAIF01012629.1	54.78	22.51	38.22	46.04	54.36	187.04
GAIF01012631.1	2.79	5.47	3.27	1.73	0.67	0
GAIF01012632.1	16.03	30.31	17.47	6.07	6.01	2.05
GAIF01012633.1	0.45	0	0	0.78	4.61	0.17
GAIF01012635.1	0	0	0	0.29	0.91	0
GAIF01012636.1	5.58	4.41	4.35	14.21	10.61	3.18
GAIF01012637.1	7.76	7.62	6.39	14.35	6.83	0.68
GAIF01012638.1	17.64	19.96	18.36	120.1	147.64	11.8
GAIF01012640.1	13.43	17.03	8.32	68.39	58.74	3.36
GAIF01012641.1	16.06	27.82	25.03	44.28	37.23	8.94
GAIF01012643.1	6.81	12.46	6.41	1.56	0.63	0.25
GAIF01012644.1	3.37	1.52	2.8	4.97	9.7	1.81
GAIF01012645.1	14.3	20.74	15.7	0	11.47	15.76
GAIF01012646.1	2.42	0.13	0.09	6.79	4.9	0.16
GAIF01012647.1	18.1	26.86	19.77	31.57	38.39	13.94
GAIF01012650.1	49.96	52.81	33.6	53.76	66.97	55.25
GAIF01012651.1	0.39	0.68	0.61	2.41	3.24	1.54
GAIF01012652.1	0.33	1.77	0.67	1.05	1.5	0.37
GAIF01012653.1	0.13	0	0.68	0.78	0.06	1.33
GAIF01012654.1	0.27	0	0	2.44	12.14	0.13
GAIF01012655.1	0.04	0.02	0	0.08	0.23	0
GAIF01012656.1	0	0.14	0	0	0	0
GAIF01012658.1	110.69	107.58	57.69	321.57	168.45	52.67
GAIF01012659.1	61.1	28.78	29.73	70.78	108.6	164.87
GAIF01012663.1	0	0.12	0.12	0	0	0
GAIF01012665.1	13.66	12.43	22.01	24.99	23.57	45.08

GAIF01012666.1	0	0.06	0.06	0.3	2.35	0.6
GAIF01012667.1	0	0	0	0	0.32	0.2
GAIF01012668.1	0	0	0	0.18	0.38	0
GAIF01012669.1	63.56	93.78	82.09	30.03	32.79	33.2
GAIF01012670.1	0.45	0	0	2.62	6.42	1
GAIF01012672.1	0.22	0	0.08	0	0	0.18
GAIF01012673.1	0	0	0	0	0.19	0.03
GAIF01012675.1	1852.86	1288.55	2450.05	1016.25	469.87	1454.78
GAIF01012676.1	0.3	0.8	0.09	3.85	16.38	0.05
GAIF01012677.1	8.33	7.87	12.35	5.93	6.55	5.89
GAIF01012678.1	0.46	1.44	1.58	2.27	2.21	0.32
GAIF01012679.1	5.93	6.62	2.41	17.05	44.86	0.41
GAIF01012680.1	0	0.08	0.03	0.2	0	0.05
GAIF01012681.1	0	0.05	0.05	0	0.13	0
GAIF01012682.1	0	0	0.06	0	0	0
GAIF01012684.1	27.53	8.83	0	11.09	30.69	38.93
GAIF01012685.1	0	0	0	0.38	1.48	0
GAIF01012686.1	0	0	0	0.06	0.07	0
GAIF01012688.1	0.06	0	0.07	0.29	0.23	0.21
GAIF01012690.1	0	0	0	0.06	0	0
GAIF01012692.1	0	0	0	0.13	0.57	0.12
GAIF01012693.1	1.19	0.91	0.13	11.22	22.53	0
GAIF01012694.1	14.56	10.51	7.99	5.93	1.42	0
GAIF01012695.1	19.35	6.82	2.82	37.42	41.2	82.78
GAIF01012696.1	1.1	0.24	1.62	1.14	0.13	0.66
GAIF01012697.1	5.03	1.5	0.64	11.27	16.52	5.81
GAIF01012698.1	0.25	0.33	0.07	0.48	1.67	0.27
GAIF01012700.1	0	0	0	0.42	0.66	0
GAIF01012701.1	0.69	0.59	1.46	1.55	2.68	0.21
GAIF01012703.1	0.35	0	0.05	1.46	7.45	0.1
GAIF01012705.1	25.38	15.75	20.41	49.61	66.33	23.11
GAIF01012706.1	0.11	0.12	0	0.26	0.49	0.06
GAIF01012707.1	0	0	0	0	0.05	0
GAIF01012708.1	0.37	0.85	0.57	0.93	0.9	0.14
GAIF01012709.1	0.1	0	0	0	0	0
GAIF01012710.1	0.12	0.39	0.2	0	0.11	0.2
GAIF01012711.1	0	0.06	0	0	0.13	0
GAIF01012712.1	0	0.17	0	0.73	3.47	0
GAIF01012714.1	1.15	2.29	0.37	15.81	29.42	0.05
GAIF01012716.1	5.14	0.52	0	5.88	17.46	2
GAIF01012718.1	0	0	0	0.69	0	0.14
GAIF01012719.1	0	0	0.16	0	0	0
GAIF01012721.1	0	0	0	0	0.1	0
GAIF01012722.1	0.43	0	0	1.93	7.84	0.96

GAIF01012724.1	7.95	3.4	2	2.12	1.25	1.01
GAIF01012725.1	0.07	0	0	0	0	0
GAIF01012726.1	19.03	36.66	42.43	49.39	45.35	16.19
GAIF01012727.1	0.46	0.1	0.1	2.64	11.41	0.51
GAIF01012729.1	28.28	38.82	54.17	112.43	42.93	18.13
GAIF01012730.1	0.14	0	0	0.06	0.56	0.06
GAIF01012732.1	10.4	8.97	6.98	38.69	30.7	3.33
GAIF01012733.1	11.6	14.27	7.17	10.3	2.63	2.45
GAIF01012734.1	0	0	0	0.17	0.18	0
GAIF01012735.1	0.92	0	0	4.82	12	4.17
GAIF01012737.1	0.09	0.1	0	0.08	0	0.04
GAIF01012738.1	0.24	0	0	0	0.23	0.03
GAIF01012740.1	0	0	0	0.14	0	0
GAIF01012741.1	0.02	0	0	0	0.32	0
GAIF01012742.1	0.13	0.29	0	3.09	16.79	0.44
GAIF01012743.1	0	0.05	0.01	0.03	0	0.03
GAIF01012744.1	0	0	0	0	0.01	0
GAIF01012746.1	0	0.04	0	0.04	0	0
GAIF01012747.1	0.44	0.2	0.22	0.43	2.75	0.04
GAIF01012748.1	0.05	0	0.08	0	0	0
GAIF01012749.1	0.12	0	0	0.48	2.17	0
GAIF01012750.1	32.99	23.45	15.81	58.39	60.98	22.11
GAIF01012751.1	1.9	2.46	1.59	0.53	0.11	0.1
GAIF01012752.1	11.14	11.76	6.71	40.13	40.53	2.52
GAIF01012754.1	128.21	161.52	150.13	146.74	59.12	26.62
GAIF01012756.1	0	0.06	0	0	0	0
GAIF01012757.1	1185.89	748.31	1677.22	516.41	135.04	1072.25
GAIF01012759.1	13.39	16.91	9.28	84.66	68.89	5.14
GAIF01012760.1	0	0	0	0	0	0.14
GAIF01012761.1	0	0.17	0	0.62	3.27	0
GAIF01012762.1	17.65	16.13	19.09	42.7	16.98	33.42
GAIF01012763.1	0.1	0.03	0	0.15	0.24	0
GAIF01012766.1	8.07	15.73	2.45	27.2	22.86	1
GAIF01012767.1	111.91	116.5	124.13	112.53	83.1	84.25
GAIF01012769.1	0.3	0.29	0.08	0.61	0.59	0.25
GAIF01012770.1	0.08	0.08	0.31	0.25	0.27	0
GAIF01012772.1	0.06	0.35	0.21	0.31	0.23	0.11
GAIF01012773.1	0.04	0.05	0.05	0.1	0.36	0
GAIF01012774.1	0	0	0	0.05	0	0
GAIF01012775.1	12.1	53.41	28.23	43.8	58.35	14.21
GAIF01012776.1	3.27	1.93	1.51	0.87	0.93	0.05
GAIF01012777.1	0.34	0.12	0.03	3.4	8.17	0.06
GAIF01012778.1	0.1	0.22	0.11	0	0	0
GAIF01012779.1	0	0.52	0.6	0	0	0

GAIF01012780.1	0.2	0.12	0	1.66	7.78	0.22
GAIF01012781.1	0.64	0.19	0.27	1.95	5.07	0.47
GAIF01012783.1	18.51	17.06	5.01	13.74	13.15	0.45
GAIF01012785.1	0	0	0	0.04	0.36	0
GAIF01012786.1	0	0	0	0	0.45	0
GAIF01012787.1	0	0.43	0	0.28	0.1	0
GAIF01012788.1	0.07	0.02	0	0	0	0
GAIF01012790.1	2.35	1.05	0.53	9.61	39.56	0.32
GAIF01012792.1	12.55	4.39	8.22	7.96	5.44	14.74
GAIF01012793.1	1.35	0.54	0.11	3.43	5.91	0.17
GAIF01012794.1	0.15	0	0	0.43	2.7	0.04
GAIF01012795.1	3.18	0.16	0	13.33	32.47	1.19
GAIF01012796.1	19.68	39.68	1.88	9.82	30.13	0.76
GAIF01012797.1	22.87	24.6	13.17	67.82	44.59	4.08
GAIF01012799.1	0.23	0.06	0	0.16	2.19	0
GAIF01012800.1	0.19	0.07	0.06	0.39	0.99	0.28
GAIF01012802.1	10.34	14.61	12.17	23.61	18.01	5.69
GAIF01012803.1	0.9	0.49	0	1.74	3.79	0.17
GAIF01012806.1	1.47	1.4	2.43	0	0	0.1
GAIF01012808.1	0.28	0	0	0.31	0.65	0.09
GAIF01012809.1	0.09	0	0	0.44	0.11	0
GAIF01012810.1	79.72	44.81	27.96	6.89	4	3.34
GAIF01012811.1	14.51	24.39	23.9	52.39	30.72	8.83
GAIF01012812.1	17.99	21.64	19.26	19.48	26.44	4.4
GAIF01012813.1	1.95	1.64	0.71	0.09	0.16	0.26
GAIF01012814.1	3.86	3.54	2.41	4.06	4.98	6.13
GAIF01012815.1	10.81	6.21	10.52	16.32	12.26	10.39
GAIF01012816.1	9.35	7.24	10.95	27.04	17.16	11.92
GAIF01012817.1	8.22	21.18	7.52	3.57	5.14	0.76
GAIF01012819.1	0.27	0.22	0	2.31	7.74	0.51
GAIF01012820.1	0	0	0.25	0	0	0
GAIF01012822.1	0	0	0	0.11	0.69	0
GAIF01012824.1	86.42	63.04	63.79	133.59	88.29	313.05
GAIF01012825.1	0.08	0.08	0	0.35	2.11	0
GAIF01012826.1	0	0	0.08	0.08	0	0
GAIF01012827.1	0	0	0.02	0.02	0.02	0
GAIF01012828.1	0	0	0.03	0	0	0
GAIF01012829.1	9.88	11.21	19.99	29.66	12.39	7.47
GAIF01012831.1	1239.99	967.31	281.01	6921.58	7984.63	763.94
GAIF01012832.1	6.68	15.11	9.51	27.86	15.44	1.67
GAIF01012833.1	9.67	20.86	15.88	13.91	13.23	5.43
GAIF01012834.1	5.3	8.57	7.89	8.93	8.95	4.66
GAIF01012836.1	0.13	0	0	0	0.69	0
GAIF01012837.1	0	0	0	0.07	0	0

GAIF01012838.1	0.46	0	0	0.32	3.76	0.91
GAIF01012843.1	20.77	20.97	24.42	17.3	20.09	23.55
GAIF01012844.1	142.07	131.47	143.44	201.65	135.2	97.62
GAIF01012845.1	0	0	0	0.59	0.68	0.19
GAIF01012846.1	147.14	80.56	133.71	3.68	3.49	8.63
GAIF01012848.1	2.31	3	1.36	11.18	6.56	1.96
GAIF01012849.1	0	0	0	0.32	0.17	0
GAIF01012850.1	0	0	0	0.86	1.48	0
GAIF01012851.1	0.29	0	0	1.01	2.1	16.14
GAIF01012853.1	0.45	0	0.08	0.27	2.76	0
GAIF01012854.1	34.71	41	33.53	34.18	38.35	22.41
GAIF01012855.1	9.66	7.05	12.06	19.76	17.55	38.67
GAIF01012856.1	0	0	0	0.06	0	0
GAIF01012857.1	0.19	0	0	1.37	0.05	0.21
GAIF01012858.1	0.64	0.51	0.19	0.72	1.65	0.59
GAIF01012859.1	0.14	0	0	0	0	0
GAIF01012862.1	0.7	0.1	0.12	1.75	5.51	0.19
GAIF01012863.1	0	0	0	0	0.4	0
GAIF01012864.1	0	0	0	0	0.31	0
GAIF01012866.1	35.97	25.59	17.46	0	0	0
GAIF01012867.1	0	0	0	0	0.06	0
GAIF01012870.1	4.14	2.88	7.97	6.16	3.23	3.45
GAIF01012871.1	11.72	9.17	0	26.13	39.79	2.14
GAIF01012872.1	0.25	0.48	0.71	1.04	0.66	1.2
GAIF01012874.1	4.64	8.09	16.98	14.18	10.4	20.04
GAIF01012876.1	1.38	0.58	0.12	2.7	4.33	0.23
GAIF01012877.1	0.12	0.16	0.04	0.14	0.16	0.12
GAIF01012878.1	0	0	0	0.09	0.22	0
GAIF01012879.1	0	0.23	0	0	3.18	0
GAIF01012881.1	8.95	2.4	3.35	18.8	38.39	80.19
GAIF01012883.1	0	0.07	0	0	1.3	0
GAIF01012884.1	0	0	0	0	0.04	0
GAIF01012885.1	2.39	0.91	3.21	2.24	0.83	1.99
GAIF01012886.1	12.55	12.85	2.73	34.06	48.45	0.35
GAIF01012887.1	0.5	0	0	1.35	3.4	3.3
GAIF01012888.1	0.17	0	0	0	0.49	0
GAIF01012889.1	0.09	0.19	0	1.84	4.86	0
GAIF01012890.1	0	0	0	0	0.06	0
GAIF01012891.1	1.16	0	0.06	4.84	7.42	0.61
GAIF01012892.1	0.44	0.12	0	2.28	7.73	0
GAIF01012893.1	0	0	0	0	0	0.14
GAIF01012894.1	14.54	5.75	5.7	20.49	12.83	10.2
GAIF01012895.1	0	0	0	0	0.31	0
GAIF01012899.1	0	0	0	0.1	0.05	0

GAIF01012900.1	0.4	0	0.68	0.8	9.28	0.51
GAIF01012902.1	0.16	0	0	0.37	1.91	0
GAIF01012903.1	0.1	0	0	0.22	0.7	0
GAIF01012904.1	0.13	0.14	0.14	2.12	2.83	0
GAIF01012906.1	0.09	0.9	0.2	0.75	2.67	0
GAIF01012907.1	0.85	0.64	0.35	3.88	7.86	1.76
GAIF01012908.1	0.07	0	0.16	0.5	1.38	0.08
GAIF01012909.1	6.54	1.42	0.57	7.59	10.07	1.44
GAIF01012910.1	10.33	20.19	1.49	22.41	48.21	0.81
GAIF01012911.1	1.68	1.01	1.24	2.94	3.27	1.12
GAIF01012912.1	0	0.05	0.06	0.06	0.61	0
GAIF01012913.1	0	0	0.08	0.08	0	0
GAIF01012914.1	0	0	0	0	0.13	0
GAIF01012915.1	8.56	10.75	6.48	20.15	17.21	0.92
GAIF01012916.1	0	0	0	0.13	0	0
GAIF01012917.1	1366.49	140.99	330.65	47.82	6.24	36.86
GAIF01012919.1	0	0	0	0.25	0.9	0
GAIF01012922.1	0	0	0	0	3.36	0
GAIF01012923.1	0.04	0	0	0	0.39	0
GAIF01012926.1	4.93	2.45	0.48	72.03	222.06	1.89
GAIF01012928.1	1.46	0.22	1.25	2.64	1.38	2.33
GAIF01012931.1	4.85	3.75	2.48	6.31	9.35	3.61
GAIF01012932.1	45.95	31.77	19.92	82.01	54.02	10.35
GAIF01012934.1	0	0	0.06	0	0	0
GAIF01012935.1	0	0	0.15	0	0.16	0.1
GAIF01012936.1	0	0.03	0.03	0.03	0.03	0
GAIF01012937.1	0.32	0	0	0	2.5	0
GAIF01012939.1	0	0	1.11	0.38	0.53	8.45
GAIF01012940.1	4.64	2.5	1.94	15.04	9.77	0.09
GAIF01012941.1	10.71	0	0	0.67	0	0
GAIF01012942.1	0	0.17	0	0.54	0.93	0.17
GAIF01012943.1	0	0	0	13.63	0	0
GAIF01012944.1	51.69	77.84	54.01	90.49	134.85	16.12
GAIF01012945.1	0	1.2	0	0	0	0
GAIF01012946.1	0.08	0.09	0	0.16	0.19	0
GAIF01012947.1	0.07	0	0	0.08	0.44	0.24
GAIF01012948.1	6.12	2.38	2.18	1.39	1.26	0.55
GAIF01012950.1	0	0.25	0.19	0.3	1.58	0
GAIF01012951.1	0	0	0	0	0.2	0
GAIF01012953.1	26.96	10.1	6.94	34.64	13.77	1.23
GAIF01012954.1	1.4	0.35	0.18	5.56	14.05	0.71
GAIF01012955.1	0.12	1.63	0.82	2.75	6	0
GAIF01012957.1	0	3.14	0	5.01	12.91	0
GAIF01012958.1	0.07	0.11	0	0.69	4.27	0.11

GAIF01012959.1	0.34	0.37	0.53	0.08	0	0.15
GAIF01012960.1	1.04	1.05	2.16	1.94	1.16	1.47
GAIF01012961.1	2.13	0.86	5.19	0.76	1.04	0.54
GAIF01012962.1	2.55	1.81	0.69	0.81	0.65	0.13
GAIF01012963.1	0.29	0.1	0	1.27	11.06	0.84
GAIF01012965.1	7.08	7.99	7.17	3.16	5.37	1.26
GAIF01012966.1	2.84	2.88	0.31	11.97	28.1	5.42
GAIF01012967.1	0.92	0.21	0.22	2.47	7.19	0.34
GAIF01012972.1	0	0	0	0.19	0.82	0
GAIF01012973.1	0	0	0	0.05	0	0
GAIF01012974.1	0.7	0.49	0.48	1.02	1.06	0.29
GAIF01012976.1	0	0	0	0.06	0.07	0
GAIF01012977.1	10.42	11.33	7.34	21.87	19.28	1.91
GAIF01012978.1	7.28	2.93	0.53	38.23	106.92	4.77
GAIF01012979.1	0	0	0	0	0.05	0
GAIF01012983.1	0.44	0	0	1.15	4.37	0
GAIF01012985.1	0.3	0.65	0	0.53	0	0.3
GAIF01012986.1	0.19	0.06	0.19	0.39	5.34	0.32
GAIF01012988.1	0	0	0	0	1.07	0.04
GAIF01012990.1	0.08	0	0	0	0	0
GAIF01012991.1	103.67	52.36	83.05	58.17	43.32	56.36
GAIF01012992.1	0	0	0	0	0	0.1
GAIF01012993.1	0.37	0	0	0	0	0
GAIF01012994.1	0	0	0	0	0.1	0
GAIF01012995.1	5439.92	6276.91	11796.25	2969.64	2023.17	7947.56
GAIF01012996.1	0	0	0	2.72	0.88	0.84
GAIF01012997.1	1.57	0.52	0.22	2.29	2.44	12.53
GAIF01012998.1	2.96	2.02	1.4	0.74	0.63	0.45
GAIF01012999.1	1.82	0.82	0.47	0.9	6.62	16.43
GAIF01013000.1	50.4	50.55	20.59	74.13	69.2	9.09
GAIF01013001.1	0.38	0.25	0.16	0.49	0.3	0.21
GAIF01013002.1	5.16	1.53	0	11.65	31.89	5.29
GAIF01013003.1	0.57	0.17	0	0.38	0.35	0
GAIF01013006.1	0	0.31	0	0	0	0
GAIF01013008.1	27.42	38.56	32.17	39.21	33.7	9.7
GAIF01013009.1	4.35	2.67	2.02	2.65	3.19	9.15
GAIF01013010.1	3.96	16.17	2.7	2.37	17.06	0
GAIF01013012.1	0	0	0	0.18	1.02	0.17
GAIF01013013.1	0	0	0	0.79	2.13	0.15
GAIF01013014.1	2.44	1.23	0.48	15.15	35.8	0.19
GAIF01013015.1	30.22	6.5	1.99	27.53	50.96	73.81
GAIF01013016.1	0.51	0	0	2.76	5.44	0
GAIF01013017.1	0	0.05	0.16	0	0	0
GAIF01013018.1	0	2.24	0	2.39	3.71	2.27

GAIF01013019.1	0.06	0.12	0.09	2.3	7.96	0.17
GAIF01013020.1	1.51	0.31	0.18	26.85	173.14	7.86
GAIF01013021.1	2.34	3.18	2.27	4.55	6.21	0.38
GAIF01013022.1	1.26	0.11	0	0.97	1.39	1.96
GAIF01013024.1	1.94	0	0	3.53	24.63	0.92
GAIF01013025.1	0.44	0.69	0.17	5.52	9.59	0.61
GAIF01013027.1	7.24	6.78	2.69	23.02	30.7	1.18
GAIF01013028.1	0	0	0	0.15	0	0
GAIF01013029.1	0.09	0	0	0.42	0.61	0
GAIF01013031.1	0.1	3.89	0.75	9	2.84	0.77
GAIF01013032.1	0	0.41	0.33	0	0	0.08
GAIF01013034.1	1.16	0.52	0.28	2.63	7.26	1.57
GAIF01013036.1	4.84	1.55	0.5	10	37.89	11.2
GAIF01013037.1	38.66	34.83	39.82	78.05	46.36	48.08
GAIF01013038.1	2.74	1.53	1.12	0.45	0.28	0.09
GAIF01013039.1	0.26	0	0.06	0.36	2.1	0
GAIF01013041.1	1.39	1.72	1.09	6.74	10.36	1.27
GAIF01013042.1	0.79	0	0	1.12	5.25	0.15
GAIF01013044.1	0	0	0	0	0.77	0.14
GAIF01013045.1	0.77	3.34	0	7.12	6.69	0
GAIF01013047.1	0.19	0	0	0.72	0.35	0
GAIF01013048.1	21.55	36.79	16.15	68.72	49.18	6.29
GAIF01013049.1	0	0.22	0.23	3.72	5.34	0
GAIF01013051.1	7.14	5.73	3.12	21.81	22.88	8.22
GAIF01013052.1	1.33	0.12	0.35	3.3	21.65	2.77
GAIF01013054.1	30.9	19.32	16.45	66.47	42.61	34.82
GAIF01013055.1	115.42	113.43	54.91	78.59	106.56	83.22
GAIF01013056.1	1.73	0.61	0.3	0	0	0.64
GAIF01013057.1	45.68	26.04	28.93	69.77	64.04	93.56
GAIF01013058.1	0.07	0.04	0.08	0.68	1.82	0.11
GAIF01013060.1	0.59	0.64	1.08	0.34	1.3	0.87
GAIF01013061.1	1.57	1.05	1.43	1.24	0	0
GAIF01013062.1	120.69	64.86	106.72	156.33	71.63	394.58
GAIF01013064.1	0	0.04	0.05	0	0	0
GAIF01013067.1	19.95	34.16	34.65	48.91	44.67	10.69
GAIF01013069.1	0	0	0	0.3	0.34	0
GAIF01013071.1	0.87	0.82	0.41	1.48	1.21	0.18
GAIF01013072.1	14.53	14.39	18.21	18.27	9.51	11.23
GAIF01013075.1	0.04	0	0	0	0	0
GAIF01013077.1	0.03	0	0.07	0	0	0
GAIF01013078.1	1.05	2.15	0.74	1.01	2.58	0.61
GAIF01013079.1	0	0	0	0	0.12	0
GAIF01013080.1	124.07	176.09	96.88	144.61	93.36	19.96
GAIF01013081.1	98.82	53.86	37.97	89.35	105.29	139.55

GAIF01013082.1	14.41	8.01	4.53	23	48.08	17.5
GAIF01013083.1	0	0	0	0	0.14	0
GAIF01013085.1	0.21	0.06	0.17	0.79	1.69	1.55
GAIF01013086.1	0.1	0.16	0.33	0.41	0.42	0.17
GAIF01013088.1	0.15	0.1	0.19	0.03	0.15	0.07
GAIF01013089.1	0.11	0	0	0	0	0
GAIF01013092.1	46.72	50.66	67.93	107.01	68.04	66.43
GAIF01013093.1	0.33	1.9	0.12	0.26	0.27	0
GAIF01013094.1	9.47	7.93	7.65	10.59	11.96	7.54
GAIF01013095.1	7.63	7.74	14.02	21.88	23.23	11.63
GAIF01013098.1	4.64	0.81	0.2	9.18	20.8	11.36
GAIF01013099.1	13.28	10.18	32.29	9.29	11.86	9.27
GAIF01013100.1	0.69	1.52	0.4	2.16	1.75	0.37
GAIF01013101.1	0.74	0	0	2.5	0	0
GAIF01013102.1	0	0.19	0	0.07	0.3	0.03
GAIF01013103.1	0	0.04	0	0	0	0
GAIF01013104.1	5733.83	4461.19	7283.82	1419.6	1351.47	891.07
GAIF01013106.1	0	0.04	0.05	0	1.05	0.01
GAIF01013107.1	0.08	0	0	0.09	0.19	0
GAIF01013109.1	0	0	0	0	0.79	0
GAIF01013111.1	22.96	16.52	12.26	56	78.13	7.24
GAIF01013112.1	2.21	2.02	2.92	0.58	1.15	0.34
GAIF01013114.1	0	1	1.21	0	0	1.01
GAIF01013115.1	0.12	0.06	0	0.06	0.1	0
GAIF01013116.1	0	0.08	0	0.26	0.74	0.09
GAIF01013117.1	0.11	0	0	0.06	0	0
GAIF01013118.1	29.97	6.71	0	8.7	9.01	74.32
GAIF01013119.1	0	0	0	0.14	0	0
GAIF01013120.1	0	0	0	0	0.13	0
GAIF01013121.1	0.63	0.23	0.23	0.73	1.13	0.23
GAIF01013122.1	1.29	0.21	0	0	1.91	0
GAIF01013123.1	0.04	0.04	0	0	0	0
GAIF01013125.1	0.78	0.47	0	0.1	0.21	0.1
GAIF01013126.1	19.32	44.02	14.92	76.48	45.24	3.04
GAIF01013127.1	0	0	0.09	0.4	0.18	0
GAIF01013128.1	0	0	0	0.61	1.91	0.15
GAIF01013129.1	0.96	0.57	0.38	4.35	9.86	0.19
GAIF01013130.1	0	0	0	0	0.48	0.55
GAIF01013133.1	0	0.4	0	0	0.19	0.24
GAIF01013134.1	6.92	11.18	6.09	18.32	13.34	2.09
GAIF01013135.1	0	0	0	0	0.2	0
GAIF01013137.1	0	0	0	0.07	0	0
GAIF01013138.1	3.36	2.18	0.31	14.15	26.24	2.27
GAIF01013139.1	0.22	0.16	0.08	1.02	7.04	0

GAIF01013142.1	5.51	1.93	1.74	9	3.78	5.17
GAIF01013143.1	0.15	0	0	0	0.18	0
GAIF01013144.1	1.73	0.94	0	1.95	3.77	0.12
GAIF01013145.1	3.88	0	0	0.8	0	0
GAIF01013147.1	0.53	0.48	0.1	0.41	0.21	0
GAIF01013149.1	15.07	13.27	12.39	30.24	27.33	4.56
GAIF01013150.1	0.38	1.91	1.28	0.45	0.48	0.9
GAIF01013152.1	0.99	0.88	7.34	0	0	9.41
GAIF01013153.1	0.06	0	0.26	0	0	0.07
GAIF01013154.1	44.64	18.36	7.46	95.39	121.6	15.87
GAIF01013155.1	17.55	12.92	10.38	50.49	52.13	9.78
GAIF01013156.1	34.98	26.25	13.74	59.22	54.5	3.66
GAIF01013157.1	0.01	0	0	0	0.03	0
GAIF01013158.1	0	0	0	0.05	0.05	0
GAIF01013159.1	7.08	3.82	4.28	16.59	10.48	3.43
GAIF01013160.1	0	0	0	0.34	0.85	0
GAIF01013161.1	0.16	0	0	0	1.16	0.36
GAIF01013163.1	1.07	0	0	0	0	0
GAIF01013165.1	0	0	0	0	0.1	0
GAIF01013166.1	0	0	0	0.3	0.39	0
GAIF01013167.1	35.36	53.48	32.12	45.39	29.55	7.29
GAIF01013169.1	35.99	22.69	14.42	47.4	26.61	5.35
GAIF01013171.1	34.17	21.03	23.59	60.79	98.15	85.13
GAIF01013172.1	390.3	286	291.45	0.72	1.26	0.21
GAIF01013173.1	3.32	1.91	0.11	17.21	41.66	0.54
GAIF01013176.1	0	0.02	0	0	0.05	0.19
GAIF01013177.1	5.27	3.57	2.32	0.95	0.42	0.39
GAIF01013178.1	5.05	4.18	5.6	11.45	6.54	2.35
GAIF01013180.1	0	0	0	0	0.01	0
GAIF01013181.1	0.41	0.19	0.05	2.58	8.96	0.09
GAIF01013182.1	1.31	1.43	0.16	3.05	3.68	0
GAIF01013183.1	0.11	0.02	0	0.1	2.66	0.02
GAIF01013185.1	54.96	62.29	85.41	114.1	81.46	35.55
GAIF01013186.1	1.65	0.67	0	10.79	38.15	0.57
GAIF01013187.1	8.7	5.03	4.18	2.52	1.11	0.24
GAIF01013188.1	91.78	85.54	129.56	928.27	457.28	491.31
GAIF01013189.1	0	0	0.04	0	0.05	0.19
GAIF01013191.1	0.44	0	0	0	1.94	0
GAIF01013192.1	0.27	0	0.15	0	0	0
GAIF01013193.1	0.23	0.27	0.08	0.36	1.59	0.14
GAIF01013194.1	0.85	0.38	0.08	0.45	0.3	0.16
GAIF01013195.1	0	0	0.06	0	0.02	0
GAIF01013196.1	0	0	0	0.19	0	0
GAIF01013197.1	0.14	0.29	1.63	2.04	1.79	1.04

GAIF01013199.1	0.51	0.08	0	0.59	1.41	0
GAIF01013200.1	93.41	45.15	37.32	12.41	10.04	2.56
GAIF01013201.1	0.17	0	0	0.6	0.21	0
GAIF01013205.1	0.04	0	0	0	0	0
GAIF01013208.1	174.44	118.68	139.16	615.17	294.64	402.59
GAIF01013209.1	0	0	0.45	0	0	0
GAIF01013210.1	0	0.16	0	0	0	0
GAIF01013212.1	0	0	0	0.6	0.53	0
GAIF01013213.1	0.23	0	0	0	0	0
GAIF01013214.1	1.05	2.74	1.54	0.49	0.51	0
GAIF01013217.1	0	0.08	0	0	0	0
GAIF01013218.1	16.41	37.55	27.78	58.01	34.74	6.8
GAIF01013219.1	0.15	0	0	0.27	1.67	0.08
GAIF01013220.1	1661.8	989.55	1274.56	1553.43	631.18	484.04
GAIF01013221.1	0	0	0.47	0	0	0
GAIF01013225.1	0.5	0.41	0.14	1.16	1.05	0.27
GAIF01013227.1	0	0	0	0	0	0.24
GAIF01013228.1	7.9	4.7	4.91	7.59	6.55	20.5
GAIF01013229.1	0.5	0.04	0.1	0.47	1.03	1.3
GAIF01013230.1	133.78	130.76	147.44	0.2	0.42	0
GAIF01013231.1	0.53	0	0	0.73	5.64	0
GAIF01013232.1	0	0	0	0.41	0	0
GAIF01013233.1	3.55	7.13	2.83	9.75	18.1	0.43
GAIF01013234.1	3.43	3.73	0.75	1.19	2.34	0
GAIF01013235.1	0.96	0.17	0.18	0	0.96	0
GAIF01013236.1	0.58	0.27	0.27	0.38	0.4	0.09
GAIF01013237.1	0.12	0.34	0.17	3	8.91	0
GAIF01013238.1	0	0.04	0	0	0.04	0
GAIF01013240.1	0.48	0	0	1.62	4.66	1.05
GAIF01013241.1	0.06	0	0	0.15	0.68	0.12
GAIF01013244.1	0.23	0.42	0.1	0.43	0.51	0.02
GAIF01013248.1	0	0.35	0.16	0.19	0.2	0.32
GAIF01013249.1	0.23	0.08	0	0.54	1.4	0.09
GAIF01013250.1	0	0	0	0.29	0.9	0
GAIF01013251.1	4.74	7.29	0.39	17.39	88.83	0
GAIF01013252.1	0.47	0.68	0.34	1.46	3.39	0.52
GAIF01013253.1	52.61	47.89	51.28	171.66	110.54	57.55
GAIF01013256.1	180.48	92.96	84.1	125.51	72.47	91.66
GAIF01013261.1	9.89	9.42	3.25	20.59	15.32	3.26
GAIF01013263.1	76.23	99	124.8	39.44	41.97	71.52
GAIF01013264.1	0.07	0.04	0	0.04	0	0
GAIF01013265.1	0.1	0.2	0	0.22	0	0
GAIF01013266.1	0	0.16	0	0.17	0.51	0
GAIF01013267.1	29.99	82.86	42.03	21.19	29.06	5.2

GAIF01013269.1	0	28.92	23.17	0	0.11	2.64
GAIF01013270.1	0.11	0.06	0.06	0	0.25	0
GAIF01013271.1	42.18	52.9	42.89	48.82	42.87	25.03
GAIF01013272.1	0	0	0	0.11	0	0
GAIF01013273.1	0.28	0.56	0.43	1.06	0.63	0
GAIF01013274.1	0	0	0.07	0	0	0
GAIF01013275.1	0	0.12	0	0.24	0.25	0
GAIF01013276.1	43.83	40.68	51.2	87.78	49.01	36.08
GAIF01013277.1	913.28	853.34	1004.77	1614.47	637.88	307.4
GAIF01013278.1	45.21	27.66	47.57	28.17	21.64	7.53
GAIF01013279.1	13.64	20.78	6.54	4.34	1.09	0
GAIF01013281.1	0.05	0.06	0	0.18	0.06	0
GAIF01013282.1	0.11	0	0.12	0.13	0.83	0.24
GAIF01013283.1	1.59	1.49	0.51	0.66	0.54	0.39
GAIF01013284.1	0	6.49	3.51	0	0	0.34
GAIF01013285.1	29.46	43.96	38.88	125.59	108.56	33.06
GAIF01013286.1	32.55	154.38	65.74	23.1	62.82	6.05
GAIF01013287.1	14.45	22.25	14.77	39.2	26.33	4.73
GAIF01013288.1	2.48	4.88	0.98	3.7	11.34	2.84
GAIF01013289.1	27.05	28.75	12.29	21.33	36.07	12.03
GAIF01013290.1	32.48	17.72	20.58	5.27	1.68	1.8
GAIF01013291.1	0.02	0	0	0	0	0
GAIF01013295.1	1.57	0.85	1.46	4.23	3.26	0.93
GAIF01013296.1	0.08	0	0	0	0.32	0.03
GAIF01013297.1	0.08	0.08	0	0.44	3.8	0.58
GAIF01013298.1	0	0	0	0	0.13	0
GAIF01013301.1	0	0	0	0.43	2.45	0
GAIF01013303.1	5.05	5.19	2.57	2.39	2.7	0.72
GAIF01013304.1	1.11	1.84	0.37	0.62	0.21	0
GAIF01013305.1	0.07	0.08	0	0.67	2.25	0.08
GAIF01013306.1	0.04	0.09	0	0	0.8	0
GAIF01013308.1	0.08	0.08	0.24	0.17	0.07	0
GAIF01013309.1	17.83	20.48	16.06	11.91	15.04	7.85
GAIF01013310.1	0	0	0	0.78	1.64	0
GAIF01013311.1	0	0.21	0	0.15	0.47	0
GAIF01013312.1	0.11	0	0	0.88	1.57	0
GAIF01013313.1	26.86	25.52	22.11	19.68	15.72	13.46
GAIF01013314.1	0	0	0	0	0.13	0
GAIF01013315.1	0	0	0.1	0	0	0
GAIF01013316.1	0.31	0	0	0.12	0.13	0
GAIF01013318.1	0	0.07	0	0.07	0.07	0
GAIF01013319.1	1.85	0.7	0.58	11.11	48.08	1.16
GAIF01013320.1	0	0.52	0.05	0	1.65	0.14
GAIF01013321.1	0.13	0.65	0.14	0.44	0.2	0.09

GAIF01013322.1	0	0.13	0	0	0	0
GAIF01013323.1	1.3	4.06	2.01	1.59	3.49	0.17
GAIF01013324.1	74.12	47.11	53.48	39.86	2.43	15.35
GAIF01013326.1	9.25	5.34	10.71	23.81	12.56	11.16
GAIF01013327.1	13.58	21.12	19.65	36.26	20.1	10.96
GAIF01013328.1	0	0	0	0.47	2.34	0
GAIF01013329.1	0	3.97	2.87	2.35	2.41	0
GAIF01013330.1	1.16	0	0	0	0	0
GAIF01013331.1	0	0	0.1	0	0	0
GAIF01013334.1	0	0.11	0.46	0	0	0.21
GAIF01013335.1	0	0	0	0	0.16	0
GAIF01013337.1	30.28	42.52	29.45	61.3	52.55	7.88
GAIF01013338.1	16.06	11.57	7.05	27.09	9.97	3.05
GAIF01013339.1	0.25	0.23	0.14	1.02	3.44	0.05
GAIF01013340.1	0.21	0.29	0.24	0.31	0.45	0.06
GAIF01013341.1	0	0	0	0	0.23	0
GAIF01013342.1	0	0	0	0	0.14	0
GAIF01013344.1	25.68	41.19	30.21	53.45	31.99	10.15
GAIF01013346.1	0	0.19	0.33	0	0	0.13
GAIF01013347.1	2.57	2.92	2	3.78	7.24	1.62
GAIF01013349.1	0	0.11	0.11	0.14	0	0.12
GAIF01013350.1	0.34	0	0.12	0.92	7.18	0.5
GAIF01013351.1	0.59	1.02	0.51	0	0.28	0.13
GAIF01013353.1	2.46	2.55	3.41	1.97	1.09	5.31
GAIF01013354.1	0.65	0.28	0	6.15	25.66	1.14
GAIF01013357.1	5.36	5.82	3.92	6.77	5.65	2.86
GAIF01013359.1	0	0	0.13	0	0	0
GAIF01013360.1	2.35	1.43	1.53	12.39	7.64	4.55
GAIF01013361.1	8.95	16.82	4.11	13.02	7.63	0.81
GAIF01013362.1	0	0	0.1	0	0.47	0
GAIF01013364.1	49.42	0.17	5.46	32.1	7.1	36.14
GAIF01013365.1	1.72	1.21	0	3.87	7.16	0
GAIF01013366.1	1.73	2.61	4.8	2.72	2.11	0.97
GAIF01013367.1	0	0	0.05	0.02	0	0
GAIF01013369.1	0	0.02	0	0.06	0.46	0
GAIF01013370.1	0	0	0	0.83	2.67	0.29
GAIF01013371.1	0	0	0	0	0.14	0
GAIF01013375.1	1.92	0.18	0	3.8	15.84	1.96
GAIF01013376.1	0.06	0.13	0.06	0.8	1.73	0
GAIF01013378.1	46.94	64.76	52.24	92.95	67.77	25.45
GAIF01013381.1	66.73	32.86	63.53	105.98	83.53	184.58
GAIF01013382.1	0.43	0	0.25	0.62	1.01	0.28
GAIF01013383.1	1.79	5.08	6	3.11	0	1.28
GAIF01013384.1	6.57	5.69	2.93	1.43	0.49	0.11

GAIF01013386.1	0.12	0	0	1.92	0	0.91
GAIF01013388.1	0	0.13	0	0	0	0
GAIF01013389.1	0.66	6.1	0.39	2.77	10.84	1.07
GAIF01013390.1	0	2.26	0	2.03	0	1.26
GAIF01013391.1	0	0	0	0.62	1.68	0
GAIF01013395.1	44.46	43.02	32.28	93.46	114.09	16.43
GAIF01013396.1	2.04	0	0	0	0	0
GAIF01013397.1	0.12	0	0.35	0.46	0	0
GAIF01013400.1	1.09	2.04	0.33	4.24	6.53	0.16
GAIF01013403.1	0	0	0	2.33	0	0.21
GAIF01013404.1	39.79	36.65	28.6	49.56	50.6	49.46
GAIF01013405.1	0.53	0.14	0.06	1.48	5.43	3.51
GAIF01013406.1	0.04	0.04	0	0.02	0.04	0.03
GAIF01013407.1	17.41	9.44	11.76	7.77	6.06	11.48
GAIF01013408.1	0.77	0.77	0.77	0.59	0.42	0.65
GAIF01013409.1	36.13	16.53	10.38	11.47	17.4	2.8
GAIF01013412.1	2.36	2.38	0.92	1.76	0.4	0.74
GAIF01013414.1	0	0	0	0.25	0.52	0.08
GAIF01013415.1	3.71	2.16	1.29	2.03	4.71	1.58
GAIF01013416.1	0.04	0	0	0.15	0.66	0
GAIF01013417.1	19.23	21.88	25.42	31	42.62	19.23
GAIF01013418.1	0	0	0.16	0	0	0
GAIF01013419.1	15.98	18.5	23.77	3.59	7.13	5.68
GAIF01013420.1	0	0	0	0	0.12	0
GAIF01013421.1	0.77	0.23	0.07	1.87	4.97	0.13
GAIF01013422.1	10.9	3.03	1.87	13.34	0.85	0.27
GAIF01013423.1	0.1	0.02	0.13	0.09	0.11	0.04
GAIF01013425.1	0	0	0	0	0.11	0
GAIF01013426.1	54.71	36.89	37.95	116.9	50.99	104.25
GAIF01013427.1	0	0.11	0	0.12	0.96	0
GAIF01013428.1	0.21	0.36	0	2.43	14.37	0
GAIF01013429.1	0.48	0.64	2.58	0.46	3.34	1.84
GAIF01013432.1	5.87	6.41	10.26	17.7	41.4	23.1
GAIF01013433.1	0	0	0	0	0.42	0
GAIF01013435.1	0	0.13	0	0	0	0
GAIF01013436.1	0.59	0.37	0.09	0.79	10.79	0.09
GAIF01013437.1	0	12.87	16.53	0	0	3.37
GAIF01013438.1	0.08	0	0	0	0.06	0
GAIF01013439.1	0	0	0	0.09	0	0
GAIF01013441.1	0.02	0.04	0	0.08	1.14	0.04
GAIF01013442.1	0.51	0.58	0.51	0.34	1.21	0.05
GAIF01013443.1	33.19	30.23	26.85	3.83	4.95	4.27
GAIF01013444.1	501.32	826.75	1792.46	351.09	200.87	479.83
GAIF01013445.1	27.64	19.44	23.75	17.32	10.06	21.07

GAIF01013446.1	0.35	0	0	0.41	4.12	2.35
GAIF01013447.1	0	0	0	0	0.44	0
GAIF01013449.1	0	0.1	0.31	0.22	0.23	0
GAIF01013452.1	5.73	7.52	3.14	51.2	60.24	6.26
GAIF01013453.1	0	0	0	0.26	4.28	0.12
GAIF01013454.1	2.85	2.32	1.17	4.68	0	2.17
GAIF01013455.1	0.2	0.38	0.29	0.3	0.3	0.17
GAIF01013456.1	0	0	0	0	0	0.1
GAIF01013457.1	0	0	0	0	0.61	0
GAIF01013458.1	0.02	0.05	0.02	0.18	1.93	0.07
GAIF01013459.1	0.48	0.41	0.21	0.22	1.49	0.21
GAIF01013460.1	2.87	3.12	0.53	3.06	5.47	0
GAIF01013462.1	0.05	0	0	0	0.4	0
GAIF01013463.1	0	0	0	0.03	0.03	0
GAIF01013464.1	2.57	4.4	1.33	3.76	2.92	0.6
GAIF01013465.1	0	0.17	0	0.54	2.22	0
GAIF01013466.1	0	0	0	0.11	0.17	0
GAIF01013469.1	0.53	0.08	0.08	3.85	11.88	0.12
GAIF01013470.1	0	0.03	0	0.27	0.8	0.07
GAIF01013471.1	4.99	2.47	2.59	4.8	1.56	3.41
GAIF01013472.1	0	0.08	0.16	0	0.24	0.05
GAIF01013474.1	10.68	14.69	4.55	25.53	26.6	1.18
GAIF01013475.1	308.52	597.1	1195.63	769.83	500.63	344.45
GAIF01013476.1	2.36	0.22	0.49	21.28	55.08	18.38
GAIF01013477.1	0.12	0.25	0	0	0	0
GAIF01013478.1	47.32	61.59	71.35	56.42	37.47	13.6
GAIF01013479.1	0	0	0	0.13	0	0
GAIF01013480.1	19.7	25.42	34.57	42.22	44.76	22.07
GAIF01013482.1	25.04	72.62	28.6	78.08	65.61	0.37
GAIF01013484.1	0.26	0.11	0	1.03	7.29	0.19
GAIF01013485.1	0.23	0	0	0.24	0	0.08
GAIF01013486.1	0	2.56	3.8	0	0	0.2
GAIF01013488.1	12.87	7.05	20.23	23.89	12.01	64.32
GAIF01013489.1	73.2	88.04	77.79	76.43	69.33	31.77
GAIF01013490.1	0.15	0.16	0	0.52	0.54	0
GAIF01013491.1	9.63	5.94	4.24	16.65	12.26	3.69
GAIF01013492.1	0.13	0	0	0	0	0.14
GAIF01013493.1	0.85	0	0	0	0	0
GAIF01013494.1	3.35	2.39	0	5.24	1.72	0
GAIF01013501.1	0.09	0	0	0	0.33	0
GAIF01013502.1	15.59	20.97	16.93	23.49	0	14.39
GAIF01013505.1	2.32	1.21	3.86	5.08	4.84	1.7
GAIF01013506.1	0	0	0	0.04	0.08	0
GAIF01013507.1	0	0.02	0	0.28	1.24	0

GAIF01013509.1	0.21	0.13	0.21	0.15	0.15	0.17
GAIF01013510.1	0	0	0	0	0.01	0
GAIF01013511.1	26.46	26.51	16.25	154.81	70.11	28.53
GAIF01013515.1	0	0.39	0	0	0	0.07
GAIF01013516.1	0	0	0	0	10.74	0
GAIF01013517.1	0.69	0.6	0	4.32	18.9	1.82
GAIF01013518.1	0	0	0	0	0.33	0
GAIF01013519.1	46.19	42.58	123.33	58.07	35	74.06
GAIF01013520.1	0	0	0	0	0.03	0
GAIF01013521.1	1.89	0.85	0.41	1.5	5.23	0.68
GAIF01013522.1	19.07	28.66	10.94	5.55	3.59	0.35
GAIF01013523.1	1.1	1.14	0.26	1.15	1.47	0
GAIF01013525.1	0	0.68	1.37	0	0	0
GAIF01013526.1	0	0.05	0.59	0	0.12	0.16
GAIF01013527.1	11.02	16.28	14.74	25.38	17.12	8.18
GAIF01013528.1	0.07	0	0	0	0.25	0
GAIF01013529.1	0.22	0.33	0.27	0.73	0.4	0.59
GAIF01013530.1	9.74	11.53	8.53	21.9	28.18	6.52
GAIF01013531.1	0.11	0.16	0	0.12	0.14	0
GAIF01013532.1	8.09	11.54	2.47	17.31	20.24	2.73
GAIF01013533.1	3.37	12.66	9.11	8.11	7.81	1.25
GAIF01013534.1	1.13	1.84	2.48	0.66	2.04	0
GAIF01013535.1	0.11	0.25	0	2.12	10.71	0.13
GAIF01013536.1	0	0	0.08	0	3.62	0
GAIF01013537.1	0	0	0	0.71	0	0
GAIF01013538.1	216.73	298.2	208.51	130.1	211.81	373.18
GAIF01013541.1	0	0	0	0	0.23	0
GAIF01013543.1	0	0.07	0.07	0.15	0	0
GAIF01013544.1	0	0.13	0	0.27	0.56	0
GAIF01013545.1	0	0.82	1.83	0	0	2.9
GAIF01013547.1	3.58	6.64	11.62	10.08	5.94	5.5
GAIF01013548.1	0	0	0	0	0.26	0.05
GAIF01013550.1	0	0	0	0.09	0.48	0
GAIF01013551.1	1.47	1.65	1.28	5.58	10.1	0.29
GAIF01013552.1	0	0	0	0	0.23	0.07
GAIF01013553.1	0.13	0	0	0	0	0
GAIF01013554.1	0	0	0	0	0.06	0
GAIF01013556.1	412.65	0	0.69	241.16	288.6	10694.26
GAIF01013557.1	0	0.1	0	0	0	0
GAIF01013558.1	0.04	0.05	0	0.05	0.05	0
GAIF01013559.1	0	4.03	2.66	0	0	0.52
GAIF01013560.1	0.27	0.31	0.49	0.21	0	0.58
GAIF01013561.1	135.23	93.98	101.63	142.88	74.78	89.32
GAIF01013562.1	0	0	0	0.77	3.98	0

GAIF01013563.1	1.71	1.1	1.66	0.47	0.12	0.44
GAIF01013564.1	96.21	178.19	233.33	175.86	351.8	58.18
GAIF01013567.1	0	0	0	0.12	0.24	0
GAIF01013569.1	13.19	19.35	6.01	35.67	57.31	24
GAIF01013570.1	73.91	121.01	153.5	166.41	136.5	26.02
GAIF01013571.1	43.03	89.27	110.36	32.26	40.14	45.02
GAIF01013573.1	0	0	0	0	0.27	0
GAIF01013575.1	8.93	6.89	4.75	19.4	8.66	2.86
GAIF01013577.1	11.29	14	12.23	3.02	1.74	0.16
GAIF01013578.1	0	0	0	0.68	5.2	1.51
GAIF01013579.1	1.56	0.52	0.07	7.65	24.69	0.22
GAIF01013580.1	0	0	0	0.13	0.78	0
GAIF01013582.1	1.04	0.16	1.18	2.5	1.27	0.02
GAIF01013584.1	0.1	0	0	0	0	0
GAIF01013585.1	0	0.23	0	0.77	0.99	0
GAIF01013587.1	0.1	0.04	0	1.32	3.69	0.31
GAIF01013588.1	5.22	0	1.12	12.35	5.91	2.03
GAIF01013589.1	0.42	0	0	0.39	2.53	0.19
GAIF01013590.1	0.61	1.33	0.67	0.71	0.73	0
GAIF01013593.1	0	0	0	0.06	0.74	0
GAIF01013594.1	0.23	0.38	0.88	0.27	0.14	0
GAIF01013595.1	22.12	13.07	23.82	54.27	52.05	23.03
GAIF01013596.1	3.72	2.9	4.19	5.83	4.35	2.82
GAIF01013597.1	0	0	0	0.14	0.5	0
GAIF01013598.1	2061.31	802.65	372.73	299.2	116.34	85.87
GAIF01013599.1	0.21	0.34	0	0	0.11	0
GAIF01013601.1	3.84	4.22	2.38	1.6	1.61	0.09
GAIF01013603.1	0	14.43	0	6.61	6.76	4.11
GAIF01013604.1	0	0.18	0	0	0	0.36
GAIF01013605.1	0.16	0.04	0	0.73	3.02	0.09
GAIF01013606.1	0	0	0	0	0.07	0
GAIF01013607.1	56.67	69.75	107.47	92.18	54.94	40
GAIF01013608.1	0.3	0.11	0	0.99	3.57	0.06
GAIF01013609.1	0.23	0	0	0.89	3.54	0.15
GAIF01013610.1	0.04	0	0	0	0.1	0
GAIF01013611.1	0	0.04	0	0	0.16	0
GAIF01013613.1	21.03	34.98	34.97	41.1	21.67	9.41
GAIF01013614.1	0	0	0	0	0.09	0
GAIF01013615.1	14.93	23.06	23.03	34.76	14.52	6.6
GAIF01013616.1	0.5	0.38	0.36	6.48	32.29	0.7
GAIF01013618.1	0	0	0.05	0	0	0
GAIF01013619.1	0.63	0	0	0.37	0.79	0
GAIF01013621.1	19.95	14.46	1.64	41.74	91.55	11.07
GAIF01013623.1	8.08	4.48	8.97	26.02	26.78	14.02

GAIF01013624.1	0.04	0	0	0	0	0
GAIF01013627.1	0.69	0.94	0.45	8.45	7.54	0
GAIF01013631.1	3.11	2.25	3.27	0.9	0.08	0.14
GAIF01013632.1	0.13	0	0	0	0	0
GAIF01013633.1	0.26	0	0.3	0.1	0.1	0.09
GAIF01013634.1	17.39	29.73	30.02	51.82	24.25	12.06
GAIF01013635.1	0.1	0.01	0.01	0.19	0.33	0.1
GAIF01013639.1	0.17	0.09	0	3.44	10.31	0.1
GAIF01013640.1	0	0	0	0	0.94	0.29
GAIF01013642.1	0.39	0.08	0	1.26	5.27	0.33
GAIF01013643.1	0.01	0	0.01	0.15	0.27	0.07
GAIF01013644.1	0.48	1.49	6.35	0.84	0.36	3.79
GAIF01013645.1	0.53	0.23	0	3.67	13.89	0
GAIF01013646.1	1.03	0.67	0.05	6.1	20.55	0.47
GAIF01013647.1	9.23	4.95	13.13	7.32	0	3.99
GAIF01013648.1	0	0.09	1.06	0	0	0
GAIF01013652.1	0	0	0	1.34	8.25	0
GAIF01013654.1	8.15	7.87	1.43	3.25	6.27	0.5
GAIF01013655.1	0	0.14	0	0.08	0	0.14
GAIF01013656.1	35.27	51.27	67.4	35.32	21.36	26.92
GAIF01013657.1	5.32	5.44	3.43	14.15	21.62	1.38
GAIF01013658.1	0	0	0	0	0.55	0
GAIF01013659.1	4.75	5.3	1.4	6.29	6.3	0.58
GAIF01013662.1	0.46	0.25	0	0	1.92	0
GAIF01013664.1	10.57	5.26	7.85	13.58	8.2	9.11
GAIF01013665.1	0.41	0.48	0.1	2.44	6.6	0.46
GAIF01013667.1	15.19	15.06	3.86	3.96	3.73	0
GAIF01013669.1	0	0.12	0.56	0	0	0.06
GAIF01013670.1	27.11	18.11	17.87	47.17	38.13	31.47
GAIF01013671.1	14.73	16.79	11.09	19.44	27.14	15.19
GAIF01013672.1	19.43	7.1	5.04	57.54	60.29	7.64
GAIF01013673.1	1.73	0.13	0.53	0.56	0	0.4
GAIF01013675.1	0.29	0.05	0.36	1.13	2.91	0.1
GAIF01013676.1	11.81	4.44	6.25	13.82	10.38	9.43
GAIF01013677.1	19.53	15.55	7.91	25.41	42.45	90.5
GAIF01013678.1	0.15	0.08	0	0	0	0.08
GAIF01013679.1	0	0	0.07	0.21	0.36	0
GAIF01013681.1	0.08	0	0	0.36	0.53	0.14
GAIF01013682.1	0	0	0	0	4.29	0
GAIF01013683.1	0.05	0	0.06	0.06	0.56	0
GAIF01013684.1	0	0	0	0.11	0.27	0
GAIF01013685.1	0	0	0	0.24	0.55	0
GAIF01013686.1	0.02	0.01	0.04	0.08	0.08	0.07
GAIF01013687.1	0	0	0	0.16	1.99	0.3

GAIF01013688.1	0.03	0.01	0	0.02	0.03	0
GAIF01013691.1	5.56	3.75	2.39	6.91	10.69	6.64
GAIF01013693.1	0	0	0	3.21	9.66	1.76
GAIF01013694.1	0.14	0	0	0.56	0.33	0.08
GAIF01013695.1	0	0	0	0	0.68	11.38
GAIF01013696.1	0.45	0.12	0	3.27	0	0
GAIF01013697.1	0	0	0	0	0.29	0.07
GAIF01013698.1	0.05	0.06	0	0.69	3.73	0.12
GAIF01013699.1	12.69	18.91	8.19	5.86	4.38	0.51
GAIF01013700.1	0	0.13	0	0.13	0	0
GAIF01013701.1	0	0	0	0.43	3.4	0
GAIF01013702.1	9.71	17.03	9.11	32.72	32.87	2.28
GAIF01013704.1	0	0	0.03	0	0	0
GAIF01013705.1	0.1	0.15	0	0.12	0.16	0
GAIF01013707.1	1.76	1.33	0.27	0.23	0.59	0
GAIF01013709.1	9.3	3.59	2.9	10.32	13.51	7.25
GAIF01013711.1	0.59	0.16	0	3.43	10.56	0
GAIF01013712.1	10.62	9.25	16.42	18.77	13.98	13.51
GAIF01013713.1	14.7	15.36	12.14	46.31	65.18	6.16
GAIF01013714.1	4.72	0.83	0.09	0	1.47	4.66
GAIF01013716.1	0.12	0.7	0	0	0.14	0
GAIF01013718.1	2.76	3.57	1.05	30.16	54.21	0.82
GAIF01013719.1	0.37	0	0	0.32	0.77	0.1
GAIF01013720.1	12.07	20.47	17.25	41.12	36.03	7.15
GAIF01013722.1	17.95	10.29	5.95	34.24	11.41	11.19
GAIF01013723.1	0	0	0	0.22	1.86	0
GAIF01013724.1	0.29	0	0	0.17	0.17	0
GAIF01013727.1	0	0.05	0.05	0.48	1.81	0
GAIF01013728.1	51.13	38.25	46.39	3.27	0.56	0.56
GAIF01013730.1	0.24	0	0	0.04	0.11	0.1
GAIF01013731.1	26.58	12.42	6.2	27.82	44.4	2.97
GAIF01013734.1	0	0.04	0	0.14	0.24	0
GAIF01013735.1	2.33	1.81	1.46	0.96	1.4	0.43
GAIF01013736.1	0.69	0.15	0	3.05	11.23	0.84
GAIF01013737.1	2.86	8.2	2.69	0	0	0.88
GAIF01013738.1	172.16	134.42	76.53	235	205.57	105.6
GAIF01013739.1	0.08	0	0	0.68	2.6	1.28
GAIF01013741.1	0	0	0	2.1	6.02	0.12
GAIF01013742.1	1.98	0.76	0.64	1.39	1.26	0.9
GAIF01013743.1	1.24	0	0	1.1	11.91	0
GAIF01013744.1	7.38	7.05	14.49	4.67	2.42	6.9
GAIF01013745.1	22.67	10.47	0.79	5.19	2.05	0
GAIF01013746.1	34.67	37.99	22.16	35.51	22.26	3.05
GAIF01013747.1	375.09	105	158.44	285.51	80.51	698.5

GAIF01013750.1	246.26	126.35	84.17	261.33	162.65	29.78
GAIF01013751.1	0	0	0.04	0.05	0	0
GAIF01013752.1	496.18	333.41	388.44	714.9	278.47	145.98
GAIF01013753.1	8.82	6.4	2.42	53.28	78.31	2.27
GAIF01013755.1	0	0.26	0.26	0	0.04	0.26
GAIF01013758.1	32.16	39.51	53.48	68.29	49.48	71.41
GAIF01013761.1	0	0.1	0	0.11	0.11	0
GAIF01013763.1	0.08	0	0	0	0	0
GAIF01013764.1	0	0	0	0.1	0.06	0
GAIF01013765.1	139.63	180.06	128.03	165.37	57.46	103.2
GAIF01013766.1	9.82	16.01	10.68	29.91	36.56	3.02
GAIF01013767.1	54.35	51.89	34.97	37.2	30.25	11.98
GAIF01013769.1	1.19	0.8	0.41	2	4.35	0.05
GAIF01013770.1	0.95	1.76	0.3	5.53	16.53	0.04
GAIF01013771.1	2.74	0.73	0.77	1.42	3.13	0.56
GAIF01013772.1	0	1.92	5.52	0.1	0.21	1.75
GAIF01013773.1	89.48	48.58	73.21	289.94	571.55	805.56
GAIF01013775.1	42.07	126.5	230.54	296.6	255.6	208.6
GAIF01013777.1	1.15	0.56	0.13	4.02	9.29	0.06
GAIF01013778.1	0	0	0	0	0	0.34
GAIF01013779.1	10.24	2.16	2.31	3.52	2.11	2.7
GAIF01013780.1	7.53	43.02	48.23	15.75	20.11	14.3
GAIF01013784.1	0	0.47	0.81	0.52	0.7	0.62
GAIF01013785.1	5.7	5.49	4.06	1.09	0	0
GAIF01013786.1	66.06	27.02	30.83	84.25	104.28	42.07
GAIF01013787.1	0	0.36	0.95	0	0	0.07
GAIF01013788.1	0.44	0.39	0	0.54	2.37	0.81
GAIF01013789.1	61.2	50.66	45.7	102.7	57.69	23.24
GAIF01013790.1	0.63	0.23	1.15	0.24	0.25	1.16
GAIF01013791.1	0.29	0.16	0.16	0.85	2.46	0.16
GAIF01013793.1	0.08	0	0	0.15	0.13	0.03
GAIF01013795.1	0	0	0	0.59	0	0
GAIF01013796.1	0.08	0	0	0.08	0.74	0.03
GAIF01013798.1	0.66	0	0.41	1.53	5.08	0.21
GAIF01013800.1	0	0	0	0.12	0.44	0.06
GAIF01013801.1	8.78	3.06	0.96	21.25	57.24	8.42
GAIF01013802.1	1.29	4.59	1.04	1.2	6.63	0.38
GAIF01013804.1	0	0	0.58	2.64	3.87	0
GAIF01013805.1	0	0	0	0	0.97	0
GAIF01013809.1	33.76	52.59	14.33	17.1	16.18	1.53
GAIF01013810.1	6.2	7.05	9.12	18.97	19.74	33.53
GAIF01013812.1	0	0	0	0	0	0.07
GAIF01013813.1	0	0	0	0.05	0	0
GAIF01013814.1	2.08	1.75	2	2.21	4.38	5.83

GAIF01013815.1	0.12	0.13	0.13	0.55	0.43	0
GAIF01013816.1	0	0.12	0	0	0.07	0
GAIF01013817.1	19.52	13.96	13.98	86.02	133.78	14.49
GAIF01013818.1	0.16	0.17	0.07	0.18	1.98	0.1
GAIF01013819.1	26.94	37.26	22.26	93.18	106.37	5.98
GAIF01013821.1	0.42	0.23	0	0.25	0.13	0.12
GAIF01013823.1	1.13	0.56	0.26	1.2	1.19	0.1
GAIF01013824.1	0	0.03	0	0.59	3.22	0.03
GAIF01013825.1	12.04	11.28	6.45	28.29	25.23	3.53
GAIF01013827.1	0.89	0.55	0.21	8.58	75.73	0.51
GAIF01013828.1	109.4	69.05	77.78	56.4	19.98	52.67
GAIF01013829.1	0.08	0	0.16	0.46	3.54	0.16
GAIF01013831.1	0	0.29	0.76	0	0.06	0
GAIF01013832.1	0	0	0	0	0.48	0
GAIF01013833.1	26.58	18.93	14.11	39.59	51.89	55.33
GAIF01013834.1	51.94	74.29	48.67	83.37	44.86	16.77
GAIF01013835.1	0.09	0	0	0	0	0
GAIF01013837.1	0	0	0.23	0	0	0
GAIF01013839.1	16.56	9.35	9.13	14.3	7.39	8.34
GAIF01013844.1	0.22	0	0	0.51	2.65	0
GAIF01013846.1	0.03	0	0.08	0.6	2.64	0.04
GAIF01013847.1	1.13	0.33	0.2	5.02	18.41	0.77
GAIF01013848.1	4.05	5.91	1.52	18.79	36.76	1.06
GAIF01013850.1	0.69	0	0.64	3.51	7.75	41.19
GAIF01013852.1	87.89	50	89.32	197.27	144.42	227.65
GAIF01013853.1	0	3.15	0	4.23	11.72	1.45
GAIF01013855.1	0.95	0	0	2.18	2.92	0
GAIF01013856.1	8.72	19.38	8.8	32.97	34.48	3.7
GAIF01013858.1	0.1	0.05	0	0	0.54	0
GAIF01013859.1	0.8	0.65	0	1.16	3.51	0
GAIF01013861.1	0.14	0	0	0.17	0	0
GAIF01013862.1	1.35	1.77	1.71	2.83	2.23	0.45
GAIF01013863.1	4.15	2.13	2.93	10.04	11.1	12.34
GAIF01013864.1	18.21	16.08	23.76	45.89	31.96	7.32
GAIF01013865.1	0.2	0.32	0.32	0	0.12	0
GAIF01013867.1	0	0.17	0	0	0.49	0
GAIF01013869.1	0	0	0	0	0	0.09
GAIF01013871.1	5.38	8.38	7.51	1.01	0.17	0
GAIF01013872.1	0.96	1.96	1.98	2.52	0.87	1.86
GAIF01013874.1	0	0	0	0.23	0.48	0.22
GAIF01013875.1	599.91	530.86	374.32	467.48	597.27	1197.67
GAIF01013879.1	9.96	19.96	16.47	44.17	15.08	7.16
GAIF01013880.1	46.24	24.84	9.97	52.25	41.72	5.51
GAIF01013882.1	2.72	3.59	1.14	1.94	4.62	0.91

GAIF01013883.1	0	0	0	0	0.09	0
GAIF01013884.1	0.22	0.15	0	0.39	0	0.09
GAIF01013885.1	0	1.98	1.33	0	0	0.13
GAIF01013886.1	0.62	0.1	0	3.53	13.99	1.18
GAIF01013887.1	0.33	0.05	0	0.16	0.23	0
GAIF01013888.1	0.69	0.8	0.54	10.34	1.07	1.57
GAIF01013890.1	1.11	0.15	0.11	0.31	0.33	0.59
GAIF01013891.1	17.54	15.73	34.02	41.48	37.72	21.4
GAIF01013893.1	4.17	3.21	0.67	26.69	46.98	23.77
GAIF01013894.1	0.01	0	0	0	0	0
GAIF01013895.1	0.2	1.46	0	2.81	11.1	0.8
GAIF01013896.1	20.08	34.94	39.83	14.9	7.21	7.11
GAIF01013897.1	0.83	0.43	0.31	5.17	17.1	0.34
GAIF01013899.1	0.06	0	0	0.15	0	0
GAIF01013900.1	35.96	17.53	15.43	92.5	124.67	20.04
GAIF01013901.1	0.1	0.04	0	0.11	0.2	0
GAIF01013902.1	0	0	0	0	0.03	0
GAIF01013903.1	28.91	35.99	35.88	24.6	17.78	20.92
GAIF01013904.1	10.94	4.49	0	40.81	98.81	27.62
GAIF01013906.1	8.45	5.73	3.8	40.75	17.06	4.42
GAIF01013907.1	0.88	0.03	1.82	2.69	5.36	1.15
GAIF01013908.1	0	0	0	0.41	0.49	0
GAIF01013909.1	146.17	156.6	212.55	128.9	93.5	124.66
GAIF01013910.1	0.06	0	0.04	0	0.08	0
GAIF01013911.1	17.36	9.51	8.49	19.19	31.66	22.12
GAIF01013912.1	0.09	0	0.94	0	0	0.09
GAIF01013913.1	0.23	0	0	0.14	0	0
GAIF01013914.1	0.31	0	0	0	0.05	0.08
GAIF01013915.1	0.18	0	0.17	0	0	0
GAIF01013918.1	0.1	0.22	0.11	0.23	0.06	0.17
GAIF01013921.1	0.22	0.08	0	1.08	5.45	0.16
GAIF01013922.1	2.2	0.12	0	2.94	12.57	5.46
GAIF01013925.1	1.22	0.59	0.89	4.07	1.46	0.6
GAIF01013926.1	0	0	0.31	0.22	0.11	0
GAIF01013927.1	222.35	120.63	118.35	123.1	42.57	26.78
GAIF01013928.1	2.63	5.22	6.15	4.51	3.58	1.51
GAIF01013930.1	1.47	2.58	2.47	0.14	1.32	0.28
GAIF01013931.1	0.11	0	0	0.53	0.27	0.12
GAIF01013932.1	0	0	0.71	0.37	0	0.09
GAIF01013933.1	7.61	7.47	2.41	17.9	22.54	0
GAIF01013934.1	10.22	13.16	47.46	24.95	15.4	33.01
GAIF01013935.1	11.17	9	5.73	1.9	11.65	5.26
GAIF01013936.1	17.32	26.65	21.65	24.05	17.22	6.94
GAIF01013937.1	25.53	30.37	12.22	57.65	60	18.09

GAIF01013938.1	0	0.7	0	1.75	7.2	0.66
GAIF01013939.1	0.04	0.08	0.08	0.31	2.14	0.04
GAIF01013941.1	0.27	0.14	0.73	0	0.16	0.07
GAIF01013942.1	12.84	19.19	7.14	39.12	38.7	2.04
GAIF01013943.1	0.19	0.06	0.06	0.34	0.86	0.08
GAIF01013944.1	44.56	38.55	12.97	42.35	48.46	3.55
GAIF01013945.1	9.92	10.68	7.62	30.85	17.41	6.82
GAIF01013946.1	0.35	0.16	0.05	1.27	8.48	0.66
GAIF01013947.1	0.16	0	0.18	0	0.19	0
GAIF01013949.1	27.73	23.33	49.28	30.73	18.97	41.27
GAIF01013950.1	205.84	171.91	75.95	55.45	45.94	60.69
GAIF01013952.1	0	0	0.13	0	0	0.39
GAIF01013953.1	4.65	1.54	2.15	5.09	3.41	4.53
GAIF01013955.1	1.21	0.1	0.1	1.08	2.13	0.41
GAIF01013957.1	0.7	0.12	0.69	1.51	1.57	0.13
GAIF01013958.1	25.23	46.09	11.85	45.37	93.21	4.76
GAIF01013960.1	25.9	12.16	4.72	67.36	52.82	8.6
GAIF01013963.1	0.86	0.29	0.94	0	0	0
GAIF01013964.1	104.08	34.28	21.8	118.86	150.79	35.45
GAIF01013965.1	0	0	0	0.23	0.35	0
GAIF01013966.1	0.77	1.32	1.13	0.6	0.62	1.98
GAIF01013967.1	0.47	0.2	0.41	0.54	3.14	0
GAIF01013968.1	0.72	0.33	0	4.47	21.35	0.13
GAIF01013969.1	217.76	112.57	36.77	210.71	560.76	104.41
GAIF01013970.1	0.36	0	0	0.28	4.35	0.53
GAIF01013971.1	0	0	0	0	5.53	0
GAIF01013973.1	0.31	0.43	0.36	0.75	1.11	0.09
GAIF01013974.1	0.13	0.28	0	0	0	0
GAIF01013975.1	1.48	0	0	5.92	15.68	1.27
GAIF01013976.1	0.03	0.29	0.1	0.42	0.33	0.19
GAIF01013977.1	0.16	0	0	0.49	2.04	0
GAIF01013978.1	0.26	0	0	0	0	0.5
GAIF01013979.1	0.3	0	0	2.07	6.99	0.49
GAIF01013980.1	5.31	3.26	1.34	21.29	18.1	0
GAIF01013981.1	0.92	0.5	1.1	0.54	2.68	0.51
GAIF01013982.1	0	0	0	0.08	0	0.08
GAIF01013984.1	0	0.15	0	0	0	0
GAIF01013987.1	0.49	0.53	0	0	0.19	0
GAIF01013988.1	0	0	0	0	0	0.22
GAIF01013990.1	0	0.47	0	0	0.25	0
GAIF01013993.1	147.34	92.94	40.39	60.59	93.52	28.71
GAIF01013995.1	5.4	9.43	5.15	17.09	8.14	2.24
GAIF01013997.1	45.05	42.76	32.82	107.58	48.29	18.65
GAIF01013998.1	0	0.05	0.21	1.48	1.54	0.14

GAIF01013999.1	44.08	51.71	29.42	88.93	51.88	13.14
GAIF01014000.1	0.06	0.14	0.07	0.33	0.3	0
GAIF01014002.1	0	0	0	0	2.48	0
GAIF01014003.1	0	0	0.07	0	0	0
GAIF01014006.1	13.11	14.26	43.19	0	0	0
GAIF01014009.1	3.02	4.64	3.16	2.9	2.18	0.34
GAIF01014011.1	0	0	0	0	0.12	0
GAIF01014013.1	62.39	40.54	19.4	185.46	410.66	64.34
GAIF01014014.1	0.15	0	0	0	0	0
GAIF01014015.1	0	0	0	0.35	0.19	0
GAIF01014016.1	0	0	0.4	0	2.06	0
GAIF01014018.1	45.29	28.95	25.44	108.51	124.04	27.49
GAIF01014020.1	0	0	0	0	0.52	0
GAIF01014021.1	23.29	27.33	38.66	39.58	13.45	14.87
GAIF01014022.1	0	0	0	2.32	8.79	0.31
GAIF01014023.1	0.12	0	0.51	0.13	0	0
GAIF01014024.1	0	0	0.1	0.61	2.26	0.1
GAIF01014025.1	10.2	6.9	4.58	21.41	13.44	7.72
GAIF01014026.1	0.2	0.06	0.4	0.28	0.15	0
GAIF01014027.1	0.34	0.37	0.38	0.4	2.27	1.32
GAIF01014028.1	0.88	0	0	2.21	8.65	12.29
GAIF01014029.1	4.82	12.88	8.91	3.48	2.11	0.08
GAIF01014030.1	16.78	19.1	27.73	24.05	11.87	20.12
GAIF01014031.1	0.05	0	0.29	2.52	6.44	0.12
GAIF01014033.1	0	0.09	0	0	0	0
GAIF01014034.1	77.52	47.98	47.86	87.72	56.04	44.49
GAIF01014035.1	0.14	0	0	1.43	6.73	0
GAIF01014036.1	0.19	0.03	0.04	0.04	0.39	0.28
GAIF01014037.1	0.11	0	0.12	0.34	0.53	0
GAIF01014038.1	0	0	0	0	0.72	0
GAIF01014039.1	28.88	30.66	15.71	96.9	78.94	9.36
GAIF01014041.1	0.24	0.92	0.93	1.68	4.65	1.06
GAIF01014042.1	20.66	21.74	12.69	21.82	15.26	3.95
GAIF01014043.1	0.35	0	0	1.79	0.38	0
GAIF01014045.1	23.73	22.31	5.85	108.63	136.99	8.39
GAIF01014046.1	1.23	0.56	0.19	2.26	6.11	0
GAIF01014048.1	28.74	18.02	16.63	49.27	68.36	30.91
GAIF01014049.1	0	14.05	57.3	0.36	0.75	42.14
GAIF01014051.1	1.19	1.18	1.67	1.81	0.22	1.33
GAIF01014052.1	0	0	0	0.13	0	0
GAIF01014053.1	9.36	11.51	4.92	19.84	35.74	1.5
GAIF01014055.1	1.93	1.4	2.21	5.74	9.66	2.75
GAIF01014057.1	9.93	21.64	9.96	25.46	39.96	0.26
GAIF01014058.1	0.04	0.05	0	0.3	0.99	0

GAIF01014059.1	4.08	5.14	0.6	4.44	1.56	0
GAIF01014060.1	0	0	0	0	0.26	0
GAIF01014061.1	6.48	4.16	6.3	4.81	5.24	2.81
GAIF01014063.1	4.71	0	0	9.67	23.06	23.13
GAIF01014064.1	0	0.33	0	1.1	2.79	0.11
GAIF01014066.1	94.04	96.06	45.67	171.73	149.78	11.57
GAIF01014067.1	6.45	8.47	5.62	25.28	25.99	2.01
GAIF01014070.1	0	0.28	0	1.73	7.05	0.51
GAIF01014072.1	0	0	0	0	0.88	0
GAIF01014073.1	0.06	0.24	0.06	0.06	0	0.06
GAIF01014074.1	5.01	2.99	0.3	49.07	136.36	7.54
GAIF01014075.1	3717.09	1869.71	1745.82	2207	2797.26	8166.96
GAIF01014078.1	0.52	0.45	0.26	0.12	0	0.04
GAIF01014079.1	82.33	65.26	53.62	7.88	0.39	0.01
GAIF01014080.1	0.8	0.72	0.08	4.67	8.97	0.24
GAIF01014081.1	6	10.64	3.52	1.69	1.61	0.19
GAIF01014082.1	13.28	15.57	20.14	54.53	48.95	12.61
GAIF01014085.1	50.39	31.44	3.32	4.33	21.77	3.13
GAIF01014086.1	2.47	0.28	0.14	0	0.16	0
GAIF01014091.1	1.16	1.5	0.08	9.45	14.51	0.08
GAIF01014092.1	37.95	45.6	28.5	133.33	49.86	15.09
GAIF01014093.1	0.33	0.47	0.48	1.01	3.13	2.27
GAIF01014094.1	7.33	14.02	4.69	0.94	3.6	0
GAIF01014095.1	0	0	0	0	0.37	0
GAIF01014096.1	13.54	7.58	14.25	12.78	5.51	11.92
GAIF01014098.1	0	0	0	0	0	0.04
GAIF01014100.1	0.07	0.21	0	0.94	2.9	0
GAIF01014101.1	0.1	0	0	0	0.6	0
GAIF01014102.1	4.31	6.5	5.21	14.02	2.59	1.93
GAIF01014103.1	108.43	44.95	65.34	7.34	9.05	6.68
GAIF01014105.1	0	0.12	0.37	0	0	0
GAIF01014107.1	0	0	0.21	0	0	0
GAIF01014108.1	38	29.03	37.48	18.47	14.22	16.84
GAIF01014109.1	137.71	130.53	116.93	89.53	51.02	20.43
GAIF01014111.1	0.63	0.89	0.5	0.47	0.05	0.1
GAIF01014112.1	1.79	1.7	1.33	3.82	5.46	8.76
GAIF01014114.1	1.5	0.38	0.87	0.82	0.43	0.49
GAIF01014115.1	31.24	17.82	18	56.24	69.76	42.95
GAIF01014116.1	0.18	0.58	0	0	0.21	0
GAIF01014118.1	0.84	2.75	0.46	2.94	3.25	0.14
GAIF01014119.1	0.09	0.17	0	0	0	0.62
GAIF01014120.1	913.64	1075.29	847.84	581.66	418.88	327.23
GAIF01014121.1	1.1	0.27	0.18	4.3	18.3	0.86
GAIF01014122.1	0	0	0.21	0	0	0

GAIF01014124.1	0.22	0	0	0	1.06	0
GAIF01014125.1	0	0	0.07	5.67	17.74	0.75
GAIF01014126.1	5.46	4.04	6.05	17.43	70.33	2.65
GAIF01014129.1	22.01	17.44	4.39	24.87	32.39	1.66
GAIF01014130.1	0.13	0	0	0.15	0.47	0
GAIF01014131.1	0.09	0.15	0.05	0	0	0
GAIF01014132.1	0	0	0	0	0.18	0
GAIF01014133.1	28.98	24.44	12.66	10.13	5.67	1.12
GAIF01014136.1	0	0.14	0.57	0.3	0.62	0.14
GAIF01014138.1	0	0.23	0.08	0	0	0.24
GAIF01014139.1	19.29	19.78	5.9	79.37	79.3	10.23
GAIF01014140.1	8.91	16.98	43.3	46.87	53.81	21.7
GAIF01014141.1	229.65	78.06	137.92	181.01	247.56	115.78
GAIF01014142.1	0.33	0.82	0.56	0.99	1.18	0.25
GAIF01014143.1	15.33	12.21	10.96	35.65	61.58	5.02
GAIF01014144.1	9.34	9.36	11.64	19.59	24.31	7.15
GAIF01014145.1	38.58	12.05	19.92	27.73	17.18	17.25
GAIF01014146.1	0	0	0	1.15	0	0.08
GAIF01014147.1	0	0	0	0.65	0	0
GAIF01014148.1	67.4	63.3	96.75	72.85	48.03	85.7
GAIF01014150.1	6.73	6.83	6.77	1.72	1.51	2.26
GAIF01014151.1	0.54	0.92	0.79	0.49	1.59	0.07
GAIF01014152.1	0.1	0	0	1.05	3.34	0.3
GAIF01014154.1	21.68	4.53	0	14.7	22.64	23.28
GAIF01014157.1	0	0	0.12	0	0	0
GAIF01014159.1	0	0	0	0	0	0.03
GAIF01014161.1	15.99	18.93	13.78	8.01	6.21	1.45
GAIF01014162.1	0.19	0.21	0	0.67	4.61	0
GAIF01014163.1	5.71	4.89	5.41	0.44	0.49	0.53
GAIF01014165.1	0	0	0	0.13	0.26	0
GAIF01014167.1	0	0	0	0	0.07	0
GAIF01014170.1	0	0	0	0.27	0	0
GAIF01014171.1	0	0	0.03	0	0	0
GAIF01014172.1	6.62	7.71	2.75	4	3.32	0.23
GAIF01014173.1	7.01	10.43	9.11	56.8	56.96	4.73
GAIF01014174.1	0.08	0	0	0.05	0.59	0.09
GAIF01014175.1	1.48	1.29	1.01	1.56	4.19	0.68
GAIF01014176.1	0.06	0	0	0	0	0
GAIF01014178.1	6119.26	7330.94	7852.09	2264.08	1832.33	1929.72
GAIF01014179.1	0	0	0	0.15	0	0
GAIF01014180.1	0.15	0	0	0.42	2.28	0.08
GAIF01014181.1	1.57	3.19	4.51	2.42	1.57	0.29
GAIF01014183.1	0	0	0	0	0.09	0
GAIF01014184.1	0.43	0.2	0.07	0.21	0.96	0

GAIF01014185.1	1240.27	714.19	377.71	82	23.11	111.78
GAIF01014186.1	0.22	0.72	0.17	0.33	0.2	0.16
GAIF01014187.1	5.91	7.16	3.24	1.09	0.16	0
GAIF01014189.1	0.42	0.37	0	2.01	6.47	0.07
GAIF01014192.1	2.1	3.7	0.66	3.6	9.16	2.32
GAIF01014193.1	0.06	0	0	0.14	0	0
GAIF01014194.1	3.86	7.33	2.89	17.55	14.43	0.82
GAIF01014195.1	0.52	0.13	0	0.51	0	0
GAIF01014196.1	3.72	1.55	0.48	8.2	12	5.53
GAIF01014197.1	0	0	0	0	0.04	0
GAIF01014198.1	0.97	0.87	2.22	1.22	3.04	1.58
GAIF01014199.1	0.72	0	0	0	3.1	0
GAIF01014200.1	11.82	13.47	6.61	6.99	3.83	0.88
GAIF01014201.1	71.32	58.47	37.34	34.32	49.76	8.42
GAIF01014202.1	5.04	3.48	4.66	9.46	16	5.55
GAIF01014204.1	0.16	0	0	0	0	0
GAIF01014205.1	2.17	19.22	0	40.92	20.85	1.98
GAIF01014207.1	0	0	0	0.23	0.1	0
GAIF01014208.1	7.06	2.14	1.47	5.4	3.48	1.32
GAIF01014209.1	9.29	7.44	2.55	40.13	36	0.85
GAIF01014210.1	2.78	0	0.87	2.53	5.99	0
GAIF01014212.1	7.64	4.42	7.74	15.6	4.97	8.72
GAIF01014214.1	11.92	18.96	7.62	69.49	57.78	3.53
GAIF01014215.1	0.28	0.08	0.23	0.16	0	0.08
GAIF01014216.1	20.66	36.93	8.95	62.11	41.8	1.92
GAIF01014217.1	33.67	26.78	26.76	21.86	12.53	18.95
GAIF01014218.1	61.41	57.89	10.86	203.76	200	5.95
GAIF01014219.1	0.06	0	0	0	0	0
GAIF01014221.1	37.52	42.39	33.2	60.12	62.16	14.8
GAIF01014223.1	0	0	0	0.04	0	0
GAIF01014224.1	64.72	95.21	90.37	167.15	66.47	29.37
GAIF01014226.1	16.78	5.88	9.32	25.21	40.04	28.4
GAIF01014228.1	51.98	34.3	24.46	100.39	74.75	15.17
GAIF01014230.1	0	0.07	0.13	0	0.74	0
GAIF01014231.1	36.04	54.64	28.78	122.12	63.5	10.92
GAIF01014232.1	0.09	0	0	0	0	0
GAIF01014234.1	6.01	5.53	5.08	2.01	1.11	0.89
GAIF01014236.1	2.15	2.34	2.36	8.03	9.42	0.8
GAIF01014238.1	0	0.09	0.11	0.02	0.02	0
GAIF01014240.1	0	0	0.12	0	0	0
GAIF01014241.1	0.72	0	0.19	2.14	2.6	1.52
GAIF01014242.1	0	0.13	0.2	0	0	0
GAIF01014243.1	1.72	0.76	0.31	3.46	10.44	0.56
GAIF01014244.1	96.95	21.76	51.86	23.96	0	13.58

GAIF01014245.1	0.19	0	0	0.28	0	0
GAIF01014246.1	0	0	0	0.15	0.15	0
GAIF01014247.1	0	0.68	0	0	0	0
GAIF01014248.1	0	0	0	0	0.26	0
GAIF01014249.1	6.3	12.91	10.57	22.82	9.79	2.83
GAIF01014250.1	0.1	0.21	0.07	0.04	0.78	0.07
GAIF01014253.1	2.29	4.18	0.2	15.92	24.86	0
GAIF01014255.1	4.94	2.38	3.36	10.89	12.63	9.25
GAIF01014257.1	0.17	0.19	0	0.6	1.85	0.28
GAIF01014258.1	0.67	0.12	0.25	0.91	0.13	0.25
GAIF01014261.1	0	0	0	0.13	0.91	0.12
GAIF01014262.1	0	0	0	0	0.48	0
GAIF01014263.1	9.28	0	2.8	11.75	5.16	8.08
GAIF01014264.1	1.13	1.06	1.22	1.23	1.49	0.21
GAIF01014265.1	0.4	0	0	1.19	2.94	1.38
GAIF01014266.1	0	0	0	0.38	0.4	0
GAIF01014267.1	0	0	0	0.37	0.13	0
GAIF01014270.1	0	0.13	0.06	0	0	0.07
GAIF01014272.1	0	0	0	0	0.22	0
GAIF01014274.1	0	0	0	1.18	5.43	0.1
GAIF01014278.1	0	0	0	0.1	0.21	0
GAIF01014280.1	0.24	0	0.05	0.61	4.89	0.78
GAIF01014282.1	0	0	0	0	0.15	0
GAIF01014283.1	0	0.76	0.57	0.2	0	0.19
GAIF01014284.1	42.95	95.51	55.14	171.54	144.11	26.38
GAIF01014285.1	24.62	13.79	4.64	32.69	37.73	11.88
GAIF01014286.1	0.15	0	0.08	0.43	1.32	0.16
GAIF01014288.1	2.75	2.31	1.24	3.34	2.71	1.52
GAIF01014290.1	26.68	18.9	3.33	33.1	23.21	41.86
GAIF01014291.1	0	0	0	0.19	0.38	0
GAIF01014293.1	0	0.28	0	0.43	1.89	0
GAIF01014294.1	0.74	0.72	0.27	0	0.3	0
GAIF01014295.1	1	0.54	1.23	0.14	0.15	0
GAIF01014297.1	8.25	6.69	4.43	34.08	28.61	5.06
GAIF01014298.1	0.87	0.32	0.26	0.98	2.28	0.03
GAIF01014299.1	0	0	0.24	0	0	0
GAIF01014300.1	0	0	0	0.13	0.83	0
GAIF01014301.1	0	0.78	0.14	0	0	0.09
GAIF01014302.1	0.14	0	0	0	0.67	0
GAIF01014303.1	0.3	0.17	0	0	0	0
GAIF01014304.1	0	0	0	0	0.16	0
GAIF01014306.1	9.64	0	3.67	3.73	5.71	12.43
GAIF01014309.1	6.07	7.8	7.61	20.17	14.82	2.44
GAIF01014313.1	9.2	9.87	5.79	8.26	1.18	0.27

GAIF01014314.1	9.31	5.73	4.44	2.56	3.48	0
GAIF01014315.1	1.46	3.82	4.45	2.62	2.21	0.32
GAIF01014317.1	10.87	9.85	13.33	1.89	0.87	0.8
GAIF01014318.1	1.89	2.39	0.54	0.35	0.73	0
GAIF01014319.1	12.49	25.8	20.76	50.77	43.02	3.67
GAIF01014320.1	0.4	7.19	0	2.06	5.11	5.8
GAIF01014322.1	0.45	0.1	0	0.63	3.28	0.1
GAIF01014323.1	0.94	0.33	0.18	6.99	23.44	1.33
GAIF01014326.1	19.25	26.91	25.04	45.33	35.62	10.82
GAIF01014327.1	0.33	0.09	0.82	0.48	0	0
GAIF01014330.1	0	0	0	0	0	0.6
GAIF01014332.1	3.22	5.76	5.19	7.06	6.03	1.26
GAIF01014333.1	0.07	0	0	0	0	0
GAIF01014334.1	0	0	0	0.19	0.86	0
GAIF01014335.1	0.04	0.04	0	0.13	0	0
GAIF01014336.1	14.78	11.41	9.72	43.07	34.42	19.13
GAIF01014337.1	0	0	0	0	0.03	0
GAIF01014338.1	0.1	0.33	0.22	0.12	0	0.11
GAIF01014339.1	0.73	2.07	0.4	8.07	12.33	0.29
GAIF01014340.1	9.52	4.89	0	19.9	0	0
GAIF01014341.1	0	0	0	0	0.14	0
GAIF01014342.1	0	0	0	0.56	2.41	0
GAIF01014343.1	0	0.06	0	0	0	0
GAIF01014344.1	0	0.04	0	0.08	0.24	0
GAIF01014346.1	3.47	28.33	7.38	57.42	44.69	2.26
GAIF01014347.1	0	3.26	7.13	0	5.13	1.22
GAIF01014348.1	11.03	8.98	14.4	35.19	31.51	9.82
GAIF01014349.1	1.46	1.32	1.25	1.78	0.93	1.12
GAIF01014350.1	3.08	2.48	1.31	2.36	3.04	0.55
GAIF01014353.1	0.33	0.12	0	2.25	12.88	0.9
GAIF01014354.1	27.77	14.95	19.74	27.75	12.32	12.38
GAIF01014356.1	10.3	9.68	11	17.34	19.74	5.42
GAIF01014357.1	0	0	0	0.23	0.41	0
GAIF01014358.1	10.53	7.35	3.86	20.61	18.81	2.12
GAIF01014360.1	0.7	0.38	0.86	0.95	1.46	1.23
GAIF01014362.1	12.35	3.9	1.92	9.43	35.06	48.72
GAIF01014363.1	43.46	47.76	19.91	171.63	62.23	17.92
GAIF01014364.1	723.66	467.32	273.19	428.4	253.58	85.17
GAIF01014365.1	12.09	8.24	6.26	14.37	23.97	3.56
GAIF01014369.1	0	0	0	0	0.72	0
GAIF01014371.1	0	0	0	0.13	0.26	0
GAIF01014372.1	38.6	12.49	7.71	32.98	57.7	36.19
GAIF01014374.1	0	0	0	0.72	1.97	0.54
GAIF01014375.1	0.38	0.33	0.41	0.26	0.27	0.17

GAIF01014376.1	0	0	0.41	1.95	2.85	0
GAIF01014377.1	0.22	0	0	1.65	3.02	0
GAIF01014378.1	0	0	0	0.12	0.51	0
GAIF01014379.1	0	0.14	0	0.15	0.08	0
GAIF01014380.1	25.94	51.05	63.75	56.94	47.05	7.33
GAIF01014381.1	0.51	0.77	0	0.79	0	0
GAIF01014384.1	8.48	3.87	4.75	9.59	4.69	8.41
GAIF01014385.1	0	0	0	0	0.17	0
GAIF01014386.1	0.15	0.27	0	0.23	1.14	0
GAIF01014388.1	37.71	45.62	32.61	15.57	9.75	5.24
GAIF01014390.1	0	0	0	0	0.03	0
GAIF01014391.1	0.05	0.05	0	0	0	0
GAIF01014392.1	0	0	0	0	0	0.1
GAIF01014393.1	1.77	12.51	3.06	4.7	14.83	0.62
GAIF01014394.1	0	0	0	0.2	1.06	0
GAIF01014395.1	62.26	60.38	34.84	124.03	53.43	19.51
GAIF01014396.1	9.54	14.49	7.7	3.74	3.27	0.97
GAIF01014397.1	0	0	0	0.08	0	0
GAIF01014398.1	0	0.06	0	0.13	0.47	0
GAIF01014399.1	0.34	0	0	0.15	0.32	0.05
GAIF01014400.1	0.22	0.31	0	1.5	2.24	0.08
GAIF01014401.1	0.42	0	0.08	0.16	0	0.08
GAIF01014402.1	0	0.09	0	0	0.09	0
GAIF01014403.1	0	0.16	0	0.17	1.45	0
GAIF01014404.1	0	0	0.12	0	0	0
GAIF01014406.1	28.22	41.93	8.09	137.13	154.99	6.57
GAIF01014407.1	0.48	0.13	0.13	1.67	0.87	0.26
GAIF01014408.1	0	0.13	0	0.14	0	0.13
GAIF01014409.1	0	0	0	0	0.45	0
GAIF01014410.1	1.05	0.59	0.15	1.99	8.36	0.49
GAIF01014411.1	0	0.41	0.69	0.15	0	0.55
GAIF01014412.1	1.69	1.61	0.52	0.94	2.26	0.62
GAIF01014413.1	31.52	29.56	50.54	51.46	23.04	32.01
GAIF01014415.1	27.33	33.78	37.1	121.26	90.46	24.31
GAIF01014416.1	0	0	0	0	0.16	0
GAIF01014418.1	0.09	0	0	0.05	0.16	0
GAIF01014420.1	0	0	0	0.26	0.41	0
GAIF01014421.1	76.36	75.86	68.68	70.61	37.52	12.44
GAIF01014422.1	0.04	0.19	0	2.51	6.6	0.06
GAIF01014423.1	2.95	2.27	0.48	7.36	23.2	1.25
GAIF01014424.1	4.65	1.22	0.93	5.88	7.62	22.81
GAIF01014425.1	372.52	258.9	220.6	4.26	4.89	5.36
GAIF01014426.1	0.14	0	0	0	0.53	0
GAIF01014428.1	0	0	0	0.02	0.02	0

GAIF01014430.1	4.94	3.66	7.17	5.6	5.76	5.43
GAIF01014432.1	27.19	14.96	14.98	61.96	59.57	65.87
GAIF01014434.1	34.37	55.29	30.14	36.18	13.4	33.71
GAIF01014435.1	4.32	1.68	2.76	5.17	4.74	1.49
GAIF01014436.1	2290.2	1769.25	3496.18	911.74	502.95	1710.11
GAIF01014437.1	0	0	0.12	0	0	0
GAIF01014438.1	0.06	0	0	0	0	0
GAIF01014439.1	3.69	2.5	2.78	6.3	18.04	21.89
GAIF01014440.1	10.5	12.63	19.67	27.29	18.47	9.71
GAIF01014441.1	1.62	0.2	0.4	4.61	12.38	1.19
GAIF01014442.1	3.19	1.67	1.56	9.87	27.88	2.56
GAIF01014444.1	1962.47	498.89	221.54	1882.52	2784.81	3166.53
GAIF01014447.1	5.28	7.66	4.3	6.22	5.87	2.03
GAIF01014448.1	0.05	0.1	0.05	0	0.17	0.05
GAIF01014449.1	10.3	15.19	3.04	4.48	16.56	1.75
GAIF01014450.1	0.1	0	0	0	0.04	0
GAIF01014451.1	0.17	0.12	0	0	0.48	0
GAIF01014453.1	0.86	0.93	1.15	0.76	1.1	0.58
GAIF01014455.1	0	0	14.4	0	15.77	0
GAIF01014457.1	0	0	0	0.75	0	0
GAIF01014458.1	0	0	0	0.26	3.69	0.25
GAIF01014459.1	0.21	0	0.23	2.4	6.22	0
GAIF01014460.1	0	0.23	0.06	0.04	1.5	0
GAIF01014461.1	5.28	8.84	0.16	16.4	56.64	1.26
GAIF01014462.1	0.11	0.08	0.12	0	0.09	0.04
GAIF01014463.1	3.31	2.15	5.06	0.67	0.09	1.86
GAIF01014464.1	4.39	2.24	4.49	12.36	8.15	19.4
GAIF01014465.1	0.07	0	0	0.25	0.7	0
GAIF01014467.1	4.1	2.3	0.17	2.08	2.59	0.09
GAIF01014470.1	1.96	1.03	0.37	2.91	11.32	0.3
GAIF01014473.1	15.98	12.65	13.29	27.76	33.36	3.43
GAIF01014475.1	0	0.4	0.34	0	0	0.07
GAIF01014476.1	14.1	6.82	11.77	21.39	27.78	12.53
GAIF01014478.1	0.08	0	0	3.38	10.92	0
GAIF01014479.1	0.31	0.61	0	0.52	0.53	0.43
GAIF01014480.1	0.19	0	0	0.66	3.21	0
GAIF01014481.1	3.69	2.87	1.74	5.2	0.95	2.32
GAIF01014482.1	0	0	0	0	0.36	0
GAIF01014485.1	1.73	0.21	0.11	0.22	4.63	0.74
GAIF01014486.1	0.52	0.57	0.19	0.3	0.11	0
GAIF01014488.1	0	0	0.84	0.73	6.72	16.55
GAIF01014491.1	17.73	12.72	10.98	3	2.65	1.76
GAIF01014492.1	0	0	0	0	0.18	0
GAIF01014496.1	0	0	0.04	0	0	0

GAIF01014497.1	0.9	0	0.25	1.02	1.39	0.15
GAIF01014501.1	0.12	0	0.2	0	1.11	0
GAIF01014502.1	0	0	0.01	26.33	0	6.67
GAIF01014503.1	0.14	0.15	0	1.94	8.71	0.61
GAIF01014504.1	10.36	5.96	0.51	20.69	30.09	5.38
GAIF01014505.1	2.25	0.89	0.06	0.39	0	0.11
GAIF01014506.1	1.59	1.09	2.3	7.25	4.66	6.77
GAIF01014507.1	0.54	0.21	0.26	0.09	0.93	0.09
GAIF01014508.1	5.34	10.78	5.44	21.54	9.17	1.96
GAIF01014509.1	0	0	0	0.11	0	0
GAIF01014510.1	0.66	0.3	0	0.41	1.8	2.7
GAIF01014511.1	0.61	0	0	4.22	21.23	0
GAIF01014513.1	1.37	0.82	1.02	1.54	1.01	0.19
GAIF01014514.1	0	0	0	0	0.1	0
GAIF01014515.1	3.55	3.99	1.54	22.09	0	1.06
GAIF01014516.1	0.16	0.09	0	0.28	0.97	0.18
GAIF01014518.1	0.1	0	0.22	0.12	0	0
GAIF01014519.1	0	0.46	0.1	0.91	0.18	0.11
GAIF01014520.1	11.23	7.24	5.35	14.11	8.56	1.37
GAIF01014521.1	8.77	14.56	10.15	25.72	13.97	4.96
GAIF01014522.1	4.57	5.56	3.81	12.44	12.8	0.35
GAIF01014523.1	0.11	0.06	0.06	0	0.2	0
GAIF01014526.1	10.49	7.64	9.15	40.28	35.91	10
GAIF01014527.1	0	0.15	0	0.16	0.97	0.15
GAIF01014528.1	6	1.38	0.4	5.49	11.37	18.03
GAIF01014529.1	19.07	19.54	15.32	40.42	32.35	114.05
GAIF01014530.1	2.51	2.81	0.8	7.07	8.12	1.39
GAIF01014531.1	0.12	0	0	0.48	2.02	0
GAIF01014532.1	5.32	13.37	7.55	25.18	18.86	1.83
GAIF01014533.1	417.89	462.74	400.78	878.02	803.7	595.08
GAIF01014535.1	0	0	0	0	0.17	0
GAIF01014536.1	4.46	14.31	8.54	6.3	8.16	1.7
GAIF01014537.1	0.51	0.66	0.39	2.59	13.41	0.11
GAIF01014538.1	84.85	97.05	75.91	245.85	125.99	38.49
GAIF01014539.1	1.92	1.37	1.21	9.29	19	2.17
GAIF01014540.1	0	0	0	0	0.12	0
GAIF01014541.1	29.79	36.9	43.71	38.33	23.4	9.31
GAIF01014542.1	0	0	0	0.06	0	0
GAIF01014543.1	4.46	5.54	2.38	0.59	0.46	0.14
GAIF01014544.1	0.06	0	0.06	0	0.1	0
GAIF01014545.1	0	0.46	0.53	0	0	0
GAIF01014547.1	18.04	18.15	29.27	27.5	13.76	19.44
GAIF01014551.1	62.68	193.3	84.68	35.59	59.95	13.99
GAIF01014552.1	0.38	0.57	0.35	1.45	5.18	0.48

GAIF01014553.1	6.33	3.06	6.75	2.04	1.48	6.2
GAIF01014554.1	0	0.05	0	0	0	0
GAIF01014556.1	0	0.01	0.09	0.2	0.58	0
GAIF01014557.1	0.37	0.89	0.2	0.42	0.55	0
GAIF01014558.1	49.77	81.03	151.88	60.9	54.5	52.11
GAIF01014559.1	47.95	20.4	13.81	93.31	64.96	31.61
GAIF01014560.1	0.29	0	0	0.72	3.05	0.43
GAIF01014561.1	0.17	0	0	0	0	0
GAIF01014563.1	1.58	0.22	0.64	3.81	25.13	3.52
GAIF01014564.1	0	0.06	0	0	0.07	0
GAIF01014566.1	1591.22	2010.59	2007.56	1029.82	802.95	654.98
GAIF01014567.1	7.48	10.59	4.97	35.54	34.76	1.92
GAIF01014568.1	0	0.83	0.18	0	0	0
GAIF01014570.1	0	0	0.28	0	0.27	0
GAIF01014573.1	10.23	13.24	20.81	13.33	12.3	5.82
GAIF01014574.1	0.03	0.04	0.07	0.15	0.12	0
GAIF01014576.1	0.44	0.14	0.07	3.53	13.57	0.84
GAIF01014579.1	9.58	2.38	0.25	21.17	65.68	15.04
GAIF01014580.1	151.56	81.25	50.52	125.94	138.24	40.36
GAIF01014581.1	19.58	21.69	14.23	59.22	36.07	14.71
GAIF01014583.1	2.21	2.04	1.74	8.99	22.99	2.34
GAIF01014584.1	0	0	0	0	0.32	0
GAIF01014585.1	0.27	0.15	0.1	1.7	6.54	0
GAIF01014586.1	0.83	0.23	0	2.89	16.34	0.08
GAIF01014587.1	10.4	10.19	28.12	9.38	4.86	10.66
GAIF01014591.1	3.61	2.47	4.79	0.49	0.61	0.93
GAIF01014593.1	3.94	9.19	0.62	28.01	50.37	1.55
GAIF01014594.1	0	0.41	0	0.86	0	0.25
GAIF01014596.1	0.49	0.86	0.54	3.09	4.15	1.2
GAIF01014597.1	0	0	0	0.69	0.7	0
GAIF01014599.1	0	0	0.05	0	0	0
GAIF01014600.1	0.61	0.38	0	0.27	0	0.06
GAIF01014602.1	0.12	0.38	0	0.14	0.42	0
GAIF01014605.1	3.2	2.65	0.23	19.91	45.4	0.07
GAIF01014606.1	11.31	8.9	1.5	24.79	39.18	2.19
GAIF01014608.1	0	0	0	0.11	0	0
GAIF01014609.1	0.16	0	1.05	0	0	0.01
GAIF01014611.1	20.76	13.57	7.03	0.39	0.44	1.02
GAIF01014613.1	0.3	0.25	0.23	0.04	0	0
GAIF01014614.1	0	0	0	0	0.29	0
GAIF01014616.1	4.34	0.38	0.61	1.38	2.69	1.39
GAIF01014617.1	56.87	41.26	22.46	2.81	2.31	9.82
GAIF01014619.1	70.82	42.58	34.4	100.66	132.53	155.48
GAIF01014620.1	0.33	0.15	0	0.99	8.55	0.52

GAIF01014622.1	0	0	0.06	0	0.07	0
GAIF01014623.1	0	0	0	0	0	0.11
GAIF01014624.1	0	0	0	0.11	0	0
GAIF01014625.1	0	0.24	0.72	0	0	0.16
GAIF01014626.1	0.34	0.51	0.44	2.55	5.75	0.46
GAIF01014628.1	0	0.05	0.05	0.35	1.25	0.38
GAIF01014630.1	0.62	0	0	0.22	4.02	2.66
GAIF01014631.1	0	0	0	0	0.07	0
GAIF01014633.1	0	0.16	0	3.59	6.9	0.16
GAIF01014634.1	1.59	1.05	0.12	15.45	37.8	0.44
GAIF01014635.1	0.44	0	0.16	0.17	0	0
GAIF01014636.1	1.37	1.89	0.53	0.06	0.74	0.04
GAIF01014637.1	6.98	4.03	4.28	19.35	50.08	44.8
GAIF01014638.1	0	0	0.16	0	0	0
GAIF01014639.1	0.14	0.25	0.47	0.18	0.92	0
GAIF01014640.1	1.21	0.44	0	0.19	0.1	0
GAIF01014641.1	1.9	0.56	0.7	6.88	19.49	3.12
GAIF01014643.1	0	0	0.3	0.11	0.07	0.1
GAIF01014644.1	209.05	366.9	230.07	283.56	360.62	48.02
GAIF01014646.1	1.81	1.5	0.09	18.07	72.79	0.28
GAIF01014647.1	7.2	2.24	3.39	5.97	2.47	3.4
GAIF01014648.1	9.8	11.2	10.48	0.58	0.6	1.38
GAIF01014649.1	29.47	21.26	20.99	14.91	22.56	5.9
GAIF01014651.1	0	0	0	0.23	1.09	0
GAIF01014652.1	0.1	0.05	0	0.31	0.43	0.07
GAIF01014653.1	8.6	9.11	9.26	28.8	18.77	6.42
GAIF01014654.1	0	0.81	0	8.77	1.75	0.86
GAIF01014656.1	2.42	3.98	4.08	5.09	5.72	2.98
GAIF01014658.1	2.07	1.12	5.48	0.66	0.24	3.74
GAIF01014660.1	0	0	0	1.15	0	0
GAIF01014662.1	0	0	0	0	0.19	0
GAIF01014663.1	0	0	0	0	0.14	0
GAIF01014665.1	16.9	20.29	37.49	21.21	16.3	33.04
GAIF01014667.1	0.19	0.21	0.16	0.2	2.47	0.08
GAIF01014669.1	19.84	15.82	28.58	18.09	12.52	20.65
GAIF01014670.1	0	0	0	0	0.31	0
GAIF01014671.1	27.07	34.09	30.69	44.14	21.5	8.47
GAIF01014674.1	0.91	0	0	5.8	16.54	0.75
GAIF01014675.1	65.4	74.23	99.04	187.65	73.37	32.74
GAIF01014676.1	0	0	0.05	0.06	0.18	0.55
GAIF01014678.1	75.13	65.73	63.79	99.15	79.68	30.9
GAIF01014679.1	137	174.97	422.28	66.44	67.45	247.26
GAIF01014681.1	1.05	2.44	0	4.89	11.89	0
GAIF01014682.1	12	9.47	5.69	23.63	11.62	5.61

GAIF01014683.1	0.23	0.1	0	0.31	1.02	0.14
GAIF01014684.1	2.8	0.28	0.28	16.38	35.32	2.38
GAIF01014685.1	0	0	0	0.88	3.47	0.3
GAIF01014687.1	0	0	0	0.01	0.01	0.02
GAIF01014688.1	0.19	0.47	0.18	0.09	0.1	0
GAIF01014689.1	14.9	11.34	9.46	34.35	15.68	7.06
GAIF01014690.1	0.8	0.19	0	0.47	1.41	0.27
GAIF01014692.1	15.24	0.35	1.42	8.17	5.26	14.74
GAIF01014693.1	23.83	33.35	25.17	80.89	75.77	12.16
GAIF01014694.1	29.06	18.07	13.33	34.12	15.13	2.26
GAIF01014696.1	7.3	0.81	0.63	9.23	23.81	13.75
GAIF01014698.1	0.04	0	0	0	0	0
GAIF01014699.1	0	0	0	0.18	0.36	0
GAIF01014700.1	0.74	0.66	0.36	1.56	2.18	0.51
GAIF01014701.1	0	0	0	0	0.53	0.16
GAIF01014702.1	0.22	0.19	0.18	0.06	0.04	0
GAIF01014703.1	0	0	0	0.05	0.34	0
GAIF01014704.1	3.73	1.52	7.05	0	0.39	0.48
GAIF01014705.1	0	0	0	0	1.19	0
GAIF01014706.1	0.84	0.33	0	10.76	26.54	0.16
GAIF01014707.1	20.11	12.83	8.35	29.81	23.99	6.94
GAIF01014708.1	0	0	0	0.19	0.39	0
GAIF01014709.1	1.05	0.14	0.49	1.09	0	0
GAIF01014711.1	1.34	0	0	0.84	13.73	2.52
GAIF01014712.1	2.58	1.93	0.27	0.27	0.07	0.08
GAIF01014714.1	2.08	1.77	0	17.22	46.01	0
GAIF01014715.1	0	0	0	0.2	1.27	0.1
GAIF01014716.1	1.25	1.22	0.36	6.03	13.37	0.29
GAIF01014717.1	0	0	0	0	2.32	0
GAIF01014718.1	0.16	0	0.54	0	1.64	0.22
GAIF01014719.1	0.62	0	0	0	0	0
GAIF01014720.1	3.29	5.8	1.83	7.91	7.53	0.51
GAIF01014721.1	2812.35	2865.07	5744.62	1525.34	976.42	3701.76
GAIF01014722.1	6.8	2.58	1.3	12.11	19.85	2.4
GAIF01014723.1	0.11	0	0	0.22	1.6	0
GAIF01014726.1	14.18	8.35	0.64	5.62	14.17	0.09
GAIF01014727.1	24.57	2.26	4.14	19.61	12.04	6.16
GAIF01014728.1	68.85	57.07	4.89	65.16	110.58	25.22
GAIF01014729.1	0	0.18	0	0.29	2.27	0
GAIF01014732.1	6.53	3.57	2.79	24.07	42.88	2.8
GAIF01014733.1	0.17	0	0	4.51	15.33	0.94
GAIF01014734.1	10.92	8.95	4.44	6.08	1.8	0.33
GAIF01014735.1	0	0	0	0.2	0.42	0
GAIF01014736.1	0.04	0.02	0.05	0.02	0.1	0.05

GAIF01014740.1	19.39	10.64	9.13	39.84	61.07	12.49
GAIF01014741.1	0.53	0.19	0.2	1.03	5.57	0.2
GAIF01014742.1	2470.16	898.89	558.9	1420.82	1428.97	2421.6
GAIF01014745.1	6.78	4.58	1.07	19.13	33.36	1.03
GAIF01014747.1	6.32	11.47	8.2	26.93	19.19	2.75
GAIF01014748.1	2.1	0.83	1.05	3.65	1.83	0.21
GAIF01014749.1	1.84	1.92	0.28	1.05	0.41	0.22
GAIF01014750.1	6.34	1.76	5.67	12.67	1.42	5.04
GAIF01014751.1	0.03	0	0.03	0.4	0.55	0
GAIF01014752.1	0.24	0	0	0	0.18	0.11
GAIF01014754.1	126.71	160.99	451.01	156.97	130.88	351.64
GAIF01014755.1	24.26	14.62	7.23	2.17	0.86	2.06
GAIF01014756.1	0.09	0.1	0.1	0.48	3.95	0.05
GAIF01014757.1	19.72	18.94	17.35	35.32	23.92	10.77
GAIF01014758.1	35.74	33.8	25.19	69.46	42.38	17.22
GAIF01014759.1	0.1	0.17	0.08	1.55	9	0.05
GAIF01014760.1	78	101.04	56.29	132.49	264.06	452.78
GAIF01014762.1	5866.49	5231.2	10663.74	3221.97	1621.06	4673.02
GAIF01014763.1	0	0	0	0.09	2.42	0.17
GAIF01014765.1	98.17	69.88	98.18	109.46	48.57	56.7
GAIF01014766.1	0.09	0.1	0	0	0	0
GAIF01014767.1	40.27	45.49	35.43	54.97	30.99	4.97
GAIF01014768.1	13.64	27.95	21.39	60.56	38.85	8.09
GAIF01014769.1	0	0	0	0	0.88	0.22
GAIF01014771.1	1.2	1.31	0.94	2.46	0.55	0
GAIF01014772.1	0	0	0	0.09	0.49	0.08
GAIF01014773.1	9.31	8.47	4.75	0	0	0
GAIF01014774.1	0	0.14	0	0	0	0
GAIF01014777.1	1.24	0.23	0.12	3.21	9.85	6.41
GAIF01014778.1	0	0.23	0	0.73	2.01	0.23
GAIF01014779.1	14.88	13.12	12.56	12.54	7.68	2.78
GAIF01014780.1	0.16	0.06	0	0	0	0.06
GAIF01014781.1	0	18.74	0	0	3.57	0
GAIF01014782.1	209.14	38.85	42.35	152.07	140.27	138.54
GAIF01014783.1	0.08	0.17	0.35	0.09	0	0.09
GAIF01014785.1	0	0	0	0	0.28	0
GAIF01014786.1	33.95	27.45	50.18	18.43	11.18	24.31
GAIF01014787.1	3.51	5.77	6.48	24.72	39.28	3.46
GAIF01014788.1	0.04	0	0.02	0.66	1.11	0.06
GAIF01014789.1	0	0	0	0	0.08	0
GAIF01014790.1	0.07	0	0	0.82	5.94	0.31
GAIF01014793.1	0	0	0	0.07	0.46	0
GAIF01014794.1	0.06	0	0	0.53	3.15	0.06
GAIF01014796.1	8.73	14.48	4.55	13.02	21.78	2.77

GAIF01014798.1	22.26	11.72	12.49	63.63	93.39	69.72
GAIF01014799.1	0	0	0	0.24	1.35	0
GAIF01014800.1	1.41	0.28	0.43	2.94	0.81	0.73
GAIF01014801.1	2.77	1.24	0.09	8.69	20.06	2.96
GAIF01014802.1	0	0	0	0.13	0	0
GAIF01014803.1	0.66	0.7	0	0.93	0.97	0
GAIF01014804.1	9.37	5.23	3.79	16.99	11.29	3.37
GAIF01014805.1	0	0	0	0	0	0.11
GAIF01014807.1	0	0.1	0	0.05	1.28	0
GAIF01014808.1	0.14	0	0	0	0.39	0.18
GAIF01014810.1	1.82	0.63	0.18	11	31.54	2.16
GAIF01014811.1	3.9	2.45	8.57	5.49	5.03	8.16
GAIF01014813.1	905.72	1066.83	759.46	578.36	782.74	346.74
GAIF01014814.1	0	0	0	0	0.08	0
GAIF01014815.1	1.74	0.47	0.85	4.92	8.02	0.5
GAIF01014816.1	0.06	0.33	0.07	0.21	0.14	0.13
GAIF01014817.1	0	0.21	0	0	0	0
GAIF01014818.1	0	0	0	0	0.23	0
GAIF01014819.1	52.45	63.36	23.03	28.41	51.86	8.99
GAIF01014820.1	0	0	0	0.14	0.76	0
GAIF01014821.1	0	0	0	0.1	0	0
GAIF01014823.1	0	0	0.61	0	0	0.37
GAIF01014824.1	0	0	0	0	0.07	0
GAIF01014825.1	0	0.87	1.02	0	0	0.44
GAIF01014826.1	0	0	0	0	0.42	0
GAIF01014827.1	0.14	0.08	0	1.11	3.2	0.15
GAIF01014828.1	33.21	18.47	5.11	9.12	9.84	1.51
GAIF01014830.1	0	0.07	0	0.08	0.18	0
GAIF01014832.1	0	0	0	0.03	0	0.01
GAIF01014833.1	36.08	13.13	18.03	86.6	107.49	174.08
GAIF01014834.1	0	0	0	0	0.16	0
GAIF01014837.1	0.16	0	0.03	0.3	0.14	0
GAIF01014838.1	26.89	16.33	0	10.92	53.81	11.55
GAIF01014839.1	10.94	8.29	5.95	19.28	20.8	5.06
GAIF01014840.1	13.48	13.15	8.1	38.41	19.28	4.5
GAIF01014841.1	0.08	0	0.18	0.2	0.89	0
GAIF01014842.1	0.56	0.09	0.07	0.62	0.77	0
GAIF01014843.1	11.3	8.1	13.71	40.76	56.76	15.14
GAIF01014844.1	1.34	1.99	0.32	6.57	25.91	0.35
GAIF01014845.1	0.53	1.55	0.48	10.63	5.6	2.24
GAIF01014848.1	62.6	49.89	69.66	28.31	17.23	10.9
GAIF01014849.1	0.22	0.45	0.11	0	0	0
GAIF01014850.1	0.54	0.7	0.27	0.85	0.5	0
GAIF01014851.1	7.04	4.56	4.01	23.18	31.84	5.86

GAIF01014852.1	0.13	0.09	0.03	0.59	2.33	0.12
GAIF01014853.1	8.9	23.95	22.13	30.59	18.69	5.45
GAIF01014855.1	0.19	0.07	0.28	1.33	2.75	0
GAIF01014856.1	160.32	10.63	11.25	351.06	884.27	4184.95
GAIF01014857.1	0.46	0	0	0.01	0.15	0.01
GAIF01014858.1	9.41	13.31	5.46	7.37	5.75	1.84
GAIF01014859.1	0.14	0	0.15	0.95	6.87	0
GAIF01014860.1	0.03	0	0	0	0	0
GAIF01014861.1	0	0	0.06	0	0	0
GAIF01014862.1	0	0	0	0.63	0.33	0
GAIF01014863.1	3152.61	2096.11	1260.72	1292.77	668.48	5317.05
GAIF01014865.1	0	0	0	0	0.3	0
GAIF01014866.1	8.15	7.04	7.55	16.98	26.65	17.77
GAIF01014867.1	0.2	0	0.03	1.2	5.05	0.29
GAIF01014868.1	1.13	0.23	0.2	6.74	25.41	0.7
GAIF01014869.1	3.31	2.44	1.04	4.39	3.56	0.39
GAIF01014870.1	0.05	0.06	0.06	0.02	0.06	0
GAIF01014871.1	0.46	0.11	0	5.93	25.94	0
GAIF01014872.1	0.03	0.04	0	0	0	0
GAIF01014873.1	0	0.61	0	0	0.03	0.24
GAIF01014874.1	0.25	0.34	0.07	2.05	7.21	1.25
GAIF01014875.1	0.05	0.97	0.23	4.35	8.75	0.06
GAIF01014876.1	73.77	92.74	68.67	93.09	72.7	30.84
GAIF01014877.1	7.56	9.08	12.17	15.46	13.85	3.97
GAIF01014878.1	144.81	16.33	31.51	5.18	0	0
GAIF01014879.1	4	13.47	6.68	0	0.01	51.24
GAIF01014881.1	32.43	47.29	35.86	8.82	29.47	2.26
GAIF01014882.1	0	0	0	0.38	1.18	0
GAIF01014884.1	0	0	0	0	0.14	0
GAIF01014885.1	0.3	1.96	0.18	0.01	2.17	0
GAIF01014886.1	0	0	0.18	0.52	3.48	0.24
GAIF01014887.1	38.14	39.12	23.64	61.5	93.28	77.79
GAIF01014888.1	7.92	16.95	9.29	9.51	18.66	1.74
GAIF01014889.1	12.91	21.94	20.36	38.9	21	5.5
GAIF01014890.1	0	0	0	0.98	4.51	0
GAIF01014894.1	8.39	3.43	3.92	11.18	9.39	12.45
GAIF01014897.1	29.9	13.7	9.31	15.5	5.05	2.13
GAIF01014898.1	0	0	0	0.31	0	0
GAIF01014901.1	176.78	111.86	47.43	187.56	164.56	113.87
GAIF01014902.1	72.11	132.01	60.9	128.32	108.06	15.21
GAIF01014903.1	0.33	0.12	0.03	0.1	1.49	0
GAIF01014904.1	0	0.12	0.36	0	0	0.48
GAIF01014905.1	9.84	0.92	0.46	5.5	7.62	7.43
GAIF01014906.1	4.72	4.18	2.32	0.72	0.6	0.14

GAIF01014907.1	22.12	37.28	34.41	62.2	51.78	15.36
GAIF01014908.1	0	0	0.48	0	0	0
GAIF01014909.1	20.9	21.3	24.61	26.1	18.19	4.69
GAIF01014910.1	0	0.05	0	0	0	0
GAIF01014913.1	1.84	6.32	0.63	6.74	12.46	0
GAIF01014915.1	0.04	0	0.05	0.15	0.4	0
GAIF01014918.1	0	0	0.3	0	0	0
GAIF01014919.1	2.12	8.34	1.47	5.37	5.14	0.4
GAIF01014920.1	1.19	0.97	0.52	1.04	1.72	0.07
GAIF01014922.1	0	0.29	0.29	0.46	0.16	0.14
GAIF01014923.1	0	0	0.27	0	0.15	0
GAIF01014924.1	0	0.68	0.34	0.54	3.36	2.23
GAIF01014926.1	0	0	0	0	0.12	0
GAIF01014928.1	0	0.06	0	0.42	1.08	0
GAIF01014930.1	0.2	0.51	0	1.03	1.08	0
GAIF01014931.1	26.99	40.6	10.89	53.73	58.37	0
GAIF01014932.1	31.93	27.82	28.7	58.09	38.39	56.64
GAIF01014933.1	4.62	1.27	0.2	3.98	12.39	1.87
GAIF01014934.1	0.15	0.08	0	0.26	3.32	0.05
GAIF01014935.1	0.38	0.48	0.25	1.07	4.87	0.13
GAIF01014939.1	0	0	0	0	2.03	0
GAIF01014940.1	0	0.31	0.15	0.65	2.62	0
GAIF01014941.1	0	0.49	0.26	0.39	0.06	0
GAIF01014942.1	0	0	0	0.62	0.86	0
GAIF01014943.1	0	0	0	0	0.13	0
GAIF01014944.1	0.49	0	0	1.69	9.23	1.87
GAIF01014945.1	10.34	6.99	6.4	20.76	14.03	12.85
GAIF01014947.1	0	0.01	0.06	0.04	0.12	0.02
GAIF01014948.1	10.3	2.19	8.66	12.29	8	3.4
GAIF01014949.1	1.14	1.41	0.54	2.26	0.78	0.45
GAIF01014951.1	0	0	0	2.62	1.59	0
GAIF01014952.1	1.62	0.88	3.26	3.13	5.19	2.67
GAIF01014953.1	10.48	10.57	7.6	11.69	18.46	29.21
GAIF01014954.1	0.61	0.17	0.25	2.83	2.57	1.68
GAIF01014958.1	34.49	42.72	7.4	167.19	256.19	2
GAIF01014960.1	6.25	5.73	2.38	3.75	3.25	0
GAIF01014961.1	16.48	0	0.45	0	0.5	5.06
GAIF01014962.1	0.08	0.11	0.02	2.79	13.49	0.18
GAIF01014963.1	0	0	0	0.73	7.82	0
GAIF01014964.1	0.23	0	0	0.45	0.22	0.25
GAIF01014966.1	0.5	0	0.18	5.5	19.81	10.82
GAIF01014969.1	30.91	20.83	17.36	8	3.17	0.78
GAIF01014970.1	13.45	10.48	6.87	4.85	2.94	0.48
GAIF01014971.1	0	0	0	0	0.14	0.13

GAIF01014972.1	129.5	44.77	53.95	77.73	86.03	144.15
GAIF01014974.1	0	1.21	0	0	0	0
GAIF01014976.1	96.04	116.9	195.82	80.18	62.08	221.94
GAIF01014980.1	0.14	0	0	0.25	1.71	0
GAIF01014981.1	0	0	0	0.1	0	0
GAIF01014983.1	4.46	3.05	6.02	19.38	19.62	6.88
GAIF01014984.1	95.67	94.43	114.39	174.35	118.88	232.66
GAIF01014985.1	0	0	0	0.01	0	0
GAIF01014986.1	0.33	0	0	0	0.4	0
GAIF01014987.1	6.66	4.91	6.07	5.5	3.94	4.11
GAIF01014988.1	0.41	0.21	0	0.87	0.71	0
GAIF01014989.1	0.62	1.09	0.42	0.91	2.54	0
GAIF01014990.1	18.34	23.15	25.17	80.59	105.89	14.08
GAIF01014991.1	0	0	0	0.5	4.89	0.1
GAIF01014992.1	0.11	0	0	0	0	0
GAIF01014993.1	2.34	0.17	0.06	1.1	2.41	0.99
GAIF01014994.1	0	0.11	0	0	0.35	0
GAIF01014995.1	0.1	0	0.46	0.12	0.5	0
GAIF01014996.1	0	0	0	0.07	0	0.07
GAIF01014997.1	1.03	1.05	0.64	2.77	6.27	0.14
GAIF01014998.1	39.51	39.72	70.69	75.27	44.57	41.14
GAIF01014999.1	7.34	12.57	6.64	23.91	15.29	2.32
GAIF01015000.1	14.51	11.11	7.32	26.59	4.25	4.31
GAIF01015003.1	0	0	0	0.12	0	0
GAIF01015004.1	1.37	2.13	0.75	3.67	1.64	0.85
GAIF01015007.1	0.42	0.14	0.09	1.56	10.35	0.21
GAIF01015008.1	0.85	1.12	0.96	0.09	0.25	0.25
GAIF01015009.1	106.58	127.15	271.09	132.23	93.64	159.6
GAIF01015010.1	0	0	0	0.16	2.17	0
GAIF01015012.1	6.55	8.9	10.39	10.35	9.74	2.61
GAIF01015013.1	1.95	1.19	2.14	8.39	4.97	6.23
GAIF01015014.1	0.49	0.34	0.1	0.94	0.54	0
GAIF01015015.1	4.99	53.45	26.32	27.2	0.03	149.65
GAIF01015016.1	0.93	0.56	0	0.77	0.38	0.06
GAIF01015017.1	0	0	0	0	0.51	0
GAIF01015018.1	0.16	0.04	0	0.09	1.37	0
GAIF01015019.1	0.17	0	0	0.07	0.2	0
GAIF01015021.1	0.03	0.07	0.1	0.04	0.08	0
GAIF01015022.1	10.03	5.25	2.31	0.14	0.6	0.96
GAIF01015023.1	0	0	0	0	0.15	0
GAIF01015024.1	0.12	0.47	0.04	0.27	0.57	0
GAIF01015026.1	0.65	2.13	0.72	3.98	2.94	1.26
GAIF01015028.1	0	0	0	0	0.06	0
GAIF01015029.1	24.63	21.64	17.36	13.84	14.83	10.56

GAIF01015030.1	0.28	0.08	0	1.21	5.02	0.23
GAIF01015031.1	3.08	3.17	1.45	5.48	6.62	0.66
GAIF01015032.1	0.77	0	0	0.85	1.94	0
GAIF01015033.1	33.42	39.46	33.54	69.9	33.29	20.79
GAIF01015035.1	0.39	0.77	0.19	1.86	2.02	0.45
GAIF01015037.1	0.08	0.05	0	1.28	7.5	0.05
GAIF01015040.1	0.53	0.48	0.25	4.73	4.92	0
GAIF01015041.1	0.11	0.97	0.25	0.13	1.75	0
GAIF01015045.1	12.06	14.3	7.3	26.8	24.06	2.19
GAIF01015046.1	18.19	19.67	14.01	24.38	14.85	6.96
GAIF01015047.1	0	0.31	0	0.33	0.95	0
GAIF01015048.1	0.23	0.08	0.04	0.62	3.76	0.04
GAIF01015050.1	556.42	449.46	190.82	174.07	250.06	55.95
GAIF01015052.1	0.12	0	0	0	0	0.04
GAIF01015053.1	10.81	9.75	15.08	19.68	15.41	22.49
GAIF01015054.1	491.68	127.83	187.32	242.91	231.94	1321.64
GAIF01015056.1	0.59	0	0	1.88	4.44	0
GAIF01015057.1	5.63	1.99	1.72	6.27	12.94	12.96
GAIF01015059.1	0.5	1.26	0	0.92	1.33	0
GAIF01015061.1	0	0	0	0.1	0	0
GAIF01015062.1	0	0.09	0.09	0.09	0.29	0.17
GAIF01015065.1	0	0	0	0	0	0.01
GAIF01015066.1	0	0	0.05	0	0	0
GAIF01015067.1	0.09	0	0	0	0.22	0
GAIF01015068.1	5.5	6.07	5.61	18.35	25.44	5.79
GAIF01015069.1	8.23	6.65	6.16	23.45	11.7	5.36
GAIF01015070.1	23.36	12.98	9.71	67.68	25.72	9.82
GAIF01015075.1	2.78	0.66	0.46	10.1	56.57	5.33
GAIF01015076.1	2.26	0.46	0	3.28	15.11	1.09
GAIF01015078.1	14.35	12.28	11.64	28.78	31.43	8.45
GAIF01015080.1	10.99	4.55	4.96	35.93	17.35	0.11
GAIF01015081.1	0.64	1.6	1.01	2.03	3.59	0.1
GAIF01015082.1	30.83	35.84	43.2	74.89	53.78	17.45
GAIF01015083.1	0	0	0.14	0	0	0
GAIF01015084.1	4.8	9.75	5.62	11.45	6.97	0.95
GAIF01015086.1	0	0	0	0	0.1	0
GAIF01015087.1	2.26	0	0	5.56	20.8	14.92
GAIF01015089.1	20.78	12.82	21.75	33.29	28.65	44.07
GAIF01015090.1	0	0	0	4.66	0	2.43
GAIF01015092.1	228.19	96.91	81.48	214.33	88.37	72.88
GAIF01015094.1	1.04	0.14	0.21	3.38	23.69	0.29
GAIF01015095.1	0.75	0.07	0.34	2.6	11.81	4.59
GAIF01015096.1	0	0	0	0	0.06	0
GAIF01015097.1	2.91	2.11	5.33	3.38	3.5	1.07

GAIF01015099.1	4.05	2.59	0.49	23.72	40.81	0.27
GAIF01015100.1	3.68	2.5	1.52	12.81	19.81	0.51
GAIF01015101.1	0.1	0.09	0	0.23	1.3	0
GAIF01015102.1	10.01	7.73	9.63	27.21	34.82	10.29
GAIF01015103.1	0	0	0	0.24	0.47	0
GAIF01015104.1	1.3	0.71	0.39	5.35	5.05	1.37
GAIF01015105.1	0	0	0	0.14	0.29	0.26
GAIF01015106.1	0.92	1.25	1.77	0.8	0	1.02
GAIF01015107.1	0.12	0.25	0	0.67	3.7	0
GAIF01015108.1	0	0	0	1.65	2.93	0
GAIF01015110.1	53.3	34.21	31.31	24.35	17.17	8.44
GAIF01015114.1	0.46	0.51	0	0	0	0.16
GAIF01015116.1	0.06	0.07	0	0.04	0.08	0
GAIF01015117.1	0	0.02	0	0	0.02	0
GAIF01015118.1	8.91	15.26	8.7	10.59	13.16	4.41
GAIF01015119.1	0	0	0.47	0	0	0
GAIF01015121.1	0	0	0	0.31	1.21	0
GAIF01015122.1	0.78	0.24	0.12	9.52	24.34	0.25
GAIF01015124.1	0.5	0.14	0	0.43	1.19	0.23
GAIF01015126.1	0.54	1.33	2.31	4.56	1.14	1.05
GAIF01015128.1	12.81	23.4	22.56	45.72	42.86	6.39
GAIF01015129.1	68.86	39.87	27.78	103.27	95.07	94.12
GAIF01015130.1	0.02	0	0	0	0.01	0
GAIF01015131.1	0.31	0.43	0	0.72	0.34	0
GAIF01015132.1	0	0	0	0.05	0	0
GAIF01015133.1	0.3	0.11	0	1.51	2.17	0
GAIF01015134.1	0.57	0.1	0	2.99	2.75	0.11
GAIF01015135.1	0	0	0	0.03	0	0
GAIF01015136.1	9.64	0.51	2.4	37.2	223.29	146.69
GAIF01015137.1	5.29	1.23	0.06	40.72	93.26	10.08
GAIF01015139.1	0	0.04	0	0.63	0	0
GAIF01015140.1	64.97	110.84	62.56	6.6	2.32	4.6
GAIF01015142.1	0.34	0	0	2.38	10	0.38
GAIF01015143.1	0.26	0	0.12	0	0.09	0
GAIF01015146.1	4.66	4.32	2.98	2.52	0.99	1.18
GAIF01015148.1	0.15	0	0	0.82	6.75	0.22
GAIF01015149.1	15.03	13.59	14.76	21.67	12.18	8.72
GAIF01015151.1	0.06	0	0	0.07	0.08	0
GAIF01015152.1	10.92	3.24	0.26	2.39	6.21	4.73
GAIF01015153.1	17.01	10.29	10.17	29.54	49.19	30.34
GAIF01015155.1	27.9	15.3	28.75	60.29	107.11	219.84
GAIF01015157.1	0	0	0.18	0	0	0
GAIF01015158.1	0	0.25	0	0	0.31	0
GAIF01015159.1	0.7	0.48	0.39	1.83	0.74	0.58

GAIF01015160.1	0	0	0.09	0	0	0
GAIF01015161.1	3.12	5.46	0	0.59	3.3	0
GAIF01015162.1	17.29	28.46	6.71	49.53	31.3	1.64
GAIF01015163.1	9.12	8.99	8.51	37.7	47.03	6.85
GAIF01015164.1	1.11	0.75	0.43	1.01	1.68	1.57
GAIF01015166.1	0.78	0.34	0.8	0.48	0.31	0.46
GAIF01015167.1	31.17	28.71	11.31	3.94	5.47	2.17
GAIF01015168.1	0	0.05	0	0	0.62	0.03
GAIF01015171.1	0	0	0	0	0.19	0
GAIF01015172.1	15.1	14.53	19.64	21.57	9.36	14.08
GAIF01015174.1	14.52	5.06	10.96	16.36	6.02	16.77
GAIF01015175.1	0.33	0.37	0.5	0	0.23	0.11
GAIF01015176.1	0.04	0	0	0.04	0	0
GAIF01015177.1	0	0	0	0.75	3.27	0.14
GAIF01015178.1	117.7	14.08	2.25	8.59	1.73	3.56
GAIF01015181.1	1.3	0	2.75	4.81	0	0
GAIF01015183.1	0.51	0	0	0.81	2.64	1.03
GAIF01015184.1	29.89	51.47	38.02	27.71	35.62	7.68
GAIF01015186.1	0.09	0.24	0	0	0.22	0
GAIF01015188.1	51.32	52.66	23.4	235.6	298.75	28
GAIF01015190.1	138.63	84.07	77.62	97.96	49.58	65.52
GAIF01015191.1	95.64	93.57	136.53	48.62	55.2	137.82
GAIF01015192.1	0.06	0.07	0.03	0.11	0.04	0
GAIF01015193.1	28.29	20.39	19.85	23.09	19.42	6.05
GAIF01015195.1	0	0	0	0	0.6	0
GAIF01015196.1	49.07	30.96	52.83	34.86	21.55	24.42
GAIF01015198.1	19.67	10.65	11.83	50.57	65.21	13.59
GAIF01015199.1	3.37	2.2	1.66	4.58	6.75	0
GAIF01015200.1	8.61	10.75	7.92	23.19	21.27	2.66
GAIF01015202.1	0.23	0.25	0	0.26	2.17	0.25
GAIF01015203.1	0	0.14	0	0	0	0
GAIF01015205.1	0	0	0	0.06	0	0
GAIF01015206.1	1.75	2.15	0.38	1.22	1.4	0
GAIF01015207.1	0	0	0	0	0.36	0
GAIF01015208.1	0.51	0.45	1.07	0.7	1.45	0.36
GAIF01015209.1	0.02	0.02	0.1	0.17	0	0.08
GAIF01015210.1	105.43	95.6	81.2	27.29	16.86	27.24
GAIF01015211.1	0	0.17	0	0.26	1.08	0
GAIF01015212.1	213.89	173.76	114.83	176.76	177.65	394.47
GAIF01015213.1	4749.08	5418.65	9745.42	2925.76	1954.33	7572.4
GAIF01015215.1	113.75	135.3	158.36	578.61	279.52	131.7
GAIF01015216.1	0	0	0.04	0	0.08	0
GAIF01015218.1	0.59	0.89	0.13	0	0	0
GAIF01015219.1	0.61	0.19	0	2.34	9.5	8.13

GAIF01015221.1	0.88	3.56	2.17	0.21	0.34	0.17
GAIF01015224.1	14.96	12.36	11.93	20.66	11.9	3.15
GAIF01015227.1	0	0.42	0	1.49	2.09	0.38
GAIF01015228.1	0	0	0	0.6	2.86	0
GAIF01015229.1	0.04	0	0	0.42	2.32	0
GAIF01015230.1	0	0	0	0	0	0.14
GAIF01015231.1	0	0	0	0	0.06	0
GAIF01015233.1	0	0.05	0.01	0.03	1.86	0.03
GAIF01015234.1	0	0.08	0	0.18	0	0
GAIF01015237.1	10.97	17.5	2.74	18.1	18.24	0.63
GAIF01015238.1	0.14	0.22	0	0	0.17	0
GAIF01015239.1	33.79	17.01	20.36	94.84	90.13	23.19
GAIF01015240.1	0.16	0	0	0.37	0.95	0.17
GAIF01015241.1	7.58	2.71	0.88	1.24	1.18	0.2
GAIF01015242.1	10.69	2.25	1.52	15.85	22.54	43.35
GAIF01015243.1	88.04	54.41	49.52	11.83	11.55	28.07
GAIF01015244.1	0.05	0	0	0.18	0.92	0
GAIF01015246.1	0.46	0.78	0.06	0.06	0.32	0.03
GAIF01015248.1	9.42	10.02	0	19.43	18.56	0.87
GAIF01015249.1	0	0	0	0.62	0.39	0
GAIF01015250.1	0	0	0	0	0.12	0
GAIF01015251.1	1.35	2.63	1.56	1.68	1.22	0.64
GAIF01015252.1	63.2	66.56	15.12	34.58	31.08	5.43
GAIF01015254.1	0	0	0	0.17	0	0
GAIF01015255.1	0.37	0.81	0.27	3.47	2.84	0
GAIF01015257.1	0.01	0	0.06	0.03	0	0
GAIF01015258.1	0	0.1	0	0	0.11	0
GAIF01015259.1	0.61	0.37	0	0.16	0.65	0
GAIF01015261.1	5.67	6.79	6.06	21.61	20.38	4.45
GAIF01015262.1	2.84	0.39	0.08	5.1	23.71	2.89
GAIF01015263.1	1.31	0.36	0.18	1.52	2.17	1.27
GAIF01015266.1	0.13	0.05	0.18	2.1	11.82	0.46
GAIF01015268.1	1.8	0.83	0.97	0.49	0.14	0
GAIF01015270.1	0	0	0	0.14	0	0
GAIF01015272.1	0	0	0	0	0.22	0
GAIF01015273.1	22.3	29.89	21.12	4.14	1.73	0.37
GAIF01015275.1	16.19	22.51	10.27	80.75	32.33	9.49
GAIF01015276.1	17.8	25.23	21.35	41.09	32.83	6.25
GAIF01015277.1	0.04	0.04	0.12	0	0	0.12
GAIF01015279.1	64.15	54.07	29.14	110.32	107.98	62.64
GAIF01015281.1	60.55	36.47	28.44	80	53.37	14.65
GAIF01015283.1	0.47	2.05	0.05	1.97	2.54	0
GAIF01015285.1	0.61	0.31	0	0.29	0.13	0
GAIF01015286.1	2.39	1.69	1.84	0.42	0.29	0.92

GAIF01015287.1	1.17	0.47	1.78	0.97	0.53	0.46
GAIF01015288.1	1.32	1.01	2.75	8.8	39.81	4.56
GAIF01015289.1	0	0	0	0	0.29	0
GAIF01015292.1	0	0	0	0.01	0	0
GAIF01015293.1	0.97	0.78	0.46	0.89	0.55	0.67
GAIF01015295.1	0	0	0	0	0	0.16
GAIF01015296.1	4.17	7.74	3.46	6.22	2.91	0.42
GAIF01015298.1	0	0.18	0	0	0.3	0
GAIF01015299.1	0	0	0	0	0.12	0
GAIF01015300.1	19.06	24.2	16.78	39.91	40.36	21.17
GAIF01015301.1	0.19	0.41	0.1	0.18	0.41	0.21
GAIF01015302.1	463.17	562.58	323.17	558.7	216.73	117.83
GAIF01015303.1	0.15	0.12	0.04	0	0.45	0.08
GAIF01015305.1	0	0.06	0.12	0	0.06	0.06
GAIF01015307.1	0	0	0	0	0.04	0
GAIF01015313.1	0	0	0	0	5.42	0
GAIF01015314.1	29.89	22.84	34.27	47.22	17.44	29.02
GAIF01015316.1	0.13	0.42	0.14	0	0	0
GAIF01015317.1	0	0.05	0	0.15	2.75	0.23
GAIF01015318.1	0	0	0	0	0	0.14
GAIF01015319.1	0.6	0	0.44	0.58	0.24	0.56
GAIF01015320.1	0.11	0.14	0	0.48	0.18	0
GAIF01015321.1	33.3	60.71	40.71	169.87	200.05	40.56
GAIF01015322.1	1.42	0.62	0	2.15	8.28	0.45
GAIF01015324.1	12.13	8.92	12.08	17.34	13.38	7.01
GAIF01015326.1	0	0	0	0.12	0.1	0
GAIF01015327.1	0	0	0	0	0.11	0
GAIF01015328.1	0	0	0.05	0	0	0
GAIF01015329.1	0	0	0	0	0.11	0
GAIF01015330.1	0.42	0	0	1.7	4.77	0.23
GAIF01015331.1	0.27	0.21	0.1	0.46	0.53	0.03
GAIF01015332.1	100.46	103.14	72.3	28.37	22.08	5.16
GAIF01015333.1	0	0	0	0	0.05	0
GAIF01015334.1	14.63	11.79	7.61	39.84	72.02	5.05
GAIF01015335.1	0.07	0	0.16	0	0	0
GAIF01015336.1	60.2	57.04	9.04	8.65	6.82	1.87
GAIF01015337.1	30.86	35.2	29.41	42.3	21.18	14.06
GAIF01015338.1	2.95	3.73	3.96	0.46	0.51	0
GAIF01015339.1	0	0	0	1.78	1.5	0
GAIF01015340.1	0	0	8.63	0	1.89	1.29
GAIF01015342.1	0.92	0.45	0.26	1.26	0.73	0.31
GAIF01015343.1	0	0	0	0	0.18	0
GAIF01015344.1	8.96	11.65	10.17	33.86	25.77	4.87
GAIF01015345.1	0.99	0.15	0.31	5.98	14.7	33.55

GAIF01015346.1	76.49	20.71	31.5	74.87	59.59	16.12
GAIF01015347.1	0.48	0.52	0.35	0	0.19	0
GAIF01015349.1	0.89	0.77	0.79	6.59	29.18	0.7
GAIF01015350.1	9.8	11.18	9.98	47.25	40.27	6.21
GAIF01015351.1	1.19	0.34	0.3	0.86	0.73	0.45
GAIF01015352.1	0	0.6	0	0	0	0.15
GAIF01015353.1	5.07	6.13	7.53	5.45	7.11	3.21
GAIF01015354.1	190.16	163.59	105.71	34.07	22.86	15.74
GAIF01015355.1	4.34	7.94	2.79	14.7	83.38	0.6
GAIF01015356.1	0.42	0.53	0.58	2.2	3.29	0.12
GAIF01015357.1	0	0	0	4.02	7.92	1.72
GAIF01015358.1	5.91	0.99	1.48	8.45	33.66	65.74
GAIF01015359.1	0.47	0.34	0.69	0.36	0	0
GAIF01015360.1	0	0	0	0	0.19	0
GAIF01015361.1	1.93	0.43	2	5.21	13.8	11.74
GAIF01015362.1	0.18	0.1	0.1	0	0	0
GAIF01015365.1	0.01	0	0	0	0	0
GAIF01015366.1	16.41	2.2	6.52	8.6	0.83	0
GAIF01015367.1	22.42	18.46	40.82	59.35	44.07	33.65
GAIF01015368.1	0.54	1.17	0	2.44	1.26	17.49
GAIF01015369.1	38.91	27.89	37.04	79.98	56.99	56.4
GAIF01015370.1	0	0	0	0	0.52	0
GAIF01015374.1	10.6	6.89	5.81	19.88	33.83	6.22
GAIF01015376.1	39.12	12.56	14.31	21.28	20.12	8.21
GAIF01015379.1	0.93	0.19	0.17	0.94	2.75	1.48
GAIF01015380.1	0.45	0	0.1	0	0	0
GAIF01015381.1	18.79	15.92	4.41	14.71	24.26	0
GAIF01015382.1	176.6	83.74	48.82	128.26	132.84	52.52
GAIF01015383.1	95.62	84.64	72.81	188.47	116.51	73.47
GAIF01015384.1	2.93	7.68	26.92	10.21	34.62	116.04
GAIF01015385.1	28.28	42.92	55.06	3.42	1.25	1.15
GAIF01015389.1	50.17	21.65	12.87	94.87	93.08	65.75
GAIF01015393.1	0	0.08	0	0.26	1.89	0.16
GAIF01015395.1	1.31	1.16	1.33	0.96	0.76	1.97
GAIF01015397.1	58.38	44.32	22.98	157.01	178.47	53.79
GAIF01015398.1	0.91	0	0	3.3	19.41	0.13
GAIF01015399.1	0.74	0.36	0.27	1.81	3.2	0.41
GAIF01015401.1	0	0	0.14	0	0	0
GAIF01015404.1	1.61	0.68	0	2.75	2.21	0.73
GAIF01015405.1	1.31	1.58	0.96	1.01	0.35	0.32
GAIF01015406.1	1.85	2.68	0.56	8.59	20.39	0
GAIF01015407.1	19.16	31.81	41.64	49.13	28.43	13.08
GAIF01015409.1	0.61	0.42	0.28	2.57	9.86	0.51
GAIF01015411.1	215.01	241.2	347.25	1539.41	774.35	387.44

GAIF01015412.1	0.35	0.06	0	1.12	8.04	1.09
GAIF01015413.1	4.64	2.18	1.05	2.29	13.11	35.99
GAIF01015415.1	0	0	0	0	0	0.86
GAIF01015417.1	1.47	0.35	0.05	1.17	1.58	0.87
GAIF01015418.1	17.41	26.71	15.45	48.32	35.93	7.76
GAIF01015419.1	552.32	210.67	120.44	304.03	173.19	263.12
GAIF01015420.1	0	0.14	0	0	0	0
GAIF01015421.1	0.47	0.14	0.05	0.7	0.21	0.09
GAIF01015422.1	9.12	5.58	6.46	7.73	4.56	3.04
GAIF01015425.1	3.8	2.85	3.97	11.76	4.9	3.09
GAIF01015426.1	0.05	0.06	0.23	0	0	0
GAIF01015427.1	11.59	13.31	5.06	37.7	30.43	3.9
GAIF01015428.1	0.31	1.36	1.2	0.73	0.19	0
GAIF01015430.1	2.05	0.9	0	6.43	6.73	0.06
GAIF01015435.1	3.89	7.37	1.97	10.03	13.67	0.09
GAIF01015436.1	0.15	0	0	0.42	0	0
GAIF01015437.1	0	0.05	0	0	0	0
GAIF01015438.1	0.55	0.15	0.46	2.41	10.8	0.15
GAIF01015439.1	1.04	0	0	2.81	4.46	1.25
GAIF01015441.1	0	0.43	0.25	0	0	0
GAIF01015442.1	124.61	168.6	70.13	340.36	243.92	20.85
GAIF01015443.1	0.33	0.24	0	1.8	4.54	0
GAIF01015444.1	5	5.79	5.24	2.96	1.87	1.59
GAIF01015447.1	8.75	4.89	2.21	19.63	29.58	2.22
GAIF01015448.1	0	0	0	0.21	0	0
GAIF01015449.1	0	0.05	0.05	0	0.05	0.1
GAIF01015450.1	46.93	47.63	35	96.51	51.21	10.93
GAIF01015451.1	0	0.31	0.24	0	0	0
GAIF01015452.1	103.26	43.71	0.07	59.19	84.11	0.01
GAIF01015453.1	1.99	1.24	1.82	0.97	0	0.69
GAIF01015454.1	0	0	0	0	0.01	0
GAIF01015455.1	0.3	0	0	0.24	0	0
GAIF01015456.1	10.88	4.08	1.69	11.13	12.19	9.62
GAIF01015457.1	0.48	0.21	0.1	4.1	24.1	0.42
GAIF01015458.1	0	0.48	0	0.03	0.71	0
GAIF01015459.1	0	0	0.03	0.01	0	0
GAIF01015461.1	0	0	0	0	22.68	32.6
GAIF01015462.1	0	0	0	0	0.02	0
GAIF01015465.1	6.73	5.78	4.47	26.34	25.73	2.33
GAIF01015466.1	37.87	39.4	49.95	33.62	20.97	16.51
GAIF01015468.1	14.89	14.76	9.52	10.06	6.8	2.6
GAIF01015469.1	0	0	0	0	0.19	0
GAIF01015470.1	0	0	0	0	1.49	0
GAIF01015477.1	0	0.25	0.13	0	0.14	0.13

GAIF01015478.1	1190.5	711.49	1053.28	586.25	311.02	533.52
GAIF01015479.1	0.01	0.06	0	0.1	1.08	0
GAIF01015480.1	5.77	7.51	6.71	13.4	6.53	4.12
GAIF01015481.1	7.61	0	0	11.85	10.56	6.23
GAIF01015482.1	0.24	0.13	0	0.14	0.14	0
GAIF01015484.1	0	0	0	0	0.27	0
GAIF01015486.1	0	0.21	0	0.23	0.71	0
GAIF01015487.1	1	2.46	1.24	2.31	0.65	0.2
GAIF01015488.1	6.83	5.15	1.68	22.81	30.43	0.69
GAIF01015489.1	0.15	0.11	0	0.06	0.93	0
GAIF01015490.1	0	0	0	0	0.22	0
GAIF01015491.1	154.34	225.87	153.41	40.69	47.73	21.41
GAIF01015493.1	0.02	0.08	0	0.22	0.88	0
GAIF01015494.1	2.94	0	0	2.81	0.55	0
GAIF01015495.1	12.69	21.27	16.11	1.3	2.39	2.86
GAIF01015496.1	8.58	3.18	1.38	15.17	30.18	1.04
GAIF01015497.1	79.96	17.49	27.02	74.01	74.56	134.2
GAIF01015498.1	0.16	0.17	0	0.56	0.39	0.18
GAIF01015500.1	55.93	56.39	72.84	27.14	17.07	12.32
GAIF01015502.1	0.38	0	0	3.58	12.57	0.99
GAIF01015504.1	0.04	0.07	0.04	0	0	0.15
GAIF01015505.1	0.12	0	0	0.85	3.09	0
GAIF01015506.1	0	0	0.01	0	0	0
GAIF01015509.1	0.6	0.47	0	2.2	12.1	3.85
GAIF01015510.1	0.21	0	0.23	0.98	1.14	0.23
GAIF01015512.1	0	0.61	0.51	0.22	0	0.31
GAIF01015515.1	0	0	0	0.09	0	0
GAIF01015516.1	0.28	0	0	24.81	0	1.86
GAIF01015517.1	1.1	0.31	0.15	2.75	12.44	0.8
GAIF01015519.1	0.46	0	0	2.11	9.65	0.32
GAIF01015520.1	30.8	8.49	8.84	30.93	40.35	0
GAIF01015521.1	10.99	12.84	9.09	58.48	66.12	9.6
GAIF01015524.1	0	0	0	0	0.27	0
GAIF01015525.1	0	0	0	0	0.09	0
GAIF01015526.1	0	0	0	0	0.22	0
GAIF01015527.1	1.19	0	0	1.87	4.12	2.48
GAIF01015528.1	13.06	4.84	4.24	21.7	11.42	12.59
GAIF01015529.1	0	0.27	0	0.23	0	0.16
GAIF01015531.1	2.68	3.31	4.82	15.28	7	2.38
GAIF01015532.1	7.91	11.65	5.18	47.94	47.8	4.25
GAIF01015533.1	0.16	0.28	0	0.42	0.64	0.38
GAIF01015536.1	0.12	0.26	0.13	1.41	2.05	0.27
GAIF01015538.1	1.18	0.46	0	3.42	21.08	0.93
GAIF01015539.1	22.21	16.54	4.05	12.22	12.48	16.68

GAIF01015540.1	0	0.46	0.11	0.56	0	0.48
GAIF01015541.1	0.98	5.32	1.25	7.76	9.02	0.18
GAIF01015542.1	4.87	18.98	0.33	10.8	14.13	0
GAIF01015543.1	0	0.03	0	0.06	0.05	0
GAIF01015544.1	82.64	56.62	49.41	60.17	52.11	8.12
GAIF01015545.1	5.25	3.16	3.65	10.43	18.56	10.86
GAIF01015547.1	0.12	0.11	0	0.18	0	0.03
GAIF01015549.1	17.45	15.78	5.7	27.14	44.02	0
GAIF01015550.1	151.12	71.82	103.67	151.8	70.78	19.6
GAIF01015552.1	0	0.11	0.22	0	0.24	0
GAIF01015553.1	4.03	4.48	1.33	5.4	1.46	0.12
GAIF01015555.1	0	0.33	0	0.17	1.09	0
GAIF01015556.1	5.41	0.44	0	0	0	0
GAIF01015558.1	52.85	439.25	93.56	0	8.96	6.64
GAIF01015561.1	5.47	5.94	4.57	4.79	6.78	1.4
GAIF01015563.1	0	0	0	0	0	0.01
GAIF01015564.1	124.92	59.02	69.05	203.38	135.32	59.98
GAIF01015565.1	0.04	0.19	0.02	0.02	0.05	0.01
GAIF01015567.1	0.13	0	0	0.05	0	0.02
GAIF01015569.1	0	0	0.14	0.25	0.78	0
GAIF01015571.1	0	0.14	0.3	0	0.26	0
GAIF01015572.1	0	0	0	0	0.07	0
GAIF01015574.1	0	0	0.23	0	0	0
GAIF01015575.1	22.86	18.93	22.68	117.55	66.25	94.86
GAIF01015576.1	0	0.06	0.02	0.28	0.57	0.06
GAIF01015577.1	3.18	10.83	0.17	14.59	36.27	8.58
GAIF01015580.1	0.02	0	0.07	0	0.04	0
GAIF01015581.1	4.92	3.27	2.25	29.31	60.99	1.13
GAIF01015582.1	1.64	0.71	0	3.55	13.29	0.6
GAIF01015583.1	0.17	0.16	0	0	0	0.51
GAIF01015585.1	0	0	0	0.09	0	0
GAIF01015586.1	130.34	52.49	49.13	84.88	99.2	149.71
GAIF01015587.1	12.66	5.58	9.21	11.92	8.64	11.5
GAIF01015588.1	0.33	0	0	1.16	6.29	0.98
GAIF01015589.1	5.81	3.33	5.57	15.47	20.36	11.04
GAIF01015590.1	2.36	1.74	0.3	5.74	11.79	0.09
GAIF01015591.1	0.15	0	0.08	0	0	0
GAIF01015592.1	0	0	0.76	0	0	0
GAIF01015594.1	0	0	0	1.01	5.45	0.1
GAIF01015596.1	0.95	0.78	0.39	0	0	0
GAIF01015598.1	46.81	22.46	5.11	126.95	158.97	18.57
GAIF01015599.1	65.39	28.13	26.56	69.05	113.68	157.19
GAIF01015601.1	48.08	50.95	27.93	54.28	50.78	26.95
GAIF01015602.1	0.13	0.02	0	0.34	0.66	0

GAIF01015603.1	0	0	0	0.7	1.27	0.27
GAIF01015605.1	0	0	0	0	0.03	0
GAIF01015606.1	12.08	10.07	12.14	15.45	30.32	26.19
GAIF01015610.1	0.02	0	0	0	0.51	0.06
GAIF01015611.1	0.07	0	0	0.73	2.14	0
GAIF01015613.1	1.71	0.93	0.12	1.11	2.18	0
GAIF01015614.1	155.2	49.46	52.69	11.12	7.5	5.74
GAIF01015615.1	0.21	0	0	0.64	0.01	0
GAIF01015616.1	0.73	2.06	0	2.97	9.89	0
GAIF01015618.1	0.08	0	0	0	0	0.04
GAIF01015619.1	7.23	4.9	1.34	12.86	28.01	3.52
GAIF01015620.1	0	0	0	0	0.02	0
GAIF01015622.1	0	0	0	0.08	0.06	0
GAIF01015623.1	62.49	25.92	101.5	41.92	17.86	81.08
GAIF01015624.1	0	0	0.27	0.7	0	0.02
GAIF01015627.1	0.03	0.02	0	0.11	0.03	0
GAIF01015628.1	0	2.92	0	0	0	0
GAIF01015630.1	0	0	0	0.91	7.19	0.24
GAIF01015631.1	0.41	0	0	0.24	0.17	0
GAIF01015632.1	0	0.2	0	0	0.23	0
GAIF01015633.1	4.7	5.26	10.2	0.37	0	0.27
GAIF01015634.1	0.67	0.73	1.19	4.79	5.27	5.46
GAIF01015635.1	30.01	32.45	43.55	40.02	31.22	24.65
GAIF01015636.1	4.42	0.99	0.43	3.87	6.96	0
GAIF01015637.1	2138.63	733.48	973.9	40.4	37.98	38.87
GAIF01015639.1	15.85	17.92	13.29	25.83	38.71	73.8
GAIF01015642.1	0.27	0.15	0	1.27	1.97	0
GAIF01015644.1	18.17	11.8	10.04	15.84	29.88	30.1
GAIF01015645.1	0.17	0	0	0	0	0
GAIF01015646.1	0.31	0.1	0	0.05	0	0
GAIF01015647.1	24.63	26.5	12.58	53.29	29.27	8.78
GAIF01015648.1	5.62	6.14	5.69	10.94	17.02	27.1
GAIF01015649.1	0	0	0.11	0.17	0.12	0
GAIF01015652.1	0	0.12	0	0	0	0
GAIF01015655.1	0	0.33	0	0.18	0	0
GAIF01015657.1	0.04	0.04	0	0	0.14	0
GAIF01015658.1	156.81	313.29	193.75	41.05	33.59	12.51
GAIF01015659.1	21.07	8.79	4.69	2.55	1.45	0.76
GAIF01015660.1	0	0	0	0	0	0.06
GAIF01015662.1	0.11	0	0.12	0	0	0
GAIF01015663.1	0.06	0	0	0	1.6	0
GAIF01015664.1	0.26	0.28	0.7	0.15	0.31	0.57
GAIF01015665.1	76.1	47.6	24.28	76.46	58.45	13.39
GAIF01015666.1	2.31	0.16	0	7.89	18.79	0.64

GAIF01015667.1	0	0	0	0	0.12	0
GAIF01015669.1	0	0.23	0.24	1.99	8.26	0.24
GAIF01015670.1	25.39	23.53	13.86	57.5	56.88	7.21
GAIF01015672.1	0	0.03	0	0.03	0.19	0.03
GAIF01015674.1	11.1	20.78	12.72	42.4	37.13	3.41
GAIF01015675.1	1.57	0.16	0.16	8.77	23.32	5.43
GAIF01015676.1	40.7	73.26	15.01	97.1	82.27	4.72
GAIF01015677.1	3.84	3.45	2.88	3.69	2.06	0.77
GAIF01015678.1	0.13	0	0	0.05	0	0
GAIF01015679.1	0	0.4	0.27	0	0	0
GAIF01015681.1	9.99	13.91	6.33	32.26	38.68	2.46
GAIF01015683.1	5.12	2.34	0.3	14.22	38.78	5.34
GAIF01015684.1	1.32	0.96	0.53	7.6	10.09	0.69
GAIF01015685.1	22.87	30.76	66.12	24.82	35	37.29
GAIF01015686.1	0.08	0	0	0	0.1	0
GAIF01015687.1	0.14	0	0	0	1.69	0
GAIF01015688.1	0.26	0	0	0	0.68	0
GAIF01015689.1	0.18	0.06	0	0.97	4.89	0.02
GAIF01015691.1	1.37	0	0	1.01	4.94	0
GAIF01015692.1	39.13	29.34	35.56	82.7	124.87	64.1
GAIF01015693.1	0	0	0	0	1.49	0
GAIF01015694.1	0	0	0	0.34	1.24	0
GAIF01015695.1	0.13	0.25	0.06	1.84	6.32	0.16
GAIF01015697.1	7.7	2.86	1.26	41.24	106.87	9.14
GAIF01015698.1	12.33	10.72	2.82	14.92	23.45	34.56
GAIF01015699.1	0.3	0.18	0	1.58	8.56	0.07
GAIF01015700.1	5.8	6.1	7.11	5.42	4.8	2.74
GAIF01015701.1	0	0	0	0	0	0.03
GAIF01015704.1	17.83	16.75	9.46	43.73	51.74	6.99
GAIF01015705.1	0	0	0	0.15	0.63	0
GAIF01015707.1	102.11	123.5	128.43	106.53	71.52	57.84
GAIF01015710.1	0	0.05	0	0.95	4.16	0
GAIF01015711.1	0	0	0	0.11	0.87	0
GAIF01015712.1	0	1.8	0	0.38	0	0
GAIF01015713.1	12.77	4.89	7.8	16.84	16.05	49.73
GAIF01015716.1	17.13	5.99	3.73	11.12	7.19	1.65
GAIF01015719.1	2.44	0.7	0.44	2.83	11.49	5.1
GAIF01015720.1	76.43	0.14	5.42	86.06	32.11	5.44
GAIF01015723.1	0.18	0	0	0	0	0
GAIF01015724.1	0	0	0	0.47	1.22	0
GAIF01015725.1	0	0	0	0.35	1.21	0.06
GAIF01015728.1	17.62	22.89	21.29	52.24	22.87	8.52
GAIF01015730.1	3.57	3.14	1.85	1.04	0.83	1.5
GAIF01015731.1	9.13	7.38	3.36	4.99	5.34	1.63

GAIF01015732.1	5.81	8.55	18.07	18.72	11.72	15.43
GAIF01015734.1	140.83	119.39	48.32	159.34	79.07	6.46
GAIF01015735.1	57.69	53.21	54.36	104.4	45.36	18.92
GAIF01015736.1	0	0.08	0	0.43	2.05	0
GAIF01015737.1	2.27	3.68	1.75	4.77	2.21	0.16
GAIF01015739.1	10.57	7.01	2.55	8.73	18.43	4.92
GAIF01015741.1	126.55	145.74	194.98	156.48	106.92	34.78
GAIF01015742.1	311.78	422.6	862.1	33.91	20.31	91.22
GAIF01015743.1	0.09	0.3	0	0.75	2	0
GAIF01015744.1	0	0	0.01	0	0	0
GAIF01015745.1	0.33	0.23	0	0	0.23	0
GAIF01015746.1	82.13	14.36	4.83	98.66	394.51	73.11
GAIF01015747.1	0.18	0	0	0	1.63	0.1
GAIF01015748.1	4.13	3.5	1.46	11.47	16.6	1.7
GAIF01015749.1	0.04	0	0	0.15	0.16	0
GAIF01015750.1	10.82	16.15	11.69	24.44	24.83	5.54
GAIF01015751.1	0.39	0.84	0.12	1.18	1.45	0
GAIF01015754.1	0	0	0	0.5	1.77	0
GAIF01015755.1	61.2	53.01	19.13	85.21	75.44	3.79
GAIF01015756.1	0.07	8.24	27.24	0.39	2.43	4.53
GAIF01015757.1	8.05	10.24	7.52	30.4	36.8	4.39
GAIF01015758.1	4.79	2.77	4.07	4.3	5.05	1.53
GAIF01015759.1	0.19	0	0	1.12	2.88	0
GAIF01015760.1	13.19	10.37	5.23	30.82	34.37	26.02
GAIF01015761.1	15.78	1.78	0.56	80.13	316.85	32.92
GAIF01015762.1	0	0.1	0	0.04	0.02	0.04
GAIF01015763.1	19.38	36.3	19.18	19.54	39.83	40.2
GAIF01015764.1	0.23	0.44	0	0.09	0.21	0
GAIF01015765.1	15.48	8.93	12.92	8.5	7.29	7.23
GAIF01015766.1	24.6	13.05	6.22	34	24.83	23.66
GAIF01015767.1	0.09	0	0	0	0	0
GAIF01015769.1	0	0	0	0	0.18	0
GAIF01015771.1	43.57	46.24	12.15	79.85	74.33	9.53
GAIF01015772.1	0.14	0	0	0.26	0.11	0.11
GAIF01015776.1	12.85	18.16	17.61	45.83	39.21	4.64
GAIF01015782.1	0	0	0	0	0.15	0
GAIF01015783.1	23.26	16.7	16.45	38.17	47.61	17.52
GAIF01015784.1	0.17	0.36	0	1.15	3.77	0
GAIF01015786.1	0	0	0	0.02	0.02	0
GAIF01015788.1	6.57	8.79	8.83	20.16	10.61	3.86
GAIF01015789.1	0.97	1.05	0.06	6.8	24.25	0
GAIF01015790.1	0	23.86	24.29	0	0.11	2.07
GAIF01015791.1	0	0.09	0	0.05	0	0
GAIF01015792.1	0.06	0	0	0	0	0

GAIF01015793.1	0.14	0	0	0.08	0.08	0
GAIF01015794.1	0.39	0.85	1	1.36	0.94	0.43
GAIF01015795.1	13.15	6.1	4.3	8.04	9.12	9.02
GAIF01015796.1	62.7	59.57	32.85	0	0.09	0
GAIF01015797.1	0	0.14	0.15	0	0	0
GAIF01015798.1	0	20.32	11.54	12.5	19.27	2.92
GAIF01015799.1	0.18	0.05	0	0.14	0.13	0
GAIF01015800.1	0.29	0.44	0.9	0.47	0.21	0.51
GAIF01015801.1	1.8	2.68	0	0	0.35	0
GAIF01015802.1	0	0	0	0.12	0.37	0.22
GAIF01015803.1	0	0	0	0	0.12	0
GAIF01015804.1	0.63	0.81	0.56	4.63	12	0.69
GAIF01015805.1	0.11	0.12	0.12	1.05	6.55	0
GAIF01015807.1	4	2.44	2.97	7.6	5.93	3.04
GAIF01015808.1	0.12	0.13	0	0.86	1.49	0.54
GAIF01015809.1	19.88	30.01	31.76	114.97	167.61	26.7
GAIF01015810.1	0	0	0	0.29	2.33	0.06
GAIF01015811.1	0.01	0	0.11	0.05	0.04	0.05
GAIF01015812.1	0	0	0	0	0.06	0
GAIF01015813.1	0.07	0.15	0.2	0.07	0.04	0
GAIF01015815.1	0.85	0.09	0.09	0.1	0.2	0.09
GAIF01015816.1	12.92	10.07	16.18	33.84	28.34	19.45
GAIF01015817.1	400.76	563.62	1157.25	208.52	223.7	650.79
GAIF01015819.1	11.55	8.01	12.5	21.57	7.58	23.91
GAIF01015820.1	0.48	0.38	0.56	2.15	5.09	0.78
GAIF01015821.1	0	0	0	0.17	1.21	0
GAIF01015822.1	0.89	0.16	0	0.34	4.49	0.16
GAIF01015825.1	62.23	97.94	91.98	62.71	43.41	16.64
GAIF01015826.1	0	0	0	0	0.32	0.1
GAIF01015827.1	0	0.02	0.02	0	0	0
GAIF01015829.1	0	0	0	0	0.21	0
GAIF01015830.1	0	0	0	0	0.07	0
GAIF01015831.1	0.63	0	0.62	0.25	0.23	0.19
GAIF01015832.1	1.99	0.83	0.32	0.17	0.04	0.04
GAIF01015833.1	33.76	13.98	17.56	26.31	9.96	2.92
GAIF01015834.1	0.11	0	0	0.13	0.14	0
GAIF01015836.1	3.42	2.36	0.13	6.89	14.69	1.79
GAIF01015837.1	56.15	66.23	99.79	2.13	2.2	9.37
GAIF01015839.1	0	0	0	0	10.72	0
GAIF01015842.1	20.84	26.77	36.78	117.17	79.73	21.52
GAIF01015843.1	0.26	0.28	0.12	0.64	5.45	0.16
GAIF01015845.1	12.01	18.6	15.17	45.39	27.35	7.5
GAIF01015848.1	0.19	0.1	0.1	2.59	3.69	0.31
GAIF01015849.1	12.94	11.06	5.63	24.75	23.77	12.78

GAIF01015850.1	11.06	16.72	23.5	1.99	0.46	1.4
GAIF01015852.1	34.61	3.97	4.96	31.24	17.46	58.91
GAIF01015853.1	0	0	0.06	0.07	0	0
GAIF01015854.1	14.42	10.26	9.16	17.21	5.35	7.07
GAIF01015855.1	0	0	0	0.1	0.11	0
GAIF01015856.1	3.86	6.57	1.66	5.45	2.83	0
GAIF01015858.1	62.61	55.18	85.88	146.28	77.42	102.77
GAIF01015859.1	85.93	21.84	14.7	78.56	63.83	8.98
GAIF01015860.1	0.05	0.03	0.08	0	0	0
GAIF01015862.1	2.09	0.18	0.18	7.48	26.93	17.4
GAIF01015864.1	0.93	0.27	0.07	9.83	30.11	0.27
GAIF01015865.1	0.1	0	0	0.77	3.38	0.07
GAIF01015866.1	129.74	133.54	96.62	205.15	141.37	71.19
GAIF01015867.1	0	0.11	0	0	0.38	0
GAIF01015869.1	0.2	0	0	0.87	1.96	0
GAIF01015870.1	0.06	0.1	0.11	0.04	0	0
GAIF01015874.1	2.06	1.61	1.68	6.67	7.94	1.57
GAIF01015875.1	0	0	0	0.83	2.34	0
GAIF01015877.1	0.48	1.46	1.08	5.09	2.91	0.3
GAIF01015878.1	0	0	0	0.26	0.27	0
GAIF01015879.1	0.12	0	0.06	0.2	0.83	0.26
GAIF01015880.1	0	0	0	0.05	0.32	0
GAIF01015882.1	0	0	0	0.14	1.02	0
GAIF01015883.1	0	0	0	0.56	1.73	0
GAIF01015885.1	3.36	1.36	2.27	6.33	9.85	1.66
GAIF01015886.1	0.39	0.21	0.21	1.23	10.52	1.06
GAIF01015887.1	0	0.32	0	0.37	2.09	0.43
GAIF01015888.1	2.73	2.3	1.38	2.42	2.25	0.51
GAIF01015889.1	0.19	0.4	0	0.21	1.1	0.35
GAIF01015890.1	0	0	0	0	2.89	0
GAIF01015891.1	0	0.14	0	0	0	0
GAIF01015892.1	0.41	0	0	2.66	4.72	0.18
GAIF01015893.1	0.4	0.07	0.05	1.95	8.33	0.5
GAIF01015894.1	0	0	0.22	0.28	2.4	1.55
GAIF01015895.1	0.13	0	0.07	0	0	0
GAIF01015897.1	0.18	0.16	0.18	0.41	0.29	0.56
GAIF01015898.1	0	0.07	0.07	0	0	0.07
GAIF01015899.1	0	0.4	0	0.08	0	0
GAIF01015900.1	0	0.21	0	0	0.12	0
GAIF01015901.1	290.78	284.23	225.57	485.59	333.92	103.86
GAIF01015903.1	17.74	25.53	20.05	70.86	45.19	11.39
GAIF01015904.1	2840.9	107.99	304.77	1848.83	257.75	2072.98
GAIF01015906.1	0.35	0	0	5.86	33	0.14
GAIF01015907.1	0.33	0.42	0.61	0.81	1.4	0

GAIF01015908.1	0	0	0	0	0	0.29
GAIF01015909.1	3.58	6.16	7.33	16.06	8.29	1.77
GAIF01015910.1	12.34	19.43	6.9	42.63	28.08	3.86
GAIF01015911.1	2.87	2.28	0.99	19.15	58.88	0.74
GAIF01015913.1	0.03	0	0	0	0	0
GAIF01015914.1	0.86	0	0.41	2.32	3.59	0.48
GAIF01015915.1	0.08	0	0	0.49	3.85	0.37
GAIF01015917.1	14.15	5.29	16.61	4.44	0.82	8.61
GAIF01015918.1	0	0.32	0	0.23	0.12	0
GAIF01015920.1	0	0	0	0	0.19	0
GAIF01015921.1	17.54	8.55	12.09	29.91	16	58.67
GAIF01015922.1	30.12	48.74	50.36	118.72	61.51	26.04
GAIF01015924.1	6.42	3.09	7.05	17.49	8.91	16.48
GAIF01015925.1	0	0	0	0.12	0.73	0
GAIF01015926.1	0	0	0	0.1	1.12	0
GAIF01015927.1	0.13	0	0	0.31	1.69	0.05
GAIF01015930.1	0.04	0	0.09	0.18	2.11	0.04
GAIF01015934.1	10.93	8.2	17.24	19.25	11.93	27.14
GAIF01015935.1	0	20.75	14.23	22.78	38.01	5.81
GAIF01015937.1	0.13	0.14	0	0.89	3.38	0.14
GAIF01015938.1	1.82	1.29	0.54	3.53	0.89	0.79
GAIF01015940.1	0.98	0.4	0.67	0.85	0.15	0.4
GAIF01015941.1	0	0	0	0.14	0	0
GAIF01015942.1	0.1	0.05	0.05	1.32	3.62	0
GAIF01015943.1	0	0	6.71	1.23	0	0
GAIF01015944.1	0.05	0	0	0	0	0
GAIF01015947.1	0	0	0	0.14	0.29	0
GAIF01015948.1	7.05	9.73	4.36	25.27	17.93	1.39
GAIF01015949.1	0.7	0	0	1.77	0	0
GAIF01015950.1	50.23	39.49	33.24	66.24	59.37	31.01
GAIF01015952.1	7.61	7.51	7.23	16.59	26.95	3.02
GAIF01015953.1	12.49	25.75	13.71	48.7	46.18	5.2
GAIF01015954.1	2.78	5.59	1.43	2.56	3.44	0
GAIF01015956.1	7.48	8.8	3.76	20.89	21.87	3.25
GAIF01015957.1	0.37	0.64	1.29	0	0	1.92
GAIF01015958.1	0.05	0.06	0	0.06	0	0
GAIF01015959.1	1.05	0.11	0.17	6.7	23.13	0.95
GAIF01015961.1	0.15	0.08	0	0.38	1.86	0
GAIF01015963.1	0	0.46	0.34	0	0	0
GAIF01015964.1	0	0.19	0	0	0	0
GAIF01015966.1	18.39	23.48	48.66	26.39	19.73	36.93
GAIF01015967.1	2979.48	1531.89	1570.7	1548.73	1765.06	12907.66
GAIF01015968.1	2.72	1.85	3.49	0.28	0.11	0.25
GAIF01015969.1	23.53	21.58	37.65	33.25	33.66	44.28

GAIF01015970.1	5.04	5.22	4.81	21.68	27.27	3.47
GAIF01015971.1	9.1	9.79	10.43	22.89	17.81	3.05
GAIF01015973.1	10.38	8.47	9.93	13.74	11.23	11.99
GAIF01015976.1	0.2	0.07	0	0	0.16	0.22
GAIF01015977.1	0.14	0.15	0.05	2.68	9.43	0.13
GAIF01015978.1	1.68	5.48	2.71	2.18	1.43	0.11
GAIF01015979.1	30.84	46.93	44.16	111.87	206.48	12.48
GAIF01015985.1	0.53	0.08	0.12	2.95	5.05	0.08
GAIF01015986.1	0.35	0.02	0.29	0	0.26	0
GAIF01015989.1	0.35	0.2	0	1.45	13.95	0
GAIF01015990.1	44.4	33.45	29.25	15.62	3.08	21.13
GAIF01015991.1	36.47	28.76	15.79	136.56	139.09	16.85
GAIF01015992.1	35.14	22.42	11.52	80.63	102.06	8.55
GAIF01015993.1	0.38	0.13	0.27	0.66	0.15	0.37
GAIF01015994.1	0.09	0	0	0	0.1	0
GAIF01015995.1	0	0	0	0	0.22	0
GAIF01015996.1	0.13	0	0	0.41	0	0.11
GAIF01015998.1	2.06	5.35	4.33	14.79	14.92	3.02
GAIF01016001.1	0	0.32	0.66	0.35	4.43	0.11
GAIF01016003.1	0	0	0.12	0	0	0
GAIF01016004.1	28.14	41.73	30.26	51.41	57.09	13.52
GAIF01016007.1	0.06	0	0	0	0	0
GAIF01016008.1	0	0.07	0.04	0.23	1.41	0.04
GAIF01016009.1	5.07	1.8	3.08	28.88	54.53	3.05
GAIF01016011.1	46.18	63.8	24.77	136.84	112.96	22.91
GAIF01016012.1	12.31	16.12	10.07	30.17	16.92	5.08
GAIF01016013.1	0	0.15	0	2.26	14.66	0.09
GAIF01016014.1	0.73	0.28	0.99	0.62	0.21	0.21
GAIF01016015.1	0	0	0	0	0.3	0
GAIF01016016.1	0.03	0.05	0.05	0.08	0.06	0.04
GAIF01016017.1	73.34	98.05	198.64	96.05	49.43	48.85
GAIF01016018.1	3.52	2.05	0.25	4.22	8.53	1.02
GAIF01016019.1	0	0	0	0	0.56	0
GAIF01016021.1	0	0	0	0.07	0	0
GAIF01016022.1	2.52	1.89	3.91	4.72	3.29	1
GAIF01016023.1	3.72	1.31	0	5.2	21.05	11.09
GAIF01016024.1	0.86	0.44	0.74	7.07	16.38	0.12
GAIF01016025.1	0	0.27	0	0	0.15	0.14
GAIF01016027.1	0	0	0	0.19	0.58	0
GAIF01016028.1	0	0.59	0	0	0	0
GAIF01016029.1	0	0.07	0	0	0	0
GAIF01016030.1	26.83	56.79	27.19	108.25	209.88	32.85
GAIF01016031.1	0	0	0	0.1	0.21	0
GAIF01016032.1	0.08	0.25	0.26	0.14	0	0

GAIF01016033.1	11.43	6.04	0	40.79	46.56	7.77
GAIF01016034.1	9.94	6.56	8.64	20.79	26.23	6.4
GAIF01016035.1	0.32	0.28	0.56	0	0	0.07
GAIF01016036.1	0	0	0	0	0.07	0
GAIF01016037.1	7.31	13.16	10.35	18.86	7.04	2.33
GAIF01016039.1	56.26	16.81	10.77	28.85	19.45	15.06
GAIF01016041.1	10.86	13.89	7.54	12.91	22.91	5.87
GAIF01016045.1	0.25	0	0	0	0.34	0
GAIF01016046.1	1.37	0.46	0.23	3.54	10.5	0.46
GAIF01016047.1	2.67	2.61	2.15	6.81	5.14	1.96
GAIF01016049.1	227.41	435.99	191	548.11	442.02	64.9
GAIF01016050.1	0	0.55	1.26	0	0	0.24
GAIF01016053.1	0.17	0.03	0.05	0.82	4.62	0.37
GAIF01016054.1	1.45	0.15	0.25	0.68	1.45	0
GAIF01016055.1	11.34	5.6	7.88	27.78	29.86	9.77
GAIF01016056.1	3.51	3.31	2.66	4.02	3.92	1.41
GAIF01016057.1	0.07	0	0	0	0	0.16
GAIF01016058.1	5.5	3.33	2.42	0.18	0.47	0.17
GAIF01016059.1	0	0	0.37	0	0	0
GAIF01016062.1	0.22	0	0	0	0	0
GAIF01016064.1	34.97	35.78	0	99.07	38.34	17
GAIF01016065.1	0	0	0	0.11	0.22	0.05
GAIF01016066.1	0.31	0.71	0.47	0.85	0.47	0
GAIF01016068.1	0	0.7	0.38	0	0	0
GAIF01016069.1	45.81	88.84	73.05	62.81	40.31	19.92
GAIF01016070.1	0.62	0.97	1.73	0.95	1.11	0.83
GAIF01016071.1	4.21	0.46	0	1.59	2.02	5.91
GAIF01016073.1	0	0	0	56.54	0.2	0
GAIF01016074.1	0.34	0.37	0	0.26	5.59	0.25
GAIF01016075.1	0.28	0.05	0.05	1.82	11.18	0.31
GAIF01016077.1	1.87	0.76	0.26	9.34	25.82	1.03
GAIF01016078.1	0.19	0.1	0.1	0.33	1.26	0
GAIF01016081.1	0.1	0	0.18	0.37	0.69	0.1
GAIF01016082.1	0.73	4.82	0	12.5	22.4	13.66
GAIF01016084.1	0	0	0	0.2	0	0
GAIF01016085.1	0.11	0	0	0.53	0.99	0
GAIF01016087.1	0.03	0.04	0.02	0.13	0.13	0
GAIF01016088.1	0	0	0	0.12	0.5	0
GAIF01016089.1	0	0.11	0.02	0.02	0.01	0.01
GAIF01016090.1	0	0	0	0.05	0.45	0
GAIF01016093.1	0	0.14	0	0	0.31	0
GAIF01016094.1	0.21	0.23	0	0	0.13	0
GAIF01016098.1	16.95	29.18	22.41	24.53	20.89	10.71
GAIF01016100.1	2.63	0.36	0.72	3.05	1.98	1.38

GAIF01016101.1	3.73	0.08	0.16	7.13	40.7	0.48
GAIF01016102.1	2.91	1.81	1.93	7.35	32.05	3.57
GAIF01016104.1	0	0	0	1.6	2.24	0
GAIF01016105.1	0	0	0	0.09	0	0
GAIF01016108.1	118.49	33.76	1.03	12.5	11.38	0
GAIF01016109.1	0	0	0	0.25	0.14	0
GAIF01016111.1	0	0	0	1.02	6.41	0.16
GAIF01016112.1	0.18	0	0.07	0.49	1.88	0
GAIF01016113.1	1.69	2.1	0	6.44	22.62	0
GAIF01016116.1	0.87	0.49	0.34	3.02	14.84	0.25
GAIF01016119.1	0.11	0.25	0	0	0.28	0
GAIF01016120.1	2.17	1.56	0.11	11.93	36.9	0.11
GAIF01016121.1	1.05	0.31	0.31	4.41	19.44	0.52
GAIF01016122.1	10.54	52.5	32.12	24.55	80.42	15.77
GAIF01016124.1	2.18	1.05	0	10.4	7.57	0
GAIF01016125.1	0	0	0	0.05	0	0
GAIF01016126.1	0	0	0	0.02	0	0
GAIF01016127.1	0.22	0	0	0	0.26	0
GAIF01016129.1	10.61	11.89	7.56	21.62	21.63	12.5
GAIF01016130.1	0	0.22	0.11	0	0	0.22
GAIF01016131.1	1.81	5	7.21	2.1	2.76	1.63
GAIF01016132.1	7.67	1.02	0.31	16.29	63.13	71.67
GAIF01016134.1	0.38	1.75	1.09	1.6	1.75	0.84
GAIF01016135.1	0	0	0	0	0.05	0
GAIF01016136.1	0	0.19	0	0	0	0
GAIF01016137.1	12.6	13.72	15.61	30.63	35.48	11.09
GAIF01016138.1	0	0	1.81	0	0	0
GAIF01016139.1	0	0.08	0	0.24	0.23	0
GAIF01016141.1	0	0.26	0	0	0	0.13
GAIF01016142.1	0	0.05	0	0.35	1.06	0
GAIF01016143.1	0	0	0.03	0	0.27	0
GAIF01016144.1	0.32	0.17	0.24	0.35	0	0.43
GAIF01016145.1	10.56	19.08	12.46	31.7	17.77	5.38
GAIF01016146.1	1.49	2.21	3.27	2.51	0.98	0.75
GAIF01016147.1	0.13	0	0	0.22	1.52	0.49
GAIF01016149.1	0	0	0	0	1.46	0
GAIF01016152.1	13.11	42.77	0	45.65	15.77	14.45
GAIF01016153.1	0	0	0	0	0.09	0
GAIF01016154.1	0.09	0	0	0	0	0
GAIF01016155.1	4.73	7.02	6.82	11.23	12.33	2.54
GAIF01016157.1	238.78	140.44	101.29	193.77	193.91	126.07
GAIF01016158.1	93.54	96.17	50.69	126.68	100.92	15.87
GAIF01016160.1	0	0	0	0	0.12	0
GAIF01016161.1	0.15	0.55	0	0	0	0

GAIF01016165.1	0	0	0	0.11	0	0
GAIF01016166.1	16.39	7.13	0	7.61	7.88	3.61
GAIF01016167.1	0.2	0	0.22	0.46	0	0.22
GAIF01016168.1	0	0	0	0.06	0.31	0
GAIF01016170.1	8.31	12.82	21.52	36.78	24.28	9.99
GAIF01016171.1	0.49	0.84	0.06	2.72	5.46	0
GAIF01016172.1	0.18	0.07	0	0.27	1.45	0.12
GAIF01016173.1	13.22	24.32	22.65	55.85	46.03	3.91
GAIF01016176.1	0	0	0	0	0.24	0
GAIF01016177.1	11.35	1.98	1.15	32.11	75.75	3.83
GAIF01016178.1	0	0	0	0.08	0.24	0.04
GAIF01016179.1	344.3	496.14	592.52	321.89	291.31	293.01
GAIF01016180.1	47.6	42.88	28.27	22.53	34.39	5.38
GAIF01016183.1	0.44	0	0	0.08	0.18	0
GAIF01016184.1	0.94	0	0	0	0	0
GAIF01016185.1	0.1	0.22	0.34	0.24	0.12	0
GAIF01016187.1	29.83	20.83	21.57	26.64	21.96	11.73
GAIF01016188.1	42.33	90.8	56.84	154.27	156.47	16.84
GAIF01016191.1	2.7	1.82	0.87	0.22	0.34	0.05
GAIF01016192.1	0.14	0.15	0	0	1.36	0
GAIF01016193.1	0.31	0.17	0.26	0.36	0.28	0.09
GAIF01016194.1	0.24	0	0	0.69	5.15	0.13
GAIF01016195.1	0.12	0.2	0.07	0.43	0.81	0.41
GAIF01016197.1	45.23	51.36	75.58	58.17	24.78	33.09
GAIF01016198.1	0.14	0.39	0	0	0.28	0.09
GAIF01016200.1	1120.08	690.43	349.82	627.39	521.45	453.26
GAIF01016201.1	2.37	2.71	0.21	5.6	5.38	0.49
GAIF01016202.1	41.37	44.19	75.61	123.67	57.13	75.11
GAIF01016203.1	0	0	0	0.07	0.07	0
GAIF01016204.1	0	0	0	0	0.17	0
GAIF01016206.1	1.74	1.34	3.97	1.73	1.52	1.45
GAIF01016207.1	0.23	0	0.04	0.23	0.65	0
GAIF01016209.1	0	0	0	0	1.03	0
GAIF01016210.1	1	0.52	1.82	1.23	0.09	0.9
GAIF01016212.1	1.72	0.84	0.27	0.98	0.12	0.98
GAIF01016213.1	0	0.03	0	0	0	0
GAIF01016214.1	73.92	75.38	60.32	101.12	80.35	27.65
GAIF01016215.1	0	0.62	0	0.45	0.27	0.28
GAIF01016216.1	0.53	0	0	0.31	1.12	4.1
GAIF01016217.1	0.14	0	0.08	0	0	0.08
GAIF01016218.1	0	0	0.41	1.53	0	0.18
GAIF01016219.1	2.73	1.09	0.3	8.67	25.21	2.11
GAIF01016223.1	45.93	70.36	69.51	96.05	50.96	22.27
GAIF01016224.1	0.13	0	0	1.35	6.62	0

GAIF01016226.1	0	0.11	0	0	0	0
GAIF01016227.1	0.02	0.04	0.02	0.04	0.02	0
GAIF01016228.1	3	1.93	0.46	13.13	15.62	9.06
GAIF01016229.1	0	0	0	0	0.24	0
GAIF01016230.1	0.08	0.16	0.08	0.18	0.91	0.04
GAIF01016231.1	85.74	86.11	32.79	171.11	171.91	15.49
GAIF01016233.1	13.1	7.4	6.2	18.64	13.21	8.31
GAIF01016234.1	0	18.27	21.37	0	0	16.84
GAIF01016235.1	0.22	0.12	0.71	0	0.13	0
GAIF01016236.1	0	0	0	0.3	0.42	0
GAIF01016238.1	20.38	19.22	17.33	21.92	24.23	36.44
GAIF01016239.1	0	0	0	0	4.74	0
GAIF01016242.1	0.06	0	0	0	0.21	0
GAIF01016244.1	0.88	0.5	1.06	0.62	1.24	0.76
GAIF01016248.1	11.77	3.12	1.09	9.04	32.75	12.91
GAIF01016252.1	0	0	0	0	0.43	0
GAIF01016253.1	0	0	0.06	0	0	0
GAIF01016255.1	0.41	0.07	0.15	3.01	14.1	1.05
GAIF01016256.1	0.38	0.63	0	4.23	13.61	0.21
GAIF01016257.1	0.93	2.4	3.45	6.41	5.82	0.94
GAIF01016260.1	0.18	0	0	0	0	0
GAIF01016262.1	0.04	0	0	0	0.13	0
GAIF01016263.1	3.46	9.35	1.84	18.54	31.3	0.37
GAIF01016264.1	0	0	0	2.93	14.57	0
GAIF01016265.1	1380.55	1002.8	425.62	902.04	829.03	446.5
GAIF01016266.1	0.54	2.04	2.19	2.39	2.55	0.72
GAIF01016268.1	0.08	0.13	0.07	0	0	0.13
GAIF01016270.1	0	0.3	0	1.12	0.17	0
GAIF01016273.1	0	0	0	0.05	0	0.05
GAIF01016275.1	0	0	0	0.24	1.62	0
GAIF01016276.1	19.63	2.51	2.94	102.02	327.5	976.78
GAIF01016277.1	10.41	1.84	0.59	98.92	493.89	10.2
GAIF01016278.1	0	0	0.11	0.85	0.49	0
GAIF01016279.1	13.67	12.6	5.24	24.58	23.8	10.78
GAIF01016280.1	0.02	0.02	0	0.21	0.4	0
GAIF01016281.1	0.05	0	0	0	0	0
GAIF01016282.1	0.7	0.15	0	15.6	55.92	2.78
GAIF01016284.1	0.07	0	0	0.93	1.79	0
GAIF01016285.1	0.07	0	0	0.21	0.99	0
GAIF01016286.1	54.24	21.75	32.64	1.81	0.44	4.15
GAIF01016289.1	0.04	7.77	0	0	3.84	0
GAIF01016291.1	2.12	3.26	0.84	30.69	64.35	0.31
GAIF01016292.1	0	0.06	0	0.17	2.86	0.13
GAIF01016293.1	7.43	5.29	5.49	14.65	9.25	6.98

GAIF01016295.1	7.22	6.32	3.3	1.63	1	0.07
GAIF01016296.1	231.43	118.08	41.23	389.24	560.95	35.24
GAIF01016297.1	62.96	79.29	59.04	67.36	55.22	27.6
GAIF01016298.1	3.81	4.55	1.76	16.98	49.99	1.36
GAIF01016299.1	23.73	44.37	24.54	26.88	8.55	1.52
GAIF01016300.1	0.45	0	0	0.78	1.16	0.41
GAIF01016301.1	0	0	0	0.51	1.2	0
GAIF01016302.1	0	0	0	0	0.5	0.09
GAIF01016303.1	0	0	0	0	0.04	0
GAIF01016304.1	0.22	0	0	0	0	0
GAIF01016305.1	0.94	0.34	0.4	2.72	6.4	0.23
GAIF01016306.1	19.99	9.74	8.07	8.27	6.91	2.03
GAIF01016307.1	0.6	0.17	0	0.65	1.75	0
GAIF01016308.1	0.14	0	0	0.05	0.08	0
GAIF01016309.1	17.18	12	5.53	27.82	32.91	2.87
GAIF01016311.1	64.87	35.56	24.84	11.51	7.79	1.36
GAIF01016312.1	1.19	0.92	0.15	7.9	20.25	0.08
GAIF01016313.1	0.29	0.2	0.19	0.47	0	0
GAIF01016314.1	8.19	11.07	11.55	6.56	4.3	2.54
GAIF01016315.1	0.14	0.1	0.1	0.65	3.66	0.21
GAIF01016316.1	4.37	0	0	4.57	13.02	15.77
GAIF01016317.1	0	0	0	0	0	0.46
GAIF01016319.1	15.66	15.24	9.34	35.6	23.91	5.44
GAIF01016321.1	1.4	1.42	4.01	3.7	1.98	1.92
GAIF01016323.1	16.73	12.43	12.14	1.11	5.08	2.3
GAIF01016325.1	0	0	0.03	0	0.15	0
GAIF01016326.1	0	0.94	0.32	0	0	0.52
GAIF01016327.1	5.52	7.87	11.43	24.97	15.34	4.41
GAIF01016329.1	0	0.42	1.28	0	0	0
GAIF01016330.1	0.17	0.47	0.1	0	0.21	0.1
GAIF01016332.1	5.45	2.01	0.14	3.21	3.73	1.85
GAIF01016334.1	0	0.23	0	0.12	0.13	0
GAIF01016335.1	0.28	0	0	0.92	3.44	0.23
GAIF01016336.1	0	0	0	0	0.56	0
GAIF01016337.1	0.44	0	1.15	0.3	0.56	0.34
GAIF01016339.1	0.32	0.26	0.18	0.19	0	0
GAIF01016341.1	1.57	0.17	0	0.73	0.57	0
GAIF01016342.1	1.13	1.73	2.09	3.72	0.9	0
GAIF01016343.1	0	0	0	0	0.72	0
GAIF01016344.1	19.36	22.16	17.21	7.12	4.51	0.68
GAIF01016345.1	26.6	48.86	22.27	83.49	77.56	7.09
GAIF01016348.1	6.76	6.93	1.9	12.98	21.16	2.26
GAIF01016350.1	13	15.22	12.55	28.6	18.25	8.97
GAIF01016351.1	1.8	2.58	2.06	3.44	2.42	0.51

GAIF01016352.1	0	0.45	0	0	0	0
GAIF01016353.1	29.23	25.95	17.3	14.14	19.08	5.91
GAIF01016354.1	62.86	24.45	20.05	1.45	2.64	0
GAIF01016355.1	0.11	0.08	0.08	2.38	2.08	0.08
GAIF01016356.1	0	0	0	0.08	0	0
GAIF01016361.1	6.38	19.04	12.76	32.1	21.65	1.98
GAIF01016363.1	0.05	0.06	0	0	0	0.12
GAIF01016364.1	0.12	0	0	0	0.43	0
GAIF01016365.1	1.33	0.87	0	6.67	6.79	2.49
GAIF01016366.1	26.61	16.77	33.3	44.05	32	58.08
GAIF01016367.1	0	0.07	0	0	0	0.07
GAIF01016368.1	11.31	18.53	11.19	43.25	41.15	6.11
GAIF01016371.1	0	0	0	1.29	0.42	0
GAIF01016372.1	0.05	0	0	0	0	0
GAIF01016374.1	0	0.28	0	0	1.5	0
GAIF01016375.1	0.16	0.18	0	0.38	0.39	0
GAIF01016376.1	7.89	0.59	0.24	5.96	36.72	19.21
GAIF01016377.1	34.34	66.16	45.14	3.84	2.3	3.01
GAIF01016379.1	22.29	17.53	16.82	15.25	3.89	3.4
GAIF01016381.1	4.57	1.97	0.52	29.46	91.69	0.77
GAIF01016382.1	18.12	14.97	6.4	24.72	34.42	1.91
GAIF01016384.1	471.24	337.32	106.24	110.47	194.96	25.6
GAIF01016386.1	1.77	0.2	0.16	0.26	0.13	0.49
GAIF01016388.1	834.6	839.93	1533.63	336.58	278.12	1098.46
GAIF01016389.1	3.27	1.89	4.94	6.53	4.43	6.32
GAIF01016393.1	4.03	11.32	0	14.32	35.46	0
GAIF01016394.1	0.05	0.06	0	0.15	0.41	0
GAIF01016395.1	0	3.13	3.44	0	0	0
GAIF01016400.1	0	0	0	0	0	0.05
GAIF01016401.1	0	0	0	0.39	0.14	0
GAIF01016402.1	0.15	0	0	0	0.09	0
GAIF01016403.1	6865.24	1091.75	2278.83	3624.84	743.97	4952.8
GAIF01016405.1	22.5	21.51	13.7	19.39	17.28	4.17
GAIF01016406.1	2.4	0	0.14	6.48	40.92	124.69
GAIF01016408.1	0.14	0.18	0.05	0	0	0
GAIF01016409.1	681.86	688.51	509.65	553.65	971.39	3961.52
GAIF01016410.1	99.21	55.26	26.92	28.43	40.92	31.05
GAIF01016411.1	42.98	37.42	35.45	48.55	36.62	10.25
GAIF01016412.1	0.57	1.37	0	0.39	0.47	0.32
GAIF01016413.1	58.31	84.95	36.51	462.11	234.9	33.51
GAIF01016415.1	10.51	7.4	4.25	23.02	19.64	3.06
GAIF01016416.1	0	0	0	0.29	0.09	0
GAIF01016417.1	2468.57	2280.88	2996.57	1080.02	574.4	466.03
GAIF01016418.1	0.44	0	0.08	1.57	9.26	0.77

GAIF01016420.1	0	0	0	0.07	0.07	0
GAIF01016421.1	0.12	0	0	0.14	0.28	0
GAIF01016422.1	13.02	15.73	10.1	3.01	4.87	0.56
GAIF01016423.1	0.22	0.63	0	0.75	0.52	0.47
GAIF01016424.1	8.94	8.01	5.34	29.84	38.28	2.59
GAIF01016425.1	13.54	14.59	2.89	15.5	23.75	3.04
GAIF01016426.1	319.86	511.26	349.48	375.93	123.32	74.74
GAIF01016427.1	7.25	4.16	5.56	6.19	1.81	2.26
GAIF01016428.1	0.17	0	0.29	0.1	0.42	0.1
GAIF01016429.1	43.12	40.08	10.29	14.14	18.31	2.59
GAIF01016430.1	0	0	0	0.11	0	0
GAIF01016431.1	25.26	21.52	4	39.53	32.95	0.7
GAIF01016433.1	0	0	0	0.2	0.42	0.39
GAIF01016434.1	2.67	2.36	0	7.24	5.36	0.25
GAIF01016435.1	200.77	244.46	88.7	308.38	252.68	68.89
GAIF01016436.1	10.22	9.76	8.76	13.82	5.74	5.7
GAIF01016437.1	2.28	2.96	0.55	0	0	0
GAIF01016438.1	131.68	59.24	25.01	226.51	153.07	6.37
GAIF01016440.1	0.64	0.45	0.07	0.3	1	0.07
GAIF01016443.1	0.16	0.34	0.17	0.54	3.92	0.17
GAIF01016444.1	3.81	5.69	4.25	9.94	16.76	0.82
GAIF01016445.1	0	0.01	0	0	0	0
GAIF01016447.1	0	0.15	0	0	0	0
GAIF01016448.1	10.88	4.25	0.59	21.59	35.7	3.49
GAIF01016450.1	0	0	0	0.19	0	0
GAIF01016452.1	0.47	0	0	0.37	0.69	0
GAIF01016454.1	0.08	0.09	0	0.88	5.75	0
GAIF01016455.1	0	0	0	0.3	0.43	0
GAIF01016456.1	0	4.79	1.77	0	0	0.81
GAIF01016459.1	1.07	0	0	2.48	9.92	0.17
GAIF01016460.1	0.21	0	0.12	0.12	0	0
GAIF01016461.1	240.82	184.12	53.44	121.53	135.58	74.35
GAIF01016463.1	0.3	1.12	0.03	2.74	5.9	0.07
GAIF01016464.1	1.05	1.28	0.14	3.03	4.56	0
GAIF01016465.1	0.35	0.35	0.06	1.89	8.38	0.06
GAIF01016468.1	0	0	0	0	0.42	0
GAIF01016469.1	1.07	0.67	0.5	0.38	0.55	0.17
GAIF01016470.1	0	0.14	0.09	0.91	2.26	0
GAIF01016471.1	217.59	129.94	27.23	59.72	95.18	6.87
GAIF01016472.1	0.36	0.58	0	0.46	0	0.23
GAIF01016475.1	4.69	5.1	4.35	13.42	11.37	2.04
GAIF01016476.1	6.55	7.99	6.52	24.95	14.46	25.32
GAIF01016477.1	1.31	1.54	0.64	3.5	8.71	0.37
GAIF01016478.1	3.82	1.94	1.91	5.7	6.53	1.77

GAIF01016479.1	0	0	0	0	0.22	0
GAIF01016480.1	0	0.07	0.14	0.04	0.27	0
GAIF01016481.1	0	0	0	0.48	1.21	0
GAIF01016483.1	0.38	0	0	0.65	3.43	0.67
GAIF01016486.1	0	0	0	0.4	0.42	0
GAIF01016487.1	3.79	2.9	2.01	0	0	1.7
GAIF01016488.1	0.75	0.8	0.05	5.31	6.44	0.28
GAIF01016489.1	0	0	0	0.09	0	0
GAIF01016490.1	0.17	0.04	0.06	0.16	0.29	0.02
GAIF01016491.1	12.96	5.78	0	48.79	75.84	0.9
GAIF01016493.1	0.53	0.92	0.16	2.82	3.67	0
GAIF01016494.1	0	0	0	0.47	2.62	0
GAIF01016495.1	0	0	0	1.08	0.22	0
GAIF01016496.1	14.55	8.57	4.36	25.72	16.45	2.82
GAIF01016497.1	70.51	51.83	51.01	137.25	86.64	19.81
GAIF01016498.1	0.06	0.03	0.03	0.25	1.32	0.03
GAIF01016499.1	336.52	326.49	656.19	139.52	86.51	253.66
GAIF01016500.1	0.03	0	0.19	0	0	0
GAIF01016501.1	0.54	0	0	0.43	0.22	0.19
GAIF01016502.1	0	0	0	0.05	0.05	0
GAIF01016503.1	1156.51	318.06	753.11	901.69	909.54	8008.38
GAIF01016504.1	3.02	1.12	1.34	1.13	0.88	1.4
GAIF01016505.1	0.04	0	0	0	0	0
GAIF01016507.1	5.26	4.08	3.3	9.3	21.68	11.04
GAIF01016508.1	0.93	1.35	1.19	3.43	1.87	0.51
GAIF01016509.1	0	0	0	0.98	5.26	0.31
GAIF01016510.1	18.32	36.4	35.39	53.56	37.94	6.38
GAIF01016512.1	0	0	0	0.02	0.05	0
GAIF01016513.1	32.72	32.3	20.26	9.65	3.86	2.56
GAIF01016514.1	5.8	3.26	2.36	17.76	24.08	3.48
GAIF01016515.1	8.55	4.79	5.43	18.62	22.32	8.61
GAIF01016516.1	2.3	6.08	1.77	6.34	12.17	0.07
GAIF01016517.1	1.66	0.26	0	3.72	14	1.05
GAIF01016518.1	0	0.38	0	0.69	1.31	0
GAIF01016520.1	0.03	0	0	0.25	0.63	0.03
GAIF01016521.1	0	0	0.22	2.75	12.12	0
GAIF01016522.1	0.77	1.85	1.12	2.6	1.99	0.38
GAIF01016523.1	15.21	19.57	9.43	127.34	98.78	6.88
GAIF01016525.1	51.05	41.05	16.15	23.46	30.81	3.08
GAIF01016526.1	15.29	28.75	16.04	59.36	39.31	5.93
GAIF01016528.1	0	0.15	0	0.76	0.25	0
GAIF01016529.1	0	1.67	0.48	0	0	0.97
GAIF01016530.1	14.61	28.77	38.58	3.56	2.93	4.78
GAIF01016532.1	0	0	0	0.15	0.79	0

GAIF01016534.1	1.02	4.23	0.62	6.05	1.05	0
GAIF01016535.1	151.4	203.34	70.89	49.52	107.5	12.01
GAIF01016536.1	0	0	0	0.51	3.72	0
GAIF01016537.1	0.65	0	0	0.49	0.31	1
GAIF01016538.1	0	0	0	0	0.07	0
GAIF01016539.1	1.73	1.64	0.58	5.15	8.14	0.5
GAIF01016541.1	0.08	0	0	0.09	1.9	0.74
GAIF01016542.1	0	0.13	0.18	0	0	0
GAIF01016543.1	0	0.19	0	0	0	0
GAIF01016544.1	1.09	1.32	1.13	1.01	1.27	0.45
GAIF01016545.1	0.29	0.11	1.08	0.4	0.35	0.32
GAIF01016547.1	0	0	0	0	0	0.35
GAIF01016549.1	13.12	30.6	9.13	67.38	172.4	133.43
GAIF01016550.1	0.02	0	0	0.08	0.86	0
GAIF01016551.1	1.15	1.51	1.11	1.42	1.72	0.99
GAIF01016553.1	16.88	15.48	16.89	26.39	12.76	3.23
GAIF01016554.1	18.83	21.85	7.82	99.16	61.84	6.1
GAIF01016555.1	1.05	0	0.14	3.65	20.32	4.62
GAIF01016557.1	2.01	2.3	0.23	4.54	2.16	0.47
GAIF01016558.1	1.04	0.09	0	3.07	10.62	0
GAIF01016559.1	0.31	0.11	0.11	0.37	0.92	0
GAIF01016561.1	0.52	0.08	0.08	0.39	2.84	1.75
GAIF01016562.1	16.03	17.02	5.7	42.2	48.25	0.77
GAIF01016563.1	0	0	0.57	0	0	0.32
GAIF01016564.1	2.41	3.08	3.1	2.17	2.58	1.94
GAIF01016567.1	0.14	0	0	0	0	0
GAIF01016568.1	0.05	0.06	0	2.07	7.1	0.12
GAIF01016569.1	53.2	4.96	2.9	98.65	429.98	266.96
GAIF01016570.1	0	0	0.05	0.2	1.09	0
GAIF01016571.1	0	0	0	0.22	0.55	0
GAIF01016572.1	11.33	14.57	22.78	48.89	53.42	14.66
GAIF01016574.1	0	8.44	0	0	5.85	10.97
GAIF01016575.1	0.78	0.34	0	0.54	0.38	0
GAIF01016576.1	0	0	0.04	0	0	0
GAIF01016577.1	0	0	0.17	0.12	0	0
GAIF01016580.1	0	0	0	0	0.14	0
GAIF01016585.1	1.79	1.49	1.96	104.03	0.33	0.45
GAIF01016586.1	0	0	0	0.4	0.41	0.08
GAIF01016587.1	9.76	11.44	18.14	14.98	7.35	11.18
GAIF01016588.1	1003.76	101.99	438.05	11.78	3.09	36.4
GAIF01016589.1	16.68	27.22	27.28	32.17	37.98	29.57
GAIF01016591.1	0	0	0	0	0.87	0
GAIF01016592.1	0.3	0	0	0.17	0.54	0.16
GAIF01016593.1	0	0.04	0	0	0	0

GAIF01016594.1	0	0	0	0	0.06	0
GAIF01016595.1	0	0	0	0.07	0	0
GAIF01016599.1	10.91	14.58	18.32	28.7	27.02	8.15
GAIF01016600.1	0	0	0	0	0.2	0
GAIF01016601.1	6.38	4.62	3.57	9.07	12.54	10.22
GAIF01016602.1	0	1.02	49.93	0	10.7	0
GAIF01016603.1	0	0	0	0.5	3.39	0.24
GAIF01016604.1	0	0	0	0.6	2.82	0
GAIF01016606.1	0	0	0	0.13	0	0
GAIF01016607.1	0.41	0.18	0.07	1.53	13.41	0.22
GAIF01016609.1	2.34	0.73	0	11.51	36.46	1.47
GAIF01016610.1	0	0.07	0	0.11	0.8	0.03
GAIF01016611.1	9.4	0	0	12.6	38.65	3.21
GAIF01016612.1	0.09	0.15	0	0.05	0.11	0
GAIF01016614.1	22.99	23.31	2.93	80.8	141.6	2.8
GAIF01016615.1	0	0.55	0.69	0	0	0.07
GAIF01016616.1	0.19	0.13	0	0.9	2.16	0.49
GAIF01016617.1	0.27	0.22	0	0.78	3.42	0.3
GAIF01016618.1	0.68	1.11	0.19	0.47	1.03	0
GAIF01016619.1	3.08	1.61	1.62	3.47	1.52	1.06
GAIF01016621.1	0	0.15	0	0	0	0
GAIF01016624.1	0	0.05	0	0	0	0
GAIF01016626.1	0	1.3	0.88	0	0	0.88
GAIF01016627.1	11.97	24.98	22.53	6.3	7.5	2.25
GAIF01016628.1	0	0	0	0.9	3.74	0
GAIF01016629.1	1.37	0.24	0	7.32	25.39	0.3
GAIF01016630.1	49.68	53.32	37.13	11.14	4.33	1.37
GAIF01016631.1	0.14	0.22	0.07	1.42	3.34	1.57
GAIF01016632.1	15.95	23.54	23.05	31.17	17.62	5.83
GAIF01016634.1	0	0.33	0	0	0.15	0
GAIF01016635.1	0.11	0.18	0.12	0.13	0.26	0
GAIF01016636.1	0	0	0	1.47	6.9	0
GAIF01016637.1	0	0	0	0	1.64	0
GAIF01016639.1	1.58	0	0	0.58	0	0
GAIF01016640.1	0	0	0	0	0.05	0
GAIF01016641.1	1.08	0.78	0.2	3.55	13.08	0.2
GAIF01016642.1	9.15	16.73	21.55	35.86	33.33	7.45
GAIF01016643.1	0.19	0	0	0	0.11	0
GAIF01016644.1	6.96	3.47	1.36	24.56	17.57	5.15
GAIF01016646.1	6.36	20.98	25.81	26.24	26.41	18.87
GAIF01016647.1	0	0	0.19	0	0	0
GAIF01016652.1	6.99	0.25	0.49	3.53	8.4	82.95
GAIF01016653.1	0.23	0	0	0	0.35	0.06
GAIF01016654.1	0.23	0	0	0.5	3.37	0

GAIF01016655.1	7.02	4.84	3.38	14.5	9.88	4.71
GAIF01016656.1	0	0	0	0	0.31	0
GAIF01016658.1	2.54	0.54	0.07	6.41	25.81	1.5
GAIF01016660.1	0	0	0	0	0	0.15
GAIF01016661.1	0	0	0	0	0.08	0
GAIF01016662.1	41.62	13.15	13.86	47.84	45.49	35.78
GAIF01016663.1	16.45	12.59	0.74	65.64	95.87	0.37
GAIF01016664.1	0.05	0	0.11	0.06	0.93	0.11
GAIF01016665.1	69.79	97.56	21.66	145.92	118.49	5.74
GAIF01016670.1	2.43	1.16	4.74	2.42	0.49	0.73
GAIF01016673.1	0.69	0.41	0.28	3.36	20.36	0.28
GAIF01016674.1	0.01	0	0.32	0.04	0	0
GAIF01016675.1	60.59	42.45	28.52	61.18	101.55	153.11
GAIF01016676.1	196.68	399.19	259.11	365.15	295.39	14.45
GAIF01016678.1	0.49	0.93	0.93	0.56	0.22	0.34
GAIF01016680.1	0.56	0	0	1.47	4.41	0
GAIF01016681.1	0	0.04	0	0	0.44	0
GAIF01016682.1	0.65	0	0	0.32	0.93	0
GAIF01016683.1	0.43	0	0	1.98	12.32	0.31
GAIF01016684.1	0.46	0.12	0.18	0.57	3.22	0.9
GAIF01016686.1	0	0	0	0.19	3.35	0.18
GAIF01016687.1	0.07	0.08	0	0	0	0
GAIF01016691.1	2.58	1.82	1.5	3	1.74	1.51
GAIF01016695.1	0	0	0.14	0.86	1.63	0
GAIF01016697.1	6.2	6.88	5.65	0.43	0.3	0.55
GAIF01016698.1	64.91	104.83	129.36	94.99	47.7	43.78
GAIF01016699.1	30	11.49	0.5	27.37	40.97	42.28
GAIF01016700.1	2.3	2.31	2.38	1.69	2.51	4.66
GAIF01016701.1	17.66	11.66	10.81	54.23	87.4	8.57
GAIF01016702.1	0	0	0	0.03	0.03	0
GAIF01016704.1	0.43	0.15	0.04	0.08	0	0.26
GAIF01016705.1	9.08	12	6.49	0	4.48	1.72
GAIF01016706.1	0	0	0	0.13	0	0
GAIF01016710.1	0	0	0	0	78.17	15.95
GAIF01016711.1	0	0	0	0.04	0.04	0
GAIF01016712.1	57.31	82.53	117.98	82.77	43.62	48.26
GAIF01016713.1	0.32	0.35	0	1.11	3.24	0
GAIF01016715.1	3.79	1.12	0.6	6.69	21.03	64.02
GAIF01016716.1	5.48	2.83	5.34	8.11	5.03	19.93
GAIF01016717.1	0	0.42	0.63	0	0	0
GAIF01016719.1	1.47	0.73	1.47	0.31	0.16	0
GAIF01016720.1	1.32	0.5	0.15	3	0.37	0.7
GAIF01016721.1	0	0	0	0.53	1.93	0.13
GAIF01016722.1	20.5	13.88	21.03	28.21	29.36	18.52

GAIF01016723.1	1.06	0.16	0.11	0	1.78	0.23
GAIF01016724.1	0.05	0.06	0	0.25	0.92	0.06
GAIF01016727.1	7.63	9.54	6.76	12.69	9.96	1.8
GAIF01016728.1	2.45	0	0.6	6.76	5.11	8.1
GAIF01016729.1	0.12	0.16	0.29	3.69	0.57	0.36
GAIF01016730.1	23.55	23.37	20.87	9.33	9.49	5.63
GAIF01016731.1	0.03	0.03	0	0	0.17	0
GAIF01016732.1	0	0.01	0	0.05	0	0
GAIF01016733.1	0	0	0	0	0.09	0
GAIF01016735.1	0	0	0	0.13	0.07	0
GAIF01016738.1	10.98	5.58	6.77	28.07	32.03	18.71
GAIF01016739.1	53.45	32.92	22.31	92.74	40.68	7.43
GAIF01016740.1	4.89	4.7	7.19	5.58	2.56	3.72
GAIF01016741.1	11.46	14.37	20.82	40.07	49.31	15.41
GAIF01016745.1	0	0	0	0.95	2.94	0
GAIF01016746.1	3.13	1.13	0.34	16.68	42.34	0.57
GAIF01016747.1	0.69	0.09	0	7.48	18.28	0.8
GAIF01016748.1	78.67	42.77	43.19	45.65	78.84	72.25
GAIF01016749.1	1.55	0.57	0.41	13.2	23.41	0.36
GAIF01016751.1	0	0	0	0	0.21	0
GAIF01016752.1	14.86	10.31	21.45	15.29	10.72	11.01
GAIF01016754.1	63.85	83.79	116.8	74.41	50.1	54.06
GAIF01016755.1	29.06	12.67	22.27	30.39	27.49	64.72
GAIF01016756.1	0.05	0	0	0.19	0	0.05
GAIF01016757.1	7.01	2.92	0	1.23	1.38	7.62
GAIF01016758.1	0.04	0	0	0.09	0.09	0
GAIF01016759.1	5.85	7.62	4.7	5.26	3.67	0.45
GAIF01016760.1	7.18	11.82	18.81	47.99	41.42	8.22
GAIF01016761.1	0.82	1.95	0.32	0.48	0.33	0.03
GAIF01016762.1	0.08	0.04	0.09	0.86	3.11	0.39
GAIF01016765.1	0	0	0	0.52	5.59	0
GAIF01016766.1	0	0.03	0	0	0	0
GAIF01016768.1	10.2	9.42	10.18	22.59	14.61	3.32
GAIF01016769.1	0.77	0.34	0.11	0.36	0.69	0
GAIF01016770.1	10.4	26.63	38.75	135.02	75.63	39.38
GAIF01016771.1	0.08	0	0	0	0.41	0
GAIF01016772.1	37.71	7.36	5.75	9.26	4.37	1.36
GAIF01016773.1	0	0	0.12	0	1.13	0
GAIF01016775.1	0	0	0	0.32	0.82	0.05
GAIF01016776.1	61.89	407.33	84.79	0.82	2.35	0.29
GAIF01016777.1	0.95	1.49	0.46	10.99	18.85	0.58
GAIF01016778.1	1.34	6.52	5.33	1.56	1.12	0
GAIF01016779.1	3.85	3.92	3.8	0	1.6	0.65
GAIF01016780.1	0	0.09	0	0	0	0

GAIF01016781.1	174.56	196.61	79.18	19.18	24.23	7.4
GAIF01016783.1	0	0	0	0	0.04	0
GAIF01016785.1	4609.67	3022.12	4903.56	2496.96	2330.87	10021.67
GAIF01016787.1	0.13	0	0.15	0.15	0	0
GAIF01016788.1	0.21	0.13	0.17	1.38	0.78	0.64
GAIF01016791.1	1.5	2.29	0.49	0.39	0.81	0
GAIF01016792.1	0	0	0	0	0.29	0.13
GAIF01016794.1	2.72	0.12	1.01	0	0.48	17.71
GAIF01016798.1	7.01	6.34	2.19	12.77	25.74	12.04
GAIF01016800.1	12.22	14.37	10.79	24.5	23.9	9.68
GAIF01016801.1	8.7	7.04	14.23	3.05	1.13	3.37
GAIF01016802.1	2.76	2.83	0.41	2.57	1.34	1.2
GAIF01016803.1	32.44	27.95	38.87	38.1	60.91	55.11
GAIF01016804.1	1.44	0.63	0.82	9.62	19.24	0.25
GAIF01016805.1	3.66	3.13	3.63	6.45	7.65	1.23
GAIF01016806.1	0.11	0.13	0	0.02	0	0.03
GAIF01016807.1	0.1	0	0	0.23	0.12	0
GAIF01016809.1	0	0	0	0	0.11	0
GAIF01016810.1	18.05	11.65	14.7	19.79	10.7	8.17
GAIF01016811.1	0	0	0	0.02	0	0
GAIF01016812.1	0	0	0	0	0.1	0
GAIF01016813.1	1.09	2.62	2.39	4.76	2.32	0.13
GAIF01016814.1	0	0	0	0.07	0.67	0
GAIF01016815.1	0.68	1.3	0.56	3.75	5.01	0.28
GAIF01016816.1	30.51	22.9	55.82	52.94	16.99	64.45
GAIF01016817.1	0.05	0	0	0	0.37	0.11
GAIF01016819.1	0.25	0.24	0.24	0.72	0	0
GAIF01016820.1	4.14	2.64	3.66	4.28	3.68	2.28
GAIF01016822.1	0.69	1.15	1.09	0.42	0.56	0.04
GAIF01016823.1	18.82	41.85	12.33	41.58	73.08	1.47
GAIF01016824.1	27.41	29.39	34.56	56.42	32.61	34.22
GAIF01016825.1	0.63	0	0	0	0.38	0
GAIF01016826.1	0	0	0	0	0.34	0
GAIF01016827.1	36.81	17.6	13.72	93.3	118.2	34.84
GAIF01016828.1	0	0.04	0	0	0	0
GAIF01016831.1	12.9	18	17.41	28.23	19.81	4.57
GAIF01016832.1	0.25	0.27	0	0.86	2.81	0.81
GAIF01016833.1	7.01	4.24	3.81	14.19	18.55	2.52
GAIF01016834.1	21.03	14.07	15.48	15.08	11.85	8.19
GAIF01016836.1	6.54	4.39	10.25	4.43	6.64	2.02
GAIF01016837.1	0.04	0	0	0.46	0.69	0
GAIF01016838.1	7.37	6.82	10.22	3.73	4.13	6.63
GAIF01016840.1	0	0	0	0.59	6.07	0.49
GAIF01016841.1	0	0	0.13	0	0	0

GAIF01016842.1	97.1	42.86	56.1	65.2	34.08	31.05
GAIF01016843.1	0	0	0	0	0	0.14
GAIF01016844.1	23.75	22.16	25.12	51.01	46.36	27.55
GAIF01016845.1	0.42	0	0	0.39	2.1	0
GAIF01016846.1	0	0.05	0	0.76	1.36	0
GAIF01016848.1	0.45	0.1	0.2	0.31	0.54	0.99
GAIF01016849.1	1.1	2.23	0.35	3.11	3.6	0.35
GAIF01016851.1	0.5	0.19	0.07	0.2	0.36	0
GAIF01016853.1	0	0	0	0	0.15	0
GAIF01016854.1	2.25	2.31	1.12	8.67	18.11	0.54
GAIF01016855.1	0	0	0	0	0.19	0
GAIF01016857.1	0.14	0.3	0.15	0	1	0
GAIF01016858.1	1.09	0.26	0	0.27	0.26	0.61
GAIF01016862.1	0.05	0	0	0	0	0
GAIF01016863.1	0.25	0.21	0.07	0.73	1.44	3.15
GAIF01016864.1	0	0	0	0	0	0.14
GAIF01016865.1	0.61	1.37	0.59	3.55	8.54	0
GAIF01016868.1	0.05	0	0	0.3	0.56	0
GAIF01016869.1	0.86	0.33	0.04	3.27	9.7	0.38
GAIF01016870.1	20.02	6.15	8.01	28.01	58.05	235.93
GAIF01016875.1	0.08	0.04	0	0.28	0.96	0.04
GAIF01016876.1	0.72	1.21	0.96	0.18	0.67	0.96
GAIF01016877.1	14.23	4.23	0.51	10.65	5.8	2.31
GAIF01016879.1	0	0	0	0	0.07	0
GAIF01016880.1	0.28	0.12	0.06	0.17	0.64	0.67
GAIF01016881.1	0	0	0	0	0.16	0
GAIF01016882.1	0.19	0.1	0.1	0.22	0.57	0
GAIF01016883.1	0	0.17	0	0	0	0
GAIF01016884.1	0	0	0	0	0	1.34
GAIF01016885.1	0.17	0.11	0.11	0	0.06	0
GAIF01016887.1	0.03	0.01	0	0	0	0
GAIF01016888.1	5.1	5.91	2.25	1.46	0.6	0.18
GAIF01016890.1	1.17	1.84	2.14	3.47	4.69	0.29
GAIF01016891.1	0.17	0.36	0.09	1.46	8.46	0.18
GAIF01016892.1	0.18	0.19	0.2	0.41	0.43	0
GAIF01016893.1	1.86	0.51	0.74	6.95	21.61	0.4
GAIF01016896.1	0.28	0.6	0.3	1.05	2.29	0.27
GAIF01016897.1	341.46	364.11	220.42	419.13	374.24	296.38
GAIF01016899.1	36.48	59.62	33.59	95.27	98.61	15.43
GAIF01016900.1	0.08	0.3	0.02	0.02	0	0
GAIF01016902.1	0.01	0.28	0.1	0.1	0.72	0.03
GAIF01016903.1	0.44	0	0	2.23	4.45	0.98
GAIF01016904.1	0.65	0.64	1.63	0.77	0.35	0.91
GAIF01016906.1	16	17.6	21.16	5.28	13.92	8.9

GAIF01016907.1	0	0.11	0	0.61	1.39	0
GAIF01016909.1	0	0	0	0	1.02	0
GAIF01016910.1	0.14	0	0	1.91	6.1	0
GAIF01016912.1	0.5	0.57	0.76	0.81	2.01	0.36
GAIF01016913.1	0.27	0.16	0	0.56	2.22	0.24
GAIF01016916.1	15.8	17.49	11.41	25.44	12.15	2.2
GAIF01016917.1	0	0	0	0	0.51	0.87
GAIF01016918.1	0	0.15	0	0	0.17	0
GAIF01016919.1	1.05	0.04	0	3.52	9.66	0.83
GAIF01016920.1	0.41	0	0	0.96	0	0
GAIF01016921.1	0	0	1.16	1.65	0.14	0
GAIF01016922.1	12.93	8.19	7.14	17.81	22.81	11.82
GAIF01016923.1	9.06	17.09	0	88.56	56.33	0
GAIF01016924.1	0.04	0	0	0	0.05	0
GAIF01016926.1	28.17	28.98	20.59	115.43	131.2	14.23
GAIF01016927.1	0.07	0.15	0.08	0.1	0.04	0
GAIF01016928.1	0.41	0	0	0	0	0
GAIF01016929.1	0	0	0	0	0.07	0
GAIF01016932.1	11.08	7.61	7.99	15.1	28.57	13.36
GAIF01016933.1	0	0	0	0.16	0	0
GAIF01016934.1	0	0	0	0	0.33	0
GAIF01016935.1	0.07	0	0	0.21	0.31	0
GAIF01016936.1	0.23	0.37	0.22	0.86	0.54	0.11
GAIF01016938.1	0.11	0	0	0.13	0	0
GAIF01016939.1	0	0.1	0	0.88	1.24	0.21
GAIF01016940.1	7.88	18.84	17.21	11.16	9.79	2.1
GAIF01016941.1	114.02	79.48	179.98	91.56	34.09	94.68
GAIF01016942.1	0.46	0.35	0.24	0.25	0.3	0.02
GAIF01016943.1	0.06	0	0	0	0.08	0
GAIF01016944.1	0.14	0	0	0	0	0.31
GAIF01016946.1	223.27	131.67	176.71	92.8	74.98	493.97
GAIF01016947.1	1.58	0.67	0.57	2.22	3.31	0.43
GAIF01016948.1	160.72	38.22	31.93	246.2	249.31	190.49
GAIF01016949.1	0.41	0.32	0	0.14	0.37	0
GAIF01016950.1	0.1	0	0	0.12	0.12	0
GAIF01016951.1	52.03	30.57	36.59	80.26	108.22	137.96
GAIF01016954.1	176.86	196.37	86.19	149.47	119.35	63.51
GAIF01016955.1	0.02	0	0.02	0.08	0.36	0.17
GAIF01016956.1	0	0.12	0	0	0	0
GAIF01016957.1	1.86	0.88	0.33	2.57	4.18	0.22
GAIF01016958.1	0	0	0	0.53	0	0
GAIF01016961.1	0	2.51	2.53	0	0.08	0.15
GAIF01016962.1	0.96	0.24	0.17	1.27	2.59	0
GAIF01016964.1	9.51	11.6	5.01	39.6	38.31	1.44

GAIF01016967.1	0	0	0	0	0.09	0
GAIF01016968.1	0.44	0.12	0	1.03	2.14	0.12
GAIF01016971.1	0.27	0	0	1.51	0	0
GAIF01016975.1	108.85	127.79	211.83	73.39	57.27	83.47
GAIF01016977.1	1.16	0.36	0.06	0.81	0.56	0
GAIF01016978.1	6.56	22.41	12.97	10.91	19.58	1.18
GAIF01016979.1	0.01	0	0	0	0.15	0
GAIF01016980.1	0	0	0	0	0.09	0
GAIF01016981.1	0	0	0	0.37	1.27	0
GAIF01016982.1	0	0	0	0.29	3.97	0.28
GAIF01016983.1	2.2	0.9	0.8	1.53	3.37	0
GAIF01016984.1	0	0	0	0	0.09	0
GAIF01016985.1	0.34	0.47	0.07	0	0.01	0.01
GAIF01016986.1	0.13	0.43	0	0.61	0.64	0
GAIF01016988.1	14.96	12.92	21.42	34.73	10.94	23.77
GAIF01016989.1	0	0	0.11	0	0.31	0
GAIF01016990.1	0.81	0	0	0.46	3.11	0
GAIF01016991.1	4.01	5.42	3.8	0.24	5.95	1.14
GAIF01016992.1	17.09	7.75	2.66	56.47	48.65	19.65
GAIF01016996.1	0.09	0	0	0	0	0
GAIF01016997.1	0	0	0	0	0.25	0
GAIF01016998.1	0.04	0.08	0.08	0.09	0.22	0
GAIF01017001.1	0	0.19	0	1	3.22	0
GAIF01017002.1	0	0	0	0.05	0.15	0
GAIF01017003.1	133.98	479.06	256.22	118.69	114.18	104.62
GAIF01017004.1	0	0	0	0.28	0.14	0
GAIF01017006.1	0	0	0	0.66	2.23	0
GAIF01017007.1	0.26	0.06	0	0.53	1.04	0.06
GAIF01017008.1	2.68	6.42	3.3	13.71	15.49	2.37
GAIF01017009.1	2.61	2.69	0.67	7.75	16.88	0.22
GAIF01017010.1	0.29	0.21	0	4.71	11.39	0.53
GAIF01017011.1	0.76	0.18	0	1.28	4.78	0.09
GAIF01017012.1	0	0	0	0	0.34	0
GAIF01017013.1	0	0	0	0	0.51	0
GAIF01017015.1	0	0	0.16	0	0	0
GAIF01017016.1	12.28	18.56	14.29	18.69	13.67	5.07
GAIF01017017.1	14.23	7.84	11.05	56.83	83.2	21.58
GAIF01017018.1	24.2	12.59	19.49	0.53	0.16	0.36
GAIF01017019.1	0	0	0	0	0.07	0
GAIF01017020.1	1.22	0.73	0.2	3.9	25.83	0.4
GAIF01017021.1	74.46	19.73	29.4	65.79	52.33	15.52
GAIF01017022.1	0	0.3	0.56	0.37	0	0
GAIF01017023.1	2.73	2.56	0	0.41	0	0
GAIF01017024.1	64.33	54.23	86.77	192.75	345.49	148.03

GAIF01017026.1	0	0.55	0.13	0.02	0.07	0.14
GAIF01017027.1	0.12	0	0	0	0.8	0.13
GAIF01017028.1	0.62	0.44	0.14	0.29	3.21	0.14
GAIF01017029.1	5.21	5.59	5.31	26.96	23.37	2.63
GAIF01017030.1	7.25	2.19	1.57	13.92	13.95	2.84
GAIF01017031.1	0.82	1.28	0.17	6.84	6.48	0.93
GAIF01017033.1	0.54	0.8	0.97	0.63	0.71	0.87
GAIF01017034.1	8.11	7.66	5.74	36.12	34.54	2.24
GAIF01017035.1	0	0.31	0	0	0.92	0
GAIF01017036.1	41.67	27	29.92	64.69	62.36	75.52
GAIF01017038.1	103.58	56.55	96.77	176.28	222.31	265.8
GAIF01017039.1	0	0	0.04	0	0	0
GAIF01017040.1	0	0	0.04	0.05	0	0
GAIF01017041.1	0	0	0.06	0.06	0.13	0.06
GAIF01017042.1	1.77	2.6	1.02	0.5	1.93	0.42
GAIF01017043.1	0.3	0.44	0.22	0	0.12	0
GAIF01017044.1	16.94	27.32	26.3	68.6	84.06	7.69
GAIF01017046.1	3.7	1.28	2.22	10.75	10.93	1.48
GAIF01017048.1	32.95	23.71	35.61	89.86	87.93	44.49
GAIF01017050.1	0.13	0	0.07	0.66	1.99	0.63
GAIF01017051.1	4.83	17.48	8.8	1.85	3.23	1.31
GAIF01017053.1	0	0	0	0	0.06	0
GAIF01017054.1	70.9	62.71	61.84	196.19	88.77	67.79
GAIF01017055.1	12.22	22.83	14.25	9.64	11.02	1.47
GAIF01017056.1	9.77	4.78	8.42	21.78	12.97	4.88
GAIF01017057.1	0.04	0.05	0	0.51	3.17	0.05
GAIF01017058.1	0.07	0	0	0.08	0.16	0
GAIF01017059.1	4.2	4.9	6.64	1.94	0.56	1.98
GAIF01017060.1	0.04	0.13	0	0	0	0
GAIF01017061.1	9.3	5.19	8.41	4.96	3.02	1.94
GAIF01017062.1	0.81	0.28	0.19	0.7	16.78	0.62
GAIF01017063.1	0.08	0.08	0	0.44	0.91	0.08
GAIF01017064.1	0.47	0.34	0.34	0.72	0.19	0
GAIF01017065.1	6.1	5.51	6.32	12.36	9.5	6.63
GAIF01017068.1	0	0	0	0.29	1.17	0
GAIF01017069.1	0.85	1.07	0.86	0.97	1.03	0.36
GAIF01017070.1	0.08	0	0	0.04	0.49	0
GAIF01017071.1	0.08	0	0	0	1.41	0
GAIF01017072.1	0	0	0	0.05	0	0
GAIF01017074.1	52.27	26.64	18.23	5	2.28	7.73
GAIF01017077.1	2.09	1.15	0.84	0.59	0.69	0.32
GAIF01017078.1	0	0	0	0	0.34	0
GAIF01017079.1	12.89	27.07	45.23	7.76	7.91	18.32
GAIF01017080.1	0	0	0.02	0	0	0

GAIF01017081.1	0.43	0.06	0	0.75	2.66	0
GAIF01017082.1	0	0	0	0.07	0.07	0
GAIF01017084.1	0.32	0	0	1.87	4.94	0
GAIF01017085.1	1.9	3.31	2.85	3.24	8.54	0.28
GAIF01017087.1	0.56	0.42	0.46	0.7	0.45	0.3
GAIF01017089.1	0	0	0	0.04	0.12	0
GAIF01017090.1	3.61	1.41	1.66	13.28	20.84	3.8
GAIF01017092.1	0.14	0	0	0.28	0.67	0
GAIF01017094.1	18.51	39.37	19.54	4.02	3.5	0.42
GAIF01017095.1	0.09	0.39	0	0	0	0
GAIF01017097.1	0	0	0	0	0.07	0
GAIF01017098.1	0.08	0	0	0.1	0.5	0
GAIF01017099.1	0.04	0	0	0	0	0
GAIF01017102.1	0	0.32	0.16	0	0	0
GAIF01017103.1	9.63	10.33	12.35	0.29	0.15	0
GAIF01017104.1	0	0	0	0	0.16	0
GAIF01017105.1	0	0	0	0.13	0.51	0
GAIF01017107.1	1.9	1.72	2.58	3.49	2.75	2.8
GAIF01017108.1	0	0	0	0.11	0.23	0.11
GAIF01017109.1	0.07	0.45	0.9	0	0.75	0
GAIF01017110.1	0.25	0	0	0.28	1.18	0
GAIF01017111.1	0	0	0	0	0.7	0
GAIF01017112.1	5.73	6.55	6.71	34.51	15.21	5.65
GAIF01017114.1	0.12	0.25	0.26	0.27	0.28	0
GAIF01017115.1	22.31	13.08	14.58	36.07	23.96	10.5
GAIF01017116.1	20.82	35.1	14.36	59.34	86.3	3.91
GAIF01017118.1	2.23	0.49	0.16	6.91	23.44	6.4
GAIF01017119.1	7.42	5.17	8.91	15.74	6.97	23.63
GAIF01017120.1	1.93	0.3	0	5.5	30.18	10.3
GAIF01017122.1	0	5.46	1.58	0	0	0.25
GAIF01017124.1	21.02	8.85	1.25	2.54	0.37	0.1
GAIF01017125.1	0	0.07	0.07	0.23	1.22	0
GAIF01017126.1	25	41.12	66.24	37.74	27.62	7.19
GAIF01017127.1	4.65	14.9	0	0	0	23.45
GAIF01017129.1	4.11	4.31	1.74	18.88	40.56	3.09
GAIF01017131.1	0.5	0.32	0.66	0.12	0	0
GAIF01017132.1	9.36	11.68	12.8	21.82	15.96	9.98
GAIF01017134.1	0.07	0.15	0.08	0.25	0.08	0.23
GAIF01017135.1	0	0	0	0.4	0.31	0
GAIF01017137.1	0.2	0.37	0.15	4.81	23.84	1.12
GAIF01017138.1	0.09	0.26	0	0.27	0	0
GAIF01017141.1	0.1	0.74	0.1	0.22	2.96	0.05
GAIF01017142.1	3.15	6.11	3.16	8.67	8.32	0.83
GAIF01017143.1	0.1	0.04	0	0	0.18	0

GAIF01017145.1	0	0	0	0	0.09	0
GAIF01017147.1	0.66	0.18	0.54	0.48	0.59	0.27
GAIF01017148.1	13.85	11.93	7.08	7.14	29.57	43.09
GAIF01017149.1	4.06	3.75	0.51	10.73	14.94	0.13
GAIF01017150.1	6.12	1.14	0.88	5.6	17.78	6.99
GAIF01017151.1	0	0.1	0	0	0.22	0
GAIF01017152.1	1031.54	294.93	432.61	803.08	1259.91	3396.16
GAIF01017153.1	0.08	0.05	0	0.45	1.3	0.09
GAIF01017158.1	0	0	0	0.49	1.89	0.75
GAIF01017159.1	14.68	20.99	15.12	64.55	39.15	5.3
GAIF01017160.1	0	0	0	0.49	0.34	0
GAIF01017162.1	138.25	110.61	74.22	149.77	234.99	7.98
GAIF01017163.1	10.69	5.23	6.55	14.3	17.56	29.24
GAIF01017166.1	14.03	6.92	6.42	26.7	43.36	26.03
GAIF01017168.1	0	0	0	0.04	0.23	0
GAIF01017170.1	0	0.17	0	0	0	0
GAIF01017171.1	29.52	0	13.85	97.04	20.38	49.02
GAIF01017172.1	53.08	54.9	25.23	99.12	139.31	78.3
GAIF01017173.1	1.19	1.25	0.26	6.4	13.35	0.39
GAIF01017174.1	0.14	0.3	0.76	1.29	0.5	0.46
GAIF01017178.1	0.88	0.89	0.41	0.45	1.48	0.09
GAIF01017179.1	0	0	0	0.07	0.55	0
GAIF01017180.1	0	0	0	0	0.14	0
GAIF01017181.1	9.77	9.95	11.88	9.52	5.85	7.52
GAIF01017184.1	0	0	0	0.12	0.12	0
GAIF01017185.1	0	0	0.08	0	0	0
GAIF01017186.1	0	0	0	0.02	0	0
GAIF01017187.1	0.06	0	0	0	0	0
GAIF01017188.1	0.31	0.33	0.91	1.73	5.11	0.63
GAIF01017189.1	0	0	0	0	0	0.09
GAIF01017190.1	84.04	29.45	30.74	51.75	72.16	51.39
GAIF01017191.1	77.67	53.22	23.78	18.2	13.8	4.12
GAIF01017192.1	0	0.14	0	0	0	0
GAIF01017193.1	0.57	0.53	0.62	1.41	0.68	0.36
GAIF01017197.1	21.28	7.72	4.6	23.79	16.45	0.03
GAIF01017198.1	0.21	0.08	0.08	0	0.75	0
GAIF01017199.1	1.38	2.33	0.76	4	1.47	0.59
GAIF01017200.1	77.9	241.97	102.03	149.88	141.6	2.88
GAIF01017202.1	0	0	0.09	0	0.34	0
GAIF01017203.1	0.17	0.07	0.06	1.29	6.21	0.15
GAIF01017204.1	11.22	8.64	7.39	28.66	21.91	1.9
GAIF01017206.1	0.17	0.11	0.12	0	1.81	0.17
GAIF01017209.1	77.23	33.77	44.19	130.98	156.55	80.93
GAIF01017211.1	60.3	50.21	62.59	23.1	19.37	84.86

GAIF01017214.1	1.34	0.38	0.16	0.66	0.26	0.16
GAIF01017215.1	30.2	44.69	31.78	27.2	14.62	7.32
GAIF01017217.1	8.66	6.73	9.05	17.3	13.21	9
GAIF01017219.1	1.22	5.32	1.7	0.88	4.01	0
GAIF01017220.1	0.4	1.15	1.08	2.82	0.33	0.61
GAIF01017221.1	0	0	0	0	0.08	0
GAIF01017223.1	0.3	0	0	1.17	0.85	0.11
GAIF01017224.1	0.58	0.79	0.95	0	0	0.16
GAIF01017225.1	0	0	0	0.76	1.57	0.24
GAIF01017226.1	10.55	6.99	1.86	42.5	54.83	2
GAIF01017228.1	0	0.08	0	0	0.09	0.19
GAIF01017230.1	133.92	243.83	200.88	0	14.79	194.23
GAIF01017231.1	0.11	0	0.12	1.82	11.07	0.25
GAIF01017232.1	7.54	4.34	6.03	6.78	5.66	5.07
GAIF01017233.1	0	0.72	0.62	0	0	0
GAIF01017235.1	768.55	700.15	619.27	168.96	85.51	38.36
GAIF01017236.1	0.42	0	0.25	1.55	6.24	1.34
GAIF01017237.1	63.75	112.65	59.09	226.85	122.61	17.41
GAIF01017238.1	0	4.04	4.19	8.25	4.17	0
GAIF01017239.1	36.44	49.27	43.51	73.66	27.83	18.33
GAIF01017240.1	0	0	0	0	0.1	0
GAIF01017241.1	3.53	3.21	5.22	1.09	0.26	1.35
GAIF01017242.1	381.88	573.55	441.22	235.55	199.08	143.62
GAIF01017244.1	0.17	0.06	0.01	1.33	3.82	0.37
GAIF01017246.1	0.06	0	0	0.07	0.69	0
GAIF01017248.1	0.22	0.1	0.88	0	0.11	0
GAIF01017250.1	15.88	16.29	11.17	42.01	41.09	7.56
GAIF01017251.1	0	0	0	0.07	0	0
GAIF01017254.1	62	62.94	58.9	134.34	101.3	34.04
GAIF01017255.1	0	0.34	0.69	0	0.13	2.31
GAIF01017256.1	0.32	0	0	0.07	1.3	0.14
GAIF01017258.1	12.11	10.19	22.08	29.18	14.84	19.9
GAIF01017259.1	0	0	0	0.63	2.44	0
GAIF01017260.1	26.6	18.8	32.26	25.89	21.11	50.94
GAIF01017262.1	8.61	6.08	7.33	21.52	14.99	11.66
GAIF01017263.1	5.36	1.73	2.92	8.11	7.37	4.78
GAIF01017264.1	12.3	4.46	2.8	26.74	24.91	3.05
GAIF01017267.1	0	0.11	0.34	0	0	0.11
GAIF01017270.1	20.75	41.79	24.28	32.53	28.66	8.85
GAIF01017271.1	3211.34	3336.5	5657.61	2104.18	1660.96	5300.65
GAIF01017272.1	0	0	0	0.21	0.27	0
GAIF01017273.1	0	0	0.09	0	0	0
GAIF01017275.1	11.72	10.2	13.19	24.46	16.82	12.8
GAIF01017276.1	3.05	1.99	1.74	10.6	4.69	0.94

GAIF01017277.1	0.04	0	0	0.04	0	0
GAIF01017281.1	17.44	15.3	12.88	76.48	40.98	24.76
GAIF01017282.1	0	0	0.14	0	0	0
GAIF01017283.1	0.58	0.06	0	1.56	4.8	0.06
GAIF01017284.1	716.79	183.45	195.75	66.76	80.4	49.15
GAIF01017285.1	0.23	0.25	0	0.13	0.14	0.25
GAIF01017286.1	10.08	12.25	8.3	21.18	14.88	2.23
GAIF01017288.1	0.95	1.47	0.44	0.63	4.06	0
GAIF01017292.1	1.67	1.32	0.4	7.56	13.39	1.92
GAIF01017293.1	0.08	0	0.17	0	0	0.08
GAIF01017295.1	41.42	55.32	53.97	123.52	51.81	29.24
GAIF01017298.1	0.22	0.08	0.2	0.74	4.75	0.08
GAIF01017299.1	0.34	0.3	0.31	0.47	0.51	0
GAIF01017300.1	4.02	4.79	7.44	5.31	12.87	4.47
GAIF01017301.1	21.3	35.15	47.72	5.03	5.81	2.59
GAIF01017302.1	0.21	0	0	0.02	1.71	0
GAIF01017303.1	67.79	95.47	119.18	137.42	102.27	54.44
GAIF01017304.1	0.29	0.11	0	1.13	4.27	0.05
GAIF01017306.1	19.56	20.98	21.67	19.33	10.54	8.45
GAIF01017309.1	22.34	38.1	23.18	35.52	18.86	2.61
GAIF01017310.1	0	0.11	0	0.26	1.26	0
GAIF01017312.1	2.41	0.62	0.38	1.46	0.69	0.13
GAIF01017313.1	1.71	0.92	0.12	5.54	0.45	0.53
GAIF01017315.1	0	0	0	0.1	0	0
GAIF01017316.1	20.82	23.6	24.38	60.7	26.34	13.81
GAIF01017319.1	0.98	0	0.12	1.14	0.76	0
GAIF01017320.1	0.05	0.03	0	0	0.13	0
GAIF01017321.1	0.6	0.94	1.45	2.23	2.39	0.58
GAIF01017322.1	4.78	2.51	5.42	1.81	2.54	2.23
GAIF01017324.1	0.43	0.05	0.03	2.44	12.37	0.23
GAIF01017325.1	0.12	0.13	0	0.87	1.56	0
GAIF01017326.1	1.03	0.13	0	6.54	18.19	0
GAIF01017327.1	9.53	6.48	0.96	0.74	0.57	0.7
GAIF01017328.1	4.49	13.18	3.57	2.94	2.38	1.04
GAIF01017329.1	0.2	0.04	0	1.13	3.59	0.13
GAIF01017332.1	32.32	27	16.98	68.2	37.29	4.36
GAIF01017333.1	9.57	9.44	7.71	27.98	16.56	5.79
GAIF01017335.1	2.65	0.77	0.52	3.42	10.92	21.25
GAIF01017336.1	0.09	0.24	0	0.86	2.14	0.19
GAIF01017337.1	2.27	0.62	1.33	0.82	0.68	0.63
GAIF01017338.1	1.22	0.53	0.71	0.45	1.06	1.05
GAIF01017339.1	0	0	0	0.03	0	0
GAIF01017341.1	0.89	3.96	4.23	2.08	0	0
GAIF01017342.1	0.25	0	0.44	0.51	0.61	0.31

GAIF01017344.1	0	0.04	0	0.41	2.75	0
GAIF01017347.1	0.03	0.11	0.08	0.73	3.45	0.08
GAIF01017348.1	1.68	1.83	0	1.95	6.88	0
GAIF01017349.1	0.5	0	0	0.34	2.39	0.61
GAIF01017350.1	0	2.58	0	2.09	6.3	0.47
GAIF01017351.1	21.17	13.62	8.18	5.67	3.84	0.69
GAIF01017352.1	0.22	0.08	0.16	0	0.53	0.08
GAIF01017353.1	0	0	0	0.07	0.39	0
GAIF01017354.1	93.11	82	43.83	34.14	37.71	19.33
GAIF01017356.1	22.11	13.85	29.59	17.55	18.11	46.38
GAIF01017359.1	14.53	10.25	10.19	24.2	16.31	14.37
GAIF01017363.1	107.86	64.22	8.78	99.11	94.13	50.56
GAIF01017364.1	0	0	0	0	0.09	0
GAIF01017365.1	2.02	1.52	1.76	1.21	3.77	0.38
GAIF01017366.1	0.2	0.22	0	0.23	0.97	0
GAIF01017367.1	0	0	0.13	0	1.87	0.53
GAIF01017370.1	0	0	0.1	0	0	0
GAIF01017372.1	0	0	0	0.21	0.19	0
GAIF01017373.1	29.93	55.23	46.65	78.2	54.52	10.58
GAIF01017374.1	0.02	0	0	0	0	0
GAIF01017376.1	0	8.07	6.05	0	0	0.7
GAIF01017377.1	2.42	4.09	0.35	9.02	17.08	2.88
GAIF01017378.1	0.18	0.05	0.05	1.13	5.71	0.05
GAIF01017379.1	0.89	0	0	0.17	1.07	0
GAIF01017380.1	0	0	0	0.05	0	0
GAIF01017381.1	8.9	16.11	15.76	25.33	30.43	19.91
GAIF01017384.1	0.06	0.07	0	0	0.11	0
GAIF01017386.1	2.32	1.08	1.82	0.58	0.2	1.28
GAIF01017387.1	400.28	357.57	628.74	96.7	51.66	132.07
GAIF01017388.1	0.64	0	0	0.55	4.69	0.18
GAIF01017389.1	25.73	29.68	62	83.71	57.01	44.38
GAIF01017390.1	0	0	0	0.07	0	0
GAIF01017391.1	35.3	19.39	25.71	47.45	38.27	36.51
GAIF01017392.1	0	0.23	0.28	0	0	0
GAIF01017393.1	1.58	0.6	0	7.89	30.53	0.82
GAIF01017394.1	0.16	0	0.17	0	0	0
GAIF01017395.1	0.84	0.08	0.08	3.17	6.57	0.62
GAIF01017396.1	0.25	0	0	0	0	0
GAIF01017398.1	0.26	0.1	0	1.18	4.3	0.46
GAIF01017399.1	0.32	0.06	0	0.25	1.16	0
GAIF01017401.1	417.29	851.31	195.06	40.66	121.47	66.96
GAIF01017403.1	0	0	0	0.15	0	0
GAIF01017405.1	6.53	4.15	2.44	0	2.2	5.16
GAIF01017406.1	0.03	0	0	0.25	0.45	0.03

GAIF01017409.1	8.94	5.2	5.46	23.1	14.23	9.96
GAIF01017410.1	4.17	13.97	11.43	5.31	6.9	1.29
GAIF01017411.1	0.74	4.6	2.77	4.91	4.82	0.74
GAIF01017413.1	0	0	0.05	0.03	0	0.02
GAIF01017415.1	0	0	0	0	0.24	0.47
GAIF01017416.1	0	0	0	0.07	0	0
GAIF01017417.1	3.56	4.32	1.94	12.25	21.92	2.77
GAIF01017419.1	0.02	0	0	0	0	0
GAIF01017420.1	0.03	0.08	0	0.36	1.16	0
GAIF01017421.1	6.57	4.82	3.24	6.86	3.75	1.81
GAIF01017422.1	94.94	81.94	59.53	30.09	13.91	5.22
GAIF01017425.1	30.19	11.96	8.47	28.64	34.16	17.95
GAIF01017426.1	0.15	0.25	0.25	1.49	5.36	0.42
GAIF01017427.1	0	0	0	0.05	0	0
GAIF01017429.1	8.83	21.65	22.54	12.15	11.85	7.92
GAIF01017431.1	31.87	41.71	41	26.19	47.85	46.7
GAIF01017432.1	56.99	59.13	50.2	130.09	124.69	94.41
GAIF01017438.1	8.11	5.77	3.16	22.07	12.94	1.62
GAIF01017439.1	20.41	25.44	13.41	58.63	60.81	18.12
GAIF01017440.1	28.82	25.71	31.33	42.04	66.05	16.97
GAIF01017441.1	0.03	0	0.03	0.03	0	0.03
GAIF01017442.1	0	0.34	0.64	0	0	0
GAIF01017443.1	0.49	0	0	1.57	9.67	4.41
GAIF01017444.1	58.99	42.53	77.24	137.3	58.24	118.73
GAIF01017446.1	1.15	0.36	0.18	3.44	14.06	0.18
GAIF01017449.1	0.08	0.07	0	0.61	0.62	0.56
GAIF01017450.1	0.05	0	0	0.53	2.64	0
GAIF01017452.1	372.97	228.11	287.9	466.67	273.32	276.16
GAIF01017453.1	12.14	28.91	11.71	9.75	16.07	0
GAIF01017454.1	3544.05	2292.82	1622.14	1953.5	1723.79	1903.86
GAIF01017455.1	15.92	14.44	6.53	14.68	29.2	19.55
GAIF01017456.1	0.89	0.33	0.38	0.39	0.86	0.38
GAIF01017458.1	0.1	0	0	0.91	7.33	0
GAIF01017459.1	1.28	0.99	0.28	5	13.79	0.35
GAIF01017460.1	0.5	0.11	0.22	0.81	11.22	0.22
GAIF01017462.1	32.23	42.34	105.53	19.33	13.22	98.08
GAIF01017463.1	0.55	0.12	0	2.33	10.98	14.7
GAIF01017464.1	0.36	0.28	0.11	0	0	0
GAIF01017465.1	7.95	4.06	4.84	18.68	28.69	60.07
GAIF01017467.1	0.2	0.22	0	0.99	5.31	0
GAIF01017470.1	0.49	0	0	0	0	0
GAIF01017472.1	0.21	0	0	0	0	0
GAIF01017473.1	0.66	0.14	0	0	0	0
GAIF01017475.1	7.73	2.28	0.71	1.92	1.55	0.71

GAIF01017477.1	0.06	0.05	0	0	0.16	0
GAIF01017478.1	0	0	0	0	0.07	0
GAIF01017480.1	0.61	0.11	0.48	1.23	0.16	0.29
GAIF01017481.1	46.42	1.82	0	38.24	104.04	71.32
GAIF01017482.1	5.91	13.33	12.27	13.21	14.28	1.16
GAIF01017483.1	4.22	3.12	2.41	10.09	12.69	5.39
GAIF01017485.1	4.81	2.83	1.63	23.71	23.02	2.2
GAIF01017486.1	0	0	0	0	0.71	0
GAIF01017487.1	0.25	0	0.2	0	0.07	0.33
GAIF01017489.1	0.06	0	0	0.07	0	0
GAIF01017490.1	0	0	0	0.42	0	0
GAIF01017491.1	0	0	0.02	0	0	0
GAIF01017492.1	0	0	0	10.87	12.43	0.61
GAIF01017493.1	0	0	0	0.13	0	0
GAIF01017494.1	72.79	27.86	18.6	112.08	159.6	84.93
GAIF01017496.1	49.58	18.78	23.51	37.92	44.13	29.54
GAIF01017498.1	0	0.19	0	0.2	0.21	0
GAIF01017500.1	0.1	0	0	0	0.13	0
GAIF01017501.1	0.04	0	0	0.01	0.12	0
GAIF01017502.1	14.1	18.58	11.21	27.4	29.86	20.25
GAIF01017506.1	0.07	0.04	0	0.04	0.3	0
GAIF01017507.1	16.07	34.55	27.71	34.22	21.45	6.13
GAIF01017508.1	0.07	0.08	0	0	0.71	0
GAIF01017509.1	0	0.26	0.52	0	0	0.04
GAIF01017510.1	13.77	5.6	5.76	21.96	18.74	12.02
GAIF01017513.1	64.98	88.71	49.6	138.65	126.61	13.37
GAIF01017514.1	1.86	1.83	2.63	1.76	1.38	2.24
GAIF01017515.1	4.18	3.61	4.19	2.71	1.33	1.63
GAIF01017516.1	0	0	0	0	0.25	0
GAIF01017517.1	1.72	0.12	0	7.63	17.89	0.36
GAIF01017518.1	0	0	0	0	0.07	0
GAIF01017519.1	0.07	0.03	0	0.24	0.67	0
GAIF01017520.1	1.33	0.78	0.34	2.21	0.13	0.21
GAIF01017521.1	1.72	1.83	3.03	0.82	1.95	0.58
GAIF01017522.1	8.15	9.08	4.67	13.04	11.69	2.15
GAIF01017523.1	1.21	0.64	0.48	1.44	1.8	0.38
GAIF01017524.1	0	0.14	0	0.37	0.47	0.05
GAIF01017525.1	296.72	104.75	113.47	168.72	241.79	207.51
GAIF01017526.1	0.68	0.83	0.28	2.81	0.91	0.34
GAIF01017527.1	0.62	0	0	1.12	6.88	0.15
GAIF01017528.1	0	0	0	0	0.84	0
GAIF01017529.1	3.73	23.39	18.47	14.69	6.61	5.4
GAIF01017530.1	0.34	0	0	3.13	8.25	2.6
GAIF01017531.1	81.6	52.62	62.88	89.48	79.71	214.18

GAIF01017532.1	0	0	0	0	4.93	0
GAIF01017533.1	35.23	30.15	31.62	56.22	63.6	21.81
GAIF01017534.1	1.95	0	0.17	7.43	24.58	12.36
GAIF01017535.1	0	0	0	0.27	0	0
GAIF01017539.1	1209.17	1005.18	2400.66	773.64	493.23	967.27
GAIF01017540.1	3.11	1.95	0.62	5.07	7.63	3.72
GAIF01017541.1	9.88	8.29	10.33	24.32	13.32	19.55
GAIF01017542.1	6.88	8.54	6.31	5.07	1.29	1.18
GAIF01017543.1	0	0	0	0	0.37	0
GAIF01017544.1	0	0	0	0	0	0.06
GAIF01017545.1	25.83	25.92	21.03	39.9	31.17	13.33
GAIF01017546.1	0	0	0.11	0	0.12	0.11
GAIF01017548.1	8.3	9.4	4.63	42.13	34.43	3.51
GAIF01017549.1	1.11	0.39	0.39	2.14	2.4	0.88
GAIF01017550.1	25.61	18.5	7.1	61.87	84.57	54.89
GAIF01017551.1	3.64	6.8	5.11	10.97	5.16	1.76
GAIF01017552.1	0.08	0	0	0.09	0.05	0
GAIF01017554.1	0.13	0.05	0	0	0	0
GAIF01017555.1	0	0	0	0.59	0	0
GAIF01017556.1	0	0.69	0	0.9	5.9	0
GAIF01017557.1	0	0	0	0.11	0.09	0.16
GAIF01017558.1	0	0	0	0	0.15	0
GAIF01017559.1	12.48	10.18	5.82	1.16	0.23	0.07
GAIF01017560.1	0	0	0	0.09	0.45	0
GAIF01017562.1	0	0	0	0	0.05	0
GAIF01017563.1	0	0	0	0.2	0	0
GAIF01017564.1	0	0	0	0.06	0	0
GAIF01017566.1	0	0.55	0.03	0	0	0
GAIF01017567.1	0	0	0	0.08	0.25	0
GAIF01017568.1	0	0.04	0	0	0.06	0
GAIF01017569.1	64.79	51.73	33.65	204.69	179.17	25.71
GAIF01017570.1	13.71	31.36	16.28	28.34	23.38	3.4
GAIF01017571.1	0.25	0.27	0	0	1.1	0.75
GAIF01017572.1	0	0	0	0.38	1.97	7.05
GAIF01017573.1	0	0	0	0	0.64	0
GAIF01017575.1	0.28	0	0	0.97	2.85	0
GAIF01017576.1	3.19	4.48	4.87	6.71	3.39	0.46
GAIF01017577.1	0	0	0.35	0	0	0
GAIF01017578.1	5.74	14.68	12.14	12.48	8.82	1.75
GAIF01017579.1	0	0	0	0	0.87	0
GAIF01017580.1	167.38	246.4	706.79	298.7	270.79	913.03
GAIF01017581.1	1.23	0	0	0	5.5	0
GAIF01017582.1	346.69	823.46	471.38	268.9	439.43	102.51
GAIF01017583.1	0.62	0.36	0.26	1.71	1.09	0.31

GAIF01017584.1	17.14	11.55	20.86	18.81	18.82	21.56
GAIF01017586.1	0.15	0	0	0	2.13	0.87
GAIF01017588.1	0	0	0	0	0.05	0
GAIF01017589.1	10.38	5.99	0.57	43.21	96.23	11.18
GAIF01017590.1	34.73	23.36	46.5	26.1	30.3	42.59
GAIF01017591.1	0	0.06	0	0.2	0.55	0
GAIF01017592.1	24.73	10.17	4.79	47.97	70.93	9.13
GAIF01017593.1	0.38	0.34	0.15	0	0	0.04
GAIF01017594.1	53.4	51.71	35.84	89.83	60.93	16.53
GAIF01017595.1	0.16	0.28	0.27	0.05	0.03	0
GAIF01017597.1	0.11	0	0	0	0	0
GAIF01017598.1	0.48	0.41	0.22	0.66	0.96	0.19
GAIF01017600.1	0.08	0	0	0.07	0.68	0.18
GAIF01017601.1	0.2	15.12	60.84	0	1.96	63.82
GAIF01017602.1	14.9	6.67	13.08	20.85	9.7	34.96
GAIF01017603.1	0	0	0	0	0.14	0
GAIF01017604.1	8.09	14.07	13.93	19.93	11.22	4.39
GAIF01017606.1	66.91	192.4	50.9	150.13	214.04	12.89
GAIF01017607.1	0	0	0	0.07	0.05	0
GAIF01017612.1	9.57	15.25	11.04	15.93	9.02	0.98
GAIF01017613.1	0.64	1.86	0.43	0.85	1.18	0
GAIF01017614.1	0	0	0.59	0	14.5	2.14
GAIF01017615.1	277.55	256.05	271.56	327.33	205.03	160.1
GAIF01017616.1	0	0	0	0.03	0	0
GAIF01017617.1	0	0	0	0	0.69	0
GAIF01017618.1	0.05	0	0	0	0	0
GAIF01017619.1	24.84	42.49	18.31	45.04	48.03	2.29
GAIF01017622.1	0	0	0	0.04	0	0
GAIF01017623.1	20.82	20.66	15.9	52	27.14	10.39
GAIF01017624.1	33.14	62.39	62.14	60	36.94	13.83
GAIF01017626.1	4.18	4.55	3.85	2.67	3.31	1.17
GAIF01017627.1	0	0	0	0	0.06	0
GAIF01017629.1	0	1.43	1.17	0	0.12	0
GAIF01017632.1	0.12	0	0.27	1.15	3.57	0
GAIF01017633.1	0	0	0	0.54	0	0.04
GAIF01017635.1	0.08	0	0	0.09	0.83	0
GAIF01017636.1	0.1	0.03	0	0.14	0	0.68
GAIF01017637.1	0	0	0	0	0.25	0
GAIF01017639.1	0	3.07	0.96	0	0.19	0.36
GAIF01017640.1	0.37	0	0	2.23	10.59	0.41
GAIF01017641.1	21.43	43.87	29.45	3.09	56.28	17.54
GAIF01017642.1	0	0	0	0.37	0	0
GAIF01017646.1	0	0	0.18	0	0.69	0.08
GAIF01017647.1	0.09	0	0	0	0.21	0

GAIF01017648.1	0	0	0	0.51	0.76	1.1
GAIF01017650.1	0.08	0.93	0.17	0.18	0.28	0.43
GAIF01017652.1	7.04	14.31	3.81	29.7	22	0.83
GAIF01017653.1	1.45	2.23	0.78	54.98	5.13	2.59
GAIF01017654.1	0	0	0	0	0.04	0.04
GAIF01017655.1	0	0	0	0.61	0.84	0
GAIF01017657.1	0	0	0	0.51	1.05	0
GAIF01017658.1	53.57	51.68	62.9	50.48	31.91	22.33
GAIF01017659.1	0	0.06	0	0	0.07	0
GAIF01017661.1	0	0	0	0.07	0.21	0.06
GAIF01017663.1	16.07	19.41	27.8	43.04	21.51	14.74
GAIF01017664.1	0.04	0.09	0.05	0.45	1.08	0.47
GAIF01017665.1	25.44	16.94	34.43	58.14	41.19	19.15
GAIF01017666.1	0.4	0.97	0	0.26	0.76	0.15
GAIF01017667.1	4.55	3.85	4.44	54.56	13.37	5.29
GAIF01017668.1	0	0.06	0.19	0.47	0.77	0
GAIF01017669.1	8.13	1.95	1.62	2.75	1.39	0.07
GAIF01017671.1	0	0.16	0	1.7	13.76	0.16
GAIF01017672.1	0	0	0.03	0.35	1.46	0.06
GAIF01017673.1	0.42	0	0.08	4.97	9.2	0.31
GAIF01017675.1	0	0.23	0.17	0.18	0.22	0
GAIF01017676.1	2.83	0.36	0.02	6.26	28.13	1.22
GAIF01017677.1	10.78	20.52	19.87	57.07	32.12	12.45
GAIF01017679.1	0	0.1	0	0.07	0.16	0
GAIF01017680.1	0	0.34	0.34	0	0	0
GAIF01017681.1	35.76	29.55	30.59	24.18	27.48	60.88
GAIF01017682.1	0	0	0	0.24	0.12	0
GAIF01017685.1	0.13	0.08	0.12	0.84	6.45	0
GAIF01017688.1	0.15	0.33	0.52	2.64	0.18	0
GAIF01017689.1	0	0.14	0	3.43	0.9	0
GAIF01017690.1	40.71	60.39	41.64	105.08	88.04	10.11
GAIF01017691.1	1.31	0.37	0.23	6.46	15.94	1.36
GAIF01017692.1	0.34	0.3	0.11	0.08	0.04	0.04
GAIF01017693.1	62.68	56.16	16.94	114	154.87	34.58
GAIF01017694.1	0.08	0	0	0.1	0.2	0.09
GAIF01017695.1	0	0	0	0	0.07	0
GAIF01017696.1	4.3	3.49	0.93	8.76	7.28	0.66
GAIF01017698.1	19	25.78	25.76	31.56	24.79	9.9
GAIF01017699.1	0	0	0	0.13	0.47	0
GAIF01017700.1	0	0.26	0	0.09	0.19	0
GAIF01017701.1	1.47	4.75	0	5.26	0.61	0
GAIF01017703.1	338.09	248.94	342.67	935.31	743.99	417.12
GAIF01017704.1	0	0	0	0	0.07	0
GAIF01017705.1	7.27	4.11	4.47	18.72	31.04	9.81

GAIF01017707.1	0	0	0	0.27	0.92	0
GAIF01017709.1	55.75	34.41	20.45	77.33	98.24	10.67
GAIF01017711.1	0.05	0.11	0	0.06	0.42	0
GAIF01017713.1	0.04	0.14	0.18	0.53	1.59	0.09
GAIF01017714.1	0	0	0	0.31	2.01	0
GAIF01017718.1	134.91	150.2	69.92	185.93	153.93	35.41
GAIF01017719.1	1.85	0.6	0.15	1.83	3.8	0.15
GAIF01017720.1	0	0	0	0	0.36	0
GAIF01017721.1	0.31	0.21	0.03	0.75	2.8	0.12
GAIF01017722.1	0	0	0	0	0.31	0
GAIF01017723.1	1.2	0	0.49	15.62	29.37	0
GAIF01017724.1	41.23	68.3	50.98	91.92	77.72	10.86
GAIF01017725.1	19.35	39.15	35.12	43.75	27.44	7.56
GAIF01017728.1	165.54	72.07	49.99	222.9	232.35	379.52
GAIF01017730.1	0.18	0.1	0	0.42	3.81	0.4
GAIF01017731.1	1.01	0.25	0	1.5	1.13	1.76
GAIF01017732.1	2.62	2.85	8.64	12.17	9.46	14.45
GAIF01017735.1	0.08	0.08	0	0.09	0.18	0
GAIF01017736.1	0.11	0	0.12	0	0	0.25
GAIF01017737.1	0	0	0	0	0.16	0
GAIF01017738.1	1.23	1.49	0.92	21.06	21.08	8.04
GAIF01017739.1	68.58	111.23	88.06	159.47	137.27	177.74
GAIF01017742.1	0.18	0.1	0	0	0.22	0
GAIF01017743.1	1.91	1.38	1.12	0.3	0	0
GAIF01017744.1	0	0	0	0.07	0.07	0
GAIF01017746.1	0.26	0	0	0	0.27	0.37
GAIF01017747.1	47.4	51.78	25.33	197.57	253.3	26.08
GAIF01017750.1	0	0.09	0	0	0	0
GAIF01017751.1	17.57	15.64	18.11	29.53	38.84	27.38
GAIF01017753.1	0.11	0	0	1.64	4.22	0.28
GAIF01017756.1	1.67	3.26	0.12	5.41	6.94	0
GAIF01017757.1	0.1	0	0	0	0	0.08
GAIF01017758.1	3.74	0.9	0.07	0	0.26	0
GAIF01017759.1	3.21	3.32	6.53	311.16	113.78	568.63
GAIF01017760.1	0.06	0.13	0	0.07	0.15	0
GAIF01017762.1	2.56	3.6	7.04	2.93	2.91	5.88
GAIF01017763.1	0.15	0	0	0	0.52	0
GAIF01017765.1	0.34	0	0	0.92	2.57	5.3
GAIF01017766.1	0	0.07	0	0.47	0.98	0
GAIF01017767.1	0	0	0	0	0.13	0
GAIF01017768.1	0.12	0	0	0	0	0
GAIF01017769.1	11.05	6.13	6.19	3.4	1.9	3.48
GAIF01017770.1	0.06	0	0	0	0	0
GAIF01017771.1	0.54	0	0	0.31	0.65	0

GAIF01017773.1	11.49	8.4	9.08	49.6	81.81	0
GAIF01017775.1	23.35	28.43	34.03	45.49	46.35	11.51
GAIF01017776.1	0	0	0	0.07	0	0
GAIF01017777.1	3.39	1.59	1.35	3.21	5.23	5.15
GAIF01017779.1	3058.94	2960.58	5391.47	987.43	589.81	1224.66
GAIF01017780.1	24.41	21.15	32.79	42.6	29.55	24.75
GAIF01017781.1	0.48	0	0.74	0.78	2.75	1.8
GAIF01017785.1	0.13	0.7	0	0.15	0.31	0
GAIF01017787.1	1.08	0.09	0.13	3.08	17.57	4.92
GAIF01017788.1	0.19	0.11	0.07	0.96	4.35	0.02
GAIF01017789.1	20.06	22.06	22.03	42.04	22.33	18.57
GAIF01017791.1	19.99	5.33	2.31	48.44	112.99	0.18
GAIF01017792.1	0	0	0	0	0.13	0
GAIF01017794.1	0	0	0	0.23	0	0.18
GAIF01017796.1	0	0	0	0	0	0.17
GAIF01017800.1	12.11	3.38	0.58	24.52	47.87	10.38
GAIF01017801.1	32.73	30.73	14.06	110.94	157.51	7.85
GAIF01017802.1	1.77	0.33	0	0.87	12.02	5.56
GAIF01017803.1	0.2	0	0	0.12	0.12	0
GAIF01017804.1	33.18	29.69	17.78	101.27	68.02	8.06
GAIF01017805.1	0	0.56	0	0	0	0
GAIF01017806.1	35.87	67.37	24.96	19.76	31.27	0
GAIF01017807.1	13.2	6.87	6.14	54.79	32.79	9.01
GAIF01017809.1	12.69	7.82	4.55	22.8	38.37	18.73
GAIF01017812.1	0	0	0	1.48	5.25	0
GAIF01017813.1	0	0	0	0.14	1.02	0.13
GAIF01017814.1	0.14	0	0	0	0	0
GAIF01017815.1	1.99	0	0.2	0	0.41	0
GAIF01017816.1	0	0	0	0.35	0	0
GAIF01017818.1	28.41	21.34	8.7	99.67	154.02	12.64
GAIF01017819.1	4.34	5.39	5.25	7.26	4.59	2.68
GAIF01017820.1	39.46	31.12	35.65	56.65	41.6	22.25
GAIF01017824.1	16.56	12.01	6.06	25.63	29.85	5.27
GAIF01017825.1	0	0.29	0.91	0	0.04	0.05
GAIF01017826.1	0	0	0.03	0	0	0
GAIF01017829.1	0	0.14	0.14	0	0	0
GAIF01017830.1	0	0.07	0	0	0	0
GAIF01017831.1	0	0	0	0.25	0.51	0
GAIF01017832.1	0.47	0.2	0	0.11	0.18	0.1
GAIF01017833.1	0.24	0.33	0.07	0.07	0	0.07
GAIF01017834.1	0	0.28	0.14	0.15	2.99	0
GAIF01017836.1	0.06	0	0	0.49	2.92	0
GAIF01017839.1	1.16	0.92	2.63	0.09	0.28	2.55
GAIF01017840.1	0.11	0.12	0.13	0	0	0

GAIF01017841.1	0.09	0.12	0.09	0.1	0.07	0
GAIF01017842.1	0.22	0.08	0	0	0.18	0.08
GAIF01017843.1	72.27	66.25	49.7	78.52	45.87	20.02
GAIF01017844.1	1.72	1.53	1.47	6.82	9.34	1.52
GAIF01017845.1	31.18	26.47	33.21	24.11	19.49	9.2
GAIF01017846.1	34.3	44.12	41.28	51.92	28.88	12.98
GAIF01017847.1	2.11	0.08	0.08	2.71	6.79	5.56
GAIF01017848.1	34.26	1.36	7.76	74.55	12.98	0
GAIF01017849.1	29.26	22.97	24.21	64.52	38.52	46.85
GAIF01017851.1	32.44	32.45	30.67	4.9	21.12	21.89
GAIF01017852.1	0.37	0	0	0.05	0.22	0
GAIF01017853.1	0.4	0.54	0.44	1.28	2.89	0.33
GAIF01017854.1	38.43	37.11	41.86	318.66	120.03	475.06
GAIF01017855.1	0.89	0.6	0.49	0.84	1.89	1.61
GAIF01017857.1	1.03	0.55	0.14	2.47	9.05	0.41
GAIF01017858.1	0	0	0	0.13	0.5	0
GAIF01017859.1	40.11	20.01	14.59	25.32	39.14	20.22
GAIF01017860.1	37.7	45.11	29.79	40.34	62.23	24.54
GAIF01017861.1	0.63	1.61	0.7	1.96	5.58	0
GAIF01017862.1	0.03	0.04	0.07	0.24	3.49	0.11
GAIF01017863.1	18.55	23.89	26.78	28.07	13.62	16.26
GAIF01017864.1	0.07	0.04	0	0.1	0.82	0
GAIF01017866.1	0.14	0.29	0	1.57	8.93	0.15
GAIF01017867.1	0.79	2.49	0	6.32	6.33	0
GAIF01017868.1	0	0	0	0	0.05	0
GAIF01017869.1	1.25	1.6	0.78	3.92	2.16	1.26
GAIF01017870.1	0.99	0	0	0.29	0	0
GAIF01017871.1	0	0	0	1.61	5.88	0
GAIF01017873.1	0.26	0.04	0.16	0.6	1.83	0.04
GAIF01017876.1	0.61	0.09	0.05	2.02	8.62	9.86
GAIF01017877.1	0	0	0	0.56	2.94	0
GAIF01017878.1	0.07	0	0	0.08	0	0
GAIF01017879.1	8.3	5.23	6.04	13.87	20.55	23.07
GAIF01017880.1	143.35	35.63	228.77	59.95	18.6	102.89
GAIF01017882.1	0	4.23	5.37	0	0.25	0.72
GAIF01017884.1	2.31	1.06	6.81	2.63	2.58	0
GAIF01017885.1	0.35	0.11	0.05	3.52	14.2	0.6
GAIF01017889.1	0.49	1.19	0	12.03	27.09	12.4
GAIF01017890.1	2.74	0.45	0	0.48	7.42	0.3
GAIF01017891.1	0	0	0	0.08	0	0
GAIF01017892.1	0.15	0	0.7	1.39	0.2	0.59
GAIF01017893.1	7.15	4.5	6.12	16.73	11.63	25.88
GAIF01017894.1	66.13	75.36	41.63	64.05	56.07	11.03
GAIF01017896.1	0	0	0	0.86	2.31	0

GAIF01017897.1	88.4	16.92	6.83	57.83	160.81	280.12
GAIF01017898.1	0.1	0	0	0	0.78	0
GAIF01017899.1	2.39	7.93	2.18	4.57	1.66	0.65
GAIF01017901.1	0	0	0	0.08	0	0
GAIF01017902.1	0.24	0.08	0	1.31	6.08	0.56
GAIF01017903.1	126.34	7.79	8.42	4.99	1.65	1.99
GAIF01017905.1	0	0	0	0.05	0	0
GAIF01017906.1	27.8	18.15	22.63	48	36.42	42.46
GAIF01017908.1	0	0	0	0	0.08	0
GAIF01017909.1	1.14	1.64	1.48	1.19	1.23	0.45
GAIF01017912.1	0	0	0	0.65	0	0
GAIF01017913.1	23.48	30.6	16.66	71.65	60.55	5.75
GAIF01017914.1	1.23	0.63	0.34	2.95	6.53	0.42
GAIF01017915.1	3.54	2.6	3.64	11.31	7.91	4.81
GAIF01017916.1	0.21	0	0	0	0.29	0.12
GAIF01017917.1	18.7	10.26	7.37	24.64	14.2	17.44
GAIF01017918.1	0	0	0	0.09	0	0
GAIF01017921.1	10.62	10.28	5.44	29.99	13.64	2.68
GAIF01017922.1	29.57	32.72	13.17	14.6	12.36	4.26
GAIF01017923.1	1.82	3.64	0	14.01	9.86	2.46
GAIF01017924.1	0	0	0	0	0.08	0
GAIF01017927.1	2.58	0	0.11	20.58	72.19	0.44
GAIF01017928.1	0	0	0.27	0	0	0
GAIF01017929.1	1.78	5.8	0.9	7.3	10.2	9.19
GAIF01017930.1	0.26	0.09	0	0.4	6.29	0
GAIF01017932.1	2.84	0.77	0.11	0.71	0.98	0
GAIF01017933.1	0	0	0	0	0.09	0
GAIF01017935.1	0.9	0	0	0	3.59	1.08
GAIF01017936.1	0	0.04	0	0.12	0.28	0
GAIF01017937.1	0.09	0.03	0	0	0.07	0
GAIF01017938.1	0.26	0.14	0	0.3	0.31	0
GAIF01017939.1	6961.96	633.15	1809.32	3815.55	412.93	2973.62
GAIF01017940.1	0	0.1	0.2	0	0	0
GAIF01017941.1	3.66	1.56	0	41.97	116	2.32
GAIF01017942.1	3.07	1.58	2.33	11.57	31.38	1.65
GAIF01017943.1	0.06	0	0	0.07	0	0
GAIF01017945.1	0.38	0.08	0.04	0.2	0.13	0
GAIF01017949.1	59.74	71.04	54.72	146.13	126.72	18.22
GAIF01017950.1	0.16	0	0	0.69	0.38	0
GAIF01017951.1	0	0.06	0	0.37	5.99	0.06
GAIF01017953.1	0	0	0	0	0.07	0
GAIF01017954.1	0	0.15	0	0	0.41	0.22
GAIF01017956.1	0	0.1	0	0	0	0
GAIF01017957.1	596.23	283.29	179.58	377.44	298.65	136.43

GAIF01017958.1	1.32	1.45	1.38	3.46	0.93	0.81
GAIF01017959.1	4.55	3.04	2.27	28.73	25.76	1.19
GAIF01017960.1	5.1	8.99	5.09	13.55	22.78	1.02
GAIF01017961.1	1.41	0.61	0.62	4.92	3.23	2.33
GAIF01017963.1	0.87	1.14	6.7	0	0	0
GAIF01017964.1	1.16	1.58	3.02	0.68	0.77	1.63
GAIF01017965.1	3.57	2.56	2.07	3.64	12.97	2.55
GAIF01017966.1	290.05	297.42	641.6	192.6	120.45	285.71
GAIF01017968.1	6.09	0.01	0	7.42	19.59	2.18
GAIF01017972.1	75.16	79.68	113.03	49.38	39.29	43
GAIF01017973.1	16.36	10.63	25.65	31.05	25.05	71.97
GAIF01017974.1	0	0	0	0	0.78	0.07
GAIF01017976.1	0.07	0.07	0.04	0.04	0.08	0
GAIF01017977.1	0	0.1	0	0	6.05	0.33
GAIF01017978.1	0.44	0	0.1	1.32	3.79	0.29
GAIF01017979.1	40.46	43.84	33.05	99.65	145.13	105.8
GAIF01017982.1	103.89	55.01	77.9	264.71	114.2	199
GAIF01017983.1	0	0	0	0.4	0	0
GAIF01017984.1	1.27	0.55	0.42	7.97	19.72	0.56
GAIF01017985.1	0	0	0	0	0.29	0.02
GAIF01017986.1	0.07	0.04	0	0.12	0	0
GAIF01017988.1	0	0	0	0	0.15	0
GAIF01017991.1	0.02	0.04	0	0	0	0
GAIF01017992.1	10.44	17.23	14.48	41.59	25.16	6.67
GAIF01017994.1	0.68	0.29	0	0.31	0.32	0
GAIF01017995.1	10.56	1.21	6.36	2.5	6.73	184.46
GAIF01017996.1	0.67	1.02	0.15	1.5	2.63	0.15
GAIF01017997.1	3.24	3.54	0.59	5.98	12.17	1.83
GAIF01017998.1	55.88	90.3	77.6	89.68	73.2	20.51
GAIF01017999.1	0.62	0.35	0.09	0.23	0.35	0
GAIF01018001.1	55.12	126.29	52.44	80.64	63.96	8.43
GAIF01018002.1	0	0	0	0.04	0.71	0
GAIF01018003.1	177.87	130.22	264.93	134.17	59.14	138.98
GAIF01018004.1	0	0	0	0.37	2.21	0
GAIF01018005.1	400.47	342.3	372.44	289.57	169.07	109.17
GAIF01018006.1	27.7	27.73	22.42	115.04	46.49	8.65
GAIF01018007.1	0.37	0.26	0.13	2.12	12.91	0.24
GAIF01018008.1	54.63	15.84	54.89	75.03	35.02	14.86
GAIF01018010.1	0	0.03	0	0	0.04	0
GAIF01018011.1	0.26	0.28	0.09	1.8	2.9	0
GAIF01018012.1	0.63	0	0.35	2.57	6.85	0.7
GAIF01018014.1	0	0	0	0	21.02	0
GAIF01018016.1	11.83	16.29	14.09	15.16	5.73	1.78
GAIF01018019.1	0	0	0	0.27	0.47	0

GAIF01018020.1	0.11	0.06	0.06	0.37	1.74	0.06
GAIF01018021.1	0	0	0	0.23	0.24	0
GAIF01018022.1	0	0	0	0	0.97	0
GAIF01018023.1	3.51	2.49	4.69	3.37	1.47	4.21
GAIF01018024.1	0.18	0.06	0.23	0.06	0.03	0
GAIF01018025.1	1.61	0.27	0.05	4.46	0.69	0.34
GAIF01018027.1	0	0.16	0.11	0	0.27	0
GAIF01018028.1	17.36	27.62	20.62	22.58	15.33	5.54
GAIF01018030.1	0.01	0	0	0.21	3.38	0
GAIF01018031.1	0	0	0.11	0.23	0.38	0.11
GAIF01018033.1	24.23	18.83	23.36	28.53	17.7	22.29
GAIF01018034.1	14.44	16.14	23.19	20.81	9.82	4.28
GAIF01018035.1	13.51	11.56	15.89	19.38	10.62	3.56
GAIF01018036.1	82.2	53.32	78	6.01	2.09	5.67
GAIF01018037.1	3.8	6.75	1.43	0	0.53	0.09
GAIF01018038.1	0.33	0	0	0	0	0
GAIF01018039.1	155.93	76.86	170.44	4.03	0	7.3
GAIF01018040.1	0	0.12	0	0	0	0
GAIF01018041.1	0	0	0	0	0.2	0
GAIF01018042.1	0.29	0.78	0.16	3.68	4.51	0.48
GAIF01018044.1	0	0	0	2.77	0	0
GAIF01018045.1	0.04	0	0	0.29	1.17	0
GAIF01018046.1	34.72	34.87	14.91	89.92	110.49	9.54
GAIF01018047.1	0.25	0.48	0.21	1.02	0.6	0.07
GAIF01018048.1	59.62	52.51	53.39	52.24	65.94	345.51
GAIF01018049.1	0.26	0.14	0.05	2.3	13.15	0.05
GAIF01018050.1	0	0	0	0	0.51	0.03
GAIF01018053.1	0	0	0	0	0.21	0
GAIF01018054.1	0.52	0.47	0.1	3.74	7.86	0.48
GAIF01018055.1	0.05	0.05	0	0.05	0.88	0
GAIF01018058.1	0.1	0	0	0	0	0
GAIF01018059.1	0.05	0	0.47	0.25	0.7	0.23
GAIF01018060.1	7.84	17.04	16.29	13.56	14.47	11.88
GAIF01018063.1	0.34	0	0.22	0.08	0.4	0.6
GAIF01018064.1	538.31	717.98	996.04	240.47	386.5	868.92
GAIF01018066.1	0.35	0.29	0.19	3.45	7.05	0
GAIF01018067.1	0.82	0	0	0.8	0.99	0
GAIF01018069.1	0.88	0.19	0.16	0.24	1.07	0.33
GAIF01018070.1	0.5	0.41	0.69	0.58	0.15	0.14
GAIF01018072.1	91.11	61.15	46.53	196.51	97.51	68.16
GAIF01018075.1	0	0	0.16	0.08	0.26	0
GAIF01018076.1	0	0.01	0	0	0	0
GAIF01018079.1	0	0	0	0.17	0.81	0
GAIF01018081.1	0	0.05	0	0	0.06	0

GAIF01018083.1	0.16	0.17	0	1.86	13.14	0
GAIF01018084.1	0	0.07	0.07	0.15	0.65	0
GAIF01018088.1	0	0	0.08	0	0.09	0
GAIF01018091.1	60.54	52.87	14.96	165.41	165.87	32.02
GAIF01018093.1	2.2	1.96	0.37	11.71	35.86	1.8
GAIF01018094.1	3.37	0.03	7.81	8.73	13.63	33.92
GAIF01018095.1	0.12	0.38	0.06	0.2	0.07	0
GAIF01018097.1	4.12	0.56	1.53	2.75	1.36	1.82
GAIF01018099.1	10.27	8.65	12.46	31.8	42.02	12.36
GAIF01018100.1	0	0.53	0.15	0	0	0
GAIF01018101.1	32.66	31.36	0	57.93	51.71	24.64
GAIF01018105.1	0.02	0	0	0.07	0.04	0.01
GAIF01018106.1	0	0	0	0	0	0.06
GAIF01018107.1	0	0.06	0.06	0	0.58	0
GAIF01018108.1	25.24	9.15	10.95	4.32	1.7	6.48
GAIF01018109.1	16.56	28.27	20.57	4.58	4.24	2.12
GAIF01018110.1	13.87	9.52	4.42	23.58	23.5	4.34
GAIF01018113.1	0	0	0	0	0.55	0
GAIF01018115.1	90.37	85.91	86.89	70.16	77.03	27.07
GAIF01018116.1	0	0	0	0.22	0.47	0
GAIF01018117.1	0	0.47	0.16	0	0	0
GAIF01018120.1	0	0	0	0	0.17	0
GAIF01018121.1	5.96	6.84	0	14.97	11.9	0
GAIF01018122.1	1.59	1.09	2.2	5.24	3.62	2.86
GAIF01018123.1	66.98	54.59	36.22	120.91	128.03	31.89
GAIF01018126.1	0.11	0.12	0	0	0.39	0
GAIF01018127.1	0.34	0	0	0	0.56	0
GAIF01018128.1	0	0	0	0.11	0.26	0
GAIF01018129.1	0	0.04	0	0	0	0
GAIF01018131.1	0	0	0	0	0.06	0
GAIF01018132.1	0	0	0	0	0.05	0.04
GAIF01018135.1	0.06	0.06	0.03	0.56	2.96	0.03
GAIF01018136.1	0.37	0.69	0.8	0.4	0.19	0
GAIF01018137.1	0.03	0	0	0	0.1	0.02
GAIF01018138.1	2.96	1.93	1.14	14.42	7.29	0.65
GAIF01018140.1	0	0	0	0	2.22	0.9
GAIF01018141.1	10.77	10.54	8.15	6.27	16.64	3.38
GAIF01018142.1	0	0.03	0.08	0	0	0
GAIF01018143.1	75.33	41.84	36.72	90.92	79	20.56
GAIF01018146.1	14.76	9.22	5.5	43.47	21.84	3.38
GAIF01018148.1	1.39	0.67	1.44	15.72	63.68	0.85
GAIF01018150.1	0.04	0.02	0.01	0.03	0.07	0.16
GAIF01018152.1	9.61	6.28	3.91	6.45	5.71	5.83
GAIF01018154.1	0.48	0.2	0.27	0.22	0.17	0.08

GAIF01018155.1	0.44	0.12	0.37	7.55	6.86	18.53
GAIF01018157.1	10.38	6.65	2.78	26.41	28.14	3.9
GAIF01018158.1	0.42	0.09	0	1.08	2.13	0
GAIF01018159.1	0.96	1.36	1.16	4.13	3.24	0.11
GAIF01018161.1	0	0.02	0	0.12	0.15	0
GAIF01018162.1	0	0	0	0	0.96	0.06
GAIF01018165.1	0	0.2	0	0.01	0.27	0
GAIF01018166.1	24.29	13.57	20.42	29.96	19.12	41.37
GAIF01018168.1	0	0	0	1.09	0	0.61
GAIF01018169.1	0	0.06	0	0.24	1.19	0
GAIF01018171.1	8.42	5.78	6.55	34.14	53.79	4.48
GAIF01018172.1	0.13	0	0	0	0	0.14
GAIF01018173.1	1.71	1.18	0.26	15.88	38.2	1.4
GAIF01018175.1	76.11	0.58	0.69	54.49	9.32	7.25
GAIF01018176.1	0	0.06	0	0	0.12	0
GAIF01018177.1	10.84	10.81	16.52	24.54	27.47	12.44
GAIF01018178.1	3569.56	2829.36	3486.29	1456.18	966.22	2046.25
GAIF01018179.1	2.97	1.24	1.1	4.08	13.47	6.12
GAIF01018180.1	146.29	21.38	28.79	505.66	335.87	609.76
GAIF01018181.1	9.14	13.42	10.02	19.8	21.78	4.83
GAIF01018182.1	5.55	7.02	9.28	33.35	51.95	8.35
GAIF01018184.1	1.3	0.98	0.18	1.84	4.36	0
GAIF01018187.1	0	0	0	0.1	0.2	0
GAIF01018190.1	2	0.84	2.15	1.14	0.19	0.97
GAIF01018192.1	0	0	0	3.23	3.75	12.79
GAIF01018193.1	3184.3	2282.46	4199.29	1541.82	1364.22	6883.82
GAIF01018194.1	0.14	0	0	0	0	0
GAIF01018195.1	5.57	6.03	1.5	9.4	54.06	10.22
GAIF01018198.1	0.16	0	0	0.79	2.21	0.26
GAIF01018199.1	0	0	0.05	0.24	1.54	0.09
GAIF01018200.1	1.82	3.16	1.4	11.39	24.63	0.6
GAIF01018202.1	23.69	25.17	18.97	44.85	67.34	12.29
GAIF01018203.1	0	0.35	0.56	0	0	0.57
GAIF01018204.1	2.77	0.3	0.1	2.25	3.99	3.15
GAIF01018205.1	1.65	1.22	0.94	1.61	2.23	1.82
GAIF01018206.1	11.17	17.11	23.56	20.13	16.13	11.26
GAIF01018207.1	26.88	8.29	0.96	107.67	515.86	84.19
GAIF01018208.1	25.65	40.3	26.99	58.47	53.42	7.65
GAIF01018209.1	0.03	0	0	0.35	1.79	0.24
GAIF01018210.1	16.8	35.44	25.87	28.73	17.72	7.1
GAIF01018211.1	0	0	0	1.73	0.45	0.55
GAIF01018212.1	0.11	0	0	0.09	0.27	0
GAIF01018213.1	0	0	0.26	0.05	0.58	0.12
GAIF01018214.1	0.07	0	0	0	0	0

GAIF01018215.1	22.58	30.33	22.42	8.01	4.58	2.14
GAIF01018216.1	13.06	13.7	7.82	36.83	50.69	11.47
GAIF01018218.1	22.91	36.22	19.07	27.03	26.17	6.1
GAIF01018219.1	0.14	0	0	0.16	0.33	0
GAIF01018220.1	52.05	38.88	99.69	147.91	68.1	157.44
GAIF01018222.1	0.62	1.57	0.37	0.66	0.6	0.18
GAIF01018223.1	31.55	46.55	47.32	82.6	46.89	12.99
GAIF01018224.1	0	0	0	0.54	0.99	0
GAIF01018225.1	0.27	0.42	0.06	0.23	0.18	1.08
GAIF01018226.1	29.01	9.75	4.13	23.71	32.49	37.95
GAIF01018227.1	0.08	0	0	0.88	0	0.08
GAIF01018228.1	107.18	15.06	47.38	58.38	6.15	132.12
GAIF01018229.1	0	0	0	0	4.52	0
GAIF01018231.1	143.25	167.71	182.23	113.65	109.83	200.77
GAIF01018232.1	0	0	0	0.61	0	0.14
GAIF01018234.1	1.71	0.79	0	13.15	18.87	0.16
GAIF01018236.1	0.2	0.22	0.18	0.14	0.2	0.02
GAIF01018237.1	0.11	0	0	0.3	1.35	0.04
GAIF01018238.1	0.93	0.2	0.1	3.66	10.71	1.43
GAIF01018239.1	62.71	60.9	43.49	104.53	119.36	90.59
GAIF01018241.1	0.27	0.15	0.08	0.72	0.91	0.3
GAIF01018242.1	0	0	0	0.3	1.38	0.05
GAIF01018243.1	69.43	102.41	169.86	74.65	62.94	75.11
GAIF01018244.1	3.61	1.88	2.38	0.17	0.52	0
GAIF01018245.1	23.68	16.62	13.53	43.31	26.16	13.65
GAIF01018246.1	1.78	2	2.37	3.46	1.6	0.98
GAIF01018247.1	12.8	8.17	10.14	23.57	41.13	17.83
GAIF01018248.1	33.81	160.42	23.17	58.56	342.35	13.78
GAIF01018251.1	0	0.09	0	0.09	0.09	0.17
GAIF01018253.1	0.41	0	0	0.24	2.22	3.39
GAIF01018255.1	0.26	0.11	0.06	2.11	7.07	0.06
GAIF01018256.1	0	0	0	0.1	0.1	0
GAIF01018261.1	0	0.31	0	0	1.69	0
GAIF01018262.1	0.54	0.18	0.08	0.25	0	0
GAIF01018263.1	0.1	0	0	0.06	0.06	0
GAIF01018265.1	43.13	67.54	62.44	58.59	57.14	12.44
GAIF01018266.1	146.75	56.94	21.54	62.71	90.34	73.76
GAIF01018267.1	0	0.08	0	0.08	0.64	0
GAIF01018268.1	0	0.04	0	0	0	0
GAIF01018271.1	0.64	1.05	0.35	0.62	0.9	0.24
GAIF01018272.1	15.24	10.19	10.38	39.98	55.84	6.44
GAIF01018274.1	53.55	32.31	37.76	112.03	61.67	49.24
GAIF01018275.1	0	0	0	0.18	0.37	0
GAIF01018276.1	0.06	0	0.06	0	0	0

GAIF01018277.1	1962.32	3087.02	5359.93	3042.32	2523.36	1546.14
GAIF01018278.1	0	0	0	0	0.42	0
GAIF01018279.1	0.27	0.44	0.3	0.94	1.78	0
GAIF01018280.1	0	0	0	0	0.09	0
GAIF01018281.1	0	0	0	0	0	0.16
GAIF01018282.1	35.58	53.26	19.05	74.03	57.43	6.98
GAIF01018283.1	0	0.97	1.55	0	0	0.25
GAIF01018284.1	0.52	0	0.01	0.46	0.31	0.09
GAIF01018285.1	0.08	0.17	0	0	0.1	0
GAIF01018286.1	0	0.2	0	0	0.11	0.2
GAIF01018287.1	1.44	1.78	0.46	6.35	13.12	0.34
GAIF01018289.1	0.02	0	0	0.03	0.07	0.03
GAIF01018292.1	69.22	24.22	138.8	46.55	12.67	48.58
GAIF01018293.1	0	0	0	0	0.14	0
GAIF01018294.1	0	0.33	0	0.53	0.55	0.67
GAIF01018295.1	0	0	0.09	0	2.93	0.09
GAIF01018296.1	0.17	0.27	0	0	0.2	0
GAIF01018298.1	0.14	0	0	0	0	0
GAIF01018299.1	0.13	0.69	0	5.76	11.01	0.14
GAIF01018300.1	1.94	1.05	0.64	5.18	10.73	0
GAIF01018302.1	0.51	1.59	2.3	0.96	1.3	3.21
GAIF01018303.1	5.75	1.68	0	4.62	7.17	1.38
GAIF01018304.1	8.65	6.27	6.91	17.19	12.53	11.99
GAIF01018305.1	17.8	20.11	11.87	34.28	22.38	2.76
GAIF01018306.1	1.13	0.33	0.45	1.69	1.39	1.42
GAIF01018307.1	0.44	0.24	0	0.26	2.39	0.73
GAIF01018308.1	12.94	4.62	1.89	65.13	79.65	29.27
GAIF01018309.1	13.74	9.03	15.61	23.26	9.53	10.41
GAIF01018312.1	0.72	0	0.8	1.05	2.4	2.99
GAIF01018313.1	0	0.03	0	0	0	0
GAIF01018314.1	1.97	2.43	3.05	6.97	1.51	2.07
GAIF01018315.1	0	0	0.05	0.33	0	0.49
GAIF01018316.1	11.78	6.82	7.12	35.56	61.31	11.72
GAIF01018317.1	0	0	0	1.56	1.18	0
GAIF01018318.1	556.29	284.41	108.34	465.5	469	87.07
GAIF01018319.1	1.77	1.82	1.1	4.27	6.29	0.74
GAIF01018320.1	0	0.04	0	0	0.11	0
GAIF01018321.1	0.08	0	0	0	0.14	0
GAIF01018323.1	2.37	2.2	2.82	4.7	4.89	1.53
GAIF01018324.1	0	0	0	0.24	0	0
GAIF01018325.1	0	0	0	0	0.65	0
GAIF01018326.1	51.15	4.9	1.99	2.18	0.94	2.03
GAIF01018327.1	0	0	0.7	0	0	0
GAIF01018329.1	0.1	0.28	0.46	0.06	0.25	0.46

GAIF01018334.1	0.44	0.43	0.39	0.39	0.27	0.32
GAIF01018337.1	101.52	82.78	46.48	178.33	210.6	83.32
GAIF01018340.1	38.51	48.27	24.73	31.93	44.77	8.31
GAIF01018341.1	0.16	0	0	0.11	0.51	0
GAIF01018344.1	0	0.14	0	0.44	0	0.54
GAIF01018346.1	775.88	12.8	0	398.29	1095.42	1571.03
GAIF01018347.1	0	0	0	0	1.29	0
GAIF01018348.1	0	0	0	0	0.05	0
GAIF01018351.1	0	0	0	0	0.17	0
GAIF01018352.1	1.28	1.22	1.14	4.92	5.39	1.15
GAIF01018354.1	1.1	0.21	0	3.26	5.94	0.47
GAIF01018355.1	0.13	0	0	1.69	3.12	0
GAIF01018358.1	0	0	0	0.03	0.06	0
GAIF01018360.1	0	0	0	0	0.14	0
GAIF01018361.1	24.17	23.34	5.25	3.32	14.22	0
GAIF01018363.1	2.36	0.47	0	11.32	28.99	0.59
GAIF01018365.1	6.15	12.92	6.35	29.78	36.65	2.45
GAIF01018367.1	4.3	2.74	2.77	0.86	0.18	0.16
GAIF01018368.1	172.46	179.04	148.69	342.29	300.31	53.35
GAIF01018369.1	37.66	51.88	19.4	261.21	208.21	6.05
GAIF01018371.1	0	3.06	1.65	0	0.11	0
GAIF01018372.1	1.43	2.55	4.23	6.55	9.5	3.28
GAIF01018374.1	2.81	1.53	0	5.07	6.27	4.06
GAIF01018375.1	10.76	2.99	0.43	19.07	25.96	1.69
GAIF01018376.1	0.13	0.07	0	0.22	3.46	0
GAIF01018378.1	0	0	0	0	0.1	0
GAIF01018380.1	19.54	6.5	5.85	27.09	20.16	17.05
GAIF01018381.1	0.04	0	0	0	0.09	0
GAIF01018383.1	1742.84	696.96	1007.88	445.85	25.3	443.8
GAIF01018384.1	12.07	15.32	12.35	1.08	0.46	0.24
GAIF01018385.1	117.92	64.45	33.48	2.61	1.84	0.51
GAIF01018386.1	0	0	0	0	0.04	0
GAIF01018388.1	0	0	0	0.18	0.62	0
GAIF01018389.1	23.75	18.83	12.43	61	69.52	8.09
GAIF01018392.1	0	0	0.17	0.18	1.15	0.18
GAIF01018393.1	0.11	0	0	0	0.28	0.88
GAIF01018395.1	7.88	10.34	4.57	38.46	26.67	1.18
GAIF01018396.1	0.04	0	0	0.02	0	0
GAIF01018397.1	0	0	0.23	0.16	1.18	0
GAIF01018398.1	215.79	274.68	161.7	228.08	170.29	33.4
GAIF01018400.1	0	0.1	0	1.34	1.65	0
GAIF01018401.1	0	2	3.45	0	0.13	0.6
GAIF01018402.1	0.98	1.16	0.11	0.12	0.38	0
GAIF01018404.1	1.68	0.56	0.28	2.7	24.58	2

GAIF01018405.1	0.29	0.18	0.03	0.04	0.08	0
GAIF01018406.1	0	0.08	0.08	0.35	1.35	0
GAIF01018407.1	0.65	1.32	0.89	2.54	6.62	0.8
GAIF01018408.1	1476.63	710.92	444.1	483.74	779.33	6226.29
GAIF01018409.1	0	0	0	0.09	0.76	0
GAIF01018410.1	33.25	34.65	29.72	59.71	47.23	93.01
GAIF01018411.1	14.81	27.6	21.34	29.93	20.08	4.02
GAIF01018412.1	541.35	485.44	605.78	99.54	49.1	98.71
GAIF01018414.1	0	0.11	0	0	0.12	0
GAIF01018415.1	0	0	0.09	0	0	0
GAIF01018416.1	0.21	0	0	0.21	1.15	0
GAIF01018419.1	36.31	23.21	16.86	50.71	33.47	31.09
GAIF01018420.1	1.17	0.22	0	2.89	10.98	0.5
GAIF01018421.1	0.35	0.88	0.13	0.81	1.26	0.26
GAIF01018422.1	1649.36	773.79	902.87	17.66	12.21	34.05
GAIF01018423.1	0.66	0.09	0	0	0	0
GAIF01018424.1	10.96	5.75	5.84	34.98	73.17	51.1
GAIF01018425.1	0.08	0.06	0	1.71	4.86	0
GAIF01018426.1	29.68	28.72	24.67	32.8	15.63	7.67
GAIF01018427.1	0	0	0	0	0.33	0
GAIF01018428.1	4.79	7.31	3.2	1	0.63	0.03
GAIF01018430.1	0.15	0.45	0	0.6	0.84	0
GAIF01018431.1	0.01	0	0.07	0.01	0	0
GAIF01018432.1	0	0	0	0	0.13	0
GAIF01018433.1	1.47	0.16	0.08	5.11	18.63	8.49
GAIF01018434.1	0.94	0.57	0.23	9.67	28.19	0.11
GAIF01018435.1	0	0	0	0.12	0.06	0
GAIF01018436.1	0	0	0	0.23	0.12	0
GAIF01018438.1	0.65	0.24	0	0	0	0.31
GAIF01018439.1	0.74	1.21	0.2	0.43	0.22	0.2
GAIF01018441.1	5.14	6.64	4.3	12.32	22.44	1.1
GAIF01018442.1	0	0.03	0	0.15	0.4	0
GAIF01018444.1	8.97	15.72	12.86	30.63	13.82	5.88
GAIF01018446.1	34.46	25.89	11.12	61.68	53.14	10.65
GAIF01018447.1	0	0.06	0.14	0	0.41	0.01
GAIF01018448.1	0	0.09	0	0.1	0	0
GAIF01018449.1	0	0	0	0	0	0.4
GAIF01018451.1	0	0	0	0	0.11	0
GAIF01018452.1	0	0	0	0.42	0	0
GAIF01018454.1	0.1	0.64	1.73	0.11	0	0.98
GAIF01018455.1	3.05	3.79	3.73	7.48	4.61	1.15
GAIF01018457.1	0.19	0	0	0	0	0
GAIF01018458.1	27.04	6.53	6.1	38.87	77.01	40.36
GAIF01018459.1	784.79	737.94	224.53	109.48	74.61	15.2

GAIF01018461.1	0.3	0	0.09	0.31	0.4	0.09
GAIF01018462.1	0	0.05	0.01	0.03	0	0.03
GAIF01018463.1	0	0	0.03	0.01	0.06	0
GAIF01018464.1	0	0	0	0.18	0.57	0
GAIF01018465.1	183.6	253.19	169.39	90.62	133.35	303.37
GAIF01018466.1	0	0	0	0.69	0.68	0
GAIF01018467.1	0.07	0.08	0	0.1	0.05	0
GAIF01018469.1	0.24	0.48	0.46	0.38	0.59	0.16
GAIF01018470.1	0.61	0.67	0.57	3.44	8.55	0.83
GAIF01018471.1	0	0	0.2	0.14	0.57	0.07
GAIF01018472.1	14.92	17.86	17.25	49.16	53.42	10.13
GAIF01018474.1	0	0	0	0.04	0	0
GAIF01018475.1	2.31	3.01	0.58	1.5	4.38	1.13
GAIF01018476.1	3.19	5.58	3.96	5.24	7.29	0.57
GAIF01018477.1	0.12	0.2	0.53	0.07	0	0
GAIF01018478.1	1.2	0	0	1.75	1.29	0
GAIF01018481.1	0	0	0	0.08	0	0
GAIF01018482.1	0	0	0	0	0.05	0
GAIF01018484.1	0	0	0	0	0	0.09
GAIF01018485.1	20.93	4.57	17.61	35.66	31.68	1.8
GAIF01018486.1	0.59	0.24	1.05	1.64	0.16	0.31
GAIF01018487.1	24.69	16.62	19.79	29.56	22.46	29.51
GAIF01018488.1	44.4	83.76	21.19	81.73	98.18	5.08
GAIF01018489.1	378.14	583.19	716.44	465.62	307.13	207.99
GAIF01018490.1	306.16	355.69	245.46	287.86	377.67	236.83
GAIF01018493.1	0.08	0.43	0.69	0	0.1	0
GAIF01018494.1	1.94	12.67	2.13	11.27	7.01	0
GAIF01018497.1	0.47	0.3	0	0.23	0.33	0
GAIF01018499.1	0.09	0	0	0	0	0.19
GAIF01018500.1	0	2.76	2	0	0	0
GAIF01018501.1	240.03	1148.6	846.73	143.05	345	324.64
GAIF01018502.1	0	0	0.11	0.12	0	0.55
GAIF01018503.1	14.92	18.52	17.37	21.8	15.31	8.93
GAIF01018504.1	22.12	39.94	22.54	47.79	46.59	5.72
GAIF01018505.1	0.85	0.08	0	1.48	5.78	2.8
GAIF01018507.1	1.54	1.52	1.69	3.08	11.42	0.62
GAIF01018510.1	46.42	37.31	53.81	135.58	71.68	149.18
GAIF01018511.1	4.55	3.5	0.54	9.04	50.82	0
GAIF01018512.1	0.68	0.21	0.46	0.92	2.62	0.25
GAIF01018514.1	0.09	0	0	0	0	0
GAIF01018517.1	0.08	0.07	0.14	0	0.08	0.01
GAIF01018521.1	1.24	0.92	0	10.67	31.78	0.25
GAIF01018523.1	0	0	0.13	0	0	0
GAIF01018524.1	0.47	1.19	3.94	1.81	2.44	2.23

GAIF01018526.1	1.33	4.76	1.6	0.46	1.44	0
GAIF01018527.1	0	35.81	30.94	0	0.13	1.95
GAIF01018528.1	0	0	0	0	0.7	0
GAIF01018529.1	0	0	0	0	0.11	0
GAIF01018530.1	0	0	0	0.08	0	0
GAIF01018531.1	0	0.25	0.06	0	0	0.13
GAIF01018532.1	4.74	11.98	2.56	30.6	36.6	0.81
GAIF01018533.1	16.66	25.94	33.07	40.33	18.93	9.81
GAIF01018534.1	36.61	75.51	41.56	138.49	191.03	14.91
GAIF01018536.1	0	0	0	0.44	7.27	0
GAIF01018537.1	0.11	0	0	0.23	0.9	0
GAIF01018538.1	1.33	0.72	0.31	1.43	1.37	1.99
GAIF01018540.1	0.32	0.28	0.35	2.6	0.8	0.19
GAIF01018541.1	2.06	1.34	0.39	0.29	0	0.09
GAIF01018543.1	0.03	0.22	0.03	0.06	0.35	0.02
GAIF01018545.1	0	0	0	15.21	15.76	0
GAIF01018546.1	0.08	0	0	0.18	0.27	0
GAIF01018547.1	1.06	0.58	0.52	0.21	0	0.13
GAIF01018549.1	11.09	3.9	0	0	0	0
GAIF01018550.1	0	0	0.14	0	0	0
GAIF01018553.1	0.05	0.16	0.21	0.11	0.04	0.12
GAIF01018555.1	0.12	0	0.04	0	0	0.07
GAIF01018556.1	0	0.06	0.06	0.06	0.14	0
GAIF01018557.1	0	0	0	0	0.04	0
GAIF01018558.1	13.8	18	7.32	57.16	66.95	4.26
GAIF01018559.1	10.35	21.18	11.23	36.77	26.83	2.99
GAIF01018561.1	4.18	4.32	2.87	4.48	3.54	0.67
GAIF01018562.1	206.24	65.56	32.15	16.84	11.9	4.67
GAIF01018563.1	0.39	0.42	0.43	0.11	0.7	0
GAIF01018565.1	0.44	0.3	0.43	0.11	0.5	0.09
GAIF01018566.1	51.06	27.91	13.35	205.55	125.8	54.01
GAIF01018567.1	12.69	6.52	8.61	23.97	11.1	3.56
GAIF01018570.1	0	0	0	0	0.1	0
GAIF01018571.1	0.24	0.03	0.1	0.67	6.84	0
GAIF01018573.1	0.29	0.11	0	0.11	0	0.11
GAIF01018574.1	0.13	0.09	0.02	0.11	0.37	0.02
GAIF01018576.1	7.09	13.02	6.57	13.79	36.35	3.13
GAIF01018577.1	0	0.1	0	0	0	0
GAIF01018578.1	0.68	0	0.13	3.71	6.18	0
GAIF01018579.1	2.37	0.05	0.39	1.29	7.04	0.06
GAIF01018580.1	0	0	0	0.24	0	0
GAIF01018581.1	0	0	0	0	0.25	0
GAIF01018582.1	5.5	3.93	2.76	2.98	5.05	2.83
GAIF01018583.1	0	0	0	0	0.01	0

GAIF01018584.1	0	0	0	0	18.6	0
GAIF01018585.1	0	0	0	0.31	0	0
GAIF01018587.1	120.83	98.34	92.85	105.47	104.94	27.71
GAIF01018588.1	1.18	0.08	0.08	0.68	3.37	0
GAIF01018590.1	1.62	0.33	0	0.83	5.38	0
GAIF01018592.1	10.42	8.57	12.47	22.95	13.18	13.25
GAIF01018595.1	0	0.08	0	0	0	0
GAIF01018596.1	1.23	1.05	2.61	0.67	0.23	0.53
GAIF01018597.1	0.31	0.67	0.15	1.97	4.38	0.11
GAIF01018598.1	0	0	0	0.18	0	0
GAIF01018601.1	0	0	0	0	0.12	0
GAIF01018603.1	0	0	0.86	0	0.44	0.05
GAIF01018604.1	0	0	0	0	0.13	0
GAIF01018605.1	0.7	0	0	1.54	13.5	1.5
GAIF01018606.1	0.18	0	0.4	0	0.87	0
GAIF01018607.1	56.63	61.08	67.46	25.64	23.6	20.15
GAIF01018609.1	0.13	0.04	0	0	0.17	0
GAIF01018610.1	0.24	0	0	0.28	0.17	0
GAIF01018611.1	847.44	644.64	257.18	1577.32	1309.97	497.32
GAIF01018613.1	0	0	0	0	0.09	0.08
GAIF01018614.1	98.42	52.32	94.83	226.55	264.9	474.83
GAIF01018615.1	0	0	0	0.28	0.29	0
GAIF01018616.1	0	0	0	0	0.1	0
GAIF01018617.1	3.39	3.58	2.61	12.51	10.37	6.19
GAIF01018619.1	84.39	117.54	224.21	116.35	88.9	188.05
GAIF01018620.1	82.68	69.71	28.16	89.01	89.45	129.18
GAIF01018622.1	21.78	20.42	41.82	30.73	19.07	40.8
GAIF01018623.1	0	0	0.01	0.03	0.02	0
GAIF01018624.1	0.16	0	0	1.4	1.85	0
GAIF01018625.1	77.64	41.33	34.72	99.49	85.2	28.7
GAIF01018626.1	0	0	4.32	3.64	13.3	0
GAIF01018627.1	2.96	1.31	0	7.38	6.98	0.58
GAIF01018628.1	0.4	0.14	0	0	0	0
GAIF01018629.1	14.42	4.4	7.49	37.57	73.48	17.6
GAIF01018630.1	0	0	0.02	0	0	0
GAIF01018631.1	0	0.05	0	0	0.13	0
GAIF01018633.1	0.2	0.43	0	1.38	4.36	1.96
GAIF01018634.1	7.49	6	6.71	22.13	15.99	7.01
GAIF01018635.1	0.12	0.89	0.46	0.1	0.41	0.21
GAIF01018637.1	14.02	14.64	10.16	16.27	9.92	1.72
GAIF01018638.1	0	0	0	0	0	0.04
GAIF01018639.1	0.17	0	0	0.31	1.74	0
GAIF01018640.1	16.78	17.9	18.01	52.62	51.62	9.87
GAIF01018641.1	0	0	0.54	1.9	19.61	0

GAIF01018643.1	5.31	4.22	4.26	9.92	15.8	9.81
GAIF01018644.1	0	0	0	0.17	0.19	0.03
GAIF01018645.1	0.38	0.24	0.26	0.65	0.22	0.16
GAIF01018647.1	8.79	7.37	6.26	5.13	4.71	1.71
GAIF01018648.1	0	0	0	0	0.5	0
GAIF01018649.1	0.12	0	0	0.96	1.71	0.65
GAIF01018650.1	76.73	28.39	72.09	5.07	50.77	0
GAIF01018652.1	0	0	0.04	0.37	2.36	0.88
GAIF01018654.1	0.35	0	0.43	0.24	0.48	0.17
GAIF01018655.1	0	0.06	0.06	0	0	0.06
GAIF01018657.1	8.73	0.85	3.45	6.89	0.74	0.77
GAIF01018659.1	0	0	0	0	0.68	0
GAIF01018661.1	1.34	1.11	1.06	0.07	0.31	0.07
GAIF01018662.1	0.63	0.99	1.21	0.48	0.46	0.37
GAIF01018663.1	0	0	0	0	1.52	0
GAIF01018665.1	0	0	0	0	0.21	0
GAIF01018668.1	93.93	73.09	56.8	127.57	83.06	48.81
GAIF01018670.1	0.06	0	0	0	0.19	0
GAIF01018671.1	0.03	0.03	0	0	0.05	0
GAIF01018672.1	0	0.16	0	0.17	0.31	0.11
GAIF01018674.1	10.39	5.68	1.52	15.39	43.77	10.08
GAIF01018676.1	0	0.07	0	0	0.44	0
GAIF01018677.1	0.31	0.23	0.46	1.27	2.33	0.4
GAIF01018679.1	0	0	0	0.13	0.13	0
GAIF01018680.1	1.5	0.21	0	2.12	0.38	0.42
GAIF01018681.1	113.06	138.22	98.73	199.16	122.98	26.16
GAIF01018682.1	0.15	0.17	0	0.49	0.55	0.04
GAIF01018684.1	2.08	0.41	0.17	4.14	11.15	2.37
GAIF01018685.1	0	0	0	0	0	0.03
GAIF01018686.1	23.16	21.13	33.91	16.99	8.84	29.19
GAIF01018687.1	0.14	0	0.05	0.99	2.77	0.42
GAIF01018688.1	0.01	0	0	0	0	0
GAIF01018689.1	0	0	0	0.08	0	0
GAIF01018690.1	1.65	1.8	1.64	6.05	13.06	1.37
GAIF01018691.1	0	0.34	0	0	0	0
GAIF01018692.1	2.87	0.1	0.74	3.28	2.24	9.28
GAIF01018693.1	4.47	3.32	3.9	22.63	32.21	2.75
GAIF01018694.1	0.5	0.18	1.1	0.95	1.54	0
GAIF01018695.1	0.65	0.66	0.92	0.16	0.06	0.26
GAIF01018697.1	0	0.04	0	0	0.39	0
GAIF01018698.1	0	0.12	0.12	1.18	0	0
GAIF01018699.1	0.1	0.22	0	0.12	0.06	0.06
GAIF01018700.1	0	0	0	0	0.01	0
GAIF01018701.1	6.67	4.96	1.12	21.91	63.45	2.23

GAIF01018703.1	0.13	0	0.14	0.15	0.16	0.29
GAIF01018705.1	0.41	0.15	0	2.09	7.81	2.13
GAIF01018709.1	0	0.21	0	0	0	0
GAIF01018710.1	29.12	2.85	2.77	15.67	0.73	2.33
GAIF01018712.1	0.2	0	0	0.47	3.04	0.11
GAIF01018713.1	0.07	0.36	0.14	0.08	0	0
GAIF01018714.1	0.14	0.42	0	0	0.17	0
GAIF01018715.1	5.1	1.27	0.97	34.98	209.55	3.08
GAIF01018717.1	0.04	0	0	0.14	0.54	0
GAIF01018718.1	0	0	0	0.15	0.61	0
GAIF01018719.1	73.67	33.45	24.07	114.23	128.91	23.43
GAIF01018720.1	0.83	0.34	1.76	0.3	0.51	0
GAIF01018721.1	15.99	2	0.78	46.71	102.33	0.62
GAIF01018722.1	0	0	0	0.3	0	0.17
GAIF01018723.1	6.87	5.19	6.14	16.08	12.32	7.7
GAIF01018724.1	59	64.93	69.78	120.77	112.15	82.36
GAIF01018725.1	0	0.13	0	2.58	11.41	0.41
GAIF01018728.1	1250.08	1306.18	1178.43	528.11	363.86	404.37
GAIF01018729.1	0	0	0	0	0.14	0
GAIF01018730.1	53.37	46.75	86.84	255.85	315.33	94.08
GAIF01018731.1	52.73	82.61	78.23	45.34	42.56	33.24
GAIF01018732.1	0	0	0	0.84	0	0.03
GAIF01018734.1	0.77	1.19	0	0.43	3.54	2.67
GAIF01018735.1	52.17	38.39	68.24	21.18	10.19	26.7
GAIF01018737.1	0.03	0	0	0.14	0.33	0.07
GAIF01018738.1	10.37	14.65	8.37	21.95	18.21	11.04
GAIF01018739.1	0.07	0	0.12	0.13	1.05	0.09
GAIF01018741.1	0	0.43	0	0	0	0
GAIF01018742.1	0.64	0.1	0.11	8.17	34.28	0.11
GAIF01018743.1	8.39	10.39	11.49	34.17	21.89	5.78
GAIF01018744.1	0.03	0.33	0.23	0.25	0.07	0
GAIF01018747.1	0.35	0	0	2.41	12.09	0
GAIF01018748.1	20.73	8.64	9.77	23.7	18.18	14.21
GAIF01018750.1	0.62	0.51	0.54	4.3	1.63	3.47
GAIF01018751.1	0.16	0.17	0.17	2.03	3.73	0
GAIF01018753.1	0.31	0.3	0.09	0.88	3.66	0.06
GAIF01018754.1	32.48	13.87	5.18	0.36	0.22	0
GAIF01018755.1	1.36	1.47	0.74	0.63	0.49	0.15
GAIF01018758.1	2.95	2.75	0.78	19.62	84.12	0.32
GAIF01018760.1	0.12	0.01	0.02	0.19	0.18	0.09
GAIF01018761.1	3.57	1.94	0	7.05	10.17	0.39
GAIF01018762.1	3.21	0	0	0.95	0.08	0
GAIF01018763.1	0	0.09	0	1.86	1.32	0.47
GAIF01018764.1	11.98	4.21	2.39	32.72	41.47	3.87

GAIF01018765.1	3.02	2.38	3.41	2	1.45	3.69
GAIF01018766.1	24.79	27.14	33.23	57.84	51.06	28.69
GAIF01018767.1	0	0.11	0	0.12	0.12	0
GAIF01018769.1	9.01	7.46	7.73	30.11	35.06	9.24
GAIF01018771.1	1.4	3.03	0.13	11.48	40.86	0.07
GAIF01018772.1	1.24	1.01	0.03	0.21	0.94	28.16
GAIF01018773.1	7.19	3.83	2.09	1.7	0.18	0.32
GAIF01018774.1	18.83	19.58	10.89	46.98	55.05	2.04
GAIF01018776.1	6.16	17.33	8.53	14.3	9.83	0.74
GAIF01018777.1	3.99	5.62	4.68	1.05	0.22	0.6
GAIF01018779.1	0	0.1	0	0	0	0
GAIF01018780.1	118.73	126.25	66.82	229.87	228.81	69.78
GAIF01018781.1	3.99	8.68	6.51	9.93	5.05	2.05
GAIF01018782.1	0.09	0	0.05	0	0.44	0
GAIF01018784.1	26.46	5.44	2.84	55.48	344.87	159.94
GAIF01018785.1	0.02	0	0	0.02	0.08	0
GAIF01018787.1	0	0	0	0.2	1.23	0
GAIF01018788.1	0	0.06	0	0	0	0
GAIF01018789.1	0.29	0.4	0.32	0.6	0.09	0.08
GAIF01018790.1	2.53	0.72	1.32	0	0	0.59
GAIF01018791.1	0.28	0.35	0.2	0.16	0	0
GAIF01018792.1	0.69	0.48	0.3	2.59	7.74	0.09
GAIF01018793.1	0.03	0	0	0	0	0
GAIF01018798.1	0.03	0	0	0	0.12	0.02
GAIF01018799.1	0.53	1.44	0.92	0.77	0.53	0.34
GAIF01018800.1	0.8	0.4	0.38	1.41	1.42	0.17
GAIF01018802.1	0	0.07	0	0	0	0
GAIF01018803.1	47.97	20.48	41.02	22.5	11.22	105.07
GAIF01018804.1	0.08	0.24	0	0.35	2.31	0.11
GAIF01018807.1	59.89	26.84	40.14	40.22	43.19	392.05
GAIF01018810.1	3.6	19.79	5.95	7.49	0.38	7.87
GAIF01018811.1	0.23	0.14	0	1.19	12.07	4.63
GAIF01018812.1	7.84	4.29	2.97	21.7	14.43	10.6
GAIF01018813.1	3.78	5.89	2.31	7.28	6.82	0.19
GAIF01018814.1	0	0	0	0	0.19	0
GAIF01018815.1	548.26	162.38	232.72	478.66	651.82	2148.54
GAIF01018816.1	0.34	0.36	0.04	0.09	0.25	0.14
GAIF01018819.1	3.2	1.06	0.18	6.03	20.38	9.13
GAIF01018821.1	22.69	32.67	20.67	95.31	89.66	9.68
GAIF01018823.1	0.62	0.45	0	2.4	8.33	0.34
GAIF01018824.1	0.04	0	0	0.02	0	0
GAIF01018828.1	0.39	0	0	0.72	3.52	0
GAIF01018830.1	0	0	0	0.21	0	0
GAIF01018832.1	0.02	0	0	0.11	0.74	0

GAIF01018833.1	0.24	0	0	0	0	0
GAIF01018834.1	0	0	0.16	0.46	2.4	0.11
GAIF01018835.1	0.03	0.03	0	0.13	0.84	0
GAIF01018836.1	0	0	0	0.09	0	0
GAIF01018837.1	0	0	0.05	0.06	0.24	0.05
GAIF01018838.1	0	0	0	0.12	0.16	0.24
GAIF01018839.1	20.12	16.27	9.78	10.19	32.26	11.17
GAIF01018840.1	0.63	0.38	0.13	5.49	19.88	0
GAIF01018841.1	14.93	10.18	13.06	7.2	3.5	4.6
GAIF01018842.1	0.16	0	0	0	0	0
GAIF01018843.1	0	0	0	0	0.62	0
GAIF01018844.1	0.32	0.31	0.27	2.07	4.63	0.31
GAIF01018845.1	3.44	4.11	1.04	8.78	17.38	2.53
GAIF01018847.1	1.11	0.1	0.41	2.88	7.33	9.59
GAIF01018849.1	0	0	0	0.04	0.14	0
GAIF01018850.1	0	0	0	0	0.11	0
GAIF01018851.1	0	0.62	1.36	0	0	0.1
GAIF01018852.1	0.84	0.63	0	0.47	2.53	0
GAIF01018853.1	0.95	1.37	0.28	13.74	34.93	0.23
GAIF01018854.1	0	0	0	0.05	0	0
GAIF01018857.1	20.54	24.35	55.64	1.17	1.42	3.34
GAIF01018858.1	0	0	0.25	0.25	0.52	0
GAIF01018859.1	27.49	21.87	44.57	76.24	30.18	58.46
GAIF01018861.1	49.89	45.79	46.19	45.94	47.4	34.14
GAIF01018862.1	144.5	169.55	505.18	131.45	88.67	265.67
GAIF01018863.1	0.86	0.86	1.49	1.65	1.92	2.06
GAIF01018864.1	0.06	0.03	0	0.2	1.13	0.16
GAIF01018865.1	23.64	30.38	16.52	12.97	23.78	21.79
GAIF01018866.1	38.63	13.81	28.75	37.15	15.11	19.68
GAIF01018868.1	25.8	4.79	13.37	1.14	0.75	1.04
GAIF01018869.1	1.77	4.05	3.31	10.3	14.94	1.96
GAIF01018870.1	0.14	0.15	0	0.16	0	0
GAIF01018871.1	3.67	3.96	1.09	3.82	10.24	0.42
GAIF01018872.1	0.16	0	0.03	0.62	0	0
GAIF01018874.1	1.31	0.24	0.24	1.86	15.54	2.98
GAIF01018875.1	1.11	1.01	1.12	1.73	2.12	0.31
GAIF01018876.1	4.71	9.21	2.36	14.67	14.73	0.1
GAIF01018877.1	4.61	2.58	2.4	1.58	3.31	0
GAIF01018880.1	8.59	4.13	4.49	10.2	12.71	7.87
GAIF01018881.1	0	0.18	0	0.11	0	0
GAIF01018882.1	6.63	16.12	15.01	17.58	11.32	8.84
GAIF01018883.1	0.9	0.29	0	3.96	11.18	0.18
GAIF01018884.1	0.02	0	0	0.03	0.07	0.03
GAIF01018885.1	8.26	4.38	6.42	10.99	5.21	13.99

GAIF01018886.1	21.3	29.34	14.57	86.15	110.02	5.32
GAIF01018887.1	1.89	2.4	1.04	5.68	8.16	0.17
GAIF01018888.1	9.91	4.93	16.09	32.79	13.69	20.1
GAIF01018889.1	0.37	0	0	0	0.95	0
GAIF01018890.1	5.66	2.96	0.3	11.07	28.93	1.28
GAIF01018891.1	0	0.11	0	0.08	0.65	0
GAIF01018892.1	1.09	0.75	0.36	7.1	11.75	0.08
GAIF01018894.1	0.04	0.11	0	0	0.14	0
GAIF01018896.1	0.15	0.65	0.5	0.17	0.36	0
GAIF01018897.1	2.54	1.38	1.63	0.25	0.06	0.06
GAIF01018898.1	34.72	30.16	16.14	107.16	52.94	5.85
GAIF01018899.1	0	0	0	0.56	0.41	0
GAIF01018901.1	0	0.1	0	0	0	0
GAIF01018904.1	0	0.16	0.78	0	0	0
GAIF01018905.1	0.73	0.3	0	2.32	11.9	0.5
GAIF01018907.1	34.09	34.28	37.32	45.16	26.77	29.17
GAIF01018908.1	100.25	142.1	172.39	149.56	120.34	48.31
GAIF01018910.1	256.57	384.86	288.97	1.04	1.08	0.14
GAIF01018911.1	0	0.13	0.05	0.57	2.06	0
GAIF01018912.1	0.11	0.12	0	0	0.96	0
GAIF01018915.1	15.33	0.3	0.84	14.44	0.82	2.39
GAIF01018916.1	1.96	1.22	0.3	14.92	35.83	0
GAIF01018918.1	0.39	0.13	0	0.05	0	0.04
GAIF01018919.1	0	0	0	0	0.33	0
GAIF01018921.1	0	0	0	0	22.33	0
GAIF01018922.1	14.18	1.03	2.82	14.38	1.84	135.71
GAIF01018924.1	0	0	0	0.03	0.06	0
GAIF01018925.1	0.04	0.22	0	0.74	4.81	0.13
GAIF01018927.1	1.71	0.83	1.88	1.99	2.29	0.21
GAIF01018928.1	4.22	1.87	0.72	1.18	1.83	1.13
GAIF01018929.1	0.05	0.22	0.27	0.06	0	0.17
GAIF01018930.1	29.86	50.15	20.8	64.68	57.73	3.74
GAIF01018932.1	1.9	1.55	1.65	0.28	0.67	0.7
GAIF01018933.1	0	0	0	0	0	0.07
GAIF01018934.1	0	6.76	3.76	0	0	0.07
GAIF01018935.1	0.03	0	0	0.28	0.73	0
GAIF01018937.1	0.21	0.22	0.19	0.6	0.99	0
GAIF01018938.1	21.24	35.89	14.48	7.21	2.42	0.26
GAIF01018939.1	0	0	0	0	0.09	0
GAIF01018940.1	0.04	0.02	0.11	1.05	0	0
GAIF01018941.1	0.03	0	0.04	0	0	0
GAIF01018942.1	15.81	9.49	5.97	41.87	56.89	50.36
GAIF01018944.1	0	3.05	1.69	0	0.06	0.23
GAIF01018946.1	0.06	0	0	0	0.07	0

GAIF01018948.1	3.56	9.02	5.7	6.07	7.25	0.99
GAIF01018951.1	0	0.05	0	0	0	0.03
GAIF01018953.1	0	0.64	1.11	0.4	0	1.39
GAIF01018954.1	0	0.04	0	0	0	0
GAIF01018955.1	0.32	0.35	0.17	0	0	0.35
GAIF01018958.1	0.47	0.51	0.26	0	0	0
GAIF01018959.1	9.82	7.12	8.18	26.44	27.69	8.21
GAIF01018961.1	0	0	0	0	0	0.23
GAIF01018962.1	0	0	0.01	0	0	0
GAIF01018963.1	1.8	1.03	1.51	2.89	6.82	1.14
GAIF01018965.1	1.25	0.63	0.64	1.12	0.58	0.85
GAIF01018966.1	0.56	0.72	0.17	4.15	14.49	0.45
GAIF01018968.1	102.1	181.96	176.8	122.38	47.76	38.24
GAIF01018969.1	3.09	1.74	4.65	10.23	8.81	14.38
GAIF01018970.1	0.13	0.02	0	0.51	1.94	0.05
GAIF01018971.1	1.55	1.51	1.13	9.21	21.22	0.77
GAIF01018972.1	0	0	0	0.03	0	0
GAIF01018973.1	0.35	4.18	1.15	3.01	2.86	0.3
GAIF01018976.1	11.94	12.02	8.53	15.69	20.25	0.52
GAIF01018977.1	0.48	0.35	0.34	0.79	1.19	0.46
GAIF01018978.1	0.08	1.96	1.62	0.66	0.39	0.09
GAIF01018979.1	0	0	0	0.26	0.05	0
GAIF01018980.1	6.67	2.27	0.57	4.35	28.94	0
GAIF01018981.1	0	0	0	0.35	0	0.13
GAIF01018982.1	0	0.25	0	0.13	0	0
GAIF01018983.1	3.89	1.36	0	3.71	10.02	0
GAIF01018984.1	0.11	0	0	0.25	1.91	0
GAIF01018985.1	3.51	3.56	1.8	0.34	0.45	0
GAIF01018986.1	0.39	0.28	0.14	0.6	3.71	0.14
GAIF01018987.1	18.49	20.31	17.65	40.93	39.46	6.78
GAIF01018988.1	5.94	18	3.52	15.05	22.17	0.17
GAIF01018989.1	0.94	0.89	0.09	3.14	15.31	0.14
GAIF01018991.1	0.73	0	0.09	1.59	4.02	0
GAIF01018992.1	118.68	74.16	44.8	141.5	111.28	161.76
GAIF01018994.1	0.17	0	0	2.32	14.53	2.78
GAIF01018995.1	11.69	23.47	12.07	44.68	25.96	3.48
GAIF01018997.1	1.66	1.4	0.2	12.12	28.97	0.47
GAIF01018998.1	3.56	3.48	0.52	11.01	13.65	1.28
GAIF01019001.1	30.96	6.57	27.5	14.09	11.19	36.37
GAIF01019002.1	0.09	0.02	0.06	0.35	2.34	0.02
GAIF01019003.1	7.96	4.33	3.1	5.13	2.09	0.4
GAIF01019004.1	24.99	19.07	23.2	59.87	33.87	21.92
GAIF01019006.1	0.17	0.06	0.19	0.98	7.28	0.37
GAIF01019007.1	0	0	0	0.03	0	0

GAIF01019008.1	0	0	0	0.11	1.07	0
GAIF01019009.1	4.46	2.34	2.33	9.37	3.84	1.63
GAIF01019010.1	1.54	2.32	0	0.44	0.48	1.93
GAIF01019012.1	0	0	0	0.4	1.24	0
GAIF01019013.1	22.36	21.69	13.28	63.44	62.92	25.01
GAIF01019014.1	0	0	0	0	36.74	15.88
GAIF01019015.1	0	0.11	0	0	0.82	0.22
GAIF01019016.1	2.06	0.7	2.28	48.71	44	325.86
GAIF01019018.1	0	0	0	8.49	14.6	2.69
GAIF01019019.1	17.94	30.64	52.32	13.03	18.94	7.27
GAIF01019021.1	0.87	1.41	1.42	2.51	0.52	0.24
GAIF01019022.1	10.75	1.8	1.53	8.46	9.78	6.52
GAIF01019023.1	0	0	0	0.18	0	0
GAIF01019026.1	0	0	0.48	0	0.13	0
GAIF01019028.1	3.31	0.38	0.19	32.47	71.96	3.85
GAIF01019029.1	3.07	3.28	2.52	5.22	3.41	1
GAIF01019032.1	0.08	0	0.05	0	0	0
GAIF01019033.1	114.27	279.06	191.16	26.4	23.12	10.99
GAIF01019034.1	0	0	0.1	0	0.21	0
GAIF01019035.1	3.73	6.53	2.44	3.96	3.05	0.19
GAIF01019036.1	0	0	0	0	1.56	0
GAIF01019037.1	5.38	5.91	0	0.62	4.39	2.13
GAIF01019038.1	3.32	4.05	2.35	4.22	4.82	0.41
GAIF01019039.1	4.55	1.62	0.52	1.07	0.28	1.41
GAIF01019041.1	0.3	0.16	0	1.46	3.93	0.33
GAIF01019042.1	0.04	0	0.15	0.1	0.11	0
GAIF01019043.1	0	0.1	0	0.05	0.05	0
GAIF01019044.1	163.61	214.4	276.38	207.39	164.76	68.7
GAIF01019046.1	0	0	0	0.71	0	0
GAIF01019047.1	0.18	0.03	0	3.53	44.15	0.21
GAIF01019050.1	0.16	0	0	0.09	0.98	0
GAIF01019051.1	55.99	56.04	73.41	65.11	54.1	21.73
GAIF01019052.1	0	0	0.16	0.35	1.11	0
GAIF01019053.1	0.27	0.13	0.13	1.21	2	0.26
GAIF01019054.1	0.12	0	0	0	0.15	0.14
GAIF01019055.1	1.22	1.28	0.5	1.9	0	0.39
GAIF01019056.1	0.27	0	0	0	0.97	0
GAIF01019057.1	4.49	2.65	2.64	15.6	21.53	9.27
GAIF01019058.1	0.34	0	0	3.25	7.31	0.61
GAIF01019059.1	0.46	0.19	0.08	1.6	4.03	0.23
GAIF01019061.1	0.04	0	0	0	0	0
GAIF01019062.1	168.39	115.43	43.12	0.23	0.79	0.22
GAIF01019064.1	0	0.17	0	0	1.13	0
GAIF01019065.1	4.31	5.61	3.01	11.44	6.75	2.01

GAIF01019067.1	16.52	15.18	15.19	40.58	25.81	12.99
GAIF01019068.1	0	0.12	0	0	0	0
GAIF01019070.1	0.46	0	0	0	0	0
GAIF01019073.1	0	0	0	0	0.05	0
GAIF01019074.1	0	0	0	0.17	0	0
GAIF01019077.1	0	6.15	1.53	15.46	10.59	13.67
GAIF01019078.1	0.15	0.13	0.5	0.23	0.24	0
GAIF01019079.1	0	0.19	0	0.01	0.68	0
GAIF01019082.1	0	0.11	0.22	0.23	0.72	0
GAIF01019083.1	1.04	1.03	0.94	1.09	1.4	1.52
GAIF01019084.1	0	0.04	0.06	0	0	0
GAIF01019085.1	22.13	22.14	17	14.79	13.68	3.39
GAIF01019086.1	2.1	1.69	2.17	1.17	0.61	1.07
GAIF01019088.1	20.43	29.99	22.15	65.71	47.88	9.19
GAIF01019089.1	2.44	1.33	4.02	9.91	4.4	1.34
GAIF01019090.1	258.03	194.16	66.16	679.45	700.66	52.28
GAIF01019091.1	95.09	70	22.43	83.46	74.63	16.76
GAIF01019092.1	3.19	4.43	1.46	13.08	28.53	0.65
GAIF01019094.1	10.02	10.4	11.09	14.72	7.55	3.99
GAIF01019095.1	0	0.33	0	0	0.3	0
GAIF01019096.1	28.82	35.53	5.02	39.56	117.48	12.97
GAIF01019097.1	9.04	15.23	4.22	23.39	21.64	0.91
GAIF01019098.1	97.4	57.03	90.48	95.65	45.05	429.38
GAIF01019099.1	87.29	53.47	54.26	74.04	69.82	85.43
GAIF01019100.1	0	0.44	0	0	0.39	0
GAIF01019101.1	24.96	34.93	16.08	9.83	2.32	0
GAIF01019102.1	1.33	4.77	7.54	8.89	9.05	0
GAIF01019103.1	0	0	0	0.1	0	0
GAIF01019105.1	0.07	0	0.07	0.08	0.08	0
GAIF01019107.1	140.55	93.02	33.16	168.04	153.34	77.8
GAIF01019108.1	0	0	0	0.16	0	0
GAIF01019110.1	8.04	12.65	4.96	6.76	44.91	7.01
GAIF01019111.1	0.09	0.04	0	0.71	2.21	0
GAIF01019112.1	2.94	3.07	3.6	1.9	1.02	1.43
GAIF01019113.1	0	0	0	0	0.21	0
GAIF01019114.1	0.25	1.08	0.54	1.51	1.64	0
GAIF01019117.1	0.82	0	0	1.67	8.35	1.39
GAIF01019118.1	0	0	0	0.1	0.72	0
GAIF01019119.1	0.28	0.36	0.06	1.1	3.21	0.12
GAIF01019121.1	0	0	0.11	0	0	0
GAIF01019122.1	0.21	0	0	0	0	0
GAIF01019125.1	6.21	5.27	6.98	11.07	12.74	10.18
GAIF01019127.1	0	0.5	0.38	0.13	0	0
GAIF01019128.1	34.31	32.77	44.4	61.32	24.65	32.62

GAIF01019130.1	0	0	0	0	0.01	0
GAIF01019131.1	3.37	3.14	0.6	9.72	14.42	0.27
GAIF01019132.1	0.56	0	0.1	0.11	0	0
GAIF01019133.1	5.71	5.17	0.57	26.23	19.68	1.93
GAIF01019134.1	0	0	0	0.15	0	0
GAIF01019135.1	0	0	0	0	0.14	0
GAIF01019136.1	0	0	0	0	0.05	0.06
GAIF01019137.1	0	0	0.11	0.47	2.17	0
GAIF01019138.1	3.17	0.58	0.81	6.58	13.85	14.71
GAIF01019139.1	0.86	1.57	0.91	3.45	1.34	0.48
GAIF01019140.1	0	0.06	0	0.06	0.13	0
GAIF01019141.1	2.28	3.2	2.06	2.8	7.14	2.41
GAIF01019142.1	0	0	0	0.3	0.62	0
GAIF01019144.1	0.07	0	0	0.08	0.09	0
GAIF01019147.1	7.19	1.44	2.63	16.6	52.66	75
GAIF01019151.1	0	1.04	0.35	0	0	0.23
GAIF01019152.1	2.89	3.92	4.51	7.41	4.21	2.14
GAIF01019153.1	0	0.91	0.98	0	0	0
GAIF01019154.1	14.22	8.46	23.38	11.32	8.67	13.31
GAIF01019155.1	2.8	0.66	0.17	3.51	7.55	5
GAIF01019156.1	7.55	5.21	3.34	17.74	26.69	14.72
GAIF01019157.1	0.12	0.2	0.14	0.15	0.31	0.07
GAIF01019158.1	0	0	0	0	0	0.12
GAIF01019159.1	10.19	1.96	0	23.65	43.84	7.8
GAIF01019161.1	2.81	3.18	0.37	5.55	7.17	0.25
GAIF01019162.1	2.38	0.25	0.19	6.54	23.81	2.69
GAIF01019163.1	54.16	30.01	4.32	10.74	9.94	0.29
GAIF01019164.1	13.21	3.46	5.29	12.39	5.17	6.93
GAIF01019165.1	0	0	0	0.31	0.64	0
GAIF01019166.1	0.64	0.48	0	3.13	7.83	0
GAIF01019167.1	5.89	1.59	0.86	1.17	0.2	0
GAIF01019168.1	0.04	0.3	0.04	0.13	1.16	0.01
GAIF01019170.1	0	0	0	0	0.07	0
GAIF01019173.1	0	0.18	0	0	0.2	0
GAIF01019175.1	0	0	0	1.11	7.29	0
GAIF01019176.1	0	0	0	0.19	0.22	0
GAIF01019177.1	5.07	7.94	4.41	25.99	27.73	2.03
GAIF01019178.1	0	0	0	0.39	1.59	0
GAIF01019179.1	5.67	1.19	0	8.46	14.24	1.01
GAIF01019181.1	0	0	0.23	0.08	0.58	0
GAIF01019183.1	8.35	6.16	3.57	34.34	13.62	5.15
GAIF01019184.1	0	0	0	0	0.07	0
GAIF01019186.1	4.74	3.75	2.37	11.8	19.49	2.47
GAIF01019187.1	44.64	58.91	127.03	52.24	34.09	86.29

GAIF01019188.1	2.11	1.8	2.19	5.58	9.37	1.41
GAIF01019189.1	13.22	9.69	16.91	50.83	31.33	11.57
GAIF01019191.1	0.14	0	0	0.17	0	0
GAIF01019193.1	1.55	0.39	0	0.97	4.89	0.26
GAIF01019194.1	2.26	0.36	0.15	3.47	9.03	6
GAIF01019195.1	0.04	0	0	0.04	0.44	0
GAIF01019196.1	22.83	17.94	5.32	6.89	3.94	0.05
GAIF01019197.1	0	0	0	0	0.17	0
GAIF01019198.1	17.54	21.88	11.03	14.91	11.77	1.38
GAIF01019199.1	0.04	0	0	0	0	0
GAIF01019200.1	0	0.44	0	0	0.06	0
GAIF01019201.1	1	1.82	1.34	0.36	0.97	0.1
GAIF01019202.1	0.86	0.35	0	0	0.26	0
GAIF01019203.1	0	0	0	0.14	0	0
GAIF01019204.1	1.88	0.72	0.7	0.11	0.04	0.1
GAIF01019205.1	234.96	94.29	202.12	58.9	52.52	423.67
GAIF01019206.1	0	0.09	0.09	0.3	2.7	0.28
GAIF01019208.1	0	0	0.01	0	0	0
GAIF01019209.1	10.25	21.2	9.48	13.78	15.66	1.27
GAIF01019210.1	0	0	0	0.05	0.1	0
GAIF01019212.1	0	0	0	0	0	0.16
GAIF01019213.1	0.27	0	0	0.04	0	0.04
GAIF01019214.1	0.43	0	0.16	0.17	0	0
GAIF01019216.1	11.99	26.13	17.78	11.87	7.23	12.67
GAIF01019217.1	0.33	0.17	0.03	2.09	7.61	0.06
GAIF01019219.1	0.89	0.35	0.48	2.11	3.59	1.23
GAIF01019221.1	0	0	0	0	1.23	0
GAIF01019222.1	30.41	24.31	7.68	66.43	107.32	6.4
GAIF01019225.1	83.47	6.2	4.15	1.89	0.9	1.06
GAIF01019226.1	2.13	0.87	0.31	3.84	10.37	1.42
GAIF01019227.1	0	0	0.03	0.1	0	0
GAIF01019229.1	0.27	0.27	0.35	0.34	0	0.14
GAIF01019230.1	0.08	0	0	2.21	3.19	0
GAIF01019231.1	0	0	0	0	0.38	0
GAIF01019233.1	42.66	16.74	17.08	62.33	31.79	34.11
GAIF01019235.1	10.82	6.18	8.75	16.07	11.3	11.72
GAIF01019236.1	0	0	0	0	0	0.49
GAIF01019237.1	46.73	23.66	25.25	24.83	22.85	21.9
GAIF01019238.1	0	0.04	0.02	0.09	0.01	0
GAIF01019239.1	0.06	0	0	0	0	0
GAIF01019241.1	0	0	4.62	4.18	1.26	0
GAIF01019242.1	3.6	1.31	0.88	18.12	36.92	0
GAIF01019244.1	0	0	0.47	0	0	0
GAIF01019245.1	0	7.23	10.98	0	0	0.24

GAIF01019246.1	0	0.29	0	0.55	1.07	0
GAIF01019247.1	0	0.33	0	0.17	0.12	0
GAIF01019248.1	5.4	2.89	1.25	6.2	5.81	0
GAIF01019249.1	0	0	0	0.43	0.44	0
GAIF01019250.1	0	0	0.03	0	0.34	0
GAIF01019253.1	1.32	0.49	0	18.69	66.76	0
GAIF01019254.1	0	0	0	0	0.49	0
GAIF01019255.1	0	0	0.12	0	0	0
GAIF01019256.1	75.17	3.45	1.97	134.92	289.9	7727.62
GAIF01019260.1	0	0	0	1.43	5.19	8.49
GAIF01019261.1	0	0	0	0	0.06	0
GAIF01019264.1	0.24	0	0	0.37	0.67	0.09
GAIF01019265.1	0.08	0.09	0.09	0.14	0.14	0
GAIF01019266.1	15.99	18.2	11.69	32.06	25.86	4.12
GAIF01019268.1	0.49	0.43	0.47	0	0	0
GAIF01019269.1	0.38	0.42	0.7	0.3	0.15	0.42
GAIF01019271.1	0.69	0.87	0	0.8	0.2	0.4
GAIF01019272.1	0.66	0.36	0.45	0.29	3.09	5.38
GAIF01019273.1	0	0.05	0	0.34	0.56	0.29
GAIF01019274.1	229.46	329.2	485.12	152.11	141.53	426.16
GAIF01019275.1	0	0	0	0	2.8	0
GAIF01019276.1	2.29	1.32	1.17	12.37	13.81	0.23
GAIF01019277.1	20.81	8.92	16.99	60.77	62.7	30.03
GAIF01019279.1	0.14	0.31	0	0.83	2.93	0.47
GAIF01019280.1	0	0.25	0	0.13	0.14	0
GAIF01019281.1	0	1.14	0	3.15	13.78	0.97
GAIF01019282.1	5.43	2.25	3.41	0.75	0.16	1.28
GAIF01019283.1	0.39	0.21	0.14	1.51	3.13	0.57
GAIF01019284.1	6.96	8.11	10	10.28	16.32	4.86
GAIF01019286.1	0.04	0	0	1.09	3.3	0.14
GAIF01019287.1	0.41	0.68	0.82	0.65	0.49	0.37
GAIF01019289.1	0.5	0.6	0.47	2.72	8.05	0.39
GAIF01019290.1	1.73	0.4	0.2	3.91	24.62	0.8
GAIF01019291.1	2.93	1.29	2.51	3.3	1.1	1.21
GAIF01019293.1	18.24	12.48	3.38	31.05	29.14	0.61
GAIF01019294.1	1.73	0.46	3.26	0.61	0.9	0
GAIF01019295.1	10.49	0	0	0	6.31	0
GAIF01019296.1	20.85	12.67	29.93	38.1	20.06	58.45
GAIF01019298.1	0	0.13	0	0	0	0
GAIF01019301.1	1.41	0.54	0.25	5.37	6.58	0.21
GAIF01019302.1	0.21	0.12	0.02	1.34	6.31	0.14
GAIF01019305.1	7.18	8.7	6.98	17.05	11.61	4
GAIF01019307.1	0	0	0	0	0.19	0
GAIF01019312.1	16.92	12.38	3.26	22.25	19.73	22.49

GAIF01019313.1	0	0	0	0	0.72	0
GAIF01019314.1	0	0	0	0	0	0.12
GAIF01019315.1	0	0.05	0.05	0.1	1.06	0
GAIF01019316.1	2.56	1.58	4.9	5.05	1.34	3.81
GAIF01019317.1	1.45	0	0.13	1.96	10.72	6.24
GAIF01019318.1	0	0	0.03	0.67	0	0.13
GAIF01019321.1	32.89	28.99	25.83	49.04	19.02	2.52
GAIF01019322.1	0.06	0.28	0.35	0	0.08	0
GAIF01019323.1	1.29	3.83	0.27	1.77	2.86	0.07
GAIF01019324.1	30.53	17.44	22.63	21.21	27.94	59.14
GAIF01019326.1	0.12	0.13	0	0	0.14	0
GAIF01019327.1	0.16	0.29	0.3	0.49	0.86	0.08
GAIF01019328.1	9.73	6.43	5.26	16.94	8.55	4.04
GAIF01019331.1	0.14	0.05	0.1	0.22	0.28	0.05
GAIF01019332.1	0	0.06	0	0.26	0.49	0
GAIF01019333.1	0.11	0.06	0	0	0.26	0.26
GAIF01019334.1	13.73	8.16	5.37	2	2.67	0.55
GAIF01019336.1	0.35	0.09	0.1	0.61	2.51	0
GAIF01019338.1	0.16	0	0	0	0	0
GAIF01019339.1	0.95	0.62	0	2.42	4.66	0.52
GAIF01019340.1	8.34	10.06	11.04	32.47	7.64	4.3
GAIF01019342.1	0.16	0	0	0	0	0
GAIF01019343.1	0	0.51	0.35	0	0	0.09
GAIF01019344.1	26.13	8.94	8.51	17.98	8.13	6.34
GAIF01019346.1	0	0	0	0.07	0.73	0
GAIF01019347.1	0	0.17	0.42	0	0	0
GAIF01019348.1	107.81	135.94	109.97	93.17	75.25	28.87
GAIF01019349.1	9.71	0.6	0.21	1.1	6.25	1.28
GAIF01019350.1	13.75	17.55	13.45	19.31	13.06	3.51
GAIF01019352.1	0.14	0.12	0.02	0.46	4.7	0
GAIF01019356.1	427.36	212.52	509.82	11.8	4.31	18.74
GAIF01019357.1	17.02	12.34	9.54	67.54	57.6	5.17
GAIF01019359.1	0	0	0	0.09	0.19	0
GAIF01019362.1	0.98	1.83	4	2.76	2.53	2.01
GAIF01019363.1	9.98	10.18	14.51	5.27	3.75	3.13
GAIF01019365.1	0	0	0	0.22	1.24	0.1
GAIF01019366.1	0	0	0.06	0	0	0
GAIF01019369.1	2.09	0	0	2.65	8.13	0
GAIF01019370.1	0.04	0	0	0.2	0.18	0
GAIF01019371.1	0	0	0	0.23	0.92	0
GAIF01019373.1	9.99	6.24	2	0.5	0.52	0
GAIF01019375.1	0.24	0	0	0	1.85	0.26
GAIF01019376.1	0	0	0	0	0.22	0
GAIF01019377.1	0.05	0	0	0.12	0	0

GAIF01019379.1	0	0	1.88	0.59	0.4	0
GAIF01019380.1	0	0	0	0	0.08	0
GAIF01019383.1	41.33	30.85	55.68	53.29	33.05	60.69
GAIF01019386.1	18.11	12.87	13.99	13.15	4.74	5.02
GAIF01019387.1	0.32	0.12	0	0.75	0	0
GAIF01019388.1	0.29	0	0	0	1.41	0
GAIF01019389.1	0.16	0.09	0.18	0.47	0.49	0.36
GAIF01019390.1	0	0	0	0	0.36	0
GAIF01019391.1	0.18	0	0	0.27	0	0
GAIF01019392.1	8.91	5.61	1.3	5.85	10.56	1.54
GAIF01019393.1	0	0.24	0.19	0.2	0.47	0.13
GAIF01019394.1	0	0	0	0	0.14	0
GAIF01019395.1	0.09	0	0	0.05	0.27	0
GAIF01019396.1	0.03	0	0	0.67	2.85	0.15
GAIF01019398.1	0	0.11	0	1.04	3.5	0.46
GAIF01019400.1	3620.82	3064.4	3392.47	2723.6	1600.53	787.94
GAIF01019401.1	1.12	0.81	0.72	3.25	7.85	2.57
GAIF01019402.1	0.08	0	0	0	0	0
GAIF01019403.1	7.89	10.48	13.09	37.62	24.32	8.55
GAIF01019404.1	0	0	0	0.07	0.07	0
GAIF01019405.1	0.22	0	0	0.9	2.83	0.24
GAIF01019406.1	1.2	0.1	0.16	5.07	34.49	1.74
GAIF01019407.1	0	0.25	0	0.24	0.6	0
GAIF01019408.1	0	0	0	0.35	1.95	0
GAIF01019409.1	1434.57	1781.02	2307.71	1047.41	441.11	347.09
GAIF01019410.1	0.37	0.34	0.46	0.17	0.2	0.07
GAIF01019412.1	1.69	0	0	1.96	2.03	0.62
GAIF01019413.1	6.59	5.03	4.42	0	12.54	0
GAIF01019415.1	10.14	16.76	1.25	20.21	25.52	1.37
GAIF01019416.1	26.8	16.16	7.68	64.03	49.88	9.91
GAIF01019417.1	0.1	0	0	0	0.13	0
GAIF01019418.1	0	0	0	4.05	0	0
GAIF01019420.1	3.98	0	0	1.61	4.78	1.46
GAIF01019421.1	0.1	0.82	0.11	52.61	39.54	22.25
GAIF01019422.1	0.15	0	0	0	1.11	0.17
GAIF01019423.1	2.69	1.58	0.89	0.75	0.84	0.65
GAIF01019424.1	6.04	9.05	2.35	39.74	62.26	8.33
GAIF01019425.1	0.11	0.28	0	0.17	0.26	0.08
GAIF01019426.1	0.09	0	0	0.64	1.89	4.29
GAIF01019427.1	0	0.03	0	0.07	0	0
GAIF01019429.1	0.16	0.09	0.27	1.04	4.68	0.09
GAIF01019430.1	159.5	209.88	297.43	228.33	220.53	145.52
GAIF01019431.1	4.34	4.11	2.63	9.96	9.68	1.98
GAIF01019432.1	154.08	63.21	181.73	108.08	25.57	39.83

GAIF01019433.1	0	0.16	0	0.17	0.98	0
GAIF01019435.1	5.56	0.22	0	7.41	32.22	2.04
GAIF01019436.1	0	0	0	0	0.06	0
GAIF01019438.1	24.63	15.57	18.09	45.34	87.02	44.89
GAIF01019439.1	9.89	5.45	2.45	11.48	17.26	5.07
GAIF01019441.1	0.11	0.04	0	0	0	0.08
GAIF01019445.1	0	0	0	0	0.02	0
GAIF01019447.1	33.55	20.78	29.14	51.17	43.51	80.35
GAIF01019449.1	0	3.83	9.5	0	0.71	9.05
GAIF01019450.1	0.58	0.32	0.11	2.08	2.74	0.16
GAIF01019451.1	16.58	16.11	22.89	16.19	21.94	9.29
GAIF01019452.1	0.02	0	0	0	0.01	0
GAIF01019454.1	0	0	0.03	0.07	0.18	0
GAIF01019455.1	0	0	0	0.11	2.49	0.54
GAIF01019457.1	0.48	0.21	0	0.67	1.63	0
GAIF01019458.1	0.04	0	0	0.05	0.35	0.41
GAIF01019459.1	1.44	1.44	0.43	2.57	5.46	0.06
GAIF01019460.1	0.77	0	0.57	8	28.9	0
GAIF01019461.1	0.35	0.14	0	2.36	7.87	0.54
GAIF01019463.1	0.54	0	0	0.63	2.38	0
GAIF01019464.1	0	0	0.06	0	0.03	0.03
GAIF01019465.1	0	0	0	0	0.49	7.68
GAIF01019466.1	1.19	0.78	0.37	4.31	10.02	0.35
GAIF01019467.1	0.24	0	0	0.14	0	0
GAIF01019469.1	3.1	9.53	7.29	4.66	5.94	1.49
GAIF01019470.1	575.74	557.92	486.5	63.43	95.39	118.47
GAIF01019471.1	0.11	0	0	0.13	0.13	0
GAIF01019472.1	0	0	0	0	0	0.04
GAIF01019473.1	82.33	77.06	68.1	19.08	12.27	3
GAIF01019474.1	0.12	0	0	0	0.63	0.26
GAIF01019475.1	0	0	0	0.3	0	0
GAIF01019477.1	0	0	0	0.21	0.46	0
GAIF01019478.1	1.05	2.54	0.71	1.64	1.79	0.22
GAIF01019479.1	14.87	9.67	16.35	10.87	15.03	33.38
GAIF01019480.1	0.57	0	0	0.13	0.55	0.25
GAIF01019482.1	5.73	7.79	31.13	0.06	0.14	0.59
GAIF01019484.1	7.38	3.91	15.18	4.25	7.88	49.15
GAIF01019485.1	0	0	0	0	0.03	0
GAIF01019486.1	0	0	0.03	0.06	0.52	0
GAIF01019487.1	0	0	0	0.12	0	0
GAIF01019489.1	9.73	4.63	9.84	6.36	11.5	11.22
GAIF01019490.1	14.94	12.2	11.82	72.56	67.22	25.28
GAIF01019492.1	69.93	111.38	95.97	99.33	65.36	115.33
GAIF01019493.1	0	0	0	0	0.12	0

GAIF01019494.1	1.68	0.25	0.33	2.83	10.81	1.09
GAIF01019495.1	4.32	0.76	1.07	0.63	0.05	0.43
GAIF01019496.1	0.1	0.1	0	0.06	0.34	0.16
GAIF01019497.1	0	0.05	0.05	0	0	0
GAIF01019498.1	0	0.09	0.38	0	0	0
GAIF01019499.1	0.06	0.07	0	0.07	0.3	0
GAIF01019500.1	2.61	6.41	2.12	14.87	19.77	0.37
GAIF01019501.1	3.89	6.62	1.37	7.83	6.7	0.32
GAIF01019502.1	1777.6	1951.14	3574.11	295.42	0	2202.26
GAIF01019505.1	6.52	3.7	2.12	8.23	9.98	3.4
GAIF01019506.1	0.93	0.3	0	2.15	9.93	0.39
GAIF01019510.1	7.1	7.02	9.21	1.62	0.39	0.95
GAIF01019511.1	70.09	33.1	33.57	29.71	34.1	144.11
GAIF01019512.1	0.07	0	0	0.1	0.11	0
GAIF01019513.1	0.98	0	0.43	0.81	1.44	0.19
GAIF01019515.1	0.27	0.33	0	0.37	0.25	0
GAIF01019518.1	0.13	0.23	0	0.5	0	0
GAIF01019519.1	0.04	0	0	0.1	1.04	0.05
GAIF01019520.1	0.86	0.49	0.3	0.97	3.55	0.81
GAIF01019521.1	24.2	18.77	24.3	50.68	42.76	20
GAIF01019522.1	0.13	0.14	0.14	0.53	2.52	0.14
GAIF01019523.1	7.04	2.5	0.41	15.06	17.13	8.67
GAIF01019524.1	56.91	62.96	81.27	96.49	53.4	38.67
GAIF01019525.1	16.16	28.12	13.87	45.92	24.16	5.89
GAIF01019528.1	39.62	45.19	26.13	188.59	99.44	37.08
GAIF01019529.1	2.55	0.3	0.08	10.22	36.04	1.25
GAIF01019530.1	36	10.42	10.49	13.49	16.95	14.2
GAIF01019535.1	0	0	0	0	0.12	0
GAIF01019536.1	0	0	0	0.16	0	0
GAIF01019537.1	0.8	0.1	0	2.16	11.73	0
GAIF01019538.1	0	0	0	0.15	0	0
GAIF01019539.1	0	0	0	3.47	2.7	2.56
GAIF01019540.1	6.19	6.57	4.86	0.31	0	0
GAIF01019541.1	0	0	0	0.12	0.21	0
GAIF01019544.1	0	0	0	0.17	0.21	0
GAIF01019545.1	0.21	1.59	0.22	6.49	8.94	0
GAIF01019547.1	26.71	24.12	28.14	46.67	32.92	20.79
GAIF01019549.1	2.92	1.08	0.76	4.04	1.17	0.09
GAIF01019550.1	0.99	0.21	0	4.12	16.97	0.22
GAIF01019553.1	0.19	0	0.16	0	0.87	0.73
GAIF01019555.1	0.34	0.09	0.24	0.51	0.14	0.06
GAIF01019556.1	0.14	0	0	0.97	4.95	0
GAIF01019558.1	8.51	13.44	10.86	29.05	24.32	0.94
GAIF01019559.1	0.05	0	0.06	0	0	0

GAIF01019560.1	28.78	5.94	7.26	31.46	21.42	19.04
GAIF01019561.1	0.64	0.87	0.53	0.19	0.77	0
GAIF01019562.1	0.16	0	0	0.38	2	0
GAIF01019563.1	0	0	0.53	1.7	0.06	1.56
GAIF01019566.1	0	0.08	0	0	0	0
GAIF01019568.1	6.59	3.75	1.45	6.66	4.55	0.5
GAIF01019569.1	1486.04	1539.71	2226.43	1542.05	1219.42	1656.96
GAIF01019571.1	0.8	0	0.09	1.97	10.72	5.3
GAIF01019572.1	2.15	7.18	3.55	0	0.09	0.32
GAIF01019573.1	0	0	0	0.04	0.04	0
GAIF01019574.1	0.2	0	0	0.22	0	0.28
GAIF01019575.1	3.31	2.52	3.33	4.87	1.67	0.82
GAIF01019576.1	18.8	12.48	19.53	36.47	21.23	44.91
GAIF01019577.1	0	0	0	0	0.04	0
GAIF01019578.1	5.97	15.05	4.23	12.46	10.82	1.19
GAIF01019579.1	0	0	0	0	1.26	0
GAIF01019580.1	0.44	0.1	0.09	0.36	0.25	0.13
GAIF01019582.1	0.29	0	0.06	1.41	0.21	0.13
GAIF01019583.1	0.41	0	0.25	0	0	0
GAIF01019585.1	1.1	0.65	0.76	0.4	0.5	0.26
GAIF01019587.1	0.5	0.2	0	2.07	2.86	0.1
GAIF01019591.1	0.32	0.23	0	0.35	0.45	0
GAIF01019593.1	0	0	0	0.14	3.4	0
GAIF01019596.1	419.21	196.22	183.14	919.3	649.27	881.21
GAIF01019598.1	103.21	123.67	66.6	178.29	95.75	25.23
GAIF01019599.1	664.01	2300.64	572.62	50.86	81.78	15.27
GAIF01019600.1	39.79	50.76	40.64	9.13	9.68	6.21
GAIF01019601.1	0.55	0.55	2.18	4.08	4.94	3.18
GAIF01019602.1	23.46	17.3	23.41	30.55	23.77	12.47
GAIF01019603.1	0	0	0	0.2	0.84	0
GAIF01019604.1	0.1	0.27	0.05	0.3	1.05	0
GAIF01019608.1	0.36	0	0	0	0	0
GAIF01019610.1	0.41	0	0	0.4	0.97	0
GAIF01019611.1	0	0.13	0.07	0	0	0.2
GAIF01019612.1	0	0.04	0	0	0	0
GAIF01019613.1	0.3	0.33	0	0.52	0.9	0
GAIF01019614.1	18.28	20.38	18.84	10.14	11.66	19.13
GAIF01019615.1	0	0	0.08	0.16	0	0
GAIF01019616.1	0.1	0	0	0	0.5	0
GAIF01019617.1	0.75	0.23	0.47	0.25	0	0
GAIF01019619.1	57.52	26.18	27.46	56.38	37.65	32.86
GAIF01019620.1	0.25	0	0	0	0	0
GAIF01019621.1	0.06	0.13	0.2	0	0.36	0
GAIF01019622.1	29.94	37.91	29.63	75.21	78.6	25.31

GAIF01019623.1	0.16	0	0	0	0	0
GAIF01019624.1	0	0.99	0.36	0	0	0.14
GAIF01019626.1	0.52	0.19	0	0.74	2.39	0.06
GAIF01019627.1	0.81	0.22	0.36	2.26	11.23	1.03
GAIF01019628.1	0.76	0.31	0.21	1.26	0.91	0.31
GAIF01019629.1	80.3	52.41	56.22	261.88	259.08	103.84
GAIF01019630.1	4.88	1.62	2.66	11.31	15.17	11.06
GAIF01019631.1	0.34	0.06	0.63	0	0	0
GAIF01019634.1	23.99	15.76	12.85	39.5	38.33	11.83
GAIF01019635.1	0	0.85	0.61	0.78	1.62	0.37
GAIF01019636.1	0.11	0.06	0.06	0.34	1.74	0.15
GAIF01019637.1	17.91	18.57	37.89	42.39	33.54	22.14
GAIF01019640.1	1.18	0.41	0.12	1.61	3.54	0.64
GAIF01019641.1	157.94	94.49	79.03	206.87	104.47	71.95
GAIF01019644.1	0	0.13	0	0	0	0.26
GAIF01019646.1	0	0	0	0	0.14	0
GAIF01019647.1	0	0.29	0	0	0	0
GAIF01019648.1	39.69	43.29	28.01	30.44	37.5	13.28
GAIF01019650.1	113.73	80.37	78.85	122.61	48.59	26.06
GAIF01019651.1	0.22	0.56	0.24	0.2	0.62	0.09
GAIF01019653.1	0	6.3	15.79	0.4	0	11.84
GAIF01019654.1	0	0	0	33.85	0	0
GAIF01019656.1	719.42	321.62	220.1	1111.88	1515.4	4374.09
GAIF01019657.1	0.05	0.05	0	0.05	0.05	0.09
GAIF01019658.1	37.7	58.78	34.43	46.04	35.15	5.31
GAIF01019659.1	0	0.32	0.15	0	0.04	0.04
GAIF01019660.1	0.35	0.28	0	1.62	7.12	0.1
GAIF01019662.1	0	0.18	0.09	0	0	0
GAIF01019663.1	1.12	1.56	1.05	7.41	11.71	3.34
GAIF01019664.1	316.16	267.92	241.1	132.94	106.67	60.8
GAIF01019667.1	21.89	12.11	7.17	17.61	21.83	38.69
GAIF01019669.1	4.56	4.25	1.23	1.35	1.68	0.05
GAIF01019674.1	0	0	0	0.32	0	0
GAIF01019675.1	29.39	16.53	10.23	33.32	20.88	5.8
GAIF01019676.1	0	0.15	0.3	3.79	11.24	0
GAIF01019677.1	0	14.93	22.75	0	0	2.36
GAIF01019678.1	0.1	0.32	0.21	0.11	0.94	0
GAIF01019679.1	0.22	0	0.12	0.07	0.14	0.14
GAIF01019680.1	9.25	7.79	7.79	30.16	24.85	18.51
GAIF01019682.1	0	0	0.01	0.03	0.17	0
GAIF01019684.1	2045.5	589.25	700.52	28.57	15.07	26.39
GAIF01019685.1	18.41	32.08	39.6	62.26	41.22	19.74
GAIF01019687.1	32.22	56.58	41.45	43.87	38.06	10.1
GAIF01019688.1	5577.53	3262.56	1802.12	2685.02	2097.63	2097.37

GAIF01019689.1	0.13	0	0	0	0.15	0
GAIF01019690.1	0.09	0.1	0	0	0	0
GAIF01019691.1	0	0	0	0	0	0.14
GAIF01019693.1	0.21	0.96	0.68	0	2	0.57
GAIF01019694.1	0.21	0.15	0	0	0	0
GAIF01019696.1	17.66	14.96	20.34	28.69	40.95	11.67
GAIF01019697.1	0	0.14	0.14	0	0	0.14
GAIF01019698.1	2.06	0.31	0.03	9.8	52.63	1.15
GAIF01019700.1	1.59	1.87	4.25	4.8	8.76	11.63
GAIF01019701.1	21.27	34.37	41.88	52.2	28.9	13.34
GAIF01019702.1	1.51	2.51	2.14	3.6	2.12	0.54
GAIF01019704.1	16.71	0	0.18	14.94	2.67	10.04
GAIF01019705.1	0.09	0	0	0.37	2.85	0
GAIF01019706.1	0	0	0	0	0.7	0
GAIF01019707.1	0	0	0	0	0.01	0
GAIF01019708.1	0.27	0	0.24	0.03	0	0
GAIF01019709.1	0	0.19	0.08	0.02	0	0
GAIF01019711.1	0.08	0	0.17	0	0	0
GAIF01019712.1	21.54	21.26	31.88	17.26	6.35	6.6
GAIF01019713.1	16.56	8.36	14.13	9.4	14.07	46.09
GAIF01019715.1	45.89	18.95	18.04	4.1	4.24	0.97
GAIF01019716.1	0	0	0	0.23	0.35	0
GAIF01019718.1	0.14	0.39	0.21	0.62	0	0.77
GAIF01019720.1	1442.57	1311.24	2851.81	561.58	279.8	693.31
GAIF01019721.1	0	0	0	0	1.17	0.27
GAIF01019723.1	0.09	0.2	0.15	0.6	3.21	0.05
GAIF01019724.1	2.23	0.45	0	6.96	26.49	3.15
GAIF01019725.1	26.89	47.44	25.69	58.49	51.85	17.22
GAIF01019726.1	31.77	30.54	37.45	39.59	22.25	14
GAIF01019727.1	0	0	0	0.52	1.76	0
GAIF01019730.1	10.65	12.19	11.86	9.68	10.12	1.52
GAIF01019732.1	32.88	35.93	12.66	47.09	234.63	52.04
GAIF01019735.1	0	2.72	1.93	0.79	0.25	0
GAIF01019736.1	5.15	2.11	1.65	18.7	2.25	3.45
GAIF01019737.1	0	0	0	0.01	0.02	0.02
GAIF01019738.1	4.67	2.35	1.82	24.65	14.83	4.89
GAIF01019740.1	0.25	0	0.14	0.22	0.08	0
GAIF01019741.1	0	0	0	0	0.58	0
GAIF01019742.1	87.74	98.52	95.63	93.06	155.04	40.37
GAIF01019743.1	7.27	7.51	5.65	18.61	14.96	5.24
GAIF01019745.1	0	0.32	0.43	0	0	0.43
GAIF01019746.1	0	0.05	0	0	0	0
GAIF01019748.1	0.52	0.9	0.91	1.83	5.41	0.25
GAIF01019749.1	1.31	1.05	1.36	0.32	0.17	0.11

GAIF01019750.1	4.67	6.23	5.62	25.24	19.11	2.87
GAIF01019751.1	0	0	0	0.25	0.48	0
GAIF01019753.1	0	0	0	0.28	1.29	0.13
GAIF01019754.1	34	17.23	23.12	33.73	13.31	5.98
GAIF01019755.1	53.44	33	37.33	48.59	15.34	51.11
GAIF01019756.1	0.06	0.07	0.13	0.14	0.07	0.2
GAIF01019757.1	57.56	30.91	56.86	38.09	26.78	152.95
GAIF01019758.1	4.01	3.86	0.25	3.69	10.55	0.25
GAIF01019760.1	0	0.11	0	0	0.47	0
GAIF01019761.1	29.52	56.68	37.17	28.67	22.93	4.36
GAIF01019763.1	0	0.21	0	0.11	0.83	0.22
GAIF01019764.1	0.58	4.74	0	1.9	3.93	0.37
GAIF01019765.1	0	0	0	0.06	0.12	0
GAIF01019767.1	0	0	0	0	0.3	0
GAIF01019768.1	26.15	24.78	16.73	20.64	21.83	18.84
GAIF01019769.1	0	0	0.16	0.27	0.19	0
GAIF01019770.1	0	0	0	0.27	0	0.78
GAIF01019771.1	19.08	26.66	12.23	40.58	33.84	2.61
GAIF01019772.1	94.74	48.24	58.64	63.57	64.61	22.52
GAIF01019773.1	0	0.54	0	0.07	0	0
GAIF01019775.1	0.41	0.44	0	1.69	2.77	0
GAIF01019776.1	0.34	0.37	0	0	2.02	0.19
GAIF01019779.1	25.06	24.75	14.74	57.14	40.81	6.31
GAIF01019781.1	360.77	166.13	88.51	64.54	8.26	75.51
GAIF01019782.1	0	0	0	0	0.05	0
GAIF01019783.1	0	0	0	0	0.12	0
GAIF01019784.1	10.43	14.67	15.49	9.97	15.49	20.96
GAIF01019785.1	3.56	2.06	0.11	2.13	5.35	2.25
GAIF01019787.1	6.83	5.93	4.87	11.05	6.99	4.16
GAIF01019788.1	0.24	0	0	0.28	0	0
GAIF01019789.1	88.76	43.87	66.44	56.19	62.87	35.57
GAIF01019791.1	0.14	0.3	0	0.56	0.91	0
GAIF01019793.1	0	0.06	0	0.14	0.86	0
GAIF01019794.1	0.09	0	0	0	0.1	0.1
GAIF01019795.1	2.91	6.26	4.76	1.47	0.44	0.94
GAIF01019796.1	0.36	0.56	0.08	2.97	6.58	0.24
GAIF01019797.1	0.12	0	0	0.16	0.37	0
GAIF01019798.1	0.04	0	0	0.04	0.15	0
GAIF01019799.1	3.64	1.88	2.84	4.51	16.91	4.15
GAIF01019800.1	0.35	0.26	0.19	0.68	2.48	0.19
GAIF01019803.1	0	0	0	0	0.14	0
GAIF01019804.1	0	0	0.09	0	0	0
GAIF01019805.1	0	0	0	0.91	1.82	3.14
GAIF01019806.1	5.36	10.9	6.53	10.95	8.44	0.95

GAIF01019809.1	2.54	3.95	2.72	0.56	1.25	0.28
GAIF01019810.1	0.25	0.11	0.22	0.23	0.42	1.63
GAIF01019812.1	60.49	23.37	26.22	100.83	110.08	304.37
GAIF01019813.1	61.73	33.01	27.55	125.95	169.61	94.3
GAIF01019814.1	1.23	0	0.24	0	0	0
GAIF01019816.1	0.25	0.05	0.11	0.88	1.88	0
GAIF01019818.1	3.82	0.59	0.6	0	0.92	0.36
GAIF01019819.1	0	0	0	0.08	0.32	0
GAIF01019820.1	0	0	0	0.43	0.3	0
GAIF01019821.1	0	0.08	0	0.68	4.93	0
GAIF01019822.1	40.03	64.58	73.88	124.02	85.2	28.24
GAIF01019823.1	0.09	0	0	0.79	0.31	0
GAIF01019825.1	0.17	0	0	0	0	0
GAIF01019826.1	0.1	0	0	0	0	0
GAIF01019828.1	0.29	0.23	0.5	1.79	4.76	0.05
GAIF01019829.1	0.04	0.04	0	0	0	0
GAIF01019831.1	0.11	0.15	0	0.04	0	0.04
GAIF01019832.1	10.41	9.28	4.9	50.42	44.5	2.06
GAIF01019833.1	0	0.57	0.01	2.38	0	0
GAIF01019834.1	1.67	1.35	2.8	1.32	0.95	1.83
GAIF01019835.1	73.54	69.2	83.07	95.36	74.39	71.97
GAIF01019837.1	0	0	0	0.04	0.04	0
GAIF01019840.1	0.58	1.49	3.8	0.2	0.44	0.44
GAIF01019843.1	113.44	144.8	79.15	104.75	108.68	32.45
GAIF01019844.1	5	1.99	2.2	0.39	0.2	0.18
GAIF01019847.1	0.03	0.05	0.17	0.05	0.16	0.01
GAIF01019850.1	0.37	0	0.04	0.75	4.57	0.2
GAIF01019852.1	207.44	157.52	355.89	60.88	47.28	217.19
GAIF01019853.1	0.09	0.03	0.05	1.14	7.12	0.05
GAIF01019854.1	0	0	0	0	0.48	0
GAIF01019856.1	0.02	0	0	0.08	0	0
GAIF01019857.1	0.12	0	0	0.08	0.4	0.27
GAIF01019858.1	0	0.06	0	0.14	1.29	2.29
GAIF01019859.1	18.92	16.95	15.5	18.14	9.24	4.95
GAIF01019860.1	0	0	0.13	0	0	0
GAIF01019862.1	0.14	0	0	0.51	1.39	0.15
GAIF01019863.1	0.27	0	0	0.8	9.07	0.15
GAIF01019865.1	15.85	20.83	12.99	19.4	20.3	2.75
GAIF01019866.1	22.83	15.33	14.92	63.02	21.51	14.66
GAIF01019867.1	0	0	0	0.23	0	0
GAIF01019868.1	0	0	0	0.02	0	0
GAIF01019870.1	0.32	0.34	0	0.12	0.37	0
GAIF01019871.1	10.89	16.06	18.51	49.53	21.35	10.36
GAIF01019872.1	35.88	14.78	12.35	26.98	30.96	37.63

GAIF01019874.1	6	5.03	4.41	5.54	4.6	2.97
GAIF01019876.1	0.07	0	0	0	0.09	0.16
GAIF01019880.1	0	0	0	0.06	0	0
GAIF01019882.1	0.28	0.13	0.16	0.12	0.09	0
GAIF01019883.1	0.23	0	0	0	0	0
GAIF01019885.1	85.15	64.96	20.58	242.22	271.95	75.43
GAIF01019886.1	10.87	14.2	10.47	18.97	15.49	5.3
GAIF01019887.1	0	0	0	0	0.81	0
GAIF01019888.1	1.31	1.56	0.28	1.1	3.98	0.97
GAIF01019889.1	0.83	0.07	0.32	0.9	11.7	1.45
GAIF01019890.1	0	0	0.13	0.13	0.97	0
GAIF01019891.1	0	0	0	0.12	0	0
GAIF01019892.1	0.29	0	0	0	0	0
GAIF01019893.1	2.86	7.17	1.37	13	33.67	0.96
GAIF01019897.1	0.13	0	0	0	0.31	0
GAIF01019898.1	2	0.57	0.35	3.78	10.23	0.58
GAIF01019900.1	0	0	0.14	0.15	0	0
GAIF01019901.1	0	0.11	0	0.46	0.39	0
GAIF01019903.1	0	0	0	0	0.38	0
GAIF01019904.1	2.75	1	0.09	4.83	13.73	7.07
GAIF01019905.1	0.14	0	0	0.34	0.71	0
GAIF01019909.1	0.24	0.48	0.08	0.12	0.21	0
GAIF01019911.1	0	0	0	0.14	0	0
GAIF01019912.1	0.81	0.24	0.49	2.15	4.49	0.24
GAIF01019913.1	18.86	11.5	29.52	31.36	11.5	30.2
GAIF01019915.1	7.36	5.43	9.64	27.97	27.72	8.23
GAIF01019916.1	581.21	358.82	804.16	1314.85	671.44	1148.56
GAIF01019917.1	0	0.15	0	0.23	0.48	0
GAIF01019919.1	1.27	0.43	0	1.59	4.11	0.11
GAIF01019920.1	12.18	6.7	9.41	24.88	32.31	17.11
GAIF01019922.1	7.85	5.23	3.65	19.27	24.87	12.87
GAIF01019923.1	0	0	0.2	0	0	0
GAIF01019924.1	0	118.62	334.64	0	0	0
GAIF01019925.1	0	0.1	0	0	0.45	0.17
GAIF01019926.1	9.43	6.58	11.42	24.76	31.05	53.75
GAIF01019927.1	24.32	1.72	0.7	42.43	149.39	20.35
GAIF01019928.1	11.39	10.57	15.01	54.61	48.12	13.44
GAIF01019929.1	0	0	0	0.07	0.83	0
GAIF01019931.1	520.55	485.25	1093.7	261.65	188.57	709.39
GAIF01019932.1	0	0	0	0.45	0.83	0
GAIF01019935.1	0	0	0.04	0	0	0
GAIF01019937.1	0.14	0	0.16	0	0	0
GAIF01019939.1	0	0.11	0	12.74	36.36	0
GAIF01019942.1	0.24	0.06	0.07	0.13	0.26	0.03

GAIF01019945.1	0	0	0.06	0.07	0.35	0
GAIF01019947.1	10.58	25.71	12.81	21.26	22.01	2.5
GAIF01019948.1	0.8	0.11	0.11	2.56	10.14	0.44
GAIF01019949.1	0.09	0.05	0.19	0	0.17	2.42
GAIF01019951.1	0.72	0.26	0	2.83	4.46	0.79
GAIF01019952.1	0	0.18	0	0.38	0.98	0
GAIF01019953.1	35.22	36.28	8.31	51.17	58.65	10.5
GAIF01019954.1	0	0.12	0	0	0	0.36
GAIF01019955.1	0	0	0	0	0.3	0
GAIF01019956.1	1.74	0.43	0	6.27	23.6	1.7
GAIF01019957.1	0.01	0.07	0	0.15	0.04	0
GAIF01019958.1	0.25	0.11	0.15	0.12	0	0
GAIF01019959.1	0	0	0.03	0	0.04	0
GAIF01019960.1	0.15	0.19	0.03	0.21	0.53	0
GAIF01019961.1	0.01	0	0	0.06	0.03	0
GAIF01019963.1	7.67	11.6	8.63	24.98	18.18	1.22
GAIF01019966.1	4.27	2.85	2.33	18.41	61.47	0.73
GAIF01019968.1	0.1	0	0	0.8	1.67	0
GAIF01019970.1	0	0.03	0	0.12	0.06	0
GAIF01019971.1	0.36	0.19	0.04	0.55	1.31	0.08
GAIF01019972.1	0.01	0	0.01	0.03	0.15	0.01
GAIF01019973.1	0	0	0	0.91	6.43	0.17
GAIF01019974.1	0.16	0	0	0.92	4.95	0.17
GAIF01019975.1	2.39	1.77	1.89	0.67	0.23	0
GAIF01019976.1	5.79	5.63	1.67	19.97	18.49	0.84
GAIF01019977.1	0	0.23	0	0	0.31	0.32
GAIF01019978.1	0	0	0	0	0	0.1
GAIF01019979.1	0	0	0.06	0	0	0
GAIF01019981.1	0	0	0	0	0.16	0
GAIF01019982.1	0.39	0.14	0	0	4.83	0.14
GAIF01019983.1	119.88	122.2	115.16	34.78	18.02	264.23
GAIF01019984.1	1.7	0	0.21	8.83	22.98	0.54
GAIF01019985.1	0	0	0	0.03	0	0
GAIF01019988.1	1.78	0.08	0.17	11.31	42.78	3.03
GAIF01019989.1	55.24	49.51	14.16	65.53	46.29	1.69
GAIF01019990.1	0	0.2	0	0.23	0.26	0
GAIF01019991.1	0.31	0	0.17	1.67	5.46	0.42
GAIF01019992.1	9.47	9.75	9.84	12.42	14.43	4.62
GAIF01019993.1	10.16	9.82	23.93	5.18	2.64	12.73
GAIF01019995.1	0	0	0	0.12	0.65	0
GAIF01019996.1	0.37	0.1	0	0.65	5.51	0.1
GAIF01019997.1	96.45	110.61	108.21	160.79	92.03	47.11
GAIF01019999.1	37.11	42.61	55.9	64.13	31.15	27.38
GAIF01020000.1	13.18	10.56	13.82	29.87	34.9	33.08

GAIF01020002.1	64.46	49.91	45.78	59.62	126.91	475.44
GAIF01020003.1	0	653.44	578.93	23.04	305.77	1509.77
GAIF01020004.1	0.64	0.94	0.51	7.4	5.5	0.7
GAIF01020005.1	24.28	20.55	13	20.82	15.1	7.25
GAIF01020006.1	1.89	4.29	2.28	11.39	9.95	0.62
GAIF01020007.1	0.02	0.03	0.05	0.02	0.08	0.02
GAIF01020010.1	0	0	0	0	0.02	0
GAIF01020013.1	0	0	0	0.36	0.13	0
GAIF01020014.1	0	0.1	0	0.06	0.23	0
GAIF01020016.1	0.08	0	0	0	0	0
GAIF01020017.1	1.68	0	0	0	0	0
GAIF01020018.1	0.27	0.15	0.45	0.16	3.37	0.28
GAIF01020019.1	0.4	0.29	0.37	1.64	5.74	0.22
GAIF01020020.1	0	0	0.05	0.1	0	0
GAIF01020022.1	0.38	0.09	0	1.61	5.9	0.59
GAIF01020023.1	24.66	18.03	19.14	14.65	13.64	3.09
GAIF01020025.1	71.21	35.46	18.32	13.14	11.97	10.55
GAIF01020027.1	0.21	0	0	0	0	0
GAIF01020029.1	0	0	0	0.08	0.44	0
GAIF01020030.1	0	0	0	0	0.49	0
GAIF01020032.1	0.19	0.21	0.31	0.66	0.34	0.1
GAIF01020033.1	1.38	1.11	1.35	0	0	0
GAIF01020034.1	0	0	0	0	0.18	0
GAIF01020036.1	8.05	2.84	4.98	4.61	3	5.62
GAIF01020037.1	0.19	0	0.35	0.07	4.81	0
GAIF01020038.1	0	0.26	0	0.16	0.08	0.05
GAIF01020039.1	0	0	0	1.25	5.33	0.34
GAIF01020040.1	7.02	5.53	7.57	9.33	3.45	8.81
GAIF01020041.1	0	0	0.23	0	0	0.06
GAIF01020042.1	28.34	9.57	16.11	18.96	14.96	16.58
GAIF01020043.1	0	0	0	0.06	0.04	0
GAIF01020044.1	1.28	1.13	0.4	7.85	28.66	0.6
GAIF01020045.1	70.05	38.01	24.03	4.64	4.46	3.16
GAIF01020047.1	141.64	182.9	354.05	89.9	68.16	150.93
GAIF01020048.1	0	0.12	0	0	0	0.23
GAIF01020049.1	0.47	0	0.11	0	0.02	0
GAIF01020051.1	11.04	16.86	15.6	13.5	5.65	4.27
GAIF01020052.1	8.66	9.61	1.55	95.34	149.71	3.03
GAIF01020054.1	2.07	0.73	0.1	1.75	0.74	0.87
GAIF01020055.1	0	0	0	0.05	0	0
GAIF01020056.1	7.29	6.97	1.78	21.28	45.29	3.88
GAIF01020057.1	0	0.02	0.44	0	0	0
GAIF01020058.1	13.64	8.53	15.98	17.64	1.18	2.83
GAIF01020059.1	0	0	0	0.62	3.57	0.06

GAIF01020060.1	0	0.64	0.37	0	0.2	0.37
GAIF01020062.1	12.15	13.45	13.14	15.79	23.61	14.26
GAIF01020063.1	0.98	0	0.1	1.14	3.32	0
GAIF01020065.1	1188.83	893.42	767.73	1542.04	1639.88	1965.24
GAIF01020067.1	5.52	6.25	1.64	1.73	1.62	0
GAIF01020068.1	6.39	10.11	8.25	18.86	13.76	2.42
GAIF01020069.1	23.65	6.24	6.24	58.84	115.77	213.98
GAIF01020070.1	1.04	0	3.07	1.93	0	2.4
GAIF01020071.1	0	0	0	0	0.05	0
GAIF01020073.1	0	0.1	0	0	0	0
GAIF01020075.1	0	0	0.05	0.08	0.23	0
GAIF01020077.1	7.53	4.5	9.32	17.22	14.32	59.77
GAIF01020082.1	29.16	17.3	10.35	18.75	17.12	7.96
GAIF01020083.1	0.33	0	0	0	0	0
GAIF01020084.1	10.06	0.76	0.67	33.61	140.54	42.64
GAIF01020085.1	24.23	59.27	37.82	24.11	13.02	2.97
GAIF01020086.1	15.66	18.72	33.37	84	40.16	24.39
GAIF01020087.1	0.27	0.76	0.64	1.14	0.71	0
GAIF01020088.1	0	0	0	0	0.09	0
GAIF01020090.1	4.15	2.48	5.27	3.93	1.47	5.73
GAIF01020091.1	52.34	17.69	19.49	134.24	134.36	100.88
GAIF01020094.1	0	0.17	0	0	0	0
GAIF01020095.1	0	0.22	1.25	0	0	0
GAIF01020097.1	0	0	0.77	0	0	0.66
GAIF01020098.1	307.66	269.95	107.61	150.39	70.2	61.51
GAIF01020099.1	0.05	0	0	0	0	0.12
GAIF01020100.1	3.51	0.21	0.1	1.88	8.37	4.06
GAIF01020102.1	162.5	150.98	115.21	241.78	172.39	14.46
GAIF01020103.1	8.12	11.03	7.67	25.43	36.31	30.33
GAIF01020104.1	17.76	12.59	14.99	22.12	15.1	33.55
GAIF01020105.1	4.95	0.83	0.66	13.32	23.54	0.78
GAIF01020106.1	0	0	0	0	1.97	0
GAIF01020107.1	0	0	0.01	0.69	1.2	0.09
GAIF01020108.1	0	0.77	0.14	2.02	0.16	0
GAIF01020109.1	0.5	0.45	0.28	0.15	0.8	0.05
GAIF01020110.1	0	0	0	0.2	0	0
GAIF01020111.1	0	0	0	0.33	2.42	0
GAIF01020112.1	0.16	0	0	1.89	4.71	0.18
GAIF01020113.1	6.24	4.48	3.33	28.59	33.83	9.34
GAIF01020114.1	0.84	0.99	1.32	0.08	0	0
GAIF01020115.1	0.39	0.21	0.36	2.8	1.72	0.57
GAIF01020116.1	116.68	99	57.54	22.19	27.27	14.57
GAIF01020118.1	0	0	0	0	0.12	0
GAIF01020119.1	0	0	0.05	0	0	0

GAIF01020120.1	0.12	0.5	0.38	0.4	0.3	0
GAIF01020121.1	5.26	4.52	2.3	15.52	9.26	1.18
GAIF01020123.1	2.83	1.35	0.91	1.09	0.93	0.58
GAIF01020125.1	6.65	12.07	8.33	1.71	0.79	0.27
GAIF01020126.1	10.13	22.41	23.47	26.64	11.14	8.53
GAIF01020129.1	26.54	51.64	16.85	66.72	71.04	1.28
GAIF01020130.1	13.42	14.65	6.1	37	59.59	1.41
GAIF01020131.1	2.24	3.94	0.82	0.25	0	0
GAIF01020132.1	0.37	0	0	0.36	0.46	0.18
GAIF01020133.1	0.12	0.13	0	0	0	0
GAIF01020134.1	13.56	9.57	14.04	16.7	8.42	6.19
GAIF01020137.1	0	0.15	0.31	0.33	0.34	0
GAIF01020139.1	0.06	3.63	0	0	0	0
GAIF01020140.1	11.37	6.27	5.87	28.08	28.92	5.06
GAIF01020147.1	0	0	0	0	0.07	0
GAIF01020148.1	14.29	7.52	4.16	4.33	7.78	1.64
GAIF01020149.1	0.62	0	0	6.26	33.82	0
GAIF01020151.1	91.37	140.38	185.72	24.8	21.58	102.6
GAIF01020152.1	0	0	0	0	0.06	0
GAIF01020153.1	16.75	33.32	5.38	48.84	76.65	2.59
GAIF01020154.1	4.93	5.82	2.33	16.64	11.76	0.97
GAIF01020155.1	8.98	5.27	3.97	10.27	14.83	3.44
GAIF01020156.1	0	0	0	0	0.52	0
GAIF01020157.1	0	0	0	0.12	0	0.11
GAIF01020159.1	3.28	7.51	6.23	14.15	6.98	2.67
GAIF01020162.1	0	0	0	0	0.09	0
GAIF01020163.1	0.22	0.15	0.1	1.5	5.16	0.3
GAIF01020164.1	0	0	0	0	0.24	0
GAIF01020165.1	0.5	0	0	2.61	9.62	0.28
GAIF01020166.1	6.33	8.57	6.55	26.64	19.99	4.6
GAIF01020167.1	2.41	2.76	2.65	0.7	0.87	1.33
GAIF01020168.1	0	0	0	0	0.57	0
GAIF01020169.1	33.75	29.03	23.45	33.73	23.94	16.19
GAIF01020171.1	51.5	57.49	37.71	76.45	63.87	32.51
GAIF01020172.1	0	0	0.18	0	0	0
GAIF01020175.1	4.69	0	0	0.49	0.45	0
GAIF01020176.1	150.74	31.43	14.41	123.84	94.58	93.38
GAIF01020181.1	7.7	9.71	4.81	2.8	4.46	0
GAIF01020182.1	0.07	0	0	0	0.89	0.08
GAIF01020183.1	0.04	0.02	0.08	0.15	0.13	0
GAIF01020184.1	26.74	26.84	13.51	27.1	24.32	1.82
GAIF01020185.1	0.09	0.1	0	0	0	0
GAIF01020186.1	0	0	0	0	0	0.01
GAIF01020187.1	0	0	2.92	0	1.03	0

GAIF01020188.1	0.74	0.66	0.36	0.23	0.21	0.14
GAIF01020189.1	0	0	0.03	0	0	0
GAIF01020190.1	0.37	0	0.17	0.31	0.9	0.21
GAIF01020192.1	12.51	9.1	5.26	1	0.52	0.08
GAIF01020193.1	2.01	1.48	2.1	6.49	12.6	2.27
GAIF01020194.1	0.74	0.42	0.07	0.19	0.98	0.14
GAIF01020196.1	16.49	7.77	0	78.48	73.72	6.12
GAIF01020197.1	0.08	0	0	0	0.43	0
GAIF01020198.1	0	0	0	0	0.25	0
GAIF01020199.1	0.56	3.2	0.78	7.93	9.89	0.3
GAIF01020202.1	4.1	0.34	0.01	1.11	2.28	0
GAIF01020203.1	2.49	1.4	0	1.07	2.1	0
GAIF01020204.1	0	0.4	0	0.43	1.02	0
GAIF01020205.1	0.03	0.05	0.01	0.05	0.2	0.23
GAIF01020206.1	0	0	0.19	0.1	0.05	0.05
GAIF01020207.1	0	0	0.06	0	1.15	0
GAIF01020208.1	0	0	0	0.01	0	0
GAIF01020209.1	3.81	1.47	4.18	3.93	2.24	14
GAIF01020211.1	0	0	24.68	0	0	0
GAIF01020214.1	0.92	0.3	0.1	4.36	14.1	0.81
GAIF01020216.1	1.22	4.25	4.3	6.26	2.92	1.54
GAIF01020217.1	0.21	0.41	0	2.03	2.91	0.09
GAIF01020218.1	29.15	73.95	59.91	74.56	73.22	16.68
GAIF01020220.1	0	0.07	0.36	0	0	1.44
GAIF01020222.1	0	0	0	0	0.17	0.23
GAIF01020223.1	13513.03	1559.52	6479.43	13354.89	1291.1	8389.23
GAIF01020224.1	0	0	0	0	0.06	0
GAIF01020225.1	0.07	0.24	0	0.17	0.53	0
GAIF01020226.1	1.87	0.84	0.05	6.02	13.24	0.37
GAIF01020228.1	2955.45	2848.21	4839.43	1278.28	623.99	1270.89
GAIF01020229.1	0	0	0	0	0.02	0
GAIF01020231.1	18.09	18.15	5.45	72.51	45.85	2.05
GAIF01020232.1	0.3	0.33	0.5	2.11	0.73	0.33
GAIF01020233.1	29.16	32.71	25.73	36.73	21.36	12.94
GAIF01020235.1	0.31	1.35	0.17	1.8	0.37	0
GAIF01020237.1	0.72	0.67	0	0.84	2.48	0.57
GAIF01020239.1	6.68	2.23	2.91	10.83	5.35	33.22
GAIF01020240.1	0	0	0	0	0.07	0
GAIF01020243.1	10	12.69	5.71	4.52	7.71	0.71
GAIF01020245.1	127.45	98.36	96.39	167.06	69.71	230.94
GAIF01020246.1	0	0	21.59	0	0	0
GAIF01020247.1	9.78	13.43	10.48	9.82	7.11	2.62
GAIF01020249.1	0	0	0	0.8	5.64	0
GAIF01020250.1	0.11	0	0	0.38	0.92	0

GAIF01020251.1	2.67	1.64	2.14	3.13	2.78	5.52
GAIF01020254.1	0	0	0	0	0.28	0
GAIF01020256.1	50.12	9.04	1.45	59.43	51.05	2.52
GAIF01020257.1	0	0	0	0	0.21	0
GAIF01020258.1	19.07	21.57	18.6	60.15	45.24	6.07
GAIF01020259.1	65.61	14.96	47.59	74.11	41.26	19.71
GAIF01020260.1	0.09	0	0.05	0.28	0.5	0
GAIF01020261.1	0	0	0	0	0	0.39
GAIF01020262.1	0.06	0.06	0	0	1.62	0
GAIF01020263.1	0.29	0	0.16	0	1.04	0
GAIF01020264.1	0.21	0	0	0	0	0
GAIF01020265.1	0.57	0.45	0.62	0.36	0.74	0
GAIF01020266.1	34.08	12.08	19.78	1.93	1	0.69
GAIF01020267.1	0.18	0.1	0.1	0.1	0.32	0.1
GAIF01020268.1	0.55	0	0.24	0	0.13	0
GAIF01020270.1	0	0	0	0	0.17	0
GAIF01020271.1	0	0	0	0	0.09	0
GAIF01020272.1	10.71	11.37	21.51	32.28	24.61	12.21
GAIF01020273.1	1.96	0.1	0	7.87	14.39	7.08
GAIF01020274.1	0	0.13	0	0.14	1.28	0
GAIF01020275.1	0.03	0	0	0.03	1.18	0.19
GAIF01020276.1	1.43	0.32	0.56	1.99	3.53	1.69
GAIF01020277.1	1.01	0.61	0.22	8.93	22.58	0.93
GAIF01020279.1	0.51	0.44	0.11	0.35	1.09	0
GAIF01020281.1	0	0.22	0	0	0	0
GAIF01020284.1	30.38	30.1	17.91	69.1	65.18	15.42
GAIF01020285.1	0.63	0	0.22	3.62	8.78	0.9
GAIF01020286.1	0	0	0	0.11	0.17	0
GAIF01020287.1	28.79	51.56	3.76	12.06	56.95	7
GAIF01020288.1	11.98	11.57	12.06	26.54	16.19	6.93
GAIF01020289.1	3.22	2.08	1.84	10.26	11.04	3.62
GAIF01020291.1	0.31	0.17	0.37	0	0	0
GAIF01020293.1	0	0	0	0	0.18	0
GAIF01020294.1	0	0	0	0	0.16	0
GAIF01020296.1	0	0	0.11	0	0	0.94
GAIF01020297.1	0.06	0.62	0.61	0.66	0.61	0.18
GAIF01020299.1	0	0	0	0.09	0	0
GAIF01020300.1	0.05	0.09	0	0.8	7.82	0
GAIF01020301.1	0.09	0	0	1.91	17.65	0.05
GAIF01020302.1	0.4	1.05	0.53	0.47	0.39	0
GAIF01020303.1	0.17	0.09	0	0	0.3	0
GAIF01020307.1	0	0	0	0	0.08	0
GAIF01020308.1	0.16	0.52	0	1.84	1.9	0.17
GAIF01020309.1	0	0	0	0.25	1.02	0

GAIF01020311.1	0	0	0	0.26	0.18	0
GAIF01020312.1	0.01	0	0	0.06	0.08	0
GAIF01020314.1	0.44	0.48	0.42	0.74	2.36	0
GAIF01020315.1	0.4	0.07	0	0.77	1.68	0.22
GAIF01020318.1	1.92	1.13	0.16	7.21	20.28	0.16
GAIF01020320.1	2.84	2.22	3.41	7.32	5.34	3.13
GAIF01020322.1	5.13	0	0	0.85	0	1.78
GAIF01020323.1	1.69	2.78	1.79	9.45	12.31	0.26
GAIF01020324.1	0	5.03	0	0	1.51	0
GAIF01020326.1	5.82	13.66	4.07	23.03	34.04	0.58
GAIF01020330.1	25.5	33.72	12.89	95.3	73.03	4.42
GAIF01020331.1	1.35	1.14	0.54	6.35	13.84	0.34
GAIF01020332.1	7.81	8.5	5.5	35.12	74.82	12.7
GAIF01020333.1	51.76	41.46	39.19	215.51	248.01	28.91
GAIF01020336.1	0.04	0	0.06	0	0	0.03
GAIF01020339.1	0.26	0.07	0	1.18	5.92	1.19
GAIF01020340.1	0	0	0	0.11	0.55	0
GAIF01020341.1	1.83	1.77	2.27	4.62	9.27	1.45
GAIF01020343.1	0	0	0	0	0.09	0
GAIF01020346.1	0	0	0	0	0.44	0
GAIF01020348.1	14.63	4.74	2.42	20.13	18.39	16.82
GAIF01020349.1	17.48	0	0	0	21.02	0
GAIF01020350.1	12.17	14.25	15.9	21.59	16.82	3.62
GAIF01020351.1	0.27	0.57	0	0	0	0
GAIF01020352.1	0	0	0	0.05	0.05	0
GAIF01020355.1	33.49	21.27	36.63	97.35	165.05	104.51
GAIF01020356.1	0	0	0	0.23	0.67	0
GAIF01020360.1	1.23	1.47	1.77	2.1	2.39	0.49
GAIF01020361.1	6.15	6.01	6.29	16.3	16.22	3.03
GAIF01020363.1	0	0	0	0	0.32	0
GAIF01020365.1	0	0	0	0	0.08	0
GAIF01020367.1	0.86	0.38	0	8.49	16.48	0.06
GAIF01020368.1	4.42	6.31	3.64	1.07	1.44	0.41
GAIF01020369.1	17.48	9.5	1.6	6.76	1.75	3.21
GAIF01020370.1	3.08	1.02	0	2.11	4.62	4.57
GAIF01020371.1	8.69	11	9.04	19.33	11.44	3.53
GAIF01020372.1	17.61	19.75	17.75	40.88	18.25	5.49
GAIF01020373.1	5.25	23.99	2.5	15.65	39.17	0.39
GAIF01020374.1	0	0	0	1.07	2.78	0
GAIF01020375.1	1.69	0	0	6.05	36.4	1.24
GAIF01020376.1	2.96	2.6	2.09	7.19	5.69	1.54
GAIF01020377.1	0.58	0.36	0	0.29	1.72	3.57
GAIF01020378.1	0	0	0	0	0.04	0
GAIF01020379.1	0	0	0	1.64	1.06	0

GAIF01020380.1	0.04	0	0	0	0	0
GAIF01020381.1	0	0	0	0	0.39	0
GAIF01020382.1	67.75	34.19	30.12	41.75	14.11	5.81
GAIF01020383.1	10.24	8.05	4.38	40.51	72.1	1.99
GAIF01020385.1	23.18	34.61	57.63	16.65	13.34	11.07
GAIF01020386.1	1.02	0	0	2.27	6.05	18.31
GAIF01020387.1	0	0	0	0.08	0	0
GAIF01020390.1	0.25	0.04	0.2	0.45	0.29	0
GAIF01020391.1	0.62	0.89	1.18	0.6	0.25	0.62
GAIF01020392.1	31.14	16.8	9.45	11.27	2.82	1.32
GAIF01020394.1	0	0	0	0.53	0.31	0
GAIF01020396.1	0	0.12	0.03	0.1	0.23	0
GAIF01020397.1	0	0	0	0.09	0.21	0
GAIF01020399.1	14.74	9.87	4.86	22.38	15.07	4.48
GAIF01020400.1	0	0	0	4.49	0	0.8
GAIF01020401.1	0	0.11	0	8.82	3.83	0.21
GAIF01020403.1	0	0.08	0	0	2.38	0
GAIF01020404.1	0	0.01	0	0	0	0
GAIF01020405.1	0.31	0.51	0	7.12	17.97	0
GAIF01020406.1	48.02	28.31	11.37	13.02	27.9	2.92
GAIF01020407.1	0.13	0	0	0	0.48	0.29
GAIF01020408.1	1.4	0.72	0.08	2.74	2.53	0.11
GAIF01020411.1	0.87	1.37	2.19	1.61	6.25	0.29
GAIF01020412.1	0.08	0	0.17	0	0	0.08
GAIF01020413.1	59.93	116.44	77.39	90.33	107.04	38.4
GAIF01020414.1	0	0	0	0	0.59	0
GAIF01020415.1	0	0.35	0.14	0.97	5.95	1.7
GAIF01020419.1	0	0.17	0	1.09	2.16	1.21
GAIF01020420.1	0.05	0	0.03	0.48	3.86	0.05
GAIF01020421.1	0	0.42	1.07	0	0	1.07
GAIF01020422.1	0.53	0	0	0.62	4.47	0.19
GAIF01020426.1	78.61	65.58	89.7	93.08	69.25	46.59
GAIF01020427.1	0.37	0	0	3.43	24.11	0.14
GAIF01020428.1	0.21	0.19	0	0.43	0.79	0
GAIF01020429.1	0	0	0	0	0.88	0
GAIF01020430.1	0.18	0.03	0	0.08	0.16	0.03
GAIF01020432.1	12.67	12.95	11.17	20.59	30.79	5.18
GAIF01020433.1	0	0	0	0	0	1.63
GAIF01020434.1	0	0	0.11	0.12	0.36	0
GAIF01020435.1	0.5	0.12	0.08	2.7	15.14	1.12
GAIF01020436.1	0	0.23	0.12	0	0	0
GAIF01020437.1	0	0	0	0.38	0	0
GAIF01020438.1	0.04	0	0	0.4	2.22	0.42
GAIF01020439.1	0	0	0	0.09	0	0.25

GAIF01020440.1	0	0	0	0.08	0	0.06
GAIF01020442.1	5.24	0.86	1.22	21.92	78.25	10.14
GAIF01020446.1	11.05	7.62	8.85	31.72	4.92	9.18
GAIF01020447.1	0	0	0	0	0.01	0
GAIF01020448.1	0	0.3	1.22	0	0	0.1
GAIF01020452.1	7.82	4.56	0	17.07	32.09	6.99
GAIF01020453.1	0.21	0.12	0.12	0.03	0	0
GAIF01020455.1	155.81	44.68	13.29	132.05	126.91	15.28
GAIF01020456.1	0	0.07	0	0	0	0
GAIF01020457.1	9.78	12.11	18.86	48.4	41.26	9.65
GAIF01020458.1	25.79	63.61	46.2	80.74	57.95	18.95
GAIF01020460.1	0.28	0.31	0.19	0.16	0.59	0.12
GAIF01020462.1	0	0.28	0.42	0	0.15	6.03
GAIF01020464.1	0	0	0	0.08	0.08	0
GAIF01020465.1	3.27	1.44	0.22	16.7	55.67	0.56
GAIF01020466.1	0.74	1.07	0.27	1.38	4.43	0.05
GAIF01020467.1	1.17	1.97	1.73	2.11	0	2.91
GAIF01020468.1	0	0	0	0	0.07	0
GAIF01020469.1	9.83	2.28	1.27	1.51	1.01	0
GAIF01020470.1	24.1	26.42	31.87	113.29	105.91	20.61
GAIF01020472.1	0.43	0.09	0	0.1	1.76	0.38
GAIF01020473.1	18.31	24.64	23.17	23.38	14.59	8.53
GAIF01020474.1	640.53	219.01	148.82	8.15	4.51	1.97
GAIF01020475.1	2.25	0.64	0	0	1.32	0.39
GAIF01020476.1	0	0	0	0	0.49	0
GAIF01020477.1	0	0	0	0.55	1.39	0
GAIF01020478.1	270.1	225.37	145.07	237.05	335.99	953.8
GAIF01020479.1	368.67	697.6	442.95	423.79	966.98	323.57
GAIF01020480.1	0	0	0.06	0	0	0
GAIF01020482.1	0	0	0	0.09	0.06	0
GAIF01020484.1	0	0	0	0	0.26	0
GAIF01020485.1	0	11.81	33.25	0	1.19	30.71
GAIF01020486.1	9.09	11.61	4.59	5.69	6.25	1.11
GAIF01020488.1	0.36	0.26	0.2	0.69	0.58	0.39
GAIF01020490.1	0.25	0.4	0.95	0.72	1.04	0.27
GAIF01020491.1	60.82	83.99	104.21	94.41	65.44	16.44
GAIF01020492.1	0	0	0	0.48	3.96	0
GAIF01020493.1	0	0	0.07	0.28	2.18	0.07
GAIF01020495.1	0	0	0	0.01	0.01	0
GAIF01020496.1	0	0.04	0	0.08	2.46	0.02
GAIF01020497.1	12.67	8.32	8.4	15.67	20.89	6.28
GAIF01020498.1	40.46	48.42	42.57	78.12	64.08	33.12
GAIF01020499.1	13.69	15.1	0	15.45	77.16	16.08
GAIF01020500.1	11.18	7.37	4.41	59.92	96.93	4.02

GAIF01020501.1	0	0.08	0.15	0.08	0.33	0
GAIF01020502.1	35.2	40.38	52.01	23.87	19.14	17.5
GAIF01020503.1	0	0	0	1.57	3.75	0
GAIF01020504.1	2.44	4.89	0.58	7.6	15.17	0
GAIF01020505.1	0	0.56	0.19	0	0	0
GAIF01020506.1	0	0.18	0	0	0.13	0
GAIF01020507.1	0.09	0	0	0	0	0
GAIF01020508.1	0	0	0.07	0.12	1.12	0
GAIF01020509.1	2.35	1.8	4.08	1.24	0.85	1.28
GAIF01020510.1	0	0	0	0.1	0	0
GAIF01020511.1	2.39	1.75	0	11.82	23.65	3.13
GAIF01020512.1	0	0	0	0	0.06	0
GAIF01020514.1	0.17	0.28	0.09	0	0	0
GAIF01020515.1	0	0	0	0	0.44	0.37
GAIF01020518.1	4.42	1.95	2.69	1.34	1.58	1.05
GAIF01020519.1	36.18	33.1	5.74	67.24	48.97	3.3
GAIF01020520.1	13.47	0	0	3.43	3.78	27.8
GAIF01020521.1	0.7	0.43	0.37	0.43	0.29	0.1
GAIF01020523.1	6.63	8.43	7.63	12.05	6.24	4.75
GAIF01020524.1	9	4.32	1.93	23.4	45.81	3.7
GAIF01020525.1	0	0	0.03	0	0.21	0
GAIF01020526.1	3.09	0	0.64	0.22	0.46	0
GAIF01020527.1	0.01	0	0	0.01	0.12	0
GAIF01020528.1	25.14	27.07	12.39	79.85	72.02	2.95
GAIF01020529.1	4.89	9.9	5.07	12.35	9.55	1.13
GAIF01020530.1	5.03	2.76	2.09	0.95	1.03	0.3
GAIF01020532.1	4.1	3.75	1.23	11.78	22.62	1.33
GAIF01020534.1	3.97	0.44	0	23.09	107.9	23.14
GAIF01020535.1	0.05	0	0	0	0.06	0
GAIF01020536.1	0	0.05	0	0.06	1.07	0
GAIF01020538.1	0.31	0.25	0.09	0.96	8.45	0.17
GAIF01020541.1	12	2.54	1.03	0.36	0.18	0.34
GAIF01020542.1	0.14	0	0	0.25	0.05	0.07
GAIF01020543.1	0.19	0	0	1.1	4.47	0.28
GAIF01020544.1	40.83	105.07	103.45	367.23	331.23	36.54
GAIF01020545.1	0	0	0	0.05	0.56	0.03
GAIF01020546.1	2.14	1.98	0.46	1.54	0.55	0
GAIF01020547.1	0.88	0.1	0	0	0	0.02
GAIF01020548.1	17.11	24.76	18.4	36.78	26.9	8.26
GAIF01020552.1	0	0.12	0	0	0	0
GAIF01020553.1	36.3	32.7	50.12	51.81	28.29	34.6
GAIF01020555.1	0.08	0.17	0.25	1.08	5.86	0.34
GAIF01020557.1	119.79	118.79	107.77	475.26	271.44	88.68
GAIF01020558.1	0	0.06	0.06	0.07	0	0.06

GAIF01020559.1	4.78	0.95	0	1.55	4.12	0
GAIF01020560.1	10.01	5.25	3.03	14.82	22.05	4.59
GAIF01020561.1	0	0.18	0.2	2.01	0.2	0.49
GAIF01020564.1	0.09	0	0.05	0	0.54	0.36
GAIF01020568.1	0	0	0	0.39	5.89	0.07
GAIF01020569.1	262.55	172.94	111.92	286.53	339.37	438.15
GAIF01020571.1	0	0	0.56	3.83	0	0
GAIF01020574.1	32.58	26.72	18.27	92.68	77.71	17.57
GAIF01020575.1	8.74	28.51	0	0	10.51	0
GAIF01020577.1	1.03	0.63	0.73	1.19	0.44	0.28
GAIF01020578.1	0.61	0.37	0.49	3.32	9.76	1.2
GAIF01020579.1	27.6	91.44	33.13	23.89	71.92	6.96
GAIF01020580.1	0	0.59	0.49	0	0	0
GAIF01020581.1	0.17	0	0.14	0.34	0.76	0
GAIF01020582.1	0.82	1.04	2.71	0	0.16	0.91
GAIF01020583.1	0	0.05	0	0	0	0
GAIF01020585.1	0.95	2.19	0.5	1.91	2.33	0.18
GAIF01020586.1	0	0	0	0	2.21	0
GAIF01020587.1	0	0	0.26	0.55	0.14	0
GAIF01020589.1	0.22	0	0	0	0.95	0
GAIF01020592.1	0	0.56	0	0	0	0
GAIF01020594.1	0	0	0	0.15	0	0
GAIF01020595.1	0	0.06	0	0	0	0
GAIF01020596.1	10.24	14.11	18.52	30.29	13.53	6.12
GAIF01020597.1	0.11	0	0	0.4	2.23	0
GAIF01020598.1	0	0	0.12	0	0.13	0
GAIF01020599.1	5.88	3.81	9.38	23.89	7.41	10.19
GAIF01020600.1	25.11	20.78	20.08	23	14.39	9.71
GAIF01020602.1	0	0	0	0	0.06	0
GAIF01020605.1	39.4	18.25	15.45	67.12	98.11	50.82
GAIF01020606.1	0.34	0.12	0	3.68	3.4	5.36
GAIF01020607.1	0	0.06	0.03	0	0	0
GAIF01020609.1	500.67	235.64	330.08	307.64	51.12	91.2
GAIF01020611.1	0.12	0.13	0	1.81	12.72	0.4
GAIF01020616.1	0	0	0.09	0	0.06	0
GAIF01020618.1	0.47	0.85	0.55	0.82	1.15	0.15
GAIF01020619.1	1.61	2.32	2.73	1.09	2.27	1.07
GAIF01020620.1	4.5	4.81	0.97	12.78	19.53	0.35
GAIF01020622.1	14.95	0	0	15	2.93	15.07
GAIF01020626.1	15.66	26.19	21.16	66.87	57.52	11.91
GAIF01020627.1	0.09	0.1	0	0.54	0.56	0
GAIF01020628.1	285.43	150.22	144.83	141.25	151.36	1154.73
GAIF01020629.1	0	0	0	0	0.14	0
GAIF01020630.1	0.64	1.27	0.05	0.23	0.47	0.22

GAIF01020631.1	8.53	7.52	0.93	17.51	25.42	4.9
GAIF01020632.1	0	0.03	0.14	0	0	0.11
GAIF01020633.1	468.45	230.58	132.81	815.66	479.36	111.71
GAIF01020637.1	0	0	4.8	15.22	10.51	0
GAIF01020638.1	4.37	8.63	1.31	20.27	25.06	0.33
GAIF01020640.1	11.96	12.44	26.4	33.34	14.85	18.62
GAIF01020641.1	0.89	1.86	1.96	9.24	5.28	1.61
GAIF01020642.1	7.61	1.09	1.28	57.52	184.75	17.23
GAIF01020643.1	0.23	0	0	0.2	0.48	0.19
GAIF01020644.1	247.65	145.79	200.46	206.51	109.06	143
GAIF01020647.1	15.21	16.32	12.64	23.31	29.26	22.7
GAIF01020650.1	16.85	37.54	7.23	15	29.12	10.49
GAIF01020651.1	1.8	3.42	1.89	1.39	1.62	0.17
GAIF01020653.1	15.52	7.18	3.26	1.8	1.3	0.36
GAIF01020655.1	12.28	9.14	14.11	43.6	33.61	23.96
GAIF01020658.1	8.23	4.84	7.32	23.01	18.04	14.18
GAIF01020659.1	0.98	0.25	0.29	0.71	4.65	0
GAIF01020660.1	13.82	12.07	9.38	2.47	1.23	0.28
GAIF01020662.1	0.62	0.45	0.23	0.5	12.33	0
GAIF01020663.1	0	0	0	0	0.01	0
GAIF01020665.1	696.88	531.67	620.34	428.66	521.33	1482.04
GAIF01020668.1	76.81	136.66	230.54	98.75	86.29	58.1
GAIF01020669.1	0.03	0	0	0.47	1.62	0
GAIF01020670.1	0	0	0	0	0.03	0
GAIF01020672.1	2.68	0.74	0.25	8.41	29.08	5.09
GAIF01020674.1	0.21	0	0	0.74	2.56	0
GAIF01020675.1	0.42	0.3	0	0.16	0.5	0
GAIF01020680.1	0	0.17	0.13	0	0.02	0.03
GAIF01020681.1	0	0	0	0.36	0.44	0.07
GAIF01020684.1	245.86	74.74	64.16	110.73	159.93	296.14
GAIF01020686.1	0	0	0	0	0.09	0
GAIF01020687.1	1.45	2.54	1.15	0.37	0.1	0.8
GAIF01020688.1	0.25	0.25	0	0	0.24	0
GAIF01020690.1	0.08	0	0	1.24	3.41	0
GAIF01020691.1	0	0.07	0	0.08	0.08	0
GAIF01020692.1	12.06	16.51	3.98	45.06	44.94	2.5
GAIF01020693.1	7.51	5.53	7.01	13.37	13.86	7.23
GAIF01020694.1	7.52	6.37	8.55	20.33	16.02	5.84
GAIF01020695.1	18.48	8.55	6.52	31.53	40.99	17.34
GAIF01020697.1	0	0	0	0.49	0.17	0.15
GAIF01020699.1	0.11	0	0.12	2.24	9.7	0.13
GAIF01020700.1	1.97	1.13	0	4.37	3.76	0.52
GAIF01020701.1	0.44	0.48	0.36	0.13	0.13	0
GAIF01020705.1	0.83	0.13	0	1.51	5.42	2.09

GAIF01020706.1	0	0	0	0	0.22	0
GAIF01020707.1	0	0	0	0.13	0.48	0.56
GAIF01020709.1	5.32	4.58	0.77	11.87	12.68	0.73
GAIF01020710.1	25.96	17.24	25.21	38.09	18.93	18.22
GAIF01020711.1	0	0	0	0.13	0	0
GAIF01020712.1	6.3	6.82	5.76	23.39	19.98	5.59
GAIF01020715.1	129.05	142.86	200.26	562.91	276.33	182.16
GAIF01020716.1	0.37	0.8	0.61	0	0.66	0
GAIF01020717.1	0	0	0	0	0.06	0
GAIF01020719.1	1.16	0	0	4.04	0	0
GAIF01020721.1	0	0.36	0.18	0.01	0	0.02
GAIF01020722.1	0	0	0.01	0	0	0
GAIF01020723.1	0.59	0.63	0.12	3.44	5.82	0.1
GAIF01020724.1	0	0	0	1.93	3.27	0.14
GAIF01020725.1	30.7	23.6	20.42	51.4	30.48	4.96
GAIF01020727.1	109.82	49.28	83.34	86.25	60.38	720.22
GAIF01020728.1	0	0	0	0	0.06	0
GAIF01020730.1	0	0	0	0.46	0.12	0
GAIF01020731.1	0	0	0	0.13	0	0
GAIF01020733.1	0.04	0	0	1.2	5.83	0.43
GAIF01020734.1	119.95	86.54	76.62	62.06	32.77	63.93
GAIF01020736.1	0	0	0	0	0.14	0.26
GAIF01020737.1	0.12	0.08	0.13	0.7	6.52	1.07
GAIF01020738.1	2.67	1.81	0	0.19	1.4	0.18
GAIF01020739.1	0	0	0.05	0	0.06	0
GAIF01020740.1	0	0.06	0.06	0.3	0.62	0
GAIF01020741.1	10.58	8.82	7.96	13.15	13.04	4.78
GAIF01020742.1	0.06	0.07	0	0.07	0	0
GAIF01020743.1	0	0	0	0.25	1.55	0.32
GAIF01020744.1	0	0	0	0.05	0.16	0
GAIF01020747.1	0	0	0	0.04	0	0
GAIF01020748.1	0.11	0.19	0.37	0.13	0.07	0.06
GAIF01020750.1	0	1.01	0.17	0.18	0	0.34
GAIF01020751.1	31.36	22.16	9.65	44.25	41.24	3.47
GAIF01020752.1	0.23	0	0.38	0	0.14	0.66
GAIF01020754.1	8.56	10.86	7.21	34.6	42.59	4.43
GAIF01020757.1	0.45	0.17	0.19	0.72	0.7	2.8
GAIF01020759.1	0.22	0	0.08	0	0.35	0.03
GAIF01020760.1	0.04	0.11	0	0.2	0	0.05
GAIF01020761.1	40.57	36.46	72.71	75.48	29.56	58.66
GAIF01020762.1	0	0	0	0.1	0	0
GAIF01020764.1	0	0.13	0.13	0	0	0
GAIF01020765.1	0	0	0.12	0	0	0.36
GAIF01020768.1	0.54	0.62	0.42	2.38	4.26	0.19

GAIF01020769.1	57.57	117.24	80.73	103.91	108.52	13.63
GAIF01020770.1	0.39	0.22	0	0	0	0
GAIF01020771.1	0.25	1.66	1.47	0.3	0	0.91
GAIF01020773.1	301.91	291.41	441.34	172.17	112.1	368.23
GAIF01020774.1	15.34	11.88	20.28	29.47	17.8	15.05
GAIF01020775.1	47.15	39.34	39.96	47.77	62.13	25.58
GAIF01020777.1	0	0	0	0	0.05	0
GAIF01020778.1	0	0	0	0.33	0.86	0.16
GAIF01020780.1	0.08	0.18	0	0	0.1	0
GAIF01020781.1	27.19	32.65	33.6	98.63	80.49	16.52
GAIF01020782.1	0.08	0.05	0	0.2	1.78	0
GAIF01020783.1	0	0	0	0.15	1.17	0
GAIF01020784.1	0	1.06	0.7	0	0	0.12
GAIF01020785.1	3.33	3.04	1.89	8.22	10.72	1.77
GAIF01020786.1	7.9	6.47	3.27	18.65	20.3	2.38
GAIF01020787.1	0.56	0	0.15	0.65	1.52	0.46
GAIF01020788.1	0.08	0	0	0	0	0
GAIF01020790.1	0	0	0	0.46	0.06	0
GAIF01020791.1	48.73	20.91	16.77	9.12	15.71	0.69
GAIF01020792.1	0.11	0	0	0.25	0	0
GAIF01020793.1	0	0	0	0.09	0.7	0
GAIF01020794.1	0	0	0	0.13	0.27	0
GAIF01020795.1	0	0.14	0	0	0	0
GAIF01020796.1	2.84	0.06	0.26	0.79	0.14	0
GAIF01020797.1	0	0.01	0.06	0	0	0.01
GAIF01020798.1	4.02	5.87	3.45	21.98	14.83	3.52
GAIF01020799.1	0.3	0.32	0	2.24	8.41	0.33
GAIF01020800.1	0	0	0	0	0.71	0
GAIF01020803.1	14.79	13.42	5.26	1.36	2.04	0.3
GAIF01020804.1	20.09	12.09	7.35	42.72	42.13	4.91
GAIF01020805.1	33.52	12.47	18.47	1.92	4.95	20.75
GAIF01020806.1	39.08	65.84	26.08	5.58	2.74	0
GAIF01020807.1	0	0.1	0	0.2	0.42	0
GAIF01020809.1	34.95	36.9	37.15	33.84	18.11	9.1
GAIF01020810.1	2.14	1.5	0.98	6.94	22.43	0.44
GAIF01020812.1	1.03	1.12	1.13	0.82	0	0.86
GAIF01020814.1	0.53	0.16	0.16	5.68	19.54	0.16
GAIF01020815.1	9.83	13	10.99	57.46	46.33	9.29
GAIF01020816.1	2.9	0	0	16.5	36.65	0
GAIF01020817.1	4.94	8.02	7.98	22.66	21.15	1.71
GAIF01020818.1	0	0.08	0	0	0	0
GAIF01020819.1	0.43	0.23	0	0.12	0	0
GAIF01020820.1	5.58	2.9	0	6.83	8.44	0
GAIF01020821.1	0	0.37	0	0	0	0

GAIF01020822.1	0	0	0	0	0.09	0
GAIF01020823.1	12.1	22.48	17.77	53.28	165.85	19.22
GAIF01020826.1	0	0	0	0	0.89	0.1
GAIF01020827.1	2.29	0.16	0.05	3.86	14.28	2.74
GAIF01020828.1	0	0	0	0	0.14	0
GAIF01020830.1	0.21	0.16	0.05	0.24	0.12	0
GAIF01020831.1	4.03	4.39	8.86	4.68	4.85	0
GAIF01020832.1	18.09	28.94	28.4	71.67	47.78	9.48
GAIF01020833.1	0	0	0	0.01	0.01	0
GAIF01020835.1	0	0	0	0	0.07	0
GAIF01020836.1	0.14	0.04	0.11	0	0.37	0.48
GAIF01020839.1	0	7.24	3.4	0	0	0.17
GAIF01020840.1	2.22	0.58	1.37	2.78	1.27	2.81
GAIF01020841.1	0	0	0.08	0	0	0
GAIF01020842.1	124.52	110.05	115.22	138.64	195.69	103.3
GAIF01020843.1	61.39	63.09	30.73	103.28	82.72	7.28
GAIF01020844.1	0.08	0	0	0	0	0
GAIF01020845.1	0.23	0	1.14	1.87	0.28	3.8
GAIF01020846.1	0.16	0.09	0.27	0.66	2.75	1.08
GAIF01020848.1	0	0	0	0.57	3.11	0
GAIF01020850.1	195.21	200.77	215.06	87.1	63.58	48.22
GAIF01020851.1	0	0.44	0.79	0	0	0
GAIF01020852.1	0.72	0	0.79	53.96	35.55	270.9
GAIF01020854.1	0.14	0	0	0	0	0
GAIF01020857.1	6.21	4.4	2.52	8.63	12.78	1.57
GAIF01020860.1	65.38	51.04	53.29	123.27	70.71	20.84
GAIF01020863.1	291.84	1.48	1.28	13.2	0.82	1.71
GAIF01020864.1	9.67	15.48	0.58	55.37	81.39	0.27
GAIF01020865.1	12.88	6.63	9.2	16.47	17.8	16.15
GAIF01020866.1	0	0	0	0	0	0.07
GAIF01020867.1	0.37	0.16	0.08	0.6	0.88	0.08
GAIF01020869.1	37.8	35.63	24	4.93	0.93	0.55
GAIF01020871.1	17.93	22.52	24.59	47.05	21.25	10.2
GAIF01020872.1	0.26	0.28	0.14	0.3	0	0.14
GAIF01020873.1	0.45	0.08	0.05	0	0.1	0.01
GAIF01020874.1	12.69	9.01	3.58	13.51	7.54	3.95
GAIF01020875.1	0.15	0.05	0	0.11	0.12	0
GAIF01020876.1	30.32	29.42	6.77	103.77	119.94	4.49
GAIF01020877.1	94.57	125.6	149.11	137.69	150.46	48.67
GAIF01020878.1	88.63	55.93	33.76	19.44	51.64	6.86
GAIF01020880.1	2.91	1.06	3.2	7.89	2.34	8.56
GAIF01020881.1	24.56	13.79	6.86	34	41.94	10.45
GAIF01020882.1	0	0	0	0.22	0	0
GAIF01020883.1	1931.86	6519.95	2229.89	1384.76	2092.21	444.71

GAIF01020884.1	0	0	0	0.11	0.11	0
GAIF01020885.1	31.35	29.32	21.19	11.07	8.23	1.76
GAIF01020886.1	0.31	0.21	0	6.4	33.46	1.67
GAIF01020888.1	0	0.38	0	0	0	0.38
GAIF01020889.1	0.18	0.06	0.2	0.09	0.06	0.12
GAIF01020891.1	0	0	0.08	0	0.13	0
GAIF01020892.1	0	0	0	0.15	0	0
GAIF01020895.1	0	0.08	0	0.08	1.26	0.15
GAIF01020896.1	0.05	0	0.11	2.51	7.93	0.53
GAIF01020897.1	26.7	0.23	2.58	18.84	7.71	51.8
GAIF01020898.1	0.51	0	0	2.63	14.37	5.24
GAIF01020899.1	0.16	0	0	1.37	7.64	0
GAIF01020901.1	0	0	0	0.12	0.13	0
GAIF01020903.1	71.18	70.59	36.06	17.55	8.22	1.8
GAIF01020904.1	10.16	5.9	4.99	38.97	39.23	5.35
GAIF01020905.1	12.36	7.22	12.14	23.16	14.82	33.37
GAIF01020906.1	4.32	1.53	0	6.4	10.06	0.92
GAIF01020907.1	0	2.05	0	0	12.82	4.02
GAIF01020909.1	0.56	0.24	0	1.43	1.35	0.25
GAIF01020910.1	0	0	0	0	0.08	0
GAIF01020911.1	49.07	22.68	14.38	109.54	56.4	102.63
GAIF01020912.1	10.03	12.03	5.64	1.72	0.9	0.45
GAIF01020913.1	0	0	0	0.02	0.11	0
GAIF01020914.1	0.69	0.29	0	1.68	3.24	0
GAIF01020917.1	0	0	0.08	0.68	0.97	0.08
GAIF01020921.1	0	0	0	0.33	2.4	0
GAIF01020922.1	1.99	0.93	3.58	1.65	1.02	0.16
GAIF01020923.1	13.68	10.14	5.66	43.4	53.09	5.29
GAIF01020925.1	0.01	0.03	0	0.07	0.1	0
GAIF01020926.1	1.79	1.58	0.19	17.92	55.88	1.78
GAIF01020927.1	0.96	0.3	0	0.79	1.81	0.3
GAIF01020930.1	17.38	15.45	14.33	10.64	7.51	3.71
GAIF01020931.1	29.62	19.69	7.18	51.21	60.28	23.86
GAIF01020932.1	11.11	14.79	25.71	36.39	16.81	15.24
GAIF01020933.1	0.32	0	0.12	0.62	2.56	0
GAIF01020934.1	2.19	0	0.25	0.47	3.76	0.18
GAIF01020936.1	0.06	0.07	0	0.74	2.6	0.14
GAIF01020937.1	0.17	0.17	0.38	0	1.49	1.18
GAIF01020940.1	4.96	14.05	6.52	12.89	17.76	1.81
GAIF01020943.1	16.73	5.35	2.4	52.96	66.54	7.29
GAIF01020944.1	0	0	0	0	11.78	0
GAIF01020945.1	4.49	0.2	0.1	4.13	5.74	5.47
GAIF01020947.1	0.86	0	0	0.33	2.06	0.94
GAIF01020948.1	0	0.04	0.17	0	0	0

GAIF01020950.1	0.16	0.23	0.12	1.88	2.76	0.18
GAIF01020951.1	208.56	214.34	186.89	56.76	26.27	8.93
GAIF01020952.1	4.63	1.31	0.22	0.47	0.85	0.44
GAIF01020953.1	7.19	10.89	5.59	9.9	18.04	2.02
GAIF01020954.1	0.72	0.22	0	6.83	17.97	0.34
GAIF01020955.1	0.23	0.04	0.38	0.47	0.09	1.21
GAIF01020956.1	37.07	24.76	33.03	58.46	37.58	57.89
GAIF01020959.1	0.09	0.03	0.05	0.31	1.58	0.81
GAIF01020960.1	0	0	0	0.35	0	0
GAIF01020963.1	0.23	0.17	0	0.82	4.51	0
GAIF01020965.1	24.1	31.57	11.13	24.87	62.24	14.26
GAIF01020966.1	0	0	0.04	0	0	0
GAIF01020968.1	3.22	0.23	0.12	11.46	73.21	1.06
GAIF01020969.1	0.08	0	0	0.09	0.33	0
GAIF01020970.1	11.76	21.08	20.76	19	16.49	3.45
GAIF01020971.1	7.24	6.93	7.03	27.57	23.67	3.73
GAIF01020972.1	0.6	0.95	0.06	5.1	6.71	1.47
GAIF01020973.1	10.24	3.53	3.37	17.73	19.68	7.31
GAIF01020974.1	312.8	389.71	230.69	266.13	219.84	163.53
GAIF01020975.1	0.14	0	0	0.46	2.06	0.11
GAIF01020976.1	4.69	2.42	7.59	2.29	1.78	0.54
GAIF01020977.1	107.26	83.36	64.16	59.08	82.68	49.42
GAIF01020978.1	1.66	1.41	1.92	2.7	1.81	2.39
GAIF01020980.1	1	1.01	0.68	3.23	1.3	0.43
GAIF01020981.1	0.69	0.15	0.15	2.09	12.66	1.53
GAIF01020982.1	1.22	0.09	0	2.83	11.43	5.29
GAIF01020984.1	0	0	0	0.12	1.77	0
GAIF01020985.1	0.63	0	0.17	0.55	2.67	0.17
GAIF01020986.1	0	0	0	0.14	0.31	0
GAIF01020988.1	97.55	112.52	43.41	114.52	124.61	25.35
GAIF01020989.1	10.87	10.46	6.42	45.73	24.04	5.47
GAIF01020990.1	0.09	0	0	0	0	0
GAIF01020991.1	0.18	1.06	0.29	0.82	2.97	0
GAIF01020992.1	0	0.3	0	2.88	8.41	0.17
GAIF01020994.1	0.24	0.07	0.46	0.07	0.07	0.13
GAIF01020997.1	413.84	396.52	402.76	882.3	925.43	1061.66
GAIF01020998.1	0.89	0.14	0.22	4.8	21.3	0.28
GAIF01020999.1	0	0	0	0	0.12	0
GAIF01021001.1	8.48	5.19	4.16	16.41	32.68	5.23
GAIF01021002.1	0.31	0	0	1	11.13	0.09
GAIF01021003.1	0	0	0	0	0.19	0
GAIF01021004.1	8.15	6.3	8.25	16.7	17.09	10.47
GAIF01021005.1	3.55	3.96	0.22	13.26	20.61	0.33
GAIF01021006.1	2.9	2.49	2.52	0.47	0.32	0.59

GAIF01021010.1	33.53	25.38	28.81	48.55	56.05	65.61
GAIF01021011.1	140.36	137.2	115.64	208.48	180.08	144.14
GAIF01021012.1	0.28	0.08	0.04	0.81	4.2	0.15
GAIF01021014.1	0	0	0.04	0	0	0
GAIF01021015.1	0.15	0.04	0.08	0	0.02	0.01
GAIF01021017.1	0	0.49	0.28	0.33	0	0.43
GAIF01021021.1	2.69	5.76	3.77	4.21	2.68	0.1
GAIF01021022.1	97.1	99.23	63.29	168.82	155.79	36.42
GAIF01021023.1	0	0	0	0	0.25	0
GAIF01021024.1	0	0	0	0.01	0.01	0
GAIF01021025.1	0	0.06	0.06	0.06	0.07	0
GAIF01021026.1	0.07	0	0	0	0	0
GAIF01021027.1	5933.86	6704.03	8958.67	3637.62	2890.18	7756.05
GAIF01021028.1	6.04	1.03	0.47	9.12	26.37	5.61
GAIF01021029.1	0	0	0	0	3.26	0
GAIF01021030.1	72.1	83.76	37.66	49.05	38.26	7.1
GAIF01021031.1	0.13	0.09	0	0.47	0.69	0.05
GAIF01021032.1	0	2.53	0.91	0	0.09	0.41
GAIF01021034.1	0.3	0	0.11	0.1	0.24	0.11
GAIF01021035.1	0.15	0	0.09	0.19	0.42	0.09
GAIF01021038.1	0	0	0	0	0.17	0
GAIF01021039.1	17.13	19.95	14.33	42.83	32	4.94
GAIF01021040.1	23.66	27.18	25.65	44.03	21.47	7.21
GAIF01021042.1	0.71	0.64	0.13	0.2	0.07	0
GAIF01021043.1	0.29	0.21	0	0	0	0
GAIF01021044.1	24.06	3.14	0.81	48.1	97.41	8.45
GAIF01021046.1	1.85	0.98	0	17.64	44.98	3.42
GAIF01021047.1	0.1	0.08	0	0.68	8.8	0.88
GAIF01021048.1	0.15	0	0	0	3.53	0.16
GAIF01021049.1	18.03	17.55	8.37	34.78	42.16	2.38
GAIF01021051.1	0.63	0.17	0	0.37	0	0.17
GAIF01021052.1	0	0	0	1.31	9.82	0
GAIF01021053.1	0	0	0	1.24	3.87	0.24
GAIF01021054.1	0	0.28	0	0	1.84	0
GAIF01021055.1	39.82	55.21	96.25	26.27	23.63	69.75
GAIF01021057.1	8.46	8.95	1.02	20.44	20.63	0
GAIF01021058.1	3.76	2	0.67	1.25	0.09	0
GAIF01021059.1	0	0	0	0	0.07	0
GAIF01021061.1	20.37	23.08	44.93	11.73	15.91	13.14
GAIF01021062.1	0.1	0	0	0	0.38	0
GAIF01021065.1	0.81	0.2	0.17	2.1	4.78	1.16
GAIF01021066.1	20.2	40.94	7.42	10.26	23.07	1.49
GAIF01021068.1	17.46	15.07	23.85	11.14	13.95	23.21
GAIF01021069.1	0	0	0	0	0.56	0

GAIF01021070.1	4.96	1.47	1.93	0.22	0.18	0.25
GAIF01021071.1	0	0	0	0	0	0.06
GAIF01021072.1	61.37	51.18	69.04	117.48	49.3	68.05
GAIF01021073.1	0.13	0	0.14	0.15	0.15	0
GAIF01021074.1	3.39	0	0.48	0	0.13	0
GAIF01021076.1	0	0.15	0	0.33	3.94	0
GAIF01021077.1	10.24	7.01	3.4	33.2	34.51	2.13
GAIF01021078.1	1.2	0.95	1.03	2.41	2.75	1.62
GAIF01021080.1	3.82	6.24	5.68	14.57	20.71	1.31
GAIF01021081.1	20.38	14.08	17.64	45.77	25.28	31.01
GAIF01021082.1	2.46	0.91	1.46	1.5	0.06	0.18
GAIF01021083.1	0.03	0.21	0.11	0.02	0.07	0.19
GAIF01021084.1	1.31	0.49	0.67	2.05	10.1	16.12
GAIF01021085.1	4.43	1.41	4.59	8.07	8.17	0
GAIF01021086.1	1.17	3.59	3.26	0.76	0.4	0.61
GAIF01021088.1	0	5.47	10.69	0	0.13	9.64
GAIF01021091.1	0	0	0	0	0.12	0
GAIF01021092.1	0	0	0	0	0.71	0.15
GAIF01021093.1	348.87	544.79	213.61	320.29	367.95	23.06
GAIF01021094.1	2.87	2.43	0.99	5.06	10.23	7.5
GAIF01021095.1	0	0	0.14	0.15	0.95	0
GAIF01021098.1	15.82	23.59	4.95	27.84	30.83	0.39
GAIF01021099.1	0.57	0.26	0.43	0	0.26	0.03
GAIF01021100.1	40.29	40.14	36.28	107.97	47.37	21.43
GAIF01021101.1	0.21	0.42	0.69	0.51	0.35	0
GAIF01021102.1	16.42	4.85	3.03	16.24	13.33	4.82
GAIF01021103.1	53.6	62.1	162.64	43.71	43.56	201.6
GAIF01021104.1	0	0	0	0	0.5	0
GAIF01021105.1	11.56	18.09	10.53	26.21	25.31	6.17
GAIF01021106.1	0.1	0	0.33	0.12	0.12	0
GAIF01021107.1	0.31	0.07	0	1.38	9.34	0
GAIF01021109.1	34.51	0.81	3.48	32.1	25.23	33.36
GAIF01021110.1	259.41	135.57	96.52	53.28	74.95	44.66
GAIF01021111.1	0	0	0	0	0.54	0.25
GAIF01021112.1	0.13	0.1	0.19	0.05	0.26	0
GAIF01021113.1	0	0	0	0.08	0.26	0
GAIF01021114.1	4.37	16.54	8.79	33.82	36.13	2.69
GAIF01021115.1	94.69	72.54	69.24	81.07	37.76	11.92
GAIF01021116.1	0	0	0.18	0.75	1.74	0
GAIF01021117.1	45.53	76.04	35.14	7.48	3.43	4.19
GAIF01021118.1	3.82	4.74	5.48	15.5	12.06	14.52
GAIF01021119.1	6.56	5.89	6.3	19.04	7.3	9.67
GAIF01021120.1	264.08	181.88	129.63	504.18	373.12	85.37
GAIF01021122.1	0	0.11	0	0.11	0.12	0

GAIF01021123.1	1.92	0.21	0.11	4.09	16.29	0.64
GAIF01021125.1	1.51	0.14	0	2.78	18.82	1.53
GAIF01021126.1	0	0	0	0	2.67	0
GAIF01021127.1	0	0.13	0.04	0	0.94	0
GAIF01021128.1	4.88	4.05	5.1	16.96	14.95	3.22
GAIF01021129.1	0	0	0	0.43	1.54	0.12
GAIF01021130.1	0	0	0.09	0	0.4	0
GAIF01021133.1	31	43.58	63.55	23.81	15.32	23.27
GAIF01021134.1	0	0	0.07	0	0.07	0
GAIF01021136.1	5.11	10.81	24.58	5.7	4.2	8.85
GAIF01021139.1	41.05	35.47	14.06	54.14	40.96	4.35
GAIF01021140.1	3.61	0.64	1.13	2.21	1.38	0.36
GAIF01021144.1	0	0	0	0	0.11	0
GAIF01021146.1	0	0.73	0.37	0	0	0
GAIF01021147.1	0.03	0.07	0.1	0.04	0.08	0
GAIF01021148.1	1.42	0.89	1.25	2.43	4.81	1.91
GAIF01021149.1	11.13	0	0.01	0.01	0.01	0
GAIF01021150.1	0.61	0.53	0.73	0.47	1.72	0
GAIF01021151.1	0	0	0	0.32	1.65	0.24
GAIF01021152.1	22.58	26.3	16.28	35.47	31.12	5.52
GAIF01021154.1	0	0	0	0.06	0.07	0
GAIF01021155.1	0.05	0	0	0.2	1.05	0
GAIF01021157.1	3.93	1.57	0.34	6.2	3.09	0
GAIF01021158.1	42.03	23.67	4.98	64.1	26.57	13.86
GAIF01021159.1	0.25	0.28	0	2.51	14.56	0.56
GAIF01021160.1	0	0.13	0	0	0.29	0
GAIF01021161.1	0.21	0	0.12	0.97	2.65	0.12
GAIF01021162.1	0.31	2.2	0	2.51	5.49	0
GAIF01021163.1	11.72	7.94	5.65	62.38	68.06	4.92
GAIF01021166.1	0.57	0.61	0.1	1.12	0.22	0
GAIF01021167.1	1422.77	1393.69	540.62	1278.54	1224.46	354.91
GAIF01021168.1	10.8	4.24	7.07	50.18	78.84	30.43
GAIF01021169.1	0.07	0	0	0	0	0
GAIF01021170.1	0.17	0.06	0	1.01	5.74	0.19
GAIF01021171.1	1.23	0.54	0	2.36	12.27	0.61
GAIF01021172.1	0.71	0.09	0.17	1.57	7.58	0.24
GAIF01021173.1	0	0.1	0	0	0	0
GAIF01021177.1	1.37	0.62	0	1.67	5.23	0
GAIF01021178.1	0.05	0	0	0.05	0	0
GAIF01021179.1	148.55	108.52	68.73	85.72	36.79	13.9
GAIF01021180.1	0.44	0.29	0	0.81	6.84	0.39
GAIF01021181.1	0.61	0	0	1.56	5.28	0.27
GAIF01021182.1	0.1	0	0	0	0.12	0
GAIF01021183.1	0.41	0	0.22	5.66	21.74	0.11

GAIF01021184.1	0	0	0	0	0.5	0
GAIF01021185.1	0	0	0	0	0.15	0
GAIF01021186.1	0	0.41	0.14	0.15	0.61	0.14
GAIF01021188.1	1.88	4.92	1.82	5.76	4.78	0.12
GAIF01021191.1	34.26	29.17	10.54	88.42	111.6	34.23
GAIF01021192.1	3.55	7.55	6.81	16.42	12.43	3.1
GAIF01021194.1	0.42	0.08	0	1.37	3.92	0
GAIF01021195.1	0	0	0	0	1.02	0.15
GAIF01021196.1	0	0	0	0	0.48	0
GAIF01021197.1	1.87	0	0	0	0	0
GAIF01021198.1	12.16	3.58	1.9	23.44	25.94	10.09
GAIF01021200.1	2.43	5.67	4.79	13.65	8.9	0.8
GAIF01021203.1	0.31	0.8	0.23	0.73	0.5	0.12
GAIF01021204.1	0.15	0.17	0	0	0.18	0
GAIF01021205.1	1.08	15.32	91.09	0	2.78	103.52
GAIF01021206.1	6.45	8.04	5.83	12.43	5	3.96
GAIF01021207.1	0	0	0	1.31	7.37	0
GAIF01021208.1	0	0	0.01	0.12	0.5	0.01
GAIF01021209.1	8.08	13.92	11.12	15.9	14.39	4.86
GAIF01021210.1	0	0	0	0	0.32	0
GAIF01021212.1	0.55	1	0.6	2.61	3.06	0.07
GAIF01021213.1	0	0	0	0.17	0.18	0
GAIF01021214.1	7.29	9.3	2.99	17.49	23.5	3.96
GAIF01021216.1	0	0.07	0	0.31	2.74	0
GAIF01021218.1	2.09	1.71	0	3.44	4.22	0.67
GAIF01021219.1	3.66	5.88	4.57	6.18	4.8	1.28
GAIF01021220.1	0.17	0.06	0.01	0	1.24	0
GAIF01021221.1	6.45	2.42	3.19	18.8	31.01	16.78
GAIF01021222.1	0.36	0.07	0	0.87	3.03	0.11
GAIF01021223.1	0	0	0	0.04	0.25	0
GAIF01021226.1	0	0	0	0	0.08	0
GAIF01021229.1	0.1	0.07	0.04	0.79	5.05	0.04
GAIF01021231.1	0.19	0	0	1.31	9.76	0.31
GAIF01021232.1	0.29	0.51	0.34	0.91	0.43	0.05
GAIF01021235.1	0	0.04	0	0	0.17	0
GAIF01021236.1	0.35	0.51	0	0.41	0.7	0
GAIF01021237.1	0.36	0.77	0.78	1.26	1.71	0.13
GAIF01021238.1	6.5	0	0	3.14	16.4	3.32
GAIF01021239.1	3.97	4.09	0.85	4.22	4.64	0.34
GAIF01021240.1	0	0	0	0	0.14	0.04
GAIF01021242.1	11.69	17.45	26.37	28.31	13.5	11.45
GAIF01021243.1	0	0.08	0	0	0	0
GAIF01021244.1	0.53	0.66	0	2.39	4.12	0.08
GAIF01021246.1	2.49	0.12	0.12	10.3	41.99	23.93

GAIF01021247.1	30.64	19.8	14.83	64.11	63.21	17.4
GAIF01021249.1	0.37	0.07	0.07	0	0	0.14
GAIF01021250.1	4.14	0.72	0.27	5.82	1.59	0
GAIF01021252.1	0	0	0	0	0.1	0
GAIF01021253.1	0.55	3.74	6.99	3.44	1.14	0.99
GAIF01021254.1	6.04	0	0	0	0	6.11
GAIF01021256.1	16.19	18.37	32.77	33.17	11.85	17.69
GAIF01021257.1	0	0	0	0.13	0.09	0
GAIF01021259.1	0	0	0	0	0.66	0
GAIF01021260.1	0.04	0.08	0.04	0.17	3.01	0.24
GAIF01021261.1	198.67	20.16	11.87	766.84	1133.84	2944.54
GAIF01021263.1	2.03	0.19	0.13	7.68	28.54	0.53
GAIF01021265.1	3.88	2.19	4.57	1.77	1.07	2.35
GAIF01021266.1	5.72	6.94	15.86	24.31	10.64	9.22
GAIF01021267.1	0.46	1	0.44	3.12	4.07	0.5
GAIF01021268.1	0.08	0	0	0.31	2.34	0.03
GAIF01021270.1	0.07	0.15	0.07	1.5	3.65	0
GAIF01021272.1	722.26	767.35	1577.24	454.23	227.81	1074.39
GAIF01021273.1	0.15	0	0	0.69	3.94	0.16
GAIF01021274.1	0.04	0.18	0	1.05	5.8	0.09
GAIF01021275.1	1057.94	3389.06	1105.28	303.8	717.67	534.57
GAIF01021276.1	1.23	0.5	0.08	4.72	8.31	0.68
GAIF01021277.1	15.99	14.17	11.38	16.85	17.92	6.86
GAIF01021278.1	0	0.23	0	0	0.16	0.11
GAIF01021282.1	0	0	0.07	0	0	0.15
GAIF01021283.1	57.74	69.35	46.26	41.73	40.97	62.55
GAIF01021284.1	0	0.25	0	0.13	0.27	0
GAIF01021286.1	5.24	0.26	0.26	11.79	64.17	48.75
GAIF01021287.1	85.69	48.51	75.16	157.7	73.14	269.94
GAIF01021288.1	49.02	16.72	16.67	72.91	47.5	80.39
GAIF01021289.1	0.16	0.13	0	0	0.29	0.04
GAIF01021290.1	11.43	10.6	11.03	39.55	38.05	19.32
GAIF01021291.1	60.78	42.55	17.28	4.77	4.4	8.36
GAIF01021292.1	0	0	0	0.11	1.33	0.1
GAIF01021296.1	497.79	550.21	835.97	594.8	495.79	899.18
GAIF01021297.1	0	0.26	0	0.28	1.46	0
GAIF01021298.1	0.33	0	0	0	0	0
GAIF01021299.1	0	0	0	0.21	0.44	0
GAIF01021300.1	0	0	0.07	0	0	0
GAIF01021301.1	1.5	0.7	1.18	0	0.7	0.47
GAIF01021302.1	24.19	19.28	21.74	22.82	12.63	12.21
GAIF01021303.1	9.29	5.87	3.76	48.18	32.33	3.34
GAIF01021304.1	0.35	0	0	0.57	0.27	0
GAIF01021306.1	1.22	0.33	0.56	4.85	15.94	1.12

GAIF01021307.1	29.13	11.25	17.13	42.98	40.99	78.73
GAIF01021308.1	37.03	22.83	7.53	45.38	58.35	22.41
GAIF01021310.1	44.75	30.98	9.96	46.89	39.5	6.39
GAIF01021311.1	6.38	4.72	6.23	16.64	22.01	11.94
GAIF01021313.1	0.44	0.36	0	3.06	5.28	0
GAIF01021314.1	0.76	0.29	0.1	1.02	1.31	0.29
GAIF01021316.1	0.07	0	0	0.04	0	0
GAIF01021319.1	0.34	0.19	0	2.24	11.45	0.19
GAIF01021320.1	30.05	72.6	42.33	65.55	59.02	5.78
GAIF01021321.1	1.14	0.11	0	2.42	0.34	1.16
GAIF01021324.1	0.25	0.53	0.4	0.28	0.3	0
GAIF01021327.1	0.09	0.4	0.2	0.11	0.22	0.1
GAIF01021328.1	0.57	0.21	0.28	2.81	5.67	0.91
GAIF01021329.1	0	0	0	0.21	0.91	0
GAIF01021330.1	0.39	0	0	0	0	0.14
GAIF01021331.1	0	0	0	0.11	0.45	0
GAIF01021332.1	0	0	0	0.06	8.06	0
GAIF01021333.1	0.03	0	0.03	0	0	0
GAIF01021334.1	13.58	9.2	11.17	20.64	22.54	7.97
GAIF01021335.1	2.17	1.34	1.43	9.83	12.12	1.44
GAIF01021336.1	0.28	1.68	0.62	0.33	1.18	0.31
GAIF01021337.1	0.38	0.07	0	0.07	0.15	0
GAIF01021338.1	11.03	7.96	4.65	0.58	1.13	0.25
GAIF01021339.1	0.13	0.14	0	0	0.31	0
GAIF01021341.1	0.06	0.03	0.06	0.71	2.54	0.06
GAIF01021342.1	30.51	50.64	22.26	27.44	35.29	31.69
GAIF01021343.1	0.18	0.43	0.1	0.08	0.48	0.1
GAIF01021344.1	20.93	21.93	14.97	37.18	30.89	4.62
GAIF01021345.1	13.32	5.38	5.05	22.08	24.25	4.99
GAIF01021346.1	2.22	3.09	0.46	5.16	10.1	0.31
GAIF01021347.1	18.15	13.93	16.73	10.79	13.78	5.94
GAIF01021348.1	0	0	0.1	0	0	0
GAIF01021349.1	0	0	0	0.44	0	0
GAIF01021352.1	2.55	0.94	0.51	22.7	46.41	0.26
GAIF01021353.1	1.01	0.56	0.3	14.12	91.94	1.56
GAIF01021354.1	0.1	0	0	0	1.86	0
GAIF01021355.1	0.12	0.39	0	0	0.29	0
GAIF01021357.1	0.83	0.38	1.3	0.14	0	0.13
GAIF01021358.1	7.9	0.78	0.64	23.24	25.43	50.23
GAIF01021359.1	10.97	21.33	10.53	51.8	61.79	4.04
GAIF01021361.1	4.67	4.9	3.55	21.86	37.15	35.26
GAIF01021362.1	0.11	0.16	0	0	0.89	0.36
GAIF01021363.1	4.35	6.02	5.11	17.42	23.59	3.14
GAIF01021364.1	0.11	0.15	0.12	0.78	0.64	0.08

GAIF01021365.1	2.11	3.66	1.39	1.3	1.35	0.46
GAIF01021366.1	0.22	0.14	0.32	0.15	0.18	0
GAIF01021367.1	0.27	0	0	0.16	0.32	0
GAIF01021369.1	0.2	0.22	0.45	0	0	0.22
GAIF01021370.1	1495.14	2724.94	1293.22	674.38	1137.92	1328.7
GAIF01021372.1	0	7.03	13.76	0	0	4.07
GAIF01021373.1	266.6	60.54	144.41	317.61	232.27	278.77
GAIF01021374.1	3.3	6.22	4.58	3.83	2.02	0.96
GAIF01021375.1	0.99	1.3	0.71	0.79	1.89	1.13
GAIF01021376.1	0.16	0.5	0.69	0.78	1.34	0
GAIF01021377.1	12.43	11.81	16.56	6.5	6.21	6.19
GAIF01021378.1	0	0	1.73	0.7	0	0.91
GAIF01021381.1	1.71	1.62	0.07	13.96	9.43	0.24
GAIF01021383.1	0	0.1	0	0	0	0
GAIF01021384.1	7.46	6.65	9.23	14.09	11.95	5.32
GAIF01021385.1	5.46	4.1	1.59	29.85	52.98	2.93
GAIF01021386.1	7.88	12.33	7.11	25.17	21.21	0
GAIF01021387.1	0.88	0	0	5.33	10.5	21.34
GAIF01021388.1	65.55	66.11	116.2	108.53	67.71	32.66
GAIF01021389.1	36.47	48.07	53.3	58.25	44.21	24.1
GAIF01021390.1	0.05	0	0	0	0.08	0
GAIF01021391.1	23.64	1.71	1.89	28.84	61.52	82.94
GAIF01021393.1	0.5	0.53	0	3.73	11.2	1.94
GAIF01021394.1	1.97	1.25	0.62	1.16	1.53	0.3
GAIF01021395.1	270.49	180.95	115.2	279.46	271.41	812.68
GAIF01021396.1	25.98	16.7	6.25	3.42	1.46	0
GAIF01021397.1	3.03	0.49	0.37	4.56	28.33	5.32
GAIF01021398.1	1.51	0.22	0	0	0.24	0
GAIF01021400.1	0	0	0	0.09	0.1	0
GAIF01021401.1	0.03	0	0	0.08	0	0
GAIF01021402.1	0	0	0	0.18	3.83	0.33
GAIF01021403.1	0.06	0.28	0.08	0.26	0	0
GAIF01021404.1	0	0	0	0.21	0.31	0
GAIF01021405.1	0	0	0	0.03	0.19	0
GAIF01021406.1	47.46	47.96	31	86.06	65.37	10.28
GAIF01021407.1	6.73	5.57	12.47	4.4	4.6	0.83
GAIF01021408.1	0.39	0	0	0.11	0.35	0
GAIF01021412.1	2.72	1.79	0.11	4.05	10.74	0.43
GAIF01021413.1	18.96	18.19	11.25	21.94	27.27	38.05
GAIF01021414.1	0.05	0	0.17	0	0	0.06
GAIF01021415.1	5.16	1.34	0.25	0.52	0	0
GAIF01021416.1	3.79	0.35	0.2	1.04	4	0.39
GAIF01021417.1	0.03	0	0	0	0	0
GAIF01021418.1	3.41	14.67	0	18.91	16.55	0

GAIF01021420.1	0.01	0.03	0.03	0.02	0.17	0
GAIF01021422.1	10.13	13.46	8.94	12.86	6.69	1.28
GAIF01021423.1	0.42	0	0.17	0.23	0	0.05
GAIF01021424.1	0	0.05	0	0	0.41	0.1
GAIF01021425.1	7.84	6.17	0.84	14.43	30.21	0.35
GAIF01021426.1	0	0	0	0.27	0	0
GAIF01021430.1	0.12	0.07	0.07	0.14	1.33	0
GAIF01021431.1	12.99	10.05	2.24	27.22	32.66	0.58
GAIF01021433.1	0.31	0	0	1.31	2.83	0
GAIF01021434.1	0	0	0	0.27	1.85	0
GAIF01021436.1	4.87	2.15	0.17	13.08	38.63	3.69
GAIF01021437.1	17.31	27.08	31.25	29.86	15.39	11.39
GAIF01021439.1	4.55	2.05	0.39	32.26	141.06	0
GAIF01021440.1	0	0	0	0.23	1.92	0
GAIF01021441.1	1.86	0	0	8.07	32.99	2.74
GAIF01021442.1	3.09	2.82	4.2	0.12	0.7	0.23
GAIF01021443.1	2.92	3.41	2.02	4.51	7.02	1.55
GAIF01021444.1	0	0	0	0	0.31	0
GAIF01021445.1	18.34	36.23	27.45	50.79	30.98	5.22
GAIF01021446.1	15.57	12.87	14.81	17.59	12.69	4.2
GAIF01021447.1	0.16	0	0	0	0	0
GAIF01021449.1	1.4	1.34	0	2.26	5.29	1.88
GAIF01021450.1	0.1	0.76	0.22	0.12	0.48	0
GAIF01021451.1	6.83	10.2	6.59	19.88	27.31	1.29
GAIF01021453.1	5.07	4.04	3.36	5.09	1.65	0.23
GAIF01021454.1	1.85	0	0	2.05	0.78	1.33
GAIF01021456.1	0	0	0	0	0.2	0
GAIF01021457.1	0.32	1	0.26	2.42	5.59	0.18
GAIF01021458.1	0	0.11	0.22	0.24	2.2	0
GAIF01021459.1	5.5	0.5	0.38	15.17	70.73	47.5
GAIF01021460.1	19.08	15.34	14.55	28.59	23.61	6
GAIF01021461.1	0.14	0.61	0	0.33	2.53	0.62
GAIF01021462.1	0	0.09	0.05	0	0	0.05
GAIF01021463.1	0.3	0.33	0.05	0.58	2.71	0
GAIF01021465.1	74.48	98.57	84.99	139.86	146.46	40.44
GAIF01021471.1	0	0	0	0	0.89	0
GAIF01021473.1	0.25	0	0	0.2	0	0
GAIF01021476.1	0	0.07	0	0	0	0.2
GAIF01021477.1	15.48	16.32	7.32	28.15	26.48	2.75
GAIF01021478.1	9.59	3.82	3.13	0	16.21	0.1
GAIF01021479.1	2.37	5.83	0.14	7.38	19.95	0
GAIF01021481.1	0	0.29	0.44	0.31	3.69	0.59
GAIF01021482.1	0	0.3	0	1.04	0	0
GAIF01021483.1	0.26	0	0	0	0	0

GAIF01021485.1	0.12	0	0	0	0	0
GAIF01021486.1	0	0.03	0	0	0	0
GAIF01021487.1	0	0.25	0.32	0	0.23	0
GAIF01021488.1	0	0	0	0	0.37	0
GAIF01021489.1	0.74	0	0	0.85	2.82	0
GAIF01021491.1	0	0	0	0	0.33	0
GAIF01021494.1	62.71	73.33	58.67	6.7	2.05	0.52
GAIF01021496.1	0.09	0	0	0	0.65	0
GAIF01021497.1	60.95	28.74	2.62	56.12	76.26	5.04
GAIF01021499.1	0	0	0	0	0.07	0.14
GAIF01021500.1	0	0	0	0	0.6	0
GAIF01021501.1	1.22	0.31	0.19	0.52	0.42	0
GAIF01021503.1	0.51	0.25	0.23	0	0	0
GAIF01021504.1	0.79	0.1	0.06	3	10.55	0.05
GAIF01021506.1	54207.21	185288.73	111235.87	65803.86	76743.3	16900.2
GAIF01021507.1	0	0	0	0.44	2.52	0
GAIF01021509.1	73.51	115.64	138.59	114.91	47.39	53.7
GAIF01021510.1	13.54	13.56	6.44	30.26	30.17	4.95
GAIF01021511.1	13.4	6.43	6.38	4.42	1.12	7.4
GAIF01021512.1	60.54	93.72	23.03	3.43	22.67	18.98
GAIF01021514.1	0.1	0	0	0	0.83	0
GAIF01021515.1	0.02	0	0	0	0.16	0.05
GAIF01021516.1	5.85	5.01	4.39	5.93	3	9.62
GAIF01021518.1	1.23	1.34	1.69	1.34	0.83	0.34
GAIF01021519.1	0	0	0	0	0.24	0
GAIF01021520.1	44.41	34.76	9.95	68.39	79.3	8.16
GAIF01021521.1	1.71	0	1.5	2.78	0.82	0.88
GAIF01021523.1	0	0.19	0.38	0.2	0	0
GAIF01021526.1	30.56	16.77	19.45	58.97	59.58	30.45
GAIF01021528.1	0	0	0	0.13	0	0.12
GAIF01021530.1	20.68	13.19	19.72	19.73	3.27	20.43
GAIF01021532.1	0.02	0	0	0.14	1.01	0.07
GAIF01021534.1	0.55	0.5	0.04	0.78	0.61	0.12
GAIF01021536.1	91.8	116.32	71.98	123.48	97.09	25.54
GAIF01021537.1	0.46	1.22	1.45	1.3	1.03	0.95
GAIF01021540.1	0.17	0.18	0.06	0.13	0	0
GAIF01021542.1	0.91	0.6	1.06	0.15	0.31	0.43
GAIF01021543.1	0	0	0	0	0.37	0
GAIF01021546.1	11.45	10.8	7.01	7.18	5	0.78
GAIF01021547.1	0	2.83	1.11	4.48	3.19	0.31
GAIF01021549.1	5.5	1.86	0.82	14.34	64.85	5.28
GAIF01021550.1	4.18	2.7	1.67	0.13	0	0.06
GAIF01021553.1	0	0	0	0.27	1.04	0
GAIF01021556.1	0	0	0	0	0.1	0

GAIF01021557.1	0.69	0.37	0.38	0.13	0.14	0
GAIF01021559.1	13.46	16.87	21.3	42.38	20.4	16.26
GAIF01021562.1	23.94	0	7.88	13.16	8.11	48.84
GAIF01021563.1	4.13	2.58	0	0.58	0	0
GAIF01021564.1	0	0	0.06	0	0	0
GAIF01021565.1	1.27	0.9	2.02	0.6	0.35	0.68
GAIF01021567.1	192.06	265.35	263.6	214.6	145.75	41.29
GAIF01021568.1	0.52	0	0	4.8	13.45	0.21
GAIF01021570.1	15.29	13.23	6.41	23.8	17.47	4.52
GAIF01021571.1	3.15	1.22	0.45	0.59	0.37	0.11
GAIF01021572.1	0	0	0	0	3.71	1.69
GAIF01021573.1	4.38	0.32	1.08	5.45	5.12	19.18
GAIF01021574.1	1.07	1.75	0.12	1.99	3.48	0.47
GAIF01021576.1	2.06	0.3	0.22	6.84	16.72	0.65
GAIF01021577.1	10.54	17.15	16.7	16.69	10.58	4.31
GAIF01021578.1	0.68	0.96	0.67	2.51	5.04	0.22
GAIF01021579.1	0	0	0	0.06	0.2	0
GAIF01021580.1	89.9	56.7	50.36	105.14	107.49	51.31
GAIF01021582.1	0	0	0	0	0.42	0.1
GAIF01021584.1	0.59	0.15	0.25	1.43	7.31	0.05
GAIF01021586.1	0.16	0.28	0.17	4.95	7.63	0.67
GAIF01021587.1	0.44	0	0	0	0	0
GAIF01021588.1	49.98	62.15	87.76	80.39	43.49	50.24
GAIF01021591.1	1.46	1.72	1.36	1.05	1.08	0.5
GAIF01021593.1	0.39	0.07	0.07	0.8	4.31	0.13
GAIF01021595.1	0.06	0.13	0.2	1.1	1.47	0.07
GAIF01021598.1	173.11	102.31	20.28	0	0.38	0
GAIF01021600.1	21.97	9.82	7.27	30.96	61.94	15.65
GAIF01021601.1	13.42	9.67	8.41	29.59	11.99	8.21
GAIF01021602.1	0.31	0	0	1.02	1.26	0
GAIF01021603.1	0	0	0.27	0	0	0
GAIF01021604.1	26.25	15.3	7.08	38.55	42.34	10.9
GAIF01021605.1	44.65	60.26	49.57	58.07	41.42	20.7
GAIF01021606.1	0.74	0.72	0.45	1	1.77	0.16
GAIF01021607.1	0.08	0	0.04	0.23	0.19	0
GAIF01021608.1	0	0	0	0	0	0.04
GAIF01021609.1	0.43	0.21	0.11	0.23	0.21	0.04
GAIF01021610.1	14.89	13.44	7.04	13.16	18.45	7.5
GAIF01021612.1	0	0.19	0	0	0	0
GAIF01021613.1	28.12	17.85	3.09	138.5	203.25	3.15
GAIF01021614.1	0	0.07	0	0.04	0.09	0
GAIF01021615.1	5.19	5.67	2.67	12.82	17.31	1.52
GAIF01021618.1	0.13	0.08	0.12	0.04	0	0.1
GAIF01021619.1	6.88	14.63	18.5	22.89	21.87	10.19

GAIF01021620.1	0.03	0	0	0	0	0
GAIF01021621.1	0	0.03	0	0.02	0.03	0.06
GAIF01021623.1	0	0	0	0	0.06	0
GAIF01021624.1	0	0	0	0.11	0.25	0.06
GAIF01021625.1	0.01	0	0.25	0	0	0
GAIF01021626.1	0	0	0	0.24	0.09	0.04
GAIF01021627.1	7.42	3.5	2.71	10.3	24.54	13.83
GAIF01021628.1	56.3	39.12	26.75	10.25	10.78	9.88
GAIF01021629.1	0.26	0	0	0.47	1.27	0.22
GAIF01021630.1	37.7	20.73	13.76	38.37	51.61	40.09
GAIF01021631.1	3.5	6.04	3.05	3.07	1.99	0.15
GAIF01021632.1	0.66	0	0	0.61	0.42	0
GAIF01021634.1	0	0	0	0	0.91	0
GAIF01021635.1	118.31	163.42	104.06	146.78	112.81	19.47
GAIF01021638.1	11.8	14.74	2.75	46.41	187.29	4.77
GAIF01021639.1	0	0	0.08	0	0	0
GAIF01021642.1	8.6	4.37	2.73	0.84	1.13	0.33
GAIF01021644.1	0	0.92	0.46	0	0	0
GAIF01021645.1	0	0	0.35	0.68	0.06	0
GAIF01021646.1	8.78	7.69	18.62	9.39	7.17	9.08
GAIF01021648.1	0	0	0	0.4	1.86	0
GAIF01021649.1	0	0	0	0	0.64	0
GAIF01021650.1	0.19	0.41	0	1.95	6.17	2.77
GAIF01021651.1	18.71	19.05	11.15	7.86	5.09	3.08
GAIF01021652.1	0.09	0	0	0.1	0.11	0
GAIF01021653.1	32.17	34.85	31.17	87.83	99.87	12.76
GAIF01021654.1	0.74	0	0	0.73	0	0
GAIF01021655.1	0	0	0	0	0.07	0
GAIF01021657.1	185.53	21.95	18.77	265.75	416.34	4458.63
GAIF01021658.1	0.1	0.52	0.31	0.22	0.91	0
GAIF01021659.1	5.81	7.75	6.06	3.3	5.15	5.2
GAIF01021660.1	0.76	0.26	0.26	2.46	9.9	0.4
GAIF01021661.1	0	0	0	0	0.41	0.35
GAIF01021662.1	0	2.94	1.64	0	0	1.33
GAIF01021663.1	0.31	0.85	0.51	0	0	0.17
GAIF01021664.1	0	0.17	0	0.81	3.06	0
GAIF01021665.1	48.38	3.14	0.81	4.9	22.37	4.15
GAIF01021667.1	0	0.35	0	0	0.58	0
GAIF01021668.1	0	0	0	0.24	0.13	0
GAIF01021669.1	0.19	0	0	0.82	1.61	0
GAIF01021670.1	19.35	18.6	17.49	46.15	37.6	11.05
GAIF01021671.1	166.16	180.76	221.34	56.56	60.17	163.89
GAIF01021673.1	0	0	0	0	1.13	0.26
GAIF01021675.1	1.47	0.71	0.66	8.02	18.66	1.32

GAIF01021676.1	46.69	75.48	58.68	40.46	29.18	17.43
GAIF01021677.1	0.03	0.01	0	0	0.05	0
GAIF01021678.1	0	0	0	70.01	0	0.14
GAIF01021679.1	177.26	145.01	269.86	161.6	153.16	452.52
GAIF01021680.1	61.1	34.71	52.33	49.75	37.57	15.39
GAIF01021682.1	0.15	0.24	0	0.41	1.54	0.44
GAIF01021684.1	16.37	25.64	57.21	14.26	23.35	8.68
GAIF01021685.1	7.57	16.77	18.78	31.16	29.06	10.39
GAIF01021686.1	22.83	33.07	15.98	59.49	66.14	5.19
GAIF01021688.1	0	0.21	0.16	0.06	0.4	0.32
GAIF01021689.1	0	0.86	0	0.18	0.98	0.42
GAIF01021690.1	10.19	10.59	14.62	2.16	7.88	38.66
GAIF01021692.1	0.43	0.16	0	2.46	24.58	0.11
GAIF01021693.1	13.82	19.7	19.21	46.2	32.32	9.22
GAIF01021694.1	1.18	0.95	1.41	0.31	1.07	0
GAIF01021695.1	1.77	0.22	0.8	5.61	9.52	1.08
GAIF01021697.1	299.45	326.33	281.75	135.86	114.53	218.32
GAIF01021698.1	0.17	0	0	0	0.2	0.18
GAIF01021699.1	0.21	0.08	0	0.65	1.42	0.08
GAIF01021700.1	19.28	21.57	14.71	3.34	9.2	6.59
GAIF01021701.1	0	1.98	0	0	0	0
GAIF01021702.1	0.1	0.02	0.11	0.14	0.15	0.04
GAIF01021703.1	0	0.85	1.82	0	0	0.11
GAIF01021705.1	589.51	493.27	279.12	672.19	755.34	652.25
GAIF01021706.1	0	0	0.19	0	0.1	0.1
GAIF01021707.1	0.33	0.36	0.05	0.24	0.55	0
GAIF01021708.1	1.39	3.67	0.22	2.87	5.15	0
GAIF01021711.1	0.17	0	0	0.39	2.41	0
GAIF01021712.1	1.23	0.18	0.06	5.6	19.91	8.66
GAIF01021713.1	0	0	0	0	0.04	0
GAIF01021716.1	10.97	17.7	7.92	54.6	34.38	2.71
GAIF01021717.1	16.72	21.79	26.15	6.25	4.44	4.61
GAIF01021718.1	0.06	0	0	0.29	1.35	0
GAIF01021720.1	0	0	0.01	0	0	0
GAIF01021722.1	0	0	0	0.27	1.51	0
GAIF01021723.1	0.34	1.57	2.05	2.17	2.46	0.09
GAIF01021724.1	0	0.14	0	0	0.08	0
GAIF01021726.1	0.11	0.06	0	1.09	4.2	0.12
GAIF01021727.1	100.18	79.23	37.24	101.82	91.68	25.15
GAIF01021728.1	0	0	0	0.25	0	0.48
GAIF01021729.1	0	0.11	0	0.36	1	0
GAIF01021731.1	0.27	0	0	0	0	0
GAIF01021733.1	0	0	0	0	2.02	0
GAIF01021734.1	19.73	21.19	4.5	29.18	30.41	3.5

GAIF01021735.1	0	0	0	0	0.01	0
GAIF01021736.1	0	0	0	0.04	0.09	0
GAIF01021737.1	4.52	7.3	2.4	16.09	22.81	0.8
GAIF01021738.1	0.5	0.37	2.74	2.23	1.84	0.11
GAIF01021739.1	19.48	9.35	12.62	15.67	25.68	12.64
GAIF01021740.1	0.04	0	0	0	0	0
GAIF01021742.1	2.19	1.65	1.66	5.67	5.06	2.23
GAIF01021744.1	1.88	0.85	2.04	1.49	1.3	0.43
GAIF01021746.1	0	0	0	0.21	0.65	0
GAIF01021747.1	1.04	0.64	0.43	3.44	10.69	0.87
GAIF01021748.1	0.1	0	0.06	0.06	0.55	0
GAIF01021750.1	13.63	25.47	18.66	43.04	33.12	5.24
GAIF01021752.1	0	0	0.02	0.09	1.18	0
GAIF01021753.1	5.98	128.66	77.33	2.41	2.73	5.83
GAIF01021755.1	0	0	0	0.23	0.42	0
GAIF01021756.1	0	0.1	0	0	0	0.1
GAIF01021757.1	0	5.34	5.15	0	0.13	0.37
GAIF01021758.1	1.56	1.19	0.16	9.82	22.52	0.12
GAIF01021759.1	0.21	0.19	0	2.38	8.06	0
GAIF01021760.1	6.11	5.56	2.37	9.64	27.98	1.06
GAIF01021761.1	0	1.18	1.34	0	0.16	2.54
GAIF01021762.1	0.03	0.08	0.02	0.05	0.07	0
GAIF01021763.1	2.86	6.64	0.86	9.05	7.97	0.57
GAIF01021764.1	0	0	0	0	0.63	0
GAIF01021765.1	3.32	4.94	0.69	3.48	0.78	1.38
GAIF01021766.1	0	0	0	2.6	0.46	1.43
GAIF01021768.1	1.26	1.44	0.92	2.68	1.6	2.16
GAIF01021769.1	0	0	0	0	0.38	0.23
GAIF01021771.1	0	0	0	0.01	0	0
GAIF01021772.1	63.86	44.86	10.69	69.37	71.46	4.62
GAIF01021773.1	0	0.08	0.08	0	0	0.31
GAIF01021776.1	0.07	0.06	0	0.23	0.37	0.06
GAIF01021777.1	3.52	0.33	0.16	12.82	49.83	9.08
GAIF01021779.1	0	0	0	0.05	0	0
GAIF01021781.1	0.2	0.17	0.1	0.52	1.54	0
GAIF01021783.1	1.06	0.96	0.19	3.09	6.61	0.29
GAIF01021784.1	0	0	0	0.12	0	0
GAIF01021785.1	4.26	2.46	1.94	17.42	15.6	1.48
GAIF01021786.1	0.11	0	0	0.23	0.44	0.15
GAIF01021787.1	10.61	12.22	16.92	43.98	26.82	9.79
GAIF01021788.1	29.51	36.82	32.31	76.13	55.13	14.72
GAIF01021789.1	0	0.04	0	0	0.87	0
GAIF01021790.1	0	0	0	0	0.12	0
GAIF01021791.1	0.14	0	0.23	0.74	3.9	0.08

GAIF01021792.1	0	0	0	0	0.79	0
GAIF01021793.1	0.14	0.15	0.1	0	0.06	0
GAIF01021795.1	35.6	23.83	29.23	82.3	43.52	71.18
GAIF01021796.1	3.87	2.9	4.13	0.95	0.72	0.84
GAIF01021797.1	0.33	0.5	0.22	3.1	4.8	0.07
GAIF01021798.1	99.38	89.25	84.92	227.71	155.11	91.53
GAIF01021799.1	0	0	0.29	0	0	0
GAIF01021800.1	0	0.44	0.24	0	0.05	0.15
GAIF01021801.1	0.2	0	0.37	0	0.44	0.46
GAIF01021803.1	0.07	0.08	0	0	0.2	0
GAIF01021805.1	0	0	0	0	0.05	0.05
GAIF01021807.1	0.08	0	0.78	0.18	0.34	0
GAIF01021808.1	3.09	0.69	1.91	5.61	6.06	6.1
GAIF01021811.1	0	0.06	0	0.06	0	0
GAIF01021812.1	0	0	0	0.03	0	0.03
GAIF01021814.1	0.92	0.23	0	3.2	11.59	1.32
GAIF01021815.1	0.27	0.48	0.1	3.3	5.93	0
GAIF01021816.1	0.11	0	0	0.39	2.02	0
GAIF01021817.1	0	0	0	0	0.24	0
GAIF01021818.1	0	0	0	0.36	3.24	0.11
GAIF01021821.1	0.89	0.85	0.85	3.87	8.43	0
GAIF01021822.1	0	0	0	0	0.28	0
GAIF01021823.1	5.6	0.48	0.07	0.42	2.75	0.37
GAIF01021824.1	0.44	0.38	0.3	1.45	7.34	3.06
GAIF01021825.1	0	0.69	0	0.48	0.74	0
GAIF01021826.1	60.69	56.2	50.85	82.42	43.5	11.94
GAIF01021827.1	0.54	0	0	0.5	0.13	0
GAIF01021828.1	43.47	46.1	16.17	17.16	11.33	2.96
GAIF01021830.1	0	91.8	55.97	0.11	1.14	3.34
GAIF01021831.1	0.07	0.04	0	0.04	0.12	0
GAIF01021832.1	0	0	0.06	0.03	0.59	0
GAIF01021834.1	5.63	8.62	6.35	27.66	31.12	6.17
GAIF01021835.1	0.15	0.39	0.11	1.37	3.2	0.06
GAIF01021836.1	0	0	0.06	0	0	0
GAIF01021837.1	7.84	15.47	20.65	18.88	17.92	6.91
GAIF01021838.1	0	0	0.2	0	0	0.1
GAIF01021840.1	0.52	0.89	0.35	0	0.1	0.53
GAIF01021841.1	0	0	0.18	0	0	0.09
GAIF01021843.1	9.8	7.58	6.01	23.95	17.13	6.19
GAIF01021844.1	0	0.21	0.32	0.38	0.21	0
GAIF01021846.1	125.47	65.05	121.8	158.31	122.97	109.73
GAIF01021847.1	0	0	0	0	0.11	0
GAIF01021848.1	0.1	0.21	0	0.22	0.11	0
GAIF01021849.1	0.74	0.23	0.12	2.34	14.06	0.35

GAIF01021850.1	0	0.56	0.6	0	0	0
GAIF01021852.1	0.03	0.06	0.03	0	0.03	0
GAIF01021853.1	6.96	6.91	7.91	26	10.56	22.38
GAIF01021854.1	8.76	1.78	4.3	11.74	24.65	74.2
GAIF01021855.1	44.05	39.6	8.25	18.13	47.41	0.23
GAIF01021856.1	0.19	0.39	0	2.74	2.5	0.91
GAIF01021857.1	3.86	4.95	1.12	6.88	13.06	1.78
GAIF01021858.1	0.05	0.04	0	0.49	3.11	0
GAIF01021859.1	0.45	0.1	0	2.15	6.97	0.1
GAIF01021860.1	2.08	2.43	0	0	1.39	0
GAIF01021861.1	1.17	0	0	1.04	7.92	0
GAIF01021862.1	0.5	0.5	0.85	0.27	0	0.2
GAIF01021863.1	0	0.18	0	2.09	13.51	0
GAIF01021864.1	0	0.09	0.46	0	0	0
GAIF01021865.1	169.7	160.85	246.85	162.93	102.49	90.66
GAIF01021870.1	12.12	12.43	1.17	29.25	34.83	23.28
GAIF01021871.1	0.24	0.64	0.19	0.24	0.32	0.15
GAIF01021872.1	5.26	2.71	4.14	3.87	4.25	7.59
GAIF01021873.1	1.49	0.59	0	5.48	8.6	0.6
GAIF01021874.1	26.36	36.62	34.62	25.43	14.26	37.82
GAIF01021876.1	0.13	0.14	0	0	0.45	0
GAIF01021878.1	0	0.13	0	0.28	0.88	0
GAIF01021881.1	0	7.8	10.29	0	0.11	0.74
GAIF01021882.1	0	0	0	0.45	0.98	0
GAIF01021883.1	0	0.1	0	0	0.22	0.1
GAIF01021884.1	0	28.51	0	0	0	0
GAIF01021885.1	0.13	0	0	1.61	6.81	0.14
GAIF01021886.1	0.25	0	0	0.64	1.04	0
GAIF01021887.1	1.15	0.39	0	2.89	3.42	0
GAIF01021888.1	0.22	0.3	0.3	0.75	1.06	0
GAIF01021889.1	30.69	15	27.23	32.59	10.79	11.27
GAIF01021890.1	0.72	0	0	1.2	0.62	0.34
GAIF01021891.1	11.71	13.69	16.73	28.44	13.25	7.48
GAIF01021892.1	0	0	0	0	0.57	0
GAIF01021893.1	16.47	15.6	5.15	31.35	34.86	5.33
GAIF01021894.1	0.09	0	0	0	0	0
GAIF01021895.1	0	0.05	0	0.28	1.29	0
GAIF01021897.1	0.15	0	0.08	0	0	0
GAIF01021898.1	2.3	2.61	0	11.1	16.77	0.3
GAIF01021899.1	0.2	0	0	0.69	1.68	0.44
GAIF01021900.1	20.66	12.02	6.95	52.41	35.01	17.31
GAIF01021901.1	0	0	0	0	0.37	0
GAIF01021903.1	0	0	0	0.48	2.17	0
GAIF01021904.1	4.12	1.63	0.26	31.33	81.93	0.52

GAIF01021910.1	1.42	0.91	2.02	2.61	2.21	1.38
GAIF01021912.1	30.34	18.03	30.5	46.36	25.75	42.55
GAIF01021913.1	5.05	3.43	3.15	3.86	4.16	0
GAIF01021914.1	40.33	59.41	19.73	68.89	107.03	28.18
GAIF01021916.1	0.25	0	0	0	0.93	0
GAIF01021917.1	2.91	0	0	6.27	14.27	3.92
GAIF01021918.1	37.45	56.01	47.11	95.39	76.28	18.34
GAIF01021920.1	0.83	0.18	0.27	6.07	18.95	0.5
GAIF01021922.1	98.97	161.71	226.27	163.29	176.32	105.45
GAIF01021923.1	2.78	8.1	0.97	8.57	19.26	0.91
GAIF01021924.1	72.92	35.54	55.96	160.77	175.53	149.93
GAIF01021925.1	5.43	2.42	1.51	20.75	26.78	1.4
GAIF01021926.1	0.67	1.65	0	0.78	3.04	0
GAIF01021928.1	0	0	0	0.59	0	0
GAIF01021932.1	9.77	18.57	13.22	35.36	32.48	3.02
GAIF01021933.1	58.09	38.73	48.05	68.9	59.12	89.32
GAIF01021935.1	3.05	0.91	0.84	13.01	35.43	26.38
GAIF01021936.1	0	0	0	0.24	1.1	0
GAIF01021938.1	0.11	0	0	0.38	0.39	0.36
GAIF01021939.1	0	0	0	0	0.05	0
GAIF01021940.1	1.69	1.42	1.09	2.76	1.29	0.76
GAIF01021941.1	0.78	0.21	0.05	1.58	3.22	0
GAIF01021942.1	0	0	0	1.7	7.03	0.11
GAIF01021943.1	0	0	0.25	0	0.14	0
GAIF01021944.1	2.35	2.37	1.09	7.86	16.43	11.39
GAIF01021945.1	0	0	0	0	0.42	0
GAIF01021946.1	0.47	0	0	0.45	0.79	0
GAIF01021947.1	0.17	0.05	0.09	1	4.52	0.19
GAIF01021949.1	2.02	7.66	3.8	4.71	0.82	3.39
GAIF01021951.1	1.76	0	1.37	0.54	0	1.67
GAIF01021952.1	8.38	11.35	4.39	13.12	15.19	0.99
GAIF01021953.1	0.71	0	0.2	0.14	0.26	0
GAIF01021954.1	0.07	0	0	0.44	1.17	0
GAIF01021955.1	0	0.14	0	0	1.32	0.07
GAIF01021957.1	0.12	0	0	0.81	6.15	0.13
GAIF01021958.1	1.04	0	0.33	4.46	1.34	0
GAIF01021959.1	0	0	0.28	0	0	0
GAIF01021960.1	0	0.03	0	0	0.08	0.28
GAIF01021966.1	13.63	3.43	3.25	92.35	86.83	12.75
GAIF01021967.1	1.47	0.71	0.27	6.34	14.7	0.18
GAIF01021968.1	0	0.82	0	0	0	0.82
GAIF01021970.1	6.66	5.11	1.82	0.98	1.4	0.05
GAIF01021971.1	0.06	0.09	0	1.16	7.26	0
GAIF01021972.1	1.98	0.57	0.11	0.97	0.5	0.58

GAIF01021973.1	0	0	0	0.12	0.45	0
GAIF01021974.1	8.22	10.64	6.33	36.42	28.06	7.28
GAIF01021975.1	0.15	0.54	0.46	0.28	0.31	0.04
GAIF01021976.1	0	0	0	4.07	13.02	0.36
GAIF01021977.1	1.05	0.3	0.23	2.59	2.56	0.04
GAIF01021978.1	0	0.13	0	0	0	0
GAIF01021979.1	0.12	0.54	0	0.14	0.74	0
GAIF01021980.1	0	0	0	0	1.05	0
GAIF01021982.1	9.31	5.25	7.87	9.37	3.13	3.16
GAIF01021983.1	7.4	3.91	4.03	23	11.83	2.06
GAIF01021984.1	13.32	18.17	9.61	1.56	2.7	0.87
GAIF01021985.1	0.22	0.12	0.24	3.24	1.45	0.12
GAIF01021986.1	40.4	42.97	25.79	49.96	44	6.52
GAIF01021987.1	0.3	0.16	0	0.17	0.54	0
GAIF01021988.1	0	0.11	0.22	0.56	0.58	0
GAIF01021989.1	0.61	0.16	0.17	1.06	0.64	0.08
GAIF01021991.1	0	0	6.81	0	0	0
GAIF01021992.1	0.14	0	0.15	0	3.84	0.15
GAIF01021993.1	0	0	0	0	1.12	0
GAIF01021994.1	15.92	15.13	10.31	22.7	24.84	17.78
GAIF01021997.1	0.6	1.69	1.71	0.37	0.34	0.7
GAIF01021998.1	8.71	6.49	0	13.94	9.43	2.15
GAIF01021999.1	0.13	0	0	1.29	15.43	0
GAIF01022000.1	0.2	0	0.07	0.77	3.81	0.15
GAIF01022001.1	0.48	0.78	0.53	0.09	0.58	0.26
GAIF01022002.1	1064.92	517.14	401.07	2353.72	3403.66	8398.98
GAIF01022003.1	2.63	1.82	0	6.39	12.51	0.53
GAIF01022004.1	2.19	0.43	0.04	11.05	43.64	0.09
GAIF01022005.1	0.29	0.12	0.14	1.97	8.08	0.1
GAIF01022006.1	0.04	0	0.03	0	0	0.03
GAIF01022007.1	77.19	19.69	37.37	115.61	32.19	58.91
GAIF01022008.1	71.68	71.56	33.14	139.65	119.76	15.17
GAIF01022010.1	0	0	0	0.13	0.41	0
GAIF01022011.1	0	0.23	0	0.37	0.49	0.28
GAIF01022012.1	0	0	0	0.19	0.53	0
GAIF01022014.1	116.48	240.24	222.97	188.22	139.01	38.97
GAIF01022015.1	0.17	0.06	0	0.81	6.07	0.07
GAIF01022016.1	45.18	59.52	36.35	23.67	17.24	0
GAIF01022017.1	0	0.06	0	0	0	0
GAIF01022020.1	0	0	0	0	1.31	0
GAIF01022021.1	37.62	10.52	10.34	37.34	47.33	0
GAIF01022022.1	3.85	4.84	5.95	0.14	0.43	0.13
GAIF01022024.1	0.03	0	0	0.33	1.51	0.1
GAIF01022026.1	0.15	0	0	0	0.3	0.06

GAIF01022027.1	0.74	0	0	0	0	0
GAIF01022030.1	26.63	27.64	24.93	38.62	32.75	7.39
GAIF01022031.1	0	0	0	0	0.14	0.07
GAIF01022033.1	11.82	14.87	13.09	61.48	39.87	2.58
GAIF01022034.1	0.24	0	0	0.28	0.48	0
GAIF01022035.1	147.74	114.21	159.16	43.86	25.3	53.25
GAIF01022036.1	35.38	24.51	23.26	55.36	39.41	14.03
GAIF01022037.1	0.48	0	0	0	0.06	0
GAIF01022039.1	1.62	0.92	0.36	13.87	41.01	1.07
GAIF01022040.1	1.1	0	0.98	3.44	5.09	0.74
GAIF01022041.1	2735.62	2089.35	1830.52	1768.03	1589.07	3606.97
GAIF01022043.1	0	0	0	0	0.21	0
GAIF01022044.1	1.87	2.2	0.4	7.99	10.7	0.83
GAIF01022045.1	11.8	11.94	6.21	41.41	23.7	7.14
GAIF01022046.1	0	0.12	0.24	0.25	0	0
GAIF01022048.1	24.48	28.47	15.17	36.05	44.53	2.89
GAIF01022049.1	0	0	0	0	1.31	0
GAIF01022050.1	0.45	0.41	0.33	1.66	1.18	1
GAIF01022052.1	0	0	0.25	0	0	0.08
GAIF01022053.1	0.12	0	0	0	0.22	0.13
GAIF01022054.1	41.06	31.87	6.71	24.47	50.69	0
GAIF01022055.1	2.97	2.06	0.58	13.28	10.95	0.73
GAIF01022056.1	12.19	24.77	13.49	13.91	10.78	3.14
GAIF01022057.1	0	0	0.13	0	0	0
GAIF01022059.1	0	0.03	0	0.03	0.1	0
GAIF01022060.1	16.36	23.47	23.91	34.83	14.85	12.17
GAIF01022061.1	0.52	0.32	0.81	0.94	1.51	0.24
GAIF01022062.1	17.37	16	27.08	60.33	31.42	24.11
GAIF01022064.1	22.07	15.41	20.02	51.37	46.94	41.35
GAIF01022065.1	439.41	629.52	1164.71	191.71	128.98	376.8
GAIF01022066.1	446.97	398.05	954.64	222.36	154.37	583.63
GAIF01022067.1	0	1.55	2.28	0	0	0.64
GAIF01022068.1	18.47	30.18	5.21	49.28	89.79	1.82
GAIF01022069.1	0	0	0	0.11	0	0
GAIF01022070.1	0	0	0.05	0.28	0.7	0.05
GAIF01022071.1	0.28	0.43	0	0.39	0.34	0
GAIF01022072.1	1.31	0.16	0	5.81	26.91	0.32
GAIF01022073.1	0	0	0	0	0.18	0
GAIF01022076.1	2.86	1.37	1.71	19.71	47.82	1.98
GAIF01022080.1	6.01	0	2.05	11.88	3.68	18.92
GAIF01022081.1	0.12	0	0	2.28	5.56	0.25
GAIF01022082.1	0	2.05	0.58	0	0	0.66
GAIF01022083.1	0.37	0.1	0.25	1.61	4.41	0.2
GAIF01022085.1	8.77	6.93	6.59	18.91	25.81	23.82

GAIF01022089.1	0	1.17	0	0	0	0
GAIF01022090.1	0	0	0	0	0.06	0
GAIF01022091.1	0.25	0	0.27	1.3	3.88	0.14
GAIF01022092.1	0	0	0	0	0	0.6
GAIF01022093.1	30.95	29.15	61.65	123.03	86.84	57.66
GAIF01022095.1	0.05	0	0	0.35	1.95	0
GAIF01022096.1	0	0	0	0.12	0.26	0
GAIF01022097.1	0	0.18	0	0.09	0.59	0
GAIF01022098.1	33.71	39.8	22.1	12.68	8.58	8.8
GAIF01022099.1	0	0.8	0.87	0	0.99	0.76
GAIF01022100.1	0.49	0.07	0	1.29	8.16	1.88
GAIF01022101.1	0.81	0.29	0	0	0	0
GAIF01022103.1	2.44	2.78	1.47	19.23	20.8	0.54
GAIF01022104.1	0.07	0.32	0.25	0.17	0	0
GAIF01022105.1	95.89	64.45	59.95	51.32	19.61	44.75
GAIF01022109.1	0.26	0.57	0.4	0.61	0.38	0
GAIF01022110.1	0	0	0	0.04	0.09	0
GAIF01022111.1	0.1	0	0	1.55	2.75	0
GAIF01022112.1	0	0	0.13	0	0	0
GAIF01022113.1	6.05	5.81	3.8	3.68	2.46	0.46
GAIF01022114.1	0	0.12	0.13	0.4	3.45	0.13
GAIF01022116.1	7.87	0	0.47	0.05	0	0
GAIF01022117.1	0	0	0	0	0.67	0
GAIF01022118.1	0	0	0.23	0	0.14	0
GAIF01022120.1	4.68	11.38	17.67	0	14.19	6.53
GAIF01022121.1	37.7	25.76	15.35	1.32	0.81	1.18
GAIF01022124.1	0	0	0	0	0	0.14
GAIF01022125.1	4.15	11.86	7.16	8.65	8.97	0.51
GAIF01022126.1	512.88	700.89	400.75	37.21	38.37	50.91
GAIF01022128.1	0.81	1.98	0.89	0.47	0.12	0
GAIF01022129.1	0.82	0	0	4.37	18.59	0.27
GAIF01022131.1	14.9	8.44	37.29	14.8	0.41	8.66
GAIF01022132.1	2.61	0.52	0.83	1.1	0.34	1.1
GAIF01022133.1	14.48	35.02	28.59	19.74	23.19	6.09
GAIF01022134.1	17.85	13.3	12.33	29.1	33.93	33.74
GAIF01022136.1	11.92	9.85	10.36	19.74	8.66	4.65
GAIF01022137.1	20.41	26.6	23	18.18	19.95	3.93
GAIF01022138.1	20.66	17.42	10.67	55.36	75.04	13.45
GAIF01022142.1	294.33	259.99	30.7	7.46	21.01	0.9
GAIF01022143.1	0.24	0.32	0	6.22	15.08	0.15
GAIF01022144.1	0	0	0	0	0.13	0
GAIF01022145.1	0	0	0	0	1.66	0
GAIF01022146.1	12.59	42.5	33.61	14.98	26.55	7.64
GAIF01022150.1	0	0	0.16	0	2.91	0.46

GAIF01022151.1	0	0.16	0.08	0	0	0
GAIF01022152.1	0	0.05	0	0.74	0.45	0.05
GAIF01022153.1	40.71	38.02	14.32	161.96	181.5	2.42
GAIF01022155.1	15.63	16.14	12.1	36.79	18.68	11.88
GAIF01022156.1	0.04	0.04	0.05	0.05	0.79	0
GAIF01022157.1	1.09	1.76	1.52	9.33	13.56	1.2
GAIF01022160.1	5.71	5.23	1.86	1.54	2.7	0.94
GAIF01022161.1	0	0.12	0.06	0	0	0.44
GAIF01022163.1	0.02	0.04	0	0.14	0.21	0
GAIF01022165.1	0.66	0.18	0.4	0.98	0.46	0.33
GAIF01022167.1	0	0	0	0.16	0.05	0
GAIF01022169.1	0.15	0	0	0.26	1.77	0.16
GAIF01022170.1	0.89	0	0	0.47	0	0
GAIF01022171.1	0.32	0	0.35	0.25	0	0.12
GAIF01022173.1	0.17	0.1	0.06	0.05	0.01	0.35
GAIF01022175.1	0.02	0	0	0	0	0
GAIF01022178.1	0.1	0.16	0.21	0.56	0.94	0.11
GAIF01022179.1	0.44	0	0.65	0.34	0	0
GAIF01022180.1	0.33	0.72	0.6	0.13	0.13	0.49
GAIF01022182.1	1.44	2.16	0.95	4.78	2.59	0.45
GAIF01022183.1	0	0	1.24	0	0	0.47
GAIF01022184.1	15.36	14.72	10.44	14.09	7.75	2.19
GAIF01022185.1	2.61	2.27	1	7.71	13.63	2.15
GAIF01022186.1	2.37	1.46	0.6	1.27	3.44	0.2
GAIF01022187.1	12.79	8.1	9.61	6.53	7.66	6.7
GAIF01022188.1	3.83	3.36	3.8	0.72	0.3	0
GAIF01022190.1	9.21	16.61	14.05	25.07	19.68	4.02
GAIF01022194.1	0	0	0	82.19	52.85	16.41
GAIF01022195.1	15.54	13.25	11.46	34.42	18.58	5.93
GAIF01022196.1	0.23	0.93	0	3.98	8.72	0
GAIF01022197.1	1.35	0.21	0	2.24	12.31	0.64
GAIF01022199.1	347.51	162.12	133.37	215.9	173.3	178.78
GAIF01022200.1	0.03	0	0.07	0.02	2.33	0
GAIF01022202.1	7.84	7.45	7.26	14.47	12.84	6.25
GAIF01022204.1	6.78	2.73	0.42	71.27	174.16	0.95
GAIF01022205.1	0	0.15	0	0.16	0	0
GAIF01022206.1	6.25	6.38	1.56	8.59	5.43	2.81
GAIF01022208.1	251.47	101.91	112.4	101.64	112.8	298
GAIF01022210.1	0.42	0.45	0.34	1.21	2.25	0.11
GAIF01022213.1	0.21	0.32	0.12	0.18	0.09	0.15
GAIF01022214.1	6.35	5.42	1.79	4.14	4.18	0.43
GAIF01022215.1	0.87	0.2	0.35	0.74	1.34	0
GAIF01022216.1	10.44	17.79	23.88	49.63	31.73	12.01
GAIF01022218.1	1.57	0.14	0.14	0.45	0	0

GAIF01022219.1	0.05	0.16	0	0.23	1.07	0.27
GAIF01022220.1	5.13	2.12	0.66	3.85	0.88	0.2
GAIF01022221.1	0	0.05	0	0	0	0
GAIF01022224.1	12.01	0	0	0	6.34	22.03
GAIF01022226.1	0.99	0.54	0	3.45	14.09	0
GAIF01022227.1	0.95	0	0.12	0	0	0.12
GAIF01022231.1	2.68	4.6	1.92	11.62	14.51	1.15
GAIF01022232.1	0	0	1.19	0	0	0
GAIF01022233.1	14.46	23.73	8.51	19.49	18.48	1.22
GAIF01022234.1	0	0.34	0.35	0	0.05	0.35
GAIF01022235.1	3600.98	666.3	1526.15	2782.22	1403.27	8658.27
GAIF01022236.1	3.37	4.74	3.89	33.13	69.9	4.86
GAIF01022237.1	0.03	0	0	0.05	0	0
GAIF01022238.1	22.11	32.38	17	63.47	50.34	5.09
GAIF01022239.1	1.52	0.22	0.28	12.09	39.64	0.61
GAIF01022240.1	0	0	0	42.84	19.43	6.08
GAIF01022242.1	102.69	101.18	166.92	49.76	31.19	83.45
GAIF01022243.1	0.11	0	0	0	0	0
GAIF01022244.1	0.73	0	0.06	0.04	0	0.25
GAIF01022246.1	0.11	0.19	0	1.2	5.39	0
GAIF01022247.1	0	0	0	0	3.88	0
GAIF01022248.1	0.06	0	0	0	0	0
GAIF01022250.1	0.67	0.21	0.17	2.33	0.6	0.45
GAIF01022251.1	0.02	0.04	0	0.14	0.5	0
GAIF01022252.1	0.48	0.79	0.19	2.56	4.01	0
GAIF01022253.1	0	0	0.07	0	0	0
GAIF01022254.1	0.16	0.09	0	0.09	0	0
GAIF01022255.1	3.26	2.25	0.37	1.41	0	0.57
GAIF01022256.1	0	0	0	0	0.11	0
GAIF01022257.1	0.76	0.5	0.67	1.41	2.56	1.67
GAIF01022258.1	0.35	0.08	0.07	0.53	0.26	1.13
GAIF01022259.1	0.78	0.98	0.55	0.39	0.1	0
GAIF01022260.1	5.2	11.54	5.63	16.32	11.34	1.26
GAIF01022261.1	0.23	0.29	0.57	0.13	0.34	0
GAIF01022262.1	29.62	26.27	17.61	47.85	36.63	15.57
GAIF01022263.1	0.13	0	0	0	1.96	0
GAIF01022264.1	42.08	22.97	40.59	122.53	192.7	34.08
GAIF01022265.1	0.86	5.04	2.89	0	0	0.21
GAIF01022266.1	0.16	0.23	0	0.06	0.37	0.06
GAIF01022267.1	11.85	13.57	9.65	29.35	37.11	4.01
GAIF01022268.1	8.02	8.82	10.96	22.46	28.16	13.47
GAIF01022270.1	12.42	12.39	15.9	27.53	14.73	6.28
GAIF01022272.1	604.13	1054.11	1173.87	233.01	234.97	390.88
GAIF01022273.1	1.99	0.12	0	0	0	0

GAIF01022274.1	0	0	0	0.16	2.47	0
GAIF01022275.1	3.54	0.7	0.15	3.24	6.9	0.69
GAIF01022276.1	0.07	0	0	0.42	1.8	0.11
GAIF01022277.1	0.17	0.09	0.09	0.59	2.84	0.09
GAIF01022278.1	0	0.03	0	0	0	0
GAIF01022279.1	0	0	0	0.04	0	0
GAIF01022280.1	14.68	16.6	13.5	43.74	54.77	12.65
GAIF01022281.1	0	0.18	0	0	0.1	0
GAIF01022283.1	0	0	0.31	0.33	0	0
GAIF01022284.1	0.92	0.52	0.7	0.28	0.19	0
GAIF01022285.1	7.39	8.59	9.36	9.89	4.66	2.16
GAIF01022286.1	0	0	0.24	0	0	0
GAIF01022287.1	0	0	0	0.03	0.09	0.03
GAIF01022288.1	28.46	8.27	6.96	158.3	311.42	11.99
GAIF01022290.1	0.26	0	0	0.15	0.15	0
GAIF01022291.1	0.54	0	0	0	0	0
GAIF01022293.1	0	0	0	0	0.13	0
GAIF01022294.1	13.13	8.87	10.39	48.46	32.33	8.25
GAIF01022295.1	0	0	0.24	0	0.26	0
GAIF01022296.1	2.53	8.74	6.28	5.37	10.59	0.73
GAIF01022297.1	0.06	0.07	0.07	0	0.22	0
GAIF01022298.1	5.62	3.78	4.53	8.51	9.45	4.19
GAIF01022301.1	0.13	0	0	0.24	0.86	0
GAIF01022302.1	0.24	0	0.08	0.19	0.21	0
GAIF01022303.1	0	0	0	0.34	0	0
GAIF01022304.1	6.88	4.05	1.97	12.81	25.45	2.18
GAIF01022305.1	0	0.06	0	0	0	0
GAIF01022307.1	24.22	31.28	24.98	56.29	45.3	15.3
GAIF01022309.1	738.19	2218.74	2282.5	337.84	306.58	400.4
GAIF01022310.1	0	0.17	0.04	0.18	0.9	0
GAIF01022313.1	0.21	0.84	1	0.24	0	0.39
GAIF01022314.1	24.5	7.49	4.87	29.79	36.34	19.16
GAIF01022315.1	5.4	4.51	2.2	25.37	24.9	1.3
GAIF01022316.1	16.67	8.57	12.67	44.76	28.53	25.54
GAIF01022318.1	1	0	0.18	2.3	13.54	0.47
GAIF01022319.1	0.13	0.04	0	1.25	4.29	0.43
GAIF01022320.1	0.06	0	0	0.07	0	0
GAIF01022322.1	0	2.13	4.53	0	0	0
GAIF01022326.1	7.8	15.46	9.08	22.97	21.34	0.72
GAIF01022329.1	54.78	71.46	46.11	114.81	71.06	13.5
GAIF01022330.1	36.35	54.76	37.9	109.72	79.5	23.84
GAIF01022331.1	0.05	0.11	0	0.06	0.24	0
GAIF01022332.1	0	0.13	0	0	0	0
GAIF01022334.1	0	0	0	0.99	0	0

GAIF01022335.1	0	0.19	0	1.46	2.29	0.14
GAIF01022336.1	0.23	0.05	0	0.96	8.04	0.4
GAIF01022337.1	0	0	0	0.8	0	0
GAIF01022338.1	2.78	0	2.13	23.2	3.23	28.25
GAIF01022339.1	2.08	0.33	0.3	5.28	15.29	8.21
GAIF01022340.1	8.13	9.16	10.2	15.62	15.08	6.24
GAIF01022341.1	0.09	0	0	0	0.11	0
GAIF01022342.1	0	5.19	7.52	0	0.77	0
GAIF01022343.1	97.03	62.48	176.41	55.42	21.94	22.7
GAIF01022344.1	0.17	0	0	0.71	2.25	0.19
GAIF01022346.1	0	0	0	0	0.31	0
GAIF01022349.1	0.64	0	0	1.25	4.65	3.43
GAIF01022351.1	0	0.06	0	0.2	2.09	0.06
GAIF01022352.1	14.26	6.43	6.18	26.28	32.37	14.17
GAIF01022354.1	1.74	0.41	0.55	6.64	13.32	0.14
GAIF01022355.1	7.87	7.47	9.58	1.49	0.86	4.42
GAIF01022356.1	20.05	29.85	32.6	24.04	12.4	7.06
GAIF01022360.1	0	0	0	0.09	0	0
GAIF01022362.1	0	0.27	0	0.14	0.21	0
GAIF01022363.1	1.48	0.16	0.11	9.78	52.56	0.65
GAIF01022364.1	13.12	11.7	8.32	34.14	54.37	11.19
GAIF01022365.1	49.22	69.74	76.56	51.13	25.19	16.92
GAIF01022366.1	0.29	0.32	0	1.35	8.27	0.21
GAIF01022368.1	0	0	1.78	0	0	0
GAIF01022370.1	0	0.31	0	0.42	1.63	0
GAIF01022373.1	29.96	21.01	30.71	47.83	53.72	84.17
GAIF01022374.1	12.73	5.8	0.23	23.59	51.59	52.03
GAIF01022375.1	0.15	0.08	0	0.09	0	0.08
GAIF01022376.1	5.81	1.79	0.79	0.49	0.2	0.2
GAIF01022377.1	0.44	0.04	0	2.02	11.13	0.92
GAIF01022379.1	1204.29	900.12	1044.32	591.29	379.88	921.06
GAIF01022381.1	0	0.1	0	88.92	433.66	13.06
GAIF01022384.1	0.71	0.12	0.24	3.32	15	2.12
GAIF01022386.1	0	0.3	0.33	0	0	0.64
GAIF01022387.1	0.37	0.1	0	0	0.32	0.1
GAIF01022388.1	0	0	0	0	0.99	0
GAIF01022390.1	3.49	4.86	2.27	0.38	0.39	0.12
GAIF01022392.1	1.16	0.28	0.12	4.95	14.51	0.24
GAIF01022393.1	4.18	0.38	0	8.95	10.27	5.16
GAIF01022394.1	0.03	0.03	0	0	0.13	0
GAIF01022396.1	9.53	8.6	17.11	25.87	13.4	32.75
GAIF01022397.1	1.57	0	0	5.28	13.79	0.17
GAIF01022398.1	0.2	0.27	0.38	0.11	0	0.05
GAIF01022399.1	0	0	0	0	0.34	0

GAIF01022400.1	0.04	0	0	0	0.05	0
GAIF01022401.1	12.54	11.84	5.96	39.76	42.51	1.53
GAIF01022405.1	0	0.06	0	0	0.13	0
GAIF01022406.1	1.15	0.61	0.07	3.93	21.53	1.63
GAIF01022407.1	0.2	0.38	0.22	1.82	1.46	0.06
GAIF01022408.1	0	0	0	0	0.06	0
GAIF01022410.1	0	0	0	0	0.02	0
GAIF01022411.1	0.12	0	0.14	0.72	1.5	0.55
GAIF01022413.1	13.55	15.41	16.54	31.34	17.58	4.13
GAIF01022414.1	1.66	0.25	0.32	4.3	7.05	1.97
GAIF01022415.1	50.41	89.84	41.99	64.22	68.85	8.55
GAIF01022416.1	3.29	3.77	0	4.42	18.49	2.96
GAIF01022417.1	0.45	0.23	0	0.49	2.24	0
GAIF01022419.1	0.12	0	0	0.43	0.44	0
GAIF01022420.1	43.34	70.75	47.33	253.59	243.81	44.77
GAIF01022421.1	14.18	17.48	10.08	5.99	3.66	0.9
GAIF01022422.1	0	0.14	0	0	0	0
GAIF01022424.1	7.75	10.19	17.99	31.7	26.77	10.29
GAIF01022426.1	255.47	229.12	201.78	166.77	109.18	69.16
GAIF01022427.1	0	0	0	0.41	0	0.06
GAIF01022428.1	14.99	8.27	6.26	18.83	39.65	25.13
GAIF01022429.1	618.5	168.71	441.82	994.45	564.56	981.91
GAIF01022430.1	7.7	8.06	10.38	25.09	20.94	5.39
GAIF01022433.1	0	0	1	0	0	0
GAIF01022434.1	0	0	0	1.44	15.03	0
GAIF01022435.1	20.03	36.17	47.94	14.96	26.21	7.32
GAIF01022436.1	34.41	30.84	40.01	37.69	50.24	47.06
GAIF01022438.1	5097.58	3156.26	3912	37.14	33.38	82.26
GAIF01022439.1	0.07	0	0	0.04	0.38	0
GAIF01022440.1	0	0	0	0.12	0	0.07
GAIF01022441.1	1.72	1.77	0.87	2.3	0.53	0.05
GAIF01022442.1	0.52	0.75	0.19	1.2	3.74	0
GAIF01022443.1	0.34	0.04	0	1.39	5.42	0
GAIF01022445.1	0	0	0	0	0.08	0
GAIF01022446.1	0	0	0	0	0.75	0
GAIF01022447.1	0	0.67	0.73	0	0.07	0.06
GAIF01022449.1	7.68	10.6	3.18	56.92	47.38	2.04
GAIF01022450.1	25.81	24.36	20.14	92.55	49.53	9.08
GAIF01022453.1	4.77	4.67	2.61	22.13	33.84	1.09
GAIF01022454.1	1.16	0	0	2.51	11.8	0.55
GAIF01022455.1	70.85	46.76	30.56	36.01	34.52	10.92
GAIF01022457.1	0.07	0.16	0.08	2.54	12.11	0.4
GAIF01022458.1	0	0.03	0	0.58	5.68	0.2
GAIF01022460.1	0.06	0	0	0	0.07	0

GAIF01022461.1	0	0	0	0	0.67	0
GAIF01022465.1	13.64	9.78	6.32	21.84	17.84	20.14
GAIF01022466.1	0.11	0.24	0.36	0	0	0
GAIF01022471.1	0	0	0	0	0.07	0
GAIF01022472.1	8.85	12.47	5.16	30.61	19.24	2.41
GAIF01022473.1	0	0	0	0	0.1	0
GAIF01022474.1	0	0	0	0.17	0	0
GAIF01022475.1	0	0	0	0	0.28	0
GAIF01022476.1	0	235.49	726.6	0	0	97.23
GAIF01022478.1	11.16	18.53	18.07	66.64	63.04	8.83
GAIF01022480.1	3.29	0.77	0.29	0.24	0.36	0
GAIF01022481.1	0	0	0	0	0.1	0
GAIF01022482.1	26.39	39.48	28.57	49.48	38.65	5.15
GAIF01022483.1	0.18	0.71	0.07	0.07	0.65	0.07
GAIF01022484.1	0.09	0	0	0.25	0.95	0
GAIF01022485.1	1.01	2.19	0.64	0.29	0.1	0
GAIF01022486.1	260.26	125.46	81.58	124.06	68.98	26.12
GAIF01022487.1	16.75	28.99	15.95	16.01	13.91	5.2
GAIF01022488.1	0	0	0	0	0.41	0
GAIF01022489.1	0.03	0.04	0	0	0.02	0
GAIF01022490.1	3.25	1.57	2.38	3.07	2.17	2.92
GAIF01022491.1	0	0	0	0.49	1.88	0
GAIF01022493.1	80.86	115.88	99.79	13.92	10.42	2.45
GAIF01022494.1	0	0	0	0	0.14	0
GAIF01022495.1	0.17	0	0	0.06	0.87	0
GAIF01022496.1	9.89	11.15	1.78	63.97	122.17	8.35
GAIF01022497.1	0	0	0.01	0.12	0.25	0.01
GAIF01022498.1	9.49	1.55	0.46	0.45	0	0
GAIF01022500.1	0.25	0.14	0	0	0.76	0
GAIF01022501.1	43.68	58.8	41.81	66.91	69.36	13.77
GAIF01022502.1	0.01	0	0.01	0.03	0.01	0
GAIF01022503.1	0.06	0	0	1.12	0	0
GAIF01022504.1	5.01	3.52	0.76	15.41	27.55	0.02
GAIF01022506.1	1.24	0.67	0	1.43	32.33	0
GAIF01022508.1	11.37	12.59	10.76	21.88	0	2
GAIF01022510.1	0	0.03	0	0.1	0	0
GAIF01022513.1	5.39	10.85	6.04	0.97	0.89	0.14
GAIF01022516.1	88.81	71.95	45.19	137.71	78.72	12.47
GAIF01022518.1	0.1	0.21	0	0.8	3.42	0
GAIF01022519.1	0	0	0	0	0.14	0
GAIF01022520.1	0	1.16	3.63	0.32	0	1.86
GAIF01022521.1	3.17	4.12	2.98	7.9	8.62	1.92
GAIF01022522.1	0.09	0	0.1	0	0	0
GAIF01022523.1	16.93	13.51	18.99	49.15	58.02	13.31

GAIF01022525.1	6.6	9.09	4.45	14.99	17.8	1.19
GAIF01022527.1	74.21	0.66	1.13	8.58	1.76	0.75
GAIF01022530.1	0	0	0	0	0.35	0
GAIF01022531.1	5.94	4.43	2.3	7.66	1.85	1.33
GAIF01022532.1	27.17	10.77	25.78	7.83	4.22	11.98
GAIF01022533.1	0.12	0.25	0	0.83	2.92	0.67
GAIF01022534.1	0	0.1	0	0	0.11	0
GAIF01022535.1	4.89	16.69	7.82	0	0.02	42.81
GAIF01022537.1	15.58	9.2	18.48	11.29	8.77	15.58
GAIF01022538.1	0	0	0	0	0.02	0
GAIF01022539.1	0.44	0.36	0	0.51	3.71	1.58
GAIF01022541.1	0.05	0	0	0.02	0	0
GAIF01022542.1	0	0.13	0	0	0.15	0
GAIF01022543.1	38.05	29.48	8.63	92.65	87.23	5.1
GAIF01022544.1	14.71	18.82	30.01	46.7	32.7	35.04
GAIF01022545.1	3.94	2.05	1.4	1.75	1.01	0.77
GAIF01022547.1	3	1.63	0.51	11.92	25.81	0.76
GAIF01022548.1	0.13	0	0	0	0	0
GAIF01022549.1	0	0	0	0.51	0.26	0
GAIF01022550.1	0	0.03	0	0	0	0
GAIF01022551.1	0.26	0.62	0.3	0.4	0.3	0.13
GAIF01022552.1	0	0.17	0	0	0	0
GAIF01022553.1	0.53	0.48	0	0.62	2.78	0.29
GAIF01022554.1	0.15	0.08	0.23	0.64	0.61	0.05
GAIF01022555.1	53.88	47.45	40.77	13.24	9.1	6.01
GAIF01022556.1	0	1.23	1.88	0	0.12	0.23
GAIF01022557.1	127.75	124.64	77.82	22.65	23.32	30.08
GAIF01022558.1	0.2	0	0.14	1.37	5.46	0.14
GAIF01022559.1	4.56	1.26	0.3	25.25	79.86	1.15
GAIF01022560.1	19.73	34.76	22.4	58.74	30.76	6.3
GAIF01022561.1	0.5	0.44	0.9	0	0.05	0.1
GAIF01022562.1	34.09	8.66	12.01	9.14	6.19	16.61
GAIF01022565.1	0.26	0	0	0.9	3.43	0.14
GAIF01022566.1	1.85	3.28	0	1.58	4.33	0
GAIF01022567.1	0	0.3	0.21	0.13	0	0.14
GAIF01022569.1	0.08	0	0	0.03	0.29	0
GAIF01022570.1	0.43	0.28	0	1.3	5.49	0
GAIF01022571.1	0.11	0.12	0	0.65	3.77	2.71
GAIF01022573.1	13.35	5.73	4.29	22.03	11.52	3.57
GAIF01022575.1	0.09	0.09	0	0	0	0
GAIF01022576.1	1.45	0.71	0.17	5.59	7.9	0.17
GAIF01022578.1	0.19	0.06	0.03	0.89	4.68	0.12
GAIF01022579.1	0.19	0	0	0	0.43	0.85
GAIF01022581.1	606.28	208.2	136.37	180.78	279.45	208.86

GAIF01022582.1	0	0	0	0	0.04	0.1
GAIF01022583.1	0	2.88	0.7	0	0.09	0.55
GAIF01022584.1	95.09	83.1	65.44	86.62	69.79	72.02
GAIF01022585.1	40.89	25.15	30.54	67.51	24.63	52.24
GAIF01022587.1	0	0	0	0	0.11	0
GAIF01022588.1	0	0	0.05	0.17	3.1	0
GAIF01022589.1	0	0	0	0.05	0.05	0
GAIF01022590.1	11.95	42.94	18.35	31.71	36.09	1.64
GAIF01022592.1	1.25	0.11	0	3.98	29.26	8.25
GAIF01022593.1	0.63	0	0	0.85	6.1	0
GAIF01022594.1	5.2	2.18	0	8.64	12.56	0.6
GAIF01022596.1	0	0	0.06	0.29	1.43	0.06
GAIF01022597.1	0	0	0	0.13	0	0
GAIF01022598.1	0	0.25	0.12	0.02	0.24	0
GAIF01022599.1	3.13	1.61	3.15	4.74	2.32	2.7
GAIF01022600.1	0	0	0	0	0.11	0
GAIF01022602.1	0.46	0.27	0.13	1.84	6.24	2.49
GAIF01022603.1	0	0	0.04	0.14	1.52	0.05
GAIF01022605.1	2.23	1.8	3.57	0.95	0.33	2.29
GAIF01022606.1	0.49	0.93	0	0.43	0	0
GAIF01022607.1	0.78	0.21	0	2.07	10.04	0.3
GAIF01022608.1	22.56	27.6	18.14	14.58	15.17	1.72
GAIF01022610.1	0.18	0.06	0.09	0.06	0.61	0
GAIF01022611.1	5.75	7.79	6.5	8.84	9.37	4.54
GAIF01022612.1	0	0.07	0	0.11	0.15	0.1
GAIF01022613.1	0.01	0	0	0	0	0
GAIF01022615.1	3.74	1.84	0.49	0.52	0.75	0.1
GAIF01022617.1	0.19	0.36	0	0.06	0.08	0.24
GAIF01022619.1	6.47	8.9	6.27	15.02	8.7	3.5
GAIF01022621.1	6.17	2.62	2.55	14.39	16.84	9.6
GAIF01022622.1	1.39	0.48	0	1.39	2.35	0.07
GAIF01022623.1	0.05	0.06	0	2.09	13.43	0
GAIF01022624.1	0	0.33	0	0.26	0	0
GAIF01022625.1	6.01	8.13	7.66	18.1	17.99	4.27
GAIF01022626.1	17.98	6.8	4.07	36.31	27.54	11.49
GAIF01022627.1	0	0	0	0	0.04	0
GAIF01022628.1	0	0.03	0.03	2.38	5.71	0
GAIF01022629.1	0.11	0.23	0.12	0.52	2.54	0.05
GAIF01022630.1	0.29	0	0	0.46	0.29	0
GAIF01022631.1	0.1	0.2	0	0	1.65	0
GAIF01022633.1	0.23	0.06	0	0.24	2.43	0
GAIF01022636.1	0.33	0.18	0	0.78	6.24	0.18
GAIF01022637.1	66.36	28.5	70.64	49.59	34.9	21.93
GAIF01022638.1	0	0	0.15	0	0	0

GAIF01022641.1	0	0	0	0	0.26	0
GAIF01022642.1	0	0	0	0	0.33	0
GAIF01022644.1	0	0	0.02	0	0	0
GAIF01022645.1	2.42	0.74	0.11	6.4	29.92	0.11
GAIF01022646.1	0.48	2.6	1.99	5.1	6.67	0
GAIF01022648.1	0	0	0	0	0.6	0
GAIF01022649.1	0.39	0.57	0.72	1.07	0.47	0
GAIF01022651.1	0	0	0	0	0.04	0
GAIF01022652.1	0	0	0	0.18	1.23	0
GAIF01022653.1	0.14	0.29	0	0	0	0
GAIF01022654.1	334.18	527.99	618.57	1007.98	674.73	43.12
GAIF01022655.1	0	0	0	0.45	7.07	0
GAIF01022656.1	19.86	43.19	18.3	13.17	19.3	2.64
GAIF01022657.1	0.1	0	0	2.48	11.99	0.35
GAIF01022660.1	2.02	1.72	4.15	4.18	7.82	4.94
GAIF01022661.1	0.07	0	0.12	1.85	7.92	1.06
GAIF01022663.1	0.14	1.34	0	0.36	0.35	0
GAIF01022665.1	13.51	16.7	4.16	12.25	20.56	6.38
GAIF01022667.1	0	0	0	0.27	0	0
GAIF01022668.1	0.96	1.97	0.42	0.86	2.06	0.29
GAIF01022669.1	0.04	0	0	0.3	1.18	0
GAIF01022670.1	0	0.31	1.3	0.8	7.44	0.08
GAIF01022671.1	1.75	0.15	0.07	3.75	18.63	13.36
GAIF01022672.1	0	0	0	0.23	0.24	0
GAIF01022675.1	0.18	0	0	2.14	5.87	0.41
GAIF01022676.1	0	0	0	0	0.11	0
GAIF01022677.1	265.72	105.97	159.98	515.72	281.67	530.42
GAIF01022679.1	2.96	3.83	3.08	13.56	14.39	2.37
GAIF01022680.1	0.04	0	0	0	0	0
GAIF01022681.1	0.15	0.17	0	0.71	0.74	0
GAIF01022682.1	0	0	0	4.4	3.04	2.17
GAIF01022683.1	37.46	49.35	30.62	16.62	9.06	30.68
GAIF01022684.1	368.27	362.47	511.63	297.46	228.81	462.84
GAIF01022685.1	0.09	0	0.19	1.23	5.73	0.15
GAIF01022686.1	0	0	0	0.2	0.44	0
GAIF01022687.1	0	0.12	0.5	0.13	0	0
GAIF01022688.1	0	0	0	0	1	0
GAIF01022689.1	14.2	16.41	15.23	25.3	24.47	7.52
GAIF01022690.1	0	0	0	0.08	0.43	0
GAIF01022691.1	5.07	3.81	4.51	10.23	3.05	2.53
GAIF01022692.1	0.68	1.12	0.88	0.53	0.14	0.25
GAIF01022693.1	0.71	0.34	0.09	2.29	6.08	1.74
GAIF01022695.1	0.38	0.55	0	0	0.3	0
GAIF01022696.1	0	0	0	0	0.35	0

GAIF01022697.1	22.07	12.41	27.71	35.48	13.42	39
GAIF01022699.1	40.96	39.96	34.41	48.53	38.83	33.32
GAIF01022700.1	1.03	0.04	0	3.03	0	0
GAIF01022702.1	0	0	0	0	0.7	0.22
GAIF01022704.1	0	0.86	1.92	0	0	1.05
GAIF01022705.1	0	0	0.19	0.2	0.24	0
GAIF01022707.1	0	0	0	0	0.12	0
GAIF01022708.1	0.71	0.34	0.48	0.22	0.11	0.07
GAIF01022710.1	0	0.07	0.04	0	0.14	0.11
GAIF01022711.1	6.77	3.18	6.7	23.67	9.85	7.83
GAIF01022712.1	0.97	4.59	2.13	5.86	8.4	0.15
GAIF01022713.1	0.21	0.23	0	1.14	6.1	0.16
GAIF01022714.1	0	0	0.14	0	0	0
GAIF01022715.1	0	0	0	0.49	0	0
GAIF01022716.1	9.18	12.48	13.97	11.27	7.19	7.75
GAIF01022717.1	0.03	16.57	10.02	0.15	29.9	2.13
GAIF01022719.1	5.83	0	0	4.51	4.77	2.14
GAIF01022720.1	11.24	14.07	11.27	26.13	23.49	3.37
GAIF01022721.1	19.87	12.04	11.96	30.85	27.23	16.75
GAIF01022722.1	0	0	0.03	0.2	0.77	0.08
GAIF01022723.1	11.24	15.49	5.99	23.76	20.11	2.52
GAIF01022724.1	13.81	11.21	7.95	45.89	58.54	3.97
GAIF01022725.1	90.94	138.25	95.44	83.26	50.47	35.55
GAIF01022726.1	0	0	0	0	3.79	0
GAIF01022727.1	0.13	0	0	0	0	0
GAIF01022729.1	0	0.22	0	0	0.82	0.43
GAIF01022730.1	0	0.18	0	0	0	0
GAIF01022731.1	40.47	37.57	62.56	90.63	53.08	70.22
GAIF01022732.1	0.32	0.64	0.21	0.32	0	0.07
GAIF01022734.1	0.12	0	0	0	0	0
GAIF01022736.1	9.93	12.31	7.91	16.41	15.45	7.87
GAIF01022737.1	0	0	0	0	0.28	0
GAIF01022740.1	0	0	0	0.17	0.17	0
GAIF01022742.1	7.54	4.38	7.63	24.21	22.49	13.91
GAIF01022743.1	0	0	0	0.19	0.1	0
GAIF01022745.1	0	0	0	0.08	0.21	0
GAIF01022746.1	0.66	0.21	0.47	1.32	5.65	0.46
GAIF01022747.1	38.09	19.11	20.39	35.35	47.56	36.56
GAIF01022748.1	0.53	0.19	0.15	0.87	2.86	0.1
GAIF01022751.1	0.48	0	0	0.08	0	0
GAIF01022753.1	0.38	0	0.34	4.43	12.95	0.25
GAIF01022754.1	129.95	190.13	259.8	118.87	102.88	179.33
GAIF01022755.1	0	0	0	2.65	8.06	0
GAIF01022757.1	0	0	0.1	0	0	0

GAIF01022758.1	10.88	12.05	4.6	1.27	3.12	0.33
GAIF01022759.1	0.24	0	0	0.21	0.93	0.2
GAIF01022760.1	60.16	12.37	3.61	497.7	817.6	373.93
GAIF01022761.1	6.11	11.67	5.11	28.24	23.53	1.31
GAIF01022762.1	5.27	4.59	9.07	7.34	5.71	3.1
GAIF01022763.1	26.59	42.41	24.86	61.09	50.94	4.85
GAIF01022764.1	2.85	2.63	0.96	8.53	5.77	0
GAIF01022766.1	8.91	6.28	5.71	18.09	25.01	1.74
GAIF01022767.1	0	4.1	2.51	0	3	0
GAIF01022768.1	1.68	2.63	0.64	6.46	12.24	0.65
GAIF01022770.1	29.74	125.35	70.39	77.22	77.2	9.21
GAIF01022771.1	2.91	2.01	0	14.03	46.62	0.86
GAIF01022772.1	2.21	0.08	0	1.17	9.8	5.96
GAIF01022773.1	0.68	0.56	0.09	2.78	8.03	0.66
GAIF01022774.1	9.4	10.53	7.93	26.03	29.61	2.58
GAIF01022775.1	0	0.27	0	0	0	0
GAIF01022777.1	0	0	0	0	0.24	0.22
GAIF01022778.1	2.4	0.71	0.22	3.68	19.97	1.16
GAIF01022779.1	0	0	0	0.11	0	0
GAIF01022780.1	0.81	0.44	1.79	0	0	0.22
GAIF01022781.1	0	0.16	0	0	0	1.59
GAIF01022782.1	0	0	0	0	0.12	0
GAIF01022785.1	42.37	26.65	14.03	7.69	7.77	2.9
GAIF01022786.1	37.27	51.4	54.09	57.08	37.48	9.21
GAIF01022788.1	0	0	0.72	0	0	0
GAIF01022789.1	0	0	0	0.06	0.07	0
GAIF01022790.1	0.49	0.61	0	3.92	5.05	0
GAIF01022791.1	37.64	18.78	14.75	0.19	0.3	0.18
GAIF01022792.1	0.07	0	0.03	0.14	0.55	0.05
GAIF01022793.1	0	0.02	0	0.22	0.3	0
GAIF01022796.1	1197.06	626.15	667.74	694.42	727.84	2522.63
GAIF01022798.1	0.44	0	0	0.26	5.59	0
GAIF01022799.1	12.66	6.9	11.89	13.6	7.95	1.28
GAIF01022801.1	0	0	0	0.17	0.17	0
GAIF01022802.1	0	0.11	0	0.12	0	0
GAIF01022803.1	72.63	85.49	135.68	41.54	44.73	40.61
GAIF01022804.1	0.37	0.05	0	2.02	9.16	0.25
GAIF01022805.1	1.46	1.15	0	6.91	24.66	0.87
GAIF01022806.1	0	0	0	0	0.49	0
GAIF01022807.1	0.23	0.51	0.39	0.51	0.42	0
GAIF01022808.1	0.59	0.33	0.28	1.01	0.26	0.02
GAIF01022810.1	0.37	0.24	0.08	2.66	12.91	0.41
GAIF01022811.1	0.28	0.31	0.1	0	0.34	0
GAIF01022812.1	25.35	29.87	24.83	49.77	23.15	8.1

GAIF01022813.1	0	0	0	0.05	0.14	0
GAIF01022814.1	20.75	47.01	14.95	0.87	0.45	0.48
GAIF01022815.1	0.11	0	0	0.14	0	0
GAIF01022816.1	3.01	0.93	0.79	5.48	5.16	7.41
GAIF01022817.1	0.2	0.22	0.11	2.32	5.71	1.82
GAIF01022820.1	3.74	15.61	14.57	10.58	3.13	9.51
GAIF01022821.1	0	0	0	0.12	0.12	0.11
GAIF01022822.1	3.17	3.29	2.37	1.84	0.43	1.19
GAIF01022823.1	0.08	0	0	0	0.05	0
GAIF01022824.1	0.15	1.99	0.32	1.03	1.38	0.05
GAIF01022825.1	0	0	0	0	0	0.34
GAIF01022826.1	0.39	0	0	1.9	3.89	0
GAIF01022827.1	358.65	214.28	114.33	39.72	41.28	49.36
GAIF01022828.1	0	0	0.08	0	0.09	0
GAIF01022829.1	17.54	20.24	21.23	21.21	15.71	13.45
GAIF01022830.1	0.91	0.33	0.67	1.41	0.18	0
GAIF01022831.1	0	0	0	0	2.28	0
GAIF01022832.1	0	0	0	0.1	0	0
GAIF01022834.1	0	0	0	0.34	0	0
GAIF01022835.1	0.26	0.24	0	1.92	4.81	0.05
GAIF01022836.1	1.72	0	0.47	12.97	54.35	8.12
GAIF01022838.1	5.79	8.8	6.49	4.43	4.44	2.22
GAIF01022840.1	0.11	0	0	0.18	0.95	0
GAIF01022841.1	0.61	1.07	0.36	5.91	1.09	0
GAIF01022842.1	0.14	0	0	0	0	0
GAIF01022845.1	6.88	12.75	14.93	15.6	6.77	12.06
GAIF01022846.1	21.25	23.74	8.5	39.96	34.74	3.81
GAIF01022847.1	0	0	0	0.1	0.13	0
GAIF01022848.1	0	0	0	0.05	0.05	0
GAIF01022852.1	0.12	0.17	0	0.25	0.24	0
GAIF01022853.1	12.5	15.91	17.81	35.3	24.31	5.18
GAIF01022856.1	0.41	0.89	0	1.6	1.66	0
GAIF01022858.1	0.1	0	0	0	0	0
GAIF01022860.1	0.86	1.2	1.82	7.9	9.2	3.43
GAIF01022861.1	0	0	0.19	0.13	0.07	0.12
GAIF01022862.1	0	0.09	0	0.67	2.38	0.18
GAIF01022863.1	0	0.1	0	0	0	0
GAIF01022864.1	8.81	19.35	3.79	3.81	1.25	0
GAIF01022866.1	0	0	0	0	0.15	0
GAIF01022867.1	13.81	23.77	2.23	106.06	179.3	3.38
GAIF01022868.1	0	0	0	0	0.06	0
GAIF01022869.1	0	0	0	0.1	0.41	0.22
GAIF01022870.1	0	0.27	0	0	0.79	1.02
GAIF01022872.1	15.15	6.04	4.16	29.1	35.73	10.63

GAIF01022873.1	0.18	0.1	0.05	0.47	5.24	0.35
GAIF01022874.1	0.25	0	0	0	0	0.14
GAIF01022875.1	0	0	0	0	0.1	0
GAIF01022876.1	0	0.84	0.37	2.53	2.13	0
GAIF01022879.1	4.19	5.03	0.72	1.68	8.63	0.14
GAIF01022880.1	13.45	11.7	14.89	12.49	12.8	22.3
GAIF01022882.1	0.18	0	0	0.2	0	0
GAIF01022883.1	3.99	5.79	0.32	9.27	12.99	0.65
GAIF01022884.1	0.91	0.18	0	2.4	4.82	3.95
GAIF01022886.1	0	0.1	0	0	0.78	0
GAIF01022887.1	0	0	0	0	0.22	0
GAIF01022888.1	0.17	0	0	0	0	0
GAIF01022889.1	0	0	0	0	0.31	0
GAIF01022891.1	0	0.15	0	0.36	1.95	0
GAIF01022892.1	18.11	14.93	6.31	3.53	5.53	2.12
GAIF01022894.1	0	0	0	0.11	0.19	0
GAIF01022895.1	0	0	0	0	0.16	0.05
GAIF01022896.1	31.81	46.55	32.12	177.94	102.24	11.23
GAIF01022899.1	0	0.26	0.12	0	10.34	0
GAIF01022900.1	0.18	0	0	0.83	3.21	0.54
GAIF01022901.1	12.12	8.82	5.8	11.87	6.08	2.57
GAIF01022904.1	0	0	0	0	0.22	0
GAIF01022905.1	1.73	0.24	1.46	0.84	0.28	0.51
GAIF01022906.1	171.24	188.19	223.52	89.3	74.67	129.7
GAIF01022907.1	0	0	0.01	0	0	0
GAIF01022908.1	5.42	5.46	4.55	13.88	15.36	2.58
GAIF01022909.1	0.02	0	0	0.02	0.43	0
GAIF01022910.1	0	3.21	0	0	0	0
GAIF01022911.1	18.13	27.46	8.92	89.32	77.9	6.66
GAIF01022912.1	0.09	0.2	0	0	0	0
GAIF01022913.1	28.14	26.49	21.24	80.66	78.96	15.83
GAIF01022914.1	14.12	5.61	6.72	19.35	13.23	15.47
GAIF01022918.1	18.18	10.56	11.83	11.94	14.04	15.15
GAIF01022919.1	0.8	0.87	0	5.67	7.57	2.51
GAIF01022920.1	0.1	0.21	0	1.1	1.65	0.11
GAIF01022922.1	0.07	0	0	0.23	0.8	1.54
GAIF01022923.1	0	0	0	0.37	0	0
GAIF01022924.1	45.29	51.83	77.92	101.82	51.95	27.93
GAIF01022925.1	31.41	38.11	46.2	78.73	48.95	15.51
GAIF01022926.1	2.76	1.59	1.31	1.08	0.91	0.52
GAIF01022927.1	11.04	6.44	3.35	8.36	8.47	0.6
GAIF01022928.1	809.11	45.78	27.9	1322.55	106.38	73.04
GAIF01022929.1	0	0	0	0	0.23	0
GAIF01022930.1	14.81	23.28	17.56	0.82	1	0.25

GAIF01022931.1	0.99	0.66	0.08	2.29	0.55	0
GAIF01022932.1	0.21	0.12	0.08	0.09	0.46	0
GAIF01022934.1	0.06	0	0	0.21	1.4	0.03
GAIF01022936.1	1.02	0.55	0	0.29	0.31	0
GAIF01022937.1	0	0.06	0	0.12	0	0
GAIF01022938.1	0.18	0	0	0.83	4.66	0.04
GAIF01022939.1	161.25	242.6	160.36	289.9	244.36	63.9
GAIF01022940.1	0	0	0	0.85	3.4	0
GAIF01022942.1	0	0	0	0.2	4.05	0
GAIF01022943.1	88.88	71.19	179.49	39.97	17.1	64.44
GAIF01022945.1	0.34	0.12	0.25	2.66	8.53	0.76
GAIF01022946.1	0.66	1.19	1.13	0.72	0.93	0.79
GAIF01022947.1	6.85	10.38	8.08	40.7	70.22	1.77
GAIF01022948.1	75.2	101.12	106.34	252	210.19	34.13
GAIF01022951.1	0.15	0.24	0.58	0.35	0.09	0.08
GAIF01022952.1	65.07	50.73	43.18	93.85	48.36	21.64
GAIF01022954.1	0	1.32	0	0.55	0	0
GAIF01022955.1	4.14	2.53	1.88	6.35	11.44	3.28
GAIF01022957.1	0.54	0.07	0.39	9.4	7.13	47.56
GAIF01022958.1	3.35	7.29	7.98	2.16	0.67	1.03
GAIF01022959.1	26.71	19.6	27.62	28.74	12.79	22.15
GAIF01022960.1	0.02	0.04	0.02	0.48	3.15	0.02
GAIF01022963.1	0.08	0.09	0	0	0.2	0
GAIF01022964.1	0	0.1	0.27	0	0.04	0.13
GAIF01022965.1	3.15	4.82	0.57	1.3	1.49	0.09
GAIF01022971.1	0	0.61	0	0.39	2.43	0.38
GAIF01022972.1	1.54	0	0.37	2.1	1.23	0
GAIF01022973.1	12.84	11.61	11.72	43.69	32.43	7.96
GAIF01022976.1	0.25	0.85	1.06	0.17	0.13	0
GAIF01022977.1	8.51	6.04	3.21	1.54	0.65	0.2
GAIF01022978.1	4.81	1.89	1.47	12.08	13.96	7.96
GAIF01022979.1	0	0	0	0.02	0.12	0
GAIF01022980.1	0.8	0	0.79	2.58	2.39	0
GAIF01022982.1	0.45	0.16	0	2.08	9.9	0
GAIF01022983.1	0.3	0.55	0.44	0.41	3.15	1.05
GAIF01022985.1	5.5	2.11	0.77	16.51	29.63	26.05
GAIF01022986.1	3.61	2.43	2.96	7.91	12.54	3.75
GAIF01022988.1	11.23	10.45	6.25	35.63	51.01	2.92
GAIF01022989.1	0	0.13	0.39	0	0	0.4
GAIF01022990.1	0	0.12	0.03	0.37	0.02	0.09
GAIF01022992.1	0	0.1	0	0.11	0	0
GAIF01022993.1	3.07	2.41	3.93	2.38	1.03	1.32
GAIF01022994.1	0	0	0	0	0.47	0
GAIF01022995.1	0.17	0	0.06	0.43	3.25	0

GAIF01022996.1	13.74	8.74	7.89	19.03	26.76	23.96
GAIF01022997.1	0	0	0	0	0	0.1
GAIF01022999.1	0.88	0.74	1.31	0.19	0.14	0.13
GAIF01023000.1	0	0.34	0.07	0.29	0	0
GAIF01023001.1	13.74	3.51	3.15	12.09	8.97	7.64
GAIF01023002.1	0	0.07	0	0.04	0.2	0
GAIF01023003.1	0	0	0	0	0.1	0.66
GAIF01023007.1	10.2	16	12.99	20.4	13.28	3.18
GAIF01023009.1	0	0.15	0.75	0	0	0
GAIF01023010.1	1.68	0.08	0	1.86	5.24	0.34
GAIF01023011.1	0	0	0	0	0.09	0
GAIF01023012.1	0.08	0.04	0	0	0	0.01
GAIF01023013.1	4.95	1.03	0.48	7.86	13.17	2.49
GAIF01023014.1	0	0.63	0.49	0	0	0.38
GAIF01023016.1	0	0	0.13	0	0	0
GAIF01023017.1	6.41	13.94	7.3	8.37	8.48	0.62
GAIF01023020.1	9.33	4.89	8.57	20.97	22.63	56.18
GAIF01023021.1	0	0.73	0.52	0	0.06	0.16
GAIF01023022.1	0	0	0	0.11	0	0
GAIF01023023.1	9.02	4.18	7.25	17	12.29	48.91
GAIF01023024.1	0	0	0	1.18	0.14	0
GAIF01023025.1	13.01	22.11	28.12	30.28	17.81	10.64
GAIF01023027.1	1.9	3.27	10.87	11.05	0	0.68
GAIF01023028.1	0.05	0	0	2.34	7.04	0.21
GAIF01023030.1	0.14	0	0	1.73	3.51	0
GAIF01023031.1	1.94	0.96	1.16	0.73	1.48	0.26
GAIF01023032.1	1.77	1.07	0.43	6.49	15.81	0.32
GAIF01023033.1	1.51	1.17	0	4.75	9.07	0.47
GAIF01023034.1	1.33	0.58	0.42	1.99	1.56	0.67
GAIF01023037.1	0.13	0	0	0.74	7.62	0
GAIF01023038.1	0.08	0.09	0	0	0	0.23
GAIF01023039.1	44.38	61.41	124.02	0	0	0
GAIF01023040.1	7.06	13.13	8.09	10.37	4.71	0.35
GAIF01023042.1	0.26	0.14	0	0	0	0.3
GAIF01023043.1	0	0.79	0.79	0.06	0.07	0.06
GAIF01023044.1	8.47	2.79	1.44	9.59	30.2	14.99
GAIF01023046.1	0	0.15	0	0.16	0.66	0.15
GAIF01023047.1	0.37	0.38	0.35	0.88	0.45	0.54
GAIF01023048.1	0	0.19	0	0	0	0
GAIF01023049.1	1.49	0.96	0.8	0.3	0.11	0
GAIF01023050.1	0	0.06	0.06	0.77	4.45	0
GAIF01023053.1	3.73	2.67	0.93	4.43	7.55	5.89
GAIF01023055.1	0.1	0.11	0.05	1.64	5.11	0.11
GAIF01023058.1	0.51	0.45	0.3	1.18	5.34	0

GAIF01023060.1	0.26	0.28	0.08	0.31	1.57	0
GAIF01023061.1	0	0	0	0.15	0	0
GAIF01023063.1	0	0	0.05	0.25	0.15	0
GAIF01023064.1	0.53	0.12	0.23	0.74	0	0.23
GAIF01023066.1	0.69	0.3	0.15	0.32	0.66	0.15
GAIF01023067.1	0	0	0	0.66	4.98	0.18
GAIF01023068.1	0.03	0.2	0	0.25	1.68	0.01
GAIF01023069.1	0.6	0.33	0	0.7	0.24	0
GAIF01023072.1	3.75	0.82	0	0.87	1.08	0
GAIF01023073.1	0.14	0.01	0.09	1.66	14.46	0.04
GAIF01023076.1	0	0	0.14	0	0.23	0
GAIF01023077.1	0.12	0	0.13	0	1.55	0.45
GAIF01023078.1	0.08	0	0	0.09	0	0
GAIF01023079.1	1.14	0.21	0	2.2	1.51	1.15
GAIF01023080.1	0.7	0.05	0.48	0.15	0.1	0.05
GAIF01023081.1	0	0	0	0.12	0.13	0
GAIF01023083.1	10.55	6.52	11.41	9.86	3.14	13.26
GAIF01023084.1	10.99	1.66	1.17	7.59	1.59	1.62
GAIF01023085.1	69.06	38.89	21.26	44.33	91.07	4.59
GAIF01023086.1	9.31	12.51	1.49	20.15	28.42	0.35
GAIF01023088.1	0	0	0	0	0.2	0
GAIF01023089.1	0.21	0	0	1.14	5.98	0.08
GAIF01023090.1	33.58	18.52	13.99	19.58	9.65	33.96
GAIF01023091.1	0	0	0	0	0.19	0
GAIF01023092.1	0.06	0.09	0.03	2.44	0.34	0.15
GAIF01023093.1	0.64	0.17	0.12	1.97	6.18	0.06
GAIF01023094.1	4.89	0.03	1.8	16.44	34.53	0.71
GAIF01023096.1	11.19	5.93	8	28.76	40.02	31
GAIF01023097.1	4.01	4.24	6.28	7.43	5.55	2.56
GAIF01023098.1	2.1	2.29	0.58	8.79	13.66	0.12
GAIF01023099.1	2.76	1.04	0.53	11.68	19.64	2.08
GAIF01023100.1	0.12	0	0	1.13	3.88	0.07
GAIF01023101.1	0	0.41	0.95	0	0	0.21
GAIF01023103.1	12.27	15.52	13.88	6.13	7.53	10.27
GAIF01023104.1	0.31	0.33	0.34	0	2.78	0
GAIF01023105.1	0.04	0.05	0	0.1	0.05	0
GAIF01023106.1	1.09	0.18	0.24	0.32	0.29	0.09
GAIF01023107.1	0	0	0	0.08	0.08	0
GAIF01023108.1	0.31	0	0	0.18	0	0
GAIF01023109.1	0.06	0.02	0	0	0.01	0
GAIF01023110.1	0	0	0	0	0.05	0
GAIF01023112.1	2.49	0.46	0	0	0	0
GAIF01023114.1	7.06	11.07	9.95	15.77	8.14	4.54
GAIF01023115.1	1.19	0.18	0	2.77	6.45	5.93

GAIF01023117.1	0.09	0.24	0.15	0.05	0.7	0
GAIF01023118.1	0	0	0	0.15	0.16	0
GAIF01023119.1	0	0	0	0	0.43	0
GAIF01023120.1	2.1	0.76	0.51	4.48	10.55	3.09
GAIF01023121.1	0.86	1.84	0.17	5.6	8.46	0.26
GAIF01023123.1	0	0	0	0	0.63	0
GAIF01023124.1	2.71	3.02	1.58	11.72	13.74	1.3
GAIF01023125.1	6.08	6.03	9.16	5.22	6.51	7.35
GAIF01023126.1	9.38	8.74	13.71	23.65	29.63	7.27
GAIF01023127.1	0.29	0.1	0	0.73	4.92	0.44
GAIF01023128.1	12.98	17.13	15.89	26.27	14.57	5.15
GAIF01023129.1	0	0.07	0	0	0.11	0
GAIF01023130.1	0.18	0.26	0.07	0.31	0	0.07
GAIF01023131.1	306.61	281.56	103.78	189.85	180.43	16.49
GAIF01023132.1	0	0	0	0	0.09	0
GAIF01023133.1	0	0	0	0	0.05	0
GAIF01023134.1	60.33	64.24	75.73	69.45	46.76	40.21
GAIF01023135.1	0	0	0	0	0.67	0
GAIF01023136.1	0.2	0.04	0.04	0.54	1.92	0.09
GAIF01023137.1	3.15	1.47	2.29	1.22	1.18	0.91
GAIF01023138.1	40.94	20.63	18.35	42.47	30.7	13.25
GAIF01023140.1	15.12	32.77	2.76	57.88	101.71	3.42
GAIF01023141.1	16.28	7.52	2.65	5.03	8.49	3.38
GAIF01023143.1	0.14	0	0	0.16	0.33	0
GAIF01023145.1	40.31	26.23	38.44	35.37	15.37	35.09
GAIF01023146.1	21.55	26.55	20.89	38.04	25.34	17.23
GAIF01023147.1	190.18	144.88	68.56	78.52	45.89	15.96
GAIF01023148.1	0	0.28	0.35	0	0.08	0.64
GAIF01023149.1	0.92	1.13	0.7	5.46	5.8	0.57
GAIF01023150.1	0.09	0.07	0	0	0	0.13
GAIF01023151.1	0.31	0	0.23	2.18	7.28	2.3
GAIF01023153.1	0	0	0	0.04	0	0
GAIF01023154.1	0	0	0	0.07	0.15	0
GAIF01023155.1	11.57	19.08	16.43	25.15	14.72	6.26
GAIF01023156.1	0.4	0.26	0.26	0.28	0.29	0
GAIF01023157.1	0	0	0.02	0.01	0	0
GAIF01023158.1	0.5	0.14	0	1.02	4.83	0
GAIF01023159.1	27.94	29.29	25.4	84.22	42.61	8.71
GAIF01023161.1	411.66	383.59	442.33	183.38	119.62	125.57
GAIF01023162.1	0	0	0	0.08	0	0
GAIF01023163.1	0	0	0.25	0	0.09	0.33
GAIF01023164.1	3.54	0.57	0.14	19.2	51	8.54
GAIF01023166.1	0	0.14	0.24	0.05	0	0
GAIF01023168.1	0	0	0	0	0	0.53

GAIF01023170.1	26.46	16.31	5.71	50.94	115.87	3.58
GAIF01023171.1	0	0	0	0.14	0	0
GAIF01023172.1	0	0.07	0	0	0.23	0
GAIF01023175.1	49.51	33.96	13.38	16.07	6.66	0.24
GAIF01023177.1	0.23	0.24	0	0.26	1.9	0.08
GAIF01023178.1	4.14	5.32	0.59	4.02	2.72	0.03
GAIF01023182.1	0.62	0.15	0.24	0.72	1.97	0.05
GAIF01023185.1	0	1.71	2.64	0	0.13	0.35
GAIF01023187.1	2.07	1.78	0.87	0.95	0.55	0
GAIF01023188.1	0	0	0	0	0.1	0
GAIF01023189.1	0	0	0	0	0.03	0
GAIF01023191.1	3.82	2.3	0.58	15.52	17.97	0.2
GAIF01023192.1	1.1	0	0.09	8.92	29.2	0
GAIF01023195.1	0.25	1.21	3.18	0	0.23	1.44
GAIF01023196.1	13.5	18.47	13.75	33.4	32.91	3.64
GAIF01023197.1	0	0	0	0.15	3.11	0.16
GAIF01023198.1	0	0	0.14	0	0	0.25
GAIF01023200.1	0.05	0.1	0.06	0	0	0
GAIF01023202.1	73.1	35.18	3.58	12.7	10.48	0.94
GAIF01023203.1	1.62	2.91	0.39	9.14	12.01	0.16
GAIF01023204.1	0	0.37	0	0	0	0
GAIF01023205.1	35.25	19.22	17.95	55.86	37.23	26.37
GAIF01023208.1	0.59	0	0	0.34	1.41	0.43
GAIF01023209.1	0.38	0.1	0	0.11	0.23	0
GAIF01023210.1	0.47	0.32	0.17	0.25	0	0
GAIF01023211.1	8.69	0	0.42	0.43	0	12.23
GAIF01023212.1	20.28	32.73	32.94	63.14	52.34	14.44
GAIF01023213.1	0.89	0	0	0	0.15	0
GAIF01023214.1	1.52	1.05	0.23	8.81	23.37	0.32
GAIF01023216.1	0.71	0	0	0.79	4.73	2.18
GAIF01023219.1	10.94	10.06	11.37	23.23	31.13	15.22
GAIF01023221.1	101.19	65.95	14	12.22	7.67	2.45
GAIF01023222.1	0	0.1	0	0	0	0
GAIF01023224.1	0	0.68	0.6	0	0.09	0.26
GAIF01023225.1	1.66	1.45	2.26	1	0.88	1.61
GAIF01023226.1	2.69	1.58	1.59	3.36	6.72	1.26
GAIF01023227.1	0	0.05	0	0	0.05	0
GAIF01023228.1	1.17	0.29	2.01	1.29	0.86	1.11
GAIF01023232.1	0.99	1.26	9.39	8.53	0	3.43
GAIF01023234.1	8.86	6.27	5.28	11.15	4.12	63.88
GAIF01023236.1	0	0	0	0.41	1.48	0
GAIF01023238.1	0.58	0.58	0.52	0.12	0.13	0.29
GAIF01023239.1	0	0	0	0	0.1	0
GAIF01023240.1	14.99	12.22	4.11	17.39	9.01	53.67

GAIF01023244.1	259.14	69.18	6.13	791.18	819.02	46.69
GAIF01023245.1	0	0	0	0.05	0.05	0
GAIF01023246.1	0.13	0.39	0.42	0.15	0.25	0.09
GAIF01023249.1	0.34	0.14	0	0.94	7.26	0.69
GAIF01023250.1	31.46	28.35	48.48	111.17	131.29	73.98
GAIF01023251.1	0.25	0	0	0.13	0.47	0.06
GAIF01023252.1	0.48	0	0	1.51	2.32	0
GAIF01023254.1	0.59	0.07	0	0.53	0.23	0.14
GAIF01023255.1	0.31	0	0	0.36	0.37	0
GAIF01023259.1	0	0.08	0	0	0.05	0
GAIF01023260.1	11.83	8.64	1.27	36.29	65.42	6.12
GAIF01023261.1	17.24	8.49	3.93	11.44	18.18	70.79
GAIF01023262.1	1.42	0.47	0.24	5.51	10.92	0
GAIF01023264.1	0	0	0	0.39	0.31	0
GAIF01023267.1	0	0	0	0.65	0.45	0
GAIF01023268.1	0	0	0	0	0.04	0
GAIF01023269.1	40.67	57.19	41.43	59.59	72.78	48.42
GAIF01023271.1	0.73	0.79	1.59	0.17	0.17	0.32
GAIF01023272.1	229.35	283	109	254.83	168.84	12.54
GAIF01023273.1	0.19	0.1	0	0.22	0.91	0.1
GAIF01023275.1	0	0	0	0.63	2.9	0
GAIF01023276.1	0	0	0	0.05	0.17	0
GAIF01023277.1	0.19	0.16	0.08	0.65	0.62	0.63
GAIF01023278.1	1.83	3.97	5.32	4.68	0	2.1
GAIF01023279.1	0.11	0.12	0	0.78	4.16	0
GAIF01023280.1	0	0	0	0.37	1.15	0.09
GAIF01023282.1	150.11	52.11	20.58	0.45	0.91	0.19
GAIF01023285.1	0.94	4.59	14.07	1.09	8.46	274.45
GAIF01023286.1	0.58	0	0	2.69	7.67	0.85
GAIF01023287.1	0	2.33	7.18	0	0	1.18
GAIF01023288.1	61.51	87.28	38.99	112.14	125.37	19.92
GAIF01023289.1	0.71	0.44	0	0.7	0.36	0
GAIF01023290.1	0.09	0.48	0.53	2.44	14.8	0.1
GAIF01023291.1	3.44	1.63	0.16	5.34	3.62	0.11
GAIF01023292.1	6.21	17.67	8.01	32.9	39.01	2.48
GAIF01023293.1	11.55	3.29	5.92	1.13	0	0
GAIF01023294.1	0.35	0.24	0.57	0.13	0.26	0.06
GAIF01023295.1	15.71	12.13	9.9	20.62	30.6	11.31
GAIF01023296.1	0	0.1	0	0	0	0
GAIF01023297.1	12.99	1.21	0.85	14.3	31.77	41.23
GAIF01023298.1	13.4	11.75	6.16	28.09	14.14	1.51
GAIF01023300.1	4.75	5.56	6.64	8.95	2.54	3.49
GAIF01023301.1	1.7	0	0	3.25	7.57	4.4
GAIF01023302.1	0.09	0.48	0.05	0.15	0.05	0

GAIF01023304.1	0.01	0	0	0.04	0.21	0
GAIF01023305.1	0	0	0	0	0.45	0
GAIF01023306.1	0.21	0	0	0.25	0	0
GAIF01023307.1	93.49	181.26	108.36	108.26	211.2	70.92
GAIF01023309.1	1.09	0.23	0.24	1.5	1.02	0.72
GAIF01023313.1	8.26	8.36	0.93	53.08	107.91	0.56
GAIF01023314.1	16.5	22.1	21.72	57.63	45.11	20.54
GAIF01023318.1	0.64	0	0.28	0	0.15	0.14
GAIF01023322.1	4.99	2.51	1.86	16.27	63.24	7.19
GAIF01023323.1	0.2	0.33	0	0.2	0	0
GAIF01023326.1	2.13	3.03	0.54	12.96	23.89	0.54
GAIF01023327.1	0.14	0.19	0.08	0.37	6.14	0.12
GAIF01023329.1	13.66	11.8	16.94	25.7	14.29	12.94
GAIF01023330.1	39.66	38.59	42.88	159.5	80.04	47.15
GAIF01023332.1	8.72	12.05	4.65	6.73	6.72	1.45
GAIF01023336.1	3.57	1.74	0.95	2.71	11.54	13.82
GAIF01023337.1	14.77	16.09	14.22	13.38	12.27	4.46
GAIF01023338.1	0	0.09	0	0.32	0.86	0
GAIF01023339.1	0	0	0	0.31	0.54	0
GAIF01023340.1	0.59	0	0.09	0.22	0.39	1.79
GAIF01023341.1	1.42	0.44	0.2	3.1	2.75	0.05
GAIF01023343.1	805.71	298.46	379.57	650.61	1238.94	9975.59
GAIF01023344.1	77.66	146.28	143.74	150.56	125.88	25.19
GAIF01023347.1	0	0	0	0.11	0.23	0
GAIF01023348.1	0.06	0	0	0.37	2.33	0.14
GAIF01023349.1	1.48	3.58	2.42	9.9	15.35	2.32
GAIF01023350.1	0.34	0.37	0.19	3.64	20.7	0.38
GAIF01023351.1	19.45	18.34	22.66	15.76	37.28	55.91
GAIF01023352.1	0.6	0.13	0	0.97	4.04	4.76
GAIF01023353.1	0.54	0.37	0.35	0.27	0.75	0.24
GAIF01023355.1	0.64	1.19	0.66	7.89	1.13	1.13
GAIF01023357.1	22.83	18.33	21.35	29.81	45.53	60.31
GAIF01023359.1	0.02	0	0.25	0.71	2.39	0.64
GAIF01023360.1	8.17	8.09	4.01	5.31	3.11	0.5
GAIF01023361.1	5.91	8.31	13.58	11.29	5.51	8.34
GAIF01023362.1	0.32	0.12	0.12	2.58	23.6	2.57
GAIF01023363.1	28.13	47.14	10.35	167.69	176.33	6.35
GAIF01023364.1	24.34	13.59	13.55	28.2	9.68	12.3
GAIF01023365.1	19.09	24.29	3.26	30.38	85.39	16.5
GAIF01023366.1	0.52	0.19	0	1.82	4.74	0.1
GAIF01023367.1	0.3	0.5	0.46	0.53	0.26	0.17
GAIF01023368.1	0	0.16	0.08	0.78	3.86	0
GAIF01023369.1	1.61	0.61	0.71	0.84	0.29	0
GAIF01023370.1	0.07	0	0	0	0	0

GAIF01023371.1	8.8	9.46	20.99	32.52	28.71	23.08
GAIF01023372.1	36.05	47.68	49.64	62.4	55.67	19.06
GAIF01023373.1	0	0.07	0	0	0	0
GAIF01023374.1	0	0.03	0	0	0.09	0
GAIF01023375.1	0.06	0	0	0.38	0.78	0.28
GAIF01023377.1	0.19	0	0	0	0	0
GAIF01023379.1	0	0	0	0	0.25	0
GAIF01023381.1	0.03	0.02	0	0.05	0.07	0
GAIF01023382.1	0	0	0	0	0.18	0
GAIF01023383.1	13.7	19.5	23.43	26.35	17.21	10.27
GAIF01023385.1	0	0	0	0.31	0.3	0
GAIF01023387.1	0	0.21	0.11	0	0	0
GAIF01023388.1	0	0	0	0.61	3.82	0.58
GAIF01023390.1	0	0	0	0.23	0.66	0.15
GAIF01023391.1	47.49	34.7	72.12	0.61	0.49	1.64
GAIF01023392.1	6.57	8.22	3.7	28.98	38.82	1.54
GAIF01023394.1	12.82	6.39	5.46	31.32	24.1	8.01
GAIF01023400.1	0.04	0.3	0.75	0	0	0.04
GAIF01023401.1	32.93	30.11	20.24	37.06	19.83	14.22
GAIF01023403.1	5.27	5.55	3.5	11.79	16.38	2.24
GAIF01023406.1	1.53	1.14	0.21	5.16	11.6	0.33
GAIF01023407.1	1.81	0	0.12	3.28	10.32	10.2
GAIF01023408.1	6.46	4.02	5.32	1.21	0.42	0.13
GAIF01023409.1	1.01	0.36	0.1	11.89	32.26	3.26
GAIF01023410.1	0	0.21	0	0	0.23	0
GAIF01023411.1	0	0.26	0	0	0	0
GAIF01023413.1	1.38	2.45	1.1	2.36	3.05	0.41
GAIF01023414.1	23.72	14.31	9.99	93.04	64.02	12.49
GAIF01023415.1	0	0	0	0.1	0.63	0.14
GAIF01023416.1	0	0	0	0.08	0	0
GAIF01023417.1	0.06	0	0	0	0	0
GAIF01023418.1	0.22	0.12	0.12	0.26	0	0
GAIF01023419.1	18.28	17.22	3.59	59.79	73.86	1.9
GAIF01023421.1	4.26	3.6	0.52	64.03	135.54	2.61
GAIF01023424.1	3.24	4.46	0.82	1.73	2.39	0.41
GAIF01023425.1	0	0.41	0.24	0.3	0	0.18
GAIF01023428.1	0	0	0	0	0.33	0
GAIF01023429.1	12.27	8.25	1.7	5.84	8.58	8.55
GAIF01023430.1	21.81	11.51	3.91	45.33	50.18	12.07
GAIF01023431.1	0.93	0.28	0.67	2.72	3.86	0.57
GAIF01023432.1	0.45	0.07	0	1.27	5.26	0.57
GAIF01023433.1	0.31	0	0	0	0.13	0
GAIF01023434.1	3.88	0.27	0	11.46	17.29	17.5
GAIF01023437.1	5.03	2.59	11	0	0	0

GAIF01023438.1	0	0.14	0	0.62	0.66	0
GAIF01023441.1	0	0.15	0	0	0.51	0
GAIF01023442.1	36	11.49	16.87	14.64	8.05	21.07
GAIF01023443.1	0.01	0.05	0.03	0	0	0
GAIF01023444.1	28.03	13.74	25.74	36.69	18.8	52.59
GAIF01023446.1	14.14	17.27	24.83	35.5	20.72	12
GAIF01023447.1	679.42	530.27	166.24	962.67	1043.92	181.91
GAIF01023449.1	0.81	0.16	0.48	4.35	1.59	0.57
GAIF01023452.1	0	0.12	0.24	0.13	0	0
GAIF01023454.1	0.6	0.37	0.19	3.96	9.63	0.09
GAIF01023456.1	68.64	65.09	48.82	150.89	64.49	21.64
GAIF01023458.1	9.78	10.34	12.95	38.89	40.8	10.13
GAIF01023459.1	0	0	0	0.11	0	0
GAIF01023461.1	0	0	9.77	0	7.27	0
GAIF01023462.1	0.2	0	0.34	0	0	0
GAIF01023463.1	7.78	12.78	3.95	25.9	24.71	1.83
GAIF01023466.1	0	0	0	0.41	0.43	0
GAIF01023467.1	13.2	7.79	7.78	12.61	5.81	7.45
GAIF01023468.1	0	0	0	0.21	1.53	0
GAIF01023469.1	25.86	38.25	22.58	62.87	68.61	36.2
GAIF01023473.1	0.11	0.12	0.06	0.25	3.44	0.65
GAIF01023474.1	46.69	118.85	102.76	56.19	42.44	4.74
GAIF01023476.1	0	0.16	0	2.91	12.95	0.11
GAIF01023477.1	0.59	0.32	0.11	0.91	2.6	0.22
GAIF01023478.1	0	0	0	1.04	0.87	0
GAIF01023479.1	0	0	0.07	0.08	0.24	0
GAIF01023480.1	0.23	0.06	0	0.2	0.28	0
GAIF01023484.1	0.03	0.06	0	0.19	0.33	0.03
GAIF01023485.1	2.01	3.47	2.4	3.12	11.71	4.81
GAIF01023486.1	18.15	19.7	10.17	11.72	12.86	2.41
GAIF01023487.1	32.64	36.1	58.84	68.12	31.33	31.04
GAIF01023488.1	0.6	0.16	0.05	3.28	16.65	0.06
GAIF01023489.1	6.15	6.68	6.21	21.97	24.29	14.19
GAIF01023490.1	19.82	18.75	30.34	51.1	31.88	29.33
GAIF01023491.1	0.06	0.13	0	0	0.07	0.2
GAIF01023493.1	0.64	0.43	0.71	0.74	0.59	0.25
GAIF01023494.1	0.34	0.26	0.48	1.3	0.3	0
GAIF01023495.1	54.13	120.59	4.36	26.35	50.96	13.78
GAIF01023501.1	0	0	0	0.14	0.15	0
GAIF01023502.1	3.67	0.88	0.89	13.2	15.84	2.86
GAIF01023503.1	0.04	0	0	0	0	0
GAIF01023504.1	0	0	0	0	0.14	0
GAIF01023505.1	1.09	0.79	0.24	5.04	12.94	0
GAIF01023506.1	0.21	0	0	1.55	5.43	0.45

GAIF01023508.1	3.93	2.95	0	0	0	0.29
GAIF01023509.1	8.98	20.99	14.31	21.27	20.41	1.9
GAIF01023510.1	3.69	4.9	2.06	6.67	9.81	0.55
GAIF01023511.1	0.27	0.59	0	0.76	0.37	0
GAIF01023512.1	49.98	15.23	23.32	31.09	29.33	31.79
GAIF01023513.1	0	0	0	0.74	2.99	1.77
GAIF01023515.1	4.95	10.06	3.22	6.56	9.44	0.5
GAIF01023516.1	0.65	0.7	0.27	2.44	6.91	0.09
GAIF01023517.1	30.51	26.74	17.43	55.25	61.09	34.2
GAIF01023519.1	0.28	0.31	0.21	0.76	0.34	0.1
GAIF01023520.1	6.28	8.32	8.23	27.39	27.05	6.98
GAIF01023521.1	0	0	0	0	0.22	0
GAIF01023522.1	1.08	0	0	0.23	0.66	0
GAIF01023523.1	0	0.03	0	0	0	0
GAIF01023524.1	4.82	2.69	3.64	7.18	14.33	3.83
GAIF01023525.1	13.73	6.74	5.42	18.08	22.99	4.1
GAIF01023527.1	103.53	36.67	10.7	18.01	10.22	1.69
GAIF01023529.1	40.04	28.98	30.86	2.01	1.48	3.07
GAIF01023530.1	0	0	0	0	0.24	0.11
GAIF01023531.1	195.18	279.59	455.21	264.52	197	196.96
GAIF01023535.1	0.21	0.23	0	0.3	0.13	0
GAIF01023536.1	0.08	0	0	0.48	2.47	0.36
GAIF01023537.1	0	0	0	0	0.72	0
GAIF01023538.1	1.32	0.08	1.38	0.16	0.01	1.32
GAIF01023539.1	5.5	7.58	6.45	16.33	11.25	1.62
GAIF01023540.1	1.05	0.08	0.08	4.6	29.76	3.79
GAIF01023541.1	0.03	0	0.03	0.03	0.03	0
GAIF01023542.1	231.9	115.87	145	288.05	106.76	157.04
GAIF01023543.1	0.15	0.49	0	0	0.75	0
GAIF01023547.1	0	0.13	0.27	0	0.15	0
GAIF01023548.1	0.23	0.12	0.25	0.13	2.34	0.13
GAIF01023549.1	0	0	0	0	0.01	0
GAIF01023550.1	0.15	0.42	1.6	0.09	0	0.25
GAIF01023552.1	0.03	0	0	0.04	0	0
GAIF01023554.1	12.58	19.55	19.06	24.05	13.9	5.89
GAIF01023555.1	2.98	3.46	1.05	21.46	39.16	1.5
GAIF01023556.1	6.56	2.19	0	2.58	1.11	0.25
GAIF01023557.1	0	0	0	0	0.09	0
GAIF01023558.1	1.55	2.92	3.62	1.8	1.36	0.68
GAIF01023561.1	0	0	0	0	0.1	0
GAIF01023562.1	0	0.14	0	0	1.11	0
GAIF01023563.1	24.03	13.25	7.38	54.06	65.06	13.56
GAIF01023564.1	148.15	131.41	56.2	243.53	292.19	94.49
GAIF01023567.1	0.96	0.83	0	4.99	15.52	0.11

GAIF01023568.1	0	0	0.45	0.87	0	0
GAIF01023570.1	22.6	132.05	56.18	4.78	15.82	4.27
GAIF01023571.1	8	5.5	10.54	26.21	18.74	14.49
GAIF01023572.1	7.42	2.86	8.19	105.44	96.39	8.25
GAIF01023573.1	492.58	458.63	403.81	946.72	1170.49	3490.42
GAIF01023574.1	0.17	0.06	0	1.44	2.64	3.01
GAIF01023576.1	0	0.07	0	0	0	0
GAIF01023577.1	0	0	0	0.18	1.2	0
GAIF01023578.1	2686.18	1749.28	2547.99	1003.14	282.7	319.8
GAIF01023580.1	62.2	6.21	0.67	5.69	23.82	1.81
GAIF01023581.1	17.94	20.75	20.76	67.67	40.73	22.82
GAIF01023582.1	21.14	18.04	54.89	21.78	12.14	11.41
GAIF01023583.1	0	0	0	0	0.14	0
GAIF01023584.1	0.33	0.09	0	0.1	0.99	0
GAIF01023585.1	20.03	25.1	31.94	119.97	81.77	66.32
GAIF01023586.1	1.77	0	0	3.71	7.37	1.89
GAIF01023587.1	0.03	0	0	0	0	0
GAIF01023590.1	9.99	11.22	8.9	5.99	4.33	1.44
GAIF01023591.1	0	0	0	0	0.06	0
GAIF01023592.1	0	0.05	0	0.44	3.11	0.06
GAIF01023593.1	0.74	1.75	0.14	0.14	0.22	0
GAIF01023594.1	12.02	12.89	24.39	27.15	19.74	17.27
GAIF01023595.1	0.11	0.48	0.12	0.51	0.26	0.24
GAIF01023596.1	0	0	0	0	0.28	0
GAIF01023597.1	45.61	49.9	29.01	39.3	39.88	41.88
GAIF01023598.1	0.11	0	0	0	0	0.25
GAIF01023600.1	3.5	1.45	0.26	12.03	52.91	1.14
GAIF01023601.1	42.1	37.74	44.36	58.75	34.72	27.29
GAIF01023602.1	0.66	2.14	1.73	0.73	0.89	1.45
GAIF01023604.1	2.47	1.49	0	11.91	40.21	2.12
GAIF01023605.1	3.3	0	0	1.87	8.79	6.88
GAIF01023608.1	26.29	38.11	30.84	70.09	40.5	16.28
GAIF01023609.1	42.27	19.36	23.24	76.79	120.7	68.22
GAIF01023610.1	70.02	55.58	49.32	47.12	6.52	76.15
GAIF01023612.1	0	0	0	0	0.13	0
GAIF01023613.1	0.05	0.05	0	0.34	1.06	0.05
GAIF01023615.1	0.2	0	0	0	0.23	0
GAIF01023616.1	10.64	9.76	10.13	50.08	45.57	12.73
GAIF01023617.1	42.64	46.72	72.3	23.86	18.46	17.65
GAIF01023618.1	304.85	140.62	88.35	362.81	191.66	184.13
GAIF01023619.1	0.17	0	0	0	0	0
GAIF01023620.1	0	0	0	0	0	1.96
GAIF01023621.1	0.14	0	0	0.05	1.32	0.15
GAIF01023622.1	0	0	0	0	0.22	0.03

GAIF01023625.1	0.15	0.19	0.52	0.6	0.48	0.7
GAIF01023626.1	0.5	0.67	0.37	0.97	1.08	2.28
GAIF01023627.1	1.57	0.14	0.14	5.02	33.39	4.47
GAIF01023628.1	0	7.68	4.61	8.33	16.53	1.81
GAIF01023629.1	0	0	0	0	0.13	0
GAIF01023631.1	0.08	0.18	0.37	0	0	0
GAIF01023632.1	94.79	107.73	174.98	110.55	67.45	104.44
GAIF01023634.1	4.84	3.83	2.8	5.56	16.24	6.45
GAIF01023636.1	2.53	2.2	0	0	1.65	2.46
GAIF01023637.1	9.24	11.89	7.86	33.24	40.02	4.15
GAIF01023639.1	4.3	6.54	6.61	0	0	5.69
GAIF01023640.1	1	0.08	0	4.89	20.95	0.55
GAIF01023641.1	12.39	3.81	2.99	6.67	6.43	7.24
GAIF01023642.1	13.68	13.17	17.04	39	29.88	14.95
GAIF01023643.1	12.04	7.46	5.19	43.71	43.33	8.06
GAIF01023645.1	54.25	57.53	22.27	41.06	37.21	5.97
GAIF01023646.1	1.4	1.59	0.52	3.79	5.46	0.06
GAIF01023647.1	40.07	28.54	14.77	31.49	50.58	91.61
GAIF01023649.1	0	0	0	0	0.12	0
GAIF01023652.1	5.62	10.2	2.77	25.95	30.13	1.39
GAIF01023653.1	0	0	0	0	0.2	0
GAIF01023654.1	22.23	33.71	28.13	87.24	53.11	10.3
GAIF01023658.1	0	0	0	0	5.99	0
GAIF01023661.1	0	0	0	0	0.19	0
GAIF01023662.1	1.07	2.46	0	3.25	6.25	0.15
GAIF01023663.1	0	0.13	0	0	0.15	0
GAIF01023667.1	0.59	0.09	0.17	0.83	4.64	0.41
GAIF01023668.1	0.16	0.12	0.06	1.49	7.38	0.18
GAIF01023671.1	0.09	0	0	0.3	0.82	0
GAIF01023672.1	0	0	0	5.81	3.45	0
GAIF01023673.1	5.52	3.16	3.19	7.31	6.64	17.61
GAIF01023674.1	0.47	1	1.77	2.65	1.72	0
GAIF01023675.1	19.84	19.24	9.81	42.34	48.66	13.76
GAIF01023676.1	0.41	0.15	0	0.16	1.81	0
GAIF01023677.1	0.12	0	0	0.07	0.07	0
GAIF01023678.1	0	0	0	0	1.55	0
GAIF01023680.1	1.08	0.88	0.3	1.9	1.84	0.49
GAIF01023681.1	0.29	0.53	1.41	0.1	0.17	0.18
GAIF01023682.1	0	0	0.27	0.11	0.28	0.29
GAIF01023683.1	23.85	8.33	9.45	12.52	23.59	9.59
GAIF01023686.1	0.81	0.23	0	0.87	1.18	0
GAIF01023687.1	51.65	73.8	132.84	47.8	23.38	52.86
GAIF01023689.1	0	0	0	0	1.27	0
GAIF01023691.1	1.58	0.92	1.33	8.16	3.35	1.47

GAIF01023692.1	0	0	0	0.1	0.17	0
GAIF01023693.1	1.49	1.29	0	4.11	15.32	0.9
GAIF01023694.1	19.8	43.32	21.43	35.47	31.41	5.32
GAIF01023695.1	0	0	0	0	0.89	0
GAIF01023696.1	0.82	0.3	0.45	0	0	0
GAIF01023697.1	80.27	64.17	78.08	72.02	108.14	65.13
GAIF01023699.1	0.6	0.34	0.19	1.96	0.86	0.59
GAIF01023700.1	120.72	112.97	38.03	174.55	229.27	29.02
GAIF01023701.1	0.06	0.05	0	0.08	0.83	0
GAIF01023704.1	0.12	0	0	0.36	2.54	0
GAIF01023705.1	29.54	32.43	61.87	57.87	27.94	44.69
GAIF01023706.1	0.32	0.08	0	0.82	5.64	0.14
GAIF01023707.1	8.2	2.67	20.69	11.41	2.96	4.52
GAIF01023709.1	0.06	0	0	0.16	0.43	0
GAIF01023711.1	1.76	0.9	0.9	2.74	6.23	1.16
GAIF01023712.1	68.22	53.29	70.39	23.26	6.26	11.32
GAIF01023713.1	0	0.05	0	0.11	0.18	0
GAIF01023715.1	22.14	32.39	33.45	17.64	14.93	5.34
GAIF01023716.1	0.17	0	0	0	0	0
GAIF01023717.1	15.1	8.64	16.48	19.51	7.72	8.81
GAIF01023718.1	0.24	0	0	1.5	6.09	0.26
GAIF01023719.1	0.09	0.05	0	2.58	0.11	0.59
GAIF01023720.1	1.59	0.23	0.23	12.76	41.13	1.8
GAIF01023721.1	0.32	0.07	0.04	1.24	3.65	0.25
GAIF01023722.1	24.46	41.97	36.18	57.29	25.98	8.08
GAIF01023724.1	0.07	0.07	0	0.08	0.32	0.11
GAIF01023725.1	0.25	0.27	0	0.14	0	0
GAIF01023726.1	0	0	0	4.08	4.91	0
GAIF01023727.1	0.13	0	0	0	0.15	0
GAIF01023728.1	0	0	0	0	0.07	0
GAIF01023730.1	0	0	0	0.07	0.07	0
GAIF01023733.1	0.1	0.11	0	0.24	0.62	0.11
GAIF01023735.1	0	0.25	0	0.14	0.98	0
GAIF01023736.1	3.59	0	0	0	3.46	7.13
GAIF01023737.1	0	0.66	0	0.08	0.49	0
GAIF01023738.1	0	0	2.39	0	0	1.54
GAIF01023739.1	167.3	178.76	477.93	19.47	20.44	113.14
GAIF01023740.1	8.74	0	22.39	20.29	7.01	0
GAIF01023742.1	0	0.15	0	0	0	0
GAIF01023743.1	20.41	6.77	4.7	1.13	2	1
GAIF01023744.1	0.35	0.54	0.13	4.03	5.09	1.69
GAIF01023745.1	0	0	0	0	0.22	0
GAIF01023746.1	0.76	1.71	2.94	1.01	2.61	1.15
GAIF01023749.1	0.89	0.41	0.21	0	0	0

GAIF01023750.1	0.35	0	2.44	2.34	0.93	0.86
GAIF01023752.1	0.29	0	0	1.7	3.87	0
GAIF01023753.1	0	2.43	7.19	0	0.05	1.54
GAIF01023754.1	0	0	0	0.96	2.66	0
GAIF01023755.1	0.41	0.05	0.11	0.7	2.66	3.58
GAIF01023758.1	0	0	0	0	0.05	0
GAIF01023761.1	2.72	6.05	2.21	8.97	3.39	0.87
GAIF01023762.1	0	0	0	0.04	0	0
GAIF01023764.1	0	0	0	0.14	0.3	0
GAIF01023765.1	0	0	0	0.08	1.31	0.07
GAIF01023768.1	0.14	0.06	0.1	0.26	0.24	0.14
GAIF01023769.1	1.98	2.31	1.27	1.68	2.51	2.05
GAIF01023772.1	0	0.02	0.02	0	0	0
GAIF01023774.1	12.67	20.1	21.75	29.61	17.85	3.67
GAIF01023775.1	0.35	0.13	0.76	0.27	0	0.25
GAIF01023777.1	0	0	0	0	0.16	0
GAIF01023780.1	0	0	0.04	0.04	0.31	0
GAIF01023781.1	0	0	0.2	0	0	0
GAIF01023783.1	1.25	0.1	0	1.04	5.9	0
GAIF01023784.1	0	0	0	0.03	0	0
GAIF01023785.1	79.68	100.12	94.24	212.57	198.23	63.34
GAIF01023786.1	0.63	0.41	0.14	0.73	2.66	2.09
GAIF01023787.1	0.57	0.85	0.08	3.29	1.96	0.16
GAIF01023788.1	10.27	0.11	0	1.84	11.1	0
GAIF01023789.1	0.1	0	0.23	0.52	1.49	0.45
GAIF01023790.1	0	0	0	0	0.34	0
GAIF01023791.1	17.71	11.35	12.1	3.27	6.56	20.74
GAIF01023792.1	0.1	0.11	0	0.23	1.45	0.11
GAIF01023793.1	34.2	42.06	20.98	70.8	75.4	5.54
GAIF01023795.1	11.85	21.61	20.85	17.17	19.81	8.11
GAIF01023796.1	2.99	2.22	1.98	1.27	3.53	1.51
GAIF01023797.1	2422.62	2338.67	2035.27	1500.29	1246.02	4790.33
GAIF01023799.1	0	0	0	0	0.75	0
GAIF01023800.1	0	0.42	0	0	0	0
GAIF01023801.1	15.71	16.81	21.2	57.14	41.91	7.46
GAIF01023802.1	0	0	0	0	0.09	0
GAIF01023803.1	0	0	0	0.27	0.14	0
GAIF01023804.1	0	0.14	0	0.14	0.23	0.13
GAIF01023807.1	2.76	9.65	4.5	3.27	5.21	0
GAIF01023809.1	0	0	0	0	1.02	0
GAIF01023811.1	38.82	61.04	25.56	28.76	50.97	22.03
GAIF01023812.1	0.25	0.08	0	0.76	1.36	0.6
GAIF01023813.1	33.62	32.32	25.64	30.12	62.84	23.68
GAIF01023814.1	0	0	0	0	0.36	0

GAIF01023815.1	0	0	0.09	0	0	0
GAIF01023816.1	0.7	0.38	0	3.85	12.29	1.73
GAIF01023817.1	5.61	6.77	4.4	2.36	1.56	0.41
GAIF01023819.1	0	0.2	0.1	0	0.66	0.1
GAIF01023820.1	6.84	11.6	4.98	13.53	12.98	1.08
GAIF01023821.1	3.72	0.19	0.22	45.25	48.39	0.32
GAIF01023822.1	0.15	0	0	1.93	6.17	0
GAIF01023823.1	1907.71	2736.87	3787.93	1211.35	979.46	712.69
GAIF01023826.1	0	0	0	0	0.22	0
GAIF01023829.1	3.35	6.54	3.27	10.73	11.26	2.05
GAIF01023830.1	0.68	0.33	0.14	0.55	1.15	0.14
GAIF01023832.1	1.16	0.46	0.12	5.12	21.97	0.58
GAIF01023833.1	0	0	0	0.12	0.25	0
GAIF01023837.1	36.96	47.5	50.77	134.5	103.36	15.66
GAIF01023838.1	16.77	14.96	30.38	41.37	22.88	23.12
GAIF01023839.1	1.18	0.41	0.12	1.61	3.54	0.64
GAIF01023840.1	1.92	2.67	1.78	8.97	2.29	1.7
GAIF01023841.1	0	0	0.09	0.1	0.3	0
GAIF01023842.1	120.45	148.2	167.55	3.64	2.19	1.87
GAIF01023843.1	30.75	17.05	14.77	23.43	19.7	16.17
GAIF01023844.1	0.11	0.37	0.13	0	0.41	0
GAIF01023845.1	0	0.25	0	0	0	0
GAIF01023846.1	0	0	0	0	0.04	0
GAIF01023848.1	14.02	13.02	12.25	32.54	21.91	6.2
GAIF01023849.1	0.05	0	0	0	0.17	0
GAIF01023851.1	123.7	75.73	26.38	141.35	150.25	25.33
GAIF01023853.1	0.22	0.05	0.37	1.69	0.93	0.49
GAIF01023854.1	76.35	21.64	14.75	8.24	9.43	4.38
GAIF01023855.1	0	0.31	0.16	0.17	0.17	0
GAIF01023857.1	11.9	19.81	19.83	35.01	21.66	4.45
GAIF01023859.1	5.07	2.17	1.45	9.97	28.07	4.69
GAIF01023861.1	6.44	5.18	7.16	6.76	9.17	10.84
GAIF01023862.1	36.71	34.02	22.92	43.31	51.76	29
GAIF01023863.1	5.89	6.67	0.54	18.8	13.57	0
GAIF01023864.1	0	0.17	0	0.16	0.33	0.15
GAIF01023865.1	0	0	0	0.16	0.16	0
GAIF01023867.1	3.58	3.98	1.53	0.91	0.73	0
GAIF01023868.1	0.04	0	0	0.05	0	0
GAIF01023869.1	0	0	0.07	0.11	0.18	0.06
GAIF01023870.1	38.49	18.12	10.02	123.69	179	32.65
GAIF01023871.1	0.34	0.19	0.57	1.87	6.21	0.25
GAIF01023872.1	0	0	0	0.07	0	0
GAIF01023873.1	18.77	18.23	11.33	24.18	13.82	6.15
GAIF01023874.1	4314.43	4447.92	8114.54	2394.74	1650.01	6122.87

GAIF01023875.1	0	0	0.03	0	0	0.12
GAIF01023876.1	0	0.04	0.11	0	0	0.12
GAIF01023878.1	4.74	10.25	25.51	6.85	4.96	5.46
GAIF01023879.1	2.29	1.52	0.56	4.73	4.14	0.84
GAIF01023880.1	0.42	0.14	0.06	1.74	7.42	0.14
GAIF01023881.1	6.92	3.94	3.18	5.95	16.24	6.61
GAIF01023882.1	22.6	21.13	9.15	69.37	90.32	3.17
GAIF01023884.1	1.16	2.43	0.64	7.29	16.3	0.27
GAIF01023886.1	0.07	0	0	0.24	0.76	0
GAIF01023887.1	12.74	8.07	2.41	38.29	87.58	2.51
GAIF01023888.1	22.78	21.41	22.47	6.76	4.84	3.49
GAIF01023890.1	0.15	0.33	0	0.27	0.37	0.08
GAIF01023891.1	0	0	0	0.11	0	0
GAIF01023892.1	0	0	0	0	2.03	0
GAIF01023893.1	0.36	0.15	0.04	0.82	4.14	0.24
GAIF01023894.1	0.14	0.24	0	0.02	0.28	0
GAIF01023895.1	0	0	0	0.13	0.94	0
GAIF01023896.1	9.69	9.33	11.4	2	0.5	0.34
GAIF01023898.1	0	0	0	107.37	662.81	3407.15
GAIF01023900.1	0.54	0	0	0.2	0.51	0.24
GAIF01023902.1	0	0	0	0.4	0.96	0
GAIF01023903.1	0.26	0.14	0.43	0.15	0.32	0
GAIF01023905.1	0.16	0	0.17	0.18	0	0
GAIF01023906.1	0.78	0.73	0.47	2.19	8.96	0.45
GAIF01023907.1	3.2	8.09	4.19	5.63	6.4	0.31
GAIF01023908.1	4.31	7.02	3.18	7.93	11.16	6.63
GAIF01023909.1	0.24	0.09	0.18	2.31	6.04	0
GAIF01023910.1	10.14	10.12	2.75	32.02	44.24	3.31
GAIF01023911.1	0.34	0	0	1.48	4.83	0.21
GAIF01023912.1	0	0	0	0.04	0.12	0.04
GAIF01023913.1	9.28	6.87	8.67	37.14	10.69	11.32
GAIF01023915.1	10.15	2.18	0.8	23.19	41.83	3.2
GAIF01023916.1	1.02	1.21	0	3.08	7.06	0.26
GAIF01023917.1	1.62	1.09	4.87	0.9	6.81	2.61
GAIF01023918.1	1.39	0.39	0.06	0	0.37	0.17
GAIF01023919.1	3.04	0	0	5.9	10.38	0.76
GAIF01023921.1	0	0	0	0	0.35	0
GAIF01023922.1	0.17	0.22	0.16	1.91	3.82	0.19
GAIF01023924.1	14.21	17.58	14.58	24.26	18.07	4.38
GAIF01023926.1	24.63	16.22	2.3	55.4	48.14	0.71
GAIF01023927.1	12.58	4.37	90.56	5.48	4.06	1.63
GAIF01023928.1	0	7.19	7.72	0	0	3.3
GAIF01023929.1	21.08	22.64	13.29	29.46	36.41	32.11
GAIF01023930.1	2.38	0.51	0.53	10.08	36.33	2.53

GAIF01023931.1	2.11	0.35	0.24	6.96	20.9	10.01
GAIF01023934.1	5.19	3.32	0	1.77	2.43	1.42
GAIF01023937.1	0.33	0	0	0.11	0.61	0
GAIF01023938.1	0	0	0	0.05	0.26	0
GAIF01023940.1	79.76	92.22	134.74	42.37	27.36	24.14
GAIF01023941.1	14.33	3.29	31.45	35.8	9.15	0
GAIF01023942.1	0	0	0	0.18	0.18	0
GAIF01023943.1	0.77	0.37	0	5.07	21.93	4.72
GAIF01023944.1	0.43	1.32	0.19	0.9	3.74	0
GAIF01023945.1	0.18	0.15	0.2	0.16	0.28	0.15
GAIF01023948.1	0	0	0	0	0.12	0
GAIF01023949.1	8762.99	4952.69	7657.25	4958.43	3248.19	10611.82
GAIF01023950.1	0	0	0	0	0.41	0
GAIF01023951.1	134.03	36.02	34.85	82.06	67.66	131.53
GAIF01023952.1	0.6	0	0.19	0.89	5.53	3.94
GAIF01023954.1	65.39	21.81	5.35	24.54	43.29	52.18
GAIF01023956.1	0	4.45	2.28	5.42	0.61	0
GAIF01023957.1	29.53	43.25	20.69	103.64	59.99	9.23
GAIF01023958.1	2.79	0.96	1.13	0.34	0.18	0
GAIF01023959.1	0	0	0	0	0.06	0
GAIF01023960.1	0.14	0.05	0	0.04	0.2	0.13
GAIF01023961.1	0	0	0	0.09	0.29	0
GAIF01023962.1	0	0	0	0	0	0.12
GAIF01023965.1	0	0.06	0	0.07	0.41	0
GAIF01023966.1	0.94	1.03	0.52	4.11	10.64	2.34
GAIF01023967.1	0	0.19	0.1	0.03	0.22	0.31
GAIF01023968.1	0	0	0.03	0	0.01	0
GAIF01023969.1	0.43	0.08	0.08	0.76	2.27	0.23
GAIF01023970.1	0	0.19	0.03	0	0.04	0.45
GAIF01023973.1	4.86	0.32	0.02	7.41	15.83	0
GAIF01023974.1	0.14	0	0.31	1.13	1.26	0.16
GAIF01023975.1	9.23	7.49	12.01	25.24	27.67	23.91
GAIF01023976.1	1.51	0.13	0	1.61	4.79	16.35
GAIF01023978.1	6.1	7.87	18.7	32.62	14.34	15.96
GAIF01023979.1	0	0	0	0.22	0	0
GAIF01023980.1	0.09	0	0	0	0.54	0.1
GAIF01023981.1	0.13	0	0	0	0	0.85
GAIF01023982.1	11.58	7.45	15.24	0.98	2.61	0
GAIF01023984.1	0	0	0	0.06	0.32	0
GAIF01023985.1	5.81	3.77	0.98	35.78	65.96	4.31
GAIF01023987.1	6	3.97	2.23	15.14	7.28	14.92
GAIF01023988.1	83.91	13.14	3.32	24.2	69.41	105.54
GAIF01023991.1	0.25	0	0.07	0.14	0.79	0.2
GAIF01023993.1	0.08	0	0.09	0	1.92	0

GAIF01023994.1	14.87	24.25	33.82	63.61	32.57	20.08
GAIF01023995.1	0.28	0.26	0.18	0	0.15	0.04
GAIF01023996.1	0.93	0.69	0.29	0.47	0.3	0.1
GAIF01023997.1	22.71	33.6	21.05	40.72	33.69	8.1
GAIF01024000.1	0.05	0.15	0.06	0.16	0.13	0
GAIF01024004.1	19.39	11.52	12.16	26.81	52.13	42.65
GAIF01024005.1	0	0	0.07	0.51	1.57	0.07
GAIF01024006.1	0.2	0.22	0.37	0.16	0.08	0.08
GAIF01024007.1	0	0.59	0	0	0	0.17
GAIF01024008.1	0	0	0	0	0.39	0.36
GAIF01024009.1	0.04	0	0	0.05	0	0
GAIF01024011.1	0	0	0	0.1	0	0
GAIF01024013.1	16.26	8.15	5.15	50.62	68.74	7.34
GAIF01024014.1	16.4	19.46	18.67	26.6	39.97	6.38
GAIF01024016.1	0.05	0.31	0.15	0.09	0.08	0
GAIF01024018.1	0	0	0	0.41	2.99	0
GAIF01024020.1	58.01	60.58	56.69	106.95	43.72	14.42
GAIF01024021.1	0	0.12	0	0.13	0.67	0
GAIF01024022.1	37.08	26.14	23.13	44.2	58.47	25.34
GAIF01024024.1	0.14	0	0	0	0.98	0
GAIF01024025.1	4.36	8.72	0.99	2.19	1.18	0
GAIF01024026.1	0	0.15	0	0.16	0.34	0
GAIF01024027.1	0.66	0	0	0.54	0	0
GAIF01024028.1	0.3	0	0	0.62	1.08	0.26
GAIF01024029.1	0	0	0	0.12	0.43	0
GAIF01024030.1	13.04	22.63	11.84	39	21.81	3.96
GAIF01024031.1	0	0	0	0	0.25	0
GAIF01024032.1	9.19	18.92	4.15	40.77	73.04	0.18
GAIF01024033.1	0.42	0.64	0.53	0.32	1.13	1.3
GAIF01024034.1	0.06	0	0	0	1.2	0
GAIF01024036.1	1009.05	205.1	278.44	609.8	993.3	3689.51
GAIF01024037.1	0.77	0.15	0	0.21	1.34	0
GAIF01024038.1	0	0	0	0.53	3.89	0
GAIF01024040.1	0.66	0.7	0	0.71	0.71	0
GAIF01024041.1	0.23	0.13	0	0.33	2.7	0.19
GAIF01024042.1	20.62	15.57	10.09	88.78	83.53	7.94
GAIF01024045.1	69.99	54.38	83.32	14.12	29.81	18.4
GAIF01024046.1	0	0	0.36	0	0	0.72
GAIF01024048.1	0	0	0	0.03	0	0
GAIF01024049.1	1.13	0.32	0.02	3.32	9.58	0.07
GAIF01024053.1	0.09	0	0.1	1.18	2.14	0.51
GAIF01024055.1	151.26	76.41	31.92	78.96	33.62	5.69
GAIF01024056.1	3.34	2.5	1.15	4.69	1.34	0.54
GAIF01024057.1	0	0.21	0.09	0.77	1.18	0.26

GAIF01024059.1	4.58	10.48	8.83	4.47	4.02	1.63
GAIF01024060.1	0.41	0	0	0.24	0.25	0
GAIF01024061.1	41.92	33.71	47.09	24.93	22.3	46.5
GAIF01024062.1	0.26	0	0	0.11	1.61	0
GAIF01024063.1	0	0	0	0	0	0.21
GAIF01024064.1	0	0	0	0	0.13	0
GAIF01024065.1	0	0	0	0	0.08	0
GAIF01024066.1	6.45	6.66	4.3	6.18	3.38	3.46
GAIF01024068.1	0.47	0	0	0	0.14	0
GAIF01024071.1	106.81	147.31	59.39	27.16	22.36	0
GAIF01024075.1	0	0	0	0	0.08	0
GAIF01024076.1	0	0	0	0.61	1.38	0
GAIF01024077.1	4.38	1.12	0.14	3.06	2.63	0.21
GAIF01024078.1	20.05	16.38	11.84	17.82	28.23	9.74
GAIF01024079.1	3.04	3.63	3.83	8.1	5.48	0.84
GAIF01024080.1	0.4	0	0	2	6.38	0.29
GAIF01024082.1	0	0	0	0.16	0.33	0
GAIF01024083.1	18.9	29.97	20.5	86.36	47.08	9.19
GAIF01024084.1	1	0.16	0.19	3.18	10.66	0.19
GAIF01024085.1	0.55	0.75	0.15	10.21	19.9	0.23
GAIF01024086.1	0	0.71	0.48	0	0	0.08
GAIF01024087.1	622.04	4348.58	1806.86	92.15	459.9	371.45
GAIF01024088.1	0.46	0.18	0.44	1.91	6.03	0.88
GAIF01024090.1	0.07	0	0.06	0.44	0.54	0
GAIF01024092.1	0	0.27	0.27	0.58	0	0.14
GAIF01024093.1	402.03	139.55	46.95	138.01	284.03	13.07
GAIF01024094.1	5.34	10.24	4.59	8.14	6.72	0.62
GAIF01024095.1	0.62	0.13	0	9.12	22.18	0
GAIF01024096.1	4.95	2.51	4.31	2.48	7.29	15.24
GAIF01024097.1	0.47	0	0	1.5	10.74	0.52
GAIF01024099.1	0	0	0	0.03	0	0
GAIF01024100.1	0.06	0	0.18	0.18	0.01	0.03
GAIF01024101.1	3.39	1.9	0.89	2.46	5.45	0.28
GAIF01024102.1	101.6	69.15	25.18	110.28	231.07	107.96
GAIF01024103.1	4.1	0	0.18	3.62	9.67	0.72
GAIF01024104.1	0	0	0	0	0	0.19
GAIF01024105.1	0.16	0	0	0.14	0.13	0.06
GAIF01024110.1	0.21	0.07	0	0.95	2.47	0.38
GAIF01024113.1	5.14	8.94	0.9	15.38	21	0.23
GAIF01024114.1	0	0	0	0.23	0	0.44
GAIF01024115.1	11.53	5.92	3.9	14.84	13.93	1.9
GAIF01024116.1	3.63	4.89	2.24	6.73	6.31	0.39
GAIF01024118.1	6.64	7.22	6.24	13.59	6.83	1.16
GAIF01024119.1	0	0	0	0.03	0.2	0.03

GAIF01024120.1	1.41	0.77	0	9.89	25.25	0
GAIF01024121.1	5.89	2.57	6.06	12.28	12.27	20.14
GAIF01024122.1	0.27	0.19	0.1	0.42	0.32	0
GAIF01024123.1	4.22	3.36	3.17	11	6.47	2.65
GAIF01024124.1	12.57	13.31	8.21	8.72	2.03	0
GAIF01024125.1	8.46	15.12	12.22	5.11	3.35	2.94
GAIF01024126.1	5.52	1.25	1.72	4.59	10.9	9.3
GAIF01024127.1	0	0	0	0.2	0	0
GAIF01024129.1	0.68	0	0.15	0.32	0.16	0
GAIF01024130.1	8.87	13.14	5.26	1.58	1.03	0.39
GAIF01024131.1	0.58	0	0	0	0	0
GAIF01024132.1	6.18	1.17	0	7.67	17.16	1.54
GAIF01024133.1	2.24	1.12	3.06	1.07	0.35	1.25
GAIF01024134.1	7.48	11.17	7.23	18.31	17.84	1.89
GAIF01024136.1	1.37	2	1.38	5.14	6.65	0.36
GAIF01024137.1	0.46	0	0	0.53	2.94	0.14
GAIF01024139.1	108.59	185.64	295.81	202.72	125	114.95
GAIF01024140.1	78.89	40.3	32.91	88.82	115.2	122.68
GAIF01024143.1	18.15	8.35	17.25	7.09	2.31	7.69
GAIF01024144.1	0.02	0.26	0	0.34	3.26	0.21
GAIF01024145.1	18.4	17.06	12.31	12.02	6.32	6.3
GAIF01024146.1	13.35	26.82	12.7	33.79	22.48	2.24
GAIF01024148.1	0	0	0	0.19	0.41	0
GAIF01024149.1	0.08	0.08	0.08	0.04	1.14	0.04
GAIF01024150.1	9.01	8.85	14.28	21.4	9.04	9.75
GAIF01024152.1	0	0.04	0.08	0	0	0
GAIF01024154.1	8.15	12.56	6.29	27.7	14.7	6.74
GAIF01024155.1	2.7	1.32	0.88	4.19	0.64	1.94
GAIF01024156.1	44.96	43.45	19.19	98.55	102.12	30.28
GAIF01024157.1	0.37	0.04	0	0.14	0.42	0
GAIF01024158.1	0.2	0.36	0	0.14	0.4	0.18
GAIF01024159.1	0.3	0	0.28	0.1	0	0.05
GAIF01024160.1	2.77	2.9	1.64	2.35	4.49	0.82
GAIF01024162.1	4.05	1.44	1.5	2.36	3.37	0.35
GAIF01024163.1	0	0	0	0.59	2.34	0
GAIF01024165.1	2.89	1.33	0.72	0.33	0.75	0.08
GAIF01024167.1	0	0.49	0.49	0	0	0.07
GAIF01024168.1	1.3	0	0.2	0.43	0	0
GAIF01024170.1	3.03	1.78	1.83	0.99	1.2	0.53
GAIF01024171.1	4.06	0	1.05	13.93	34.72	0
GAIF01024172.1	484.76	549.95	256.94	531.82	344.05	330.62
GAIF01024173.1	0	0	0	0.04	0	0
GAIF01024175.1	0.06	0.02	0	0	0.06	0
GAIF01024177.1	12.89	12.75	8.32	7.1	4.89	1.14

GAIF01024179.1	0.22	0.15	0.06	0.64	1.72	0
GAIF01024181.1	88.89	157.2	68.67	215.54	171.47	9.12
GAIF01024183.1	101.97	170.11	203.41	93.35	80.23	96.18
GAIF01024184.1	0	0	0.32	0	0.35	0
GAIF01024185.1	4.15	3.57	1.19	6.58	12.98	1.51
GAIF01024186.1	8.31	4.63	2.34	4.58	3.81	0.29
GAIF01024188.1	0.12	0.06	0.32	0.58	0.99	0.13
GAIF01024192.1	0	0.13	0	0	8.7	0
GAIF01024193.1	0.17	0.14	0	0.29	0.4	0
GAIF01024194.1	0	0	0	0.07	0	0
GAIF01024195.1	0	4.78	2.07	0	0.25	0.63
GAIF01024196.1	9.95	12.39	4.22	72.47	83.52	2.09
GAIF01024197.1	3208.92	3825.81	5792.21	2448.77	1658.83	3877.09
GAIF01024198.1	21.58	29.38	31.93	51.96	23.24	13.94
GAIF01024199.1	2.11	0.11	0.12	3.34	10.77	1.7
GAIF01024200.1	3.58	5.55	4.07	5.36	4.6	0.27
GAIF01024202.1	0	0	0	0.27	2.91	0
GAIF01024203.1	0.13	0.09	0	0.39	0	0
GAIF01024204.1	0.12	0.19	0	0.07	0.42	0
GAIF01024205.1	1.4	0.97	0.14	7.26	6.76	3.17
GAIF01024206.1	0	0	0.09	0	0	0
GAIF01024207.1	0	0	0	0	0.66	0
GAIF01024208.1	25.26	6.22	3.68	0	0	0
GAIF01024209.1	0.26	0.28	0	0.45	0.15	0
GAIF01024210.1	6.25	7.37	0.88	31.78	91.61	2.29
GAIF01024211.1	1.17	0.4	0.09	0.86	0.38	1.3
GAIF01024213.1	0	0	0	0	0.22	0
GAIF01024214.1	0.32	1.05	0	0	0	0
GAIF01024215.1	0	0	0	0.02	0.02	0
GAIF01024216.1	4.27	2.17	3.09	4.11	9.07	4.5
GAIF01024220.1	4.55	1.6	2.74	4.26	4.23	1.62
GAIF01024221.1	0	0	0	0	0.12	0
GAIF01024223.1	99.12	88.66	28.07	10.51	6.18	31.76
GAIF01024226.1	2.3	1.82	1.55	3.94	1.95	4.44
GAIF01024227.1	1.71	0	0	1.17	1.52	0.42
GAIF01024228.1	3.2	0.16	0.26	7.15	21.7	8.05
GAIF01024230.1	0	0	0	0.4	0	0
GAIF01024233.1	0.09	0	0	0.05	0	0.05
GAIF01024235.1	7.98	9.37	2.1	12.82	14.58	3.96
GAIF01024236.1	3.26	4.54	4.74	6.79	3.52	2.11
GAIF01024237.1	0	0	0	0	38.72	0
GAIF01024238.1	0	0	0	0	0.29	0
GAIF01024239.1	0.04	0.02	0.2	0.08	0.37	0.03
GAIF01024241.1	0.02	0	0	0.03	0.02	0

GAIF01024242.1	1.61	0.53	0	4.88	6.32	0
GAIF01024243.1	0	0.48	0	0	2.34	0.07
GAIF01024244.1	13.42	6.9	12.51	25.15	18.13	1.61
GAIF01024245.1	0	0	0	0	0.09	0
GAIF01024246.1	7.5	13.22	10.68	24.7	16.33	2.9
GAIF01024247.1	0.16	0.26	0	0.03	0.03	0.31
GAIF01024248.1	2.6	3.83	2.04	0.79	0.3	0.2
GAIF01024249.1	5.27	5.18	4.36	10.14	10.16	2.31
GAIF01024250.1	0.03	0	0	0.06	0.33	0
GAIF01024251.1	95.05	72.31	110	107.9	68.53	48.22
GAIF01024252.1	0	0	0	0.24	0	0
GAIF01024253.1	8.53	12	7.22	23.41	14.06	3.24
GAIF01024254.1	0	0.11	0.34	0.22	0.18	0.23
GAIF01024255.1	8.35	4.95	4.75	11.02	25.95	30.5
GAIF01024256.1	0.06	0.06	0	0.49	0.61	0.06
GAIF01024258.1	0.43	0	0	0.99	6.66	0
GAIF01024259.1	0.22	0	0	0	0	0.05
GAIF01024260.1	0.3	0	0	0.82	0.85	0.07
GAIF01024261.1	8.64	2.84	1.3	34.21	54.29	22.82
GAIF01024262.1	2.67	0.09	0	1.13	7.18	0.64
GAIF01024263.1	0	0	0	0	0.19	0
GAIF01024266.1	11.45	18.3	17	1.72	1.78	0.89
GAIF01024267.1	0	0	0	0	0.25	0
GAIF01024269.1	0	0.8	0.36	3.07	13.69	1.12
GAIF01024270.1	505.64	34.28	23.25	331.7	315.89	445.42
GAIF01024271.1	24.82	36.49	20.55	4.9	15.48	4.63
GAIF01024272.1	11.01	8.05	17.34	16.81	9.5	61.81
GAIF01024273.1	1.35	1.38	1.57	2.58	3.62	0.17
GAIF01024274.1	0	0	0	0.18	0.09	0.04
GAIF01024275.1	0.37	0.21	0.77	0.22	0.2	0
GAIF01024277.1	4.73	7.09	3.91	3.05	2.48	0.36
GAIF01024279.1	6.47	5.21	6.45	9.32	10.67	2.25
GAIF01024280.1	0	2.03	0	0	0	0.22
GAIF01024281.1	0.05	0	0	0.25	0.97	0.17
GAIF01024282.1	0	0	0	0.63	0.49	0.07
GAIF01024284.1	28.17	11.22	3.71	4.2	3.12	0.25
GAIF01024285.1	7.34	1.67	1.06	40.8	85.62	1.78
GAIF01024286.1	0	0	0	0.24	0.61	0.11
GAIF01024287.1	0	0.15	0.31	0	0	0
GAIF01024288.1	18.88	36.72	7.11	46.39	56.53	0.61
GAIF01024290.1	28.34	28.32	22.08	27.34	24.58	10.69
GAIF01024291.1	0	0	0	0	0.08	0
GAIF01024297.1	0	0	0	0.03	0.1	0
GAIF01024298.1	0.07	0	0	0	0	0

GAIF01024299.1	45.78	31.73	19.12	168.31	149.71	26.94
GAIF01024300.1	0	0	0	0.13	0.2	0
GAIF01024301.1	0.38	0	0	0.15	0.3	0
GAIF01024302.1	0	0	0	0	0.28	0
GAIF01024303.1	1.04	0.66	0.54	4.61	9.55	0.34
GAIF01024304.1	14.61	6.71	15.21	1.13	0	0.6
GAIF01024305.1	54.75	52.95	39.84	32.03	31.52	24.58
GAIF01024306.1	1	0.39	0	0.39	1.76	0
GAIF01024308.1	0.07	0	0.64	0.08	0	0.08
GAIF01024309.1	0	0	0	0	0.17	0
GAIF01024310.1	1.15	2.37	0.53	0.63	0	0
GAIF01024311.1	0.73	0.62	0.36	1.22	2.54	0.36
GAIF01024312.1	13.74	20.9	16.11	3.4	4.19	1.79
GAIF01024313.1	0	0	0.09	0	0	0.09
GAIF01024315.1	3.5	4.1	2.61	18.26	19.79	2.62
GAIF01024316.1	0	2.08	0	0	0	0
GAIF01024317.1	0	0	0	0.15	1.24	0
GAIF01024318.1	37.77	9.26	4.87	27.81	66.53	12.4
GAIF01024319.1	2.17	0.37	0	3.18	8.1	0.38
GAIF01024320.1	3.59	2.52	1.21	5.68	6.2	0.41
GAIF01024321.1	1.18	2.19	0.39	6.59	11.52	1.3
GAIF01024322.1	0.91	0.11	0.63	0.12	3.51	1
GAIF01024323.1	0	0.03	0	0	0	0
GAIF01024325.1	0.13	0.03	0.57	0	1.42	0.28
GAIF01024326.1	6.4	11.76	2.78	22.19	18.98	1.37
GAIF01024327.1	4.36	2.79	4.2	11.04	8.61	7.36
GAIF01024329.1	0	0	0	0	3.77	0.18
GAIF01024330.1	1.92	1.79	0.75	9.39	16.49	0.45
GAIF01024333.1	0	3.35	2.09	0.19	0.37	0.98
GAIF01024334.1	0.12	0.08	0	0.15	0.75	0.16
GAIF01024335.1	15.41	16.72	4.26	9.16	20.12	10.29
GAIF01024336.1	0.12	0	0	0	0	0
GAIF01024338.1	2.31	1.82	1.02	18.85	22.78	0.4
GAIF01024339.1	0	0	0.08	0	0.11	0.05
GAIF01024341.1	9.16	5.46	16.12	0	0	9.42
GAIF01024342.1	0.41	1.31	0	0.97	0	0
GAIF01024343.1	0	0	0.23	0	0.27	0
GAIF01024344.1	61.97	9.68	0.84	92.41	86.96	1.49
GAIF01024345.1	0.66	0.16	0	0.33	2.77	0.83
GAIF01024346.1	0.36	0.49	0	0	0.88	0
GAIF01024347.1	0.15	0	0.81	0.19	0	0.36
GAIF01024348.1	19.89	23.32	35	44.36	23.3	13
GAIF01024350.1	70.08	77.39	107.8	359.95	312.79	94.3
GAIF01024351.1	9.72	11.07	3	8.33	10.7	1.89

GAIF01024352.1	0.43	0.23	0	1.79	9.22	0.24
GAIF01024354.1	0	4.61	5.92	0.11	0	0.74
GAIF01024356.1	0	0	0	0	0.13	0
GAIF01024357.1	0.04	0.09	0	0	0.05	0.05
GAIF01024360.1	0	0	0	0.01	0.54	0
GAIF01024362.1	30	40.62	38.15	35.17	23.67	7.25
GAIF01024363.1	0.22	0.24	0.25	1.92	3.54	0.25
GAIF01024364.1	0.36	0.08	0	0.42	5.07	0.32
GAIF01024367.1	0.65	0.57	0.14	3.02	8.75	0
GAIF01024368.1	98.17	42.63	60.71	83.34	37.33	16.77
GAIF01024373.1	0.91	0.57	0.57	1.81	2.82	1.44
GAIF01024374.1	0	0	0	0.23	0.65	0
GAIF01024375.1	0	0	0	0	0	0.06
GAIF01024376.1	0	0	0	0	0.95	0
GAIF01024377.1	39.43	21.24	5.25	249.13	148.59	151.17
GAIF01024380.1	38.61	27.55	26.89	81.48	49.55	16.03
GAIF01024381.1	0	0.12	0	0	0	0
GAIF01024382.1	0.13	0	0	0	0.3	0
GAIF01024383.1	0.61	0.33	0.34	1.05	1.7	0
GAIF01024384.1	1.3	1.23	0	14.26	32.02	1.03
GAIF01024385.1	10.43	7.12	10.69	17.65	17.42	14.27
GAIF01024386.1	1.21	0.83	0.49	1.85	4.76	0.84
GAIF01024387.1	0.04	0.04	0.04	0.49	3.64	0.17
GAIF01024388.1	132.05	84.39	52.66	111.93	87.93	43.02
GAIF01024389.1	0	0	0	39.54	64.11	0
GAIF01024390.1	14.28	6.97	8.58	27.82	28.42	9.57
GAIF01024392.1	1892.36	1896.92	2935.52	843.72	483.91	318.31
GAIF01024394.1	0	0	0	0	0.11	0.1
GAIF01024395.1	2.44	1	0.34	0.94	0.73	1.01
GAIF01024398.1	2.01	0.18	0.18	7.37	27.24	9.86
GAIF01024399.1	0.15	0	0	0.34	0	0.32
GAIF01024404.1	0	0	0	0.06	0.13	0
GAIF01024405.1	0	0	0	0	4.51	0
GAIF01024406.1	0	0.03	0	0.15	0	0.16
GAIF01024407.1	0.39	0.16	0	2.68	7.15	0.04
GAIF01024408.1	9.42	5.69	5.77	17.77	27.81	12.13
GAIF01024409.1	14.61	15.63	9.35	6.75	3.69	1.41
GAIF01024410.1	8.09	5.95	6.97	15.31	16.91	8.5
GAIF01024411.1	0	0	0	0.15	0.16	0
GAIF01024414.1	10.24	13.72	20.7	31.62	14.16	7.72
GAIF01024417.1	0.28	0	0.08	0.76	7.4	0.45
GAIF01024418.1	0.1	0.21	0.21	1.11	1.15	0
GAIF01024419.1	0.13	0	0.19	0.17	1.05	0
GAIF01024420.1	0	0	0	0.26	2.21	0

GAIF01024421.1	0	0.05	0.05	0	0.05	0
GAIF01024422.1	0	0.34	0.27	0	0	0
GAIF01024423.1	0	0.04	0	0	0	0
GAIF01024425.1	23.66	27.68	19.44	39.82	28.73	7.34
GAIF01024428.1	17.88	11.09	7.36	18.32	23.35	4.31
GAIF01024429.1	0	0	0	0.12	0	0.11
GAIF01024430.1	3.69	6.03	2.49	1.39	2.8	0.21
GAIF01024431.1	344.04	552.38	406.13	183.8	192.12	59.03
GAIF01024432.1	0.09	0.56	0	1.09	3.7	0
GAIF01024433.1	0	0	0	0	3.75	0
GAIF01024435.1	19.48	6.79	2.26	75.9	79.76	9.42
GAIF01024436.1	0.39	0.39	0.31	0.5	0.35	0.05
GAIF01024437.1	66.56	130.99	93.86	143.85	124.82	24.13
GAIF01024438.1	0	0.25	1.1	0	0	0.51
GAIF01024439.1	1.5	0.96	0.82	0.55	0.98	0
GAIF01024440.1	0	0	0	0.32	0.35	0
GAIF01024441.1	0	0	0	0	0.21	0.19
GAIF01024443.1	26.98	13.81	23.5	23.5	8.93	21.47
GAIF01024448.1	0.24	0	0	0.97	1.87	0.13
GAIF01024451.1	15.66	22.24	9.06	42.63	29.59	12.62
GAIF01024453.1	0.06	0	0	0.14	0.15	0.19
GAIF01024454.1	23.52	5.91	7.43	12.74	9.27	3.23
GAIF01024455.1	0	0.04	0.04	0	0	0
GAIF01024456.1	0.11	0.12	0.36	0	0.26	0.12
GAIF01024457.1	0	0	0	0	0	0.1
GAIF01024458.1	17.96	36.18	10.35	4.37	3.28	0
GAIF01024459.1	17.5	14.02	1.01	30.33	37.53	5.2
GAIF01024460.1	17.32	11.45	7.12	23.05	46.67	18.83
GAIF01024461.1	0.11	0	0	0.12	1.68	0.12
GAIF01024462.1	0.48	0.1	0.21	3.22	20.39	0.95
GAIF01024463.1	2.73	0.11	0.34	7.42	24.01	1.93
GAIF01024464.1	40.42	35.98	42.35	23.5	16.21	10.33
GAIF01024465.1	0	0	0	0.21	1.3	0
GAIF01024466.1	0.05	0	0	1.19	5.41	0.11
GAIF01024467.1	0.14	0	0	0.33	1.99	0.05
GAIF01024468.1	0.67	0	0	0	3.15	0
GAIF01024469.1	8.31	7.57	11.1	12.25	5.94	4.46
GAIF01024471.1	0.68	0	0	1.6	2.58	0
GAIF01024472.1	9.38	8.95	5.72	17.22	17.71	2.72
GAIF01024474.1	11.88	11.16	9.4	27.19	24.89	6.27
GAIF01024475.1	0.1	0	0	0.42	0.63	0
GAIF01024477.1	0	0	0	0	0.03	0
GAIF01024478.1	0.97	0.04	0.04	0	0	0
GAIF01024479.1	0.26	0	0	0	0.45	0.1

GAIF01024481.1	0	0	0	0	0.75	0
GAIF01024482.1	0	0	0	1.66	8.97	0
GAIF01024483.1	12.77	9.54	13.65	17.77	26.67	63.72
GAIF01024485.1	0	0.31	0	0	0	0
GAIF01024486.1	0.07	0.13	0	0.61	0.38	0
GAIF01024487.1	9.76	5.46	3.5	14.75	24.22	45.69
GAIF01024490.1	0.16	0.35	0.09	0.75	1.93	0.35
GAIF01024492.1	0.09	0	0	0	0	0
GAIF01024494.1	0.35	0.48	0.11	1.09	1.45	0.32
GAIF01024495.1	58.03	87.68	40.05	70.16	74.32	22.95
GAIF01024497.1	2.15	1.15	1.81	6.1	6.41	0.98
GAIF01024499.1	0	0	0	0.15	0	0
GAIF01024500.1	0	2.7	0	0	1	0
GAIF01024503.1	0	0	0	0.15	0.35	0
GAIF01024504.1	74.99	82.02	37.91	112.74	109.11	74.05
GAIF01024505.1	0	0	0	0	0.19	0
GAIF01024506.1	0.11	0	0	0.36	0	0.34
GAIF01024509.1	11.48	6.77	9.93	4.56	1.69	2.71
GAIF01024510.1	0.17	0.09	0	0.09	0	0
GAIF01024511.1	0	0	0	0	0.14	0
GAIF01024512.1	0	0	0.1	0	0	0
GAIF01024515.1	0.12	0	0	0	0	0
GAIF01024516.1	0	0	0.06	0	0.21	0.13
GAIF01024520.1	1737.23	2721.19	1198.79	883.14	2065.22	1612.19
GAIF01024522.1	1.42	0.9	2.94	0	0	0
GAIF01024523.1	0.43	0	0	1.69	3.61	0
GAIF01024524.1	0	0	0	0.07	0	0
GAIF01024525.1	11.19	8.14	7.63	17.52	11.73	5.91
GAIF01024526.1	60.96	73.95	46.36	308.76	152.63	28.52
GAIF01024527.1	0.27	0.27	0.24	3.61	11.78	0.06
GAIF01024529.1	0.06	0.21	0.28	0.51	0.83	0.07
GAIF01024530.1	0	0	0	0.07	0	0
GAIF01024531.1	667.83	99.8	71.98	1036.53	150.62	17.03
GAIF01024532.1	0	0.26	0.84	0	0	0.26
GAIF01024533.1	1.31	0.81	0.47	2.49	6.7	1.89
GAIF01024534.1	3.4	2.18	2.88	22.77	22.11	0.17
GAIF01024535.1	0	0	0	0.8	1.8	0
GAIF01024538.1	0.03	0.3	0.01	0.02	0.02	0
GAIF01024539.1	0.65	0.3	0.65	1.3	0.97	0.8
GAIF01024541.1	71.71	51.99	54.28	240.69	367.24	90.78
GAIF01024542.1	43.43	28.4	35.22	4.78	3.93	1.56
GAIF01024544.1	36.44	26.21	36.76	39.73	31.83	33.34
GAIF01024545.1	2558.74	11908.8	6904.05	165.52	338.66	208.93
GAIF01024546.1	0	0	0.06	0	0	0

GAIF01024548.1	0	0.11	0	0.12	0.84	0
GAIF01024549.1	0	0.06	0.19	0	0	0
GAIF01024550.1	0.02	0	0	0	0	0
GAIF01024552.1	3.4	6.49	10.33	8.69	10.63	2.64
GAIF01024553.1	23.86	34.52	44.01	40.78	28.51	9.64
GAIF01024554.1	0.47	0	0.4	0	2.59	2.59
GAIF01024555.1	0	0.06	0	0	0	0
GAIF01024556.1	52.85	24.84	46.61	167.44	176.75	681.64
GAIF01024557.1	0.26	0	0.01	0.69	4.84	0
GAIF01024560.1	14.45	14.68	9.16	39.81	48.71	5.49
GAIF01024561.1	0.09	0.1	0	0.1	0	0
GAIF01024562.1	0	0	0	0.56	0	0
GAIF01024563.1	3.12	0.68	0.19	4.21	17.12	5.13
GAIF01024564.1	10.49	5.7	28.79	12.17	25.23	5.78
GAIF01024565.1	0	0.52	0.35	0	0.06	0.23
GAIF01024566.1	0.06	0	0	0	0	0
GAIF01024568.1	62.29	30.14	28.97	130.56	175.57	104.36
GAIF01024569.1	0.22	0.13	0.56	0.27	1.08	0.22
GAIF01024571.1	0	0.02	0	0.16	1.09	0
GAIF01024572.1	0.14	0	0	0.37	1.36	0
GAIF01024573.1	4.62	2.91	1.3	8.23	14.49	16.25
GAIF01024575.1	19.81	12.23	17.89	28.97	24.1	14.47
GAIF01024577.1	5.6	8.35	8.08	20.8	12.03	2.73
GAIF01024578.1	0	0	0	0.27	1.54	0.13
GAIF01024579.1	0.03	0	0	0.39	0	0
GAIF01024580.1	0.72	0.66	0	2.51	16.23	0.51
GAIF01024582.1	36.44	32.53	19.59	16.75	17.64	10.64
GAIF01024583.1	0.29	0.18	0	0.16	1.91	0
GAIF01024584.1	0.06	0.27	0.14	0	0.08	0.07
GAIF01024585.1	0.58	0	1.04	0	0	0
GAIF01024586.1	0	0	0	0	0.09	0
GAIF01024588.1	0	0	0	0.09	0	0
GAIF01024590.1	0	0	0	0.11	0	0
GAIF01024591.1	0	5.4	1.19	0	0.06	0.22
GAIF01024592.1	6.25	3.26	5.36	12.62	16.68	12.46
GAIF01024593.1	0.23	0	0.13	0	0	0
GAIF01024594.1	0	0	0	0	0.15	0
GAIF01024596.1	0	0	0	0.56	0.43	0
GAIF01024600.1	0.74	0.42	0.14	4.79	6.44	0.11
GAIF01024601.1	0	0	0.15	0	0	0
GAIF01024602.1	0	0	0	0	0.15	0
GAIF01024606.1	75.44	118.17	81.25	90.05	116.08	130.35
GAIF01024607.1	0.11	0.08	0.08	0.53	1.38	0
GAIF01024608.1	0	0	0	0	0.04	0.04

GAIF01024609.1	16.31	7.12	4.65	8.79	13.59	6.93
GAIF01024611.1	2.13	3.03	1.61	12.18	22.25	0.49
GAIF01024612.1	0.36	0	0	0	0.74	0
GAIF01024613.1	0.24	0	0	0.28	0.88	0
GAIF01024615.1	1.35	0	1.45	0	0	0
GAIF01024616.1	0.03	0	0	0	0	0
GAIF01024617.1	2.44	2.05	0.08	2.51	3.02	0
GAIF01024618.1	0	0	0.16	0	0	0.11
GAIF01024619.1	1.9	0.94	0.05	0.6	1.83	1.8
GAIF01024620.1	0.72	0.11	0.11	1.19	6.91	0.79
GAIF01024621.1	0	0	0	0.13	0.59	0
GAIF01024622.1	0.13	0	0	0	2.21	0
GAIF01024623.1	2.35	2.62	1.15	3.73	1.93	0.54
GAIF01024624.1	450.33	210.42	208.27	61.2	31.3	190.28
GAIF01024625.1	0	0.02	0	0	0	0
GAIF01024626.1	3.46	2.82	0	5.29	9.85	2.48
GAIF01024627.1	0.02	0.05	0	0.03	0.06	0
GAIF01024628.1	16.91	13.33	16.01	51.73	35.95	10.79
GAIF01024629.1	9.94	7.48	6.15	17.52	26.84	5.95
GAIF01024631.1	0.11	0	0	0.12	0.32	0.12
GAIF01024632.1	0	0.81	0.33	0	0	0
GAIF01024634.1	0	0.07	0	0	0	0
GAIF01024636.1	0	0.08	0	0.09	0.66	0
GAIF01024637.1	0	0.34	0	0.12	0	0
GAIF01024638.1	18.99	8.59	2.71	80.72	181.81	33.45
GAIF01024639.1	0	0.37	0	0	0.21	0
GAIF01024640.1	0.92	0.69	0.08	0.66	0.34	0
GAIF01024641.1	0.42	0.45	0.23	0.26	0	0.53
GAIF01024642.1	0	0	0	0	0.19	0
GAIF01024643.1	0.66	0.3	0	1.95	10.87	0.19
GAIF01024644.1	0	0.18	0	0.51	0.25	0
GAIF01024645.1	0.39	0	0	0.18	2.5	0
GAIF01024646.1	0.5	0.26	0.16	6.28	32.04	0
GAIF01024648.1	0	0	0.07	0	0	0
GAIF01024649.1	0.14	0.06	0.03	0.38	1.11	0.24
GAIF01024650.1	0	0	0	0	0	0.23
GAIF01024652.1	0	0	0	0	0.53	0
GAIF01024654.1	10.26	2.89	0.8	26.69	63.46	7.57
GAIF01024655.1	0	0	0	0	0.07	0
GAIF01024657.1	0	0.01	0	0	0	0
GAIF01024659.1	2.59	2.4	0.41	12.25	34.4	4.54
GAIF01024660.1	6.98	4.12	6.97	23.08	19.97	7.51
GAIF01024661.1	17.14	20.08	40.18	34.37	23.91	20.99
GAIF01024663.1	0	0	0	0	0.29	0

GAIF01024665.1	0.5	0.14	0	0.58	1.66	0
GAIF01024667.1	0.37	0.14	0	7.8	20.05	0.27
GAIF01024668.1	0	0	0.05	0	0	0
GAIF01024669.1	0	0.13	0	0.28	1.74	0
GAIF01024670.1	0	0	0	0.29	0	0
GAIF01024672.1	0.23	0	0.25	0.44	1.93	0
GAIF01024673.1	8.23	5.12	5.81	16.6	21.67	4.86
GAIF01024674.1	1.74	5.55	2.92	0.95	0.12	0.11
GAIF01024675.1	0	0	0	0	2.16	0.2
GAIF01024677.1	1.35	0.35	0	0.75	1.55	0.5
GAIF01024678.1	25.56	28.28	14.77	85.24	87.98	34.43
GAIF01024679.1	0.32	0.35	0	2.7	12.55	0
GAIF01024681.1	0.23	0.26	0	1.36	6.5	0
GAIF01024682.1	2.63	0.59	0.85	0	2.03	1.02
GAIF01024683.1	10.11	7.08	14.38	28.71	26.16	12.45
GAIF01024684.1	8.52	8.47	5.99	20.05	9.48	1.72
GAIF01024687.1	49.99	106.78	115.81	148.24	162.86	86.98
GAIF01024688.1	2.39	0.82	0.14	5.83	16.16	2.91
GAIF01024689.1	0.05	0	0	0.06	0	0
GAIF01024690.1	0	0	0	0	0	0.24
GAIF01024691.1	10.2	3.02	3.56	14.6	32.75	17.87
GAIF01024692.1	3.58	4.12	1.69	0.91	0.09	0.08
GAIF01024693.1	0	0.2	0.12	0	0	1.9
GAIF01024694.1	7.77	6.57	6.95	16.34	9.81	6.02
GAIF01024695.1	0.04	0	0	0	0.05	0.05
GAIF01024696.1	45.19	24.74	61.21	89.27	50.14	78.48
GAIF01024698.1	18.58	4.89	10.02	5.69	3.93	7.86
GAIF01024699.1	0.51	0.55	0	5.08	18.25	0
GAIF01024700.1	1.34	0.79	0.8	4.23	9.21	0.4
GAIF01024701.1	101.12	9.35	0.48	28.72	93.02	0
GAIF01024706.1	0	0.02	0	0	0.4	0
GAIF01024707.1	19.69	14.92	16.37	72.47	42.69	9.64
GAIF01024708.1	0	0	0.1	0	0	0.1
GAIF01024711.1	8.16	6.76	9.08	21.97	24.71	17.84
GAIF01024712.1	0.11	0.17	0	0.04	0.05	0
GAIF01024714.1	2.92	2.82	0.21	0.39	0.24	8.16
GAIF01024715.1	0.1	0	0.06	0	0	0
GAIF01024716.1	0	0.13	0.19	0.77	1.72	0
GAIF01024717.1	18.4	11.14	14.12	16.44	16.13	8.55
GAIF01024719.1	0.18	0.1	0.07	0.9	4.33	0.49
GAIF01024721.1	4.65	1.7	1.92	3.55	1.76	0
GAIF01024722.1	7.22	6.83	2.07	8.02	20.02	2.42
GAIF01024723.1	6.67	0.47	0.16	36.93	165.9	2.64
GAIF01024724.1	0.2	0	0	0.23	0.72	0

GAIF01024725.1	0	0	0	0	0.1	0
GAIF01024726.1	16.16	18.86	11.57	58.83	34.84	6.13
GAIF01024727.1	0	0.06	0.06	0.6	2.11	0.17
GAIF01024728.1	0	0	0	0.1	0.03	0
GAIF01024729.1	21.66	13.7	12.65	29.38	35.02	12.92
GAIF01024730.1	111.16	119.09	19.43	1.55	1.2	0.38
GAIF01024732.1	21.16	31.93	8.44	54.61	60.62	1.32
GAIF01024733.1	0	0	0	0.31	1.01	0.18
GAIF01024734.1	9.5	5.58	7.18	9.86	10.87	13.43
GAIF01024736.1	0	0	0.09	0	0.21	0
GAIF01024737.1	0	0	0	0	0.15	0
GAIF01024739.1	24.4	17.35	17.3	34.73	35.43	12.31
GAIF01024740.1	0.06	0.62	0.52	0.18	0.35	0.63
GAIF01024741.1	17.83	9.28	7.07	2.38	1.41	0.61
GAIF01024742.1	0.2	0.5	0	9.87	78.84	0
GAIF01024744.1	0	0.14	0	0	0	0.14
GAIF01024745.1	0	0.14	0	0	0.89	0
GAIF01024746.1	0	0	0	0	1	0
GAIF01024747.1	0	0	0	0	0.39	0
GAIF01024748.1	0.4	0	0	0.43	1.34	0
GAIF01024749.1	225.19	305.21	152.24	1056.43	987.45	95.63
GAIF01024752.1	0	0	0	0.2	0.42	0
GAIF01024755.1	22.57	15.58	11.98	13.31	16.84	14.52
GAIF01024757.1	19.86	9.93	5.69	52.89	55.34	8.66
GAIF01024758.1	13.73	1.7	2.61	11.62	23.38	2.99
GAIF01024759.1	0	0	0	0.12	0.38	0
GAIF01024761.1	0	0	0.06	0.27	0.83	0
GAIF01024762.1	0.08	0.09	0	0	0	0
GAIF01024763.1	0	0	0	1.15	6.53	0
GAIF01024764.1	13.9	15	28.01	32.2	20.99	17.43
GAIF01024765.1	3.48	45.59	5.46	1.15	9.57	0.18
GAIF01024766.1	0	0	0	0.45	1.06	0.03
GAIF01024767.1	77.82	100.36	101.73	106.87	90.98	45.24
GAIF01024768.1	0.16	0	0	0	0	0
GAIF01024770.1	0.14	0	0.04	0.21	0.13	0.27
GAIF01024772.1	6.42	6.83	7.69	14.95	24.33	7.54
GAIF01024773.1	0.18	0.04	0	0.89	5.32	0.04
GAIF01024775.1	0.19	0	0	0.04	0.09	0
GAIF01024776.1	0	0	0	0.37	0.47	0.09
GAIF01024778.1	0.19	0.07	0	0.22	0.52	0
GAIF01024779.1	0	0	0	0.19	0.1	0
GAIF01024780.1	0.02	0.1	0	0.25	1.9	0.03
GAIF01024781.1	0.36	0.52	0.26	0.14	0.14	0.4
GAIF01024783.1	19.17	1.87	2.13	108.74	449.93	58.63

GAIF01024784.1	0	0.13	0	0.14	0.64	0
GAIF01024786.1	0	0	0	1.09	3.63	0.18
GAIF01024787.1	0	0.09	0	0	0.1	0
GAIF01024789.1	0.45	0.49	0	0.37	1.16	0
GAIF01024790.1	0	0	0.1	0	0	0
GAIF01024791.1	0	0.04	0	0	0	0
GAIF01024792.1	0.21	0	0	0.12	0	0.11
GAIF01024793.1	0.08	0.09	0	0.28	0.59	0.09
GAIF01024795.1	42.2	51.66	49.55	11.14	6.87	10.54
GAIF01024796.1	0	0	0.11	0	0	0
GAIF01024800.1	0	0	0	0	0.27	0.07
GAIF01024801.1	3851.94	3912.41	3730.03	1968.72	1954.94	4152.31
GAIF01024803.1	0.36	0.1	0.1	0.84	6.52	0.2
GAIF01024804.1	10.84	7.83	10.01	29.29	23.74	9.52
GAIF01024805.1	0.08	0	0	0.16	0.19	0
GAIF01024806.1	0.24	0	0	2.52	6.53	0
GAIF01024807.1	0	0	1.68	0	0	0
GAIF01024809.1	6.04	7.49	0.94	17.73	13.82	3.63
GAIF01024810.1	0.68	1.62	1	0	2.73	1
GAIF01024811.1	18.11	3.1	7.56	16.11	5.91	12.9
GAIF01024812.1	0.11	0	0	0.04	0.48	0
GAIF01024813.1	0	0.17	0	0.54	1.5	0
GAIF01024814.1	0	0	0	0.35	1.29	0
GAIF01024818.1	258.33	159.34	138.9	65.99	181.01	527.55
GAIF01024821.1	12.13	6.77	11.37	23.09	37.1	28.79
GAIF01024823.1	35.75	43.5	42.93	68.88	72.96	27.96
GAIF01024825.1	7.89	33.66	63.07	0	0	17.4
GAIF01024826.1	0.35	1.39	1.05	0.56	0.23	0
GAIF01024827.1	26.92	22.93	7.28	39.7	45.4	4.2
GAIF01024829.1	0.09	0.2	0	0.33	0	0
GAIF01024831.1	22.06	19.99	10.93	2.4	3.05	0.98
GAIF01024833.1	0	0	0	0	0.15	0
GAIF01024834.1	0	1.3	0.16	0	0.04	0
GAIF01024837.1	12.42	12.44	15.3	24.61	12.57	7.95
GAIF01024840.1	6.41	11.06	0.05	1.48	3.88	0.11
GAIF01024841.1	1.07	1.74	0.42	0.35	0.37	0.25
GAIF01024842.1	0.38	0	0.25	0.18	0	0
GAIF01024843.1	0.99	0.93	0.48	6.5	9.64	0.73
GAIF01024844.1	0.27	0.22	0	0.24	2.72	0
GAIF01024845.1	0	0	0	0	0.25	0.18
GAIF01024846.1	0.04	0.04	0	0.04	0.09	0
GAIF01024848.1	0.11	0.06	0	0	0.07	0
GAIF01024849.1	9.45	14.12	18.51	18.64	12.6	6.15
GAIF01024850.1	6.7	10.15	2.11	1.08	2.28	0

GAIF01024851.1	0	0	0	0.04	0	0
GAIF01024855.1	0.05	0	0	0	0	0
GAIF01024856.1	7.33	8.05	6.52	17.44	11.72	3.07
GAIF01024857.1	40.3	36.74	83.14	104.9	54.62	122.39
GAIF01024860.1	9.59	10.03	11.21	23.56	32.82	24.62
GAIF01024862.1	0	0	0	0.17	0.36	0.16
GAIF01024864.1	0.1	0.11	0.06	0	0	0.06
GAIF01024866.1	3.06	0	0.2	0.9	5.35	1.96
GAIF01024867.1	143.88	71.54	37.95	89.23	69.7	24.13
GAIF01024868.1	2.38	1.04	2.21	3.93	12.86	0.23
GAIF01024869.1	7.06	0.3	0.41	21.68	56.3	119.46
GAIF01024871.1	47.48	57.09	58.96	39.33	38.73	63.62
GAIF01024873.1	2.83	1.82	0.66	4.19	9.29	0.14
GAIF01024874.1	0	0	0.15	0.32	1.5	0
GAIF01024875.1	9.05	6.34	7.72	2.26	0.65	0.07
GAIF01024876.1	89.47	85.85	264.76	90.99	50.9	272.31
GAIF01024877.1	0	0	0.36	0.13	0.4	0
GAIF01024878.1	0	0	0	0.08	0.12	0
GAIF01024879.1	12.94	17.01	21.79	45.5	34.5	8.56
GAIF01024880.1	33.99	42.35	47.4	7.04	5.74	3.64
GAIF01024881.1	2.26	1.59	0.25	4.45	11.72	0.25
GAIF01024882.1	0	0.17	0.69	0	0	0
GAIF01024883.1	9.21	0.97	0.27	8.53	22.34	1.25
GAIF01024884.1	3.88	1.79	1.43	0.9	1.05	0.27
GAIF01024886.1	0.45	0.51	0.25	0.98	1.44	0
GAIF01024887.1	0.05	0.34	0.14	0.05	0.15	0.12
GAIF01024888.1	10.14	14.61	10.01	16.41	18	4.15
GAIF01024889.1	2.46	2.51	1.07	11.52	25.65	2.02
GAIF01024890.1	0	0.27	0.24	0	0	0
GAIF01024892.1	0	0.29	0	0	0.15	0
GAIF01024894.1	47.6	92.29	23.56	182.99	183.94	5.61
GAIF01024895.1	8.22	7.02	7.71	17.31	20.23	15.88
GAIF01024896.1	0.01	0	0	0.02	0.54	0
GAIF01024898.1	1.02	0.02	0.69	0.12	2.45	0
GAIF01024899.1	0	0	0	0	0.05	0
GAIF01024900.1	1.15	0.73	0.64	1.67	0.88	0.56
GAIF01024901.1	0	0.02	0.2	0.02	0.27	0
GAIF01024902.1	2.64	4.14	1.32	1.17	0.47	0.18
GAIF01024903.1	0	0	0	0	0.08	0
GAIF01024904.1	3.96	1.08	5.52	4.03	1.78	10.38
GAIF01024905.1	0	0	0	0.12	0	0
GAIF01024906.1	0	0.5	1.22	0.47	1.2	0.77
GAIF01024907.1	0.35	0.5	0.89	0.67	0.14	0.13
GAIF01024908.1	0	0.41	0	0.46	1.02	0

GAIF01024909.1	267.47	179.31	47.82	439.38	360.97	29.16
GAIF01024910.1	0	0	0.08	0	0	0
GAIF01024913.1	0	0	0.09	0.09	0	0
GAIF01024914.1	0.55	0.3	0	170.72	224.85	388.62
GAIF01024915.1	0	0	0	0.46	0.96	0.15
GAIF01024916.1	0.15	0.07	0.07	0.63	0.18	0.05
GAIF01024917.1	0	0.1	0.05	0	0.16	0
GAIF01024918.1	0.43	0.09	0.28	0.1	0	0
GAIF01024919.1	148.4	99.29	32.34	78.99	100.65	97.45
GAIF01024920.1	18.75	0.66	1.32	3.15	0.09	0.17
GAIF01024921.1	0.38	0	0	0.09	0.28	0
GAIF01024922.1	19.99	10.69	4.78	49.91	69.82	15.89
GAIF01024923.1	19.51	0	0.96	7.89	1.19	3.62
GAIF01024924.1	29.62	9.89	12.69	61.22	44.48	141.94
GAIF01024925.1	0.27	0.07	0.07	0.93	1.68	0.44
GAIF01024926.1	10.56	0	16.03	0	0	9.17
GAIF01024927.1	1.45	0.14	0.14	3.41	5.88	1.05
GAIF01024928.1	25.72	15	14.63	86.63	109.6	16.68
GAIF01024931.1	156.4	175.5	136.46	210.84	257.46	76.71
GAIF01024932.1	0.54	0.48	0.49	0.63	0.77	0
GAIF01024933.1	0.04	0	0	0	0	0
GAIF01024934.1	0.3	0	0	2.63	10.35	2.99
GAIF01024935.1	44.91	14.78	5.59	29	35.26	3.56
GAIF01024937.1	1.09	1.98	2.1	1.85	2.14	0.45
GAIF01024938.1	0	0	0	0	0.06	0
GAIF01024939.1	1.8	1.07	2.35	2.58	0.6	2.84
GAIF01024940.1	3.01	2.25	0.82	16.78	26.65	2.28
GAIF01024943.1	2.97	8	0.84	21.93	43.33	0.24
GAIF01024944.1	0	0	0	0	0.02	0
GAIF01024945.1	13.86	13.96	18.31	29.29	23.45	39.07
GAIF01024947.1	0	0	0.22	1	3.94	0
GAIF01024949.1	0	0	0	0.18	0.25	0
GAIF01024952.1	0.43	0.31	0	0.77	1.09	0.16
GAIF01024953.1	0	0	0	0.13	0	0
GAIF01024955.1	0.09	0	0	0	0.98	0
GAIF01024956.1	12.22	19.73	17.1	23.32	13.41	2.83
GAIF01024958.1	2.65	1.19	2.85	1.71	0.78	1.54
GAIF01024959.1	2.8	3.26	0.24	6.34	9.33	2.76
GAIF01024960.1	0	0.05	0	0	0	0
GAIF01024961.1	0.35	2.23	5.03	1.09	0	1.05
GAIF01024962.1	20.35	0.28	1.13	3.52	0.19	0.11
GAIF01024963.1	0	0.05	0	0.14	0.05	0.05
GAIF01024964.1	0.11	0	0.06	0.13	0.26	0
GAIF01024965.1	0.1	0.06	0.15	0.18	0.09	0

GAIF01024967.1	0	0	0	0	0	0.34
GAIF01024968.1	18.63	28.02	25.83	38.2	43.62	23
GAIF01024969.1	0.16	0.09	0.09	0.05	0	0.04
GAIF01024971.1	0	0.09	0.11	0.02	0.02	0.13
GAIF01024972.1	0.42	0.1	0.1	0.9	4.69	2.56
GAIF01024973.1	8.39	3.14	8.32	7.88	12.65	11.64
GAIF01024976.1	0	0	0	0.05	0.05	0
GAIF01024977.1	21.52	16.55	14.96	24.57	37.81	5.62
GAIF01024980.1	0	0	0	0.26	1.22	0.62
GAIF01024981.1	11.42	12.51	9.02	26.2	22.63	3.28
GAIF01024982.1	0	0	0	0	0.07	0.02
GAIF01024989.1	0.28	0	0	1.46	2.77	2.38
GAIF01024990.1	0.16	0.11	0.17	1.13	4.09	2.34
GAIF01024991.1	2.34	1.02	0.26	15.73	58.16	0.26
GAIF01024992.1	14.64	18.4	27.82	55.46	35.71	14.46
GAIF01024993.1	0.66	0	0	0.48	2.39	2.56
GAIF01024995.1	2193.22	2312	3369.52	1346.63	1041.5	4217.97
GAIF01024996.1	0.31	0.17	0	2.36	9.59	0
GAIF01024997.1	18.47	21.34	17.49	28.23	19.24	7.85
GAIF01024999.1	1.24	1.62	1.63	3.45	0.6	2.19
GAIF01025000.1	0	0.22	0	0.72	3.98	0.23
GAIF01025001.1	0	0	0	0.45	2.82	4.39
GAIF01025004.1	0	0	0	3.75	0	0.63
GAIF01025005.1	0.02	0.02	0.03	0	0.02	0
GAIF01025007.1	0.1	0	0	0	0	0
GAIF01025008.1	0	0.38	0	1.09	0	0
GAIF01025010.1	0	0.13	0	0.01	0.55	0
GAIF01025011.1	0	0	0	0	0	0.15
GAIF01025012.1	0.44	0.08	0.11	0.08	0	0.02
GAIF01025013.1	20.41	15.78	19.92	36.32	18.31	7.35
GAIF01025014.1	0.09	0	0	0	0.11	0
GAIF01025015.1	0.04	0	0	0.2	0.94	0.09
GAIF01025016.1	0.03	0.22	0	0	0	0
GAIF01025017.1	39.43	40.09	15.97	64.08	81.61	3.16
GAIF01025018.1	0.23	0.14	0.35	0.08	0.25	0.05
GAIF01025019.1	0.18	0.08	0.04	0.62	1.83	0
GAIF01025020.1	0.06	0	0.17	0.74	2.52	0.27
GAIF01025021.1	103.57	52.52	50.12	223.88	260.71	98.96
GAIF01025024.1	0.24	0	0	2.11	10.88	0
GAIF01025025.1	3.99	5.18	1.63	34.93	43.12	1.28
GAIF01025026.1	0.88	0.25	0	5.49	15.79	0.06
GAIF01025027.1	25.03	18.37	29.33	32.72	38.61	77.17
GAIF01025029.1	0.19	0.46	0.05	4.51	10.53	0.05
GAIF01025030.1	12.17	12.57	20.15	14.87	14.36	6.95

GAIF01025031.1	0	0	0.15	0.72	2.81	0
GAIF01025033.1	0.88	0.3	0.23	3.73	17.3	0
GAIF01025034.1	1.45	2.78	0.47	6.32	5.73	0.19
GAIF01025035.1	3.01	0.89	0	7.3	8.39	4.52
GAIF01025036.1	0.18	0	0	0	0.14	0
GAIF01025037.1	24.89	26.27	8.14	66.98	96.76	0.73
GAIF01025039.1	0	0	0.14	0	0	0.14
GAIF01025040.1	1.97	2.04	2.36	1.73	1.79	1.21
GAIF01025041.1	0	0	0	0	0.22	0
GAIF01025042.1	0.09	0	0.03	0.17	0.11	0
GAIF01025043.1	0	0.55	0.62	0.02	0.03	0.22
GAIF01025045.1	4.2	3.05	2.5	0.2	0	0
GAIF01025046.1	14.28	8.29	12.96	16.3	8.24	10.07
GAIF01025047.1	0	0.19	0	0.6	3.75	0
GAIF01025048.1	0.31	0.64	0.11	0	0	0
GAIF01025049.1	0.14	0.08	0.16	0.74	0	0
GAIF01025053.1	7.21	9.84	5.1	2	1.86	0.85
GAIF01025055.1	5.79	5.92	11.66	25.85	25.18	4.07
GAIF01025059.1	0.26	0.11	0	1.09	6.17	0.21
GAIF01025060.1	0	0.1	0.05	0.05	0	0
GAIF01025061.1	0.45	0.33	0.17	0.26	0.81	0.25
GAIF01025063.1	10.78	18.76	9.33	34.44	20.75	2.09
GAIF01025064.1	0	0.45	0.76	0	0	0.19
GAIF01025065.1	6.29	6.37	5.24	23.92	15.54	0
GAIF01025066.1	0	0	0	0	0	0.27
GAIF01025067.1	0.14	0.18	0	1.85	11.31	0.42
GAIF01025068.1	0	0	0	1.87	5.57	0
GAIF01025069.1	15.58	13.37	28.16	25.63	39.16	46.75
GAIF01025070.1	0.16	0	0	0.17	0	0
GAIF01025071.1	2.05	0.39	0.17	2.59	12.99	1.34
GAIF01025072.1	2703.69	1849.49	1436.23	1289.79	674.28	2768.84
GAIF01025073.1	0	0	0	0	0.07	0
GAIF01025074.1	0.07	1.04	0.31	0.54	1.03	0.11
GAIF01025075.1	0	0	0	0	0.11	0
GAIF01025076.1	14.68	9.22	1.29	13.41	20.18	9.34
GAIF01025078.1	0.59	0.2	0.17	2.47	5.94	0.21
GAIF01025079.1	45.35	49.15	68.9	80.59	46.15	50.79
GAIF01025080.1	0	0	0	0.31	0	0
GAIF01025081.1	8.01	29.5	13.85	15.97	6.39	9.08
GAIF01025082.1	0.82	0.22	0.39	3.63	11.84	0.57
GAIF01025084.1	3.42	0	0.08	3.31	0.35	2.89
GAIF01025085.1	0	0.05	0	0.06	0	0
GAIF01025086.1	0.2	0	0.15	0	0	0.15
GAIF01025087.1	0.92	0.22	1.22	0	0.1	0.02

GAIF01025088.1	0	1.22	0.32	0	0	0.13
GAIF01025090.1	71.38	62.83	54.48	85.56	73.41	149.33
GAIF01025091.1	10.53	16.83	24.59	1.22	0.58	0.52
GAIF01025092.1	4.57	4.1	8.28	1.01	3.49	1.52
GAIF01025093.1	0.2	0.18	0.07	0.03	0.9	0.04
GAIF01025095.1	9	9.62	25.27	25.68	14.02	15.61
GAIF01025099.1	63.62	78.25	50.62	62.52	1.14	11.43
GAIF01025100.1	1.46	1.19	1.55	1.03	0.75	1.15
GAIF01025101.1	0	0.18	0.18	0.19	0	0
GAIF01025102.1	8.58	9.47	10.78	21.44	29.97	4.79
GAIF01025103.1	0	0	0.03	0.07	0.04	0
GAIF01025104.1	0	0.08	0	0.09	0	0
GAIF01025105.1	18.93	25.35	30.95	51.03	43	16.98
GAIF01025108.1	49.82	1.19	8.49	58.07	22.91	67.44
GAIF01025109.1	0.11	0	0	0.69	1.77	0
GAIF01025110.1	0.69	0.25	0	0.18	1.02	0
GAIF01025111.1	0.15	0.08	0.08	0.89	2.24	0.08
GAIF01025112.1	0	0	0	0.39	1.86	0.12
GAIF01025113.1	151.49	178.68	185.67	245.67	123.57	73.97
GAIF01025114.1	26.91	19.56	34.74	57.14	33.63	33.13
GAIF01025117.1	0	0	0	0	0.6	0
GAIF01025119.1	70.62	126.99	68.31	85.14	74.6	9.78
GAIF01025120.1	0	0.1	0	0	1.02	0
GAIF01025121.1	6.38	5.61	8.53	11.77	14.17	8.41
GAIF01025122.1	1.1	0.54	0.22	11.4	47.35	1.44
GAIF01025123.1	27.09	32.1	39.06	80.25	31.66	28.46
GAIF01025124.1	58.36	29.61	15.68	77.24	36.21	39.94
GAIF01025125.1	0	0.07	0	0	0	0
GAIF01025126.1	0.14	0	0.3	7.58	11.62	0
GAIF01025128.1	0	0	0	0	0.03	0
GAIF01025129.1	0.14	0	0.03	0.5	1.93	0.47
GAIF01025130.1	0.25	0	0	0.19	0.1	0
GAIF01025131.1	8.91	6.21	1.43	32.12	83.29	29.56
GAIF01025132.1	0	0	0	0.02	0.09	0
GAIF01025133.1	0.09	0	0	0.52	3.74	0
GAIF01025134.1	1.91	0	0	6.97	41.5	1.05
GAIF01025135.1	0	0	0	0	0.38	0
GAIF01025136.1	0	0	0	1.07	3.88	0.31
GAIF01025137.1	1010.03	291.72	252.83	347.19	252.42	206.78
GAIF01025138.1	1550.48	1495.79	687.65	5575.28	5291.88	1876.85
GAIF01025140.1	0.12	0	0	0	0	0
GAIF01025141.1	0	0	0.16	0.06	1.08	0
GAIF01025143.1	0	0.06	0	0	0	0.03
GAIF01025145.1	7.75	8.09	7.77	7.39	8.22	5.43

GAIF01025146.1	0	0.12	0	0.25	0.6	0
GAIF01025148.1	0	0	0	0.11	0	0.04
GAIF01025150.1	28.5	36.77	28.41	48.6	31.82	9.85
GAIF01025151.1	60.79	53.37	64.81	36.24	51.45	16.96
GAIF01025155.1	0	0	0	0	1.09	1.16
GAIF01025158.1	287.21	171.52	241.08	203.31	95.26	78.41
GAIF01025159.1	0	0	0	0.13	0	0
GAIF01025160.1	0.95	0.47	0	2.62	4.49	0.1
GAIF01025161.1	13.92	3.77	3.28	12.19	11.67	22.69
GAIF01025162.1	0.18	0.01	0	0.03	0.57	0
GAIF01025163.1	67.26	43.7	101.21	52.37	32.69	26.07
GAIF01025164.1	0.57	0.77	0	9.55	38.75	1.72
GAIF01025166.1	0	0	0	0.62	1.34	0
GAIF01025167.1	0.09	0	0	0	0	0
GAIF01025168.1	19.96	12.03	24.06	43.69	28.31	38.85
GAIF01025170.1	0.12	0	0.13	0.28	1.02	0
GAIF01025171.1	0	0	0	0	0.21	0.19
GAIF01025172.1	0	0	0	1	2.38	0.04
GAIF01025173.1	0	0.18	0.04	0	1.6	0
GAIF01025175.1	0	0	0	0.2	0	0
GAIF01025176.1	1.59	0.12	0.12	3.33	9.51	0.31
GAIF01025177.1	7.48	8.13	1.35	2.73	1.86	0
GAIF01025178.1	1.29	0.9	0.88	55.91	53.15	18.68
GAIF01025179.1	14.46	13.22	18.28	17.35	11.26	12.73
GAIF01025180.1	0	0	0	0	0.31	0
GAIF01025183.1	3.64	2.34	1.18	0.94	0.81	0
GAIF01025184.1	0	0	0	0.06	0.25	0
GAIF01025185.1	0	0	0	0	0.72	0
GAIF01025186.1	0.35	0.22	0	0.77	0.58	0
GAIF01025187.1	0.35	0	0	1.36	8.32	1.81
GAIF01025188.1	0	0	0	0.04	0.04	0
GAIF01025189.1	1.2	3.07	0.84	2.98	3.7	0
GAIF01025191.1	21.03	18.89	18.49	35.49	29.1	21.6
GAIF01025192.1	6.17	9.18	8.11	21.65	22.3	1.52
GAIF01025193.1	5.11	11.06	8.63	36.1	22.45	5.47
GAIF01025195.1	0.6	0.84	1.04	1.5	1.24	0.28
GAIF01025196.1	31.21	81.25	44.89	70.94	89.4	9.2
GAIF01025197.1	32.36	42.14	22.23	35.26	44.57	7.61
GAIF01025198.1	0	0.06	0	0.07	0.53	0
GAIF01025201.1	0	0.05	0	0	0.06	0
GAIF01025203.1	12.23	18.06	20.82	18.26	12.35	8.61
GAIF01025207.1	0.11	0.12	0	2.07	5.9	0.12
GAIF01025208.1	0.01	0	0	0	0.73	0.03
GAIF01025209.1	0	2.43	0	3.07	0	0

GAIF01025210.1	310.66	352.29	860.87	977.58	475.06	946.05
GAIF01025211.1	22.29	14.06	10.31	33.39	31.4	10.35
GAIF01025212.1	0.18	0.38	0.05	0.19	0.1	0
GAIF01025213.1	0	0.04	0	0.1	0.32	0
GAIF01025214.1	0	0	0	0	0.23	0
GAIF01025215.1	2.53	0	0	9.24	16.93	2.9
GAIF01025216.1	0.27	0.27	0.35	0.34	0.61	0.14
GAIF01025218.1	0	0	0	0.1	0.59	0
GAIF01025220.1	0	0	0	0	0.77	0
GAIF01025221.1	0.71	0.28	0.22	5.06	16.4	0.34
GAIF01025222.1	11.18	5.03	10.34	20.01	12.1	19.71
GAIF01025223.1	16.18	361.74	192.87	0.9	10.61	6.14
GAIF01025224.1	0	0	0.05	0	0.05	0
GAIF01025226.1	0.09	0.2	0.2	0.1	0.34	0
GAIF01025227.1	0.26	0	0	3.32	15.44	0
GAIF01025228.1	27.96	31.6	25.43	63.77	75.96	46.52
GAIF01025230.1	6.47	0.09	0	1.59	0	0
GAIF01025232.1	20.48	4.99	12.8	17.64	13.09	16.08
GAIF01025233.1	11.13	2.63	4.14	14.97	19.5	44.08
GAIF01025234.1	6.35	3.57	4.58	14.78	10.01	5.36
GAIF01025235.1	0	0	0	0.4	2.49	0
GAIF01025236.1	20.8	19.6	12.05	108.47	135.3	8.89
GAIF01025238.1	0.42	0.21	0.14	1.31	10.16	0.71
GAIF01025239.1	14.59	6.62	6.53	19.04	18.19	11.91
GAIF01025240.1	0.42	0.31	0.62	0	0.51	1.86
GAIF01025241.1	0.99	1.08	1.33	1.35	0.2	1.28
GAIF01025242.1	0.81	0	0	4.41	18.35	4.59
GAIF01025243.1	11.15	16.33	11.06	29.22	14.22	2.87
GAIF01025244.1	1.89	1.28	0.49	2.97	15.59	0.41
GAIF01025245.1	0.48	0.97	1.43	1.83	2.96	0.45
GAIF01025247.1	0	0	0	0.7	2.9	0.11
GAIF01025249.1	18.03	33.68	15.48	37.77	28.01	3.3
GAIF01025251.1	7.44	2.73	1.79	19.22	16.81	4.69
GAIF01025254.1	0.72	0.06	0	0.45	0.13	0
GAIF01025256.1	0	0	0.07	0.24	1.32	0.49
GAIF01025257.1	2.01	1.22	1.23	0	0	0.09
GAIF01025259.1	0.88	0	0	0.82	8.69	8.94
GAIF01025261.1	0.93	2.32	1.47	2.67	2.04	0
GAIF01025262.1	0.03	0	0	0	0	0
GAIF01025263.1	0.44	0	0	1.33	3.28	0.1
GAIF01025264.1	0.37	0	0	0.61	1.19	0.06
GAIF01025265.1	29.16	35.26	31.13	45.01	37.01	8.91
GAIF01025267.1	0.13	0	0	0.29	3.04	1.39
GAIF01025268.1	0.06	0.13	0	0.13	1.8	0.24

GAIF01025269.1	0.22	0	0	0	0	0
GAIF01025271.1	0.64	0.1	0.91	3.63	18.94	0
GAIF01025272.1	0.03	0	0.04	0	0	0
GAIF01025273.1	1.54	0.47	0	3.34	3.05	1.42
GAIF01025274.1	18.93	18.42	4.6	51.33	74.83	3.95
GAIF01025275.1	0	7.06	1.85	0	0	0.09
GAIF01025277.1	0	0.07	0.07	0	0	0
GAIF01025279.1	1.11	0.24	0.12	2.45	7.36	0.25
GAIF01025280.1	3.12	4.28	4.32	9.53	6.95	1.66
GAIF01025281.1	0	0	0.07	0.58	0	0.07
GAIF01025283.1	25.62	27.56	28.48	3.32	2.67	12.44
GAIF01025284.1	6.48	5.77	1.03	6.37	16.51	5.63
GAIF01025285.1	0	0	0	0.05	0.05	0
GAIF01025288.1	0.52	0.34	0	1.09	1.51	0
GAIF01025289.1	26.35	41.3	59.62	76.53	36.74	30.46
GAIF01025290.1	0.06	0	1.4	5.1	3.6	0
GAIF01025292.1	0.11	0.06	0.06	0.19	0.49	0
GAIF01025293.1	0	0	0	0	0.15	0
GAIF01025295.1	345.35	88.59	84.08	389.58	401.79	1475.75
GAIF01025296.1	17.36	10.21	3.51	0.79	0.82	0.38
GAIF01025297.1	0	0	0	0.22	0.23	0
GAIF01025298.1	0.54	0.2	0.18	0.54	0.82	0.05
GAIF01025299.1	515.08	89.51	67.01	62.53	55.51	135.93
GAIF01025301.1	276.75	92.47	32.76	122.68	149.96	193
GAIF01025302.1	0	0.7	0.4	0	0.05	0.22
GAIF01025303.1	0	0	0	0	0.37	0
GAIF01025308.1	0	0	0	0	4.1	0.2
GAIF01025309.1	7.62	6.79	1.93	27.17	21.36	1.41
GAIF01025310.1	12.72	12.61	8.49	2.13	0.49	0.67
GAIF01025311.1	22.79	24.89	20.33	36.5	30.75	8.32
GAIF01025312.1	0.15	0	0.16	0	0.12	0
GAIF01025313.1	0	0.48	0	3.58	11.66	0
GAIF01025314.1	0.75	1.26	0.67	0.7	0	0
GAIF01025317.1	146.39	114.39	39.22	230.68	248.02	57.8
GAIF01025318.1	0.08	0	0	0	0	0.05
GAIF01025320.1	0	0.07	0	0.45	1.64	0.14
GAIF01025321.1	0	0.06	0	0	0	0
GAIF01025323.1	0	0.1	0.05	0	0	0.05
GAIF01025324.1	0.1	0.22	0.19	1.12	4.19	0
GAIF01025327.1	0	0.07	0	0.23	0.08	0.07
GAIF01025328.1	6.99	3.34	6.05	12.73	21.01	31.59
GAIF01025329.1	2.62	1.57	0	15.03	28.93	0.41
GAIF01025331.1	0	0	0	0.12	0.65	0.06
GAIF01025332.1	0.03	0.04	0.02	0.18	0	0.11

GAIF01025333.1	0.3	0.11	0.11	0	0	0.11
GAIF01025334.1	0.06	0.12	0.12	0	0.2	0
GAIF01025335.1	0	0.14	0	0	0	0
GAIF01025337.1	15.18	14.76	14.94	14.14	10.85	7.9
GAIF01025338.1	123.78	101.51	59.88	147.31	143.81	220.8
GAIF01025339.1	36.78	24.88	28	0	0	0
GAIF01025340.1	0.2	0	0.59	0	0.7	0.42
GAIF01025341.1	0	0	0	0	0.06	0
GAIF01025342.1	0	0	0	0	0	0.09
GAIF01025343.1	7.34	6.74	3.93	16.27	14.34	5.58
GAIF01025344.1	0	0	0	0.11	0	0
GAIF01025345.1	1329.39	236.68	625.31	1139.77	707.93	600.28
GAIF01025347.1	0.34	0	1.64	0.53	1.36	0
GAIF01025349.1	82.53	45.09	67.99	80.74	39.21	268.79
GAIF01025350.1	0.38	0.1	0.1	0.09	2.87	0.1
GAIF01025351.1	0.8	0	0	0.46	0.08	0
GAIF01025352.1	10.26	13.93	4.21	55.26	69.07	1.8
GAIF01025353.1	0	0.15	0.04	0	0.23	0
GAIF01025354.1	0	0.12	0	0.1	0.36	0
GAIF01025355.1	0	0.04	0	0	0	0
GAIF01025356.1	0	0	0.17	0	0.52	0
GAIF01025357.1	0	0	0	0.25	1.04	0.12
GAIF01025358.1	0	0.06	0	1.21	7.86	0
GAIF01025359.1	0.07	0.4	0.08	1.5	4.76	0.56
GAIF01025360.1	45.18	38.89	26.29	286.74	271.57	39.97
GAIF01025361.1	21.95	16.34	19.47	34.7	23.81	72.66
GAIF01025362.1	0.33	0.61	0.47	0.15	0.4	0
GAIF01025363.1	5.82	1.61	0.5	46.07	103.27	1.75
GAIF01025364.1	0	0	0	0.11	0	0
GAIF01025365.1	16.32	14.12	15.3	20.81	14.76	7.95
GAIF01025366.1	8032.16	2553.07	1495.39	4367.19	4247.65	2384.03
GAIF01025368.1	0	0.5	0	0	0	0.51
GAIF01025369.1	0	0	0	0.06	0	0
GAIF01025370.1	0	0	0	0.1	0	0
GAIF01025371.1	0	0	0	0.08	0.08	0.04
GAIF01025372.1	0.92	1.61	1.52	1.78	1.45	0.94
GAIF01025375.1	0.31	0	0.17	0	2.04	0.17
GAIF01025376.1	0	0	0.14	0	0	0
GAIF01025377.1	0	0	0	0.1	0.1	0
GAIF01025378.1	35.88	31.98	20.1	50.9	50.43	17.22
GAIF01025379.1	221.53	303.57	131.8	20.82	22.77	22.74
GAIF01025380.1	0	0.79	0	3.11	8.48	0
GAIF01025381.1	1555.97	1020.14	676.48	1900.51	2382.78	1926.7
GAIF01025382.1	37.87	54.9	24.16	83.88	50.65	4.31

GAIF01025383.1	0	0	0.04	0.2	1.16	0.08
GAIF01025384.1	0.06	0.12	0	0.07	1.04	0
GAIF01025385.1	32.11	26.23	24.23	22.28	23.3	17.34
GAIF01025386.1	0.46	0.13	0.05	2.86	8.72	0.26
GAIF01025387.1	92.98	106.84	50.67	39.22	80.89	6.6
GAIF01025389.1	0.03	0.11	0.04	0.04	0.08	0
GAIF01025390.1	9.67	6.08	17.55	18.63	38.64	70.09
GAIF01025391.1	0.92	0.94	0	0.6	0	0.3
GAIF01025392.1	17.54	26.91	18.52	30.66	34.17	12.72
GAIF01025395.1	0	0.18	0	0.81	2.04	0
GAIF01025396.1	4.3	5.38	5.21	12.13	0	21.07
GAIF01025397.1	0.02	0.02	0	0.01	0	0
GAIF01025398.1	0.52	0	0.57	0.48	0.62	0.11
GAIF01025399.1	0.06	0.06	0	0.33	2.52	0
GAIF01025400.1	0	0	0	0.08	0	0
GAIF01025401.1	0.95	0	0.03	2.41	12.79	2.21
GAIF01025402.1	0.65	0	0.26	0.28	0.14	0
GAIF01025403.1	0.32	0	0	0.19	0	0
GAIF01025404.1	0	0	0	0	0.24	0
GAIF01025406.1	5.17	4.12	9.7	24.63	14.89	20.07
GAIF01025408.1	0.17	0.05	0	0.4	0.63	0
GAIF01025409.1	43.13	67.93	54.36	59.83	46	13.51
GAIF01025410.1	0	0	0.04	0	2	0
GAIF01025411.1	0	0	0	0	4.04	1.35
GAIF01025413.1	11.23	3.19	3.86	18.15	0	0.6
GAIF01025414.1	21.4	38.99	31.68	34.7	37.84	29.66
GAIF01025415.1	0.21	0.14	0.17	0.92	3.52	0.15
GAIF01025416.1	0.94	0.46	1.04	0.12	0	0.35
GAIF01025417.1	0.75	0	0.09	1.36	5.13	2.03
GAIF01025418.1	0	0.3	0	0	0	0.57
GAIF01025419.1	0	0	0	0	0.27	0
GAIF01025421.1	0	0	0	0	0.13	0
GAIF01025422.1	20.9	23.14	19.62	17.83	30.88	78.88
GAIF01025423.1	43.8	50.79	30.08	90.45	91.1	11.76
GAIF01025424.1	1.33	1.34	1.38	6.42	6.45	2.08
GAIF01025425.1	14.71	7.15	5.23	2.91	2.01	0
GAIF01025426.1	13.97	20.11	7.3	48.46	51.46	3.37
GAIF01025427.1	0.03	0	0	0	0.03	0
GAIF01025428.1	0	0	0.11	0.11	0.47	0.22
GAIF01025429.1	0	1.82	1.84	0	0	0
GAIF01025431.1	151.72	238.14	255.68	26.99	23.22	42.55
GAIF01025432.1	1.9	2.59	2.56	3.28	2.41	0.72
GAIF01025433.1	0.57	0	0	5.53	20.35	1.92
GAIF01025434.1	0	0	0	0.21	0	0

GAIF01025435.1	35.99	1.77	0.96	39.35	101.43	300.49
GAIF01025436.1	2.22	0.72	2.66	8.08	6.3	9.16
GAIF01025437.1	0	2.95	2.7	0	0	0.27
GAIF01025438.1	0	0	0	0.04	0.04	0
GAIF01025440.1	0	0	0	0	2.37	0
GAIF01025441.1	0	0	0	0.31	0.94	0.66
GAIF01025442.1	0	0	0	0	0.06	0
GAIF01025443.1	30.19	15.15	14.63	64.31	88.49	30.24
GAIF01025445.1	0.08	0.44	0	1.13	1.75	0.11
GAIF01025446.1	0	0	0	0.08	0.34	0
GAIF01025447.1	0.11	0.59	0.48	0.88	2.1	0
GAIF01025449.1	4471.65	12473.25	10042.73	4311.24	2417.63	1147.56
GAIF01025450.1	0	0	0	0.2	0	0
GAIF01025451.1	145.44	80.87	39.6	110.75	127.16	0.21
GAIF01025452.1	0.16	0.26	0	1.21	7.52	3.27
GAIF01025453.1	0.42	0	0.35	0.61	0.51	0.93
GAIF01025455.1	0.06	0.03	0	0.82	3.19	0.07
GAIF01025456.1	18.19	7.24	17.68	6.74	8.94	27.56
GAIF01025458.1	0.93	0.97	0.4	0.76	0	0
GAIF01025459.1	7.04	5.23	13.85	14.36	11.13	9.8
GAIF01025460.1	18.6	9.68	8.48	32.52	58.59	17.75
GAIF01025461.1	0.1	0	0	0	0	0.11
GAIF01025462.1	10.4	9.67	9.45	8.13	2.58	2.39
GAIF01025464.1	44.03	36.17	24.92	110.55	77.78	20.06
GAIF01025466.1	0	0	0	0	1.06	0
GAIF01025468.1	50.56	72.16	23.17	132.51	139.14	2.13
GAIF01025470.1	25.59	16.77	28.33	31.29	21.07	102.97
GAIF01025472.1	262.56	279.14	164.48	328.08	254.1	71.37
GAIF01025473.1	0	0	0	0.26	1.22	0
GAIF01025474.1	0	0.36	0.34	3.92	14.57	0
GAIF01025477.1	0.32	0	0	0.89	3.93	0.14
GAIF01025479.1	0.11	0.07	0.01	0	0.03	0
GAIF01025480.1	0.61	0.44	0.17	2.99	3.34	0.11
GAIF01025481.1	0.35	0.76	0.11	1.92	1.57	0.11
GAIF01025483.1	2.86	0.59	0.4	3.04	7.75	15.49
GAIF01025485.1	1.11	0.19	0.09	1.44	2.37	3.06
GAIF01025487.1	0	0	0.13	0	0.98	0
GAIF01025488.1	0	0.84	0.09	0	0	0.19
GAIF01025489.1	70.59	39.35	23.82	4.21	2.32	2.18
GAIF01025490.1	0.93	0.76	0	0.91	1.16	0
GAIF01025491.1	0.21	0.09	0	0.37	1.75	0
GAIF01025492.1	0	0.04	0	0	0.23	0
GAIF01025493.1	0.05	0.06	0	0.53	3.1	0.06
GAIF01025494.1	0	0.06	0	0.18	3.88	0

GAIF01025496.1	0.16	0	0.17	0.43	0.82	0
GAIF01025497.1	32.78	41.11	14.07	47.35	48.14	2.63
GAIF01025498.1	0	0	0	0	3.56	1.39
GAIF01025499.1	0.36	0.46	0.03	0.73	0.97	0.3
GAIF01025501.1	11	13.41	23.19	26.31	24.82	14.69
GAIF01025503.1	68.15	178.56	80.6	56.09	116.46	16.91
GAIF01025504.1	0.17	0.12	0.06	0.28	0.34	0
GAIF01025506.1	2.49	0	13.2	4.33	1.16	8.83
GAIF01025507.1	0.3	0.07	0	0	0	0.55
GAIF01025508.1	0	0	0	0.21	0.43	0.07
GAIF01025509.1	0	0	0	0.08	0.12	0
GAIF01025510.1	0	0.05	0	0	0.1	0
GAIF01025511.1	0.07	0.08	0	0	0.17	0
GAIF01025512.1	0	0	0	0.22	0	0
GAIF01025513.1	25.58	19.03	26.91	121.12	41.62	17.36
GAIF01025514.1	19.38	13.74	16.57	38.44	20.8	35.69
GAIF01025515.1	0	0.06	0	0	1.26	0
GAIF01025516.1	0.18	0	0	0.2	0.15	0.17
GAIF01025517.1	0.15	0.17	0	0.45	0.87	0
GAIF01025518.1	0.29	0.32	0.16	2.12	7.21	0.56
GAIF01025519.1	68.03	82.85	52.87	76.9	108.47	24.09
GAIF01025522.1	0.9	0	0	0	1.64	0.17
GAIF01025524.1	65.41	23.18	65.58	92.18	55.57	14.42
GAIF01025525.1	0.07	0.05	0	0.58	1.85	0.02
GAIF01025526.1	0.3	0	0	0	0.24	1.11
GAIF01025527.1	4.8	0.24	0	21.61	42.41	9.29
GAIF01025529.1	0.21	0.03	0.03	0.76	3.05	0.07
GAIF01025530.1	0	0	0	0.63	0.42	0.61
GAIF01025531.1	2.69	0.2	0.41	12.65	23.65	68.6
GAIF01025532.1	10.18	10.38	15.32	22.81	11.13	12.56
GAIF01025533.1	0.43	0.29	0.11	0.66	0.36	0
GAIF01025535.1	1.02	0.42	0.96	0	0.34	0
GAIF01025536.1	0	0.22	0.13	2.18	9.08	0.45
GAIF01025539.1	0.29	0.1	0	1.85	4.1	2.11
GAIF01025540.1	0.06	0.06	0	0.13	1.48	0
GAIF01025541.1	0.51	2.01	0	3.46	5.19	0.11
GAIF01025542.1	0	0.7	0.29	0	2.14	0.82
GAIF01025544.1	0.11	0	0	0	0	0.18
GAIF01025545.1	0.74	0.2	0	4.31	8.04	0.41
GAIF01025546.1	0	0.07	0	0.32	0.12	0
GAIF01025547.1	0.11	0.3	0.25	0	0.74	0
GAIF01025548.1	10.1	13.22	11.44	31.17	27.15	2.78
GAIF01025549.1	12.33	16.38	5.06	34.21	49.67	4.43
GAIF01025550.1	0.17	0.06	0	0	0	0

GAIF01025553.1	0	0.1	0	0.11	1.02	0
GAIF01025554.1	0	0	0	0.06	0.07	0
GAIF01025555.1	0	0.08	0.08	0.42	0.78	0
GAIF01025556.1	17.6	14.21	25.59	31.75	31.96	35.36
GAIF01025557.1	0	0	0	0	0.21	0
GAIF01025558.1	0	0	0	0.99	0.38	0.23
GAIF01025559.1	4.15	5.68	1.98	7.17	11.41	0.72
GAIF01025561.1	185.52	153.29	131.04	296.59	159.11	91.88
GAIF01025562.1	0.12	0.07	0	0.57	5.49	0
GAIF01025563.1	1.12	0.69	0.31	5.4	23.02	2.22
GAIF01025564.1	3.04	0	3.6	6.49	36.05	6.69
GAIF01025565.1	17.04	16.16	6.75	61.47	62.5	3.61
GAIF01025566.1	7.03	4.14	4.1	25.74	26.85	7.58
GAIF01025567.1	0.56	0.23	0.06	1.95	5.67	0.18
GAIF01025568.1	15.01	14.79	5.09	15.56	43.82	1.52
GAIF01025569.1	0	0	0	0	0.06	0
GAIF01025570.1	0.35	0	0	0	2.09	0.58
GAIF01025571.1	3.44	0.92	0.29	19.51	45.42	2.17
GAIF01025573.1	7.76	6.76	5.7	10.02	11.13	11.8
GAIF01025575.1	34.73	46.13	23.11	26.64	53.32	34.3
GAIF01025578.1	0	0	0.05	0	0.87	0
GAIF01025579.1	3.28	4.56	0.6	5.29	7.67	0.6
GAIF01025581.1	0	0	0	0	0.69	0
GAIF01025582.1	0.65	0	0.24	1.5	8.86	0.41
GAIF01025583.1	103.05	29.57	20.63	15.64	12.67	9.86
GAIF01025585.1	0.68	0.8	0.7	6.13	9.14	1.94
GAIF01025586.1	0	0.24	0	0.77	2.41	0.25
GAIF01025587.1	2.83	2.31	0	3.12	7.83	0.94
GAIF01025588.1	6.86	5.91	6.42	15.81	12.92	3.88
GAIF01025589.1	16.95	13.03	13.01	12.13	4.29	3.09
GAIF01025590.1	74.75	18.34	19.33	80.26	74.29	79.35
GAIF01025591.1	1.49	0.27	0.09	4.94	8.55	0
GAIF01025592.1	267.56	418.01	28.5	354.22	633.2	45.25
GAIF01025594.1	0	0	0	13.11	0	0
GAIF01025595.1	21.84	32.01	27.21	3.27	1.03	1.49
GAIF01025596.1	0.05	0.08	0	0.84	0.77	0.19
GAIF01025597.1	7.6	8.29	4.07	22.88	39.94	3.34
GAIF01025598.1	0.89	1.77	0.13	3.4	4.87	0
GAIF01025599.1	0	0	0.1	0	0.11	0
GAIF01025600.1	0.62	0	0.27	4.03	0.75	2.46
GAIF01025601.1	13.14	13.38	5.08	35.62	45.07	1.6
GAIF01025602.1	1.35	1.37	0.99	5.19	4.89	0.8
GAIF01025603.1	0.6	2.16	0	9.43	28.44	0
GAIF01025604.1	0	1.03	1.22	0.27	1.72	0

GAIF01025606.1	1.12	1.01	0.27	0.97	0.26	0.27
GAIF01025607.1	8.73	17.23	10.56	10.23	10.01	2.27
GAIF01025609.1	0.32	0.31	0.34	0.75	2.88	0.16
GAIF01025610.1	8.89	11.28	12.09	7.13	4.46	2.1
GAIF01025611.1	11.33	11.45	9.39	19.43	14.07	4.27
GAIF01025613.1	822.03	1008.62	762.7	369.78	294.56	194.67
GAIF01025614.1	0.05	0	0	0.24	1.12	0
GAIF01025616.1	7.9	5.72	2.84	14.32	14.19	1.56
GAIF01025617.1	82.45	47.4	23.14	110.49	90.42	104.66
GAIF01025618.1	0	0	0	0	0.13	0
GAIF01025619.1	0.73	1.09	0.96	3.92	1.81	0.05
GAIF01025621.1	0.55	0	0.14	8.08	1.85	2.65
GAIF01025623.1	61.13	96.48	278.66	81.6	62.39	164.64
GAIF01025624.1	15.78	10.61	12.69	3.77	1.01	0.66
GAIF01025627.1	9.61	8.46	8.94	6.69	4.86	3.79
GAIF01025628.1	0	0.06	0	0.39	2.89	0.12
GAIF01025629.1	55.15	36.23	37.21	53.89	94.62	65.44
GAIF01025630.1	15.24	17.28	20.76	16.08	9.8	4.94
GAIF01025632.1	0	0.26	0	0	0.15	0
GAIF01025634.1	0.94	0.27	0	3.62	2.55	0.34
GAIF01025636.1	4.81	7.03	7.55	14.04	11.74	3.64
GAIF01025637.1	4602.61	5069.76	7486.76	2578.36	2038.67	8683.24
GAIF01025638.1	0	0	0	0	0.16	0
GAIF01025639.1	8.65	0	0.35	4.76	0.19	0.87
GAIF01025641.1	9.54	9.03	16.57	48.39	23.49	37.02
GAIF01025642.1	0.08	0.83	0.13	0.79	1.59	0.18
GAIF01025643.1	1.29	0.23	0.09	2.7	7.59	8.78
GAIF01025645.1	0.15	0.36	0.13	0.53	0.51	0.02
GAIF01025647.1	4.66	3.1	0	7.65	7.99	0
GAIF01025648.1	0	0	0.07	0.04	0.05	0
GAIF01025649.1	8.04	17.43	10.67	42.63	17.86	5.04
GAIF01025650.1	249.2	136.5	163.43	8.69	5.79	21.77
GAIF01025651.1	0.14	0.16	0.13	0.03	0.03	0.03
GAIF01025652.1	0.03	0.21	0.07	0.11	0.08	0
GAIF01025654.1	0.57	0.35	0	1.1	0.75	0.97
GAIF01025655.1	2.01	1.75	3.16	6.98	7.62	3.64
GAIF01025657.1	5.07	13.66	7.38	19.97	18.42	0.5
GAIF01025658.1	0	2.39	1.12	0	0.05	0.27
GAIF01025660.1	0	0	0	0.03	0	0
GAIF01025661.1	1.15	2.28	0.42	1.05	1.21	0
GAIF01025662.1	0.62	0.55	0.23	4.53	8.59	0.11
GAIF01025664.1	1.21	4.07	1.33	10.99	9.66	0.49
GAIF01025665.1	30.81	23.1	77.72	15.2	6.24	33.07
GAIF01025666.1	0.25	0.5	0.02	1.5	3.11	0

GAIF01025667.1	0	0	0.02	0.01	0.02	0
GAIF01025668.1	0.69	0.25	0	0.62	0.63	2.26
GAIF01025670.1	84.62	101.79	33.49	189.55	160.04	65.33
GAIF01025672.1	41.1	26.35	35.63	1.29	0.67	0.31
GAIF01025674.1	0.11	0.24	0	0	0	0
GAIF01025676.1	43.39	41.09	52.61	40.81	53.38	62.57
GAIF01025677.1	0	0.15	0	0.17	1.46	0
GAIF01025680.1	3.02	4.95	1.06	7.46	10.95	0.06
GAIF01025681.1	0.07	0	0.08	0.43	0.09	0
GAIF01025682.1	141.55	194.72	204.81	248.56	259.15	205.76
GAIF01025683.1	0	0	0.09	0	0.18	0
GAIF01025685.1	0.74	0.59	0	0.75	2.06	0
GAIF01025687.1	0.13	0	0	0.31	0.97	0
GAIF01025689.1	8.96	14.62	4.78	7.69	10.57	18.68
GAIF01025691.1	2.31	0.81	0	7.95	27.29	2
GAIF01025692.1	0.27	0	0	0	0.48	0
GAIF01025694.1	1.63	0.14	0.14	8.75	33.54	0.42
GAIF01025695.1	2.09	1.57	0.46	11.91	20.9	7.07
GAIF01025696.1	0.6	0.84	1.42	1.4	0.42	0.2
GAIF01025697.1	152.59	129.95	148.66	124.38	57.7	38.64
GAIF01025698.1	0	0	0	0	0.01	0
GAIF01025701.1	0.29	0.42	0.14	4.5	2.73	0.13
GAIF01025702.1	0.24	0	0	1.11	2.89	0.2
GAIF01025703.1	0.85	0.09	0.18	0.23	0.5	0.34
GAIF01025704.1	0	0	0	0	0.16	0
GAIF01025705.1	0	0.13	0	0.28	0.5	0.07
GAIF01025706.1	0	0.08	0.08	2.37	15.06	0.24
GAIF01025707.1	48.9	45.39	96.83	39.85	42.65	119.61
GAIF01025708.1	0	0.26	0	0	0	0
GAIF01025709.1	0	0.11	0	0.12	0	0
GAIF01025710.1	0.24	0.05	0.05	1.47	7.74	0.86
GAIF01025711.1	0	0	0	0	0.42	0
GAIF01025712.1	0.5	0.12	0.26	3.71	7.91	0.64
GAIF01025713.1	0.12	0.14	0	0	0	0
GAIF01025714.1	1.88	1.87	0.34	4.72	10.53	1.72
GAIF01025715.1	18.41	19.3	24	18.2	16.55	8.43
GAIF01025716.1	64.22	69.69	23.05	310.94	497.01	6.72
GAIF01025717.1	0	0	0.12	0.11	0.07	0.06
GAIF01025718.1	0	0	0	0	0.09	0
GAIF01025719.1	0.07	0.08	0	0	0.09	0.08
GAIF01025721.1	1.3	1.3	2.15	0.13	0.26	0
GAIF01025722.1	0	0	0	0	1.63	0
GAIF01025723.1	0.11	0	0	0.13	1.17	0
GAIF01025724.1	1.41	0	0	0.88	1.56	1.33

GAIF01025725.1	0	0	0	0.87	1.59	0
GAIF01025727.1	69.75	42.83	34.78	243.18	113.52	53.76
GAIF01025729.1	0.82	0.41	0.72	1.19	0.36	0.27
GAIF01025730.1	0	0	0	0.29	0.65	0.19
GAIF01025732.1	0	0	0	0.41	0.46	0
GAIF01025733.1	1.33	1.21	1.8	3.35	2.42	0.74
GAIF01025735.1	0	0.33	0	0.26	0.85	0
GAIF01025736.1	1.33	2.67	0.98	6.95	15.39	1.04
GAIF01025737.1	18	13.56	7.3	66.78	51.27	5.64
GAIF01025738.1	1.03	0	0	0.18	0	0
GAIF01025739.1	263.08	113.49	143.98	172.96	148.27	507.12
GAIF01025740.1	1.24	0.86	0.46	0.72	0.5	1.34
GAIF01025741.1	0.26	0.83	0.7	0.3	0.46	0.28
GAIF01025742.1	0.04	0.05	0	0	0.42	0
GAIF01025743.1	16.48	7.01	12.19	34.72	24.45	80.85
GAIF01025744.1	3.66	4.96	0	39.49	61.63	0.53
GAIF01025745.1	55.7	92.88	101.74	65.04	49.56	78.67
GAIF01025746.1	8.59	6.63	5.86	8.67	3.12	2.84
GAIF01025747.1	18.75	21.96	6.33	78.8	109.8	4.81
GAIF01025748.1	0	0	0	0.1	0	0
GAIF01025750.1	0	0	0	0	0.17	0.03
GAIF01025752.1	29.8	25.86	19.91	28.11	28.6	4.69
GAIF01025753.1	0.47	0.11	0	0.3	0.26	0.06
GAIF01025755.1	17.75	25.93	61.93	24.67	11.07	19.12
GAIF01025758.1	27.01	19.33	17.59	32.57	32.63	13.66
GAIF01025759.1	656.48	755.36	1060.78	139.76	76.05	49.11
GAIF01025761.1	0	0	0	0.67	0	0
GAIF01025764.1	0.1	0.2	0	0	0.34	0.21
GAIF01025765.1	0.05	0	0	0	0	0
GAIF01025766.1	0.11	0.36	0.61	0.13	0.13	0
GAIF01025768.1	0.15	0.5	0.17	0.72	0	0.17
GAIF01025769.1	0	0	0	0	0.06	0.12
GAIF01025773.1	0	0.08	0.11	0.03	0	0
GAIF01025774.1	0	0	0	0	1.02	0
GAIF01025775.1	0	0.12	0	0.95	1.32	0.06
GAIF01025776.1	6.9	7.67	10.49	14.06	13.83	8.53
GAIF01025777.1	29.99	41.41	19.59	35.24	28.46	8.51
GAIF01025781.1	0	0	0.17	0.9	2.43	0
GAIF01025782.1	0	0	0	0	0.24	0
GAIF01025783.1	5.2	3.72	2.55	19.84	38.49	4.52
GAIF01025784.1	0	1.77	1.1	0	0.07	0.76
GAIF01025785.1	1.83	1.18	0.57	1.47	3.9	0.26
GAIF01025786.1	1.23	2.52	0.45	0.47	0.82	0.08
GAIF01025787.1	0	0	0	0.26	1.8	0.06

GAIF01025789.1	0	0	0	0.07	0	0
GAIF01025790.1	0	0	0	0.09	0.36	0.08
GAIF01025791.1	0.8	0.27	0.47	0.5	0.37	0.95
GAIF01025794.1	0	0	0	0	0.01	0
GAIF01025795.1	0.5	0.34	0.34	0.14	0	0.21
GAIF01025797.1	0.72	0.68	0	0.81	1.79	1.59
GAIF01025798.1	6.66	0.55	0.61	16.53	0.34	0.31
GAIF01025800.1	0	0	0.02	0	0	0.05
GAIF01025802.1	123.89	68.79	67.61	34.08	0	0
GAIF01025803.1	0	0	0	0.27	0.19	0.09
GAIF01025804.1	0.8	0.12	0	4.55	17.35	0.63
GAIF01025806.1	0.03	0.21	0.05	0.97	8.63	0.07
GAIF01025808.1	0	0	0	0	0	0.02
GAIF01025809.1	0	0	0	0.13	0	0
GAIF01025811.1	1.39	1.37	0	5.49	17.64	0.73
GAIF01025812.1	15.01	7.02	8.97	21.74	21.22	29.6
GAIF01025813.1	4.55	3.64	2.69	1.95	1.16	0.68
GAIF01025814.1	0	0	0	0.09	0	0
GAIF01025815.1	1.02	0	0.03	0.12	0.71	0
GAIF01025817.1	0	0	0	0	0.62	0
GAIF01025818.1	0	0	0	0	0.49	0
GAIF01025819.1	3.25	1.3	0.97	0.45	0.32	0.18
GAIF01025820.1	4410.28	17042.3	21053.5	4534.51	3980.4	2330.89
GAIF01025821.1	0.45	0.22	0.67	0.64	0.64	0.78
GAIF01025823.1	0	0.08	0.05	0.17	0.16	0
GAIF01025824.1	0.05	0.05	0.05	0.9	3.73	0.8
GAIF01025825.1	0	0	0	0.3	1.63	0.03
GAIF01025826.1	7.51	2.14	14.28	0.19	0.39	0.72
GAIF01025827.1	1.2	0.19	0.1	1.14	3.22	0.44
GAIF01025829.1	0	0	0	0.46	0.5	0
GAIF01025830.1	0	0	0	0	0.16	0
GAIF01025831.1	1.72	1.56	0.47	1.4	0.59	0.03
GAIF01025832.1	4.15	0	0	2.92	0	0
GAIF01025833.1	21.12	17.24	25.03	71.11	39.94	109.73
GAIF01025834.1	0	0.27	0.32	0	0	0
GAIF01025836.1	646.33	583.52	680.99	311.73	236.49	320.5
GAIF01025839.1	0.1	0.11	0	0.57	2.72	0
GAIF01025840.1	16.87	5.8	12.04	26.68	28.51	66.88
GAIF01025844.1	1.24	0.45	0.58	6.16	7.44	1.38
GAIF01025845.1	0	0	0.07	0	0	0
GAIF01025846.1	3.71	27.72	22.19	0.02	17.81	0
GAIF01025847.1	0	0.04	0	0	0	0
GAIF01025848.1	0	0.25	0	2.12	6.47	1.89
GAIF01025850.1	0.61	1.34	0	0.36	0.37	0

GAIF01025851.1	0.06	0.07	0.1	0.04	0	0
GAIF01025852.1	11.8	10.24	7.34	29.54	37.1	3.53
GAIF01025853.1	0	0	0	0.1	1.78	0.19
GAIF01025854.1	0.12	0	0	0	0.26	0
GAIF01025856.1	0	0.12	0.09	0	0	0
GAIF01025859.1	0	1.18	1.88	0	0	0
GAIF01025860.1	0	0	0	0.16	0.98	0
GAIF01025861.1	14.11	13.7	14.48	36.72	43.92	11.8
GAIF01025862.1	8.33	12.35	4.68	22.79	24.63	4.76
GAIF01025863.1	8.54	13.03	11	5.28	0	1.35
GAIF01025864.1	0	0	0	0.4	1.08	0
GAIF01025865.1	36.05	9.38	18.22	107.78	31.5	6.7
GAIF01025866.1	1.35	0.13	0	0.51	8.48	6.33
GAIF01025869.1	0	0	0	0.83	0.58	1.66
GAIF01025870.1	0.13	0	0	0.15	0.15	0
GAIF01025871.1	52.62	21.95	4.23	67.78	85.23	10.5
GAIF01025872.1	0	0.04	0.04	1.21	0.88	0.04
GAIF01025873.1	0.31	0.45	0.11	0.48	0.13	0
GAIF01025875.1	20.51	13.11	16.28	22.77	11.87	14.59
GAIF01025876.1	0	0.07	0.1	0.14	0.5	0.17
GAIF01025877.1	0	0	0	0	0.07	0
GAIF01025878.1	0.09	0.03	0	0.26	2.61	0
GAIF01025880.1	12.51	15.52	12.18	32.18	13.2	5.07
GAIF01025882.1	0.04	0.04	0.04	0.27	1.27	0.28
GAIF01025883.1	23.2	25.38	35.08	38.6	20.88	40.2
GAIF01025885.1	47.6	62.98	70.37	122.76	79.81	38.58
GAIF01025886.1	0.28	0.18	0.03	2.13	9.36	0.12
GAIF01025887.1	0.53	0	0.32	0	0	0
GAIF01025888.1	0.57	0.62	0	4.07	19.97	0.69
GAIF01025889.1	0.18	0.05	0	1.43	5.96	0
GAIF01025890.1	18.08	14.02	15.12	51	45.11	19.19
GAIF01025891.1	0.79	0.88	0.77	0.3	0.87	0.2
GAIF01025892.1	2.7	1.32	4.07	1.17	0.24	2.16
GAIF01025893.1	18.88	20	23.3	47.94	36.35	24.05
GAIF01025894.1	0.12	0.11	0.04	0.85	3.24	0.09
GAIF01025895.1	0.03	0.04	0.04	0.61	1.84	1.31
GAIF01025898.1	0.04	0.02	0	0.41	0.22	0.24
GAIF01025899.1	0.04	0.09	0	0	0.05	0
GAIF01025901.1	0.11	0	0	0	0	0
GAIF01025902.1	41.21	48.86	50.04	71.23	33.51	14.01
GAIF01025903.1	0.66	1.23	0.67	2.73	7.87	0
GAIF01025904.1	0	0	0	0	0.08	0
GAIF01025905.1	2.22	1.01	0.13	6.36	16.7	13.89
GAIF01025906.1	24.74	37.41	13.26	28.2	16.17	0.97

GAIF01025907.1	1.24	0.71	0.58	8.72	29.17	0.39
GAIF01025908.1	10.56	14	3.49	22.5	44.02	40.82
GAIF01025909.1	38.46	26.86	57.15	114.3	50.01	187.96
GAIF01025911.1	167.95	121.28	144.9	64.55	20.8	33.33
GAIF01025912.1	0	0.26	0	0.84	2.04	0
GAIF01025913.1	0	0	0	0	0.15	0
GAIF01025914.1	1.6	0.69	0.88	3.21	0.92	0.37
GAIF01025915.1	0.04	0.02	0	0.04	0.04	0
GAIF01025916.1	0	0	0.1	0.1	0.16	0
GAIF01025917.1	0.2	0.08	0	0	0	0.12
GAIF01025918.1	15.08	24.53	9.05	27.8	26.72	2.66
GAIF01025919.1	10.06	11.25	10.94	2.09	0.34	0.63
GAIF01025921.1	0	0.31	0	0	1.86	0
GAIF01025922.1	14.14	10.62	6.9	34.92	14.68	8.53
GAIF01025924.1	0	0	0.21	0.15	1.94	0.07
GAIF01025926.1	0	0	0	0.04	0.13	0.04
GAIF01025927.1	1.12	1.22	1.43	5.05	8.77	0.12
GAIF01025929.1	16.71	19.58	14.91	45.07	18.44	5.04
GAIF01025930.1	0.33	0.36	0	0.46	0.24	0
GAIF01025933.1	1.6	0.43	0.27	2.67	3.42	1.27
GAIF01025934.1	0.4	0.2	0.2	0.07	0.18	0
GAIF01025935.1	1.68	0	0	7.41	13.1	1.21
GAIF01025937.1	0.07	0.07	0	0.16	1.36	0
GAIF01025940.1	0	0	0.12	0.37	0	0
GAIF01025941.1	0.1	0.13	0.09	0.03	0.09	0.03
GAIF01025942.1	7.99	1.62	4.16	7.74	4.28	1.99
GAIF01025943.1	0.09	0.05	0	0	0	0
GAIF01025944.1	175.69	221.5	215.64	141.51	98.61	83.64
GAIF01025946.1	0.62	0.91	0.85	0.81	1.73	0.18
GAIF01025947.1	1.09	0.59	0.76	0.85	2.81	0.46
GAIF01025948.1	0.46	0	0.06	0.06	0.16	0.03
GAIF01025951.1	0	0	0	0.2	0.2	0
GAIF01025952.1	20.42	24.26	11.45	75.5	65.41	4.5
GAIF01025953.1	5.93	5.88	3.95	35.06	25.15	5.09
GAIF01025954.1	0	0	0	0.14	0.57	0
GAIF01025956.1	0	0	0	0.03	0	0
GAIF01025957.1	9.17	6.37	7.57	1.57	3.26	1.14
GAIF01025958.1	14.4	21.12	15.4	50.53	58.93	7.78
GAIF01025960.1	5.85	6.93	6.49	2.02	1.25	0.51
GAIF01025961.1	5054.85	5670.63	7570.49	3049.75	2439.7	4646.57
GAIF01025962.1	0	0.11	0	0	2.23	0
GAIF01025963.1	0.65	1.16	0.69	1.56	1.79	0.21
GAIF01025965.1	0	0.49	0	0.83	0.21	0
GAIF01025966.1	0.05	0.02	0	0.09	0	0.05

GAIF01025967.1	0.08	0.17	0.09	0	0.82	0
GAIF01025968.1	0	0.07	0	0.08	0.54	0
GAIF01025969.1	0	0	0	0	0.05	0
GAIF01025970.1	0.05	0	0	0.06	0.71	0
GAIF01025971.1	8.81	7.01	5.16	5.82	3.69	1.14
GAIF01025973.1	0	0	0.03	0.18	0.42	0.09
GAIF01025975.1	0	0.1	0	0.33	0.73	0
GAIF01025977.1	0.11	0.24	0	0.13	1.22	0.12
GAIF01025978.1	0.25	0.25	0.18	1.79	8.18	2.16
GAIF01025979.1	0.44	0.58	0	2.47	4.9	0.1
GAIF01025981.1	0	0	0	0	0.03	0.03
GAIF01025982.1	0.6	0.35	0.18	0.25	0.85	0.3
GAIF01025983.1	0.19	0.06	0.03	0.9	1.16	0
GAIF01025984.1	6.36	10.01	8.18	48.55	21.97	9.98
GAIF01025985.1	4.52	3.26	2.32	2.03	0.77	0
GAIF01025987.1	0.09	0.14	0.18	0.38	0.24	0.19
GAIF01025991.1	0	0	0	0	0.04	0.04
GAIF01025994.1	0.28	0.15	0.04	0	0	0.19
GAIF01025997.1	0	8.28	4.16	5.08	9.22	1.02
GAIF01025998.1	0	0	0	0.08	0.37	0
GAIF01026000.1	0	0	0	0	0.29	0
GAIF01026002.1	0.71	1.25	1.9	1.03	3.51	1.36
GAIF01026003.1	30.06	18.69	21.9	34.96	28.19	7.47
GAIF01026004.1	0.4	0.14	0	0.7	0.69	0.6
GAIF01026007.1	0	0.01	0	0	0	0
GAIF01026008.1	0	0	0	0	0.5	0
GAIF01026009.1	0	0.24	0.24	0	0	0.12
GAIF01026011.1	0	0.07	0	0	0.04	0
GAIF01026012.1	54.41	43.79	104.23	160.93	157.43	588.86
GAIF01026013.1	0.1	16.18	16.68	0	0.5	1.37
GAIF01026014.1	0	0	0	0.1	0.63	0
GAIF01026015.1	0.54	0.47	0.35	1.62	1.42	0.24
GAIF01026017.1	7.62	7.56	6.23	8.61	7.57	3.89
GAIF01026018.1	0.44	0	0	0.85	2.83	0.32
GAIF01026019.1	0	0.08	0	0.18	1.19	0
GAIF01026020.1	0	0	0	0.2	0.2	0
GAIF01026021.1	0	0	0	0	0.21	0
GAIF01026022.1	0.79	0.86	1.67	3.09	5.38	2.62
GAIF01026023.1	3.75	4.63	8.03	7.5	2.96	4.26
GAIF01026024.1	0.51	0.55	1.11	1.17	1.42	0.19
GAIF01026025.1	0.64	0.43	0.7	0.11	0.06	0.05
GAIF01026027.1	0	0.16	0	0.09	0.27	0
GAIF01026028.1	0.54	0.07	0	0.35	0.44	0.13
GAIF01026029.1	0	0	0	0.08	0	0

GAIF01026031.1	4.77	6.18	2.45	24.48	17.9	1.42
GAIF01026032.1	11.22	13.27	17.91	14.53	2.07	7.61
GAIF01026033.1	2.31	0	0	0.67	0	0
GAIF01026034.1	0	0	0	0.44	0.95	0
GAIF01026035.1	0	0	0.18	0	0	0
GAIF01026037.1	0.53	0.38	0	0	0.84	0.69
GAIF01026038.1	0.3	0	0	0.17	0.54	0
GAIF01026039.1	1.25	2.03	0.46	0.36	0.62	0.34
GAIF01026040.1	45.83	58.73	39.81	107.9	87.36	11.99
GAIF01026041.1	0	0	0	0.1	0.17	0
GAIF01026042.1	5.59	11.46	2.8	7.6	4.22	0
GAIF01026043.1	0.03	0	0	0	0	0
GAIF01026044.1	0.18	0	0	0.21	1.1	0
GAIF01026045.1	0	0.2	0	0	0	0
GAIF01026046.1	0	0	0	0	0.15	0.14
GAIF01026047.1	0	0.2	0	0	0	0
GAIF01026048.1	21.38	42.09	22.47	69.3	90.1	13.78
GAIF01026049.1	17.39	21.89	35.86	43.67	14.11	23.82
GAIF01026050.1	8.87	18.33	7.69	45.77	51.73	2.05
GAIF01026051.1	1.35	0.07	0	2.82	15.17	5.43
GAIF01026052.1	4.3	1.52	0	6.37	17.6	3.18
GAIF01026053.1	0.33	0	0.18	0	0	0
GAIF01026054.1	0.09	0.05	0.05	1.5	4.62	0.1
GAIF01026056.1	1722.87	2314.97	2239.59	1051.91	776.99	825.13
GAIF01026057.1	0	0	0	0.44	2.12	0
GAIF01026058.1	0	0.09	0	0	0	0
GAIF01026059.1	0	0.14	0	0	0	0
GAIF01026061.1	0.11	0.12	0	0.93	6.76	0.38
GAIF01026062.1	16.57	9.97	15.3	81.25	41.45	20.7
GAIF01026063.1	0.2	0.41	0.63	0.15	0.31	0.26
GAIF01026064.1	1.26	0.85	0.17	5.2	5.58	0.95
GAIF01026065.1	297.71	233.29	800.44	73.19	46.08	483.52
GAIF01026067.1	19.34	53.92	39.57	52.83	58.6	45.79
GAIF01026068.1	0.13	0.37	0.11	0.33	0.14	0.21
GAIF01026069.1	0	0.07	0	0	0	0
GAIF01026070.1	0	0	0	0.43	2.33	0
GAIF01026071.1	0.51	0.55	0	0.3	0.46	0
GAIF01026072.1	0	0.34	0.09	0	0	0
GAIF01026073.1	0	0	0	0.2	0.11	0
GAIF01026075.1	0.34	0.15	0.07	1.4	4.28	0.52
GAIF01026080.1	0	0	0	0	0.17	0
GAIF01026082.1	10.29	9.74	9.08	21.93	26.26	4.58
GAIF01026085.1	0	0	0	0.13	0	0
GAIF01026086.1	0	0	0.07	0.07	0.18	0

GAIF01026090.1	0	0.05	0	0	0	0
GAIF01026091.1	0.34	0.09	0.05	0.8	2.12	0.05
GAIF01026092.1	0	0	0	0.09	0.09	0
GAIF01026095.1	5.39	10.53	4.87	9.16	14.85	4.39
GAIF01026096.1	102.82	88.99	159.22	102.11	102.41	334.29
GAIF01026097.1	0	0.1	0	0.21	0.97	0
GAIF01026100.1	0	0	0.06	0	0	0
GAIF01026101.1	19.07	2.35	9.06	27.19	34.9	186.29
GAIF01026103.1	0.21	0.11	0	0	0.22	0
GAIF01026104.1	2.74	0.21	0.1	5.48	41.78	13.73
GAIF01026106.1	0.2	0.42	0.18	0.34	0.37	0.28
GAIF01026107.1	1.28	0.32	0.11	10.06	44.85	1.38
GAIF01026108.1	29.64	26.78	15.46	100.35	42.9	8.37
GAIF01026109.1	0.09	0	0	0	0	0
GAIF01026110.1	52.97	52.2	48.3	53.05	51.45	62.45
GAIF01026111.1	358.45	185.48	148.47	247.57	110.29	106.58
GAIF01026112.1	0	0	0	0.64	0	0.3
GAIF01026114.1	1162.84	631.55	511.27	751.43	819.1	771.18
GAIF01026115.1	0.19	0	0	0	0	0
GAIF01026116.1	0	0	0.09	0.08	0.58	0
GAIF01026118.1	785.79	313.63	683.11	380.93	135.75	331.96
GAIF01026119.1	0.28	0.1	0	0.86	1.89	0
GAIF01026120.1	0.12	0.16	0	0.11	0	0
GAIF01026121.1	13.41	17.06	9.91	14.24	15.87	1.9
GAIF01026122.1	60.78	25.84	29.81	86.15	62.77	60.72
GAIF01026123.1	1.93	4.29	2.86	8.16	10.19	0.64
GAIF01026124.1	0.34	0	0	0.66	2.44	0
GAIF01026125.1	0.46	0	0.13	0.79	0	0.13
GAIF01026126.1	0.17	0.09	0.28	0	0	0
GAIF01026127.1	0	0	0.08	0	0	0
GAIF01026129.1	0.5	0.18	0.37	0	0.2	0.18
GAIF01026131.1	0.15	0	0	1.04	3.34	0
GAIF01026134.1	14.24	3.7	0	86.16	51.56	2.51
GAIF01026135.1	27.3	33.3	19.16	99.46	47.71	11.37
GAIF01026136.1	0.48	0.69	0.17	0	0.38	0
GAIF01026137.1	0.46	0.43	0.07	3.51	10.68	0.29
GAIF01026141.1	0.36	0.4	0	0	0.71	0
GAIF01026142.1	0.05	0	0	0	0	0
GAIF01026143.1	0.27	0.3	0.2	0	0.11	0.1
GAIF01026144.1	29.66	18.91	16.88	45.89	24.44	10.49
GAIF01026145.1	0.43	0	0.16	0	0	0.09
GAIF01026146.1	32.16	70.28	16.7	35.65	59.12	1.59
GAIF01026147.1	12.54	18.09	22.94	35.73	16.53	10.63
GAIF01026148.1	0.13	0	0	0	0	0

GAIF01026149.1	0.2	0	0	0	0	0
GAIF01026150.1	0.15	0.09	0	0	0	0
GAIF01026151.1	0	0	0	0.54	1.68	0
GAIF01026152.1	4.24	7.21	0.61	3.42	8.3	0
GAIF01026155.1	0	0	0	0.08	0	0
GAIF01026156.1	1.24	0.84	0.56	2.49	5.36	0
GAIF01026157.1	3.81	4.56	3.07	6.49	6.42	1.4
GAIF01026158.1	0.13	0	0.43	0.76	2.6	0.14
GAIF01026159.1	0	0	0	0	0.1	0
GAIF01026160.1	0.25	0	0.07	1.32	4.78	2.16
GAIF01026161.1	0	0	0	0	0.46	0
GAIF01026162.1	84.79	56.04	51.2	77.87	76.06	26.2
GAIF01026163.1	0	0.11	0	1.04	4.07	0.11
GAIF01026164.1	48.79	61.37	59.79	61.26	50.99	35.21
GAIF01026165.1	0	0.11	0	0	0	0.11
GAIF01026166.1	42.07	17.62	28.4	8.07	3.71	14.34
GAIF01026168.1	0.25	0	0	0	0	0
GAIF01026169.1	41.26	17.4	20.26	49.52	66.81	53.97
GAIF01026170.1	0.21	0	0	0	1.13	0
GAIF01026171.1	0.76	0	0	0	1.41	0
GAIF01026174.1	897.79	12.93	106.83	18.71	2.9	17.1
GAIF01026175.1	0	0	0	0.05	0.11	0
GAIF01026176.1	0.01	0	0	0.07	0.07	0
GAIF01026177.1	5.46	10.32	1.68	8.06	16.15	1.9
GAIF01026178.1	0	0.17	0	0.71	2.86	0.17
GAIF01026180.1	0.8	1.71	0.68	4.82	4.96	0.2
GAIF01026181.1	0.07	0.04	0.12	0.26	0.14	0.41
GAIF01026182.1	4.13	4.01	2.41	21.28	15.45	1.28
GAIF01026183.1	8.55	7.31	6.24	17.33	24.94	10.57
GAIF01026184.1	1.11	1.57	3.68	1.78	0	1.93
GAIF01026186.1	1.98	1.52	0.73	4.47	8.02	0
GAIF01026188.1	0	0	0	0	0.04	0
GAIF01026189.1	0	0	0	0.04	0.21	0
GAIF01026190.1	0	0	0	0.32	2.43	0
GAIF01026191.1	0.14	0	0	0	0	0.31
GAIF01026192.1	0.24	0.79	0.8	4.34	5.37	0.67
GAIF01026194.1	0	0	0	0.16	0	0
GAIF01026195.1	3.74	1.61	0.82	16.7	28.29	0.55
GAIF01026198.1	0.11	0	0	0	0.26	0
GAIF01026199.1	915.25	390.16	215.61	575.19	492.55	172.47
GAIF01026200.1	1.11	3.11	1.36	1.37	1.47	0.07
GAIF01026202.1	48.27	52.18	128.27	17.81	13.49	57.52
GAIF01026206.1	202.16	27.42	5.04	187.96	773.59	325.23
GAIF01026208.1	2.05	1.91	0.81	5.62	8.12	1.94

GAIF01026209.1	6.45	17.04	16.67	19.7	22.2	3.97
GAIF01026210.1	0	0	0	0.39	2.02	0.18
GAIF01026211.1	0	0.24	0	0.26	0.53	0
GAIF01026212.1	0	0.12	0	0	0.13	0.15
GAIF01026214.1	3.22	3.83	0	17.37	25.93	0.91
GAIF01026215.1	3.9	2.74	3.45	3.58	3.4	10.3
GAIF01026217.1	0	0.18	0	0.19	0.44	0
GAIF01026218.1	12.69	25.76	20.96	27.71	16.43	6.36
GAIF01026219.1	111.25	125.85	278.78	101.22	83.46	204.77
GAIF01026220.1	229.59	209.79	93.69	57.37	47.92	6.23
GAIF01026222.1	514.53	400.02	592.29	235.15	165.98	364.87
GAIF01026223.1	2.22	0.99	0.14	1.21	2.67	2.88
GAIF01026225.1	0.15	0	0	0.34	0.18	0.16
GAIF01026226.1	0	2.02	0	0	3.37	0
GAIF01026227.1	29.7	24.42	52.51	0	0.2	0.09
GAIF01026228.1	0	0	0.48	1.19	10.34	0
GAIF01026230.1	170.71	92.36	63.17	79.24	31.63	19.14
GAIF01026231.1	0.41	0	0	0.67	1.86	0
GAIF01026232.1	0	0	0	0.57	1.58	0
GAIF01026234.1	1.99	0	0	4.35	0	0
GAIF01026236.1	0.23	0.08	0.33	0	0.37	0
GAIF01026237.1	0	0.05	0	0.11	0.5	0
GAIF01026238.1	0.78	0.28	0.21	0.15	0.08	0
GAIF01026239.1	0	0.12	0	0.91	3.35	0.49
GAIF01026240.1	0.83	1.26	0	1.92	1.59	0
GAIF01026241.1	23.18	26.13	15.3	39.58	40.2	4.8
GAIF01026242.1	0.34	0.14	0	1.37	4.06	0.37
GAIF01026244.1	0	0	0	0	0.38	0
GAIF01026245.1	0	0	0.39	0.54	1.1	0.24
GAIF01026249.1	0	0	0	0	0.88	0
GAIF01026250.1	0	0	0	0.12	0	0
GAIF01026251.1	5.37	12.74	18.63	20.47	14.89	5.59
GAIF01026253.1	0.73	0.4	0	2.55	5.5	0.3
GAIF01026254.1	0	1	0	0.75	144.17	0
GAIF01026255.1	71.36	110.25	124.9	106.92	104.27	47.08
GAIF01026256.1	0	0	0	0	0.03	0
GAIF01026257.1	1.21	0.12	0.08	2.06	1.27	0.62
GAIF01026258.1	0	0.21	0	0	0	0
GAIF01026259.1	0.26	0.48	0.37	0.08	0.26	0.21
GAIF01026260.1	0.51	0.17	0	0	0.32	0.3
GAIF01026263.1	0	0	0	0.14	0.22	0
GAIF01026264.1	4.16	0.87	2.52	6.87	1.03	0.6
GAIF01026265.1	0	0	0	0	0	0.05
GAIF01026266.1	54.99	63.07	77.18	28.2	21.52	41.79

GAIF01026267.1	7.3	8.79	6.94	11.12	15.29	1.15
GAIF01026268.1	0.04	0	0.09	0.15	0.36	0
GAIF01026269.1	0.23	0	0.19	0.67	1.87	0.06
GAIF01026272.1	1.5	1.03	1.15	2.42	1.1	0.45
GAIF01026273.1	18.64	24.96	25.97	41.68	32.73	17.4
GAIF01026274.1	13.87	15.21	15.61	30.6	8.82	6.82
GAIF01026275.1	13.29	17.49	8.95	42.32	49.98	2.25
GAIF01026276.1	1.5	1.67	0.82	9.06	17.99	0.23
GAIF01026277.1	0.16	0	0	0	2.39	0
GAIF01026278.1	0	0	0	0.03	0	0
GAIF01026279.1	3.04	6.45	6.11	11.66	11.39	1.75
GAIF01026280.1	16.92	24.58	13.64	31.43	24.5	3.22
GAIF01026281.1	7.46	6.33	9.91	25.09	37.26	10.55
GAIF01026282.1	1.05	1.28	0	2.9	1.95	0
GAIF01026285.1	0.47	0.26	0.21	0	0	0
GAIF01026286.1	0.75	0.41	0.07	1.09	0.53	0.07
GAIF01026289.1	0	0.14	0	0.3	0.31	0.14
GAIF01026291.1	10.76	7.54	11.45	24.53	31.45	31.17
GAIF01026292.1	0.15	0.17	0	0.53	1.76	0.24
GAIF01026294.1	0	0.17	0.7	0	0	0.17
GAIF01026295.1	0.11	0	0	1.1	4.04	0
GAIF01026296.1	0	0	0	0	0.34	0
GAIF01026297.1	0	0.18	0.09	0	0	0
GAIF01026298.1	44.4	16.22	22.7	20.59	4.24	6.34
GAIF01026299.1	0.23	0.32	0	1.02	1.97	0
GAIF01026301.1	0.94	2.91	1.72	1.95	0.66	0.32
GAIF01026303.1	0.55	0.44	0	4.17	8.6	0.19
GAIF01026304.1	0	0.5	0.34	0	0	0
GAIF01026305.1	0.73	0	0	0.45	1.58	0.11
GAIF01026307.1	13.81	13.24	9.67	25.39	31.37	23.95
GAIF01026309.1	0	0	0	0	0.56	0
GAIF01026311.1	27.57	18.96	14.89	36.79	16.08	6.47
GAIF01026312.1	0	0.56	0.05	0	0	0
GAIF01026313.1	0	0.04	0	0	0.29	0
GAIF01026314.1	3.65	11.65	11.67	45.03	37.16	4.2
GAIF01026315.1	0	0	0.18	0	0.97	0
GAIF01026317.1	0	0	0	0.1	0	0
GAIF01026318.1	1642.04	1277.73	1399.4	6.21	4.81	4.94
GAIF01026319.1	0	0	0	0	0.15	0
GAIF01026322.1	0.82	0.18	0.72	0.57	0.59	1.45
GAIF01026325.1	64.75	36.43	4.82	124.23	186.71	41.32
GAIF01026326.1	0	0	0	0	0.17	0.05
GAIF01026328.1	0	2.25	3.55	5.14	10.82	2.72
GAIF01026330.1	0	5.81	4.32	0	0.08	0.46

GAIF01026331.1	124.48	55.08	37.21	226.7	216.47	69.78
GAIF01026333.1	0.1	0.11	0	0.24	1.96	0
GAIF01026334.1	0.18	0.09	0.23	0.27	0.29	0
GAIF01026335.1	0	0	0	0.09	0	0
GAIF01026336.1	0.14	0.22	0.07	0.95	1.56	0.08
GAIF01026337.1	0	0	0	0	0.07	0
GAIF01026340.1	0	0	0	0	1.02	0
GAIF01026342.1	0.49	0.18	0.09	1.09	6.84	0.23
GAIF01026343.1	0	0	0	0.06	0.06	0
GAIF01026344.1	0.14	0.04	0	0.09	0.06	0
GAIF01026345.1	0	0	0	0.56	4.27	0
GAIF01026346.1	0	0	0	0.16	0.11	0
GAIF01026347.1	0.08	0	0	0	0.03	0
GAIF01026348.1	170.48	88.65	25.77	215.04	159.28	27.82
GAIF01026349.1	0.1	0.11	0.65	0.23	1.78	4.79
GAIF01026350.1	5.56	9.79	10.06	9.12	10.63	1.98
GAIF01026351.1	21.75	16.15	19.68	48.87	51.79	11.61
GAIF01026352.1	0	0	0	0.17	0.11	0
GAIF01026353.1	0.32	0.52	0.35	0.3	0.23	0.04
GAIF01026354.1	0	0	0	0.32	0.99	0
GAIF01026355.1	1.3	0.14	0	3.31	24.32	0.29
GAIF01026356.1	9.84	5.35	0.86	32.56	49.5	0.6
GAIF01026357.1	0.48	1.54	0.03	4.75	11.44	0.13
GAIF01026358.1	1.25	0	0	0.34	0.63	0
GAIF01026359.1	0.59	0.38	0	3.82	7.91	0.65
GAIF01026360.1	0	0	0	0	1.16	0
GAIF01026361.1	1.6	0.75	1.23	5.36	1.47	1.05
GAIF01026362.1	0.16	0.08	0	0.38	0	0.12
GAIF01026363.1	8.35	3.71	5.62	30.82	26	2.93
GAIF01026364.1	0.04	0	0	0	0	0.03
GAIF01026365.1	17.5	11.67	13.74	28.47	20.46	12.27
GAIF01026366.1	0	0	0	0.14	0	0
GAIF01026369.1	28.61	38.29	54.3	115.95	86.79	29.13
GAIF01026370.1	0.11	0.37	0	0.78	0.54	0
GAIF01026371.1	68.23	41.74	21.11	60.99	47.85	39.75
GAIF01026372.1	2.34	1.47	2.65	2.29	0.72	0.61
GAIF01026373.1	1.64	0	0.16	4.77	40.52	16
GAIF01026374.1	0	0	0	0	1	0
GAIF01026375.1	0.13	0	0	0.3	0	0
GAIF01026376.1	0.68	0.83	0.83	1.76	1.59	0.28
GAIF01026378.1	0	0	0	0	4.82	0
GAIF01026379.1	0	0	0	0	0.12	0
GAIF01026381.1	2.38	0.76	1.08	6.01	11.28	0
GAIF01026382.1	0	0	0	0	0.11	0

GAIF01026383.1	0	0	0	0.12	0.25	0
GAIF01026384.1	0.56	0	0.15	1.12	9.05	0
GAIF01026385.1	0	0	1.05	1.11	2.3	0
GAIF01026387.1	0.03	0	0	0.03	0	0.03
GAIF01026388.1	837.34	359.55	331.85	233.87	192.83	977.23
GAIF01026389.1	0	0	0	0	0.25	0
GAIF01026391.1	0	0	0	0.07	0.17	0
GAIF01026392.1	0.16	0.09	0.12	0.28	0.23	0.12
GAIF01026393.1	24.96	27.64	19.68	39.72	36.95	7.95
GAIF01026394.1	0	0	0	0.18	0.13	0
GAIF01026395.1	0	0	0	0	0.15	0
GAIF01026396.1	10.12	5.07	6.23	0.08	0	0
GAIF01026397.1	0	0	0	0	0.05	0
GAIF01026398.1	56.74	46.14	50.21	39.81	29.9	9.05
GAIF01026399.1	0	0	0	0	0.17	0
GAIF01026400.1	0	0	0	0	0	0.14
GAIF01026401.1	15.77	23.12	23.34	32.14	19.15	8.04
GAIF01026402.1	4.14	6.27	1.83	1.32	0.47	0.15
GAIF01026403.1	0	0	0	0	0.1	0
GAIF01026404.1	0	0	0	0.77	3.5	0
GAIF01026405.1	0.11	0	0	0	0	0
GAIF01026406.1	0.06	0	0	0	0	0
GAIF01026408.1	0.05	0.11	0	0	0	0
GAIF01026409.1	5.66	3.31	3.81	9.19	3.74	4.68
GAIF01026410.1	0	0	0.41	0	0	0
GAIF01026411.1	7.6	6.86	10.17	20.47	8.03	8.61
GAIF01026413.1	0	0	0	0.03	0	0
GAIF01026416.1	0	0.09	0	0	0	0
GAIF01026419.1	0	0	0.13	0	0	0
GAIF01026421.1	2.81	2.31	1.56	25.8	59.22	5.58
GAIF01026422.1	0.14	0.28	0.8	0.27	0.1	0.25
GAIF01026423.1	9.59	10.05	6.48	11.23	32.88	7.94
GAIF01026425.1	1.35	0.62	0.27	0.67	1.23	0.72
GAIF01026427.1	0.13	0.14	0.14	0	0.75	0.23
GAIF01026428.1	0.13	0	0	0	0	0
GAIF01026429.1	1.07	1.49	0	6.54	10.44	0.67
GAIF01026431.1	2.86	6.95	8.97	0	0	3.34
GAIF01026432.1	0.2	0.07	0.07	0.24	1.22	0
GAIF01026433.1	0	0.12	0	0	0.12	0
GAIF01026434.1	0	0	0.26	0	0	0
GAIF01026435.1	0.01	0	0	0.41	0	0.31
GAIF01026436.1	7	6.17	6.06	6.56	1.76	1.68
GAIF01026438.1	14.48	10.75	11.8	24.17	16.9	10.38
GAIF01026439.1	505.52	182.64	173.65	567.14	504.75	2185.91

GAIF01026443.1	108.17	96.5	38.14	1.53	5.05	3.31
GAIF01026444.1	0	0	0	0	0.19	0
GAIF01026445.1	9.74	6.57	10.02	18.87	16.93	12.45
GAIF01026449.1	0	0	0	0.21	0	0
GAIF01026450.1	10.05	7.17	5.6	16.07	25	11.04
GAIF01026451.1	0.78	0.77	0.07	1.69	10.27	1.44
GAIF01026452.1	0.23	0	0.16	1.31	13.09	0
GAIF01026453.1	2.42	0.22	2	16.15	29.01	1.13
GAIF01026454.1	0.15	0.17	0	0.38	0.62	0.23
GAIF01026455.1	151.65	201.6	179.68	0.64	1.49	1.52
GAIF01026456.1	0	0.05	0	0.22	0.87	0.11
GAIF01026458.1	1.5	0.13	0	8.17	32.89	4.45
GAIF01026459.1	2.77	2.51	5.53	6.41	4.87	0
GAIF01026460.1	0	0.33	0.12	0	0.23	0
GAIF01026462.1	0	0.08	0.08	0.18	0.1	0.08
GAIF01026463.1	6.94	6.5	17.72	21.77	19	19.91
GAIF01026464.1	0	0	0	0.28	0.4	0.26
GAIF01026465.1	0	0.02	0.02	0.27	2.09	0.02
GAIF01026468.1	0.22	0.09	0	0.06	0.21	0.04
GAIF01026469.1	17.31	11.73	1.65	40.48	31.9	59.71
GAIF01026470.1	0.73	0.07	0.07	0.23	0	0.07
GAIF01026471.1	0.53	0.16	0.08	0.62	2.28	0
GAIF01026472.1	0.54	0.07	0.04	0.35	1.38	0
GAIF01026473.1	0.28	0.15	1.24	0	0.17	0.31
GAIF01026475.1	0.33	0.2	0.12	0.16	0.98	0
GAIF01026476.1	0	0	0	0	0	0.05
GAIF01026477.1	1.14	3.6	3.88	3.05	2.88	0.25
GAIF01026478.1	0.95	0.65	0	6.69	27.73	1.9
GAIF01026481.1	0	0	0	0	0.11	0
GAIF01026482.1	6.61	8.66	5.36	11.85	9.21	1.35
GAIF01026483.1	6.55	5.69	0.52	2.64	2.35	0
GAIF01026484.1	53.86	53.99	25.7	126.31	124.07	16.59
GAIF01026485.1	1.28	0.68	0.99	0.62	0.71	0.9
GAIF01026486.1	25.04	22.14	3.94	28.18	34.82	15.27
GAIF01026488.1	0.08	0	0.22	0.66	0.15	0.69
GAIF01026489.1	424.22	576.74	567.09	293.53	181.06	150.35
GAIF01026490.1	0	0.24	0	0	0.31	0
GAIF01026492.1	0	0	0	0.11	0.11	0
GAIF01026493.1	0	0	0	0	0.04	0
GAIF01026494.1	13.61	8.49	3.6	17.77	14.99	4.82
GAIF01026495.1	0.26	0.04	0.04	0.99	4.97	0.04
GAIF01026496.1	0	0	0	0.28	3.51	0.27
GAIF01026497.1	0	0	0.14	0	0	0
GAIF01026500.1	6876.39	6094.17	7738.02	2036.11	1410.29	4041.88

GAIF01026501.1	3.86	1.63	0.82	1.86	7.74	3.77
GAIF01026502.1	0	0.05	0	0.06	0	0
GAIF01026503.1	0	0	0.23	0	0.13	0
GAIF01026505.1	0	0	0	0.15	1.08	0
GAIF01026506.1	0	0	0	0.1	0.13	0
GAIF01026508.1	0.11	0.05	0	0.76	0.58	0
GAIF01026511.1	0.04	0.04	0	0.04	0	0
GAIF01026514.1	91.54	52.29	22.49	144.37	91.08	5.76
GAIF01026515.1	5090.11	16933.1	22379	6070.28	3654.15	2917.15
GAIF01026516.1	0.24	0.26	0.27	1.97	9.4	1.12
GAIF01026517.1	58.14	29.66	28.89	86.81	78.85	78.7
GAIF01026518.1	10.81	11.66	9.8	1.2	0.62	1.24
GAIF01026519.1	0.37	0.48	0.4	0	0.09	0
GAIF01026521.1	2.54	2.27	1.68	0.53	0.27	0
GAIF01026523.1	0	0	0	0	0	1.85
GAIF01026525.1	0.67	0.31	0.14	0.07	0.19	0
GAIF01026526.1	68.59	43.87	31	42.14	43.67	13.34
GAIF01026527.1	1.06	1.11	0.44	7.25	19.21	0.2
GAIF01026528.1	51.95	69.13	55.38	100.88	82.74	23.18
GAIF01026530.1	42.46	25.76	21.52	113.57	66.68	19.16
GAIF01026531.1	7.49	3.4	5.57	12.04	29.02	3.91
GAIF01026532.1	37.62	41.29	87.75	71.41	28.05	77.5
GAIF01026533.1	0	0.18	0.18	0	0.35	0
GAIF01026534.1	0.17	0	0	0.6	5.15	0.11
GAIF01026536.1	2.1	1.77	1.22	0.24	0.81	0.17
GAIF01026537.1	1.37	2	0.37	0.67	4.56	0.64
GAIF01026538.1	0.58	0.32	0.21	2.92	4.78	0
GAIF01026540.1	0.05	0	0	0.06	0.19	0
GAIF01026541.1	0.82	0.02	0.04	1.19	1.2	0
GAIF01026542.1	0.35	0.29	0	1.64	7.73	0.68
GAIF01026544.1	314.73	160.49	100.05	793.5	1407.12	1949.02
GAIF01026546.1	25.7	22.77	19.79	40.19	46.15	72.65
GAIF01026547.1	0.12	0.83	2.5	0.16	0.03	0.14
GAIF01026550.1	0	0	0	0	2.67	0
GAIF01026553.1	1.05	1.72	1.97	0.94	2.59	6.53
GAIF01026556.1	0	0	0	0.3	0.06	0
GAIF01026557.1	3.84	4.03	1.16	13.05	22.11	0.44
GAIF01026558.1	43.43	12.23	15.67	4.49	3.64	9.73
GAIF01026560.1	0	0	0	0	0.23	0
GAIF01026562.1	0.34	0.28	0	1.96	11.69	0.56
GAIF01026563.1	0	0.49	0	0.39	1.69	0
GAIF01026564.1	0.21	3.53	3.92	6.74	6.54	2.24
GAIF01026565.1	226.52	242.4	306.02	121.78	85.04	134.48
GAIF01026566.1	0	0.12	0	0.53	0	0

GAIF01026567.1	0.24	1.32	0.53	0.14	0	0.53
GAIF01026568.1	0	1	0	2.17	0	0
GAIF01026569.1	0.21	0.44	0.44	2.9	0.9	0.85
GAIF01026570.1	0.05	0	0	0.33	0.92	0
GAIF01026571.1	128.77	320.01	224.11	327.63	256.07	84.16
GAIF01026572.1	3.92	2.1	5.08	7.69	12.65	5.02
GAIF01026574.1	0	0	0	0	0.01	0
GAIF01026576.1	104.17	74.76	69.44	62.17	38.29	43.16
GAIF01026578.1	0	0	0	40.59	0	0
GAIF01026579.1	0.32	0.41	0.07	3.45	14.45	0.07
GAIF01026580.1	0.64	0.39	0.88	0.41	0	0.2
GAIF01026581.1	0.15	0	0.08	0.62	4.51	0.08
GAIF01026582.1	17.66	7.54	4.39	15.27	29.03	59.17
GAIF01026583.1	0	0	0	0	0	0.14
GAIF01026585.1	0	0.1	0	0	0	0
GAIF01026586.1	0.07	0	0	0	0	0
GAIF01026587.1	0	0	0	0.09	0.09	0
GAIF01026589.1	0	1.69	1.25	0	0	0
GAIF01026590.1	22.28	14.98	11.98	26.38	36.52	18.15
GAIF01026591.1	0	1.54	0	6.58	5.11	0
GAIF01026592.1	0	0	0	0.14	0.76	0
GAIF01026593.1	0	0	0	0	0.47	0.22
GAIF01026594.1	3.61	2.41	1.83	8.7	3	1.22
GAIF01026596.1	2.85	2.75	0	0	0	0.48
GAIF01026598.1	6.43	4.31	3.78	18.43	21.06	2.91
GAIF01026601.1	11.72	9.95	12.36	32.24	9.12	20.74
GAIF01026603.1	61.45	88.54	63.02	145.48	106.98	17.74
GAIF01026604.1	0	0.25	0.08	0.18	0.28	0
GAIF01026605.1	0	0	0	0	0.06	0
GAIF01026606.1	3.17	0.81	3.39	0.56	0.68	0.84
GAIF01026607.1	0	0.51	1.25	0.08	0	0.89
GAIF01026608.1	0.51	1.6	0.21	0.15	0.54	0
GAIF01026609.1	5.81	4.9	4.81	7.57	7.41	5.62
GAIF01026610.1	0.26	0	0	0.3	3.13	0.14
GAIF01026611.1	0.29	0	0	0	0	0.05
GAIF01026612.1	4.65	2.18	2.24	10.37	3.8	4.29
GAIF01026613.1	0	0	0	0.21	0.44	0
GAIF01026614.1	0.08	0.18	0.14	0	0.05	0
GAIF01026616.1	0.9	0.97	0.25	11.19	126.09	2.11
GAIF01026617.1	0	0	0	0	0.03	0
GAIF01026618.1	154.09	0.94	40.51	419.21	122.34	402.25
GAIF01026619.1	0	0.02	0	0	0	0
GAIF01026620.1	0.26	0	0	0	0.79	0.1
GAIF01026621.1	0	0	0	0.2	1.43	0

GAIF01026622.1	4.18	3.59	1.15	12.11	15.69	9.39
GAIF01026623.1	0.48	0.17	0.07	3.85	16.21	0.18
GAIF01026626.1	0	0.26	0	0.28	0	0
GAIF01026627.1	11.13	9.25	8.81	27.21	28.11	8.31
GAIF01026628.1	0	0	0	0.18	0.55	0
GAIF01026629.1	0.11	0	0	0.23	0.58	0.49
GAIF01026630.1	0	0	0	0	0.31	0
GAIF01026632.1	0	0	0.07	0	0.07	0
GAIF01026635.1	0	0.06	0.21	0	0	0.09
GAIF01026636.1	0.03	0	0	0.07	0	0
GAIF01026637.1	17.99	19.35	16.06	5.51	3.09	1.2
GAIF01026638.1	0.15	0.03	0.06	0.26	1	0.11
GAIF01026640.1	29.78	45.97	33.9	18.29	48.25	28.26
GAIF01026641.1	2.59	1.79	3.49	0.27	0.14	0
GAIF01026642.1	7.39	5.3	1.2	2.65	0.84	0
GAIF01026643.1	33.91	34.72	22.37	48.17	31.01	3.83
GAIF01026644.1	10.62	14.67	6.25	11.91	14.87	1.01
GAIF01026645.1	0	0.07	0.15	0.24	0	0
GAIF01026647.1	0.35	0.04	0.04	1.64	0.14	0
GAIF01026649.1	0.19	0.11	0	1.15	6.55	0.24
GAIF01026650.1	0.43	0	0	2.71	4.11	0
GAIF01026651.1	15.5	6.71	4.84	42.95	73.3	56.06
GAIF01026652.1	0	0	0	0.19	0.49	0
GAIF01026653.1	0	0	0	0	0	0.06
GAIF01026654.1	1.11	4.72	6.91	5.64	0	0
GAIF01026655.1	0.12	0.13	0.09	1.53	9.27	0
GAIF01026656.1	6.89	6.83	7.23	9.23	4.65	2.59
GAIF01026657.1	0	0.04	0	0	0	0
GAIF01026659.1	0.12	0.1	0	1.53	1.6	0
GAIF01026660.1	0.06	0	0	0	0	0
GAIF01026661.1	10.54	8.46	7.93	43.56	51.6	5.04
GAIF01026665.1	0.09	0	0	0	0.43	0
GAIF01026666.1	0.38	0	0	0.06	0.58	0
GAIF01026667.1	437.04	426.91	173.83	15.22	7.49	1.91
GAIF01026668.1	1.01	0.71	0	2.87	0.07	1.16
GAIF01026672.1	333.35	272.83	142.75	209.6	162.7	83.78
GAIF01026675.1	51.73	54.33	45.44	58.24	40.78	25.91
GAIF01026678.1	0	0	0	0	0	0.12
GAIF01026679.1	0.22	0	0	0.51	0.75	0.12
GAIF01026683.1	0.07	0	0	0.37	0.96	0
GAIF01026684.1	0	0	0	0	0.33	0
GAIF01026686.1	0	0	0	0.08	0.32	0
GAIF01026688.1	0.3	0.3	0.04	0	0	0
GAIF01026689.1	0.06	0.07	0.07	0.72	0.97	0

GAIF01026690.1	0.1	0	0.16	2.2	8.59	0.14
GAIF01026691.1	2.33	1.74	1.28	2.03	5.42	6.89
GAIF01026692.1	0	0	0	2.6	1.59	0
GAIF01026694.1	0.03	0	0	0	0	0
GAIF01026695.1	0.07	0	0	0	0	0
GAIF01026696.1	17.42	27.36	37.82	42.68	25.14	17.49
GAIF01026697.1	111.2	44.95	45.49	128.89	146	85.18
GAIF01026700.1	0	0	0	0	0.44	0
GAIF01026701.1	20.32	27.83	28.4	43.53	49.68	10.14
GAIF01026703.1	0.52	0.07	0	1.66	1.57	0.07
GAIF01026705.1	0	0.05	0	0	0	0
GAIF01026706.1	0.71	0.31	0	0	0.17	0
GAIF01026707.1	8.04	3.86	5.03	14.46	23.56	12.8
GAIF01026708.1	173.72	195.97	133.35	142.46	91.09	15.48
GAIF01026709.1	0.05	0.05	0.05	0	0.06	0.05
GAIF01026710.1	0	0	0	0	0.06	0
GAIF01026711.1	0.15	0.17	0	0	0	0.06
GAIF01026713.1	9.72	34.03	29.4	5.74	10.12	0.51
GAIF01026714.1	45.89	28.42	40.1	58.95	23.61	70.69
GAIF01026716.1	1.87	0.07	0.08	6.86	10	1.44
GAIF01026717.1	67.26	39.42	16.21	66.93	114.58	101.04
GAIF01026718.1	0	0.2	0.21	0	0	0
GAIF01026719.1	3.44	3.59	0.05	8.87	38.81	17.21
GAIF01026720.1	0	0	0.06	0.07	0	0
GAIF01026722.1	0	0.18	0.35	0	0	0.53
GAIF01026723.1	3.06	4.52	2.07	9.99	14.14	1.76
GAIF01026724.1	0.34	0	0	1.3	9.54	0.25
GAIF01026726.1	0.85	0.31	0.1	0	0	0.1
GAIF01026727.1	29.18	58.87	29.72	72.85	49.11	6.27
GAIF01026728.1	6.11	8.48	7.77	0.16	0.17	1.49
GAIF01026729.1	57.79	43.82	21.97	90.15	122.45	16.35
GAIF01026730.1	0.07	0	0	0.47	0.32	0
GAIF01026731.1	0	0	0	0.09	0	0
GAIF01026732.1	10.56	5.08	5.51	21.51	30.58	7.54
GAIF01026733.1	0.1	0.34	0	0	0.54	0
GAIF01026735.1	0	0	0	0	0.17	0
GAIF01026737.1	0.19	0.72	0.21	0.44	2.16	0
GAIF01026738.1	0.2	0	0	1.73	8.39	0
GAIF01026740.1	0.36	0.23	0.11	0.6	1.93	0.17
GAIF01026741.1	0	0	0	0	1.7	1.54
GAIF01026742.1	11.68	1.81	4.63	6.89	8.48	3.19
GAIF01026744.1	0	1.33	0.19	0	0	0
GAIF01026745.1	99.66	126.53	90.4	178.79	177.59	142.86
GAIF01026746.1	3.47	3.75	2.33	6.95	3.82	0.04

GAIF01026747.1	194.41	120.91	107.4	14.22	10.98	31.68
GAIF01026750.1	0	0	0	0.13	1.01	0
GAIF01026751.1	12.75	18.25	6.38	0.91	0.58	0.23
GAIF01026752.1	0	0	0	0.02	0.04	0
GAIF01026753.1	6.85	3.6	1.92	13.46	13.89	5.13
GAIF01026755.1	27.31	19.35	18.09	32.46	25.72	6.62
GAIF01026756.1	0.14	0	0	0.08	0.43	0.08
GAIF01026758.1	39.61	34.27	21.55	51.06	52.55	54.54
GAIF01026760.1	0.95	0.39	0.65	0.14	0.29	0.13
GAIF01026761.1	0	0	0	0.31	0	0
GAIF01026762.1	38.7	30.62	23.49	46.95	84.57	10.61
GAIF01026764.1	0.35	0	0	0.21	4.25	0.78
GAIF01026765.1	0	0.1	0.41	0	0	0.1
GAIF01026766.1	0.09	0	0	0	0	0
GAIF01026767.1	2	1.22	3.6	18.23	7.55	2.71
GAIF01026768.1	7.09	1.6	4.56	2.41	1.35	2.19
GAIF01026769.1	1.5	1.17	0.08	8.55	14.15	0.17
GAIF01026771.1	0	0.02	0	0.02	0	0
GAIF01026773.1	0	0	0	0	0.03	0
GAIF01026775.1	0.14	0.01	0	0	0.07	0.04
GAIF01026776.1	30.51	38.4	9.31	2.55	8.93	2.76
GAIF01026777.1	0	0.04	0	0	0.08	0
GAIF01026778.1	0	0.58	0.07	0	0.08	0
GAIF01026779.1	0	0	0.03	0	5.63	16.43
GAIF01026781.1	0	0.17	0.17	0.18	0	0
GAIF01026782.1	12.57	11.8	6.28	49.04	49.02	2.24
GAIF01026784.1	0.5	1.35	0.55	0	0.22	0.05
GAIF01026786.1	0.12	0.04	0.04	0.27	0.89	0.17
GAIF01026787.1	0.02	0	0	0.02	0.02	0
GAIF01026788.1	0	0.04	0.26	0.2	0.5	0
GAIF01026789.1	0	0.1	0	0	0	0
GAIF01026790.1	0	0.21	0	0.33	1.26	0.1
GAIF01026791.1	0	0	0	0.26	0	0
GAIF01026793.1	1.24	1.76	0.41	0.58	1.2	0
GAIF01026794.1	0.14	0.15	0	0.49	2.03	0
GAIF01026795.1	0.04	0	0	0.54	1.49	0
GAIF01026797.1	0.05	0.17	0.17	0	0	0
GAIF01026798.1	1.58	0.98	0.12	6.3	30.89	0.25
GAIF01026799.1	0.19	0.21	0	3.89	22.14	0.05
GAIF01026800.1	2.67	3.71	0.61	3.89	3.81	0.07
GAIF01026801.1	0.59	0.66	0.18	0.5	1.37	0.64
GAIF01026803.1	0.06	0	0	0.07	0	0
GAIF01026804.1	21.61	18.4	12.61	12.07	12.03	1.39
GAIF01026805.1	0	0.15	0	1.64	4.77	0

GAIF01026806.1	12.82	9.38	11.8	37.56	44.52	14.22
GAIF01026807.1	0.03	0	0	0	0.04	0
GAIF01026808.1	0	0	0.29	0	0.1	0.58
GAIF01026809.1	0.12	0	0	2.94	6.79	0
GAIF01026810.1	0.29	0.59	0.09	0.63	2.74	0.18
GAIF01026811.1	4.81	5.78	3.43	18.84	8.98	2.75
GAIF01026812.1	1.08	0.17	0	3.23	10.96	0.17
GAIF01026815.1	0	0	0	6.92	15.41	0.29
GAIF01026816.1	0.06	0.37	0.28	0.59	1.09	0
GAIF01026817.1	0	0	0.58	1.13	0.93	8.62
GAIF01026819.1	0.03	0	0	0.97	16.89	0
GAIF01026820.1	22.15	12.46	8.95	10.51	4.56	7.21
GAIF01026821.1	10.88	16.44	8.62	127.31	152.53	6.23
GAIF01026822.1	0.13	0.14	0	0.15	1.25	0
GAIF01026823.1	0.84	1.06	1.07	0.32	0.17	0.15
GAIF01026824.1	9.3	7.58	10.44	26.15	26.58	12.03
GAIF01026827.1	1.01	1.2	0.2	4.15	1.32	0.71
GAIF01026828.1	5.2	9.53	10.43	18.51	8.57	3.8
GAIF01026829.1	0	0.28	0	0.91	3.46	0
GAIF01026830.1	82.8	47.38	61.44	45.38	53.09	62.38
GAIF01026831.1	0.49	0	0.13	2.4	10.7	1.34
GAIF01026832.1	11.64	3.93	0	0	0	0
GAIF01026833.1	0	0	0	0	0.31	0
GAIF01026834.1	5.82	7.62	3.91	5.51	3.14	0.92
GAIF01026835.1	2.2	2.81	4.56	8	3.38	2.35
GAIF01026836.1	0.07	0.16	0	0.08	0.52	0
GAIF01026837.1	0.04	0	0.11	0.03	0.1	0
GAIF01026839.1	142.18	38.62	50.8	33.07	30.68	147.29
GAIF01026840.1	16.78	0	0	0	2.71	0
GAIF01026841.1	8.71	6.93	11.12	22.85	27.26	11.94
GAIF01026842.1	0.1	0	0	0.6	0.27	0
GAIF01026844.1	0	0	0	0	0.08	0
GAIF01026845.1	0	0	0.19	1.99	7.09	0
GAIF01026848.1	0	0.16	0.32	0	0	0.16
GAIF01026853.1	0	0	0	0	0.26	0
GAIF01026855.1	0	0	0	0	0.9	0
GAIF01026857.1	0	0	0.08	0	0.31	0
GAIF01026858.1	0.45	1.06	0.49	0.81	2.54	0.13
GAIF01026859.1	0.47	0.34	0	2.52	21.3	0.34
GAIF01026860.1	0	0	0	0	0.72	0
GAIF01026861.1	0	0	0	0.18	0	0
GAIF01026862.1	4.42	1.89	0.35	12.27	46.7	21.83
GAIF01026863.1	0	0	0.06	0	0	0
GAIF01026864.1	5.93	7.9	1.4	9.37	9.24	2.85

GAIF01026866.1	15.8	4.5	10.06	34.51	59.2	129.32
GAIF01026868.1	0	0	0	0	1.26	0
GAIF01026869.1	0	0.12	0.37	0	0.27	0
GAIF01026870.1	0	0.41	0.21	0.22	0.92	0.42
GAIF01026871.1	3404.29	2124.42	1321.34	2166.24	2672.79	4810.47
GAIF01026873.1	10.16	13.98	10.11	30.55	25.8	4.47
GAIF01026875.1	0.17	0.19	0	0.4	4.45	0
GAIF01026876.1	0.88	0.48	0.05	5.09	13.93	0
GAIF01026877.1	5.27	4.91	5.12	1.4	0.9	0.17
GAIF01026878.1	0.16	0.11	0.02	0.31	0	0
GAIF01026879.1	0.14	0	0.15	0	0	0.45
GAIF01026881.1	0	0.15	1.22	0	0	2.6
GAIF01026882.1	0.14	0	0	0	0	0
GAIF01026884.1	0	0	0	0	0	0.18
GAIF01026886.1	1.81	2.31	1.77	0.57	0.52	0
GAIF01026889.1	0.56	0.12	0	1.43	7.15	0
GAIF01026892.1	24.65	8.13	4.16	43.67	63.04	11.15
GAIF01026893.1	0.92	0.22	1.63	0	0	0
GAIF01026896.1	0	0.16	0	0.36	0.16	0
GAIF01026898.1	0.03	0	0	0	0	0
GAIF01026899.1	23.94	31.48	5.14	199.89	268.24	7.45
GAIF01026900.1	969.18	1217.97	917.11	608.84	1010.51	5970.35
GAIF01026901.1	2.55	4.08	3.85	5	5.37	0.79
GAIF01026902.1	27.44	20.8	37.87	53.32	24.89	35.77
GAIF01026903.1	77.22	23.85	13.73	96.04	44.04	8.09
GAIF01026904.1	15.33	6.4	3.4	0.08	0.13	0.04
GAIF01026905.1	8.3	4.39	7.18	3.38	1.91	1.47
GAIF01026906.1	0	0	0	0.24	0.99	0
GAIF01026907.1	0.04	0	0	0.05	0	0
GAIF01026908.1	7	4.3	9.19	5.83	6.22	8.56
GAIF01026909.1	0	1.32	3.71	0	0	5.22
GAIF01026910.1	1.16	0.56	0	0.87	0.71	0.16
GAIF01026911.1	17.59	7.24	7.15	1.49	2.26	3.97
GAIF01026914.1	870.04	593.57	355.37	91.26	67.48	33.85
GAIF01026915.1	0	0.02	0.01	0	0	0
GAIF01026917.1	0	0	0.05	0	0.05	0.25
GAIF01026922.1	0	0.1	0	0	0.02	0
GAIF01026923.1	0	0.38	0.38	1.4	2.99	0
GAIF01026924.1	5.24	6.21	5.55	0.73	0.26	0.46
GAIF01026926.1	4.1	3.96	2.02	15.86	18.65	1.34
GAIF01026928.1	21.33	3.53	4.31	18.73	15.68	15.77
GAIF01026929.1	0	0	0	0.54	2.78	0.68
GAIF01026930.1	0.49	1.58	0	2.82	2.34	0.54
GAIF01026931.1	0	0	0	0.08	0	0

GAIF01026932.1	2091.25	2462.57	2504.63	2404.4	2145.49	3362.74
GAIF01026934.1	0	0	0	0.08	0	0
GAIF01026935.1	16.12	1.1	1.11	37.71	131.14	409.95
GAIF01026936.1	12.04	8.22	7.34	2.68	4.05	2.04
GAIF01026937.1	0.07	0.04	0	0.17	0	0.04
GAIF01026941.1	80.65	31.88	26.86	84.77	118.57	114.8
GAIF01026943.1	0	0	0.11	0	0.62	0
GAIF01026945.1	32.84	26.52	45.63	26.02	20.78	31.67
GAIF01026946.1	61.72	61.2	48.14	266.17	197.01	38.67
GAIF01026947.1	0.18	0.27	0	0.78	2.06	0.13
GAIF01026948.1	0.07	0	0	0	0	0
GAIF01026949.1	16.12	6.38	3.16	28.25	3.76	0
GAIF01026951.1	0	0	0	0.15	0	0
GAIF01026953.1	25.09	14.39	19.98	35.19	20.39	15.34
GAIF01026955.1	0.89	0.32	0.22	6.65	23.35	1.66
GAIF01026956.1	8.7	4.88	4.93	12.52	12.41	4.75
GAIF01026957.1	2.72	0.11	0.32	5.53	12.86	4.61
GAIF01026958.1	0.12	0	0	0.14	1.16	0
GAIF01026959.1	0	0	0	0.3	0	0
GAIF01026960.1	1.46	0.93	0	0.8	0.34	0.01
GAIF01026962.1	0.59	0.12	0.38	0.31	0.52	0.26
GAIF01026964.1	43.14	31.9	60.6	35.76	17.24	32.33
GAIF01026965.1	0.14	0.05	0.1	0.11	0.11	0.05
GAIF01026966.1	1.88	5.17	5	2.33	3.33	0.74
GAIF01026967.1	8.6	13.68	3.85	31.44	47.75	2.13
GAIF01026968.1	56.11	46.89	35.16	51.1	81.36	35.34
GAIF01026969.1	0	1.21	0.99	0	0.05	0.05
GAIF01026972.1	0	0	0.23	0.28	0	0
GAIF01026973.1	0.71	1.51	0.57	1	0.43	0.19
GAIF01026974.1	0.04	0	0	0	0	0
GAIF01026976.1	13.97	19.83	19.99	30.99	21.65	10.22
GAIF01026978.1	0	0	0	0	0.52	0.48
GAIF01026979.1	0.57	0.28	0	1.58	2.96	0
GAIF01026980.1	0	0.07	0	0	0	0.07
GAIF01026982.1	0.13	0	0	0	1.12	0
GAIF01026983.1	0.06	0	0	0	0	0
GAIF01026984.1	0.45	1.72	0	1.38	1.01	0
GAIF01026985.1	43.28	63.81	27.53	66.81	77.4	18.71
GAIF01026986.1	0.53	0.12	0.12	0.87	4.75	0.24
GAIF01026987.1	0	0.1	0	0	0	0
GAIF01026988.1	0.41	0.63	0.26	2.5	7	2.05
GAIF01026989.1	6.53	2.35	0.35	2.75	5.89	0.81
GAIF01026990.1	0	0.08	0	0	0	0
GAIF01026991.1	0	0.09	0	0	0	0

GAIF01026994.1	0	0	0	0	0.46	3.2
GAIF01026996.1	0	0	0	0.09	2.03	0
GAIF01026997.1	0	0	0	0	0.32	0
GAIF01026998.1	2.71	2.82	2.13	7.79	5.04	4.47
GAIF01026999.1	7.45	5	5.57	14.13	9.57	3.77
GAIF01027001.1	164.22	221.81	110.11	160.94	187.81	226.77
GAIF01027002.1	1.45	1.67	0.4	7.46	11.32	0.2
GAIF01027003.1	0	0.03	0.04	0.74	2.07	0
GAIF01027005.1	4.83	0.8	0.09	6.37	14.38	0
GAIF01027007.1	0	0	0	0	0.68	0.21
GAIF01027008.1	0	0	0	0.5	0.37	0
GAIF01027010.1	2.11	0.31	0	3.43	11.68	22.34
GAIF01027012.1	0.35	0	0	0.76	1.08	0
GAIF01027014.1	0.25	0.27	0.18	0.29	3	0
GAIF01027015.1	0.14	0.16	0.16	0.17	0.47	0
GAIF01027016.1	4.16	5.07	3.39	5.78	6.06	0.28
GAIF01027017.1	0.07	0	0	0	0.25	0
GAIF01027018.1	29.18	29.79	14.54	26.69	19.67	5.9
GAIF01027019.1	0.39	0.29	0	0.92	4.91	1.89
GAIF01027020.1	3.42	2.83	0.9	14.6	31.25	0.75
GAIF01027022.1	1.02	0.05	0.03	0.98	1.34	0.17
GAIF01027023.1	8.12	7.52	13.86	1.05	0.12	0.22
GAIF01027025.1	0.35	0.48	0.38	5.39	6.69	0.61
GAIF01027026.1	0	0	0	3.29	0	0
GAIF01027027.1	0	0	0	1.28	0.94	3.22
GAIF01027028.1	9.33	14.1	9.91	31.05	44.57	3.48
GAIF01027029.1	0	0	0	0	0.82	0.04
GAIF01027030.1	112.75	41.08	54.91	45.53	23.01	32.92
GAIF01027031.1	1.32	1.32	1.44	2.58	1.46	0.89
GAIF01027032.1	10.37	10.99	12.4	10.82	9	3.91
GAIF01027033.1	10.18	2.11	2.13	17.08	33.4	17.75
GAIF01027035.1	0	0	0	0.03	0.11	0
GAIF01027037.1	4.53	4.53	2.34	24.51	51.64	0.97
GAIF01027041.1	0.78	0.24	0.31	0.97	1.07	0.09
GAIF01027042.1	2.93	0.5	0.05	14.26	20.98	0.15
GAIF01027043.1	23.17	22.29	9.29	22.66	16.06	0
GAIF01027044.1	0	0	0	0	0.22	0
GAIF01027046.1	9.85	31.58	100.99	4.98	4.63	28.96
GAIF01027047.1	6.17	1.02	4.86	6.01	3.25	1.06
GAIF01027049.1	37.82	49.96	51.18	80.28	42.65	37.61
GAIF01027050.1	0.71	0.26	0.05	2.36	8.64	0
GAIF01027052.1	0.98	0	0	0.76	4.19	2.88
GAIF01027053.1	50.45	48.37	67.01	151.38	73.72	70.2
GAIF01027054.1	0	0	0	0.07	0.67	0

GAIF01027055.1	0.11	0	0	0	0	0
GAIF01027056.1	0.35	0.35	0.14	2.31	5.67	0.11
GAIF01027057.1	10.03	14.15	20.45	11.16	11.22	3.13
GAIF01027058.1	2.25	1.04	0.21	1.43	2.89	0.9
GAIF01027059.1	58.59	67.2	44.82	10.92	11.26	0
GAIF01027061.1	0	0	0.11	0.24	0.25	0
GAIF01027062.1	75.06	53.19	90.75	162.3	148.25	69.09
GAIF01027063.1	1.66	2.58	0	1.98	4.14	0
GAIF01027064.1	0.53	0.03	0.03	0.38	1.1	0.45
GAIF01027065.1	18.28	25.44	21.22	44.81	41.67	10.35
GAIF01027067.1	0	0.25	0	0	0	0
GAIF01027068.1	0	0	0	0.04	0	0
GAIF01027069.1	0	0	0	0	0.44	0
GAIF01027070.1	0.63	0	1.04	0.51	0	0
GAIF01027071.1	19.53	17.53	25.72	1.69	0.84	1.84
GAIF01027072.1	0	0.13	0	0	0.32	0.05
GAIF01027073.1	28.84	19.6	21.73	70.55	82.17	30.91
GAIF01027074.1	0.41	0	0	1.1	3.26	2.1
GAIF01027075.1	79.27	28.98	32.55	159.71	66.56	46.03
GAIF01027076.1	0.66	0.51	0	0.11	0	0
GAIF01027079.1	7.36	14.17	5.05	34.84	37.52	5.02
GAIF01027080.1	0	0	0	0	1.41	1.29
GAIF01027081.1	0	0	0	0.03	0.14	0
GAIF01027083.1	0	0.02	0	0	0.06	0.05
GAIF01027084.1	0.12	0	0	1.77	6.2	0.13
GAIF01027085.1	22.43	24.39	38.08	66.77	29.65	19.8
GAIF01027086.1	0.16	0.61	0.18	0.34	4.67	0.88
GAIF01027087.1	0.57	0.07	0	0.07	0.15	0
GAIF01027089.1	0.34	0	0	2.01	4.22	0.72
GAIF01027090.1	0	0	0	0	0.37	0
GAIF01027091.1	0.12	0.04	0.08	0.07	0.26	0
GAIF01027092.1	0	0.13	0.14	0	0	0
GAIF01027093.1	44.76	57.24	30.74	114.58	90.55	7.11
GAIF01027096.1	13.2	8.29	2.47	13.69	19.83	1.24
GAIF01027097.1	7.07	5.37	2.91	3.37	2.82	0.79
GAIF01027098.1	0.35	0.47	0.29	2.05	6.64	0.25
GAIF01027100.1	11.59	9.35	7.49	4.38	4.67	1.34
GAIF01027101.1	0	0	0	0.82	0.85	0
GAIF01027102.1	0.08	0.05	0	0.54	0.71	0.32
GAIF01027103.1	64.03	54.91	18.74	68.96	63.48	12.54
GAIF01027105.1	17.27	25.18	13.86	9.49	15.55	2.26
GAIF01027106.1	0	0	0	0	0	0.01
GAIF01027108.1	10.37	14.89	11.76	56.88	66.57	4.49
GAIF01027109.1	0.21	0	0.17	0	0	0.05

GAIF01027110.1	0.8	1	1.08	3.12	1.48	0.58
GAIF01027111.1	0.16	0.02	0	0.43	1.26	0
GAIF01027112.1	0.19	0	0	0.22	0.23	0
GAIF01027113.1	21.07	22.11	21.1	28.32	29.45	17.52
GAIF01027114.1	3.55	1.84	0.99	1.2	1.45	0.9
GAIF01027115.1	0	0	0	0.07	0.14	0
GAIF01027116.1	0	0.04	0.13	0	0	0.86
GAIF01027117.1	0.15	0	0	1.88	4.88	0.08
GAIF01027118.1	22.07	18.48	6.73	48.83	70.96	24.15
GAIF01027122.1	13.76	7.91	8.78	12.66	17.24	20.6
GAIF01027123.1	0.06	0.12	0	0.51	3.32	0.55
GAIF01027125.1	0	0	0	0	0.15	0
GAIF01027127.1	0.24	0	0.16	0.68	0.46	0
GAIF01027128.1	85.55	129.17	168.29	3.81	3.71	11.02
GAIF01027129.1	0	0	0	0	0.1	0
GAIF01027131.1	0	5.88	0	0	0	0
GAIF01027132.1	0.05	0	0	0.06	0.12	0
GAIF01027133.1	0	0	0	0	0	0.07
GAIF01027136.1	2.65	1.94	1.85	1.43	0.53	0
GAIF01027137.1	3.24	2.58	1.64	7.96	14.4	1.37
GAIF01027138.1	4.53	3.37	0	6.09	21.2	0.29
GAIF01027140.1	1.41	2.31	1.72	10.55	21.63	1.15
GAIF01027141.1	248.21	215.94	442.65	247.34	12.65	133.12
GAIF01027142.1	0	0.09	0	0	0	0
GAIF01027144.1	0.95	1.16	0.7	1.01	0.91	0.48
GAIF01027145.1	14.33	12.11	27.3	29.26	14.33	63.65
GAIF01027146.1	45.21	55.75	52.06	65.59	36.97	22.79
GAIF01027148.1	12.44	10.8	1.67	84.43	161.2	10.91
GAIF01027149.1	0.6	0.52	0.4	1.4	0.58	0
GAIF01027151.1	0	0	0	0	0.11	0
GAIF01027152.1	9.66	5.93	6.72	27.08	35.19	6.22
GAIF01027153.1	167.05	160.73	155.69	10.58	10.56	23.07
GAIF01027158.1	46.62	69.7	22.39	71.02	52.56	28.9
GAIF01027159.1	0	0	0	0	0	0.38
GAIF01027160.1	0	0.05	0	0	0	0
GAIF01027161.1	5.6	5.2	4.52	19.13	16.45	2.27
GAIF01027162.1	0.18	0	0	0	0	0
GAIF01027163.1	6.8	9.42	4.21	19.92	15.53	3.76
GAIF01027166.1	7.38	10.85	17.47	11.28	9.87	2.52
GAIF01027168.1	0	0	0	0	9.99	4.76
GAIF01027169.1	0.45	0.47	0	0.78	0	0
GAIF01027171.1	0	0.05	0.05	0	0.25	0
GAIF01027173.1	0.27	0	0	0.27	0	0
GAIF01027174.1	0	0	0	0.21	0	0

GAIF01027175.1	46.54	47.59	56.75	4.21	2.7	1.96
GAIF01027176.1	0.12	0.78	1.18	0.97	0.72	0
GAIF01027177.1	19.96	19.79	28.57	32.61	14.59	19
GAIF01027178.1	0	10.43	0	0	1.19	0
GAIF01027180.1	2.59	2.97	0.7	6.66	14.21	0
GAIF01027184.1	11.68	9.63	12.18	18.36	8.02	11.27
GAIF01027186.1	140.5	134.3	271.72	143.41	119.71	157.04
GAIF01027187.1	0	0	0	1.62	8.08	0
GAIF01027188.1	0	0	0	0.47	0.98	0.22
GAIF01027189.1	0.37	0.25	0.82	2.22	4.03	0.58
GAIF01027190.1	0	0.26	0	0	0	0.37
GAIF01027191.1	46.83	78.12	73.23	35.05	18.93	30.8
GAIF01027194.1	0.93	0.25	0.51	1.21	5.99	0
GAIF01027195.1	0	0	0	0	0.08	0
GAIF01027199.1	107.94	27.84	37.43	115.04	131.86	72
GAIF01027201.1	73.02	81.33	41.7	117.4	87.53	5.3
GAIF01027203.1	0	1.8	1.92	0	0	0.1
GAIF01027204.1	0	0	0	0	0.05	0
GAIF01027206.1	21.05	11.92	20.56	45	55.3	16.39
GAIF01027207.1	243.04	55.46	63.89	196.74	184.28	83.45
GAIF01027208.1	21.6	0	0	0	0	0
GAIF01027211.1	9.61	12.87	14.99	28.62	11.06	7.37
GAIF01027212.1	101.56	98.1	152.08	38.15	13.06	24.46
GAIF01027213.1	0.34	0.18	0	0.39	7.34	3.17
GAIF01027214.1	0.33	0.21	0.36	0.05	0.06	0
GAIF01027215.1	4.31	4.97	1.45	15.66	12	0.82
GAIF01027216.1	1.41	2.04	0.62	8.16	3.95	0.31
GAIF01027217.1	2.4	2.21	1.01	2.04	1.78	0.2
GAIF01027218.1	0	0	0.11	0	0	0
GAIF01027219.1	0	7.96	4.36	0	0	0.59
GAIF01027220.1	0.11	0	0	0.26	0.14	0
GAIF01027223.1	4.17	9.81	9.07	15.87	12.02	0.69
GAIF01027224.1	0	0	0	0.06	0.14	0
GAIF01027225.1	20.5	23.39	15.57	53.45	34.28	10.42
GAIF01027226.1	2.76	12.36	4.15	23.48	20.14	1.23
GAIF01027228.1	0	0.36	0	0	0	0
GAIF01027230.1	4.32	2.28	2.31	2.84	0.84	0.77
GAIF01027231.1	2.66	0	1.26	0	1.57	0
GAIF01027232.1	1.7	1.7	1.15	2.27	0.63	0.14
GAIF01027233.1	11.29	4.98	7.86	23.05	22.19	23.02
GAIF01027234.1	17.41	11.29	26.66	37.58	82.85	145.98
GAIF01027237.1	1.01	0.57	1.03	0.36	1.34	1.81
GAIF01027238.1	0.09	0	0	0.05	0	0
GAIF01027239.1	0	0	0	2.52	0	0

GAIF01027242.1	5.77	6.69	4.36	6.71	4.34	1.03
GAIF01027243.1	464.67	143.64	172.55	8.23	7.04	0.92
GAIF01027244.1	16.92	32.34	17.42	79.22	69.78	1.4
GAIF01027246.1	1.2	0	16.26	5.02	0	0
GAIF01027247.1	0.28	0.41	0.1	0.76	2.26	0
GAIF01027248.1	0	0.07	0	0	0.12	0
GAIF01027249.1	0.54	0.25	0.04	0.25	1.39	0.11
GAIF01027251.1	0	0	0	0	0.05	0
GAIF01027252.1	0	0	0	0	1.66	0
GAIF01027254.1	0	0	0	0	0	0.66
GAIF01027255.1	0.29	0.33	0.09	0.72	0.41	0.07
GAIF01027257.1	17.89	37	27.73	36.62	52.46	3.93
GAIF01027258.1	9.5	19.97	8.81	37.4	24.97	2.64
GAIF01027259.1	31.79	43.07	37.19	4.54	3.45	0.29
GAIF01027260.1	0.02	0	0	0	0	0
GAIF01027262.1	0.15	0.16	0.11	2.95	4.02	0
GAIF01027263.1	0	0	0	0.28	0	0
GAIF01027264.1	0	0	0	0.19	0.69	0
GAIF01027265.1	5.31	1.82	3.4	10.59	10.1	6.1
GAIF01027266.1	18.62	44.94	44.57	43.88	19.33	9.31
GAIF01027267.1	15.18	5.18	17.24	25.53	21.22	45.67
GAIF01027268.1	3.39	3.27	2.7	0.74	1.3	1.47
GAIF01027270.1	14.54	0	17.93	0	22.89	0
GAIF01027271.1	6.49	7.87	2.5	9.34	8.82	0.22
GAIF01027272.1	3.04	0.58	0.07	0.55	1.36	0.07
GAIF01027273.1	15.92	17.31	16.21	33.08	28.56	7.92
GAIF01027274.1	0	0	0	0.27	0	0
GAIF01027275.1	0	0	0	0.75	3.13	0
GAIF01027276.1	0.11	0.23	0.23	3.46	8.96	0.23
GAIF01027277.1	34.41	37	19.59	8.43	7.66	1.26
GAIF01027278.1	0	0.14	0.58	0	0	0
GAIF01027279.1	37.42	23.81	12.6	13.84	12.09	3.15
GAIF01027280.1	0	0	0	0.06	0.18	0
GAIF01027281.1	0	0.11	0	0.11	0.82	0
GAIF01027283.1	2.93	2.37	2.02	0.87	1.81	0.09
GAIF01027284.1	0	0	0	0.16	0	0
GAIF01027285.1	1.81	0.1	0.31	0	0	0
GAIF01027286.1	0	0.07	0	0	0.08	0
GAIF01027288.1	3.83	5.13	3.2	3.84	3.66	1.84
GAIF01027289.1	165.69	153.47	84.97	253.97	323.06	201.27
GAIF01027290.1	268.1	211.7	72	93.81	147.33	26.14
GAIF01027291.1	0.4	0.22	0	2.22	14.3	0
GAIF01027293.1	0	3.08	11.89	6.63	5.65	0
GAIF01027294.1	0.28	0.29	0.13	1.65	5.21	0.18

GAIF01027295.1	0.11	0	0	0	0	0
GAIF01027296.1	0.89	1.1	0	2.82	6.53	0
GAIF01027297.1	5.25	2.91	2.28	8.28	21.8	7.97
GAIF01027299.1	0.02	0.11	0.04	0.04	0.14	0
GAIF01027300.1	46.63	84.17	27.24	37.13	31.36	1.54
GAIF01027301.1	2211.03	871.55	1416.37	1217.97	1426.62	8506.42
GAIF01027305.1	33.94	18.17	14.28	98.58	100.4	44.18
GAIF01027306.1	0.36	0	0	0	0	0.8
GAIF01027307.1	33.5	17.88	11.65	77.1	75.13	7.86
GAIF01027309.1	14.13	4.6	5.43	42.99	81.73	24.42
GAIF01027311.1	7.55	10.23	6.79	31.36	42.21	5.35
GAIF01027312.1	11.58	1.32	0.55	41.24	67.24	5.36
GAIF01027313.1	8.25	13.84	9.75	9.6	24.61	6.89
GAIF01027314.1	3400.09	4983.03	8884.64	2012.54	1396.13	5617.1
GAIF01027315.1	98.82	190.72	307.06	117.41	69.26	75.74
GAIF01027316.1	6.45	3.26	4.63	10.86	7.32	4.41
GAIF01027318.1	0	0	0	0	0.36	0
GAIF01027319.1	213.3	8.24	9.89	472.6	919.42	11.47
GAIF01027321.1	57.05	66.65	103.97	83.95	38.64	60.84
GAIF01027322.1	15.29	11.63	5.03	8.2	9.88	3.79
GAIF01027324.1	1	4.1	2.16	7.45	15.42	0
GAIF01027325.1	4.22	11.49	7.67	12.43	16.07	1.06
GAIF01027326.1	0.19	0	0.21	0	0	0
GAIF01027328.1	80.78	52.07	31.24	92.08	88.04	9.47
GAIF01027329.1	52.03	35.43	27.54	50.89	21.8	4.48
GAIF01027331.1	0	0	0	0	0.33	0
GAIF01027332.1	0	0	0	0	0.13	0
GAIF01027335.1	15.27	10.83	19.07	39.84	45.4	27.4
GAIF01027339.1	0	0.6	1.07	0	0	0
GAIF01027340.1	0.76	1.83	1.51	2.66	4.59	0
GAIF01027341.1	0.6	0.39	0	0.56	2.17	0.4
GAIF01027342.1	0	0	0.13	0	0	0
GAIF01027343.1	13.59	12.09	9.61	31.42	21.42	10.78
GAIF01027344.1	9.62	10.14	4.4	48.52	48.44	2.65
GAIF01027345.1	0.32	0	0	0.92	0	0
GAIF01027347.1	190.78	180.82	150.89	95.08	70.23	166.39
GAIF01027350.1	0.53	0.41	0.38	0.06	0.12	0.29
GAIF01027352.1	0	0	0.01	0	0	0
GAIF01027353.1	0.3	0.57	0.31	0.17	0.24	0.08
GAIF01027354.1	42.12	18.51	50.93	10.34	2.28	24.02
GAIF01027355.1	0	0	0	0	0.13	0
GAIF01027357.1	0.54	0.3	0.04	0.18	0.42	0
GAIF01027358.1	4.46	11.67	9.55	13.54	14.86	1.44
GAIF01027359.1	6.85	0.51	0.84	7.68	0.58	0.45

GAIF01027362.1	136.01	138.49	153.67	214	191.55	476.46
GAIF01027364.1	0.23	0	0	0.13	0	0
GAIF01027365.1	16.09	12.19	13.42	40.93	46.59	7.54
GAIF01027367.1	0	0	0	0	0.7	0
GAIF01027368.1	0.17	0	0.55	0.39	2.01	0
GAIF01027372.1	30.75	16.12	33.68	85.39	76.15	117.27
GAIF01027373.1	7.01	10.44	7.92	15.7	34.63	10.43
GAIF01027374.1	46.88	66.76	39.96	104.88	49.92	9.82
GAIF01027375.1	0.16	0	0	0	0.19	0
GAIF01027378.1	4.12	17.16	1.09	17.73	38.73	0.3
GAIF01027379.1	39.91	33.15	32.52	95.51	55.38	20.07
GAIF01027380.1	0	0.2	0	1.23	7.57	0
GAIF01027381.1	0	0	0	0	0.12	0
GAIF01027382.1	0.06	0	0	0	0	0
GAIF01027383.1	1.4	1.52	0.77	4.46	12.61	1.93
GAIF01027384.1	0.34	0	0	0.83	6.52	0.78
GAIF01027387.1	0	0.11	0.11	0.12	0	0.11
GAIF01027388.1	0	0	0	0	0.13	0
GAIF01027389.1	0	0	0	0.06	0.06	0
GAIF01027390.1	0.18	0	0	0	0	0
GAIF01027391.1	0	0.13	0	0.29	0	0.14
GAIF01027392.1	5.51	0.7	0.32	16.25	115.79	1.74
GAIF01027393.1	17.33	22.75	60.86	15.39	7.44	24.69
GAIF01027395.1	1.09	1.27	1.77	1.46	1.31	0.22
GAIF01027398.1	0.66	0.73	0	4.38	6.94	0.36
GAIF01027399.1	0	0	0.1	0.1	1.46	0
GAIF01027400.1	18.08	13.3	32.99	60.18	30.22	61.83
GAIF01027401.1	1.51	3.14	6.42	5.17	0	6.51
GAIF01027402.1	0	0.03	0	0	0	0
GAIF01027403.1	3.47	4.59	6.75	2.08	1.4	1.62
GAIF01027404.1	2.2	2.22	1.38	4.37	1.32	0.69
GAIF01027405.1	2.07	1.4	0.43	10.71	14.52	0.85
GAIF01027406.1	0	0.09	0	0.1	0.1	0
GAIF01027408.1	1.31	0.55	0.67	4.8	15.78	15.47
GAIF01027410.1	0	0	0	0	0.07	0
GAIF01027412.1	0.16	0	0	0	1.72	0
GAIF01027413.1	1.13	0.54	0.41	6.82	31.12	0.96
GAIF01027414.1	13.26	10.58	10.31	55.8	103.15	10.23
GAIF01027415.1	0	0	0	0	0.18	0
GAIF01027416.1	6.86	4.18	5.58	38.03	59.93	8.32
GAIF01027417.1	0	0	0	0.47	2.65	0
GAIF01027419.1	0.46	0.12	0	0.4	7.32	2.53
GAIF01027420.1	19.32	15.51	14.32	32.14	26.98	10.09
GAIF01027421.1	2.29	2.91	2.65	3.95	3.53	0.5

GAIF01027424.1	41.37	100.67	141.67	74.62	53.46	29.49
GAIF01027425.1	1.77	2.73	2.39	1.1	2.42	3.9
GAIF01027427.1	0	0	0.03	0.03	0	0
GAIF01027429.1	0.86	0.13	0	0	0	0.39
GAIF01027430.1	0.05	0	0	0	0.06	0
GAIF01027433.1	0	0	0	0	0.26	0
GAIF01027434.1	5.53	6.56	9.35	23.96	11.89	4.45
GAIF01027436.1	0	0	0	0	0.37	0
GAIF01027437.1	0.44	0.42	0.34	1.92	0	0
GAIF01027438.1	0.05	0	0	0	0	0
GAIF01027440.1	3.23	6.33	2.64	10.87	22.08	0.63
GAIF01027444.1	0	0	0	0	0	0.12
GAIF01027448.1	0	0	0	0.34	0	0
GAIF01027449.1	0.44	0.18	0.42	0.13	0	0.24
GAIF01027450.1	40.69	42.56	71.74	35.42	23.29	51.75
GAIF01027451.1	0	0.11	0	0	0.79	0.43
GAIF01027452.1	15.33	9.4	8.23	47.62	30.56	3.1
GAIF01027453.1	85.54	111.59	38.85	38.38	40	0
GAIF01027454.1	34.97	27.97	35.85	70.1	31.88	18.79
GAIF01027457.1	47.15	28.12	59.93	112.4	62.21	132.84
GAIF01027458.1	0	0.6	0	1.54	0.2	0
GAIF01027459.1	11.4	11.58	14.48	28.54	12.59	7.84
GAIF01027460.1	0.32	0.17	0.28	0.44	1.18	0.03
GAIF01027462.1	1.37	0.5	1.94	1.59	3.57	5.83
GAIF01027466.1	0	0	0	0.16	0	0
GAIF01027467.1	0.05	0	0.05	0	0.06	0
GAIF01027468.1	5.06	6.78	5.15	21.76	20.05	2.35
GAIF01027472.1	29.3	22.81	4.5	61.41	50.69	0
GAIF01027474.1	0.09	0.05	0.03	0.21	0.04	0.08
GAIF01027475.1	0.06	0	0	0	0	0
GAIF01027476.1	1.93	0.18	0.18	11.78	30.81	15.63
GAIF01027478.1	0.24	0	0	0	0.43	0
GAIF01027479.1	0	0	0	0.25	0.26	0
GAIF01027480.1	0	0	0	0	0.11	0
GAIF01027481.1	12.89	9.39	6.37	15.33	17.46	12.41
GAIF01027483.1	2.36	1.21	1.7	3.13	6.5	3.24
GAIF01027484.1	9.3	8.8	0.38	1.6	2.49	0
GAIF01027485.1	0.09	0.08	0	0	0.7	0.08
GAIF01027486.1	0.02	0	0	4.19	12.17	0.09
GAIF01027487.1	32.06	14.23	16.75	13.31	11.99	45.96
GAIF01027488.1	0	0	0	0.24	0.38	0
GAIF01027489.1	3.44	4.54	9.12	5.11	5.57	13.98
GAIF01027490.1	1.25	0.1	0	2.12	15.73	0.25
GAIF01027492.1	0.12	0	0	0	0	0

GAIF01027493.1	0	0	0	0	0.08	0
GAIF01027494.1	0	0	0	0	0.2	0
GAIF01027495.1	0	0.06	0	0.33	1.25	0
GAIF01027497.1	0	0	0	0	0.1	0
GAIF01027498.1	27.57	38.97	29	27.07	17.44	15.61
GAIF01027499.1	3.96	7.6	6.05	14.42	7.92	1.18
GAIF01027501.1	33.37	35.68	15.56	61.68	54.67	16.52
GAIF01027502.1	10.56	19.87	5.94	6.13	6.67	0.3
GAIF01027505.1	0	0	0	0.08	0.04	0
GAIF01027508.1	0	0	0	0	0.21	0
GAIF01027513.1	0	0	0	0.05	0.06	0
GAIF01027515.1	115.12	189.9	220.19	339.97	311.76	132.02
GAIF01027516.1	26.1	42.84	8.4	31.56	23.98	0.8
GAIF01027517.1	0.09	0	0	0.4	0.21	0
GAIF01027518.1	0	0	0	0	0.22	0
GAIF01027520.1	49.4	34.6	31.33	98.19	75.81	11.91
GAIF01027524.1	0.14	0.6	1.51	0.48	0.17	0.61
GAIF01027525.1	21.97	29.23	19.28	46.59	51.43	6.75
GAIF01027526.1	4.2	1.07	0.92	12.03	38.57	8.8
GAIF01027528.1	0.18	0	0.02	0.84	5.79	0.13
GAIF01027529.1	103.96	116.74	44	11.92	5.45	3.16
GAIF01027530.1	11.36	22.39	10.1	78.78	105.56	4.26
GAIF01027531.1	0.14	0	0	0.5	1.72	0
GAIF01027533.1	296.51	295.58	561.74	195.41	114.74	168.83
GAIF01027534.1	223.49	140.39	258.05	18.49	7.79	22.55
GAIF01027535.1	0	0	0	0.28	1.35	0
GAIF01027537.1	27.83	10.96	6.35	59.08	83.03	29.3
GAIF01027538.1	0	0	0	0.04	0.13	0
GAIF01027539.1	0.15	1.37	0.32	0.28	0.52	0
GAIF01027540.1	0	0.03	0	0.08	0	0
GAIF01027541.1	0	0	0	0.15	0.3	0
GAIF01027542.1	1.51	0.68	0.05	1.52	5.81	0.48
GAIF01027543.1	0.03	0.04	0	0.06	0.18	0
GAIF01027544.1	686.2	257.67	188.82	771.6	668.55	1386.75
GAIF01027545.1	226.57	179.9	173.64	118.8	113.07	79.27
GAIF01027546.1	7.38	0	0.6	3.78	2.94	6.53
GAIF01027547.1	10.27	13.58	17.37	20.03	12.7	5.75
GAIF01027549.1	0.8	0.24	0.44	1.77	2.88	0.83
GAIF01027551.1	117.77	121.16	75.69	171.22	179.53	79.51
GAIF01027552.1	0	0.07	0	0	0	0
GAIF01027553.1	0	0	0.17	0.18	0.76	0
GAIF01027555.1	0.17	0	0	0.4	0.72	0
GAIF01027556.1	5.5	3.91	4.52	16.75	13.82	4.2
GAIF01027557.1	0.5	0.22	0.88	0	0	0

GAIF01027558.1	0	0.13	0	0.12	0.77	0
GAIF01027559.1	12.71	7.13	8.1	18.63	22.78	7.47
GAIF01027560.1	0.31	0.46	0.06	0.55	0	0.06
GAIF01027561.1	0	0	0	0	2.32	0
GAIF01027562.1	0.11	0	0.03	32.68	15.37	0.04
GAIF01027563.1	8.14	10.53	7.79	21.29	33.2	11.74
GAIF01027564.1	0	0	0	0	0.24	0
GAIF01027567.1	0.16	0.09	0.09	0.24	0.73	0
GAIF01027568.1	11.22	8.43	6.24	27.18	20.39	7.01
GAIF01027569.1	0	0.14	0.03	0.08	0.08	0
GAIF01027571.1	0	0.41	0	0	0	0
GAIF01027573.1	0.05	0	0	0	0	0
GAIF01027575.1	0	0	0.04	0	0	0
GAIF01027576.1	0.18	0	0	0.2	0.21	0
GAIF01027577.1	2.2	0.43	0.1	0.11	0	0
GAIF01027578.1	0.15	0.48	0.48	0.34	0.71	0
GAIF01027581.1	35.09	7.39	8.97	4.04	1.01	0.35
GAIF01027582.1	0.66	0.43	0	0.31	0	0
GAIF01027584.1	34.04	44.48	25.86	68.65	57.73	14.4
GAIF01027586.1	0	0.08	0	0.16	1.17	0.31
GAIF01027587.1	0	0.31	0	0.33	1.04	0
GAIF01027588.1	0	0.14	0	0	0	0
GAIF01027590.1	100.75	34.7	36.13	149.24	204.23	91.93
GAIF01027591.1	3593.17	3842.46	4814.58	2310.06	1718.35	2256.96
GAIF01027592.1	0.13	0	0	1.32	5.64	0
GAIF01027593.1	0	0.02	0.02	0	0.02	0
GAIF01027594.1	1.3	1.67	1.56	3.16	3.13	0.52
GAIF01027595.1	146.96	95.56	86.71	276.39	332.52	177.24
GAIF01027596.1	0.1	0	0	0	0.17	0.05
GAIF01027597.1	14.59	13.33	9.5	47.28	24.55	3.7
GAIF01027598.1	0.31	2.12	0.95	0.17	1.82	0.34
GAIF01027602.1	0.53	0.39	0	0	0	0.07
GAIF01027603.1	0	0	0	0.14	0.84	0
GAIF01027604.1	0	0	0.14	0	0.25	0
GAIF01027605.1	7.13	2.24	4.67	8.91	1.81	16.92
GAIF01027606.1	12.42	6.87	6.89	29.15	30.25	35.02
GAIF01027607.1	0	0	0.04	0	0	0
GAIF01027608.1	0.13	0	0	0.23	0	0
GAIF01027609.1	0.29	0.06	0.19	1.06	3.38	0.25
GAIF01027610.1	7.28	7.19	6.53	22.83	18.93	1.94
GAIF01027611.1	0.49	0	0	0	0	0
GAIF01027612.1	0	0	0	0.07	0.51	0.07
GAIF01027614.1	0.96	0.82	0.88	0.04	0.04	0.12
GAIF01027615.1	0	0	0	0.04	0.48	0

GAIF01027617.1	198.33	370.37	91.08	175.29	269.75	31.83
GAIF01027619.1	73.97	105.81	68.99	358.69	335.27	49.14
GAIF01027620.1	0	0	0	0	0.12	0
GAIF01027621.1	0.66	0.08	0	0.76	3.41	0.08
GAIF01027622.1	2.62	1.53	0	6.79	10.31	2.36
GAIF01027626.1	12.77	12.41	12.02	5.38	6.3	3.72
GAIF01027627.1	0	0.05	0.1	0.26	1.17	0
GAIF01027629.1	0	0.19	0.26	0.2	0.14	0
GAIF01027630.1	0.2	0.72	0.29	1.55	2.96	0.15
GAIF01027631.1	0.26	0	0	0	0	0
GAIF01027632.1	0	0	0.14	0.3	0	0
GAIF01027633.1	0	0	0.73	1.03	2.65	0.2
GAIF01027634.1	145.65	66.44	73.29	101.03	108.02	44.96
GAIF01027637.1	0.23	0.6	0.17	0	0.18	0
GAIF01027638.1	3.47	8.89	4.67	7.17	0	0
GAIF01027640.1	0	0	0	0.05	0.1	0
GAIF01027641.1	59.94	31.17	31.64	74.49	62.64	109.19
GAIF01027642.1	0.08	0.72	0.09	1.43	0.49	0.18
GAIF01027643.1	0	18.99	28.17	0.14	0.29	1.96
GAIF01027645.1	0	0	0.1	0.05	0	0
GAIF01027647.1	0	0	0	0.69	1.48	0.04
GAIF01027649.1	3.1	1.64	1.09	11.87	15.11	1.72
GAIF01027650.1	0.13	0.21	0	0.06	0.89	0
GAIF01027651.1	0	0.17	0	0	0	0
GAIF01027652.1	0	0	0	0.66	2.11	0.08
GAIF01027653.1	0.07	0	0	0	0.17	0
GAIF01027656.1	0.25	0.4	0.38	0.74	4.57	0.14
GAIF01027658.1	0	0.01	0	0	0.17	0
GAIF01027662.1	1.84	2.08	2.52	0	0	0.08
GAIF01027663.1	2.2	0.96	1.81	3.46	2.51	1.72
GAIF01027664.1	154.98	238.17	254.37	159.51	139.66	79.93
GAIF01027665.1	0.41	0	0.12	0	0	0
GAIF01027666.1	0	0	0.02	0	0	0
GAIF01027668.1	12.26	8.35	5.54	8.4	6.93	8.19
GAIF01027669.1	0.91	0.45	0.98	0	1.4	0
GAIF01027670.1	5.35	20.09	5.16	0.32	0.25	0.53
GAIF01027671.1	7.82	6.65	8.15	16.77	10.93	6.17
GAIF01027673.1	0	0	0	0	1.96	0
GAIF01027674.1	0	0	0	0.05	0	0
GAIF01027675.1	1.78	1.23	0.7	4.41	0	0.28
GAIF01027678.1	0.04	0	0	0	0	0
GAIF01027679.1	14.56	16.37	19.76	24.66	27.88	8.99
GAIF01027681.1	0.13	0	0.16	0	1.25	2.33
GAIF01027682.1	0	0	0	0	0.93	0

GAIF01027683.1	23.35	13.97	22.24	27.58	3.4	47.64
GAIF01027685.1	0.73	0.49	0.53	0.32	0.04	0.08
GAIF01027686.1	11.86	8.44	8.21	17.37	8.83	7.78
GAIF01027688.1	0.03	0	0	0.18	0.91	0
GAIF01027690.1	3.75	6.91	6.44	3.9	4.16	0.74
GAIF01027691.1	0.02	0	0.12	0.58	0	0
GAIF01027692.1	111.74	46.33	45.74	28.61	0	61.21
GAIF01027693.1	9.98	22.62	19.87	43.45	27.9	4.29
GAIF01027696.1	0	0	0	0	0.16	0
GAIF01027697.1	0	0	0	0.43	0.53	0
GAIF01027699.1	0	0	0.1	1.88	2.41	0.1
GAIF01027701.1	0	0	0	0.24	0.57	0
GAIF01027702.1	0	0.07	0	0	0	0
GAIF01027703.1	0.19	0.15	0	2.25	11.21	0.28
GAIF01027705.1	18.72	17.59	19.89	46.01	25.73	8.33
GAIF01027706.1	2.21	2.24	0.59	7.16	5.22	0.92
GAIF01027707.1	6.6	8.39	8.8	9.77	5.22	5.24
GAIF01027709.1	0	0	0	0.1	0.52	0.09
GAIF01027710.1	64.55	84.88	39.86	20.33	53.37	4.45
GAIF01027713.1	0	0	0.06	0	0	0
GAIF01027714.1	0.34	0.17	0	0	0.81	0.36
GAIF01027715.1	0.05	0	0	0	0.96	0
GAIF01027716.1	8.77	3.62	3.86	15.24	21.03	11.16
GAIF01027717.1	0	0	0	0.08	0	0
GAIF01027718.1	4.86	11.56	0.99	16.67	36.86	0.75
GAIF01027720.1	7.77	8.79	8.39	31.08	24.83	3.13
GAIF01027722.1	4.04	6.78	5.83	7.37	4.72	0.38
GAIF01027723.1	31	52.38	13.8	133.1	185.35	8.46
GAIF01027724.1	0.53	0.48	0	1.13	2.76	0
GAIF01027725.1	23.81	24.72	8.44	20.69	40.46	16.03
GAIF01027726.1	1.84	0.33	0.78	1.42	1.97	0.34
GAIF01027727.1	1.14	0.25	0.07	4.48	18.47	0.68
GAIF01027728.1	3.83	3.33	4.2	1.78	0.46	1.27
GAIF01027730.1	0.05	0	0	0.31	0.06	0
GAIF01027731.1	0	0.27	0	1.63	5.84	0
GAIF01027732.1	0.82	0.36	1.26	0.57	0	1.08
GAIF01027733.1	0	0.01	0.23	0.31	0	0.08
GAIF01027734.1	76.54	109.87	32.94	273.53	274.37	4.98
GAIF01027735.1	0	0	0	0	0.59	0
GAIF01027736.1	27.83	40.65	17.89	30.67	79.57	13.09
GAIF01027737.1	8.19	16.17	13.1	2.41	2.9	1.54
GAIF01027740.1	262.59	140.27	224.04	167.88	123.14	180.89
GAIF01027741.1	2.4	0	1.14	0	0	0
GAIF01027742.1	0.05	0	0	0.27	0	0

GAIF01027744.1	0	0	0.04	0	0	0
GAIF01027745.1	14.97	24.96	13.03	16.02	16.09	2.95
GAIF01027747.1	0.09	0.1	0.29	0.11	0.4	0
GAIF01027748.1	2.89	2.93	2.71	13.57	15.01	2
GAIF01027749.1	0	0	0.06	0	0	0
GAIF01027751.1	2013.3	2447.41	2762.56	1339.25	604.43	391.98
GAIF01027753.1	0	0	0	0.16	0	0
GAIF01027755.1	0.9	0	0	7.91	26.81	0.69
GAIF01027758.1	1.42	1.97	0	2.55	1.27	0.89
GAIF01027761.1	4.28	4.08	2.2	2.33	1.53	0.22
GAIF01027762.1	1.03	0.22	0.6	1.12	2.24	0.53
GAIF01027763.1	0.1	0.11	0	0.8	2.83	0
GAIF01027764.1	5.32	5.24	1.91	3.37	1.7	0
GAIF01027765.1	11.59	23.47	21.52	29	23.49	3.94
GAIF01027767.1	0	0.12	0	0	0	0
GAIF01027768.1	0	0	0	0.1	0.43	0.09
GAIF01027769.1	23.48	18.76	11.13	53.91	34.79	9.5
GAIF01027770.1	0	0.22	0.05	0.06	0	0
GAIF01027771.1	0	0.21	0	0	0	0
GAIF01027772.1	0	2.29	0.41	0	0	0
GAIF01027773.1	1.74	0.18	0.27	0.19	0.9	0.27
GAIF01027774.1	128.48	82.59	70.36	17.46	8.74	7.3
GAIF01027775.1	0	0	0.04	0	0	0
GAIF01027777.1	0	0	0	0	0.3	0
GAIF01027778.1	0	0	0	0.53	0.26	0
GAIF01027779.1	2.85	1.44	0.18	3.82	4.14	2.16
GAIF01027780.1	0	0	0	0	0	0.19
GAIF01027781.1	118.63	87.15	47.09	87.73	53.76	21.11
GAIF01027782.1	0	0	0	0	0.07	0.07
GAIF01027783.1	0	0	0	0	0.03	0
GAIF01027784.1	0	0	0	0	1.94	0.18
GAIF01027785.1	18.34	23.55	18.43	34.88	20.54	8.82
GAIF01027786.1	0.39	0.12	0	1.04	2.02	0.12
GAIF01027787.1	0	0.8	0	0	0.05	0
GAIF01027788.1	2.99	4.57	0	5.83	6.42	0
GAIF01027789.1	0	0	0	0	0.05	0
GAIF01027790.1	60.45	24.48	24.4	83.93	372.41	33.24
GAIF01027791.1	0	0.1	0	0	0.22	0
GAIF01027793.1	0	0.05	0	0	0.06	0
GAIF01027794.1	24.71	25.65	30.45	56.26	31.12	19.33
GAIF01027795.1	0	0	0	1.53	11.34	0
GAIF01027797.1	40.66	4.22	1.79	36.04	60.45	8.78
GAIF01027799.1	0	0.05	0	0	0	0.81
GAIF01027800.1	3.02	1.21	0.36	9.09	11.98	2.82

GAIF01027801.1	0	0	0	0.43	0.66	0
GAIF01027802.1	0.74	2.24	1.02	3.06	3.56	0.67
GAIF01027803.1	9.14	7.08	4.98	2.67	1.14	1.3
GAIF01027804.1	0	0.14	0	0	0	0
GAIF01027805.1	0	0.07	0	0.52	2.01	0
GAIF01027807.1	0.5	0.11	0	0	0	0
GAIF01027808.1	0.03	0	0.03	0	0.17	0
GAIF01027809.1	77.28	114.33	45.85	211.92	176.12	11.47
GAIF01027810.1	4.37	0.44	0.45	13.46	31.5	6.01
GAIF01027811.1	24.74	23.88	9.44	25.17	27.02	2.81
GAIF01027814.1	1.31	0.74	0.18	5.07	14.09	0.54
GAIF01027815.1	0.47	0	0.1	0.11	0.46	0.1
GAIF01027818.1	0	0	0	0.86	0.87	0.34
GAIF01027820.1	5.07	2.19	7.21	13.04	9.28	43.49
GAIF01027821.1	0	0	0	0	0.66	0
GAIF01027824.1	3.43	9.22	7.87	5.82	5.38	1.27
GAIF01027825.1	0	0	0	0.16	0.17	0
GAIF01027826.1	0	0	0	0.75	0	0
GAIF01027827.1	0.44	0.32	0	0.17	0.18	0.16
GAIF01027828.1	0	0.16	0	0.17	0	0.08
GAIF01027829.1	0	0	0	0	0.12	0
GAIF01027830.1	1.18	0.13	0	5.06	18.12	0.39
GAIF01027831.1	5.4	8.08	7.69	11.06	10.47	2.41
GAIF01027832.1	19.02	15.4	0	21.96	41.2	0
GAIF01027833.1	0.06	0	0	0	0.07	0
GAIF01027834.1	0	0	0	0.33	0	0
GAIF01027835.1	19.78	25.13	11.84	56.74	23.61	4.8
GAIF01027836.1	5.31	4.72	5.71	0.15	0.16	0.22
GAIF01027837.1	0.13	0.43	0.15	1.85	4.31	0.29
GAIF01027840.1	23.98	22.16	16.45	10.16	6.2	2.64
GAIF01027841.1	0.42	0	0	1.58	3.15	0
GAIF01027842.1	0	0	0	0	0.06	0
GAIF01027843.1	0	0	0.12	0	0	0
GAIF01027844.1	0.03	0	0	0	0	0.02
GAIF01027845.1	0.57	0.39	0.16	0.08	0.09	0
GAIF01027846.1	0	0	0	0.27	0.27	0
GAIF01027848.1	21.42	19.08	24.27	23.1	15.44	11.13
GAIF01027849.1	22.09	15.2	39.82	47.43	17.63	54.84
GAIF01027850.1	393.87	394.97	406.09	133.77	149.13	91.34
GAIF01027851.1	1.04	0.69	0	1.39	5.76	0.09
GAIF01027852.1	1.38	0	0	0	0.65	0.5
GAIF01027853.1	0	0	0	0	0.5	0
GAIF01027856.1	12.71	13.79	19.22	26.21	23.22	19.85
GAIF01027857.1	3.25	2.53	1.33	6.42	10.22	1.98

GAIF01027858.1	0	0.3	0.41	0.11	0	0.21
GAIF01027861.1	156.44	114.19	120.17	128.62	63.15	31.75
GAIF01027862.1	0.71	0.4	0	8.75	31.23	0.87
GAIF01027866.1	2122.83	673.2	740.9	1338.01	1800.15	5888.59
GAIF01027870.1	2.55	15.37	0	26.57	11.16	7.61
GAIF01027871.1	54.91	81.4	52.97	80.71	29.44	5.77
GAIF01027873.1	0	0	0.07	1.06	5.53	0.06
GAIF01027874.1	3.66	3.17	2.48	11.15	14.83	0.77
GAIF01027876.1	0	0	0	0	0.64	0
GAIF01027879.1	0	0.67	0.27	0	0	1.22
GAIF01027880.1	0.33	0	0.35	0.24	1.63	0
GAIF01027882.1	0	1.1	0.51	0	0	0.09
GAIF01027883.1	21.37	31.79	25.45	79.66	71.74	12.3
GAIF01027884.1	7.29	10.79	7.68	14.68	10.12	2.45
GAIF01027885.1	1.8	1.03	0.29	2.68	3.69	0.47
GAIF01027887.1	0.03	0.03	0.01	0.01	0	0.05
GAIF01027888.1	3.52	1.41	0.09	13.65	33.73	1.05
GAIF01027889.1	5.08	0.95	0.41	7.08	29.22	17.16
GAIF01027892.1	31.37	48.91	21.41	154.61	70.08	16.09
GAIF01027893.1	2.35	0.22	0.09	7.33	34.97	1.07
GAIF01027894.1	0	0	0.07	0	0	0
GAIF01027895.1	0	0	0	0.29	0.33	0
GAIF01027896.1	100.68	74.94	62.25	179.24	175.43	37.79
GAIF01027897.1	11.92	5.07	8.27	5.75	8.01	43.09
GAIF01027898.1	0	0.07	0	0.39	2.93	0
GAIF01027899.1	0.51	0.8	0.62	0.46	0.41	0.13
GAIF01027900.1	0.31	0.06	0.17	2.96	19.34	1.13
GAIF01027901.1	0.8	0.47	0.13	3.83	11.11	0.07
GAIF01027902.1	0	0	0	0	0	0.3
GAIF01027904.1	0	0	0	0.62	0.94	0.21
GAIF01027905.1	0.27	0.03	0.07	0.21	1.51	0
GAIF01027907.1	20.98	11.41	0	24.35	0	0
GAIF01027908.1	0.46	1.68	0.17	2.69	0.74	0.68
GAIF01027911.1	0	0.26	0	0.33	0	0
GAIF01027912.1	274.08	78.44	65.04	383.7	468.71	313.35
GAIF01027913.1	1.07	0	0.45	0.72	0	0.01
GAIF01027914.1	16.46	24.23	10.65	6.15	3.21	0.12
GAIF01027915.1	0	0	0	0.68	0.51	0
GAIF01027916.1	36.66	77.69	71.96	105.48	95.08	25.92
GAIF01027917.1	5.37	2.17	1.64	9.27	16.21	9.01
GAIF01027918.1	0.69	0.75	0.25	8.91	30.19	0.25
GAIF01027919.1	3.3	2.39	0.87	12.25	19.3	1.48
GAIF01027920.1	0.15	0	0.09	0.28	4.78	0
GAIF01027922.1	51.68	61.27	24	109.08	103.54	20.59

GAIF01027923.1	8.15	6.55	5.75	16.36	32.37	19.98
GAIF01027924.1	0.06	0.43	0	0.42	0.22	0
GAIF01027925.1	11.14	28.91	20.21	33.51	35.67	4.13
GAIF01027928.1	20.79	10.96	6.43	34.04	46.06	22.4
GAIF01027929.1	3.27	2.66	1.65	4.2	8.24	2.08
GAIF01027930.1	51.44	27.81	40.75	84.69	82.41	147.54
GAIF01027931.1	0.38	0.05	0.09	0.73	0.22	0.37
GAIF01027932.1	0	0	0	0.11	0.11	0
GAIF01027933.1	0	0	0	0.32	0	0
GAIF01027934.1	0	0.09	0	0.37	0.77	0
GAIF01027935.1	0	0	0	0.73	0.15	0
GAIF01027937.1	7.83	17.16	8.37	33.4	29.77	1.9
GAIF01027938.1	0.89	1.11	0	3.78	7.92	0
GAIF01027940.1	0	0.05	0	0	0	0
GAIF01027941.1	0.03	0	0	0.05	0.08	0
GAIF01027943.1	7.8	0.63	1.35	2.38	0.3	0
GAIF01027944.1	0.08	0	0	0.1	0.1	0
GAIF01027945.1	0	0.25	0	0.78	2.85	0.05
GAIF01027946.1	0	0	0	0.73	0	0.61
GAIF01027947.1	12.77	15.46	4.34	31.73	40.68	2.15
GAIF01027949.1	0.07	0	0	0	0.08	0
GAIF01027950.1	0.04	0.14	0	0.3	0.05	0
GAIF01027951.1	9.31	7.36	14.67	13.1	10.18	10.78
GAIF01027952.1	0	2.28	1.07	0.22	2.59	0
GAIF01027953.1	0.39	0.23	0.09	1.4	5.06	0.24
GAIF01027954.1	0	0.05	0	0	0	0
GAIF01027955.1	14.05	16.27	10.54	30.08	24.88	5.67
GAIF01027956.1	37.34	26.35	22.8	46.62	28.63	5.43
GAIF01027959.1	0.92	1.17	1.21	0.86	0.36	0.69
GAIF01027960.1	3560.4	3751.91	6300.93	1758.56	1451.2	5768.97
GAIF01027962.1	0	0	0	0	0.08	0
GAIF01027963.1	4.67	3.79	5.35	24.17	21.9	17.58
GAIF01027966.1	0	0	0.14	0.15	0	0
GAIF01027967.1	26.65	28.22	23	49.81	55.14	19.52
GAIF01027968.1	13.86	14.23	9.82	24.93	34.23	24.99
GAIF01027969.1	0	0	0	0	0.26	0
GAIF01027970.1	30.33	33.46	17.63	89.73	36.03	6.55
GAIF01027972.1	1.08	0.51	0	2.7	13.23	0.85
GAIF01027974.1	1.24	8.44	6.19	4.13	2.44	1.04
GAIF01027975.1	16.95	19.06	11.48	33.5	16.77	5.76
GAIF01027976.1	0.05	0	0	0.49	0.71	0
GAIF01027977.1	43.49	54.79	68.16	43.36	29.44	29.02
GAIF01027978.1	0.38	0	0	0.29	0.98	0
GAIF01027979.1	7.59	1.56	0.24	19.41	64.16	12.86

GAIF01027981.1	4.39	1	5.13	0.57	0.6	1.56
GAIF01027982.1	0.11	0	0	0	0	0
GAIF01027983.1	5.77	4.42	1.36	20.37	30.74	4.48
GAIF01027984.1	0.41	0.54	0.88	0.36	0.57	0.05
GAIF01027985.1	0	0	0	0	0.22	0
GAIF01027986.1	3.8	0.22	0.44	3.94	20.18	6.83
GAIF01027987.1	407.48	215.78	375.13	486.96	373.59	3116.81
GAIF01027989.1	4.34	2.02	1.36	11.15	8.45	2.39
GAIF01027992.1	50.42	49.36	20.79	75.45	52.36	18.62
GAIF01027993.1	0.45	0.2	0.49	0.99	2.27	0.25
GAIF01027994.1	22.52	9.12	8.81	32.34	32.73	69.63
GAIF01027995.1	0.58	2.15	0.26	1.48	10.9	0
GAIF01027996.1	9.81	7.55	15.55	29.81	15.84	12.64
GAIF01027999.1	2.43	0.97	0.21	7.06	16.62	0.56
GAIF01028001.1	0	0	0	0	0.12	0
GAIF01028002.1	0.11	0.12	0.12	0.38	1.43	0
GAIF01028003.1	17.85	29.09	22.04	31.46	18.94	5.76
GAIF01028004.1	0	0	0	0.98	2.68	0
GAIF01028007.1	0	0	0.03	0	0.03	0
GAIF01028010.1	0	0	0	1.47	1.07	0.14
GAIF01028011.1	0.05	0	0.23	2.27	8.11	0.06
GAIF01028012.1	0.28	0	0	0	0.45	0.31
GAIF01028014.1	0.01	0	0.01	0	0	0
GAIF01028016.1	0.08	0	0	0	0	0
GAIF01028017.1	0.02	0.04	0	0.05	0.07	0
GAIF01028018.1	0.06	0	0	0.03	0.08	0
GAIF01028019.1	15.85	3.62	12.19	8.08	4.01	7.34
GAIF01028020.1	0.04	0	0.05	0.1	0.05	0
GAIF01028021.1	12.33	9.06	27.43	8.46	0.33	4.16
GAIF01028022.1	0.03	0.15	0	0.04	0	0
GAIF01028023.1	0.06	0.1	0.03	0.81	7.51	0.31
GAIF01028026.1	0	10.69	16.63	0	7.88	10.84
GAIF01028027.1	0.58	0.16	0	0.67	5.52	0
GAIF01028028.1	0	0	0	0	0.07	0
GAIF01028029.1	0.12	0	0	0.02	0.35	0
GAIF01028030.1	0.06	0.03	0.03	0.26	1.54	0.09
GAIF01028031.1	13.78	8.72	7.9	20.08	21.01	10.08
GAIF01028032.1	109.54	141.65	210.7	131.65	96.25	37.44
GAIF01028034.1	0.39	0.22	0.17	0	7.32	0.37
GAIF01028039.1	0	0.03	0	0.03	0.34	0
GAIF01028040.1	0.83	0.9	0	0.12	0.25	0
GAIF01028041.1	0	0	0	0	0.07	0
GAIF01028042.1	7.15	5	3.19	11.11	15.02	2.49
GAIF01028043.1	1.2	0	0	0	0	0

GAIF01028044.1	0	0	0	0.03	0.18	0
GAIF01028045.1	605.05	544.69	274.68	475.08	928.55	245.67
GAIF01028047.1	0	0.13	0	0.14	0.3	0
GAIF01028048.1	0	0	0	0	0.52	0
GAIF01028049.1	78.61	79.17	45	7.34	8.41	15.71
GAIF01028050.1	0	0	0.76	0	0	0
GAIF01028051.1	2.97	4.28	1.22	0.27	0.14	0
GAIF01028052.1	2.24	6.89	4.2	0.61	0.32	0.15
GAIF01028053.1	72.03	31.59	42.37	12.03	17.54	10.02
GAIF01028054.1	0.84	0.83	0.08	6.71	13.08	0.34
GAIF01028055.1	10.37	19.7	23.23	2.98	2.06	0.85
GAIF01028056.1	2107.51	1864.68	4651.18	737.87	396.57	1456.14
GAIF01028060.1	1.12	1.3	1.59	2.25	3.23	1.33
GAIF01028061.1	3.68	0	0	0	4.04	5.93
GAIF01028062.1	0	2.1	1.77	0	0	0.06
GAIF01028063.1	35.88	44.65	36.51	41.19	20.59	10.77
GAIF01028064.1	6.1	10.15	5.07	25.77	20.45	2.86
GAIF01028066.1	0.23	0.39	0.27	0.23	0.15	0.07
GAIF01028068.1	50.57	62.24	35.96	54.29	50.78	15.32
GAIF01028069.1	8.56	8.7	10.96	13.91	18.78	8.57
GAIF01028070.1	0	0	0	0.18	0	0
GAIF01028071.1	0	0.34	0	0	0	0
GAIF01028073.1	0	0.36	0	0	0.4	0
GAIF01028075.1	26.41	21.92	31.04	46.69	21.17	23.25
GAIF01028079.1	2344.69	1114.46	1732.63	1489.85	1811.49	7087.78
GAIF01028080.1	0	0.12	0	0	0.2	0
GAIF01028082.1	0.61	0.65	0.69	0.69	1.33	0.27
GAIF01028084.1	18.43	23.11	17.8	96.25	85.76	5.06
GAIF01028085.1	9.1	19.83	13.31	18.02	16.1	4.85
GAIF01028086.1	33.07	42.74	15.88	171.43	181.07	35.45
GAIF01028087.1	176.06	59.84	164.36	194.12	106.12	105.58
GAIF01028089.1	31.13	60.58	34.55	75.23	67.83	18.13
GAIF01028090.1	81.58	84.04	226.11	33.1	12.27	102.55
GAIF01028091.1	0	0	0	0	0.84	0
GAIF01028092.1	0.27	0.79	0.4	5.56	16.73	0.2
GAIF01028094.1	0	0	0	0.05	0.16	0
GAIF01028096.1	0.06	0.06	0	0.38	2.66	0.3
GAIF01028097.1	0	0	0	0.08	0	0
GAIF01028098.1	10.68	9.36	0	10.26	17.18	14.09
GAIF01028099.1	0.27	0	0.18	0	0	0
GAIF01028100.1	0.63	0.27	0.14	1.31	0.6	0.14
GAIF01028101.1	1.84	0.19	0.38	0.31	4.53	0.68
GAIF01028102.1	13.79	6.29	5.14	26.27	22.82	8.58
GAIF01028103.1	12.68	21.45	5.94	28.53	45.35	13.98

GAIF01028105.1	0	0	0	0	1.68	0
GAIF01028107.1	11.37	11.44	10.18	25.41	44.33	6.16
GAIF01028108.1	3.31	4.49	3.4	14.67	16.99	2.22
GAIF01028109.1	0.33	0.52	0	0.18	0.65	0
GAIF01028110.1	0	0	0	0.65	1.89	0.28
GAIF01028112.1	0.43	0.13	0.27	0.21	0.67	0
GAIF01028113.1	0.06	0	0	0.07	0	0
GAIF01028114.1	0.22	0	0	0.38	0.91	0.89
GAIF01028115.1	0	0	0	0	0	0.24
GAIF01028116.1	0	0	0	0	4.22	0
GAIF01028118.1	8.02	9.13	5.56	3.77	3.05	0.47
GAIF01028120.1	32.71	31.74	49.49	46.58	18.41	34.8
GAIF01028122.1	223.82	240.89	687.53	49.8	52.6	325.45
GAIF01028123.1	0	0	0	0	0.19	0
GAIF01028125.1	0	0	0	0	0	0.04
GAIF01028126.1	1.78	1.8	0.42	2.22	2.76	0.7
GAIF01028127.1	3.29	2.73	2.1	0.91	0.6	0
GAIF01028133.1	0.04	0.04	0.08	0.73	2.45	0.04
GAIF01028134.1	0	0	0	0.13	0	0
GAIF01028135.1	23.29	6.31	6.37	3.05	3.32	11.34
GAIF01028136.1	0	0.16	0	0	0	0
GAIF01028137.1	0	0	0	0	1.13	0
GAIF01028141.1	0.02	0.03	0.03	0.01	0.03	0.03
GAIF01028142.1	0.12	0.03	0.02	0	1.13	0
GAIF01028147.1	42.91	24.68	18.23	138.12	211.93	182.72
GAIF01028148.1	0	0	0	0	0	0.2
GAIF01028149.1	99.26	169.56	260.76	581.91	414.28	137.33
GAIF01028150.1	0.15	0.05	0	0	0	0
GAIF01028151.1	0	0	0	0	0.39	0
GAIF01028152.1	1.61	2.1	0.35	6.91	6.39	0.71
GAIF01028153.1	749.97	638.86	637.87	84.79	1.18	38.33
GAIF01028154.1	0.25	0.07	0.2	1.22	4.69	0.41
GAIF01028155.1	71.13	11.66	5.04	5.67	4.5	1.76
GAIF01028158.1	0.14	0.22	0	0	0.09	0.52
GAIF01028159.1	23.81	20.87	17.02	3.59	1.4	0.39
GAIF01028164.1	0	0	0	0	0.39	0
GAIF01028165.1	0	0	0	0	2.06	0
GAIF01028166.1	0.77	5.89	1.14	36.05	91.02	1.57
GAIF01028168.1	2.12	1.35	0.15	2.98	0.43	0.11
GAIF01028169.1	1.13	1.58	0	1.54	0.72	0.22
GAIF01028170.1	0.42	0.58	0.12	0	0	0
GAIF01028172.1	0	0.08	0	0	0	0
GAIF01028173.1	0	0	0	0	0.05	0
GAIF01028174.1	0	0	0	0.31	0	0

GAIF01028175.1	1.68	0	1.59	0.78	0.31	2.04
GAIF01028177.1	0.32	0.07	0.49	0	0	0
GAIF01028179.1	0	0.87	0.75	0	0	0.21
GAIF01028180.1	0.37	0.13	0.14	1.28	8.86	0.27
GAIF01028181.1	0.08	0	0	0	0	0
GAIF01028182.1	10.82	7.16	7.07	4.97	2.6	1.72
GAIF01028183.1	0.32	0	0	0	0	0
GAIF01028184.1	0	0.04	0.04	0	0.03	0
GAIF01028186.1	3	7.39	0.44	26.46	51.95	0.33
GAIF01028188.1	0.82	0.23	0.08	2.24	30.78	1.67
GAIF01028189.1	64.43	62.53	16.41	214.06	229.93	27.71
GAIF01028190.1	0.39	0.11	0.05	1.01	4.1	0.19
GAIF01028191.1	21.28	35.69	27.78	28.84	11.94	7.29
GAIF01028192.1	0.22	0	0.2	0	2.84	0
GAIF01028194.1	0	0	0	0.49	1.39	0.08
GAIF01028195.1	0	0.62	0.21	0	0	0
GAIF01028196.1	0.7	0.42	0.23	0.62	0	0.09
GAIF01028197.1	831.22	259.34	450.19	553.08	951.48	5996.96
GAIF01028198.1	0.16	0.06	0	0	0	0
GAIF01028201.1	12.02	5.5	9.37	0.61	0.38	1.16
GAIF01028202.1	2.24	1.86	1.15	8.39	12.73	0.66
GAIF01028203.1	3.84	3.19	0.49	8.86	6.96	0.02
GAIF01028204.1	0	0.87	0	0	3.25	1.17
GAIF01028207.1	0	0.05	0	0	0	0
GAIF01028209.1	0	0	0	0.46	1.9	0.11
GAIF01028210.1	8.23	11.5	0.65	15.86	12.01	0.52
GAIF01028211.1	0.22	0.18	0	0	0	0.06
GAIF01028212.1	5.46	4.7	5.9	19.4	34.06	8.02
GAIF01028213.1	0.07	0.16	0.08	0.51	0.53	0
GAIF01028214.1	0	0.06	1.14	0	0	1.13
GAIF01028215.1	7.91	5.62	9.21	28.64	43.37	11.13
GAIF01028217.1	0	0	0	0.18	0.35	0
GAIF01028218.1	0	0.08	0	0	0.18	0
GAIF01028219.1	0.22	0.28	0	0.04	2.92	0.47
GAIF01028220.1	0	0	0	0	0.08	0
GAIF01028221.1	14.45	16.89	15.04	26.99	14.98	5.4
GAIF01028222.1	2.73	1.79	0.59	4.29	8.9	1.76
GAIF01028223.1	0.81	0.72	0.1	1.53	1.18	0.16
GAIF01028224.1	53.28	90.03	52.34	195.93	224.02	28.96
GAIF01028225.1	0.09	0	0.1	3.77	18.26	0.1
GAIF01028226.1	0	0	0	0.01	0.06	0
GAIF01028227.1	0.65	0.45	0.13	2.81	5.4	0.13
GAIF01028229.1	0.32	1.05	0	0	0	0
GAIF01028230.1	38.68	45.23	31.08	49.15	40.63	9.12

GAIF01028233.1	2.38	1.49	0.28	12.74	56.9	0.22
GAIF01028234.1	8.52	19.92	21.97	19.66	11.2	6.66
GAIF01028235.1	0	0	0	0	0.09	0
GAIF01028236.1	0	0	0	0	0.15	0
GAIF01028239.1	0.12	0.2	0.13	0	0	0.13
GAIF01028240.1	1.02	0.41	0	3.84	13.91	3.5
GAIF01028241.1	0	0.1	0	0	0	0.15
GAIF01028243.1	0	1.01	0.96	0	0.19	0.06
GAIF01028245.1	0	0	0.12	0	0.26	0
GAIF01028247.1	2.66	0.8	0.41	7.37	14.2	1.46
GAIF01028248.1	82.05	96.01	94.79	180.92	129.02	43.23
GAIF01028249.1	0	0.25	0	3.52	11.8	0
GAIF01028250.1	0	0	0	0.11	0.14	0.07
GAIF01028253.1	0	17.3	11.79	0	0.39	1.07
GAIF01028254.1	0	0	0.09	0	0	0
GAIF01028255.1	48.23	55.39	35.93	184.19	114.82	19.77
GAIF01028256.1	4950.56	6159.78	10957.84	3299.88	2629.92	9905.74
GAIF01028258.1	9.1	2.24	1.19	9.66	8.8	1.72
GAIF01028259.1	0	0.06	0	0	0.09	0
GAIF01028261.1	0.38	0.29	0.16	0.9	1.15	0
GAIF01028262.1	0.12	0.04	0	0.32	0.38	0
GAIF01028263.1	4.78	2.5	3.71	18.32	29.4	12.66
GAIF01028264.1	0	0.07	0.57	0.07	0	0
GAIF01028266.1	91.81	34.42	16.38	116.91	68.29	7.85
GAIF01028267.1	1.47	1.94	3.98	5.94	3.61	2.89
GAIF01028268.1	0	0	0	0.48	2.15	0
GAIF01028270.1	0.69	0.15	0.69	3.46	5.93	13.62
GAIF01028271.1	0.1	0.11	0	0	0.12	0
GAIF01028273.1	2.11	2.33	1.84	2.05	1.83	1.6
GAIF01028274.1	0.59	0	0.08	1.29	3.43	2.92
GAIF01028275.1	0.01	0	0.01	0.06	0	0
GAIF01028276.1	0.72	0.39	0.31	2.5	6.38	1.66
GAIF01028279.1	0.29	0	0	1.58	0	0.47
GAIF01028283.1	0.14	0	0	0	0.08	0
GAIF01028284.1	0	0	0	0	0.1	0
GAIF01028285.1	47.46	21.43	18.99	31.14	12.4	17.97
GAIF01028286.1	0	0	0	0	1.84	0
GAIF01028288.1	3.12	1.86	1.03	14.08	94.91	4.71
GAIF01028290.1	0.07	0.03	0.05	0.75	0	0.09
GAIF01028292.1	20.35	38.28	13.56	12.35	15.47	2.96
GAIF01028294.1	4.8	13.57	9.08	26.37	17.84	4.97
GAIF01028295.1	1.4	0.17	0	11.76	50.8	0.34
GAIF01028296.1	9.17	12.83	6.59	28.2	38.27	4.31
GAIF01028297.1	0	0	0	0	0.16	0

GAIF01028299.1	44.98	43.68	41.25	126.36	74.2	52.59
GAIF01028300.1	8.07	0.74	0.53	2.38	1.06	0.22
GAIF01028301.1	0	0.24	0.41	0.7	1.27	0
GAIF01028303.1	7.16	8.03	9.77	15.9	4.41	10.48
GAIF01028304.1	0	0	0	0	0	0.3
GAIF01028305.1	0.12	0.19	0	0	0.28	0
GAIF01028306.1	0	0	0	0.09	0	0
GAIF01028307.1	3.86	0.23	0.47	0.5	1.81	0.47
GAIF01028308.1	12.9	10.04	10.98	12.02	8.68	8.22
GAIF01028311.1	0	0.15	0	1.27	1.95	0.15
GAIF01028312.1	2.11	1.93	0.46	22.72	54.7	2.79
GAIF01028313.1	1.16	0.93	0	6.38	17.97	1.19
GAIF01028316.1	0.43	0.47	0.39	1.04	3.71	0.65
GAIF01028317.1	1.16	0	0	0	0	0
GAIF01028318.1	0.07	0	0.07	0.3	0.94	0
GAIF01028322.1	0.58	0	0	0.67	0.22	0.63
GAIF01028323.1	33.28	29.73	51.62	68.51	42.63	37.63
GAIF01028324.1	1.82	0.85	0.14	0.15	0.47	0
GAIF01028325.1	0	0	0	0	0.04	0
GAIF01028326.1	0.81	1.05	1.18	0.71	0.13	0.25
GAIF01028327.1	66	272.52	95.17	19.69	25	2.32
GAIF01028328.1	0.23	0	0	0.18	0.46	0
GAIF01028329.1	0.15	0.08	0	0	1.2	0
GAIF01028330.1	0.13	0	0	0	0.9	0
GAIF01028331.1	0.44	0	0.48	2.03	4.2	0.96
GAIF01028333.1	99.5	80.14	60.83	20.01	8.23	3.34
GAIF01028334.1	0	0	0	0.05	0	0
GAIF01028335.1	4.93	3.66	3.44	4.11	8.37	2.81
GAIF01028339.1	0	1.84	0	0	0	0
GAIF01028340.1	2.12	2.15	3.64	5.37	5.35	0.38
GAIF01028341.1	0	0	0	0	0.11	0
GAIF01028342.1	0.34	0	0	0	0	0.09
GAIF01028343.1	0	0	0	0.05	0	0
GAIF01028344.1	0	0.18	0.25	0	0.1	0
GAIF01028345.1	116.5	60.04	65.31	176.72	77.39	215.12
GAIF01028346.1	0.06	0.03	0	0.07	0.27	0
GAIF01028347.1	0	0.06	0	0	0	0
GAIF01028348.1	0.04	0	0	0	0	0
GAIF01028349.1	0	0.05	0	0	0.06	0.22
GAIF01028353.1	0	0.47	2.01	0	0	0.48
GAIF01028354.1	0.02	0.12	0.08	0	0.52	0.08
GAIF01028356.1	0	0	0	0	0	0.04
GAIF01028357.1	10.21	14.66	21.05	19.88	13.32	10.52
GAIF01028359.1	163.24	184.2	40.66	30.23	17.82	3.45

GAIF01028360.1	13.81	3.97	3.34	10.66	13.2	16.66
GAIF01028362.1	1.91	4.16	1.16	0.3	0.18	0.18
GAIF01028363.1	0.02	0.14	0.02	0.05	0.7	0.01
GAIF01028364.1	0.1	0.1	0.1	0.44	1.38	0.32
GAIF01028365.1	0.96	1.05	0	6.72	17.41	0.15
GAIF01028366.1	0	0	0	0	1.45	0
GAIF01028367.1	0	0.04	0.08	0	0.43	0.16
GAIF01028368.1	0.65	2.13	0.99	0.25	0.31	0
GAIF01028369.1	0	0	0	0	0.07	0
GAIF01028370.1	0.79	1.54	0.52	1.83	5.11	0.35
GAIF01028371.1	0	0.02	0	0	0	0.02
GAIF01028372.1	0.69	3	0.16	3.2	4.04	0.11
GAIF01028373.1	0	0	0	0.41	2.51	0
GAIF01028375.1	0.59	0.14	0	0.39	0.43	0
GAIF01028376.1	21.09	10.82	10.3	19.7	22.23	29.67
GAIF01028377.1	0	0	0	0.18	1.63	0.09
GAIF01028378.1	6.15	9.82	6.02	25.66	21.59	3.05
GAIF01028379.1	11.06	9.68	4.57	19.31	20.21	2.57
GAIF01028380.1	0	0	0	0.63	0.49	0
GAIF01028381.1	1.42	0.39	0	0	0	0.42
GAIF01028382.1	3.73	2.31	3.35	1.34	2.17	2.59
GAIF01028383.1	0	0	0	0	0.16	0
GAIF01028385.1	14.42	11.15	5.56	29.25	27.45	8.16
GAIF01028386.1	223.34	318.58	141.31	0.17	0.18	0.5
GAIF01028389.1	4.59	2.09	0.47	1.24	1.54	0.47
GAIF01028390.1	23.46	19.82	17.56	39.84	27.13	4.24
GAIF01028394.1	129.89	35.86	84.84	114.82	129.75	612.02
GAIF01028396.1	0.47	0.31	1.03	0.11	0	0
GAIF01028397.1	3.61	0	0	4.97	16.87	6.85
GAIF01028398.1	0.22	0	0	0	0.07	0
GAIF01028400.1	0.04	0.04	0.08	0.08	0.13	0.04
GAIF01028401.1	7.69	5.57	2.76	1.01	0.62	0.06
GAIF01028403.1	0	0	0	0	0.09	0
GAIF01028404.1	11.46	12.92	8.73	36.61	59.13	3.77
GAIF01028405.1	0	0	0	0.17	0	0
GAIF01028406.1	213.84	215.68	362.81	22.15	5.98	20.69
GAIF01028407.1	0	0	0	0.68	1.9	0
GAIF01028408.1	0	0	0	0	0	0.07
GAIF01028409.1	0.6	0.39	0	3.73	12.17	1.57
GAIF01028410.1	0	0	0.07	0	0.08	0.07
GAIF01028411.1	0	0	0	0.82	2.09	0
GAIF01028412.1	0	0	0.12	0.13	0	0
GAIF01028413.1	0.16	0	0	0.55	0.27	0.94
GAIF01028414.1	60.49	75.65	106.45	83.91	78.24	145.07

GAIF01028415.1	23.38	9.99	14.13	14.37	14.55	65.28
GAIF01028417.1	38.46	50.16	28.66	45.36	41.8	6.54
GAIF01028418.1	14.17	17.97	20.45	55.24	46.38	8.27
GAIF01028419.1	0.38	0	0	1.52	4.04	0
GAIF01028420.1	3.26	2.26	1.08	9.19	8.76	7.64
GAIF01028421.1	11.66	21.81	14.61	4.88	2.06	0.65
GAIF01028422.1	1.02	0.99	0.22	2.36	4.16	1.46
GAIF01028428.1	0.25	0.09	0.14	0.97	1.3	0
GAIF01028430.1	0	0	0	0	0.82	0
GAIF01028431.1	0.12	0.13	0	0.14	0.5	0
GAIF01028432.1	26.76	17.27	27.51	27.8	16.85	28.27
GAIF01028434.1	56.14	64.91	30.32	103.08	84.91	7.45
GAIF01028435.1	5.24	0	0	31.98	74.37	8.48
GAIF01028436.1	4.32	7.39	3.09	10.66	6.67	2.82
GAIF01028439.1	0.02	0	0	0	0	0
GAIF01028440.1	1.55	2.81	0.95	4.94	11.19	0.76
GAIF01028441.1	269.33	163.77	122.27	121.68	34.49	10.07
GAIF01028442.1	0.28	0.1	0	0.11	0.11	0.31
GAIF01028443.1	0	0	0	0	0.11	0
GAIF01028444.1	0.46	0.48	0.81	2.53	14.54	6.69
GAIF01028446.1	0	0	0	0	0.3	0
GAIF01028447.1	0	0.1	0	0	0.21	0
GAIF01028448.1	0.16	0.03	0.02	0.04	0.35	0.21
GAIF01028450.1	0.34	0.5	0.13	0	0.28	0.13
GAIF01028451.1	16.62	21.94	15.36	82.09	57.36	9.12
GAIF01028452.1	75.58	91.38	87.79	24.93	19.42	30.59
GAIF01028453.1	9.99	8.35	18.38	17.73	16.62	12.06
GAIF01028454.1	1.7	1.56	0.67	0	0.08	0
GAIF01028455.1	1.74	1.3	0.6	0	0.65	0.12
GAIF01028456.1	0	0	0.17	0	0.76	0
GAIF01028457.1	0.37	0.68	0.96	0	0	0.41
GAIF01028458.1	0	0	0	0.28	1.5	0
GAIF01028459.1	1.97	0.11	0.11	6.4	12.32	10.53
GAIF01028460.1	0.76	0.69	1.11	0.44	0.31	1.4
GAIF01028462.1	0	0	0	0	0.48	0
GAIF01028464.1	34.8	39.74	41.22	51.71	29.51	6.13
GAIF01028465.1	9.72	4.35	4.3	11.89	4.95	0.69
GAIF01028466.1	3.21	0.75	0	9.69	57.32	3.93
GAIF01028467.1	0	0.23	0	0.24	0.26	1.01
GAIF01028468.1	0.05	0.09	0.06	0.55	2.07	0
GAIF01028470.1	1.19	0.78	2.35	3.41	3.72	11.11
GAIF01028471.1	28.27	20.68	9.06	48	36.72	13.42
GAIF01028472.1	0	0	0	0.32	0.08	0.14
GAIF01028474.1	0.31	0	0.17	0.18	9	0

GAIF01028476.1	0.01	0	0	0	0	0
GAIF01028477.1	0	0	0	0.19	0	0
GAIF01028479.1	182.76	64.37	97.21	131.29	90.37	381
GAIF01028480.1	1.32	0.16	0.12	3.07	14.66	7.8
GAIF01028483.1	5.64	3.35	1.11	14.45	84.38	0.55
GAIF01028486.1	547.03	292.65	186.58	193.64	120.55	72.67
GAIF01028487.1	240.23	96.94	84.27	180.13	168.94	272.58
GAIF01028489.1	8.36	6.32	6.39	9.21	13.97	13.53
GAIF01028490.1	1.06	0	0.29	4.31	14.69	1.85
GAIF01028491.1	0.04	0.09	0	0.23	1.34	0
GAIF01028493.1	1.92	2.58	0.34	15.47	20.73	0
GAIF01028495.1	4947.3	18121.64	17796.84	3481.86	4771.59	1732.47
GAIF01028497.1	0	0.09	0	0.1	0.42	0
GAIF01028498.1	0.09	0.19	0	0.4	2.19	0
GAIF01028500.1	11.47	7.32	12.47	12.93	7.3	15.56
GAIF01028502.1	0	0	0	1.41	5.39	0
GAIF01028504.1	0	0	0	0.27	0	0.26
GAIF01028505.1	15.04	14.96	16.59	31.89	41.99	5.03
GAIF01028506.1	2.37	3.08	1.57	2.11	3.08	0.7
GAIF01028507.1	12.43	10.94	20.9	33.31	29.36	44.02
GAIF01028508.1	0.63	0	0	1.18	1.34	0.6
GAIF01028509.1	0.16	0.08	0	0.02	1.18	0
GAIF01028510.1	2.52	0.41	0	12.28	48.76	2.08
GAIF01028511.1	1.66	0.55	0.85	2.69	3.02	3.96
GAIF01028512.1	374.93	1102.98	1274.78	252.7	298.15	359.9
GAIF01028513.1	0.29	0	0	1	2.5	0
GAIF01028514.1	1.12	0.6	0.92	0.69	0	0.47
GAIF01028515.1	10.25	4.02	5.55	19.42	26.97	12.42
GAIF01028517.1	0.11	0	0	0	0	0
GAIF01028519.1	26.1	22.35	53.47	94.61	96	52.42
GAIF01028520.1	1.05	0.24	0.12	8.65	32.33	5.93
GAIF01028521.1	0.07	0.16	0	1.3	3.33	0
GAIF01028523.1	16.41	14.19	11.24	35.32	19.83	7.45
GAIF01028524.1	1.2	0	0.05	1.92	8.1	5.24
GAIF01028525.1	3.99	2.4	2.61	22.87	51.68	10.86
GAIF01028526.1	0	0	0	0	0.1	0
GAIF01028530.1	0.11	0	0	0	1.03	0
GAIF01028531.1	0	0	0.05	0	0.06	0
GAIF01028533.1	0	0	0	0	0.15	0
GAIF01028535.1	0	0	0	0.16	0	0.08
GAIF01028538.1	0.08	0.26	0.04	0.11	0.8	0
GAIF01028539.1	0.18	0.12	0	2.56	13.34	0.2
GAIF01028540.1	0.17	0.03	0.06	0.99	4.53	0.16
GAIF01028543.1	0.05	0	0.18	0.19	0.26	0.18

GAIF01028545.1	0.18	0.07	0	0.38	0.65	0
GAIF01028547.1	2.43	0.58	0.07	24.94	84.39	1.39
GAIF01028548.1	70.83	18.05	32.69	48.73	33.89	99.43
GAIF01028549.1	13.19	4.78	9.66	17.04	10.65	72.37
GAIF01028551.1	1.12	1.26	2.19	1.38	1	0.01
GAIF01028552.1	2.7	0.49	0.49	20.18	64.64	20.24
GAIF01028553.1	1.5	0.38	0.19	7.99	22.07	1.17
GAIF01028555.1	1	0.09	0.12	1.2	0.24	0
GAIF01028560.1	11.5	19.08	0.6	51.53	28.86	1.75
GAIF01028561.1	0.68	0.78	0.41	2.49	1.83	0.17
GAIF01028562.1	0.14	0	0.15	0.16	0.17	0.61
GAIF01028563.1	74.13	32.16	4.2	29.15	67.88	10.75
GAIF01028564.1	1.15	1.18	0.21	2.18	4.32	0.04
GAIF01028567.1	0.05	0.35	0.06	0.31	2.27	0.18
GAIF01028568.1	0.36	1.81	0.26	1.18	1.89	0
GAIF01028570.1	2.53	4.44	2.32	3.43	1.52	0
GAIF01028571.1	11.26	4.47	3.35	18.91	19.45	0.97
GAIF01028572.1	0	0	0	0.9	8.81	1.89
GAIF01028573.1	51.55	64.15	23.84	9.45	8.31	3.44
GAIF01028574.1	0	0	0	12.99	2.95	3.2
GAIF01028575.1	0	0	0	0	0.21	0
GAIF01028576.1	0.59	0.12	0.04	1.16	10.58	1.34
GAIF01028578.1	1.48	0.06	0.13	0.12	0.07	0.02
GAIF01028582.1	0.78	0	0.19	2.21	7.19	0.48
GAIF01028583.1	0	0	0	0	1.35	0
GAIF01028584.1	4.12	4.61	0	11.63	13.71	5.28
GAIF01028585.1	0.4	0	0.24	0.16	0.32	0.11
GAIF01028586.1	4.96	2.05	12.33	13.05	10.09	2.06
GAIF01028587.1	0	0	0	0.05	0	0
GAIF01028590.1	0	0	0	0	0.15	0
GAIF01028592.1	0.04	0	0.05	0	1.74	0
GAIF01028597.1	1.14	0.4	0.24	0.55	4	2.42
GAIF01028598.1	31.56	63.75	48.09	92.54	86.56	21.15
GAIF01028599.1	0	0	0	0	0.11	0
GAIF01028600.1	0.33	0	0	0	0	0
GAIF01028601.1	0.53	0	0.18	0	0	0.66
GAIF01028602.1	0.05	0	0.05	0.13	0.19	0
GAIF01028603.1	20.45	25.98	18.99	52.95	57.76	10.33
GAIF01028604.1	51.16	35.75	37.65	31.8	13.01	12.97
GAIF01028605.1	0	0	0	0.39	2.12	0.09
GAIF01028608.1	1.72	2.01	1.25	1.46	1.31	0.54
GAIF01028609.1	2.59	3.36	1.48	14.69	21.93	0.96
GAIF01028610.1	0.18	0	0	0	0	0.07
GAIF01028611.1	0.02	0	0.03	0.28	1.02	0.03

GAIF01028614.1	0	0	0	0.18	2.54	0.17
GAIF01028616.1	0	0.75	0.76	0	0	0.41
GAIF01028617.1	0.27	0	0	0.15	1.12	0
GAIF01028618.1	0	0	0	0.01	0	0
GAIF01028619.1	6.35	5.22	3.32	22.89	32.17	1.12
GAIF01028620.1	1.94	2.63	1.46	2.53	2.33	0.4
GAIF01028621.1	22.8	27.44	21.01	60.32	32.8	4.49
GAIF01028623.1	12.5	9.42	6.93	5.43	3.81	1.87
GAIF01028624.1	4.88	12.26	12.53	12.78	8.55	3.43
GAIF01028625.1	0	0.04	0.18	0.09	0	0
GAIF01028626.1	0.33	0.12	0.12	1.41	1.33	0
GAIF01028627.1	0.69	0.11	0	0	0.04	0
GAIF01028628.1	10.95	6.78	0	4.49	10.95	0
GAIF01028630.1	0.12	0	0	0	0.1	0
GAIF01028631.1	23.43	13.47	12.37	50.05	48.5	6.88
GAIF01028634.1	0.47	0.43	0.04	2.14	8.48	0.12
GAIF01028636.1	0	0.14	0	0	0	0
GAIF01028637.1	0	0	0.13	0	1.31	0
GAIF01028639.1	9.17	16.41	24.04	44.47	27.58	14.67
GAIF01028640.1	0.01	0	0	0	0	0
GAIF01028642.1	23.89	15.41	17.13	33.63	34.58	23.73
GAIF01028643.1	1.57	1.23	0.88	5.64	7.36	0.42
GAIF01028644.1	1.79	0.59	0	6.34	21.31	0.37
GAIF01028645.1	10.09	12.84	10.73	2.67	2.12	0
GAIF01028648.1	37.36	48.52	71.6	22.73	1.16	10.19
GAIF01028649.1	2.23	1.74	0.3	4.7	3.53	0.08
GAIF01028650.1	0	0.17	0.17	0	0	0
GAIF01028653.1	0	0	0.08	0.16	0.04	0
GAIF01028654.1	0.19	0	0	0	0.12	0.07
GAIF01028655.1	3.01	3.68	3.92	0.55	0.34	0
GAIF01028656.1	0	0	0	0.09	0.09	0
GAIF01028660.1	1.6	2.38	1.23	1.92	1.09	0.06
GAIF01028661.1	0	0	0	0	0.05	0
GAIF01028663.1	10.62	22.96	15.55	35.72	30.12	3.32
GAIF01028664.1	0.18	0	0	0.38	1.46	0.35
GAIF01028665.1	36.48	85.22	34.7	80.11	76.4	7.75
GAIF01028666.1	5.75	8.12	5.76	8.23	9.67	0.59
GAIF01028667.1	0	0.14	0	0.52	0	0
GAIF01028668.1	80.68	48.31	9.53	13.68	32.46	2.93
GAIF01028671.1	15.08	22.1	20.57	27.47	37.99	4.82
GAIF01028672.1	0	0	0.26	0	1.88	0.27
GAIF01028673.1	0	0	0	0.06	0	0
GAIF01028674.1	37.8	41.53	37.75	97.6	47.08	18.9
GAIF01028675.1	7.75	7.52	5.27	18.63	6.32	7.02

GAIF01028676.1	0.03	0	0	0.94	7.77	0.1
GAIF01028678.1	0.01	0.02	0.05	0.09	0.62	0.46
GAIF01028679.1	0.05	0.03	0	0.06	1.15	0.04
GAIF01028680.1	0	0	0	0	0.12	0.11
GAIF01028681.1	137.96	161.54	200.17	146.56	103.64	113.31
GAIF01028682.1	30.89	26.4	15.39	84.81	72.62	8.11
GAIF01028683.1	20.3	19.29	24.9	28.7	15.38	12.03
GAIF01028684.1	28.97	20.64	28.26	25.15	30.4	15.67
GAIF01028685.1	11.78	6.11	3.38	42.26	36.6	4.19
GAIF01028686.1	3.38	1.73	1.52	6.41	3.82	4.34
GAIF01028687.1	1.17	1.51	0.63	7.42	12.26	0.1
GAIF01028690.1	0	0	0.03	0	0	0
GAIF01028692.1	0	1.4	1.34	0	0	0.27
GAIF01028693.1	4.74	2.13	3.12	11.41	14.62	5.06
GAIF01028694.1	13.09	18.13	7.73	22.2	27.55	1.1
GAIF01028695.1	40.37	37.01	43.28	49.24	7.18	2.24
GAIF01028696.1	6.55	4.78	5.26	21.24	34.8	37.26
GAIF01028697.1	15.13	14.67	6.44	24.78	30.5	5.1
GAIF01028698.1	0	0.49	0	0.47	1.83	0.31
GAIF01028699.1	0	0	0.13	0	0	0
GAIF01028700.1	0.12	0	0	0	0	0
GAIF01028701.1	28.8	21.03	19.68	17.53	12.9	13.23
GAIF01028702.1	0.02	0	0	0	0.02	0
GAIF01028704.1	0.12	0	0.07	0.21	0.51	0.27
GAIF01028705.1	19.75	1.19	2.88	28.12	36.42	77.1
GAIF01028706.1	0	0	0	0	0.08	0
GAIF01028707.1	0.47	0.13	0	0.27	7.73	0
GAIF01028709.1	0	0	0	0.05	0.05	0
GAIF01028710.1	0.05	0	0	0	0	0
GAIF01028711.1	0	0	0	0.02	0	0
GAIF01028712.1	0	0.08	0	0.08	0	0
GAIF01028713.1	0.73	1.03	0.83	0.12	0	0.06
GAIF01028715.1	8.16	5.4	7.53	10.62	10.97	2.38
GAIF01028717.1	0.5	0.23	0.16	4.77	11.17	0.08
GAIF01028718.1	124.24	98.98	65.57	118.89	144.5	19.48
GAIF01028720.1	0.4	0	0	0	0	0
GAIF01028721.1	2.4	4.06	4.1	0.93	1.6	0.29
GAIF01028722.1	84.8	174.29	103.48	152.05	200.71	22.57
GAIF01028724.1	0	0	0	0	0.14	0
GAIF01028725.1	18.86	18.69	16.62	52.61	43.29	8.4
GAIF01028728.1	14	10.89	4.68	21.1	20.63	1.02
GAIF01028729.1	37.09	36.78	47.24	59.92	49.6	37.28
GAIF01028730.1	10.62	9.3	16.08	30.09	21.24	19.34
GAIF01028731.1	0.15	0	0	0.45	3.25	0.15

GAIF01028733.1	0	0	0	0.04	0.08	0
GAIF01028734.1	0.26	0.5	4.05	0	0	7.83
GAIF01028735.1	0.18	0.13	0.13	0.35	0.66	0
GAIF01028737.1	0.37	0.89	0	2.44	8.89	2.01
GAIF01028738.1	0.12	0	0.27	0.43	3.55	0
GAIF01028739.1	0.13	0.14	0.2	0	0	0.15
GAIF01028740.1	4.71	4.09	3.38	9.53	4.95	8.37
GAIF01028743.1	0	0.05	0	0	0.32	0
GAIF01028745.1	1.29	0.47	0.24	0.25	0.69	0.87
GAIF01028746.1	0.31	0.47	0.07	3.93	17.61	0
GAIF01028747.1	0.27	0.25	0.2	0.34	1.83	0
GAIF01028750.1	0	0	0	0.21	0.21	0
GAIF01028752.1	0.16	0	0	0.28	1.27	0.36
GAIF01028753.1	0.17	0	0.12	0	0.68	0
GAIF01028754.1	0	0.03	0	0	0	0
GAIF01028755.1	0.01	0	0	0.29	0.22	0
GAIF01028756.1	0.41	0.3	0	0.48	0.33	0
GAIF01028758.1	6.49	2.64	3.26	11.17	5.93	9.31
GAIF01028761.1	0	0	0.04	0.31	0.86	0.23
GAIF01028762.1	41.3	63.05	89.81	113.6	112.3	79.77
GAIF01028763.1	0.43	0.87	0.28	7.27	48.79	0.12
GAIF01028764.1	2.27	0.61	0.27	7.75	26.17	1.67
GAIF01028765.1	22.22	17.97	41.9	15.35	5.03	18.12
GAIF01028766.1	6.23	1.14	0.51	1.89	1.05	0.13
GAIF01028767.1	0	0	0	0	0	0.1
GAIF01028768.1	540.53	422.27	557.95	305.49	146.82	451.02
GAIF01028770.1	0.27	0	0	4.32	14.95	0.3
GAIF01028771.1	0.07	0.25	0	0.25	0.47	0.09
GAIF01028773.1	51.13	47.28	15.86	63.02	65.89	12.27
GAIF01028775.1	0.08	0	0	0	0	0
GAIF01028776.1	0.72	0	1.48	1.98	1.3	2.35
GAIF01028777.1	5.21	2.43	1.36	1.87	2.99	0
GAIF01028779.1	6.45	2.63	2.65	5.98	2.52	7.81
GAIF01028780.1	0	0.1	0	0	0	0
GAIF01028781.1	7.52	4.03	6.65	1.17	0.67	0.37
GAIF01028782.1	20.54	24.33	20.04	45.49	46.66	9.08
GAIF01028783.1	0	0	0	0	0.16	0
GAIF01028784.1	0.13	0	0	0	0	0
GAIF01028786.1	13.12	20.54	26	43.24	20.04	14.24
GAIF01028787.1	55.51	48.71	7.98	73.97	102.84	58.57
GAIF01028788.1	0.27	0	0.2	0	0	0
GAIF01028789.1	0	0.07	0.07	0.1	0.3	0.15
GAIF01028791.1	99.76	179.71	221.59	63.92	66.81	79.89
GAIF01028792.1	0	0	0	0	0.29	0

GAIF01028794.1	0	0.05	0	0.16	0.62	0
GAIF01028795.1	0	0	0	0	0.12	0
GAIF01028796.1	0.25	0.36	0.75	0.57	0.84	0.11
GAIF01028797.1	0.26	0.09	0	0.86	1.96	0.57
GAIF01028798.1	6.71	6.07	3.53	39.43	57.2	4.35
GAIF01028799.1	0	0	0	0.32	0.49	0
GAIF01028800.1	0	0	0	0.15	0.71	0
GAIF01028804.1	0.73	0.62	0.6	1.32	1.33	0.29
GAIF01028805.1	0	0	0	0.08	0	0
GAIF01028807.1	3.17	0.82	0.38	6.04	16.67	2.28
GAIF01028808.1	0	0	0	0	0.04	0
GAIF01028810.1	0	0	0	0.04	0	0
GAIF01028811.1	1.3	0.95	0	4.4	15.42	0
GAIF01028812.1	10.04	24.11	17.79	21.46	20.81	2.08
GAIF01028814.1	0.2	0.43	0	1.37	1.36	0.22
GAIF01028815.1	0.07	0.08	0	0.14	0.77	0.05
GAIF01028817.1	0	0	0	1.02	0	0
GAIF01028818.1	3.22	1.05	0.58	6.21	8.63	1.98
GAIF01028819.1	0.2	0.04	0.04	0.08	0.9	0.04
GAIF01028820.1	0.39	0.36	0.02	1.03	1.91	0
GAIF01028821.1	78.35	82.82	49.59	85.3	45.27	6.44
GAIF01028822.1	0.06	0	0.05	0	1.09	0.03
GAIF01028823.1	47.72	58.25	26.95	96.44	44.19	8.27
GAIF01028824.1	0	0.08	0	0	0	0
GAIF01028825.1	0.37	0.27	0	0.58	3.54	0
GAIF01028826.1	35.42	44.51	21.34	7.91	4.32	0.72
GAIF01028827.1	1.25	1.46	0.82	3.12	0.03	1.13
GAIF01028829.1	0	0	0	0.04	0	0
GAIF01028830.1	12.24	12.72	8.39	30.97	38.92	21.45
GAIF01028833.1	0.41	0.34	0.05	1.01	1.37	1.27
GAIF01028834.1	0	0	0	0.02	0.07	0
GAIF01028835.1	225.19	256.87	494.19	231.85	183.45	562.77
GAIF01028838.1	19.46	15.79	22.46	42.18	48.24	43.5
GAIF01028839.1	0	0.33	0.17	0	3.82	1.32
GAIF01028840.1	8.79	12.82	6	44.99	34.12	1.76
GAIF01028841.1	0.03	0.02	0.01	0.08	0.05	0
GAIF01028843.1	1.03	0.66	0.51	2.65	10.99	0.82
GAIF01028848.1	17.94	14.7	14.41	40.73	23.45	10.03
GAIF01028849.1	0	0	0	0	0.1	0
GAIF01028850.1	0.12	0.13	0	1.51	7.5	0.09
GAIF01028851.1	0.92	0.21	0.07	2.73	14.48	0.36
GAIF01028852.1	13.42	27.37	4.05	12.4	20.84	3.59
GAIF01028854.1	0	0.38	0	2.32	1.27	0.65
GAIF01028855.1	0	0	0	0	0.56	0

GAIF01028856.1	3.29	11.86	0	33.29	21.38	3.16
GAIF01028857.1	0.53	0.34	0.4	1.05	0.77	0
GAIF01028860.1	1.49	0	0.68	0	0	0
GAIF01028861.1	186.06	291.34	25.87	18.76	92.56	27.47
GAIF01028862.1	0	0	0	0	0.29	0
GAIF01028863.1	0	0	0	1.64	10.69	0
GAIF01028864.1	0.79	0.61	0.62	0.52	0.14	0
GAIF01028868.1	0.12	0.04	0.04	0.04	0	0
GAIF01028869.1	4.13	6.91	14.06	2.44	2.56	12.02
GAIF01028870.1	24.09	0.01	0.02	26.15	0.01	0
GAIF01028871.1	0	0	0	0	0.17	0
GAIF01028872.1	0	0.3	0	0	0	0.1
GAIF01028873.1	0	0	0	0.13	0.77	0
GAIF01028874.1	0	0	0	0	0	0.03
GAIF01028875.1	0.08	0.04	0	1.07	2.5	0.14
GAIF01028876.1	0	0	0.08	0	0	0
GAIF01028877.1	17.87	8.16	20.34	27.01	15.92	173.71
GAIF01028879.1	0	0.15	0	0	0	0
GAIF01028880.1	0.13	0.14	0	1.83	7.28	0.58
GAIF01028881.1	0.2	0.11	0	0.23	0.24	0
GAIF01028882.1	0.36	0	0	0.79	5.46	0.35
GAIF01028887.1	4.16	7.03	4.26	6.94	5.17	0.67
GAIF01028889.1	1.67	0.43	0.09	0.46	1.72	0
GAIF01028890.1	0.83	2.35	0.37	0.77	0.4	0
GAIF01028891.1	0	0.21	0.42	1.54	1.6	0
GAIF01028894.1	4.35	4.6	3.16	1.64	0.64	0.19
GAIF01028895.1	0.44	0.37	0.12	0.06	0.27	0.27
GAIF01028896.1	0	0	0.03	0	0.05	0
GAIF01028897.1	35.89	67.75	62.46	63.22	53.13	32.98
GAIF01028898.1	5.55	6.73	8.2	10.77	5.9	4
GAIF01028899.1	29.85	42.5	7.32	39.32	68.27	9
GAIF01028901.1	0.13	0	0	1.75	7.25	0
GAIF01028902.1	0	0.05	0.22	0	0	0
GAIF01028903.1	37.36	25.05	4.06	71.98	86.63	10.06
GAIF01028906.1	0	0	0	0	0.3	0
GAIF01028909.1	0	0	0.05	0	0.12	0
GAIF01028912.1	0.22	0	0	0	0	0
GAIF01028913.1	0.78	0.09	0.1	0.5	0.21	0
GAIF01028914.1	0	0.1	0.06	0	0.3	0.17
GAIF01028916.1	17.04	17.96	10.28	4.15	3.67	1.62
GAIF01028917.1	20.49	9.14	4.77	4.6	1.59	4.06
GAIF01028918.1	1.65	0.09	0	0.16	0.14	0
GAIF01028919.1	0.04	0	0	0.26	0.69	0.02
GAIF01028921.1	0	0	0	0	0.22	0.09

GAIF01028922.1	21.67	22.32	21.04	78.47	85.69	7.6
GAIF01028925.1	37.78	50.46	29.88	61.34	52.29	4.42
GAIF01028926.1	195.66	199.53	184.44	174.5	0	0.05
GAIF01028927.1	2.31	2.23	2.99	6.45	10.77	2.76
GAIF01028928.1	238.49	114.34	111.51	139.45	81.05	163.52
GAIF01028929.1	0.31	0	0	0.18	3.73	0.34
GAIF01028930.1	37.75	78.27	29.87	69.12	77.81	3.34
GAIF01028931.1	0.43	0.16	0	0.33	0.35	0.32
GAIF01028932.1	8.05	4.43	12.33	1.27	0.24	2.3
GAIF01028934.1	0.94	1.24	0.24	3.64	2.64	0
GAIF01028936.1	6.71	7.93	4.48	38.33	39.45	2.5
GAIF01028937.1	1.08	0.14	0.07	0.29	2.89	0.28
GAIF01028938.1	0.67	0.32	0.41	0.51	0.8	0
GAIF01028939.1	0	0	0	0.32	0.98	0
GAIF01028940.1	35.22	33.81	26.92	51.69	78.86	26.67
GAIF01028941.1	16.92	79.33	113.46	22.07	32.96	30.18
GAIF01028942.1	8.74	3.09	0.81	43.33	219.44	5.88
GAIF01028943.1	7.04	8.87	8.18	22.15	19.51	3.96
GAIF01028944.1	0	0	0.03	0.26	2.38	0.14
GAIF01028945.1	1.89	0	0	0.67	0.16	0.08
GAIF01028947.1	58.32	96.76	43.76	114.2	124.75	5.92
GAIF01028948.1	0.14	0	0	0	0	0
GAIF01028949.1	0	0	0	0	0.17	0
GAIF01028951.1	65.76	18	46.93	55.78	27.2	47.13
GAIF01028952.1	0	0.2	0	0	0	0
GAIF01028954.1	0.09	0	0.07	0	0	0
GAIF01028956.1	0.12	0.66	0	1.55	1.02	0
GAIF01028957.1	3.86	2.74	0.16	20.15	87.96	3.27
GAIF01028958.1	6.29	3.61	1.52	15.43	3.86	2.44
GAIF01028960.1	15.57	17.46	20.81	23.4	17.71	9.45
GAIF01028961.1	166.55	102.74	98.58	151.94	183.08	292.82
GAIF01028962.1	0.45	0	0	0	0.11	0
GAIF01028963.1	84.95	40.95	18.15	14.59	1.8	12.96
GAIF01028965.1	3.08	7.51	3.84	2.9	1.6	0.18
GAIF01028967.1	0.43	0.08	0.22	0	0	0.08
GAIF01028969.1	0.45	0.1	0	0.72	6.43	0.98
GAIF01028972.1	6.48	6.59	8.05	1.38	2.99	1.2
GAIF01028974.1	1.61	1.38	1.56	1.06	0.63	0.22
GAIF01028978.1	0.43	0	0	0.24	0.31	0.16
GAIF01028979.1	0	0	0	0	0.21	0
GAIF01028980.1	0.22	0	0.12	0.26	0.81	0
GAIF01028984.1	0	0	0	0.09	0	0
GAIF01028986.1	15.03	6.91	2.91	0.74	0.51	0.23
GAIF01028987.1	10.75	2.86	0.67	33.67	49.32	48.98

GAIF01028988.1	0	0.3	0	0.17	2.03	0
GAIF01028989.1	67.76	92.58	54.89	111.93	141.47	9.98
GAIF01028990.1	14.89	6.89	8.06	6.52	12.29	15.15
GAIF01028991.1	1.1	1.27	0.55	1.65	3.4	0.75
GAIF01028993.1	0.24	0	0	0.28	0	0
GAIF01028994.1	0	0	0	0.1	0.31	0.09
GAIF01028996.1	1.22	1.23	0.86	3.75	6.93	0.29
GAIF01028998.1	0	0	0	0	0.19	0
GAIF01028999.1	0.15	0.08	0	0.22	1.1	0.09
GAIF01029000.1	0	0	0.04	0	0.25	0
GAIF01029001.1	11.01	12.51	10.27	3.57	2.44	0.54
GAIF01029003.1	0	0.58	0	0.13	0.15	0
GAIF01029004.1	0.17	0.2	0.11	1	3.93	0.16
GAIF01029005.1	0.04	0	0.01	0.19	0	0.39
GAIF01029006.1	1.74	1.22	0	2.73	3.39	3.9
GAIF01029008.1	0.13	0	0	0.65	0.31	0
GAIF01029009.1	18.5	12.46	15.29	38.79	30.7	17.9
GAIF01029010.1	7.26	7.51	7.57	15.23	11.06	2.19
GAIF01029012.1	0	0	0.34	0	0	0
GAIF01029013.1	22.31	14.56	11.16	89.86	216.56	23.22
GAIF01029015.1	0.04	0	0	0.05	0	0
GAIF01029016.1	0.98	0.14	0.14	0.23	0.08	0
GAIF01029017.1	5.26	9.76	11.3	31.34	22.4	3.67
GAIF01029018.1	0	0	0	0	0	0.12
GAIF01029019.1	1.88	1.43	0.36	3.27	7.82	0.47
GAIF01029020.1	0.17	0.04	0	0	0.08	0
GAIF01029022.1	0	0	0	0.06	0.13	0
GAIF01029023.1	0	0.13	0	0	0	0
GAIF01029024.1	19.34	11.93	8.45	36.02	56.42	45.67
GAIF01029025.1	0.23	0.12	0	0.08	0.26	0.07
GAIF01029026.1	116.93	52.34	52.1	208.78	166.65	51.75
GAIF01029027.1	3.65	2.55	1.49	3.87	8.3	1.24
GAIF01029028.1	1.62	0.15	0	7.36	21.27	0.45
GAIF01029029.1	16.51	29.23	44.26	42.03	25.07	22.47
GAIF01029030.1	5.01	3.03	4.28	0	0	0.46
GAIF01029031.1	0.2	0.29	0	0.23	0.69	0.14
GAIF01029032.1	53.44	38.47	36.67	18.65	5.02	3.68
GAIF01029034.1	0	0	0	0.09	0.29	0
GAIF01029035.1	0.07	0.07	0	0.08	0.24	0
GAIF01029039.1	0.15	0	0.25	0	0	0
GAIF01029040.1	4.68	3.32	1.2	0.63	0.39	0.12
GAIF01029042.1	0.23	0.09	0.01	1.68	2.71	0.01
GAIF01029043.1	10.34	7.34	3.46	6	8.85	2.41
GAIF01029044.1	14.67	19.01	24.91	49.75	42.08	32.84

GAIF01029045.1	0	0	0	1.06	3.93	0
GAIF01029046.1	0	0.2	0	0	0	0
GAIF01029047.1	4.94	5.08	5.24	12.43	13.43	22.94
GAIF01029048.1	15.24	10.38	8.49	24.47	39.57	54.71
GAIF01029049.1	0.86	0.37	0.53	0.33	0.12	0.42
GAIF01029050.1	0.6	0.48	0.48	0	0	0.12
GAIF01029051.1	15.5	10.5	10.28	28.58	24.48	18.7
GAIF01029052.1	2	0	0	4.46	5.65	2.18
GAIF01029053.1	1298.4	4406	5313.07	847.57	1653.75	5955.31
GAIF01029054.1	1.33	0	0	0.17	3.02	0
GAIF01029055.1	63.7	52.54	81.6	118.22	80.14	70.93
GAIF01029056.1	45.82	14.42	70.68	47.07	12.81	10.93
GAIF01029058.1	0.87	0.29	0.23	2.99	12.89	3.52
GAIF01029059.1	9.45	10.61	14.58	12.54	10.69	7.54
GAIF01029061.1	0.19	0	0	0	0	0.21
GAIF01029062.1	0	0	0	0	0.08	0
GAIF01029063.1	14.04	6.32	8.91	20.41	16.17	61.71
GAIF01029064.1	4.37	0.22	0	5.98	22.68	10.73
GAIF01029065.1	31.07	29.32	18.9	41.63	23.61	7.23
GAIF01029066.1	0	0.39	0.83	0	0	0.06
GAIF01029067.1	0	0	0	0.99	5.57	0
GAIF01029068.1	0.1	0	0	0	0	0
GAIF01029069.1	11.77	8.77	1.27	4.89	5.23	0.24
GAIF01029070.1	0.1	0	0	0	0	0
GAIF01029071.1	0.16	0.17	0	0.36	0.56	1.03
GAIF01029074.1	7.77	5.5	6.2	30.33	18.83	6.88
GAIF01029075.1	0.26	1.37	0	1.52	1.21	0
GAIF01029076.1	0.76	1.03	0	1.32	1.36	1.16
GAIF01029079.1	454.92	466.81	739.86	429.79	260.16	625.33
GAIF01029080.1	2.26	2.57	5.83	9.93	7.8	3.68
GAIF01029081.1	0.16	0	0	0	0.07	0
GAIF01029082.1	0	0.3	0.07	0.07	0	0
GAIF01029084.1	4.47	1.38	0	1.45	1.84	1.02
GAIF01029085.1	5.14	0.55	0.64	0.08	0	0.32
GAIF01029086.1	30.09	15.43	17.93	26.44	21.71	6.16
GAIF01029088.1	39.45	52.67	31.34	203.97	114.63	11.91
GAIF01029089.1	0	0.23	0	0	0.38	0
GAIF01029090.1	1.84	0.6	0.91	2.56	9.27	1.92
GAIF01029091.1	0	0	0.14	0	0.46	0
GAIF01029093.1	1.88	0.97	0.1	1.14	1.29	0
GAIF01029094.1	26.8	47.44	28.44	32.98	47.01	15.22
GAIF01029095.1	4.5	0	14.75	8.6	8.66	9.48
GAIF01029096.1	26.31	28.75	23.97	56.74	69.8	16.22
GAIF01029097.1	0	0	0	0.09	0.37	0

GAIF01029098.1	0	0	0	0	0.01	0
GAIF01029099.1	0	0	0	0.11	0	0.06
GAIF01029100.1	4.78	14	6.64	14.44	14.58	1.93
GAIF01029101.1	4345.07	5055.81	6982.31	2986.65	2029.74	3977.64
GAIF01029102.1	3.69	1.92	0.74	13.28	14.29	0.41
GAIF01029105.1	24.13	17.59	10.74	152.85	76.57	7.75
GAIF01029106.1	37.21	22.32	16.33	142.65	95.7	223.87
GAIF01029108.1	13.78	8.56	15.21	31.13	11.75	31.89
GAIF01029109.1	0.08	0.08	0	0.44	2.56	0.25
GAIF01029110.1	343.84	145.19	170.76	410.26	165.28	255.92
GAIF01029111.1	3.42	0.3	0.69	5.48	11.85	0
GAIF01029114.1	0	0	0	0.11	0.11	0
GAIF01029115.1	0.77	0.5	0.63	15.64	1.66	0
GAIF01029117.1	0.25	0.23	0.38	0.13	0.1	0.05
GAIF01029119.1	0	0.08	0.25	0.17	0.14	0
GAIF01029120.1	0.12	0.11	0.85	0.09	0.1	0.33
GAIF01029121.1	0	0	0	0	0.9	0
GAIF01029123.1	0.12	0	0	0.14	0.04	0
GAIF01029124.1	11.43	9.06	0	16.26	24.37	0
GAIF01029125.1	0	0	0	0.64	0.84	0
GAIF01029126.1	1.67	0	0	14.2	14.92	3.24
GAIF01029128.1	0	1.74	2.02	0.09	0	0.18
GAIF01029129.1	0	0.09	0	0	0.17	0.16
GAIF01029131.1	6.14	6.33	6.51	16.83	15.15	8.75
GAIF01029132.1	1.05	1	2.92	1.67	3.83	1.57
GAIF01029133.1	0.12	0	0	0.43	4.04	0.69
GAIF01029134.1	0.14	0	0	0.33	0.34	0
GAIF01029135.1	13.05	16.98	14.46	20.38	20.26	7.22
GAIF01029137.1	1.28	1.82	1.73	4.81	7.95	0.87
GAIF01029139.1	0	0.24	1.36	0	0	0.16
GAIF01029140.1	42.04	15.8	26.64	19.82	9.03	39.58
GAIF01029141.1	28.52	10.43	9.12	41.55	75.68	80.89
GAIF01029142.1	0	0	0	0.19	0.58	0
GAIF01029144.1	1.36	0.46	0.7	0.1	0.27	0.09
GAIF01029145.1	0.74	0.6	0.07	7.09	26.21	0.07
GAIF01029146.1	3.06	2.3	1.39	7.85	17.03	2.56
GAIF01029148.1	1.27	0.87	0.44	1.08	1.84	0.15
GAIF01029149.1	0.99	0.22	0	0.23	0.48	0
GAIF01029150.1	6.55	5.52	2.81	8.78	10.23	1.24
GAIF01029151.1	27.77	30.88	17.05	3.96	3.8	0.28
GAIF01029153.1	0	0	0	0.12	0	0
GAIF01029154.1	0.05	0	0	0	0.19	0
GAIF01029157.1	0.07	0.03	0	0	0.34	0
GAIF01029158.1	0.23	0.13	0.13	1.87	8.19	1.53

GAIF01029161.1	0.05	0	0.08	0.14	0	0
GAIF01029162.1	3.2	0.8	0.18	1.14	5.53	0.36
GAIF01029163.1	0	0	0	0	0.19	0
GAIF01029164.1	0	0	0.25	0	0.28	0.13
GAIF01029165.1	0	0	0	0	0.1	0
GAIF01029166.1	0.03	0.05	0.04	0.19	0.45	0
GAIF01029167.1	0.22	0.06	0	1.59	5.02	0.18
GAIF01029168.1	0	0	0	0	0.07	0
GAIF01029169.1	0	0	0	0	0	0.06
GAIF01029170.1	29.75	22.78	25.46	0	3.79	7.71
GAIF01029171.1	13.06	17.48	9.29	66.64	41.79	5.06
GAIF01029172.1	0	0.11	0.12	0.73	6.2	0.12
GAIF01029173.1	4.21	2.05	2.26	4.8	0.77	15.1
GAIF01029176.1	0	0	0.22	0	0	0.11
GAIF01029177.1	0.71	0.12	0	2.67	13.05	0.38
GAIF01029178.1	18.37	8.86	11.03	27.67	33.63	62.88
GAIF01029179.1	4.8	3.61	0.41	26.15	44.9	2.85
GAIF01029180.1	1.84	1.2	0.08	0.28	0.65	0
GAIF01029181.1	0	0.53	0.4	0.14	0.15	0
GAIF01029182.1	4.58	8.27	6.98	0.97	0.25	0
GAIF01029183.1	40.85	63.76	39.53	86.56	51.52	12.39
GAIF01029185.1	1.6	0.59	0	0	0	0
GAIF01029186.1	0.43	0.09	0	0.5	1.45	0.47
GAIF01029187.1	0.03	0.24	0.14	0	0	0.03
GAIF01029188.1	0	0.17	0	0.12	0.15	0
GAIF01029189.1	134.24	127.6	341.51	73.15	49.13	297.69
GAIF01029191.1	0.11	0.24	0.01	0	0	0
GAIF01029192.1	0.15	0.06	0	0	0	0.4
GAIF01029195.1	396.85	187.76	215.09	99.01	58.07	63.45
GAIF01029196.1	1.55	2.59	0.57	27.51	23.03	0.92
GAIF01029197.1	0	0	0.08	0	0	0
GAIF01029198.1	0	0	0	0.22	1.35	0
GAIF01029201.1	151.08	262.62	235.71	71.68	84.58	51.13
GAIF01029202.1	0	0.23	0	0	0.17	0
GAIF01029203.1	0	0	0	0.12	0.97	0.11
GAIF01029206.1	4.55	7.25	12.87	8.88	8.15	7.23
GAIF01029208.1	20.14	29.55	20.67	22.1	20.51	12.2
GAIF01029209.1	0.14	0.08	0	0.04	0.2	0.13
GAIF01029210.1	15.52	18.25	11.57	73.71	78.84	6.17
GAIF01029213.1	0.08	0.18	0	0	1.02	0
GAIF01029214.1	24.83	49.69	28.89	37.29	23.49	3.94
GAIF01029215.1	0.13	0	0	0.95	4.91	0
GAIF01029216.1	0	0.23	0.23	0.2	0.39	0.12
GAIF01029217.1	0.22	0.43	0	0.46	0	0

GAIF01029219.1	29.7	29.82	16.05	85.69	47.73	6.61
GAIF01029220.1	0.21	0	0	0	0.99	0
GAIF01029221.1	0.54	0.32	0	7.32	13.02	0
GAIF01029223.1	10.9	4.82	13.25	23.29	32.52	29.4
GAIF01029225.1	1.19	0.43	0	0.69	2.87	0
GAIF01029227.1	47.31	27.03	22.15	67.34	37.56	21.97
GAIF01029228.1	0	0	0	0.13	0	0
GAIF01029229.1	0.08	0	0.09	0	0.1	0
GAIF01029230.1	10.35	8.7	9.71	21.62	24.88	13.06
GAIF01029231.1	29.47	28.39	29.96	54.93	34.02	21.08
GAIF01029232.1	0.31	0.11	0.11	1.19	5.91	0.34
GAIF01029235.1	5.26	12.82	3.56	28.89	43.67	0.62
GAIF01029236.1	2.39	1.3	0	9.32	17.35	0.12
GAIF01029237.1	0.14	0	0	0	0	0
GAIF01029238.1	30.82	17.25	7.74	64.98	108.2	12.26
GAIF01029239.1	0	0.04	0	0	0	0
GAIF01029240.1	14.06	9.92	7.97	24.75	34.66	28.99
GAIF01029241.1	6.79	7.08	7.3	46.05	28.41	9.59
GAIF01029242.1	92.19	53.31	54.2	97.29	82.6	41.58
GAIF01029243.1	16.18	12.28	14.63	63.76	146.09	50.12
GAIF01029244.1	77.07	88.75	54.68	14.16	30.95	44.67
GAIF01029245.1	0	0.1	0	0	0	0
GAIF01029247.1	0	0	0	1.37	0.79	0.62
GAIF01029248.1	7.09	72.37	64.85	0.98	1.02	3.73
GAIF01029249.1	0	0	0.17	0	0	0
GAIF01029250.1	0.18	0.2	0.1	0.21	0	0.2
GAIF01029251.1	0.13	0	0	0	0	0.07
GAIF01029252.1	12.85	4.67	2.82	24.6	45.78	3.4
GAIF01029253.1	0.13	0.15	0	2.17	11.73	0
GAIF01029254.1	6.91	1.73	0	24.64	54.68	8.32
GAIF01029255.1	6.99	0.37	1.5	211.61	189.73	80.64
GAIF01029256.1	0.7	0.24	3.18	0.17	0	3.06
GAIF01029257.1	36.97	36.79	26.82	86.55	95.44	16.48
GAIF01029258.1	0.15	0.08	0.31	0.09	0.03	0
GAIF01029259.1	1.04	0.51	0	1.05	1.27	4.91
GAIF01029261.1	0	0.35	0.12	0	0.26	0
GAIF01029262.1	0	0	0	0.14	0.59	0.14
GAIF01029263.1	0	0	0	0	0.31	0
GAIF01029265.1	21.36	22.29	53.89	19.92	28.79	178.05
GAIF01029266.1	59.14	48.15	26.45	88.78	74.16	19.86
GAIF01029267.1	0	0	0	0.06	0.13	0
GAIF01029268.1	12.74	17.74	13.17	13.99	6.56	1.19
GAIF01029269.1	0.31	0.08	0	0.31	0	0.13
GAIF01029270.1	0.08	0	0	0	0.1	0

GAIF01029271.1	64.06	68.42	107.27	152.08	109.19	30.06
GAIF01029272.1	0.68	0.9	1.11	0.99	0.97	0.27
GAIF01029274.1	0.78	0.39	0.13	4.06	29.78	0.13
GAIF01029275.1	0	0	0	0	0.16	0
GAIF01029277.1	0.11	0.04	0	0.54	1.57	0.05
GAIF01029278.1	18.13	22.66	33.99	49.6	18.55	19.46
GAIF01029279.1	0.24	0	0	0	0.78	0.2
GAIF01029280.1	0	0	0	0.03	0.12	0.11
GAIF01029281.1	0	0	0.4	0	0	0.3
GAIF01029282.1	0.22	0.14	0.4	0.3	1.47	0.2
GAIF01029283.1	0	0	0.11	1.33	3.25	0.23
GAIF01029284.1	0	0	0	0.07	0	0
GAIF01029286.1	0.88	0.26	0.13	0.01	0.02	0
GAIF01029288.1	0	0	0	0.17	0.09	0
GAIF01029289.1	8.5	4.66	4.49	3.53	3.42	2.44
GAIF01029291.1	0.52	0.14	0.27	0.06	0.44	0.12
GAIF01029293.1	0.44	0.94	1.91	0	0.1	0.16
GAIF01029294.1	0.19	0.2	0.02	1.3	4.23	0.09
GAIF01029295.1	0	0.11	0	0	0	0
GAIF01029297.1	27.04	17.48	25.38	36.69	17.74	38.28
GAIF01029301.1	0.06	10.81	8.75	0	0.08	0.47
GAIF01029302.1	12.44	16.37	19.67	12.02	15.67	7.27
GAIF01029303.1	2.64	3.4	0.82	4.7	5.38	0.64
GAIF01029304.1	0	0	0	0.12	0.67	0
GAIF01029305.1	0	0.13	0	0	0.36	0
GAIF01029306.1	0	0	0	0	0.4	0
GAIF01029307.1	0	0	0	0.49	0	0
GAIF01029308.1	17.18	15.97	17.36	21.53	35.69	98.76
GAIF01029309.1	25.01	38.89	19.59	189.51	120.4	16.16
GAIF01029312.1	0.46	0.38	0.12	0.23	1.31	0.12
GAIF01029313.1	32.69	22.51	17.65	54.65	54.19	34.86
GAIF01029314.1	0	0.18	0	0	0	0
GAIF01029315.1	138.11	115.67	52.97	75.44	211.66	74.48
GAIF01029316.1	0.06	0	0	0	0	0.13
GAIF01029320.1	11.76	3.57	6.01	10.16	2.47	3.62
GAIF01029321.1	0.17	0.44	0	4.51	17.64	0
GAIF01029322.1	0.34	0	0	0.55	2.02	0
GAIF01029324.1	0.13	0.1	0.1	0.26	0.88	0.15
GAIF01029325.1	0	0.45	0	0	0	0.41
GAIF01029326.1	0.48	0.52	0.63	0.67	1.27	0.32
GAIF01029328.1	29.75	23.45	30.56	41.59	17.78	9.59
GAIF01029329.1	1.33	2.65	1.22	2.32	1.33	1.22
GAIF01029330.1	0	0	0	0	0.21	0
GAIF01029331.1	4.01	2.86	3.04	19.64	29.25	13.2

GAIF01029334.1	0.32	0.46	0.22	8.66	14.15	0.96
GAIF01029335.1	1.2	0.26	0	0	0.29	0.26
GAIF01029336.1	0.04	0	0	0	0	0
GAIF01029337.1	1.6	0.74	0	7.68	14.82	0
GAIF01029338.1	0.16	0.1	0.14	1.79	8.45	0.14
GAIF01029342.1	2.74	3.87	2.86	9.04	6.73	1.43
GAIF01029343.1	0.09	0	0	0.4	0	0
GAIF01029344.1	19.35	19.68	9.85	139.64	72.81	9.5
GAIF01029345.1	0.26	0.29	0.11	1.34	2.3	0.04
GAIF01029346.1	2.66	1.02	2.06	2.36	0.75	1.03
GAIF01029347.1	155.5	50.55	27.21	287.64	269.83	25.26
GAIF01029348.1	0.07	0	0	0	0.07	0
GAIF01029349.1	0	0	0	0	0.02	0.01
GAIF01029351.1	0.12	0.96	0.26	0.14	0.14	0.02
GAIF01029353.1	1.24	2.7	1.51	7.68	10.28	0.15
GAIF01029354.1	0	0	0	0.41	2	0.13
GAIF01029357.1	0	0	0.1	0	0	0
GAIF01029359.1	0.12	0	0	1.44	5.61	0.32
GAIF01029363.1	123.72	123.47	88.27	92.75	320.97	144.95
GAIF01029366.1	0	0	0	0.17	0.33	0.24
GAIF01029367.1	11.41	11.2	14.35	44.22	28.11	16.48
GAIF01029368.1	0	0	0	0.08	0.13	0
GAIF01029369.1	0.11	0.09	0.14	0	0	0
GAIF01029371.1	0.05	0.08	0	0	0	0
GAIF01029372.1	10.58	14.34	6.55	33.69	26.43	2.33
GAIF01029374.1	0	0	0	0.05	0	0
GAIF01029377.1	1.52	3.87	1.49	3.98	4.69	2.48
GAIF01029383.1	0.14	0.15	0	0	1.83	0
GAIF01029386.1	45.88	23.98	42.81	166.01	423.15	171.76
GAIF01029387.1	1.81	2.85	0.44	5.96	7.76	0.11
GAIF01029388.1	25.97	19.53	24.38	35.14	15.21	7.56
GAIF01029389.1	0	0	0	0	0.1	0
GAIF01029391.1	62.55	95.42	139.76	103.91	55.8	50.52
GAIF01029392.1	17.2	33.25	32.86	73.6	63.57	15.94
GAIF01029393.1	0	0	0	0.07	0.28	0
GAIF01029396.1	16.93	15.63	21.98	29.54	14.09	7.75
GAIF01029397.1	0	0.38	0	0.21	0	0
GAIF01029399.1	0	0	0	0.13	0	0
GAIF01029400.1	0	0.07	0.07	0.14	0.08	0
GAIF01029402.1	13.61	6.87	7.19	13.3	21.04	11.62
GAIF01029403.1	2.38	1.77	1.1	0.73	0.75	0.55
GAIF01029404.1	0.32	0.27	0.27	4.94	4.57	0.14
GAIF01029405.1	0	0.06	0	0.38	0.12	0
GAIF01029407.1	0.71	0.19	0.39	0.62	1.04	0.79

GAIF01029408.1	2.82	0.14	0	4.16	9.66	10.3
GAIF01029409.1	46.27	8.06	0.87	74.24	222.57	29.53
GAIF01029410.1	0	0.16	0	0.17	2.66	0.16
GAIF01029411.1	2.45	3.5	3.19	1.95	1.29	2.36
GAIF01029412.1	0.87	1.13	0.06	0.77	0.88	0
GAIF01029413.1	0	0	0.05	0	0.11	0
GAIF01029414.1	0.22	0.16	0	1.26	3.49	0.08
GAIF01029417.1	0.14	0	0	0	0.17	0
GAIF01029418.1	23.43	30.28	52.08	61.1	70.96	32.96
GAIF01029419.1	0	0	0.02	0	0	0
GAIF01029420.1	0	0	0	0.53	2.06	0
GAIF01029422.1	0	0	0	0	0.24	0
GAIF01029423.1	25.81	30	22.08	35.69	32.28	11.42
GAIF01029425.1	2.25	3.09	7.53	4.15	5.4	0.1
GAIF01029426.1	0	0	0	0	0.11	0.1
GAIF01029428.1	0.14	11.45	49.59	0	1.44	73.93
GAIF01029429.1	13.25	15.98	9.87	18.51	10.58	3.3
GAIF01029430.1	0.12	0.13	0	0	0.55	0
GAIF01029431.1	37.31	58.6	8.45	58.1	86.61	2.02
GAIF01029432.1	0.15	0	0.17	1.42	6.81	0
GAIF01029434.1	0.21	0.44	0.9	1.19	6.76	0.23
GAIF01029436.1	0	0	0	0.28	0.33	0
GAIF01029437.1	4051.56	3330.68	3903.64	2332.04	1845.32	4128.77
GAIF01029438.1	0	0	0	0.07	0	0
GAIF01029439.1	0	0.01	0	0	1.82	0
GAIF01029441.1	11.35	5.34	4.13	7.65	15.11	10.01
GAIF01029442.1	0.44	0.32	0	2.81	8.39	0.24
GAIF01029445.1	0.33	0.51	0.14	0	1.82	0
GAIF01029446.1	0.48	0.19	0	2.51	6.72	0.24
GAIF01029449.1	18.34	11.71	25.12	24.8	22.56	76.02
GAIF01029451.1	2.89	0.37	0	22.3	77.9	3
GAIF01029453.1	9.09	11.59	20.99	34.87	35.88	20.55
GAIF01029457.1	0	0.02	0	0	0	0
GAIF01029460.1	19.32	20.82	25.85	37.23	44.35	10.37
GAIF01029461.1	75.94	92.05	44.11	235.4	237.42	25.01
GAIF01029462.1	0.15	0	0	0	0.84	0
GAIF01029463.1	5.9	0.23	1.36	0.36	0.12	0.11
GAIF01029464.1	7.92	25.1	44.45	2.73	4.68	2.89
GAIF01029465.1	0.06	0.07	0.13	0.21	0.14	0
GAIF01029466.1	0	0.27	0.2	0.49	1.82	0
GAIF01029467.1	0	0.06	0	0.12	0.25	0.06
GAIF01029468.1	0	0	0	0	0.05	0
GAIF01029469.1	37.89	38.45	20.02	4.91	7.42	9.23
GAIF01029470.1	0	0	0	0	0.17	0.79

GAIF01029471.1	0	0	0	0	0.37	0
GAIF01029473.1	0.7	0.5	0.19	26.58	22.15	19.59
GAIF01029476.1	1.32	0.93	0.58	2.67	1.74	1.38
GAIF01029477.1	0.78	0.53	0.27	0.56	2.4	0.09
GAIF01029478.1	23.48	30.63	31.24	30.29	27.94	50.9
GAIF01029480.1	0.12	0	0	0.57	3.11	0.13
GAIF01029482.1	51.43	37.19	27.75	52.43	43.16	35.49
GAIF01029483.1	26.22	57.03	50.8	76.09	15.77	14.45
GAIF01029485.1	0	0.01	0	0	0	0
GAIF01029486.1	0	0	0	0	0.05	0
GAIF01029488.1	0.07	0.04	0	0	0	0.09
GAIF01029489.1	0.12	0.07	0	0	0.15	0
GAIF01029490.1	0.19	0.68	0.26	0	0.39	0.22
GAIF01029491.1	0	0.47	0	1.49	0	0
GAIF01029492.1	1.01	1	2.88	5.29	1.69	0.65
GAIF01029493.1	0	0	0	0	0.19	0
GAIF01029496.1	3.11	1.1	1.21	12.95	4.53	4.07
GAIF01029498.1	0.24	0	0	0	0	0
GAIF01029499.1	25.21	19.83	6.99	83.26	74.67	4.9
GAIF01029500.1	0	1.28	2.23	0	0.13	0.12
GAIF01029502.1	0.61	0.3	0.48	0	0	0.49
GAIF01029503.1	0	0.08	0	0	0	0
GAIF01029504.1	4.05	13.21	5.52	1.3	2.35	0.31
GAIF01029505.1	2.31	2.85	2.03	9.36	7.97	1.47
GAIF01029507.1	0.28	0.09	0.04	1.54	5.95	0.53
GAIF01029508.1	1.9	2.67	1.21	0	0.77	0.27
GAIF01029511.1	0.35	0	0	2.9	9.19	0.58
GAIF01029512.1	0	0.11	0.21	0	0.06	0
GAIF01029513.1	0.14	0	0	0.17	0.17	0.16
GAIF01029515.1	0.05	0	0.03	0	0	0
GAIF01029517.1	1.07	0.54	1.34	0.83	0.09	1.03
GAIF01029518.1	37.59	24.78	47.12	101.94	38.15	82.11
GAIF01029520.1	0	0.14	0.1	0	0.05	0
GAIF01029521.1	1631.07	268.96	567.82	0	0	135.11
GAIF01029522.1	0	0.18	0	2.32	5.71	0.56
GAIF01029523.1	3454.13	3041.15	5034.64	43.33	24.52	31.09
GAIF01029524.1	0	0	0	0.03	0	0
GAIF01029525.1	55.97	51.26	30.18	43.81	24.25	18.72
GAIF01029526.1	0	0	0	0	0.43	0
GAIF01029527.1	9.09	4.46	7.88	9.21	7.25	10.78
GAIF01029528.1	0.19	0	0	1.29	6.13	0.1
GAIF01029529.1	1.19	0	0	0.36	2.57	2.39
GAIF01029530.1	52.83	31.71	30.9	23.82	33.58	32.48
GAIF01029531.1	0	0.09	0	0	0.1	0.18

GAIF01029532.1	0	0	0	0	0.25	0
GAIF01029533.1	35.76	26.76	44.42	86.34	32.09	66.18
GAIF01029534.1	0.48	0	2.92	2.34	1.45	1.69
GAIF01029535.1	1.05	0.26	0.16	4.92	24.22	1
GAIF01029536.1	6.5	3.33	0	10.48	15.34	1.05
GAIF01029538.1	0	0	0	0	0.24	0
GAIF01029540.1	20.11	24.61	17.48	9.17	3.22	0
GAIF01029542.1	0	0	0	0	4.22	0
GAIF01029543.1	1.37	0	0	4.53	2.57	0
GAIF01029544.1	0.29	0.06	0	0.27	1.66	0
GAIF01029545.1	21.91	23.77	41.61	32.12	21.81	20.97
GAIF01029546.1	0	0.04	0	0	0.2	0
GAIF01029547.1	6.03	7.16	5.12	8.28	2.64	3.32
GAIF01029548.1	6.21	3.52	9.55	4.62	2.6	3.35
GAIF01029549.1	6.12	13.53	7.9	2.14	0.7	0
GAIF01029550.1	0.11	0	0	0	0.08	0.04
GAIF01029551.1	0.03	0.03	0	0	0.07	0
GAIF01029552.1	2.29	2.18	0.52	6.54	14.58	0.21
GAIF01029553.1	0.14	0.15	0	0.16	2.8	0
GAIF01029554.1	0	0.06	0	0.06	0	0
GAIF01029555.1	0	0	0	0.12	0	0
GAIF01029556.1	1.12	0.07	0	2.44	12.07	6.79
GAIF01029557.1	0.01	0	0.13	0	0.12	0
GAIF01029559.1	0.52	0.6	0.97	0	0	0.28
GAIF01029560.1	15.06	10.92	6.24	36.14	34.97	1.97
GAIF01029561.1	0	0	0.26	0	0	0
GAIF01029562.1	0	0	0	0	0.14	0
GAIF01029563.1	2.6	1.58	7.29	1.46	1.16	2.79
GAIF01029564.1	9.31	4.4	2.53	45.25	51.3	1.43
GAIF01029568.1	2.12	3.58	1.67	4.96	3.66	0.45
GAIF01029570.1	38.93	40.39	29.44	55.18	36.77	24.31
GAIF01029571.1	0	6.44	5.48	0	0	0.96
GAIF01029572.1	0.14	0	0	0.58	1.09	0.38
GAIF01029574.1	175.29	84.05	130.81	17.46	12.11	13.98
GAIF01029575.1	0.47	0.68	0	0.36	0	0
GAIF01029576.1	21.29	20.03	19.57	44.42	21.14	8.83
GAIF01029578.1	2.76	0.9	0.91	3.74	11.15	48.8
GAIF01029579.1	1.41	1.44	1.45	5.63	11.35	1.75
GAIF01029580.1	0	0	0	0	0.27	0
GAIF01029581.1	9.57	19.27	7.16	23.01	17.06	1.5
GAIF01029582.1	496.46	409.93	468.15	0	0.96	0.14
GAIF01029583.1	1021.9	518.8	633.05	198.55	245.87	1638.25
GAIF01029585.1	1.74	1.59	1.19	7.76	9.85	0
GAIF01029588.1	9.39	9.53	1.95	5.57	2.13	0

GAIF01029589.1	18.12	10.59	12.06	25.69	29.41	3.15
GAIF01029590.1	0	0	2.27	0	0	0.55
GAIF01029593.1	0	0	0	0.01	0.06	0
GAIF01029594.1	3.73	10.43	7.84	9.77	5.83	1
GAIF01029595.1	0	0	0.38	0	0	0
GAIF01029596.1	0	2.1	1.48	0	0	0.43
GAIF01029598.1	0.14	0	0.32	0	0	0
GAIF01029599.1	3.09	0.49	0.17	2.51	4.27	0.85
GAIF01029601.1	1.92	1.46	1.27	4.8	2.31	0.64
GAIF01029603.1	1.89	0.35	0.93	5.27	0	0.39
GAIF01029604.1	0	0	0	0.19	0.46	0.06
GAIF01029605.1	15.4	16.24	26.27	33.91	20.52	28.52
GAIF01029606.1	0.2	0.21	0.22	1.48	5.42	0.32
GAIF01029608.1	0.41	0.17	0	0.23	0	0.53
GAIF01029610.1	1.31	0	0	1.33	9.84	0.54
GAIF01029611.1	0.18	0.68	0	0.32	0	0
GAIF01029612.1	0	0.01	0	0	0	0.01
GAIF01029613.1	1.91	1.86	0	0.93	1.09	0
GAIF01029614.1	1.39	1.81	0	0.48	0.33	0
GAIF01029615.1	731.33	554.64	655.36	29.31	14.39	11.17
GAIF01029616.1	83.58	85.41	56.38	102.17	82.8	136.73
GAIF01029617.1	41.04	0.01	1.93	41.26	46.54	22.5
GAIF01029619.1	5.82	6.76	3.93	9.16	8.53	0.57
GAIF01029620.1	0	0	0	0	0.16	0.09
GAIF01029621.1	0.17	0.07	0	0.41	0.07	0
GAIF01029624.1	0.11	0	0	0.08	0.22	0
GAIF01029625.1	0	0	0	0	0.26	0
GAIF01029626.1	0	0	0.03	0.07	0	0
GAIF01029627.1	0.6	0.39	0.13	0.84	0.43	0.13
GAIF01029629.1	11.32	1.77	2.68	11.88	26.09	11.21
GAIF01029630.1	0	0	0	0	0.13	0
GAIF01029631.1	60.12	44.77	31.97	81.36	93.03	13.37
GAIF01029632.1	9.52	10.04	5.54	19.96	14.91	1.72
GAIF01029633.1	0.13	0	0	0.13	0.36	0
GAIF01029634.1	0	0	0	1.4	0	0
GAIF01029635.1	17.1	6.05	4.85	24.93	20.01	13.2
GAIF01029637.1	0.02	0.03	0	0.38	2.78	0.05
GAIF01029638.1	0.84	0	0	0.73	2.54	1.4
GAIF01029639.1	92.27	62.18	54	61.93	92.12	41.06
GAIF01029643.1	0	0.51	0	0.27	0.14	0.52
GAIF01029644.1	0	0.07	0.2	0	0	0
GAIF01029645.1	0	0	0	0	0.04	0
GAIF01029646.1	0	0	0	0	0.13	0
GAIF01029647.1	0	0	0	0	0.02	0

GAIF01029648.1	0	0	0.32	0	0.13	0.17
GAIF01029653.1	14.9	15.41	7.19	10.42	11.53	0.99
GAIF01029654.1	1.26	0.34	0	9.13	33.66	1.04
GAIF01029657.1	0	0	0.55	0	0	0
GAIF01029658.1	0	0.48	0.75	0.6	3.03	0.42
GAIF01029659.1	1.62	3.1	0.38	9.81	18.86	0.1
GAIF01029660.1	0.15	0	0	0.09	1.24	0
GAIF01029661.1	1.88	1.71	2.85	5.64	5.23	2.77
GAIF01029662.1	1.68	0.55	0.65	1.23	1.32	0.39
GAIF01029663.1	24.5	47.03	13.74	54.51	59.85	2.03
GAIF01029664.1	0.3	0	0	0	0.78	0
GAIF01029665.1	66.89	103.15	23.27	242.26	159.28	5.21
GAIF01029668.1	4.58	4.99	3.59	17.69	16.92	2.49
GAIF01029669.1	34.78	31.8	23.74	10.39	6.53	17.53
GAIF01029670.1	0.79	0.54	0.36	0.13	0.76	0.29
GAIF01029672.1	10.83	15.97	25.9	4.3	5.71	1.64
GAIF01029675.1	44.05	53	68.78	138.64	57.61	33.36
GAIF01029676.1	14.59	17.77	20.11	71.74	66.3	12.65
GAIF01029678.1	0.29	0.14	0	0.48	1.1	0
GAIF01029679.1	11.86	9.08	8.12	3.84	5.65	1.54
GAIF01029680.1	0	0	0.3	0	0.11	0
GAIF01029681.1	76.87	36.46	18.89	118.55	58.76	5.94
GAIF01029682.1	2.35	2.84	1.21	24.43	33.77	7.3
GAIF01029683.1	3.46	1.5	2.66	1.81	0.21	1.33
GAIF01029684.1	0	0	0	0.87	1.01	0
GAIF01029685.1	1.28	2.31	0.47	1.88	1.84	0
GAIF01029686.1	18	24.26	24.78	36.66	46.87	31.06
GAIF01029688.1	59.73	61.92	70.29	105.17	61.64	54.06
GAIF01029690.1	0	0	0	0	0.12	0
GAIF01029692.1	0	0	0	0	0.44	0.4
GAIF01029693.1	0.33	0	0	0.15	2.27	0.14
GAIF01029694.1	0.26	0.3	0.14	0.75	0.66	0.17
GAIF01029695.1	0	0	0	0	0	0.02
GAIF01029696.1	12.73	42.57	62.44	28.42	20.35	11.19
GAIF01029697.1	9.31	7.98	3.05	3.18	4.39	0.11
GAIF01029698.1	0	0	0.17	0	0	0
GAIF01029699.1	0.17	0	0.18	0.32	2.35	0
GAIF01029700.1	4.39	9.14	13.48	17.52	0	0.91
GAIF01029701.1	41.6	75	35.61	109.88	112.77	7.88
GAIF01029704.1	1.35	0.29	0.36	1.12	0.68	0.03
GAIF01029705.1	0.04	0	0	0.04	0.09	0.04
GAIF01029706.1	0	0	0.18	0	0.43	0
GAIF01029710.1	22.45	9.33	5.68	16.89	12.37	11.18
GAIF01029712.1	0.41	0.03	0.04	2.63	9.67	1.2

GAIF01029716.1	0.89	0.31	0.08	0.78	1.82	1.57
GAIF01029718.1	10.17	10.28	11.41	56.57	56.76	12.38
GAIF01029719.1	0	0.3	0	0.16	0	0
GAIF01029720.1	0	6.48	0	2.03	4.66	0
GAIF01029722.1	0	0	0.21	0	0	0
GAIF01029723.1	0	0	0.07	0	0	0.15
GAIF01029724.1	0	0	0	0	0.02	0
GAIF01029726.1	15.57	11.63	3.36	76.21	82.01	4.38
GAIF01029729.1	7.47	0.94	0	28.7	53.92	3.84
GAIF01029730.1	0.23	0.13	0	0.41	1.27	0.26
GAIF01029731.1	304.43	365.14	441.89	247.33	154.08	148.92
GAIF01029732.1	0	0	0	0.86	5.81	0.28
GAIF01029733.1	0	0	0.11	0	0	0
GAIF01029734.1	0	0	0	0	0	0.18
GAIF01029735.1	0	0	0	0	0.28	0
GAIF01029739.1	1.09	0.37	0	5.66	25.49	0.19
GAIF01029740.1	29.43	52.27	39.68	30.92	28.03	7.93
GAIF01029741.1	17.11	17.13	6.18	5.1	4.97	0.26
GAIF01029742.1	0	0	0.1	0	0.23	0
GAIF01029743.1	5.51	1.54	0	9.76	32.79	0.39
GAIF01029744.1	0.95	0.62	0	6.65	17.87	0.58
GAIF01029745.1	1.27	0.99	0.4	2.14	4.57	0.51
GAIF01029746.1	0.25	0.81	0	2.29	1.19	0.27
GAIF01029747.1	0.03	0.03	0	0	0	0
GAIF01029748.1	8.17	2.94	0	6.51	8.65	1.88
GAIF01029749.1	0.06	0	0	0.7	3.43	0
GAIF01029750.1	192.5	149.58	154.41	421.95	381.47	358.93
GAIF01029751.1	0	0	0.05	0.14	0.05	0.13
GAIF01029752.1	0	0	0	0.06	0	0
GAIF01029753.1	0.59	0.25	0.11	2.52	6.61	4.86
GAIF01029754.1	0.1	0.22	0.15	0.04	0	0
GAIF01029755.1	0	0	0	0.08	0	0
GAIF01029756.1	0.61	0.84	0.09	0.85	1.61	0
GAIF01029757.1	2.84	1.57	2.49	8.56	16.33	1.06
GAIF01029758.1	0.22	0.42	0.36	0.06	0.07	0.48
GAIF01029760.1	0	0	0	0	0.23	0
GAIF01029761.1	0.36	0.13	0	0.14	0.14	0
GAIF01029762.1	1.08	0.64	0.41	1.08	2.2	0.81
GAIF01029764.1	0	0.14	0	0	0	0
GAIF01029765.1	8.22	0	0	12.95	4.69	2.82
GAIF01029766.1	2.57	1.31	2.6	0.23	0.06	0.33
GAIF01029767.1	0.18	0	0.2	0.32	0.22	0
GAIF01029769.1	57.41	102.71	76.11	22.05	19.32	9.03
GAIF01029770.1	0	0	3.68	0	0.26	0

GAIF01029772.1	7.24	5.03	6.64	16.43	20.4	5.94
GAIF01029773.1	18.6	15.09	12.87	52.61	52.11	4.71
GAIF01029774.1	7.45	9.85	7.02	25.74	16.68	2.77
GAIF01029775.1	8.37	3.44	3.47	10.52	7.91	42.64
GAIF01029776.1	0	0.04	0.15	1.88	12.69	0.17
GAIF01029777.1	0	0	0	0	0.16	0
GAIF01029778.1	0	0	0.05	0	1.35	0.49
GAIF01029779.1	0.04	0.13	0	0.42	0	0.07
GAIF01029780.1	0.2	0.05	0.05	0	0	0
GAIF01029784.1	0	0	0	0	0.35	0
GAIF01029786.1	0	0.08	0	0	0	0
GAIF01029789.1	27.52	29.23	23.99	84.7	83.99	28.54
GAIF01029790.1	0	0	0	0.08	0.08	0
GAIF01029792.1	0.07	0.29	0	0.08	0	0.07
GAIF01029793.1	0.1	0.34	0.11	0.85	3.26	0.35
GAIF01029794.1	0.09	0.1	0	0.21	0.43	0
GAIF01029795.1	1.99	0.71	0	3.48	2.77	0.72
GAIF01029796.1	0	0.47	0	2.02	10.15	1.2
GAIF01029797.1	2.4	1.83	0.75	1.7	3.83	0.52
GAIF01029801.1	2816.83	536.63	354.26	1596.34	1833.89	1458.29
GAIF01029802.1	60.15	37.92	57.12	55.78	0	76.8
GAIF01029803.1	0	0	0	0	0.82	0
GAIF01029804.1	89.25	44.74	62.99	11.43	5.85	8.94
GAIF01029805.1	18.95	16.54	23.55	24.17	31.89	6.02
GAIF01029806.1	0	0	0	0	0	0.17
GAIF01029808.1	0.2	0.13	0	0.73	2.83	0
GAIF01029809.1	13.77	15.29	7.94	31.54	25.04	7.97
GAIF01029810.1	0	0.31	0.62	0	0	0
GAIF01029811.1	79.48	74.84	86.3	65.9	62.52	61.08
GAIF01029812.1	68.99	36.66	44.49	57.06	75.54	75.71
GAIF01029814.1	0	0.07	0	0.12	0.56	0
GAIF01029815.1	18.49	18.61	36.95	62.33	62.36	30.7
GAIF01029816.1	24.26	18.77	27.78	21.56	21.09	8.96
GAIF01029817.1	0	0	0	0	0.01	0
GAIF01029818.1	1.36	0.92	2.1	0.59	0.61	0.93
GAIF01029820.1	21.87	14.51	25.37	43.59	32.69	53.65
GAIF01029822.1	205.38	70.27	26.84	34.64	31.78	0.93
GAIF01029825.1	3.44	4.64	0.69	7.25	6.89	0.11
GAIF01029826.1	4.84	0	0	8.76	2.02	1
GAIF01029827.1	12.22	13.02	17.35	47.18	65.05	7.66
GAIF01029828.1	0	0	0	0	0.97	0.17
GAIF01029829.1	1.95	0	0	3.76	7.21	1.43
GAIF01029830.1	0.26	0	1.97	0.3	0	0.28
GAIF01029831.1	0.27	0.12	0.13	1.59	5.15	0

GAIF01029832.1	0	0	0.04	0	0.08	0
GAIF01029833.1	33.31	21.08	10	36.19	23.46	63.28
GAIF01029834.1	11.15	5.68	5.65	34.13	20.9	17.97
GAIF01029835.1	0.53	0.31	0.05	0.11	0.75	0.11
GAIF01029836.1	0	1.3	0	0	0.83	0
GAIF01029837.1	0	0	0	0	1.61	1.33
GAIF01029838.1	11.24	9.71	9.13	30.87	9.78	7.13
GAIF01029840.1	0	0.18	0	0.72	7.4	0
GAIF01029843.1	0	0.14	0	0.4	1.86	0.09
GAIF01029844.1	0	0.09	0	0	0.1	0
GAIF01029846.1	9.64	8.44	9.16	27.24	17.7	39.45
GAIF01029847.1	0	0.02	0	0	0	0
GAIF01029848.1	1.85	2.11	0.74	0.94	11.38	0.84
GAIF01029849.1	12.22	7.63	9.22	28.52	40.42	10.37
GAIF01029850.1	0.1	0.09	0	0.24	3.11	0
GAIF01029851.1	32.62	30.58	8.49	36.72	72.06	69.62
GAIF01029853.1	0.21	0.11	0.45	0	0.12	0
GAIF01029854.1	0.5	1.09	0.55	3.5	4.84	0
GAIF01029855.1	25.21	43.12	22.41	51.97	49.6	9.54
GAIF01029856.1	0	0	0	0.06	0	0
GAIF01029857.1	0.12	0.35	0	0.53	3.78	0.21
GAIF01029858.1	0	0.03	0	0.03	0.07	0
GAIF01029859.1	0	0	0	0	0.17	0
GAIF01029863.1	0	0.14	0	0.45	2.64	0
GAIF01029864.1	0	0.48	0	1.16	2.54	0.88
GAIF01029865.1	124.22	156.12	202.42	472.19	259.29	61.29
GAIF01029866.1	0.07	1.45	1.33	0.15	0.16	0.47
GAIF01029867.1	0.51	0.21	0.09	3.76	14.28	0.17
GAIF01029868.1	11.37	13.06	5.45	14.87	17.33	0.88
GAIF01029872.1	38.42	9.75	13	13.82	83.65	240.58
GAIF01029873.1	0.34	0.09	0.47	0.1	0.21	0
GAIF01029874.1	73.98	83.87	82.41	116.12	122.57	31.47
GAIF01029875.1	0	0	0	0	1.19	0.22
GAIF01029876.1	0	0	0	0	0.4	0
GAIF01029877.1	1.42	1.11	0.17	0.46	0.19	0
GAIF01029878.1	1.07	0.95	0.11	10.17	36.08	0
GAIF01029879.1	0	0	0	1.25	6.21	0.71
GAIF01029880.1	0.14	0.03	0.16	0.51	0.31	0.45
GAIF01029881.1	2.38	1.3	1.24	2.13	2.54	0.85
GAIF01029883.1	0	0	0	0.15	0.4	0
GAIF01029885.1	1.14	4.5	0	0	0	0
GAIF01029887.1	111.39	246.22	115.47	123.24	376.37	16.15
GAIF01029889.1	39	33.71	8.87	104.04	102.18	2.7
GAIF01029890.1	0	0.73	0.11	0	0	0

GAIF01029891.1	0.3	0.21	0	0.92	3.09	0.11
GAIF01029892.1	9.99	6.92	7.96	20.39	26.46	13.7
GAIF01029894.1	0	1.47	0.95	0	0.15	0
GAIF01029895.1	0.14	0.16	0	0	1.42	0.16
GAIF01029897.1	161.47	72.69	71.64	162.43	223.55	676.05
GAIF01029901.1	10.97	12.24	13.39	40.25	37.46	10.57
GAIF01029904.1	1.72	0.87	0	18.3	88.09	1.4
GAIF01029905.1	5.52	3.29	11.32	5.53	0.24	2.62
GAIF01029906.1	0.14	0.16	0	0.5	1.89	0.32
GAIF01029908.1	9.61	7.61	7.74	29.94	37.21	3.31
GAIF01029909.1	0	1.02	1.99	0	0	0.58
GAIF01029910.1	0	0	0	0	0.69	0
GAIF01029912.1	0.26	0.06	0.28	0.36	1.67	0
GAIF01029913.1	0.1	0	0	0.18	0.32	0
GAIF01029914.1	0	0	0	0.03	0	0
GAIF01029915.1	17.88	25.26	13.16	63.46	35.64	7.57
GAIF01029917.1	0	1.07	1.25	1.05	0	0
GAIF01029918.1	1.14	2.18	0.89	1.82	1.43	0.3
GAIF01029919.1	0.74	2.13	0.01	1.2	0.78	1.22
GAIF01029921.1	0	0.09	0.19	0.2	0.2	0
GAIF01029922.1	17.68	8.75	7.18	54.49	71.51	19.77
GAIF01029923.1	0	0	0	2.92	7.06	0
GAIF01029924.1	18.36	33.69	45.78	95.36	90.61	21.96
GAIF01029925.1	5.04	3.03	3.96	6.38	5.98	5.42
GAIF01029926.1	0	0	0	0.48	1.36	0.45
GAIF01029927.1	0	22.48	15.79	36.2	40.96	15.1
GAIF01029929.1	0	0	0	0	0.12	0
GAIF01029930.1	0	0	0	0	0.06	0
GAIF01029931.1	0	0.12	0.25	0.78	2.56	0.25
GAIF01029932.1	33.11	25.78	29.95	88.14	43.72	22.09
GAIF01029933.1	0.3	0.13	0.2	0	0.15	0.13
GAIF01029934.1	0.13	0.14	0	0.15	0.62	0
GAIF01029935.1	14.1	8.71	6.1	22.25	6.23	1.55
GAIF01029936.1	26.7	20.56	11.76	79.1	51.58	6.16
GAIF01029938.1	44.17	33.63	31.88	199.46	109.2	38.99
GAIF01029941.1	0.12	0	0	0.87	0	0.14
GAIF01029943.1	23.19	34.48	25.99	40.01	27.69	5.45
GAIF01029944.1	0.06	0	0	0	0	0
GAIF01029945.1	0.19	0.1	0	1.65	4.98	0.1
GAIF01029946.1	3.15	5.87	3.07	2.6	0	4.15
GAIF01029949.1	0	0	0	0.98	0	0
GAIF01029950.1	0.13	0.07	0	0.37	0.76	0.07
GAIF01029951.1	0	0.12	0	0.12	2.3	0
GAIF01029952.1	6.6	2.7	4.41	13.1	7.7	8.11

GAIF01029953.1	0	0	0	0.06	0.06	0
GAIF01029954.1	3.6	3.19	0.68	16.29	25.91	0.26
GAIF01029956.1	0	0	0	0	0.62	1.24
GAIF01029957.1	1.95	0	3.27	8.06	20.47	1.35
GAIF01029958.1	1.62	1.33	0.9	1.68	2.15	0.22
GAIF01029959.1	0	0	0	0	0	0.07
GAIF01029961.1	0.12	0	0	0	0	0
GAIF01029962.1	0	0	0	0	0.48	0
GAIF01029963.1	0	0.06	0	0	0	0
GAIF01029964.1	0	0.07	0.8	0.08	0	0.07
GAIF01029965.1	0.22	0.12	0	1.65	8.94	0
GAIF01029966.1	0	0	0	0.19	0	0.06
GAIF01029968.1	0.18	0	0	0.21	0	0
GAIF01029969.1	1.56	0.81	0	11.78	15.99	0
GAIF01029970.1	0.04	0	0	0	0	0
GAIF01029971.1	26.48	18.33	12.72	21.52	0.61	25.02
GAIF01029972.1	16	4.86	4.97	20.63	12.16	4.05
GAIF01029973.1	16.3	16.12	33.56	58.18	41.81	55.03
GAIF01029974.1	199.5	28.53	84.47	118.25	76.11	86.72
GAIF01029975.1	0.21	0	0	0.73	2.54	0
GAIF01029977.1	13.78	19.51	19.01	7.48	7.45	16.16
GAIF01029978.1	37.29	38.25	21.89	60.59	35.22	6.13
GAIF01029981.1	0	0	0	0	0.05	0
GAIF01029983.1	0.31	0	0	0	0	0
GAIF01029984.1	0.08	0.06	0	0.76	2.18	0.17
GAIF01029985.1	10.09	9.57	5.58	33.45	28.18	5.02
GAIF01029987.1	0.5	1.62	1.09	2.68	0.79	0.18
GAIF01029988.1	0.12	0.14	0	0.29	0.12	0
GAIF01029991.1	0.1	0.04	0	0.27	3.38	0.04
GAIF01029993.1	0.45	0.06	0	0.19	1.75	0
GAIF01029995.1	0	0	0	0.02	0.14	0
GAIF01029996.1	37.5	14.99	24.82	50.82	22.68	217.71
GAIF01029997.1	0	0	0	0	0.04	0
GAIF01029998.1	21.53	29.43	20.28	32.76	32.09	6.43
GAIF01029999.1	0	0.19	0	0	0.21	0.19
GAIF01030000.1	53.75	95.69	99.8	144.21	133.05	20.91
GAIF01030001.1	9.8	7.49	4.36	48.92	53.76	9.93
GAIF01030002.1	2.91	0.69	0.5	8.34	21.12	0.4
GAIF01030003.1	50.12	24.95	23.16	27.75	16.98	12.19
GAIF01030004.1	6.34	8.35	11.39	5.82	3.94	3.96
GAIF01030005.1	0	0	0	0.06	0.26	0
GAIF01030006.1	0.09	0	0	0.99	4.53	0.09
GAIF01030009.1	0.38	1.27	0	1.11	1.65	0
GAIF01030011.1	2.17	1.59	1.33	1.31	1.81	0.41

GAIF01030012.1	3895.42	4573.13	9061.9	2388.85	1591.28	10373.48
GAIF01030013.1	2.9	4.7	6.09	898.83	807.35	460.61
GAIF01030014.1	0.17	0	0	0	0	0
GAIF01030015.1	0.31	0	0	0.83	0.25	0
GAIF01030016.1	0.03	0.11	0	0	0	0.08
GAIF01030017.1	0	0	0.34	0	0	0
GAIF01030019.1	39.06	10.97	14.55	23.11	15.29	20.57
GAIF01030021.1	0.2	0	0.11	0	0.59	0
GAIF01030022.1	0.38	0.04	0	1.61	4.66	0.04
GAIF01030023.1	0	0	0.13	0	0	0
GAIF01030024.1	4.32	6.93	8.05	1.36	0.35	0.24
GAIF01030025.1	2.78	5.95	3.14	2.15	2.53	1.21
GAIF01030026.1	0	0	0	0	0.12	0
GAIF01030028.1	0	0	0	0.12	0.19	0.06
GAIF01030030.1	0.1	0	0	0.57	0.72	0
GAIF01030031.1	2226.12	6268.11	7947.57	2416.26	1550.56	973.79
GAIF01030034.1	53.02	23.93	16.2	82.31	32.56	6.26
GAIF01030035.1	7.74	8.42	11.43	25.03	15.83	4.05
GAIF01030036.1	0	0	0	0.45	1.16	0
GAIF01030037.1	63.9	38.11	122.61	25.86	6.89	18.95
GAIF01030039.1	7.18	2.5	0.42	28.42	57.51	1.27
GAIF01030040.1	1.43	0.35	0.2	5.57	22.7	0.15
GAIF01030041.1	18.23	19.96	32.29	54.45	47.8	27.59
GAIF01030043.1	0	0.05	0	0.05	0.05	0
GAIF01030044.1	73.74	86.98	54.82	199.34	189.36	28.21
GAIF01030045.1	0.05	0	0	0.12	0.12	0
GAIF01030046.1	28.13	17.37	17.01	42.56	68.15	14.38
GAIF01030048.1	53.03	73.97	86.94	100.75	64.42	35.54
GAIF01030049.1	5.08	4.89	5.08	15.15	30.13	2.03
GAIF01030050.1	0.31	0.37	0.22	0.72	1.5	0.11
GAIF01030051.1	144.28	238.71	287.27	138.91	109.02	64.17
GAIF01030052.1	0	0.43	0.97	0	0	0.38
GAIF01030053.1	0.71	0.92	0.62	4.49	3.87	0.01
GAIF01030057.1	0.24	0.4	0	0.43	0	0.4
GAIF01030058.1	0.42	0.75	0	1.61	6.67	0
GAIF01030060.1	0.18	0.4	0.2	0.21	6.16	0
GAIF01030061.1	0	0	0	1.59	4.17	0.17
GAIF01030062.1	20.8	32.81	31.11	48.51	48.87	13.46
GAIF01030063.1	0.25	0.68	0	0.29	0	0
GAIF01030064.1	0	0	0	0	0.06	0
GAIF01030065.1	12.16	3.58	1.9	23.44	25.94	10.09
GAIF01030066.1	1.19	0.1	0.08	1.53	2.02	0.91
GAIF01030070.1	0.1	0.06	0.09	0.06	0.12	0.05
GAIF01030071.1	0	0.47	1.1	0.06	0.55	0.34

GAIF01030072.1	2.52	4.57	0.23	7.08	13.9	0.23
GAIF01030073.1	0	0	0	0	0.12	0
GAIF01030074.1	0	0	0	0.24	1.72	0
GAIF01030075.1	0.55	0.17	0.23	2.07	7.82	0.16
GAIF01030077.1	0	0	0	0	0.2	0
GAIF01030078.1	0.05	0	0	0	0.23	0
GAIF01030079.1	3.28	3.56	3.6	3.8	23.65	0
GAIF01030081.1	2.46	2.71	1.89	12	2.57	2.05
GAIF01030082.1	601.84	252.11	108.28	438.02	403.15	85.97
GAIF01030083.1	78.34	47.08	84.22	77.69	10.89	31.91
GAIF01030085.1	0	0	0.09	0	0.14	0.04
GAIF01030086.1	0.7	0.57	0.57	0.2	0	0
GAIF01030089.1	964.42	255.56	293.01	497.88	855.19	3027.87
GAIF01030090.1	2.06	0.3	0.45	8.38	23.53	1.34
GAIF01030091.1	0.09	0	0	0	0	0
GAIF01030092.1	0	0.07	0	0.11	0	0
GAIF01030093.1	0	0	0	0.06	0.06	0
GAIF01030094.1	1.35	2.84	1.39	1.17	0.81	0.65
GAIF01030096.1	57.3	83.42	92.5	122.27	97.53	93.53
GAIF01030097.1	0	0	0	0.14	0.07	0
GAIF01030098.1	19.3	27.02	33.47	44.67	26.81	10.23
GAIF01030099.1	140.3	144.73	154.03	243.16	292.56	453.64
GAIF01030100.1	0.34	0	0	0.74	5.45	0.53
GAIF01030102.1	0	2.7	4.71	10.93	5.01	8.1
GAIF01030103.1	15.45	0.14	2.12	15.07	1.48	35.66
GAIF01030105.1	0	0	0	0	0.1	0
GAIF01030106.1	17.46	9.63	15.66	39.96	28.99	40.66
GAIF01030107.1	7.95	17.96	4.34	33.29	35.88	13.09
GAIF01030111.1	4.49	0	0.21	2.71	0.11	0.93
GAIF01030113.1	0.03	0	0	0	0	0
GAIF01030114.1	5.6	8.22	2.8	10.48	18.92	3.84
GAIF01030116.1	0	0	0	0	0.24	0
GAIF01030119.1	1.45	0.87	0.72	1.35	1.22	0.8
GAIF01030120.1	0	0.11	0.12	0	0	0
GAIF01030122.1	0	0.16	0	0	0	0
GAIF01030123.1	0.62	1.17	1.48	1.07	0.06	0.7
GAIF01030125.1	1.22	0.03	0	0.34	2.39	0
GAIF01030127.1	16.48	24.89	10.73	7.66	11.12	3.48
GAIF01030128.1	41.68	54.49	62.1	43.54	20.89	16.3
GAIF01030129.1	0.32	0.09	0.09	0.66	0.58	0.04
GAIF01030130.1	3.41	1.93	0.64	15.95	17.89	0.7
GAIF01030133.1	2.93	0.38	0	1.36	10.57	13.69
GAIF01030134.1	0	0	0	0	1.03	0
GAIF01030135.1	0.4	0.07	0.04	4.52	17.3	0.07

GAIF01030136.1	0	0	0	0.14	0.14	0
GAIF01030139.1	24.17	14.15	12.21	51.19	55.07	16.16
GAIF01030140.1	0.05	0	0	0.05	0.22	0
GAIF01030141.1	0.2	0.17	0.12	0.58	0.75	0.05
GAIF01030143.1	0	0	0.06	0.18	0.7	0
GAIF01030145.1	34.25	87.04	56.35	35.7	38.3	7.11
GAIF01030148.1	0	0	0	0	0	0.02
GAIF01030150.1	0.92	0.14	0	0.46	1.9	1.16
GAIF01030152.1	0.08	0	0.08	0.09	1.02	0
GAIF01030153.1	54.43	14.43	43.77	60.27	33.9	20.12
GAIF01030154.1	358.24	400.43	410.64	0.17	0.53	0.41
GAIF01030155.1	28.97	15.05	6.82	4.28	3.44	0.5
GAIF01030157.1	0	0	0	0.25	0	0
GAIF01030158.1	0	0	0	0	0	0.1
GAIF01030159.1	0	4.11	3.58	3.14	5.97	1.09
GAIF01030164.1	0.2	0.26	0.35	0.1	0.45	0
GAIF01030165.1	1.08	0.64	0	2.42	6.22	1.66
GAIF01030167.1	0	0	0.03	0	0	0
GAIF01030168.1	0.17	0	0	0	0	0
GAIF01030170.1	2.72	3.26	3.61	3.85	2.75	0.26
GAIF01030171.1	0.14	0	0.07	0.16	0	0.07
GAIF01030172.1	0	0.07	0	0.07	0	0
GAIF01030173.1	95.22	26.46	72.98	147.47	95.94	15.96
GAIF01030174.1	2.76	7.93	3.26	4.11	0	1.51
GAIF01030177.1	20.42	23.63	35.25	70.33	32.08	20.51
GAIF01030179.1	0	0	0	0	0.33	0
GAIF01030180.1	0.1	0.34	0.23	0.12	0	0
GAIF01030181.1	12.6	0.22	5.42	26.01	7.85	40
GAIF01030182.1	10.16	2.59	1.15	13.66	15.7	0.9
GAIF01030183.1	0.11	0	0	0.63	4.44	0.12
GAIF01030184.1	1.63	1.61	0	4.73	9.88	0.33
GAIF01030186.1	198.88	19.69	37.37	377.26	152.64	58.91
GAIF01030188.1	0	0	0	0	1.33	0
GAIF01030189.1	2.47	3.78	1.24	10.34	16.14	1
GAIF01030190.1	17.07	25.34	13.57	40.31	44.05	5.57
GAIF01030191.1	0.51	0.14	0.14	1.11	1.61	0.56
GAIF01030192.1	3.79	5.2	4.32	5.39	3.27	0.98
GAIF01030193.1	0	0.17	0	0	0	0
GAIF01030194.1	0.63	0	0	2.45	25.48	1.86
GAIF01030196.1	14.37	20.62	17.78	59.67	72.97	7.94
GAIF01030197.1	0	0	0	0	0.1	0
GAIF01030198.1	0	0	0	0.1	0.7	0
GAIF01030201.1	0.5	0.27	0.27	0.58	0.45	0.14
GAIF01030202.1	0.04	0	0	0.04	0.15	0

GAIF01030203.1	0.21	0.54	0	0.14	1.1	0.3
GAIF01030204.1	21.99	29.93	14.57	49.76	58.54	15.75
GAIF01030205.1	1.29	0.16	1.73	0.83	0.69	4.43
GAIF01030207.1	0.53	0.29	0.19	3.88	27.54	0.39
GAIF01030208.1	1389.8	3072.53	2860.11	604.15	777.04	818.1
GAIF01030209.1	629.75	542.5	275.48	507.4	1479	105.2
GAIF01030210.1	0.89	1.66	0.91	3.61	6.04	0.63
GAIF01030213.1	0.76	4.89	5.85	198.8	66.86	79.28
GAIF01030214.1	27.67	15.83	25.55	18.36	2.78	18.64
GAIF01030215.1	0	0	0.08	1.02	3.96	0.16
GAIF01030217.1	0	0	0	0.01	0	0
GAIF01030218.1	6.25	3.68	4.38	0.61	0.55	1.58
GAIF01030219.1	18.93	11.63	4.75	22.77	15.74	2.52
GAIF01030221.1	0.16	0.17	0	0	1.33	0
GAIF01030222.1	58.82	43.02	38.75	57.92	58.73	32.22
GAIF01030223.1	0	0.27	0	0.57	0.29	0
GAIF01030224.1	0.1	0	0.23	2.06	8.39	0.34
GAIF01030225.1	3.49	4.03	4.14	16.55	21.09	2.54
GAIF01030227.1	0.08	0.04	0.13	0	5.64	0.09
GAIF01030228.1	3.78	6.56	7.53	5.35	4.84	1.69
GAIF01030229.1	0.09	0.14	0	0.1	0.34	0
GAIF01030232.1	2.62	11.41	0	9.13	12.61	0
GAIF01030233.1	1.04	0.63	0.51	0.4	0.19	0.13
GAIF01030235.1	0	0.12	0.12	0	0	0
GAIF01030236.1	81.19	55.67	54.78	69	24.1	19.92
GAIF01030238.1	1.93	0.88	1.22	0.35	0.24	0.11
GAIF01030239.1	0	0.2	0	1.36	5.57	0.12
GAIF01030240.1	11.46	10.36	5.41	15.53	19.47	21.86
GAIF01030241.1	11.05	17.3	11.53	33.85	22.73	2.57
GAIF01030242.1	0	0	0	0	0.17	0
GAIF01030243.1	3.87	3.1	3.88	8.59	5.75	7.53
GAIF01030244.1	0	0.22	0	0.72	5.45	0
GAIF01030245.1	15.76	16.55	11.26	39.83	59.27	8.45
GAIF01030246.1	0.37	0.24	0.16	2.81	6.62	0.24
GAIF01030247.1	30.71	32.01	36.81	45.24	27.81	21.08
GAIF01030248.1	171.78	61.23	21.87	190.07	135.79	14.76
GAIF01030249.1	152.5	70.92	82.26	130.71	96.89	207.1
GAIF01030250.1	126.26	173.03	266.42	77.67	49.07	28.6
GAIF01030251.1	0	0	0.04	0	0	0
GAIF01030252.1	4.51	4.74	3.95	2.29	2.07	0.56
GAIF01030254.1	0	0.05	0	0	0	0.05
GAIF01030256.1	0.3	0.17	0	1.5	3.57	0.08
GAIF01030257.1	10.1	4.42	6.78	6.45	3.15	5.45
GAIF01030258.1	0	0	0	0	0.06	0

GAIF01030259.1	17.49	18.5	27.75	40.53	15.99	13.97
GAIF01030260.1	6.4	7.32	4.48	6.01	2.77	0.97
GAIF01030261.1	0.21	0.23	0	0.48	0.75	0.23
GAIF01030262.1	3.76	9.93	3.15	16.25	28.5	0.17
GAIF01030264.1	0.08	0.09	0	0.64	1.04	0.26
GAIF01030265.1	0.38	0	0	0.87	0.3	0
GAIF01030266.1	0	0	0	0	0.31	0
GAIF01030267.1	0.9	0.63	0.32	10.87	22.45	0.07
GAIF01030270.1	0.75	0.16	0	2.71	12.42	0.08
GAIF01030271.1	0	0	0	0.04	0	0
GAIF01030273.1	1.68	1.51	1.04	3.49	3.53	0.04
GAIF01030274.1	10.22	16.34	10.22	11.95	10.64	1.43
GAIF01030277.1	0.63	0.66	0.45	2.98	2.72	0.43
GAIF01030278.1	9.5	9.2	4.74	2.56	1.92	0.43
GAIF01030280.1	0	0	0	0.01	0	0
GAIF01030281.1	0	0	0	0.04	0.17	0
GAIF01030282.1	18.81	13.7	11.71	10.58	6.06	4.27
GAIF01030283.1	1.7	0.67	0.51	8	16.02	1.53
GAIF01030284.1	0	0	0	0.17	0	0
GAIF01030285.1	0	0	0	0	0.22	0
GAIF01030287.1	35.25	26.29	27.67	44.9	39.89	17.06
GAIF01030291.1	0	0	0	0.25	0.22	0
GAIF01030292.1	3.38	0.91	0	2.34	3.37	0.54
GAIF01030293.1	0.44	0	0.09	0.37	2.08	0
GAIF01030295.1	0	0	0	0	0.44	0
GAIF01030297.1	15.76	20.26	5.92	10.62	10.06	1.41
GAIF01030299.1	2.25	0.13	0.13	5.63	18.96	0.3
GAIF01030301.1	135.06	249.31	266.8	118.71	103.51	41.87
GAIF01030302.1	0.72	0.87	0.09	1.45	8.94	0.27
GAIF01030303.1	0.53	0	0.46	1.6	0	2.36
GAIF01030304.1	0	0.33	0	0	0	0
GAIF01030305.1	0	0	0	15.42	0	2.55
GAIF01030306.1	3.27	3.77	0.86	6.14	3.38	0.22
GAIF01030307.1	5.15	3.21	0.7	7.82	13.04	7.56
GAIF01030308.1	241.81	164.02	149.99	50.97	139.01	181.83
GAIF01030309.1	0	0.28	0	0	0	0
GAIF01030310.1	0	0	0	0	0.03	0
GAIF01030312.1	4.4	12.09	4.33	4.14	6.84	1.72
GAIF01030313.1	13.94	7.73	5.82	0.99	1.16	0.86
GAIF01030314.1	9.29	1.42	0	20.03	53.65	19.57
GAIF01030316.1	0.9	1.49	0.76	2.08	1.4	0
GAIF01030317.1	0.49	0	0	0.59	1.8	0
GAIF01030319.1	2.17	0.51	0	2.4	3.6	0.46
GAIF01030320.1	0	0.07	0.26	0.41	0.36	0

GAIF01030321.1	2.92	0.67	0.72	0.4	0.13	0.19
GAIF01030322.1	0.08	0	0	0.49	1.43	1.68
GAIF01030325.1	0.06	0.26	0.53	3.61	10.22	0.13
GAIF01030327.1	0.14	0	0	0.36	1.73	0
GAIF01030328.1	0	0	0	0	0	0.1
GAIF01030329.1	0	0.1	0.09	0.09	0.1	0.09
GAIF01030330.1	0.11	0.12	0	0	0	0
GAIF01030331.1	1.97	1.55	0.51	0.1	0.05	0.69
GAIF01030333.1	0	0	0	0	0.2	0
GAIF01030334.1	34.88	28.37	38.37	55.81	1.36	31.98
GAIF01030335.1	0.42	0.77	0.62	1.49	3.07	0.16
GAIF01030337.1	0.8	0.09	0.31	0.24	0	0.13
GAIF01030338.1	0	0	0.09	0.09	0	0
GAIF01030339.1	1.13	0	0	0.97	3.54	0
GAIF01030341.1	4.38	2.42	4.75	5.37	3.68	11.12
GAIF01030342.1	4.61	4.55	1.84	2.43	2.27	0.92
GAIF01030343.1	9.43	11.67	12.69	10.17	8.16	2.38
GAIF01030344.1	132.1	154.61	225.48	106.27	76.86	77.22
GAIF01030345.1	0	0	0	0	0.13	0
GAIF01030346.1	4.41	9.09	10.74	6.22	3.04	8.87
GAIF01030347.1	0	2.61	1.82	0	0.07	0
GAIF01030348.1	0	0.04	0	0	0.14	0
GAIF01030350.1	14.05	25.31	28.87	10.12	31.55	92
GAIF01030351.1	29.35	25.01	12.95	76.72	102.9	10.99
GAIF01030353.1	2.82	1.88	0.05	0.8	9.98	0.65
GAIF01030355.1	8.74	9.5	0	0	0	9.58
GAIF01030356.1	0.61	0.22	0.89	0	0	0
GAIF01030357.1	3.41	2.92	3.27	5.89	28.51	16.61
GAIF01030359.1	0.19	0.05	0.06	0.35	0.73	0.7
GAIF01030363.1	4.69	0.37	0.19	2.37	4.33	4.16
GAIF01030364.1	0	0.12	0.06	0.06	0.13	0
GAIF01030365.1	49.91	73.37	79.82	85.53	52.83	22.79
GAIF01030366.1	12.31	11.25	7.18	37.04	25.54	3.84
GAIF01030368.1	0	0	0	0.13	0.27	0
GAIF01030369.1	1.64	3.98	0.74	9.61	12.63	0.11
GAIF01030370.1	0.31	0	0.73	3.34	4.18	12.84
GAIF01030371.1	77.01	34.24	0.67	38.77	130.69	7.98
GAIF01030373.1	0.61	0.57	0.72	2.45	6.68	0.2
GAIF01030376.1	12.61	11.76	19.69	18.82	11.99	9.28
GAIF01030379.1	0	0	0	0.04	0.16	0.1
GAIF01030381.1	0	0	0.07	0	0	0
GAIF01030383.1	22.89	28.75	21.3	56.52	44.03	14.19
GAIF01030385.1	0	0.11	0	0.34	0.65	0.09
GAIF01030386.1	0	0	0	2.15	7.02	58.93

GAIF01030387.1	0	0	0	4.06	1.87	0
GAIF01030388.1	0	0.05	0.09	0	0	0
GAIF01030389.1	0.23	0	0	0.22	0.45	0
GAIF01030393.1	0.29	0.1	0	1.23	6.61	0
GAIF01030394.1	0.68	0.41	0	2.61	22.44	0.18
GAIF01030395.1	46.92	39.53	29.68	12.13	9.88	10.87
GAIF01030396.1	0	3.02	0.95	0	0.07	0.14
GAIF01030397.1	0.32	0.09	0.09	0.65	3.19	0.27
GAIF01030398.1	0	0	0	0.11	0.01	0
GAIF01030399.1	0	0	0	0.04	0.05	0
GAIF01030400.1	1.74	0.91	2.59	8.21	6.75	0.66
GAIF01030401.1	0.02	0.02	0	0.02	0	0.02
GAIF01030402.1	0.01	0.01	0.02	0	0	0
GAIF01030403.1	19.76	43.16	10.78	31.98	42.98	2.64
GAIF01030404.1	14.22	15.69	13.98	30.62	19.97	5.03
GAIF01030405.1	0	0	0	0	0.33	0
GAIF01030406.1	7.39	12.85	5.66	16.84	12.57	2.47
GAIF01030408.1	258.02	266.11	222.32	177.3	124.74	137.75
GAIF01030409.1	0	0	0	0	1.69	1.38
GAIF01030411.1	0.11	0.06	0	0	0	0
GAIF01030412.1	0	0	0	0	0.09	0
GAIF01030413.1	4.18	1.92	1.53	0.32	0.22	0
GAIF01030414.1	7.9	17.47	8.78	6.49	7.91	3.05
GAIF01030415.1	0	0	0	0	0.14	0
GAIF01030416.1	4.25	0.56	1.84	7.17	11.53	29.97
GAIF01030417.1	9.9	9.68	13.04	32.16	20.23	15.57
GAIF01030418.1	0	0	0	0	0.54	0
GAIF01030419.1	9.15	4.11	0	6.77	6.98	0.57
GAIF01030421.1	4.85	0.66	0.24	10.97	39.58	1.52
GAIF01030422.1	0	0	0	0.48	1.16	0
GAIF01030424.1	40.98	29.41	32.32	43.9	48.86	23.03
GAIF01030425.1	0	0.1	0	0	0.74	0
GAIF01030427.1	0.33	0.19	0.17	0.76	5.97	0.7
GAIF01030429.1	0.91	0.74	0.5	3.16	5.04	0.37
GAIF01030430.1	74.84	95.94	182.41	107.09	69.94	70.23
GAIF01030431.1	0	0	0	0	0	0.2
GAIF01030432.1	0.1	0.03	0	0.19	0.88	0
GAIF01030433.1	0.13	0.06	0.06	0.86	3.36	0
GAIF01030434.1	7.25	2.91	5.57	31.85	65.24	7.35
GAIF01030435.1	98.64	69.98	39.23	32.68	35.22	6.33
GAIF01030436.1	0.38	0.2	0.52	0.22	0.34	0.1
GAIF01030437.1	1.98	0.87	0.5	0.13	0.2	0.14
GAIF01030438.1	0.31	1.23	0.56	0.95	1.81	0.28
GAIF01030439.1	64.15	64.44	22.59	0.03	0.64	0

GAIF01030441.1	1.06	0.48	0.23	1.35	1.04	0
GAIF01030442.1	0	0	0	0.03	0.16	0
GAIF01030443.1	0.2	0	0	2.4	0	1.43
GAIF01030444.1	0.59	0.52	8.67	7.39	1.61	3.71
GAIF01030446.1	0	0.13	0	0.13	0.36	0
GAIF01030447.1	0.04	0	0.02	0.11	0.25	0.02
GAIF01030448.1	0	0.38	0.43	0	0.05	0.29
GAIF01030449.1	0	0	0	0.12	0	0
GAIF01030450.1	180.32	198.24	467.31	155.27	121.09	401.87
GAIF01030451.1	9.58	5.57	6.27	37.85	82.58	26.7
GAIF01030452.1	0.14	0.1	0	1.78	3.58	0.31
GAIF01030453.1	0.17	0.1	0	0.1	0	0
GAIF01030455.1	0.36	0	0	0.19	0	50.05
GAIF01030456.1	13.07	16.58	8.31	60.67	36.12	6.19
GAIF01030457.1	23.19	18.48	9.27	38.15	41.69	7.17
GAIF01030458.1	0	0	0	0.03	0.03	0
GAIF01030459.1	254.16	339.39	167.66	224.02	185.69	31.29
GAIF01030460.1	0	4.52	0	0	0.13	0
GAIF01030462.1	2.95	1.7	2.1	7.11	5.69	2.65
GAIF01030463.1	13.3	12.43	15.64	24.08	35.66	109.03
GAIF01030464.1	0.19	0.2	0.05	1.96	4.9	0.1
GAIF01030465.1	7.52	4.27	8.31	14.06	20.1	100.03
GAIF01030467.1	0.05	0.05	0	0.47	2.88	0.05
GAIF01030469.1	0	0	0	0.02	0	0
GAIF01030470.1	3.48	1.08	0	4.43	0.71	0
GAIF01030471.1	0.07	0	0	0.12	0.38	0
GAIF01030472.1	0	0.14	0	0.29	0.82	0.21
GAIF01030473.1	1.33	0.33	1.8	1.23	0.94	1.28
GAIF01030474.1	5.33	5.77	8.09	2.59	5.62	5.74
GAIF01030475.1	0	0	0	0	0.08	0
GAIF01030477.1	1.4	1.87	0	0	0	0
GAIF01030478.1	0	0.13	0	0	0.95	0
GAIF01030479.1	1.26	0.32	1.55	1.03	0.36	0
GAIF01030480.1	0.04	0	0	0.05	0	0
GAIF01030481.1	46.94	13.84	3.97	370.57	853.56	25.61
GAIF01030482.1	0	0	0	0	0.12	0
GAIF01030483.1	0.26	0	0.35	2.87	3.1	4.01
GAIF01030485.1	0.52	0	0	0	0	0
GAIF01030486.1	0.06	0	0.04	0	0.02	0
GAIF01030487.1	0	1.86	1.03	0	0	0.38
GAIF01030488.1	15.2	7.12	6.71	4.66	3.38	2.26
GAIF01030489.1	129.13	144.46	264.81	126.01	94.35	142.49
GAIF01030490.1	0.07	0	0	0.4	0.99	0
GAIF01030491.1	19.12	7.86	4.71	25.11	33.45	18.69

GAIF01030493.1	0	0	0	0.14	0.44	0
GAIF01030494.1	0	12.58	0	31.6	0	16.11
GAIF01030495.1	0.18	0	0	0.1	0	0
GAIF01030496.1	0	0	0	0.45	2.4	0.22
GAIF01030497.1	3.01	4.05	1.47	4.6	3.31	0.53
GAIF01030500.1	224.22	86.25	170.21	202.74	204.77	493.24
GAIF01030501.1	10.5	13.86	4.33	31.51	25.72	1.43
GAIF01030502.1	0	0	0	0	0.04	0
GAIF01030504.1	0	0	0	0	0.02	0
GAIF01030505.1	0.08	0.04	0	0.8	3.81	0.13
GAIF01030506.1	3.38	4.04	6.19	3.27	1.52	3.75
GAIF01030507.1	0.43	0	0.75	0.59	0	0
GAIF01030511.1	0	0	0	1.34	3.06	0.91
GAIF01030513.1	0.06	0.16	0.16	0.43	0.78	0.05
GAIF01030514.1	54.59	65.71	76.95	81.15	96.23	114.67
GAIF01030515.1	12.73	14.74	14.31	29.62	22.03	4.83
GAIF01030516.1	6.55	9.53	13.01	17.26	11.19	10.22
GAIF01030519.1	2.99	0.13	0.22	11.37	45.34	3.78
GAIF01030520.1	0.44	0.18	0	1.87	10.42	0.31
GAIF01030521.1	0	0	0	0	0	0.42
GAIF01030524.1	0	0.31	0	0.11	0.8	0
GAIF01030525.1	12.77	2.07	0.52	1.22	1.95	0
GAIF01030526.1	0	0	0	0	1.3	0
GAIF01030527.1	29.94	30.22	36.81	35.93	21.92	8.1
GAIF01030528.1	0	0.14	0.14	0.44	3.69	1.27
GAIF01030530.1	0.39	0	0	0.09	0	0
GAIF01030531.1	3.58	6.15	8.36	9.64	13.04	1.53
GAIF01030532.1	0.83	0.48	0.47	1.68	2.17	1.05
GAIF01030534.1	0.52	0.07	0.02	3.85	18.07	0.22
GAIF01030538.1	0	0	0	0.15	4.86	0
GAIF01030539.1	35.71	21.97	23.08	66.26	36.45	14.5
GAIF01030540.1	7.2	12.29	5.93	1.28	2.65	0.22
GAIF01030541.1	42.92	55.06	29.65	30.32	20.53	1.78
GAIF01030542.1	0	0	0.11	0	0.25	0
GAIF01030543.1	0.11	0	0	0.13	0.67	0
GAIF01030544.1	0.36	0.71	0.15	2.22	2.75	0.2
GAIF01030546.1	7.85	12.19	15.12	11.34	6.55	8.65
GAIF01030547.1	2.05	0.28	1.16	3.38	0	0.78
GAIF01030549.1	0	0	0.14	0	0	0
GAIF01030551.1	720.35	1261.81	1283.78	94.3	73.92	88.29
GAIF01030552.1	0.58	0.16	0.08	0.08	0.35	0
GAIF01030553.1	0	0	0	0	0.22	0
GAIF01030554.1	0.07	0	0	1.41	3.73	0.07
GAIF01030555.1	19.39	6.18	3.18	23.74	12.8	11.09

GAIF01030556.1	0.15	0	0	0.34	2.82	0.16
GAIF01030558.1	0.37	1	0.34	0.07	0	0.07
GAIF01030559.1	199.53	315.36	526.84	970.48	584.94	355.53
GAIF01030561.1	0.13	0	0	0.45	1.67	0
GAIF01030562.1	15.67	7.45	6.62	20.81	32.57	22.98
GAIF01030565.1	0.93	1.01	0.93	4.96	4.37	0.33
GAIF01030567.1	67.36	77.44	29.15	236.48	158.95	9.45
GAIF01030568.1	23.89	29.84	28.95	80.7	55.03	26.16
GAIF01030569.1	0	0.25	0.26	0.27	0.28	0.13
GAIF01030570.1	0	0	0	0	0	21.42
GAIF01030571.1	0	0	0.2	0	0	0
GAIF01030573.1	0.36	0.21	0.04	1.06	1.31	0
GAIF01030574.1	3.84	2.52	0.9	4.59	3.57	0
GAIF01030575.1	82.49	66.42	39.5	249.54	186.16	13.52
GAIF01030578.1	0	0	0.05	0.1	0.16	0
GAIF01030579.1	0	0	0.05	0	0	0
GAIF01030581.1	0	0	0	0	0.24	0
GAIF01030582.1	0.15	0	0	0	0.1	0
GAIF01030583.1	52.45	39.19	24.59	57.3	42.6	8.12
GAIF01030586.1	66.51	99.85	93.34	142.2	128.03	67.53
GAIF01030587.1	0.32	0	0	0.06	0.32	0
GAIF01030588.1	0	0	1.34	0	0	0
GAIF01030589.1	0.75	0.25	0.11	5.03	13.95	5.29
GAIF01030590.1	0.2	0	0.14	0	1.48	0
GAIF01030591.1	0	0.04	0	0.05	0	0
GAIF01030593.1	0	0	0	0	0.09	0
GAIF01030594.1	0.15	0	0	0	0.73	0
GAIF01030596.1	0.35	0.23	0.3	0.45	0.33	0.15
GAIF01030597.1	1.01	1.9	0.32	6.39	4.35	0.56
GAIF01030599.1	0	0	0	0	0.05	0
GAIF01030601.1	31.79	6.6	9.24	21.57	5.39	3.44
GAIF01030603.1	0	0	0	0.37	0	0
GAIF01030604.1	0	0.08	0	0	0	0.02
GAIF01030605.1	8.34	7.69	25.73	27.38	19.48	17.85
GAIF01030606.1	0.03	0.06	0	0.1	0.17	0
GAIF01030607.1	21.18	5.79	2.65	2.65	3.51	8.53
GAIF01030609.1	0	0	0.23	0	0.3	0
GAIF01030612.1	0.08	0.51	0.43	2.27	0.38	0
GAIF01030614.1	44.6	38.91	36.21	126.08	55.88	35.45
GAIF01030615.1	0.16	0.23	0.29	0.73	5.07	0.06
GAIF01030616.1	3.28	1.56	0.34	2.38	0.12	0.11
GAIF01030617.1	1.53	0	0	0	6.46	4.31
GAIF01030618.1	0.4	0	1.34	0.57	0	0.64
GAIF01030620.1	0	0	0.05	0.06	0.29	0

GAIF01030621.1	0.1	0.19	1.17	0	0.04	0.23
GAIF01030622.1	9.88	9.43	3.59	15.27	16.65	0.7
GAIF01030623.1	0	0.12	0	0	1.68	0
GAIF01030624.1	0	0	0	0	0.16	0
GAIF01030625.1	1.02	1.22	1.28	0.9	0.89	0.77
GAIF01030626.1	1.87	5.15	4.05	6.37	0.82	0
GAIF01030629.1	1.72	0.87	0	4.52	9.65	0
GAIF01030630.1	63.62	111.77	54.77	12.93	3.37	0.93
GAIF01030631.1	1.86	2.3	0.91	0.67	0.85	0.11
GAIF01030632.1	0	0	0	1.17	3.38	0.18
GAIF01030633.1	9.78	9.85	3.74	19.47	22.55	0.49
GAIF01030634.1	11.02	14.81	12.09	28.83	17.53	3.7
GAIF01030637.1	0.08	0	0	0	0	0
GAIF01030639.1	0.17	0.06	0.01	0	0.68	0
GAIF01030642.1	0.05	0	0	0.12	0.19	0.91
GAIF01030643.1	0.07	0.11	0	0.01	0	0
GAIF01030644.1	0.19	0.08	0	0.98	4.23	0.24
GAIF01030645.1	0.59	0	0	0	0	0.07
GAIF01030646.1	0	0.08	0.05	0	0.2	0
GAIF01030649.1	0	0.09	0	0	0	0
GAIF01030651.1	0	0	0	0.21	0.14	0
GAIF01030653.1	0	1.62	2.94	0	0.12	0.65
GAIF01030654.1	716.76	1072.59	477.74	220.59	220.91	364.06
GAIF01030655.1	4.87	3.85	2.43	1.2	0.83	0.05
GAIF01030656.1	1.95	1.74	0.72	2.49	4.11	1.23
GAIF01030657.1	18.68	18.52	12.52	20.03	21.19	3.19
GAIF01030658.1	0	0.14	0	0	0.16	0
GAIF01030660.1	0	0	0	0.19	0	0
GAIF01030661.1	1.27	0.55	0	0.39	0.2	0
GAIF01030662.1	0.16	0	0.35	1.66	8.61	0
GAIF01030665.1	0	0	0.02	0.1	0.5	0.12
GAIF01030666.1	8.47	11.33	11.84	12.53	6.49	4.34
GAIF01030667.1	10.08	1.33	4.82	0	1.09	7.78
GAIF01030668.1	0	0	0	0.04	0.02	0
GAIF01030670.1	0	0	0	0	0.06	0
GAIF01030671.1	7.59	7.42	11	15.07	9.93	12.74
GAIF01030672.1	0.13	0.25	0.15	0.19	0.22	0.17
GAIF01030673.1	0	0.5	0.5	0	0	1.09
GAIF01030674.1	25.74	28.09	26.44	48.23	41.13	16.01
GAIF01030675.1	0	0	0	0.07	0	0
GAIF01030676.1	41.33	12.39	12.24	34.3	48.24	196.12
GAIF01030677.1	0.71	0.52	0.21	1.16	3.08	0
GAIF01030678.1	0	0.1	0	0	0	0
GAIF01030679.1	20.79	12.32	6.05	54.61	63.31	13.95

GAIF01030680.1	18.21	21.44	5.62	23.3	24.47	1.68
GAIF01030681.1	3.44	2.02	2.55	0.43	0.45	0.31
GAIF01030682.1	0	0	0	0.04	0	0
GAIF01030684.1	0	0	0	0	0.28	0
GAIF01030685.1	0.26	0	0	0	0	0
GAIF01030686.1	1.55	0.1	0.03	10.66	23.27	2.09
GAIF01030687.1	0	0.07	0	0	0.18	0
GAIF01030688.1	11.15	12.01	11.84	48.85	56.78	6.62
GAIF01030689.1	1.71	1.29	0.33	1.38	2.37	0.61
GAIF01030690.1	0.09	0	0	0.29	0	0
GAIF01030691.1	14.25	18.02	11.1	40.76	21.54	7.37
GAIF01030692.1	151.74	184.68	518.26	138.83	99.97	343.86
GAIF01030693.1	12.9	13.59	10.65	30.71	22.06	3.37
GAIF01030694.1	6.97	4.95	4.47	17.97	19.84	5.08
GAIF01030695.1	16.9	26.14	32	33.3	16.23	11.5
GAIF01030698.1	37.27	43.23	54.74	127.82	78.26	40.15
GAIF01030699.1	0.08	0	0	0.15	1.28	0
GAIF01030700.1	0.35	0	0.37	0	0	1.18
GAIF01030701.1	0	0	0	0	0.2	0
GAIF01030703.1	16.12	36.06	26.85	46.46	24.35	11
GAIF01030704.1	0.23	0.47	0.45	1.35	2.41	0.05
GAIF01030705.1	0.39	0.12	0.12	1.45	2.87	0.1
GAIF01030707.1	166.58	102.79	70.73	880	795.19	171.58
GAIF01030709.1	0.12	0.98	0.67	0.58	0.18	0.12
GAIF01030711.1	14.95	20.31	4.86	23.42	34.8	17.38
GAIF01030712.1	28.4	18.11	15.35	27.2	29.59	10.24
GAIF01030713.1	0.19	0.73	1.31	0	0.23	0.42
GAIF01030714.1	0	0.11	0.14	0	0	0
GAIF01030715.1	1.51	0.55	0.37	0.49	1.42	0.09
GAIF01030716.1	18.12	24.62	19.89	1.28	0.88	0.54
GAIF01030717.1	5.69	1.54	2.85	3.71	2.94	0.77
GAIF01030718.1	2.65	4.1	4.71	1.82	4.02	1.03
GAIF01030720.1	20.42	18.19	21.32	31.12	20.31	20.96
GAIF01030721.1	3.8	2.97	2	5.85	2.39	0.73
GAIF01030722.1	0.18	0.2	0	1.67	3.83	0.2
GAIF01030723.1	0	0	0	0	0.1	0
GAIF01030725.1	0	0.77	0.56	0	0	0.06
GAIF01030727.1	0	0	0	0.12	0.24	0.11
GAIF01030729.1	0	0	0	0.19	0.47	0.05
GAIF01030731.1	1880.6	1215.89	3052.69	501.86	112.01	336.74
GAIF01030732.1	59.16	79.54	54.44	131.77	73.41	15.83
GAIF01030734.1	0.96	6.44	2.52	13.25	10.4	2.96
GAIF01030736.1	0	0	0	0	0.07	0
GAIF01030737.1	0.06	0.19	0.26	0.41	0.21	0.19

GAIF01030739.1	0	0.93	7.69	1.85	5.43	4.2
GAIF01030741.1	0	0	0	0.33	0	0
GAIF01030742.1	3.98	0	0	5.14	27.73	1.03
GAIF01030743.1	0.32	0.71	0	0.24	0.49	0.09
GAIF01030744.1	1.92	0.25	0	5.97	18.05	1.29
GAIF01030745.1	5.84	6.46	7.37	21.05	10.41	3.78
GAIF01030746.1	0	0	0.16	0	0	0
GAIF01030748.1	7.08	8	5.21	10.06	4.3	2.05
GAIF01030749.1	24.34	176.12	437.72	1.15	2.08	53.51
GAIF01030750.1	0	0	0.12	2.38	5.06	0.24
GAIF01030753.1	2.6	5.62	0	14.42	18.18	0
GAIF01030754.1	3.92	0.4	0	2.82	2.5	0.81
GAIF01030756.1	12.18	18.26	6.33	19.42	40.81	1.31
GAIF01030757.1	0	0	0.05	0.29	2.22	0.03
GAIF01030760.1	1.61	4.92	2.14	0.93	4.25	0.37
GAIF01030762.1	39.94	41.49	55.03	21.01	9.82	14.69
GAIF01030763.1	47.27	12.34	18.68	4.39	0.97	0.99
GAIF01030765.1	0.44	0.47	0	1.52	3.15	0.24
GAIF01030766.1	0	1.4	2.16	0	0	0.17
GAIF01030768.1	4.45	8.85	4.03	11.99	32.08	0.31
GAIF01030769.1	129.57	152.37	92.87	0	0.15	0.14
GAIF01030770.1	0.6	0.09	0.09	1	2.78	0
GAIF01030771.1	0.82	1.19	0.15	0.16	0.66	0.3
GAIF01030772.1	1.98	1.32	2.84	7.42	5.86	2.85
GAIF01030773.1	0.08	0.37	0.29	0.09	0.18	0.25
GAIF01030774.1	0.18	0.07	0	0.14	0.07	0.07
GAIF01030775.1	5.52	17.64	5.66	16.24	9.62	0.24
GAIF01030776.1	27.65	3.57	0.7	31.96	58.35	1.56
GAIF01030779.1	0.66	0.38	0.17	2.21	8.43	0.44
GAIF01030780.1	730.52	424.98	157.67	358.72	286.58	250.12
GAIF01030781.1	2.79	2.74	1.46	14.64	26.83	0.77
GAIF01030782.1	0.01	0.83	2.51	0.01	0.11	0.12
GAIF01030784.1	4.85	4.8	3.53	2.17	1.67	1.22
GAIF01030785.1	31.48	27.89	23.69	47.25	22.07	7.94
GAIF01030787.1	1.94	5.23	4.94	2.26	1.85	0.56
GAIF01030788.1	1.74	4.76	1.91	3.98	9.38	0.35
GAIF01030789.1	28.42	27.28	26.95	29.71	20.05	15.75
GAIF01030790.1	0	0.11	0	0.68	2.48	0.42
GAIF01030791.1	0	0	0.16	0	0.27	0
GAIF01030792.1	0.16	0	0	2.92	10.5	0.61
GAIF01030794.1	0	0	0.18	0.05	0.05	0.05
GAIF01030795.1	0	0	0.03	0	0	0
GAIF01030797.1	28.98	59.55	31.37	69.75	49.28	7.02
GAIF01030798.1	0.5	0.99	1.18	0.86	1.29	0.55

GAIF01030799.1	0.05	0.12	0.18	1.06	4.26	0.35
GAIF01030801.1	0	0	0	0.38	0.59	0.18
GAIF01030802.1	0.76	0.26	0.04	0.74	2.4	0.09
GAIF01030805.1	157	452.31	195.87	95.11	313.55	55.92
GAIF01030806.1	480.38	56.36	97.32	472.75	461.28	6983.56
GAIF01030807.1	32.84	25.07	26.73	27.05	20.79	13.01
GAIF01030808.1	18.47	37.73	72.89	25.13	20.96	23.14
GAIF01030809.1	0.25	0.15	0.13	1.29	4.82	0.13
GAIF01030810.1	0	0	0	0	0.65	0
GAIF01030811.1	0.13	0	0	0.08	0.04	0.04
GAIF01030813.1	0	0	0	0	0.42	0
GAIF01030814.1	9.46	7.6	20.98	1.6	0.24	1.3
GAIF01030815.1	5.38	7.94	6.3	8.5	5.76	2.33
GAIF01030816.1	0	0	0.34	0	0	0
GAIF01030817.1	7.66	5.62	4.54	36.61	35.27	6.07
GAIF01030819.1	0	0	0	0	10.38	0
GAIF01030820.1	14.37	13.37	9.14	28.1	17.55	9.29
GAIF01030825.1	0.1	0.22	0.22	0.24	0	0.45
GAIF01030826.1	0	0	0	0.09	0.09	0
GAIF01030827.1	0.24	0	0	0	0.29	1.08
GAIF01030828.1	0	0.04	0	0.43	0.99	0
GAIF01030829.1	3	3.38	7.83	1.21	0.87	4.81
GAIF01030830.1	5	2.45	1.37	16.12	37.13	6.33
GAIF01030832.1	0.33	0.72	0.36	0.29	0	0.36
GAIF01030835.1	9.81	17.3	16.02	26.85	15.35	8.24
GAIF01030836.1	15.18	7.21	7.99	34.19	76.4	4.1
GAIF01030838.1	0	0	0	3.65	3.92	0
GAIF01030839.1	28.55	21.94	47.7	84.82	65.04	45.02
GAIF01030840.1	0.48	0.43	0	1.11	0	0
GAIF01030841.1	0	0	0	0.04	0.05	0.06
GAIF01030842.1	0	0	0	1.56	0	0
GAIF01030843.1	0.07	0	0	0	0	0
GAIF01030845.1	5.18	2.4	2.48	3.86	3.3	3.79
GAIF01030848.1	58.47	79.86	92.81	91.82	65.06	73.23
GAIF01030849.1	0.09	0.04	0	0.15	0.44	0.16
GAIF01030850.1	1.15	0.21	0.58	0.29	0.71	0.6
GAIF01030851.1	0.15	0.32	0	0	0.18	0
GAIF01030853.1	0	0.14	0	1.29	6.35	0
GAIF01030856.1	0.35	0.13	0	1.66	7.42	0.77
GAIF01030859.1	56.08	57.13	81.58	131.16	103.65	86.89
GAIF01030863.1	0.1	0	0	0.12	0.97	0
GAIF01030864.1	21.51	26.21	27.68	36.23	19.29	12.66
GAIF01030865.1	23.38	2.8	8.48	17.43	11.09	38.77
GAIF01030866.1	13.48	10.69	11.74	19.97	8.03	24.7

GAIF01030868.1	0	0	0.16	0	0	0
GAIF01030869.1	28.74	30.06	7.87	18.27	27.63	6.63
GAIF01030871.1	0	0	0	0	0	0.56
GAIF01030872.1	0	0	0.37	0.15	0	0
GAIF01030873.1	464.64	225.67	462.16	349.27	137.54	213.95
GAIF01030874.1	137.44	322.94	193.13	17.05	23.08	26.86
GAIF01030876.1	0	0	0	0.42	2.18	0
GAIF01030877.1	10.51	9.41	16.18	11.12	3.31	11.28
GAIF01030878.1	19	13.82	11.17	29.72	47.94	6.97
GAIF01030880.1	4.35	2.87	6.37	1.38	0.48	1.6
GAIF01030881.1	0	0	0.27	0.19	7.36	1.53
GAIF01030882.1	3017.55	1826.06	785.37	1790.58	1544.97	528.67
GAIF01030883.1	0	0.1	0.05	0	0.55	0.1
GAIF01030886.1	38.45	33.91	42.34	28.75	25.36	17.38
GAIF01030887.1	56.18	43.78	26.4	147.95	179.97	15.9
GAIF01030888.1	0	0.11	0	0.06	0	0
GAIF01030889.1	6.75	0.99	0	8.26	25.23	35.59
GAIF01030890.1	2.34	1.19	0.64	8.07	12.85	1.69
GAIF01030891.1	15.97	16.82	42.15	31.95	23.55	28.57
GAIF01030892.1	0	0	0	0.04	0	0
GAIF01030893.1	1.67	1.83	1.86	5.78	3.65	20.48
GAIF01030894.1	0	0.1	0	0.74	2.64	0.3
GAIF01030898.1	0	0	0	0.23	1.42	0
GAIF01030899.1	4.88	33.26	12.16	1.16	9.41	1.93
GAIF01030901.1	17.59	26.93	18.1	4.49	7.22	1.36
GAIF01030902.1	9.68	4.68	2.42	23.06	32.3	5.77
GAIF01030903.1	8.73	10.44	9.63	21.08	19.42	7.02
GAIF01030906.1	0	12.95	3.89	0	0	0.19
GAIF01030907.1	0	0	0	0	0.91	0.25
GAIF01030908.1	0	0.24	0	0.26	0.39	0.1
GAIF01030909.1	0	0.1	0.05	0.17	0.29	0
GAIF01030912.1	14.12	2.29	1.69	107.43	95.66	0.65
GAIF01030914.1	0.01	0	0.04	0.12	0	0.01
GAIF01030915.1	0.08	0.07	0	0.08	0	0.04
GAIF01030916.1	1.8	0.29	0.15	1.73	6.37	3.46
GAIF01030918.1	0	0	0	0	0.18	0
GAIF01030919.1	0	0.13	0.05	0.25	0.45	0.09
GAIF01030920.1	0	0	0	0.22	0	0
GAIF01030922.1	0.12	0	0	0	0.14	0
GAIF01030924.1	0.4	0.24	0.35	0.81	3.54	0.55
GAIF01030928.1	6.86	2.99	0	14.52	16.29	7.72
GAIF01030929.1	0.58	0	0.11	1.86	6.97	0.11
GAIF01030930.1	0.04	0.04	0	0.13	0.18	0
GAIF01030933.1	0	0	0.2	0	0	0

GAIF01030934.1	0	0.11	0	0	0	0
GAIF01030935.1	0.66	2.59	1.9	1.62	0.37	0
GAIF01030937.1	0	0	0	0.63	3.43	0
GAIF01030940.1	116.42	43.06	38.16	121.67	174.24	87.82
GAIF01030941.1	0	3.11	0	0	37.51	0
GAIF01030942.1	13.26	18.14	3.21	19.74	40.07	0.77
GAIF01030943.1	0.06	0	0	0.07	0.22	0
GAIF01030946.1	0.11	0.12	0.06	0.25	0	0
GAIF01030948.1	0.2	0	0.27	0.61	0.43	0.21
GAIF01030951.1	0	0.16	0	0.17	0.18	0
GAIF01030952.1	0.2	0	0.22	0.72	1.99	0.11
GAIF01030955.1	0.22	0.05	0.17	0	0.17	0
GAIF01030957.1	2.63	0.23	0.31	2.46	3.31	1.75
GAIF01030958.1	9.5	13.77	4.91	7.92	11.04	1.28
GAIF01030960.1	7.43	3.86	2.46	20.08	23.31	10.02
GAIF01030961.1	0	0	0	0	0	0.08
GAIF01030962.1	0	0	0	0	0	0.07
GAIF01030963.1	0.54	0.08	0.34	1.61	1.58	0
GAIF01030966.1	0.12	0	0	0.13	0.56	0
GAIF01030967.1	0.21	0.22	0	0.03	0	0
GAIF01030968.1	0.54	0.23	0.2	2.38	1.47	0.47
GAIF01030970.1	19.33	19.03	15.79	40.25	25.95	10.18
GAIF01030971.1	0.05	0.32	0.31	0	0	0.05
GAIF01030972.1	0	0.07	0	0	0	0
GAIF01030973.1	491.43	426.27	638.87	382.44	145.79	242.83
GAIF01030974.1	0.46	2.01	0.07	0.15	0.27	0.34
GAIF01030975.1	0.04	0.06	0.08	0.17	0.22	0.18
GAIF01030978.1	20.58	4.6	4.94	0.19	0.06	0.06
GAIF01030979.1	1.24	2.36	0.14	4.28	14.6	0.61
GAIF01030980.1	0.5	0	0	1	3.62	0.16
GAIF01030982.1	0.07	0	0	0	0	0
GAIF01030983.1	0.25	0.38	0.49	1.68	5.45	0.3
GAIF01030984.1	0.66	1.33	1.02	1.08	2.07	0.58
GAIF01030985.1	0	0	0	0	0.28	0
GAIF01030986.1	0	0	0	0.05	0	0
GAIF01030990.1	0.93	0.23	0	2.24	10.15	0.2
GAIF01030991.1	0.1	0.15	0	1.35	4.37	0.08
GAIF01030992.1	8.15	5.01	4.83	32.2	57.94	22.39
GAIF01030993.1	0	0	0.04	0	0	0
GAIF01030994.1	0.92	0.29	0	1.73	5.2	0.23
GAIF01030995.1	18.53	20.05	13.7	34.44	29.45	2.7
GAIF01030996.1	0.33	0	0	0.12	0	0
GAIF01030997.1	0	0	0	0	0.19	0
GAIF01030998.1	18.47	26.3	13.44	47.47	26.36	4.85

GAIF01030999.1	0	0.07	0.07	0	0	0
GAIF01031000.1	0	0	0	0	0.06	0
GAIF01031002.1	15.25	25.44	25.37	37.36	21.04	10.42
GAIF01031003.1	0.32	0.21	0	1.26	3.56	0.07
GAIF01031004.1	0.21	0.08	0.2	0.35	0.37	0.04
GAIF01031005.1	22.29	22.32	21.64	12.64	10.14	11.27
GAIF01031006.1	1.32	0.51	1.06	1.23	0.91	0.64
GAIF01031007.1	1.81	3.7	2.95	1.64	3.01	0
GAIF01031009.1	15.91	21.64	28.74	66.22	54.47	21.15
GAIF01031010.1	4.24	1.64	0.77	0.46	0.18	0.24
GAIF01031011.1	0	0	0	0	2.44	0.56
GAIF01031012.1	22.03	33.74	10.66	35.35	33.31	0.68
GAIF01031013.1	8.33	4.38	0.29	28.99	81.24	14.51
GAIF01031014.1	0	0	0	0.72	0.62	0
GAIF01031021.1	107.88	51.3	50.28	18.94	10.07	1.64
GAIF01031022.1	0.23	0	0	0.18	1	0.17
GAIF01031024.1	9.88	7.39	1.27	46	35.28	4.27
GAIF01031026.1	0	0.04	0	0	0	0
GAIF01031027.1	0	0.08	0.23	0	0.12	0.08
GAIF01031028.1	0	0.47	0.27	0	0	0.56
GAIF01031029.1	0.15	0.04	0	0.38	1.96	0.16
GAIF01031031.1	0.15	0.1	0.16	2.02	6.17	0.05
GAIF01031032.1	9.67	14.24	1.56	25.52	43.01	0.81
GAIF01031033.1	1.3	1.48	1.93	1.49	1.79	0.66
GAIF01031035.1	0	0	0	0	1.23	0
GAIF01031036.1	5.72	11.87	9.73	8.5	6.8	3.68
GAIF01031038.1	0	0.21	0.07	0.15	1.06	0.07
GAIF01031040.1	0	0.27	0	0.03	0.91	0
GAIF01031041.1	0.76	0	0	0.22	2.43	0.07
GAIF01031043.1	0	0	0	0	0.02	0
GAIF01031044.1	11.23	23.43	12.67	24.04	15.93	3.52
GAIF01031045.1	8.21	10.01	4.4	9.43	14.14	0.48
GAIF01031046.1	11.76	9.25	20.64	29.46	0	22.9
GAIF01031047.1	0.29	0.23	0	0.92	7.14	0
GAIF01031048.1	0.6	0.22	0.07	3.86	18.58	0.22
GAIF01031049.1	0	0	0	0	0.63	0
GAIF01031052.1	0.06	0.06	0	0.32	0.26	0
GAIF01031054.1	0.16	0.36	0	0	0	0
GAIF01031058.1	0.04	0	0	0.09	0.24	0
GAIF01031059.1	10.46	22.33	25.68	12.18	59.6	9.57
GAIF01031060.1	0.16	0.81	3.71	0.19	0.19	0
GAIF01031061.1	0	0	0	0.25	0	0
GAIF01031062.1	0	0.41	0.41	0	0	0
GAIF01031063.1	0.71	0	0.14	1.26	4.84	0.07

GAIF01031064.1	0.22	0.24	0.26	0.09	0	0
GAIF01031065.1	58.22	66.11	90.02	37.19	28.84	72.33
GAIF01031066.1	0.36	0.25	0	0	0.08	0
GAIF01031067.1	0.47	0	0	0	0	0
GAIF01031070.1	0	0	0	0	0.63	0.15
GAIF01031073.1	7.73	14.12	9.02	5.61	3.82	0.08
GAIF01031075.1	0.13	0	0	0.07	0.68	0.07
GAIF01031076.1	0	0	0	0	1.02	0
GAIF01031077.1	22.31	13.4	9.18	55.71	43.55	5.46
GAIF01031078.1	0.27	0	0.06	1.14	8.04	0.28
GAIF01031080.1	2.89	0.89	0	8.06	34.47	0.56
GAIF01031082.1	1.31	0	0	5.59	18.95	0.32
GAIF01031083.1	0.07	0.14	0	0	0	0
GAIF01031084.1	0	0	0.04	0	0	0
GAIF01031086.1	1.27	0.62	1.18	3.52	8.02	0.81
GAIF01031088.1	44.01	0	3.72	30.19	10.3	27.26
GAIF01031089.1	139.04	71.69	118.83	120.45	98.01	256.35
GAIF01031091.1	8.24	13.53	5.86	19.32	28.18	0.89
GAIF01031092.1	0	0	0	0.16	1.72	0
GAIF01031093.1	16.09	13.6	13.13	5.02	3.57	3.57
GAIF01031095.1	7.47	5.31	12.58	15.84	13.06	4.94
GAIF01031096.1	70.02	59.48	54.39	47.03	40.15	15.87
GAIF01031099.1	31.02	30.91	62.41	67.15	31.93	45.32
GAIF01031100.1	0	0	0	0.23	0.78	0
GAIF01031102.1	2.72	4.96	3.39	5.13	3.01	1.38
GAIF01031105.1	0	0	0.07	0	0	0
GAIF01031106.1	0.06	0.01	0.07	0.13	1.5	1.13
GAIF01031107.1	24.35	9.26	2.06	175.39	498.2	3.33
GAIF01031108.1	0	0.09	0	0	0.1	0
GAIF01031110.1	8.41	11.31	2.21	2.85	3.03	0
GAIF01031115.1	38.95	38.4	54.98	30.05	27.76	40.41
GAIF01031118.1	0	0.27	0	0	2.11	0
GAIF01031119.1	268.23	69.84	67.33	70.94	35.21	33.01
GAIF01031120.1	0	0	0	0.13	0.84	0
GAIF01031121.1	0.07	0	0	0	0	0
GAIF01031122.1	13.41	10.95	2.63	5.73	3.64	0.33
GAIF01031123.1	4.39	3.83	6.3	2.11	1.42	2.64
GAIF01031124.1	0.64	0	0.42	0	1.99	0.28
GAIF01031125.1	0	0.05	0	0.05	1.96	0.08
GAIF01031127.1	15.26	15.09	6.55	2.33	1.1	0.13
GAIF01031128.1	0	0.25	1.11	0	0	0
GAIF01031130.1	1.26	0.76	0.8	0	0	0
GAIF01031132.1	17.77	21.21	27.26	25.06	24.97	9.71
GAIF01031133.1	0	0	0	0	0.05	0

GAIF01031135.1	9.5	3.53	4.67	3.34	1.8	1.65
GAIF01031136.1	0	0.75	0.93	0	0	1.19
GAIF01031137.1	0.13	0.27	0	0.44	0.45	0.14
GAIF01031138.1	6.82	13.58	14.26	49.2	29.26	4.53
GAIF01031140.1	21.9	16.18	13.89	22.33	20.92	6
GAIF01031141.1	14.51	9.04	9.86	15.78	15.24	16.9
GAIF01031142.1	0.45	0.41	0.09	0.55	0.5	0.13
GAIF01031144.1	0.04	0.01	0	0	0.08	0
GAIF01031146.1	0.27	0.1	0	1.88	6.56	0.2
GAIF01031147.1	0.28	0.45	0.3	0	0	0.15
GAIF01031150.1	0.57	0.27	0.09	1.81	2.96	0.36
GAIF01031151.1	0	0	0	0.21	0.44	0
GAIF01031153.1	0	0	0	0	0.11	0
GAIF01031154.1	0.13	0	0	0.72	2.64	0
GAIF01031155.1	0.71	0	1.49	1.87	0	1.28
GAIF01031156.1	36.97	32.33	23.07	24.32	38.14	12.5
GAIF01031157.1	7.84	0	0	12.81	19.02	49.48
GAIF01031158.1	5.18	0.97	0	18.37	34.34	3.1
GAIF01031159.1	0.06	0	0.06	0	0.21	0
GAIF01031160.1	0.67	1.56	0.94	0.44	1.38	0.21
GAIF01031161.1	0.13	0	0.15	0	0	0
GAIF01031162.1	73.35	47.96	34.09	59.17	44.85	23.07
GAIF01031163.1	0.07	0.08	0	0.12	1.25	0
GAIF01031164.1	14.83	12.84	9.24	5.7	9.34	23.6
GAIF01031165.1	0	0.05	0	0	0.18	0
GAIF01031171.1	4.56	0.37	0.75	1.85	1.64	3.39
GAIF01031173.1	0	0.07	0	0.17	0.26	0
GAIF01031174.1	0.33	0.53	0	3.11	6.55	0
GAIF01031175.1	14.41	8.81	10.42	18.2	16.36	8.66
GAIF01031177.1	3.83	1.9	2.04	3.62	1.99	1.31
GAIF01031181.1	0.15	0.13	0	0.18	0.59	0
GAIF01031182.1	0.41	0.43	0.59	1.49	1.55	1.79
GAIF01031183.1	0	0	0	0	0.03	0
GAIF01031184.1	0	0	0	0	0.11	0
GAIF01031185.1	54.58	29.38	15.31	9.46	11.12	0.86
GAIF01031186.1	18.66	8.33	10.94	52.12	92.96	31.33
GAIF01031187.1	0	0.05	0	0	0.3	0
GAIF01031188.1	0	0	0.28	0	0	0
GAIF01031190.1	23.3	42.84	8.64	45.57	58.63	1.29
GAIF01031191.1	13.08	9.48	8.57	9.91	3.82	4.3
GAIF01031192.1	1.35	0.63	0.61	1.18	3.59	0.58
GAIF01031193.1	0.72	0.91	0.4	0.28	1.01	0.26
GAIF01031194.1	0	0	0	0	0.6	0.46
GAIF01031195.1	0	0	0	0	0.27	0

GAIF01031197.1	11.99	3.64	16.63	9.29	2.78	8.39
GAIF01031200.1	0	0.02	0	0	0	0
GAIF01031201.1	3.46	2.18	1.52	18.12	38.54	1.81
GAIF01031202.1	0.33	0	0	0.19	1.8	0
GAIF01031205.1	3.67	0.3	0.2	1.07	1.99	0.4
GAIF01031206.1	1.58	0	0	0.52	6.95	0.73
GAIF01031209.1	0	0.32	0.11	1.69	2.8	0
GAIF01031210.1	0	0.06	0	0	0.13	0
GAIF01031211.1	0	0	0	0.3	0.93	0
GAIF01031212.1	17.07	20.48	17.88	29.89	26.6	3.78
GAIF01031213.1	26.08	28.45	14.03	28.94	15.99	2.62
GAIF01031214.1	0.84	0.78	0	2.78	8.77	5.01
GAIF01031217.1	7.17	17.15	14.61	20.47	13.62	3.47
GAIF01031218.1	4.79	3.46	6.77	7.67	9.25	4.24
GAIF01031219.1	0	0.16	0.16	0	0	0
GAIF01031222.1	15.44	20.82	17.08	39.74	23.02	17.52
GAIF01031223.1	17.96	9.32	19.42	50.02	43.06	189.44
GAIF01031224.1	0	0	0	0.11	0.06	0
GAIF01031225.1	0	0	0	0	0.09	0.02
GAIF01031226.1	0	0	0	0	0.05	0
GAIF01031227.1	0	0.06	0	0	0	0
GAIF01031228.1	0.05	0.27	0.05	0	0.06	0.16
GAIF01031229.1	3.54	2.91	1.18	4.97	12.37	0.71
GAIF01031230.1	19.94	14.53	18.08	18.01	2.53	6.03
GAIF01031232.1	0.3	0.16	0.83	2.63	6.17	15.3
GAIF01031233.1	0	0	0	0.12	0	0
GAIF01031234.1	0	0	0	0.28	0	0
GAIF01031235.1	40	28.21	34.95	31.87	19.28	22.36
GAIF01031237.1	0	0	0	0	3.29	0
GAIF01031238.1	0	0.21	0.77	5.08	2.3	3.07
GAIF01031239.1	56.53	74.93	55.23	145.11	132.36	37.07
GAIF01031240.1	4.57	2.52	6.36	4.83	3.04	5.72
GAIF01031241.1	180.85	225.78	259.39	548.91	303.15	33.91
GAIF01031244.1	0.07	0.08	0	0	0	0
GAIF01031245.1	14.04	6.56	7.02	17.29	8.8	11.48
GAIF01031247.1	0	0	0	0	0.15	0
GAIF01031248.1	0	0	0	0.38	0	0
GAIF01031249.1	0.04	0	0	0.24	1.93	0
GAIF01031251.1	0	0	0	0	0.07	0
GAIF01031252.1	14.69	24.73	21.73	108.44	146.07	13.57
GAIF01031255.1	0.06	0	0	0	0	0
GAIF01031256.1	0.32	0	0.59	0.75	0.78	0.12
GAIF01031257.1	4.31	3.56	2.45	19.51	18.61	1.97
GAIF01031261.1	0.31	1.17	0	0.18	0.43	0

GAIF01031262.1	13.05	30.82	16.43	8.15	5.67	0.3
GAIF01031263.1	0	0.16	0.32	0.5	0.35	0.16
GAIF01031265.1	0.02	0.05	0	0.63	1.9	0
GAIF01031268.1	0.12	0.2	0	0	0	0
GAIF01031269.1	0	0	0	0	0.08	0
GAIF01031270.1	8.55	8.1	1.54	86.28	66.18	26.41
GAIF01031274.1	559.84	395.93	384.81	304.52	260.76	279.63
GAIF01031275.1	10.63	4.58	2.14	24.57	45.78	5.42
GAIF01031276.1	0	0	0	0	11.75	0
GAIF01031277.1	0	0	0	0.8	2.11	0
GAIF01031279.1	19.79	19.05	17.91	15.51	7.72	5.66
GAIF01031280.1	0.05	0.16	0.11	0.11	0.23	0.32
GAIF01031282.1	1.82	0	1.66	3.81	0	0
GAIF01031283.1	0.81	1.22	0.67	0	0.45	0
GAIF01031284.1	0	0.07	0	0	0	0
GAIF01031285.1	1.35	1.07	0.13	0.57	0.59	0
GAIF01031286.1	0	0	0	0.15	0.11	0
GAIF01031288.1	0	0	0	0.16	0.41	0
GAIF01031289.1	18.78	7.11	3.07	120.76	237.26	3.74
GAIF01031290.1	0.25	0.04	0	0.92	5.41	0.28
GAIF01031291.1	0.84	0.7	0	2.58	1.88	0.99
GAIF01031293.1	0.39	0.17	0.22	0.37	0	0
GAIF01031294.1	125.54	108.16	66.25	194.46	206.14	159.98
GAIF01031297.1	1.83	1.98	3.32	2.65	0.96	15.4
GAIF01031298.1	0.06	0	0	0	0	0
GAIF01031299.1	261.4	136.28	52.23	284.96	264.83	13.82
GAIF01031301.1	2.42	3.91	2.34	1.64	1.05	1.91
GAIF01031302.1	0.03	0.04	0.04	0.04	0	0.04
GAIF01031303.1	1.87	1.16	0.78	5.07	5.36	0.59
GAIF01031304.1	76.91	56.02	82.05	24.99	10.97	23.87
GAIF01031305.1	0.18	0.1	0	2.94	8.23	0.1
GAIF01031307.1	0.11	0.05	0.02	0.04	0.2	0
GAIF01031308.1	32.95	18.96	21.5	39.46	22.36	75.13
GAIF01031309.1	0	0.12	0	0	0.26	0
GAIF01031311.1	0	0	0	1.44	3.64	4.81
GAIF01031313.1	0	1.93	2.79	0	0	0.24
GAIF01031315.1	0	0	0	0.34	0.32	0
GAIF01031316.1	5.69	12.92	6.07	44.48	47.31	2.53
GAIF01031317.1	0	0	0	0	0	0.23
GAIF01031320.1	6.78	4.84	7.72	34.04	0	9.22
GAIF01031321.1	0.77	0.29	0.04	0.4	1.02	0.81
GAIF01031322.1	0.07	0	0	0.23	1.13	0.06
GAIF01031323.1	324.47	394.01	400.36	478.93	367.78	253.55
GAIF01031324.1	0	0	0	0	13.91	0

GAIF01031327.1	0	0.11	0	0.34	0	0
GAIF01031328.1	0	0.08	0	1.4	7.24	0.25
GAIF01031330.1	1.92	0.92	1.88	6.65	6.43	6.83
GAIF01031331.1	68.19	38.82	163.23	90.15	2.49	36.85
GAIF01031333.1	6.7	6.81	3.35	24.95	11.41	1.39
GAIF01031334.1	20.15	8.34	10.13	29.12	31.89	21.34
GAIF01031335.1	36.4	17.12	17.34	50.18	22.4	50.51
GAIF01031336.1	48.7	117.02	120.34	64.5	69.8	19.9
GAIF01031337.1	1.75	0.47	0.82	5.45	12.04	0.59
GAIF01031339.1	4.88	0.42	0.19	5.01	0.5	0.04
GAIF01031340.1	0.43	0.1	0.16	1.12	1.63	0.16
GAIF01031341.1	0.54	0.6	0.41	1.2	6.33	0
GAIF01031342.1	0.1	0.02	0.03	0.58	2.52	0.05
GAIF01031343.1	1.44	2.05	6.11	10	17.05	4.35
GAIF01031344.1	0	0	0	0.28	0.14	0
GAIF01031346.1	33.03	41.56	43.64	55.69	34.95	25.73
GAIF01031348.1	0	0	0	0.44	0.1	0.09
GAIF01031349.1	0.05	0	0.06	0	0	0
GAIF01031350.1	4.35	3.9	0.81	9.24	7.24	1.73
GAIF01031351.1	0	0	0.08	0	0.29	0
GAIF01031352.1	0	0.14	0	0.15	0	0
GAIF01031353.1	77.23	44.27	38.94	107.26	49.68	146.88
GAIF01031354.1	15.26	8.34	13.27	14.39	9.32	11.31
GAIF01031355.1	15.28	14.2	6.4	20.39	22.02	2.03
GAIF01031356.1	0.36	0	0	0.41	0	0.2
GAIF01031357.1	0.31	0.47	0.74	1.35	1.62	0.27
GAIF01031358.1	0.04	0.05	0	0	0	0
GAIF01031359.1	2.22	0.26	0	11.02	35.63	0.8
GAIF01031360.1	88.68	86.99	74.41	5.56	3.11	9.02
GAIF01031361.1	0.24	0.44	0.71	0.75	1.07	0.27
GAIF01031363.1	0	0.07	0	0.22	0.15	0
GAIF01031364.1	0.16	0	0	0.19	0	0.19
GAIF01031365.1	2.85	1.17	0.59	3.53	15.63	5.84
GAIF01031367.1	7.18	9.07	11.95	6.66	3.76	4.21
GAIF01031368.1	17.64	20.03	39.96	55.73	36.5	79.7
GAIF01031369.1	0.7	0.77	0.65	0.57	1.29	0.12
GAIF01031370.1	0.59	0.85	0	0.68	0.47	0
GAIF01031371.1	1.84	2.61	1.93	3	5.35	0
GAIF01031372.1	0	0.19	0	0	0.49	0
GAIF01031373.1	0	0	0	0.16	0.36	0
GAIF01031375.1	0	0	0	0.19	0.82	0
GAIF01031376.1	0.24	0.48	0.17	0.74	0.26	0.26
GAIF01031377.1	0.85	0	0	0	0	0
GAIF01031379.1	6.67	3.32	5.15	2.81	0.94	6.94

GAIF01031380.1	0	0	0	0	0.02	0
GAIF01031381.1	0	0	0	0.32	0.32	0
GAIF01031383.1	0	0	0	1.85	7.65	0
GAIF01031386.1	0.12	0	0.13	0	0.15	0
GAIF01031387.1	8.93	12.57	8.24	8.48	4.39	1.12
GAIF01031389.1	0	0.32	0	0	0	0
GAIF01031391.1	10.5	13.19	9.1	38.98	16.01	3.75
GAIF01031392.1	4.98	2.17	1.23	6.11	6.48	3.91
GAIF01031394.1	0	0	0	0	0.12	0
GAIF01031397.1	0.11	0	0.24	0	0.19	0
GAIF01031399.1	18.55	60.49	7.22	52.61	96.76	1.51
GAIF01031400.1	0	0	0	0.2	0	0
GAIF01031404.1	0.09	0	0	0.21	1.74	0
GAIF01031405.1	9.91	5.79	12.11	16.37	10.86	8.84
GAIF01031406.1	5.73	10.85	7.52	17.52	11.06	2.68
GAIF01031407.1	0	0	0	0	0.08	0
GAIF01031409.1	0.72	0.61	0.18	2.9	6.4	0.22
GAIF01031410.1	0	0	0.08	0.15	0.35	0
GAIF01031413.1	11.3	10.08	7.09	20.21	23.25	2.29
GAIF01031414.1	0	0.98	1.13	0	0	0
GAIF01031415.1	18.8	14.55	8.94	36.61	25.6	2.77
GAIF01031416.1	10.98	21.95	19.77	39.32	21.38	6.53
GAIF01031417.1	0.24	0	0	0.28	1.53	0.16
GAIF01031418.1	0	0	0.15	0	0	0
GAIF01031419.1	5.41	7.97	5.64	38.47	25.45	3.07
GAIF01031420.1	17.77	9.6	7.35	2.79	2.43	1.05
GAIF01031423.1	0	0	0	0	2.03	0
GAIF01031424.1	1.87	2.05	2.28	2.46	1.59	1.04
GAIF01031425.1	0.06	0	0	0.07	0.07	0
GAIF01031427.1	0	0.3	0.3	0	0	0
GAIF01031428.1	0	0	0	0.91	1.22	0
GAIF01031429.1	0.14	0	0	0.08	0.08	0
GAIF01031430.1	0	0	0	0.04	0	0
GAIF01031434.1	0.15	0.05	0	0.29	0.3	0
GAIF01031435.1	384.34	224.38	219.05	40.71	39.31	302.11
GAIF01031436.1	43.6	73.8	31.78	13.59	23.58	7.5
GAIF01031437.1	7.52	4.8	3.05	17.65	29.79	3.96
GAIF01031438.1	0.07	0	0.04	1.14	5.06	0.54
GAIF01031440.1	0	0	0	0.51	0.26	0
GAIF01031441.1	0	0	0.14	0.16	0.24	0
GAIF01031443.1	0	0	0	0.26	0	0
GAIF01031444.1	5.86	1.75	2.41	0.57	0	0.51
GAIF01031445.1	0.77	0.99	1.03	0	0.28	0.37
GAIF01031446.1	0.4	0.16	0.13	1.92	7.54	0.14

GAIF01031447.1	0	0	0	0	0.79	0
GAIF01031448.1	0.67	0.62	0.28	1.19	3.46	0.06
GAIF01031450.1	2.82	18.47	2.89	0.5	4.16	0.44
GAIF01031451.1	1.67	0.61	0	6.21	35.66	3.07
GAIF01031452.1	0.04	0	0.03	0.07	0.06	0
GAIF01031453.1	30.83	20.77	19.21	73	55.24	16.38
GAIF01031454.1	209.89	270.91	744.64	0	2.57	101.79
GAIF01031457.1	0.18	0	0	0	0	0
GAIF01031458.1	0.19	0	0.1	0.11	0	0
GAIF01031460.1	16.48	8.24	7.44	27.92	32.75	22.95
GAIF01031461.1	0	0	0.06	0.3	1.57	0
GAIF01031462.1	216.22	207.05	114.48	466.43	420.37	62.02
GAIF01031464.1	0	0.34	0.11	0	0	0.06
GAIF01031465.1	91.17	36.37	49.32	122.39	69.34	372.25
GAIF01031467.1	39.48	129.61	88.88	63.48	26.09	56.49
GAIF01031468.1	0	0.27	0	0.57	0.3	0
GAIF01031469.1	0.19	0	0	0.86	5.05	0.49
GAIF01031470.1	245.39	273.16	126.01	476.46	221.65	44.48
GAIF01031472.1	0.11	0.12	0.12	1.04	4.44	0
GAIF01031474.1	53.08	41.51	46.42	14.11	14.5	20.39
GAIF01031475.1	0	0	0	0	0.13	0
GAIF01031476.1	68.79	78.67	152.99	30.75	22.79	54.62
GAIF01031477.1	38.79	27.98	25.27	156.1	172.41	28.08
GAIF01031478.1	65.93	34.12	25.4	164.39	161.29	28.93
GAIF01031479.1	0	0	0	0	0.18	0
GAIF01031481.1	1610.4	451.33	650.06	1553.83	873.71	21116.1
GAIF01031483.1	0	0	0	0	0.1	0
GAIF01031484.1	26.85	31.08	17.14	37.51	31.2	9.3
GAIF01031485.1	26.35	45.19	53.78	67.97	40.62	20.66
GAIF01031486.1	0.03	0	0	0.12	0.24	0.29
GAIF01031487.1	0	0	0	0.02	0.09	0
GAIF01031488.1	2.08	0.76	0	0	0	0
GAIF01031490.1	15.84	1.56	0.2	1.58	7.93	0.59
GAIF01031491.1	7.57	2.71	2.17	9.42	15.36	8.1
GAIF01031492.1	0	0	0	0.07	0.56	0
GAIF01031493.1	1.32	0.34	0.32	3.49	24.46	0.82
GAIF01031494.1	5.37	2.58	1.06	6.48	9.13	0.87
GAIF01031495.1	0	0	0.13	0	0	0
GAIF01031496.1	4.89	10.96	7.53	5.43	4.13	0.46
GAIF01031497.1	0.54	0	0	0	1.3	1.19
GAIF01031503.1	7.57	3.43	5.68	2.34	0.91	1.11
GAIF01031504.1	0.72	1.42	1.08	5.13	7.14	0.63
GAIF01031505.1	3.49	1.11	2.31	5.68	10.78	21.25
GAIF01031506.1	0	0	0	0	0	0.51

GAIF01031507.1	2.99	3.17	3.02	1.55	1.23	0.26
GAIF01031508.1	0.79	0.29	0	0.75	1.31	0.15
GAIF01031509.1	10.8	3.46	3.02	7.26	5.25	1.54
GAIF01031510.1	1.01	1.22	2.35	1.3	1.49	1.73
GAIF01031511.1	45	69.31	30.58	7.82	4.57	1.02
GAIF01031513.1	0.03	0.03	0.03	0.36	2.27	0.21
GAIF01031517.1	0.04	0	0	0.09	0	0.04
GAIF01031518.1	0	0.13	0	0	0	0
GAIF01031519.1	0.03	0.01	0	0.02	0.03	0
GAIF01031521.1	0	0.3	0	0	0	0
GAIF01031522.1	0	0	0	0.27	1.46	0.05
GAIF01031523.1	171.25	246.5	180.18	299.55	236.33	68.31
GAIF01031526.1	0	0.21	0	0	0	0
GAIF01031527.1	0	0	0.17	0	0	0
GAIF01031529.1	0	0	0	0.46	0.95	0
GAIF01031530.1	16.43	17.14	19.76	36.09	30.07	4.73
GAIF01031531.1	10.61	3.15	0.28	67.63	201.74	1.84
GAIF01031532.1	0.06	0	0.04	0	0	0
GAIF01031534.1	15.83	16.33	19.03	16.88	26.93	11.58
GAIF01031535.1	0	2.55	0.66	0	0	0.12
GAIF01031536.1	0	0	0	0	0	2.3
GAIF01031538.1	0.03	0.04	0.23	0.12	0.04	0.08
GAIF01031539.1	0.12	0.03	0	0.54	2.18	0.07
GAIF01031540.1	0	0	0	0.2	0	0
GAIF01031541.1	17.65	4.63	9.74	28.11	21.68	2.49
GAIF01031543.1	75.73	34.27	16.86	79.06	83.65	29.62
GAIF01031544.1	0	0.13	0	0	1.04	0
GAIF01031545.1	2.91	3.03	3.2	7.78	3.96	1.26
GAIF01031546.1	33.74	0	1.8	19.57	7.36	34.51
GAIF01031547.1	0.08	0.07	0	0	0.01	0
GAIF01031548.1	0.08	0	0	0	0	0
GAIF01031550.1	37.74	24.21	16.3	35.96	2.23	2.94
GAIF01031551.1	3.67	2.39	1.96	7.27	8.94	1.91
GAIF01031552.1	7.29	4.47	8.97	17.2	10.32	11.32
GAIF01031553.1	0	0.16	0	0.6	0.03	0.08
GAIF01031554.1	0	0	0	0	0.16	0
GAIF01031556.1	0	0	0	0	0.08	0
GAIF01031558.1	0.09	0	0	0.69	5.98	0.15
GAIF01031560.1	0.09	0	0	0.76	3.2	0
GAIF01031562.1	0	0.76	0	0.32	0	0
GAIF01031563.1	0	0	0	0	0.21	0
GAIF01031566.1	0.11	0	0	0.25	2.72	0.12
GAIF01031568.1	0	0.74	0	0	0	0
GAIF01031569.1	0	0	0	0	0.1	0

GAIF01031570.1	0.19	0.07	0.17	0.15	0	0.12
GAIF01031571.1	2.39	0.69	3.15	0	0	0
GAIF01031572.1	5.07	4.18	0.81	10.65	16.98	15.82
GAIF01031574.1	5.41	0.43	0.09	16.98	45.91	15.36
GAIF01031575.1	1.36	1.26	1.71	3.44	5.08	1.44
GAIF01031576.1	0	0	0.04	0	0	0
GAIF01031577.1	0.12	0	0.16	1.1	2.77	0.39
GAIF01031579.1	1.17	1.32	0.99	5.55	6.6	0.38
GAIF01031580.1	0	0	0.42	0	0	0
GAIF01031582.1	0.19	0	0	0.49	0.66	0
GAIF01031583.1	7.09	9.3	3.57	0	0	0.25
GAIF01031584.1	0	0	0	0	0.2	0
GAIF01031586.1	0.38	0.4	0.03	1.68	4.21	0
GAIF01031589.1	1.57	0.86	0.38	0.2	0.42	0.1
GAIF01031592.1	0.13	0	0	0.16	0.97	0
GAIF01031593.1	34.67	40.39	29.51	36.95	42.09	66.84
GAIF01031594.1	0.06	0	0	0	0.18	0.12
GAIF01031596.1	1.56	0.37	0.72	11.21	16.85	1.26
GAIF01031598.1	21.85	29.92	28.37	65.84	58.57	15.14
GAIF01031599.1	0	0	0.14	0	0	0.61
GAIF01031601.1	21.92	19.72	27.34	18.95	19.11	35.98
GAIF01031602.1	3.62	0.74	0.29	38.51	164.49	16.44
GAIF01031603.1	20.76	14.58	13.71	21.23	19.84	15.12
GAIF01031604.1	2.67	2.36	0.85	1.38	1.43	0.15
GAIF01031605.1	0	14.91	6.6	0	0.39	0
GAIF01031606.1	22.89	14.21	20.02	37.82	40.26	20.66
GAIF01031607.1	0.92	0.6	1.05	0.14	0.3	0.1
GAIF01031608.1	0.31	0	0.11	0.6	1.24	0
GAIF01031609.1	0.4	0.73	0.22	1.48	1.77	0
GAIF01031611.1	0.09	0.07	0.2	0.86	3	0.37
GAIF01031613.1	1326.04	1166.66	828.16	1889.77	1729.78	2125.71
GAIF01031614.1	0.03	0.04	0	0.16	0.16	0
GAIF01031615.1	0	0.21	0	0	0	0
GAIF01031616.1	0.11	0.16	0.25	0.82	4.1	0.16
GAIF01031617.1	4.26	2.12	0	8.42	13.42	0
GAIF01031618.1	87.2	60.63	73.23	95.28	39.15	18.28
GAIF01031620.1	8.77	6.42	6.93	11.53	18.66	4.34
GAIF01031625.1	0	0	0	0	0	0.15
GAIF01031628.1	0	0	0	0	0.86	0
GAIF01031629.1	187.18	184.75	99.06	265.91	223.4	59.08
GAIF01031630.1	0.28	0	0	0	0	0
GAIF01031631.1	1.13	0.68	0.44	0.39	1.23	0.06
GAIF01031632.1	0	0	0	0	0	0.04
GAIF01031634.1	0	0	0	0	0.17	0

GAIF01031635.1	112.44	111.17	81.47	179.12	71.59	46.85
GAIF01031637.1	0	0	0	0.19	0	0
GAIF01031638.1	0	0.62	0.16	11.66	55.5	0.31
GAIF01031639.1	5.35	3.25	1.62	7.72	3.85	0.82
GAIF01031640.1	1.06	0.96	0.39	1.84	1.69	4.86
GAIF01031641.1	3.39	8.7	11.8	1.17	1.68	1.01
GAIF01031642.1	99	56.52	69.11	31.36	14.86	37.14
GAIF01031644.1	3.66	3.84	1.47	1.61	0.59	0.1
GAIF01031646.1	0.13	0.13	0.02	0	0	0.05
GAIF01031647.1	0	0	0	0	0.1	0
GAIF01031651.1	7.2	21.79	24.64	30.98	35.63	19.16
GAIF01031652.1	0.16	0.02	0.11	0	0	0.02
GAIF01031653.1	12.55	11.68	4.44	70.51	75.53	2.77
GAIF01031655.1	0	0.12	0.26	0.27	0.13	0
GAIF01031656.1	0.03	0	0	0	0	0
GAIF01031658.1	0.52	2.04	1.74	2.62	1.9	1.17
GAIF01031660.1	1.85	0	0	0	2.49	6.95
GAIF01031661.1	0.29	0	0	0.19	0.36	0
GAIF01031663.1	15.65	13.12	6.61	95.35	62.1	7.61
GAIF01031664.1	4.8	3.6	4.45	0.8	0.38	0.14
GAIF01031665.1	0	0.31	0	0.06	0.06	0
GAIF01031668.1	1.51	0.82	0.09	3.46	10.55	0.28
GAIF01031669.1	7.66	7.91	5.75	41.87	63.35	4.37
GAIF01031671.1	0.61	0.36	0.25	7.43	22.08	0.98
GAIF01031672.1	4.38	9.94	3.77	11.81	15.03	0.24
GAIF01031673.1	0	0	0	0	0.08	0
GAIF01031674.1	12.01	3.82	9.42	8.14	12.56	22.93
GAIF01031675.1	0.83	0	0	0.6	9.08	0.1
GAIF01031676.1	0.57	0.05	0	0.77	2	0
GAIF01031677.1	172.69	46.75	40.23	19.64	16.16	9.24
GAIF01031678.1	0.45	0.13	0	1.13	0.98	0
GAIF01031679.1	3.88	1.36	0	2.29	8.26	2.11
GAIF01031680.1	0.13	0.14	0	1.9	7.58	0.2
GAIF01031681.1	1140.81	280.78	369.63	794.39	1482.31	3320.1
GAIF01031682.1	0.34	0.19	0.09	1.18	2.73	0
GAIF01031683.1	0.96	0	0	0.1	0	0
GAIF01031684.1	0	0	0	0.15	0.03	0.05
GAIF01031686.1	0	0	0	0	1.08	0
GAIF01031688.1	7.74	0.47	0.05	0.93	3.56	0.33
GAIF01031689.1	38.3	31.93	53.19	31.1	36.47	27.97
GAIF01031690.1	0	0	0	0.23	0.57	0
GAIF01031691.1	0	0	0	0	0.3	0
GAIF01031692.1	0.11	0.24	0	0	0	0
GAIF01031693.1	0.12	0.04	0	0.95	4.61	0

GAIF01031695.1	0.65	0.2	0.1	4.82	14.96	0.57
GAIF01031696.1	0	0	0	0.26	0	0
GAIF01031697.1	1.17	1.01	0.57	4.22	5.62	0.3
GAIF01031699.1	9.07	4.04	0.68	17.76	72.5	9.48
GAIF01031700.1	4.12	2.17	1.73	3.01	1.78	7.4
GAIF01031701.1	20.04	7.92	9.98	22.35	37.45	38.36
GAIF01031705.1	11.13	3.27	2.88	0	0	0
GAIF01031707.1	5.3	2.88	5.9	5.98	3.63	2.51
GAIF01031708.1	0.02	0	0	0.28	1	0.05
GAIF01031709.1	1.27	1.9	0.52	3.68	2.1	1.57
GAIF01031710.1	0	0	0	0	0.14	0
GAIF01031711.1	117.35	103.43	114	128.97	54.03	36.42
GAIF01031712.1	0.34	0.11	0	0	0	0
GAIF01031713.1	0.26	1.68	0.99	2.24	1.86	0.14
GAIF01031715.1	1.33	1.13	1.96	3.2	5.74	0.07
GAIF01031716.1	185.75	176.95	101.76	110.19	101.38	117.02
GAIF01031717.1	10.04	15.3	12.27	11.4	6.5	1.66
GAIF01031718.1	0.11	0.78	0	0.13	0.54	0.09
GAIF01031722.1	0	0	0	0	0.15	0.04
GAIF01031725.1	3.78	2.06	6.11	6.09	2.15	14.81
GAIF01031728.1	0	0	0	2.76	15.23	0
GAIF01031729.1	30.29	29.76	27.84	36.98	25.9	16.81
GAIF01031731.1	23.4	5.49	1.92	19.87	30.67	37.74
GAIF01031732.1	2.65	1.39	1.87	6.3	4.67	2.51
GAIF01031733.1	39.47	19.01	13.05	42.72	50.04	22.14
GAIF01031734.1	0.23	0.5	0	0.26	1.35	0.38
GAIF01031736.1	3.62	2.89	1.4	35.14	30.7	1.28
GAIF01031738.1	0.09	0	0	0.42	0.43	0.39
GAIF01031741.1	0	0	0	0	0.05	0
GAIF01031743.1	0	0.06	0	0.73	1.26	0
GAIF01031745.1	5.02	1.99	3.89	17.42	3.37	9.96
GAIF01031746.1	0.08	0.09	0	0.48	1.24	0
GAIF01031747.1	0	0.11	0	0.58	1.79	0
GAIF01031748.1	1.19	0	0	3.46	7.88	0.66
GAIF01031749.1	0.13	0.26	0.19	0.45	0.08	0.25
GAIF01031750.1	0.1	0	0	0	0	0
GAIF01031751.1	0	0.14	0	0	0.7	0
GAIF01031752.1	0	0	0.21	0	0	0
GAIF01031753.1	0.03	0	0	0.12	0.53	0.03
GAIF01031755.1	0.83	0.2	0.2	3.31	9.62	0
GAIF01031756.1	0.2	0.05	0.08	4.79	15.12	0.8
GAIF01031757.1	0	0	0	0.2	0.12	0
GAIF01031760.1	6.22	1.07	10.53	0	15.46	0
GAIF01031761.1	5.82	0.76	0.51	0.54	3.22	6.61

GAIF01031762.1	0.97	0.35	0.18	0.38	0	0
GAIF01031763.1	1.03	0.64	0	3.06	6.53	0.16
GAIF01031764.1	0	0	0	0.3	1.43	0
GAIF01031766.1	0.06	0	0.13	0.07	0.21	0
GAIF01031767.1	1.89	2.53	0.49	5.02	6.67	0.12
GAIF01031768.1	0.05	0.06	0	0.19	0.91	0.06
GAIF01031769.1	17.7	16.87	9.02	35.47	52.36	12.77
GAIF01031770.1	8.77	5.15	9.24	35.49	19.39	13.32
GAIF01031771.1	0	0	0	0.06	0.38	0
GAIF01031772.1	0.08	0.28	0.28	0.49	1.12	0
GAIF01031773.1	19.65	19.78	30	57.16	80.01	27.49
GAIF01031774.1	0	16.61	2.3	0	0.18	0.83
GAIF01031775.1	16.66	16.49	10.64	4.05	2.68	0.5
GAIF01031776.1	3.05	3.3	8.5	4.71	0.13	4.47
GAIF01031777.1	0	0	0	0	0.03	0
GAIF01031779.1	0	0	0.12	0	0	0
GAIF01031782.1	0	5.98	3.18	0	0	0.8
GAIF01031783.1	0	0.1	0.05	0	0.11	0
GAIF01031784.1	1.92	2.51	2.66	1.07	0.8	0.86
GAIF01031785.1	0	0	0.99	0	0.51	1.33
GAIF01031787.1	2.6	0.47	0	21.23	111.57	1.31
GAIF01031788.1	0.15	0	0	1.06	7.3	0.5
GAIF01031790.1	12.89	22.53	16.3	22.91	14.52	7.37
GAIF01031792.1	365.33	438.15	635.27	580.85	408.36	278.88
GAIF01031793.1	16.43	31.57	16.38	19.26	13.43	2.02
GAIF01031794.1	3.82	6.47	10.42	5.02	3.67	5.54
GAIF01031795.1	76.77	144.9	136.35	179.38	183.32	40.13
GAIF01031799.1	1	0.72	0.35	1.89	2.2	0.26
GAIF01031800.1	2.53	1.61	0.36	6.48	16.46	5.33
GAIF01031801.1	0	0.29	0.2	0.35	1.82	0
GAIF01031802.1	0.15	0	0	0	0	0
GAIF01031803.1	0.03	0	0.09	0.03	0.03	0
GAIF01031805.1	16.83	11.7	20.71	38.06	22.14	17.08
GAIF01031807.1	0	0.91	2.62	0	0	0.17
GAIF01031808.1	0	0.17	0	0	0.19	0
GAIF01031809.1	3.08	0	10.5	15.04	0	3.78
GAIF01031810.1	0	0.28	0	0.24	0.08	0.07
GAIF01031812.1	1.23	1.47	1.77	2.1	2.39	0.49
GAIF01031813.1	0.48	0	0.17	0.74	3.05	0
GAIF01031814.1	27.78	11.04	4.44	50.55	66.26	21.95
GAIF01031815.1	0.24	0.13	0	1.1	2.68	0
GAIF01031817.1	0.8	0	0	5.15	25.87	0
GAIF01031818.1	56.91	53.43	28.8	69.74	72.95	9.04
GAIF01031819.1	50.37	56.69	17.45	80.44	73.11	29.92

GAIF01031821.1	0.55	0.6	0.53	4.63	9.43	0
GAIF01031822.1	8.68	13.37	9.87	22.03	13.59	3.88
GAIF01031823.1	281.29	34.16	75.97	3.88	2.13	7.94
GAIF01031824.1	0.42	0.09	0	1.28	4.58	0.75
GAIF01031825.1	0.28	0	0	0	0	0
GAIF01031826.1	0.56	0	0	0	0	0
GAIF01031827.1	15.54	16.24	0.5	62.7	76.62	2.78
GAIF01031828.1	1	2.1	0.83	2.48	5.74	0.2
GAIF01031830.1	0.79	0	0	0.55	5.5	0.35
GAIF01031832.1	9.79	8.45	9.94	1.49	0.33	0.3
GAIF01031834.1	0	0	0	1.4	16.34	0
GAIF01031836.1	0	0	0	0.44	1.83	0
GAIF01031837.1	0	0	0	0.28	0.29	0
GAIF01031838.1	6.89	1.38	3.05	2.39	0.29	0.07
GAIF01031839.1	1.1	1.2	1.45	0.85	1.59	0.41
GAIF01031842.1	34.43	22.28	30.73	52.42	40.71	97.45
GAIF01031844.1	168.16	56.29	30.69	299.09	232.64	38.52
GAIF01031845.1	0.27	1.51	0.83	2.75	6.16	1.21
GAIF01031847.1	0.13	0.43	0	0.61	0.63	0.14
GAIF01031849.1	4.89	0	1.03	12.95	6.53	7.15
GAIF01031850.1	0.41	0.05	0	0.43	1.27	0
GAIF01031852.1	0	0	0	0	0.21	0
GAIF01031853.1	0	1.02	0.12	0	0.07	0.06
GAIF01031854.1	13.78	21.74	12.01	45.3	21.13	3.13
GAIF01031855.1	0	0.7	0.18	0	3.68	0
GAIF01031856.1	0.25	0.07	0	1.72	7.7	0.41
GAIF01031858.1	0.06	0	0	0.2	0	0.06
GAIF01031859.1	0.85	0	0.48	1.03	1.66	0
GAIF01031860.1	0	0	0	0.16	0	0
GAIF01031861.1	0	0	0	0.2	0	0
GAIF01031862.1	9.17	8.3	0.57	40.91	57.47	0.1
GAIF01031863.1	3.16	3.5	6.81	0	0	0
GAIF01031864.1	6.71	0.31	0	20.71	51.17	2.08
GAIF01031868.1	52.13	82	132.74	27.6	28.06	61.12
GAIF01031869.1	0.08	0	0	0	0	0
GAIF01031872.1	0.68	0	0	0	0	0
GAIF01031873.1	0	0.05	0	0.05	0	0
GAIF01031874.1	0	0	0	0.09	0.46	0
GAIF01031875.1	0	0	0	1.64	4.16	0.17
GAIF01031876.1	2.46	10.17	9.37	4.05	4.55	2
GAIF01031878.1	0	1.09	0	0	0	0
GAIF01031880.1	0	0	0	0.41	0.21	0
GAIF01031882.1	0.32	0.06	0.18	99.4	4.85	25.83
GAIF01031883.1	0	0	0.22	0	0.74	0

GAIF01031888.1	10.2	7.26	14.78	27.38	23.91	12.58
GAIF01031889.1	0.55	0.07	0	0.35	0.22	0.4
GAIF01031893.1	8.56	6.96	7.62	10.07	3.25	1.39
GAIF01031894.1	0.12	0.13	0	0.28	1.02	0.33
GAIF01031895.1	61.49	81.49	53.74	84.38	50.93	17.3
GAIF01031896.1	0.15	0.08	0	0.09	0.72	0
GAIF01031898.1	0	0	0	0.02	0.08	0
GAIF01031899.1	71.7	102.43	102.88	95.46	46.39	44.52
GAIF01031901.1	0	0	0	0	0.15	0
GAIF01031902.1	11.01	3.98	6.33	30.47	52.26	15.91
GAIF01031903.1	28.02	51.72	48.83	61.41	45.48	13.32
GAIF01031904.1	0	0	0	2.61	1.24	0
GAIF01031906.1	466.71	2818.8	1285.23	148.86	264.34	82.49
GAIF01031908.1	41.83	58.43	47.37	36.3	33.64	2.87
GAIF01031909.1	0.84	0.09	0.09	0.39	0.1	0
GAIF01031911.1	2.07	3.26	2.36	9.66	6.27	0.77
GAIF01031912.1	0.05	0	0	0.05	0.34	0.05
GAIF01031913.1	17.61	24.49	18.89	35.93	24.66	2.51
GAIF01031915.1	11.13	10.55	14.73	22.86	13.46	12.71
GAIF01031916.1	0	0	0	2.5	5.65	1.1
GAIF01031917.1	0	0	0	0.05	0	0
GAIF01031920.1	15.03	21.78	31.2	20.01	10.19	12.63
GAIF01031921.1	5.26	5.06	4.19	0.74	0	1.33
GAIF01031923.1	0.21	0.2	0	2.29	11.72	0
GAIF01031924.1	0	0	0.05	0.06	0.06	0
GAIF01031925.1	0	0.21	0	0	0	0
GAIF01031926.1	0	0	0	0	0.14	0
GAIF01031927.1	0.27	0.3	0.04	1.86	3.54	0
GAIF01031929.1	91.97	50.97	32.23	93.65	29.95	14.04
GAIF01031930.1	0.16	2.05	0.41	2.86	9.17	0.77
GAIF01031931.1	40.86	30.21	25.9	18.34	7.12	12.08
GAIF01031933.1	0.11	0	0	2.06	7.88	0
GAIF01031934.1	0	0.06	0	0.07	0.32	0
GAIF01031936.1	95.46	70.48	38.95	18.24	26.87	3.84
GAIF01031937.1	1.5	1.98	0.41	3.28	2.83	0.82
GAIF01031939.1	0.48	0.1	0.11	4.46	17.89	1.27
GAIF01031941.1	1.81	1.36	0.15	13.41	37.08	0.84
GAIF01031942.1	0	0.19	0	0	0	0
GAIF01031944.1	0.26	0.75	0.15	0.89	0.73	0
GAIF01031945.1	0	0	0	0	0.78	0.19
GAIF01031947.1	0.53	0	0.32	0	0	0.52
GAIF01031948.1	93.6	8.1	5.69	13.89	40.77	45.42
GAIF01031949.1	0.13	0.14	0	0.29	2.5	0.07
GAIF01031950.1	0.04	0.05	0	0.81	2.5	0.29

GAIF01031951.1	0.04	0.04	0	0.14	0.9	0.13
GAIF01031952.1	432.39	123.22	146.46	197.88	183.05	167.33
GAIF01031953.1	11.61	4.24	5.92	10.18	10.58	12.63
GAIF01031955.1	13.36	21.7	9.72	36.75	28.33	2.32
GAIF01031956.1	0	0	0.22	0.24	0	0
GAIF01031957.1	0	1.67	3.97	0	0	0.52
GAIF01031958.1	0.61	0.18	0.05	2.42	12.7	4.75
GAIF01031960.1	0	0	0.32	0	0	0
GAIF01031961.1	0	0.11	0	0	0	0
GAIF01031962.1	91.91	100.63	84.32	40.78	41.55	15.15
GAIF01031963.1	0	0	0	0	0.28	0
GAIF01031964.1	0	0	0	0	0.01	0
GAIF01031965.1	15.2	35.82	11.11	67.2	64.11	3.43
GAIF01031966.1	0	0.1	0.05	0.6	0.23	0
GAIF01031967.1	0.21	0.58	0.7	0.25	0.13	0.35
GAIF01031969.1	0	0.2	0	0.43	0.22	0
GAIF01031970.1	31.88	29.58	18.32	112	64.16	15.69
GAIF01031972.1	318.85	347.15	131.12	30.83	82.87	25.67
GAIF01031973.1	0.11	0	0	11.38	0	0
GAIF01031976.1	0.45	0.1	0	1.23	2.56	0
GAIF01031977.1	2.86	5.02	4.54	4.47	3.55	0.57
GAIF01031979.1	0	0	0	0.37	0.49	0
GAIF01031980.1	0.1	0	0.4	0.11	0.31	0
GAIF01031981.1	0	0	0	0.2	0	0
GAIF01031982.1	0	0.14	0	0.59	2.77	0
GAIF01031984.1	0.9	0.98	1.78	0.42	0.65	1.39
GAIF01031987.1	0	0	0.14	0	0	0
GAIF01031988.1	0	0.22	0	0	0.12	0
GAIF01031989.1	0	0	0	0	0.21	0.03
GAIF01031990.1	0	0.32	0.13	0	0	0.46
GAIF01031992.1	0.13	0	0	0	0	0.07
GAIF01031993.1	60.5	62.09	49.9	83.35	48.17	72.63
GAIF01031994.1	259.32	93.92	92.98	142.91	125.96	274.11
GAIF01031995.1	1.01	3.19	5.26	0	2.3	0
GAIF01031996.1	0.28	0	0	0	0	0.08
GAIF01031997.1	0.09	0	0	0.96	3.27	0
GAIF01031999.1	158.35	158.86	361.07	89.25	50.21	160.23
GAIF01032000.1	0.43	0.16	0	2.15	6.69	7.71
GAIF01032001.1	0	0.15	0	0	0.12	0
GAIF01032002.1	34.1	64.39	17.81	180.51	94.58	4.9
GAIF01032005.1	161.27	231.66	249.44	97.37	65.82	21.97
GAIF01032007.1	15.67	18.73	7.35	46.23	29.77	2.9
GAIF01032008.1	1270.14	327.78	139.6	335.95	337.43	80.75
GAIF01032010.1	0	0.09	0	0.13	0.04	0.04

GAIF01032011.1	43.7	33.49	40.9	47.51	22.52	20.75
GAIF01032013.1	0	0	0	0.16	0	0
GAIF01032014.1	0	0	0	0.31	1.26	0
GAIF01032016.1	0.56	0.12	0	1.17	6.94	0.12
GAIF01032017.1	0.02	0	0	0.06	0.02	0
GAIF01032018.1	0	0	0	0	0	0.25
GAIF01032020.1	6.85	4.8	4.18	2.83	0.73	0
GAIF01032021.1	0	0	0	0.14	0.14	0
GAIF01032022.1	13.44	15.96	5.23	42.58	36.06	3.1
GAIF01032024.1	0.06	0	0	0	0.1	0
GAIF01032025.1	5906.96	6762.02	16125.26	3529.25	2495.76	17948.05
GAIF01032026.1	0	0	0.46	0.96	1	0.26
GAIF01032027.1	0	0.06	0	0	0	0
GAIF01032028.1	7.02	5.05	7.18	19.67	21.26	13.11
GAIF01032029.1	4.62	0.52	0	5.62	8.59	2.13
GAIF01032030.1	8.14	10.98	10.16	15.75	8.77	6.91
GAIF01032031.1	11.73	5.76	0	6.21	3.14	0
GAIF01032033.1	1.09	3.42	0	1.71	0	0
GAIF01032036.1	153.59	295.82	214.79	124.56	158.64	128.85
GAIF01032037.1	0.74	0	0	0.49	0.76	0
GAIF01032039.1	90.07	57.8	38.96	61.05	39.98	20.7
GAIF01032041.1	1.11	0.6	0.12	1.03	2.01	0.12
GAIF01032043.1	0.34	0.09	0	1.17	6.02	0.32
GAIF01032044.1	0	0.07	0.15	5.56	25.18	0.08
GAIF01032045.1	5	0.16	0	9.05	22.47	0.16
GAIF01032046.1	26.34	30.57	65.86	23.48	18.12	83.67
GAIF01032047.1	16.85	18.21	17.5	60.83	76.95	7.83
GAIF01032048.1	0.79	1.45	0.09	0.73	2.55	0
GAIF01032049.1	0.8	0.7	0.49	2.02	20.17	0.39
GAIF01032050.1	0	0	0	0.11	0	0
GAIF01032053.1	72.62	38.53	15.01	17.76	18.09	7.48
GAIF01032055.1	0.86	1.59	2.33	5.09	5.99	1.17
GAIF01032056.1	0.28	0	0.31	1.29	4.18	0.46
GAIF01032057.1	13.94	0	0.01	4.09	0	0
GAIF01032060.1	0	0	0	0.05	0	0
GAIF01032061.1	2.44	4.2	1.99	7.59	8.06	0.26
GAIF01032062.1	0	0	0	0.04	0.04	0
GAIF01032063.1	0	0	0	0.2	0	0.34
GAIF01032064.1	328.58	408.37	191.72	963.57	912.97	65.36
GAIF01032065.1	0	0	0	0	0.26	0.01
GAIF01032066.1	0	0	0.1	0	0	0
GAIF01032068.1	97.35	70.03	62.1	0.23	0.24	0.11
GAIF01032069.1	0.48	0.39	0.27	0	0	0
GAIF01032070.1	1.41	0.76	0	2.24	3.38	0

GAIF01032071.1	0.35	1.31	0.47	1.5	6.02	0.1
GAIF01032072.1	0.6	0.12	0.03	0.33	8.33	0
GAIF01032073.1	0.2	0.11	0.34	6.89	0	0
GAIF01032075.1	93.71	89.6	139.55	140.63	123.21	131.33
GAIF01032076.1	0.3	0.27	0.15	0.43	1.54	0.07
GAIF01032081.1	2.56	1.82	1.83	1.87	1.18	0.7
GAIF01032082.1	0.46	0.22	0	2.63	4.58	0
GAIF01032083.1	5.69	8.29	6.72	12.04	9.34	1.71
GAIF01032084.1	2.26	0.62	0.31	8.05	17.7	5.62
GAIF01032085.1	12.04	17.97	6.23	14.68	12.02	1.98
GAIF01032086.1	0.2	0.16	0	0.68	2.18	0
GAIF01032087.1	1.84	3.25	2.93	9.37	6.06	3.66
GAIF01032089.1	0	0	0	0	0.05	0
GAIF01032091.1	0.16	0.17	0.09	0	0	0.71
GAIF01032093.1	0	0	0.1	0	0	0
GAIF01032096.1	0	0	0	0	0	0.07
GAIF01032097.1	9.1	12.4	20.22	15.25	12.5	0.01
GAIF01032098.1	0.4	0	0.18	0.39	2.25	0
GAIF01032100.1	33.35	35.65	39.11	74.8	46.11	28.68
GAIF01032101.1	21.08	28.92	8.69	71.97	77.58	5.47
GAIF01032103.1	0.15	0.43	0.27	0.23	1.5	0.28
GAIF01032104.1	0.16	0	0	0.47	1.56	0
GAIF01032105.1	0.31	0.71	0	1.14	12.03	0
GAIF01032106.1	0.05	0.05	0	0.2	0.23	0.03
GAIF01032107.1	0	0	0	0	0.11	0
GAIF01032108.1	3.98	0.94	1.18	6.62	17.45	7.8
GAIF01032109.1	0.27	0.42	0.48	0.89	4.41	0.12
GAIF01032110.1	0	0.54	1.64	0	0.15	0.14
GAIF01032111.1	1.4	1.27	0	0.85	2.29	0.6
GAIF01032112.1	0	0.22	0.05	0.05	0	0.04
GAIF01032113.1	0	0.29	0	0	0	0.3
GAIF01032114.1	0	0	0	0	0.35	0
GAIF01032116.1	1.38	0	0.17	4.88	21.08	2.3
GAIF01032118.1	0	0	0.21	0	0.24	0
GAIF01032119.1	13.16	26.28	11.99	33.55	28.07	3.72
GAIF01032120.1	0.2	0.21	0.22	0	0.23	0
GAIF01032121.1	29.86	12.85	3.74	11.55	20.5	1.75
GAIF01032122.1	0	0	0	0.43	0	0.13
GAIF01032124.1	1.37	3.12	1.58	0.63	1.07	0.6
GAIF01032125.1	0.01	0	0	0.02	0.18	0
GAIF01032126.1	56.59	28.08	11.27	196.32	115.71	18.56
GAIF01032127.1	632.86	504.28	928.38	49.87	21.07	69.02
GAIF01032128.1	0	0	0	0	0.06	0
GAIF01032130.1	0	0.67	0.68	0	0	0.68

GAIF01032131.1	0.56	0.68	0	0	0	0
GAIF01032132.1	1.69	2.41	1.96	2.31	5.67	0.43
GAIF01032133.1	0	0	0	0.3	1.86	0
GAIF01032134.1	12.57	12.21	12.38	25.58	30.27	3.96
GAIF01032136.1	0	0	0	0.08	0.16	0
GAIF01032137.1	0.27	0.67	0.83	0.16	0	0.23
GAIF01032138.1	0.09	0.06	0.1	0.31	0.67	0.06
GAIF01032139.1	26.84	35.31	29.35	24.43	39.92	8.58
GAIF01032140.1	13.44	18.3	12.29	68.45	41.24	4.95
GAIF01032141.1	5.14	9.01	9	28.7	17.38	2.14
GAIF01032143.1	0	0	0.02	0.01	0	0.05
GAIF01032145.1	5.54	4.85	4.59	12.86	9.22	2.67
GAIF01032146.1	127.23	55.84	30.84	264.15	290.49	57.86
GAIF01032147.1	1.6	0.31	0.27	3.8	17.08	6.18
GAIF01032148.1	0.53	0.33	0.25	0.09	0.18	0
GAIF01032149.1	2655.64	2970.15	3277.03	814.45	306.94	171.46
GAIF01032151.1	0	0.49	0	0	1.5	0
GAIF01032153.1	0.03	0.09	0.06	0.2	1.69	0.09
GAIF01032154.1	29.05	41.54	8.7	6.42	6.86	0.89
GAIF01032155.1	418.49	219.84	290.52	49	48.42	69.32
GAIF01032156.1	0.06	0.09	0.01	1.87	8.2	0.11
GAIF01032157.1	253.63	199.29	115.8	0.1	0.6	0
GAIF01032158.1	0.07	0	0	0.42	1.23	0.08
GAIF01032159.1	0.38	0.42	0.24	1.33	2.11	0
GAIF01032160.1	0	0.08	0	0	0.61	0
GAIF01032161.1	0.23	0.33	0.25	0.51	0.17	0.23
GAIF01032162.1	33.06	46.84	33.23	56.21	42.14	9.24
GAIF01032163.1	11.46	0	0	38.87	31.18	60.28
GAIF01032164.1	23.99	15.07	13.17	34.63	42.73	26.3
GAIF01032165.1	0	0	0	0.1	0.05	0.05
GAIF01032169.1	14.84	3.5	7.54	13.9	25.49	30.7
GAIF01032170.1	0.24	0	0	0.47	1.94	0
GAIF01032171.1	0.41	0.13	0.08	2.16	8.3	0.84
GAIF01032174.1	0	0	0.13	0	0	0.26
GAIF01032176.1	0	0	0.08	0	0	0
GAIF01032177.1	3.53	7.79	8.03	0.42	0.31	0.17
GAIF01032179.1	0.54	0.59	0.15	7.7	36.3	0.45
GAIF01032180.1	76.43	50.74	20.68	118.77	75.76	5.86
GAIF01032182.1	0.76	0.2	0.06	1.02	2.46	0.94
GAIF01032183.1	0.15	0.24	0	1.11	1.59	0.73
GAIF01032184.1	0	0.1	0	0.31	1.41	0
GAIF01032187.1	0.2	0.34	0	0.92	0.69	0.2
GAIF01032188.1	0.89	0	0.23	2.23	14.03	12.1
GAIF01032189.1	0	0.02	0.04	0.09	0.33	0.01

GAIF01032193.1	1.61	3.84	1.67	3.63	5.79	1.06
GAIF01032194.1	0.13	0.07	0.07	0.23	1.42	0.13
GAIF01032195.1	63.31	83.91	53.49	211.12	168.65	26.05
GAIF01032196.1	76.24	199.14	17.5	32.39	143.76	22.97
GAIF01032198.1	160.01	103.39	185.94	90.63	72.48	85.21
GAIF01032200.1	0	0	0	0.21	1.17	0.13
GAIF01032202.1	0	0	0	0	0.59	0
GAIF01032203.1	0.21	0.18	0.05	2.6	4.83	0.5
GAIF01032204.1	0.77	0.52	0.33	2.42	7.66	0.79
GAIF01032206.1	0	0	0	0	0.04	0
GAIF01032207.1	0	0	0	0.09	0.6	0
GAIF01032208.1	1.28	0.13	0	2.97	8.24	0.9
GAIF01032209.1	0	0	0	0.11	0	0
GAIF01032210.1	0.99	1.08	0.62	1.18	1.74	0
GAIF01032211.1	0	0.02	0	0.02	0.18	0
GAIF01032213.1	6.02	9.35	17.5	13.91	4.12	7.58
GAIF01032215.1	0.9	0.9	0.25	3.85	6.8	0
GAIF01032216.1	132.85	38.02	38.38	232.6	226.2	45.34
GAIF01032219.1	0.63	1.38	0.23	7.31	4.88	0.33
GAIF01032221.1	4.4	13.3	1.82	23.02	91.25	0.71
GAIF01032223.1	18.74	12.07	14.35	19.42	11.17	27.93
GAIF01032224.1	91.27	66.54	62.77	117.85	101.44	73.43
GAIF01032225.1	0.42	0.13	0.07	2.58	9.61	2.05
GAIF01032226.1	49.23	49.11	51.28	40.98	63.72	27.15
GAIF01032228.1	0	0.14	0.03	1.26	7.45	1.61
GAIF01032231.1	0.01	0.33	0	0.06	0	0.11
GAIF01032232.1	0	0	0	0	0.11	0
GAIF01032233.1	0	0	0	0	0.48	0
GAIF01032234.1	122.38	38.02	166.21	13.41	13.02	19.27
GAIF01032235.1	394.24	374.98	545.84	249.03	182.28	834.48
GAIF01032236.1	12.91	24.82	21.81	32.04	28.24	8.54
GAIF01032238.1	0.7	0	0.05	1.41	3.95	5.76
GAIF01032240.1	0	0	0	0	0.04	0
GAIF01032241.1	1.03	1.91	0.4	2.04	1.53	0.47
GAIF01032244.1	3.88	0.55	1.33	0	0	0
GAIF01032245.1	1.52	0	0.65	0	0	0
GAIF01032247.1	0	0.32	0.21	0.22	0.47	0
GAIF01032248.1	51.88	24.6	24.94	75.45	75.44	73.75
GAIF01032249.1	0.12	0.07	0	0.07	0.44	0
GAIF01032250.1	0	0	0	0	0	0.42
GAIF01032251.1	3.33	2.29	0.5	1.53	1.81	0.58
GAIF01032252.1	134.47	81.26	55.64	135.24	180.92	119.94
GAIF01032253.1	23.44	24.5	22.09	31.88	36.3	42.52
GAIF01032254.1	0.08	0.04	0	0.14	0.45	0

GAIF01032255.1	20.64	19.27	23.5	54.08	37.53	18.15
GAIF01032256.1	4.17	1.6	1.02	15.42	46.99	3.42
GAIF01032257.1	7.75	6.76	2.09	16.03	30.59	3.14
GAIF01032258.1	0	0	0	0	0.07	0
GAIF01032259.1	0	0	0	0.35	1.84	0
GAIF01032260.1	0	0	0	15.22	0	0
GAIF01032261.1	0.59	0.3	0.28	0.38	4.16	0.05
GAIF01032263.1	0.09	0.2	0	1.67	6.59	0.1
GAIF01032264.1	0.47	0	0	3.25	6.13	0.17
GAIF01032265.1	3.75	2.67	1.51	9.51	26.87	2.05
GAIF01032266.1	0	0	0	0	0.12	0
GAIF01032267.1	0.72	0.81	0.36	1.37	3.92	0.15
GAIF01032268.1	0.17	0	0.12	0	0.14	0
GAIF01032269.1	4.74	3.14	2.99	2.49	0.84	0.41
GAIF01032271.1	3.08	0.51	0.11	0	1.21	0
GAIF01032272.1	69.29	116.43	125.53	103.57	70.39	34.61
GAIF01032273.1	0.04	0	0	0	0	0
GAIF01032274.1	28.43	34.96	29.27	66.22	28.06	14.05
GAIF01032277.1	0	0.93	1.1	0	0	0.06
GAIF01032279.1	11.68	61.92	14.8	2.88	4.71	0.21
GAIF01032280.1	12.79	7.85	4.56	45.88	36.66	1.15
GAIF01032281.1	0.56	0.11	0	2.42	7.21	2.3
GAIF01032282.1	2.15	4.47	0	9.83	11.97	0
GAIF01032284.1	0	0.94	0.44	0	0	0
GAIF01032286.1	22.2	30.28	13.66	91.25	53.95	5.81
GAIF01032289.1	0	0	0	0	0.07	0
GAIF01032290.1	0.82	0.3	0.78	0.38	0.07	0.48
GAIF01032292.1	0	0	0	0	0.04	0
GAIF01032293.1	0	0	0	0	0.04	0
GAIF01032295.1	1.6	0.5	0.63	1.06	0.55	0.25
GAIF01032298.1	0.24	0	0	0.62	3.37	0.27
GAIF01032299.1	3.07	2.25	5.13	15.97	8.88	2.64
GAIF01032300.1	26.73	23.92	24.73	27.17	22.79	48.91
GAIF01032301.1	0	0.59	0.61	0	0.66	0
GAIF01032303.1	0.65	6.04	4.09	3.45	5.75	2.85
GAIF01032304.1	11.77	9.37	9.26	22.13	21.68	12.24
GAIF01032305.1	12.64	16.12	3.47	0	0.15	0
GAIF01032307.1	0.6	0.52	0	0.42	0.14	0.13
GAIF01032308.1	0	0	0	0.03	0	0
GAIF01032309.1	1.99	3.99	2.39	2.31	4.59	1.14
GAIF01032313.1	1.3	0.88	0.85	3.07	4.79	0.72
GAIF01032316.1	33.98	36.03	35.22	105.54	96.18	17.07
GAIF01032317.1	4.49	2.38	4.68	3.34	0.42	2.41
GAIF01032318.1	7.47	19.24	13.13	5.42	3.58	0.39

GAIF01032319.1	5.67	1.77	0.34	4.49	6	0.15
GAIF01032320.1	0	0	0.06	0.19	1.34	0
GAIF01032321.1	0.11	0	0	0	0.13	0.12
GAIF01032322.1	0	0	0	0.28	0	0
GAIF01032324.1	3.6	6.8	2.64	18.65	22.93	33.69
GAIF01032325.1	0.16	0	0	0.38	3.31	0
GAIF01032326.1	0.68	0.58	1.5	1.36	1.11	7.99
GAIF01032328.1	0	0	0	0	0.29	0
GAIF01032331.1	5.4	6.36	7.33	20.88	11.54	2.73
GAIF01032334.1	3.27	1.14	0.9	16.15	34.17	0.77
GAIF01032335.1	0	0	0	0	0.18	0
GAIF01032337.1	4.08	4.54	3.21	3.62	1.91	1.8
GAIF01032339.1	0.18	0	0	1.7	6.6	0
GAIF01032340.1	4.39	3.45	1.77	4.82	3.39	1.03
GAIF01032341.1	0	0	0	0	0.31	0
GAIF01032342.1	3.02	2.03	0.59	4.46	4.18	0.76
GAIF01032343.1	6.87	4.02	5.39	4.35	3.43	2.61
GAIF01032345.1	0	0	0.14	0	0	0
GAIF01032346.1	0.75	0.87	0.35	6.52	18.79	0.16
GAIF01032347.1	4.46	4.09	4.52	14.02	26.43	22.94
GAIF01032348.1	13.5	6.31	4.3	12.61	18.52	5.03
GAIF01032349.1	63.31	31.46	27.67	52.79	49.55	13.17
GAIF01032350.1	85.88	148.4	148.89	107.53	101.54	150.61
GAIF01032351.1	0	0	0.57	3.78	0	1.93
GAIF01032352.1	0.87	0.48	0.11	3.61	9.42	0.26
GAIF01032353.1	0	0	0	0.11	0.63	0.05
GAIF01032354.1	0	0	0	0	0	0.64
GAIF01032357.1	0	0	0	0.39	0.18	0
GAIF01032359.1	0.07	1.45	0.81	0	0.09	0.25
GAIF01032360.1	7.1	6.85	2.11	7.26	7.43	0.51
GAIF01032361.1	0.01	0	0.05	0.07	0	0.01
GAIF01032362.1	1.61	0.4	0	1.05	0	0.03
GAIF01032363.1	0	1.42	0.15	0	0	0
GAIF01032365.1	262.07	191.04	305.1	109.74	83.43	182.38
GAIF01032366.1	0.57	0	0.63	0.46	0	0
GAIF01032369.1	4.48	3.08	3.83	4.25	4.03	5.04
GAIF01032371.1	10.37	9.34	4.49	16.66	12.1	1.5
GAIF01032372.1	0.13	0	0	0.39	0.24	0.15
GAIF01032373.1	3.3	13.27	8.37	0	0.85	0
GAIF01032374.1	1.57	0.37	0.53	1.58	3.16	0.83
GAIF01032375.1	16.3	5.25	3.45	2.24	0.87	0.4
GAIF01032376.1	0.13	0	0	0.25	1.78	0.06
GAIF01032377.1	0.46	0.04	1.18	0.31	0	0
GAIF01032378.1	1.52	2.46	5.4	4.41	4.12	15.54

GAIF01032379.1	14.62	16.63	17.86	68.31	28.13	12.97
GAIF01032380.1	0	0	0	0.24	0	0
GAIF01032381.1	11.42	17.79	20.74	30.59	17.7	8.96
GAIF01032382.1	0.4	0.22	0.22	1.38	5.12	2.18
GAIF01032383.1	4.45	6.37	2.67	1.13	0.47	0.05
GAIF01032384.1	0.05	4.16	2.32	0	0.06	0.33
GAIF01032385.1	0	0.05	0	0	0.07	0
GAIF01032386.1	0.03	0.07	0.14	0.68	3.96	0.1
GAIF01032387.1	0	0	0	0	0.05	0
GAIF01032388.1	6.21	5.74	7.35	14.89	14.31	3.71
GAIF01032389.1	5.48	19.96	8.49	9.59	10.33	1.77
GAIF01032390.1	0	0	0.15	0.56	0.38	0.15
GAIF01032394.1	0.25	0.27	0.28	0.15	0.5	0.42
GAIF01032396.1	0.01	0.09	0	0.37	2.94	0.01
GAIF01032397.1	0.8	0.05	0	1.02	3.5	1.27
GAIF01032398.1	0.58	0.47	1.5	0.17	0.35	0.16
GAIF01032400.1	0	0	0.41	0	0	0
GAIF01032401.1	1.88	1.46	0	6.84	19.17	5.46
GAIF01032402.1	1.26	0	0	1.05	5.3	1.14
GAIF01032403.1	4.34	5.03	5.92	5.94	8.09	3.01
GAIF01032405.1	0.05	0.34	0.11	0.47	0	0
GAIF01032406.1	0	0.55	0.47	0	0	0.04
GAIF01032407.1	0	0	1.82	0	0	2.98
GAIF01032408.1	42.58	41.91	32.82	59.33	30.24	15.92
GAIF01032410.1	0	0.15	0	0	0.62	0
GAIF01032411.1	1.94	0.5	0.78	0.25	0	0.07
GAIF01032412.1	0.68	0.73	1.07	0.74	1.22	4.94
GAIF01032413.1	51.17	83.62	69.09	160.1	162.45	76.73
GAIF01032415.1	0	0	0.25	0	0	0
GAIF01032417.1	0.05	0.18	0	0	0	0.06
GAIF01032418.1	8.49	7.59	10.44	20.67	17.37	6.7
GAIF01032421.1	0.05	0	0.03	0	0.03	0
GAIF01032423.1	0	0	0	0	0.07	0
GAIF01032424.1	0	0	0.01	0.02	0.01	0
GAIF01032425.1	0.13	0.21	0	1.32	4.31	0.04
GAIF01032427.1	0.17	0	0	0.88	2.34	0
GAIF01032428.1	0.04	0	0	0	0	0
GAIF01032429.1	0	0	0	0	0.06	0
GAIF01032430.1	4.86	6.47	3.79	25.9	17.03	8.08
GAIF01032432.1	10.85	8.16	4.18	23.84	13.46	1.78
GAIF01032433.1	26.85	33.29	15.75	38.84	37.79	6.82
GAIF01032434.1	19.94	22.79	26.26	41.48	37.98	6.92
GAIF01032435.1	0.69	0.75	0.15	3.37	12.63	0
GAIF01032436.1	8.54	24.69	17.35	34.42	39.13	4.77

GAIF01032437.1	0.16	0.09	0	0.45	4.61	0.26
GAIF01032438.1	0.15	0	0	0	0	0.11
GAIF01032439.1	0.47	0.29	0.07	0.08	0	0.07
GAIF01032440.1	23.71	23.32	32.84	38.98	18.89	31.73
GAIF01032441.1	4.2	1.93	0	6.85	15.61	0
GAIF01032442.1	3.11	4.02	1.93	1.22	0.7	0.14
GAIF01032444.1	1.73	1.89	1.9	4.92	7.65	5.31
GAIF01032446.1	0.67	1.27	0.43	3.63	4.77	0.06
GAIF01032447.1	6.01	12.16	6.01	1.59	0.79	0.07
GAIF01032449.1	0	0.05	0	0.15	1.65	0
GAIF01032450.1	0	0	0	0.39	1.54	0
GAIF01032451.1	1.77	0.13	0	4.47	17.62	2.17
GAIF01032452.1	0.5	1.24	0.16	0.33	0	0
GAIF01032453.1	0	0	0	0.84	8.91	1.26
GAIF01032454.1	0.14	0.6	0	0.48	0.33	0.91
GAIF01032455.1	37.95	34.97	44.83	98.76	62.81	35.83
GAIF01032456.1	120.89	134.71	134.98	87.07	97.24	141.62
GAIF01032458.1	184.44	61.37	19.11	255.86	295.09	94.46
GAIF01032460.1	0	0	0	0	0.11	0
GAIF01032461.1	0	0	0	0.14	0.56	0
GAIF01032462.1	0.41	3.22	1.35	0.24	0	0
GAIF01032465.1	0	0.12	0.06	0.4	0.27	0
GAIF01032466.1	25.15	19.49	23.59	33.54	20.22	48.89
GAIF01032468.1	0	0	0	0.14	0	0
GAIF01032470.1	16.21	25.03	25.21	31.48	16.76	14.53
GAIF01032472.1	0.57	0.12	0	0.95	6.33	0.46
GAIF01032473.1	0.13	0.07	0	0.74	5.19	0
GAIF01032474.1	5.46	1.72	0.3	67.64	206.77	3.93
GAIF01032475.1	0	0.06	0	0	0	0
GAIF01032476.1	13.82	11.55	9.49	28.12	51.03	16.02
GAIF01032477.1	1.24	1.9	0.34	4.66	8.67	0
GAIF01032480.1	6.21	5.26	5.62	4.04	2.71	2.64
GAIF01032481.1	0	0	0	0.15	0.4	0.07
GAIF01032482.1	5.45	2.79	1.78	32.21	49.12	2.94
GAIF01032484.1	0	0.09	0	0.24	1.42	0
GAIF01032485.1	0.84	0.91	0.61	1.37	2.34	0.15
GAIF01032486.1	14.63	0.22	0.45	0.24	0.25	0
GAIF01032488.1	4.36	0	0	0	0	0
GAIF01032493.1	0.15	0.33	0.17	0.78	4.07	0.7
GAIF01032496.1	26.36	24.14	26.26	35.83	22.92	20.15
GAIF01032497.1	0.04	0	0	0	0.03	0
GAIF01032498.1	0.23	0.42	0.17	0.69	0.53	0.14
GAIF01032500.1	0.25	0	0.19	1.32	0.52	0
GAIF01032506.1	0	0.32	0.16	9.13	2.5	4.09

GAIF01032507.1	0	0	0	0.29	3.36	0.14
GAIF01032508.1	0.22	0.09	0.09	1.98	7.67	0.36
GAIF01032509.1	0.04	0	0	0	0	0
GAIF01032510.1	0.53	0	0	0	0.86	2.93
GAIF01032511.1	0	0.6	1.05	0	0	2.54
GAIF01032512.1	16.99	18.53	11.84	37.6	23.02	3.5
GAIF01032513.1	0.63	0	1.85	0	0	0
GAIF01032515.1	10.45	9.01	15.21	25.79	11.45	25.94
GAIF01032516.1	0.07	0.08	0	0.1	0.05	0
GAIF01032518.1	0	0	0	0.13	0	0
GAIF01032519.1	0	0	0.11	0	0	0
GAIF01032520.1	0.06	0.07	0	2.17	9.63	0.07
GAIF01032521.1	34.59	42.91	58.7	97	45.12	35.69
GAIF01032522.1	24.05	17.66	11.95	157.32	77.49	28.13
GAIF01032523.1	18.06	14.85	21.72	2.14	25.5	9.84
GAIF01032524.1	0	0	0	0	0.7	0
GAIF01032525.1	0	0	0.68	0	0.51	0
GAIF01032526.1	12.3	13.03	9.84	33.24	17.6	3.47
GAIF01032527.1	249.59	114.37	325.98	210.21	31.49	135.54
GAIF01032529.1	0	0	0	0.23	0	0
GAIF01032530.1	6.66	14.42	4.57	57.32	66.87	2.19
GAIF01032531.1	23.42	15.45	12.48	48.96	36.47	7.18
GAIF01032532.1	535.1	404.35	354.25	175.19	118.27	264.8
GAIF01032534.1	0.15	0.04	0.2	0.09	0.31	0
GAIF01032536.1	0.69	0	0	0.6	2.5	0.89
GAIF01032537.1	0.02	0.04	0	0.05	0.12	0
GAIF01032538.1	0	0	0	0	0.27	0
GAIF01032539.1	0	6.9	0.81	1.82	12.82	0.64
GAIF01032541.1	0.52	0.14	0	13.99	24.64	9.57
GAIF01032543.1	1.68	3.97	1	3.01	3.35	0.26
GAIF01032544.1	2.17	1.18	1.31	5.28	10.67	0.95
GAIF01032545.1	38.42	27.39	28.04	70.43	78.8	31.71
GAIF01032547.1	0	0	0	0.39	2.6	0
GAIF01032548.1	0	0	0	0.17	0.62	0
GAIF01032550.1	3.83	2.32	2.02	2.53	1.86	0.21
GAIF01032551.1	0.02	0.05	0	0.16	0.87	0
GAIF01032552.1	0.08	0	0.25	0.1	0	0
GAIF01032553.1	0	0	0	0.28	0	0
GAIF01032554.1	10.8	16.92	6.42	50.29	59.82	1.34
GAIF01032555.1	0	0.06	0.06	0.33	0.83	0.19
GAIF01032556.1	8.97	7.96	10.19	41.25	21.34	6.82
GAIF01032557.1	50.68	27.12	24.59	41.12	88.84	375.72
GAIF01032558.1	0	0.06	0	0	0.07	0
GAIF01032560.1	16	6.86	5.8	5.39	3.29	8.01

GAIF01032561.1	19.88	16.68	14.3	16.13	5.57	1.91
GAIF01032562.1	0	0	0	0.02	0.02	0
GAIF01032563.1	2.11	4.73	0.85	8.29	9.87	0
GAIF01032564.1	0.17	0	0	0.12	0.84	0
GAIF01032565.1	0.05	0	0	0.38	1.3	0
GAIF01032567.1	0.57	0.61	0.52	0.55	0.57	0.31
GAIF01032568.1	0.18	0.14	0	0.12	1.41	0.45
GAIF01032572.1	2.94	2.5	3.63	5.17	4.29	0.76
GAIF01032573.1	0	0.57	1.91	0	0	4.21
GAIF01032574.1	10.91	7.91	6.44	6.43	1.39	2.28
GAIF01032575.1	0.12	0	0	0.14	0.79	0
GAIF01032577.1	1.32	3.39	8.82	0.56	1.44	8.72
GAIF01032578.1	0.03	0.07	0	0	0.04	0
GAIF01032579.1	1.66	2.92	2.38	1.78	0.61	0.56
GAIF01032580.1	0.14	0.01	0.01	0.12	0.12	0.15
GAIF01032582.1	0	7.1	12.21	0	0	1.52
GAIF01032584.1	103.49	67.16	129.18	7	2.6	6.87
GAIF01032588.1	0	0	0.14	0	0	0
GAIF01032590.1	0	0	0.04	0	0	0
GAIF01032592.1	81.51	90.59	47.3	98.27	67.19	26.52
GAIF01032593.1	0.08	0.04	0	0.55	1.61	0.09
GAIF01032595.1	0.41	0.06	0.05	0	0.07	0.03
GAIF01032597.1	0	0	0	0	0.13	0
GAIF01032598.1	0.57	0	0	2.66	7.57	0.32
GAIF01032599.1	158.66	130.29	57.37	222.08	162.02	36.48
GAIF01032600.1	0.12	0.13	0	1.44	3.28	0.82
GAIF01032602.1	22.47	12.67	9.65	59.96	58.93	13.2
GAIF01032603.1	0	0	0	0.36	3.21	0
GAIF01032604.1	0.03	0.05	0.07	0.59	3.26	0.12
GAIF01032605.1	4.05	3.49	2.96	2.18	4.05	2.63
GAIF01032606.1	0	0	0	0.08	1.57	0.16
GAIF01032608.1	118.82	143.28	198.84	258.52	117.2	154.95
GAIF01032609.1	1.23	0.54	0.17	9.52	28.05	12.64
GAIF01032610.1	2.73	3.1	0.31	3.56	19.79	7.79
GAIF01032612.1	8.08	3.2	1.29	17.39	23.44	10.8
GAIF01032614.1	0	0	0	0.18	0.18	0
GAIF01032615.1	0.88	0.19	0.13	2.79	4.86	6.52
GAIF01032616.1	0.79	0.51	0.69	0.46	2.57	4.37
GAIF01032617.1	0.04	0	0	0	0.09	0
GAIF01032619.1	0	0.89	0	0	0	0
GAIF01032620.1	0.23	0.13	0.13	0.04	1.06	0
GAIF01032621.1	14.25	19.83	7.7	50.91	37.29	1.19
GAIF01032622.1	0.01	7.19	1.6	0.1	21.59	0.82
GAIF01032625.1	214.83	128.16	151.93	271.25	855.62	533.88

GAIF01032627.1	0.21	0.04	0	0.32	1.62	0.27
GAIF01032628.1	44.6	30.56	28.87	45.18	47.84	60.54
GAIF01032629.1	84.41	16.63	9.31	61.13	100.25	4.19
GAIF01032630.1	0.12	0	0	0	0.64	0
GAIF01032631.1	0.61	0.08	0.58	5.79	31.56	0.5
GAIF01032632.1	0.09	0.2	0	1.1	7.04	0.08
GAIF01032637.1	0	0.17	0.18	0	0	0.09
GAIF01032638.1	22.07	34.64	20.55	69.13	59.78	6.12
GAIF01032639.1	0.22	0	0	2.32	6.41	3.55
GAIF01032640.1	0.04	0	0	0	0	0
GAIF01032641.1	0	0	0	0	0.13	0
GAIF01032642.1	0	0	0	0.24	0.76	0
GAIF01032644.1	0.25	0.03	0.74	0.12	0.07	0.34
GAIF01032645.1	18.24	12.45	5.4	33.2	36.82	7.34
GAIF01032646.1	1.12	0.17	0.18	0.19	0.19	0.53
GAIF01032647.1	0.85	0.12	0	0.79	7.25	0.93
GAIF01032650.1	0	0.21	0	0	0	0
GAIF01032651.1	0.92	0.08	3.72	3.6	1.36	9.17
GAIF01032653.1	114.5	47.33	48.86	190.16	73	8.88
GAIF01032654.1	0	6.92	4.42	0	0	0.72
GAIF01032655.1	25.15	14.06	14.59	38.88	41.32	8.58
GAIF01032656.1	0.22	0.24	0.06	0.51	2.32	0.12
GAIF01032657.1	0	0.11	0	0.82	0.97	0.22
GAIF01032659.1	1.56	2.17	0.41	4.25	7.31	0.47
GAIF01032662.1	12.58	7.47	3	35.82	57.9	1.33
GAIF01032663.1	6.03	4.04	3.46	13.08	16.94	11.45
GAIF01032664.1	0.73	1.14	1.57	1.09	1.17	0.72
GAIF01032668.1	6.84	15.28	9.43	34.4	53.39	18.57
GAIF01032669.1	104.9	97.85	107.72	91.07	54.81	35.84
GAIF01032670.1	0.08	0	0	0.45	2.04	0
GAIF01032671.1	0	0	0	0	0.11	0
GAIF01032672.1	9.14	3.89	0.65	31.76	40.15	4.84
GAIF01032673.1	4.55	4.95	2.44	1.87	0.6	0.12
GAIF01032674.1	0	0	0	0.16	1.02	0
GAIF01032675.1	3.82	8.47	2.64	1.81	3.4	2.03
GAIF01032676.1	0	0.04	0	0	0	0
GAIF01032677.1	0	0	0	1.71	4.52	0.74
GAIF01032678.1	4.16	1.12	4.57	4.48	10.2	2.09
GAIF01032679.1	0.13	0.09	0.18	0.15	0	0.14
GAIF01032680.1	0.05	0	0	0	0.12	0
GAIF01032681.1	0.16	0.32	0	0.06	1.74	0
GAIF01032682.1	0.67	0.44	0.15	1.24	0.97	0
GAIF01032683.1	0.34	0	0	2.04	4.15	0
GAIF01032684.1	0	3.3	4.61	0	0	0.96

GAIF01032686.1	44.31	40.8	31.58	53.79	55.82	8.36
GAIF01032688.1	0	0.1	0	0	0.32	0
GAIF01032690.1	0	0	0	0	0.65	0
GAIF01032691.1	0.88	0.41	0.27	1.35	7.84	1.11
GAIF01032692.1	4.8	16.1	11.19	17.02	17.92	1.9
GAIF01032693.1	116.88	121.54	62.31	470.03	287.14	26.69
GAIF01032694.1	0.45	0.16	0	0.7	2.73	0.17
GAIF01032695.1	0	0	0	0.09	0.19	0
GAIF01032697.1	10.16	8.24	6.43	5.4	0.41	0.95
GAIF01032698.1	6.71	4.24	0.46	27.78	62.25	0
GAIF01032699.1	90.21	185.48	164.49	172.4	111.33	22.23
GAIF01032701.1	1.39	0.15	0.38	0.72	0.33	0
GAIF01032702.1	0.92	0.42	0.39	0.63	4.63	0
GAIF01032704.1	0	0	0	1.36	1.35	0
GAIF01032706.1	0	0	30.67	0	0	0
GAIF01032707.1	11.58	13.03	11.18	1.7	1.24	0.32
GAIF01032708.1	0.08	0	0	0.59	1.01	0
GAIF01032710.1	0.06	0	0	0.07	0.03	0
GAIF01032711.1	3.46	2.53	3.27	0.76	0.26	1.11
GAIF01032713.1	1.44	0	0	0	0.37	0
GAIF01032716.1	0.38	0.52	0	1.88	8.92	1.05
GAIF01032718.1	0	0.12	0.12	0.38	2.11	0.12
GAIF01032719.1	1.91	0.49	0	4.23	3.81	1.4
GAIF01032720.1	7.76	7.21	5.01	1.31	0.62	0.16
GAIF01032724.1	0	0	0	0.18	0.82	0
GAIF01032727.1	2.34	0	7.68	0	0.78	0
GAIF01032728.1	14.08	0.07	0	26.21	11.34	2.76
GAIF01032730.1	7.15	8.57	5	22.45	17.78	3.33
GAIF01032731.1	26.24	15.77	12.03	11.77	8.91	17.27
GAIF01032733.1	0.09	0	0	0.05	0.22	0.05
GAIF01032734.1	0	0	0	0	1.36	0
GAIF01032735.1	0	0.35	0	1.25	2.65	0
GAIF01032737.1	0.76	0.31	0.31	2.22	13.66	0.42
GAIF01032739.1	2.52	0	0	6.58	36.09	3.85
GAIF01032740.1	0.18	0	0	0	0.43	0
GAIF01032742.1	1.02	2.7	0.43	8.46	13.03	0.07
GAIF01032743.1	10.28	6.13	8.38	17.9	15.36	8.06
GAIF01032744.1	4.42	8.44	5.43	32.71	43.32	1.91
GAIF01032745.1	0.6	0.16	0	7.1	37.5	1.15
GAIF01032746.1	0	0	0.15	0.43	2.84	0.25
GAIF01032748.1	33.61	49.29	45.7	89.54	48.19	21.75
GAIF01032749.1	0.21	0	0	0.98	1.33	0
GAIF01032750.1	22.04	18.85	23.74	38.81	26.15	47.21
GAIF01032751.1	10.36	11.33	3.37	4.05	3.04	0.26

GAIF01032752.1	0.09	0	0	0	0.55	0
GAIF01032753.1	0.1	0	0	0.12	0.18	0
GAIF01032755.1	0.38	0.21	0	1.1	1.14	0.21
GAIF01032757.1	0.7	0.57	0	1.62	8.58	0.19
GAIF01032758.1	20.25	14.79	29.51	66.29	0	25.05
GAIF01032759.1	0.78	0	0	1.99	1.87	0.17
GAIF01032761.1	0.12	0.22	0.04	0.56	1.21	0
GAIF01032762.1	17.02	39.95	14.4	42.98	51.16	6.6
GAIF01032763.1	0.45	0.1	0	0.48	0.88	0.17
GAIF01032765.1	3.46	2.83	1.82	4.37	7.4	0.66
GAIF01032766.1	4.5	3.63	4.3	14.99	9.25	5.76
GAIF01032767.1	187.65	141.61	34.92	0	0.47	0
GAIF01032768.1	0	0	0	0.04	0	0
GAIF01032770.1	0	0	0	0.11	2.12	0.31
GAIF01032771.1	0	0	0	0.15	1.11	0
GAIF01032775.1	0	0.28	0	0.29	4.13	0
GAIF01032776.1	1.23	0.24	0.08	6.3	19.89	0.43
GAIF01032777.1	0	0	0	0.21	0.33	0
GAIF01032778.1	0.43	0.31	0.14	0.2	0.1	0
GAIF01032779.1	537.5	469.73	492.77	99.08	58.7	50.63
GAIF01032780.1	0.07	0	0	0	0.18	0
GAIF01032781.1	0	7.04	6.45	0	0	1.31
GAIF01032782.1	14.07	11.81	10.86	26.51	37.36	13.82
GAIF01032783.1	0.46	0	0	2.16	12.29	0.26
GAIF01032784.1	0.12	0	0	0	0.62	0
GAIF01032787.1	5.09	3.18	2.46	3.11	1.87	1.45
GAIF01032788.1	2.47	3.28	4.85	5.95	2.68	1.7
GAIF01032791.1	0.06	0	0	0.14	0.42	0
GAIF01032792.1	4.52	8.53	2.15	6.93	11.02	3.75
GAIF01032795.1	0	0	0	0	0.04	0
GAIF01032796.1	0	0	0	0.87	0.38	0
GAIF01032797.1	5.12	4.32	4.71	7.65	3.14	2.77
GAIF01032798.1	0	0	0	0.19	0.19	0
GAIF01032799.1	56.14	71.24	66.78	59.07	56.65	29.95
GAIF01032800.1	0.09	0	0	0.9	0.83	1.51
GAIF01032801.1	0.91	1.31	0	1.54	1.59	0
GAIF01032802.1	0	0	0.12	0	0	0
GAIF01032803.1	0	0	0	0	0.3	0
GAIF01032804.1	2.82	1.79	0	9.53	38.1	1.03
GAIF01032805.1	766.98	516.89	259.25	3976	3246.02	302.7
GAIF01032808.1	86.67	40.17	74.33	178.25	252.92	149.95
GAIF01032810.1	0	0	0	0	0.06	0
GAIF01032811.1	4.66	10.34	2.51	18.79	15.73	0.2
GAIF01032812.1	0	0.06	0	0.24	3.94	0

GAIF01032814.1	38.33	3.01	5.67	7.41	3.78	28.68
GAIF01032815.1	4.15	9.89	7.51	5.48	7.32	2.43
GAIF01032819.1	0	0	0	0	0.4	0
GAIF01032823.1	0	0.23	0	0	0	0
GAIF01032826.1	1.34	0.15	0	0.31	12.44	0
GAIF01032827.1	35.49	33.17	28.58	86.5	106.82	25
GAIF01032830.1	0	0	0	0	0.54	0
GAIF01032832.1	0	0	0	0	0.9	0
GAIF01032833.1	0.62	0.27	0.26	0.08	0	0
GAIF01032836.1	17.2	29.47	18.13	51.15	24.93	8.66
GAIF01032837.1	1.43	2.33	0.16	6.63	2.75	0
GAIF01032838.1	13.44	18.5	14.16	50.75	44.38	5
GAIF01032839.1	0.64	0.51	0.87	0.97	0.58	0.18
GAIF01032840.1	0.23	0.19	0	0	0.56	0.33
GAIF01032842.1	0	0	0	0	0.14	0
GAIF01032843.1	0.53	0.77	0.78	0.74	1.1	0.36
GAIF01032844.1	0.11	0.12	0.25	0.13	0.67	0
GAIF01032846.1	93.46	108.57	103.74	70.39	67.99	124.58
GAIF01032848.1	0	0	0.07	0.12	0.37	0.11
GAIF01032852.1	295.8	171.92	120.77	239.62	247.29	456.39
GAIF01032854.1	10.55	14.52	11.52	24.31	22.05	4.79
GAIF01032855.1	16.24	21.69	32.67	27.07	17.94	22.8
GAIF01032861.1	0.29	0	0	0.21	0.98	0
GAIF01032862.1	47.33	66.45	76.5	69.38	65.21	83.88
GAIF01032863.1	0.21	0	0	0.16	0.19	0
GAIF01032864.1	54.02	37.66	30.83	51.99	55.64	85.22
GAIF01032865.1	0	0	0	0.31	0.22	0.21
GAIF01032870.1	44.04	52.76	42.79	48.6	33.9	22.32
GAIF01032871.1	0.15	0.33	0.49	0.35	0.36	0.33
GAIF01032872.1	52.45	136.79	103.64	95.62	37.84	53.54
GAIF01032873.1	0.11	0.24	0.25	0	0	0.06
GAIF01032874.1	5.2	6.06	3.6	21.71	22.65	1.39
GAIF01032875.1	0	0	0	0.04	0.02	0
GAIF01032876.1	59.81	80.71	70.39	65.9	48.83	30.45
GAIF01032877.1	40.18	42.16	31.61	34.93	43.16	29.85
GAIF01032878.1	76.71	33.33	15.03	96.03	56.7	7.99
GAIF01032879.1	9.16	10.24	1.7	4.34	3.26	0
GAIF01032880.1	5.28	3.3	1.6	18.66	20.96	15.24
GAIF01032881.1	0	0	0	0.57	0	0.56
GAIF01032882.1	0	0	0	0	0.04	0
GAIF01032883.1	6.62	8.85	6.27	16.72	28	34.74
GAIF01032884.1	122.2	147.61	111.55	105.03	80.21	106.48
GAIF01032885.1	14.74	3.66	1.98	22.74	46.91	81.16
GAIF01032886.1	10.7	5.26	1.45	17.71	20.47	0.59

GAIF01032887.1	0	0.57	0.26	0	0	0.14
GAIF01032888.1	0.04	0	0.01	0.43	3.16	0
GAIF01032889.1	0	0.33	0.05	0	0	0
GAIF01032891.1	1.87	3.84	4.31	19.37	19.48	1.3
GAIF01032893.1	0	0.03	0	0	0	0
GAIF01032895.1	0.81	0.76	0.86	0.54	0	0.48
GAIF01032897.1	0	0.03	0	0	0	0
GAIF01032898.1	4.09	4.57	2.51	3.63	2.17	0.26
GAIF01032899.1	7.25	6.84	4.31	2.49	1.16	0.33
GAIF01032900.1	0.31	0.23	0	0.84	7.24	0.11
GAIF01032901.1	0	0	0	0	0.08	0
GAIF01032902.1	25.41	54.33	41.96	68.73	74.95	7.58
GAIF01032903.1	0.07	0	0	0	0	0
GAIF01032904.1	10.12	14.22	12.18	28.62	23.9	4.17
GAIF01032905.1	304.46	153.18	110.46	607.96	1274.32	88.59
GAIF01032906.1	0	0	0	0.15	0	0
GAIF01032907.1	241.24	25.47	27.34	299.57	510.17	4316.36
GAIF01032909.1	12.47	7.57	3.79	6.08	2.63	1.83
GAIF01032910.1	3137.82	2661.39	2124.23	1896.63	1773.03	4997.19
GAIF01032912.1	0	0	0	0.18	0.47	0
GAIF01032913.1	370.06	438.6	329.1	106.19	59.54	79.83
GAIF01032916.1	0	0	0	0.54	2.66	0.1
GAIF01032917.1	14.21	20.56	19.14	36.99	30.57	8.98
GAIF01032918.1	0	0	0.39	0.12	0.12	0
GAIF01032921.1	10.74	5.26	6.83	16.19	17.13	8.56
GAIF01032922.1	22.95	1.51	1.64	28.82	4.02	5.94
GAIF01032923.1	0	0	0	0	0.17	0
GAIF01032924.1	0	0	0	0.15	0	0
GAIF01032925.1	0.6	0.6	0.38	0.41	0.24	0.22
GAIF01032928.1	16.5	26.1	18.6	41.71	30.08	6.41
GAIF01032930.1	1.19	0	0.65	0.23	0.95	0
GAIF01032932.1	0.17	0.37	0.46	1.28	2.95	0.19
GAIF01032933.1	7.69	8.2	13.09	39.8	55.81	65.88
GAIF01032934.1	0	0.22	0	0.49	2.23	0.44
GAIF01032935.1	0	0	0	0	0.52	0
GAIF01032936.1	1.18	2.31	0.43	7.03	7.85	0.43
GAIF01032937.1	0	0.29	0	0	0	0
GAIF01032938.1	2.3	0.17	0.17	12.5	44.19	4.74
GAIF01032939.1	0.32	0.69	0.35	0	0.19	0
GAIF01032941.1	0	0	0.06	0.74	0.09	0
GAIF01032942.1	0.06	0	0	0	0.46	0.21
GAIF01032943.1	17.01	10.63	12.9	9.54	2.21	2.27
GAIF01032944.1	5.33	8.36	6.19	1.9	2.1	0.57
GAIF01032948.1	3.59	5.66	1.43	10.71	9.31	0.34

GAIF01032949.1	4.63	3.65	5.73	0.47	0.05	0
GAIF01032950.1	0.15	0	0	0	0	0
GAIF01032952.1	0.68	0	0.87	0.25	0.56	0
GAIF01032953.1	0	0	0	0	0.17	0
GAIF01032954.1	14.49	7.07	14.7	11.14	4.96	5.04
GAIF01032955.1	0.56	0.17	0.31	1.55	3.88	0.65
GAIF01032956.1	28.43	21.55	5.31	76.63	86.26	3.87
GAIF01032961.1	0	0	0.15	0	0	0
GAIF01032964.1	1.29	0.77	0.6	1.45	19.08	5.15
GAIF01032966.1	16.88	29.75	24.54	88.91	48.02	6.8
GAIF01032968.1	0.04	0	0	0.3	0.62	0
GAIF01032969.1	0.21	0	0	0	0.07	0
GAIF01032972.1	0	0	0.03	0	0	0
GAIF01032973.1	0.2	0.32	0	2.53	0.12	0
GAIF01032974.1	0.4	0	0	2.03	8.42	0.15
GAIF01032975.1	10.81	6.68	2.71	24.41	40.56	3.25
GAIF01032976.1	0	0	0	0	1.47	0.37
GAIF01032977.1	0.07	0	0	0.65	1.52	0
GAIF01032979.1	0	0	0	0.09	0	0
GAIF01032980.1	0.23	0.13	0.26	1.63	2.39	0.26
GAIF01032981.1	432.06	84.12	69.34	240	73.93	46.56
GAIF01032983.1	0	0	0	0.15	0.46	0.07
GAIF01032985.1	132.28	69.24	50.18	152.73	160.22	538.84
GAIF01032987.1	19.89	23.25	10.61	44.04	40.2	3.18
GAIF01032988.1	0.09	0	0.09	0.1	0.21	0
GAIF01032989.1	0.27	0.3	0.99	0.68	2.11	0.17
GAIF01032990.1	0.2	0.65	0	1.85	0.84	0.22
GAIF01032991.1	21.61	21.09	10.48	62.02	59.97	6.46
GAIF01032992.1	0.44	0	0.69	0	0.38	0
GAIF01032994.1	1.48	1.69	0	17.99	43.85	0.49
GAIF01032995.1	0	0	0	0	0.01	0
GAIF01032996.1	9.32	10.83	6.73	24.09	28.26	7.29
GAIF01032997.1	7.95	6.08	0.16	0.29	0	0.07
GAIF01032998.1	30.42	38.4	35.21	61.09	36.65	11.62
GAIF01032999.1	38.91	39.98	22.09	40.14	21.32	6.73
GAIF01033001.1	0	0	0	0	1.27	0
GAIF01033003.1	1.09	1.08	0.44	0.46	1.32	0
GAIF01033004.1	6.62	7.33	4.93	1.65	1.56	0.65
GAIF01033005.1	9.27	6.91	1	3.39	1	0
GAIF01033006.1	0	0.34	0	0.36	0.06	0
GAIF01033007.1	0.06	0	0	0.59	1.69	0.25
GAIF01033008.1	11.64	5	6.23	17.71	24.82	28.13
GAIF01033010.1	26.56	19.07	12.81	0.73	0.38	0.35
GAIF01033011.1	0.46	0	0	1.06	2.19	0.5

GAIF01033013.1	0.21	0	0	0.64	0.69	0.46
GAIF01033014.1	0.21	0	0.19	0.05	0	0.14
GAIF01033015.1	10.18	5.48	10.66	22.96	19.53	71.31
GAIF01033018.1	0	0	0	0	0.06	0
GAIF01033019.1	13.24	6.16	9.45	1.46	0.26	0.39
GAIF01033020.1	0	0.2	0	0.36	1.37	0
GAIF01033021.1	0.19	0.33	0	0.04	0	0.08
GAIF01033022.1	1.87	2.07	1.41	9.39	10.45	0.44
GAIF01033023.1	28.91	32.99	20.95	204.07	194.26	24.91
GAIF01033024.1	1.29	0.47	0.24	4.37	15.51	0.36
GAIF01033025.1	0	0.14	0	0.17	0	0
GAIF01033026.1	6.91	10.2	4.66	0.22	0.38	0
GAIF01033028.1	5.28	3.42	2.81	11.83	18.86	7.58
GAIF01033029.1	0.02	0	0	0	0.07	0.01
GAIF01033030.1	0	0.09	0	0.89	4.21	0
GAIF01033031.1	0	0.01	0	0.06	0.43	0
GAIF01033032.1	0.16	0.09	0	0.85	6.74	0
GAIF01033033.1	0.08	0	0	1.22	1.87	0
GAIF01033034.1	2.4	2.72	3.07	14.27	9.38	1.98
GAIF01033036.1	0.63	0.11	0.11	0.27	0.37	0.14
GAIF01033037.1	2.54	4.22	0.44	19.25	27.66	0.25
GAIF01033039.1	4.66	4.77	10.84	2.71	1.21	5.29
GAIF01033040.1	73.82	47.87	38.26	12.98	5.8	2.47
GAIF01033042.1	6.45	4.52	5.39	15.35	6.81	8.95
GAIF01033044.1	1.4	0.49	0.2	5.69	17.25	0.1
GAIF01033045.1	71.66	43.89	22.73	117.1	121.9	75.52
GAIF01033046.1	1.37	1.08	0.27	0.43	1.2	2.2
GAIF01033048.1	22.09	11.57	14.65	32.17	18.27	22.55
GAIF01033050.1	0	0	0	0	5.03	0
GAIF01033051.1	184.18	177.38	95.84	422.27	436.59	146.63
GAIF01033052.1	0.63	0.1	0.06	6	20.91	0.23
GAIF01033053.1	0.04	0.13	0.09	0	0	0
GAIF01033054.1	9	6.43	2.46	13.13	8.33	1.64
GAIF01033055.1	0	0	0	0	0	0.12
GAIF01033056.1	7.96	13.06	12.34	23.68	21.05	3.31
GAIF01033059.1	0.03	0	0	0.04	0.38	0
GAIF01033060.1	0	0	0	0	2.98	0
GAIF01033064.1	0.28	0.36	0.18	0.58	1.07	0.61
GAIF01033065.1	2.66	3.81	3.97	0.74	0.26	0.23
GAIF01033066.1	0.08	0	0	0	0	0
GAIF01033067.1	8.9	5.39	3.5	34.18	65.53	18.66
GAIF01033069.1	2.41	1.87	3.03	0	0	0
GAIF01033070.1	2.12	0	0	5.57	36.88	1.09
GAIF01033072.1	7.04	18.7	11.75	11.72	10.44	8.29

GAIF01033074.1	0	0	0	0.14	0.1	0
GAIF01033075.1	0	0	0	0.07	0	0
GAIF01033077.1	88.16	81.24	84.72	66.36	53.66	46.38
GAIF01033079.1	106.6	63.94	29.56	111.03	95.67	44.16
GAIF01033081.1	30.25	11.29	5.62	4.84	3.38	0.49
GAIF01033082.1	0.56	0.26	0	0.83	2.67	0.26
GAIF01033083.1	0.15	0.08	0	1.21	4.05	0
GAIF01033084.1	24.31	37.63	26.74	131.09	99.46	21.09
GAIF01033085.1	0	0	0	9	0	7.02
GAIF01033086.1	1.48	0.47	0.05	16.42	45.97	0.34
GAIF01033087.1	0.05	0.13	0.25	0.07	0.25	0.06
GAIF01033090.1	5.02	2.81	1.45	8.21	2.78	0.53
GAIF01033091.1	1.55	0.56	0.09	1.9	6.21	0
GAIF01033092.1	0.12	0	0	1.52	7.8	0.46
GAIF01033093.1	0.53	0.21	0.25	4.32	113.15	22.17
GAIF01033094.1	0	0	0	0.88	0	0
GAIF01033095.1	0	0.87	0.44	0	0	0.06
GAIF01033096.1	0.16	0.17	0.06	0.97	5.53	0
GAIF01033097.1	0.07	0	0	0	0	0
GAIF01033098.1	0.81	0.53	0	2.31	2.69	0
GAIF01033099.1	0	4.77	7.71	0	12.69	0
GAIF01033101.1	11.93	24.01	22.63	49.11	42.03	5.23
GAIF01033102.1	0.51	0.55	0.72	1.1	2.1	0.08
GAIF01033104.1	0.15	0.04	0.27	0.19	0.73	0
GAIF01033105.1	0.46	0	0	0.86	4.13	0.39
GAIF01033106.1	0	0.12	0.49	0.45	0.34	0
GAIF01033107.1	0.54	0	0.3	0	0.33	0
GAIF01033109.1	1.82	1.64	2.79	7.48	3.33	1.83
GAIF01033110.1	53.38	74.54	82.98	66.73	41.17	35.69
GAIF01033112.1	3	7.3	0.29	13.64	40.07	0.19
GAIF01033113.1	0.03	0.03	0	0.09	0.36	0.03
GAIF01033115.1	25.73	13.07	10.67	1.01	0.37	0.56
GAIF01033116.1	0	0.02	0	0	0.32	0
GAIF01033117.1	5819.41	10827.75	7036.54	3409.97	5517.41	5891.88
GAIF01033118.1	30.37	9.9	5.68	36.21	40.91	2.14
GAIF01033120.1	0.49	0	0.31	2.04	13.71	0
GAIF01033122.1	10.21	10.27	10.09	16.88	15.65	2.81
GAIF01033123.1	7.6	5.33	7.52	24.03	14.26	13.56
GAIF01033125.1	0.04	0.08	0.04	0.69	3.46	0.91
GAIF01033126.1	3.04	2.73	2.52	4.8	7.6	0.12
GAIF01033127.1	1.28	2.46	0.58	4.54	6.16	0
GAIF01033128.1	0	0	0	0	0	0.05
GAIF01033129.1	0	0.14	0	0	0.78	0
GAIF01033131.1	42.48	0.77	0.56	3.29	2.43	11.82

GAIF01033132.1	15.83	11.42	16.58	21.68	36.01	55.78
GAIF01033133.1	8.04	7.12	1.37	7.11	8.33	0
GAIF01033136.1	0	0	0	0.22	0	0
GAIF01033137.1	7.84	0.03	0	0.01	0.21	0.05
GAIF01033138.1	2.25	1.58	1.54	0.68	0.87	0.95
GAIF01033139.1	2.47	0.96	0.19	13.3	39.43	0.78
GAIF01033140.1	77.55	82.73	105.01	150.39	63.95	45.84
GAIF01033141.1	52.45	0	38.39	0.96	0	0
GAIF01033142.1	0	0.76	0.65	0	0	0.63
GAIF01033143.1	0.01	0	0	0.27	0	0
GAIF01033145.1	0.13	0.05	0.07	0.64	4.29	0.14
GAIF01033146.1	1.35	0.68	1.16	1.64	2.33	0
GAIF01033148.1	560.68	376.74	374.03	1378.91	818.79	1541.75
GAIF01033149.1	0	0	0	2.57	7.51	0
GAIF01033150.1	0	0	0	0	0	0.2
GAIF01033151.1	11.71	7.5	2.49	18.61	20.25	1.61
GAIF01033152.1	1.67	0	0.18	8.53	27.32	3.01
GAIF01033153.1	0.13	0.41	0.14	0	0	0
GAIF01033154.1	32.42	34.97	38.43	44.7	48.72	80.74
GAIF01033155.1	0	0	0	0.06	0	0
GAIF01033156.1	2.12	0.28	0.71	4.92	11.82	0.92
GAIF01033157.1	0	0	0	0.02	0.24	0
GAIF01033159.1	4.6	2.46	0	8.3	7.49	3.36
GAIF01033160.1	11.91	9.02	7.21	20.78	22.72	5.34
GAIF01033162.1	0.22	0.1	0.02	1.31	4.34	0.1
GAIF01033163.1	232.17	56.04	70.19	201.76	220.26	745.36
GAIF01033164.1	0.07	0	0	0.3	0	0.16
GAIF01033165.1	0	0	0	0	0.38	0
GAIF01033166.1	0	0	0.05	0	0	0
GAIF01033167.1	0.14	0.23	0.24	0.5	0	0
GAIF01033169.1	212.73	289.68	78.54	575.41	503.41	31.2
GAIF01033170.1	0	0	0	0.16	0.09	0.15
GAIF01033171.1	0	0	0	0	0.02	0
GAIF01033173.1	4.38	1.13	0.1	7.91	35.03	12.64
GAIF01033174.1	1.02	0.14	0.28	9.22	36.53	0.98
GAIF01033175.1	0.22	0.2	0	1.71	7.96	1.15
GAIF01033176.1	0	0	0	0.08	0.16	0
GAIF01033178.1	0	0.11	0	4.02	11.2	0.11
GAIF01033182.1	0.06	0	0	1.35	5.21	0.43
GAIF01033183.1	3.39	5.96	0.95	6.15	13.48	0.27
GAIF01033184.1	0	0	0	0	0.02	0
GAIF01033186.1	4.4	12.2	12.74	23.85	22.8	1.1
GAIF01033187.1	108.7	126.64	126.48	146.22	76.76	32.59
GAIF01033188.1	12.68	20.49	24.17	32.4	17.16	9.71

GAIF01033189.1	0.19	0.04	0.12	0.13	0.13	0
GAIF01033191.1	0.43	0.23	0.24	1.41	4.74	0.39
GAIF01033192.1	56.33	72.18	80.45	90.15	94.94	58.21
GAIF01033193.1	0.07	0	0.08	0.24	1.18	0
GAIF01033194.1	5.62	3.71	8.34	11.19	8.36	38.03
GAIF01033196.1	0.6	0.09	0	1.5	7.06	1.43
GAIF01033197.1	3.55	1.81	1.81	7.98	12.39	1.23
GAIF01033198.1	1.32	0.36	1.81	2.11	0.2	1.46
GAIF01033201.1	78.67	0	0	45.65	55.19	231.2
GAIF01033203.1	0	0.31	0	0.33	0	0
GAIF01033204.1	0	0	0.24	0.25	0.13	0.12
GAIF01033205.1	0.41	0.68	0.82	0.65	0.49	0.37
GAIF01033207.1	4.27	2.3	1.6	8.73	5.69	0.95
GAIF01033208.1	0	0	0	0	0.23	0
GAIF01033209.1	0.66	0.11	0.23	4.54	21.98	1.09
GAIF01033210.1	0	0	0	0.01	0.05	0
GAIF01033213.1	14.47	19.27	11.39	27.41	25.99	3.23
GAIF01033214.1	2.62	1.59	1.57	0.69	0.53	0
GAIF01033216.1	0.72	0.13	0	0.27	1.19	0
GAIF01033218.1	0	0	0	0	0.05	0
GAIF01033219.1	1.74	0	1.17	1.61	0.81	0
GAIF01033220.1	0	0	0.19	0.62	0.11	0
GAIF01033221.1	0	0	0	0	0.07	0
GAIF01033222.1	0.52	0.28	0.57	0	0.31	1
GAIF01033223.1	0	0	0	0.32	0.5	0
GAIF01033224.1	57.94	40.61	33.75	162.92	286.12	96.7
GAIF01033225.1	0.03	0	0	0.16	0.4	0
GAIF01033226.1	22.25	14.64	11.74	35.49	42.17	24.67
GAIF01033227.1	20.36	18.61	27.83	17.18	13.58	8.46
GAIF01033228.1	100.21	69.67	56.19	11.83	6.26	5.76
GAIF01033229.1	10.09	15.28	13.33	21.06	10.91	4.08
GAIF01033230.1	0	0	0	0	0.61	0
GAIF01033232.1	0.47	0.2	0.1	2.08	7.59	0
GAIF01033233.1	0.52	1.18	1.06	8.04	13.56	0.24
GAIF01033234.1	0	5.93	5.27	0	0	0
GAIF01033235.1	0.08	0	0	0.64	0	0.17
GAIF01033236.1	0	0	0	0	0	0.18
GAIF01033237.1	0.06	0	0	0	0.15	0
GAIF01033238.1	0	0	0.1	0	0.11	0
GAIF01033239.1	10.06	6.59	7.71	4.9	7	18.96
GAIF01033240.1	0.06	0	0.06	2.45	11.14	0.06
GAIF01033242.1	0	0	0	0.19	1.07	0
GAIF01033243.1	6.28	4.5	2.68	13.17	18.95	6.15
GAIF01033245.1	3.91	10.63	11.8	30.26	44.9	7.15

GAIF01033246.1	0	0	0	0	0.58	0.18
GAIF01033247.1	171.46	102.25	68.85	148.38	101.83	38.35
GAIF01033248.1	0.55	0.13	0.34	1.6	1.1	0.3
GAIF01033249.1	25.8	30.76	27.68	50.46	42.25	17.31
GAIF01033250.1	0	0	0	0	0.35	0.32
GAIF01033251.1	27.07	36.79	5.57	35.34	54.48	0
GAIF01033252.1	0	0	0	0	0.28	0
GAIF01033253.1	32.68	8.77	9.4	30.63	47.51	26.31
GAIF01033255.1	0	0	0	1.2	3.52	0
GAIF01033257.1	60.61	157.74	87.51	85.62	113.61	15.01
GAIF01033258.1	0.62	0.11	0	1.67	2.1	0
GAIF01033259.1	24.59	2.92	1.87	80.33	284.65	634.76
GAIF01033261.1	0	0	0.06	0.11	1.61	0
GAIF01033262.1	0.11	0	0	0.62	1.47	0
GAIF01033264.1	0.06	0.98	0.87	0	0	0.06
GAIF01033265.1	6.07	5.57	5.51	20.63	31.07	1.5
GAIF01033266.1	0.36	0.17	0	0.87	0.12	0.16
GAIF01033269.1	0.28	0.4	0.41	0.43	0	0
GAIF01033270.1	23.92	21.42	24.65	37.64	23.9	8.82
GAIF01033271.1	5.03	5.58	5.44	15.79	24.66	5.28
GAIF01033272.1	5.37	1.5	1.84	2.48	0	1.79
GAIF01033273.1	0.02	0.04	0	0	0.25	0
GAIF01033274.1	0	0	0	0	0.05	0
GAIF01033275.1	19.9	22.03	22.24	36.37	18.84	12.6
GAIF01033280.1	0.12	0.08	0.03	1.62	10.75	0.08
GAIF01033281.1	0.83	0.31	0.11	4.14	13.59	0.26
GAIF01033284.1	4.6	0	0	0	0	0
GAIF01033286.1	0	0.04	0	0	0.05	0
GAIF01033287.1	0	0	0	0.06	0.06	0
GAIF01033288.1	0.23	0.1	0	0.47	5.32	0.35
GAIF01033289.1	0.02	0.02	0	0.13	1.44	0.08
GAIF01033290.1	0	0.16	0	0.86	1.25	0
GAIF01033292.1	0.07	0	0	0	0	0
GAIF01033293.1	0	0	0	0	0.74	0
GAIF01033294.1	0.05	0	0	0	0	0
GAIF01033295.1	0	0	0	0	0.14	0
GAIF01033296.1	8.75	18.35	21.56	15.55	10.76	8.79
GAIF01033297.1	52.89	49.39	14.38	85.06	80.19	27.84
GAIF01033298.1	0	0	0	0	0	8.26
GAIF01033299.1	0	0	0	0.1	1.92	0
GAIF01033301.1	0.14	0.15	0	2.8	14.65	0
GAIF01033303.1	0	0	0	0.08	0.16	0
GAIF01033304.1	1.94	0.21	0.3	6.27	27.58	10.4
GAIF01033305.1	27.7	64.58	51.6	106.71	75.71	10.41

GAIF01033306.1	0.81	0	0	0.81	0.28	0.38
GAIF01033307.1	0.98	0.27	0.27	2.1	5.46	0.32
GAIF01033308.1	0	0	0	0	0.24	0
GAIF01033310.1	0.04	0.06	0.09	0.51	2.72	0.09
GAIF01033312.1	0.03	0	0.02	0.02	0.04	0
GAIF01033313.1	0	0.11	0	0.12	1.14	0
GAIF01033314.1	0	0	0	0	0.15	0.07
GAIF01033315.1	21.67	21.68	31.37	11.35	3.92	7.82
GAIF01033316.1	16.71	12.29	17.59	34.8	32.33	48.84
GAIF01033317.1	0.78	0.49	0.09	8.41	14.53	0.14
GAIF01033318.1	0	0	1.28	0.77	0.91	0
GAIF01033319.1	0	0	0	0.01	0.07	0
GAIF01033321.1	7.57	11.1	18.41	18.84	11.71	10.66
GAIF01033322.1	0.2	1.02	0.18	5.09	2.32	0.15
GAIF01033323.1	24.96	17.05	16.87	28.01	17.61	11.94
GAIF01033324.1	3.08	2.28	0.38	4.71	10.52	3.47
GAIF01033325.1	0.68	0.27	0.23	0.17	0.4	0.2
GAIF01033326.1	0.26	0.67	0	0.34	0.41	0
GAIF01033327.1	0	0.09	0	0	0.01	0
GAIF01033329.1	0.22	0.97	1.22	0.09	0.18	0.82
GAIF01033331.1	0.42	0.22	0.15	0.27	0	0
GAIF01033332.1	45.22	53.84	28.91	133.2	92.76	9.46
GAIF01033333.1	0.54	1.42	0	0.19	0.16	0.79
GAIF01033334.1	3.25	1.12	0	3.94	5.45	0.51
GAIF01033335.1	4.96	2.49	8.79	2.51	1.53	4.34
GAIF01033336.1	3.77	5.53	5.31	8.63	6.63	1.8
GAIF01033337.1	6.62	11.99	6.6	15.71	12.17	2.35
GAIF01033338.1	9.57	11.86	9.55	25.83	12.65	3.58
GAIF01033339.1	1.11	0.83	1.34	2.12	2.08	0.6
GAIF01033340.1	0.04	0	0	0	0	0
GAIF01033341.1	9.27	13.23	14.63	12.33	16.96	14.26
GAIF01033342.1	23.52	13.32	16.69	46.08	52.48	49.66
GAIF01033343.1	1.16	0.74	0.35	2.13	6.06	0.18
GAIF01033345.1	5.22	6.15	0.72	0.62	0	0.92
GAIF01033347.1	51.53	23.97	27.28	42.14	19.36	10.03
GAIF01033349.1	13.71	18.69	10.09	14.22	10.46	1.61
GAIF01033350.1	0.12	0.33	0.42	1.7	4.44	0.13
GAIF01033351.1	1.02	0.51	0.41	1.78	1.84	1.17
GAIF01033352.1	13.26	31.82	29.2	28.88	32.74	5.58
GAIF01033353.1	28.51	18.74	17.03	30.97	31.24	25.32
GAIF01033354.1	0.24	2.57	0.39	4.38	7.66	0.13
GAIF01033355.1	0.44	0	0	0	1.58	0.96
GAIF01033356.1	0.12	0.2	0	0.28	0.53	0
GAIF01033358.1	0	0	0	0	0.4	0

GAIF01033359.1	0.19	0.13	0	0	0	0
GAIF01033360.1	0	0	0	0	0.36	0
GAIF01033363.1	2.17	0.48	0.33	1.44	8.03	0.36
GAIF01033364.1	39.72	63.08	25.28	60.09	64.45	16.28
GAIF01033365.1	0.08	0	0.09	0.59	2.23	0
GAIF01033366.1	49.67	98.76	64.66	133.43	117.89	15.9
GAIF01033367.1	1.41	2.25	0.4	2.79	5.4	1.01
GAIF01033368.1	14.39	2.07	8.9	37.27	36.75	12.94
GAIF01033369.1	0	0	0	0.06	0	0
GAIF01033370.1	16.94	16.35	12.38	33	24.77	5.78
GAIF01033371.1	1.32	1.15	3.77	6.13	8.26	1.75
GAIF01033373.1	409.1	832.56	909.85	70.16	50.43	41.05
GAIF01033374.1	0.09	0.05	0	0	0.1	0
GAIF01033375.1	0.16	0.43	0	0.09	0.95	0
GAIF01033376.1	4.68	6.11	6.17	18.48	18.02	10.32
GAIF01033377.1	4.96	7.87	6.91	11.51	9.51	0.59
GAIF01033378.1	0	0	0.11	0.18	1.04	0
GAIF01033379.1	6.95	9.27	3.42	11.62	15.66	0.3
GAIF01033380.1	16.42	11.9	27.08	40.41	36.62	67.5
GAIF01033381.1	17.15	23.34	29.38	30.47	15.53	7.93
GAIF01033382.1	0.48	0.13	0	1.95	16.76	0.13
GAIF01033386.1	9.69	29.53	44.06	10.29	11.05	48.61
GAIF01033388.1	23.37	30.44	31.1	64.59	34.67	12.05
GAIF01033390.1	4.49	7.15	4.37	12.45	17.07	2.81
GAIF01033391.1	0	0	0	0.12	0.06	0
GAIF01033392.1	0.09	0	0.15	1.62	3.35	0.1
GAIF01033393.1	33.96	46.62	52.45	52.88	59.53	88.54
GAIF01033395.1	5.44	13.78	4.75	11.21	18.99	0.69
GAIF01033399.1	19.9	1.33	0.87	40.86	233.72	7.87
GAIF01033401.1	0	0	0	0	0.05	0
GAIF01033402.1	0	0	0	0	0.28	0
GAIF01033404.1	36.09	41.28	11.47	71.93	70.56	8.28
GAIF01033405.1	0	0	0.23	0.36	0	1.04
GAIF01033406.1	3070.58	2183.21	2288.74	1565.85	1506.04	4965.05
GAIF01033408.1	0	0	0	0.11	0.34	0
GAIF01033409.1	0	0	0	0	0.19	0
GAIF01033410.1	0.35	0.26	0	0	0.14	0
GAIF01033411.1	2	5.22	3.19	5.92	7.76	0.88
GAIF01033413.1	7.42	5.44	1.28	9.24	17.74	0.69
GAIF01033414.1	61.3	22.66	44.03	93.2	34.29	91.58
GAIF01033415.1	0.14	0	0	0	1.12	0.08
GAIF01033416.1	4.93	1.43	0.6	34.22	171.74	0.97
GAIF01033417.1	0.09	0	0	0.59	1.86	0
GAIF01033419.1	2.78	1.68	0.76	0.72	0.65	0

GAIF01033420.1	0.15	0	0	0	0	0
GAIF01033421.1	0.13	0	0	0.3	0.16	0
GAIF01033422.1	0.05	0	0	0.66	1.81	0.06
GAIF01033423.1	0	0	0.12	0.04	0	0
GAIF01033424.1	0.19	0.94	0.95	0.22	0.12	0
GAIF01033428.1	0	0	0.05	0.12	0.42	0.22
GAIF01033429.1	0.37	0.46	0.23	0.67	3.92	0
GAIF01033430.1	36.3	0.36	0.92	0.78	0	0
GAIF01033431.1	0	0	0	0.21	0.44	0
GAIF01033433.1	0	0	0.21	0	1.14	0.42
GAIF01033435.1	0.11	0	0.28	0.74	0.48	0
GAIF01033436.1	0	0	0.13	0	0	0
GAIF01033437.1	0.13	0	0	0	0	0.29
GAIF01033438.1	1.23	0.4	1.42	0.74	0.36	1.08
GAIF01033439.1	5.22	3.89	4.82	5.84	2.85	1.24
GAIF01033440.1	1.05	0	0.19	1.02	2.18	3.67
GAIF01033441.1	0.56	0.17	0.34	7.61	34.98	3.18
GAIF01033442.1	3.89	3.32	6.1	0.78	0.04	0.3
GAIF01033443.1	0.88	0.55	0	2.34	4.85	0.28
GAIF01033444.1	1.83	2.38	2.91	0.42	0.11	0
GAIF01033445.1	3.11	3.39	3.17	6.01	3	1.55
GAIF01033446.1	11.72	8.26	14.36	142.3	80.24	106.24
GAIF01033448.1	88.4	23.91	31.26	47.29	38.3	11.36
GAIF01033449.1	0.16	0	0	0.25	0	0
GAIF01033451.1	0.04	0	0	0.04	0	0
GAIF01033452.1	0	0	0	0	0.14	0
GAIF01033453.1	35.86	35.07	18.19	20.58	19.56	1.27
GAIF01033454.1	15.82	17.52	5.54	37.98	31.86	2.18
GAIF01033458.1	11.39	0.01	0.11	16.35	14.87	7.11
GAIF01033459.1	64.9	86.6	40.35	216.64	106.67	22.65
GAIF01033461.1	24.02	1.54	1.51	36.52	31.71	8.34
GAIF01033462.1	0.5	0	0.19	1.47	7.08	1.33
GAIF01033464.1	0.44	0.17	0	0.61	1.32	0.49
GAIF01033465.1	0.24	0.18	0.42	0.85	0.57	0.73
GAIF01033468.1	0.39	0	0	0.19	1.75	0
GAIF01033469.1	0	0.03	0	0	0.03	0
GAIF01033470.1	0	0	0.12	0	0.25	0
GAIF01033471.1	0	0	0	0	0.28	0
GAIF01033472.1	0	0.06	0	0.17	1.73	0
GAIF01033473.1	10.68	8.81	5.51	43.28	69.1	2.89
GAIF01033475.1	0.1	0	0	0	0	0
GAIF01033478.1	0	0	0	0.59	1.52	0
GAIF01033480.1	0	0	0	0.1	0	0
GAIF01033481.1	0.35	2.35	4.65	2.52	2.17	0.87

GAIF01033482.1	33.33	52.87	6.89	0.58	0.66	0.42
GAIF01033483.1	0	0.34	0.51	0.91	0.19	0.52
GAIF01033484.1	20.5	20.14	10.17	16.45	20.37	3.32
GAIF01033485.1	49.34	67.28	136.85	123.31	53.5	82.25
GAIF01033487.1	0	0	0.07	0.14	0	0.14
GAIF01033488.1	0.73	0.2	0.13	5.25	17.51	1.55
GAIF01033490.1	0	0	0.06	0	0.38	0
GAIF01033491.1	0	0	0	0.11	0.42	0
GAIF01033492.1	28.91	12.11	48.83	55.43	64.67	31
GAIF01033493.1	0	0	0.08	0	0	0
GAIF01033494.1	2.93	5.56	6.81	5.56	4.42	8.86
GAIF01033495.1	22.88	7.78	3.45	34.84	26.96	52.36
GAIF01033496.1	2.58	1.93	1.25	9.36	10.14	36.9
GAIF01033497.1	48.61	63.71	51.17	123.8	149.31	33.61
GAIF01033499.1	6.29	8.14	3.94	31.42	25.45	2.68
GAIF01033500.1	17.42	14.79	21.23	5.75	3.07	6.17
GAIF01033501.1	0.05	0.1	0.05	0.16	1.33	0
GAIF01033503.1	0	0.44	0.06	0.51	1.15	0.09
GAIF01033504.1	14.2	19.42	21.32	19.15	14.88	7.31
GAIF01033507.1	0	0	0	0	0	0.1
GAIF01033508.1	0.08	0.03	0.03	0	0	0
GAIF01033511.1	73.24	54.76	91.15	69.64	47.41	42.02
GAIF01033512.1	1.18	0.77	0.39	1.51	0.28	0.26
GAIF01033513.1	0	0.04	0	0	0	0
GAIF01033514.1	0.03	0	0.07	0	0.23	0
GAIF01033515.1	6.53	4.03	1.31	11.79	17.62	1.91
GAIF01033516.1	17.77	5.73	0	7.08	6.67	1.88
GAIF01033518.1	0.45	0	0	0	0.44	0
GAIF01033519.1	8.24	3.09	5.6	18.34	31.15	31.14
GAIF01033520.1	1.73	2.82	0	0	1.73	0
GAIF01033523.1	23.54	25.54	22	3.06	1.98	2.54
GAIF01033524.1	14.1	24.74	15.88	22.09	11.83	2.7
GAIF01033525.1	11.55	18.55	6.21	48.14	67.04	1.37
GAIF01033526.1	2.22	0	0.26	53.17	111.13	42.61
GAIF01033527.1	0.07	0	0	0	0.46	0
GAIF01033528.1	0.09	0	0	0	0.11	0
GAIF01033529.1	0	0.26	0	0	0	0
GAIF01033532.1	0.28	0	0.05	0.71	3.12	0
GAIF01033533.1	7.71	6.73	5.06	21.28	43.12	3.4
GAIF01033534.1	0	0	0	0.3	0	0
GAIF01033535.1	62.85	23.68	11.11	90.65	76.19	14.43
GAIF01033537.1	0	0	0	0.05	0	0
GAIF01033538.1	0	0	0	0.02	0	0
GAIF01033540.1	0	0.04	0	0	0.01	0

GAIF01033541.1	0.15	0	0.08	2.69	3.99	0.34
GAIF01033543.1	3.07	0.17	0.34	0.54	0.75	1.38
GAIF01033544.1	158.08	38.19	34.75	1.94	1.02	0.64
GAIF01033547.1	0	0	0.12	2.63	11.67	0.12
GAIF01033549.1	19.72	20.5	21.86	27.27	15.11	11.08
GAIF01033550.1	0.27	0.07	0.03	0.75	3.65	0
GAIF01033551.1	0	0.05	0	0.15	0	0
GAIF01033552.1	11.15	3.73	13.51	4.18	4.84	7.78
GAIF01033553.1	0.46	0	0	0.09	0.09	0
GAIF01033554.1	2.25	0.46	0	1.1	2.99	0.33
GAIF01033555.1	0	0.27	0.14	0	0	0
GAIF01033556.1	0	0	0	0.04	0	0
GAIF01033557.1	65.89	68.59	0.65	122.57	193.91	12.45
GAIF01033558.1	0	0	0	0	0.09	0
GAIF01033559.1	311.83	227.66	85.91	668.13	1080.98	59.53
GAIF01033561.1	0	0	0	0	0	0.05
GAIF01033562.1	0	0.07	0	0	0	0.07
GAIF01033563.1	0	0	0.2	0	0	0
GAIF01033564.1	0.26	1.43	0	1.83	1.54	0.11
GAIF01033565.1	63.34	22.39	16.23	111.57	126.07	54.49
GAIF01033566.1	0	0	0	0	0.9	0.25
GAIF01033567.1	12.02	12.52	20.46	27.67	35.22	17.22
GAIF01033568.1	0	0	0	1	0.74	0
GAIF01033569.1	0	0	0	0	0.3	0
GAIF01033570.1	0.34	0	0	0.1	0.17	0.06
GAIF01033571.1	0	0	0.01	0.14	0.27	0
GAIF01033572.1	0.96	0	2.07	0.63	0	0.73
GAIF01033573.1	178.5	225.13	348.82	139.83	128.49	408.67
GAIF01033574.1	36.16	37.64	34.84	64.96	42.62	12.57
GAIF01033575.1	2.87	0.68	0.14	10.42	21.3	0.55
GAIF01033576.1	0.13	0.52	0.72	0	0.15	0.25
GAIF01033577.1	0	0	0	0	2.95	0
GAIF01033578.1	6.79	4.8	2.63	19.4	27.66	2.45
GAIF01033579.1	0.07	0	0	0.08	1.08	0.08
GAIF01033580.1	0	0	0	0	0.18	0
GAIF01033581.1	5.41	1.38	0.06	3.28	7.2	1.78
GAIF01033583.1	15.31	0	7.48	11.25	20.18	21.48
GAIF01033584.1	1.11	1.34	0.81	5.16	9.21	1.77
GAIF01033585.1	0.1	0.05	0.05	0.73	2.46	0.27
GAIF01033586.1	0	0	0	0	0.1	0
GAIF01033587.1	0.4	0.1	0.03	0.09	0	0.06
GAIF01033588.1	30.4	9.97	4.74	19.46	66.88	18
GAIF01033590.1	0	0	0.16	0	0.51	0
GAIF01033591.1	0	0	0	0.52	1.88	0.12

GAIF01033592.1	0	0.04	0	0	0	0
GAIF01033594.1	0	0	0	0.51	0.88	0
GAIF01033595.1	0	0	0.1	0	0	0
GAIF01033596.1	0.18	0.09	0.05	1.13	5.17	0.41
GAIF01033597.1	0	0	0.53	0.28	0	0.63
GAIF01033598.1	724.29	1592.93	376.09	724.36	1331.04	620.21
GAIF01033599.1	0.79	0.17	0.17	3.29	8.56	6.79
GAIF01033601.1	3.75	7.93	6.25	19.19	11.35	1.33
GAIF01033602.1	122.92	63.84	40.94	26.33	26.13	13.17
GAIF01033606.1	11.9	18.26	14.55	17.57	30.79	6.81
GAIF01033608.1	0.07	0.33	0.41	0.43	0.57	0
GAIF01033609.1	0	0	0	0.17	0	0
GAIF01033610.1	57.15	35.82	23.41	73.57	86.64	88.82
GAIF01033612.1	17.17	9.99	1.28	60.49	59.09	0
GAIF01033613.1	4.73	4.02	2.64	13.36	2.07	0.95
GAIF01033616.1	0	0	0	0.52	0.44	0.51
GAIF01033618.1	0.23	0	0	0	0	0
GAIF01033619.1	4.01	0	0	22.32	54.85	26.07
GAIF01033620.1	0	0	0	0	0.08	0
GAIF01033621.1	18.49	8.44	12.4	55.71	121.56	173.17
GAIF01033623.1	9.38	3.87	1.78	0	0	0
GAIF01033625.1	0	0.21	0	0	0	0.11
GAIF01033626.1	0	0	0	0	0.13	0
GAIF01033629.1	0	0.91	0.28	0	0.16	0
GAIF01033630.1	47.38	39.04	7.77	54.04	76.73	40.45
GAIF01033631.1	0.36	0	0	0	0	0
GAIF01033632.1	15.48	23.9	52.19	23.72	25.51	19.03
GAIF01033633.1	0	0	0	0.33	0	0
GAIF01033635.1	0.04	0	0	0.04	0.44	0.04
GAIF01033636.1	10.54	13.24	15.68	22.4	21.03	8.73
GAIF01033637.1	0	0	0	0.64	1	0
GAIF01033638.1	0.12	0	0	0.14	0.3	0
GAIF01033639.1	0.53	1.29	0.8	1.77	1.88	0.25
GAIF01033641.1	0.63	0	0	0	0	0
GAIF01033642.1	2.28	0.53	0.36	7	27.44	13.29
GAIF01033643.1	14.77	18.08	18.5	27.24	15.14	5.02
GAIF01033644.1	0	0.18	0.43	0	0	0.68
GAIF01033646.1	1973.88	1962.99	376.79	134.12	232.41	115.95
GAIF01033647.1	0.01	0	0	0	0	0
GAIF01033648.1	7.26	2.62	1.54	35.97	72.34	2.21
GAIF01033649.1	0.07	0	0	0	0	0
GAIF01033650.1	0.34	0	0.26	2.12	4.73	0.61
GAIF01033651.1	0	0	0	0	2.38	0
GAIF01033652.1	0.04	0	0	0.05	0.31	0.1

GAIF01033653.1	0	0	0	0.11	0.11	0
GAIF01033654.1	0	0	0.12	0	0	0
GAIF01033657.1	159.43	117.48	135.78	57.59	70.81	263.59
GAIF01033658.1	0	0.01	0.01	0.45	2.06	0.01
GAIF01033659.1	0	0	0	6.07	20.63	0
GAIF01033660.1	30.69	29.84	17.23	61.69	70.31	5.24
GAIF01033661.1	1	0.07	0.24	5.76	41.05	1.63
GAIF01033662.1	0	0	0.11	0	0.12	0
GAIF01033663.1	0.26	0.22	0	5.17	16.01	0.06
GAIF01033664.1	107.65	118.82	102.64	92.36	43.71	20.72
GAIF01033665.1	0.32	0.52	0.53	0.93	4.8	0.53
GAIF01033666.1	0	0	0	0.11	0	0
GAIF01033667.1	0	0.11	0	0	0.12	0
GAIF01033668.1	0	0	0	0	0.35	0
GAIF01033669.1	0.97	1.38	0.93	8.29	8.23	0.22
GAIF01033670.1	0.29	0.04	0	1.37	7.84	0.36
GAIF01033671.1	1	0.72	1.1	3.09	1.8	1.47
GAIF01033672.1	40.62	34.26	15.9	31.43	42.69	14.06
GAIF01033673.1	0.93	0.11	0.06	4.2	14.32	2.97
GAIF01033674.1	0.5	0.18	0	2.53	9.69	0.37
GAIF01033675.1	0.05	0	0	0	0.22	0
GAIF01033678.1	0.44	0.12	0	1.03	5.45	0.12
GAIF01033679.1	0.04	0.02	0	0.15	0.51	0.04
GAIF01033680.1	0.24	0.04	0.11	0.32	0.7	0.11
GAIF01033681.1	34.41	45.02	29.21	71.52	52.77	12.66
GAIF01033682.1	3.28	0.76	0.54	1.29	0	0
GAIF01033683.1	0	0	0	0	1.61	0
GAIF01033684.1	0	0	0	0.06	0	0
GAIF01033687.1	2.23	1.91	1.16	13.65	23.39	0.85
GAIF01033688.1	0.33	0.55	0.21	0.84	0.53	0.1
GAIF01033690.1	10.3	11.25	11.02	7.4	1.91	2.49
GAIF01033691.1	44.16	53.83	51.53	154.15	86.85	29.03
GAIF01033692.1	15.08	6.43	5.24	18.83	24.37	32.39
GAIF01033693.1	50.29	77.82	71.89	91.88	46.97	15.72
GAIF01033695.1	6.32	5.07	4.7	10.49	17.62	19.17
GAIF01033696.1	44.1	36.48	19.56	40.06	59.99	18.41
GAIF01033697.1	55.52	14.28	18.63	14.92	16.28	86.27
GAIF01033698.1	2.58	0.16	0.25	2.11	7.3	7.77
GAIF01033699.1	0	0	0.05	0	0	0
GAIF01033700.1	0	0	0	0	0.29	0
GAIF01033701.1	0	0	0	0	0.04	0
GAIF01033704.1	0	15.29	0	21.97	19.95	5.49
GAIF01033706.1	1.85	2.85	1.77	2.32	1.11	0.34
GAIF01033707.1	15.24	20.45	16.83	34.13	30.22	6.58

GAIF01033708.1	28.86	12.92	9.58	0	14.68	64.47
GAIF01033709.1	0.57	2.65	3.58	3.65	0.64	0
GAIF01033711.1	0.67	0.05	0.05	1.55	11.67	1.26
GAIF01033714.1	2.84	1.12	2.24	3.76	6.07	4.9
GAIF01033716.1	92.88	48.92	78.23	81.77	79.54	70.41
GAIF01033717.1	2	1.1	0.77	3.71	9.2	0.81
GAIF01033718.1	0	0	0	0	0.4	0
GAIF01033719.1	0	0.06	0.11	0	0	0
GAIF01033720.1	5.59	10.63	9.85	27.3	15.82	3.73
GAIF01033721.1	0.92	1.3	4.34	3.44	2.8	17.49
GAIF01033722.1	0.07	0	0	0	0	0
GAIF01033723.1	0.3	0	0	0.35	1.33	0
GAIF01033724.1	6.22	2.98	0.93	13.98	25.61	2.26
GAIF01033727.1	793	1773.63	2224.44	343.1	720.9	723.85
GAIF01033728.1	0.53	0.11	0.05	0.24	0.1	0.02
GAIF01033729.1	0.21	0	0	1.64	7.34	0.06
GAIF01033730.1	11.35	18.34	5.33	48.37	34.72	6.07
GAIF01033731.1	4.37	2.14	1.18	7.78	7.79	1.49
GAIF01033732.1	0	0	0	0	75.83	0
GAIF01033733.1	24.67	22.39	17.9	56.51	22.7	10.4
GAIF01033734.1	0.1	0.63	0.46	1.28	8.04	0.69
GAIF01033735.1	0.08	0	0	0	0.27	0
GAIF01033736.1	0	0	0	0	0.33	0
GAIF01033737.1	0	0.06	0	0	0	0
GAIF01033738.1	0	0	0	1.64	11.29	0
GAIF01033740.1	0.71	0.77	0	4.92	2.55	0
GAIF01033741.1	1.21	0.12	0	3.7	9.91	0
GAIF01033742.1	62.09	60.6	32.8	19.92	17.29	6.02
GAIF01033743.1	0	0	0	0.01	0	0.03
GAIF01033744.1	14.28	20.89	18	32.65	0	8.27
GAIF01033745.1	58.74	108.27	132.67	131.73	136.43	59.01
GAIF01033747.1	0	0	0	6.89	0	0.35
GAIF01033748.1	1.14	0.38	0	3.68	14.72	2.62
GAIF01033750.1	0.18	0	0	0	0	0
GAIF01033751.1	0	0	0	0.35	0.18	0
GAIF01033753.1	0	0	0	0	0.06	0
GAIF01033754.1	0	0	0	0.06	0.12	0
GAIF01033755.1	162.55	181.35	177.49	8.99	11.6	29.51
GAIF01033756.1	0.27	0	0	1.42	7.06	0
GAIF01033759.1	2.46	2.03	1	0	0.27	0
GAIF01033760.1	21.38	8.37	15.02	10.24	6.65	6.67
GAIF01033761.1	0.17	0	0	0	0.07	0
GAIF01033765.1	0	0	0	0.04	0.31	0
GAIF01033766.1	18.35	10.85	17.79	7.87	5.05	7.85

GAIF01033767.1	0.14	0	0	0.09	0.76	0
GAIF01033769.1	0	0	0	0	1.59	0.15
GAIF01033770.1	0	0.1	0	0.86	0.34	0.21
GAIF01033771.1	0.07	0	0	0.12	0.86	0
GAIF01033772.1	0.14	0.08	0	0	0.08	0
GAIF01033774.1	10.95	12.8	6.31	27.17	34.34	2.34
GAIF01033775.1	0	2.24	0	1.5	0	0
GAIF01033776.1	0	0	0	0.07	0	0
GAIF01033777.1	0	0	0	0.48	1.88	0
GAIF01033778.1	6.05	5.71	0.26	56.96	8.33	0
GAIF01033779.1	0	0	0.14	0	0	0
GAIF01033780.1	0.14	0.2	0	0.01	0	0.03
GAIF01033782.1	16.62	19.32	13.53	29.27	18.61	3.47
GAIF01033784.1	0.49	0.04	0.01	0.28	1.59	0.36
GAIF01033785.1	0	0.15	0.15	0	0	0
GAIF01033786.1	16.18	10.76	19.81	28.24	14.14	45.42
GAIF01033787.1	7.97	3	6.87	11.95	12.16	14.86
GAIF01033788.1	30.9	16.12	29.71	21.31	14.7	37.9
GAIF01033789.1	0	0	0	0.69	0.31	0.56
GAIF01033790.1	42.11	16.86	13.85	47.61	46.65	16.31
GAIF01033791.1	0	0	0.08	2.21	5.06	0
GAIF01033792.1	24.91	59	54.43	14.39	24.61	13.02
GAIF01033793.1	0	0	0.07	0.07	0	0
GAIF01033794.1	0.19	0	0	0	0.11	0
GAIF01033795.1	0.09	0.19	0	0.21	0.43	0
GAIF01033796.1	0	0	0	0	0	0.09
GAIF01033797.1	0	0.09	0	0	0	0
GAIF01033798.1	2135.33	1044.77	2153.22	594.28	98.83	220.81
GAIF01033799.1	0	0	0	0	4.06	0.03
GAIF01033800.1	7.53	6.62	2.29	10.04	17.53	1.94
GAIF01033801.1	0.6	0.39	1.08	1.16	0.8	1.01
GAIF01033802.1	0	0	0	0.3	1.78	0
GAIF01033804.1	0.15	0.28	0.05	0.21	0.21	0
GAIF01033805.1	0	0	0	0	0.18	0
GAIF01033807.1	2.28	519.04	1123.64	12.9	86.88	88.55
GAIF01033811.1	0.88	0.38	0.1	0.97	3.84	0.34
GAIF01033812.1	105.97	137.1	85.99	261.19	207.29	23.53
GAIF01033814.1	0.09	0.11	0.02	0.51	5.63	0.23
GAIF01033815.1	2.17	0.99	0	1.04	5.22	1.59
GAIF01033816.1	0.07	0.28	0.05	0.66	5.47	0.82
GAIF01033817.1	1.38	0.75	0.51	6.95	12.66	0.12
GAIF01033819.1	0	0	0	0	0.17	0
GAIF01033820.1	3.03	0	0.42	0	0	0
GAIF01033821.1	0	0.07	0	0.98	1.26	0

GAIF01033823.1	0.06	0	0	0.13	0.11	0.06
GAIF01033825.1	12.06	3.18	3.15	16.77	27.07	17.17
GAIF01033826.1	13.12	13.13	13.16	13.08	5.21	3.51
GAIF01033829.1	0.22	0.23	0.12	0	0	0
GAIF01033830.1	2.59	0.52	0.31	4.93	12.12	0.69
GAIF01033832.1	30.67	48.69	28.69	7.12	2.8	0.14
GAIF01033833.1	8.25	11.1	11.73	25.35	10.21	3.99
GAIF01033834.1	0.09	0.13	0	0.07	0.11	0.1
GAIF01033835.1	0.25	0.14	0	0.87	6.03	0
GAIF01033836.1	0	0	0.06	0	0	0
GAIF01033838.1	0.47	0	0.35	0.55	3.22	0.52
GAIF01033840.1	0	0	0	0	0.96	0
GAIF01033841.1	1.5	0	0.88	1.54	2.14	2.2
GAIF01033842.1	0.23	0.09	0	0	0	0
GAIF01033843.1	1.56	0.66	1.71	0.39	0.73	0
GAIF01033844.1	5.1	6.87	4.49	5.17	3.03	4.38
GAIF01033847.1	35.25	28.91	36.04	95.9	60.63	66.54
GAIF01033848.1	0.29	0.56	0.48	0.51	0.8	0
GAIF01033851.1	0	0.07	0	0.08	0	0
GAIF01033852.1	614.29	239.84	289.66	1167.43	1547.67	8387.38
GAIF01033853.1	0.1	0	0.23	0.48	1.37	0
GAIF01033854.1	0.16	0	0	0.07	0	0
GAIF01033855.1	0	0	0.05	0.05	0	0
GAIF01033856.1	21.17	31.18	45.75	11.09	13	8.54
GAIF01033857.1	12.68	25.46	25.33	36.95	28.43	6.93
GAIF01033858.1	0	0	0	0	0.03	0
GAIF01033859.1	0	0	0	0.03	0.4	0
GAIF01033860.1	3.2	1.27	0.16	7.94	6.47	1.92
GAIF01033861.1	2.28	0.88	2.36	11.69	6.14	14.8
GAIF01033862.1	2.6	2.21	2.6	3.67	0	0
GAIF01033864.1	20.28	36.89	23.89	23.48	18.69	4.24
GAIF01033865.1	0.22	0.09	0.09	0.47	1.88	0.09
GAIF01033866.1	12.98	5.12	3.92	2.64	2.44	2.15
GAIF01033867.1	18.41	24.19	12.11	100.32	84.92	6.33
GAIF01033868.1	0.04	0.04	0	0.23	1.74	0.08
GAIF01033869.1	0.06	0.07	0	0.07	0	0
GAIF01033871.1	0.13	0.04	0	0.38	1.08	0
GAIF01033872.1	5.43	1.81	0	5.88	5.78	1.22
GAIF01033873.1	0.05	0.1	0.27	0.35	0.37	0
GAIF01033874.1	0.03	0	0	0	0.04	0
GAIF01033875.1	0.77	0.95	1.24	3	4.14	1.42
GAIF01033878.1	1.15	0.29	0.2	3.5	12.71	0.29
GAIF01033879.1	0	0	0	0.05	0.11	0
GAIF01033880.1	35.84	21.19	11.19	41.49	30.14	10.28

GAIF01033881.1	0	0	0	0	0.35	0
GAIF01033882.1	0	0	0	0.12	1.03	0
GAIF01033884.1	0.08	0.17	0	0	0.09	0
GAIF01033885.1	0.21	1.14	0.12	2.79	4.96	0.12
GAIF01033886.1	0	0.04	0	0.1	0.35	0
GAIF01033887.1	2.54	0.12	0.06	8.08	40.66	4.24
GAIF01033888.1	0.09	0	0	0.45	1.86	0
GAIF01033889.1	0	1.06	0	0	1.13	5.3
GAIF01033891.1	0	0	0.23	0	0.11	0
GAIF01033892.1	0.37	0.2	0	0	0	0
GAIF01033893.1	9.56	3.68	3.07	1.37	2.3	1.3
GAIF01033895.1	190.58	43.55	75.3	160.01	36.95	45.67
GAIF01033901.1	8.05	19.59	1.12	3.52	4.42	0
GAIF01033902.1	18.48	15.91	15.44	26.32	21.55	7.12
GAIF01033903.1	0	1.59	1.6	0.09	0.1	0
GAIF01033904.1	17.24	19.09	14.75	38.22	34.31	8.43
GAIF01033905.1	3.51	2.92	2.87	9.01	8.08	6.58
GAIF01033906.1	0	0.23	0.19	0	0	0
GAIF01033907.1	0	0.21	0	0.51	1.35	0
GAIF01033911.1	0	0	0.63	0	0	0.63
GAIF01033912.1	4.04	13.17	4.43	14.02	9.7	8.89
GAIF01033913.1	0.15	0.11	0	1.46	3.39	0.17
GAIF01033914.1	4.43	2.85	2.29	12.07	14.89	4.01
GAIF01033916.1	0.1	0.33	0.55	0.12	0.24	0.11
GAIF01033917.1	0	0	0	0	0.73	0
GAIF01033918.1	9.74	17.66	30.84	20.84	15.85	8.42
GAIF01033919.1	3.59	3.95	3.44	13.26	10.87	2.16
GAIF01033923.1	0.14	0.1	0.1	0.54	1.39	0.15
GAIF01033924.1	0.13	0	0.05	0.25	2.24	0.23
GAIF01033925.1	0	2.12	1.46	0.16	0	0.08
GAIF01033926.1	9.05	7.69	4.92	8.27	8.9	0.55
GAIF01033927.1	57.81	100.59	53	83.43	76.55	17.04
GAIF01033928.1	0	0	0.09	0.05	0.85	0
GAIF01033929.1	0	0	0	0	0.14	0
GAIF01033930.1	12.92	13.24	25.86	35.3	40.87	47.82
GAIF01033931.1	8.44	5.46	7.81	19.6	17.52	10.03
GAIF01033932.1	0.05	0	0	0.19	0.06	0
GAIF01033933.1	0	0	0	0	0.11	0.1
GAIF01033934.1	8.03	12.71	12.51	39.52	19.9	7
GAIF01033935.1	30.3	23.62	17.12	62.74	60.34	9.19
GAIF01033936.1	3.69	2.75	0	5.95	14.26	0.91
GAIF01033937.1	18.5	20.02	10.2	35.75	31.77	5.22
GAIF01033938.1	84.94	24.95	285.35	1.83	0	39.94
GAIF01033939.1	0	0	0	0	0	0.5

GAIF01033940.1	0.08	0	0	0.39	1.7	0
GAIF01033943.1	0	0	0	0.04	0.07	0
GAIF01033944.1	0	0	0	0	0.06	0.35
GAIF01033945.1	0.41	0.33	0.22	0	0	0
GAIF01033946.1	5.68	6.25	2.76	18.05	13.6	1.14
GAIF01033947.1	0.1	0.05	0	0	0.24	0
GAIF01033948.1	16.78	9.59	9.77	46.88	101.96	86.35
GAIF01033949.1	14.2	10.8	6.53	62.02	79.16	4.15
GAIF01033950.1	19.34	4.97	5.35	20.66	10.5	4.67
GAIF01033951.1	0.9	0	0.41	0	0.13	0
GAIF01033954.1	0.17	0	0	0	0.79	0
GAIF01033957.1	1.05	3.07	2.93	3.34	6.03	0.29
GAIF01033958.1	2.1	4.69	1.47	5.59	8.06	0.55
GAIF01033959.1	0.21	0.23	0	0.25	1.78	0
GAIF01033960.1	0.27	0.02	0	0.11	0.35	0
GAIF01033962.1	13.59	8.71	14.26	10.46	4.77	4.08
GAIF01033963.1	5.56	2.13	1.33	7.19	13.93	4.48
GAIF01033964.1	6.86	13.36	3.05	28.99	34.33	1.43
GAIF01033965.1	0.3	0	0	0.12	0	0.11
GAIF01033966.1	786.77	454.1	236.38	91.67	91.55	142.27
GAIF01033967.1	0	0	0	0.62	2.47	0.4
GAIF01033970.1	10.33	9.46	10.11	23.08	18.32	10.86
GAIF01033971.1	537.64	573.5	1732.1	481.2	326.07	1162.68
GAIF01033973.1	0	0	0	0.04	0.16	0
GAIF01033974.1	0	1.14	0	0	0.75	0
GAIF01033975.1	0.72	0.44	0	1.23	3.71	2.43
GAIF01033976.1	61.55	73.89	135.78	70.7	55.46	172.41
GAIF01033978.1	135.89	196.85	424.51	111.01	84.87	159.86
GAIF01033979.1	0	19.01	0	20.29	63.07	0
GAIF01033980.1	0	0.05	0	0	0	0
GAIF01033981.1	0	0.05	0	0	0.05	0
GAIF01033982.1	30.93	40.76	42.89	37.67	33.51	30.5
GAIF01033985.1	66.38	75.67	97.91	74.69	38.39	30.34
GAIF01033986.1	18.8	14.68	15.02	27.69	20.53	3.24
GAIF01033987.1	0.34	0	0	0.26	2.45	0
GAIF01033988.1	0.95	0.13	0.14	0.45	0.4	0.22
GAIF01033990.1	0	0	0	0.15	0.02	0
GAIF01033993.1	1.28	0.04	0.04	0.42	0.32	0.1
GAIF01033994.1	24.91	34.05	35.98	40.46	44.05	11.54
GAIF01033995.1	14.67	13.55	9.56	10.15	6.5	5.26
GAIF01033997.1	5.91	3.84	2.54	1.13	0.7	0
GAIF01033998.1	9.91	3.85	3.42	46.33	19.92	7.33
GAIF01033999.1	70.32	69.38	53.71	102.69	55.46	22.53
GAIF01034000.1	0	0.05	0	0	0	0

GAIF01034001.1	3.18	3.54	6.01	1.46	0	3.57
GAIF01034002.1	0	0	0	0	0.08	0
GAIF01034004.1	1.18	0.64	0.24	6.16	18.88	0.08
GAIF01034005.1	30.16	21.15	20.85	45.99	12.92	7.61
GAIF01034006.1	43.94	32.06	16.65	79.34	95.08	18.83
GAIF01034007.1	0.07	0.08	0.13	2.25	8.58	0.26
GAIF01034008.1	57.38	88.59	152.09	63.29	49.5	145.44
GAIF01034010.1	0	0.25	0	0	0	0
GAIF01034011.1	0.27	0.36	0	0	0	0
GAIF01034012.1	0	0.16	0	0	0.49	0
GAIF01034013.1	0.33	0.89	0.72	1.14	0.39	1.08
GAIF01034015.1	0	0	0	0	1.16	0
GAIF01034017.1	0	0.18	0	0	0	0
GAIF01034020.1	0.04	0	0	0.05	1.5	0
GAIF01034021.1	4.36	12.31	7.31	15.72	24.84	2.78
GAIF01034022.1	0.1	0	0	0.47	2.38	0
GAIF01034023.1	194.23	107.33	52.13	17.41	14.67	4.82
GAIF01034024.1	0	0	0	0.11	0	0
GAIF01034026.1	0.68	1.11	0.25	2.64	6.15	4.76
GAIF01034029.1	0.18	0.07	0	1.13	6.07	0
GAIF01034030.1	0	0	0.08	0	0.07	0.07
GAIF01034032.1	0.42	0	0	3.15	12.43	0.12
GAIF01034034.1	0	0	0	0	0.36	0
GAIF01034035.1	0.18	0	0.1	0.11	0.11	0.1
GAIF01034036.1	4.06	1.13	0.53	20.69	76.9	60.33
GAIF01034037.1	0	0	0	0.34	0.65	0
GAIF01034038.1	16.74	16.01	19.58	91.42	127.81	14.19
GAIF01034039.1	0	0	0	0	0.05	0
GAIF01034040.1	0	0	0	0.04	0.18	0
GAIF01034041.1	12.01	11.63	5.76	34.93	54.13	3.58
GAIF01034042.1	10.25	9.72	21.12	5.48	1.41	3.57
GAIF01034043.1	0	1.06	0	2.7	0.69	0.24
GAIF01034044.1	7.71	13.65	8.83	8.49	5.73	0.88
GAIF01034045.1	0.08	0.09	0	0.39	1.6	0
GAIF01034047.1	0	0	0	0.97	3.56	1.05
GAIF01034048.1	0	0.13	0	0	0	0
GAIF01034049.1	0	0	0	0	0.14	0.06
GAIF01034050.1	0	0	0	1.07	2.57	0
GAIF01034053.1	0.16	0	0.09	0.09	0	0
GAIF01034054.1	9.61	3.01	1.01	12.68	13.14	0.44
GAIF01034055.1	5.78	8.46	4.39	6.53	4.43	1.04
GAIF01034056.1	0.08	0	0	0	0	0
GAIF01034057.1	0	0	0	0	2.1	0
GAIF01034058.1	0.67	0.18	0	0.97	0.61	0

GAIF01034059.1	7.8	12.39	18.17	13.69	8.2	8.83
GAIF01034060.1	0.89	0.29	4.07	11.4	0.05	1.33
GAIF01034062.1	19.34	20.72	25.58	52.28	52.42	28.58
GAIF01034064.1	209.14	145.3	92.69	226.26	358.23	128.53
GAIF01034066.1	1.44	0.26	0.13	4.05	16	0.9
GAIF01034067.1	0	0	0	0	0.36	0
GAIF01034068.1	0.14	0	0	0.65	2.18	0.31
GAIF01034071.1	0	0.13	0	0	0.01	0
GAIF01034072.1	1.78	1.6	0.23	0.24	0.13	0
GAIF01034073.1	11.36	19.25	33.87	16.04	12.85	12.88
GAIF01034074.1	18.61	32.84	35.8	12.86	17.05	2.48
GAIF01034075.1	0.43	0.16	0	5.8	21.13	0.31
GAIF01034076.1	0	0	0	0	0.04	0
GAIF01034077.1	33.34	43.55	55.23	46.34	45.97	29.74
GAIF01034078.1	2.17	0.17	0.26	0.09	0.37	0.68
GAIF01034079.1	0	0.02	0	0	0.15	0
GAIF01034084.1	1.72	0	0.47	8.33	18.98	0.32
GAIF01034085.1	1.74	0.23	0.35	0.43	0.98	0.1
GAIF01034086.1	12.18	11.18	10.07	40.12	26.1	3.9
GAIF01034087.1	0	0.16	0	2.16	8.6	0.16
GAIF01034088.1	40.34	46.04	7.51	54.58	41.84	2.51
GAIF01034089.1	4.44	5.06	5.99	12.97	13.51	17.51
GAIF01034091.1	27.41	46.99	15.55	23.09	42.82	3.35
GAIF01034092.1	2.42	0.07	0.31	0.57	4.09	2.65
GAIF01034094.1	0	0	0	0.05	0.23	0
GAIF01034099.1	45	14.38	9.14	41.91	41.06	33.36
GAIF01034100.1	0	0	0	0	0.77	0
GAIF01034101.1	0.25	0.01	0	0.47	1.96	0
GAIF01034102.1	0	0	0	0	0.38	0
GAIF01034103.1	0	0	0	0	0.1	0
GAIF01034104.1	0.64	0.1	0	3.62	20.08	0.05
GAIF01034105.1	3.21	1.39	2.41	4.05	6.5	3.1
GAIF01034106.1	0.03	0	0	0.06	0	0
GAIF01034107.1	0.78	0	0	0.23	2.57	0
GAIF01034109.1	9.24	3.28	3.12	13.68	5.41	1.24
GAIF01034110.1	28.69	51.51	30.91	34.09	22.79	5.67
GAIF01034111.1	1.21	2.49	0.05	1.02	2.21	0
GAIF01034112.1	5.38	11.59	13.02	1.82	1.66	2.12
GAIF01034113.1	19.41	37.53	32.94	34.68	28.91	14.27
GAIF01034114.1	0.53	0	0	1.08	18.19	0
GAIF01034117.1	0	0.05	0	0.03	0.03	0
GAIF01034119.1	105.71	71.45	31.89	36.91	14.63	2.68
GAIF01034120.1	0.17	0	0.09	0.1	0.81	0.18
GAIF01034121.1	17.4	23.4	18.67	22.34	21.54	9.43

GAIF01034123.1	0.53	0.11	0.11	1.52	1.28	0.48
GAIF01034124.1	0.03	0	0.03	0.07	0.01	0
GAIF01034125.1	0.14	0.1	0	0.04	0.43	0
GAIF01034126.1	0.19	0	0	0	0.12	0
GAIF01034128.1	0	0.53	0	0.13	0	0.25
GAIF01034129.1	0.08	0	0.36	0	0	0
GAIF01034130.1	0.11	0.05	0	0	0.58	0
GAIF01034131.1	0	0.1	0	0	0	0
GAIF01034133.1	1.5	1.18	1.1	6.59	1.3	3.31
GAIF01034135.1	23.66	30.24	27.87	22.68	23.53	6.22
GAIF01034136.1	1.03	1.58	1.3	0	0	0.02
GAIF01034137.1	0	0	0.2	0	0.33	0
GAIF01034138.1	0	0	0	0	0.35	0
GAIF01034139.1	0	0	0	102.42	54.7	18.41
GAIF01034140.1	179.11	195.59	162.83	431.52	223.82	132.61
GAIF01034141.1	0.15	0	0	0	0.18	0
GAIF01034143.1	0.42	0.15	0	1.61	4.5	0.31
GAIF01034144.1	0.8	0.28	0.6	2.23	3.01	1.84
GAIF01034145.1	0	0	0	0	0.08	0
GAIF01034146.1	0.05	0	0	0.12	0.69	0
GAIF01034147.1	0.12	0	0	0	0.07	0
GAIF01034148.1	9.96	4.76	3.35	7.12	13.59	6.79
GAIF01034149.1	11.47	9.28	16.41	16.8	12.95	11.09
GAIF01034150.1	1.44	0.87	0.79	0.24	0.07	0
GAIF01034152.1	0	0	0	0.18	0.75	0
GAIF01034153.1	0.16	0.14	0	0	0	0
GAIF01034154.1	0.06	0	0	0	0	0
GAIF01034158.1	12.47	1.99	6.06	10.79	7.36	11.57
GAIF01034159.1	7.07	2.97	1.49	19.37	30.28	0.62
GAIF01034160.1	8.45	14.47	13.03	38.39	21.59	5.68
GAIF01034161.1	649.44	695.61	1557.09	530.06	302.38	934.18
GAIF01034162.1	5.91	0	1.2	3.83	2.5	19.63
GAIF01034163.1	0.13	0	0	0.6	2.82	0
GAIF01034164.1	0.53	0.63	0.27	3.71	10.1	0.08
GAIF01034165.1	23.48	35.5	12.97	74.07	110.25	15
GAIF01034166.1	27.37	19.79	34.93	26.44	9.69	8.42
GAIF01034168.1	56.07	46.94	52.93	80.46	53.7	59.39
GAIF01034171.1	12.19	10.64	12.48	10.72	14.33	12.41
GAIF01034172.1	834.27	948.89	1711.6	0	1.52	0.7
GAIF01034173.1	5.39	3.05	1.91	0	6.26	4.26
GAIF01034174.1	8.01	16.98	4.4	46.1	41.26	1.58
GAIF01034175.1	0.02	0.25	0.39	0.51	0.07	0.13
GAIF01034176.1	0	0.15	0.12	0.34	0.57	0.07
GAIF01034178.1	0	0	0	0.2	0.82	0

GAIF01034179.1	0	0	0.04	0.07	0	0
GAIF01034180.1	0.09	0	0	0.81	2.68	0.14
GAIF01034181.1	0.16	0.83	0.61	0.41	1.2	0.72
GAIF01034182.1	0.8	0.88	0.25	1.4	0	0
GAIF01034183.1	0.13	0.09	0	0.83	3.4	1.63
GAIF01034185.1	13.21	0.48	1.25	21.12	26.72	13.5
GAIF01034186.1	2.96	2.28	0.5	7.83	21.28	1.15
GAIF01034187.1	0	0	0	0	0.04	0
GAIF01034188.1	99.29	82.35	104.83	5.88	2.64	7.4
GAIF01034189.1	1.41	3.39	1.15	3.2	3.38	0.08
GAIF01034190.1	0.06	0	0	0.07	0.21	0
GAIF01034191.1	64.48	42.01	45.64	78.19	39.07	121.15
GAIF01034193.1	0	0.44	0	0	0	0.28
GAIF01034194.1	1.67	1.81	0.87	2.65	5.17	1.26
GAIF01034195.1	0	0	0	0	0.53	0
GAIF01034197.1	2.01	0.95	0.22	4.75	9.59	13.74
GAIF01034198.1	0.04	0.13	0	0	0	0
GAIF01034199.1	8.95	1.82	0.44	23.85	48.04	15.21
GAIF01034201.1	25	24.15	14.57	16.21	19.82	13.08
GAIF01034202.1	1.62	1.76	0.15	4.54	5.68	0
GAIF01034203.1	0	0	0	4.82	0.41	2.91
GAIF01034204.1	4.72	5.69	4.2	5.92	3.48	0.47
GAIF01034205.1	3.64	2.89	2.92	11.93	6.56	2.33
GAIF01034206.1	0	0.12	0.25	0	0	0.12
GAIF01034207.1	0	0	0	0	0	0.15
GAIF01034208.1	0.2	0.29	0.07	1.61	1.35	1.16
GAIF01034209.1	0.54	0.98	0.4	0.63	0	0.2
GAIF01034210.1	12.77	6.08	3.06	3.26	2.49	3.76
GAIF01034213.1	0.07	0.04	0.16	0.34	0.22	0.2
GAIF01034214.1	0.13	0.15	0.07	0.39	3.73	0.07
GAIF01034216.1	72.33	28.93	34.86	49.66	16.09	44.95
GAIF01034217.1	0	0	0	0.09	0.56	0
GAIF01034218.1	0	0.19	0	1.71	3.19	0
GAIF01034219.1	0.68	0.48	0.16	0.86	0.96	0.2
GAIF01034224.1	0.45	0	0	0.17	0.16	0
GAIF01034226.1	1.03	1.67	1.44	4.83	7.13	0.73
GAIF01034229.1	3.5	1.65	1.89	1.4	2.39	0.52
GAIF01034230.1	28.03	22.06	36.7	33.81	16.03	34.9
GAIF01034231.1	0.24	0.2	0.05	1.69	3.74	0.16
GAIF01034233.1	250.07	186.66	161.3	1527.37	1486.98	189.74
GAIF01034234.1	1.84	0.17	0.15	0.36	0	0.38
GAIF01034236.1	0	0	0.05	0	0	0
GAIF01034238.1	0	0	0	0	0.1	0
GAIF01034241.1	0.29	0.53	1.14	0.11	0.57	0

GAIF01034242.1	0.46	0.09	0	0.97	1.7	4.08
GAIF01034243.1	0.38	0.19	0.06	2.47	6.97	0.1
GAIF01034244.1	0	0.14	0	0	0	0
GAIF01034246.1	0.03	0	0	0	0	0
GAIF01034247.1	0	0.17	0.08	0	0.51	0
GAIF01034249.1	0	0	0	0	1.03	0
GAIF01034250.1	0	0	0	0	0.07	0
GAIF01034251.1	0	0.37	0.07	0	0	0
GAIF01034252.1	1.37	0.75	0.48	0.65	0.67	0.34
GAIF01034255.1	142.53	59.28	122.42	43.85	39.43	406.37
GAIF01034256.1	12.42	20.71	23.16	12.03	2.51	8.35
GAIF01034257.1	4.59	5.38	2.62	1.42	0.89	0.15
GAIF01034259.1	0	0	0	0	0	0.02
GAIF01034260.1	0	0	0	0	0	0.09
GAIF01034261.1	1.05	0.76	0.89	0.94	4.47	0.51
GAIF01034262.1	5.92	1.97	2.2	19.3	30.52	10.58
GAIF01034263.1	34.97	15.14	110.3	24.97	19.03	847.75
GAIF01034264.1	66.44	37.64	31.56	74.76	72.92	94.62
GAIF01034267.1	0.51	0.14	0.18	1.04	2.23	0.13
GAIF01034268.1	0.18	0	0	1.3	0.28	1.42
GAIF01034269.1	0.02	0	0	0.02	0	0
GAIF01034270.1	5.49	1.17	1.2	8.03	2.64	6.12
GAIF01034271.1	0.46	0.38	0	0.98	3.41	0
GAIF01034272.1	0	0	0	0	0.05	0
GAIF01034273.1	4.14	0.43	0.11	6.3	35.36	11.42
GAIF01034274.1	52.92	41.67	29	72.44	62.44	28.08
GAIF01034275.1	0.51	0.15	0.06	0.08	0.58	0
GAIF01034276.1	0	0	0.1	0	1.94	0
GAIF01034277.1	0.13	0	0	0	0.47	0
GAIF01034278.1	2.92	6.69	2.37	1.32	0.8	0
GAIF01034279.1	0	0.03	0	0.14	1.98	0
GAIF01034281.1	0	0.33	0	0.47	2.07	0
GAIF01034282.1	30.82	33.59	18.41	7.33	3.02	0.3
GAIF01034287.1	2.91	192.16	124.76	19.08	115.94	45.65
GAIF01034288.1	0.05	0.06	0.03	0	0.33	0.15
GAIF01034290.1	0.21	0	0	0.71	1.25	0.13
GAIF01034291.1	7.01	5.58	4.39	13.87	14.43	6.38
GAIF01034292.1	0	0	0	0.03	0	0
GAIF01034293.1	9.59	4.46	3.68	15.57	27.9	16.74
GAIF01034294.1	6.87	7.29	4.74	30.02	34.6	2.13
GAIF01034296.1	2.27	3.36	0.31	0.99	2.73	0.03
GAIF01034297.1	0.02	0	0	0.04	0.26	0
GAIF01034298.1	0	0	0	0.74	5.35	0
GAIF01034299.1	61.58	98.57	17.44	89.39	289.08	51.28

GAIF01034300.1	3.56	0.97	1.46	1.6	2.97	0.81
GAIF01034301.1	0.46	0.17	0.17	3.76	13.16	0
GAIF01034302.1	16.12	6.38	4.58	31.52	43.51	7.54
GAIF01034303.1	0	0	0	0	0.08	0
GAIF01034304.1	0	0	0	0.24	0	0.3
GAIF01034306.1	118.87	88.65	41.5	379.27	290.56	35.53
GAIF01034310.1	0	0.03	0	0	0	0
GAIF01034311.1	15.73	15.71	16.53	58.94	47.54	19.52
GAIF01034312.1	1.49	1.25	0.25	0.67	1.24	0
GAIF01034313.1	52.93	69.29	40.38	7.23	2.64	0.87
GAIF01034314.1	0.32	0.52	0	2.03	7.25	0.17
GAIF01034315.1	0.86	1.21	0.27	0.72	0.15	0
GAIF01034316.1	0.52	1.18	0.18	6.93	13.25	0.18
GAIF01034317.1	0.56	0	0	2.08	4.45	2.97
GAIF01034318.1	0.16	0.09	0	1.24	1.58	5.44
GAIF01034319.1	0.56	0.61	2.77	1.63	0.67	0.31
GAIF01034323.1	3.63	12.38	6.08	1.17	0.73	0.33
GAIF01034325.1	11.36	11.65	8.23	41.28	53.6	7.91
GAIF01034326.1	13.8	16.38	17.08	31.07	37.33	10.43
GAIF01034327.1	0.26	0	0	0	0	0
GAIF01034328.1	4.97	5.03	0.62	15.79	17.78	0.05
GAIF01034329.1	616.08	336.68	448.84	406.74	373.95	2285.78
GAIF01034330.1	0.12	0	0	0	1.5	0
GAIF01034331.1	1.43	1.05	1.73	3.61	5.15	2.53
GAIF01034333.1	0.08	0	0	0	0.09	0.09
GAIF01034334.1	0	0	0	0	0.06	0
GAIF01034335.1	111.85	91.99	51.95	165.51	179.44	117.22
GAIF01034337.1	0.12	0.13	0	2.23	11.68	0.66
GAIF01034339.1	21.82	10.2	17.09	10.04	9.28	2.13
GAIF01034341.1	0	0.01	0	0	1.3	1.11
GAIF01034342.1	0	0.06	0	0.06	0.12	0
GAIF01034343.1	0.92	0.24	1.07	1.49	0.95	1.53
GAIF01034344.1	0.26	0.1	0	0.41	1.48	0
GAIF01034345.1	0	0.08	0	0.09	0.56	0
GAIF01034347.1	0.12	0	0	0	0	0
GAIF01034349.1	0.14	0.15	0.23	0.12	1.25	0
GAIF01034350.1	0.4	0	0	0.92	2.44	2.5
GAIF01034355.1	0	0.21	0	0.55	2.1	0
GAIF01034356.1	0.1	0.19	0	0.03	0.06	0
GAIF01034357.1	0	0	0	0.3	0	0
GAIF01034358.1	25.74	29.85	20.19	41.05	42.54	18.96
GAIF01034359.1	47.3	26.15	34.77	106.59	166.48	291.83
GAIF01034362.1	6.66	2.75	1.51	0	0.14	0
GAIF01034364.1	0	0	0	0.19	0	0

GAIF01034365.1	0	0	0	0.06	0	0
GAIF01034366.1	0	0.11	0	0.06	0	0
GAIF01034367.1	18.39	11.68	2.45	54.86	45.93	0.45
GAIF01034368.1	57.86	54.22	105.71	60.74	35.47	32.76
GAIF01034369.1	0.1	0	0	0.11	0.42	0
GAIF01034370.1	2.75	5.36	5.13	0	0	0
GAIF01034371.1	0	2.45	4.66	0	0.1	0.18
GAIF01034372.1	4.08	1.98	1.12	13.69	32.16	7.71
GAIF01034374.1	0	0	0	0	0.13	0.04
GAIF01034375.1	0.07	0.16	0	0	0.53	0
GAIF01034376.1	13.21	7.73	8.49	24.67	33.19	8.74
GAIF01034377.1	3.41	6.17	1.29	12.49	23.71	1.02
GAIF01034379.1	29.36	24.55	20.14	33.33	29.55	69.89
GAIF01034380.1	9.88	9.43	3.59	15.27	16.65	0.7
GAIF01034381.1	133.37	83.42	62.14	7.68	5.22	14.88
GAIF01034382.1	1.65	1.02	0.21	2.11	1.32	0.38
GAIF01034384.1	0.09	0	0	0.9	0.21	0
GAIF01034385.1	2.26	2.09	0.8	0.71	0.34	0
GAIF01034386.1	4.9	1.51	0.46	3.43	2.88	0.16
GAIF01034387.1	0	0	0	0	0.81	0
GAIF01034388.1	0.18	0	0	0.26	1.31	0
GAIF01034390.1	0.03	0	0	0.38	0	0
GAIF01034391.1	0.11	0	0	0	0	0
GAIF01034392.1	37.07	62.18	26	71.51	66.45	5.99
GAIF01034393.1	0.25	0.14	0	0.29	0	0
GAIF01034394.1	0	0	0	0	0.05	0
GAIF01034395.1	293.65	431.32	170.03	279.93	314.04	45.69
GAIF01034396.1	0	0	0.07	0.36	1.35	0.28
GAIF01034397.1	0.08	0	0	0.03	0	0
GAIF01034398.1	10.59	5.62	7.91	19.24	9.59	11.45
GAIF01034400.1	1.38	1.67	0.28	1.19	2.4	0.11
GAIF01034401.1	0.03	0	0	0	0.04	0
GAIF01034402.1	175.12	186.55	68.29	176.43	260.94	39.95
GAIF01034403.1	0	0.82	0.25	0.09	0.09	0.5
GAIF01034404.1	14.76	13.98	12.77	32.02	18.73	8.13
GAIF01034405.1	0.09	0	0	0	0	0
GAIF01034406.1	0	0	0	0	0.03	0
GAIF01034407.1	0.23	0.25	0.05	0.47	0.43	0.05
GAIF01034408.1	61.07	63.27	47.91	40.69	25.11	6.25
GAIF01034409.1	22.74	14.73	26.22	16.72	7.98	13.47
GAIF01034412.1	28.45	37.51	26.63	62.15	59.57	6.91
GAIF01034413.1	35.44	21.13	9.04	16.94	25.09	45.22
GAIF01034415.1	5.58	0	0	180.02	427.35	0.02
GAIF01034416.1	0	0	0	0.44	0.22	2.37

GAIF01034418.1	13.48	3.54	2.41	19.95	17.76	3.51
GAIF01034419.1	75.27	44.01	32.89	133.7	148.7	14.82
GAIF01034420.1	7.88	10.18	7.72	42.02	45.16	4.49
GAIF01034421.1	0.3	0.33	0	0	0.16	0
GAIF01034422.1	0.09	0	0.05	0.1	0.57	0.02
GAIF01034424.1	0	0	0	0	0.29	0
GAIF01034425.1	0	0.23	0	5.78	12.81	0
GAIF01034426.1	0	0	0	0	0.29	0
GAIF01034427.1	0	0	0	0	0.4	0
GAIF01034428.1	2.52	2.53	2.56	5.53	6.06	0.49
GAIF01034429.1	11.02	1.14	0.54	16.3	64.75	9.21
GAIF01034430.1	28.85	21.25	8.31	60.5	17.75	5
GAIF01034431.1	0	0	0.11	6.11	11.12	0
GAIF01034432.1	0	0	0.12	0	0	0
GAIF01034433.1	0.13	0	0	0	0	0.15
GAIF01034435.1	6.99	13.25	12.74	6.52	8.63	2.54
GAIF01034436.1	9.46	10.01	0	12.08	1.93	23.58
GAIF01034438.1	12.24	0.17	0	0.89	0	0
GAIF01034439.1	0	0	0	0	0.73	0
GAIF01034440.1	0	0	0.1	0.07	0.18	0
GAIF01034441.1	0.2	0.36	0.29	0.46	0.08	1.68
GAIF01034442.1	82.06	19.72	38.83	105.81	107.37	517.73
GAIF01034443.1	48.19	17.88	14.55	108.51	219.78	490.26
GAIF01034445.1	1.19	0.74	0.74	0.1	0	0
GAIF01034447.1	1.64	0.47	0	2.76	1.64	0.75
GAIF01034448.1	1.29	0.14	0	0.98	0	0.01
GAIF01034449.1	0	0.09	0	0.05	0	0
GAIF01034450.1	17.99	36.89	51.08	10.62	10.04	12.9
GAIF01034451.1	0.2	0.24	0.07	0.33	1.13	0.09
GAIF01034452.1	0	0	0.84	0	0	0.17
GAIF01034454.1	0.23	0	0	0	0	0
GAIF01034455.1	0	0	0	0.26	0.87	0
GAIF01034456.1	0	0	0	0	0.06	0.16
GAIF01034458.1	0.08	0.08	0.18	1.63	6.91	0.08
GAIF01034459.1	46.85	19.64	22.82	59.06	77.99	323.06
GAIF01034462.1	108.7	137.86	138.58	104.73	98.04	67.46
GAIF01034463.1	0.95	0.97	0	19.06	81.81	0
GAIF01034464.1	0.1	0.03	0	0.34	0.14	0.19
GAIF01034465.1	19.35	19.26	22.05	54.71	42.02	7.62
GAIF01034466.1	0.29	0	0	0.68	0.52	0
GAIF01034468.1	0	0.11	0	0	0	0
GAIF01034469.1	0	0	0	0.57	4.53	0
GAIF01034470.1	0.5	0.73	0.05	0.53	1.05	0.14
GAIF01034471.1	14.77	3.32	0.45	12.16	16.97	1.55

GAIF01034472.1	0.33	0.25	0	1.24	2.41	0
GAIF01034473.1	0	0	0	0.11	0	0
GAIF01034474.1	53.37	71.6	84.96	225.97	171.27	109.32
GAIF01034476.1	0	0	0	0	0.45	0
GAIF01034479.1	0	2.62	4.19	0	0.36	0.33
GAIF01034480.1	0	0	0	1.42	0.87	0
GAIF01034481.1	0.18	0	0.07	0.21	0.14	0.26
GAIF01034482.1	0	1.18	1.43	0	0	0.24
GAIF01034483.1	0.67	0	0	3.5	13.44	0.76
GAIF01034484.1	0.46	0.04	0.04	1.77	9.57	0.04
GAIF01034485.1	43.99	31.06	37.21	128.76	163.97	77.41
GAIF01034487.1	51.34	50.78	32.9	81.48	78.17	142.51
GAIF01034488.1	0	0	0.06	0	0	0.99
GAIF01034489.1	0.16	0.12	0.23	0.62	0.64	0.23
GAIF01034490.1	47.28	57.09	90.04	204.52	170.15	72.46
GAIF01034491.1	21.25	25.35	19.81	57.48	46.3	5.13
GAIF01034492.1	0	18.77	4.97	16.89	5.92	6.01
GAIF01034493.1	1.61	0.95	0.48	0.37	0.42	0.97
GAIF01034494.1	0.38	0	0	0.73	1.71	0.28
GAIF01034495.1	22.74	17.08	24.15	25.21	20.12	115.91
GAIF01034497.1	84.52	82.21	160.68	202.35	73.62	44.25
GAIF01034498.1	10.62	14.04	2.95	20.02	34.75	3.23
GAIF01034499.1	25.78	8.56	17.7	10.29	0	16.98
GAIF01034501.1	47.97	39.52	45.5	27.57	19.03	16.69
GAIF01034504.1	0.29	0.77	0	0.01	0	0.09
GAIF01034505.1	0	0.05	0	0.14	2.11	0.13
GAIF01034506.1	5.29	2.12	3.93	1.56	0.36	1.3
GAIF01034507.1	0	0.22	0	0	0.58	0
GAIF01034508.1	0	1.71	6.96	0	0	29.64
GAIF01034510.1	0.18	0.23	0.1	0	0.04	0.03
GAIF01034512.1	3.88	2.89	4.97	2.74	2.26	4.32
GAIF01034513.1	0	0.62	0.88	0	0.21	0
GAIF01034514.1	12.25	11.17	21	28.33	21.65	17.66
GAIF01034515.1	0	0	0	0.1	0.1	0
GAIF01034518.1	0.24	0.27	0	2.84	14.11	2.16
GAIF01034519.1	0.45	0.25	0	1.97	5.86	1
GAIF01034520.1	1.32	0	0.21	1.64	7.88	5.3
GAIF01034521.1	0	0.17	0	0	0	0
GAIF01034522.1	1.37	2.98	2.42	4.43	3.06	0.86
GAIF01034523.1	0	0	0.82	0	0.95	0.65
GAIF01034524.1	0	0.16	0	0.75	0.17	0
GAIF01034525.1	5.17	0	1.23	0.87	0	0.14
GAIF01034526.1	4.87	6.45	5.25	14.07	10.73	3.43
GAIF01034527.1	25.29	31.81	32.47	82.17	68.39	11.38

GAIF01034528.1	0.26	0.47	1.34	0.5	1.03	0.18
GAIF01034529.1	0.52	0.28	0	2.88	11.15	1.01
GAIF01034531.1	0	0	0	0	0.21	0
GAIF01034532.1	86.92	77.46	78.16	115.75	57.29	34.89
GAIF01034534.1	0.11	0.23	0.1	0.52	1.74	0
GAIF01034535.1	0	0	0	4.88	0	0
GAIF01034537.1	0	0	0	0.1	1.19	0.2
GAIF01034538.1	0	0	0	0	0	0.03
GAIF01034539.1	0.06	0.07	0	0.07	0.15	0.14
GAIF01034541.1	39.5	27.29	15.12	238.97	138.18	33.7
GAIF01034544.1	0.03	0	0	0	0.04	0.03
GAIF01034547.1	0.39	0.21	0	0.8	5.32	0.43
GAIF01034551.1	20	23.77	19.83	21.61	30.2	108.55
GAIF01034552.1	0	0	0	0.1	0.11	0
GAIF01034553.1	0.51	0	0	1.18	7.56	0.75
GAIF01034554.1	110.94	45.99	72.28	98.12	45.82	22.29
GAIF01034555.1	0.58	0.08	9.46	5	2.46	32.18
GAIF01034557.1	7.65	2.36	2.43	4.99	4.97	2.66
GAIF01034558.1	0.03	0	0	0	0.01	0
GAIF01034559.1	1.09	0.79	1.2	1.41	0.44	0.27
GAIF01034560.1	27.24	38.53	33.16	70.92	53.83	23.66
GAIF01034561.1	16	13.55	25.39	45.05	19.76	19.82
GAIF01034562.1	0.78	1.18	0.34	0.54	1.87	0
GAIF01034563.1	10.02	11.85	5.92	31.55	34.12	2.95
GAIF01034564.1	0.12	0.09	0	0.05	0	0
GAIF01034567.1	0	0	0	0.18	0.12	0
GAIF01034568.1	0	0.21	0.03	1.37	2.84	0.11
GAIF01034570.1	0.26	0.14	0	1.38	4.3	0
GAIF01034571.1	0.72	0.22	0	3.56	14.4	1.02
GAIF01034573.1	0	0	0.58	0	0	0
GAIF01034574.1	1.32	0.91	0.52	5.92	12.21	7.72
GAIF01034575.1	0	0	0	0.1	0.04	0
GAIF01034577.1	0	0.08	0	0.08	0	0
GAIF01034578.1	0.63	0.14	0	2.63	11.81	0.28
GAIF01034580.1	7.09	5.35	5.02	11.55	14.86	9.58
GAIF01034581.1	68.67	4.94	4.57	16.58	27.53	64.63
GAIF01034582.1	0.28	0.18	0.3	0.83	1.4	0.12
GAIF01034583.1	0	0.06	0.2	0	0.06	0.06
GAIF01034585.1	0	0	0	0	0	0.11
GAIF01034586.1	0	0	0	0	0.15	0
GAIF01034588.1	0.97	0.33	0.13	0.14	0.44	0.07
GAIF01034589.1	65.57	58.69	25.66	45.38	30.43	8.23
GAIF01034590.1	0.33	0.36	0.18	2.07	3.71	0.19
GAIF01034593.1	0.06	0	0	0.3	1.41	0

GAIF01034594.1	0	0	0	0.1	1.66	0
GAIF01034596.1	0	0	0	0	0.67	0
GAIF01034597.1	0.23	0	0	0	0	0
GAIF01034599.1	0.07	0	0	0	0.04	0
GAIF01034600.1	12.5	21.75	26.55	24.52	11.17	14.63
GAIF01034601.1	29.61	30.88	32.14	54.42	31.9	14.8
GAIF01034602.1	5.97	3.16	3.28	0	0	25.56
GAIF01034603.1	0.09	0.25	0	0	0	0
GAIF01034604.1	0.02	0.02	0	0.11	0.46	0
GAIF01034606.1	1.11	0.44	0	0	1.33	0
GAIF01034607.1	22.61	36.04	54.94	33.2	15.29	20.82
GAIF01034609.1	0	0	0	0.32	0.08	0
GAIF01034611.1	0.55	0	0	0	1.33	0
GAIF01034612.1	4.27	2.88	3.96	2.34	0.8	2.81
GAIF01034614.1	0.17	0.19	0.11	1.62	1.41	0.23
GAIF01034615.1	0.11	0.12	0.08	0.3	0.84	0
GAIF01034616.1	0.14	0	0	0	0	0
GAIF01034617.1	18.86	21.31	29.56	41.65	35.63	18.42
GAIF01034620.1	11.68	7.88	7.65	41.75	45.44	6.42
GAIF01034622.1	0.2	0.09	0	0	0.38	0
GAIF01034623.1	0	0	0.62	0	0	0
GAIF01034624.1	0.3	0.13	0.4	0.77	3.55	1.73
GAIF01034625.1	0.24	0	0	0	1.24	0
GAIF01034626.1	39.72	39.56	29.9	69.39	51.69	28.22
GAIF01034627.1	141.92	199.55	255.28	200.01	164.61	138.02
GAIF01034628.1	2.72	2.6	1.27	6.23	4.99	1.21
GAIF01034630.1	20.93	11.58	13.6	1.15	0	3.41
GAIF01034632.1	12.98	16.84	8.42	2.71	5.57	0.86
GAIF01034633.1	27.98	14.46	13.29	25.43	22.38	18.66
GAIF01034634.1	31.45	9.26	7.79	21.6	22.72	27.75
GAIF01034636.1	0	0	0	0	0.04	0
GAIF01034637.1	0	0.25	0	0.48	1.29	0
GAIF01034638.1	31.01	16.77	27.72	22.25	20.21	24.27
GAIF01034639.1	1.5	0.59	0	2.53	15.54	6.3
GAIF01034640.1	0	0	0	0.21	0.22	0
GAIF01034642.1	231.16	73.25	46.36	432.72	97.86	6.46
GAIF01034643.1	0	0	0	0	0.3	0
GAIF01034644.1	2.94	5.62	0.57	4.99	10.33	1.33
GAIF01034645.1	18.05	19.98	6.75	68.41	52.63	3.65
GAIF01034646.1	50.65	54.34	7.35	1.11	1.32	59.3
GAIF01034647.1	1.74	6.81	5.35	8.2	11.97	0.44
GAIF01034648.1	17.16	27.83	13.24	45.85	40.38	9.51
GAIF01034649.1	3.47	0.63	0.19	6.45	17.95	10.33
GAIF01034650.1	0.01	0	0	0.27	0	0

GAIF01034651.1	0.37	0	0	0.85	0.22	0.4
GAIF01034653.1	0	0.1	0	0	0	0.03
GAIF01034654.1	0.81	1.58	2.37	0.8	1.36	2.82
GAIF01034655.1	1.07	1.29	0.13	0.83	6.01	0.13
GAIF01034656.1	9.52	11.05	5.52	32.63	31.65	5.16
GAIF01034658.1	10.78	18.33	11.54	7.15	6.27	1
GAIF01034659.1	0	0.05	0	0.18	0.19	0
GAIF01034660.1	0	0	0	0	0.47	0
GAIF01034663.1	0.77	0.37	0.19	1.4	5.28	1.8
GAIF01034664.1	0.35	0	0.13	0.41	5.11	0.13
GAIF01034665.1	0.14	0.15	0	0.97	4.45	0
GAIF01034666.1	0	0	0	0.71	0.93	0
GAIF01034667.1	0	0.04	0.07	0.37	0.58	0
GAIF01034668.1	6.34	5.12	4.98	15.03	8.16	3.31
GAIF01034669.1	0.33	0	0	0.38	0.5	0
GAIF01034672.1	37.15	46.85	51.4	27.8	31.88	52.74
GAIF01034673.1	0	0	0	0	0.02	0
GAIF01034674.1	0	0.07	0.21	0	0	0
GAIF01034675.1	0	0.15	0	0.32	0.84	0
GAIF01034676.1	2.72	2.27	3.47	6.32	11.57	2.51
GAIF01034677.1	0.53	0.23	0.35	0	0	0.35
GAIF01034678.1	17.54	14.36	10.21	15.69	27.44	34.23
GAIF01034679.1	0.44	0.16	0	0.51	1.4	0
GAIF01034682.1	0	0	0	0.05	0	0
GAIF01034683.1	0.45	0.1	0.2	0.1	0.11	0.1
GAIF01034684.1	3.72	1.85	0.8	11.92	25.03	0.49
GAIF01034685.1	6.37	5.54	6.87	14.52	7.62	6.02
GAIF01034686.1	0.64	0.26	0.53	0.84	0.1	0.09
GAIF01034687.1	0.55	0.15	0	0.16	0	0
GAIF01034688.1	0.04	0.12	0.3	0.21	0.28	0
GAIF01034689.1	0.09	0.2	0.1	1.41	6.94	0.15
GAIF01034690.1	0	0	0	0	0.28	0
GAIF01034692.1	0.11	0.03	0.06	0	0.7	0.03
GAIF01034693.1	3.73	8.41	3.07	1.51	0.67	0
GAIF01034694.1	0	0	0.26	0	0	0
GAIF01034695.1	4.32	12.29	6.74	12.76	15.69	1
GAIF01034697.1	1.36	1.61	0.78	0.34	0	0
GAIF01034701.1	0	0	0	0.33	0.94	0
GAIF01034702.1	0	0	0	0	0	0.14
GAIF01034703.1	1.96	0.45	0.23	2.39	1.98	0.11
GAIF01034704.1	0.25	0.8	0.27	1.14	2.95	0.27
GAIF01034705.1	19.25	21.7	35.1	21.41	11.57	18.32
GAIF01034706.1	0.34	0.11	0	2.26	6.3	0.19
GAIF01034707.1	41.83	26.67	113.27	65.18	47.59	138.85

GAIF01034708.1	16.14	15.96	15.22	37.4	25.82	9.71
GAIF01034709.1	0.14	0	0	0.8	4.33	0.15
GAIF01034710.1	0	0	0	0.06	0.06	0
GAIF01034714.1	19.6	18.55	4.08	34.56	45.05	1.67
GAIF01034715.1	9.9	9.1	9.52	25.81	21.53	5.12
GAIF01034717.1	0	0.07	0	0	0	0
GAIF01034718.1	0	0	0	0	0.11	0
GAIF01034720.1	45.52	29.37	20.75	104.27	103.51	23.77
GAIF01034721.1	0	0	0	0.39	0.47	0
GAIF01034722.1	0.28	0.2	0	1.74	7.55	0.1
GAIF01034723.1	0.2	0.22	0	0.41	0.15	0
GAIF01034725.1	64.07	117.78	61.48	139.47	115.57	10.33
GAIF01034726.1	7.28	3.41	6.02	8.75	13.79	57.14
GAIF01034727.1	0.03	0	0	0	0.02	0.02
GAIF01034729.1	52.44	27.34	18.87	63.45	41.51	6.9
GAIF01034730.1	22.56	34.58	24.09	39.28	45.15	78.04
GAIF01034731.1	0.11	0	0	0	0	0
GAIF01034735.1	0.93	0.25	0.13	3.09	21.88	0.89
GAIF01034737.1	0	0.07	0	0.18	0	0.07
GAIF01034738.1	9.74	11.97	5.22	1.61	0.98	0
GAIF01034740.1	0.19	0.25	0.13	4.83	12.9	0.63
GAIF01034741.1	0.26	0.12	0.04	0.21	0.09	0
GAIF01034742.1	7.22	15.7	5.44	4.63	7.06	0.95
GAIF01034743.1	9.95	5.48	0	23.34	19.44	0.63
GAIF01034744.1	0	0.12	0	0.54	2.13	0.35
GAIF01034746.1	0	0.05	0	0.22	0.52	0
GAIF01034747.1	29.29	25.15	40.32	23.52	29.33	75.26
GAIF01034749.1	0	0	0	1.05	1.99	0
GAIF01034750.1	0.11	0.08	0	0.13	0.06	0
GAIF01034752.1	0	0	0	0	0.11	0
GAIF01034753.1	0	0	0.06	0.09	0.34	0
GAIF01034754.1	12.88	6.4	7.29	18.84	27.57	10.97
GAIF01034755.1	0.17	1.37	0.78	0.12	0.12	0.11
GAIF01034756.1	26.24	9.13	4.13	66.66	115.72	24.35
GAIF01034757.1	3.9	7.48	4.98	9.63	11.53	1.36
GAIF01034758.1	0.31	0	0	0	0	0
GAIF01034760.1	0	0.17	0	0	0	0
GAIF01034762.1	0	0.23	0.08	0.16	0.5	1.6
GAIF01034763.1	23.06	26.8	24.34	43.37	35.11	8.27
GAIF01034764.1	7.28	5.62	2	13.9	32.49	10.35
GAIF01034765.1	0.13	0.28	0.16	0.31	0	0.24
GAIF01034766.1	0	0	0	0.22	0	0
GAIF01034770.1	0	0.41	0.26	0	0	0
GAIF01034772.1	1.72	3.68	4.2	14.05	13.88	1.31

GAIF01034773.1	14.88	12.12	12.61	8.93	12.52	12.12
GAIF01034774.1	0.11	0.04	0	0.96	4.03	0.32
GAIF01034775.1	0	0	0.06	0	0.07	0
GAIF01034777.1	0.53	0.34	0.1	2.96	8.71	0.08
GAIF01034778.1	0.05	0	0	0.11	0.49	0
GAIF01034779.1	10.87	9.96	2.79	46.95	56.38	8.11
GAIF01034780.1	0	0	0.07	0	0	0
GAIF01034781.1	4.16	0.29	0	1.56	1.45	3.55
GAIF01034782.1	0.05	0.05	0	0	0	0
GAIF01034784.1	0	0	0	0.04	0.04	0
GAIF01034786.1	16.55	13.17	14.6	26.75	13.86	14.33
GAIF01034788.1	11.82	8.88	18.58	24.04	8.42	18.65
GAIF01034789.1	0	0	0	0.08	0	0
GAIF01034791.1	0	0.03	0	0.32	2.2	0
GAIF01034792.1	0	0	0	0	0.43	0.13
GAIF01034794.1	1.28	1.42	2.23	3.59	1.73	1.48
GAIF01034795.1	0.58	0.21	0.24	1.84	0.93	0.52
GAIF01034796.1	0	0.9	1.6	2.15	0.66	0.88
GAIF01034797.1	0	0.13	0.11	0.01	0.2	0
GAIF01034798.1	6.91	1.48	1.86	11.76	24.64	2.87
GAIF01034799.1	0	0	0	0.11	0.12	0
GAIF01034800.1	185.65	122.97	90.36	138.51	110.3	112.68
GAIF01034802.1	0	0	0	0.42	0	0
GAIF01034804.1	0.06	0	0.04	0.04	0.08	0
GAIF01034805.1	1.25	1.03	1.42	3.26	2.86	0.71
GAIF01034806.1	29.01	11.68	6.5	78.94	64.08	21.79
GAIF01034809.1	0.15	0.16	0.49	0.17	0	0
GAIF01034810.1	0	0.11	0	0.82	2.93	0
GAIF01034812.1	43.87	2.89	7.59	26.85	7.99	29.74
GAIF01034813.1	73.06	57.71	16.22	126.34	122.16	26.16
GAIF01034814.1	2.25	0.72	0.29	10.29	19.73	0.87
GAIF01034815.1	34.09	44.34	18.29	46.31	41.35	4.59
GAIF01034816.1	0	0	0	0	0.1	0
GAIF01034817.1	2.85	2.78	2.89	5.6	3.6	5.56
GAIF01034818.1	14.08	7.55	6.07	29.33	31.19	7.59
GAIF01034819.1	15.01	2.91	1.62	11.8	14.41	5.1
GAIF01034821.1	2174.45	2099.43	2875.37	1128	856.21	2709.22
GAIF01034823.1	0.59	0.41	0.04	3.26	3.43	0.02
GAIF01034824.1	0	0	0	0	0.04	0
GAIF01034825.1	0.58	0.9	0.38	0.4	4.73	0.64
GAIF01034828.1	0	0	0	0.17	0	0
GAIF01034829.1	0	0.56	0.9	0	0	0.17
GAIF01034831.1	3.22	5.15	1.47	6.33	6.45	0.39
GAIF01034832.1	0	0	0.08	0	0.09	0

GAIF01034833.1	372.66	222.96	257.47	726.27	387.09	402.07
GAIF01034834.1	54.55	40.24	48.54	17.79	16.07	15.45
GAIF01034835.1	0	0.08	0	0	0	0
GAIF01034836.1	0.27	0	0	0.15	0	0
GAIF01034837.1	1.46	1.06	0.93	0.49	0.06	0
GAIF01034838.1	5.09	5.41	6.73	4.56	0.83	0.89
GAIF01034839.1	19.02	9.04	7.06	3.03	0.77	0.12
GAIF01034840.1	0.07	0	0.11	0.15	0.11	0
GAIF01034841.1	136.93	44.17	14.87	597.14	638.38	99.98
GAIF01034842.1	0	0	0	0	0.39	0.02
GAIF01034844.1	5.47	0.33	0.66	2.14	13.66	5.45
GAIF01034846.1	0.02	0.03	0.03	0.18	0.77	0.05
GAIF01034848.1	52.45	57.03	43.19	33.25	138.07	58
GAIF01034849.1	0	0	0	0.28	0.3	0
GAIF01034850.1	0	0.04	0	0	0	0
GAIF01034852.1	0.06	0	0	0.58	2.73	0.03
GAIF01034853.1	1.59	1.73	1.62	3.13	4.28	1.49
GAIF01034854.1	0	0.26	0.13	0	0	0
GAIF01034855.1	10.47	15.73	9.03	40.63	45.81	10.66
GAIF01034856.1	0	0	0	0	0.07	0
GAIF01034857.1	0.11	0.12	0.12	2.25	4.27	0.83
GAIF01034858.1	0	0	0	0	0.06	0
GAIF01034859.1	60	44.87	32.36	91.56	110.11	43.44
GAIF01034860.1	1	0	0.7	0.13	0.09	0.48
GAIF01034861.1	0	0	0.12	0	0	0
GAIF01034862.1	6.55	5.92	7.13	22.56	20.55	4.35
GAIF01034863.1	0	0	0.07	0.07	0.15	0
GAIF01034864.1	0	0	0	0.59	0.7	0.11
GAIF01034866.1	0.03	0.03	0.03	0.06	0.29	0
GAIF01034867.1	28.57	4.65	8.2	0	0	0
GAIF01034869.1	1.84	0.75	0.76	1.87	5.8	0.76
GAIF01034870.1	5.57	6.65	8.3	25.61	28.94	3.66
GAIF01034872.1	0.81	0.09	0.09	4.06	15.85	0.28
GAIF01034873.1	0.77	0	0	4.33	20.16	0.57
GAIF01034874.1	0	0	0	0	0.07	0
GAIF01034875.1	0	0	0	0.03	0.07	0
GAIF01034876.1	0.1	0	0	0.04	0.12	0
GAIF01034877.1	68.2	55.01	55.41	74.55	48.67	29.22
GAIF01034878.1	0	0.07	0.07	0	0	0
GAIF01034879.1	11.44	5.98	0.95	3.81	4.59	0.16
GAIF01034880.1	0.06	0.07	0	0.29	1.91	0.07
GAIF01034882.1	54.69	40.65	24.46	69.67	47.8	22.5
GAIF01034883.1	25.54	21.78	19.43	81.37	47.71	33.46
GAIF01034884.1	0	0	0	0	0.03	0

GAIF01034886.1	0	0	0	0	0.38	0
GAIF01034887.1	0	0	0	0	0.09	0
GAIF01034888.1	0.29	0.24	0.08	0.51	0.62	0.16
GAIF01034890.1	454.33	407.3	313.22	400.8	306.74	182.87
GAIF01034891.1	0.13	0.14	0	0.15	0.16	0.44
GAIF01034894.1	0	0	0	0.1	0	0.34
GAIF01034896.1	0	0	0	0	0.36	0
GAIF01034897.1	0.07	0	0	0.01	1.96	0.07
GAIF01034898.1	0.33	0.49	0.37	0.13	0.14	0
GAIF01034899.1	0.05	0.28	0.46	0.12	0.16	0.31
GAIF01034900.1	0.25	1.27	0.59	0.05	0	0.18
GAIF01034902.1	0.18	0.13	0.07	0.21	1.13	0.02
GAIF01034903.1	0	0	0.03	0	0	0
GAIF01034904.1	0.12	0.13	0	0.25	0.2	0
GAIF01034905.1	31.61	73.99	18.7	73.67	75.31	5.84
GAIF01034908.1	0	0	0.15	0	0	0
GAIF01034910.1	0	0	0.86	0	0	0.37
GAIF01034911.1	1.84	0.25	0	0.53	0	0
GAIF01034914.1	26.89	31.51	36.05	37.87	30.5	10.64
GAIF01034915.1	0.07	0	0	0.25	1.1	0
GAIF01034916.1	0	0	0	0	0	0.07
GAIF01034918.1	8.42	5.07	2.3	0.14	0.3	0.6
GAIF01034919.1	0	0	0	0.03	0.23	0
GAIF01034920.1	152.4	336.42	624.6	165.72	135.57	140.32
GAIF01034922.1	0	0.2	0.2	0	0	0
GAIF01034923.1	0	0	0	0	0.31	0
GAIF01034925.1	0.08	0.03	0	0.31	1.34	0
GAIF01034926.1	0	0	0	0	0.25	0
GAIF01034927.1	0	0	0	0.05	0.14	0
GAIF01034929.1	0	0.08	0	0.08	0.17	0
GAIF01034930.1	1.36	0.13	0.27	3.58	11	0
GAIF01034931.1	1.91	0.23	0.37	0.59	2.81	0.05
GAIF01034932.1	1.33	0	0.37	2.42	13.16	0.98
GAIF01034933.1	1.32	0.33	0.1	4.09	28.65	6.43
GAIF01034934.1	11.08	4.02	4.83	17.78	7.71	4.01
GAIF01034935.1	0	0	0	0.13	0	0
GAIF01034936.1	0	0	0	0.11	0	0
GAIF01034937.1	0.11	0	0	0	0.33	0
GAIF01034938.1	4.71	2.39	3.81	6.21	13.79	3.63
GAIF01034940.1	99.95	186.95	171.4	13.87	15.2	12.04
GAIF01034941.1	0.62	1.09	2.33	1.69	0.9	1.93
GAIF01034942.1	0.23	0.27	0	1.18	3.06	0.56
GAIF01034943.1	25.07	33.4	27.2	142.42	166.19	14.64
GAIF01034944.1	0.46	0	0	0	1.74	0.31

GAIF01034945.1	0	0	0	0	0.12	0
GAIF01034946.1	5.53	4.58	4.7	0	0	0.52
GAIF01034947.1	0.07	0	0	0.26	0.63	0
GAIF01034948.1	0.12	0.14	0.01	0	0.19	0.29
GAIF01034949.1	74.44	45.97	69.68	180.14	98.31	84.59
GAIF01034950.1	0	6.89	4.3	0.08	0	0.39
GAIF01034951.1	8.61	5.71	4.21	0.16	2.53	1.56
GAIF01034952.1	23.51	13.27	0	42.08	57.74	8.47
GAIF01034953.1	0	0.14	0.14	0.15	0.76	0.14
GAIF01034954.1	0	0	0	0.23	0.24	0
GAIF01034956.1	1.1	0.73	0.1	1.3	0.68	0.16
GAIF01034957.1	0	0	0	0	0.12	0
GAIF01034958.1	2.37	0.5	0.1	3.34	18.75	1.28
GAIF01034959.1	37	25.65	17.24	80.68	77.33	8.41
GAIF01034962.1	0	0	0	0	0.2	0
GAIF01034963.1	0.64	0.35	0.36	1.47	1.46	0.44
GAIF01034965.1	4.01	3.19	3.31	1.31	7.57	1.06
GAIF01034966.1	0.23	0	0	0	0	0
GAIF01034967.1	3.05	0.64	0.21	1.43	2.03	5.37
GAIF01034968.1	0	0	0	0.16	0	0
GAIF01034969.1	0.36	0	0	0.17	0.86	0
GAIF01034970.1	0.24	0	0	0	0.14	0
GAIF01034974.1	0	0	0	0.31	0.3	0
GAIF01034975.1	0.5	0.04	0.13	1.68	1.48	0.51
GAIF01034977.1	8.69	3.99	8.73	12.93	8.39	58.68
GAIF01034979.1	4.1	0.54	2.07	0.29	0.2	0.27
GAIF01034981.1	0.51	0.14	0.14	6.43	20.59	1.32
GAIF01034983.1	0	0	0	0.26	0.41	0
GAIF01034984.1	2.44	2.88	2.98	15.5	36.45	2.47
GAIF01034985.1	119	85.51	148.33	282.44	12.69	82.25
GAIF01034986.1	0.22	0.05	0	0.48	1.52	0.02
GAIF01034989.1	0.08	0.25	0.02	0.04	0	0.02
GAIF01034992.1	0.37	0	0	1.29	5.65	0
GAIF01034994.1	0.55	0	0	0	1.55	0
GAIF01034995.1	1.82	1.16	0.33	2.47	4.2	0
GAIF01034996.1	0	0	0	0	0.08	0
GAIF01034997.1	1.11	0.59	0.94	3.31	7.52	0
GAIF01034998.1	0	0	0.32	0.13	0	0
GAIF01035000.1	1.02	0.55	0.24	0.25	0.13	0
GAIF01035003.1	5.74	0.79	0.11	0.61	0.88	0.81
GAIF01035006.1	0.88	0.34	0.97	1.15	2.91	2.1
GAIF01035008.1	0.17	0.09	0	0	0	0
GAIF01035009.1	7.17	9.37	5.43	16.8	7.77	2.2
GAIF01035010.1	1.48	2.42	0.58	9.7	12.09	0

GAIF01035011.1	21.43	13.98	3.28	10.74	17.25	1.45
GAIF01035014.1	0.04	0.15	0.03	0.19	0.15	0
GAIF01035015.1	38.84	33.27	18.74	10.12	8.1	5.73
GAIF01035016.1	1.1	0.24	0.32	0	0.09	0
GAIF01035019.1	12.15	8.51	12.34	34.37	13.4	17.48
GAIF01035020.1	0	0	0	3.48	3.85	0
GAIF01035021.1	0.89	0.18	0.08	0.69	1.88	0.86
GAIF01035023.1	0	0.17	0.11	0	0	0
GAIF01035026.1	0.09	0	0.14	0.16	0.91	0.14
GAIF01035028.1	35.82	40.87	32.63	23.86	14.49	5.67
GAIF01035030.1	0.03	0	0.02	0	0	0
GAIF01035032.1	9.63	18.4	12.14	28.05	13.02	5.51
GAIF01035034.1	24.47	26.39	28.93	88.3	33.47	23.49
GAIF01035036.1	0.04	0.02	0	0	0.03	0
GAIF01035037.1	0.1	0	0	0	0	0
GAIF01035039.1	0	0	0	0.15	0	0
GAIF01035041.1	0	0	0	0	0.43	0.15
GAIF01035042.1	1.16	0.31	0.19	0.5	0.78	0
GAIF01035043.1	12.94	16.23	14.56	15.73	18.82	5.35
GAIF01035044.1	0.18	0	0.41	0	0.67	0
GAIF01035045.1	0.22	0.4	0.22	1.56	1.92	0.26
GAIF01035046.1	0	0	0	3.26	3.22	0
GAIF01035049.1	0	0	0	0	0.16	0
GAIF01035050.1	6.57	3.34	9.61	16.97	7.67	43.49
GAIF01035051.1	0	0.05	0	0.04	0	0
GAIF01035052.1	0	0	0	0	0.42	0
GAIF01035053.1	0.12	0	0	1.71	4.29	0.27
GAIF01035054.1	22.39	32.73	22.24	94.56	135.74	8.04
GAIF01035055.1	0.31	0.08	0	0.09	0	0.26
GAIF01035056.1	0.12	1.23	0	0	0.37	0
GAIF01035057.1	5.45	6.42	3.65	21.7	16.83	9.86
GAIF01035058.1	0.07	0.02	0.04	0	0	0
GAIF01035059.1	9.89	0	0	35.67	29.52	7.87
GAIF01035060.1	2.4	0.77	4.17	1.49	3.1	5.78
GAIF01035061.1	1.24	1.85	0.68	0.18	0	0
GAIF01035063.1	0.12	0	0.1	0	0	0.09
GAIF01035064.1	2.11	4.13	0.89	0	0	0
GAIF01035065.1	0.09	0	0	0.2	0.05	0
GAIF01035067.1	0	0	0.08	0	0	0
GAIF01035068.1	170.47	196.58	78.23	210.43	153.23	12.87
GAIF01035069.1	0	0.12	0.06	0	0	0.18
GAIF01035070.1	42.92	30.51	13.6	118.29	163.3	11.02
GAIF01035071.1	0.02	0.03	0.05	0.02	0.08	0.02
GAIF01035072.1	0.63	0.34	0	0.36	0	0

GAIF01035073.1	0	0	0	0.19	0.21	0.21
GAIF01035075.1	0.74	2.41	0.65	8.05	9.05	0
GAIF01035078.1	0.34	1.01	0.2	4.38	14.13	0.29
GAIF01035080.1	0	0	0	0.58	3.03	0.35
GAIF01035085.1	6.43	9.98	7.74	15.65	11.04	4.69
GAIF01035086.1	0	0	0.1	0	0.15	0
GAIF01035088.1	0	0	0	0	0.07	0
GAIF01035090.1	0.21	0.12	0.23	0.87	6.48	0.23
GAIF01035091.1	0.1	0.11	0.57	0	1.37	0
GAIF01035092.1	0.03	0	0	0.03	0	0
GAIF01035093.1	0.67	1.06	0.36	0.41	0.41	0.18
GAIF01035095.1	0	0	0.07	0	0	0
GAIF01035096.1	48.84	42.31	21.95	9.81	0	0.2
GAIF01035099.1	23.93	12.99	10.79	5.86	5.3	5.57
GAIF01035100.1	7.99	4.43	0	38.88	104.95	0.53
GAIF01035101.1	0.15	0	0	0	0.18	0
GAIF01035102.1	0	0.41	0.15	0.04	0.04	0
GAIF01035103.1	9.29	5.94	0	10.79	27.15	0
GAIF01035104.1	0.26	0	0.09	2.03	4.9	1.78
GAIF01035105.1	0	0.08	0.08	0	0.06	0.03
GAIF01035106.1	20.21	17	10.93	59.14	46.04	33.26
GAIF01035108.1	0	0	0	0	0.31	0
GAIF01035110.1	0.53	0.46	0.08	9.43	25.77	0.19
GAIF01035111.1	18.03	14.62	0.94	34.06	56.85	3.53
GAIF01035112.1	83.59	76.97	59.43	140.62	95.45	22.72
GAIF01035114.1	32.01	33.58	21.61	54.51	40.99	3.97
GAIF01035115.1	0	0	0.02	0.01	0.02	0
GAIF01035116.1	2.52	0.69	0.05	0.3	0.37	0.02
GAIF01035117.1	1.21	1.27	0.34	9.43	17	1.78
GAIF01035119.1	0	0	0	0.25	0.13	0.59
GAIF01035120.1	0.04	0.05	0.05	0.64	2.29	0
GAIF01035121.1	86.23	138.52	64.95	19.86	20.38	7.77
GAIF01035123.1	16.92	15.61	10.33	41.39	40.87	5.63
GAIF01035124.1	0	0	0	0.01	0	0.09
GAIF01035127.1	0	0	0	0	0.09	0
GAIF01035129.1	0	0	0	0	0.36	0
GAIF01035131.1	0	0.05	0.28	0	0	0.32
GAIF01035132.1	0	0	0	0.2	0.15	0
GAIF01035134.1	0.04	0	0	0.05	0.08	0
GAIF01035139.1	18.63	20.68	21.91	91.46	86.65	12.49
GAIF01035142.1	66.11	94.85	42.42	35.67	18.38	6.48
GAIF01035143.1	0.89	0.86	0.99	0.9	0.53	0.32
GAIF01035144.1	0	0	0	0.05	0	0
GAIF01035146.1	0	0	0	0.61	0.26	0

GAIF01035147.1	2.41	1.71	2.46	19.19	38.84	5.07
GAIF01035148.1	2.29	1.04	0.21	14.86	48.94	1.68
GAIF01035149.1	8.83	10.01	15.49	22.83	13.03	8.34
GAIF01035150.1	28.13	5.66	9.12	2.55	0.61	0.57
GAIF01035151.1	0.62	0.65	0.38	1.04	0.31	0.12
GAIF01035152.1	0	0.05	0	0.47	2	0
GAIF01035153.1	0	0.14	0	0.05	0.49	0
GAIF01035154.1	0	0	0.5	0.81	0.47	0.37
GAIF01035156.1	0.09	0	0.48	0.27	0.25	0.18
GAIF01035157.1	0	0	0	0	1.48	0
GAIF01035158.1	0.03	0	0	0	0	0.03
GAIF01035160.1	65.58	79.7	53.51	99.11	91.06	58.89
GAIF01035161.1	0	0	0	0.2	0	0
GAIF01035162.1	0	0	0	0.2	0	0
GAIF01035163.1	4893.71	4311.04	6189.77	2682.09	2459.8	14775.2
GAIF01035167.1	0	0	0	0.12	0	0
GAIF01035168.1	0.07	0	0	0	0	0.08
GAIF01035169.1	0.14	0	0.15	0.16	0	0
GAIF01035170.1	0.6	1.54	0.26	0	0.44	0
GAIF01035172.1	5.99	3.72	7.87	16.26	12.47	2.47
GAIF01035173.1	2.67	1.86	1.69	5.23	6.46	0.42
GAIF01035174.1	0	1.75	1.4	0.32	0.67	0
GAIF01035176.1	7.85	7.81	0	12.81	26.77	12.48
GAIF01035177.1	0.08	0	0	0	0	0
GAIF01035178.1	4.26	7.43	1.13	12.11	19.89	0.41
GAIF01035179.1	36.5	29.05	11.45	47.13	40.1	7.17
GAIF01035180.1	2.47	1.2	0.32	0.81	3.17	0.12
GAIF01035182.1	70.81	0.1	1.35	27.94	0.23	0.94
GAIF01035183.1	0.27	0.09	0	0.1	0.21	0.17
GAIF01035185.1	0.64	0	0	3.79	21.98	0.3
GAIF01035186.1	0.16	0.16	0.16	1.89	4.3	0.04
GAIF01035188.1	28.53	0	0.94	13.02	5.49	9.85
GAIF01035189.1	9.23	20.5	8.61	1.35	0.93	0.16
GAIF01035191.1	0	0	0	0	0.3	0
GAIF01035192.1	7.71	12.41	10.03	14.08	11.67	1.73
GAIF01035196.1	0	0	0	0	0.34	0
GAIF01035197.1	22.21	13.81	16.51	29.49	33.53	25
GAIF01035198.1	0	0	0	0	0.37	0
GAIF01035199.1	0	0.17	0	0	2.9	0
GAIF01035200.1	2.24	0.74	0.77	5.86	21.99	4.31
GAIF01035201.1	0	4.72	2.99	0.08	0.16	0.69
GAIF01035204.1	0	0	0.04	0.07	0.1	0
GAIF01035205.1	0	0.62	0.56	0.15	0.15	0.35
GAIF01035206.1	0	0.03	0	0.49	2.25	0

GAIF01035207.1	0	0	0	0.03	0	0.06
GAIF01035208.1	0.06	0	0.15	0.2	0.31	0.04
GAIF01035209.1	0.18	0	0	0.87	3.4	0.1
GAIF01035210.1	0	0	0	0.63	1.81	0
GAIF01035214.1	0.28	0	0	0	0	0
GAIF01035215.1	0	0	0	0	1.66	0
GAIF01035217.1	0.32	0.05	0.14	0.73	3.12	0
GAIF01035218.1	0.19	0.07	0.03	0	0	0
GAIF01035220.1	24.21	37.45	38.61	78.13	37.51	17.52
GAIF01035221.1	0.07	0	0.37	0.31	0.32	0
GAIF01035222.1	0.14	0.06	0.05	2.32	14.95	0.05
GAIF01035224.1	0	0	0	0	0.15	0
GAIF01035225.1	8.79	4.47	10.79	13.37	5.15	6.05
GAIF01035226.1	9.85	31.58	100.99	4.98	4.63	28.96
GAIF01035227.1	0.72	0.06	0	0.06	0	0
GAIF01035228.1	7.94	1.34	1.15	97.12	313.35	4.11
GAIF01035229.1	0	0.1	0	0	0	0
GAIF01035230.1	0.87	0.8	0.15	2.56	2.41	0
GAIF01035231.1	0	0	0	0.56	0	0
GAIF01035232.1	0.13	0	0	0	1.27	0
GAIF01035233.1	9.74	10.25	13.54	21.25	11.24	8.83
GAIF01035234.1	2.41	1.85	2.65	5.26	3.24	0.47
GAIF01035235.1	0.09	0.13	0.07	0.18	0.11	0
GAIF01035238.1	0	0.14	0.14	0	0	0.14
GAIF01035239.1	125.81	248.54	289.69	83.98	48.03	19.57
GAIF01035241.1	343.26	202.8	84.95	76.12	163.14	9.24
GAIF01035242.1	0	0	0	0	0.07	0
GAIF01035244.1	15.04	17.93	9.52	35.15	41.73	11.76
GAIF01035246.1	25.03	19.35	24.46	32.07	13.57	14.99
GAIF01035248.1	4.54	10.47	3.66	14.83	15.14	2.86
GAIF01035252.1	0.46	0.17	0	0	1.11	0.34
GAIF01035253.1	0.33	0.36	0	0.38	1.18	0
GAIF01035255.1	1.24	1.48	0.95	5.04	9.41	1.23
GAIF01035256.1	3.47	3.02	2.83	7.33	10.74	1.64
GAIF01035257.1	0.09	0.36	0.03	0.9	2.71	0
GAIF01035258.1	0	0	0	0	0.15	0
GAIF01035259.1	0.24	0.18	0.09	1.27	3.01	0.04
GAIF01035260.1	0.77	0.43	0	6.06	19.01	0.04
GAIF01035262.1	14.71	6.34	0.59	15.28	29.46	3.11
GAIF01035263.1	34.38	25.57	14.94	13.27	16.77	11.04
GAIF01035266.1	15.66	18.23	26.83	64.78	73.32	11.2
GAIF01035267.1	0	0	0	0	1.43	0
GAIF01035268.1	51.34	41.5	19.44	12.53	11.67	3.29
GAIF01035269.1	15.34	11.61	9.96	30.73	29.61	6.91

GAIF01035270.1	2.32	4.19	7.24	7.53	3.68	6.28
GAIF01035271.1	2.65	2.11	3.84	4.79	4.91	11.46
GAIF01035272.1	0.09	0	0	0	0	0
GAIF01035275.1	0.26	0.14	0.14	0.59	0.31	0.42
GAIF01035276.1	9.68	12.93	6.23	6.85	3.66	1.07
GAIF01035277.1	0.05	0	0	0	0	0
GAIF01035278.1	0	0.24	0	0	0.34	0
GAIF01035279.1	0	0	0	0	0.17	0
GAIF01035282.1	0.58	0.32	0.16	1.68	9.25	0.48
GAIF01035283.1	1.17	0.16	0	0	0.18	0
GAIF01035284.1	0	0.07	0	0	0.07	0
GAIF01035287.1	4.33	4.88	2.02	2.79	2.99	1.94
GAIF01035288.1	0.09	0	0	0	0	0
GAIF01035289.1	0	0	0	0.85	3.28	0
GAIF01035290.1	17.12	14.25	11.63	52.37	104.68	77.1
GAIF01035292.1	9.14	6.79	0.59	19.27	75.21	0.59
GAIF01035294.1	0.08	0.05	0	0.1	0.1	0
GAIF01035295.1	0	2.72	3.23	0	0	1.08
GAIF01035296.1	0	0	0.05	0	0	0.05
GAIF01035299.1	0.1	0.02	0	1.06	5.07	0.25
GAIF01035300.1	330.07	63.59	106.13	313.82	113.23	1036.04
GAIF01035301.1	4.22	2.6	0	0	0.26	0
GAIF01035303.1	50.35	43.7	49.5	55.72	31.71	10.02
GAIF01035304.1	0	0	0	0.14	0.57	0.13
GAIF01035306.1	117.98	117.03	69.42	352.81	534.07	63
GAIF01035307.1	3.61	3.02	2.86	5.21	2.92	0.6
GAIF01035308.1	4.7	0.97	1.23	8.16	16.63	1.48
GAIF01035309.1	0.24	0	0	0.14	0.29	0
GAIF01035310.1	27.17	25.7	19.25	181	102.53	21.71
GAIF01035311.1	2.19	3.1	0.79	10.74	11.07	0.98
GAIF01035312.1	0	0.11	0.05	0.17	0.12	0
GAIF01035313.1	17.28	0	0	0	0	0
GAIF01035314.1	4.92	7.96	6.94	5.96	7.5	2.28
GAIF01035316.1	0	0	0	2.59	7.91	0.99
GAIF01035317.1	0.31	0	0	3.17	9.57	2.17
GAIF01035319.1	0	0.05	0.05	0.11	1.13	0
GAIF01035320.1	0.02	0.05	0.11	0.32	1.6	0.03
GAIF01035323.1	32.41	34.58	10.41	7.8	3.42	0.28
GAIF01035325.1	0	0	0	0.1	0.09	0
GAIF01035326.1	0.15	0.08	0	0.09	1.79	0
GAIF01035328.1	18.26	21.16	15.41	0.21	0.07	0.13
GAIF01035329.1	33.54	51.64	46.05	41.82	41.13	8.44
GAIF01035330.1	0	14.58	0	0	29.26	70.29
GAIF01035332.1	0.07	0.08	0	0.68	2.02	0.16

GAIF01035333.1	129.12	113.06	117.03	325.55	213.87	111.27
GAIF01035334.1	0	0	0	0.38	0	0
GAIF01035335.1	8.13	13.26	10.24	20.41	10.79	4.35
GAIF01035336.1	46.97	96.03	95.88	83.6	66.22	13.38
GAIF01035337.1	0.16	0.06	0	2.64	6.67	1.81
GAIF01035338.1	120.67	267.91	58.41	93.94	22.04	36.59
GAIF01035339.1	60	22.6	11.38	143.2	131.06	6.05
GAIF01035340.1	37.46	24.44	49.35	86.96	63.07	148.63
GAIF01035341.1	0.13	0.09	0.06	0.28	1.6	0.12
GAIF01035342.1	2.86	3.07	1.72	2.94	3.06	0
GAIF01035343.1	0	0	0	0.07	0	0
GAIF01035344.1	0.93	0.46	0	0.88	2.54	0
GAIF01035345.1	24.13	19.64	11.79	40.85	46.39	4.86
GAIF01035346.1	21.67	23.56	15.54	43.74	47.95	8.53
GAIF01035347.1	0	0	0	0	0.18	0
GAIF01035349.1	9.4	3.82	0	31.72	40.59	5.41
GAIF01035351.1	0.17	0	0	0	0	0
GAIF01035352.1	0	0	0	0.89	2.3	0
GAIF01035353.1	0	0	0	0.29	0.24	0
GAIF01035354.1	0.75	0.68	0.9	1.97	1.28	0.69
GAIF01035356.1	14.06	25.61	11.73	35.07	20.53	2.41
GAIF01035357.1	0.18	0	0.1	0.72	3.32	0
GAIF01035358.1	108.5	529.73	254.66	55.88	131.45	18.36
GAIF01035359.1	0	0	0	0.19	0	0
GAIF01035360.1	124.15	24.95	79.63	290.54	219.17	738.47
GAIF01035362.1	82.04	264.79	195.61	41.12	54.97	44.46
GAIF01035363.1	0.23	0.33	0.28	0.6	0.47	0
GAIF01035364.1	0	0	0	0	0.08	0
GAIF01035366.1	0	0.1	0	0.12	0.27	0
GAIF01035367.1	0	0.03	0.03	0.03	0.07	0.03
GAIF01035368.1	39.91	43.87	65.68	42.37	28.78	24.26
GAIF01035370.1	0	0.09	0	0.01	0.16	0
GAIF01035371.1	37.69	51.95	66.04	36.84	22.52	10.23
GAIF01035372.1	0	0	0	0	0.19	0
GAIF01035373.1	0	0	0.14	0	0	0
GAIF01035374.1	0	0	0	0.18	1.57	0
GAIF01035376.1	0	0	0	0.79	2.05	0.19
GAIF01035378.1	2.41	0	0	4.44	11	30.3
GAIF01035379.1	45.55	37.21	50.35	22.54	10.66	11.21
GAIF01035381.1	0	0	0	0	0.67	0
GAIF01035383.1	0.6	0	0	1.14	6.98	1.99
GAIF01035384.1	3.74	3.34	2.42	11.31	8.99	10.15
GAIF01035385.1	17.25	10.01	21.77	29.77	13.44	21.71
GAIF01035386.1	2.97	2.3	1.99	11.47	6.73	0.74

GAIF01035387.1	0	0.34	0	0.36	3.01	0
GAIF01035388.1	0.91	0.5	0.5	0.71	0	0.17
GAIF01035389.1	0	0	0	0.16	0.96	0.04
GAIF01035390.1	0.6	0	0	0	0	0
GAIF01035391.1	0.15	0.08	0.08	0.09	0.53	0
GAIF01035392.1	0	0	0	0.35	0.82	0
GAIF01035393.1	0.02	0.08	0.03	0.06	0.09	0.03
GAIF01035395.1	13.44	14.17	15.85	43.46	18.76	12.23
GAIF01035396.1	2.87	2.21	0.65	6.73	10.99	4.3
GAIF01035397.1	26.31	28.02	16.01	3.98	1.22	0.3
GAIF01035398.1	1186.5	406.25	579.05	918.88	1014.63	10021.76
GAIF01035400.1	0	0	0	0	0.12	0
GAIF01035401.1	2.27	3.27	1.13	10.78	9.59	2.76
GAIF01035402.1	0	0.42	0	0	0	0
GAIF01035403.1	0	0	0	0.59	2.92	0.42
GAIF01035405.1	0	0	0	0.14	0.24	0
GAIF01035407.1	0	0	0	0.04	1.36	0
GAIF01035408.1	4.45	5.68	4.95	12.95	12.71	2.62
GAIF01035409.1	0	0	0	0	0.11	0
GAIF01035410.1	1.56	0.53	0.22	1.34	0.85	1.08
GAIF01035411.1	0.55	0.1	0	1.6	4.3	0
GAIF01035413.1	0.04	0	0	0	0.05	0
GAIF01035414.1	0	0	0	0	0.2	0
GAIF01035415.1	0	0	0.05	0.05	0	0
GAIF01035416.1	0	0	0	0	0.36	0
GAIF01035418.1	0	0	0	0.1	0.11	0
GAIF01035419.1	0.96	0.81	1.55	0	0	0
GAIF01035420.1	116.45	52.43	45.43	131.46	89.86	88.67
GAIF01035421.1	0.49	0.27	0	4.01	10.23	0.41
GAIF01035422.1	10.17	5.27	6.38	14.95	15.14	4.7
GAIF01035423.1	0	0.08	0	0	0	0
GAIF01035425.1	3.08	1.67	0.66	9.49	3.34	1.94
GAIF01035426.1	0.48	0.26	0.26	6.12	24.08	0
GAIF01035429.1	0.79	0.74	0	4.87	12.78	0
GAIF01035430.1	0.53	0	0	0.61	2.53	0
GAIF01035431.1	0	0	0	0.03	0.06	0
GAIF01035432.1	0.7	0.18	0.06	0.46	1.49	0.12
GAIF01035433.1	8	14.16	5.24	53.84	56.76	1.97
GAIF01035435.1	0.47	0.13	0	0	0.07	0
GAIF01035438.1	0	16.76	9.77	0	0	0.33
GAIF01035439.1	9.84	3.1	0	2.99	4.06	1.47
GAIF01035440.1	6.21	3.67	2.34	48.41	6.4	9.78
GAIF01035441.1	1.18	0.11	0	0.23	0	0
GAIF01035442.1	0	0	0.9	0.26	0	0.1

GAIF01035443.1	0	0	0	0	1.44	0
GAIF01035444.1	0	0.14	0.29	0.1	0.07	0.07
GAIF01035446.1	0	0.04	0	0	0	0
GAIF01035448.1	0.67	0.14	0.14	4.79	12.8	0.65
GAIF01035449.1	0	0	0	0.13	0.53	0
GAIF01035450.1	2.55	1.68	0.1	17.95	50.98	0.3
GAIF01035452.1	0.23	0	0	0.52	8.43	0
GAIF01035453.1	0.04	0.01	0.02	0.01	0	0.03
GAIF01035455.1	0	0	0	0	0.26	0
GAIF01035458.1	0.13	0	0	0	0	0
GAIF01035460.1	0.5	0.34	0.25	1.36	9.56	0.61
GAIF01035462.1	0.98	0.07	0	0.08	0.25	0.1
GAIF01035463.1	80.98	59.44	71.6	137.07	53.94	28.77
GAIF01035464.1	4.29	9.6	6.46	8.17	8.54	0.56
GAIF01035465.1	9.78	9.4	4.41	3.58	1.36	0.23
GAIF01035466.1	0	0	0	0	0	0.44
GAIF01035468.1	0	0	0.46	1.66	0	0
GAIF01035470.1	3.32	1.83	1.67	5.61	4.6	8.62
GAIF01035471.1	0.27	0	0	0.31	0.65	0
GAIF01035472.1	0.02	0.01	0	0	0	0.04
GAIF01035473.1	101.45	36.76	48.06	61.62	12.28	46.25
GAIF01035474.1	7.04	8.15	10.09	22.62	16.15	6.11
GAIF01035479.1	22.21	14.56	20.77	17.76	10.54	6.32
GAIF01035480.1	0	0	0	0.07	0.15	0
GAIF01035483.1	0.03	0	0	0.09	0	0
GAIF01035484.1	5.94	4.31	2.51	3.62	9.34	7.98
GAIF01035486.1	0	0.4	0.49	0.09	0	0.24
GAIF01035489.1	0.84	0.27	0.72	1.71	0.71	0.41
GAIF01035490.1	0	0	0.13	2.42	7.99	0
GAIF01035491.1	0	6.33	6.39	0	0	0
GAIF01035492.1	6.76	10.6	4.97	12.58	4.77	1.99
GAIF01035494.1	0.51	0	0.37	1.26	0	0.06
GAIF01035495.1	1.18	0.3	0.4	2.54	10.63	23.2
GAIF01035496.1	0.51	0.2	0	1.88	3.79	0
GAIF01035498.1	1.07	2.06	0.54	0.56	0.95	0
GAIF01035499.1	0.39	0	0.14	0.08	1.02	0
GAIF01035500.1	0	0	0	0.14	1.57	0
GAIF01035501.1	15.21	27.67	8.63	96.3	60.43	5.4
GAIF01035502.1	0	0	0	0.05	0.11	0
GAIF01035503.1	21.93	26.78	10.4	14.61	17.25	1.04
GAIF01035504.1	0.05	0.12	0.12	0.75	3.35	0
GAIF01035506.1	18.98	9.58	4.69	0.52	1.61	0.16
GAIF01035507.1	0.06	0	0	0.07	0	0
GAIF01035508.1	0.89	1.09	0.22	8.18	12.4	0.36

GAIF01035509.1	2.98	7.22	6.36	7.08	4	0
GAIF01035510.1	0	0	0	0.08	1.98	0
GAIF01035511.1	0.46	0.2	0.3	1.17	1.33	0.1
GAIF01035512.1	11.59	19.42	26.92	42.92	35.64	14.47
GAIF01035514.1	51.73	12.7	5.52	42.94	67.08	18.54
GAIF01035515.1	13.15	5.61	8.73	28.09	24.18	33.13
GAIF01035516.1	0.21	0.22	0.11	0.24	0.25	0
GAIF01035517.1	87.31	70.88	41.16	86.4	64.67	21.51
GAIF01035518.1	0.14	0.11	0	0.15	2.15	0
GAIF01035519.1	3.36	3.66	8.18	8.93	2.78	4.27
GAIF01035520.1	0.28	0.2	0.31	0	0	0
GAIF01035521.1	16.3	18.58	18.23	16.75	15.15	8.07
GAIF01035522.1	0	0.11	0.33	1.39	5.65	0.44
GAIF01035523.1	0.39	0.21	0	0.68	3.69	0.05
GAIF01035525.1	2.38	0.69	1.04	2.85	3.43	1.49
GAIF01035526.1	22.75	55.07	21.92	82.18	170.84	21.53
GAIF01035527.1	0	0.09	0	0.19	0	0
GAIF01035529.1	20.79	25.87	26.79	34.39	30.97	11.38
GAIF01035530.1	50.52	73.67	75.29	64.68	50.64	23.77
GAIF01035531.1	15.26	13.34	20.21	47.41	40.49	139.51
GAIF01035532.1	0.12	0.13	0.19	0.34	0.14	0.13
GAIF01035533.1	0.51	0.75	0.09	0.8	0.31	0.38
GAIF01035535.1	29.98	14.62	14.63	48.47	42.84	17.2
GAIF01035536.1	0.09	0.02	0	0.02	0	0.01
GAIF01035537.1	24.78	25.25	13.11	102.26	116.05	5.69
GAIF01035539.1	14.25	11.61	13.99	28.68	16.39	10.22
GAIF01035540.1	0	0.16	0.48	4.5	4.74	0.48
GAIF01035542.1	3.78	1.36	1.53	3.62	1.99	0.69
GAIF01035545.1	0.06	0.07	0	0.18	0.79	0
GAIF01035546.1	0	0	0	0.14	0	0
GAIF01035547.1	3.54	0.28	0	7.27	22.45	0
GAIF01035548.1	0	0	0	0	0	0.02
GAIF01035550.1	0	0	0	0.14	0.87	0
GAIF01035551.1	0.09	0	0.19	0.12	0.31	0.07
GAIF01035552.1	20.29	10.36	8.85	57.9	70.05	18.05
GAIF01035553.1	891.99	147.8	19.97	2156	1423.32	50.57
GAIF01035554.1	0	0	0	0	0	0.31
GAIF01035555.1	3.48	0.73	0	13.68	21.91	0.89
GAIF01035556.1	0	0	0	0	0.28	0
GAIF01035557.1	142.26	151.97	162.37	96.42	147.58	216.5
GAIF01035558.1	0.35	0	0	0.4	2.63	0
GAIF01035560.1	0	0	0	0.91	0	0
GAIF01035561.1	0.03	0	0.31	0	0	0
GAIF01035562.1	0.28	0.23	0.56	2.74	0.42	0.64

GAIF01035563.1	0	0	0	0.13	0	0
GAIF01035565.1	151.13	0	73.07	11.01	0	0
GAIF01035566.1	0	0	0	0	0.18	0
GAIF01035567.1	0	0.12	0	0	0	0
GAIF01035568.1	0.27	0.37	0	0.6	0.37	0
GAIF01035569.1	0.73	0.69	0.45	0.71	1.1	0.79
GAIF01035570.1	28.14	25.76	20.61	38.24	35.92	12.63
GAIF01035571.1	0	0.15	0.22	0.08	1.15	0
GAIF01035572.1	0	0.01	0.09	0.04	0.09	0
GAIF01035574.1	14.11	16.74	7.29	55.08	64	4.82
GAIF01035575.1	0.9	0.37	0.09	0	0.36	0
GAIF01035576.1	2.17	1.26	2.47	4.29	11.59	8.95
GAIF01035577.1	0	0	0	0	20.77	0
GAIF01035579.1	1.9	2.35	3.23	0.3	0.16	0.29
GAIF01035580.1	0	0	0	0.2	0.69	0
GAIF01035581.1	0.11	0.11	0	1.13	2.36	0.03
GAIF01035582.1	14.97	7.79	11.33	4.55	2.04	5.75
GAIF01035583.1	0.98	0.3	0	2.75	16.82	7.4
GAIF01035585.1	5.53	5.7	2.88	0.6	2.23	0
GAIF01035586.1	0	0	0	0	0.08	0
GAIF01035587.1	0	0.21	0.43	0.33	2.37	0.32
GAIF01035588.1	0	0	0	0	0	0.03
GAIF01035589.1	0.24	0.05	0.21	1.35	7.65	0.21
GAIF01035590.1	0.05	0	0	0	0	0
GAIF01035591.1	0.8	0.68	0.68	0.21	0.11	0.29
GAIF01035592.1	0.09	0	0.05	0.14	0.36	0
GAIF01035595.1	0.02	0.02	0	0.02	0.03	0
GAIF01035596.1	0.81	1.87	0.25	3.61	2.81	0
GAIF01035597.1	13.77	8.14	7.78	18.95	24.98	18.44
GAIF01035598.1	0.18	0	0	0.42	0	0
GAIF01035599.1	5.2	7.03	0.7	4.27	2.14	0
GAIF01035600.1	3.05	2.27	1.85	0.37	0.1	0
GAIF01035602.1	0	0	0	0.09	0.28	0
GAIF01035605.1	0	0.06	0	0	0	0
GAIF01035606.1	0	0	0.18	0.19	0.4	0.18
GAIF01035607.1	0	0	0	0	0.01	0
GAIF01035608.1	38.84	40.68	40.16	108.54	70.47	29.89
GAIF01035609.1	13.58	5.27	8.3	21.72	39.14	40.88
GAIF01035610.1	0	0.98	0	7.21	1.09	2.96
GAIF01035611.1	104.36	137.29	196.24	246.97	196.84	41.75
GAIF01035613.1	0.39	0.14	0	0	0.26	0
GAIF01035614.1	0	0	0.05	0.48	0	0
GAIF01035615.1	5.81	5.86	5.35	0.02	0.39	0
GAIF01035616.1	0	0.06	0	0.06	0.38	0

GAIF01035618.1	0	0	0.11	0	0	0
GAIF01035619.1	16.01	21.28	22.17	12.59	14.67	7.12
GAIF01035620.1	0.08	0.09	0	0	0.2	0
GAIF01035621.1	222.96	255.73	350.25	337.83	358.38	921.81
GAIF01035622.1	0.12	0	0	0.14	0.44	0
GAIF01035623.1	42.02	0.43	4.97	31.56	12.59	8.46
GAIF01035626.1	0	0.07	0	0	0	0
GAIF01035627.1	0	0	0	0	0.55	0
GAIF01035630.1	4.44	6.3	4.98	11.54	7.78	2.77
GAIF01035631.1	2.53	0.65	0.61	9.2	36.52	17.63
GAIF01035632.1	0	0	0	0.09	0	0
GAIF01035633.1	0.79	0	0	2.88	7.05	0.5
GAIF01035634.1	0.12	0.32	0	0.41	0.07	0.32
GAIF01035635.1	0	1.38	0.56	0.88	2.14	0.42
GAIF01035636.1	0.4	0.11	0.11	0.35	1.14	2.26
GAIF01035637.1	0	0.32	0.11	0.68	0.7	0
GAIF01035638.1	0	0.05	0	0	0	0
GAIF01035641.1	36.24	69.6	80.47	253.79	142.29	28.48
GAIF01035642.1	8.5	4.67	4.52	2.64	0.53	1.06
GAIF01035644.1	0	0.09	0	0.2	0.2	0
GAIF01035645.1	0	0	0	0.36	2.27	0
GAIF01035646.1	0.86	0.76	0.47	4.18	10.85	0.71
GAIF01035647.1	0.52	0.22	0.05	1.75	15.34	1.57
GAIF01035648.1	0.23	0.15	0.18	0.14	0.22	0
GAIF01035649.1	0.16	0	0.52	0	0	0.35
GAIF01035650.1	34.88	23.17	42.75	64.04	23.64	39.56
GAIF01035651.1	24.89	13.94	16.29	27.87	18.09	41.93
GAIF01035653.1	11.86	5.56	5.48	36.26	53.02	12.19
GAIF01035655.1	40.81	70.82	97.38	105.28	98.31	31.96
GAIF01035656.1	1.69	0.44	0.08	3.04	9.33	7.13
GAIF01035658.1	0.14	0	0	0.64	8.41	0
GAIF01035660.1	0	0	0	0	1.17	0
GAIF01035661.1	5.82	2.62	0.22	8.85	27.5	4.64
GAIF01035662.1	14.02	13.26	8.42	37.63	28.41	2.82
GAIF01035663.1	0.28	0.67	0	0.4	0	0.17
GAIF01035666.1	0	0.35	0	0	0.13	0
GAIF01035667.1	0.36	0.06	0.06	2.48	13.54	0.39
GAIF01035668.1	4.58	10.43	2.99	16.21	23.77	0.64
GAIF01035669.1	8.75	4.79	10.12	25.73	12.29	22.81
GAIF01035670.1	0.01	0	0	0.27	0.92	0
GAIF01035671.1	7.16	4.97	3.97	0.95	0.49	0.15
